# Microbial species delineation using whole genome sequences

**DOI:** 10.1093/nar/gkv657

**Published:** 2015-07-06

**Authors:** Neha J. Varghese, Supratim Mukherjee, Natalia Ivanova, Konstantinos T. Konstantinidis, Kostas Mavrommatis, Nikos C. Kyrpides, Amrita Pati

**Affiliations:** 1 Microbial and Metagenome Superprogram, DOE Joint Genomic Institute, Walnut Creek, CA 94598, USA; 2 Department of Civil and Environmental Engineering, Georgia Institute of Technology, Atlanta, GA 30332-0355, USA; 3 Celgene Corp., San Francisco, CA 94158, USA

## Abstract

Increased sequencing of microbial genomes has revealed that prevailing prokaryotic species assignments can be inconsistent with whole genome information for a significant number of species. The long-standing need for a systematic and scalable species assignment technique can be met by the genome-wide Average Nucleotide Identity (gANI) metric, which is widely acknowledged as a robust measure of genomic relatedness. In this work, we demonstrate that the combination of gANI and the alignment fraction (AF) between two genomes accurately reflects their genomic relatedness. We introduce an efficient implementation of AF,gANI and discuss its successful application to 86.5M genome pairs between 13,151 prokaryotic genomes assigned to 3032 species. Subsequently, by comparing the genome clusters obtained from complete linkage clustering of these pairs to existing taxonomy, we observed that nearly 18% of all prokaryotic species suffer from anomalies in species definition. Our results can be used to explore central questions such as whether microorganisms form a continuum of genetic diversity or distinct species represented by distinct genetic signatures. We propose that this precise and objective AF,gANI-based species definition: the MiSI (Microbial Species Identifier) method, be used to address previous inconsistencies in species classification and as the primary guide for new taxonomic species assignment, supplemented by the traditional polyphasic approach, as required.

## INTRODUCTION

Microbes dominate the Tree of Life both in numbers and diversity, rendering their natural classification both difficult and important. In animals, a species is generally defined as a group of organisms that can interbreed (biological species concept) (1), however, this definition cannot be directly applied to assemblages of asexual organisms. As a result, microbial taxonomy employs a polyphasic approach (2,3), which integrates genotypic, phenotypic and chemotaxonomic information about an organism and delineates microbial species based on a consensus of available data. This polyphasic approach relies on a toolbox of methods including DNA–DNA hybridization (DDH), G+C content variation, sequence comparison of select DNA markers (including 16S rRNA), identification of certain metabolites such as fatty acids, polar lipids, cell wall composition and exopolysaccharides, as well as morphological, biochemical and enzymological characterization. It is not uncommon that these different methods in the polyphasic taxonomy toolbox do not fully agree about the suggested classification of the same isolates. For instance, tight genotypic clustering of strains can contradict their biochemical versatility and phenotypic variability, and vice versa. This can indicate both potentially unusual and interesting biological processes as well as experimental errors. However, since the choice of specific methods and the weight assigned to each data type is left to the discretion of the researchers and may vary depending on the organism and the scope of the study, inconsistencies in classification may result. The polyphasic approach, which evolved prior to the emergence of high-throughput sequencing technologies, has enabled prokaryotic classification so far. However, the complete genome of an organism is its ultimate genetic signature and sequencing complete genomes is fast, accurate and no longer cost-prohibitive. Whole-genome based grouping may still be unable to account for physiological variations emerging from a single gene or a small subset of genes, but it maximizes the information taken into account in computing genomic distance and the time is right for transitioning into a more unbiased approach for organism-to-species assignment that leverages whole-genomic information and keeps pace with the discovery of novel microbes.

In this work, we introduce the MiSI (Microbial Species Identifier) method and evaluate whether it can be used as the fundamental guide for measuring genetic distinctiveness of isolates and explore its application toward correcting inconsistent classifications of prokaryotic species and assigning species to newly sequenced organisms. Traditional polyphasic approaches might still be required for classification at higher taxonomic levels and strain level separation. Whole-genome based Average Nucleotide Identity (gANI) has been proposed as a measure of similarity between two genomes by Konstantinidis and Tiedje (4). Here, we augment this approach and have developed a scalable method to compute gANI for 86.5 million genome pairs in the publicly available Integrated Microbial Genomes (IMG) database (5). We calculate gANI for a pair of genomes by averaging the nucleotide identity of orthologous genes identified as bidirectional best hits (BBHs) using fast, yet sensitive, similarity searches (6). In addition to gANI, we consider the fraction of orthologous genes (Alignment Fraction, AF) as a complementary measure of genetic relatedness of a pair of genomes based on gene content. We systematically explore and statistically characterize the relationship between gANI and AF for a large set of diverse bacterial and archaeal genomes. We demonstrate that together, these two measures accurately capture genetic distance between the genomes and are robust in the face of increased sampling of microbial biodiversity. They correlate, in general, well with the existing classifications and allow selection of thresholds, which, if applied uniformly to existing taxa, can lead to consistent species assignments that are predictive of the level of intra-species genetic diversity. We argue that the domain wide application of ANI, which was infeasible a few years ago, has become practical, effective and scalable with the novel variation of AF and gANI computation, as well as value-enriched and made necessary by the recent increase of phylogenetic coverage with whole genome sequences (7,8).

## MATERIALS AND METHODS

### Selection of intra-species genome pairs for determination of AF and gANI species delineating cutoffs

13,512 publicly available bacterial and archaeal genomes from IMG were filtered to retain only the 13,151 genomes that have an acceptable coding density in the range of 700–1200 genes per MB of sequence and less than 2500 scaffolds. The coding density filter was applied in order to minimize the impact of having draft genomes and errors in annotation on predicted coding sequences. For the creation of cliques and clique-groups, all 13,151 genomes were used, which contributed to 86.5M pairs. In order to identify values of AF and gANI that determine whether genomes in a pair belong to the same species, a high-quality subset of the 86.5M pairs was utilized. 2,153 genomes that were taxonomically unclassified or had undesignated species (sp.) were filtered out, thus retaining pairs for 10,998 genomes (Supplementary Table S4) for this analysis. Of these, 8,673 genomes (766 species, Supplementary Table S4) belonged to species with at least two sequenced genomes, thereby forming 1,130,980 intra-species genome pairs (Supplementary Table S4). These pairs were further filtered to retain 1,128,084 pairs having pairwise AF greater than or equal to 0.6. The 31 species that are lost as a result of the AF filter mostly have only two members per species and most pairs have very low gANI (Data Set S3). For determining intra-species AF and gANI cutoffs, the aforementioned 1,130,980 and 1,128,084 pairs, respectively, were utilized.

### AF and gANI computation

The average nucleotide identity was determined for all the genomes in our data set against all other genomes. The algorithm implemented to determine the AF and gANI between two genomes is a modification of the original method proposed by Konstantinidis and Tiedje (4). In our implementation, protein-coding genes of genome A and genome B were compared at the nucleotide level using the high performance similarity search tool, NSimScan (http://www.scidm.org/). The results are then filtered to retain only the BBHs that display at least 70% sequence identity over at least 70% of the length of the shorter sequence in each BBH pair. The gANI of genome A to genome B is defined as the sum of the percent identity times the alignment length for all BBH's, divided by the sum of the lengths of the BBH genes. The AF is computed by dividing the sum of the lengths of all BBH genes by the sum of the length of all the genes in genome A. This computation is performed separately in both directions: from Genome A to genome B and from Genome B to Genome A. The code to perform gANI,AF computation is available for free download at https://ani.jgi-psf.org/html/download.php.

### Implementation of maximal clique enumeration (MCE)

Genome pairs corresponding to the aforementioned 13,151 genomes were used as the input to a PERL script that uses the C++ Bron–Kerbosch module to construct maximal cliques. The Bron–Kerbosch algorithm finds maximal cliques in an undirected graph (10). As its output, it lists all subsets of vertices with the two properties, that each pair of vertices in one of the listed subsets is connected by an edge, and no listed subset can have any additional vertices added to it while preserving its complete connectivity. In this case, each genome is treated as a vertex and an undirected edge is present between two genomes when their minimum pairwise AF is at least 0.6 and their minimum pairwise gANI is at least 96.5.

### Selection of pairs for 16S based validation of AF and gANI species delineating cutoffs

The AF and gANI cutoffs identified for species delineation were further validated in this work using 16S distances. Of the 1,130,980 intra-species pairs, 607,012 pairs that had pairwise 16S distances computed within IMG were used. Of these, 605,717 intra-species pairs that had pairwise 16S distance ≤ 0.03 were used to validate the AF cutoff of 0.6 (Supplementary Figure S1a). Subsequently, 604,384 intra-species pairs that had pairwise 16S distance ≤ 0.03 and AF ≥ 0.6 were used to validate the gANI cutoff of 96.5 (Supplementary Figure S1b).

### Determining the species assignment of the genomes in our data set

The reference taxonomic classifications for genomes evaluated in this work were taken from NCBI's taxonomy database (9,10). Since the traditional approach to assignment of prokaryotic species is polyphasic and there is no central repository that tracks exactly how each strain received its species definition, it is frequently unclear how the strains that have been subjected to genome sequencing have been assigned to their named taxon at the time of their deposition to NCBI's Taxonomy database.

## RESULTS

### Algorithm and data sets for pairwise AF and gANI computation

13,512 publicly available bacterial and archaeal genomes from the Integrated Microbial Genomes (IMG) system were used as the starting point for our analysis. Since AF,gANI computation relies on the identification of orthologs between genomes, gene fragmentation caused by sequencing artifacts and errors in gene prediction are likely to distort the gANI and AF values. Therefore, only 13,151 genomes out of the 13,512 genomes having an acceptable coding density in the range of 700–1200 genes per Mb of sequence and less than 2500 scaffolds were retained.

To determine the AF and gANI between a pair of genomes, orthologs are identified as BBHs derived from nucleotide alignments that display 70% or more sequence identity and at least 70% alignment along the shorter sequence in each BBH pair. AF and gANI are computed as averages over the identities of these BBHs. The AF measures the fraction of coding sequence of each genome that aligns in the aforementioned BBHs, with AF = 0 denoting the absence of BBHs and AF = 1 corresponding to complete conservation of all protein coding genes. Thus, for the purpose of assigning species to a genome, AF serves as an initial filter for identifying candidate species definitions using genome pairs that qualify minimum AF requirements. The final species assignment then relies on gANI, which measures the extent of conservation between orthologous sequences. The gANI of genome A to genome B is defined as the sum of the identities divided by the sum of the lengths of the BBH genes.

The algorithm implemented in the MiSI method to determine the AF and gANI values between two genomes contains two notable modifications of the original method proposed by Konstantinidis and Tiedje (4). First, nucleotide sequences of genes were used instead of segments sampled over the entire genome. Second, pairwise nucleotide sequence alignment was performed using NSimScan (http://www.scidm.org/), which is based on the same dictionary lookup idea as NCBI BLAST (11) but is 10 to 100 times faster because of its careful design and implementation. This translates to >10-fold speedup for pairwise intra-species AF,gANI computations and 5-fold speedup for pairwise inter-species AF,gANI computations when compared with the original method (Supplementary Table S1), enabling the computation of 86.5 million pairwise AF,gANI values in approximately 48 hours of walltime on a compute cluster comprised of Intel Xeon L5520 2.27 GHz cores. The total computation time was 190,000 cpu core hours. Detailed hardware configuration of the compute cluster is available at http://www.nersc.gov/users/computational-systems/genepool/configuration/.

### Determination of species-level AF and gANI threshold values

**Figure 1.**
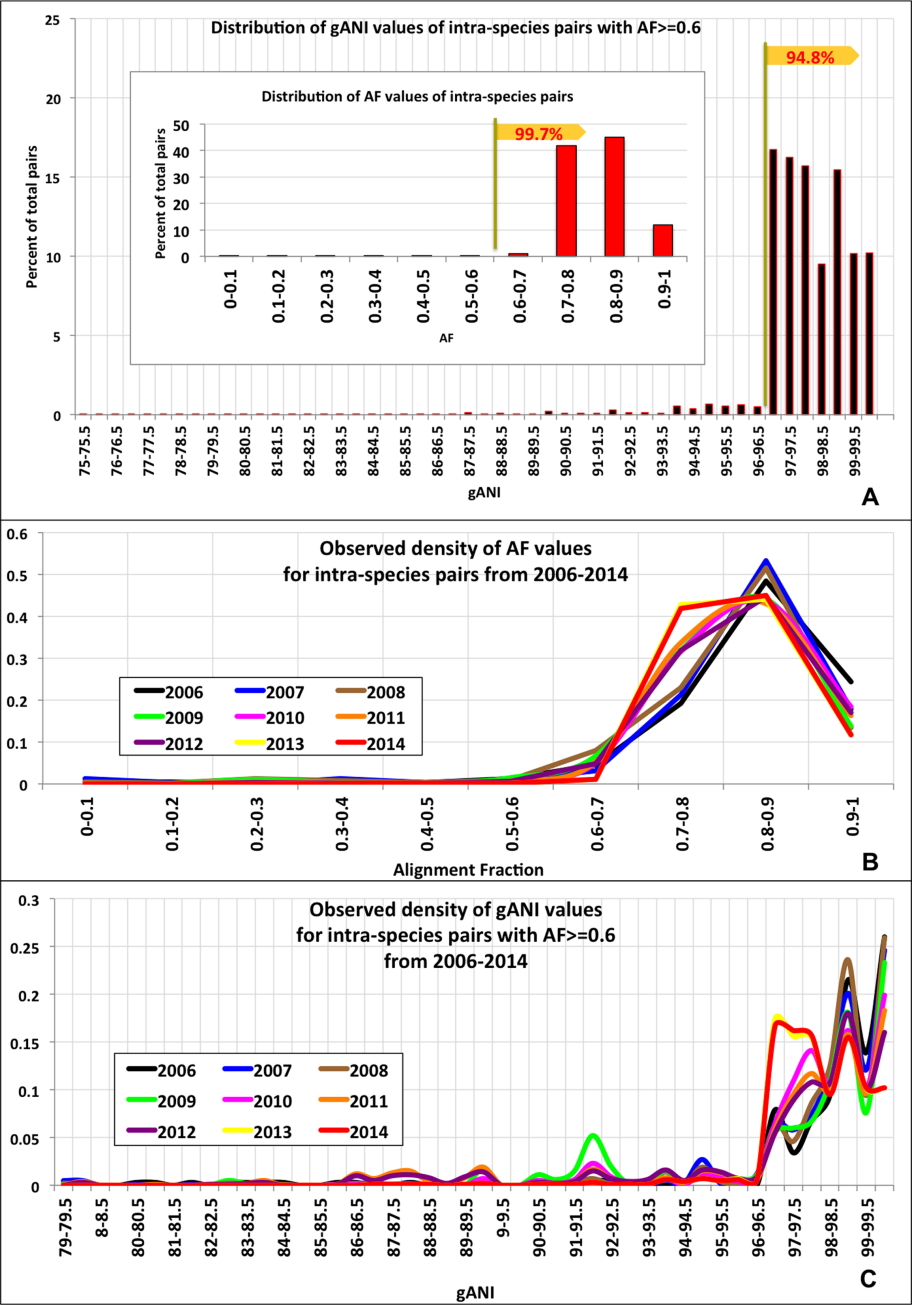
Determination of species-level AF and gANI values **(A)** Distribution of AF values of all intra-species pairs (inset) and gANI values of those intra-species pairs that have AF ≥ 0.6 **(B)** Distribution of AF values evaluated at multiple time points (yearly, 2006–2014). **(C)** Distribution of gANI values evaluated at multiple time points (yearly, 2006–2014).

As the first step, gANI and AF values above which two genomes are considered related enough to be assigned to the same species were identified. To determine these thresholds, AF and gANI values for 1.1 million pairs of genomes belonging to the same species per existing taxonomy were plotted. Figure 1A shows the distribution of AF and gANI for these intra-species genome pairs. For 99.7% of all intra-species pairs, at least 60% of their gene content was found in orthologous gene pairs, resulting in AF values greater than 0.6. Additionally, we plotted the observed density of the AF values for intra-species pairs from 2006 to 2014 (Figure 1B) using incremental snapshots of IMG and the majority of these pairs (99.7% in 2014) had AF values of 0.6 or higher. A small number of species (31 species, Supplementary Table S2) consist of genome pairs having AF less than 0.6. *Pseudomonas syringae* and *Clostridium botulinum* are examples of two such species that consist of genome pairs having less than 60% of their genes related, with some *C. botulinum* genome pairs having as little as 23% of genes in orthologous pairs (Supplementary Figure S2). AF values below 0.6 reflect substantial divergence between the strains assigned to the same species compared to a great majority of named species, and denote cases that could be potentially adjusted toward a more predictive, genome-based taxonomy. This has been observed in *C. botulinum* where strains within this species cluster into 4 phylogenetic lineages that represent distinct physiological characteristics such as the toxin they produce (12,13). The gANI plot reveals a similar trend where 95% of the intra-species pairs exhibit nucleotide identity greater than 96.5 while the remaining (5%) constitute a long tail. Misidentified organisms account for a large part of this tail (SI, Figure S3). Additionally, the distribution of gANI values was evaluated yearly from 2006 to 2014 (Figure 1C) using incremental snapshots of IMG and the majority (94.8% in 2014) of intra-species pairs consistently demonstrated gANI values of 96.5 or higher. Therefore, AF and gANI values of 0.6 and 96.5, respectively, were identified as minimum thresholds to assign a genome pair to the same species. We also established that 16S distance, which is a commonly used method from the polyphasic approach for species assignment, is highly correlated with these cutoffs (Supplementary Figure S1). Of all intra-species genome pairs showing 16S distance 0.03 or less, 99.8% have AF at least 0.6, 92.2% have gANI of at least 96.5, while 92.2% qualify the aforementioned cut-offs for both AF and gANI.

### Clustering of genomes in the MiSI method

**Figure 2.**
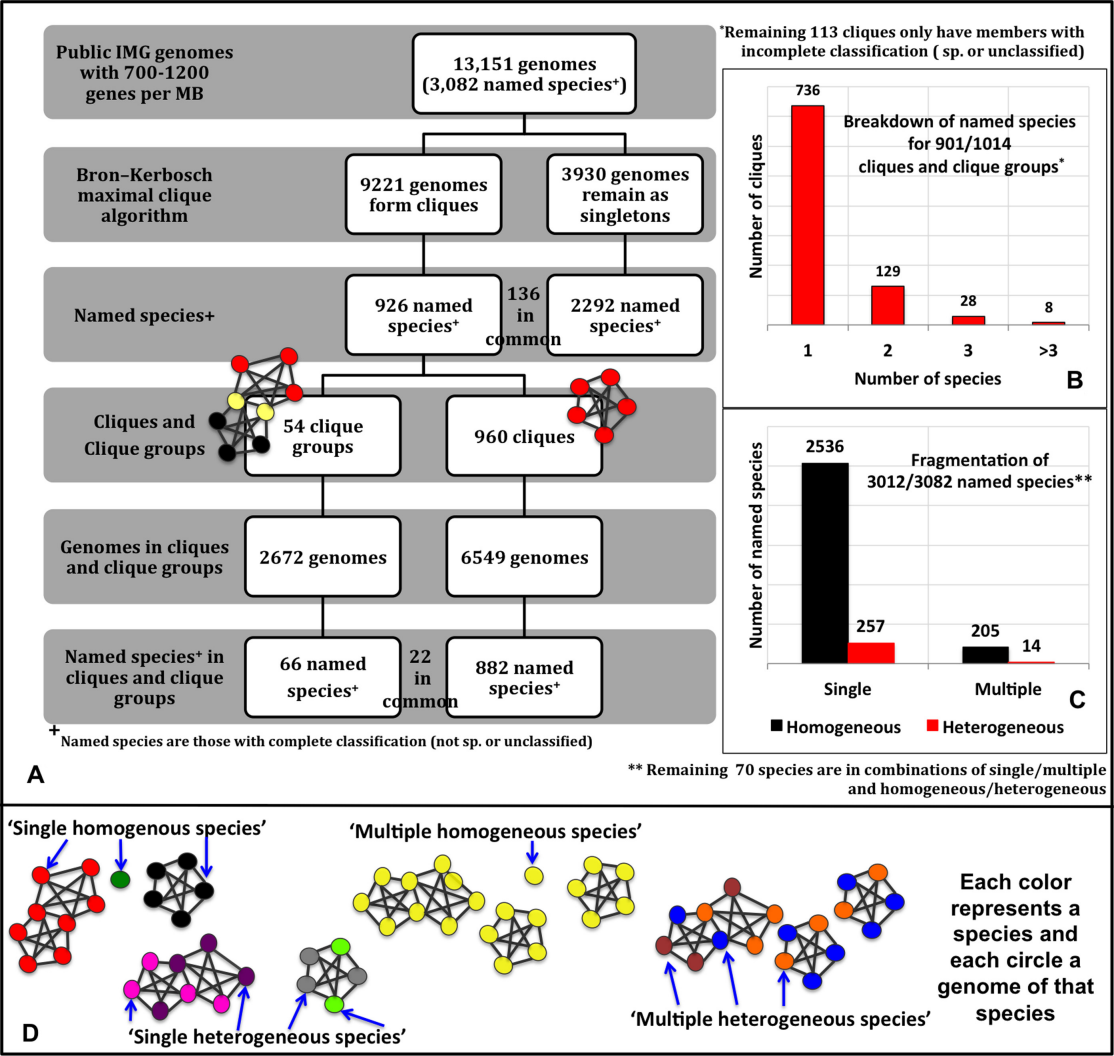
**(A)** Breakdown of genomes and species into cliques, clique groups and singletons. This figure shows the number of genomes and named species at each step of our method. The genomes/species in common are annotated between the boxes. **(B)** Breakdown of named species for 901 clusters. This graph shows how many clusters are populated by one species, two species and so on. **(C)** Breakdown of species into different categories. This graph shows how many species fall into the ‘single homogeneous’, ‘multiple homogeneous’, ‘single heterogeneous’ and ‘multiple heterogeneous’ species category. **(D)** Pictorial description of the species categories. Each color represents a species and each circle a genome of that species. Species ‘black’ is present only in one clique and that clique only has genomes of that species, making it a ‘single homogeneous species’. Species ‘pink’ has genomes in only one clique group, but that clique group has genomes from other species (‘purple’), thus making it a ‘single heterogeneous species’. The genomes of species ‘yellow’ are present in multiple clusters, but each cluster they belong to only have genomes of that species, making it a ‘multiple homogeneous species’. Species ‘blue’ has genomes in multiple clusters and each cluster has genomes of other species (orange, brown), thus making species ‘blue’ a ‘multiple heterogeneous’ species.

To examine the differences between the species assignments based on the conventional polyphasic approach and the aforementioned AF, gANI cut-offs, we clustered all sequenced microbial genomes based on the above-mentioned cut-offs. Since the clustering is agnostic of existing taxonomy, it provides the opportunity for unbiased exploration of how genomes group based solely on their genomic relatedness and can have implications on whether or not natural groups of prokaryotic genomes exist. For this, we applied MCE, a form of complete linkage clustering (14), to genome pairs linked with species-level AF and gANI. MCE generates two types of clusters: (i) ‘cliques’ which are complete graphs, where each vertex represents a genome and every vertex is linked to every other vertex and (ii) ‘clique-groups’ which are formed when multiple maximal clique configurations are possible, resulting in cliques having genomes in common (Figure 2A).

MCE was applied to genome pairs having bi-directional AF of at least 0.6 and bi-directional gANI of at least 96.5. Within a clique, species-level AF,gANI linkage is preserved across all pairs of genomes above the aforementioned thresholds indicating high similarity in genetic content, making it the ideal object to represent a microbial species. Genomes belonging to a single taxonomic species often disperse into multiple cliques/singletons when AF,gANI-based clustering is applied. This is due largely to the presence of divergent strains that have pairwise AF,gANI below the determined thresholds. Overlapping cliques are consolidated into clique-groups as a post-processing step after MCE. Application of more relaxed models of clustering such as single and average linkage clustering ‘hides’ such weak links by placing them within a cluster and leads to loss of valuable information about divergent strains (See SI, Figure S4, where different clustering methods are compared).

1.1M genome pairs meet the required thresholds and each of the 9221 genomes that constitute these pairs has significant AF,gANI to at least one other genome, contributing to 960 cliques and 54 clique groups, while approximately 3930 genomes remain singletons. Figure 2A breaks down in detail, the cliques, clique-groups and singletons in terms of genomes and species. The fact that, only 7% of the species are in clique-groups while the remaining are in individual cliques, lends support to the existence of species boundaries. Henceforth, the term ‘cluster’ will be used as a common term to indicate a clique or a clique-group. Approximately 72.6% of all clusters (736 of 1014) are composed of organisms with the same taxonomic species (Figure 2B), revealing that existing species assignments are largely consistent to one another with respect to their intra-species diversity. However, a subset of the species are distributed among multiple cliques so that for only 53.4% of clusters there is a one-to-one relationship between the cluster and existing species assignments. Of the 3082 named species in this study, 790 are in clusters, while 2156 are singletons. Representatives of 136 species are present both in cluster(s) and as singleton(s). Thus, genomes of currently named species fall into four categories with respect to their distribution in clusters. A species is considered (i) ‘single homogeneous’ when it is present as a singleton or in only one cluster with all other genomes from the same species (82.3%); (ii) ‘multiple homogeneous’ when it is present in multiple clusters with other genomes from the same species, or as multiple singletons or a combination of a singleton and a ‘clean’ clique (6.6%); (iii) ‘single heterogeneous’ when it is present in a single cluster with genomes from other species (8.4%); (iv) ‘multiple heterogeneous’ when it is present in multiple clusters with genomes from other species (0.4%) (Figure 2D).

Species that are categorized as ‘single-homogeneous’ are in complete agreement with existing taxonomic definitions. Genomes of ‘multiple homogeneous’ species may have undergone significant genomic divergence, possibly forming one or more cores of new species, which has gone unnoticed. The genomes belonging to the other two categories are assumed to be misidentified strains. The breakdown of all species into the above categories is provided in Figure 2B and Figure 2C.

The robustness of the AF and gANI cut-offs is underscored by the fact that the number and composition of the clusters remains unchanged across different modes of clustering and across a spectrum of thresholds (SI). The novelty of generating maximal cliques using the massive data set of more than 13,000 genomes reveals species boundaries and identifies divergent strains for the first time across such a large phylogenetic space.

### Validation of results (Biological significance of cliques and clique groups)

**Figure 3.**
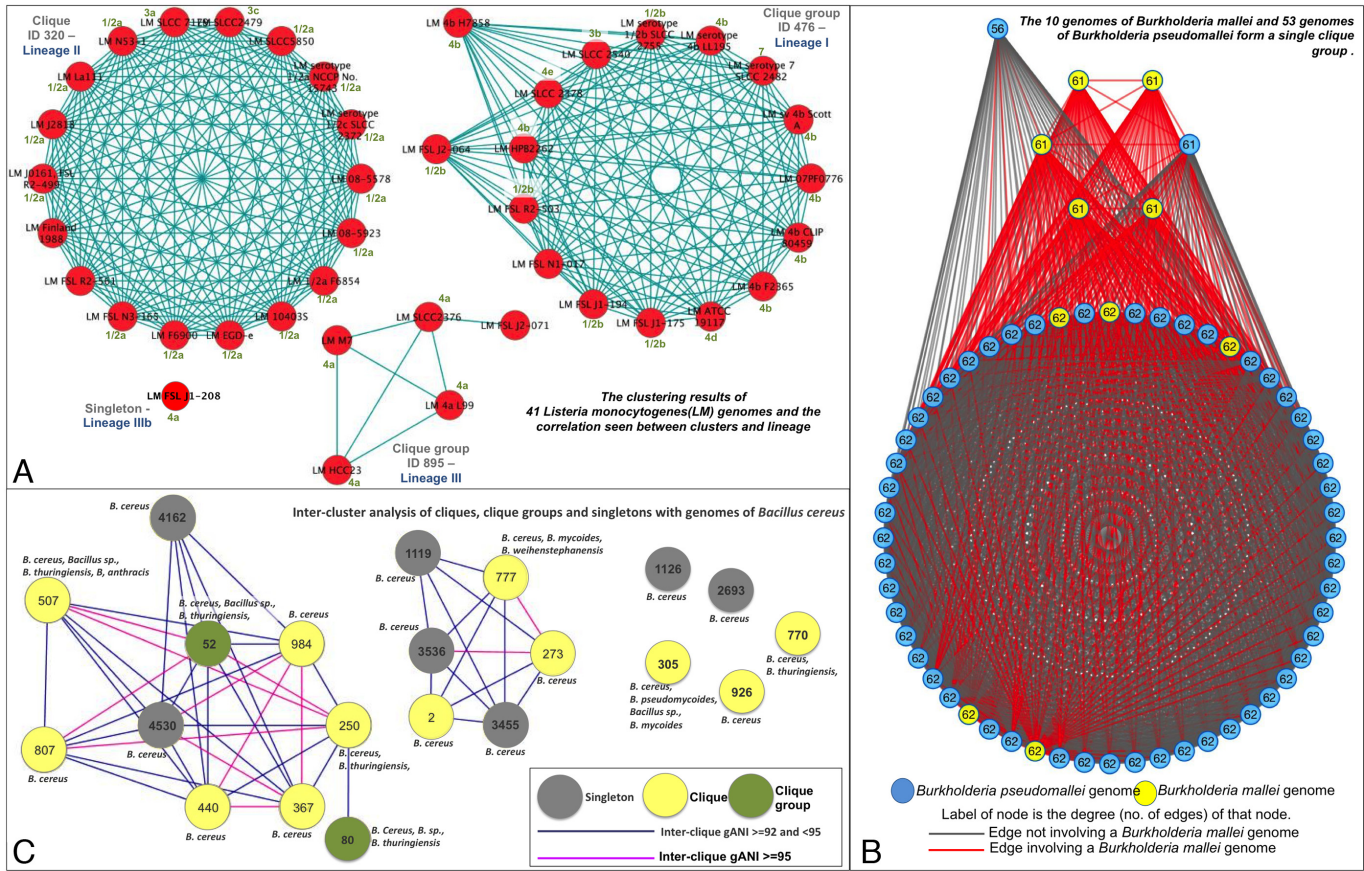
Validation of results. (**A**) The clustering results of 41 *Listeria monocytogenes (LM)* genomes and the correlation seen between clusters and lineage. Each red circle depicts a genome and each blue line depicts an gANI ≥ 96.5 and an AF ≥ 0.5 between the two genomes it joins. Genome name is annotated in black where LM stands for *Listeria Monocytogenes*, the lineage associated with each group is specified in dark blue and known serotype information for each genome is specified in green **(B)** Cluster based analysis of *Bacillus cereus* group. Each circle represents a cluster or singleton, with a gray circle depicting a singleton, a yellow circle depicting a clique and a green circle depicting a clique group. A pink line depicts the inter-cluster gANI if it falls between 92 and 95, and blue line if it is between 95 and 96.5. The species present in each cluster is annotated with text above/below/next to the cluster. **(C)** Pictorial description of the *Burkholderia mallei* and *Burkholderia pseudomallei* clique group. Each circle represents a genome, where a yellow circle represents a *B.mallei* genome and a blue circle represents a *B.pseudomallei* genome. Each line or edge depicts an gANI ≥ 96.5 and AF ≥ 0.5 between the two genomes it joins. More specifically, a red line is an edge involving a *B.mallei* genome and a gray line is an edge that does not. The degree of the genome/node (the number of genomes with which it has the required gANI and AF) is annotated in black.

*Listeria monocytogenes* is an example of a species that is ‘multiple homogeneous’. The 41 genomes of this species are distributed into two clique-groups, one clique and one singleton, and each cluster is populated only with genomes from this species. Several studies, that explored the ribotypes, serotypes and virulence gene polymorphisms of *L. monocytogenes*, identified three distinct lineages with differences in pathogenic potential (15). Analysis of the *Listeria monocytogenes* clique and clique-groups shows their complete agreement with the classification of strains into the three lineages (Figure 3A, Supplementary Table S3). Further, the genome that remains as a singleton (*Listeria monocytogenes* FSL J1–208) has been shown to have a smaller chromosome size and belongs to a virulent uncommon phylogenetic lineage IV (16). Both genomic and phenotypic evidence indicate that the strains of *L. monocytogenes* form distinct separate groups and suggest that this species has to be revisited. However, other methods employed by the polyphasic approach, such as 16S-distance (Supplementary Figure S5) and phylogenetic marker gene based ANI (pMG-ANI) (17), fail to capture this divergence presumably due to reduced resolution, placing all strains of *L. monocytogenes* in a single cluster.

*Burkholderia mallei* and *Burkholderia pseudomallei* fall into the category of ‘single heterogeneous’ species. Detection and differentiation of these two species has been discussed heavily in scientific literature (18–21). The 10 genomes of *B. mallei* and 53 genomes of *B. pseudomallei* form a single clique-group with an intra-clique average gANI of 99.6. *B. pseudomallei* is a gram-negative bacterium recovered from water and wet soils while *B. mallei* is an obligate mammalian pathogen that has evolved from the former through systematic genome reduction to adapt to an animal host (22). A comparison of the genome sequences between the two named species revealed that they are 99% identical within conserved regions and mainly differ in genome size. This identity is captured by gANI as we place them in a single cluster, however we also capture the difference in size since they form a clique-group with missing links due largely to low AFs in inter-*mallei*-*pseudomallei* genome pairs (Figure 3B). Thus, we have correctly identified *B. mallei* and *B. pseudomallei* as a group of genomes in an evolutionary genetic continuum where a subset has diverged from the others, and, if the goal is to have relative uniform species designations and taxonomy, strains of these two species should be classified as a single species. The final unification of the two species and the exact definition of species will depend on pathogenicity of individual strains, the Code of Nomenclature governing prokaryotes and the presence of type strains.

Species that fall into the ‘multiple heterogeneous’ category consist of genomes that exist in multiple clusters that are also populated with genomes belonging to other species. The *Bacillus cereus* sensu lato presents one such well-known species complex (23,24). Strains of *B. cereus (Bc), B. thuringiensis (Bt), B. mycoides (Bm), B. pseudomycoides (Bp), B. weihenstephanensis (Bw)* and *B. anthracis (Ba)* are often intermingled within cliques (Figure 3C). Our cliques correlate strongly with available metadata on pathogenicity, isolation source and habitats of the strains (23–25). The largest species cluster (70 genomes, clique-group #80) consists of agricultural biopesticide insect pathogenic *Bt* and closely related *Bc* strains isolated from the environment or infected human wounds. The second largest cluster (45 genomes, clique #507) is dominated by *Ba* and includes a handful of pathogenic strains of *Bc* and *Bt*, which are characterized by the production of anthrax-like toxins and/or polyglutamate capsule. The third largest cluster (21 genomes, heterogeneous clique #777) contains strains of *Bc, Bm* and *Bw*, which were isolated from the environment (forest/soil) or food (dairy) and do not have demonstrated pathogenic properties. Although Clique #770 and clique-group #52 contain 17 genomes each, the clique consists of only environmental (soil) strains while the clique-group is made up of emetic strains. Additionally, 9 cliques/clique-groups and 7 singletons are present. We also observe that *Bc subsp. cytotoxis NVH 391–98*, which has been identified as an outlier in literature (25), is classified as a singleton in this study.

The intra-cluster gANI for the aforementioned clusters is consistently very high, well above 96.5, strongly supporting the hypothesis that genomes in each cluster should be reclassified to a single species. For example, clique #305 is comprised of 8 *Bacillus* genomes isolated from soil, including one *Bp*, two *Bm*, three *Bc* and two without species definitions. This clique is home to the type strain for *Bacillus pseudomycoides*, namely, *Bacillus pseudomycoides DSM 12442*. Therefore, all participating genomes in this cluster should be considered with respect to being reclassified as *Bacillus pseudomycoides*. Further, Figure 3C shows clusters that represent closely related sub-species (pink edges, gANI ≥ 95) versus clusters that represent moderately related sub-species (blue edges, 92 ≤ gANI ≤ 95).

### Advantages of the MiSI method

The implementation of AF,gANI in MiSI is fast, scalable and an accurate reflection of the genetic similarity between a pair of genomes. Species assignment using the MiSI method is affected very little or not at all by ‘draftiness’ of a genome since our analysis showed that increasing the number of scaffolds of a genome up to 2500 scaffolds and genome reduction up to 25% had no effect on the genome being placed in the accurate species cluster. (Supplementary Material and Supplementary Figure S10)

In addition, the comparison of our method to other ANI-based methods that determine genomic distance, such as Jspecies (26) and SpecI (17), has been discussed in detail in SI, and highlights the definite advantages of using MiSI over others (Supplementary Figure S6). Jspecies utilizes fragments from the entire genome and offers a choice of alignment methods (Blast (ANIb) and Mummer (ANIm)). When genomes are closely related, ANIb and ANIm match gANI values closely, but as the relatedness of genomes decreases, the disparity between the values increases. Additionally, Jspecies was unable to compute ANIs for a large number of genome pairs and crashed. SpecI utilizes phylogenetic marker genes to compute ANI and thus offers lower resolution since it utilizes a very small fraction of all available genomic sequence for an organism. For the average bacterial genome, the time taken to compute pMG based ANI and gANI are comparable (SI), with the latter providing significantly higher resolution.

**Figure 4.**
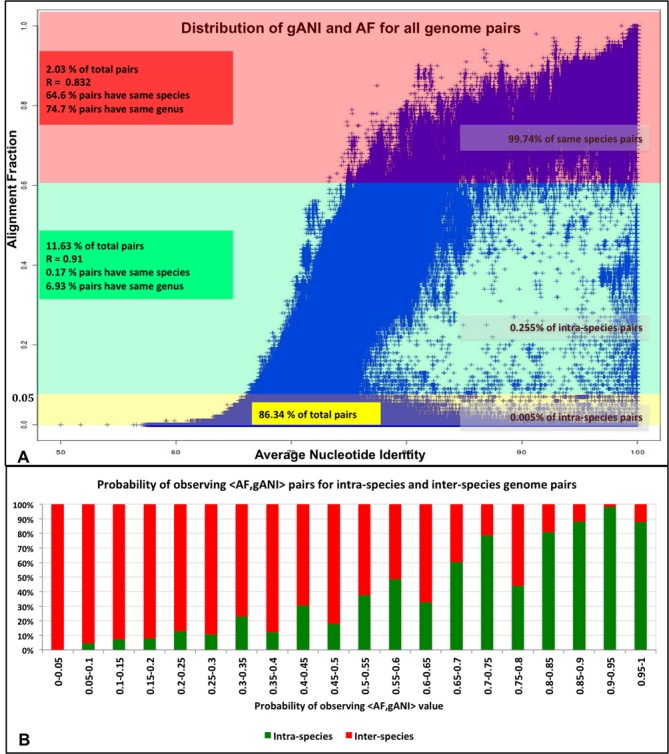
Relationship between gANI and AF. (**A**) Distribution of AF and gANI values for all pairs of genomes. (**B**) Probabilistic modeling of <AF,gANI> tuples for intra-species and inter-species pairs, P_r_[AF = a,ANI = b] = P_r_[AF = a]* P_r_[ANI = b|AF = a].

As first application of this method we have determined an empirical distribution that can used to compute the probability that a pair of genomes belongs to the same species, given an AF and gANI value. Since the plot of gANI against AF for 86.5M pairs of genomes (Figure 4A) suggests that the two variables are highly correlated (SI), the empirical probability of gANI = b being generated by an intra-species pair is computed conditionally on the value of AF being a. In summary,

(1) \begin{equation*} \begin{array}{*{20}l} {{\rm P}_{\rm r}^{{\rm intra - species}} [{\rm AF} = {\rm a},{\rm ANI} = {\rm b}] = } \\ {{\rm P}_{\rm r}^{{\rm intra - species}} [{\rm AF} = {\rm a}]*{\rm P}_{\rm r}^{{\rm intra - species}} [{\rm ANI} = {\rm b}|{\rm AF} = {\rm a}]} \\ \end{array} \end{equation*}

To determine how likely a pair with P_r_^intra-species^[AF = a,ANI = b] = x is to belong to the same species, Figure 4B may be used. It shows the ranges of final probabilities for ‘real’ intra-species pairs when an AF cut-off of 0.6 is used. We observe that higher probabilities have greater likelihood of corresponding to intra-species pairs with the 1% false positive rate probability level being 0.6. The code available at (https://ani.jgi-psf.org/html/download.php) can be used to compute pairwise AF and gANI values between a pair of genomes. The values can be used to look up the intra-species probability in the table available at https://ani.jgi-psf.org/download_files/ProbabilityTable.txt or can be used as input values to implemented web application (https://ani.jgi-psf.org/html/prob.php) that uses this empirical data to allow an external user to determine the intra-species probability.

The species-level cliques and clique-groups have multiple varied applications. MCE provides the ability to sharpen our view of the evolutionary relationships between genomes, since it identifies not only highly conserved species-cores in the form of cliques, but also species in the form of clique-groups and species separation in the form of multiple homogeneous species that could be revisited taxonomically. Since clique-groups are composed of multiple cliques that share common genomes, the linkage between all the genomes in a clique-group is not preserved, and thus they are ideal for detecting species with divergent genomes. Subsequently, average inter-cluster and intra-cluster gANI was also computed within and between all cliques, clique groups and singletons, as described in Methods in order to discover the extent of genetic conservation within and between clusters and between clusters and singletons (Supplementary Figure S7).

Genome cliques identified in this work are valuable in the context of establishing whether an uncultured organism represented as a single-cell genome or a genome isolated from a metagenome belongs to an existing species or is a novel candidate species. This decision can be formed based on the average AF,gANI of an uncultured genome to existing cliques and clique-groups. Single-cell genomes are often partial, so the usual AF cut-offs do not apply to single-cells.

2,367 of sequenced genomes belonging to 10% of known genera lack species assignments. Using cliques, 326 of these unclassified genomes (denoted as ‘*sp*.’ or ‘unclassified’) can be assigned to the existing species based on their clustering patterns (Data Set S1). Furthermore, there are 109 cliques and 4 clique-groups that are populated entirely by genomes without any species definitions and thus, represent novel species awaiting taxonomic descriptions (SI, Data Set S2).

The composition of cliques also highlights the unexpected distribution of type strains. ‘Type Strain’ is defined as a living culture chosen to represent a prokaryotic species; therefore, the type strain of a species should not be identical or highly similar with the type strain of any other species (27). Indeed we find that of the 445 cliques containing at least one type strain, 414 contain a single type strain. 31 cliques contain type strains from multiple species, that might be the result of either classification based on clinical evidence or incorrect practices where the classification of a single strain species was forced with low evidence. These cliques have been described in detail at https://ani.jgi-psf.org/html/analyses.php?page=typestrains. An example is Clique 757 (Supplementary Figure S8), which consists of five genomes, three of which are types strains belonging to three different species (Caldicellulosirupter kristjanssonii 177R1B, Caldicellulosirupter acetigenus DSM7040, Caldicellulosirupter lactoaceticus 6A). Strain 177R1B was proposed as a type strain of a new species on the basis of low DDH rates with C. lactoaceticus 6A (28). Such low DDH rates are questionable since these genomes display AF of 0.81 and gANI of 98.51, and probably attributed to the high experimental noise of the DDH method (unlike the MiSI method). Additionally, ∼40% of the singletons (1551/3930) are type strains suggesting that greater coverage of sequencing around these ‘type species’ may be required to provide a more complete view of the diversity of the corresponding species. Since type strains serve as the nomenclatural type of a species and a reference point, greater care must be exercised to ensure that they are accurately named.

The cliques and clique-groups generated in this work will be maintained as part of the IMG system and associated analysis tools will be served to the community through IMG. We propose that these tools be used by the community as the basis for identification of species of newly sequenced genomes. Further, the groupings identified and genomes and species used in our analysis have been represented in detail at https://ani.jgi-psf.org. The website provides the opportunity for researchers to explore specific genomes or species of interest and identity groups of genomes or species of particular significance. The software provided can also be used in order to determine the similarity of newly sequenced genomes to existing reference genomes, an application that is being actively used in the sequencing pipeline at the Joint Genome Institute.

Further, these groups will form the stepping-stones for our intended future directions that include the overlaying of these clusters with phenotypic and biochemical information. Since this task is manually intensive, and involves the collection of extensive metadata by way of deep literature survey, it is beyond the scope of this manuscript.

## DISCUSSION

Several reports have already illustrated that microbial taxonomic assignments are inconsistent with genetic information for a large number of species (17,29,30). According to a recent ASM report, ‘*in moving forward with microbial taxonomy, it is critical to determine whether microorganisms cluster in groups with meaningful commonalities or to determine what commonalities may be best used to cluster microorganisms into meaningful groups*’ (29). For the first time, gANI was applied across all available sequenced prokaryotic genomes and its potential to cluster microorganisms into such ‘meaningful groups’ was explored. We have shown that the combination of AF and gANI provides an objective and robust measure of genetic relatedness. The MiSI genome groups have been compared with ‘named’ species; similarity of 16S rDNA, and similarity of conserved core pMGs and the advantages of our method has been demonstrated (Supplementary Information). The examination of cliques highlights several inconsistencies in the traditional classification of strains into species based on the polyphasic approach. Genomes from 17.7% of all species were assigned into cliques based on the MiSI method in a manner that disagreed with existing taxonomy. This percentage becomes as high as 49% if only species that participate in cliques are considered. 3% of all species (99/3082) are represented in multiple cliques/clique-groups suggesting that multiple genetically divergent strains were assigned to these species. Furthermore 2% of species (66/3082) participate in clique-groups, which represent species that are in the process of forming one or more new species cores. These species are comprised predominantly of host-associated microbial genomes (ani.jgi-psf.org). We also observed that these species were not a direct result of over-sampling of associated species since species with various degrees of sampling are uniformly represented in clique-groups; no correlation was also observed between high coverage of a species in terms of number of sequenced strains and its participation in higher number of homogeneous cliques (Supplementary Figure S9). Although we recognize that biases in sampling and cultivation play a role in the structure of the MCE clusters and the connectivity of genomes, all of the artifacts discussed above provide valuable insight into the evolutionary dynamics of prokaryotes and can be used to explore central questions such as whether microorganisms form a continuum of genetic diversity or distinct species represented by distinct genetic signatures.

## Supplementary Material

SUPPLEMENTARY DATA
