# Supplementary material for: Microbial species delineation using whole genome sequences
Source: Nucleic Acids Res. 2015 Jul 6;43(14):6761–71. doi: 10.1093/nar/gkv657 (PMC4538840; doi:10.1093/nar/gkv657)
Supplement: SUPPLEMENTARY DATA [file supp_43_14_6761__index.html]

Microbial species delineation using whole genome sequences — Microbial species delineation using whole genome sequences — SUPPLEMENTARY DATA 

# Microbial species delineation using whole genome sequences

## SUPPLEMENTARY DATA

- SUPPLEMENTARY DATA
- SUPPLEMENTARY DATA
- SUPPLEMENTARY DATA
- SUPPLEMENTARY DATA
