# Supplementary material for: Microbial species delineation using whole genome sequences
Source: Nucleic Acids Res. 2015 Jul 6;43(14):6761–71. doi: 10.1093/nar/gkv657 (PMC4538840; doi:10.1093/nar/gkv657)
Supplement: SUPPLEMENTARY DATA [file supp_gkv657_nar-03569-n-2014-File006.docx]

**Supplementary Information**

***Correlation between AF and gANI***

In Figure 4a, three distinct AF intervals are observed: [0,0.1), [0.1,0.6), [0.6,1]. As expected, the interval [0,0.1) boasts the highest concentration of data points (86.34%) with AF and gANI displaying no linear correlation. In the intervals [0.1,0.6) and [0.6,1], a very strong positive correlation is observed between AF and gANI values suggesting a strong dependence between the fraction of orthologous genes and the extent of their conservation. In the AF interval of [0.6,1], 74.7% of the points represent pairs of genomes belonging to the same genus, while 64.6% of these points represent pairs of genomes belonging to the same species. This interval also includes the highest concentration of intra-species genome pairs with 99.74% of all intra-species genome pairs being located here. A strong positive correlation (R=0.83, p=0) is observed between AF and gANI in this interval as well.

***Analysis of NCBI-taxonomy-based intra-species genome pairs that do not display AF and gANI above the recommended cutoffs:***

The 1,130,980 NCBI-taxonomy-based intra-species genome pairs that were used to determine AF and gANI thresholds were further examined with respect to the cutoffs. The gANI value for 1,128,084 intra-species pairs (8,558 genomes) that had an AF>=0.6 were plotted. We found that 5,225 genomes (252 species) forms at least one intra-species pair with gANI below the previously identified cutoff of 96.5 and thus contribute to the tail seen in Figure S3. 4,673 of these genomes also form at least one intra-species pair with gANI of 96.5 or more. *Escherichia coli* genomes form the majority of this list, which includes 161 individual species that are mostly pathogens and include strains of *Escherichia*, *Salmonella*, *Acinetobacter,* *Staphylococcus, Helicobacter, Leptospira, Vibrio,* and *Bacillus* among others*.* These depict an evolutionary scenario where, although most members within the species core share high genomic similarity/relatedness, some strains have diverged and are evolving rapidly. For example the *Bacillus cereus* group has confounded microbiologists for years and yet there is no consensus to separate individual members of the group into well-defined species(1, 2). For divergent groups such as these, the MiSI method can be applied to separate members of such species into highly conserved, well-defined species clusters that are reproducible with the sampling of more genomes from the same species. The remaining 552 genomes (91 species) form pairs with gANI less than 96.5. 17 *Streptococcus mitis* genomes top this list of 91 distinct species. Comparative genomic analysis of several *S. mitis* genomes from different geographical regions has shown a highly variable accessory genome attributed to extensive gene transfer events(3). Overall, these genomes can be considered to be more diverse than the average “species” with the latter being indicated by connectivity based on AF>=0.6 and gANI>=96.5. Such species with divergent subsets should be split into different species if the goal is to have a more predictable and systematic genome-level classification. These represent 6.5% of all genomes examined above, and should be investigated with respect to discrepancies in their current species assignments and divergence from the other members of their respective species clusters.

***Effect of using different clustering approaches at different threshold combinations***

We explored the impact of using different clustering methods towards identification of species-level genome groups. In particular, we compared the use of single-linkage (SL), average-linkage (AL) and complete-linkage clustering (MCE) for creating clusters. To do so, we first generated single-linkage, average-linkage and complete-linkage clusters at every combination of gANI values in the set {93, 94, 95, 96, 96.5, 97, 98} and AF values in the set {0.5, 0.6, 0.7}. Thus each clustering method was implemented for 21 combinations of AF and gANI. We then generated a 13,151 x 13,151 genome-linkage matrix for each combination of AF and gANI values for each mode of clustering. Each row/column in the genome-linkage matrix represents a genome and a cell (i,j) is assigned a value of 1 if genome i and genome j are assigned the same cluster. For each AF and gANI pair, the number of permutations in the genome-matrix in moving from single-linkage clustering to average-linkage clustering and from average-linkage clustering to complete-linkage clustering were recorded. These numbers are shown in Figure S4. We see that for gANI 96.5, the clusters create remain the most stable across all methods. On further analysis of the clusters formed at gANI 96.5 and AF 0.6, we observed that the clusters computed using all three methods at our predetermined thresholds were found to be exactly the same if cliques and clique groups are treated equally. This demonstrates the robustness of AF and gANI cutoffs determined statistically in order to ascertain the high level of genomic identity indicative of species in a given pair of genomes. Results of this clustering analysis are available upon request.

***Robustness of the MiSI method to incomplete and draft genomes***

The rapid pace of improvements in genome sequencing is unmatched by genome assembly techniques and today assembly into “high quality draft” genomes is much more feasible than generating “finished” genomes. Our analysis for determining the AF and gANI species delineating cutoffs, and for forming species level clusters, used genomes in 2,500 or fewer scaffolds, which amounted to 99.5% of all available bacterial genomes. The Integrated Microbial Genomes (IMG) system hosts JGI-sequenced genomes, user-submitted genomes, and publically released NCBI genomes. Currently, it contains 28,508 bacterial genomes, of which, only 141 genomes are in more than 2,500 scaffolds (most of these 141 genomes were submitted by external users of IMG for their own studies and were marked as “low quality” per IMG’s internal pipelines that determine the quality of gene calls and sequences for a given genome). Therefore, our cutoff on the number of scaffolds for determining “draftiness” is very inclusive.

To validate this cutoff of 2,500 scaffolds, and to explore the impact of “draftiness” on the correct placement of a genome in its species cluster, the following analysis was performed.

First, the effect of “draftiness” on genome completeness was examined. “Draftiness” was measured in terms of the number of scaffolds while genome completeness was captured as the percentage of the 40 phylogenetic marker genes (represented by COGs) (4) that could be detected in each genome. “Draftiness” was plotted against “completeness” for the 13,151 genomes included in this study and a significant correlation between the two wasn’t observed (Figure S10). In the absence of significant correlation between these two variables, it can be concluded that the existence of a genome in a large number of scaffolds does not imply that the genome is “incomplete” and/or missing necessary marker genes.

Further, “draftiness” manifests as missing genes and partially called genes. Our method utilizes bidirectional best hits (BBHs) as the basis for calculating AF and gANI values. Since qualifying BBHs are required to have at least 70% coverage of alignment on the shorter gene in the BBH pair, the effect of partial genes is mitigated to a large extent, making MiSI robust to genomes “draftiness”. We see this robustness being displayed in several cliques where genomes of a single scaffold have been clustered correctly with genomes that exist in multiple scaffolds. One such example is the *Lactococcus lactis* species, the genomes of which are present in two cliques made up of 6 genomes each. Clique #54 contains strains of *L.lactis.cremoris* while clique #569 contains strains of *L.lactis. lactis* and one *L.lactis. cremoris* which on further investigation was revealed to be a *L.lactis. lactis* that was incorrectly named in IMG (NCBI project ID 67863). Only one member of clique #569 (*L. lactis. lactis* Il1403) has all 40 required pMG’s and was finished .The remaining members are in multiple scaffolds and have less than 40 pMG’s.

In order to quantitatively test the effect of draft genomes, we fragmented the “finished and complete” genome sequence of *L. lactis cremoris* A76 (IMG ID 2511231149) present in clique #54 to generate 15 artificial genome files, with the genome being divided into different number of fragments in each file (2, 4, 8, 16, 32, 64, 128, 256, 512, 1024, 2048, 2500, 3000, 3500, 4000 fragments each). Gene calls from Prodigal (4) were used to calculate the AF and gANI of each artificial genome to all the other genomes present in our database. The results show that the genomes that have been fragmented up to 2500 scaffolds display the required AF and gANI to all the other members of clique #54 and to no other. Fragmentation from 3000 scaffolds upwards was unsuccessful in placing the genome in the correct cluster.

Finally, we also tested the effect of “incompleteness” on the robustness of AF and gANI values derived from a genome. Our objective here was to identify the extent to which a genome could be reduced with the MiSI method placing it in the correct species cluster. To test this, we used the same “finished and complete” genome sequence of *Lactococcus lactis cremoris A76* and randomly reduced 5% to 95% of the genome, in 5% increments and thus generated 19 artificial genomes having varied percentages of the complete genome sequence. For each of these artificial genomes, Prodigal was used to call genes and gANI and AF values of each artificial genome to all other genomes in our dataset were computed. The artificial genomes reduced by 5,10,15 and 20 percent displayed the required AF and gANI to all other members of clique #54 and to no other. The artificial genomes reduced by 25 percent had the required AF and gANI values to three genomes in clique #54 and thus would form a clique-group instead of a clique. The artificial genomes reduced by 30 percent or more did not pass the thresholds to any other genome in the dataset. This shows that the process of species assignment via the MiSI method remains unaffected through genome reduction by as much as 25%. Below this value, a relaxation of the AF threshold would be required to accurate place the reduced genome.

***Comparison of MiSI to other methods***

*Comparison to JSpecies****.*** The inability to compute ANI in JSpecies for a large set of genomes required selection of a subset of genomes to carry out this analysis. Fourteen strains of *Buchnera aphidicola* were used to explore the difference between gANI computed in MiSI and ANI computed using JSpecies. In this set, the gANI of six of these genomes to all other genomes of the same species is low (72-86) while the remaining eight genomes display significantly high gANI (~99) and high AF to each other. Thus the latter eight genomes form a clique while the former six genomes remain as singletons. For pairs within this set, we used the JSpecies software(5) to calculate ANI based on whole genome comparison, using two different methods: ANIm (using MUMmer)(6) and ANIb (using BLAST). Both these values were compared to the corresponding gANI of the pair as shown in Figure S6a.

As can be seen in the figure, the pairwise genome comparisons that we predict to have a gANI ~99, generate precisely the same values for ANIm and ANIb. As an ANI boundary of ~95-96 has been recommended for taxonomically circumscribing prokaryotic species, ANIm and ANIb concur that these genomes belong to the same species. As the compared genomes become more divergent, the values predicted by the three methods differ more significantly. In this low gANI boundary, the results we produce, are bounded by ANIm and ANIb. The most important observation remains, that when our method predicts a low gANI, which essentially lead to the separation of these genomes into singletons, the other two methods do not predict an ANI that is higher than 95%. While our method detects the higher extent of divergence between pairs of genomes, these other methods only detect high levels of similarity. Since all three methods concur on which pairwise comparison assigns two genomes as the same species and which does not, we conclude that the cluster structuring in MiSI is not negatively biased by our choice of CDS over whole genome.

*Comparison to SpecI***.** ANI based on phylogenetic marker genes (pMGs) was used to generate species clusters in the SpecI method(7). Although, pMG-based ANIs are typically higher than our gANI since they are more conserved at the sequence level, the same species-level ANI cut-off of 96.5 was identified using SpecI. The main difference between the method proposed by Bork et al(7) and our method using nsimscan(8), is the number of genes compared between each pair of genomes. While MiSI includes all the genes defined in a genome, SpecI first identifies and extracts the phylogenetic marker genes (pMG) present in the genomes and calculates the ANI using only these pMG’s. Thus, we compared the results of computing the ANI between two genomes, using just the pMG’s versus using all the genes of genome.

As the first step, we used the Perl script available at the SpecI download page (<http://vm-lux.embl.de/~mende/specI//downloads.html>): MOCATFetchMGs03.pl to identify and extract the pMG’s in each of the 13,151 genomes in our dataset. For the purpose of calculation of gANI, only the top hit to each of the 40 COG’s (representing the pMG’s) was retained. The distribution of the percent of pMG’s identified is shown in Figure S6b. Out of the 13,151 genomes, 8,669 genomes had one or more pMG’s present in the genome. The remaining 4482 genomes have at least one pMG but these are too divergent to be detected by the pMG models under regular cut-offs.

Among these genomes, we examined the case of *Lactobacillus reuteri*. In our dataset, we have total of 11 *L. reuteri* genomes distributed as such: seven genomes are in clique-group with intra-gANI of 97.45 and intra-AF of 0.75, two genomes are in a clique and the remaining two are singletons. SpecI, on the other hand, has a single cluster (cluster1457) with 8 *L. reuteri* genomes. Of the 11 *L. reuteri* genomes in our dataset, eight have 40 pMG’s, while the remaining three genomes have no pMG’s. Further investigation into this set of genomes revealed that the marker genes were present within the genomes, but were unable to be identified by the pMG identification tool implemented by specI. The nucleotide and protein sequences of all the genes of each of the genomes were extracted, and every single genome was run through the SpecI webserver. All the genomes that have 40 pMG's were assigned to the SpecI Lactobacillus cluster. The genomes that showed an absence of pMG’s was not assigned to any cluster and was further deemed to belong to a “novel” genus. MiSI on the other hand, was able to correctly identify that these genomes belong to a *Lactobacillus reuteri* clique-group. This further highlights another drawback of the SpecI tool, wherein, if a user has multiple genomes that are not in the SpecI database, or are not closely related to the genomes in SpecI database, SpecI does not provide the means to test if the input genomes themselves are closely related to one and another.

Next, we picked intra-species pairs, that included only genomes with complete classification and had one or more pMG’s, and for each pair the gANI was computed using the pMG’s identified in each genome of the pair. Upon comparing pMG-ANI and gANI of these 1.1M intra-species pairs, we found that 94.6% of intra-species pairs have ANI>=96.5 while 2.9% of intra-species pairs are found to have ANI<96.5 using both methods. The remaining 2.5% of pairs have pMG-ANI>=96.5 but gANI<96.5. These pairs reflect rapid speciation events highlighting divergent strains, which evade detection if only pMGs are considered. As expected, species clusters based on gANI display higher fragmentation than those based on pMG-ANI (Figure S6c).

We then benchmarked both the approaches across different sets of genome pairs that varied from intra-species pairs to inter-species pairs, with genome size varying from 1MB to 6MB. Since, genetic changes contributing to speciation are unlikely to be concentrated in conserved pMGs, utilizing all CDSs for ANI computation not only maximizes the inclusion of these genetic changes, but also comes at very little or no additional computational cost: the time taken to recognize and extract pMGs and compute ANI is comparable to the time taken for computing gANI for bacterial genomes smaller than 4.5M. For larger genomes, pMG-based ANI is only slightly faster (Table S4).

*Comparison between MiSI groupings and groupings based on 16S rDNA similarity.* This work also facilitates systematic comparisons between genome groups generated by the MiSI method and groupings based on 16S gene similarity. Since gANI and AF considers the entire genome, the MiSI distance between genomes are typically larger than 16S distances. With the exception of the rare cases of horizontally transferred of 16S genes(9–11), clustering using 16S distances is expected to yield fewer clusters than clustering using the MiSI method. Indeed, we found that 6.4% of genomes that have a defined 16s distance in IMG, that form cliques based on our AF and gANI cutoffs would group differently when grouped using 16S-based similarity. With the latter as the reference, the MiSI classification would generate some false negative species associations, but hardly any false-positives. With the MiSI similarity as the reference, a 16S-based classification would generate a sizeable portion of false-positive species associations and not many false negatives. Although the clustering in MiSI is stricter, it is a more consistent representation of evolution than the distance captured by a single or a set of marker gene since it accounts for both the nucleotide identity across the entire genome and the fraction of the genome that aligns.

***An empirical distribution to determine whether a pair of genomes belongs to the same species***

To understand the relationship between AF and gANI in intra-species vs. inter-species genome pairs, paired gANI and AF values were plotted for 86.5M pairs of genomes (Figure 4a). The resulting scatterplot displays distinct trends in three intervals of AF: [0,0.1), [0.1,0.6), [0.6,1]. As expected, the interval [0,0.1) boasts the highest concentration of data points (86.34%) with gANI and AF displaying no linear correlation. In the intervals [0.1,0.6) and [0.6,1], a very strong positive correlation is observed between AF and gANI values suggesting a strong dependence between the fraction of orthologous genes and the extent of their conservation. Therefore, AF and gANI are best modeled as dependent variables in a probabilistic modeling context with AF being modeled as an independent random variable and gANI being modeled as a random variable dependent on AF.

To determine whether two genomes with pairwise AF=a and gANI=b belong to the same species, empirical probabilities computed from domain wide data may be used (Dataset S3). The probability of AF=a being generated by an intra-species pair is independently computed. Then the empirical probability of gANI=b being generated by an intra-species pair is computed conditionally on the value of AF being a. In summary,

P_r_^intra-species^[AF=a,ANI=b]= P_r_^intra-species^ [AF=a]* P_r_^intra-species^ [ANI=b|AF=a] (1).

To determine how likely a pair with P_r_^intra-species^[AF=a,ANI=b]=x is to belong to the same species, Figure 4b may be used. It shows the ranges of final probabilities for “real” intra-species pairs when an AF cut-off of 0.6 is used. We observe that higher probabilities have greater likelihood of corresponding to intra-species pairs with the 1% false positive rate probability level being 0.6.

***Misidentification from the perspective of cliques***

Misidentifications can also be viewed from the perspective of cliques (Figure 2b, a subset of 278 cliques with multiple taxonomic species). Species inhabiting such multi-taxonomic cliques include three cliques populated by multiple named species of *Rickettsia*; four cliques inhabited by multiple named species of *Mycobacterium*; three cliques populated by multiple named species of *Thermoanaerobacter* and one clique inhabited by 9 named species of *Brucella*. *Burkholderia*, *Streptomyces*, *Desulfurococcus*, *Salinispora*, *Shigella*, *Streptococcus*, *Xanthomonas*, *Lactobacillus*, *Pseudomonas* are other genera that are represented in the multi-taxonomic cliques.

***Identification of putative novel species***

Cliques that are populated entirely by genomes without any species definitions are surmised to be novel species. Clustering at AF>=0.6 and gANI>=96.5 generates 109 such cliques (Table S3). *Thioalkalivibrio* genomes dominate these cliques with 56 genomes present in 10/109 cliques. Other unclassified cliques are dominated by taxonomic species of *Pseudomonas*, *Streptomyces*, and *Butyrivibrio*. Some of these cliques are connected with other cliques or singletons with gANI>=95 but below 96.5 and high inter AF, making it possible to find species closely-related to these undefined groups. For example, clique 961, which is made up of two *Ralstonia sp.,* exhibits average inter-gANI of 95.6 and inter-AF of 0.77 with a *Ralstonia pickettii* singleton, suggesting that the genomes in these cliques are close to *Ralstonia pickettii*.

**Reference:**

1. Vilas-Boas,G., Sanchis,V., Lereclus,D., Lemos,M.V.F. and Bourguet,D. (2002) Genetic differentiation between sympatric populations of Bacillus cereus and Bacillus thuringiensis. *Appl. Environ. Microbiol.*, **68**, 1414–1424.

2. Vilas-Bôas,G.T., Peruca,A.P.S. and Arantes,O.M.N. (2007) Biology and taxonomy of Bacillus cereus , Bacillus anthracis , and Bacillus thuringiensis. *Can. J. Microbiol.*, **53**, 673–687.

3. Denapaite,D., Brückner,R., Nuhn,M., Reichmann,P., Henrich,B., Maurer,P., Schähle,Y., Selbmann,P., Zimmermann,W., Wambutt,R., *et al.* (2010) The Genome of Streptococcus mitis B6 - What Is a Commensal? *PLoS ONE*, **5**, e9426.

4. Hyatt,D., Chen,G.-L., LoCascio,P.F., Land,M.L., Larimer,F.W. and Hauser,L.J. (2010) Prodigal: prokaryotic gene recognition and translation initiation site identification. *BMC Bioinformatics*, **11**, 119.

5. Richter,M. and Rosselló-Móra,R. (2009) Shifting the genomic gold standard for the prokaryotic species definition. *Proc. Natl. Acad. Sci.*, 10.1073/pnas.0906412106.

6. Kurtz,S., Phillippy,A., Delcher,A.L., Smoot,M., Shumway,M., Antonescu,C. and Salzberg,S.L. (2004) Versatile and open software for comparing large genomes. *Genome Biol.*, **5**, R12.

7. Mende,D.R., Sunagawa,S., Zeller,G. and Bork,P. (2013) Accurate and universal delineation of prokaryotic species. *Nat. Methods*, **10**, 881–884.

8. Kaznadzey,D. QSimScan (Quick SIMilarity SCANner).

9. Wright,A.-D.G. (2006) Phylogenetic relationships within the order Halobacteriales inferred from 16S rRNA gene sequences. *Int. J. Syst. Evol. Microbiol.*, **56**, 1223–1227.

10. Jain,R., Rivera,M.C. and Lake,J.A. (1999) Horizontal gene transfer among genomes: the complexity hypothesis. *Proc. Natl. Acad. Sci. U. S. A.*, **96**, 3801–3806.

11. Wagner,A. and de la Chaux,N. (2008) Distant horizontal gene transfer is rare for multiple families of prokaryotic insertion sequences. *Mol. Genet. Genomics MGG*, **280**, 397–408.

**Supplementary Figures:**


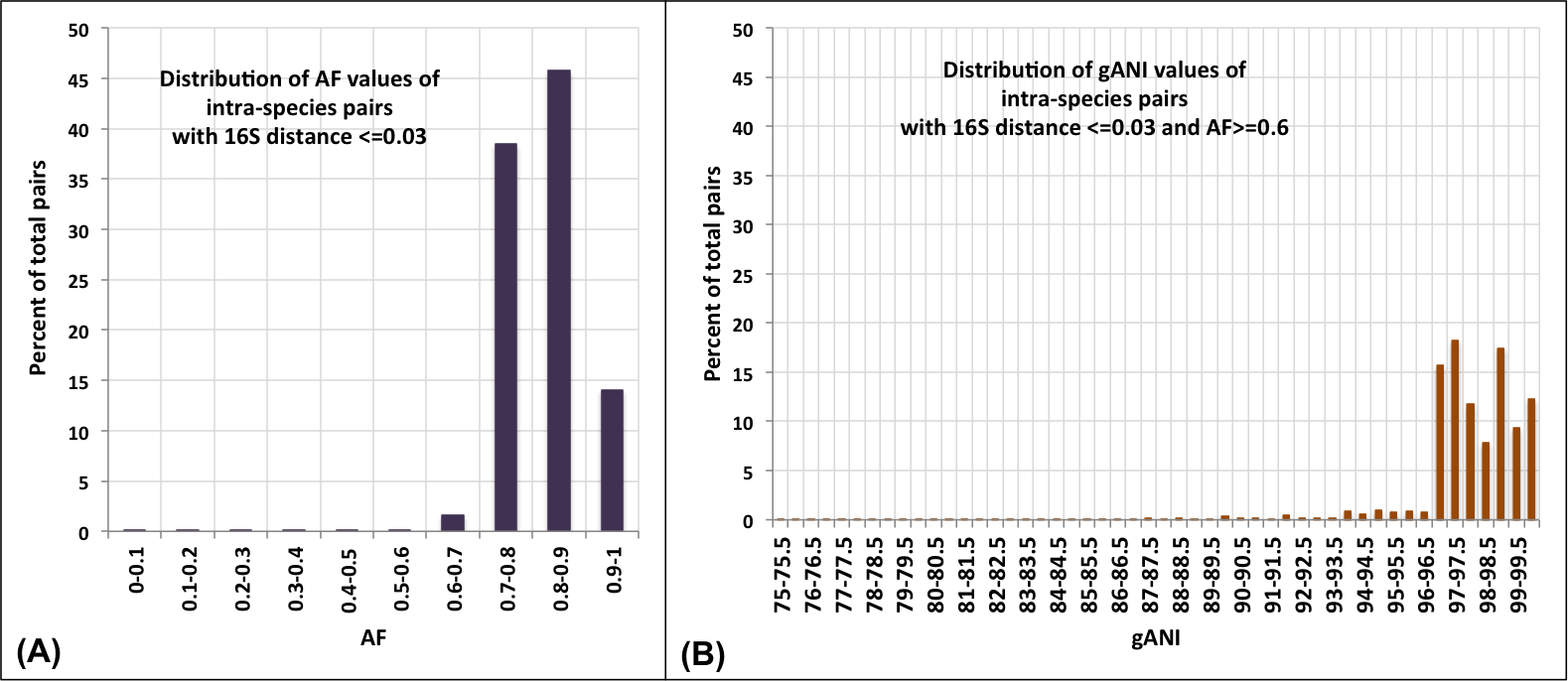


**Figure S1: (A) Distribution of AF values of intra-species pairs that have a 16s distance less than 0.03 (corresponds to 97% identity) (B) Distribution of the gANI values of those intra-species pairs that have a 16s distance less than 0.03 and an AF greater than 0.6**. The identification of the pairs used in each plot has been described in Methods. The plots serve as a validation of the cutoffs identified since the same thresholds are identified using those intra-species pairs that have the required 16s distance to each other.


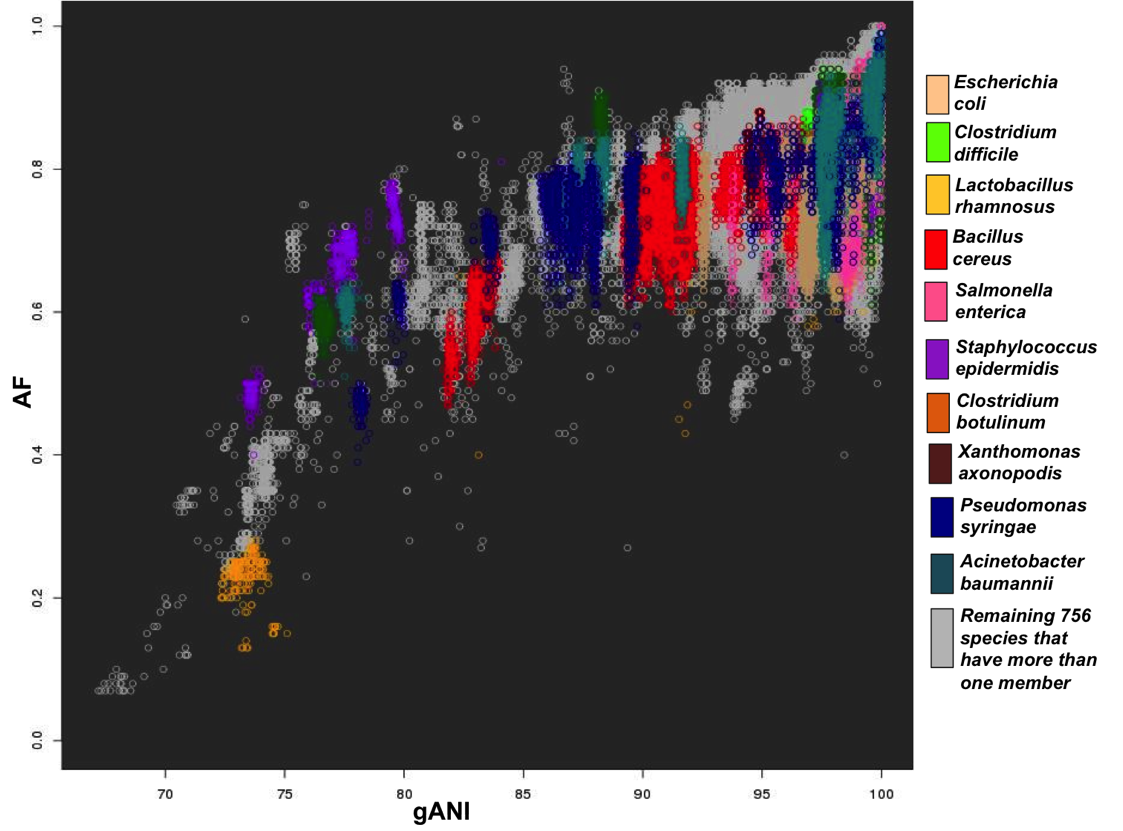


**Figure S2: AF vs gANI values of ~1.3 million intra-species pairs.** The points associated with 10 species, i.e. all the intra-species pairs of 10 species have been given a separate color as specified in the legend to the right. All the intra-species points associated with the remaining species are colored gray.


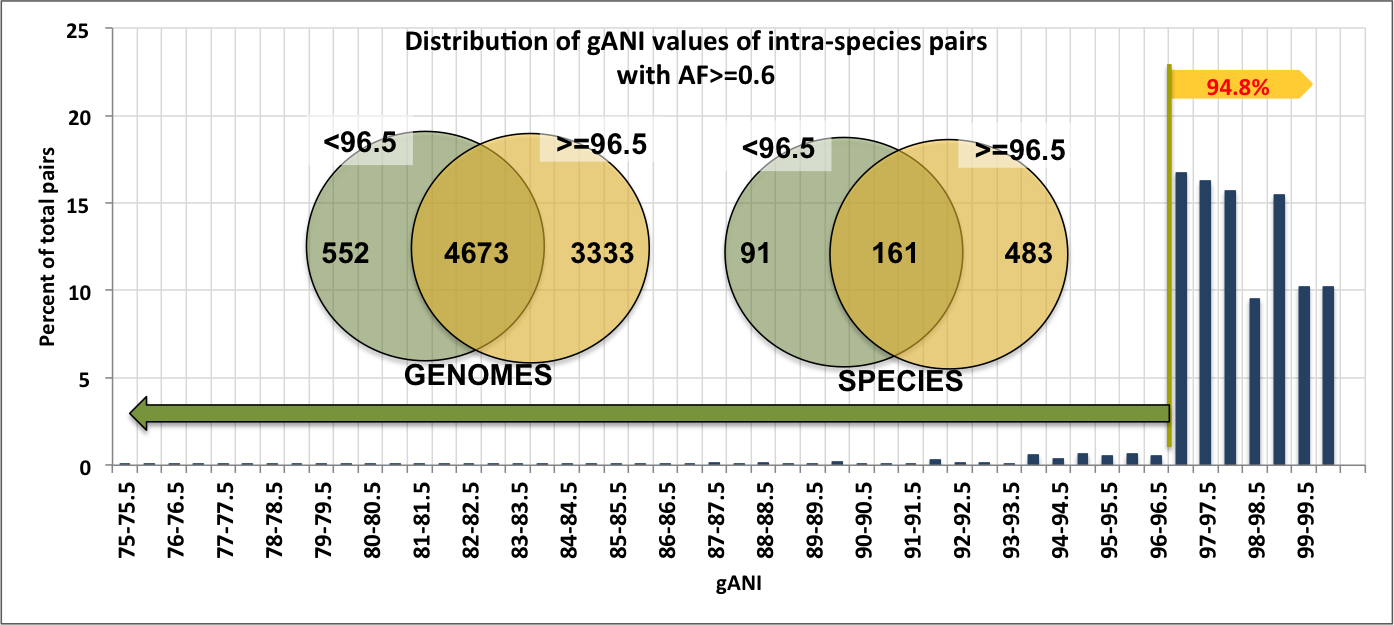


**Figure S3: Analysis of genomes and species that contribute to the tail.** The distribution of gANI values of intra-species pairs that have an AF>=0.6 are plotted in dark blue, where the Y axis shows the percent of total pairs seen in each bin of gANI. The percent of total pairs that have gANI>=96.5 are shown on the yellow flag. The number of genomes that only contribute to pairs that have a gANI>=96.5 is shown in the yellow circle in the left Venn diagram, the number of genomes that only contribute to pairs that have a gANI<96.5 is shown in the green circle, while the number of genomes that contribute to pairs with gANI both above and below the threshold is shown in the overlap. The same analysis was carried out for named species and the numbers for these are annotated in the Venn diagram to the right.


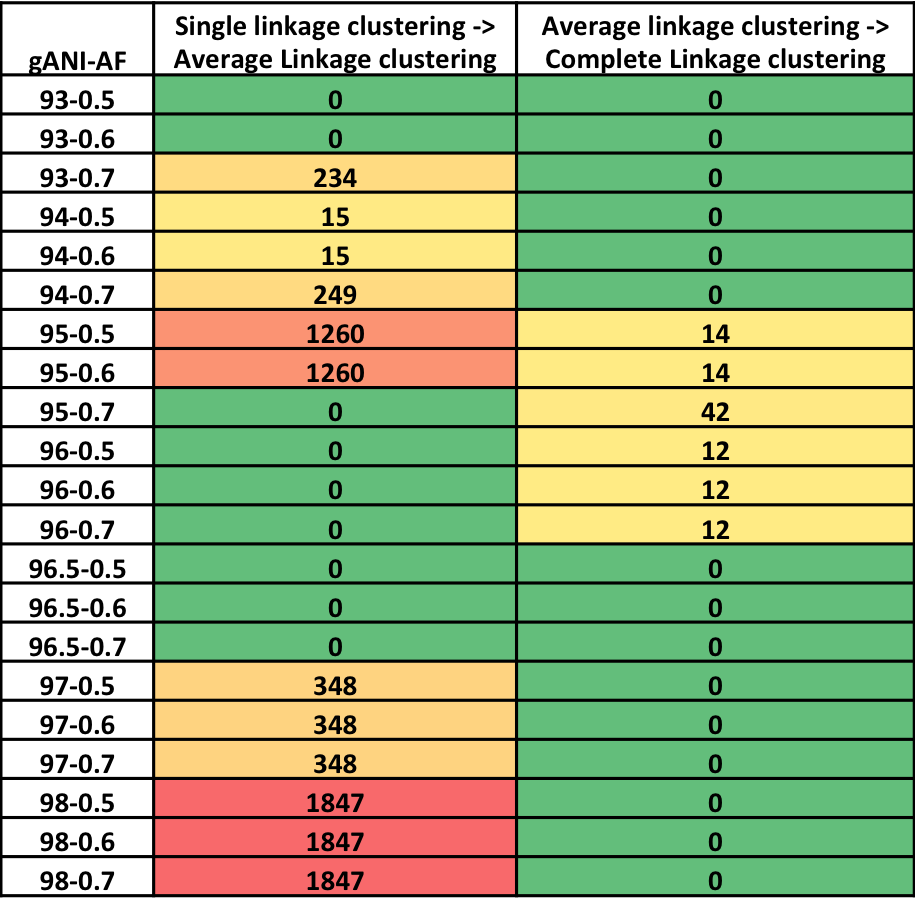


**Figure S4: Effect of using different clustering approaches.** The table shows the comparison of the clusters formed using single-linkage, average-linkage and complete-linkage (MCE) at different AF and gANI combinations. Each row is a combination, and each column shows the number of differences between the genome-linkage matrix generated using the clusters formed between the two clustering methods.


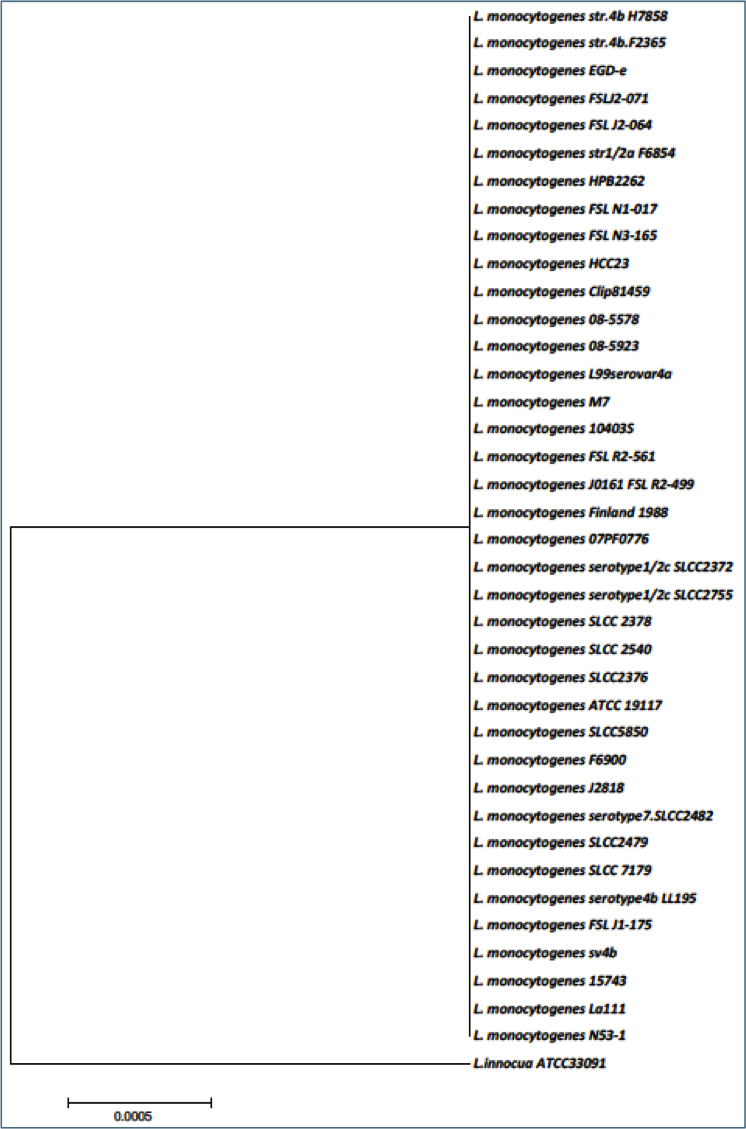


**Figure S5: 16S neighbor joining tree of *Listeria monocytogenes* using *L. innocua* as an outgroup.**


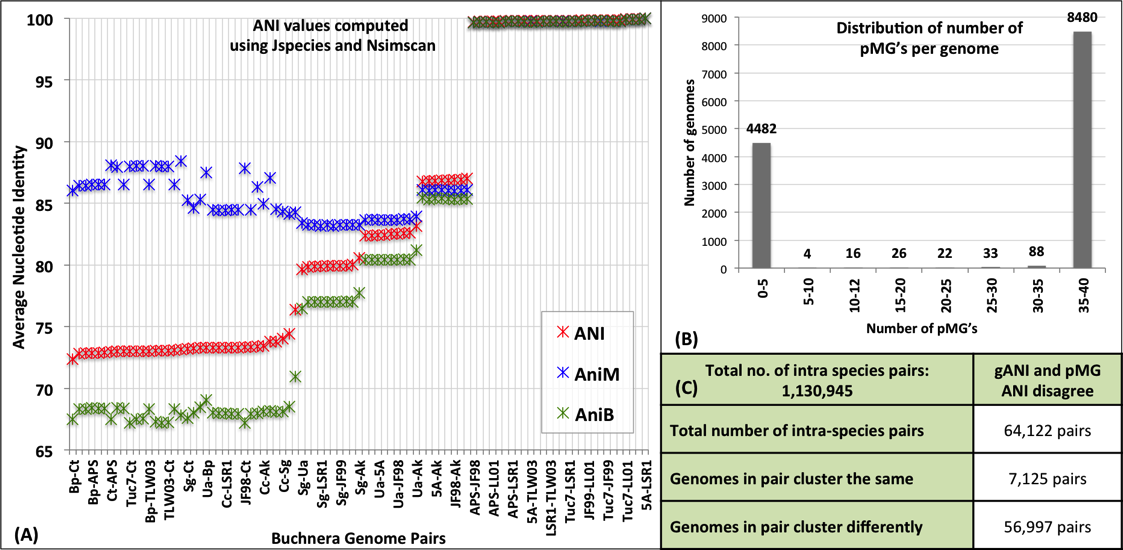


**Figure S6: Comparison with other methods (A) Difference between ANI values computed using Jspecies Blast/Mummer in Jspecies and our approach using NSimScan.** Fourteen strains of *Buchnera aphidicola* were used to explore the difference between gANI computed using MiSI and ANI computed using JSpecies. The X-axis plots each genome pairs and shows the strains of the genomes that contribute to that pair. The Y-axis plots the ANI observed. Each “x” marks the ANI value observed for that pair, with green indicating the AniB value (ANI value using BLAST), blue indicating the AniM value (ANI value using MUMmer) and red indicating the gANI value computed using MiSI. **(B) Distribution of the number of pMG’s identified using specI’s tool in 13,151 genomes.** The number of pMG’s identified is shown on the X axis in bins, while the Y axis shows the number of genomes that have the number of pMG’s in each bin. **(C)** Breakdown based on species-delineating gANI threshold to determine the differences between using pMG-based ANI or gANI towards the formation of clique, clique groups and singletons.


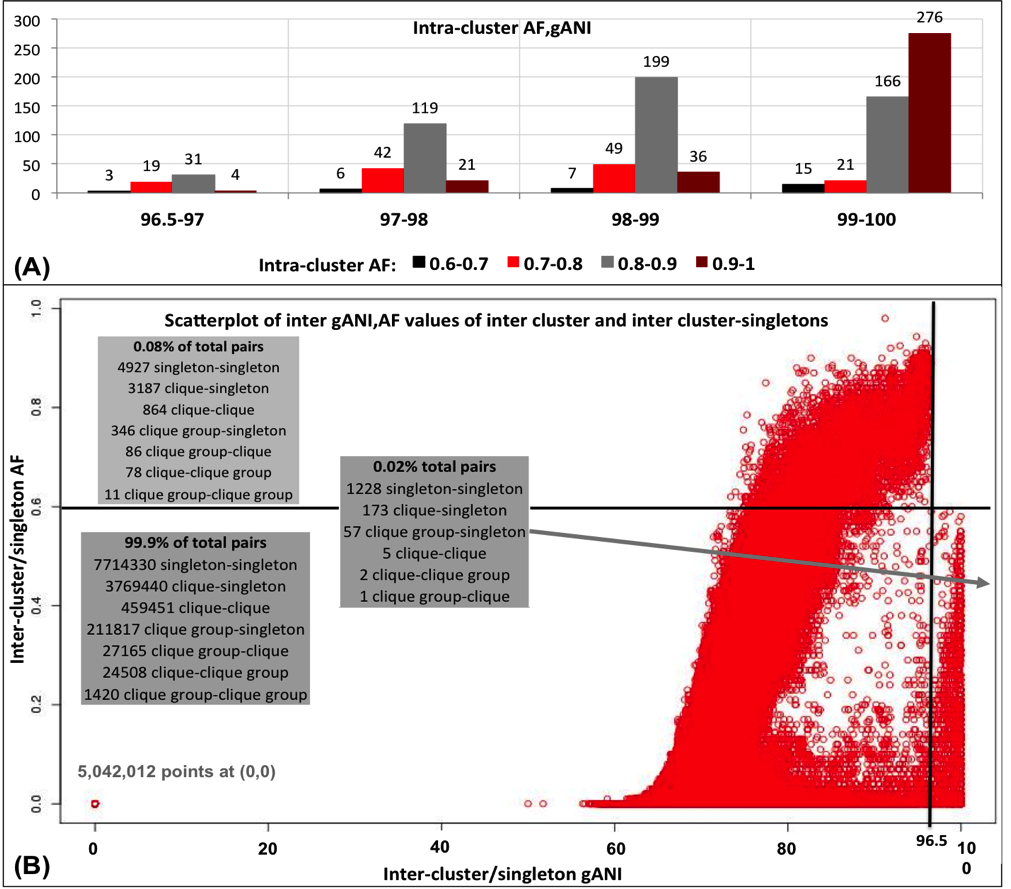


**Figure S7: Intra-cluster and inter-cluster/singleton AF,gANI values**. **(A)** The graph shows the distribution of gANI values and the corresponding AF values divided into fixed size bins. No intra-clique AF value below 0.6 or gANI value below 96.5 was observed. Each AF bin is assigned a different color. **(B)** The graph plots the observed inter-cluster/singleton gANI values on the X-axis and the corresponding inter-cluster/singleton AF values on the Y-axis. The thresholds of gANI 96.5 and AF 0.6 have been marked with a black line dividing the graph into four quadrants. The percent of total pairs in each quadrant is annotated in the grey boxes, along with a breakdown of the how many types of combinations are seen in that quadrant.


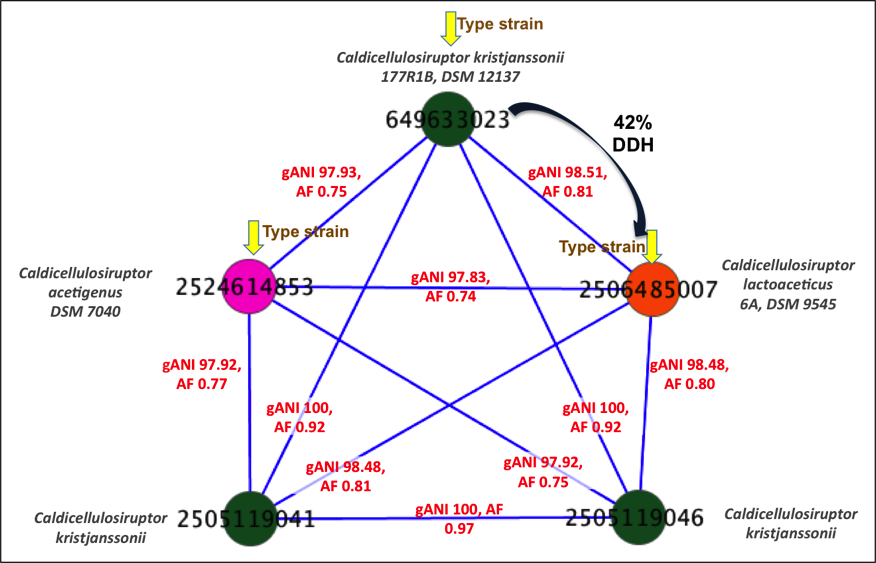


**Figure S8: Pictorial depiction of clique 757 containing 5 genomes with 3 type strains that represent three different species.** Each circle represents a genome present in clique 757, while each blue line depicts the presence of an edge between two genomes if those two genomes have gANI>=96.5 and AF>=0.6. The circles are colored based on species, where green represents *Caldicellulosiruptor kristjanssonii*, pink represents *Caldicellulosiruptor acetigenus* and orange represents *Caldicellulosiruptor lactoaceticu*s. Type strains are shown using the yellow arrows. The DDH values between the type strains have been annotated.


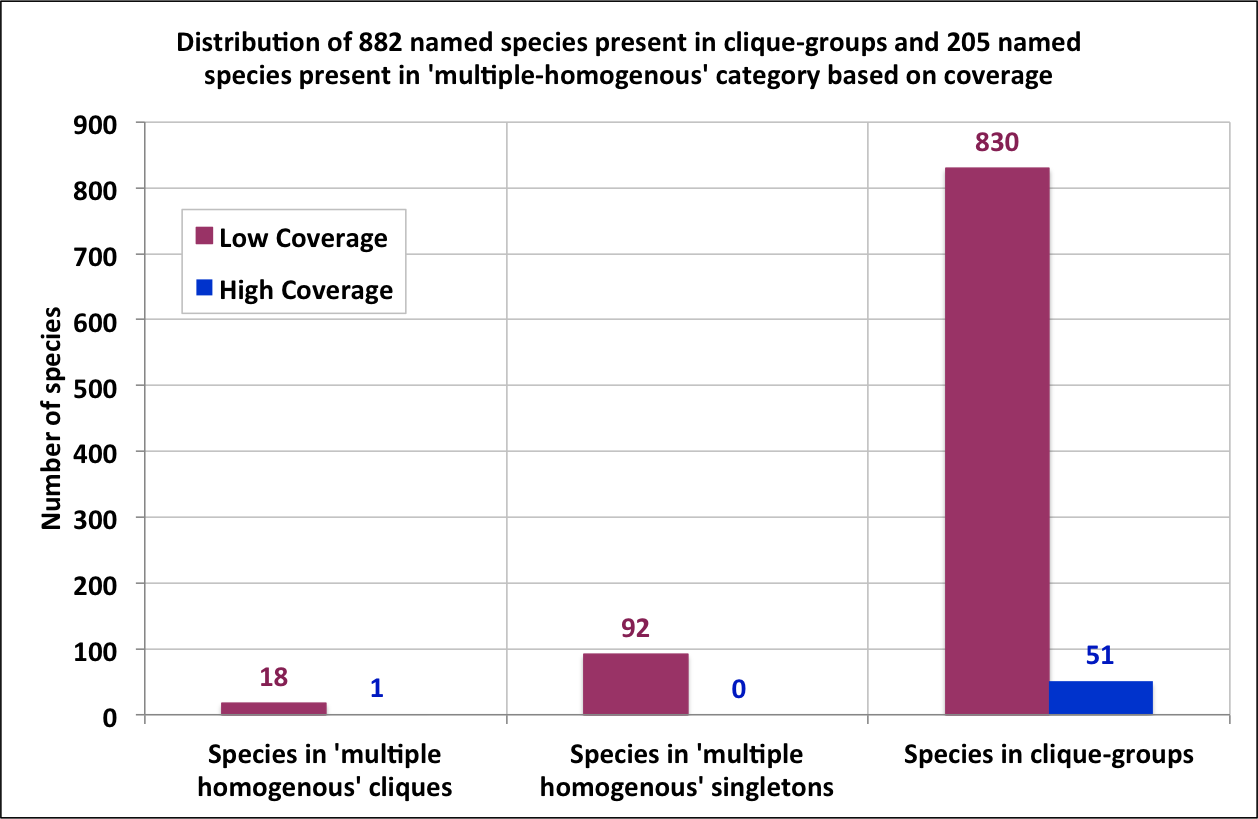


**Figure S9:** **Distribution of 882 named species present in clique-groups and 205 named species present in ‘multiple-homogeneous’ category based on coverage**. Coverage of a species is defined as the number of genomes of that species that is present within our dataset. Low coverage indicates that the species has less than 20 genome representatives in our dataset, while high coverage indicates that the species has more than or equal to 20 genome representatives in our dataset.


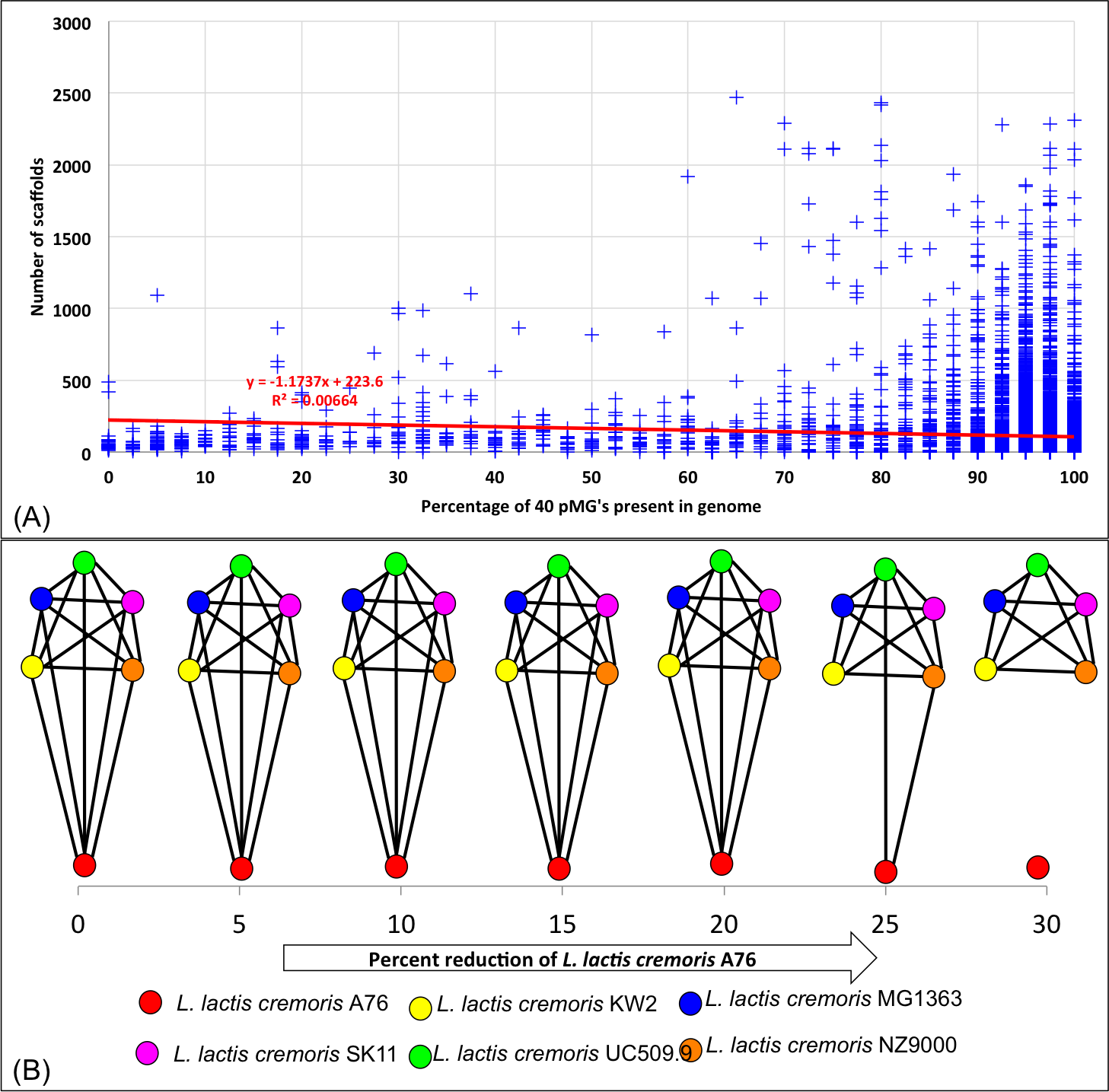


**Figure S10: Robustness of the MiSI method against incomplete genomes. (A) Scatterplot of completeness of each genome in our dataset versus its corresponding number of scaffolds.**  The plot shows the percentage of 40 pMG’s for each of the 13,151 genomes on the X-axis and the number of scaffolds that genome in present in on the Y-axis. Every plus on the graph represents a single genome. The percentage was computed by determining how many of the 40 COG’s of the pMG’s were present in the genome. **(B) Pictorial description of clustering results *of L.lactis cremoris* A76 genome over a series of genome reductions**. Each circle represents a genome with the color of the circle corresponding to the strain. A black line between two circles represents an edge that indicates that the two genomes have AF and gANI values above our thresholds. Each point on the X-axis corresponds to a percent reduction in the genome sequence of *L.lactis cremoris* A76 and the graph above the point describes how that reduced genome connects to the rest of the genomes in our dataset.


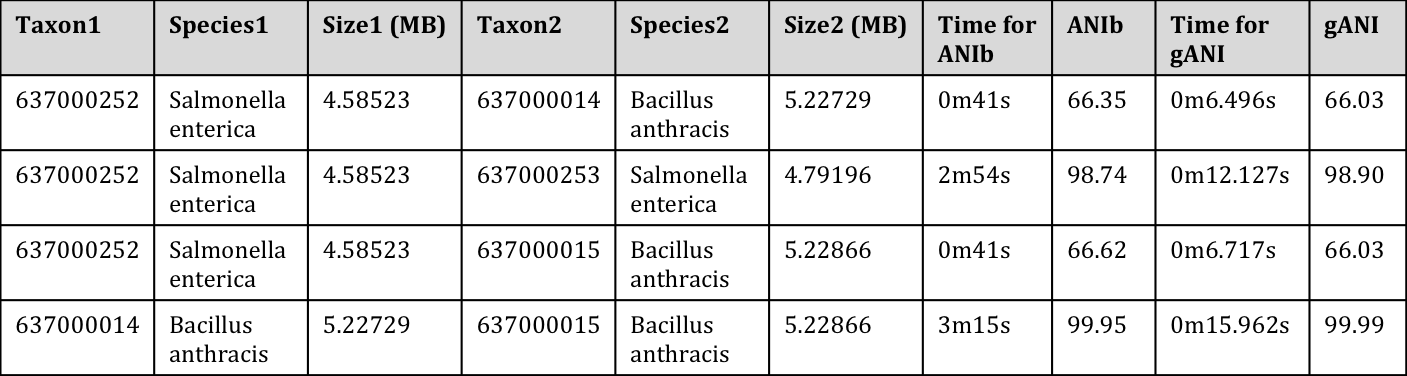


**Table S1**: **Results on comparison of time taken to compute ANI using two different approaches**. In the first approach, we synthesized four artificial genome sequence made of concatenated 1020 nucleotide fragments that are overlapping in the original genome sequence using a 500bp sliding window. This was used as input to Jspecies to calculate ANIb (ANI using BLAST). The time taken to do so is compared to calculation of gANI using our approach.


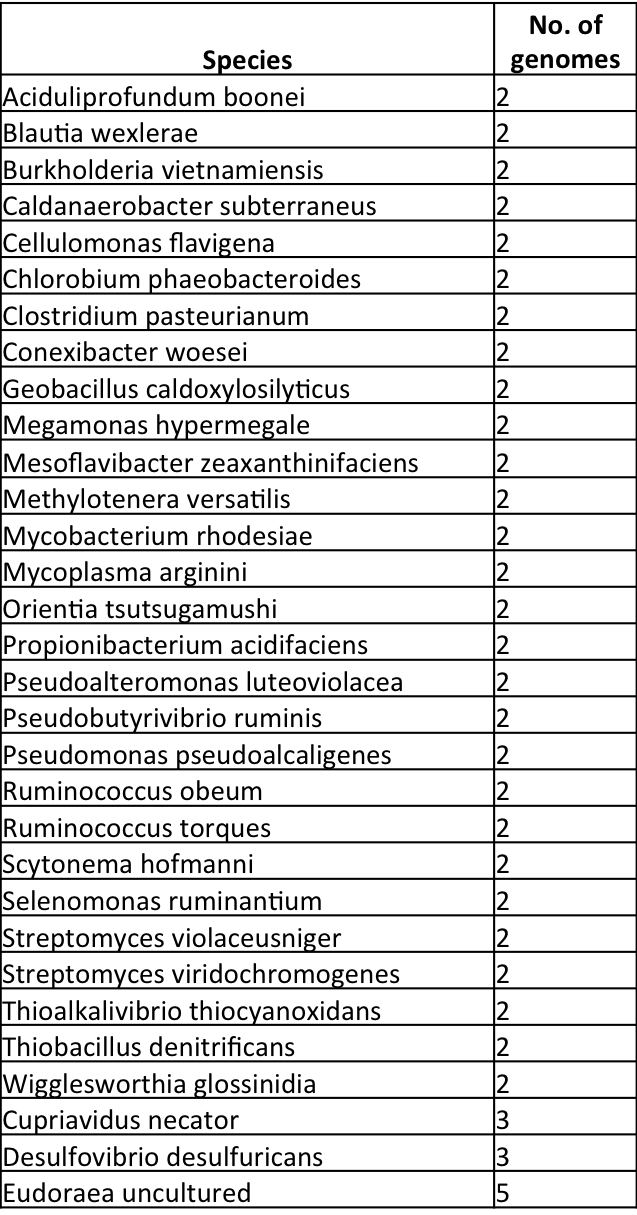


**Table S2:** **The 31 species that is lost as a result of the AF filter.**


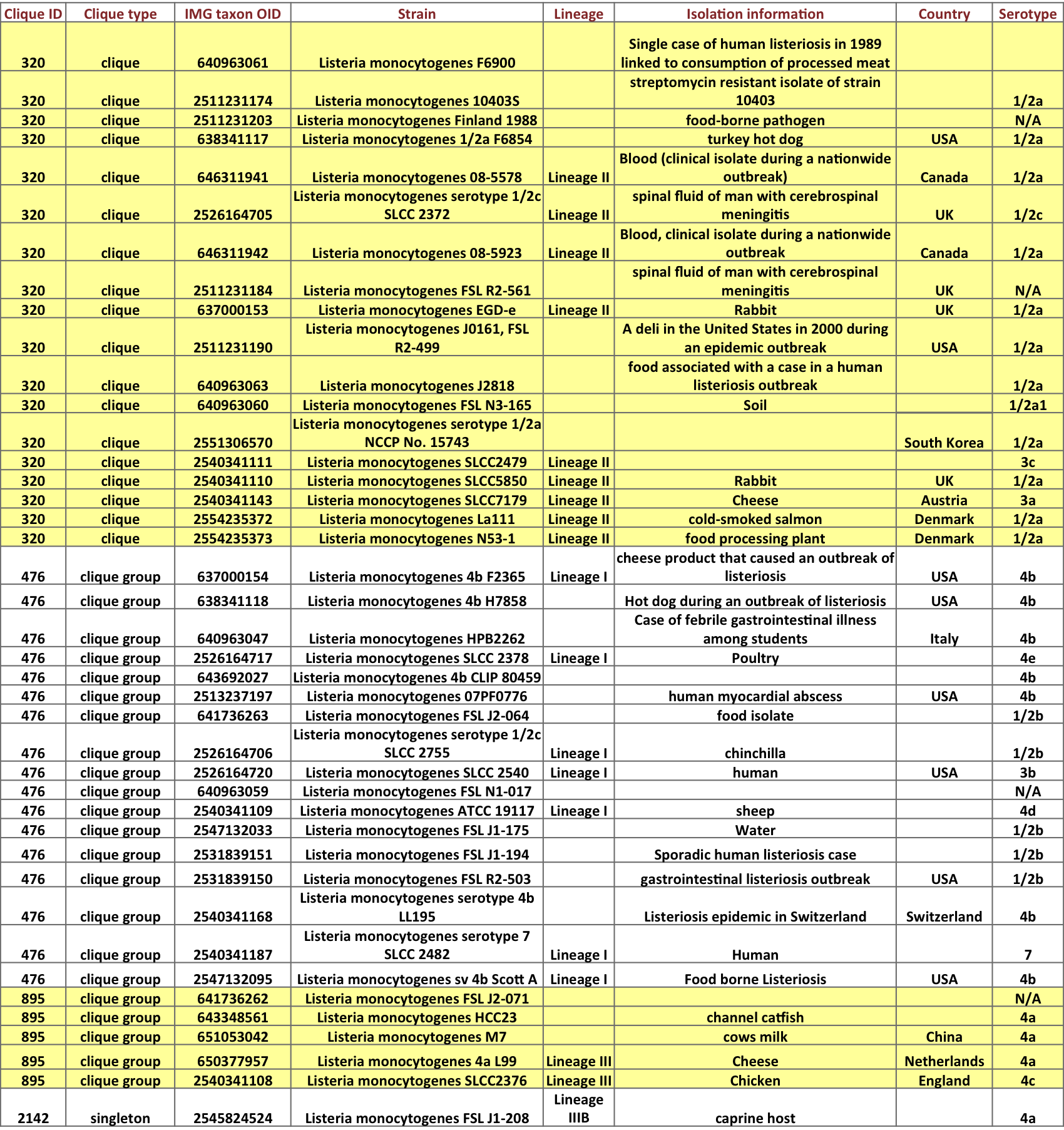


**Table S3: Membership of the individual clique and clique groups and singleton formed by the *Listeria monocytogenes* strains included within our analysis, showing the correspondence to previously identified lineages.**


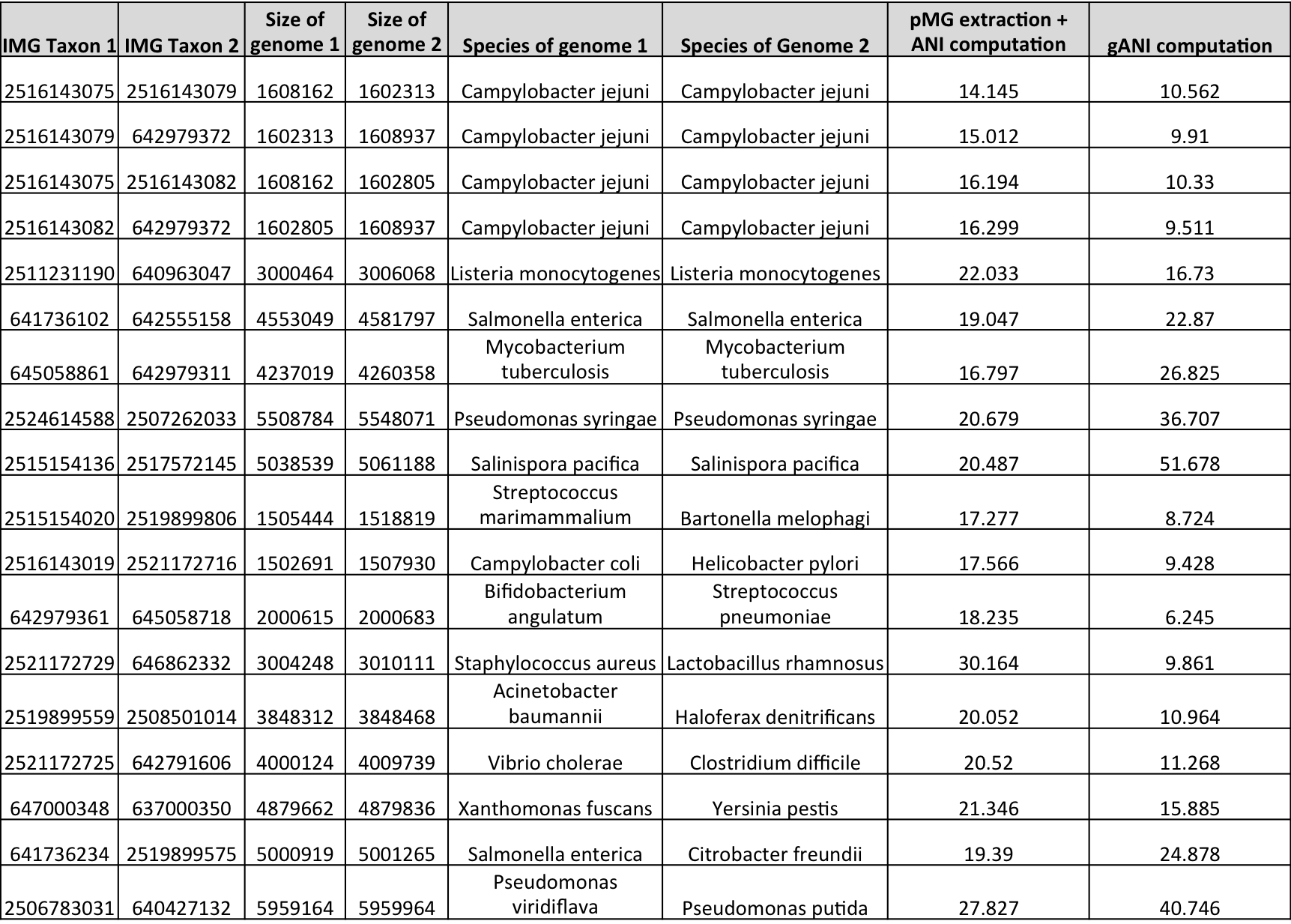


**Table S4: Comparison of time taken to compute pMG-based ANI and gANI between pairs of similar sized genomes.** In this table, the size is specified in bases, while the last two columns show the real time in seconds taken by each of the approaches. The seventh column in this table is an addition of the time taken to identify the pMG’s using the SpecI’s Perl script, with the time taken to compute gANI using only the pMG’s extracted.

**Supplementary Datasets:**

**Dataset S1**: 326 genomes (highlighted in red) with undefined species (sp. or unclassified) can be assigned species definitions. (External Excel File)

**Dataset S2:** The 109 cliques and 4 clique groups that are populated entirely by genomes without any species definitions are putative novel species. (External Excel File)

**Dataset S3:** This table provides the ability to determine whether two genomes with pairwise AF=a and gANI=b belong to the same species and has been computed using empirical probabilities from domain wide data implemented in the aforementioned formula. (External Excel File)
