# Supplementary material for: Microbial species delineation using whole genome sequences
Source: Nucleic Acids Res. 2015 Jul 6;43(14):6761–71. doi: 10.1093/nar/gkv657 (PMC4538840; doi:10.1093/nar/gkv657)
Supplement: SUPPLEMENTARY DATA [file supp_gkv657_nar-03569-n-2014-File007.pdf]

Dataset S1: The 326 genomes(higlighted in red) with incomplete classification (sp. Or unclassified) that can be assigned to species based on their clique membership.

Cluster ID	Cluster Type	IMG Taxon ID	Species	Taxon Display Name
3	clique	2547132273	Escherichia sp.	Escherichia sp. TW15838
3	clique	2547132275	Escherichia sp.	Escherichia sp. TW10509
3	clique	2534681675	Escherichia coli	Escherichia coli O2:H4 1.2741
3	clique	2534682161	Escherichia coli	Escherichia coli STEC_7v
4	clique	648276711	Peptoniphilus sp.	Peptoniphilus sp. F0141
4	clique	647000289	Peptoniphilus lacrimalis	Peptoniphilus lacrimalis 315-B
4	clique	2519899691	Peptoniphilus lacrimalis	Peptoniphilus lacrimalis DSM 7455
12	clique	2541047518	Clostridium sp.	Clostridium bifermentans ATCC 638
12	clique	2541047517	Clostridium bifermentans	Clostridium bifermentans ATCC 19299
13	clique	649633021	Burkholderia sp.	Burkholderia sp. CCGE1001
13	clique	2526164713	Burkholderia sp.	Burkholderia sp. JPY366
13	clique	2518645580	Burkholderia phenoliruptrix	Burkholderia phenoliruptrix BR3459
13	clique	2554235175	Burkholderia phenoliruptrix	Burkholderia phenoliruptrix AC1100
16	clique	2541046973	Bacteroidetes sp.	Bacteroidetes sp. F0290
16	clique	2556793004	Phocaeicola abscessus	Phocaeicola abscessus CCUG 55929
20	clique	2531839610	Thermotoga sp.	Thermotoga sp. EMP
20	clique	637000321	Thermotoga maritima	Thermotoga maritima MSB8
20	clique	2519899531	Thermotoga maritima	Thermotoga maritima MSB8, DSM 3109
20	clique	2554235387	Thermotoga maritima	Thermotoga maritima MSB8
32	clique	2551306100	Bacillus sp.	Bacillus sp. CPSM8
32	clique	2554235236	Bacillus sp.	Bacillus sp. SB47
32	clique	2551306715	Bacillus licheniformis	Bacillus licheniformis 9945A
41	clique	649989958	unclassified	Lachnospiraceae bacterium sp. 8_1_57FAA
41	clique	651324046	unclassified	Lachnospiraceae bacterium 1_1_57FAA
41	clique	651324050	unclassified	Lachnospiraceae bacterium 3_1_46FAA
41	clique	640963025	Ruminococcus torques	Ruminococcus torques ATCC 27756
45	clique	2551306542	unclassified	Herbaspirillum sp. B65
45	clique	2551306545	unclassified	Herbaspirillum sp. B501
45	clique	2551306410	Herbaspirillum seropedicae	Herbaspirillum seropedicae Os45
45	clique	2551306416	Herbaspirillum seropedicae	Herbaspirillum seropedicae Os34
46	clique	646206273	Bacteroides sp.	Bacteroides sp. 3_2_5
46	clique	647533110	Bacteroides sp.	Bacteroides sp. 2_1_16
46	clique	651324014	Bacteroides sp.	Bacteroides sp. 2_1_56FAA
46	clique	637000024	Bacteroides fragilis	Bacteroides fragilis NCTC 9343
46	clique	637000025	Bacteroides fragilis	Bacteroides fragilis YCH46
46	clique	650377910	Bacteroides fragilis	Bacteroides fragilis 638R
46	clique	2531839304	Bacteroides fragilis	Bacteroides fragilis CL03T12C07
46	clique	2531839305	Bacteroides fragilis	Bacteroides fragilis CL07T00C01
46	clique	2531839306	Bacteroides fragilis	Bacteroides fragilis CL07T12C05
46	clique	2534681944	Bacteroides fragilis	Bacteroides fragilis HMW 615
46	clique	2537561570	Bacteroides fragilis	Bacteroides fragilis CL05T12C13
46	clique	2537562066	Bacteroides fragilis	Bacteroides fragilis CL03T00C08
47	clique	2554235436	Candidatus Portiera sp.	Candidatus Portiera aleyrodidarum BT-B-HRs
47	clique	2517093038	Candidatus Portiera aleyrodidarum	Candidatus Portiera aleyrodidarum BT-B
47	clique	2518645538	Candidatus Portiera aleyrodidarum	Candidatus Portiera aleyrodidarum BT-Q-AWRs
47	clique	2522572174	Candidatus Portiera aleyrodidarum	Candidatus Portiera aleyrodidarum BT-QVLC
57	clique	2548877023	Chlorogloeopsis sp.	Chlorogloeopsis sp. PCC 9212
57	clique	2509601037	Chlorogloeopsis fritschii	Chlorogloeopsis fritschii PCC 6912 (CLC Draft 2)
57	clique	2551306142	Chlorogloeopsis fritschii	Chlorogloeopsis fritschii PCC 6912
66	clique	2551306396	Paenibacillus sp.	Paenibacillus sp. ICGEB2008
66	clique	2547132099	Paenibacillus polymyxa	Paenibacillus polymyxa N.R. Smith 1105, ATCC 842
66	clique	2548876929	Paenibacillus polymyxa	Paenibacillus polymyxa OSY-DF
67	clique	2510065002	Arsenophonus sp.	Arsenophonus sp. ArN
67	clique	2524614872	Arsenophonus nasoniae	Arsenophonus nasoniae DSM 15247
68	clique	2554235432	Haemophilus sp.	Haemophilus parasuis ZJ0906
68	clique	641736156	Haemophilus parasuis	Haemophilus parasuis 29755
68	clique	643348556	Haemophilus parasuis	Haemophilus parasuis SH0165
68	clique	2541047150	Haemophilus parasuis	Haemophilus parasuis gx033
70	clique	2545824642	Lactobacillus sp.	Lactobacillus rhamnosus LOCK900
70	clique	2554235437	Lactobacillus sp.	Lactobacillus rhamnosus LOCK908
70	clique	642979366	Lactobacillus rhamnosus	Lactobacillus rhamnosus HN001
70	clique	643886127	Lactobacillus rhamnosus	Lactobacillus rhamnosus LMS2-1
70	clique	644736382	Lactobacillus rhamnosus	Lactobacillus rhamnosus GG
70	clique	644736383	Lactobacillus rhamnosus	Lactobacillus rhamnosus Lc 705
70	clique	646862332	Lactobacillus rhamnosus	Lactobacillus rhamnosus GG, ATCC 53103
70	clique	2511231185	Lactobacillus rhamnosus	Lactobacillus rhamnosus K, ATCC 8530
70	clique	2519899592	Lactobacillus rhamnosus	Lactobacillus rhamnosus LRHMDP3
70	clique	2519899593	Lactobacillus rhamnosus	Lactobacillus rhamnosus LRHMDP2
70	clique	2529293053	Lactobacillus rhamnosus	Lactobacillus rhamnosus R0011
70	clique	2534681855	Lactobacillus rhamnosus	Lactobacillus rhamnosus ATCC 21052
70	clique	2554235212	Lactobacillus rhamnosus	Lactobacillus rhamnosus CRL1505
71	clique	2509276012	Bacillus sp.	Bacillus sp. J13
71	clique	2506783046	Paenibacillus lactis	Paenibacillus lactis 154
72	clique	647533209	Ruegeria sp.	Ruegeria sp. TrichCH4B
72	clique	2504756012	Ruegeria mobilis	Ruegeria mobilis 45A6
72	clique	2534682167	Ruegeria mobilis	Ruegeria mobilis F1926
74	clique	2541047020	Microbacterium sp.	Microbacterium sp. oral taxon 186 F0373
74	clique	2531839539	Microbacterium laevaniformans	Microbacterium laevaniformans OR221
75	clique	2545824768	Lactobacillus sp.	Lactobacillus plantarum IPLA88
75	clique	637000141	Lactobacillus plantarum	Lactobacillus plantarum WCFS1
75	clique	643886053	Lactobacillus plantarum	Lactobacillus plantarum ATCC 14917
75	clique	644736381	Lactobacillus plantarum	Lactobacillus plantarum JDM1

75	clique	648028037	Lactobacillus plantarum	Lactobacillus plantarum plantarum ST-III
75	clique	2505119027	Lactobacillus plantarum	Lactobacillus plantarum VBLLa 11-47
75	clique	2521172735	Lactobacillus plantarum	Lactobacillus plantarum plantarum NC8
75	clique	2540341093	Lactobacillus plantarum	Lactobacillus plantarum ZJ316
75	clique	2554235316	Lactobacillus plantarum	Lactobacillus plantarum plantarum P8
98	clique	2531839270	Klebsiella sp.	Klebsiella sp. OBRC7
98	clique	2511231124	Klebsiella oxytoca	Klebsiella oxytoca KCTC 1686
98	clique	2518645552	Klebsiella oxytoca	Klebsiella oxytoca E718
98	clique	2531839401	Klebsiella oxytoca	Klebsiella oxytoca 10-5242
100	clique	2540341040	Pseudoalteromonas sp.	Pseudoalteromonas sp. BSi20429
100	clique	2541047156	Pseudoalteromonas arctica	Pseudoalteromonas arctica A 37-1-2
108	clique	651324053	unclassified	Lachnospiraceae bacterium 5_1_57FAA
108	clique	641736197	Clostridium scindens	Clostridium scindens ATCC 35704
110	clique	2529293266	Bacteroides sp.	Bacteroides sp. HPS0048
110	clique	2537561664	Bacteroides nordii	Bacteroides nordii CL02T12C05
113	clique	2537561888	Eubacterium sp.	Eubacterium sp. AS15
113	clique	648276673	Eubacterium yurii	Eubacterium yurii margaretiae ATCC 43715
115	clique	643692015	Chloroflexus sp.	Chloroflexus sp. Y-400-fl
115	clique	641228485	Chloroflexus aurantiacus	Chloroflexus aurantiacus J-10-fl
120	clique	649633088	Rahnella sp.	Rahnella sp. Y9602
120	clique	2513237194	Rahnella aquatilis	Rahnella aquatilis HX2
121	clique	651324054	unclassified	Lachnospiraceae bacterium 6_1_63FAA
121	clique	643886146	Blautia hansenii	Blautia hansenii VPI C7-24, DSM 20583
130	clique	651324019	Capnocytophaga sp.	Capnocytophaga sp. oral taxon 329 F0087
130	clique	2513237390	Paraprevotella clara	Paraprevotella clara YIT 11840
133	clique	2511231094	Pseudogulbenkiania sp.	Pseudogulbenkiania sp. NH8B
133	clique	643886068	Pseudogulbenkiania ferrooxidans	Pseudogulbenkiania ferrooxidans 2002
136	clique	647000294	Prevotella sp.	Prevotella sp. F0295
136	clique	2518645604	Prevotella loescheii	Prevotella loescheii DSM 19665
137	clique	649989930	Eggerthella sp.	Eggerthella sp. 1_3_56FAA
137	clique	651324028	Eggerthella sp.	Eggerthella sp. HGA1
137	clique	644736358	Eggerthella lenta	Eggerthella lenta VPI 0255, DSM 2243
140	clique	2513237299	Synergistes sp.	Synergistes sp. 3_1_syn1
140	clique	2510065052	Cloacibacillus evryensis	Cloacibacillus evryensis 158, DSM 19522
144	clique	2521172563	Thioalkalivibrio sp.	Thioalkalivibrio sp. ARh5
144	clique	2523231052	Thioalkalivibrio sp.	Thioalkalivibrio sp. ARh3
144	clique	2519899645	Thioalkalivibrio thiocyanoxidans	Thioalkalivibrio thiocyanoxidans ARh2
146	clique	2524614702	Dorea sp.	Dorea sp. AGR2135
146	clique	641736133	Dorea formicigenerans	Dorea formicigenerans ATCC 27755
146	clique	2513237343	Dorea formicigenerans	Dorea formicigenerans 4_6_53AFAA
147	clique	646206262	Fusobacterium sp.	Fusobacterium sp. 2_1_31
147	clique	2534681820	Fusobacterium periodonticum	Fusobacterium periodonticum D10
168	clique	647533241	Veillonella sp.	Veillonella sp. 3_1_44
168	clique	647533242	Veillonella sp.	Veillonella sp. 6_1_27
168	clique	647000331	Veillonella parvula	Veillonella parvula ATCC 17745
170	clique	2519899624	Citrobacter sp.	Citrobacter sp. A1
170	clique	2519899729	Citrobacter sp.	Citrobacter sp. L17
170	clique	2519899575	Citrobacter freundii	Citrobacter freundii ATCC 8090
170	clique	2531839651	Citrobacter freundii	Citrobacter freundii GTC 09479
170	clique	2534682223	Citrobacter freundii	Citrobacter freundii GTC 09629
177	clique	2522125170	Microbacterium sp.	Microbacterium sp. 11MF
177	clique	2521172614	Microbacterium paraoxydans	Microbacterium paraoxydans 77MFTsu3.2
181	clique	2513237322	Bilophila sp.	Bilophila sp. 4_1_30
181	clique	649989917	Bilophila wadsworthia	Bilophila wadsworthia 3_1_6
187	clique	651324080	Paenibacillus sp.	Paenibacillus sp. HGF5
187	clique	646311929	Paenibacillus lautus	Paenibacillus lautus Y412MC10
203	clique	2512047053	Alistipes sp.	Alistipes sp. HGB5
203	clique	2509601035	Alistipes finegoldii	Alistipes finegoldii AHN 2437, DSM 17242
221	clique	643886056	Acidaminococcus sp.	Acidaminococcus sp. D21
221	clique	2541047012	Acidaminococcus sp.	Acidaminococcus sp. HPA0509
221	clique	2511231106	Acidaminococcus intestini	Acidaminococcus intestini RyC-MR95
221	clique	2524614855	Acidaminococcus intestini	Acidaminococcus intestini DSM 21505
223	clique	643692055	Wolbachia sp.	Wolbachia sp. wRi
223	clique	2540341171	Wolbachia sp.	Wolbachia endosymbiont of Drosophila simulans wHa
223	clique	637000339	Wolbachia endosymbiont	Wolbachia endosymbiont of Drosophila melanogaster
225	clique	2529293077	Slackia sp.	Slackia sp. CM382
225	clique	645951837	Slackia exigua	Slackia exigua ATCC 700122
233	clique	2531839536	Acinetobacter sp.	Acinetobacter sp. NBRC 100985
233	clique	2551306081	Acinetobacter venetianus	Acinetobacter venetianus RAG-1
233	clique	2551306104	Acinetobacter venetianus	Acinetobacter venetianus VE-C3
237	clique	647000328	Streptomyces sp.	Streptomyces griseus XyelbKG-1 1
237	clique	641522653	Streptomyces griseus	Streptomyces griseus griseus NBRC 13350
243	clique	646206251	Coprobacillus sp.	Coprobacillus sp. D7
243	clique	2513237364	Coprobacillus sp.	Coprobacillus sp. 8_2_54BFAA
243	clique	2513237376	Coprobacillus sp.	Coprobacillus sp. 3_3_56FAA
243	clique	641736198	Clostridium ramosum	Clostridium ramosum VPI 0427, DSM 1402
254	clique	651324091	Propionibacterium sp.	Propionibacterium sp. 409-HC1
254	clique	651324092	Propionibacterium sp.	Propionibacterium sp. 434-HC2
254	clique	2512047068	Propionibacterium sp.	Propionibacterium sp. CC003-HC2
254	clique	2513237388	Propionibacterium sp.	Propionibacterium sp. 5_U_42AFAA
254	clique	637000215	Propionibacterium acnes	Propionibacterium acnes KPA171202
254	clique	646564561	Propionibacterium acnes	Propionibacterium acnes SK137
254	clique	647000296	Propionibacterium acnes	Propionibacterium acnes J139
254	clique	647000297	Propionibacterium acnes	Propionibacterium acnes J165
254	clique	647000298	Propionibacterium acnes	Propionibacterium acnes SK187

254	clique	651053058	Propionibacterium acnes	Propionibacterium acnes 266
254	clique	651053059	Propionibacterium acnes	Propionibacterium acnes 6609
254	clique	2511231063	Propionibacterium acnes	Propionibacterium acnes TypeIA2 P.acn33
254	clique	2511231095	Propionibacterium acnes	Propionibacterium acnes TypeIA2 P.acn31
254	clique	2511231125	Propionibacterium acnes	Propionibacterium acnes TypeIA2 P.acn17
254	clique	2511231219	Propionibacterium acnes	Propionibacterium acnes ATCC 11828
254	clique	2515154214	Propionibacterium acnes	Propionibacterium acnes DSM 1897
254	clique	2516143011	Propionibacterium acnes	Propionibacterium acnes PRP-38
254	clique	2522572173	Propionibacterium acnes	Propionibacterium acnes C1
254	clique	2529292840	Propionibacterium acnes	Propionibacterium acnes HL002PA2
254	clique	2529292847	Propionibacterium acnes	Propionibacterium acnes HL063PA2
254	clique	2529292952	Propionibacterium acnes	Propionibacterium acnes HL082PA2
254	clique	2531839222	Propionibacterium acnes	Propionibacterium acnes HL045PA1
254	clique	2531839223	Propionibacterium acnes	Propionibacterium acnes HL013PA1
254	clique	2531839225	Propionibacterium acnes	Propionibacterium acnes HL020PA1
254	clique	2531839226	Propionibacterium acnes	Propionibacterium acnes HL053PA2
254	clique	2531839313	Propionibacterium acnes	Propionibacterium acnes HL087PA2
254	clique	2531839314	Propionibacterium acnes	Propionibacterium acnes HL087PA3
254	clique	2531839315	Propionibacterium acnes	Propionibacterium acnes HL110PA4
254	clique	2531839316	Propionibacterium acnes	Propionibacterium acnes HL087PA1
254	clique	2531839317	Propionibacterium acnes	Propionibacterium acnes HL092PA1
254	clique	2531839318	Propionibacterium acnes	Propionibacterium acnes HL083PA1
254	clique	2531839319	Propionibacterium acnes	Propionibacterium acnes HL060PA1
254	clique	2531839320	Propionibacterium acnes	Propionibacterium acnes HL038PA1
254	clique	2531839323	Propionibacterium acnes	Propionibacterium acnes HL083PA2
254	clique	2531839324	Propionibacterium acnes	Propionibacterium acnes HL025PA1
254	clique	2531839421	Propionibacterium acnes	Propionibacterium acnes HL036PA3
254	clique	2531839423	Propionibacterium acnes	Propionibacterium acnes HL030PA2
254	clique	2531839424	Propionibacterium acnes	Propionibacterium acnes HL001PA1
254	clique	2531839425	Propionibacterium acnes	Propionibacterium acnes HL036PA1
254	clique	2531839426	Propionibacterium acnes	Propionibacterium acnes HL036PA2
254	clique	2531839427	Propionibacterium acnes	Propionibacterium acnes HL050PA1
254	clique	2531839428	Propionibacterium acnes	Propionibacterium acnes HL050PA2
254	clique	2531839429	Propionibacterium acnes	Propionibacterium acnes HL050PA3
254	clique	2531839430	Propionibacterium acnes	Propionibacterium acnes HL030PA1
254	clique	2531839451	Propionibacterium acnes	Propionibacterium acnes FZ1/2/0
254	clique	2534681798	Propionibacterium acnes	Propionibacterium acnes HL037PA1
254	clique	2534682005	Propionibacterium acnes	Propionibacterium acnes HL078PA1
254	clique	2537561578	Propionibacterium acnes	Propionibacterium acnes HL110PA3
254	clique	2537561579	Propionibacterium acnes	Propionibacterium acnes HL110PA2
254	clique	2537561580	Propionibacterium acnes	Propionibacterium acnes HL043PA2
254	clique	2537561581	Propionibacterium acnes	Propionibacterium acnes HL043PA1
254	clique	2537561582	Propionibacterium acnes	Propionibacterium acnes HL013PA2
254	clique	2537561583	Propionibacterium acnes	Propionibacterium acnes HL110PA1
254	clique	2537561584	Propionibacterium acnes	Propionibacterium acnes HL074PA1
254	clique	2537561585	Propionibacterium acnes	Propionibacterium acnes HL072PA2
254	clique	2537561586	Propionibacterium acnes	Propionibacterium acnes HL072PA1
254	clique	2537561594	Propionibacterium acnes	Propionibacterium acnes HL099PA1
254	clique	2537561595	Propionibacterium acnes	Propionibacterium acnes HL097PA1
254	clique	2537561596	Propionibacterium acnes	Propionibacterium acnes HL103PA1
254	clique	2537561597	Propionibacterium acnes	Propionibacterium acnes HL096PA3
254	clique	2537561598	Propionibacterium acnes	Propionibacterium acnes HL096PA2
254	clique	2537561671	Propionibacterium acnes	Propionibacterium acnes HL046PA2
254	clique	2537561672	Propionibacterium acnes	Propionibacterium acnes HL046PA1
254	clique	2537561673	Propionibacterium acnes	Propionibacterium acnes HL005PA2
254	clique	2537561674	Propionibacterium acnes	Propionibacterium acnes HL005PA1
254	clique	2537561675	Propionibacterium acnes	Propionibacterium acnes HL059PA1
254	clique	2537561676	Propionibacterium acnes	Propionibacterium acnes HL025PA2
254	clique	2537561677	Propionibacterium acnes	Propionibacterium acnes HL086PA1
254	clique	2537561678	Propionibacterium acnes	Propionibacterium acnes HL059PA2
254	clique	2537561681	Propionibacterium acnes	Propionibacterium acnes HL005PA4
254	clique	2537561682	Propionibacterium acnes	Propionibacterium acnes HL005PA3
254	clique	2537561736	Propionibacterium acnes	Propionibacterium acnes HL082PA1
254	clique	2537561743	Propionibacterium acnes	Propionibacterium acnes HL056PA1
254	clique	2537561815	Propionibacterium acnes	Propionibacterium acnes HL007PA1
254	clique	2537561816	Propionibacterium acnes	Propionibacterium acnes HL002PA3
254	clique	2537561817	Propionibacterium acnes	Propionibacterium acnes HL053PA1
254	clique	2537561818	Propionibacterium acnes	Propionibacterium acnes HL002PA1
254	clique	2537561819	Propionibacterium acnes	Propionibacterium acnes HL063PA1
254	clique	2537561820	Propionibacterium acnes	Propionibacterium acnes HL027PA2
254	clique	2537561821	Propionibacterium acnes	Propionibacterium acnes HL027PA1
254	clique	2537561822	Propionibacterium acnes	Propionibacterium acnes HL067PA1
254	clique	2537562124	Propionibacterium acnes	Propionibacterium acnes SK182
254	clique	2540341064	Propionibacterium acnes	Propionibacterium acnes HL096PA1
259	clique	2545824769	Pasteurella sp.	Pasteurella multocida RIIF
259	clique	2545824770	Pasteurella sp.	Pasteurella multocida P1933
259	clique	2545824771	Pasteurella sp.	Pasteurella multocida 1500C
259	clique	2545824772	Pasteurella sp.	Pasteurella multocida 2000
259	clique	2545824773	Pasteurella sp.	Pasteurella multocida 671/90
259	clique	637000203	Pasteurella multocida	Pasteurella multocida multocida Pm70
259	clique	2512047043	Pasteurella multocida	Pasteurella multocida 36950
259	clique	2512564074	Pasteurella multocida	Pasteurella multocida multocida HN06
259	clique	2513237228	Pasteurella multocida	Pasteurella multocida multocida P-3480
259	clique	2519103085	Pasteurella multocida	Pasteurella multocida multocida P52VAC
259	clique	2529293056	Pasteurella multocida	Pasteurella multocida multocida Anand1_goat

259	clique	2529293057	Pasteurella multocida	Pasteurella multocida gallicida Anand1_poultry
265	clique	2551306356	Pseudomonas sp.	Pseudomonas sp. S5.2
265	clique	650716075	Pseudomonas mendocina	Pseudomonas mendocina NK-01
272	clique	2551306543	Pantoea sp.	Pantoea sp. B40
272	clique	646564558	Pantoea ananatis	Pantoea ananatis LMG 20103
272	clique	651053056	Pantoea ananatis	Pantoea ananatis AJ13355
272	clique	2511231160	Pantoea ananatis	Pantoea ananatis PA13
272	clique	2511231178	Pantoea ananatis	Pantoea ananatis LMG 5342
275	clique	650716007	Agrobacterium sp.	Agrobacterium sp. H13-3
275	clique	2521172663	Agrobacterium sp.	Agrobacterium sp. 10MFCol1.1
275	clique	2537562123	Agrobacterium tumefaciens	Agrobacterium tumefaciens 5A
275	clique	2554235003	Agrobacterium tumefaciens	Agrobacterium tumefaciens WRT31
276	clique	2554235291	unclassified	Sphingomonas sp. YL-JM2C
276	clique	640427144	Sphingomonas wittichii	Sphingomonas wittichii RW1
276	clique	2548876930	Sphingomonas wittichii	Sphingomonas wittichii DP58
278	clique	2521172537	Klebsiella sp.	Klebsiella sp. BRL6-2
278	clique	2531839402	Klebsiella oxytoca	Klebsiella oxytoca 10-5243
278	clique	2531839404	Klebsiella oxytoca	Klebsiella oxytoca 10-5245
278	clique	2547132040	Klebsiella oxytoca	Klebsiella oxytoca 10-5248
278	clique	2548876782	Klebsiella oxytoca	Klebsiella oxytoca 11492-1
284	clique	2551306561	Aeromonas sp.	Aeromonas sp. MDS8
284	clique	2529292917	Aeromonas hydrophila	Aeromonas hydrophila SSU
284	clique	2551306511	Aeromonas hydrophila	Aeromonas hydrophila 277
284	clique	2551306512	Aeromonas hydrophila	Aeromonas hydrophila 113
284	clique	2551306513	Aeromonas hydrophila	Aeromonas hydrophila 259
284	clique	2551306514	Aeromonas hydrophila	Aeromonas hydrophila 187
284	clique	2551306515	Aeromonas hydrophila	Aeromonas hydrophila 173
284	clique	2551306648	Aeromonas hydrophila	Aeromonas hydrophila 14
286	clique	2554235234	Klebsiella sp.	Klebsiella oxytoca SA2
286	clique	2519899599	Klebsiella oxytoca	Klebsiella oxytoca M5al
292	clique	643886214	Acinetobacter sp.	Acinetobacter sp. ATCC 27244
292	clique	647000202	Acinetobacter haemolyticus	Acinetobacter haemolyticus ATCC 19194
292	clique	2537561554	Acinetobacter haemolyticus	Acinetobacter haemolyticus CIP 64.3
292	clique	2537561834	Acinetobacter haemolyticus	Acinetobacter haemolyticus NIPH 261
292	clique	2545824737	Acinetobacter haemolyticus	Acinetobacter haemolyticus MTCC 9819
292	clique	2551306342	Acinetobacter haemolyticus	Acinetobacter haemolyticus TG19599
292	clique	2551306343	Acinetobacter haemolyticus	Acinetobacter haemolyticus TG21157
292	clique	2551306346	Acinetobacter haemolyticus	Acinetobacter haemolyticus TG19602
293	clique	645058833	Fusobacterium sp.	Fusobacterium sp. D11
293	clique	646206252	Fusobacterium sp.	Fusobacterium nucleatum animalis 7_1
293	clique	647533170	Fusobacterium sp.	Fusobacterium sp. 3_1_33
293	clique	651324032	Fusobacterium sp.	Fusobacterium sp. 11_3_2
293	clique	651324033	Fusobacterium sp.	Fusobacterium sp. 21_1A
293	clique	2554235311	Fusobacterium sp.	Fusobacterium sp. 4_8
293	clique	2531839191	Fusobacterium nucleatum	Fusobacterium nucleatum polymorphum F0401
293	clique	2537561939	Fusobacterium nucleatum	Fusobacterium nucleatum animalis F0419
293	clique	2554235040	Fusobacterium nucleatum	Fusobacterium nucleatum animalis ChDC F324
298	clique	2529292817	Capnocytophaga sp.	Capnocytophaga sp. oral taxon 380 F0488
298	clique	2531839279	Capnocytophaga sp.	Capnocytophaga sp. oral taxon 335 F0486
298	clique	2531839552	Capnocytophaga sp.	Capnocytophaga sp. oral taxon 324 F0483
298	clique	2540341000	Capnocytophaga sp.	Capnocytophaga sp. oral taxon 412 F0487
298	clique	2541047019	Capnocytophaga sp.	Capnocytophaga sp. oral taxon 336 F0502
298	clique	644736338	Capnocytophaga ochracea	Capnocytophaga ochracea VPI 2845, DSM 7271
298	clique	649989920	Capnocytophaga ochracea	Capnocytophaga ochracea F0287
298	clique	2531839278	Capnocytophaga ochracea	Capnocytophaga ochracea Holt 25, ATCC 33596
301	clique	2541047255	Clostridium sp.	Clostridium difficile CD8
301	clique	2541047256	Clostridium sp.	Clostridium difficile CD9
301	clique	2541047257	Clostridium sp.	Clostridium difficile CD18
301	clique	2541047259	Clostridium sp.	Clostridium difficile CD17
301	clique	2541047260	Clostridium sp.	Clostridium difficile CD13
301	clique	2541047263	Clostridium sp.	Clostridium difficile CD40
301	clique	2541047265	Clostridium sp.	Clostridium difficile CD34
301	clique	2541047269	Clostridium sp.	Clostridium difficile CD43
301	clique	2541047270	Clostridium sp.	Clostridium difficile CD49
301	clique	2541047271	Clostridium sp.	Clostridium difficile CD47
301	clique	2541047272	Clostridium sp.	Clostridium difficile CD46
301	clique	2541047273	Clostridium sp.	Clostridium difficile CD45
301	clique	2541047274	Clostridium sp.	Clostridium difficile CD51
301	clique	2541047278	Clostridium sp.	Clostridium difficile CD70
301	clique	2541047281	Clostridium sp.	Clostridium difficile CD104
301	clique	2541047282	Clostridium sp.	Clostridium difficile CD109
301	clique	2541047283	Clostridium sp.	Clostridium difficile CD90
301	clique	2541047284	Clostridium sp.	Clostridium difficile CD111
301	clique	2541047286	Clostridium sp.	Clostridium difficile CD129
301	clique	2541047288	Clostridium sp.	Clostridium difficile CD131
301	clique	2541047289	Clostridium sp.	Clostridium difficile CD159
301	clique	2541047291	Clostridium sp.	Clostridium difficile CD149
301	clique	2541047292	Clostridium sp.	Clostridium difficile CD144
301	clique	2541047296	Clostridium sp.	Clostridium difficile CD169
301	clique	2541047300	Clostridium sp.	Clostridium difficile CD178
301	clique	2541047301	Clostridium sp.	Clostridium difficile CD181
301	clique	2541047302	Clostridium sp.	Clostridium difficile CD200
301	clique	2541047303	Clostridium sp.	Clostridium difficile CD201
301	clique	2541047307	Clostridium sp.	Clostridium difficile 655
301	clique	2541047310	Clostridium sp.	Clostridium difficile 842

301	clique	2541047313 Clostridium sp.	Clostridium difficile 6057
301	clique	2541047314 Clostridium sp.	Clostridium difficile DA00044
301	clique	2541047317 Clostridium sp.	Clostridium difficile DA00114
301	clique	2541047318 Clostridium sp.	Clostridium difficile DA00126
301	clique	2541047319 Clostridium sp.	Clostridium difficile DA00128
301	clique	2541047320 Clostridium sp.	Clostridium difficile DA00129
301	clique	2541047321 Clostridium sp.	Clostridium difficile DA00141
301	clique	2541047322 Clostridium sp.	Clostridium difficile DA00131
301	clique	2541047325 Clostridium sp.	Clostridium difficile DA00142
301	clique	2541047327 Clostridium sp.	Clostridium difficile DA00149
301	clique	2541047329 Clostridium sp.	Clostridium difficile DA00160
301	clique	2541047330 Clostridium sp.	Clostridium difficile DA00167
301	clique	2541047332 Clostridium sp.	Clostridium difficile DA00174
301	clique	2541047333 Clostridium sp.	Clostridium difficile DA00189
301	clique	2541047335 Clostridium sp.	Clostridium difficile DA00191
301	clique	2541047337 Clostridium sp.	Clostridium difficile DA00196
301	clique	2541047339 Clostridium sp.	Clostridium difficile DA00197
301	clique	2541047341 Clostridium sp.	Clostridium difficile DA00203
301	clique	2541047344 Clostridium sp.	Clostridium difficile DA00244
301	clique	2541047346 Clostridium sp.	Clostridium difficile DA00232
301	clique	2541047347 Clostridium sp.	Clostridium difficile DA00216
301	clique	2541047348 Clostridium sp.	Clostridium difficile DA00215
301	clique	2541047349 Clostridium sp.	Clostridium difficile DA00245
301	clique	2541047352 Clostridium sp.	Clostridium difficile DA00275
301	clique	2541047354 Clostridium sp.	Clostridium difficile DA00273
301	clique	2541047356 Clostridium sp.	Clostridium difficile DA00313
301	clique	2541047358 Clostridium sp.	Clostridium difficile DA00310
301	clique	2541047360 Clostridium sp.	Clostridium difficile DA00306
301	clique	2541047361 Clostridium sp.	Clostridium difficile F253
301	clique	2541047362 Clostridium sp.	Clostridium difficile F249
301	clique	2541047365 Clostridium sp.	Clostridium difficile Y10
301	clique	2541047369 Clostridium sp.	Clostridium difficile Y41
301	clique	2541047373 Clostridium sp.	Clostridium difficile Y215
301	clique	2541047374 Clostridium sp.	Clostridium difficile Y231
301	clique	2541047375 Clostridium sp.	Clostridium difficile Y312
301	clique	2541047377 Clostridium sp.	Clostridium difficile Y307
301	clique	2541047378 Clostridium sp.	Clostridium difficile Y247
301	clique	2541047382 Clostridium sp.	Clostridium difficile Y381
301	clique	2541047384 Clostridium sp.	Clostridium difficile Y401
301	clique	2541047386 Clostridium sp.	Clostridium difficile P5
301	clique	2541047388 Clostridium sp.	Clostridium difficile P7
301	clique	2541047392 Clostridium sp.	Clostridium difficile P19
301	clique	2541047393 Clostridium sp.	Clostridium difficile P21
301	clique	2541047396 Clostridium sp.	Clostridium difficile P25
301	clique	2541047397 Clostridium sp.	Clostridium difficile P20
301	clique	2541047400 Clostridium sp.	Clostridium difficile P30
301	clique	2541047405 Clostridium sp.	Clostridium difficile P42
301	clique	2541047406 Clostridium sp.	Clostridium difficile P45
301	clique	2541047409 Clostridium sp.	Clostridium difficile P49
301	clique	2541047413 Clostridium sp.	Clostridium difficile P59
301	clique	2541047415 Clostridium sp.	Clostridium difficile P72
301	clique	2541047416 Clostridium sp.	Clostridium difficile P70
301	clique	2541047417 Clostridium sp.	Clostridium difficile P69
301	clique	2541047419 Clostridium sp.	Clostridium difficile P74
301	clique	2541047420 Clostridium sp.	Clostridium difficile P75
301	clique	2541047488 Clostridium sp.	Clostridium difficile CD196
301	clique	2541047496 Clostridium sp.	Clostridium difficile P11
301	clique	2541047511 Clostridium sp.	Clostridium difficile F548
301	clique	2541047514 Clostridium sp.	Clostridium difficile F200
301	clique	638341066 Clostridium difficile	Clostridium difficile QCD-32g58
301	clique	640069308 Clostridium difficile	Clostridium difficile 630 (epidemic type X)
301	clique	641736216 Clostridium difficile	Clostridium difficile QCD-76w55, NAP1
301	clique	641736217 Clostridium difficile	Clostridium difficile QCD-63q42
301	clique	641736218 Clostridium difficile	Clostridium difficile QCD-37x79, NAP1a/001
301	clique	641736219 Clostridium difficile	Clostridium difficile QCD-97b34, NAP1b/006
301	clique	642791606 Clostridium difficile	Clostridium difficile CIP 107932
301	clique	642791631 Clostridium difficile	Clostridium difficile QCD-23m63
301	clique	645058803 Clostridium difficile	Clostridium difficile QCD-66c26
301	clique	646311914 Clostridium difficile	Clostridium difficile CD196
301	clique	646311915 Clostridium difficile	Clostridium difficile R20291
301	clique	647000226 Clostridium difficile	Clostridium difficile BI9
301	clique	647000227 Clostridium difficile	Clostridium difficile NAP07
301	clique	647000228 Clostridium difficile	Clostridium difficile NAP08
301	clique	2512047057 Clostridium difficile	Clostridium difficile VPI 10463, ATCC 43255
301	clique	2515154168 Clostridium difficile	Clostridium difficile ATCC 9689
301	clique	2526164710 Clostridium difficile	Clostridium difficile DSM 1296
301	clique	2534681960 Clostridium difficile	Clostridium difficile 002-P50-2011
301	clique	2534681961 Clostridium difficile	Clostridium difficile 050-P50-2011
301	clique	2537561695 Clostridium difficile	Clostridium difficile 70-100-2010
301	clique	2541047254 Clostridium difficile	Clostridium difficile CD3
301	clique	2541047258 Clostridium difficile	Clostridium difficile CD21
301	clique	2541047261 Clostridium difficile	Clostridium difficile CD22
301	clique	2541047262 Clostridium difficile	Clostridium difficile CD39
301	clique	2541047264 Clostridium difficile	Clostridium difficile CD38
301	clique	2541047266 Clostridium difficile	Clostridium difficile CD44

301	clique	2541047267	Clostridium difficile	Clostridium difficile CD42
301	clique	2541047268	Clostridium difficile	Clostridium difficile CD41
301	clique	2541047275	Clostridium difficile	Clostridium difficile CD88
301	clique	2541047276	Clostridium difficile	Clostridium difficile CD86
301	clique	2541047277	Clostridium difficile	Clostridium difficile CD69
301	clique	2541047279	Clostridium difficile	Clostridium difficile CD68
301	clique	2541047280	Clostridium difficile	Clostridium difficile CD92
301	clique	2541047285	Clostridium difficile	Clostridium difficile CD113
301	clique	2541047287	Clostridium difficile	Clostridium difficile CD127
301	clique	2541047290	Clostridium difficile	Clostridium difficile CD132
301	clique	2541047293	Clostridium difficile	Clostridium difficile CD133
301	clique	2541047294	Clostridium difficile	Clostridium difficile CD165
301	clique	2541047297	Clostridium difficile	Clostridium difficile CD166
301	clique	2541047298	Clostridium difficile	Clostridium difficile CD170
301	clique	2541047299	Clostridium difficile	Clostridium difficile CD175
301	clique	2541047304	Clostridium difficile	Clostridium difficile CD206
301	clique	2541047305	Clostridium difficile	Clostridium difficile CD211
301	clique	2541047306	Clostridium difficile	Clostridium difficile CD212
301	clique	2541047308	Clostridium difficile	Clostridium difficile 824
301	clique	2541047309	Clostridium difficile	Clostridium difficile 840
301	clique	2541047311	Clostridium difficile	Clostridium difficile 6041
301	clique	2541047312	Clostridium difficile	Clostridium difficile 6042
301	clique	2541047315	Clostridium difficile	Clostridium difficile DA00062
301	clique	2541047316	Clostridium difficile	Clostridium difficile DA00065
301	clique	2541047323	Clostridium difficile	Clostridium difficile DA00132
301	clique	2541047324	Clostridium difficile	Clostridium difficile DA00134
301	clique	2541047326	Clostridium difficile	Clostridium difficile DA00145
301	clique	2541047328	Clostridium difficile	Clostridium difficile DA00154
301	clique	2541047331	Clostridium difficile	Clostridium difficile DA00165
301	clique	2541047334	Clostridium difficile	Clostridium difficile DA00183
301	clique	2541047336	Clostridium difficile	Clostridium difficile DA00193
301	clique	2541047338	Clostridium difficile	Clostridium difficile DA00195
301	clique	2541047340	Clostridium difficile	Clostridium difficile DA00211
301	clique	2541047342	Clostridium difficile	Clostridium difficile DA00210
301	clique	2541047343	Clostridium difficile	Clostridium difficile DA00212
301	clique	2541047345	Clostridium difficile	Clostridium difficile DA00238
301	clique	2541047350	Clostridium difficile	Clostridium difficile DA00246
301	clique	2541047351	Clostridium difficile	Clostridium difficile DA00305
301	clique	2541047353	Clostridium difficile	Clostridium difficile DA00261
301	clique	2541047355	Clostridium difficile	Clostridium difficile DA00256
301	clique	2541047357	Clostridium difficile	Clostridium difficile F152
301	clique	2541047359	Clostridium difficile	Clostridium difficile DA00307
301	clique	2541047363	Clostridium difficile	Clostridium difficile F314
301	clique	2541047366	Clostridium difficile	Clostridium difficile Y21
301	clique	2541047367	Clostridium difficile	Clostridium difficile Y171
301	clique	2541047368	Clostridium difficile	Clostridium difficile Y155
301	clique	2541047370	Clostridium difficile	Clostridium difficile Y165
301	clique	2541047371	Clostridium difficile	Clostridium difficile Y202
301	clique	2541047376	Clostridium difficile	Clostridium difficile Y270
301	clique	2541047379	Clostridium difficile	Clostridium difficile Y266
301	clique	2541047380	Clostridium difficile	Clostridium difficile Y343
301	clique	2541047381	Clostridium difficile	Clostridium difficile Y358
301	clique	2541047385	Clostridium difficile	Clostridium difficile P3
301	clique	2541047387	Clostridium difficile	Clostridium difficile P6
301	clique	2541047389	Clostridium difficile	Clostridium difficile P9
301	clique	2541047390	Clostridium difficile	Clostridium difficile P13
301	clique	2541047391	Clostridium difficile	Clostridium difficile P15
301	clique	2541047394	Clostridium difficile	Clostridium difficile P23
301	clique	2541047395	Clostridium difficile	Clostridium difficile P24
301	clique	2541047398	Clostridium difficile	Clostridium difficile P32
301	clique	2541047399	Clostridium difficile	Clostridium difficile P29
301	clique	2541047401	Clostridium difficile	Clostridium difficile P31
301	clique	2541047403	Clostridium difficile	Clostridium difficile P38
301	clique	2541047404	Clostridium difficile	Clostridium difficile P36
301	clique	2541047407	Clostridium difficile	Clostridium difficile P46
301	clique	2541047408	Clostridium difficile	Clostridium difficile P50
301	clique	2541047410	Clostridium difficile	Clostridium difficile P48
301	clique	2541047411	Clostridium difficile	Clostridium difficile P51
301	clique	2541047412	Clostridium difficile	Clostridium difficile P78
301	clique	2541047414	Clostridium difficile	Clostridium difficile P61
301	clique	2541047418	Clostridium difficile	Clostridium difficile P71
301	clique	2541047421	Clostridium difficile	Clostridium difficile P77
301	clique	2541047489	Clostridium difficile	Clostridium difficile 342
301	clique	2541047490	Clostridium difficile	Clostridium difficile P1
301	clique	2541047494	Clostridium difficile	Clostridium difficile P2
301	clique	2541047495	Clostridium difficile	Clostridium difficile P73
301	clique	2541047497	Clostridium difficile	Clostridium difficile P8
301	clique	2541047510	Clostridium difficile	Clostridium difficile F601
301	clique	2541047512	Clostridium difficile	Clostridium difficile F525
301	clique	2541047513	Clostridium difficile	Clostridium difficile F480
301	clique	2547132210	Clostridium difficile	Clostridium difficile 6503
301	clique	2547132211	Clostridium difficile	Clostridium difficile 6466
301	clique	2548876768	Clostridium difficile	Clostridium difficile CD37
308	clique	2501846301	Xanthomonas sp.	Xanthomonas sp. 5
308	clique	651324111	Xanthomonas vesicatoria	Xanthomonas vesicatoria Maraite, ATCC 35937

311	clique	645058782 Bacteroides sp.	Bacteroides sp. 2_1_7
311	clique	647533112 Bacteroides sp.	Bacteroides sp. 2_1_33B
311	clique	648861001 Bacteroides sp.	Bacteroides sp. 20_3
311	clique	648861002 Bacteroides sp.	Bacteroides sp. 3_1_19
311	clique	646206279 Parabacteroides sp.	Parabacteroides sp. D13
311	clique	640753039 Parabacteroides distasonis	Parabacteroides distasonis ATCC 8503
311	clique	2534682208 Parabacteroides distasonis	Parabacteroides distasonis CL09T03C24
311	clique	2534682209 Parabacteroides distasonis	Parabacteroides distasonis CL03T12C09
312	clique	2504756062 Paenibacillus sp.	Paenibacillus sp. J6
312	clique	2505679063 Paenibacillus sp.	Paenibacillus sp. J10
312	clique	2508501032 Paenibacillus sp.	Paenibacillus sp. J14
312	clique	2528768218 Paenibacillus sp.	Paenibacillus sp. J14
312	clique	2528768219 Paenibacillus sp.	Paenibacillus sp. J6
312	clique	2505679060 Paenibacillus barengoltzii	Paenibacillus barengoltzii J12
312	clique	2528768208 Paenibacillus barengoltzii	Paenibacillus barengoltzii J12
313	clique	645951866 Fusobacterium sp.	Fusobacterium sp. 3_1_5R
313	clique	645951804 Fusobacterium gonidiaformans	Fusobacterium gonidiaformans ATCC 25563
316	clique	646311966 Vibrio sp.	Vibrio sp. Ex25
316	clique	2545555817 Vibrio sp.	Vibrio sp. Ex25
316	clique	2534682140 Vibrio alginolyticus	Vibrio alginolyticus E0666
324	clique	2522572104 Burkholderia sp.	Burkholderia sp. URHA0054
324	clique	641736151 Burkholderia graminis	Burkholderia graminis C4D1M
340	clique	2512047052 Actinomyces sp.	Actinomyces sp. F0384
340	clique	649989905 Actinomyces viscosus	Actinomyces viscosus C505
342	clique	2516493017 Desulfitobacterium sp.	Desulfitobacterium sp. LBE
342	clique	637000093 Desulfitobacterium hafniense	Desulfitobacterium hafniense Y51
342	clique	643348537 Desulfitobacterium hafniense	Desulfitobacterium hafniense DCB-2
342	clique	2513020008 Desulfitobacterium hafniense	Desulfitobacterium hafniense TCE-1
342	clique	2513237149 Desulfitobacterium hafniense	Desulfitobacterium hafniense PCP-1
342	clique	2516653041 Desulfitobacterium hafniense	Desulfitobacterium hafniense TCP-A
342	clique	2522572064 Desulfitobacterium hafniense	Desulfitobacterium hafniense DP7 (draft 151 contigs)
350	clique	646206263 Bacteroides sp.	Bacteroides sp. 9_1_42FAA
350	clique	647533113 Bacteroides sp.	Bacteroides sp. 3_1_33FAA
350	clique	642979370 Bacteroides dorei	Bacteroides dorei DSM 17855
350	clique	646206258 Bacteroides dorei	Bacteroides dorei 5_1_36/D4
350	clique	2531839307 Bacteroides dorei	Bacteroides dorei CL02T12C06
350	clique	2531839308 Bacteroides dorei	Bacteroides dorei CL03T12C01
350	clique	2537562062 Bacteroides dorei	Bacteroides dorei CL02T00C15
351	clique	2551306537 Enterobacter sp.	Enterobacter sp. B509
351	clique	2547132296 Enterobacter mori	Enterobacter mori LMG 25706
361	clique	2516653053 Deinococcus sp.	Deinococcus sp. 2009
361	clique	2524614857 Deinococcus ficus	Deinococcus ficus DSM 19119
370	clique	2526164723 Pectobacterium sp.	Pectobacterium sp. SCC3193
370	clique	646311947 Pectobacterium wasabiae	Pectobacterium wasabiae WPP163
380	clique	2537561632 Capnocytophaga sp.	Capnocytophaga sp. CM59
380	clique	2541046980 Capnocytophaga granulosa	Capnocytophaga granulosa ATCC 51502
384	clique	2513237088 Rhizobium sp.	Rhizobium sp. STM6155
384	clique	2534682333 Rhizobium mesoamericanum	Rhizobium mesoamericanum STM3625
400	clique	649633069 unclassified	Micromonospora sp. L5
400	clique	2501939600 unclassified	Micromonospora L5
400	clique	648028042 Micromonospora aurantiaca	Micromonospora aurantiaca ATCC 27029
410	clique	2546826724 Methylobacterium sp.	Methylobacterium sp. EUR3 AL-11
410	clique	2547132193 Methylobacterium sp.	Methylobacterium sp. L2-4
410	clique	641522638 Methylobacterium radiotolerans	Methylobacterium radiotolerans JCM 2831
411	clique	639633059 Shewanella sp.	Shewanella sp. W3-18-1
411	clique	2510436004 Shewanella putrefaciens	Shewanella putrefaciens 200 (Missing data)
411	clique	2524023073 Shewanella putrefaciens	Shewanella putrefaciens CN-32
419	clique	2505679059 Ochrobactrum sp.	Ochrobactrum sp. J50
419	clique	2504756061 Ochrobactrum intermedium	Ochrobactrum intermedium J2
431	clique	2516653074 Burkholderia sp.	Burkholderia sp. WSM4176
431	clique	2501025500 Burkholderia tuberum	Burkholderia tuberum STM678
436	clique	2551306696 Caldicellulosiruptor sp.	Caldicellulosiruptor sp. F32
436	clique	640427106 Caldicellulosiruptor saccharolyticus	Caldicellulosiruptor saccharolyticus DSM 8903
444	clique	648276706 Oscillatoria sp.	Oscillatoria sp. PCC 6506
444	clique	2508501075 Oscillatoria formosa	Oscillatoria formosa PCC 6407
452	clique	2541047099 Pseudomonas sp.	Pseudomonas sp. CF150
452	clique	2541047103 Pseudomonas sp.	Pseudomonas sp. CFT9
452	clique	2521172707 Pseudomonas fluorescens	Pseudomonas fluorescens A506
455	clique	2508501008 unclassified	Azospira sp. ZAP
455	clique	2508501046 Azospira suillum	Azospira suillum PS
462	clique	2505679024 Fischerella sp.	Fischerella sp. JSC-11
462	clique	2548876998 Fischerella thermalis	Fischerella thermalis PCC 7521
480	clique	2529293097 Pelosinus fermentans	Pelosinus fermentans A11
480	clique	2529293098 Pelosinus fermentans	Pelosinus fermentans A12
480	clique	2529293099 Pelosinus fermentans	Pelosinus fermentans R7, DSM 17108
480	clique	2534682259 Pelosinus fermentans	Pelosinus fermentans B3
480	clique	2537561517 Pelosinus fermentans	Pelosinus fermentans B4
480	clique	2511231046 Pelosinus sp.	Pelosinus sp. HCF1
481	clique	2503198003 Clostridium sp.	Clostridium sp. CP1
481	clique	2531839097 Clostridium pasteurianum	Clostridium pasteurianum DSM 525
482	clique	651285008 Streptococcus sp.	Streptococcus sp. C150
482	clique	2516143101 Streptococcus salivarius	Streptococcus salivarius PS4
484	clique	2537561772 Campylobacter sp.	Campylobacter sp. FOBRC14
484	clique	640753010 Campylobacter curvus	Campylobacter curvus 525.92
489	clique	2541047041 unclassified	Leptospira sp. sv. Kenya Sh9

489	clique	639633032	Leptospira borgpetersenii	Leptospira borgpetersenii sv Hardjo-bovis JB197
489	clique	639633033	Leptospira borgpetersenii	Leptospira borgpetersenii sv Hardjo-bovis L550
489	clique	2519103083	Leptospira borgpetersenii	Leptospira borgpetersenii sv. Castellonis 200801910
489	clique	2519103130	Leptospira borgpetersenii	Leptospira borgpetersenii UI 09149
489	clique	2526164610	Leptospira borgpetersenii	Leptospira borgpetersenii sv. Mini 201000851
489	clique	2526164642	Leptospira borgpetersenii	Leptospira borgpetersenii sv. Javanica MK146
489	clique	2526164643	Leptospira borgpetersenii	Leptospira borgpetersenii Brem 328
489	clique	2526164644	Leptospira borgpetersenii	Leptospira borgpetersenii Brem 307
489	clique	2528311142	Leptospira borgpetersenii	Leptospira borgpetersenii sv. Javanica UI 09931
489	clique	2537561563	Leptospira borgpetersenii	Leptospira borgpetersenii Noumea 25
489	clique	2537561607	Leptospira borgpetersenii	Leptospira borgpetersenii 200801926
489	clique	2541047033	Leptospira borgpetersenii	Leptospira borgpetersenii sv. Hardjo-bovis Sponselee
489	clique	2548876766	Leptospira borgpetersenii	Leptospira borgpetersenii sv. Kenya TE 0159
489	clique	2548876767	Leptospira borgpetersenii	Leptospira borgpetersenii sv. Javanica L0864
489	clique	2551306643	Leptospira borgpetersenii	Leptospira borgpetersenii sv. Hardjo-bovis Lely 607
489	clique	2551306644	Leptospira borgpetersenii	Leptospira borgpetersenii sv. Javanica L0066
492	clique	2512047080	Vibrio sp.	Vibrio sp. N418
492	clique	2512047079	Vibrio scophthalmi	Vibrio scophthalmi LMG 19158
495	clique	648276708	Pantoea sp.	Pantoea sp. aB
495	clique	2541047590	Pantoea agglomerans	Pantoea agglomerans 299R
513	clique	648861006	unclassified	Burkholderiales bacterium 1_1_47
513	clique	651324084	Parasutterella excrementihominis	Parasutterella excrementihominis YIT 11859
531	clique	2541047040	unclassified	Leptospira sp. P2653
531	clique	2526164577	Leptospira weilii	Leptospira weilii 2006001855
531	clique	2534682340	Leptospira weilii	Leptospira weilii UI 13098
531	clique	2534682341	Leptospira weilii	Leptospira weilii LNT 1234
531	clique	2534682342	Leptospira weilii	Leptospira weilii Ecochallenge
531	clique	2548876967	Leptospira weilii	Leptospira weilii LNT 1194
531	clique	2548876968	Leptospira weilii	Leptospira weilii UI 14631
541	clique	2513237394	Neisseria sp.	Neisseria sp. GT4A_CT1
541	clique	651324075	Neisseria macacae	Neisseria macacae ATCC 33926
552	clique	651324015	unclassified	Bradyrhizobiaceae bacterium SG-6C
552	clique	2537561906	Afipia clevelandensis	Afipia clevelandensis ATCC 49720
559	clique	2513237395	Lactobacillus sp.	Lactobacillus sp. 7_1_47FAA
559	clique	645951832	Lactobacillus iners	Lactobacillus iners DSM 13335
559	clique	648276682	Lactobacillus iners	Lactobacillus iners AB-1
559	clique	649989959	Lactobacillus iners	Lactobacillus iners ATCC 55195
559	clique	649989960	Lactobacillus iners	Lactobacillus iners LEAF 2052A-d
559	clique	649989961	Lactobacillus iners	Lactobacillus iners LEAF 2053A-b
559	clique	649989962	Lactobacillus iners	Lactobacillus iners LEAF 2062A-h1
559	clique	649989963	Lactobacillus iners	Lactobacillus iners LEAF 3008A-a
559	clique	649989965	Lactobacillus iners	Lactobacillus iners LactinV 03V1-b
559	clique	649989966	Lactobacillus iners	Lactobacillus iners LactinV 09V1-c
559	clique	649989967	Lactobacillus iners	Lactobacillus iners LactinV 11V1-d
559	clique	649989968	Lactobacillus iners	Lactobacillus iners SPIN 2503V10-D
559	clique	651324062	Lactobacillus iners	Lactobacillus iners SPIN 1401G
559	clique	651324063	Lactobacillus iners	Lactobacillus iners UPII 143-D
559	clique	651324064	Lactobacillus iners	Lactobacillus iners UPII 60-B
577	clique	2519899622	Pseudomonas sp.	Pseudomonas sp. Ag1
577	clique	2531839262	Pseudomonas fluorescens	Pseudomonas fluorescens BBc6R8
590	clique	2540341169	Serratia sp.	Serratia sp. WW4
590	clique	2554235221	Serratia sp.	Serratia marcescens LCT-SM166
590	clique	2554235222	Serratia sp.	Serratia marcescens LCT-SM262
590	clique	2529293103	Serratia marcescens	Serratia marcescens VGH107
590	clique	2548876920	Serratia marcescens	Serratia marcescens LCT-SM213
595	clique	2502171177	Arcobacter sp.	Arcobacter sp. 1
595	clique	640753004	Arcobacter butzleri	Arcobacter butzleri RM4018
595	clique	649989908	Arcobacter butzleri	Arcobacter butzleri JV22
595	clique	2511231182	Arcobacter butzleri	Arcobacter butzleri ED-1
595	clique	2541046991	Arcobacter butzleri	Arcobacter butzleri 7h1h
600	clique	2513237383	Fusobacterium sp.	Fusobacterium sp. 12_1B
600	clique	645951859	Fusobacterium ulcerans	Fusobacterium ulcerans ATCC 49185
608	clique	2554235440	Mannheimia sp.	Mannheimia haemolytica USMARC_2286
608	clique	647000269	Mannheimia haemolytica	Mannheimia haemolytica A2 BOVINE
608	clique	647000270	Mannheimia haemolytica	Mannheimia haemolytica A2 OVINE
608	clique	647533181	Mannheimia haemolytica	Mannheimia haemolytica PHL213
608	clique	2523533563	Mannheimia haemolytica	Mannheimia haemolytica
608	clique	2537561899	Mannheimia haemolytica	Mannheimia haemolytica 6 H23
608	clique	2545824631	Mannheimia haemolytica	Mannheimia haemolytica USDA-ARS-USMARC-185
608	clique	2545824632	Mannheimia haemolytica	Mannheimia haemolytica USDA-ARS-USMARC-183
608	clique	2554235380	Mannheimia haemolytica	Mannheimia haemolytica D153
608	clique	2554235406	Mannheimia haemolytica	Mannheimia haemolytica D174
608	clique	2554235407	Mannheimia haemolytica	Mannheimia haemolytica D171
614	clique	647533114	Bacteroides sp.	Bacteroides sp. D20
614	clique	649989914	Bacteroides sp.	Bacteroides sp. 4_1_36
614	clique	641380447	Bacteroides uniformis	Bacteroides uniformis ATCC 8492
614	clique	2534681854	Bacteroides uniformis	Bacteroides uniformis CL03T12C37
614	clique	2537561752	Bacteroides uniformis	Bacteroides uniformis CL03T00C23
616	clique	2501846304	Xanthomonas sp.	Xanthomonas sp. 7
616	clique	651324109	Xanthomonas perforans	Xanthomonas gardneri PDDCC 1620, ATCC 19865
617	clique	2523231035	Gordonia sp.	Gordonia sp. KTR9
617	clique	2545824722	Gordonia terrae	Gordonia terrae C-6
620	clique	650377931	Erwinia sp.	Erwinia sp. Ejp617
620	clique	646564532	Erwinia pyrifoliae	Erwinia pyrifoliae Ep1/96
620	clique	646862321	Erwinia pyrifoliae	Erwinia pyrifoliae DSM 12163

629	clique	649989907	Anaerostipes sp.	Anaerostipes sp. 3_2_56FAA
629	clique	641736227	Anaerostipes caccae	Anaerostipes caccae DSM 14662
633	clique	2517572184	Streptomyces sp.	Streptomyces sp. CNY228
633	clique	2526164526	Streptomyces sp.	Streptomyces sp. LaPpAH-201
633	clique	2547132081	Streptomyces sp.	Streptomyces sp. S4
633	clique	645058823	Streptomyces albus	Streptomyces albus J1074
633	clique	2541047081	Streptomyces albus	Streptomyces albus J1074
634	clique	651324107	Turicibacter sp.	Turicibacter sp. HGF1
634	clique	647000330	Turicibacter sanguinis	Turicibacter sanguinis PC909
640	clique	2531839311	Acinetobacter sp.	Acinetobacter sp. HA
640	clique	2551306381	Acinetobacter schindleri	Acinetobacter schindleri TG19614
642	clique	2519899605	Staphylococcus sp.	Staphylococcus sp. OJ82
642	clique	2529293016	Staphylococcus equorum	Staphylococcus equorum equorum Mu2
644	clique	2508501006	Pseudomonas sp.	Pseudomonas sp. PK
644	clique	2518645567	Pseudomonas stutzeri	Pseudomonas stutzeri AN10, CCUG 29243
648	clique	2531839264	Rhodococcus sp.	Rhodococcus sp. JVH1
648	clique	2548877052	Rhodococcus sp.	Rhodococcus sp. DK17
648	clique	637000234	Rhodococcus jostii	Rhodococcus jostii RHA1
654	clique	2527291525	unclassified	Cloacimonetes bacterium JGI 0000039-G13 (Combined_Assembly_WWE1_1__WWE1)
654	clique	642555115	Candidatus Cloacamonas acidaminovorans	Candidatus Cloacamonas acidaminovorans
674	clique	2519899510	Methylotenera sp.	Methylotenera sp. 73s
674	clique	2516493010	Methylotenera versatilis	Methylotenera versatilis 79
677	clique	2537561631	Streptococcus sp.	Streptococcus sp. BS35b
677	clique	2537561966	Streptococcus oralis	Streptococcus oralis SK1074
683	clique	643886005	Clostridium sp.	Clostridium sp M62/1
683	clique	650377923	Clostridium cf. saccharolyticum	Clostridium cf. saccharolyticum K10
691	clique	639857033	Pseudoalteromonas sp.	Pseudoalteromonas sp. TW-7
691	clique	2540341038	Pseudoalteromonas sp.	Pseudoalteromonas sp. BSi20480
691	clique	2541047158	Pseudoalteromonas marina	Pseudoalteromonas marina mano4
692	clique	2531839499	Anoxybacillus sp.	Anoxybacillus sp. DT3-1
692	clique	2551306365	Geobacillus caldoxylosilyticus	Geobacillus caldoxylosilyticus G10
699	clique	2519899670	Nocardiopsis sp.	Nocardiopsis sp. CNS639
699	clique	646564557	Nocardiopsis dassonvillei	Nocardiopsis dassonvillei dassonvillei DSM 43111
701	clique	2534682194	Burkholderia sp.	Burkholderia sp. BT03
701	clique	2537561811	Burkholderia terrae	Burkholderia terrae BS001
702	clique	2512875014	Desulfitobacterium sp.	Desulfitobacterium sp. PCE1, DSM 10344
702	clique	2507262031	Desulfitobacterium dehalogenans	Desulfitobacterium dehalogenans JW/IU-DC1, ATCC 51507
709	clique	650716032	Delftia sp.	Delftia sp. Cs1-4
709	clique	641228489	Delftia acidovorans	Delftia acidovorans SPH-1
712	clique	2551306544	Mycobacterium sp.	Mycobacterium sp. VKM Ac-1817D
712	clique	2519899744	Mycobacterium fortuitum	Mycobacterium fortuitum fortuitum DSM 46621
720	clique	2551306111	Pseudoalteromonas sp.	Pseudoalteromonas sp. NJ631
720	clique	2519899641	Pseudoalteromonas piscicida	Pseudoalteromonas piscicida ATCC 15057
720	clique	2541047159	Pseudoalteromonas piscicida	Pseudoalteromonas piscicida JCM 20779
724	clique	646206256	Citrobacter sp.	Citrobacter sp. 30_2
724	clique	2513237266	Citrobacter freundii	Citrobacter freundii 4_7_47CFAA
734	clique	2510917027	Brevibacillus sp.	Brevibacillus sp. CF112
734	clique	2524023183	Brevibacillus agri	Brevibacillus agri BAB-2500
735	clique	642555106	Anaeromyxobacter sp.	Anaeromyxobacter sp. K
735	clique	643348507	Anaeromyxobacter dehalogenans	Anaeromyxobacter dehalogenans 2CP-1
738	clique	641522631	Halobacterium salinarum	Halobacterium salinarum R1, DSM 671
738	clique	638154504	Halobacterium sp.	Halobacterium sp. NRC-1
739	clique	2540341240	Microbacterium sp.	Microbacterium sp. KROCY2
739	clique	2519899570	Kocuria palustris	Kocuria palustris TAGA27
745	clique	650716094	Tepidanaerobacter sp.	Tepidanaerobacter sp. Re1
745	clique	2540341113	Tepidanaerobacter acetatoxydans	Tepidanaerobacter acetatoxydans Re1
751	clique	2537562058	Acinetobacter sp.	Acinetobacter sp. WC-743
751	clique	2547132297	Acinetobacter berezeniae	Acinetobacter bereziniae Brisou 64, LMG 1003
762	clique	646206274	Bacteroides sp.	Bacteroides sp. 4_3_47FAA
762	clique	649989913	Bacteroides sp.	Bacteroides sp. 3_1_40A
762	clique	640753008	Bacteroides vulgatus	Bacteroides vulgatus ATCC 8482
762	clique	647000214	Bacteroides vulgatus	Bacteroides vulgatus PC510
762	clique	2503754051	Bacteroides vulgatus	Bacteroides vulgatus 274-1D4 (mouse strain)
762	clique	2510065017	Bacteroides vulgatus	Bacteroides vulgatus RJ2H1
762	clique	2510065018	Bacteroides vulgatus	Bacteroides vulgatus RJ2L3
762	clique	2537561756	Bacteroides vulgatus	Bacteroides vulgatus CL09T03C04
774	clique	2519899750	Rhodobacter sp.	Rhodobacter sp. AKP1
774	clique	640069327	Rhodobacter sphaeroides	Rhodobacter sphaeroides 2.4.1, ATCC BAA-808
774	clique	640069328	Rhodobacter sphaeroides	Rhodobacter sphaeroides ATCC 17029
774	clique	643348570	Rhodobacter sphaeroides	Rhodobacter sphaeroides KD131
774	clique	651324095	Rhodobacter sphaeroides	Rhodobacter sphaeroides WS8N
774	clique	2512875023	Rhodobacter sphaeroides	Rhodobacter sphaeroides 2.4.1
775	clique	2519103095	Burkholderia sp.	Burkholderia sp. KJ006
775	clique	640069307	Burkholderia vietnamiensis	Burkholderia vietnamiensis G4
782	clique	2528768092	Ralstonia sp.	Ralstonia sp. JGI 0001001-B07 (contamination screened)
782	clique	2528768093	Ralstonia sp.	Ralstonia sp. JGI 0001001-C06 (contamination screened)
782	clique	2528768035	unclassified	beta proteobacterium JGI 0001001-A11 (contamination screened)
782	clique	642555151	Ralstonia pickettii	Ralstonia pickettii 12J
782	clique	2522125080	Ralstonia pickettii	Ralstonia pickettii OR214
783	clique	2507149004	Alcanivorax sp.	Alcanivorax sp.
783	clique	2507149008	Alcanivorax sp.	Alcanivorax sp. sk2-jrc
783	clique	637000004	Alcanivorax borkumensis	Alcanivorax borkumensis SK2
784	clique	2518645600	Pseudomonas sp.	Pseudomonas sp. URMO17WK12:I1
784	clique	2523533528	Pseudomonas sp.	Pseudomonas sp. URMO17WK12:I4
784	clique	2523533568	Pseudomonas sp.	Pseudomonas sp. URMO17WK12:I3

784	clique	2505679082	Pseudomonas fulva	Pseudomonas fulva 12-X
786	clique	2531839272	Prevotella sp.	Prevotella sp. MSX73
786	clique	647533195	Prevotella buccae	Prevotella buccae D17
786	clique	649989986	Prevotella buccae	Prevotella buccae ATCC 33574
802	clique	2517093029	Natrinema sp.	Natrinema sp. J7-2
802	clique	2554235484	Natrinema gari	Natrinema gari JCM 14663
804	clique	2511231129	Vibrio sp.	Vibrio sp. EJY3
804	clique	2554235216	Vibrio natriegens	Vibrio natriegens RF-FC 7
811	clique	2554235460	Psychrobacter sp.	Psychrobacter sp. G
811	clique	637000227	Psychrobacter cryohalolentis	Psychrobacter cryohalolentis K5
813	clique	649989910	Bacillus sp.	Bacillus sp. BT1B_CT2
813	clique	639279303	Bacillus licheniformis	Bacillus licheniformis DSM 13 Goettingen
813	clique	639279304	Bacillus licheniformis	Bacillus licheniformis DSM 13 Novozymes
813	clique	2519899833	Bacillus licheniformis	Bacillus licheniformis WX-02
813	clique	2548877019	Bacillus licheniformis	Bacillus licheniformis 10-1-A
813	clique	2548877020	Bacillus licheniformis	Bacillus licheniformis 5-2-D
813	clique	2551306495	Bacillus licheniformis	Bacillus licheniformis CGMCC 3963
816	clique	2548876977	Rhodococcus sp.	Rhodococcus sp. P14
816	clique	2554235023	Rhodococcus ruber	Rhodococcus ruber Chol-4
819	clique	642791603	Geobacillus sp.	Geobacillus sp. G11MC16
819	clique	640069312	Geobacillus thermodenitrificans	Geobacillus thermodenitrificans NG80-2
819	clique	2554235472	Geobacillus thermodenitrificans	Geobacillus thermodenitrificans DSM 465
821	clique	2513237386	Actinomyces sp.	Actinomyces sp. F0330
821	clique	2541047505	Actinomyces johnsonii	Actinomyces johnsonii F0510
821	clique	2541048002	Actinomyces johnsonii	Actinomyces johnsonii F0542
830	clique	641736270	Clostridium sp.	Clostridium sp. SS2/1
830	clique	649989957	unclassified	Lachnospiraceae bacterium sp. 5_1_63FAA
830	clique	650377990	unclassified	Clostridiales sp. SSC/2
830	clique	2534681714	Anaerostipes hadrus	Anaerostipes hadrus comb. nov. VPI 82-52, DSM 3319
834	clique	646206272	Bacteroides sp.	Bacteroides sp. 1_1_6
834	clique	648861000	Bacteroides sp.	Bacteroides sp. 1_1_14
834	clique	637000026	Bacteroides thetaiotaomicron	Bacteroides thetaiotaomicron VPI-5482
834	clique	2513020028	Bacteroides thetaiotaomicron	Bacteroides thetaiotaomicron NLAE-zl-C523 (Illumina Draft assembly with Velvet)
834	clique	2513020029	Bacteroides thetaiotaomicron	Bacteroides thetaiotaomicron NLAE-zl-P32 (Illumina Draft assembly with Velvet)
834	clique	2513020032	Bacteroides thetaiotaomicron	Bacteroides thetaiotaomicron NLAE-zl-P699
834	clique	2514885004	Bacteroides thetaiotaomicron	Bacteroides thetaiotaomicron NLAE-zl-G288
834	clique	2515154063	Bacteroides thetaiotaomicron	Bacteroides thetaiotaomicron NLAE-zl-H207
834	clique	2515154066	Bacteroides thetaiotaomicron	Bacteroides thetaiotaomicron NLAE-zl-H463
834	clique	2515154067	Bacteroides thetaiotaomicron	Bacteroides thetaiotaomicron NLAE-zl-H492
843	clique	2551306406	Vibrio sp.	Vibrio sp. 712i1
843	clique	638341222	Vibrio alginolyticus	Vibrio alginolyticus 12G01
843	clique	647000332	Vibrio alginolyticus	Vibrio alginolyticus 40B
844	clique	651324008	Agrobacterium sp.	Agrobacterium sp. ATCC 31749
844	clique	639279301	Agrobacterium tumefaciens	Agrobacterium tumefaciens C58-UWash
844	clique	639279302	Agrobacterium tumefaciens	Agrobacterium tumefaciens C58-UWash
847	clique	649989921	Clostridium sp.	Clostridium sp. HGF2
847	clique	2541047402	Clostridium sp.	Clostridium difficile P28
847	clique	2513237332	unclassified	Erysipelotrichaceae bacterium sp. 21_3
847	clique	2513237345	unclassified	Erysipelotrichaceae sp. 2_2_44A
847	clique	2531839268	unclassified	Erysipelotrichaceae bacterium sp. 6_1_45
847	clique	2534682210	Clostridium innocuum	Clostridium innocuum 2959
858	clique	646206253	Fusobacterium sp.	Fusobacterium sp. 4_1_13
858	clique	647533169	Fusobacterium sp.	Fusobacterium sp. 3_1_27
858	clique	647533171	Fusobacterium sp.	Fusobacterium sp. 3_1_36A2
858	clique	2554235746	Fusobacterium sp.	Fusobacterium sp. 3_1_36A2
858	clique	638341097	Fusobacterium nucleatum	Fusobacterium nucleatum vincentii ATCC 49256
858	clique	2519899747	Fusobacterium nucleatum	Fusobacterium nucleatum fusiforme ATCC 51190
858	clique	2554235041	Fusobacterium nucleatum	Fusobacterium nucleatum vincentii ChDC F8
875	clique	2524614673	Streptomyces sp.	Streptomyces sp. PgraA7
875	clique	645058822	Streptomyces roseosporus	Streptomyces roseosporus NRRL 15998
875	clique	645058827	Streptomyces roseosporus	Streptomyces roseosporus NRRL 11379
885	clique	651324049	unclassified	Lachnospiraceae bacterium 2_1_58FAA
885	clique	640963057	Ruminococcus gnavus	Ruminococcus gnavus ATCC 29149
886	clique	649989909	Bacillus sp.	Bacillus sp. 2_A_57_CT2
886	clique	2551306096	Bacillus oceanisediminis	Bacillus oceanisediminis 2691
896	clique	2524614873	Teredinibacter sp.	Teredinibacter sp. 991H.S.0a.06
896	clique	2513237099	Teredinibacter turnerae	Teredinibacter turnerae T7902
896	clique	2519899663	Teredinibacter turnerae	Teredinibacter turnerae T0609
896	clique	2519899664	Teredinibacter turnerae	Teredinibacter turnerae T8412
896	clique	2523533596	Teredinibacter turnerae	Teredinibacter turnerae T8513
897	clique	647533203	unclassified	Rhodobacterales sp. Y4I
897	clique	2521172619	Phaeobacter daeponensis	Phaeobacter daeponensis TF-218, DSM 23529 (scaffold version)
902	clique	2551306348	Dickeya sp.	Dickeya chrysanthemi NCPPB 516
902	clique	644736355	Dickeya zeae	Dickeya zeae Ech1591
908	clique	2548876840	Anaerococcus sp.	Anaerococcus sp. ph10
908	clique	645058761	Anaerococcus vaginalis	Anaerococcus vaginalis ATCC 51170
912	clique	2551306658	unclassified	Herbaspirillum sp. B39
912	clique	2551306420	Herbaspirillum huttiense	Herbaspirillum huttiense putei IAM 15032
938	clique	2513237367	Clostridium sp.	Clostridium sp. 7_3_54FAA
938	clique	649989922	Clostridium symbiosum	Clostridium symbiosum WAL-14163
938	clique	649989923	Clostridium symbiosum	Clostridium symbiosum WAL-14673
945	clique	2510065064	Pseudoxanthomonas sp.	Pseudoxanthomonas sp. J31
945	clique	2551306371	Pseudoxanthomonas sp.	Pseudoxanthomonas sp. GW2
945	clique	2505679064	Pseudoxanthomonas taiwanensis	Pseudoxanthomonas taiwanensis J19
948	clique	2548877171	Bacteroides sp.	Bacteroides sp. 14(A)

948	clique	643886111	Bacteroides cellulosilyticus	Bacteroides cellulosilyticus DSM 14838
948	clique	2531839301	Bacteroides cellulosilyticus	Bacteroides cellulosilyticus CL02T12C19
955	clique	639633003	Acidovorax sp.	Acidovorax sp. JS42
955	clique	643348541	Acidovorax ebreus	Acidovorax ebreus TPSY
958	clique	2534682224	Lactobacillus sp.	Lactobacillus sp. 66c
958	clique	2537562121	Lactobacillus equicursoris	Lactobacillus equicursoris CIP 110162
960	clique	2541047028	Ralstonia sp.	Ralstonia sp. GA3-3
960	clique	640427136	Ralstonia eutropha	Ralstonia eutropha H16
978	clique	649633068	Methylovorus sp.	Methylovorus sp. MP688
978	clique	644736389	Methylovorus glucosetrophus	Methylovorus glucosetrophus SIP3-4
993	clique	645951850	Fusobacterium sp.	Fusobacterium sp. D12
993	clique	2531839544	Fusobacterium necrophorum	Fusobacterium necrophorum Fnf1007
993	clique	2537561963	Fusobacterium necrophorum	Fusobacterium necrophorum funduliforme ATCC 51357
995	clique	2548876792	Xanthomonas sp.	Xanthomonas sp. NCPPB 1132
995	clique	2548876789	Xanthomonas sacchari	Xanthomonas sacchari NCPPB 4393
1009	clique	2545824673	Enterococcus sp.	Enterococcus flavescens ATCC 49996
1009	clique	647533127	Enterococcus casseliflavus	Enterococcus casseliflavus EC10
1009	clique	647533128	Enterococcus casseliflavus	Enterococcus casseliflavus EC20
1009	clique	647533129	Enterococcus casseliflavus	Enterococcus casseliflavus EC30
1009	clique	2540341159	Enterococcus casseliflavus	Enterococcus casseliflavus EC20
1009	clique	2541047578	Enterococcus casseliflavus	Enterococcus casseliflavus 14-MB-W-14
