# Supplementary material for: Microbial species delineation using whole genome sequences
Source: Nucleic Acids Res. 2015 Jul 6;43(14):6761–71. doi: 10.1093/nar/gkv657 (PMC4538840; doi:10.1093/nar/gkv657)
Supplement: SUPPLEMENTARY DATA [file supp_gkv657_nar-03569-n-2014-File008.pdf]

Dataset S2: 109 cliques and 4 clique groups that are populated entirely by genomes without any species definitions are putative novel species

Cluster ID	Cluster type	Species present in clique (number of genomes of each species)
8	clique-group	unclassified(3)
14	clique	Thioalkalivibrio sp.(11)
23	clique	Thioalkalivibrio sp.(3)
31	clique	Burkholderia sp.(2)
65	clique	Streptomyces sp.(5)
90	clique	Streptomyces sp.(3)
101	clique	Ancylobacter sp.(1),unclassified(1)
105	clique	Eubacterium sp.(2)
112	clique	unclassified(2)
117	clique	Pseudoalteromonas sp.(2)
139	clique	Burkholderia sp.(2)
156	clique	unclassified(1),Sulfurimonas sp.(1)
179	clique	unclassified(2)
193	clique	Rhodococcus sp.(2)
196	clique	Thioalkalivibrio sp.(2)
197	clique	Selenomonas sp.(2)
199	clique	Pseudomonas sp.(3)
207	clique	Butyrivibrio sp.(2)
210	clique	Thioalkalivibrio sp.(3)
228	clique	Rhizobium sp.(3)
234	clique	Thioalkalivibrio sp.(11)
283	clique	Pseudoalteromonas sp.(3)
296	clique	unclassified(2),Coprococcus sp.(2)
306	clique	Pseudomonas sp.(3)
309	clique	unclassified(2)
321	clique	Pseudoalteromonas sp.(2)
338	clique	Mycobacterium sp.(3)
347	clique	unclassified(2)
348	clique	Arthrobacter sp.(2)
372	clique	Streptomyces sp.(4)
375	clique	unclassified(2)
385	clique	Bacillus sp.(2)
388	clique	Methylophilus sp.(2)
391	clique	Paenibacillus sp.(2)
392	clique	Cyanothece sp.(2)
398	clique	Enterobacter sp.(2)
401	clique	Dehalobacter sp.(2)
403	clique	Methanobrevibacter sp.(2)

405	clique	Pseudomonas sp.(2)
418	clique	unclassified(2)
424	clique	unclassified(2)
429	clique	Streptomyces sp.(5)
457	clique	Actinomyces sp.(2)
461	clique	Hyphomicrobium sp.(2)
466	clique	Vibrio sp.(4)
467	clique-group	Streptococcus sp.(6)
485	clique	Butyrivibrio sp.(2)
494	clique	Blattabacterium sp.(2)
497	clique	Synechocystis sp.(6)
499	clique	Nocardioides sp.(2)
514	clique	Sulfitobacter sp.(2)
520	clique	Thioalkalivibrio sp.(12)
521	clique	unclassified(1),Methylotenera sp.(1)
537	clique-group	unclassified(10)
545	clique	unclassified(4)
556	clique	unclassified(2)
565	clique	Thioalkalivibrio sp.(7)
579	clique	Streptococcus sp.(2)
592	clique-group	unclassified(6)
596	clique	Streptomyces sp.(3)
601	clique	Butyrivibrio sp.(2)
624	clique	Actinomyces sp.(1),Atopobium sp.(1)
625	clique	Agrobacterium sp.(3)
647	clique	Aminobacter sp.(3)
649	clique	Thauera sp.(2)
657	clique	unclassified(2)
660	clique	Mycobacterium sp.(2)
675	clique	Shewanella sp.(2)
678	clique	Butyrivibrio sp.(2)
708	clique	unclassified(2)
711	clique	Burkholderia sp.(2)
722	clique	Bradyrhizobium sp.(2)
730	clique	unclassified(2)
748	clique	Pseudomonas sp.(3)
760	clique	Clostridium sp.(1),Candidatus Arthromitus sp.(5)
763	clique	Pseudomonas sp.(2)
772	clique	Pseudomonas sp.(7)
776	clique	Paracoccus sp.(3)
790	clique	Hydrogenobaculum sp.(4)

792	clique	unclassified(2)
808	clique	Xanthomonas sp.(3)
809	clique	Bacillus sp.(2)
814	clique	Synechococcus sp.(4)
838	clique	Thioalkalivibrio sp.(3)
850	clique	Blattabacterium sp.(2)
853	clique	unclassified(1),Eubacterium sp.(1)
854	clique	Thioalkalivibrio sp.(2)
862	clique	Pseudomonas sp.(4)
865	clique	Olleya sp.(2)
872	clique	Rhodanobacter sp.(4)
878	clique	Pseudomonas sp.(2)
884	clique	Methylobacterium sp.(2)
914	clique	Methyloversatilis sp.(1),unclassified(1)
918	clique	unclassified(2)
921	clique	Oribacterium sp.(2)
922	clique	unclassified(2)
923	clique	Paracoccus sp.(2)
931	clique	unclassified(2)
934	clique	Thioalkalivibrio sp.(2)
935	clique	Methylobacterium sp.(2)
939	clique	unclassified(1),Chloroflexus sp.(1)
944	clique	Streptomyces sp.(2)
949	clique	Eubacteriaceae bacterium sp.(1),Eubacterium sp.(1)
951	clique	Arenimonas sp.(1),Rhizobacter sp.(1),Lysobacter sp.(1)
961	clique	Ralstonia sp.(2)
963	clique	Cyanothece sp.(2)
972	clique	Ruminococcus sp.(1),Blautia sp.(1)
975	clique	Luteimonas sp.(2)
983	clique	Arcobacter sp.(2)
987	clique	Desulfovibrio sp.(2)
996	clique	Pseudomonas sp.(2)
1011	clique	Coprobacillus sp.(2)
1012	clique	Cryocola sp.(1),Leifsonia sp.(1)
