# Supplementary material for: Microbial species delineation using whole genome sequences
Source: Nucleic Acids Res. 2015 Jul 6;43(14):6761–71. doi: 10.1093/nar/gkv657 (PMC4538840; doi:10.1093/nar/gkv657)
Supplement: SUPPLEMENTARY DATA [file supp_gkv657_nar-03569-n-2014-File009.pdf]

Dataset S3: This table provided the ability to determine whether two genomes with pairwise AF=a and gANI=b belong to the same species and has been computed using empirical probabilities from domain wide data

$P_r^{intra\text{-}species}[AF=a,ANI=b]=P_r^{intra\text{-}species}[AF=a]*P_r^{intra\text{-}species}[ANI=b|AF=a]$

AF=a,ANI=b	$P_r^{intra\text{-}species}[AF=a,ANI=b]$
0.00,0.0	0
0.00,0.1	0
0.00,0.2	0
0.00,0.3	0
0.00,0.4	0
0.00,0.5	0
0.00,0.6	0
0.00,0.7	0
0.00,0.8	0
0.00,0.9	0
0.00,1.0	0
0.00,1.1	0
0.00,1.2	0
0.00,1.3	0
0.00,1.4	0
0.00,1.5	0
0.00,1.6	0
0.00,1.7	0
0.00,1.8	0
0.00,1.9	0
0.00,2.0	0
0.00,2.1	0
0.00,2.2	0
0.00,2.3	0
0.00,2.4	0
0.00,2.5	0
0.00,2.6	0
0.00,2.7	0
0.00,2.8	0
0.00,2.9	0
0.00,3.0	0
0.00,3.1	0
0.00,3.2	0
0.00,3.3	0

0.00,3.4	0
0.00,3.5	0
0.00,3.6	0
0.00,3.7	0
0.00,3.8	0
0.00,3.9	0
0.00,4.0	0
0.00,4.1	0
0.00,4.2	0
0.00,4.3	0
0.00,4.4	0
0.00,4.5	0
0.00,4.6	0
0.00,4.7	0
0.00,4.8	0
0.00,4.9	0
0.00,5.0	0
0.00,5.1	0
0.00,5.2	0
0.00,5.3	0
0.00,5.4	0
0.00,5.5	0
0.00,5.6	0
0.00,5.7	0
0.00,5.8	0
0.00,5.9	0
0.00,6.0	0
0.00,6.1	0
0.00,6.2	0
0.00,6.3	0
0.00,6.4	0
0.00,6.5	0
0.00,6.6	0
0.00,6.7	0
0.00,6.8	0
0.00,6.9	0
0.00,7.0	0
0.00,7.1	0
0.00,7.2	0
0.00,7.3	0
0.00,7.4	0
0.00,7.5	0

0.00,7.6	0
0.00,7.7	0
0.00,7.8	0
0.00,7.9	0
0.00,8.0	0
0.00,8.1	0
0.00,8.2	0
0.00,8.3	0
0.00,8.4	0
0.00,8.5	0
0.00,8.6	0
0.00,8.7	0
0.00,8.8	0
0.00,8.9	0
0.00,9.0	0
0.00,9.1	0
0.00,9.2	0
0.00,9.3	0
0.00,9.4	0
0.00,9.5	0
0.00,9.6	0
0.00,9.7	0
0.00,9.8	0
0.00,9.9	0
0.00,10.0	0
0.00,10.1	0
0.00,10.2	0
0.00,10.3	0
0.00,10.4	0
0.00,10.5	0
0.00,10.6	0
0.00,10.7	0
0.00,10.8	0
0.00,10.9	0
0.00,11.0	0
0.00,11.1	0
0.00,11.2	0
0.00,11.3	0
0.00,11.4	0
0.00,11.5	0
0.00,11.6	0
0.00,11.7	0

0.00,11.8	0
0.00,11.9	0
0.00,12.0	0
0.00,12.1	0
0.00,12.2	0
0.00,12.3	0
0.00,12.4	0
0.00,12.5	0
0.00,12.6	0
0.00,12.7	0
0.00,12.8	0
0.00,12.9	0
0.00,13.0	0
0.00,13.1	0
0.00,13.2	0
0.00,13.3	0
0.00,13.4	0
0.00,13.5	0
0.00,13.6	0
0.00,13.7	0
0.00,13.8	0
0.00,13.9	0
0.00,14.0	0
0.00,14.1	0
0.00,14.2	0
0.00,14.3	0
0.00,14.4	0
0.00,14.5	0
0.00,14.6	0
0.00,14.7	0
0.00,14.8	0
0.00,14.9	0
0.00,15.0	0
0.00,15.1	0
0.00,15.2	0
0.00,15.3	0
0.00,15.4	0
0.00,15.5	0
0.00,15.6	0
0.00,15.7	0
0.00,15.8	0
0.00,15.9	0

0.00,16.0	0
0.00,16.1	0
0.00,16.2	0
0.00,16.3	0
0.00,16.4	0
0.00,16.5	0
0.00,16.6	0
0.00,16.7	0
0.00,16.8	0
0.00,16.9	0
0.00,17.0	0
0.00,17.1	0
0.00,17.2	0
0.00,17.3	0
0.00,17.4	0
0.00,17.5	0
0.00,17.6	0
0.00,17.7	0
0.00,17.8	0
0.00,17.9	0
0.00,18.0	0
0.00,18.1	0
0.00,18.2	0
0.00,18.3	0
0.00,18.4	0
0.00,18.5	0
0.00,18.6	0
0.00,18.7	0
0.00,18.8	0
0.00,18.9	0
0.00,19.0	0
0.00,19.1	0
0.00,19.2	0
0.00,19.3	0
0.00,19.4	0
0.00,19.5	0
0.00,19.6	0
0.00,19.7	0
0.00,19.8	0
0.00,19.9	0
0.00,20.0	0
0.00,20.1	0

0.00,20.2	0
0.00,20.3	0
0.00,20.4	0
0.00,20.5	0
0.00,20.6	0
0.00,20.7	0
0.00,20.8	0
0.00,20.9	0
0.00,21.0	0
0.00,21.1	0
0.00,21.2	0
0.00,21.3	0
0.00,21.4	0
0.00,21.5	0
0.00,21.6	0
0.00,21.7	0
0.00,21.8	0
0.00,21.9	0
0.00,22.0	0
0.00,22.1	0
0.00,22.2	0
0.00,22.3	0
0.00,22.4	0
0.00,22.5	0
0.00,22.6	0
0.00,22.7	0
0.00,22.8	0
0.00,22.9	0
0.00,23.0	0
0.00,23.1	0
0.00,23.2	0
0.00,23.3	0
0.00,23.4	0
0.00,23.5	0
0.00,23.6	0
0.00,23.7	0
0.00,23.8	0
0.00,23.9	0
0.00,24.0	0
0.00,24.1	0
0.00,24.2	0
0.00,24.3	0

0.00,24.4	0
0.00,24.5	0
0.00,24.6	0
0.00,24.7	0
0.00,24.8	0
0.00,24.9	0
0.00,25.0	0
0.00,25.1	0
0.00,25.2	0
0.00,25.3	0
0.00,25.4	0
0.00,25.5	0
0.00,25.6	0
0.00,25.7	0
0.00,25.8	0
0.00,25.9	0
0.00,26.0	0
0.00,26.1	0
0.00,26.2	0
0.00,26.3	0
0.00,26.4	0
0.00,26.5	0
0.00,26.6	0
0.00,26.7	0
0.00,26.8	0
0.00,26.9	0
0.00,27.0	0
0.00,27.1	0
0.00,27.2	0
0.00,27.3	0
0.00,27.4	0
0.00,27.5	0
0.00,27.6	0
0.00,27.7	0
0.00,27.8	0
0.00,27.9	0
0.00,28.0	0
0.00,28.1	0
0.00,28.2	0
0.00,28.3	0
0.00,28.4	0
0.00,28.5	0

0.00,28.6	0
0.00,28.7	0
0.00,28.8	0
0.00,28.9	0
0.00,29.0	0
0.00,29.1	0
0.00,29.2	0
0.00,29.3	0
0.00,29.4	0
0.00,29.5	0
0.00,29.6	0
0.00,29.7	0
0.00,29.8	0
0.00,29.9	0
0.00,30.0	0
0.00,30.1	0
0.00,30.2	0
0.00,30.3	0
0.00,30.4	0
0.00,30.5	0
0.00,30.6	0
0.00,30.7	0
0.00,30.8	0
0.00,30.9	0
0.00,31.0	0
0.00,31.1	0
0.00,31.2	0
0.00,31.3	0
0.00,31.4	0
0.00,31.5	0
0.00,31.6	0
0.00,31.7	0
0.00,31.8	0
0.00,31.9	0
0.00,32.0	0
0.00,32.1	0
0.00,32.2	0
0.00,32.3	0
0.00,32.4	0
0.00,32.5	0
0.00,32.6	0
0.00,32.7	0

0.00,32.8	0
0.00,32.9	0
0.00,33.0	0
0.00,33.1	0
0.00,33.2	0
0.00,33.3	0
0.00,33.4	0
0.00,33.5	0
0.00,33.6	0
0.00,33.7	0
0.00,33.8	0
0.00,33.9	0
0.00,34.0	0
0.00,34.1	0
0.00,34.2	0
0.00,34.3	0
0.00,34.4	0
0.00,34.5	0
0.00,34.6	0
0.00,34.7	0
0.00,34.8	0
0.00,34.9	0
0.00,35.0	0
0.00,35.1	0
0.00,35.2	0
0.00,35.3	0
0.00,35.4	0
0.00,35.5	0
0.00,35.6	0
0.00,35.7	0
0.00,35.8	0
0.00,35.9	0
0.00,36.0	0
0.00,36.1	0
0.00,36.2	0
0.00,36.3	0
0.00,36.4	0
0.00,36.5	0
0.00,36.6	0
0.00,36.7	0
0.00,36.8	0
0.00,36.9	0

0.00,37.0	0
0.00,37.1	0
0.00,37.2	0
0.00,37.3	0
0.00,37.4	0
0.00,37.5	0
0.00,37.6	0
0.00,37.7	0
0.00,37.8	0
0.00,37.9	0
0.00,38.0	0
0.00,38.1	0
0.00,38.2	0
0.00,38.3	0
0.00,38.4	0
0.00,38.5	0
0.00,38.6	0
0.00,38.7	0
0.00,38.8	0
0.00,38.9	0
0.00,39.0	0
0.00,39.1	0
0.00,39.2	0
0.00,39.3	0
0.00,39.4	0
0.00,39.5	0
0.00,39.6	0
0.00,39.7	0
0.00,39.8	0
0.00,39.9	0
0.00,40.0	0
0.00,40.1	0
0.00,40.2	0
0.00,40.3	0
0.00,40.4	0
0.00,40.5	0
0.00,40.6	0
0.00,40.7	0
0.00,40.8	0
0.00,40.9	0
0.00,41.0	0
0.00,41.1	0

0.00,41.2	0
0.00,41.3	0
0.00,41.4	0
0.00,41.5	0
0.00,41.6	0
0.00,41.7	0
0.00,41.8	0
0.00,41.9	0
0.00,42.0	0
0.00,42.1	0
0.00,42.2	0
0.00,42.3	0
0.00,42.4	0
0.00,42.5	0
0.00,42.6	0
0.00,42.7	0
0.00,42.8	0
0.00,42.9	0
0.00,43.0	0
0.00,43.1	0
0.00,43.2	0
0.00,43.3	0
0.00,43.4	0
0.00,43.5	0
0.00,43.6	0
0.00,43.7	0
0.00,43.8	0
0.00,43.9	0
0.00,44.0	0
0.00,44.1	0
0.00,44.2	0
0.00,44.3	0
0.00,44.4	0
0.00,44.5	0
0.00,44.6	0
0.00,44.7	0
0.00,44.8	0
0.00,44.9	0
0.00,45.0	0
0.00,45.1	0
0.00,45.2	0
0.00,45.3	0

0.00,45.4	0
0.00,45.5	0
0.00,45.6	0
0.00,45.7	0
0.00,45.8	0
0.00,45.9	0
0.00,46.0	0
0.00,46.1	0
0.00,46.2	0
0.00,46.3	0
0.00,46.4	0
0.00,46.5	0
0.00,46.6	0
0.00,46.7	0
0.00,46.8	0
0.00,46.9	0
0.00,47.0	0
0.00,47.1	0
0.00,47.2	0
0.00,47.3	0
0.00,47.4	0
0.00,47.5	0
0.00,47.6	0
0.00,47.7	0
0.00,47.8	0
0.00,47.9	0
0.00,48.0	0
0.00,48.1	0
0.00,48.2	0
0.00,48.3	0
0.00,48.4	0
0.00,48.5	0
0.00,48.6	0
0.00,48.7	0
0.00,48.8	0
0.00,48.9	0
0.00,49.0	0
0.00,49.1	0
0.00,49.2	0
0.00,49.3	0
0.00,49.4	0
0.00,49.5	0

0.00,49.6	0
0.00,49.7	0
0.00,49.8	0
0.00,49.9	0
0.00,50.0	0
0.00,50.1	0
0.00,50.2	0
0.00,50.3	0
0.00,50.4	0
0.00,50.5	0
0.00,50.6	0
0.00,50.7	0
0.00,50.8	0
0.00,50.9	0
0.00,51.0	0
0.00,51.1	0
0.00,51.2	0
0.00,51.3	0
0.00,51.4	0
0.00,51.5	0
0.00,51.6	0
0.00,51.7	0
0.00,51.8	0
0.00,51.9	0
0.00,52.0	0
0.00,52.1	0
0.00,52.2	0
0.00,52.3	0
0.00,52.4	0
0.00,52.5	0
0.00,52.6	0
0.00,52.7	0
0.00,52.8	0
0.00,52.9	0
0.00,53.0	0
0.00,53.1	0
0.00,53.2	0
0.00,53.3	0
0.00,53.4	0
0.00,53.5	0
0.00,53.6	0
0.00,53.7	0

0.00,53.8	0
0.00,53.9	0
0.00,54.0	0
0.00,54.1	0
0.00,54.2	0
0.00,54.3	0
0.00,54.4	0
0.00,54.5	0
0.00,54.6	0
0.00,54.7	0
0.00,54.8	0
0.00,54.9	0
0.00,55.0	0
0.00,55.1	0
0.00,55.2	0
0.00,55.3	0
0.00,55.4	0
0.00,55.5	0
0.00,55.6	0
0.00,55.7	0
0.00,55.8	0
0.00,55.9	0
0.00,56.0	0
0.00,56.1	0
0.00,56.2	0
0.00,56.3	0
0.00,56.4	0
0.00,56.5	0
0.00,56.6	0
0.00,56.7	0
0.00,56.8	0
0.00,56.9	0
0.00,57.0	0
0.00,57.1	0
0.00,57.2	0
0.00,57.3	0
0.00,57.4	0
0.00,57.5	0
0.00,57.6	0
0.00,57.7	0
0.00,57.8	0
0.00,57.9	0

0.00,58.0	0
0.00,58.1	0
0.00,58.2	0
0.00,58.3	0
0.00,58.4	0
0.00,58.5	0
0.00,58.6	0
0.00,58.7	0
0.00,58.8	0
0.00,58.9	0
0.00,59.0	0
0.00,59.1	0
0.00,59.2	0
0.00,59.3	0
0.00,59.4	0
0.00,59.5	0
0.00,59.6	0
0.00,59.7	0
0.00,59.8	0
0.00,59.9	0
0.00,60.0	0
0.00,60.1	0
0.00,60.2	0
0.00,60.3	0
0.00,60.4	0
0.00,60.5	0
0.00,60.6	0
0.00,60.7	0
0.00,60.8	0
0.00,60.9	0
0.00,61.0	0
0.00,61.1	0
0.00,61.2	0
0.00,61.3	0
0.00,61.4	0
0.00,61.5	0
0.00,61.6	0
0.00,61.7	0
0.00,61.8	0
0.00,61.9	0
0.00,62.0	0
0.00,62.1	0

0.00,62.2	0
0.00,62.3	0
0.00,62.4	0
0.00,62.5	0
0.00,62.6	0
0.00,62.7	0
0.00,62.8	0
0.00,62.9	0
0.00,63.0	0
0.00,63.1	0
0.00,63.2	0
0.00,63.3	0
0.00,63.4	0
0.00,63.5	0
0.00,63.6	0
0.00,63.7	0
0.00,63.8	0
0.00,63.9	0
0.00,64.0	0
0.00,64.1	0
0.00,64.2	0
0.00,64.3	0
0.00,64.4	0
0.00,64.5	0
0.00,64.6	0
0.00,64.7	0
0.00,64.8	0
0.00,64.9	0
0.00,65.0	0
0.00,65.1	0
0.00,65.2	0
0.00,65.3	0
0.00,65.4	0
0.00,65.5	0
0.00,65.6	0
0.00,65.7	0
0.00,65.8	0
0.00,65.9	0
0.00,66.0	0
0.00,66.1	0
0.00,66.2	0
0.00,66.3	0

0.00,66.4	0
0.00,66.5	0
0.00,66.6	0
0.00,66.7	0
0.00,66.8	0
0.00,66.9	0
0.00,67.0	0
0.00,67.1	0
0.00,67.2	0
0.00,67.3	0
0.00,67.4	0
0.00,67.5	0
0.00,67.6	0
0.00,67.7	0
0.00,67.8	0
0.00,67.9	0
0.00,68.0	0
0.00,68.1	0
0.00,68.2	0
0.00,68.3	0
0.00,68.4	0
0.00,68.5	0
0.00,68.6	0
0.00,68.7	0
0.00,68.8	0
0.00,68.9	0
0.00,69.0	0
0.00,69.1	0
0.00,69.2	0
0.00,69.3	0
0.00,69.4	0
0.00,69.5	0
0.00,69.6	0
0.00,69.7	0
0.00,69.8	0
0.00,69.9	0
0.00,70.0	0
0.00,70.1	0
0.00,70.2	0
0.00,70.3	0
0.00,70.4	0
0.00,70.5	0

0.00,70.6	0
0.00,70.7	0
0.00,70.8	0
0.00,70.9	0
0.00,71.0	0
0.00,71.1	0
0.00,71.2	0
0.00,71.3	0
0.00,71.4	0
0.00,71.5	0
0.00,71.6	0
0.00,71.7	0
0.00,71.8	0
0.00,71.9	0
0.00,72.0	0
0.00,72.1	0
0.00,72.2	0
0.00,72.3	0
0.00,72.4	0
0.00,72.5	0
0.00,72.6	0
0.00,72.7	0
0.00,72.8	0
0.00,72.9	0
0.00,73.0	0
0.00,73.1	0
0.00,73.2	0
0.00,73.3	0
0.00,73.4	0
0.00,73.5	0
0.00,73.6	0
0.00,73.7	0
0.00,73.8	0
0.00,73.9	0
0.00,74.0	0
0.00,74.1	0
0.00,74.2	0
0.00,74.3	0
0.00,74.4	0
0.00,74.5	0
0.00,74.6	0
0.00,74.7	0

0.00,74.8	0
0.00,74.9	0
0.00,75.0	0
0.00,75.1	0
0.00,75.2	0
0.00,75.3	0
0.00,75.4	0
0.00,75.5	0
0.00,75.6	0
0.00,75.7	0
0.00,75.8	0
0.00,75.9	0
0.00,76.0	0
0.00,76.1	0
0.00,76.2	0
0.00,76.3	0
0.00,76.4	0
0.00,76.5	0
0.00,76.6	0
0.00,76.7	0
0.00,76.8	0
0.00,76.9	0
0.00,77.0	0
0.00,77.1	0
0.00,77.2	0
0.00,77.3	0
0.00,77.4	0
0.00,77.5	0
0.00,77.6	0
0.00,77.7	0
0.00,77.8	0
0.00,77.9	0
0.00,78.0	0
0.00,78.1	0
0.00,78.2	0
0.00,78.3	0
0.00,78.4	0
0.00,78.5	0
0.00,78.6	0
0.00,78.7	0
0.00,78.8	0
0.00,78.9	0

0.00,79.0	0
0.00,79.1	0
0.00,79.2	0
0.00,79.3	0
0.00,79.4	0
0.00,79.5	0
0.00,79.6	0
0.00,79.7	0
0.00,79.8	0
0.00,79.9	0
0.00,80.0	0
0.00,80.1	0
0.00,80.2	0
0.00,80.3	0
0.00,80.4	0
0.00,80.5	0
0.00,80.6	0
0.00,80.7	0
0.00,80.8	0
0.00,80.9	0
0.00,81.0	0
0.00,81.1	0
0.00,81.2	0
0.00,81.3	0
0.00,81.4	0
0.00,81.5	0
0.00,81.6	0
0.00,81.7	0
0.00,81.8	0
0.00,81.9	0
0.00,82.0	0
0.00,82.1	0
0.00,82.2	0
0.00,82.3	0
0.00,82.4	0
0.00,82.5	0
0.00,82.6	0
0.00,82.7	0
0.00,82.8	0
0.00,82.9	0
0.00,83.0	0
0.00,83.1	0

0.00,83.2	0
0.00,83.3	0
0.00,83.4	0
0.00,83.5	0
0.00,83.6	0
0.00,83.7	0
0.00,83.8	0
0.00,83.9	0
0.00,84.0	0
0.00,84.1	0
0.00,84.2	0
0.00,84.3	0
0.00,84.4	0
0.00,84.5	0
0.00,84.6	0
0.00,84.7	0
0.00,84.8	0
0.00,84.9	0
0.00,85.0	0
0.00,85.1	0
0.00,85.2	0
0.00,85.3	0
0.00,85.4	0
0.00,85.5	0
0.00,85.6	0
0.00,85.7	0
0.00,85.8	0
0.00,85.9	0
0.00,86.0	0
0.00,86.1	0
0.00,86.2	0
0.00,86.3	0
0.00,86.4	0
0.00,86.5	0
0.00,86.6	0
0.00,86.7	0
0.00,86.8	0
0.00,86.9	0
0.00,87.0	0
0.00,87.1	0
0.00,87.2	0
0.00,87.3	0

0.00,87.4	0
0.00,87.5	0
0.00,87.6	0
0.00,87.7	0
0.00,87.8	0
0.00,87.9	0
0.00,88.0	0
0.00,88.1	0
0.00,88.2	0
0.00,88.3	0
0.00,88.4	0
0.00,88.5	0
0.00,88.6	0
0.00,88.7	0
0.00,88.8	0
0.00,88.9	0
0.00,89.0	0
0.00,89.1	0
0.00,89.2	0
0.00,89.3	0
0.00,89.4	0
0.00,89.5	0
0.00,89.6	0
0.00,89.7	0
0.00,89.8	0
0.00,89.9	0
0.00,90.0	0
0.00,90.1	0
0.00,90.2	0
0.00,90.3	0
0.00,90.4	0
0.00,90.5	0
0.00,90.6	0
0.00,90.7	0
0.00,90.8	0
0.00,90.9	0
0.00,91.0	0
0.00,91.1	0
0.00,91.2	0
0.00,91.3	0
0.00,91.4	0
0.00,91.5	0

0.00,91.6	0
0.00,91.7	0
0.00,91.8	0
0.00,91.9	0
0.00,92.0	0
0.00,92.1	0
0.00,92.2	0
0.00,92.3	0
0.00,92.4	0
0.00,92.5	0
0.00,92.6	0
0.00,92.7	0
0.00,92.8	0
0.00,92.9	0
0.00,93.0	0
0.00,93.1	0
0.00,93.2	0
0.00,93.3	0
0.00,93.4	0
0.00,93.5	0
0.00,93.6	0
0.00,93.7	0
0.00,93.8	0
0.00,93.9	0
0.00,94.0	0
0.00,94.1	0
0.00,94.2	0
0.00,94.3	0
0.00,94.4	0
0.00,94.5	0
0.00,94.6	0
0.00,94.7	0
0.00,94.8	0
0.00,94.9	0
0.00,95.0	0
0.00,95.1	0
0.00,95.2	0
0.00,95.3	0
0.00,95.4	0
0.00,95.5	0
0.00,95.6	0
0.00,95.7	0

0.00,95.8	0
0.00,95.9	0
0.00,96.0	0
0.00,96.1	0
0.00,96.2	0
0.00,96.3	0
0.00,96.4	0
0.00,96.5	0
0.00,96.6	0
0.00,96.7	0
0.00,96.8	0
0.00,96.9	0
0.00,97.0	0
0.00,97.1	0
0.00,97.2	0
0.00,97.3	0
0.00,97.4	0
0.00,97.5	0
0.00,97.6	0
0.00,97.7	0
0.00,97.8	0
0.00,97.9	0
0.00,98.0	0
0.00,98.1	0
0.00,98.2	0
0.00,98.3	0
0.00,98.4	0
0.00,98.5	0
0.00,98.6	0
0.00,98.7	0
0.00,98.8	0
0.00,98.9	0
0.00,99.0	0
0.00,99.1	0
0.00,99.2	0
0.00,99.3	0
0.00,99.4	0
0.00,99.5	0
0.00,99.6	0
0.00,99.7	0
0.00,99.8	0
0.00,99.9	0

0.00,100.0	0
0.01,0.0	0
0.01,0.1	0
0.01,0.2	0
0.01,0.3	0
0.01,0.4	0
0.01,0.5	0
0.01,0.6	0
0.01,0.7	0
0.01,0.8	0
0.01,0.9	0
0.01,1.0	0
0.01,1.1	0
0.01,1.2	0
0.01,1.3	0
0.01,1.4	0
0.01,1.5	0
0.01,1.6	0
0.01,1.7	0
0.01,1.8	0
0.01,1.9	0
0.01,2.0	0
0.01,2.1	0
0.01,2.2	0
0.01,2.3	0
0.01,2.4	0
0.01,2.5	0
0.01,2.6	0
0.01,2.7	0
0.01,2.8	0
0.01,2.9	0
0.01,3.0	0
0.01,3.1	0
0.01,3.2	0
0.01,3.3	0
0.01,3.4	0
0.01,3.5	0
0.01,3.6	0
0.01,3.7	0
0.01,3.8	0
0.01,3.9	0
0.01,4.0	0

0.01,4.1	0
0.01,4.2	0
0.01,4.3	0
0.01,4.4	0
0.01,4.5	0
0.01,4.6	0
0.01,4.7	0
0.01,4.8	0
0.01,4.9	0
0.01,5.0	0
0.01,5.1	0
0.01,5.2	0
0.01,5.3	0
0.01,5.4	0
0.01,5.5	0
0.01,5.6	0
0.01,5.7	0
0.01,5.8	0
0.01,5.9	0
0.01,6.0	0
0.01,6.1	0
0.01,6.2	0
0.01,6.3	0
0.01,6.4	0
0.01,6.5	0
0.01,6.6	0
0.01,6.7	0
0.01,6.8	0
0.01,6.9	0
0.01,7.0	0
0.01,7.1	0
0.01,7.2	0
0.01,7.3	0
0.01,7.4	0
0.01,7.5	0
0.01,7.6	0
0.01,7.7	0
0.01,7.8	0
0.01,7.9	0
0.01,8.0	0
0.01,8.1	0
0.01,8.2	0

0.01,8.3	0
0.01,8.4	0
0.01,8.5	0
0.01,8.6	0
0.01,8.7	0
0.01,8.8	0
0.01,8.9	0
0.01,9.0	0
0.01,9.1	0
0.01,9.2	0
0.01,9.3	0
0.01,9.4	0
0.01,9.5	0
0.01,9.6	0
0.01,9.7	0
0.01,9.8	0
0.01,9.9	0
0.01,10.0	0
0.01,10.1	0
0.01,10.2	0
0.01,10.3	0
0.01,10.4	0
0.01,10.5	0
0.01,10.6	0
0.01,10.7	0
0.01,10.8	0
0.01,10.9	0
0.01,11.0	0
0.01,11.1	0
0.01,11.2	0
0.01,11.3	0
0.01,11.4	0
0.01,11.5	0
0.01,11.6	0
0.01,11.7	0
0.01,11.8	0
0.01,11.9	0
0.01,12.0	0
0.01,12.1	0
0.01,12.2	0
0.01,12.3	0
0.01,12.4	0

0.01,12.5	0
0.01,12.6	0
0.01,12.7	0
0.01,12.8	0
0.01,12.9	0
0.01,13.0	0
0.01,13.1	0
0.01,13.2	0
0.01,13.3	0
0.01,13.4	0
0.01,13.5	0
0.01,13.6	0
0.01,13.7	0
0.01,13.8	0
0.01,13.9	0
0.01,14.0	0
0.01,14.1	0
0.01,14.2	0
0.01,14.3	0
0.01,14.4	0
0.01,14.5	0
0.01,14.6	0
0.01,14.7	0
0.01,14.8	0
0.01,14.9	0
0.01,15.0	0
0.01,15.1	0
0.01,15.2	0
0.01,15.3	0
0.01,15.4	0
0.01,15.5	0
0.01,15.6	0
0.01,15.7	0
0.01,15.8	0
0.01,15.9	0
0.01,16.0	0
0.01,16.1	0
0.01,16.2	0
0.01,16.3	0
0.01,16.4	0
0.01,16.5	0
0.01,16.6	0

0.01,16.7	0
0.01,16.8	0
0.01,16.9	0
0.01,17.0	0
0.01,17.1	0
0.01,17.2	0
0.01,17.3	0
0.01,17.4	0
0.01,17.5	0
0.01,17.6	0
0.01,17.7	0
0.01,17.8	0
0.01,17.9	0
0.01,18.0	0
0.01,18.1	0
0.01,18.2	0
0.01,18.3	0
0.01,18.4	0
0.01,18.5	0
0.01,18.6	0
0.01,18.7	0
0.01,18.8	0
0.01,18.9	0
0.01,19.0	0
0.01,19.1	0
0.01,19.2	0
0.01,19.3	0
0.01,19.4	0
0.01,19.5	0
0.01,19.6	0
0.01,19.7	0
0.01,19.8	0
0.01,19.9	0
0.01,20.0	0
0.01,20.1	0
0.01,20.2	0
0.01,20.3	0
0.01,20.4	0
0.01,20.5	0
0.01,20.6	0
0.01,20.7	0
0.01,20.8	0

0.01,20.9	0
0.01,21.0	0
0.01,21.1	0
0.01,21.2	0
0.01,21.3	0
0.01,21.4	0
0.01,21.5	0
0.01,21.6	0
0.01,21.7	0
0.01,21.8	0
0.01,21.9	0
0.01,22.0	0
0.01,22.1	0
0.01,22.2	0
0.01,22.3	0
0.01,22.4	0
0.01,22.5	0
0.01,22.6	0
0.01,22.7	0
0.01,22.8	0
0.01,22.9	0
0.01,23.0	0
0.01,23.1	0
0.01,23.2	0
0.01,23.3	0
0.01,23.4	0
0.01,23.5	0
0.01,23.6	0
0.01,23.7	0
0.01,23.8	0
0.01,23.9	0
0.01,24.0	0
0.01,24.1	0
0.01,24.2	0
0.01,24.3	0
0.01,24.4	0
0.01,24.5	0
0.01,24.6	0
0.01,24.7	0
0.01,24.8	0
0.01,24.9	0
0.01,25.0	0

0.01,25.1	0
0.01,25.2	0
0.01,25.3	0
0.01,25.4	0
0.01,25.5	0
0.01,25.6	0
0.01,25.7	0
0.01,25.8	0
0.01,25.9	0
0.01,26.0	0
0.01,26.1	0
0.01,26.2	0
0.01,26.3	0
0.01,26.4	0
0.01,26.5	0
0.01,26.6	0
0.01,26.7	0
0.01,26.8	0
0.01,26.9	0
0.01,27.0	0
0.01,27.1	0
0.01,27.2	0
0.01,27.3	0
0.01,27.4	0
0.01,27.5	0
0.01,27.6	0
0.01,27.7	0
0.01,27.8	0
0.01,27.9	0
0.01,28.0	0
0.01,28.1	0
0.01,28.2	0
0.01,28.3	0
0.01,28.4	0
0.01,28.5	0
0.01,28.6	0
0.01,28.7	0
0.01,28.8	0
0.01,28.9	0
0.01,29.0	0
0.01,29.1	0
0.01,29.2	0

0.01,29.3	0
0.01,29.4	0
0.01,29.5	0
0.01,29.6	0
0.01,29.7	0
0.01,29.8	0
0.01,29.9	0
0.01,30.0	0
0.01,30.1	0
0.01,30.2	0
0.01,30.3	0
0.01,30.4	0
0.01,30.5	0
0.01,30.6	0
0.01,30.7	0
0.01,30.8	0
0.01,30.9	0
0.01,31.0	0
0.01,31.1	0
0.01,31.2	0
0.01,31.3	0
0.01,31.4	0
0.01,31.5	0
0.01,31.6	0
0.01,31.7	0
0.01,31.8	0
0.01,31.9	0
0.01,32.0	0
0.01,32.1	0
0.01,32.2	0
0.01,32.3	0
0.01,32.4	0
0.01,32.5	0
0.01,32.6	0
0.01,32.7	0
0.01,32.8	0
0.01,32.9	0
0.01,33.0	0
0.01,33.1	0
0.01,33.2	0
0.01,33.3	0
0.01,33.4	0

0.01,33.5	0
0.01,33.6	0
0.01,33.7	0
0.01,33.8	0
0.01,33.9	0
0.01,34.0	0
0.01,34.1	0
0.01,34.2	0
0.01,34.3	0
0.01,34.4	0
0.01,34.5	0
0.01,34.6	0
0.01,34.7	0
0.01,34.8	0
0.01,34.9	0
0.01,35.0	0
0.01,35.1	0
0.01,35.2	0
0.01,35.3	0
0.01,35.4	0
0.01,35.5	0
0.01,35.6	0
0.01,35.7	0
0.01,35.8	0
0.01,35.9	0
0.01,36.0	0
0.01,36.1	0
0.01,36.2	0
0.01,36.3	0
0.01,36.4	0
0.01,36.5	0
0.01,36.6	0
0.01,36.7	0
0.01,36.8	0
0.01,36.9	0
0.01,37.0	0
0.01,37.1	0
0.01,37.2	0
0.01,37.3	0
0.01,37.4	0
0.01,37.5	0
0.01,37.6	0

0.01,37.7	0
0.01,37.8	0
0.01,37.9	0
0.01,38.0	0
0.01,38.1	0
0.01,38.2	0
0.01,38.3	0
0.01,38.4	0
0.01,38.5	0
0.01,38.6	0
0.01,38.7	0
0.01,38.8	0
0.01,38.9	0
0.01,39.0	0
0.01,39.1	0
0.01,39.2	0
0.01,39.3	0
0.01,39.4	0
0.01,39.5	0
0.01,39.6	0
0.01,39.7	0
0.01,39.8	0
0.01,39.9	0
0.01,40.0	0
0.01,40.1	0
0.01,40.2	0
0.01,40.3	0
0.01,40.4	0
0.01,40.5	0
0.01,40.6	0
0.01,40.7	0
0.01,40.8	0
0.01,40.9	0
0.01,41.0	0
0.01,41.1	0
0.01,41.2	0
0.01,41.3	0
0.01,41.4	0
0.01,41.5	0
0.01,41.6	0
0.01,41.7	0
0.01,41.8	0

0.01,41.9	0
0.01,42.0	0
0.01,42.1	0
0.01,42.2	0
0.01,42.3	0
0.01,42.4	0
0.01,42.5	0
0.01,42.6	0
0.01,42.7	0
0.01,42.8	0
0.01,42.9	0
0.01,43.0	0
0.01,43.1	0
0.01,43.2	0
0.01,43.3	0
0.01,43.4	0
0.01,43.5	0
0.01,43.6	0
0.01,43.7	0
0.01,43.8	0
0.01,43.9	0
0.01,44.0	0
0.01,44.1	0
0.01,44.2	0
0.01,44.3	0
0.01,44.4	0
0.01,44.5	0
0.01,44.6	0
0.01,44.7	0
0.01,44.8	0
0.01,44.9	0
0.01,45.0	0
0.01,45.1	0
0.01,45.2	0
0.01,45.3	0
0.01,45.4	0
0.01,45.5	0
0.01,45.6	0
0.01,45.7	0
0.01,45.8	0
0.01,45.9	0
0.01,46.0	0

0.01,46.1	0
0.01,46.2	0
0.01,46.3	0
0.01,46.4	0
0.01,46.5	0
0.01,46.6	0
0.01,46.7	0
0.01,46.8	0
0.01,46.9	0
0.01,47.0	0
0.01,47.1	0
0.01,47.2	0
0.01,47.3	0
0.01,47.4	0
0.01,47.5	0
0.01,47.6	0
0.01,47.7	0
0.01,47.8	0
0.01,47.9	0
0.01,48.0	0
0.01,48.1	0
0.01,48.2	0
0.01,48.3	0
0.01,48.4	0
0.01,48.5	0
0.01,48.6	0
0.01,48.7	0
0.01,48.8	0
0.01,48.9	0
0.01,49.0	0
0.01,49.1	0
0.01,49.2	0
0.01,49.3	0
0.01,49.4	0
0.01,49.5	0
0.01,49.6	0
0.01,49.7	0
0.01,49.8	0
0.01,49.9	0
0.01,50.0	0
0.01,50.1	0
0.01,50.2	0

0.01,50.3	0
0.01,50.4	0
0.01,50.5	0
0.01,50.6	0
0.01,50.7	0
0.01,50.8	0
0.01,50.9	0
0.01,51.0	0
0.01,51.1	0
0.01,51.2	0
0.01,51.3	0
0.01,51.4	0
0.01,51.5	0
0.01,51.6	0
0.01,51.7	0
0.01,51.8	0
0.01,51.9	0
0.01,52.0	0
0.01,52.1	0
0.01,52.2	0
0.01,52.3	0
0.01,52.4	0
0.01,52.5	0
0.01,52.6	0
0.01,52.7	0
0.01,52.8	0
0.01,52.9	0
0.01,53.0	0
0.01,53.1	0
0.01,53.2	0
0.01,53.3	0
0.01,53.4	0
0.01,53.5	0
0.01,53.6	0
0.01,53.7	0
0.01,53.8	0
0.01,53.9	0
0.01,54.0	0
0.01,54.1	0
0.01,54.2	0
0.01,54.3	0
0.01,54.4	0

0.01,54.5	0
0.01,54.6	0
0.01,54.7	0
0.01,54.8	0
0.01,54.9	0
0.01,55.0	0
0.01,55.1	0
0.01,55.2	0
0.01,55.3	0
0.01,55.4	0
0.01,55.5	0
0.01,55.6	0
0.01,55.7	0
0.01,55.8	0
0.01,55.9	0
0.01,56.0	0
0.01,56.1	0
0.01,56.2	0
0.01,56.3	0
0.01,56.4	0
0.01,56.5	0
0.01,56.6	0
0.01,56.7	0
0.01,56.8	0
0.01,56.9	0
0.01,57.0	0
0.01,57.1	0
0.01,57.2	0
0.01,57.3	0
0.01,57.4	0
0.01,57.5	0
0.01,57.6	0
0.01,57.7	0
0.01,57.8	0
0.01,57.9	0
0.01,58.0	0
0.01,58.1	0
0.01,58.2	0
0.01,58.3	0
0.01,58.4	0
0.01,58.5	0
0.01,58.6	0

0.01,58.7	0
0.01,58.8	0
0.01,58.9	0
0.01,59.0	0
0.01,59.1	0
0.01,59.2	0
0.01,59.3	0
0.01,59.4	0
0.01,59.5	0
0.01,59.6	0
0.01,59.7	0
0.01,59.8	0
0.01,59.9	0
0.01,60.0	0
0.01,60.1	0
0.01,60.2	0
0.01,60.3	0
0.01,60.4	0
0.01,60.5	0
0.01,60.6	0
0.01,60.7	0
0.01,60.8	0
0.01,60.9	0
0.01,61.0	0
0.01,61.1	0
0.01,61.2	0
0.01,61.3	0
0.01,61.4	0
0.01,61.5	0
0.01,61.6	0
0.01,61.7	0
0.01,61.8	0
0.01,61.9	0
0.01,62.0	0
0.01,62.1	0
0.01,62.2	0
0.01,62.3	0
0.01,62.4	0
0.01,62.5	0
0.01,62.6	0
0.01,62.7	0
0.01,62.8	0

0.01,62.9	0
0.01,63.0	0
0.01,63.1	0
0.01,63.2	0
0.01,63.3	0
0.01,63.4	0
0.01,63.5	0
0.01,63.6	0
0.01,63.7	0
0.01,63.8	0
0.01,63.9	0
0.01,64.0	0
0.01,64.1	0
0.01,64.2	0
0.01,64.3	0
0.01,64.4	0
0.01,64.5	0
0.01,64.6	0
0.01,64.7	0
0.01,64.8	0
0.01,64.9	0
0.01,65.0	0
0.01,65.1	0
0.01,65.2	0
0.01,65.3	0
0.01,65.4	0
0.01,65.5	0
0.01,65.6	0
0.01,65.7	0
0.01,65.8	0
0.01,65.9	0
0.01,66.0	0
0.01,66.1	0
0.01,66.2	0
0.01,66.3	0
0.01,66.4	0
0.01,66.5	0
0.01,66.6	0
0.01,66.7	0
0.01,66.8	0
0.01,66.9	0
0.01,67.0	0

0.01,67.1	0
0.01,67.2	0
0.01,67.3	0
0.01,67.4	0
0.01,67.5	0
0.01,67.6	0
0.01,67.7	0
0.01,67.8	0
0.01,67.9	0
0.01,68.0	0
0.01,68.1	0
0.01,68.2	0
0.01,68.3	0
0.01,68.4	0
0.01,68.5	0
0.01,68.6	0
0.01,68.7	0
0.01,68.8	0
0.01,68.9	0
0.01,69.0	0
0.01,69.1	0
0.01,69.2	0
0.01,69.3	0
0.01,69.4	0
0.01,69.5	0
0.01,69.6	0
0.01,69.7	0
0.01,69.8	0
0.01,69.9	0
0.01,70.0	0
0.01,70.1	0
0.01,70.2	0
0.01,70.3	0
0.01,70.4	0
0.01,70.5	0
0.01,70.6	0
0.01,70.7	0
0.01,70.8	0
0.01,70.9	0
0.01,71.0	0
0.01,71.1	0
0.01,71.2	0

0.01,71.3	0
0.01,71.4	0
0.01,71.5	0
0.01,71.6	0
0.01,71.7	0
0.01,71.8	0
0.01,71.9	0
0.01,72.0	0
0.01,72.1	0
0.01,72.2	0
0.01,72.3	0
0.01,72.4	0
0.01,72.5	0
0.01,72.6	0
0.01,72.7	0
0.01,72.8	0
0.01,72.9	0
0.01,73.0	0
0.01,73.1	0
0.01,73.2	0
0.01,73.3	0
0.01,73.4	0
0.01,73.5	0
0.01,73.6	0
0.01,73.7	0
0.01,73.8	0
0.01,73.9	0
0.01,74.0	0
0.01,74.1	0
0.01,74.2	0
0.01,74.3	0
0.01,74.4	0
0.01,74.5	0
0.01,74.6	0
0.01,74.7	0
0.01,74.8	0
0.01,74.9	0
0.01,75.0	0
0.01,75.1	0
0.01,75.2	0
0.01,75.3	0
0.01,75.4	0

0.01,75.5	0
0.01,75.6	0
0.01,75.7	0
0.01,75.8	0
0.01,75.9	0
0.01,76.0	0
0.01,76.1	0
0.01,76.2	0
0.01,76.3	0
0.01,76.4	0
0.01,76.5	0
0.01,76.6	0
0.01,76.7	0
0.01,76.8	0
0.01,76.9	0
0.01,77.0	0
0.01,77.1	0
0.01,77.2	0
0.01,77.3	0
0.01,77.4	0
0.01,77.5	0
0.01,77.6	0
0.01,77.7	0
0.01,77.8	0
0.01,77.9	0
0.01,78.0	0
0.01,78.1	0
0.01,78.2	0
0.01,78.3	0
0.01,78.4	0
0.01,78.5	0
0.01,78.6	0
0.01,78.7	0
0.01,78.8	0
0.01,78.9	0
0.01,79.0	0
0.01,79.1	0
0.01,79.2	0
0.01,79.3	0
0.01,79.4	0
0.01,79.5	0
0.01,79.6	0

0.01,79.7	0
0.01,79.8	0
0.01,79.9	0
0.01,80.0	0
0.01,80.1	0
0.01,80.2	0
0.01,80.3	0
0.01,80.4	0
0.01,80.5	0
0.01,80.6	0
0.01,80.7	0
0.01,80.8	0
0.01,80.9	0
0.01,81.0	0
0.01,81.1	0
0.01,81.2	0
0.01,81.3	0
0.01,81.4	0
0.01,81.5	0
0.01,81.6	0
0.01,81.7	0
0.01,81.8	0
0.01,81.9	0
0.01,82.0	0
0.01,82.1	0
0.01,82.2	0
0.01,82.3	0
0.01,82.4	0
0.01,82.5	0
0.01,82.6	0
0.01,82.7	0
0.01,82.8	0
0.01,82.9	0
0.01,83.0	0
0.01,83.1	0
0.01,83.2	0
0.01,83.3	0
0.01,83.4	0
0.01,83.5	0
0.01,83.6	0
0.01,83.7	0
0.01,83.8	0

0.01,83.9	0
0.01,84.0	0
0.01,84.1	0
0.01,84.2	0
0.01,84.3	0
0.01,84.4	0
0.01,84.5	0
0.01,84.6	0
0.01,84.7	0
0.01,84.8	0
0.01,84.9	0
0.01,85.0	0
0.01,85.1	0
0.01,85.2	0
0.01,85.3	0
0.01,85.4	0
0.01,85.5	0
0.01,85.6	0
0.01,85.7	0
0.01,85.8	0
0.01,85.9	0
0.01,86.0	0
0.01,86.1	0
0.01,86.2	0
0.01,86.3	0
0.01,86.4	0
0.01,86.5	0
0.01,86.6	0
0.01,86.7	0
0.01,86.8	0
0.01,86.9	0
0.01,87.0	0
0.01,87.1	0
0.01,87.2	0
0.01,87.3	0
0.01,87.4	0
0.01,87.5	0
0.01,87.6	0
0.01,87.7	0
0.01,87.8	0
0.01,87.9	0
0.01,88.0	0

0.01,88.1	0
0.01,88.2	0
0.01,88.3	0
0.01,88.4	0
0.01,88.5	0
0.01,88.6	0
0.01,88.7	0
0.01,88.8	0
0.01,88.9	0
0.01,89.0	0
0.01,89.1	0
0.01,89.2	0
0.01,89.3	0
0.01,89.4	0
0.01,89.5	0
0.01,89.6	0
0.01,89.7	0
0.01,89.8	0
0.01,89.9	0
0.01,90.0	0
0.01,90.1	0
0.01,90.2	0
0.01,90.3	0
0.01,90.4	0
0.01,90.5	0
0.01,90.6	0
0.01,90.7	0
0.01,90.8	0
0.01,90.9	0
0.01,91.0	0
0.01,91.1	0
0.01,91.2	0
0.01,91.3	0
0.01,91.4	0
0.01,91.5	0
0.01,91.6	0
0.01,91.7	0
0.01,91.8	0
0.01,91.9	0
0.01,92.0	0
0.01,92.1	0
0.01,92.2	0

0.01,92.3	0
0.01,92.4	0
0.01,92.5	0
0.01,92.6	0
0.01,92.7	0
0.01,92.8	0
0.01,92.9	0
0.01,93.0	0
0.01,93.1	0
0.01,93.2	0
0.01,93.3	0
0.01,93.4	0
0.01,93.5	0
0.01,93.6	0
0.01,93.7	0
0.01,93.8	0
0.01,93.9	0
0.01,94.0	0
0.01,94.1	0
0.01,94.2	0
0.01,94.3	0
0.01,94.4	0
0.01,94.5	0
0.01,94.6	0
0.01,94.7	0
0.01,94.8	0
0.01,94.9	0
0.01,95.0	0
0.01,95.1	0
0.01,95.2	0
0.01,95.3	0
0.01,95.4	0
0.01,95.5	0
0.01,95.6	0
0.01,95.7	0
0.01,95.8	0
0.01,95.9	0
0.01,96.0	0
0.01,96.1	0
0.01,96.2	0
0.01,96.3	0
0.01,96.4	0

0.01,96.5	0
0.01,96.6	0
0.01,96.7	0
0.01,96.8	0
0.01,96.9	0
0.01,97.0	0
0.01,97.1	0
0.01,97.2	0
0.01,97.3	0
0.01,97.4	0
0.01,97.5	0
0.01,97.6	0
0.01,97.7	0
0.01,97.8	0
0.01,97.9	0
0.01,98.0	0
0.01,98.1	0
0.01,98.2	0
0.01,98.3	0
0.01,98.4	0
0.01,98.5	0
0.01,98.6	0
0.01,98.7	0
0.01,98.8	0
0.01,98.9	0
0.01,99.0	0
0.01,99.1	0
0.01,99.2	0
0.01,99.3	0
0.01,99.4	0
0.01,99.5	0
0.01,99.6	0
0.01,99.7	0
0.01,99.8	0
0.01,99.9	0
0.01,100.0	0
0.02,0.0	0
0.02,0.1	0
0.02,0.2	0
0.02,0.3	0
0.02,0.4	0
0.02,0.5	0

0.02,0.6	0
0.02,0.7	0
0.02,0.8	0
0.02,0.9	0
0.02,1.0	0
0.02,1.1	0
0.02,1.2	0
0.02,1.3	0
0.02,1.4	0
0.02,1.5	0
0.02,1.6	0
0.02,1.7	0
0.02,1.8	0
0.02,1.9	0
0.02,2.0	0
0.02,2.1	0
0.02,2.2	0
0.02,2.3	0
0.02,2.4	0
0.02,2.5	0
0.02,2.6	0
0.02,2.7	0
0.02,2.8	0
0.02,2.9	0
0.02,3.0	0
0.02,3.1	0
0.02,3.2	0
0.02,3.3	0
0.02,3.4	0
0.02,3.5	0
0.02,3.6	0
0.02,3.7	0
0.02,3.8	0
0.02,3.9	0
0.02,4.0	0
0.02,4.1	0
0.02,4.2	0
0.02,4.3	0
0.02,4.4	0
0.02,4.5	0
0.02,4.6	0
0.02,4.7	0

0.02,4.8	0
0.02,4.9	0
0.02,5.0	0
0.02,5.1	0
0.02,5.2	0
0.02,5.3	0
0.02,5.4	0
0.02,5.5	0
0.02,5.6	0
0.02,5.7	0
0.02,5.8	0
0.02,5.9	0
0.02,6.0	0
0.02,6.1	0
0.02,6.2	0
0.02,6.3	0
0.02,6.4	0
0.02,6.5	0
0.02,6.6	0
0.02,6.7	0
0.02,6.8	0
0.02,6.9	0
0.02,7.0	0
0.02,7.1	0
0.02,7.2	0
0.02,7.3	0
0.02,7.4	0
0.02,7.5	0
0.02,7.6	0
0.02,7.7	0
0.02,7.8	0
0.02,7.9	0
0.02,8.0	0
0.02,8.1	0
0.02,8.2	0
0.02,8.3	0
0.02,8.4	0
0.02,8.5	0
0.02,8.6	0
0.02,8.7	0
0.02,8.8	0
0.02,8.9	0

0.02,9.0	0
0.02,9.1	0
0.02,9.2	0
0.02,9.3	0
0.02,9.4	0
0.02,9.5	0
0.02,9.6	0
0.02,9.7	0
0.02,9.8	0
0.02,9.9	0
0.02,10.0	0
0.02,10.1	0
0.02,10.2	0
0.02,10.3	0
0.02,10.4	0
0.02,10.5	0
0.02,10.6	0
0.02,10.7	0
0.02,10.8	0
0.02,10.9	0
0.02,11.0	0
0.02,11.1	0
0.02,11.2	0
0.02,11.3	0
0.02,11.4	0
0.02,11.5	0
0.02,11.6	0
0.02,11.7	0
0.02,11.8	0
0.02,11.9	0
0.02,12.0	0
0.02,12.1	0
0.02,12.2	0
0.02,12.3	0
0.02,12.4	0
0.02,12.5	0
0.02,12.6	0
0.02,12.7	0
0.02,12.8	0
0.02,12.9	0
0.02,13.0	0
0.02,13.1	0

0.02,13.2	0
0.02,13.3	0
0.02,13.4	0
0.02,13.5	0
0.02,13.6	0
0.02,13.7	0
0.02,13.8	0
0.02,13.9	0
0.02,14.0	0
0.02,14.1	0
0.02,14.2	0
0.02,14.3	0
0.02,14.4	0
0.02,14.5	0
0.02,14.6	0
0.02,14.7	0
0.02,14.8	0
0.02,14.9	0
0.02,15.0	0
0.02,15.1	0
0.02,15.2	0
0.02,15.3	0
0.02,15.4	0
0.02,15.5	0
0.02,15.6	0
0.02,15.7	0
0.02,15.8	0
0.02,15.9	0
0.02,16.0	0
0.02,16.1	0
0.02,16.2	0
0.02,16.3	0
0.02,16.4	0
0.02,16.5	0
0.02,16.6	0
0.02,16.7	0
0.02,16.8	0
0.02,16.9	0
0.02,17.0	0
0.02,17.1	0
0.02,17.2	0
0.02,17.3	0

0.02,17.4	0
0.02,17.5	0
0.02,17.6	0
0.02,17.7	0
0.02,17.8	0
0.02,17.9	0
0.02,18.0	0
0.02,18.1	0
0.02,18.2	0
0.02,18.3	0
0.02,18.4	0
0.02,18.5	0
0.02,18.6	0
0.02,18.7	0
0.02,18.8	0
0.02,18.9	0
0.02,19.0	0
0.02,19.1	0
0.02,19.2	0
0.02,19.3	0
0.02,19.4	0
0.02,19.5	0
0.02,19.6	0
0.02,19.7	0
0.02,19.8	0
0.02,19.9	0
0.02,20.0	0
0.02,20.1	0
0.02,20.2	0
0.02,20.3	0
0.02,20.4	0
0.02,20.5	0
0.02,20.6	0
0.02,20.7	0
0.02,20.8	0
0.02,20.9	0
0.02,21.0	0
0.02,21.1	0
0.02,21.2	0
0.02,21.3	0
0.02,21.4	0
0.02,21.5	0

0.02,21.6	0
0.02,21.7	0
0.02,21.8	0
0.02,21.9	0
0.02,22.0	0
0.02,22.1	0
0.02,22.2	0
0.02,22.3	0
0.02,22.4	0
0.02,22.5	0
0.02,22.6	0
0.02,22.7	0
0.02,22.8	0
0.02,22.9	0
0.02,23.0	0
0.02,23.1	0
0.02,23.2	0
0.02,23.3	0
0.02,23.4	0
0.02,23.5	0
0.02,23.6	0
0.02,23.7	0
0.02,23.8	0
0.02,23.9	0
0.02,24.0	0
0.02,24.1	0
0.02,24.2	0
0.02,24.3	0
0.02,24.4	0
0.02,24.5	0
0.02,24.6	0
0.02,24.7	0
0.02,24.8	0
0.02,24.9	0
0.02,25.0	0
0.02,25.1	0
0.02,25.2	0
0.02,25.3	0
0.02,25.4	0
0.02,25.5	0
0.02,25.6	0
0.02,25.7	0

0.02,25.8	0
0.02,25.9	0
0.02,26.0	0
0.02,26.1	0
0.02,26.2	0
0.02,26.3	0
0.02,26.4	0
0.02,26.5	0
0.02,26.6	0
0.02,26.7	0
0.02,26.8	0
0.02,26.9	0
0.02,27.0	0
0.02,27.1	0
0.02,27.2	0
0.02,27.3	0
0.02,27.4	0
0.02,27.5	0
0.02,27.6	0
0.02,27.7	0
0.02,27.8	0
0.02,27.9	0
0.02,28.0	0
0.02,28.1	0
0.02,28.2	0
0.02,28.3	0
0.02,28.4	0
0.02,28.5	0
0.02,28.6	0
0.02,28.7	0
0.02,28.8	0
0.02,28.9	0
0.02,29.0	0
0.02,29.1	0
0.02,29.2	0
0.02,29.3	0
0.02,29.4	0
0.02,29.5	0
0.02,29.6	0
0.02,29.7	0
0.02,29.8	0
0.02,29.9	0

0.02,30.0	0
0.02,30.1	0
0.02,30.2	0
0.02,30.3	0
0.02,30.4	0
0.02,30.5	0
0.02,30.6	0
0.02,30.7	0
0.02,30.8	0
0.02,30.9	0
0.02,31.0	0
0.02,31.1	0
0.02,31.2	0
0.02,31.3	0
0.02,31.4	0
0.02,31.5	0
0.02,31.6	0
0.02,31.7	0
0.02,31.8	0
0.02,31.9	0
0.02,32.0	0
0.02,32.1	0
0.02,32.2	0
0.02,32.3	0
0.02,32.4	0
0.02,32.5	0
0.02,32.6	0
0.02,32.7	0
0.02,32.8	0
0.02,32.9	0
0.02,33.0	0
0.02,33.1	0
0.02,33.2	0
0.02,33.3	0
0.02,33.4	0
0.02,33.5	0
0.02,33.6	0
0.02,33.7	0
0.02,33.8	0
0.02,33.9	0
0.02,34.0	0
0.02,34.1	0

0.02,34.2	0
0.02,34.3	0
0.02,34.4	0
0.02,34.5	0
0.02,34.6	0
0.02,34.7	0
0.02,34.8	0
0.02,34.9	0
0.02,35.0	0
0.02,35.1	0
0.02,35.2	0
0.02,35.3	0
0.02,35.4	0
0.02,35.5	0
0.02,35.6	0
0.02,35.7	0
0.02,35.8	0
0.02,35.9	0
0.02,36.0	0
0.02,36.1	0
0.02,36.2	0
0.02,36.3	0
0.02,36.4	0
0.02,36.5	0
0.02,36.6	0
0.02,36.7	0
0.02,36.8	0
0.02,36.9	0
0.02,37.0	0
0.02,37.1	0
0.02,37.2	0
0.02,37.3	0
0.02,37.4	0
0.02,37.5	0
0.02,37.6	0
0.02,37.7	0
0.02,37.8	0
0.02,37.9	0
0.02,38.0	0
0.02,38.1	0
0.02,38.2	0
0.02,38.3	0

0.02,38.4	0
0.02,38.5	0
0.02,38.6	0
0.02,38.7	0
0.02,38.8	0
0.02,38.9	0
0.02,39.0	0
0.02,39.1	0
0.02,39.2	0
0.02,39.3	0
0.02,39.4	0
0.02,39.5	0
0.02,39.6	0
0.02,39.7	0
0.02,39.8	0
0.02,39.9	0
0.02,40.0	0
0.02,40.1	0
0.02,40.2	0
0.02,40.3	0
0.02,40.4	0
0.02,40.5	0
0.02,40.6	0
0.02,40.7	0
0.02,40.8	0
0.02,40.9	0
0.02,41.0	0
0.02,41.1	0
0.02,41.2	0
0.02,41.3	0
0.02,41.4	0
0.02,41.5	0
0.02,41.6	0
0.02,41.7	0
0.02,41.8	0
0.02,41.9	0
0.02,42.0	0
0.02,42.1	0
0.02,42.2	0
0.02,42.3	0
0.02,42.4	0
0.02,42.5	0

0.02,42.6	0
0.02,42.7	0
0.02,42.8	0
0.02,42.9	0
0.02,43.0	0
0.02,43.1	0
0.02,43.2	0
0.02,43.3	0
0.02,43.4	0
0.02,43.5	0
0.02,43.6	0
0.02,43.7	0
0.02,43.8	0
0.02,43.9	0
0.02,44.0	0
0.02,44.1	0
0.02,44.2	0
0.02,44.3	0
0.02,44.4	0
0.02,44.5	0
0.02,44.6	0
0.02,44.7	0
0.02,44.8	0
0.02,44.9	0
0.02,45.0	0
0.02,45.1	0
0.02,45.2	0
0.02,45.3	0
0.02,45.4	0
0.02,45.5	0
0.02,45.6	0
0.02,45.7	0
0.02,45.8	0
0.02,45.9	0
0.02,46.0	0
0.02,46.1	0
0.02,46.2	0
0.02,46.3	0
0.02,46.4	0
0.02,46.5	0
0.02,46.6	0
0.02,46.7	0

0.02,46.8	0
0.02,46.9	0
0.02,47.0	0
0.02,47.1	0
0.02,47.2	0
0.02,47.3	0
0.02,47.4	0
0.02,47.5	0
0.02,47.6	0
0.02,47.7	0
0.02,47.8	0
0.02,47.9	0
0.02,48.0	0
0.02,48.1	0
0.02,48.2	0
0.02,48.3	0
0.02,48.4	0
0.02,48.5	0
0.02,48.6	0
0.02,48.7	0
0.02,48.8	0
0.02,48.9	0
0.02,49.0	0
0.02,49.1	0
0.02,49.2	0
0.02,49.3	0
0.02,49.4	0
0.02,49.5	0
0.02,49.6	0
0.02,49.7	0
0.02,49.8	0
0.02,49.9	0
0.02,50.0	0
0.02,50.1	0
0.02,50.2	0
0.02,50.3	0
0.02,50.4	0
0.02,50.5	0
0.02,50.6	0
0.02,50.7	0
0.02,50.8	0
0.02,50.9	0

0.02,51.0	0
0.02,51.1	0
0.02,51.2	0
0.02,51.3	0
0.02,51.4	0
0.02,51.5	0
0.02,51.6	0
0.02,51.7	0
0.02,51.8	0
0.02,51.9	0
0.02,52.0	0
0.02,52.1	0
0.02,52.2	0
0.02,52.3	0
0.02,52.4	0
0.02,52.5	0
0.02,52.6	0
0.02,52.7	0
0.02,52.8	0
0.02,52.9	0
0.02,53.0	0
0.02,53.1	0
0.02,53.2	0
0.02,53.3	0
0.02,53.4	0
0.02,53.5	0
0.02,53.6	0
0.02,53.7	0
0.02,53.8	0
0.02,53.9	0
0.02,54.0	0
0.02,54.1	0
0.02,54.2	0
0.02,54.3	0
0.02,54.4	0
0.02,54.5	0
0.02,54.6	0
0.02,54.7	0
0.02,54.8	0
0.02,54.9	0
0.02,55.0	0
0.02,55.1	0

0.02,55.2	0
0.02,55.3	0
0.02,55.4	0
0.02,55.5	0
0.02,55.6	0
0.02,55.7	0
0.02,55.8	0
0.02,55.9	0
0.02,56.0	0
0.02,56.1	0
0.02,56.2	0
0.02,56.3	0
0.02,56.4	0
0.02,56.5	0
0.02,56.6	0
0.02,56.7	0
0.02,56.8	0
0.02,56.9	0
0.02,57.0	0
0.02,57.1	0
0.02,57.2	0
0.02,57.3	0
0.02,57.4	0
0.02,57.5	0
0.02,57.6	0
0.02,57.7	0
0.02,57.8	0
0.02,57.9	0
0.02,58.0	0
0.02,58.1	0
0.02,58.2	0
0.02,58.3	0
0.02,58.4	0
0.02,58.5	0
0.02,58.6	0
0.02,58.7	0
0.02,58.8	0
0.02,58.9	0
0.02,59.0	0
0.02,59.1	0
0.02,59.2	0
0.02,59.3	0

0.02,59.4	0
0.02,59.5	0
0.02,59.6	0
0.02,59.7	0
0.02,59.8	0
0.02,59.9	0
0.02,60.0	0
0.02,60.1	0
0.02,60.2	0
0.02,60.3	0
0.02,60.4	0
0.02,60.5	0
0.02,60.6	0
0.02,60.7	0
0.02,60.8	0
0.02,60.9	0
0.02,61.0	0
0.02,61.1	0
0.02,61.2	0
0.02,61.3	0
0.02,61.4	0
0.02,61.5	0
0.02,61.6	0
0.02,61.7	0
0.02,61.8	0
0.02,61.9	0
0.02,62.0	0
0.02,62.1	0
0.02,62.2	0
0.02,62.3	0
0.02,62.4	0
0.02,62.5	0
0.02,62.6	0
0.02,62.7	0
0.02,62.8	0
0.02,62.9	0
0.02,63.0	0
0.02,63.1	0
0.02,63.2	0
0.02,63.3	0
0.02,63.4	0
0.02,63.5	0

0.02,63.6	0
0.02,63.7	0
0.02,63.8	0
0.02,63.9	0
0.02,64.0	0
0.02,64.1	0
0.02,64.2	0
0.02,64.3	0
0.02,64.4	0
0.02,64.5	0
0.02,64.6	0
0.02,64.7	0
0.02,64.8	0
0.02,64.9	0
0.02,65.0	0
0.02,65.1	0
0.02,65.2	0
0.02,65.3	0
0.02,65.4	0
0.02,65.5	0
0.02,65.6	0
0.02,65.7	0
0.02,65.8	0
0.02,65.9	0
0.02,66.0	0
0.02,66.1	0
0.02,66.2	0
0.02,66.3	0
0.02,66.4	0
0.02,66.5	0
0.02,66.6	0
0.02,66.7	0
0.02,66.8	0
0.02,66.9	0
0.02,67.0	0
0.02,67.1	0
0.02,67.2	0
0.02,67.3	0
0.02,67.4	0
0.02,67.5	0
0.02,67.6	0
0.02,67.7	0

0.02,67.8	0
0.02,67.9	0
0.02,68.0	0
0.02,68.1	0
0.02,68.2	0
0.02,68.3	0
0.02,68.4	0
0.02,68.5	0
0.02,68.6	0
0.02,68.7	0
0.02,68.8	0
0.02,68.9	0
0.02,69.0	0
0.02,69.1	0
0.02,69.2	0
0.02,69.3	0
0.02,69.4	0
0.02,69.5	0
0.02,69.6	0
0.02,69.7	0
0.02,69.8	0
0.02,69.9	0
0.02,70.0	0
0.02,70.1	0
0.02,70.2	0
0.02,70.3	0
0.02,70.4	0
0.02,70.5	0
0.02,70.6	0
0.02,70.7	0
0.02,70.8	0
0.02,70.9	0
0.02,71.0	0
0.02,71.1	0
0.02,71.2	0
0.02,71.3	0
0.02,71.4	0
0.02,71.5	0
0.02,71.6	0
0.02,71.7	0
0.02,71.8	0
0.02,71.9	0

0.02,72.0	0
0.02,72.1	0
0.02,72.2	0
0.02,72.3	0
0.02,72.4	0
0.02,72.5	0
0.02,72.6	0
0.02,72.7	0
0.02,72.8	0
0.02,72.9	0
0.02,73.0	0
0.02,73.1	0
0.02,73.2	0
0.02,73.3	0
0.02,73.4	0
0.02,73.5	0
0.02,73.6	0
0.02,73.7	0
0.02,73.8	0
0.02,73.9	0
0.02,74.0	0
0.02,74.1	0
0.02,74.2	0
0.02,74.3	0
0.02,74.4	0
0.02,74.5	0
0.02,74.6	0
0.02,74.7	0
0.02,74.8	0
0.02,74.9	0
0.02,75.0	0
0.02,75.1	0
0.02,75.2	0
0.02,75.3	0
0.02,75.4	0
0.02,75.5	0
0.02,75.6	0
0.02,75.7	0
0.02,75.8	0
0.02,75.9	0
0.02,76.0	0
0.02,76.1	0

0.02,76.2	0
0.02,76.3	0
0.02,76.4	0
0.02,76.5	0
0.02,76.6	0
0.02,76.7	0
0.02,76.8	0
0.02,76.9	0
0.02,77.0	0
0.02,77.1	0
0.02,77.2	0
0.02,77.3	0
0.02,77.4	0
0.02,77.5	0
0.02,77.6	0
0.02,77.7	0
0.02,77.8	0
0.02,77.9	0
0.02,78.0	0
0.02,78.1	0
0.02,78.2	0
0.02,78.3	0
0.02,78.4	0
0.02,78.5	0
0.02,78.6	0
0.02,78.7	0
0.02,78.8	0
0.02,78.9	0
0.02,79.0	0
0.02,79.1	0
0.02,79.2	0
0.02,79.3	0
0.02,79.4	0
0.02,79.5	0
0.02,79.6	0
0.02,79.7	0
0.02,79.8	0
0.02,79.9	0
0.02,80.0	0
0.02,80.1	0
0.02,80.2	0
0.02,80.3	0

0.02,80.4	0
0.02,80.5	0
0.02,80.6	0
0.02,80.7	0
0.02,80.8	0
0.02,80.9	0
0.02,81.0	0
0.02,81.1	0
0.02,81.2	0
0.02,81.3	0
0.02,81.4	0
0.02,81.5	0
0.02,81.6	0
0.02,81.7	0
0.02,81.8	0
0.02,81.9	0
0.02,82.0	0
0.02,82.1	0
0.02,82.2	0
0.02,82.3	0
0.02,82.4	0
0.02,82.5	0
0.02,82.6	0
0.02,82.7	0
0.02,82.8	0
0.02,82.9	0
0.02,83.0	0
0.02,83.1	0
0.02,83.2	0
0.02,83.3	0
0.02,83.4	0
0.02,83.5	0
0.02,83.6	0
0.02,83.7	0
0.02,83.8	0
0.02,83.9	0
0.02,84.0	0
0.02,84.1	0
0.02,84.2	0
0.02,84.3	0
0.02,84.4	0
0.02,84.5	0

0.02,84.6	0
0.02,84.7	0
0.02,84.8	0
0.02,84.9	0
0.02,85.0	0
0.02,85.1	0
0.02,85.2	0
0.02,85.3	0
0.02,85.4	0
0.02,85.5	0
0.02,85.6	0
0.02,85.7	0
0.02,85.8	0
0.02,85.9	0
0.02,86.0	0
0.02,86.1	0
0.02,86.2	0
0.02,86.3	0
0.02,86.4	0
0.02,86.5	0
0.02,86.6	0
0.02,86.7	0
0.02,86.8	0
0.02,86.9	0
0.02,87.0	0
0.02,87.1	0
0.02,87.2	0
0.02,87.3	0
0.02,87.4	0
0.02,87.5	0
0.02,87.6	0
0.02,87.7	0
0.02,87.8	0
0.02,87.9	0
0.02,88.0	0
0.02,88.1	0
0.02,88.2	0
0.02,88.3	0
0.02,88.4	0
0.02,88.5	0
0.02,88.6	0
0.02,88.7	0

0.02,88.8	0
0.02,88.9	0
0.02,89.0	0
0.02,89.1	0
0.02,89.2	0
0.02,89.3	0
0.02,89.4	0
0.02,89.5	0
0.02,89.6	0
0.02,89.7	0
0.02,89.8	0
0.02,89.9	0
0.02,90.0	0
0.02,90.1	0
0.02,90.2	0
0.02,90.3	0
0.02,90.4	0
0.02,90.5	0
0.02,90.6	0
0.02,90.7	0
0.02,90.8	0
0.02,90.9	0
0.02,91.0	0
0.02,91.1	0
0.02,91.2	0
0.02,91.3	0
0.02,91.4	0
0.02,91.5	0
0.02,91.6	0
0.02,91.7	0
0.02,91.8	0
0.02,91.9	0
0.02,92.0	0
0.02,92.1	0
0.02,92.2	0
0.02,92.3	0
0.02,92.4	0
0.02,92.5	0
0.02,92.6	0
0.02,92.7	0
0.02,92.8	0
0.02,92.9	0

0.02,93.0	0
0.02,93.1	0
0.02,93.2	0
0.02,93.3	0
0.02,93.4	0
0.02,93.5	0
0.02,93.6	0
0.02,93.7	0
0.02,93.8	0
0.02,93.9	0
0.02,94.0	0
0.02,94.1	0
0.02,94.2	0
0.02,94.3	0
0.02,94.4	0
0.02,94.5	0
0.02,94.6	0
0.02,94.7	0
0.02,94.8	0
0.02,94.9	0
0.02,95.0	0
0.02,95.1	0
0.02,95.2	0
0.02,95.3	0
0.02,95.4	0
0.02,95.5	0
0.02,95.6	0
0.02,95.7	0
0.02,95.8	0
0.02,95.9	0
0.02,96.0	0
0.02,96.1	0
0.02,96.2	0
0.02,96.3	0
0.02,96.4	0
0.02,96.5	0
0.02,96.6	0
0.02,96.7	0
0.02,96.8	0
0.02,96.9	0
0.02,97.0	0
0.02,97.1	0

0.02,97.2	0
0.02,97.3	0
0.02,97.4	0
0.02,97.5	0
0.02,97.6	0
0.02,97.7	0
0.02,97.8	0
0.02,97.9	0
0.02,98.0	0
0.02,98.1	0
0.02,98.2	0
0.02,98.3	0
0.02,98.4	0
0.02,98.5	0
0.02,98.6	0
0.02,98.7	0
0.02,98.8	0
0.02,98.9	0
0.02,99.0	0
0.02,99.1	0
0.02,99.2	0
0.02,99.3	0
0.02,99.4	0
0.02,99.5	0
0.02,99.6	0
0.02,99.7	0
0.02,99.8	0
0.02,99.9	0
0.02,100.0	0
0.03,0.0	0
0.03,0.1	0
0.03,0.2	0
0.03,0.3	0
0.03,0.4	0
0.03,0.5	0
0.03,0.6	0
0.03,0.7	0
0.03,0.8	0
0.03,0.9	0
0.03,1.0	0
0.03,1.1	0
0.03,1.2	0

0.03,1.3	0
0.03,1.4	0
0.03,1.5	0
0.03,1.6	0
0.03,1.7	0
0.03,1.8	0
0.03,1.9	0
0.03,2.0	0
0.03,2.1	0
0.03,2.2	0
0.03,2.3	0
0.03,2.4	0
0.03,2.5	0
0.03,2.6	0
0.03,2.7	0
0.03,2.8	0
0.03,2.9	0
0.03,3.0	0
0.03,3.1	0
0.03,3.2	0
0.03,3.3	0
0.03,3.4	0
0.03,3.5	0
0.03,3.6	0
0.03,3.7	0
0.03,3.8	0
0.03,3.9	0
0.03,4.0	0
0.03,4.1	0
0.03,4.2	0
0.03,4.3	0
0.03,4.4	0
0.03,4.5	0
0.03,4.6	0
0.03,4.7	0
0.03,4.8	0
0.03,4.9	0
0.03,5.0	0
0.03,5.1	0
0.03,5.2	0
0.03,5.3	0
0.03,5.4	0

0.03,5.5	0
0.03,5.6	0
0.03,5.7	0
0.03,5.8	0
0.03,5.9	0
0.03,6.0	0
0.03,6.1	0
0.03,6.2	0
0.03,6.3	0
0.03,6.4	0
0.03,6.5	0
0.03,6.6	0
0.03,6.7	0
0.03,6.8	0
0.03,6.9	0
0.03,7.0	0
0.03,7.1	0
0.03,7.2	0
0.03,7.3	0
0.03,7.4	0
0.03,7.5	0
0.03,7.6	0
0.03,7.7	0
0.03,7.8	0
0.03,7.9	0
0.03,8.0	0
0.03,8.1	0
0.03,8.2	0
0.03,8.3	0
0.03,8.4	0
0.03,8.5	0
0.03,8.6	0
0.03,8.7	0
0.03,8.8	0
0.03,8.9	0
0.03,9.0	0
0.03,9.1	0
0.03,9.2	0
0.03,9.3	0
0.03,9.4	0
0.03,9.5	0
0.03,9.6	0

0.03,9.7	0
0.03,9.8	0
0.03,9.9	0
0.03,10.0	0
0.03,10.1	0
0.03,10.2	0
0.03,10.3	0
0.03,10.4	0
0.03,10.5	0
0.03,10.6	0
0.03,10.7	0
0.03,10.8	0
0.03,10.9	0
0.03,11.0	0
0.03,11.1	0
0.03,11.2	0
0.03,11.3	0
0.03,11.4	0
0.03,11.5	0
0.03,11.6	0
0.03,11.7	0
0.03,11.8	0
0.03,11.9	0
0.03,12.0	0
0.03,12.1	0
0.03,12.2	0
0.03,12.3	0
0.03,12.4	0
0.03,12.5	0
0.03,12.6	0
0.03,12.7	0
0.03,12.8	0
0.03,12.9	0
0.03,13.0	0
0.03,13.1	0
0.03,13.2	0
0.03,13.3	0
0.03,13.4	0
0.03,13.5	0
0.03,13.6	0
0.03,13.7	0
0.03,13.8	0

0.03,13.9	0
0.03,14.0	0
0.03,14.1	0
0.03,14.2	0
0.03,14.3	0
0.03,14.4	0
0.03,14.5	0
0.03,14.6	0
0.03,14.7	0
0.03,14.8	0
0.03,14.9	0
0.03,15.0	0
0.03,15.1	0
0.03,15.2	0
0.03,15.3	0
0.03,15.4	0
0.03,15.5	0
0.03,15.6	0
0.03,15.7	0
0.03,15.8	0
0.03,15.9	0
0.03,16.0	0
0.03,16.1	0
0.03,16.2	0
0.03,16.3	0
0.03,16.4	0
0.03,16.5	0
0.03,16.6	0
0.03,16.7	0
0.03,16.8	0
0.03,16.9	0
0.03,17.0	0
0.03,17.1	0
0.03,17.2	0
0.03,17.3	0
0.03,17.4	0
0.03,17.5	0
0.03,17.6	0
0.03,17.7	0
0.03,17.8	0
0.03,17.9	0
0.03,18.0	0

0.03,18.1	0
0.03,18.2	0
0.03,18.3	0
0.03,18.4	0
0.03,18.5	0
0.03,18.6	0
0.03,18.7	0
0.03,18.8	0
0.03,18.9	0
0.03,19.0	0
0.03,19.1	0
0.03,19.2	0
0.03,19.3	0
0.03,19.4	0
0.03,19.5	0
0.03,19.6	0
0.03,19.7	0
0.03,19.8	0
0.03,19.9	0
0.03,20.0	0
0.03,20.1	0
0.03,20.2	0
0.03,20.3	0
0.03,20.4	0
0.03,20.5	0
0.03,20.6	0
0.03,20.7	0
0.03,20.8	0
0.03,20.9	0
0.03,21.0	0
0.03,21.1	0
0.03,21.2	0
0.03,21.3	0
0.03,21.4	0
0.03,21.5	0
0.03,21.6	0
0.03,21.7	0
0.03,21.8	0
0.03,21.9	0
0.03,22.0	0
0.03,22.1	0
0.03,22.2	0

0.03,22.3	0
0.03,22.4	0
0.03,22.5	0
0.03,22.6	0
0.03,22.7	0
0.03,22.8	0
0.03,22.9	0
0.03,23.0	0
0.03,23.1	0
0.03,23.2	0
0.03,23.3	0
0.03,23.4	0
0.03,23.5	0
0.03,23.6	0
0.03,23.7	0
0.03,23.8	0
0.03,23.9	0
0.03,24.0	0
0.03,24.1	0
0.03,24.2	0
0.03,24.3	0
0.03,24.4	0
0.03,24.5	0
0.03,24.6	0
0.03,24.7	0
0.03,24.8	0
0.03,24.9	0
0.03,25.0	0
0.03,25.1	0
0.03,25.2	0
0.03,25.3	0
0.03,25.4	0
0.03,25.5	0
0.03,25.6	0
0.03,25.7	0
0.03,25.8	0
0.03,25.9	0
0.03,26.0	0
0.03,26.1	0
0.03,26.2	0
0.03,26.3	0
0.03,26.4	0

0.03,26.5	0
0.03,26.6	0
0.03,26.7	0
0.03,26.8	0
0.03,26.9	0
0.03,27.0	0
0.03,27.1	0
0.03,27.2	0
0.03,27.3	0
0.03,27.4	0
0.03,27.5	0
0.03,27.6	0
0.03,27.7	0
0.03,27.8	0
0.03,27.9	0
0.03,28.0	0
0.03,28.1	0
0.03,28.2	0
0.03,28.3	0
0.03,28.4	0
0.03,28.5	0
0.03,28.6	0
0.03,28.7	0
0.03,28.8	0
0.03,28.9	0
0.03,29.0	0
0.03,29.1	0
0.03,29.2	0
0.03,29.3	0
0.03,29.4	0
0.03,29.5	0
0.03,29.6	0
0.03,29.7	0
0.03,29.8	0
0.03,29.9	0
0.03,30.0	0
0.03,30.1	0
0.03,30.2	0
0.03,30.3	0
0.03,30.4	0
0.03,30.5	0
0.03,30.6	0

0.03,30.7	0
0.03,30.8	0
0.03,30.9	0
0.03,31.0	0
0.03,31.1	0
0.03,31.2	0
0.03,31.3	0
0.03,31.4	0
0.03,31.5	0
0.03,31.6	0
0.03,31.7	0
0.03,31.8	0
0.03,31.9	0
0.03,32.0	0
0.03,32.1	0
0.03,32.2	0
0.03,32.3	0
0.03,32.4	0
0.03,32.5	0
0.03,32.6	0
0.03,32.7	0
0.03,32.8	0
0.03,32.9	0
0.03,33.0	0
0.03,33.1	0
0.03,33.2	0
0.03,33.3	0
0.03,33.4	0
0.03,33.5	0
0.03,33.6	0
0.03,33.7	0
0.03,33.8	0
0.03,33.9	0
0.03,34.0	0
0.03,34.1	0
0.03,34.2	0
0.03,34.3	0
0.03,34.4	0
0.03,34.5	0
0.03,34.6	0
0.03,34.7	0
0.03,34.8	0

0.03,34.9	0
0.03,35.0	0
0.03,35.1	0
0.03,35.2	0
0.03,35.3	0
0.03,35.4	0
0.03,35.5	0
0.03,35.6	0
0.03,35.7	0
0.03,35.8	0
0.03,35.9	0
0.03,36.0	0
0.03,36.1	0
0.03,36.2	0
0.03,36.3	0
0.03,36.4	0
0.03,36.5	0
0.03,36.6	0
0.03,36.7	0
0.03,36.8	0
0.03,36.9	0
0.03,37.0	0
0.03,37.1	0
0.03,37.2	0
0.03,37.3	0
0.03,37.4	0
0.03,37.5	0
0.03,37.6	0
0.03,37.7	0
0.03,37.8	0
0.03,37.9	0
0.03,38.0	0
0.03,38.1	0
0.03,38.2	0
0.03,38.3	0
0.03,38.4	0
0.03,38.5	0
0.03,38.6	0
0.03,38.7	0
0.03,38.8	0
0.03,38.9	0
0.03,39.0	0

0.03,39.1	0
0.03,39.2	0
0.03,39.3	0
0.03,39.4	0
0.03,39.5	0
0.03,39.6	0
0.03,39.7	0
0.03,39.8	0
0.03,39.9	0
0.03,40.0	0
0.03,40.1	0
0.03,40.2	0
0.03,40.3	0
0.03,40.4	0
0.03,40.5	0
0.03,40.6	0
0.03,40.7	0
0.03,40.8	0
0.03,40.9	0
0.03,41.0	0
0.03,41.1	0
0.03,41.2	0
0.03,41.3	0
0.03,41.4	0
0.03,41.5	0
0.03,41.6	0
0.03,41.7	0
0.03,41.8	0
0.03,41.9	0
0.03,42.0	0
0.03,42.1	0
0.03,42.2	0
0.03,42.3	0
0.03,42.4	0
0.03,42.5	0
0.03,42.6	0
0.03,42.7	0
0.03,42.8	0
0.03,42.9	0
0.03,43.0	0
0.03,43.1	0
0.03,43.2	0

0.03,43.3	0
0.03,43.4	0
0.03,43.5	0
0.03,43.6	0
0.03,43.7	0
0.03,43.8	0
0.03,43.9	0
0.03,44.0	0
0.03,44.1	0
0.03,44.2	0
0.03,44.3	0
0.03,44.4	0
0.03,44.5	0
0.03,44.6	0
0.03,44.7	0
0.03,44.8	0
0.03,44.9	0
0.03,45.0	0
0.03,45.1	0
0.03,45.2	0
0.03,45.3	0
0.03,45.4	0
0.03,45.5	0
0.03,45.6	0
0.03,45.7	0
0.03,45.8	0
0.03,45.9	0
0.03,46.0	0
0.03,46.1	0
0.03,46.2	0
0.03,46.3	0
0.03,46.4	0
0.03,46.5	0
0.03,46.6	0
0.03,46.7	0
0.03,46.8	0
0.03,46.9	0
0.03,47.0	0
0.03,47.1	0
0.03,47.2	0
0.03,47.3	0
0.03,47.4	0

0.03,47.5	0
0.03,47.6	0
0.03,47.7	0
0.03,47.8	0
0.03,47.9	0
0.03,48.0	0
0.03,48.1	0
0.03,48.2	0
0.03,48.3	0
0.03,48.4	0
0.03,48.5	0
0.03,48.6	0
0.03,48.7	0
0.03,48.8	0
0.03,48.9	0
0.03,49.0	0
0.03,49.1	0
0.03,49.2	0
0.03,49.3	0
0.03,49.4	0
0.03,49.5	0
0.03,49.6	0
0.03,49.7	0
0.03,49.8	0
0.03,49.9	0
0.03,50.0	0
0.03,50.1	0
0.03,50.2	0
0.03,50.3	0
0.03,50.4	0
0.03,50.5	0
0.03,50.6	0
0.03,50.7	0
0.03,50.8	0
0.03,50.9	0
0.03,51.0	0
0.03,51.1	0
0.03,51.2	0
0.03,51.3	0
0.03,51.4	0
0.03,51.5	0
0.03,51.6	0

0.03,51.7	0
0.03,51.8	0
0.03,51.9	0
0.03,52.0	0
0.03,52.1	0
0.03,52.2	0
0.03,52.3	0
0.03,52.4	0
0.03,52.5	0
0.03,52.6	0
0.03,52.7	0
0.03,52.8	0
0.03,52.9	0
0.03,53.0	0
0.03,53.1	0
0.03,53.2	0
0.03,53.3	0
0.03,53.4	0
0.03,53.5	0
0.03,53.6	0
0.03,53.7	0
0.03,53.8	0
0.03,53.9	0
0.03,54.0	0
0.03,54.1	0
0.03,54.2	0
0.03,54.3	0
0.03,54.4	0
0.03,54.5	0
0.03,54.6	0
0.03,54.7	0
0.03,54.8	0
0.03,54.9	0
0.03,55.0	0
0.03,55.1	0
0.03,55.2	0
0.03,55.3	0
0.03,55.4	0
0.03,55.5	0
0.03,55.6	0
0.03,55.7	0
0.03,55.8	0

0.03,55.9	0
0.03,56.0	0
0.03,56.1	0
0.03,56.2	0
0.03,56.3	0
0.03,56.4	0
0.03,56.5	0
0.03,56.6	0
0.03,56.7	0
0.03,56.8	0
0.03,56.9	0
0.03,57.0	0
0.03,57.1	0
0.03,57.2	0
0.03,57.3	0
0.03,57.4	0
0.03,57.5	0
0.03,57.6	0
0.03,57.7	0
0.03,57.8	0
0.03,57.9	0
0.03,58.0	0
0.03,58.1	0
0.03,58.2	0
0.03,58.3	0
0.03,58.4	0
0.03,58.5	0
0.03,58.6	0
0.03,58.7	0
0.03,58.8	0
0.03,58.9	0
0.03,59.0	0
0.03,59.1	0
0.03,59.2	0
0.03,59.3	0
0.03,59.4	0
0.03,59.5	0
0.03,59.6	0
0.03,59.7	0
0.03,59.8	0
0.03,59.9	0
0.03,60.0	0

0.03,60.1	0
0.03,60.2	0
0.03,60.3	0
0.03,60.4	0
0.03,60.5	0
0.03,60.6	0
0.03,60.7	0
0.03,60.8	0
0.03,60.9	0
0.03,61.0	0
0.03,61.1	0
0.03,61.2	0
0.03,61.3	0
0.03,61.4	0
0.03,61.5	0
0.03,61.6	0
0.03,61.7	0
0.03,61.8	0
0.03,61.9	0
0.03,62.0	0
0.03,62.1	0
0.03,62.2	0
0.03,62.3	0
0.03,62.4	0
0.03,62.5	0
0.03,62.6	0
0.03,62.7	0
0.03,62.8	0
0.03,62.9	0
0.03,63.0	0
0.03,63.1	0
0.03,63.2	0
0.03,63.3	0
0.03,63.4	0
0.03,63.5	0
0.03,63.6	0
0.03,63.7	0
0.03,63.8	0
0.03,63.9	0
0.03,64.0	0
0.03,64.1	0
0.03,64.2	0

0.03,64.3	0
0.03,64.4	0
0.03,64.5	0
0.03,64.6	0
0.03,64.7	0
0.03,64.8	0
0.03,64.9	0
0.03,65.0	0
0.03,65.1	0
0.03,65.2	0
0.03,65.3	0
0.03,65.4	0
0.03,65.5	0
0.03,65.6	0
0.03,65.7	0
0.03,65.8	0
0.03,65.9	0
0.03,66.0	0
0.03,66.1	0
0.03,66.2	0
0.03,66.3	0
0.03,66.4	0
0.03,66.5	0
0.03,66.6	0
0.03,66.7	0
0.03,66.8	0
0.03,66.9	0
0.03,67.0	0
0.03,67.1	0
0.03,67.2	0
0.03,67.3	0
0.03,67.4	0
0.03,67.5	0
0.03,67.6	0
0.03,67.7	0
0.03,67.8	0
0.03,67.9	0
0.03,68.0	0
0.03,68.1	0
0.03,68.2	0
0.03,68.3	0
0.03,68.4	0

0.03,68.5	0
0.03,68.6	0
0.03,68.7	0
0.03,68.8	0
0.03,68.9	0
0.03,69.0	0
0.03,69.1	0
0.03,69.2	0
0.03,69.3	0
0.03,69.4	0
0.03,69.5	0
0.03,69.6	0
0.03,69.7	0
0.03,69.8	0
0.03,69.9	0
0.03,70.0	0
0.03,70.1	0
0.03,70.2	0
0.03,70.3	0
0.03,70.4	0
0.03,70.5	0
0.03,70.6	0
0.03,70.7	0
0.03,70.8	0
0.03,70.9	0
0.03,71.0	0
0.03,71.1	0
0.03,71.2	0
0.03,71.3	0
0.03,71.4	0
0.03,71.5	0
0.03,71.6	0
0.03,71.7	0
0.03,71.8	0
0.03,71.9	0
0.03,72.0	0
0.03,72.1	0
0.03,72.2	0
0.03,72.3	0
0.03,72.4	0
0.03,72.5	0
0.03,72.6	0

0.03,72.7	0
0.03,72.8	0
0.03,72.9	0
0.03,73.0	0
0.03,73.1	0
0.03,73.2	0
0.03,73.3	0
0.03,73.4	0
0.03,73.5	0
0.03,73.6	0
0.03,73.7	0
0.03,73.8	0
0.03,73.9	0
0.03,74.0	0
0.03,74.1	0
0.03,74.2	0
0.03,74.3	0
0.03,74.4	0
0.03,74.5	0
0.03,74.6	0
0.03,74.7	0
0.03,74.8	0
0.03,74.9	0
0.03,75.0	0
0.03,75.1	0
0.03,75.2	0
0.03,75.3	0
0.03,75.4	0
0.03,75.5	0
0.03,75.6	0
0.03,75.7	0
0.03,75.8	0
0.03,75.9	0
0.03,76.0	0
0.03,76.1	0
0.03,76.2	0
0.03,76.3	0
0.03,76.4	0
0.03,76.5	0
0.03,76.6	0
0.03,76.7	0
0.03,76.8	0

0.03,76.9	0
0.03,77.0	0
0.03,77.1	0
0.03,77.2	0
0.03,77.3	0
0.03,77.4	0
0.03,77.5	0
0.03,77.6	0
0.03,77.7	0
0.03,77.8	0
0.03,77.9	0
0.03,78.0	0
0.03,78.1	0
0.03,78.2	0
0.03,78.3	0
0.03,78.4	0
0.03,78.5	0
0.03,78.6	0
0.03,78.7	0
0.03,78.8	0
0.03,78.9	0
0.03,79.0	0
0.03,79.1	0
0.03,79.2	0
0.03,79.3	0
0.03,79.4	0
0.03,79.5	0
0.03,79.6	0
0.03,79.7	0
0.03,79.8	0
0.03,79.9	0
0.03,80.0	0
0.03,80.1	0
0.03,80.2	0
0.03,80.3	0
0.03,80.4	0
0.03,80.5	0
0.03,80.6	0
0.03,80.7	0
0.03,80.8	0
0.03,80.9	0
0.03,81.0	0

0.03,81.1	0
0.03,81.2	0
0.03,81.3	0
0.03,81.4	0
0.03,81.5	0
0.03,81.6	0
0.03,81.7	0
0.03,81.8	0
0.03,81.9	0
0.03,82.0	0
0.03,82.1	0
0.03,82.2	0
0.03,82.3	0
0.03,82.4	0
0.03,82.5	0
0.03,82.6	0
0.03,82.7	0
0.03,82.8	0
0.03,82.9	0
0.03,83.0	0
0.03,83.1	0
0.03,83.2	0
0.03,83.3	0
0.03,83.4	0
0.03,83.5	0
0.03,83.6	0
0.03,83.7	0
0.03,83.8	0
0.03,83.9	0
0.03,84.0	0
0.03,84.1	0
0.03,84.2	0
0.03,84.3	0
0.03,84.4	0
0.03,84.5	0
0.03,84.6	0
0.03,84.7	0
0.03,84.8	0
0.03,84.9	0
0.03,85.0	0
0.03,85.1	0
0.03,85.2	0

0.03,85.3	0
0.03,85.4	0
0.03,85.5	0
0.03,85.6	0
0.03,85.7	0
0.03,85.8	0
0.03,85.9	0
0.03,86.0	0
0.03,86.1	0
0.03,86.2	0
0.03,86.3	0
0.03,86.4	0
0.03,86.5	0
0.03,86.6	0
0.03,86.7	0
0.03,86.8	0
0.03,86.9	0
0.03,87.0	0
0.03,87.1	0
0.03,87.2	0
0.03,87.3	0
0.03,87.4	0
0.03,87.5	0
0.03,87.6	0
0.03,87.7	0
0.03,87.8	0
0.03,87.9	0
0.03,88.0	0
0.03,88.1	0
0.03,88.2	0
0.03,88.3	0
0.03,88.4	0
0.03,88.5	0
0.03,88.6	0
0.03,88.7	0
0.03,88.8	0
0.03,88.9	0
0.03,89.0	0
0.03,89.1	0
0.03,89.2	0
0.03,89.3	0
0.03,89.4	0

0.03,89.5	0
0.03,89.6	0
0.03,89.7	0
0.03,89.8	0
0.03,89.9	0
0.03,90.0	0
0.03,90.1	0
0.03,90.2	0
0.03,90.3	0
0.03,90.4	0
0.03,90.5	0
0.03,90.6	0
0.03,90.7	0
0.03,90.8	0
0.03,90.9	0
0.03,91.0	0
0.03,91.1	0
0.03,91.2	0
0.03,91.3	0
0.03,91.4	0
0.03,91.5	0
0.03,91.6	0
0.03,91.7	0
0.03,91.8	0
0.03,91.9	0
0.03,92.0	0
0.03,92.1	0
0.03,92.2	0
0.03,92.3	0
0.03,92.4	0
0.03,92.5	0
0.03,92.6	0
0.03,92.7	0
0.03,92.8	0
0.03,92.9	0
0.03,93.0	0
0.03,93.1	0
0.03,93.2	0
0.03,93.3	0
0.03,93.4	0
0.03,93.5	0
0.03,93.6	0

0.03,93.7	0
0.03,93.8	0
0.03,93.9	0
0.03,94.0	0
0.03,94.1	0
0.03,94.2	0
0.03,94.3	0
0.03,94.4	0
0.03,94.5	0
0.03,94.6	0
0.03,94.7	0
0.03,94.8	0
0.03,94.9	0
0.03,95.0	0
0.03,95.1	0
0.03,95.2	0
0.03,95.3	0
0.03,95.4	0
0.03,95.5	0
0.03,95.6	0
0.03,95.7	0
0.03,95.8	0
0.03,95.9	0
0.03,96.0	0
0.03,96.1	0
0.03,96.2	0
0.03,96.3	0
0.03,96.4	0
0.03,96.5	0
0.03,96.6	0
0.03,96.7	0
0.03,96.8	0
0.03,96.9	0
0.03,97.0	0
0.03,97.1	0
0.03,97.2	0
0.03,97.3	0
0.03,97.4	0
0.03,97.5	0
0.03,97.6	0
0.03,97.7	0
0.03,97.8	0

0.03,97.9	0
0.03,98.0	0
0.03,98.1	0
0.03,98.2	0
0.03,98.3	0
0.03,98.4	0
0.03,98.5	0
0.03,98.6	0
0.03,98.7	0
0.03,98.8	0
0.03,98.9	0
0.03,99.0	0
0.03,99.1	0
0.03,99.2	0
0.03,99.3	0
0.03,99.4	0
0.03,99.5	0
0.03,99.6	0
0.03,99.7	0
0.03,99.8	0
0.03,99.9	0
0.03,100.0	0
0.04,0.0	0
0.04,0.1	0
0.04,0.2	0
0.04,0.3	0
0.04,0.4	0
0.04,0.5	0
0.04,0.6	0
0.04,0.7	0
0.04,0.8	0
0.04,0.9	0
0.04,1.0	0
0.04,1.1	0
0.04,1.2	0
0.04,1.3	0
0.04,1.4	0
0.04,1.5	0
0.04,1.6	0
0.04,1.7	0
0.04,1.8	0
0.04,1.9	0

0.04,2.0	0
0.04,2.1	0
0.04,2.2	0
0.04,2.3	0
0.04,2.4	0
0.04,2.5	0
0.04,2.6	0
0.04,2.7	0
0.04,2.8	0
0.04,2.9	0
0.04,3.0	0
0.04,3.1	0
0.04,3.2	0
0.04,3.3	0
0.04,3.4	0
0.04,3.5	0
0.04,3.6	0
0.04,3.7	0
0.04,3.8	0
0.04,3.9	0
0.04,4.0	0
0.04,4.1	0
0.04,4.2	0
0.04,4.3	0
0.04,4.4	0
0.04,4.5	0
0.04,4.6	0
0.04,4.7	0
0.04,4.8	0
0.04,4.9	0
0.04,5.0	0
0.04,5.1	0
0.04,5.2	0
0.04,5.3	0
0.04,5.4	0
0.04,5.5	0
0.04,5.6	0
0.04,5.7	0
0.04,5.8	0
0.04,5.9	0
0.04,6.0	0
0.04,6.1	0

0.04,6.2	0
0.04,6.3	0
0.04,6.4	0
0.04,6.5	0
0.04,6.6	0
0.04,6.7	0
0.04,6.8	0
0.04,6.9	0
0.04,7.0	0
0.04,7.1	0
0.04,7.2	0
0.04,7.3	0
0.04,7.4	0
0.04,7.5	0
0.04,7.6	0
0.04,7.7	0
0.04,7.8	0
0.04,7.9	0
0.04,8.0	0
0.04,8.1	0
0.04,8.2	0
0.04,8.3	0
0.04,8.4	0
0.04,8.5	0
0.04,8.6	0
0.04,8.7	0
0.04,8.8	0
0.04,8.9	0
0.04,9.0	0
0.04,9.1	0
0.04,9.2	0
0.04,9.3	0
0.04,9.4	0
0.04,9.5	0
0.04,9.6	0
0.04,9.7	0
0.04,9.8	0
0.04,9.9	0
0.04,10.0	0
0.04,10.1	0
0.04,10.2	0
0.04,10.3	0

0.04,10.4	0
0.04,10.5	0
0.04,10.6	0
0.04,10.7	0
0.04,10.8	0
0.04,10.9	0
0.04,11.0	0
0.04,11.1	0
0.04,11.2	0
0.04,11.3	0
0.04,11.4	0
0.04,11.5	0
0.04,11.6	0
0.04,11.7	0
0.04,11.8	0
0.04,11.9	0
0.04,12.0	0
0.04,12.1	0
0.04,12.2	0
0.04,12.3	0
0.04,12.4	0
0.04,12.5	0
0.04,12.6	0
0.04,12.7	0
0.04,12.8	0
0.04,12.9	0
0.04,13.0	0
0.04,13.1	0
0.04,13.2	0
0.04,13.3	0
0.04,13.4	0
0.04,13.5	0
0.04,13.6	0
0.04,13.7	0
0.04,13.8	0
0.04,13.9	0
0.04,14.0	0
0.04,14.1	0
0.04,14.2	0
0.04,14.3	0
0.04,14.4	0
0.04,14.5	0

0.04,14.6	0
0.04,14.7	0
0.04,14.8	0
0.04,14.9	0
0.04,15.0	0
0.04,15.1	0
0.04,15.2	0
0.04,15.3	0
0.04,15.4	0
0.04,15.5	0
0.04,15.6	0
0.04,15.7	0
0.04,15.8	0
0.04,15.9	0
0.04,16.0	0
0.04,16.1	0
0.04,16.2	0
0.04,16.3	0
0.04,16.4	0
0.04,16.5	0
0.04,16.6	0
0.04,16.7	0
0.04,16.8	0
0.04,16.9	0
0.04,17.0	0
0.04,17.1	0
0.04,17.2	0
0.04,17.3	0
0.04,17.4	0
0.04,17.5	0
0.04,17.6	0
0.04,17.7	0
0.04,17.8	0
0.04,17.9	0
0.04,18.0	0
0.04,18.1	0
0.04,18.2	0
0.04,18.3	0
0.04,18.4	0
0.04,18.5	0
0.04,18.6	0
0.04,18.7	0

0.04,18.8	0
0.04,18.9	0
0.04,19.0	0
0.04,19.1	0
0.04,19.2	0
0.04,19.3	0
0.04,19.4	0
0.04,19.5	0
0.04,19.6	0
0.04,19.7	0
0.04,19.8	0
0.04,19.9	0
0.04,20.0	0
0.04,20.1	0
0.04,20.2	0
0.04,20.3	0
0.04,20.4	0
0.04,20.5	0
0.04,20.6	0
0.04,20.7	0
0.04,20.8	0
0.04,20.9	0
0.04,21.0	0
0.04,21.1	0
0.04,21.2	0
0.04,21.3	0
0.04,21.4	0
0.04,21.5	0
0.04,21.6	0
0.04,21.7	0
0.04,21.8	0
0.04,21.9	0
0.04,22.0	0
0.04,22.1	0
0.04,22.2	0
0.04,22.3	0
0.04,22.4	0
0.04,22.5	0
0.04,22.6	0
0.04,22.7	0
0.04,22.8	0
0.04,22.9	0

0.04,23.0	0
0.04,23.1	0
0.04,23.2	0
0.04,23.3	0
0.04,23.4	0
0.04,23.5	0
0.04,23.6	0
0.04,23.7	0
0.04,23.8	0
0.04,23.9	0
0.04,24.0	0
0.04,24.1	0
0.04,24.2	0
0.04,24.3	0
0.04,24.4	0
0.04,24.5	0
0.04,24.6	0
0.04,24.7	0
0.04,24.8	0
0.04,24.9	0
0.04,25.0	0
0.04,25.1	0
0.04,25.2	0
0.04,25.3	0
0.04,25.4	0
0.04,25.5	0
0.04,25.6	0
0.04,25.7	0
0.04,25.8	0
0.04,25.9	0
0.04,26.0	0
0.04,26.1	0
0.04,26.2	0
0.04,26.3	0
0.04,26.4	0
0.04,26.5	0
0.04,26.6	0
0.04,26.7	0
0.04,26.8	0
0.04,26.9	0
0.04,27.0	0
0.04,27.1	0

0.04,27.2	0
0.04,27.3	0
0.04,27.4	0
0.04,27.5	0
0.04,27.6	0
0.04,27.7	0
0.04,27.8	0
0.04,27.9	0
0.04,28.0	0
0.04,28.1	0
0.04,28.2	0
0.04,28.3	0
0.04,28.4	0
0.04,28.5	0
0.04,28.6	0
0.04,28.7	0
0.04,28.8	0
0.04,28.9	0
0.04,29.0	0
0.04,29.1	0
0.04,29.2	0
0.04,29.3	0
0.04,29.4	0
0.04,29.5	0
0.04,29.6	0
0.04,29.7	0
0.04,29.8	0
0.04,29.9	0
0.04,30.0	0
0.04,30.1	0
0.04,30.2	0
0.04,30.3	0
0.04,30.4	0
0.04,30.5	0
0.04,30.6	0
0.04,30.7	0
0.04,30.8	0
0.04,30.9	0
0.04,31.0	0
0.04,31.1	0
0.04,31.2	0
0.04,31.3	0

0.04,31.4	0
0.04,31.5	0
0.04,31.6	0
0.04,31.7	0
0.04,31.8	0
0.04,31.9	0
0.04,32.0	0
0.04,32.1	0
0.04,32.2	0
0.04,32.3	0
0.04,32.4	0
0.04,32.5	0
0.04,32.6	0
0.04,32.7	0
0.04,32.8	0
0.04,32.9	0
0.04,33.0	0
0.04,33.1	0
0.04,33.2	0
0.04,33.3	0
0.04,33.4	0
0.04,33.5	0
0.04,33.6	0
0.04,33.7	0
0.04,33.8	0
0.04,33.9	0
0.04,34.0	0
0.04,34.1	0
0.04,34.2	0
0.04,34.3	0
0.04,34.4	0
0.04,34.5	0
0.04,34.6	0
0.04,34.7	0
0.04,34.8	0
0.04,34.9	0
0.04,35.0	0
0.04,35.1	0
0.04,35.2	0
0.04,35.3	0
0.04,35.4	0
0.04,35.5	0

0.04,35.6	0
0.04,35.7	0
0.04,35.8	0
0.04,35.9	0
0.04,36.0	0
0.04,36.1	0
0.04,36.2	0
0.04,36.3	0
0.04,36.4	0
0.04,36.5	0
0.04,36.6	0
0.04,36.7	0
0.04,36.8	0
0.04,36.9	0
0.04,37.0	0
0.04,37.1	0
0.04,37.2	0
0.04,37.3	0
0.04,37.4	0
0.04,37.5	0
0.04,37.6	0
0.04,37.7	0
0.04,37.8	0
0.04,37.9	0
0.04,38.0	0
0.04,38.1	0
0.04,38.2	0
0.04,38.3	0
0.04,38.4	0
0.04,38.5	0
0.04,38.6	0
0.04,38.7	0
0.04,38.8	0
0.04,38.9	0
0.04,39.0	0
0.04,39.1	0
0.04,39.2	0
0.04,39.3	0
0.04,39.4	0
0.04,39.5	0
0.04,39.6	0
0.04,39.7	0

0.04,39.8	0
0.04,39.9	0
0.04,40.0	0
0.04,40.1	0
0.04,40.2	0
0.04,40.3	0
0.04,40.4	0
0.04,40.5	0
0.04,40.6	0
0.04,40.7	0
0.04,40.8	0
0.04,40.9	0
0.04,41.0	0
0.04,41.1	0
0.04,41.2	0
0.04,41.3	0
0.04,41.4	0
0.04,41.5	0
0.04,41.6	0
0.04,41.7	0
0.04,41.8	0
0.04,41.9	0
0.04,42.0	0
0.04,42.1	0
0.04,42.2	0
0.04,42.3	0
0.04,42.4	0
0.04,42.5	0
0.04,42.6	0
0.04,42.7	0
0.04,42.8	0
0.04,42.9	0
0.04,43.0	0
0.04,43.1	0
0.04,43.2	0
0.04,43.3	0
0.04,43.4	0
0.04,43.5	0
0.04,43.6	0
0.04,43.7	0
0.04,43.8	0
0.04,43.9	0

0.04,44.0	0
0.04,44.1	0
0.04,44.2	0
0.04,44.3	0
0.04,44.4	0
0.04,44.5	0
0.04,44.6	0
0.04,44.7	0
0.04,44.8	0
0.04,44.9	0
0.04,45.0	0
0.04,45.1	0
0.04,45.2	0
0.04,45.3	0
0.04,45.4	0
0.04,45.5	0
0.04,45.6	0
0.04,45.7	0
0.04,45.8	0
0.04,45.9	0
0.04,46.0	0
0.04,46.1	0
0.04,46.2	0
0.04,46.3	0
0.04,46.4	0
0.04,46.5	0
0.04,46.6	0
0.04,46.7	0
0.04,46.8	0
0.04,46.9	0
0.04,47.0	0
0.04,47.1	0
0.04,47.2	0
0.04,47.3	0
0.04,47.4	0
0.04,47.5	0
0.04,47.6	0
0.04,47.7	0
0.04,47.8	0
0.04,47.9	0
0.04,48.0	0
0.04,48.1	0

0.04,48.2	0
0.04,48.3	0
0.04,48.4	0
0.04,48.5	0
0.04,48.6	0
0.04,48.7	0
0.04,48.8	0
0.04,48.9	0
0.04,49.0	0
0.04,49.1	0
0.04,49.2	0
0.04,49.3	0
0.04,49.4	0
0.04,49.5	0
0.04,49.6	0
0.04,49.7	0
0.04,49.8	0
0.04,49.9	0
0.04,50.0	0
0.04,50.1	0
0.04,50.2	0
0.04,50.3	0
0.04,50.4	0
0.04,50.5	0
0.04,50.6	0
0.04,50.7	0
0.04,50.8	0
0.04,50.9	0
0.04,51.0	0
0.04,51.1	0
0.04,51.2	0
0.04,51.3	0
0.04,51.4	0
0.04,51.5	0
0.04,51.6	0
0.04,51.7	0
0.04,51.8	0
0.04,51.9	0
0.04,52.0	0
0.04,52.1	0
0.04,52.2	0
0.04,52.3	0

0.04,52.4	0
0.04,52.5	0
0.04,52.6	0
0.04,52.7	0
0.04,52.8	0
0.04,52.9	0
0.04,53.0	0
0.04,53.1	0
0.04,53.2	0
0.04,53.3	0
0.04,53.4	0
0.04,53.5	0
0.04,53.6	0
0.04,53.7	0
0.04,53.8	0
0.04,53.9	0
0.04,54.0	0
0.04,54.1	0
0.04,54.2	0
0.04,54.3	0
0.04,54.4	0
0.04,54.5	0
0.04,54.6	0
0.04,54.7	0
0.04,54.8	0
0.04,54.9	0
0.04,55.0	0
0.04,55.1	0
0.04,55.2	0
0.04,55.3	0
0.04,55.4	0
0.04,55.5	0
0.04,55.6	0
0.04,55.7	0
0.04,55.8	0
0.04,55.9	0
0.04,56.0	0
0.04,56.1	0
0.04,56.2	0
0.04,56.3	0
0.04,56.4	0
0.04,56.5	0

0.04,56.6	0
0.04,56.7	0
0.04,56.8	0
0.04,56.9	0
0.04,57.0	0
0.04,57.1	0
0.04,57.2	0
0.04,57.3	0
0.04,57.4	0
0.04,57.5	0
0.04,57.6	0
0.04,57.7	0
0.04,57.8	0
0.04,57.9	0
0.04,58.0	0
0.04,58.1	0
0.04,58.2	0
0.04,58.3	0
0.04,58.4	0
0.04,58.5	0
0.04,58.6	0
0.04,58.7	0
0.04,58.8	0
0.04,58.9	0
0.04,59.0	0
0.04,59.1	0
0.04,59.2	0
0.04,59.3	0
0.04,59.4	0
0.04,59.5	0
0.04,59.6	0
0.04,59.7	0
0.04,59.8	0
0.04,59.9	0
0.04,60.0	0
0.04,60.1	0
0.04,60.2	0
0.04,60.3	0
0.04,60.4	0
0.04,60.5	0
0.04,60.6	0
0.04,60.7	0

0.04,60.8	0
0.04,60.9	0
0.04,61.0	0
0.04,61.1	0
0.04,61.2	0
0.04,61.3	0
0.04,61.4	0
0.04,61.5	0
0.04,61.6	0
0.04,61.7	0
0.04,61.8	0
0.04,61.9	0
0.04,62.0	0
0.04,62.1	0
0.04,62.2	0
0.04,62.3	0
0.04,62.4	0
0.04,62.5	0
0.04,62.6	0
0.04,62.7	0
0.04,62.8	0
0.04,62.9	0
0.04,63.0	0
0.04,63.1	0
0.04,63.2	0
0.04,63.3	0
0.04,63.4	0
0.04,63.5	0
0.04,63.6	0
0.04,63.7	0
0.04,63.8	0
0.04,63.9	0
0.04,64.0	0
0.04,64.1	0
0.04,64.2	0
0.04,64.3	0
0.04,64.4	0
0.04,64.5	0
0.04,64.6	0
0.04,64.7	0
0.04,64.8	0
0.04,64.9	0

0.04,65.0	0
0.04,65.1	0
0.04,65.2	0
0.04,65.3	0
0.04,65.4	0
0.04,65.5	0
0.04,65.6	0
0.04,65.7	0
0.04,65.8	0
0.04,65.9	0
0.04,66.0	0
0.04,66.1	0
0.04,66.2	0
0.04,66.3	0
0.04,66.4	0
0.04,66.5	0
0.04,66.6	0
0.04,66.7	0
0.04,66.8	0
0.04,66.9	0
0.04,67.0	0
0.04,67.1	0
0.04,67.2	0
0.04,67.3	0
0.04,67.4	0
0.04,67.5	0
0.04,67.6	0
0.04,67.7	0
0.04,67.8	0
0.04,67.9	0
0.04,68.0	0
0.04,68.1	0
0.04,68.2	0
0.04,68.3	0
0.04,68.4	0
0.04,68.5	0
0.04,68.6	0
0.04,68.7	0
0.04,68.8	0
0.04,68.9	0
0.04,69.0	0
0.04,69.1	0

0.04,69.2	0
0.04,69.3	0
0.04,69.4	0
0.04,69.5	0
0.04,69.6	0
0.04,69.7	0
0.04,69.8	0
0.04,69.9	0
0.04,70.0	0
0.04,70.1	0
0.04,70.2	0
0.04,70.3	0
0.04,70.4	0
0.04,70.5	0
0.04,70.6	0
0.04,70.7	0
0.04,70.8	0
0.04,70.9	0
0.04,71.0	0
0.04,71.1	0
0.04,71.2	0
0.04,71.3	0
0.04,71.4	0
0.04,71.5	0
0.04,71.6	0
0.04,71.7	0
0.04,71.8	0
0.04,71.9	0
0.04,72.0	0
0.04,72.1	0
0.04,72.2	0
0.04,72.3	0
0.04,72.4	0
0.04,72.5	0
0.04,72.6	0
0.04,72.7	0
0.04,72.8	0
0.04,72.9	0
0.04,73.0	0
0.04,73.1	0
0.04,73.2	0
0.04,73.3	0

0.04,73.4	0
0.04,73.5	0
0.04,73.6	0
0.04,73.7	0
0.04,73.8	0
0.04,73.9	0
0.04,74.0	0
0.04,74.1	0
0.04,74.2	0
0.04,74.3	0
0.04,74.4	0
0.04,74.5	0
0.04,74.6	0
0.04,74.7	0
0.04,74.8	0
0.04,74.9	0
0.04,75.0	0
0.04,75.1	0
0.04,75.2	0
0.04,75.3	0
0.04,75.4	0
0.04,75.5	0
0.04,75.6	0
0.04,75.7	0
0.04,75.8	0
0.04,75.9	0
0.04,76.0	0
0.04,76.1	0
0.04,76.2	0
0.04,76.3	0
0.04,76.4	0
0.04,76.5	0
0.04,76.6	0
0.04,76.7	0
0.04,76.8	0
0.04,76.9	0
0.04,77.0	0
0.04,77.1	0
0.04,77.2	0
0.04,77.3	0
0.04,77.4	0
0.04,77.5	0

0.04,77.6	0
0.04,77.7	0
0.04,77.8	0
0.04,77.9	0
0.04,78.0	0
0.04,78.1	0
0.04,78.2	0
0.04,78.3	0
0.04,78.4	0
0.04,78.5	0
0.04,78.6	0
0.04,78.7	0
0.04,78.8	0
0.04,78.9	0
0.04,79.0	0
0.04,79.1	0
0.04,79.2	0
0.04,79.3	0
0.04,79.4	0
0.04,79.5	0
0.04,79.6	0
0.04,79.7	0
0.04,79.8	0
0.04,79.9	0
0.04,80.0	0
0.04,80.1	0
0.04,80.2	0
0.04,80.3	0
0.04,80.4	0
0.04,80.5	0
0.04,80.6	0
0.04,80.7	0
0.04,80.8	0
0.04,80.9	0
0.04,81.0	0
0.04,81.1	0
0.04,81.2	0
0.04,81.3	0
0.04,81.4	0
0.04,81.5	0
0.04,81.6	0
0.04,81.7	0

0.04,81.8	0
0.04,81.9	0
0.04,82.0	0
0.04,82.1	0
0.04,82.2	0
0.04,82.3	0
0.04,82.4	0
0.04,82.5	0
0.04,82.6	0
0.04,82.7	0
0.04,82.8	0
0.04,82.9	0
0.04,83.0	0
0.04,83.1	0
0.04,83.2	0
0.04,83.3	0
0.04,83.4	0
0.04,83.5	0
0.04,83.6	0
0.04,83.7	0
0.04,83.8	0
0.04,83.9	0
0.04,84.0	0
0.04,84.1	0
0.04,84.2	0
0.04,84.3	0
0.04,84.4	0
0.04,84.5	0
0.04,84.6	0
0.04,84.7	0
0.04,84.8	0
0.04,84.9	0
0.04,85.0	0
0.04,85.1	0
0.04,85.2	0
0.04,85.3	0
0.04,85.4	0
0.04,85.5	0
0.04,85.6	0
0.04,85.7	0
0.04,85.8	0
0.04,85.9	0

0.04,86.0	0
0.04,86.1	0
0.04,86.2	0
0.04,86.3	0
0.04,86.4	0
0.04,86.5	0
0.04,86.6	0
0.04,86.7	0
0.04,86.8	0
0.04,86.9	0
0.04,87.0	0
0.04,87.1	0
0.04,87.2	0
0.04,87.3	0
0.04,87.4	0
0.04,87.5	0
0.04,87.6	0
0.04,87.7	0
0.04,87.8	0
0.04,87.9	0
0.04,88.0	0
0.04,88.1	0
0.04,88.2	0
0.04,88.3	0
0.04,88.4	0
0.04,88.5	0
0.04,88.6	0
0.04,88.7	0
0.04,88.8	0
0.04,88.9	0
0.04,89.0	0
0.04,89.1	0
0.04,89.2	0
0.04,89.3	0
0.04,89.4	0
0.04,89.5	0
0.04,89.6	0
0.04,89.7	0
0.04,89.8	0
0.04,89.9	0
0.04,90.0	0
0.04,90.1	0

0.04,90.2	0
0.04,90.3	0
0.04,90.4	0
0.04,90.5	0
0.04,90.6	0
0.04,90.7	0
0.04,90.8	0
0.04,90.9	0
0.04,91.0	0
0.04,91.1	0
0.04,91.2	0
0.04,91.3	0
0.04,91.4	0
0.04,91.5	0
0.04,91.6	0
0.04,91.7	0
0.04,91.8	0
0.04,91.9	0
0.04,92.0	0
0.04,92.1	0
0.04,92.2	0
0.04,92.3	0
0.04,92.4	0
0.04,92.5	0
0.04,92.6	0
0.04,92.7	0
0.04,92.8	0
0.04,92.9	0
0.04,93.0	0
0.04,93.1	0
0.04,93.2	0
0.04,93.3	0
0.04,93.4	0
0.04,93.5	0
0.04,93.6	0
0.04,93.7	0
0.04,93.8	0
0.04,93.9	0
0.04,94.0	0
0.04,94.1	0
0.04,94.2	0
0.04,94.3	0

0.04,94.4	0
0.04,94.5	0
0.04,94.6	0
0.04,94.7	0
0.04,94.8	0
0.04,94.9	0
0.04,95.0	0
0.04,95.1	0
0.04,95.2	0
0.04,95.3	0
0.04,95.4	0
0.04,95.5	0
0.04,95.6	0
0.04,95.7	0
0.04,95.8	0
0.04,95.9	0
0.04,96.0	0
0.04,96.1	0
0.04,96.2	0
0.04,96.3	0
0.04,96.4	0
0.04,96.5	0
0.04,96.6	0
0.04,96.7	0
0.04,96.8	0
0.04,96.9	0
0.04,97.0	0
0.04,97.1	0
0.04,97.2	0
0.04,97.3	0
0.04,97.4	0
0.04,97.5	0
0.04,97.6	0
0.04,97.7	0
0.04,97.8	0
0.04,97.9	0
0.04,98.0	0
0.04,98.1	0
0.04,98.2	0
0.04,98.3	0
0.04,98.4	0
0.04,98.5	0

0.04,98.6	0
0.04,98.7	0
0.04,98.8	0
0.04,98.9	0
0.04,99.0	0
0.04,99.1	0
0.04,99.2	0
0.04,99.3	0
0.04,99.4	0
0.04,99.5	0
0.04,99.6	0
0.04,99.7	0
0.04,99.8	0
0.04,99.9	0
0.04,100.0	0
0.05,0.0	0
0.05,0.1	0
0.05,0.2	0
0.05,0.3	0
0.05,0.4	0
0.05,0.5	0
0.05,0.6	0
0.05,0.7	0
0.05,0.8	0
0.05,0.9	0
0.05,1.0	0
0.05,1.1	0
0.05,1.2	0
0.05,1.3	0
0.05,1.4	0
0.05,1.5	0
0.05,1.6	0
0.05,1.7	0
0.05,1.8	0
0.05,1.9	0
0.05,2.0	0
0.05,2.1	0
0.05,2.2	0
0.05,2.3	0
0.05,2.4	0
0.05,2.5	0
0.05,2.6	0

0.05,2.7	0
0.05,2.8	0
0.05,2.9	0
0.05,3.0	0
0.05,3.1	0
0.05,3.2	0
0.05,3.3	0
0.05,3.4	0
0.05,3.5	0
0.05,3.6	0
0.05,3.7	0
0.05,3.8	0
0.05,3.9	0
0.05,4.0	0
0.05,4.1	0
0.05,4.2	0
0.05,4.3	0
0.05,4.4	0
0.05,4.5	0
0.05,4.6	0
0.05,4.7	0
0.05,4.8	0
0.05,4.9	0
0.05,5.0	0
0.05,5.1	0
0.05,5.2	0
0.05,5.3	0
0.05,5.4	0
0.05,5.5	0
0.05,5.6	0
0.05,5.7	0
0.05,5.8	0
0.05,5.9	0
0.05,6.0	0
0.05,6.1	0
0.05,6.2	0
0.05,6.3	0
0.05,6.4	0
0.05,6.5	0
0.05,6.6	0
0.05,6.7	0
0.05,6.8	0

0.05,6.9	0
0.05,7.0	0
0.05,7.1	0
0.05,7.2	0
0.05,7.3	0
0.05,7.4	0
0.05,7.5	0
0.05,7.6	0
0.05,7.7	0
0.05,7.8	0
0.05,7.9	0
0.05,8.0	0
0.05,8.1	0
0.05,8.2	0
0.05,8.3	0
0.05,8.4	0
0.05,8.5	0
0.05,8.6	0
0.05,8.7	0
0.05,8.8	0
0.05,8.9	0
0.05,9.0	0
0.05,9.1	0
0.05,9.2	0
0.05,9.3	0
0.05,9.4	0
0.05,9.5	0
0.05,9.6	0
0.05,9.7	0
0.05,9.8	0
0.05,9.9	0
0.05,10.0	0
0.05,10.1	0
0.05,10.2	0
0.05,10.3	0
0.05,10.4	0
0.05,10.5	0
0.05,10.6	0
0.05,10.7	0
0.05,10.8	0
0.05,10.9	0
0.05,11.0	0

0.05,11.1	0
0.05,11.2	0
0.05,11.3	0
0.05,11.4	0
0.05,11.5	0
0.05,11.6	0
0.05,11.7	0
0.05,11.8	0
0.05,11.9	0
0.05,12.0	0
0.05,12.1	0
0.05,12.2	0
0.05,12.3	0
0.05,12.4	0
0.05,12.5	0
0.05,12.6	0
0.05,12.7	0
0.05,12.8	0
0.05,12.9	0
0.05,13.0	0
0.05,13.1	0
0.05,13.2	0
0.05,13.3	0
0.05,13.4	0
0.05,13.5	0
0.05,13.6	0
0.05,13.7	0
0.05,13.8	0
0.05,13.9	0
0.05,14.0	0
0.05,14.1	0
0.05,14.2	0
0.05,14.3	0
0.05,14.4	0
0.05,14.5	0
0.05,14.6	0
0.05,14.7	0
0.05,14.8	0
0.05,14.9	0
0.05,15.0	0
0.05,15.1	0
0.05,15.2	0

0.05,15.3	0
0.05,15.4	0
0.05,15.5	0
0.05,15.6	0
0.05,15.7	0
0.05,15.8	0
0.05,15.9	0
0.05,16.0	0
0.05,16.1	0
0.05,16.2	0
0.05,16.3	0
0.05,16.4	0
0.05,16.5	0
0.05,16.6	0
0.05,16.7	0
0.05,16.8	0
0.05,16.9	0
0.05,17.0	0
0.05,17.1	0
0.05,17.2	0
0.05,17.3	0
0.05,17.4	0
0.05,17.5	0
0.05,17.6	0
0.05,17.7	0
0.05,17.8	0
0.05,17.9	0
0.05,18.0	0
0.05,18.1	0
0.05,18.2	0
0.05,18.3	0
0.05,18.4	0
0.05,18.5	0
0.05,18.6	0
0.05,18.7	0
0.05,18.8	0
0.05,18.9	0
0.05,19.0	0
0.05,19.1	0
0.05,19.2	0
0.05,19.3	0
0.05,19.4	0

0.05,19.5	0
0.05,19.6	0
0.05,19.7	0
0.05,19.8	0
0.05,19.9	0
0.05,20.0	0
0.05,20.1	0
0.05,20.2	0
0.05,20.3	0
0.05,20.4	0
0.05,20.5	0
0.05,20.6	0
0.05,20.7	0
0.05,20.8	0
0.05,20.9	0
0.05,21.0	0
0.05,21.1	0
0.05,21.2	0
0.05,21.3	0
0.05,21.4	0
0.05,21.5	0
0.05,21.6	0
0.05,21.7	0
0.05,21.8	0
0.05,21.9	0
0.05,22.0	0
0.05,22.1	0
0.05,22.2	0
0.05,22.3	0
0.05,22.4	0
0.05,22.5	0
0.05,22.6	0
0.05,22.7	0
0.05,22.8	0
0.05,22.9	0
0.05,23.0	0
0.05,23.1	0
0.05,23.2	0
0.05,23.3	0
0.05,23.4	0
0.05,23.5	0
0.05,23.6	0

0.05,23.7	0
0.05,23.8	0
0.05,23.9	0
0.05,24.0	0
0.05,24.1	0
0.05,24.2	0
0.05,24.3	0
0.05,24.4	0
0.05,24.5	0
0.05,24.6	0
0.05,24.7	0
0.05,24.8	0
0.05,24.9	0
0.05,25.0	0
0.05,25.1	0
0.05,25.2	0
0.05,25.3	0
0.05,25.4	0
0.05,25.5	0
0.05,25.6	0
0.05,25.7	0
0.05,25.8	0
0.05,25.9	0
0.05,26.0	0
0.05,26.1	0
0.05,26.2	0
0.05,26.3	0
0.05,26.4	0
0.05,26.5	0
0.05,26.6	0
0.05,26.7	0
0.05,26.8	0
0.05,26.9	0
0.05,27.0	0
0.05,27.1	0
0.05,27.2	0
0.05,27.3	0
0.05,27.4	0
0.05,27.5	0
0.05,27.6	0
0.05,27.7	0
0.05,27.8	0

0.05,27.9	0
0.05,28.0	0
0.05,28.1	0
0.05,28.2	0
0.05,28.3	0
0.05,28.4	0
0.05,28.5	0
0.05,28.6	0
0.05,28.7	0
0.05,28.8	0
0.05,28.9	0
0.05,29.0	0
0.05,29.1	0
0.05,29.2	0
0.05,29.3	0
0.05,29.4	0
0.05,29.5	0
0.05,29.6	0
0.05,29.7	0
0.05,29.8	0
0.05,29.9	0
0.05,30.0	0
0.05,30.1	0
0.05,30.2	0
0.05,30.3	0
0.05,30.4	0
0.05,30.5	0
0.05,30.6	0
0.05,30.7	0
0.05,30.8	0
0.05,30.9	0
0.05,31.0	0
0.05,31.1	0
0.05,31.2	0
0.05,31.3	0
0.05,31.4	0
0.05,31.5	0
0.05,31.6	0
0.05,31.7	0
0.05,31.8	0
0.05,31.9	0
0.05,32.0	0

0.05,32.1	0
0.05,32.2	0
0.05,32.3	0
0.05,32.4	0
0.05,32.5	0
0.05,32.6	0
0.05,32.7	0
0.05,32.8	0
0.05,32.9	0
0.05,33.0	0
0.05,33.1	0
0.05,33.2	0
0.05,33.3	0
0.05,33.4	0
0.05,33.5	0
0.05,33.6	0
0.05,33.7	0
0.05,33.8	0
0.05,33.9	0
0.05,34.0	0
0.05,34.1	0
0.05,34.2	0
0.05,34.3	0
0.05,34.4	0
0.05,34.5	0
0.05,34.6	0
0.05,34.7	0
0.05,34.8	0
0.05,34.9	0
0.05,35.0	0
0.05,35.1	0
0.05,35.2	0
0.05,35.3	0
0.05,35.4	0
0.05,35.5	0
0.05,35.6	0
0.05,35.7	0
0.05,35.8	0
0.05,35.9	0
0.05,36.0	0
0.05,36.1	0
0.05,36.2	0

0.05,36.3	0
0.05,36.4	0
0.05,36.5	0
0.05,36.6	0
0.05,36.7	0
0.05,36.8	0
0.05,36.9	0
0.05,37.0	0
0.05,37.1	0
0.05,37.2	0
0.05,37.3	0
0.05,37.4	0
0.05,37.5	0
0.05,37.6	0
0.05,37.7	0
0.05,37.8	0
0.05,37.9	0
0.05,38.0	0
0.05,38.1	0
0.05,38.2	0
0.05,38.3	0
0.05,38.4	0
0.05,38.5	0
0.05,38.6	0
0.05,38.7	0
0.05,38.8	0
0.05,38.9	0
0.05,39.0	0
0.05,39.1	0
0.05,39.2	0
0.05,39.3	0
0.05,39.4	0
0.05,39.5	0
0.05,39.6	0
0.05,39.7	0
0.05,39.8	0
0.05,39.9	0
0.05,40.0	0
0.05,40.1	0
0.05,40.2	0
0.05,40.3	0
0.05,40.4	0

0.05,40.5	0
0.05,40.6	0
0.05,40.7	0
0.05,40.8	0
0.05,40.9	0
0.05,41.0	0
0.05,41.1	0
0.05,41.2	0
0.05,41.3	0
0.05,41.4	0
0.05,41.5	0
0.05,41.6	0
0.05,41.7	0
0.05,41.8	0
0.05,41.9	0
0.05,42.0	0
0.05,42.1	0
0.05,42.2	0
0.05,42.3	0
0.05,42.4	0
0.05,42.5	0
0.05,42.6	0
0.05,42.7	0
0.05,42.8	0
0.05,42.9	0
0.05,43.0	0
0.05,43.1	0
0.05,43.2	0
0.05,43.3	0
0.05,43.4	0
0.05,43.5	0
0.05,43.6	0
0.05,43.7	0
0.05,43.8	0
0.05,43.9	0
0.05,44.0	0
0.05,44.1	0
0.05,44.2	0
0.05,44.3	0
0.05,44.4	0
0.05,44.5	0
0.05,44.6	0

0.05,44.7	0
0.05,44.8	0
0.05,44.9	0
0.05,45.0	0
0.05,45.1	0
0.05,45.2	0
0.05,45.3	0
0.05,45.4	0
0.05,45.5	0
0.05,45.6	0
0.05,45.7	0
0.05,45.8	0
0.05,45.9	0
0.05,46.0	0
0.05,46.1	0
0.05,46.2	0
0.05,46.3	0
0.05,46.4	0
0.05,46.5	0
0.05,46.6	0
0.05,46.7	0
0.05,46.8	0
0.05,46.9	0
0.05,47.0	0
0.05,47.1	0
0.05,47.2	0
0.05,47.3	0
0.05,47.4	0
0.05,47.5	0
0.05,47.6	0
0.05,47.7	0
0.05,47.8	0
0.05,47.9	0
0.05,48.0	0
0.05,48.1	0
0.05,48.2	0
0.05,48.3	0
0.05,48.4	0
0.05,48.5	0
0.05,48.6	0
0.05,48.7	0
0.05,48.8	0

0.05,48.9	0
0.05,49.0	0
0.05,49.1	0
0.05,49.2	0
0.05,49.3	0
0.05,49.4	0
0.05,49.5	0
0.05,49.6	0
0.05,49.7	0
0.05,49.8	0
0.05,49.9	0
0.05,50.0	0
0.05,50.1	0
0.05,50.2	0
0.05,50.3	0
0.05,50.4	0
0.05,50.5	0
0.05,50.6	0
0.05,50.7	0
0.05,50.8	0
0.05,50.9	0
0.05,51.0	0
0.05,51.1	0
0.05,51.2	0
0.05,51.3	0
0.05,51.4	0
0.05,51.5	0
0.05,51.6	0
0.05,51.7	0
0.05,51.8	0
0.05,51.9	0
0.05,52.0	0
0.05,52.1	0
0.05,52.2	0
0.05,52.3	0
0.05,52.4	0
0.05,52.5	0
0.05,52.6	0
0.05,52.7	0
0.05,52.8	0
0.05,52.9	0
0.05,53.0	0

0.05,53.1	0
0.05,53.2	0
0.05,53.3	0
0.05,53.4	0
0.05,53.5	0
0.05,53.6	0
0.05,53.7	0
0.05,53.8	0
0.05,53.9	0
0.05,54.0	0
0.05,54.1	0
0.05,54.2	0
0.05,54.3	0
0.05,54.4	0
0.05,54.5	0
0.05,54.6	0
0.05,54.7	0
0.05,54.8	0
0.05,54.9	0
0.05,55.0	0
0.05,55.1	0
0.05,55.2	0
0.05,55.3	0
0.05,55.4	0
0.05,55.5	0
0.05,55.6	0
0.05,55.7	0
0.05,55.8	0
0.05,55.9	0
0.05,56.0	0
0.05,56.1	0
0.05,56.2	0
0.05,56.3	0
0.05,56.4	0
0.05,56.5	0
0.05,56.6	0
0.05,56.7	0
0.05,56.8	0
0.05,56.9	0
0.05,57.0	0
0.05,57.1	0
0.05,57.2	0

0.05,57.3	0
0.05,57.4	0
0.05,57.5	0
0.05,57.6	0
0.05,57.7	0
0.05,57.8	0
0.05,57.9	0
0.05,58.0	0
0.05,58.1	0
0.05,58.2	0
0.05,58.3	0
0.05,58.4	0
0.05,58.5	0
0.05,58.6	0
0.05,58.7	0
0.05,58.8	0
0.05,58.9	0
0.05,59.0	0
0.05,59.1	0
0.05,59.2	0
0.05,59.3	0
0.05,59.4	0
0.05,59.5	0
0.05,59.6	0
0.05,59.7	0
0.05,59.8	0
0.05,59.9	0
0.05,60.0	0
0.05,60.1	0
0.05,60.2	0
0.05,60.3	0
0.05,60.4	0
0.05,60.5	0
0.05,60.6	0
0.05,60.7	0
0.05,60.8	0
0.05,60.9	0
0.05,61.0	0
0.05,61.1	0
0.05,61.2	0
0.05,61.3	0
0.05,61.4	0

0.05,61.5	0
0.05,61.6	0
0.05,61.7	0
0.05,61.8	0
0.05,61.9	0
0.05,62.0	0
0.05,62.1	0
0.05,62.2	0
0.05,62.3	0
0.05,62.4	0
0.05,62.5	0
0.05,62.6	0
0.05,62.7	0
0.05,62.8	0
0.05,62.9	0
0.05,63.0	0
0.05,63.1	0
0.05,63.2	0
0.05,63.3	0
0.05,63.4	0
0.05,63.5	0
0.05,63.6	0
0.05,63.7	0
0.05,63.8	0
0.05,63.9	0
0.05,64.0	0
0.05,64.1	0
0.05,64.2	0
0.05,64.3	0
0.05,64.4	0
0.05,64.5	0
0.05,64.6	0
0.05,64.7	0
0.05,64.8	0
0.05,64.9	0
0.05,65.0	0
0.05,65.1	0
0.05,65.2	0
0.05,65.3	0
0.05,65.4	0
0.05,65.5	0
0.05,65.6	0

0.05,65.7	0
0.05,65.8	0
0.05,65.9	0
0.05,66.0	0
0.05,66.1	0
0.05,66.2	0
0.05,66.3	0
0.05,66.4	0
0.05,66.5	0
0.05,66.6	0
0.05,66.7	0
0.05,66.8	0
0.05,66.9	0
0.05,67.0	0
0.05,67.1	0
0.05,67.2	0
0.05,67.3	0
0.05,67.4	0
0.05,67.5	0
0.05,67.6	0
0.05,67.7	0
0.05,67.8	0
0.05,67.9	0
0.05,68.0	0
0.05,68.1	0
0.05,68.2	0
0.05,68.3	0
0.05,68.4	0
0.05,68.5	0
0.05,68.6	0
0.05,68.7	0
0.05,68.8	0
0.05,68.9	0
0.05,69.0	0
0.05,69.1	0
0.05,69.2	0
0.05,69.3	0
0.05,69.4	0
0.05,69.5	0
0.05,69.6	0
0.05,69.7	0
0.05,69.8	0

0.05,69.9	0
0.05,70.0	0
0.05,70.1	0
0.05,70.2	0
0.05,70.3	0
0.05,70.4	0
0.05,70.5	0
0.05,70.6	0
0.05,70.7	0
0.05,70.8	0
0.05,70.9	0
0.05,71.0	0
0.05,71.1	0
0.05,71.2	0
0.05,71.3	0
0.05,71.4	0
0.05,71.5	0
0.05,71.6	0
0.05,71.7	0
0.05,71.8	0
0.05,71.9	0
0.05,72.0	0
0.05,72.1	0
0.05,72.2	0
0.05,72.3	0
0.05,72.4	0
0.05,72.5	0
0.05,72.6	0
0.05,72.7	0
0.05,72.8	0
0.05,72.9	0
0.05,73.0	0
0.05,73.1	0
0.05,73.2	0
0.05,73.3	0
0.05,73.4	0
0.05,73.5	0
0.05,73.6	0
0.05,73.7	0
0.05,73.8	0
0.05,73.9	0
0.05,74.0	0

0.05,74.1	0
0.05,74.2	0
0.05,74.3	0
0.05,74.4	0
0.05,74.5	0
0.05,74.6	0
0.05,74.7	0
0.05,74.8	0
0.05,74.9	0
0.05,75.0	0
0.05,75.1	0
0.05,75.2	0
0.05,75.3	0
0.05,75.4	0
0.05,75.5	0
0.05,75.6	0
0.05,75.7	0
0.05,75.8	0
0.05,75.9	0
0.05,76.0	0
0.05,76.1	0
0.05,76.2	0
0.05,76.3	0
0.05,76.4	0
0.05,76.5	0
0.05,76.6	0
0.05,76.7	0
0.05,76.8	0
0.05,76.9	0
0.05,77.0	0
0.05,77.1	0
0.05,77.2	0
0.05,77.3	0
0.05,77.4	0
0.05,77.5	0
0.05,77.6	0
0.05,77.7	0
0.05,77.8	0
0.05,77.9	0
0.05,78.0	0
0.05,78.1	0
0.05,78.2	0

0.05,78.3	0
0.05,78.4	0
0.05,78.5	0
0.05,78.6	0
0.05,78.7	0
0.05,78.8	0
0.05,78.9	0
0.05,79.0	0
0.05,79.1	0
0.05,79.2	0
0.05,79.3	0
0.05,79.4	0
0.05,79.5	0
0.05,79.6	0
0.05,79.7	0
0.05,79.8	0
0.05,79.9	0
0.05,80.0	0
0.05,80.1	0
0.05,80.2	0
0.05,80.3	0
0.05,80.4	0
0.05,80.5	0
0.05,80.6	0
0.05,80.7	0
0.05,80.8	0
0.05,80.9	0
0.05,81.0	0
0.05,81.1	0
0.05,81.2	0
0.05,81.3	0
0.05,81.4	0
0.05,81.5	0
0.05,81.6	0
0.05,81.7	0
0.05,81.8	0
0.05,81.9	0
0.05,82.0	0
0.05,82.1	0
0.05,82.2	0
0.05,82.3	0
0.05,82.4	0

0.05,82.5	0
0.05,82.6	0
0.05,82.7	0
0.05,82.8	0
0.05,82.9	0
0.05,83.0	0
0.05,83.1	0
0.05,83.2	0
0.05,83.3	0
0.05,83.4	0
0.05,83.5	0
0.05,83.6	0
0.05,83.7	0
0.05,83.8	0
0.05,83.9	0
0.05,84.0	0
0.05,84.1	0
0.05,84.2	0
0.05,84.3	0
0.05,84.4	0
0.05,84.5	0
0.05,84.6	0
0.05,84.7	0
0.05,84.8	0
0.05,84.9	0
0.05,85.0	0
0.05,85.1	0
0.05,85.2	0
0.05,85.3	0
0.05,85.4	0
0.05,85.5	0
0.05,85.6	0
0.05,85.7	0
0.05,85.8	0
0.05,85.9	0
0.05,86.0	0
0.05,86.1	0
0.05,86.2	0
0.05,86.3	0
0.05,86.4	0
0.05,86.5	0
0.05,86.6	0

0.05,86.7	0
0.05,86.8	0
0.05,86.9	0
0.05,87.0	0
0.05,87.1	0
0.05,87.2	0
0.05,87.3	0
0.05,87.4	0
0.05,87.5	0
0.05,87.6	0
0.05,87.7	0
0.05,87.8	0
0.05,87.9	0
0.05,88.0	0
0.05,88.1	0
0.05,88.2	0
0.05,88.3	0
0.05,88.4	0
0.05,88.5	0
0.05,88.6	0
0.05,88.7	0
0.05,88.8	0
0.05,88.9	0
0.05,89.0	0
0.05,89.1	0
0.05,89.2	0
0.05,89.3	0
0.05,89.4	0
0.05,89.5	0
0.05,89.6	0
0.05,89.7	0
0.05,89.8	0
0.05,89.9	0
0.05,90.0	0
0.05,90.1	0
0.05,90.2	0
0.05,90.3	0
0.05,90.4	0
0.05,90.5	0
0.05,90.6	0
0.05,90.7	0
0.05,90.8	0

0.05,90.9	0
0.05,91.0	0
0.05,91.1	0
0.05,91.2	0
0.05,91.3	0
0.05,91.4	0
0.05,91.5	0
0.05,91.6	0
0.05,91.7	0
0.05,91.8	0
0.05,91.9	0
0.05,92.0	0
0.05,92.1	0
0.05,92.2	0
0.05,92.3	0
0.05,92.4	0
0.05,92.5	0
0.05,92.6	0
0.05,92.7	0
0.05,92.8	0
0.05,92.9	0
0.05,93.0	0
0.05,93.1	0
0.05,93.2	0
0.05,93.3	0
0.05,93.4	0
0.05,93.5	0
0.05,93.6	0
0.05,93.7	0
0.05,93.8	0
0.05,93.9	0
0.05,94.0	0
0.05,94.1	0
0.05,94.2	0
0.05,94.3	0
0.05,94.4	0
0.05,94.5	0
0.05,94.6	0
0.05,94.7	0
0.05,94.8	0
0.05,94.9	0
0.05,95.0	0

0.05,95.1	0
0.05,95.2	0
0.05,95.3	0
0.05,95.4	0
0.05,95.5	0
0.05,95.6	0
0.05,95.7	0
0.05,95.8	0
0.05,95.9	0
0.05,96.0	0
0.05,96.1	0
0.05,96.2	0
0.05,96.3	0
0.05,96.4	0
0.05,96.5	0
0.05,96.6	0
0.05,96.7	0
0.05,96.8	0
0.05,96.9	0
0.05,97.0	0
0.05,97.1	0
0.05,97.2	0
0.05,97.3	0
0.05,97.4	0
0.05,97.5	0
0.05,97.6	0
0.05,97.7	0
0.05,97.8	0
0.05,97.9	0
0.05,98.0	0
0.05,98.1	0
0.05,98.2	0
0.05,98.3	0
0.05,98.4	0
0.05,98.5	0
0.05,98.6	0
0.05,98.7	0
0.05,98.8	0
0.05,98.9	0
0.05,99.0	0
0.05,99.1	0
0.05,99.2	0

0.05,99.3	0
0.05,99.4	0
0.05,99.5	0
0.05,99.6	0
0.05,99.7	0
0.05,99.8	0
0.05,99.9	0
0.05,100.0	0
0.06,0.0	0
0.06,0.1	0
0.06,0.2	0
0.06,0.3	0
0.06,0.4	0
0.06,0.5	0
0.06,0.6	0
0.06,0.7	0
0.06,0.8	0
0.06,0.9	0
0.06,1.0	0
0.06,1.1	0
0.06,1.2	0
0.06,1.3	0
0.06,1.4	0
0.06,1.5	0
0.06,1.6	0
0.06,1.7	0
0.06,1.8	0
0.06,1.9	0
0.06,2.0	0
0.06,2.1	0
0.06,2.2	0
0.06,2.3	0
0.06,2.4	0
0.06,2.5	0
0.06,2.6	0
0.06,2.7	0
0.06,2.8	0
0.06,2.9	0
0.06,3.0	0
0.06,3.1	0
0.06,3.2	0
0.06,3.3	0

0.06,3.4	0
0.06,3.5	0
0.06,3.6	0
0.06,3.7	0
0.06,3.8	0
0.06,3.9	0
0.06,4.0	0
0.06,4.1	0
0.06,4.2	0
0.06,4.3	0
0.06,4.4	0
0.06,4.5	0
0.06,4.6	0
0.06,4.7	0
0.06,4.8	0
0.06,4.9	0
0.06,5.0	0
0.06,5.1	0
0.06,5.2	0
0.06,5.3	0
0.06,5.4	0
0.06,5.5	0
0.06,5.6	0
0.06,5.7	0
0.06,5.8	0
0.06,5.9	0
0.06,6.0	0
0.06,6.1	0
0.06,6.2	0
0.06,6.3	0
0.06,6.4	0
0.06,6.5	0
0.06,6.6	0
0.06,6.7	0
0.06,6.8	0
0.06,6.9	0
0.06,7.0	0
0.06,7.1	0
0.06,7.2	0
0.06,7.3	0
0.06,7.4	0
0.06,7.5	0

0.06,7.6	0
0.06,7.7	0
0.06,7.8	0
0.06,7.9	0
0.06,8.0	0
0.06,8.1	0
0.06,8.2	0
0.06,8.3	0
0.06,8.4	0
0.06,8.5	0
0.06,8.6	0
0.06,8.7	0
0.06,8.8	0
0.06,8.9	0
0.06,9.0	0
0.06,9.1	0
0.06,9.2	0
0.06,9.3	0
0.06,9.4	0
0.06,9.5	0
0.06,9.6	0
0.06,9.7	0
0.06,9.8	0
0.06,9.9	0
0.06,10.0	0
0.06,10.1	0
0.06,10.2	0
0.06,10.3	0
0.06,10.4	0
0.06,10.5	0
0.06,10.6	0
0.06,10.7	0
0.06,10.8	0
0.06,10.9	0
0.06,11.0	0
0.06,11.1	0
0.06,11.2	0
0.06,11.3	0
0.06,11.4	0
0.06,11.5	0
0.06,11.6	0
0.06,11.7	0

0.06,11.8	0
0.06,11.9	0
0.06,12.0	0
0.06,12.1	0
0.06,12.2	0
0.06,12.3	0
0.06,12.4	0
0.06,12.5	0
0.06,12.6	0
0.06,12.7	0
0.06,12.8	0
0.06,12.9	0
0.06,13.0	0
0.06,13.1	0
0.06,13.2	0
0.06,13.3	0
0.06,13.4	0
0.06,13.5	0
0.06,13.6	0
0.06,13.7	0
0.06,13.8	0
0.06,13.9	0
0.06,14.0	0
0.06,14.1	0
0.06,14.2	0
0.06,14.3	0
0.06,14.4	0
0.06,14.5	0
0.06,14.6	0
0.06,14.7	0
0.06,14.8	0
0.06,14.9	0
0.06,15.0	0
0.06,15.1	0
0.06,15.2	0
0.06,15.3	0
0.06,15.4	0
0.06,15.5	0
0.06,15.6	0
0.06,15.7	0
0.06,15.8	0
0.06,15.9	0

0.06,16.0	0
0.06,16.1	0
0.06,16.2	0
0.06,16.3	0
0.06,16.4	0
0.06,16.5	0
0.06,16.6	0
0.06,16.7	0
0.06,16.8	0
0.06,16.9	0
0.06,17.0	0
0.06,17.1	0
0.06,17.2	0
0.06,17.3	0
0.06,17.4	0
0.06,17.5	0
0.06,17.6	0
0.06,17.7	0
0.06,17.8	0
0.06,17.9	0
0.06,18.0	0
0.06,18.1	0
0.06,18.2	0
0.06,18.3	0
0.06,18.4	0
0.06,18.5	0
0.06,18.6	0
0.06,18.7	0
0.06,18.8	0
0.06,18.9	0
0.06,19.0	0
0.06,19.1	0
0.06,19.2	0
0.06,19.3	0
0.06,19.4	0
0.06,19.5	0
0.06,19.6	0
0.06,19.7	0
0.06,19.8	0
0.06,19.9	0
0.06,20.0	0
0.06,20.1	0

0.06,20.2	0
0.06,20.3	0
0.06,20.4	0
0.06,20.5	0
0.06,20.6	0
0.06,20.7	0
0.06,20.8	0
0.06,20.9	0
0.06,21.0	0
0.06,21.1	0
0.06,21.2	0
0.06,21.3	0
0.06,21.4	0
0.06,21.5	0
0.06,21.6	0
0.06,21.7	0
0.06,21.8	0
0.06,21.9	0
0.06,22.0	0
0.06,22.1	0
0.06,22.2	0
0.06,22.3	0
0.06,22.4	0
0.06,22.5	0
0.06,22.6	0
0.06,22.7	0
0.06,22.8	0
0.06,22.9	0
0.06,23.0	0
0.06,23.1	0
0.06,23.2	0
0.06,23.3	0
0.06,23.4	0
0.06,23.5	0
0.06,23.6	0
0.06,23.7	0
0.06,23.8	0
0.06,23.9	0
0.06,24.0	0
0.06,24.1	0
0.06,24.2	0
0.06,24.3	0

0.06,24.4	0
0.06,24.5	0
0.06,24.6	0
0.06,24.7	0
0.06,24.8	0
0.06,24.9	0
0.06,25.0	0
0.06,25.1	0
0.06,25.2	0
0.06,25.3	0
0.06,25.4	0
0.06,25.5	0
0.06,25.6	0
0.06,25.7	0
0.06,25.8	0
0.06,25.9	0
0.06,26.0	0
0.06,26.1	0
0.06,26.2	0
0.06,26.3	0
0.06,26.4	0
0.06,26.5	0
0.06,26.6	0
0.06,26.7	0
0.06,26.8	0
0.06,26.9	0
0.06,27.0	0
0.06,27.1	0
0.06,27.2	0
0.06,27.3	0
0.06,27.4	0
0.06,27.5	0
0.06,27.6	0
0.06,27.7	0
0.06,27.8	0
0.06,27.9	0
0.06,28.0	0
0.06,28.1	0
0.06,28.2	0
0.06,28.3	0
0.06,28.4	0
0.06,28.5	0

0.06,28.6	0
0.06,28.7	0
0.06,28.8	0
0.06,28.9	0
0.06,29.0	0
0.06,29.1	0
0.06,29.2	0
0.06,29.3	0
0.06,29.4	0
0.06,29.5	0
0.06,29.6	0
0.06,29.7	0
0.06,29.8	0
0.06,29.9	0
0.06,30.0	0
0.06,30.1	0
0.06,30.2	0
0.06,30.3	0
0.06,30.4	0
0.06,30.5	0
0.06,30.6	0
0.06,30.7	0
0.06,30.8	0
0.06,30.9	0
0.06,31.0	0
0.06,31.1	0
0.06,31.2	0
0.06,31.3	0
0.06,31.4	0
0.06,31.5	0
0.06,31.6	0
0.06,31.7	0
0.06,31.8	0
0.06,31.9	0
0.06,32.0	0
0.06,32.1	0
0.06,32.2	0
0.06,32.3	0
0.06,32.4	0
0.06,32.5	0
0.06,32.6	0
0.06,32.7	0

0.06,32.8	0
0.06,32.9	0
0.06,33.0	0
0.06,33.1	0
0.06,33.2	0
0.06,33.3	0
0.06,33.4	0
0.06,33.5	0
0.06,33.6	0
0.06,33.7	0
0.06,33.8	0
0.06,33.9	0
0.06,34.0	0
0.06,34.1	0
0.06,34.2	0
0.06,34.3	0
0.06,34.4	0
0.06,34.5	0
0.06,34.6	0
0.06,34.7	0
0.06,34.8	0
0.06,34.9	0
0.06,35.0	0
0.06,35.1	0
0.06,35.2	0
0.06,35.3	0
0.06,35.4	0
0.06,35.5	0
0.06,35.6	0
0.06,35.7	0
0.06,35.8	0
0.06,35.9	0
0.06,36.0	0
0.06,36.1	0
0.06,36.2	0
0.06,36.3	0
0.06,36.4	0
0.06,36.5	0
0.06,36.6	0
0.06,36.7	0
0.06,36.8	0
0.06,36.9	0

0.06,37.0	0
0.06,37.1	0
0.06,37.2	0
0.06,37.3	0
0.06,37.4	0
0.06,37.5	0
0.06,37.6	0
0.06,37.7	0
0.06,37.8	0
0.06,37.9	0
0.06,38.0	0
0.06,38.1	0
0.06,38.2	0
0.06,38.3	0
0.06,38.4	0
0.06,38.5	0
0.06,38.6	0
0.06,38.7	0
0.06,38.8	0
0.06,38.9	0
0.06,39.0	0
0.06,39.1	0
0.06,39.2	0
0.06,39.3	0
0.06,39.4	0
0.06,39.5	0
0.06,39.6	0
0.06,39.7	0
0.06,39.8	0
0.06,39.9	0
0.06,40.0	0
0.06,40.1	0
0.06,40.2	0
0.06,40.3	0
0.06,40.4	0
0.06,40.5	0
0.06,40.6	0
0.06,40.7	0
0.06,40.8	0
0.06,40.9	0
0.06,41.0	0
0.06,41.1	0

0.06,41.2	0
0.06,41.3	0
0.06,41.4	0
0.06,41.5	0
0.06,41.6	0
0.06,41.7	0
0.06,41.8	0
0.06,41.9	0
0.06,42.0	0
0.06,42.1	0
0.06,42.2	0
0.06,42.3	0
0.06,42.4	0
0.06,42.5	0
0.06,42.6	0
0.06,42.7	0
0.06,42.8	0
0.06,42.9	0
0.06,43.0	0
0.06,43.1	0
0.06,43.2	0
0.06,43.3	0
0.06,43.4	0
0.06,43.5	0
0.06,43.6	0
0.06,43.7	0
0.06,43.8	0
0.06,43.9	0
0.06,44.0	0
0.06,44.1	0
0.06,44.2	0
0.06,44.3	0
0.06,44.4	0
0.06,44.5	0
0.06,44.6	0
0.06,44.7	0
0.06,44.8	0
0.06,44.9	0
0.06,45.0	0
0.06,45.1	0
0.06,45.2	0
0.06,45.3	0

0.06,45.4	0
0.06,45.5	0
0.06,45.6	0
0.06,45.7	0
0.06,45.8	0
0.06,45.9	0
0.06,46.0	0
0.06,46.1	0
0.06,46.2	0
0.06,46.3	0
0.06,46.4	0
0.06,46.5	0
0.06,46.6	0
0.06,46.7	0
0.06,46.8	0
0.06,46.9	0
0.06,47.0	0
0.06,47.1	0
0.06,47.2	0
0.06,47.3	0
0.06,47.4	0
0.06,47.5	0
0.06,47.6	0
0.06,47.7	0
0.06,47.8	0
0.06,47.9	0
0.06,48.0	0
0.06,48.1	0
0.06,48.2	0
0.06,48.3	0
0.06,48.4	0
0.06,48.5	0
0.06,48.6	0
0.06,48.7	0
0.06,48.8	0
0.06,48.9	0
0.06,49.0	0
0.06,49.1	0
0.06,49.2	0
0.06,49.3	0
0.06,49.4	0
0.06,49.5	0

0.06,49.6	0
0.06,49.7	0
0.06,49.8	0
0.06,49.9	0
0.06,50.0	0
0.06,50.1	0
0.06,50.2	0
0.06,50.3	0
0.06,50.4	0
0.06,50.5	0
0.06,50.6	0
0.06,50.7	0
0.06,50.8	0
0.06,50.9	0
0.06,51.0	0
0.06,51.1	0
0.06,51.2	0
0.06,51.3	0
0.06,51.4	0
0.06,51.5	0
0.06,51.6	0
0.06,51.7	0
0.06,51.8	0
0.06,51.9	0
0.06,52.0	0
0.06,52.1	0
0.06,52.2	0
0.06,52.3	0
0.06,52.4	0
0.06,52.5	0
0.06,52.6	0
0.06,52.7	0
0.06,52.8	0
0.06,52.9	0
0.06,53.0	0
0.06,53.1	0
0.06,53.2	0
0.06,53.3	0
0.06,53.4	0
0.06,53.5	0
0.06,53.6	0
0.06,53.7	0

0.06,53.8	0
0.06,53.9	0
0.06,54.0	0
0.06,54.1	0
0.06,54.2	0
0.06,54.3	0
0.06,54.4	0
0.06,54.5	0
0.06,54.6	0
0.06,54.7	0
0.06,54.8	0
0.06,54.9	0
0.06,55.0	0
0.06,55.1	0
0.06,55.2	0
0.06,55.3	0
0.06,55.4	0
0.06,55.5	0
0.06,55.6	0
0.06,55.7	0
0.06,55.8	0
0.06,55.9	0
0.06,56.0	0
0.06,56.1	0
0.06,56.2	0
0.06,56.3	0
0.06,56.4	0
0.06,56.5	0
0.06,56.6	0
0.06,56.7	0
0.06,56.8	0
0.06,56.9	0
0.06,57.0	0
0.06,57.1	0
0.06,57.2	0
0.06,57.3	0
0.06,57.4	0
0.06,57.5	0
0.06,57.6	0
0.06,57.7	0
0.06,57.8	0
0.06,57.9	0

0.06,58.0	0
0.06,58.1	0
0.06,58.2	0
0.06,58.3	0
0.06,58.4	0
0.06,58.5	0
0.06,58.6	0
0.06,58.7	0
0.06,58.8	0
0.06,58.9	0
0.06,59.0	0
0.06,59.1	0
0.06,59.2	0
0.06,59.3	0
0.06,59.4	0
0.06,59.5	0
0.06,59.6	0
0.06,59.7	0
0.06,59.8	0
0.06,59.9	0
0.06,60.0	0
0.06,60.1	0
0.06,60.2	0
0.06,60.3	0
0.06,60.4	0
0.06,60.5	0
0.06,60.6	0
0.06,60.7	0
0.06,60.8	0
0.06,60.9	0
0.06,61.0	0
0.06,61.1	0
0.06,61.2	0
0.06,61.3	0
0.06,61.4	0
0.06,61.5	0
0.06,61.6	0
0.06,61.7	0
0.06,61.8	0
0.06,61.9	0
0.06,62.0	0
0.06,62.1	0

0.06,62.2	0
0.06,62.3	0
0.06,62.4	0
0.06,62.5	0
0.06,62.6	0
0.06,62.7	0
0.06,62.8	0
0.06,62.9	0
0.06,63.0	0
0.06,63.1	0
0.06,63.2	0
0.06,63.3	0
0.06,63.4	0
0.06,63.5	0
0.06,63.6	0
0.06,63.7	0
0.06,63.8	0
0.06,63.9	0
0.06,64.0	0
0.06,64.1	0
0.06,64.2	0
0.06,64.3	0
0.06,64.4	0
0.06,64.5	0
0.06,64.6	0
0.06,64.7	0
0.06,64.8	0
0.06,64.9	0
0.06,65.0	0
0.06,65.1	0
0.06,65.2	0
0.06,65.3	0
0.06,65.4	0
0.06,65.5	0
0.06,65.6	0
0.06,65.7	0
0.06,65.8	0
0.06,65.9	0
0.06,66.0	0
0.06,66.1	0
0.06,66.2	0
0.06,66.3	0

0.06,66.4	0
0.06,66.5	0
0.06,66.6	0
0.06,66.7	0
0.06,66.8	0
0.06,66.9	0
0.06,67.0	0
0.06,67.1	0
0.06,67.2	0
0.06,67.3	0
0.06,67.4	0
0.06,67.5	0
0.06,67.6	0
0.06,67.7	0
0.06,67.8	0
0.06,67.9	0
0.06,68.0	0
0.06,68.1	0
0.06,68.2	0
0.06,68.3	0
0.06,68.4	0
0.06,68.5	0
0.06,68.6	0
0.06,68.7	0
0.06,68.8	0
0.06,68.9	0
0.06,69.0	0
0.06,69.1	0
0.06,69.2	0
0.06,69.3	0
0.06,69.4	0
0.06,69.5	0
0.06,69.6	0
0.06,69.7	0
0.06,69.8	0
0.06,69.9	0
0.06,70.0	0
0.06,70.1	0
0.06,70.2	0
0.06,70.3	0
0.06,70.4	0
0.06,70.5	0

0.06,70.6	0
0.06,70.7	0
0.06,70.8	0
0.06,70.9	0
0.06,71.0	0
0.06,71.1	0
0.06,71.2	0
0.06,71.3	0
0.06,71.4	0
0.06,71.5	0
0.06,71.6	0
0.06,71.7	0
0.06,71.8	0
0.06,71.9	0
0.06,72.0	0
0.06,72.1	0
0.06,72.2	0
0.06,72.3	0
0.06,72.4	0
0.06,72.5	0
0.06,72.6	0
0.06,72.7	0
0.06,72.8	0
0.06,72.9	0
0.06,73.0	0
0.06,73.1	0
0.06,73.2	0
0.06,73.3	0
0.06,73.4	0
0.06,73.5	0
0.06,73.6	0
0.06,73.7	0
0.06,73.8	0
0.06,73.9	0
0.06,74.0	0
0.06,74.1	0
0.06,74.2	0
0.06,74.3	0
0.06,74.4	0
0.06,74.5	0
0.06,74.6	0
0.06,74.7	0

0.06,74.8	0
0.06,74.9	0
0.06,75.0	0
0.06,75.1	0
0.06,75.2	0
0.06,75.3	0
0.06,75.4	0
0.06,75.5	0
0.06,75.6	0
0.06,75.7	0
0.06,75.8	0
0.06,75.9	0
0.06,76.0	0
0.06,76.1	0
0.06,76.2	0
0.06,76.3	0
0.06,76.4	0
0.06,76.5	0
0.06,76.6	0
0.06,76.7	0
0.06,76.8	0
0.06,76.9	0
0.06,77.0	0
0.06,77.1	0
0.06,77.2	0
0.06,77.3	0
0.06,77.4	0
0.06,77.5	0
0.06,77.6	0
0.06,77.7	0
0.06,77.8	0
0.06,77.9	0
0.06,78.0	0
0.06,78.1	0
0.06,78.2	0
0.06,78.3	0
0.06,78.4	0
0.06,78.5	0
0.06,78.6	0
0.06,78.7	0
0.06,78.8	0
0.06,78.9	0

0.06,79.0	0
0.06,79.1	0
0.06,79.2	0
0.06,79.3	0
0.06,79.4	0
0.06,79.5	0
0.06,79.6	0
0.06,79.7	0
0.06,79.8	0
0.06,79.9	0
0.06,80.0	0
0.06,80.1	0
0.06,80.2	0
0.06,80.3	0
0.06,80.4	0
0.06,80.5	0
0.06,80.6	0
0.06,80.7	0
0.06,80.8	0
0.06,80.9	0
0.06,81.0	0
0.06,81.1	0
0.06,81.2	0
0.06,81.3	0
0.06,81.4	0
0.06,81.5	0
0.06,81.6	0
0.06,81.7	0
0.06,81.8	0
0.06,81.9	0
0.06,82.0	0
0.06,82.1	0
0.06,82.2	0
0.06,82.3	0
0.06,82.4	0
0.06,82.5	0
0.06,82.6	0
0.06,82.7	0
0.06,82.8	0
0.06,82.9	0
0.06,83.0	0
0.06,83.1	0

0.06,83.2	0
0.06,83.3	0
0.06,83.4	0
0.06,83.5	0
0.06,83.6	0
0.06,83.7	0
0.06,83.8	0
0.06,83.9	0
0.06,84.0	0
0.06,84.1	0
0.06,84.2	0
0.06,84.3	0
0.06,84.4	0
0.06,84.5	0
0.06,84.6	0
0.06,84.7	0
0.06,84.8	0
0.06,84.9	0
0.06,85.0	0
0.06,85.1	0
0.06,85.2	0
0.06,85.3	0
0.06,85.4	0
0.06,85.5	0
0.06,85.6	0
0.06,85.7	0
0.06,85.8	0
0.06,85.9	0
0.06,86.0	0
0.06,86.1	0
0.06,86.2	0
0.06,86.3	0
0.06,86.4	0
0.06,86.5	0
0.06,86.6	0
0.06,86.7	0
0.06,86.8	0
0.06,86.9	0
0.06,87.0	0
0.06,87.1	0
0.06,87.2	0
0.06,87.3	0

0.06,87.4	0
0.06,87.5	0
0.06,87.6	0
0.06,87.7	0
0.06,87.8	0
0.06,87.9	0
0.06,88.0	0
0.06,88.1	0
0.06,88.2	0
0.06,88.3	0
0.06,88.4	0
0.06,88.5	0
0.06,88.6	0
0.06,88.7	0
0.06,88.8	0
0.06,88.9	0
0.06,89.0	0
0.06,89.1	0
0.06,89.2	0
0.06,89.3	0
0.06,89.4	0
0.06,89.5	0
0.06,89.6	0
0.06,89.7	0
0.06,89.8	0
0.06,89.9	0
0.06,90.0	0
0.06,90.1	0
0.06,90.2	0
0.06,90.3	0
0.06,90.4	0
0.06,90.5	0
0.06,90.6	0
0.06,90.7	0
0.06,90.8	0
0.06,90.9	0
0.06,91.0	0
0.06,91.1	0
0.06,91.2	0
0.06,91.3	0
0.06,91.4	0
0.06,91.5	0

0.06,91.6	0
0.06,91.7	0
0.06,91.8	0
0.06,91.9	0
0.06,92.0	0
0.06,92.1	0
0.06,92.2	0
0.06,92.3	0
0.06,92.4	0
0.06,92.5	0
0.06,92.6	0
0.06,92.7	0
0.06,92.8	0
0.06,92.9	0
0.06,93.0	0
0.06,93.1	0
0.06,93.2	0
0.06,93.3	0
0.06,93.4	0
0.06,93.5	0
0.06,93.6	0
0.06,93.7	0
0.06,93.8	0
0.06,93.9	0
0.06,94.0	0
0.06,94.1	0
0.06,94.2	0
0.06,94.3	0
0.06,94.4	0
0.06,94.5	0
0.06,94.6	0
0.06,94.7	0
0.06,94.8	0
0.06,94.9	0
0.06,95.0	0
0.06,95.1	0
0.06,95.2	0
0.06,95.3	0
0.06,95.4	0
0.06,95.5	0
0.06,95.6	0
0.06,95.7	0

0.06,95.8	0
0.06,95.9	0
0.06,96.0	0
0.06,96.1	0
0.06,96.2	0
0.06,96.3	0
0.06,96.4	0
0.06,96.5	0
0.06,96.6	0
0.06,96.7	0
0.06,96.8	0
0.06,96.9	0
0.06,97.0	0
0.06,97.1	0
0.06,97.2	0
0.06,97.3	0
0.06,97.4	0
0.06,97.5	0
0.06,97.6	0
0.06,97.7	0
0.06,97.8	0
0.06,97.9	0
0.06,98.0	0
0.06,98.1	0
0.06,98.2	0
0.06,98.3	0
0.06,98.4	0
0.06,98.5	0
0.06,98.6	0
0.06,98.7	0
0.06,98.8	0
0.06,98.9	0
0.06,99.0	0
0.06,99.1	0
0.06,99.2	0
0.06,99.3	0
0.06,99.4	0
0.06,99.5	0
0.06,99.6	0
0.06,99.7	0
0.06,99.8	0
0.06,99.9	0

0.06,100.0	0
0.07,0.0	0
0.07,0.1	0
0.07,0.2	0
0.07,0.3	0
0.07,0.4	0
0.07,0.5	0
0.07,0.6	0
0.07,0.7	0
0.07,0.8	0
0.07,0.9	0
0.07,1.0	0
0.07,1.1	0
0.07,1.2	0
0.07,1.3	0
0.07,1.4	0
0.07,1.5	0
0.07,1.6	0
0.07,1.7	0
0.07,1.8	0
0.07,1.9	0
0.07,2.0	0
0.07,2.1	0
0.07,2.2	0
0.07,2.3	0
0.07,2.4	0
0.07,2.5	0
0.07,2.6	0
0.07,2.7	0
0.07,2.8	0
0.07,2.9	0
0.07,3.0	0
0.07,3.1	0
0.07,3.2	0
0.07,3.3	0
0.07,3.4	0
0.07,3.5	0
0.07,3.6	0
0.07,3.7	0
0.07,3.8	0
0.07,3.9	0
0.07,4.0	0

0.07,4.1	0
0.07,4.2	0
0.07,4.3	0
0.07,4.4	0
0.07,4.5	0
0.07,4.6	0
0.07,4.7	0
0.07,4.8	0
0.07,4.9	0
0.07,5.0	0
0.07,5.1	0
0.07,5.2	0
0.07,5.3	0
0.07,5.4	0
0.07,5.5	0
0.07,5.6	0
0.07,5.7	0
0.07,5.8	0
0.07,5.9	0
0.07,6.0	0
0.07,6.1	0
0.07,6.2	0
0.07,6.3	0
0.07,6.4	0
0.07,6.5	0
0.07,6.6	0
0.07,6.7	0
0.07,6.8	0
0.07,6.9	0
0.07,7.0	0
0.07,7.1	0
0.07,7.2	0
0.07,7.3	0
0.07,7.4	0
0.07,7.5	0
0.07,7.6	0
0.07,7.7	0
0.07,7.8	0
0.07,7.9	0
0.07,8.0	0
0.07,8.1	0
0.07,8.2	0

0.07,8.3	0
0.07,8.4	0
0.07,8.5	0
0.07,8.6	0
0.07,8.7	0
0.07,8.8	0
0.07,8.9	0
0.07,9.0	0
0.07,9.1	0
0.07,9.2	0
0.07,9.3	0
0.07,9.4	0
0.07,9.5	0
0.07,9.6	0
0.07,9.7	0
0.07,9.8	0
0.07,9.9	0
0.07,10.0	0
0.07,10.1	0
0.07,10.2	0
0.07,10.3	0
0.07,10.4	0
0.07,10.5	0
0.07,10.6	0
0.07,10.7	0
0.07,10.8	0
0.07,10.9	0
0.07,11.0	0
0.07,11.1	0
0.07,11.2	0
0.07,11.3	0
0.07,11.4	0
0.07,11.5	0
0.07,11.6	0
0.07,11.7	0
0.07,11.8	0
0.07,11.9	0
0.07,12.0	0
0.07,12.1	0
0.07,12.2	0
0.07,12.3	0
0.07,12.4	0

0.07,12.5	0
0.07,12.6	0
0.07,12.7	0
0.07,12.8	0
0.07,12.9	0
0.07,13.0	0
0.07,13.1	0
0.07,13.2	0
0.07,13.3	0
0.07,13.4	0
0.07,13.5	0
0.07,13.6	0
0.07,13.7	0
0.07,13.8	0
0.07,13.9	0
0.07,14.0	0
0.07,14.1	0
0.07,14.2	0
0.07,14.3	0
0.07,14.4	0
0.07,14.5	0
0.07,14.6	0
0.07,14.7	0
0.07,14.8	0
0.07,14.9	0
0.07,15.0	0
0.07,15.1	0
0.07,15.2	0
0.07,15.3	0
0.07,15.4	0
0.07,15.5	0
0.07,15.6	0
0.07,15.7	0
0.07,15.8	0
0.07,15.9	0
0.07,16.0	0
0.07,16.1	0
0.07,16.2	0
0.07,16.3	0
0.07,16.4	0
0.07,16.5	0
0.07,16.6	0

0.07,16.7	0
0.07,16.8	0
0.07,16.9	0
0.07,17.0	0
0.07,17.1	0
0.07,17.2	0
0.07,17.3	0
0.07,17.4	0
0.07,17.5	0
0.07,17.6	0
0.07,17.7	0
0.07,17.8	0
0.07,17.9	0
0.07,18.0	0
0.07,18.1	0
0.07,18.2	0
0.07,18.3	0
0.07,18.4	0
0.07,18.5	0
0.07,18.6	0
0.07,18.7	0
0.07,18.8	0
0.07,18.9	0
0.07,19.0	0
0.07,19.1	0
0.07,19.2	0
0.07,19.3	0
0.07,19.4	0
0.07,19.5	0
0.07,19.6	0
0.07,19.7	0
0.07,19.8	0
0.07,19.9	0
0.07,20.0	0
0.07,20.1	0
0.07,20.2	0
0.07,20.3	0
0.07,20.4	0
0.07,20.5	0
0.07,20.6	0
0.07,20.7	0
0.07,20.8	0

0.07,20.9	0
0.07,21.0	0
0.07,21.1	0
0.07,21.2	0
0.07,21.3	0
0.07,21.4	0
0.07,21.5	0
0.07,21.6	0
0.07,21.7	0
0.07,21.8	0
0.07,21.9	0
0.07,22.0	0
0.07,22.1	0
0.07,22.2	0
0.07,22.3	0
0.07,22.4	0
0.07,22.5	0
0.07,22.6	0
0.07,22.7	0
0.07,22.8	0
0.07,22.9	0
0.07,23.0	0
0.07,23.1	0
0.07,23.2	0
0.07,23.3	0
0.07,23.4	0
0.07,23.5	0
0.07,23.6	0
0.07,23.7	0
0.07,23.8	0
0.07,23.9	0
0.07,24.0	0
0.07,24.1	0
0.07,24.2	0
0.07,24.3	0
0.07,24.4	0
0.07,24.5	0
0.07,24.6	0
0.07,24.7	0
0.07,24.8	0
0.07,24.9	0
0.07,25.0	0

0.07,25.1	0
0.07,25.2	0
0.07,25.3	0
0.07,25.4	0
0.07,25.5	0
0.07,25.6	0
0.07,25.7	0
0.07,25.8	0
0.07,25.9	0
0.07,26.0	0
0.07,26.1	0
0.07,26.2	0
0.07,26.3	0
0.07,26.4	0
0.07,26.5	0
0.07,26.6	0
0.07,26.7	0
0.07,26.8	0
0.07,26.9	0
0.07,27.0	0
0.07,27.1	0
0.07,27.2	0
0.07,27.3	0
0.07,27.4	0
0.07,27.5	0
0.07,27.6	0
0.07,27.7	0
0.07,27.8	0
0.07,27.9	0
0.07,28.0	0
0.07,28.1	0
0.07,28.2	0
0.07,28.3	0
0.07,28.4	0
0.07,28.5	0
0.07,28.6	0
0.07,28.7	0
0.07,28.8	0
0.07,28.9	0
0.07,29.0	0
0.07,29.1	0
0.07,29.2	0

0.07,29.3	0
0.07,29.4	0
0.07,29.5	0
0.07,29.6	0
0.07,29.7	0
0.07,29.8	0
0.07,29.9	0
0.07,30.0	0
0.07,30.1	0
0.07,30.2	0
0.07,30.3	0
0.07,30.4	0
0.07,30.5	0
0.07,30.6	0
0.07,30.7	0
0.07,30.8	0
0.07,30.9	0
0.07,31.0	0
0.07,31.1	0
0.07,31.2	0
0.07,31.3	0
0.07,31.4	0
0.07,31.5	0
0.07,31.6	0
0.07,31.7	0
0.07,31.8	0
0.07,31.9	0
0.07,32.0	0
0.07,32.1	0
0.07,32.2	0
0.07,32.3	0
0.07,32.4	0
0.07,32.5	0
0.07,32.6	0
0.07,32.7	0
0.07,32.8	0
0.07,32.9	0
0.07,33.0	0
0.07,33.1	0
0.07,33.2	0
0.07,33.3	0
0.07,33.4	0

0.07,33.5	0
0.07,33.6	0
0.07,33.7	0
0.07,33.8	0
0.07,33.9	0
0.07,34.0	0
0.07,34.1	0
0.07,34.2	0
0.07,34.3	0
0.07,34.4	0
0.07,34.5	0
0.07,34.6	0
0.07,34.7	0
0.07,34.8	0
0.07,34.9	0
0.07,35.0	0
0.07,35.1	0
0.07,35.2	0
0.07,35.3	0
0.07,35.4	0
0.07,35.5	0
0.07,35.6	0
0.07,35.7	0
0.07,35.8	0
0.07,35.9	0
0.07,36.0	0
0.07,36.1	0
0.07,36.2	0
0.07,36.3	0
0.07,36.4	0
0.07,36.5	0
0.07,36.6	0
0.07,36.7	0
0.07,36.8	0
0.07,36.9	0
0.07,37.0	0
0.07,37.1	0
0.07,37.2	0
0.07,37.3	0
0.07,37.4	0
0.07,37.5	0
0.07,37.6	0

0.07,37.7	0
0.07,37.8	0
0.07,37.9	0
0.07,38.0	0
0.07,38.1	0
0.07,38.2	0
0.07,38.3	0
0.07,38.4	0
0.07,38.5	0
0.07,38.6	0
0.07,38.7	0
0.07,38.8	0
0.07,38.9	0
0.07,39.0	0
0.07,39.1	0
0.07,39.2	0
0.07,39.3	0
0.07,39.4	0
0.07,39.5	0
0.07,39.6	0
0.07,39.7	0
0.07,39.8	0
0.07,39.9	0
0.07,40.0	0
0.07,40.1	0
0.07,40.2	0
0.07,40.3	0
0.07,40.4	0
0.07,40.5	0
0.07,40.6	0
0.07,40.7	0
0.07,40.8	0
0.07,40.9	0
0.07,41.0	0
0.07,41.1	0
0.07,41.2	0
0.07,41.3	0
0.07,41.4	0
0.07,41.5	0
0.07,41.6	0
0.07,41.7	0
0.07,41.8	0

0.07,41.9	0
0.07,42.0	0
0.07,42.1	0
0.07,42.2	0
0.07,42.3	0
0.07,42.4	0
0.07,42.5	0
0.07,42.6	0
0.07,42.7	0
0.07,42.8	0
0.07,42.9	0
0.07,43.0	0
0.07,43.1	0
0.07,43.2	0
0.07,43.3	0
0.07,43.4	0
0.07,43.5	0
0.07,43.6	0
0.07,43.7	0
0.07,43.8	0
0.07,43.9	0
0.07,44.0	0
0.07,44.1	0
0.07,44.2	0
0.07,44.3	0
0.07,44.4	0
0.07,44.5	0
0.07,44.6	0
0.07,44.7	0
0.07,44.8	0
0.07,44.9	0
0.07,45.0	0
0.07,45.1	0
0.07,45.2	0
0.07,45.3	0
0.07,45.4	0
0.07,45.5	0
0.07,45.6	0
0.07,45.7	0
0.07,45.8	0
0.07,45.9	0
0.07,46.0	0

0.07,46.1	0
0.07,46.2	0
0.07,46.3	0
0.07,46.4	0
0.07,46.5	0
0.07,46.6	0
0.07,46.7	0
0.07,46.8	0
0.07,46.9	0
0.07,47.0	0
0.07,47.1	0
0.07,47.2	0
0.07,47.3	0
0.07,47.4	0
0.07,47.5	0
0.07,47.6	0
0.07,47.7	0
0.07,47.8	0
0.07,47.9	0
0.07,48.0	0
0.07,48.1	0
0.07,48.2	0
0.07,48.3	0
0.07,48.4	0
0.07,48.5	0
0.07,48.6	0
0.07,48.7	0
0.07,48.8	0
0.07,48.9	0
0.07,49.0	0
0.07,49.1	0
0.07,49.2	0
0.07,49.3	0
0.07,49.4	0
0.07,49.5	0
0.07,49.6	0
0.07,49.7	0
0.07,49.8	0
0.07,49.9	0
0.07,50.0	0
0.07,50.1	0
0.07,50.2	0

0.07,50.3	0
0.07,50.4	0
0.07,50.5	0
0.07,50.6	0
0.07,50.7	0
0.07,50.8	0
0.07,50.9	0
0.07,51.0	0
0.07,51.1	0
0.07,51.2	0
0.07,51.3	0
0.07,51.4	0
0.07,51.5	0
0.07,51.6	0
0.07,51.7	0
0.07,51.8	0
0.07,51.9	0
0.07,52.0	0
0.07,52.1	0
0.07,52.2	0
0.07,52.3	0
0.07,52.4	0
0.07,52.5	0
0.07,52.6	0
0.07,52.7	0
0.07,52.8	0
0.07,52.9	0
0.07,53.0	0
0.07,53.1	0
0.07,53.2	0
0.07,53.3	0
0.07,53.4	0
0.07,53.5	0
0.07,53.6	0
0.07,53.7	0
0.07,53.8	0
0.07,53.9	0
0.07,54.0	0
0.07,54.1	0
0.07,54.2	0
0.07,54.3	0
0.07,54.4	0

0.07,54.5	0
0.07,54.6	0
0.07,54.7	0
0.07,54.8	0
0.07,54.9	0
0.07,55.0	0
0.07,55.1	0
0.07,55.2	0
0.07,55.3	0
0.07,55.4	0
0.07,55.5	0
0.07,55.6	0
0.07,55.7	0
0.07,55.8	0
0.07,55.9	0
0.07,56.0	0
0.07,56.1	0
0.07,56.2	0
0.07,56.3	0
0.07,56.4	0
0.07,56.5	0
0.07,56.6	0
0.07,56.7	0
0.07,56.8	0
0.07,56.9	0
0.07,57.0	0
0.07,57.1	0
0.07,57.2	0
0.07,57.3	0
0.07,57.4	0
0.07,57.5	0
0.07,57.6	0
0.07,57.7	0
0.07,57.8	0
0.07,57.9	0
0.07,58.0	0
0.07,58.1	0
0.07,58.2	0
0.07,58.3	0
0.07,58.4	0
0.07,58.5	0
0.07,58.6	0

0.07,58.7	0
0.07,58.8	0
0.07,58.9	0
0.07,59.0	0
0.07,59.1	0
0.07,59.2	0
0.07,59.3	0
0.07,59.4	0
0.07,59.5	0
0.07,59.6	0
0.07,59.7	0
0.07,59.8	0
0.07,59.9	0
0.07,60.0	0
0.07,60.1	0
0.07,60.2	0
0.07,60.3	0
0.07,60.4	0
0.07,60.5	0
0.07,60.6	0
0.07,60.7	0
0.07,60.8	0
0.07,60.9	0
0.07,61.0	0
0.07,61.1	0
0.07,61.2	0
0.07,61.3	0
0.07,61.4	0
0.07,61.5	0
0.07,61.6	0
0.07,61.7	0
0.07,61.8	0
0.07,61.9	0
0.07,62.0	0
0.07,62.1	0
0.07,62.2	0
0.07,62.3	0
0.07,62.4	0
0.07,62.5	0
0.07,62.6	0
0.07,62.7	0
0.07,62.8	0

0.07,62.9	0
0.07,63.0	0
0.07,63.1	0
0.07,63.2	0
0.07,63.3	0
0.07,63.4	0
0.07,63.5	0
0.07,63.6	0
0.07,63.7	0
0.07,63.8	0
0.07,63.9	0
0.07,64.0	0
0.07,64.1	0
0.07,64.2	0
0.07,64.3	0
0.07,64.4	0
0.07,64.5	0
0.07,64.6	0
0.07,64.7	0
0.07,64.8	0
0.07,64.9	0
0.07,65.0	0
0.07,65.1	0
0.07,65.2	0
0.07,65.3	0
0.07,65.4	0
0.07,65.5	0
0.07,65.6	0
0.07,65.7	0
0.07,65.8	0
0.07,65.9	0
0.07,66.0	0
0.07,66.1	0
0.07,66.2	0
0.07,66.3	0
0.07,66.4	0
0.07,66.5	0
0.07,66.6	0
0.07,66.7	0
0.07,66.8	0
0.07,66.9	0
0.07,67.0	0

0.07,67.1	0
0.07,67.2	0
0.07,67.3	0
0.07,67.4	0
0.07,67.5	0
0.07,67.6	0
0.07,67.7	0
0.07,67.8	0
0.07,67.9	0
0.07,68.0	0
0.07,68.1	0
0.07,68.2	0
0.07,68.3	0
0.07,68.4	0
0.07,68.5	0
0.07,68.6	0
0.07,68.7	0
0.07,68.8	0
0.07,68.9	0
0.07,69.0	0
0.07,69.1	0
0.07,69.2	0
0.07,69.3	0
0.07,69.4	0
0.07,69.5	0
0.07,69.6	0
0.07,69.7	0
0.07,69.8	0
0.07,69.9	0
0.07,70.0	0
0.07,70.1	0
0.07,70.2	0
0.07,70.3	0
0.07,70.4	0
0.07,70.5	0
0.07,70.6	0
0.07,70.7	0
0.07,70.8	0
0.07,70.9	0
0.07,71.0	0
0.07,71.1	0
0.07,71.2	0

0.07,71.3	0
0.07,71.4	0
0.07,71.5	0
0.07,71.6	0
0.07,71.7	0
0.07,71.8	0
0.07,71.9	0
0.07,72.0	0
0.07,72.1	0
0.07,72.2	0
0.07,72.3	0
0.07,72.4	0
0.07,72.5	0
0.07,72.6	0
0.07,72.7	0
0.07,72.8	0
0.07,72.9	0
0.07,73.0	0
0.07,73.1	0
0.07,73.2	0
0.07,73.3	0
0.07,73.4	0
0.07,73.5	0
0.07,73.6	0
0.07,73.7	0
0.07,73.8	0
0.07,73.9	0
0.07,74.0	0
0.07,74.1	0
0.07,74.2	0
0.07,74.3	0
0.07,74.4	0
0.07,74.5	0
0.07,74.6	0
0.07,74.7	0
0.07,74.8	0
0.07,74.9	0
0.07,75.0	0
0.07,75.1	0
0.07,75.2	0
0.07,75.3	0
0.07,75.4	0

0.07,75.5	0
0.07,75.6	0
0.07,75.7	0
0.07,75.8	0
0.07,75.9	0
0.07,76.0	0
0.07,76.1	0
0.07,76.2	0
0.07,76.3	0
0.07,76.4	0
0.07,76.5	0
0.07,76.6	0
0.07,76.7	0
0.07,76.8	0
0.07,76.9	0
0.07,77.0	0
0.07,77.1	0
0.07,77.2	0
0.07,77.3	0
0.07,77.4	0
0.07,77.5	0
0.07,77.6	0
0.07,77.7	0
0.07,77.8	0
0.07,77.9	0
0.07,78.0	0
0.07,78.1	0
0.07,78.2	0
0.07,78.3	0
0.07,78.4	0
0.07,78.5	0
0.07,78.6	0
0.07,78.7	0
0.07,78.8	0
0.07,78.9	0
0.07,79.0	0
0.07,79.1	0
0.07,79.2	0
0.07,79.3	0
0.07,79.4	0
0.07,79.5	0
0.07,79.6	0

0.07,79.7	0
0.07,79.8	0
0.07,79.9	0
0.07,80.0	0
0.07,80.1	0
0.07,80.2	0
0.07,80.3	0
0.07,80.4	0
0.07,80.5	0
0.07,80.6	0
0.07,80.7	0
0.07,80.8	0
0.07,80.9	0
0.07,81.0	0
0.07,81.1	0
0.07,81.2	0
0.07,81.3	0
0.07,81.4	0
0.07,81.5	0
0.07,81.6	0
0.07,81.7	0
0.07,81.8	0
0.07,81.9	0
0.07,82.0	0
0.07,82.1	0
0.07,82.2	0
0.07,82.3	0
0.07,82.4	0
0.07,82.5	0
0.07,82.6	0
0.07,82.7	0
0.07,82.8	0
0.07,82.9	0
0.07,83.0	0
0.07,83.1	0
0.07,83.2	0
0.07,83.3	0
0.07,83.4	0
0.07,83.5	0
0.07,83.6	0
0.07,83.7	0
0.07,83.8	0

0.07,83.9	0
0.07,84.0	0
0.07,84.1	0
0.07,84.2	0
0.07,84.3	0
0.07,84.4	0
0.07,84.5	0
0.07,84.6	0
0.07,84.7	0
0.07,84.8	0
0.07,84.9	0
0.07,85.0	0
0.07,85.1	0
0.07,85.2	0
0.07,85.3	0
0.07,85.4	0
0.07,85.5	0
0.07,85.6	0
0.07,85.7	0
0.07,85.8	0
0.07,85.9	0
0.07,86.0	0
0.07,86.1	0
0.07,86.2	0
0.07,86.3	0
0.07,86.4	0
0.07,86.5	0
0.07,86.6	0
0.07,86.7	0
0.07,86.8	0
0.07,86.9	0
0.07,87.0	0
0.07,87.1	0
0.07,87.2	0
0.07,87.3	0
0.07,87.4	0
0.07,87.5	0
0.07,87.6	0
0.07,87.7	0
0.07,87.8	0
0.07,87.9	0
0.07,88.0	0

0.07,88.1	0
0.07,88.2	0
0.07,88.3	0
0.07,88.4	0
0.07,88.5	0
0.07,88.6	0
0.07,88.7	0
0.07,88.8	0
0.07,88.9	0
0.07,89.0	0
0.07,89.1	0
0.07,89.2	0
0.07,89.3	0
0.07,89.4	0
0.07,89.5	0
0.07,89.6	0
0.07,89.7	0
0.07,89.8	0
0.07,89.9	0
0.07,90.0	0
0.07,90.1	0
0.07,90.2	0
0.07,90.3	0
0.07,90.4	0
0.07,90.5	0
0.07,90.6	0
0.07,90.7	0
0.07,90.8	0
0.07,90.9	0
0.07,91.0	0
0.07,91.1	0
0.07,91.2	0
0.07,91.3	0
0.07,91.4	0
0.07,91.5	0
0.07,91.6	0
0.07,91.7	0
0.07,91.8	0
0.07,91.9	0
0.07,92.0	0
0.07,92.1	0
0.07,92.2	0

0.07,92.3	0
0.07,92.4	0
0.07,92.5	0
0.07,92.6	0
0.07,92.7	0
0.07,92.8	0
0.07,92.9	0
0.07,93.0	0
0.07,93.1	0
0.07,93.2	0
0.07,93.3	0
0.07,93.4	0
0.07,93.5	0
0.07,93.6	0
0.07,93.7	0
0.07,93.8	0
0.07,93.9	0
0.07,94.0	0
0.07,94.1	0
0.07,94.2	0
0.07,94.3	0
0.07,94.4	0
0.07,94.5	0
0.07,94.6	0
0.07,94.7	0
0.07,94.8	0
0.07,94.9	0
0.07,95.0	0
0.07,95.1	0
0.07,95.2	0
0.07,95.3	0
0.07,95.4	0
0.07,95.5	0
0.07,95.6	0
0.07,95.7	0
0.07,95.8	0
0.07,95.9	0
0.07,96.0	0
0.07,96.1	0
0.07,96.2	0
0.07,96.3	0
0.07,96.4	0

0.07,96.5	0
0.07,96.6	0
0.07,96.7	0
0.07,96.8	0
0.07,96.9	0
0.07,97.0	0
0.07,97.1	0
0.07,97.2	0
0.07,97.3	0
0.07,97.4	0
0.07,97.5	0
0.07,97.6	0
0.07,97.7	0
0.07,97.8	0
0.07,97.9	0
0.07,98.0	0
0.07,98.1	0
0.07,98.2	0
0.07,98.3	0
0.07,98.4	0
0.07,98.5	0
0.07,98.6	0
0.07,98.7	0
0.07,98.8	0
0.07,98.9	0
0.07,99.0	0
0.07,99.1	0
0.07,99.2	0
0.07,99.3	0
0.07,99.4	0
0.07,99.5	0
0.07,99.6	0
0.07,99.7	0
0.07,99.8	0
0.07,99.9	0
0.07,100.0	0
0.08,0.0	0
0.08,0.1	0
0.08,0.2	0
0.08,0.3	0
0.08,0.4	0
0.08,0.5	0

0.08,0.6	0
0.08,0.7	0
0.08,0.8	0
0.08,0.9	0
0.08,1.0	0
0.08,1.1	0
0.08,1.2	0
0.08,1.3	0
0.08,1.4	0
0.08,1.5	0
0.08,1.6	0
0.08,1.7	0
0.08,1.8	0
0.08,1.9	0
0.08,2.0	0
0.08,2.1	0
0.08,2.2	0
0.08,2.3	0
0.08,2.4	0
0.08,2.5	0
0.08,2.6	0
0.08,2.7	0
0.08,2.8	0
0.08,2.9	0
0.08,3.0	0
0.08,3.1	0
0.08,3.2	0
0.08,3.3	0
0.08,3.4	0
0.08,3.5	0
0.08,3.6	0
0.08,3.7	0
0.08,3.8	0
0.08,3.9	0
0.08,4.0	0
0.08,4.1	0
0.08,4.2	0
0.08,4.3	0
0.08,4.4	0
0.08,4.5	0
0.08,4.6	0
0.08,4.7	0

0.08,4.8	0
0.08,4.9	0
0.08,5.0	0
0.08,5.1	0
0.08,5.2	0
0.08,5.3	0
0.08,5.4	0
0.08,5.5	0
0.08,5.6	0
0.08,5.7	0
0.08,5.8	0
0.08,5.9	0
0.08,6.0	0
0.08,6.1	0
0.08,6.2	0
0.08,6.3	0
0.08,6.4	0
0.08,6.5	0
0.08,6.6	0
0.08,6.7	0
0.08,6.8	0
0.08,6.9	0
0.08,7.0	0
0.08,7.1	0
0.08,7.2	0
0.08,7.3	0
0.08,7.4	0
0.08,7.5	0
0.08,7.6	0
0.08,7.7	0
0.08,7.8	0
0.08,7.9	0
0.08,8.0	0
0.08,8.1	0
0.08,8.2	0
0.08,8.3	0
0.08,8.4	0
0.08,8.5	0
0.08,8.6	0
0.08,8.7	0
0.08,8.8	0
0.08,8.9	0

0.08,9.0	0
0.08,9.1	0
0.08,9.2	0
0.08,9.3	0
0.08,9.4	0
0.08,9.5	0
0.08,9.6	0
0.08,9.7	0
0.08,9.8	0
0.08,9.9	0
0.08,10.0	0
0.08,10.1	0
0.08,10.2	0
0.08,10.3	0
0.08,10.4	0
0.08,10.5	0
0.08,10.6	0
0.08,10.7	0
0.08,10.8	0
0.08,10.9	0
0.08,11.0	0
0.08,11.1	0
0.08,11.2	0
0.08,11.3	0
0.08,11.4	0
0.08,11.5	0
0.08,11.6	0
0.08,11.7	0
0.08,11.8	0
0.08,11.9	0
0.08,12.0	0
0.08,12.1	0
0.08,12.2	0
0.08,12.3	0
0.08,12.4	0
0.08,12.5	0
0.08,12.6	0
0.08,12.7	0
0.08,12.8	0
0.08,12.9	0
0.08,13.0	0
0.08,13.1	0

0.08,13.2	0
0.08,13.3	0
0.08,13.4	0
0.08,13.5	0
0.08,13.6	0
0.08,13.7	0
0.08,13.8	0
0.08,13.9	0
0.08,14.0	0
0.08,14.1	0
0.08,14.2	0
0.08,14.3	0
0.08,14.4	0
0.08,14.5	0
0.08,14.6	0
0.08,14.7	0
0.08,14.8	0
0.08,14.9	0
0.08,15.0	0
0.08,15.1	0
0.08,15.2	0
0.08,15.3	0
0.08,15.4	0
0.08,15.5	0
0.08,15.6	0
0.08,15.7	0
0.08,15.8	0
0.08,15.9	0
0.08,16.0	0
0.08,16.1	0
0.08,16.2	0
0.08,16.3	0
0.08,16.4	0
0.08,16.5	0
0.08,16.6	0
0.08,16.7	0
0.08,16.8	0
0.08,16.9	0
0.08,17.0	0
0.08,17.1	0
0.08,17.2	0
0.08,17.3	0

0.08,17.4	0
0.08,17.5	0
0.08,17.6	0
0.08,17.7	0
0.08,17.8	0
0.08,17.9	0
0.08,18.0	0
0.08,18.1	0
0.08,18.2	0
0.08,18.3	0
0.08,18.4	0
0.08,18.5	0
0.08,18.6	0
0.08,18.7	0
0.08,18.8	0
0.08,18.9	0
0.08,19.0	0
0.08,19.1	0
0.08,19.2	0
0.08,19.3	0
0.08,19.4	0
0.08,19.5	0
0.08,19.6	0
0.08,19.7	0
0.08,19.8	0
0.08,19.9	0
0.08,20.0	0
0.08,20.1	0
0.08,20.2	0
0.08,20.3	0
0.08,20.4	0
0.08,20.5	0
0.08,20.6	0
0.08,20.7	0
0.08,20.8	0
0.08,20.9	0
0.08,21.0	0
0.08,21.1	0
0.08,21.2	0
0.08,21.3	0
0.08,21.4	0
0.08,21.5	0

0.08,21.6	0
0.08,21.7	0
0.08,21.8	0
0.08,21.9	0
0.08,22.0	0
0.08,22.1	0
0.08,22.2	0
0.08,22.3	0
0.08,22.4	0
0.08,22.5	0
0.08,22.6	0
0.08,22.7	0
0.08,22.8	0
0.08,22.9	0
0.08,23.0	0
0.08,23.1	0
0.08,23.2	0
0.08,23.3	0
0.08,23.4	0
0.08,23.5	0
0.08,23.6	0
0.08,23.7	0
0.08,23.8	0
0.08,23.9	0
0.08,24.0	0
0.08,24.1	0
0.08,24.2	0
0.08,24.3	0
0.08,24.4	0
0.08,24.5	0
0.08,24.6	0
0.08,24.7	0
0.08,24.8	0
0.08,24.9	0
0.08,25.0	0
0.08,25.1	0
0.08,25.2	0
0.08,25.3	0
0.08,25.4	0
0.08,25.5	0
0.08,25.6	0
0.08,25.7	0

0.08,25.8	0
0.08,25.9	0
0.08,26.0	0
0.08,26.1	0
0.08,26.2	0
0.08,26.3	0
0.08,26.4	0
0.08,26.5	0
0.08,26.6	0
0.08,26.7	0
0.08,26.8	0
0.08,26.9	0
0.08,27.0	0
0.08,27.1	0
0.08,27.2	0
0.08,27.3	0
0.08,27.4	0
0.08,27.5	0
0.08,27.6	0
0.08,27.7	0
0.08,27.8	0
0.08,27.9	0
0.08,28.0	0
0.08,28.1	0
0.08,28.2	0
0.08,28.3	0
0.08,28.4	0
0.08,28.5	0
0.08,28.6	0
0.08,28.7	0
0.08,28.8	0
0.08,28.9	0
0.08,29.0	0
0.08,29.1	0
0.08,29.2	0
0.08,29.3	0
0.08,29.4	0
0.08,29.5	0
0.08,29.6	0
0.08,29.7	0
0.08,29.8	0
0.08,29.9	0

0.08,30.0	0
0.08,30.1	0
0.08,30.2	0
0.08,30.3	0
0.08,30.4	0
0.08,30.5	0
0.08,30.6	0
0.08,30.7	0
0.08,30.8	0
0.08,30.9	0
0.08,31.0	0
0.08,31.1	0
0.08,31.2	0
0.08,31.3	0
0.08,31.4	0
0.08,31.5	0
0.08,31.6	0
0.08,31.7	0
0.08,31.8	0
0.08,31.9	0
0.08,32.0	0
0.08,32.1	0
0.08,32.2	0
0.08,32.3	0
0.08,32.4	0
0.08,32.5	0
0.08,32.6	0
0.08,32.7	0
0.08,32.8	0
0.08,32.9	0
0.08,33.0	0
0.08,33.1	0
0.08,33.2	0
0.08,33.3	0
0.08,33.4	0
0.08,33.5	0
0.08,33.6	0
0.08,33.7	0
0.08,33.8	0
0.08,33.9	0
0.08,34.0	0
0.08,34.1	0

0.08,34.2	0
0.08,34.3	0
0.08,34.4	0
0.08,34.5	0
0.08,34.6	0
0.08,34.7	0
0.08,34.8	0
0.08,34.9	0
0.08,35.0	0
0.08,35.1	0
0.08,35.2	0
0.08,35.3	0
0.08,35.4	0
0.08,35.5	0
0.08,35.6	0
0.08,35.7	0
0.08,35.8	0
0.08,35.9	0
0.08,36.0	0
0.08,36.1	0
0.08,36.2	0
0.08,36.3	0
0.08,36.4	0
0.08,36.5	0
0.08,36.6	0
0.08,36.7	0
0.08,36.8	0
0.08,36.9	0
0.08,37.0	0
0.08,37.1	0
0.08,37.2	0
0.08,37.3	0
0.08,37.4	0
0.08,37.5	0
0.08,37.6	0
0.08,37.7	0
0.08,37.8	0
0.08,37.9	0
0.08,38.0	0
0.08,38.1	0
0.08,38.2	0
0.08,38.3	0

0.08,38.4	0
0.08,38.5	0
0.08,38.6	0
0.08,38.7	0
0.08,38.8	0
0.08,38.9	0
0.08,39.0	0
0.08,39.1	0
0.08,39.2	0
0.08,39.3	0
0.08,39.4	0
0.08,39.5	0
0.08,39.6	0
0.08,39.7	0
0.08,39.8	0
0.08,39.9	0
0.08,40.0	0
0.08,40.1	0
0.08,40.2	0
0.08,40.3	0
0.08,40.4	0
0.08,40.5	0
0.08,40.6	0
0.08,40.7	0
0.08,40.8	0
0.08,40.9	0
0.08,41.0	0
0.08,41.1	0
0.08,41.2	0
0.08,41.3	0
0.08,41.4	0
0.08,41.5	0
0.08,41.6	0
0.08,41.7	0
0.08,41.8	0
0.08,41.9	0
0.08,42.0	0
0.08,42.1	0
0.08,42.2	0
0.08,42.3	0
0.08,42.4	0
0.08,42.5	0

0.08,42.6	0
0.08,42.7	0
0.08,42.8	0
0.08,42.9	0
0.08,43.0	0
0.08,43.1	0
0.08,43.2	0
0.08,43.3	0
0.08,43.4	0
0.08,43.5	0
0.08,43.6	0
0.08,43.7	0
0.08,43.8	0
0.08,43.9	0
0.08,44.0	0
0.08,44.1	0
0.08,44.2	0
0.08,44.3	0
0.08,44.4	0
0.08,44.5	0
0.08,44.6	0
0.08,44.7	0
0.08,44.8	0
0.08,44.9	0
0.08,45.0	0
0.08,45.1	0
0.08,45.2	0
0.08,45.3	0
0.08,45.4	0
0.08,45.5	0
0.08,45.6	0
0.08,45.7	0
0.08,45.8	0
0.08,45.9	0
0.08,46.0	0
0.08,46.1	0
0.08,46.2	0
0.08,46.3	0
0.08,46.4	0
0.08,46.5	0
0.08,46.6	0
0.08,46.7	0

0.08,46.8	0
0.08,46.9	0
0.08,47.0	0
0.08,47.1	0
0.08,47.2	0
0.08,47.3	0
0.08,47.4	0
0.08,47.5	0
0.08,47.6	0
0.08,47.7	0
0.08,47.8	0
0.08,47.9	0
0.08,48.0	0
0.08,48.1	0
0.08,48.2	0
0.08,48.3	0
0.08,48.4	0
0.08,48.5	0
0.08,48.6	0
0.08,48.7	0
0.08,48.8	0
0.08,48.9	0
0.08,49.0	0
0.08,49.1	0
0.08,49.2	0
0.08,49.3	0
0.08,49.4	0
0.08,49.5	0
0.08,49.6	0
0.08,49.7	0
0.08,49.8	0
0.08,49.9	0
0.08,50.0	0
0.08,50.1	0
0.08,50.2	0
0.08,50.3	0
0.08,50.4	0
0.08,50.5	0
0.08,50.6	0
0.08,50.7	0
0.08,50.8	0
0.08,50.9	0

0.08,51.0	0
0.08,51.1	0
0.08,51.2	0
0.08,51.3	0
0.08,51.4	0
0.08,51.5	0
0.08,51.6	0
0.08,51.7	0
0.08,51.8	0
0.08,51.9	0
0.08,52.0	0
0.08,52.1	0
0.08,52.2	0
0.08,52.3	0
0.08,52.4	0
0.08,52.5	0
0.08,52.6	0
0.08,52.7	0
0.08,52.8	0
0.08,52.9	0
0.08,53.0	0
0.08,53.1	0
0.08,53.2	0
0.08,53.3	0
0.08,53.4	0
0.08,53.5	0
0.08,53.6	0
0.08,53.7	0
0.08,53.8	0
0.08,53.9	0
0.08,54.0	0
0.08,54.1	0
0.08,54.2	0
0.08,54.3	0
0.08,54.4	0
0.08,54.5	0
0.08,54.6	0
0.08,54.7	0
0.08,54.8	0
0.08,54.9	0
0.08,55.0	0
0.08,55.1	0

0.08,55.2	0
0.08,55.3	0
0.08,55.4	0
0.08,55.5	0
0.08,55.6	0
0.08,55.7	0
0.08,55.8	0
0.08,55.9	0
0.08,56.0	0
0.08,56.1	0
0.08,56.2	0
0.08,56.3	0
0.08,56.4	0
0.08,56.5	0
0.08,56.6	0
0.08,56.7	0
0.08,56.8	0
0.08,56.9	0
0.08,57.0	0
0.08,57.1	0
0.08,57.2	0
0.08,57.3	0
0.08,57.4	0
0.08,57.5	0
0.08,57.6	0
0.08,57.7	0
0.08,57.8	0
0.08,57.9	0
0.08,58.0	0
0.08,58.1	0
0.08,58.2	0
0.08,58.3	0
0.08,58.4	0
0.08,58.5	0
0.08,58.6	0
0.08,58.7	0
0.08,58.8	0
0.08,58.9	0
0.08,59.0	0
0.08,59.1	0
0.08,59.2	0
0.08,59.3	0

0.08,59.4	0
0.08,59.5	0
0.08,59.6	0
0.08,59.7	0
0.08,59.8	0
0.08,59.9	0
0.08,60.0	0
0.08,60.1	0
0.08,60.2	0
0.08,60.3	0
0.08,60.4	0
0.08,60.5	0
0.08,60.6	0
0.08,60.7	0
0.08,60.8	0
0.08,60.9	0
0.08,61.0	0
0.08,61.1	0
0.08,61.2	0
0.08,61.3	0
0.08,61.4	0
0.08,61.5	0
0.08,61.6	0
0.08,61.7	0
0.08,61.8	0
0.08,61.9	0
0.08,62.0	0
0.08,62.1	0
0.08,62.2	0
0.08,62.3	0
0.08,62.4	0
0.08,62.5	0
0.08,62.6	0
0.08,62.7	0
0.08,62.8	0
0.08,62.9	0
0.08,63.0	0
0.08,63.1	0
0.08,63.2	0
0.08,63.3	0
0.08,63.4	0
0.08,63.5	0

0.08,63.6	0
0.08,63.7	0
0.08,63.8	0
0.08,63.9	0
0.08,64.0	0
0.08,64.1	0
0.08,64.2	0
0.08,64.3	0
0.08,64.4	0
0.08,64.5	0
0.08,64.6	0
0.08,64.7	0
0.08,64.8	0
0.08,64.9	0
0.08,65.0	0
0.08,65.1	0
0.08,65.2	0
0.08,65.3	0
0.08,65.4	0
0.08,65.5	0
0.08,65.6	0
0.08,65.7	0
0.08,65.8	0
0.08,65.9	0
0.08,66.0	0
0.08,66.1	0
0.08,66.2	0
0.08,66.3	0
0.08,66.4	0
0.08,66.5	0
0.08,66.6	0
0.08,66.7	0
0.08,66.8	0
0.08,66.9	0
0.08,67.0	0
0.08,67.1	0
0.08,67.2	0
0.08,67.3	0
0.08,67.4	0
0.08,67.5	0
0.08,67.6	0
0.08,67.7	0

0.08,67.8	0
0.08,67.9	0
0.08,68.0	0
0.08,68.1	0
0.08,68.2	0
0.08,68.3	0
0.08,68.4	0
0.08,68.5	0
0.08,68.6	0
0.08,68.7	0
0.08,68.8	0
0.08,68.9	0
0.08,69.0	0
0.08,69.1	0
0.08,69.2	0
0.08,69.3	0
0.08,69.4	0
0.08,69.5	0
0.08,69.6	0
0.08,69.7	0
0.08,69.8	0
0.08,69.9	0
0.08,70.0	0
0.08,70.1	0
0.08,70.2	0
0.08,70.3	0
0.08,70.4	0
0.08,70.5	0
0.08,70.6	0
0.08,70.7	0
0.08,70.8	0
0.08,70.9	0
0.08,71.0	0
0.08,71.1	0
0.08,71.2	0
0.08,71.3	0
0.08,71.4	0
0.08,71.5	0
0.08,71.6	0
0.08,71.7	0
0.08,71.8	0
0.08,71.9	0

0.08,72.0	0
0.08,72.1	0
0.08,72.2	0
0.08,72.3	0
0.08,72.4	0
0.08,72.5	0
0.08,72.6	0
0.08,72.7	0
0.08,72.8	0
0.08,72.9	0
0.08,73.0	0
0.08,73.1	0
0.08,73.2	0
0.08,73.3	0
0.08,73.4	0
0.08,73.5	0
0.08,73.6	0
0.08,73.7	0
0.08,73.8	0
0.08,73.9	0
0.08,74.0	0
0.08,74.1	0
0.08,74.2	0
0.08,74.3	0
0.08,74.4	0
0.08,74.5	0
0.08,74.6	0
0.08,74.7	0
0.08,74.8	0
0.08,74.9	0
0.08,75.0	0
0.08,75.1	0
0.08,75.2	0
0.08,75.3	0
0.08,75.4	0
0.08,75.5	0
0.08,75.6	0
0.08,75.7	0
0.08,75.8	0
0.08,75.9	0
0.08,76.0	0
0.08,76.1	0

0.08,76.2	0
0.08,76.3	0
0.08,76.4	0
0.08,76.5	0
0.08,76.6	0
0.08,76.7	0
0.08,76.8	0
0.08,76.9	0
0.08,77.0	0
0.08,77.1	0
0.08,77.2	0
0.08,77.3	0
0.08,77.4	0
0.08,77.5	0
0.08,77.6	0
0.08,77.7	0
0.08,77.8	0
0.08,77.9	0
0.08,78.0	0
0.08,78.1	0
0.08,78.2	0
0.08,78.3	0
0.08,78.4	0
0.08,78.5	0
0.08,78.6	0
0.08,78.7	0
0.08,78.8	0
0.08,78.9	0
0.08,79.0	0
0.08,79.1	0
0.08,79.2	0
0.08,79.3	0
0.08,79.4	0
0.08,79.5	0
0.08,79.6	0
0.08,79.7	0
0.08,79.8	0
0.08,79.9	0
0.08,80.0	0
0.08,80.1	0
0.08,80.2	0
0.08,80.3	0

0.08,80.4	0
0.08,80.5	0
0.08,80.6	0
0.08,80.7	0
0.08,80.8	0
0.08,80.9	0
0.08,81.0	0
0.08,81.1	0
0.08,81.2	0
0.08,81.3	0
0.08,81.4	0
0.08,81.5	0
0.08,81.6	0
0.08,81.7	0
0.08,81.8	0
0.08,81.9	0
0.08,82.0	0
0.08,82.1	0
0.08,82.2	0
0.08,82.3	0
0.08,82.4	0
0.08,82.5	0
0.08,82.6	0
0.08,82.7	0
0.08,82.8	0
0.08,82.9	0
0.08,83.0	0
0.08,83.1	0
0.08,83.2	0
0.08,83.3	0
0.08,83.4	0
0.08,83.5	0
0.08,83.6	0
0.08,83.7	0
0.08,83.8	0
0.08,83.9	0
0.08,84.0	0
0.08,84.1	0
0.08,84.2	0
0.08,84.3	0
0.08,84.4	0
0.08,84.5	0

0.08,84.6	0
0.08,84.7	0
0.08,84.8	0
0.08,84.9	0
0.08,85.0	0
0.08,85.1	0
0.08,85.2	0
0.08,85.3	0
0.08,85.4	0
0.08,85.5	0
0.08,85.6	0
0.08,85.7	0
0.08,85.8	0
0.08,85.9	0
0.08,86.0	0
0.08,86.1	0
0.08,86.2	0
0.08,86.3	0
0.08,86.4	0
0.08,86.5	0
0.08,86.6	0
0.08,86.7	0
0.08,86.8	0
0.08,86.9	0
0.08,87.0	0
0.08,87.1	0
0.08,87.2	0
0.08,87.3	0
0.08,87.4	0
0.08,87.5	0
0.08,87.6	0
0.08,87.7	0
0.08,87.8	0
0.08,87.9	0
0.08,88.0	0
0.08,88.1	0
0.08,88.2	0
0.08,88.3	0
0.08,88.4	0
0.08,88.5	0
0.08,88.6	0
0.08,88.7	0

0.08,88.8	0
0.08,88.9	0
0.08,89.0	0
0.08,89.1	0
0.08,89.2	0
0.08,89.3	0
0.08,89.4	0
0.08,89.5	0
0.08,89.6	0
0.08,89.7	0
0.08,89.8	0
0.08,89.9	0
0.08,90.0	0
0.08,90.1	0
0.08,90.2	0
0.08,90.3	0
0.08,90.4	0
0.08,90.5	0
0.08,90.6	0
0.08,90.7	0
0.08,90.8	0
0.08,90.9	0
0.08,91.0	0
0.08,91.1	0
0.08,91.2	0
0.08,91.3	0
0.08,91.4	0
0.08,91.5	0
0.08,91.6	0
0.08,91.7	0
0.08,91.8	0
0.08,91.9	0
0.08,92.0	0
0.08,92.1	0
0.08,92.2	0
0.08,92.3	0
0.08,92.4	0
0.08,92.5	0
0.08,92.6	0
0.08,92.7	0
0.08,92.8	0
0.08,92.9	0

0.08,93.0	0
0.08,93.1	0
0.08,93.2	0
0.08,93.3	0
0.08,93.4	0
0.08,93.5	0
0.08,93.6	0
0.08,93.7	0
0.08,93.8	0
0.08,93.9	0
0.08,94.0	0
0.08,94.1	0
0.08,94.2	0
0.08,94.3	0
0.08,94.4	0
0.08,94.5	0
0.08,94.6	0
0.08,94.7	0
0.08,94.8	0
0.08,94.9	0
0.08,95.0	0
0.08,95.1	0
0.08,95.2	0
0.08,95.3	0
0.08,95.4	0
0.08,95.5	0
0.08,95.6	0
0.08,95.7	0
0.08,95.8	0
0.08,95.9	0
0.08,96.0	0
0.08,96.1	0
0.08,96.2	0
0.08,96.3	0
0.08,96.4	0
0.08,96.5	0
0.08,96.6	0
0.08,96.7	0
0.08,96.8	0
0.08,96.9	0
0.08,97.0	0
0.08,97.1	0

0.08,97.2	0
0.08,97.3	0
0.08,97.4	0
0.08,97.5	0
0.08,97.6	0
0.08,97.7	0
0.08,97.8	0
0.08,97.9	0
0.08,98.0	0
0.08,98.1	0
0.08,98.2	0
0.08,98.3	0
0.08,98.4	0
0.08,98.5	0
0.08,98.6	0
0.08,98.7	0
0.08,98.8	0
0.08,98.9	0
0.08,99.0	0
0.08,99.1	0
0.08,99.2	0
0.08,99.3	0
0.08,99.4	0
0.08,99.5	0
0.08,99.6	0
0.08,99.7	0
0.08,99.8	0
0.08,99.9	0
0.08,100.0	0
0.09,0.0	0
0.09,0.1	0
0.09,0.2	0
0.09,0.3	0
0.09,0.4	0
0.09,0.5	0
0.09,0.6	0
0.09,0.7	0
0.09,0.8	0
0.09,0.9	0
0.09,1.0	0
0.09,1.1	0
0.09,1.2	0

0.09,1.3	0
0.09,1.4	0
0.09,1.5	0
0.09,1.6	0
0.09,1.7	0
0.09,1.8	0
0.09,1.9	0
0.09,2.0	0
0.09,2.1	0
0.09,2.2	0
0.09,2.3	0
0.09,2.4	0
0.09,2.5	0
0.09,2.6	0
0.09,2.7	0
0.09,2.8	0
0.09,2.9	0
0.09,3.0	0
0.09,3.1	0
0.09,3.2	0
0.09,3.3	0
0.09,3.4	0
0.09,3.5	0
0.09,3.6	0
0.09,3.7	0
0.09,3.8	0
0.09,3.9	0
0.09,4.0	0
0.09,4.1	0
0.09,4.2	0
0.09,4.3	0
0.09,4.4	0
0.09,4.5	0
0.09,4.6	0
0.09,4.7	0
0.09,4.8	0
0.09,4.9	0
0.09,5.0	0
0.09,5.1	0
0.09,5.2	0
0.09,5.3	0
0.09,5.4	0

0.09,5.5	0
0.09,5.6	0
0.09,5.7	0
0.09,5.8	0
0.09,5.9	0
0.09,6.0	0
0.09,6.1	0
0.09,6.2	0
0.09,6.3	0
0.09,6.4	0
0.09,6.5	0
0.09,6.6	0
0.09,6.7	0
0.09,6.8	0
0.09,6.9	0
0.09,7.0	0
0.09,7.1	0
0.09,7.2	0
0.09,7.3	0
0.09,7.4	0
0.09,7.5	0
0.09,7.6	0
0.09,7.7	0
0.09,7.8	0
0.09,7.9	0
0.09,8.0	0
0.09,8.1	0
0.09,8.2	0
0.09,8.3	0
0.09,8.4	0
0.09,8.5	0
0.09,8.6	0
0.09,8.7	0
0.09,8.8	0
0.09,8.9	0
0.09,9.0	0
0.09,9.1	0
0.09,9.2	0
0.09,9.3	0
0.09,9.4	0
0.09,9.5	0
0.09,9.6	0

0.09,9.7	0
0.09,9.8	0
0.09,9.9	0
0.09,10.0	0
0.09,10.1	0
0.09,10.2	0
0.09,10.3	0
0.09,10.4	0
0.09,10.5	0
0.09,10.6	0
0.09,10.7	0
0.09,10.8	0
0.09,10.9	0
0.09,11.0	0
0.09,11.1	0
0.09,11.2	0
0.09,11.3	0
0.09,11.4	0
0.09,11.5	0
0.09,11.6	0
0.09,11.7	0
0.09,11.8	0
0.09,11.9	0
0.09,12.0	0
0.09,12.1	0
0.09,12.2	0
0.09,12.3	0
0.09,12.4	0
0.09,12.5	0
0.09,12.6	0
0.09,12.7	0
0.09,12.8	0
0.09,12.9	0
0.09,13.0	0
0.09,13.1	0
0.09,13.2	0
0.09,13.3	0
0.09,13.4	0
0.09,13.5	0
0.09,13.6	0
0.09,13.7	0
0.09,13.8	0

0.09,13.9	0
0.09,14.0	0
0.09,14.1	0
0.09,14.2	0
0.09,14.3	0
0.09,14.4	0
0.09,14.5	0
0.09,14.6	0
0.09,14.7	0
0.09,14.8	0
0.09,14.9	0
0.09,15.0	0
0.09,15.1	0
0.09,15.2	0
0.09,15.3	0
0.09,15.4	0
0.09,15.5	0
0.09,15.6	0
0.09,15.7	0
0.09,15.8	0
0.09,15.9	0
0.09,16.0	0
0.09,16.1	0
0.09,16.2	0
0.09,16.3	0
0.09,16.4	0
0.09,16.5	0
0.09,16.6	0
0.09,16.7	0
0.09,16.8	0
0.09,16.9	0
0.09,17.0	0
0.09,17.1	0
0.09,17.2	0
0.09,17.3	0
0.09,17.4	0
0.09,17.5	0
0.09,17.6	0
0.09,17.7	0
0.09,17.8	0
0.09,17.9	0
0.09,18.0	0

0.09,18.1	0
0.09,18.2	0
0.09,18.3	0
0.09,18.4	0
0.09,18.5	0
0.09,18.6	0
0.09,18.7	0
0.09,18.8	0
0.09,18.9	0
0.09,19.0	0
0.09,19.1	0
0.09,19.2	0
0.09,19.3	0
0.09,19.4	0
0.09,19.5	0
0.09,19.6	0
0.09,19.7	0
0.09,19.8	0
0.09,19.9	0
0.09,20.0	0
0.09,20.1	0
0.09,20.2	0
0.09,20.3	0
0.09,20.4	0
0.09,20.5	0
0.09,20.6	0
0.09,20.7	0
0.09,20.8	0
0.09,20.9	0
0.09,21.0	0
0.09,21.1	0
0.09,21.2	0
0.09,21.3	0
0.09,21.4	0
0.09,21.5	0
0.09,21.6	0
0.09,21.7	0
0.09,21.8	0
0.09,21.9	0
0.09,22.0	0
0.09,22.1	0
0.09,22.2	0

0.09,22.3	0
0.09,22.4	0
0.09,22.5	0
0.09,22.6	0
0.09,22.7	0
0.09,22.8	0
0.09,22.9	0
0.09,23.0	0
0.09,23.1	0
0.09,23.2	0
0.09,23.3	0
0.09,23.4	0
0.09,23.5	0
0.09,23.6	0
0.09,23.7	0
0.09,23.8	0
0.09,23.9	0
0.09,24.0	0
0.09,24.1	0
0.09,24.2	0
0.09,24.3	0
0.09,24.4	0
0.09,24.5	0
0.09,24.6	0
0.09,24.7	0
0.09,24.8	0
0.09,24.9	0
0.09,25.0	0
0.09,25.1	0
0.09,25.2	0
0.09,25.3	0
0.09,25.4	0
0.09,25.5	0
0.09,25.6	0
0.09,25.7	0
0.09,25.8	0
0.09,25.9	0
0.09,26.0	0
0.09,26.1	0
0.09,26.2	0
0.09,26.3	0
0.09,26.4	0

0.09,26.5	0
0.09,26.6	0
0.09,26.7	0
0.09,26.8	0
0.09,26.9	0
0.09,27.0	0
0.09,27.1	0
0.09,27.2	0
0.09,27.3	0
0.09,27.4	0
0.09,27.5	0
0.09,27.6	0
0.09,27.7	0
0.09,27.8	0
0.09,27.9	0
0.09,28.0	0
0.09,28.1	0
0.09,28.2	0
0.09,28.3	0
0.09,28.4	0
0.09,28.5	0
0.09,28.6	0
0.09,28.7	0
0.09,28.8	0
0.09,28.9	0
0.09,29.0	0
0.09,29.1	0
0.09,29.2	0
0.09,29.3	0
0.09,29.4	0
0.09,29.5	0
0.09,29.6	0
0.09,29.7	0
0.09,29.8	0
0.09,29.9	0
0.09,30.0	0
0.09,30.1	0
0.09,30.2	0
0.09,30.3	0
0.09,30.4	0
0.09,30.5	0
0.09,30.6	0

0.09,30.7	0
0.09,30.8	0
0.09,30.9	0
0.09,31.0	0
0.09,31.1	0
0.09,31.2	0
0.09,31.3	0
0.09,31.4	0
0.09,31.5	0
0.09,31.6	0
0.09,31.7	0
0.09,31.8	0
0.09,31.9	0
0.09,32.0	0
0.09,32.1	0
0.09,32.2	0
0.09,32.3	0
0.09,32.4	0
0.09,32.5	0
0.09,32.6	0
0.09,32.7	0
0.09,32.8	0
0.09,32.9	0
0.09,33.0	0
0.09,33.1	0
0.09,33.2	0
0.09,33.3	0
0.09,33.4	0
0.09,33.5	0
0.09,33.6	0
0.09,33.7	0
0.09,33.8	0
0.09,33.9	0
0.09,34.0	0
0.09,34.1	0
0.09,34.2	0
0.09,34.3	0
0.09,34.4	0
0.09,34.5	0
0.09,34.6	0
0.09,34.7	0
0.09,34.8	0

0.09,34.9	0
0.09,35.0	0
0.09,35.1	0
0.09,35.2	0
0.09,35.3	0
0.09,35.4	0
0.09,35.5	0
0.09,35.6	0
0.09,35.7	0
0.09,35.8	0
0.09,35.9	0
0.09,36.0	0
0.09,36.1	0
0.09,36.2	0
0.09,36.3	0
0.09,36.4	0
0.09,36.5	0
0.09,36.6	0
0.09,36.7	0
0.09,36.8	0
0.09,36.9	0
0.09,37.0	0
0.09,37.1	0
0.09,37.2	0
0.09,37.3	0
0.09,37.4	0
0.09,37.5	0
0.09,37.6	0
0.09,37.7	0
0.09,37.8	0
0.09,37.9	0
0.09,38.0	0
0.09,38.1	0
0.09,38.2	0
0.09,38.3	0
0.09,38.4	0
0.09,38.5	0
0.09,38.6	0
0.09,38.7	0
0.09,38.8	0
0.09,38.9	0
0.09,39.0	0

0.09,39.1	0
0.09,39.2	0
0.09,39.3	0
0.09,39.4	0
0.09,39.5	0
0.09,39.6	0
0.09,39.7	0
0.09,39.8	0
0.09,39.9	0
0.09,40.0	0
0.09,40.1	0
0.09,40.2	0
0.09,40.3	0
0.09,40.4	0
0.09,40.5	0
0.09,40.6	0
0.09,40.7	0
0.09,40.8	0
0.09,40.9	0
0.09,41.0	0
0.09,41.1	0
0.09,41.2	0
0.09,41.3	0
0.09,41.4	0
0.09,41.5	0
0.09,41.6	0
0.09,41.7	0
0.09,41.8	0
0.09,41.9	0
0.09,42.0	0
0.09,42.1	0
0.09,42.2	0
0.09,42.3	0
0.09,42.4	0
0.09,42.5	0
0.09,42.6	0
0.09,42.7	0
0.09,42.8	0
0.09,42.9	0
0.09,43.0	0
0.09,43.1	0
0.09,43.2	0

0.09,43.3	0
0.09,43.4	0
0.09,43.5	0
0.09,43.6	0
0.09,43.7	0
0.09,43.8	0
0.09,43.9	0
0.09,44.0	0
0.09,44.1	0
0.09,44.2	0
0.09,44.3	0
0.09,44.4	0
0.09,44.5	0
0.09,44.6	0
0.09,44.7	0
0.09,44.8	0
0.09,44.9	0
0.09,45.0	0
0.09,45.1	0
0.09,45.2	0
0.09,45.3	0
0.09,45.4	0
0.09,45.5	0
0.09,45.6	0
0.09,45.7	0
0.09,45.8	0
0.09,45.9	0
0.09,46.0	0
0.09,46.1	0
0.09,46.2	0
0.09,46.3	0
0.09,46.4	0
0.09,46.5	0
0.09,46.6	0
0.09,46.7	0
0.09,46.8	0
0.09,46.9	0
0.09,47.0	0
0.09,47.1	0
0.09,47.2	0
0.09,47.3	0
0.09,47.4	0

0.09,47.5	0
0.09,47.6	0
0.09,47.7	0
0.09,47.8	0
0.09,47.9	0
0.09,48.0	0
0.09,48.1	0
0.09,48.2	0
0.09,48.3	0
0.09,48.4	0
0.09,48.5	0
0.09,48.6	0
0.09,48.7	0
0.09,48.8	0
0.09,48.9	0
0.09,49.0	0
0.09,49.1	0
0.09,49.2	0
0.09,49.3	0
0.09,49.4	0
0.09,49.5	0
0.09,49.6	0
0.09,49.7	0
0.09,49.8	0
0.09,49.9	0
0.09,50.0	0
0.09,50.1	0
0.09,50.2	0
0.09,50.3	0
0.09,50.4	0
0.09,50.5	0
0.09,50.6	0
0.09,50.7	0
0.09,50.8	0
0.09,50.9	0
0.09,51.0	0
0.09,51.1	0
0.09,51.2	0
0.09,51.3	0
0.09,51.4	0
0.09,51.5	0
0.09,51.6	0

0.09,51.7	0
0.09,51.8	0
0.09,51.9	0
0.09,52.0	0
0.09,52.1	0
0.09,52.2	0
0.09,52.3	0
0.09,52.4	0
0.09,52.5	0
0.09,52.6	0
0.09,52.7	0
0.09,52.8	0
0.09,52.9	0
0.09,53.0	0
0.09,53.1	0
0.09,53.2	0
0.09,53.3	0
0.09,53.4	0
0.09,53.5	0
0.09,53.6	0
0.09,53.7	0
0.09,53.8	0
0.09,53.9	0
0.09,54.0	0
0.09,54.1	0
0.09,54.2	0
0.09,54.3	0
0.09,54.4	0
0.09,54.5	0
0.09,54.6	0
0.09,54.7	0
0.09,54.8	0
0.09,54.9	0
0.09,55.0	0
0.09,55.1	0
0.09,55.2	0
0.09,55.3	0
0.09,55.4	0
0.09,55.5	0
0.09,55.6	0
0.09,55.7	0
0.09,55.8	0

0.09,55.9	0
0.09,56.0	0
0.09,56.1	0
0.09,56.2	0
0.09,56.3	0
0.09,56.4	0
0.09,56.5	0
0.09,56.6	0
0.09,56.7	0
0.09,56.8	0
0.09,56.9	0
0.09,57.0	0
0.09,57.1	0
0.09,57.2	0
0.09,57.3	0
0.09,57.4	0
0.09,57.5	0
0.09,57.6	0
0.09,57.7	0
0.09,57.8	0
0.09,57.9	0
0.09,58.0	0
0.09,58.1	0
0.09,58.2	0
0.09,58.3	0
0.09,58.4	0
0.09,58.5	0
0.09,58.6	0
0.09,58.7	0
0.09,58.8	0
0.09,58.9	0
0.09,59.0	0
0.09,59.1	0
0.09,59.2	0
0.09,59.3	0
0.09,59.4	0
0.09,59.5	0
0.09,59.6	0
0.09,59.7	0
0.09,59.8	0
0.09,59.9	0
0.09,60.0	0

0.09,60.1	0
0.09,60.2	0
0.09,60.3	0
0.09,60.4	0
0.09,60.5	0
0.09,60.6	0
0.09,60.7	0
0.09,60.8	0
0.09,60.9	0
0.09,61.0	0
0.09,61.1	0
0.09,61.2	0
0.09,61.3	0
0.09,61.4	0
0.09,61.5	0
0.09,61.6	0
0.09,61.7	0
0.09,61.8	0
0.09,61.9	0
0.09,62.0	0
0.09,62.1	0
0.09,62.2	0
0.09,62.3	0
0.09,62.4	0
0.09,62.5	0
0.09,62.6	0
0.09,62.7	0
0.09,62.8	0
0.09,62.9	0
0.09,63.0	0
0.09,63.1	0
0.09,63.2	0
0.09,63.3	0
0.09,63.4	0
0.09,63.5	0
0.09,63.6	0
0.09,63.7	0
0.09,63.8	0
0.09,63.9	0
0.09,64.0	0
0.09,64.1	0
0.09,64.2	0

0.09,64.3	0
0.09,64.4	0
0.09,64.5	0
0.09,64.6	0
0.09,64.7	0
0.09,64.8	0
0.09,64.9	0
0.09,65.0	0
0.09,65.1	0
0.09,65.2	0
0.09,65.3	0
0.09,65.4	0
0.09,65.5	0
0.09,65.6	0
0.09,65.7	0
0.09,65.8	0
0.09,65.9	0
0.09,66.0	0
0.09,66.1	0
0.09,66.2	0
0.09,66.3	0
0.09,66.4	0
0.09,66.5	0
0.09,66.6	0
0.09,66.7	0
0.09,66.8	0
0.09,66.9	0
0.09,67.0	0
0.09,67.1	0
0.09,67.2	0
0.09,67.3	0
0.09,67.4	0
0.09,67.5	0
0.09,67.6	0
0.09,67.7	0
0.09,67.8	0
0.09,67.9	0
0.09,68.0	0
0.09,68.1	0
0.09,68.2	0
0.09,68.3	0
0.09,68.4	0

0.09,68.5	0
0.09,68.6	0
0.09,68.7	0
0.09,68.8	0
0.09,68.9	0
0.09,69.0	0
0.09,69.1	0
0.09,69.2	0
0.09,69.3	0
0.09,69.4	0
0.09,69.5	0
0.09,69.6	0
0.09,69.7	0
0.09,69.8	0
0.09,69.9	0
0.09,70.0	0
0.09,70.1	0
0.09,70.2	0
0.09,70.3	0
0.09,70.4	0
0.09,70.5	0
0.09,70.6	0
0.09,70.7	0
0.09,70.8	0
0.09,70.9	0
0.09,71.0	0
0.09,71.1	0
0.09,71.2	0
0.09,71.3	0
0.09,71.4	0
0.09,71.5	0
0.09,71.6	0
0.09,71.7	0
0.09,71.8	0
0.09,71.9	0
0.09,72.0	0
0.09,72.1	0
0.09,72.2	0
0.09,72.3	0
0.09,72.4	0
0.09,72.5	0
0.09,72.6	0

0.09,72.7	0
0.09,72.8	0
0.09,72.9	0
0.09,73.0	0
0.09,73.1	0
0.09,73.2	0
0.09,73.3	0
0.09,73.4	0
0.09,73.5	0
0.09,73.6	0
0.09,73.7	0
0.09,73.8	0
0.09,73.9	0
0.09,74.0	0
0.09,74.1	0
0.09,74.2	0
0.09,74.3	0
0.09,74.4	0
0.09,74.5	0
0.09,74.6	0
0.09,74.7	0
0.09,74.8	0
0.09,74.9	0
0.09,75.0	0
0.09,75.1	0
0.09,75.2	0
0.09,75.3	0
0.09,75.4	0
0.09,75.5	0
0.09,75.6	0
0.09,75.7	0
0.09,75.8	0
0.09,75.9	0
0.09,76.0	0
0.09,76.1	0
0.09,76.2	0
0.09,76.3	0
0.09,76.4	0
0.09,76.5	0
0.09,76.6	0
0.09,76.7	0
0.09,76.8	0

0.09,76.9	0
0.09,77.0	0
0.09,77.1	0
0.09,77.2	0
0.09,77.3	0
0.09,77.4	0
0.09,77.5	0
0.09,77.6	0
0.09,77.7	0
0.09,77.8	0
0.09,77.9	0
0.09,78.0	0
0.09,78.1	0
0.09,78.2	0
0.09,78.3	0
0.09,78.4	0
0.09,78.5	0
0.09,78.6	0
0.09,78.7	0
0.09,78.8	0
0.09,78.9	0
0.09,79.0	0
0.09,79.1	0
0.09,79.2	0
0.09,79.3	0
0.09,79.4	0
0.09,79.5	0
0.09,79.6	0
0.09,79.7	0
0.09,79.8	0
0.09,79.9	0
0.09,80.0	0
0.09,80.1	0
0.09,80.2	0
0.09,80.3	0
0.09,80.4	0
0.09,80.5	0
0.09,80.6	0
0.09,80.7	0
0.09,80.8	0
0.09,80.9	0
0.09,81.0	0

0.09,81.1	0
0.09,81.2	0
0.09,81.3	0
0.09,81.4	0
0.09,81.5	0
0.09,81.6	0
0.09,81.7	0
0.09,81.8	0
0.09,81.9	0
0.09,82.0	0
0.09,82.1	0
0.09,82.2	0
0.09,82.3	0
0.09,82.4	0
0.09,82.5	0
0.09,82.6	0
0.09,82.7	0
0.09,82.8	0
0.09,82.9	0
0.09,83.0	0
0.09,83.1	0
0.09,83.2	0
0.09,83.3	0
0.09,83.4	0
0.09,83.5	0
0.09,83.6	0
0.09,83.7	0
0.09,83.8	0
0.09,83.9	0
0.09,84.0	0
0.09,84.1	0
0.09,84.2	0
0.09,84.3	0
0.09,84.4	0
0.09,84.5	0
0.09,84.6	0
0.09,84.7	0
0.09,84.8	0
0.09,84.9	0
0.09,85.0	0
0.09,85.1	0
0.09,85.2	0

0.09,85.3	0
0.09,85.4	0
0.09,85.5	0
0.09,85.6	0
0.09,85.7	0
0.09,85.8	0
0.09,85.9	0
0.09,86.0	0
0.09,86.1	0
0.09,86.2	0
0.09,86.3	0
0.09,86.4	0
0.09,86.5	0
0.09,86.6	0
0.09,86.7	0
0.09,86.8	0
0.09,86.9	0
0.09,87.0	0
0.09,87.1	0
0.09,87.2	0
0.09,87.3	0
0.09,87.4	0
0.09,87.5	0
0.09,87.6	0
0.09,87.7	0
0.09,87.8	0
0.09,87.9	0
0.09,88.0	0
0.09,88.1	0
0.09,88.2	0
0.09,88.3	0
0.09,88.4	0
0.09,88.5	0
0.09,88.6	0
0.09,88.7	0
0.09,88.8	0
0.09,88.9	0
0.09,89.0	0
0.09,89.1	0
0.09,89.2	0
0.09,89.3	0
0.09,89.4	0

0.09,89.5	0
0.09,89.6	0
0.09,89.7	0
0.09,89.8	0
0.09,89.9	0
0.09,90.0	0
0.09,90.1	0
0.09,90.2	0
0.09,90.3	0
0.09,90.4	0
0.09,90.5	0
0.09,90.6	0
0.09,90.7	0
0.09,90.8	0
0.09,90.9	0
0.09,91.0	0
0.09,91.1	0
0.09,91.2	0
0.09,91.3	0
0.09,91.4	0
0.09,91.5	0
0.09,91.6	0
0.09,91.7	0
0.09,91.8	0
0.09,91.9	0
0.09,92.0	0
0.09,92.1	0
0.09,92.2	0
0.09,92.3	0
0.09,92.4	0
0.09,92.5	0
0.09,92.6	0
0.09,92.7	0
0.09,92.8	0
0.09,92.9	0
0.09,93.0	0
0.09,93.1	0
0.09,93.2	0
0.09,93.3	0
0.09,93.4	0
0.09,93.5	0
0.09,93.6	0

0.09,93.7	0
0.09,93.8	0
0.09,93.9	0
0.09,94.0	0
0.09,94.1	0
0.09,94.2	0
0.09,94.3	0
0.09,94.4	0
0.09,94.5	0
0.09,94.6	0
0.09,94.7	0
0.09,94.8	0
0.09,94.9	0
0.09,95.0	0
0.09,95.1	0
0.09,95.2	0
0.09,95.3	0
0.09,95.4	0
0.09,95.5	0
0.09,95.6	0
0.09,95.7	0
0.09,95.8	0
0.09,95.9	0
0.09,96.0	0
0.09,96.1	0
0.09,96.2	0
0.09,96.3	0
0.09,96.4	0
0.09,96.5	0
0.09,96.6	0
0.09,96.7	0
0.09,96.8	0
0.09,96.9	0
0.09,97.0	0
0.09,97.1	0
0.09,97.2	0
0.09,97.3	0
0.09,97.4	0
0.09,97.5	0
0.09,97.6	0
0.09,97.7	0
0.09,97.8	0

0.09,97.9	0
0.09,98.0	0
0.09,98.1	0
0.09,98.2	0
0.09,98.3	0
0.09,98.4	0
0.09,98.5	0
0.09,98.6	0
0.09,98.7	0
0.09,98.8	0
0.09,98.9	0
0.09,99.0	0
0.09,99.1	0
0.09,99.2	0
0.09,99.3	0
0.09,99.4	0
0.09,99.5	0
0.09,99.6	0
0.09,99.7	0
0.09,99.8	0
0.09,99.9	0
0.09,100.0	0
0.10,0.0	0
0.10,0.1	0
0.10,0.2	0
0.10,0.3	0
0.10,0.4	0
0.10,0.5	0
0.10,0.6	0
0.10,0.7	0
0.10,0.8	0
0.10,0.9	0
0.10,1.0	0
0.10,1.1	0
0.10,1.2	0
0.10,1.3	0
0.10,1.4	0
0.10,1.5	0
0.10,1.6	0
0.10,1.7	0
0.10,1.8	0
0.10,1.9	0

0.10,2.0	0
0.10,2.1	0
0.10,2.2	0
0.10,2.3	0
0.10,2.4	0
0.10,2.5	0
0.10,2.6	0
0.10,2.7	0
0.10,2.8	0
0.10,2.9	0
0.10,3.0	0
0.10,3.1	0
0.10,3.2	0
0.10,3.3	0
0.10,3.4	0
0.10,3.5	0
0.10,3.6	0
0.10,3.7	0
0.10,3.8	0
0.10,3.9	0
0.10,4.0	0
0.10,4.1	0
0.10,4.2	0
0.10,4.3	0
0.10,4.4	0
0.10,4.5	0
0.10,4.6	0
0.10,4.7	0
0.10,4.8	0
0.10,4.9	0
0.10,5.0	0
0.10,5.1	0
0.10,5.2	0
0.10,5.3	0
0.10,5.4	0
0.10,5.5	0
0.10,5.6	0
0.10,5.7	0
0.10,5.8	0
0.10,5.9	0
0.10,6.0	0
0.10,6.1	0

0.10,6.2	0
0.10,6.3	0
0.10,6.4	0
0.10,6.5	0
0.10,6.6	0
0.10,6.7	0
0.10,6.8	0
0.10,6.9	0
0.10,7.0	0
0.10,7.1	0
0.10,7.2	0
0.10,7.3	0
0.10,7.4	0
0.10,7.5	0
0.10,7.6	0
0.10,7.7	0
0.10,7.8	0
0.10,7.9	0
0.10,8.0	0
0.10,8.1	0
0.10,8.2	0
0.10,8.3	0
0.10,8.4	0
0.10,8.5	0
0.10,8.6	0
0.10,8.7	0
0.10,8.8	0
0.10,8.9	0
0.10,9.0	0
0.10,9.1	0
0.10,9.2	0
0.10,9.3	0
0.10,9.4	0
0.10,9.5	0
0.10,9.6	0
0.10,9.7	0
0.10,9.8	0
0.10,9.9	0
0.10,10.0	0
0.10,10.1	0
0.10,10.2	0
0.10,10.3	0

0.10,10.4	0
0.10,10.5	0
0.10,10.6	0
0.10,10.7	0
0.10,10.8	0
0.10,10.9	0
0.10,11.0	0
0.10,11.1	0
0.10,11.2	0
0.10,11.3	0
0.10,11.4	0
0.10,11.5	0
0.10,11.6	0
0.10,11.7	0
0.10,11.8	0
0.10,11.9	0
0.10,12.0	0
0.10,12.1	0
0.10,12.2	0
0.10,12.3	0
0.10,12.4	0
0.10,12.5	0
0.10,12.6	0
0.10,12.7	0
0.10,12.8	0
0.10,12.9	0
0.10,13.0	0
0.10,13.1	0
0.10,13.2	0
0.10,13.3	0
0.10,13.4	0
0.10,13.5	0
0.10,13.6	0
0.10,13.7	0
0.10,13.8	0
0.10,13.9	0
0.10,14.0	0
0.10,14.1	0
0.10,14.2	0
0.10,14.3	0
0.10,14.4	0
0.10,14.5	0

0.10,14.6	0
0.10,14.7	0
0.10,14.8	0
0.10,14.9	0
0.10,15.0	0
0.10,15.1	0
0.10,15.2	0
0.10,15.3	0
0.10,15.4	0
0.10,15.5	0
0.10,15.6	0
0.10,15.7	0
0.10,15.8	0
0.10,15.9	0
0.10,16.0	0
0.10,16.1	0
0.10,16.2	0
0.10,16.3	0
0.10,16.4	0
0.10,16.5	0
0.10,16.6	0
0.10,16.7	0
0.10,16.8	0
0.10,16.9	0
0.10,17.0	0
0.10,17.1	0
0.10,17.2	0
0.10,17.3	0
0.10,17.4	0
0.10,17.5	0
0.10,17.6	0
0.10,17.7	0
0.10,17.8	0
0.10,17.9	0
0.10,18.0	0
0.10,18.1	0
0.10,18.2	0
0.10,18.3	0
0.10,18.4	0
0.10,18.5	0
0.10,18.6	0
0.10,18.7	0

0.10,18.8	0
0.10,18.9	0
0.10,19.0	0
0.10,19.1	0
0.10,19.2	0
0.10,19.3	0
0.10,19.4	0
0.10,19.5	0
0.10,19.6	0
0.10,19.7	0
0.10,19.8	0
0.10,19.9	0
0.10,20.0	0
0.10,20.1	0
0.10,20.2	0
0.10,20.3	0
0.10,20.4	0
0.10,20.5	0
0.10,20.6	0
0.10,20.7	0
0.10,20.8	0
0.10,20.9	0
0.10,21.0	0
0.10,21.1	0
0.10,21.2	0
0.10,21.3	0
0.10,21.4	0
0.10,21.5	0
0.10,21.6	0
0.10,21.7	0
0.10,21.8	0
0.10,21.9	0
0.10,22.0	0
0.10,22.1	0
0.10,22.2	0
0.10,22.3	0
0.10,22.4	0
0.10,22.5	0
0.10,22.6	0
0.10,22.7	0
0.10,22.8	0
0.10,22.9	0

0.10,23.0	0
0.10,23.1	0
0.10,23.2	0
0.10,23.3	0
0.10,23.4	0
0.10,23.5	0
0.10,23.6	0
0.10,23.7	0
0.10,23.8	0
0.10,23.9	0
0.10,24.0	0
0.10,24.1	0
0.10,24.2	0
0.10,24.3	0
0.10,24.4	0
0.10,24.5	0
0.10,24.6	0
0.10,24.7	0
0.10,24.8	0
0.10,24.9	0
0.10,25.0	0
0.10,25.1	0
0.10,25.2	0
0.10,25.3	0
0.10,25.4	0
0.10,25.5	0
0.10,25.6	0
0.10,25.7	0
0.10,25.8	0
0.10,25.9	0
0.10,26.0	0
0.10,26.1	0
0.10,26.2	0
0.10,26.3	0
0.10,26.4	0
0.10,26.5	0
0.10,26.6	0
0.10,26.7	0
0.10,26.8	0
0.10,26.9	0
0.10,27.0	0
0.10,27.1	0

0.10,27.2	0
0.10,27.3	0
0.10,27.4	0
0.10,27.5	0
0.10,27.6	0
0.10,27.7	0
0.10,27.8	0
0.10,27.9	0
0.10,28.0	0
0.10,28.1	0
0.10,28.2	0
0.10,28.3	0
0.10,28.4	0
0.10,28.5	0
0.10,28.6	0
0.10,28.7	0
0.10,28.8	0
0.10,28.9	0
0.10,29.0	0
0.10,29.1	0
0.10,29.2	0
0.10,29.3	0
0.10,29.4	0
0.10,29.5	0
0.10,29.6	0
0.10,29.7	0
0.10,29.8	0
0.10,29.9	0
0.10,30.0	0
0.10,30.1	0
0.10,30.2	0
0.10,30.3	0
0.10,30.4	0
0.10,30.5	0
0.10,30.6	0
0.10,30.7	0
0.10,30.8	0
0.10,30.9	0
0.10,31.0	0
0.10,31.1	0
0.10,31.2	0
0.10,31.3	0

0.10,31.4	0
0.10,31.5	0
0.10,31.6	0
0.10,31.7	0
0.10,31.8	0
0.10,31.9	0
0.10,32.0	0
0.10,32.1	0
0.10,32.2	0
0.10,32.3	0
0.10,32.4	0
0.10,32.5	0
0.10,32.6	0
0.10,32.7	0
0.10,32.8	0
0.10,32.9	0
0.10,33.0	0
0.10,33.1	0
0.10,33.2	0
0.10,33.3	0
0.10,33.4	0
0.10,33.5	0
0.10,33.6	0
0.10,33.7	0
0.10,33.8	0
0.10,33.9	0
0.10,34.0	0
0.10,34.1	0
0.10,34.2	0
0.10,34.3	0
0.10,34.4	0
0.10,34.5	0
0.10,34.6	0
0.10,34.7	0
0.10,34.8	0
0.10,34.9	0
0.10,35.0	0
0.10,35.1	0
0.10,35.2	0
0.10,35.3	0
0.10,35.4	0
0.10,35.5	0

0.10,35.6	0
0.10,35.7	0
0.10,35.8	0
0.10,35.9	0
0.10,36.0	0
0.10,36.1	0
0.10,36.2	0
0.10,36.3	0
0.10,36.4	0
0.10,36.5	0
0.10,36.6	0
0.10,36.7	0
0.10,36.8	0
0.10,36.9	0
0.10,37.0	0
0.10,37.1	0
0.10,37.2	0
0.10,37.3	0
0.10,37.4	0
0.10,37.5	0
0.10,37.6	0
0.10,37.7	0
0.10,37.8	0
0.10,37.9	0
0.10,38.0	0
0.10,38.1	0
0.10,38.2	0
0.10,38.3	0
0.10,38.4	0
0.10,38.5	0
0.10,38.6	0
0.10,38.7	0
0.10,38.8	0
0.10,38.9	0
0.10,39.0	0
0.10,39.1	0
0.10,39.2	0
0.10,39.3	0
0.10,39.4	0
0.10,39.5	0
0.10,39.6	0
0.10,39.7	0

0.10,39.8	0
0.10,39.9	0
0.10,40.0	0
0.10,40.1	0
0.10,40.2	0
0.10,40.3	0
0.10,40.4	0
0.10,40.5	0
0.10,40.6	0
0.10,40.7	0
0.10,40.8	0
0.10,40.9	0
0.10,41.0	0
0.10,41.1	0
0.10,41.2	0
0.10,41.3	0
0.10,41.4	0
0.10,41.5	0
0.10,41.6	0
0.10,41.7	0
0.10,41.8	0
0.10,41.9	0
0.10,42.0	0
0.10,42.1	0
0.10,42.2	0
0.10,42.3	0
0.10,42.4	0
0.10,42.5	0
0.10,42.6	0
0.10,42.7	0
0.10,42.8	0
0.10,42.9	0
0.10,43.0	0
0.10,43.1	0
0.10,43.2	0
0.10,43.3	0
0.10,43.4	0
0.10,43.5	0
0.10,43.6	0
0.10,43.7	0
0.10,43.8	0
0.10,43.9	0

0.10,44.0	0
0.10,44.1	0
0.10,44.2	0
0.10,44.3	0
0.10,44.4	0
0.10,44.5	0
0.10,44.6	0
0.10,44.7	0
0.10,44.8	0
0.10,44.9	0
0.10,45.0	0
0.10,45.1	0
0.10,45.2	0
0.10,45.3	0
0.10,45.4	0
0.10,45.5	0
0.10,45.6	0
0.10,45.7	0
0.10,45.8	0
0.10,45.9	0
0.10,46.0	0
0.10,46.1	0
0.10,46.2	0
0.10,46.3	0
0.10,46.4	0
0.10,46.5	0
0.10,46.6	0
0.10,46.7	0
0.10,46.8	0
0.10,46.9	0
0.10,47.0	0
0.10,47.1	0
0.10,47.2	0
0.10,47.3	0
0.10,47.4	0
0.10,47.5	0
0.10,47.6	0
0.10,47.7	0
0.10,47.8	0
0.10,47.9	0
0.10,48.0	0
0.10,48.1	0

0.10,48.2	0
0.10,48.3	0
0.10,48.4	0
0.10,48.5	0
0.10,48.6	0
0.10,48.7	0
0.10,48.8	0
0.10,48.9	0
0.10,49.0	0
0.10,49.1	0
0.10,49.2	0
0.10,49.3	0
0.10,49.4	0
0.10,49.5	0
0.10,49.6	0
0.10,49.7	0
0.10,49.8	0
0.10,49.9	0
0.10,50.0	0
0.10,50.1	0
0.10,50.2	0
0.10,50.3	0
0.10,50.4	0
0.10,50.5	0
0.10,50.6	0
0.10,50.7	0
0.10,50.8	0
0.10,50.9	0
0.10,51.0	0
0.10,51.1	0
0.10,51.2	0
0.10,51.3	0
0.10,51.4	0
0.10,51.5	0
0.10,51.6	0
0.10,51.7	0
0.10,51.8	0
0.10,51.9	0
0.10,52.0	0
0.10,52.1	0
0.10,52.2	0
0.10,52.3	0

0.10,52.4	0
0.10,52.5	0
0.10,52.6	0
0.10,52.7	0
0.10,52.8	0
0.10,52.9	0
0.10,53.0	0
0.10,53.1	0
0.10,53.2	0
0.10,53.3	0
0.10,53.4	0
0.10,53.5	0
0.10,53.6	0
0.10,53.7	0
0.10,53.8	0
0.10,53.9	0
0.10,54.0	0
0.10,54.1	0
0.10,54.2	0
0.10,54.3	0
0.10,54.4	0
0.10,54.5	0
0.10,54.6	0
0.10,54.7	0
0.10,54.8	0
0.10,54.9	0
0.10,55.0	0
0.10,55.1	0
0.10,55.2	0
0.10,55.3	0
0.10,55.4	0
0.10,55.5	0
0.10,55.6	0
0.10,55.7	0
0.10,55.8	0
0.10,55.9	0
0.10,56.0	0
0.10,56.1	0
0.10,56.2	0
0.10,56.3	0
0.10,56.4	0
0.10,56.5	0

0.10,56.6	0
0.10,56.7	0
0.10,56.8	0
0.10,56.9	0
0.10,57.0	0
0.10,57.1	0
0.10,57.2	0
0.10,57.3	0
0.10,57.4	0
0.10,57.5	0
0.10,57.6	0
0.10,57.7	0
0.10,57.8	0
0.10,57.9	0
0.10,58.0	0
0.10,58.1	0
0.10,58.2	0
0.10,58.3	0
0.10,58.4	0
0.10,58.5	0
0.10,58.6	0
0.10,58.7	0
0.10,58.8	0
0.10,58.9	0
0.10,59.0	0
0.10,59.1	0
0.10,59.2	0
0.10,59.3	0
0.10,59.4	0
0.10,59.5	0
0.10,59.6	0
0.10,59.7	0
0.10,59.8	0
0.10,59.9	0
0.10,60.0	0
0.10,60.1	0
0.10,60.2	0
0.10,60.3	0
0.10,60.4	0
0.10,60.5	0
0.10,60.6	0
0.10,60.7	0

0.10,60.8	0
0.10,60.9	0
0.10,61.0	0
0.10,61.1	0
0.10,61.2	0
0.10,61.3	0
0.10,61.4	0
0.10,61.5	0
0.10,61.6	0
0.10,61.7	0
0.10,61.8	0
0.10,61.9	0
0.10,62.0	0
0.10,62.1	0
0.10,62.2	0
0.10,62.3	0
0.10,62.4	0
0.10,62.5	0
0.10,62.6	0
0.10,62.7	0
0.10,62.8	0
0.10,62.9	0
0.10,63.0	0
0.10,63.1	0
0.10,63.2	0
0.10,63.3	0
0.10,63.4	0
0.10,63.5	0
0.10,63.6	0
0.10,63.7	0
0.10,63.8	0
0.10,63.9	0
0.10,64.0	0
0.10,64.1	0
0.10,64.2	0
0.10,64.3	0
0.10,64.4	0
0.10,64.5	0
0.10,64.6	0
0.10,64.7	0
0.10,64.8	0
0.10,64.9	0

0.10,65.0	0
0.10,65.1	0
0.10,65.2	0
0.10,65.3	0
0.10,65.4	0
0.10,65.5	0
0.10,65.6	0
0.10,65.7	0
0.10,65.8	0
0.10,65.9	0
0.10,66.0	0
0.10,66.1	0
0.10,66.2	0
0.10,66.3	0
0.10,66.4	0
0.10,66.5	0
0.10,66.6	0
0.10,66.7	0
0.10,66.8	0
0.10,66.9	0
0.10,67.0	0
0.10,67.1	0
0.10,67.2	0
0.10,67.3	0
0.10,67.4	0
0.10,67.5	0
0.10,67.6	0
0.10,67.7	0
0.10,67.8	0
0.10,67.9	0
0.10,68.0	0
0.10,68.1	0
0.10,68.2	0
0.10,68.3	0
0.10,68.4	0
0.10,68.5	0
0.10,68.6	0
0.10,68.7	0
0.10,68.8	0
0.10,68.9	0
0.10,69.0	0
0.10,69.1	0

0.10,69.2	0
0.10,69.3	0
0.10,69.4	0
0.10,69.5	0
0.10,69.6	0
0.10,69.7	0
0.10,69.8	0
0.10,69.9	0
0.10,70.0	0
0.10,70.1	0
0.10,70.2	0
0.10,70.3	0
0.10,70.4	0
0.10,70.5	0
0.10,70.6	0
0.10,70.7	0
0.10,70.8	0
0.10,70.9	0
0.10,71.0	0
0.10,71.1	0
0.10,71.2	0
0.10,71.3	0
0.10,71.4	0
0.10,71.5	0
0.10,71.6	0
0.10,71.7	0
0.10,71.8	0
0.10,71.9	0
0.10,72.0	0
0.10,72.1	0
0.10,72.2	0
0.10,72.3	0
0.10,72.4	0
0.10,72.5	0
0.10,72.6	0
0.10,72.7	0
0.10,72.8	0
0.10,72.9	0
0.10,73.0	0
0.10,73.1	0
0.10,73.2	0
0.10,73.3	0

0.10,73.4	0
0.10,73.5	0
0.10,73.6	0
0.10,73.7	0
0.10,73.8	0
0.10,73.9	0
0.10,74.0	0
0.10,74.1	0
0.10,74.2	0
0.10,74.3	0
0.10,74.4	0
0.10,74.5	0
0.10,74.6	0
0.10,74.7	0
0.10,74.8	0
0.10,74.9	0
0.10,75.0	0
0.10,75.1	0
0.10,75.2	0
0.10,75.3	0
0.10,75.4	0
0.10,75.5	0
0.10,75.6	0
0.10,75.7	0
0.10,75.8	0
0.10,75.9	0
0.10,76.0	0
0.10,76.1	0
0.10,76.2	0
0.10,76.3	0
0.10,76.4	0
0.10,76.5	0
0.10,76.6	0
0.10,76.7	0
0.10,76.8	0
0.10,76.9	0
0.10,77.0	0
0.10,77.1	0
0.10,77.2	0
0.10,77.3	0
0.10,77.4	0
0.10,77.5	0

0.10,77.6	0
0.10,77.7	0
0.10,77.8	0
0.10,77.9	0
0.10,78.0	0
0.10,78.1	0
0.10,78.2	0
0.10,78.3	0
0.10,78.4	0
0.10,78.5	0
0.10,78.6	0
0.10,78.7	0
0.10,78.8	0
0.10,78.9	0
0.10,79.0	0
0.10,79.1	0
0.10,79.2	0
0.10,79.3	0
0.10,79.4	0
0.10,79.5	0
0.10,79.6	0
0.10,79.7	0
0.10,79.8	0
0.10,79.9	0
0.10,80.0	0
0.10,80.1	0
0.10,80.2	0
0.10,80.3	0
0.10,80.4	0
0.10,80.5	0
0.10,80.6	0
0.10,80.7	0
0.10,80.8	0
0.10,80.9	0
0.10,81.0	0
0.10,81.1	0
0.10,81.2	0
0.10,81.3	0
0.10,81.4	0
0.10,81.5	0
0.10,81.6	0
0.10,81.7	0

0.10,81.8	0
0.10,81.9	0
0.10,82.0	0
0.10,82.1	0
0.10,82.2	0
0.10,82.3	0
0.10,82.4	0
0.10,82.5	0
0.10,82.6	0
0.10,82.7	0
0.10,82.8	0
0.10,82.9	0
0.10,83.0	0
0.10,83.1	0
0.10,83.2	0
0.10,83.3	0
0.10,83.4	0
0.10,83.5	0
0.10,83.6	0
0.10,83.7	0
0.10,83.8	0
0.10,83.9	0
0.10,84.0	0
0.10,84.1	0
0.10,84.2	0
0.10,84.3	0
0.10,84.4	0
0.10,84.5	0
0.10,84.6	0
0.10,84.7	0
0.10,84.8	0
0.10,84.9	0
0.10,85.0	0
0.10,85.1	0
0.10,85.2	0
0.10,85.3	0
0.10,85.4	0
0.10,85.5	0
0.10,85.6	0
0.10,85.7	0
0.10,85.8	0
0.10,85.9	0

0.10,86.0	0
0.10,86.1	0
0.10,86.2	0
0.10,86.3	0
0.10,86.4	0
0.10,86.5	0
0.10,86.6	0
0.10,86.7	0
0.10,86.8	0
0.10,86.9	0
0.10,87.0	0
0.10,87.1	0
0.10,87.2	0
0.10,87.3	0
0.10,87.4	0
0.10,87.5	0
0.10,87.6	0
0.10,87.7	0
0.10,87.8	0
0.10,87.9	0
0.10,88.0	0
0.10,88.1	0
0.10,88.2	0
0.10,88.3	0
0.10,88.4	0
0.10,88.5	0
0.10,88.6	0
0.10,88.7	0
0.10,88.8	0
0.10,88.9	0
0.10,89.0	0
0.10,89.1	0
0.10,89.2	0
0.10,89.3	0
0.10,89.4	0
0.10,89.5	0
0.10,89.6	0
0.10,89.7	0
0.10,89.8	0
0.10,89.9	0
0.10,90.0	0
0.10,90.1	0

0.10,90.2	0
0.10,90.3	0
0.10,90.4	0
0.10,90.5	0
0.10,90.6	0
0.10,90.7	0
0.10,90.8	0
0.10,90.9	0
0.10,91.0	0
0.10,91.1	0
0.10,91.2	0
0.10,91.3	0
0.10,91.4	0
0.10,91.5	0
0.10,91.6	0
0.10,91.7	0
0.10,91.8	0
0.10,91.9	0
0.10,92.0	0
0.10,92.1	0
0.10,92.2	0
0.10,92.3	0
0.10,92.4	0
0.10,92.5	0
0.10,92.6	0
0.10,92.7	0
0.10,92.8	0
0.10,92.9	0
0.10,93.0	0
0.10,93.1	0
0.10,93.2	0
0.10,93.3	0
0.10,93.4	0
0.10,93.5	0
0.10,93.6	0
0.10,93.7	0
0.10,93.8	0
0.10,93.9	0
0.10,94.0	0
0.10,94.1	0
0.10,94.2	0
0.10,94.3	0

0.10,94.4	0
0.10,94.5	0
0.10,94.6	0
0.10,94.7	0
0.10,94.8	0
0.10,94.9	0
0.10,95.0	0
0.10,95.1	0
0.10,95.2	0
0.10,95.3	0
0.10,95.4	0
0.10,95.5	0
0.10,95.6	0
0.10,95.7	0
0.10,95.8	0
0.10,95.9	0
0.10,96.0	0
0.10,96.1	0
0.10,96.2	0
0.10,96.3	0
0.10,96.4	0
0.10,96.5	0
0.10,96.6	0
0.10,96.7	0
0.10,96.8	0
0.10,96.9	0
0.10,97.0	0
0.10,97.1	0
0.10,97.2	0
0.10,97.3	0
0.10,97.4	0
0.10,97.5	0
0.10,97.6	0
0.10,97.7	0
0.10,97.8	0
0.10,97.9	0
0.10,98.0	0
0.10,98.1	0
0.10,98.2	0
0.10,98.3	0
0.10,98.4	0
0.10,98.5	0

0.10,98.6	0
0.10,98.7	0
0.10,98.8	0
0.10,98.9	0
0.10,99.0	0
0.10,99.1	0
0.10,99.2	0
0.10,99.3	0
0.10,99.4	0
0.10,99.5	0
0.10,99.6	0
0.10,99.7	0
0.10,99.8	0
0.10,99.9	0
0.10,100.0	0
0.11,0.0	0
0.11,0.1	0
0.11,0.2	0
0.11,0.3	0
0.11,0.4	0
0.11,0.5	0
0.11,0.6	0
0.11,0.7	0
0.11,0.8	0
0.11,0.9	0
0.11,1.0	0
0.11,1.1	0
0.11,1.2	0
0.11,1.3	0
0.11,1.4	0
0.11,1.5	0
0.11,1.6	0
0.11,1.7	0
0.11,1.8	0
0.11,1.9	0
0.11,2.0	0
0.11,2.1	0
0.11,2.2	0
0.11,2.3	0
0.11,2.4	0
0.11,2.5	0
0.11,2.6	0

0.11,2.7	0
0.11,2.8	0
0.11,2.9	0
0.11,3.0	0
0.11,3.1	0
0.11,3.2	0
0.11,3.3	0
0.11,3.4	0
0.11,3.5	0
0.11,3.6	0
0.11,3.7	0
0.11,3.8	0
0.11,3.9	0
0.11,4.0	0
0.11,4.1	0
0.11,4.2	0
0.11,4.3	0
0.11,4.4	0
0.11,4.5	0
0.11,4.6	0
0.11,4.7	0
0.11,4.8	0
0.11,4.9	0
0.11,5.0	0
0.11,5.1	0
0.11,5.2	0
0.11,5.3	0
0.11,5.4	0
0.11,5.5	0
0.11,5.6	0
0.11,5.7	0
0.11,5.8	0
0.11,5.9	0
0.11,6.0	0
0.11,6.1	0
0.11,6.2	0
0.11,6.3	0
0.11,6.4	0
0.11,6.5	0
0.11,6.6	0
0.11,6.7	0
0.11,6.8	0

0.11,6.9	0
0.11,7.0	0
0.11,7.1	0
0.11,7.2	0
0.11,7.3	0
0.11,7.4	0
0.11,7.5	0
0.11,7.6	0
0.11,7.7	0
0.11,7.8	0
0.11,7.9	0
0.11,8.0	0
0.11,8.1	0
0.11,8.2	0
0.11,8.3	0
0.11,8.4	0
0.11,8.5	0
0.11,8.6	0
0.11,8.7	0
0.11,8.8	0
0.11,8.9	0
0.11,9.0	0
0.11,9.1	0
0.11,9.2	0
0.11,9.3	0
0.11,9.4	0
0.11,9.5	0
0.11,9.6	0
0.11,9.7	0
0.11,9.8	0
0.11,9.9	0
0.11,10.0	0
0.11,10.1	0
0.11,10.2	0
0.11,10.3	0
0.11,10.4	0
0.11,10.5	0
0.11,10.6	0
0.11,10.7	0
0.11,10.8	0
0.11,10.9	0
0.11,11.0	0

0.11,11.1	0
0.11,11.2	0
0.11,11.3	0
0.11,11.4	0
0.11,11.5	0
0.11,11.6	0
0.11,11.7	0
0.11,11.8	0
0.11,11.9	0
0.11,12.0	0
0.11,12.1	0
0.11,12.2	0
0.11,12.3	0
0.11,12.4	0
0.11,12.5	0
0.11,12.6	0
0.11,12.7	0
0.11,12.8	0
0.11,12.9	0
0.11,13.0	0
0.11,13.1	0
0.11,13.2	0
0.11,13.3	0
0.11,13.4	0
0.11,13.5	0
0.11,13.6	0
0.11,13.7	0
0.11,13.8	0
0.11,13.9	0
0.11,14.0	0
0.11,14.1	0
0.11,14.2	0
0.11,14.3	0
0.11,14.4	0
0.11,14.5	0
0.11,14.6	0
0.11,14.7	0
0.11,14.8	0
0.11,14.9	0
0.11,15.0	0
0.11,15.1	0
0.11,15.2	0

0.11,15.3	0
0.11,15.4	0
0.11,15.5	0
0.11,15.6	0
0.11,15.7	0
0.11,15.8	0
0.11,15.9	0
0.11,16.0	0
0.11,16.1	0
0.11,16.2	0
0.11,16.3	0
0.11,16.4	0
0.11,16.5	0
0.11,16.6	0
0.11,16.7	0
0.11,16.8	0
0.11,16.9	0
0.11,17.0	0
0.11,17.1	0
0.11,17.2	0
0.11,17.3	0
0.11,17.4	0
0.11,17.5	0
0.11,17.6	0
0.11,17.7	0
0.11,17.8	0
0.11,17.9	0
0.11,18.0	0
0.11,18.1	0
0.11,18.2	0
0.11,18.3	0
0.11,18.4	0
0.11,18.5	0
0.11,18.6	0
0.11,18.7	0
0.11,18.8	0
0.11,18.9	0
0.11,19.0	0
0.11,19.1	0
0.11,19.2	0
0.11,19.3	0
0.11,19.4	0

0.11,19.5	0
0.11,19.6	0
0.11,19.7	0
0.11,19.8	0
0.11,19.9	0
0.11,20.0	0
0.11,20.1	0
0.11,20.2	0
0.11,20.3	0
0.11,20.4	0
0.11,20.5	0
0.11,20.6	0
0.11,20.7	0
0.11,20.8	0
0.11,20.9	0
0.11,21.0	0
0.11,21.1	0
0.11,21.2	0
0.11,21.3	0
0.11,21.4	0
0.11,21.5	0
0.11,21.6	0
0.11,21.7	0
0.11,21.8	0
0.11,21.9	0
0.11,22.0	0
0.11,22.1	0
0.11,22.2	0
0.11,22.3	0
0.11,22.4	0
0.11,22.5	0
0.11,22.6	0
0.11,22.7	0
0.11,22.8	0
0.11,22.9	0
0.11,23.0	0
0.11,23.1	0
0.11,23.2	0
0.11,23.3	0
0.11,23.4	0
0.11,23.5	0
0.11,23.6	0

0.11,23.7	0
0.11,23.8	0
0.11,23.9	0
0.11,24.0	0
0.11,24.1	0
0.11,24.2	0
0.11,24.3	0
0.11,24.4	0
0.11,24.5	0
0.11,24.6	0
0.11,24.7	0
0.11,24.8	0
0.11,24.9	0
0.11,25.0	0
0.11,25.1	0
0.11,25.2	0
0.11,25.3	0
0.11,25.4	0
0.11,25.5	0
0.11,25.6	0
0.11,25.7	0
0.11,25.8	0
0.11,25.9	0
0.11,26.0	0
0.11,26.1	0
0.11,26.2	0
0.11,26.3	0
0.11,26.4	0
0.11,26.5	0
0.11,26.6	0
0.11,26.7	0
0.11,26.8	0
0.11,26.9	0
0.11,27.0	0
0.11,27.1	0
0.11,27.2	0
0.11,27.3	0
0.11,27.4	0
0.11,27.5	0
0.11,27.6	0
0.11,27.7	0
0.11,27.8	0

0.11,27.9	0
0.11,28.0	0
0.11,28.1	0
0.11,28.2	0
0.11,28.3	0
0.11,28.4	0
0.11,28.5	0
0.11,28.6	0
0.11,28.7	0
0.11,28.8	0
0.11,28.9	0
0.11,29.0	0
0.11,29.1	0
0.11,29.2	0
0.11,29.3	0
0.11,29.4	0
0.11,29.5	0
0.11,29.6	0
0.11,29.7	0
0.11,29.8	0
0.11,29.9	0
0.11,30.0	0
0.11,30.1	0
0.11,30.2	0
0.11,30.3	0
0.11,30.4	0
0.11,30.5	0
0.11,30.6	0
0.11,30.7	0
0.11,30.8	0
0.11,30.9	0
0.11,31.0	0
0.11,31.1	0
0.11,31.2	0
0.11,31.3	0
0.11,31.4	0
0.11,31.5	0
0.11,31.6	0
0.11,31.7	0
0.11,31.8	0
0.11,31.9	0
0.11,32.0	0

0.11,32.1	0
0.11,32.2	0
0.11,32.3	0
0.11,32.4	0
0.11,32.5	0
0.11,32.6	0
0.11,32.7	0
0.11,32.8	0
0.11,32.9	0
0.11,33.0	0
0.11,33.1	0
0.11,33.2	0
0.11,33.3	0
0.11,33.4	0
0.11,33.5	0
0.11,33.6	0
0.11,33.7	0
0.11,33.8	0
0.11,33.9	0
0.11,34.0	0
0.11,34.1	0
0.11,34.2	0
0.11,34.3	0
0.11,34.4	0
0.11,34.5	0
0.11,34.6	0
0.11,34.7	0
0.11,34.8	0
0.11,34.9	0
0.11,35.0	0
0.11,35.1	0
0.11,35.2	0
0.11,35.3	0
0.11,35.4	0
0.11,35.5	0
0.11,35.6	0
0.11,35.7	0
0.11,35.8	0
0.11,35.9	0
0.11,36.0	0
0.11,36.1	0
0.11,36.2	0

0.11,36.3	0
0.11,36.4	0
0.11,36.5	0
0.11,36.6	0
0.11,36.7	0
0.11,36.8	0
0.11,36.9	0
0.11,37.0	0
0.11,37.1	0
0.11,37.2	0
0.11,37.3	0
0.11,37.4	0
0.11,37.5	0
0.11,37.6	0
0.11,37.7	0
0.11,37.8	0
0.11,37.9	0
0.11,38.0	0
0.11,38.1	0
0.11,38.2	0
0.11,38.3	0
0.11,38.4	0
0.11,38.5	0
0.11,38.6	0
0.11,38.7	0
0.11,38.8	0
0.11,38.9	0
0.11,39.0	0
0.11,39.1	0
0.11,39.2	0
0.11,39.3	0
0.11,39.4	0
0.11,39.5	0
0.11,39.6	0
0.11,39.7	0
0.11,39.8	0
0.11,39.9	0
0.11,40.0	0
0.11,40.1	0
0.11,40.2	0
0.11,40.3	0
0.11,40.4	0

0.11,40.5	0
0.11,40.6	0
0.11,40.7	0
0.11,40.8	0
0.11,40.9	0
0.11,41.0	0
0.11,41.1	0
0.11,41.2	0
0.11,41.3	0
0.11,41.4	0
0.11,41.5	0
0.11,41.6	0
0.11,41.7	0
0.11,41.8	0
0.11,41.9	0
0.11,42.0	0
0.11,42.1	0
0.11,42.2	0
0.11,42.3	0
0.11,42.4	0
0.11,42.5	0
0.11,42.6	0
0.11,42.7	0
0.11,42.8	0
0.11,42.9	0
0.11,43.0	0
0.11,43.1	0
0.11,43.2	0
0.11,43.3	0
0.11,43.4	0
0.11,43.5	0
0.11,43.6	0
0.11,43.7	0
0.11,43.8	0
0.11,43.9	0
0.11,44.0	0
0.11,44.1	0
0.11,44.2	0
0.11,44.3	0
0.11,44.4	0
0.11,44.5	0
0.11,44.6	0

0.11,44.7	0
0.11,44.8	0
0.11,44.9	0
0.11,45.0	0
0.11,45.1	0
0.11,45.2	0
0.11,45.3	0
0.11,45.4	0
0.11,45.5	0
0.11,45.6	0
0.11,45.7	0
0.11,45.8	0
0.11,45.9	0
0.11,46.0	0
0.11,46.1	0
0.11,46.2	0
0.11,46.3	0
0.11,46.4	0
0.11,46.5	0
0.11,46.6	0
0.11,46.7	0
0.11,46.8	0
0.11,46.9	0
0.11,47.0	0
0.11,47.1	0
0.11,47.2	0
0.11,47.3	0
0.11,47.4	0
0.11,47.5	0
0.11,47.6	0
0.11,47.7	0
0.11,47.8	0
0.11,47.9	0
0.11,48.0	0
0.11,48.1	0
0.11,48.2	0
0.11,48.3	0
0.11,48.4	0
0.11,48.5	0
0.11,48.6	0
0.11,48.7	0
0.11,48.8	0

0.11,48.9	0
0.11,49.0	0
0.11,49.1	0
0.11,49.2	0
0.11,49.3	0
0.11,49.4	0
0.11,49.5	0
0.11,49.6	0
0.11,49.7	0
0.11,49.8	0
0.11,49.9	0
0.11,50.0	0
0.11,50.1	0
0.11,50.2	0
0.11,50.3	0
0.11,50.4	0
0.11,50.5	0
0.11,50.6	0
0.11,50.7	0
0.11,50.8	0
0.11,50.9	0
0.11,51.0	0
0.11,51.1	0
0.11,51.2	0
0.11,51.3	0
0.11,51.4	0
0.11,51.5	0
0.11,51.6	0
0.11,51.7	0
0.11,51.8	0
0.11,51.9	0
0.11,52.0	0
0.11,52.1	0
0.11,52.2	0
0.11,52.3	0
0.11,52.4	0
0.11,52.5	0
0.11,52.6	0
0.11,52.7	0
0.11,52.8	0
0.11,52.9	0
0.11,53.0	0

0.11,53.1	0
0.11,53.2	0
0.11,53.3	0
0.11,53.4	0
0.11,53.5	0
0.11,53.6	0
0.11,53.7	0
0.11,53.8	0
0.11,53.9	0
0.11,54.0	0
0.11,54.1	0
0.11,54.2	0
0.11,54.3	0
0.11,54.4	0
0.11,54.5	0
0.11,54.6	0
0.11,54.7	0
0.11,54.8	0
0.11,54.9	0
0.11,55.0	0
0.11,55.1	0
0.11,55.2	0
0.11,55.3	0
0.11,55.4	0
0.11,55.5	0
0.11,55.6	0
0.11,55.7	0
0.11,55.8	0
0.11,55.9	0
0.11,56.0	0
0.11,56.1	0
0.11,56.2	0
0.11,56.3	0
0.11,56.4	0
0.11,56.5	0
0.11,56.6	0
0.11,56.7	0
0.11,56.8	0
0.11,56.9	0
0.11,57.0	0
0.11,57.1	0
0.11,57.2	0

0.11,57.3	0
0.11,57.4	0
0.11,57.5	0
0.11,57.6	0
0.11,57.7	0
0.11,57.8	0
0.11,57.9	0
0.11,58.0	0
0.11,58.1	0
0.11,58.2	0
0.11,58.3	0
0.11,58.4	0
0.11,58.5	0
0.11,58.6	0
0.11,58.7	0
0.11,58.8	0
0.11,58.9	0
0.11,59.0	0
0.11,59.1	0
0.11,59.2	0
0.11,59.3	0
0.11,59.4	0
0.11,59.5	0
0.11,59.6	0
0.11,59.7	0
0.11,59.8	0
0.11,59.9	0
0.11,60.0	0
0.11,60.1	0
0.11,60.2	0
0.11,60.3	0
0.11,60.4	0
0.11,60.5	0
0.11,60.6	0
0.11,60.7	0
0.11,60.8	0
0.11,60.9	0
0.11,61.0	0
0.11,61.1	0
0.11,61.2	0
0.11,61.3	0
0.11,61.4	0

0.11,61.5	0
0.11,61.6	0
0.11,61.7	0
0.11,61.8	0
0.11,61.9	0
0.11,62.0	0
0.11,62.1	0
0.11,62.2	0
0.11,62.3	0
0.11,62.4	0
0.11,62.5	0
0.11,62.6	0
0.11,62.7	0
0.11,62.8	0
0.11,62.9	0
0.11,63.0	0
0.11,63.1	0
0.11,63.2	0
0.11,63.3	0
0.11,63.4	0
0.11,63.5	0
0.11,63.6	0
0.11,63.7	0
0.11,63.8	0
0.11,63.9	0
0.11,64.0	0
0.11,64.1	0
0.11,64.2	0
0.11,64.3	0
0.11,64.4	0
0.11,64.5	0
0.11,64.6	0
0.11,64.7	0
0.11,64.8	0
0.11,64.9	0
0.11,65.0	0
0.11,65.1	0
0.11,65.2	0
0.11,65.3	0
0.11,65.4	0
0.11,65.5	0
0.11,65.6	0

0.11,65.7	0
0.11,65.8	0
0.11,65.9	0
0.11,66.0	0
0.11,66.1	0
0.11,66.2	0
0.11,66.3	0
0.11,66.4	0
0.11,66.5	0
0.11,66.6	0
0.11,66.7	0
0.11,66.8	0
0.11,66.9	0
0.11,67.0	0
0.11,67.1	0
0.11,67.2	0
0.11,67.3	0
0.11,67.4	0
0.11,67.5	0
0.11,67.6	0
0.11,67.7	0
0.11,67.8	0
0.11,67.9	0
0.11,68.0	0
0.11,68.1	0
0.11,68.2	0
0.11,68.3	0
0.11,68.4	0
0.11,68.5	0
0.11,68.6	0
0.11,68.7	0
0.11,68.8	0
0.11,68.9	0
0.11,69.0	0
0.11,69.1	0
0.11,69.2	0
0.11,69.3	0
0.11,69.4	0
0.11,69.5	0
0.11,69.6	0
0.11,69.7	0
0.11,69.8	0

0.11,69.9	0
0.11,70.0	0
0.11,70.1	0
0.11,70.2	0
0.11,70.3	0
0.11,70.4	0
0.11,70.5	0
0.11,70.6	0
0.11,70.7	0
0.11,70.8	0
0.11,70.9	0
0.11,71.0	0
0.11,71.1	0
0.11,71.2	0
0.11,71.3	0
0.11,71.4	0
0.11,71.5	0
0.11,71.6	0
0.11,71.7	0
0.11,71.8	0
0.11,71.9	0
0.11,72.0	0
0.11,72.1	0
0.11,72.2	0
0.11,72.3	0
0.11,72.4	0
0.11,72.5	0
0.11,72.6	0
0.11,72.7	0
0.11,72.8	0
0.11,72.9	0
0.11,73.0	0
0.11,73.1	0
0.11,73.2	0
0.11,73.3	0
0.11,73.4	0
0.11,73.5	0
0.11,73.6	0
0.11,73.7	0
0.11,73.8	0
0.11,73.9	0
0.11,74.0	0

0.11,74.1	0
0.11,74.2	0
0.11,74.3	0
0.11,74.4	0
0.11,74.5	0
0.11,74.6	0
0.11,74.7	0
0.11,74.8	0
0.11,74.9	0
0.11,75.0	0
0.11,75.1	0
0.11,75.2	0
0.11,75.3	0
0.11,75.4	0
0.11,75.5	0
0.11,75.6	0
0.11,75.7	0
0.11,75.8	0
0.11,75.9	0
0.11,76.0	0
0.11,76.1	0
0.11,76.2	0
0.11,76.3	0
0.11,76.4	0
0.11,76.5	0
0.11,76.6	0
0.11,76.7	0
0.11,76.8	0
0.11,76.9	0
0.11,77.0	0
0.11,77.1	0
0.11,77.2	0
0.11,77.3	0
0.11,77.4	0
0.11,77.5	0
0.11,77.6	0
0.11,77.7	0
0.11,77.8	0
0.11,77.9	0
0.11,78.0	0
0.11,78.1	0
0.11,78.2	0

0.11,78.3	0
0.11,78.4	0
0.11,78.5	0
0.11,78.6	0
0.11,78.7	0
0.11,78.8	0
0.11,78.9	0
0.11,79.0	0
0.11,79.1	0
0.11,79.2	0
0.11,79.3	0
0.11,79.4	0
0.11,79.5	0
0.11,79.6	0
0.11,79.7	0
0.11,79.8	0
0.11,79.9	0
0.11,80.0	0
0.11,80.1	0
0.11,80.2	0
0.11,80.3	0
0.11,80.4	0
0.11,80.5	0
0.11,80.6	0
0.11,80.7	0
0.11,80.8	0
0.11,80.9	0
0.11,81.0	0
0.11,81.1	0
0.11,81.2	0
0.11,81.3	0
0.11,81.4	0
0.11,81.5	0
0.11,81.6	0
0.11,81.7	0
0.11,81.8	0
0.11,81.9	0
0.11,82.0	0
0.11,82.1	0
0.11,82.2	0
0.11,82.3	0
0.11,82.4	0

0.11,82.5	0
0.11,82.6	0
0.11,82.7	0
0.11,82.8	0
0.11,82.9	0
0.11,83.0	0
0.11,83.1	0
0.11,83.2	0
0.11,83.3	0
0.11,83.4	0
0.11,83.5	0
0.11,83.6	0
0.11,83.7	0
0.11,83.8	0
0.11,83.9	0
0.11,84.0	0
0.11,84.1	0
0.11,84.2	0
0.11,84.3	0
0.11,84.4	0
0.11,84.5	0
0.11,84.6	0
0.11,84.7	0
0.11,84.8	0
0.11,84.9	0
0.11,85.0	0
0.11,85.1	0
0.11,85.2	0
0.11,85.3	0
0.11,85.4	0
0.11,85.5	0
0.11,85.6	0
0.11,85.7	0
0.11,85.8	0
0.11,85.9	0
0.11,86.0	0
0.11,86.1	0
0.11,86.2	0
0.11,86.3	0
0.11,86.4	0
0.11,86.5	0
0.11,86.6	0

0.11,86.7	0
0.11,86.8	0
0.11,86.9	0
0.11,87.0	0
0.11,87.1	0
0.11,87.2	0
0.11,87.3	0
0.11,87.4	0
0.11,87.5	0
0.11,87.6	0
0.11,87.7	0
0.11,87.8	0
0.11,87.9	0
0.11,88.0	0
0.11,88.1	0
0.11,88.2	0
0.11,88.3	0
0.11,88.4	0
0.11,88.5	0
0.11,88.6	0
0.11,88.7	0
0.11,88.8	0
0.11,88.9	0
0.11,89.0	0
0.11,89.1	0
0.11,89.2	0
0.11,89.3	0
0.11,89.4	0
0.11,89.5	0
0.11,89.6	0
0.11,89.7	0
0.11,89.8	0
0.11,89.9	0
0.11,90.0	0
0.11,90.1	0
0.11,90.2	0
0.11,90.3	0
0.11,90.4	0
0.11,90.5	0
0.11,90.6	0
0.11,90.7	0
0.11,90.8	0

0.11,90.9	0
0.11,91.0	0
0.11,91.1	0
0.11,91.2	0
0.11,91.3	0
0.11,91.4	0
0.11,91.5	0
0.11,91.6	0
0.11,91.7	0
0.11,91.8	0
0.11,91.9	0
0.11,92.0	0
0.11,92.1	0
0.11,92.2	0
0.11,92.3	0
0.11,92.4	0
0.11,92.5	0
0.11,92.6	0
0.11,92.7	0
0.11,92.8	0
0.11,92.9	0
0.11,93.0	0
0.11,93.1	0
0.11,93.2	0
0.11,93.3	0
0.11,93.4	0
0.11,93.5	0
0.11,93.6	0
0.11,93.7	0
0.11,93.8	0
0.11,93.9	0
0.11,94.0	0
0.11,94.1	0
0.11,94.2	0
0.11,94.3	0
0.11,94.4	0
0.11,94.5	0
0.11,94.6	0
0.11,94.7	0
0.11,94.8	0
0.11,94.9	0
0.11,95.0	0

0.11,95.1	0
0.11,95.2	0
0.11,95.3	0
0.11,95.4	0
0.11,95.5	0
0.11,95.6	0
0.11,95.7	0
0.11,95.8	0
0.11,95.9	0
0.11,96.0	0
0.11,96.1	0
0.11,96.2	0
0.11,96.3	0
0.11,96.4	0
0.11,96.5	0
0.11,96.6	0
0.11,96.7	0
0.11,96.8	0
0.11,96.9	0
0.11,97.0	0
0.11,97.1	0
0.11,97.2	0
0.11,97.3	0
0.11,97.4	0
0.11,97.5	0
0.11,97.6	0
0.11,97.7	0
0.11,97.8	0
0.11,97.9	0
0.11,98.0	0
0.11,98.1	0
0.11,98.2	0
0.11,98.3	0
0.11,98.4	0
0.11,98.5	0
0.11,98.6	0
0.11,98.7	0
0.11,98.8	0
0.11,98.9	0
0.11,99.0	0
0.11,99.1	0
0.11,99.2	0

0.11,99.3	0
0.11,99.4	0
0.11,99.5	0
0.11,99.6	0
0.11,99.7	0
0.11,99.8	0
0.11,99.9	0
0.11,100.0	0
0.12,0.0	0
0.12,0.1	0
0.12,0.2	0
0.12,0.3	0
0.12,0.4	0
0.12,0.5	0
0.12,0.6	0
0.12,0.7	0
0.12,0.8	0
0.12,0.9	0
0.12,1.0	0
0.12,1.1	0
0.12,1.2	0
0.12,1.3	0
0.12,1.4	0
0.12,1.5	0
0.12,1.6	0
0.12,1.7	0
0.12,1.8	0
0.12,1.9	0
0.12,2.0	0
0.12,2.1	0
0.12,2.2	0
0.12,2.3	0
0.12,2.4	0
0.12,2.5	0
0.12,2.6	0
0.12,2.7	0
0.12,2.8	0
0.12,2.9	0
0.12,3.0	0
0.12,3.1	0
0.12,3.2	0
0.12,3.3	0

0.12,3.4	0
0.12,3.5	0
0.12,3.6	0
0.12,3.7	0
0.12,3.8	0
0.12,3.9	0
0.12,4.0	0
0.12,4.1	0
0.12,4.2	0
0.12,4.3	0
0.12,4.4	0
0.12,4.5	0
0.12,4.6	0
0.12,4.7	0
0.12,4.8	0
0.12,4.9	0
0.12,5.0	0
0.12,5.1	0
0.12,5.2	0
0.12,5.3	0
0.12,5.4	0
0.12,5.5	0
0.12,5.6	0
0.12,5.7	0
0.12,5.8	0
0.12,5.9	0
0.12,6.0	0
0.12,6.1	0
0.12,6.2	0
0.12,6.3	0
0.12,6.4	0
0.12,6.5	0
0.12,6.6	0
0.12,6.7	0
0.12,6.8	0
0.12,6.9	0
0.12,7.0	0
0.12,7.1	0
0.12,7.2	0
0.12,7.3	0
0.12,7.4	0
0.12,7.5	0

0.12,7.6	0
0.12,7.7	0
0.12,7.8	0
0.12,7.9	0
0.12,8.0	0
0.12,8.1	0
0.12,8.2	0
0.12,8.3	0
0.12,8.4	0
0.12,8.5	0
0.12,8.6	0
0.12,8.7	0
0.12,8.8	0
0.12,8.9	0
0.12,9.0	0
0.12,9.1	0
0.12,9.2	0
0.12,9.3	0
0.12,9.4	0
0.12,9.5	0
0.12,9.6	0
0.12,9.7	0
0.12,9.8	0
0.12,9.9	0
0.12,10.0	0
0.12,10.1	0
0.12,10.2	0
0.12,10.3	0
0.12,10.4	0
0.12,10.5	0
0.12,10.6	0
0.12,10.7	0
0.12,10.8	0
0.12,10.9	0
0.12,11.0	0
0.12,11.1	0
0.12,11.2	0
0.12,11.3	0
0.12,11.4	0
0.12,11.5	0
0.12,11.6	0
0.12,11.7	0

0.12,11.8	0
0.12,11.9	0
0.12,12.0	0
0.12,12.1	0
0.12,12.2	0
0.12,12.3	0
0.12,12.4	0
0.12,12.5	0
0.12,12.6	0
0.12,12.7	0
0.12,12.8	0
0.12,12.9	0
0.12,13.0	0
0.12,13.1	0
0.12,13.2	0
0.12,13.3	0
0.12,13.4	0
0.12,13.5	0
0.12,13.6	0
0.12,13.7	0
0.12,13.8	0
0.12,13.9	0
0.12,14.0	0
0.12,14.1	0
0.12,14.2	0
0.12,14.3	0
0.12,14.4	0
0.12,14.5	0
0.12,14.6	0
0.12,14.7	0
0.12,14.8	0
0.12,14.9	0
0.12,15.0	0
0.12,15.1	0
0.12,15.2	0
0.12,15.3	0
0.12,15.4	0
0.12,15.5	0
0.12,15.6	0
0.12,15.7	0
0.12,15.8	0
0.12,15.9	0

0.12,16.0	0
0.12,16.1	0
0.12,16.2	0
0.12,16.3	0
0.12,16.4	0
0.12,16.5	0
0.12,16.6	0
0.12,16.7	0
0.12,16.8	0
0.12,16.9	0
0.12,17.0	0
0.12,17.1	0
0.12,17.2	0
0.12,17.3	0
0.12,17.4	0
0.12,17.5	0
0.12,17.6	0
0.12,17.7	0
0.12,17.8	0
0.12,17.9	0
0.12,18.0	0
0.12,18.1	0
0.12,18.2	0
0.12,18.3	0
0.12,18.4	0
0.12,18.5	0
0.12,18.6	0
0.12,18.7	0
0.12,18.8	0
0.12,18.9	0
0.12,19.0	0
0.12,19.1	0
0.12,19.2	0
0.12,19.3	0
0.12,19.4	0
0.12,19.5	0
0.12,19.6	0
0.12,19.7	0
0.12,19.8	0
0.12,19.9	0
0.12,20.0	0
0.12,20.1	0

0.12,20.2	0
0.12,20.3	0
0.12,20.4	0
0.12,20.5	0
0.12,20.6	0
0.12,20.7	0
0.12,20.8	0
0.12,20.9	0
0.12,21.0	0
0.12,21.1	0
0.12,21.2	0
0.12,21.3	0
0.12,21.4	0
0.12,21.5	0
0.12,21.6	0
0.12,21.7	0
0.12,21.8	0
0.12,21.9	0
0.12,22.0	0
0.12,22.1	0
0.12,22.2	0
0.12,22.3	0
0.12,22.4	0
0.12,22.5	0
0.12,22.6	0
0.12,22.7	0
0.12,22.8	0
0.12,22.9	0
0.12,23.0	0
0.12,23.1	0
0.12,23.2	0
0.12,23.3	0
0.12,23.4	0
0.12,23.5	0
0.12,23.6	0
0.12,23.7	0
0.12,23.8	0
0.12,23.9	0
0.12,24.0	0
0.12,24.1	0
0.12,24.2	0
0.12,24.3	0

0.12,24.4	0
0.12,24.5	0
0.12,24.6	0
0.12,24.7	0
0.12,24.8	0
0.12,24.9	0
0.12,25.0	0
0.12,25.1	0
0.12,25.2	0
0.12,25.3	0
0.12,25.4	0
0.12,25.5	0
0.12,25.6	0
0.12,25.7	0
0.12,25.8	0
0.12,25.9	0
0.12,26.0	0
0.12,26.1	0
0.12,26.2	0
0.12,26.3	0
0.12,26.4	0
0.12,26.5	0
0.12,26.6	0
0.12,26.7	0
0.12,26.8	0
0.12,26.9	0
0.12,27.0	0
0.12,27.1	0
0.12,27.2	0
0.12,27.3	0
0.12,27.4	0
0.12,27.5	0
0.12,27.6	0
0.12,27.7	0
0.12,27.8	0
0.12,27.9	0
0.12,28.0	0
0.12,28.1	0
0.12,28.2	0
0.12,28.3	0
0.12,28.4	0
0.12,28.5	0

0.12,28.6	0
0.12,28.7	0
0.12,28.8	0
0.12,28.9	0
0.12,29.0	0
0.12,29.1	0
0.12,29.2	0
0.12,29.3	0
0.12,29.4	0
0.12,29.5	0
0.12,29.6	0
0.12,29.7	0
0.12,29.8	0
0.12,29.9	0
0.12,30.0	0
0.12,30.1	0
0.12,30.2	0
0.12,30.3	0
0.12,30.4	0
0.12,30.5	0
0.12,30.6	0
0.12,30.7	0
0.12,30.8	0
0.12,30.9	0
0.12,31.0	0
0.12,31.1	0
0.12,31.2	0
0.12,31.3	0
0.12,31.4	0
0.12,31.5	0
0.12,31.6	0
0.12,31.7	0
0.12,31.8	0
0.12,31.9	0
0.12,32.0	0
0.12,32.1	0
0.12,32.2	0
0.12,32.3	0
0.12,32.4	0
0.12,32.5	0
0.12,32.6	0
0.12,32.7	0

0.12,32.8	0
0.12,32.9	0
0.12,33.0	0
0.12,33.1	0
0.12,33.2	0
0.12,33.3	0
0.12,33.4	0
0.12,33.5	0
0.12,33.6	0
0.12,33.7	0
0.12,33.8	0
0.12,33.9	0
0.12,34.0	0
0.12,34.1	0
0.12,34.2	0
0.12,34.3	0
0.12,34.4	0
0.12,34.5	0
0.12,34.6	0
0.12,34.7	0
0.12,34.8	0
0.12,34.9	0
0.12,35.0	0
0.12,35.1	0
0.12,35.2	0
0.12,35.3	0
0.12,35.4	0
0.12,35.5	0
0.12,35.6	0
0.12,35.7	0
0.12,35.8	0
0.12,35.9	0
0.12,36.0	0
0.12,36.1	0
0.12,36.2	0
0.12,36.3	0
0.12,36.4	0
0.12,36.5	0
0.12,36.6	0
0.12,36.7	0
0.12,36.8	0
0.12,36.9	0

0.12,37.0	0
0.12,37.1	0
0.12,37.2	0
0.12,37.3	0
0.12,37.4	0
0.12,37.5	0
0.12,37.6	0
0.12,37.7	0
0.12,37.8	0
0.12,37.9	0
0.12,38.0	0
0.12,38.1	0
0.12,38.2	0
0.12,38.3	0
0.12,38.4	0
0.12,38.5	0
0.12,38.6	0
0.12,38.7	0
0.12,38.8	0
0.12,38.9	0
0.12,39.0	0
0.12,39.1	0
0.12,39.2	0
0.12,39.3	0
0.12,39.4	0
0.12,39.5	0
0.12,39.6	0
0.12,39.7	0
0.12,39.8	0
0.12,39.9	0
0.12,40.0	0
0.12,40.1	0
0.12,40.2	0
0.12,40.3	0
0.12,40.4	0
0.12,40.5	0
0.12,40.6	0
0.12,40.7	0
0.12,40.8	0
0.12,40.9	0
0.12,41.0	0
0.12,41.1	0

0.12,41.2	0
0.12,41.3	0
0.12,41.4	0
0.12,41.5	0
0.12,41.6	0
0.12,41.7	0
0.12,41.8	0
0.12,41.9	0
0.12,42.0	0
0.12,42.1	0
0.12,42.2	0
0.12,42.3	0
0.12,42.4	0
0.12,42.5	0
0.12,42.6	0
0.12,42.7	0
0.12,42.8	0
0.12,42.9	0
0.12,43.0	0
0.12,43.1	0
0.12,43.2	0
0.12,43.3	0
0.12,43.4	0
0.12,43.5	0
0.12,43.6	0
0.12,43.7	0
0.12,43.8	0
0.12,43.9	0
0.12,44.0	0
0.12,44.1	0
0.12,44.2	0
0.12,44.3	0
0.12,44.4	0
0.12,44.5	0
0.12,44.6	0
0.12,44.7	0
0.12,44.8	0
0.12,44.9	0
0.12,45.0	0
0.12,45.1	0
0.12,45.2	0
0.12,45.3	0

0.12,45.4	0
0.12,45.5	0
0.12,45.6	0
0.12,45.7	0
0.12,45.8	0
0.12,45.9	0
0.12,46.0	0
0.12,46.1	0
0.12,46.2	0
0.12,46.3	0
0.12,46.4	0
0.12,46.5	0
0.12,46.6	0
0.12,46.7	0
0.12,46.8	0
0.12,46.9	0
0.12,47.0	0
0.12,47.1	0
0.12,47.2	0
0.12,47.3	0
0.12,47.4	0
0.12,47.5	0
0.12,47.6	0
0.12,47.7	0
0.12,47.8	0
0.12,47.9	0
0.12,48.0	0
0.12,48.1	0
0.12,48.2	0
0.12,48.3	0
0.12,48.4	0
0.12,48.5	0
0.12,48.6	0
0.12,48.7	0
0.12,48.8	0
0.12,48.9	0
0.12,49.0	0
0.12,49.1	0
0.12,49.2	0
0.12,49.3	0
0.12,49.4	0
0.12,49.5	0

0.12,49.6	0
0.12,49.7	0
0.12,49.8	0
0.12,49.9	0
0.12,50.0	0
0.12,50.1	0
0.12,50.2	0
0.12,50.3	0
0.12,50.4	0
0.12,50.5	0
0.12,50.6	0
0.12,50.7	0
0.12,50.8	0
0.12,50.9	0
0.12,51.0	0
0.12,51.1	0
0.12,51.2	0
0.12,51.3	0
0.12,51.4	0
0.12,51.5	0
0.12,51.6	0
0.12,51.7	0
0.12,51.8	0
0.12,51.9	0
0.12,52.0	0
0.12,52.1	0
0.12,52.2	0
0.12,52.3	0
0.12,52.4	0
0.12,52.5	0
0.12,52.6	0
0.12,52.7	0
0.12,52.8	0
0.12,52.9	0
0.12,53.0	0
0.12,53.1	0
0.12,53.2	0
0.12,53.3	0
0.12,53.4	0
0.12,53.5	0
0.12,53.6	0
0.12,53.7	0

0.12,53.8	0
0.12,53.9	0
0.12,54.0	0
0.12,54.1	0
0.12,54.2	0
0.12,54.3	0
0.12,54.4	0
0.12,54.5	0
0.12,54.6	0
0.12,54.7	0
0.12,54.8	0
0.12,54.9	0
0.12,55.0	0
0.12,55.1	0
0.12,55.2	0
0.12,55.3	0
0.12,55.4	0
0.12,55.5	0
0.12,55.6	0
0.12,55.7	0
0.12,55.8	0
0.12,55.9	0
0.12,56.0	0
0.12,56.1	0
0.12,56.2	0
0.12,56.3	0
0.12,56.4	0
0.12,56.5	0
0.12,56.6	0
0.12,56.7	0
0.12,56.8	0
0.12,56.9	0
0.12,57.0	0
0.12,57.1	0
0.12,57.2	0
0.12,57.3	0
0.12,57.4	0
0.12,57.5	0
0.12,57.6	0
0.12,57.7	0
0.12,57.8	0
0.12,57.9	0

0.12,58.0	0
0.12,58.1	0
0.12,58.2	0
0.12,58.3	0
0.12,58.4	0
0.12,58.5	0
0.12,58.6	0
0.12,58.7	0
0.12,58.8	0
0.12,58.9	0
0.12,59.0	0
0.12,59.1	0
0.12,59.2	0
0.12,59.3	0
0.12,59.4	0
0.12,59.5	0
0.12,59.6	0
0.12,59.7	0
0.12,59.8	0
0.12,59.9	0
0.12,60.0	0
0.12,60.1	0
0.12,60.2	0
0.12,60.3	0
0.12,60.4	0
0.12,60.5	0
0.12,60.6	0
0.12,60.7	0
0.12,60.8	0
0.12,60.9	0
0.12,61.0	0
0.12,61.1	0
0.12,61.2	0
0.12,61.3	0
0.12,61.4	0
0.12,61.5	0
0.12,61.6	0
0.12,61.7	0
0.12,61.8	0
0.12,61.9	0
0.12,62.0	0
0.12,62.1	0

0.12,62.2	0
0.12,62.3	0
0.12,62.4	0
0.12,62.5	0
0.12,62.6	0
0.12,62.7	0
0.12,62.8	0
0.12,62.9	0
0.12,63.0	0
0.12,63.1	0
0.12,63.2	0
0.12,63.3	0
0.12,63.4	0
0.12,63.5	0
0.12,63.6	0
0.12,63.7	0
0.12,63.8	0
0.12,63.9	0
0.12,64.0	0
0.12,64.1	0
0.12,64.2	0
0.12,64.3	0
0.12,64.4	0
0.12,64.5	0
0.12,64.6	0
0.12,64.7	0
0.12,64.8	0
0.12,64.9	0
0.12,65.0	0
0.12,65.1	0
0.12,65.2	0
0.12,65.3	0
0.12,65.4	0
0.12,65.5	0
0.12,65.6	0
0.12,65.7	0
0.12,65.8	0
0.12,65.9	0
0.12,66.0	0
0.12,66.1	0
0.12,66.2	0
0.12,66.3	0

0.12,66.4	0
0.12,66.5	0
0.12,66.6	0
0.12,66.7	0
0.12,66.8	0
0.12,66.9	0
0.12,67.0	0
0.12,67.1	0
0.12,67.2	0
0.12,67.3	0
0.12,67.4	0
0.12,67.5	0
0.12,67.6	0
0.12,67.7	0
0.12,67.8	0
0.12,67.9	0
0.12,68.0	0
0.12,68.1	0
0.12,68.2	0
0.12,68.3	0
0.12,68.4	0
0.12,68.5	0
0.12,68.6	0
0.12,68.7	0
0.12,68.8	0
0.12,68.9	0
0.12,69.0	0
0.12,69.1	0
0.12,69.2	0
0.12,69.3	0
0.12,69.4	0
0.12,69.5	0
0.12,69.6	0
0.12,69.7	0
0.12,69.8	0
0.12,69.9	0
0.12,70.0	0
0.12,70.1	0
0.12,70.2	0
0.12,70.3	0
0.12,70.4	0
0.12,70.5	0

0.12,70.6	0
0.12,70.7	0
0.12,70.8	0
0.12,70.9	0
0.12,71.0	0
0.12,71.1	0
0.12,71.2	0
0.12,71.3	0
0.12,71.4	0
0.12,71.5	0
0.12,71.6	0
0.12,71.7	0
0.12,71.8	0
0.12,71.9	0
0.12,72.0	0
0.12,72.1	0
0.12,72.2	0
0.12,72.3	0
0.12,72.4	0
0.12,72.5	0
0.12,72.6	0
0.12,72.7	0
0.12,72.8	0
0.12,72.9	0
0.12,73.0	0
0.12,73.1	0
0.12,73.2	0
0.12,73.3	0
0.12,73.4	0
0.12,73.5	0
0.12,73.6	0
0.12,73.7	0
0.12,73.8	0
0.12,73.9	0
0.12,74.0	0
0.12,74.1	0
0.12,74.2	0
0.12,74.3	0
0.12,74.4	0
0.12,74.5	0
0.12,74.6	0
0.12,74.7	0

0.12,74.8	0
0.12,74.9	0
0.12,75.0	0
0.12,75.1	0
0.12,75.2	0
0.12,75.3	0
0.12,75.4	0
0.12,75.5	0
0.12,75.6	0
0.12,75.7	0
0.12,75.8	0
0.12,75.9	0
0.12,76.0	0
0.12,76.1	0
0.12,76.2	0
0.12,76.3	0
0.12,76.4	0
0.12,76.5	0
0.12,76.6	0
0.12,76.7	0
0.12,76.8	0
0.12,76.9	0
0.12,77.0	0
0.12,77.1	0
0.12,77.2	0
0.12,77.3	0
0.12,77.4	0
0.12,77.5	0
0.12,77.6	0
0.12,77.7	0
0.12,77.8	0
0.12,77.9	0
0.12,78.0	0
0.12,78.1	0
0.12,78.2	0
0.12,78.3	0
0.12,78.4	0
0.12,78.5	0
0.12,78.6	0
0.12,78.7	0
0.12,78.8	0
0.12,78.9	0

0.12,79.0	0
0.12,79.1	0
0.12,79.2	0
0.12,79.3	0
0.12,79.4	0
0.12,79.5	0
0.12,79.6	0
0.12,79.7	0
0.12,79.8	0
0.12,79.9	0
0.12,80.0	0
0.12,80.1	0
0.12,80.2	0
0.12,80.3	0
0.12,80.4	0
0.12,80.5	0
0.12,80.6	0
0.12,80.7	0
0.12,80.8	0
0.12,80.9	0
0.12,81.0	0
0.12,81.1	0
0.12,81.2	0
0.12,81.3	0
0.12,81.4	0
0.12,81.5	0
0.12,81.6	0
0.12,81.7	0
0.12,81.8	0
0.12,81.9	0
0.12,82.0	0
0.12,82.1	0
0.12,82.2	0
0.12,82.3	0
0.12,82.4	0
0.12,82.5	0
0.12,82.6	0
0.12,82.7	0
0.12,82.8	0
0.12,82.9	0
0.12,83.0	0
0.12,83.1	0

0.12,83.2	0
0.12,83.3	0
0.12,83.4	0
0.12,83.5	0
0.12,83.6	0
0.12,83.7	0
0.12,83.8	0
0.12,83.9	0
0.12,84.0	0
0.12,84.1	0
0.12,84.2	0
0.12,84.3	0
0.12,84.4	0
0.12,84.5	0
0.12,84.6	0
0.12,84.7	0
0.12,84.8	0
0.12,84.9	0
0.12,85.0	0
0.12,85.1	0
0.12,85.2	0
0.12,85.3	0
0.12,85.4	0
0.12,85.5	0
0.12,85.6	0
0.12,85.7	0
0.12,85.8	0
0.12,85.9	0
0.12,86.0	0
0.12,86.1	0
0.12,86.2	0
0.12,86.3	0
0.12,86.4	0
0.12,86.5	0
0.12,86.6	0
0.12,86.7	0
0.12,86.8	0
0.12,86.9	0
0.12,87.0	0
0.12,87.1	0
0.12,87.2	0
0.12,87.3	0

0.12,87.4	0
0.12,87.5	0
0.12,87.6	0
0.12,87.7	0
0.12,87.8	0
0.12,87.9	0
0.12,88.0	0
0.12,88.1	0
0.12,88.2	0
0.12,88.3	0
0.12,88.4	0
0.12,88.5	0
0.12,88.6	0
0.12,88.7	0
0.12,88.8	0
0.12,88.9	0
0.12,89.0	0
0.12,89.1	0
0.12,89.2	0
0.12,89.3	0
0.12,89.4	0
0.12,89.5	0
0.12,89.6	0
0.12,89.7	0
0.12,89.8	0
0.12,89.9	0
0.12,90.0	0
0.12,90.1	0
0.12,90.2	0
0.12,90.3	0
0.12,90.4	0
0.12,90.5	0
0.12,90.6	0
0.12,90.7	0
0.12,90.8	0
0.12,90.9	0
0.12,91.0	0
0.12,91.1	0
0.12,91.2	0
0.12,91.3	0
0.12,91.4	0
0.12,91.5	0

0.12,91.6	0
0.12,91.7	0
0.12,91.8	0
0.12,91.9	0
0.12,92.0	0
0.12,92.1	0
0.12,92.2	0
0.12,92.3	0
0.12,92.4	0
0.12,92.5	0
0.12,92.6	0
0.12,92.7	0
0.12,92.8	0
0.12,92.9	0
0.12,93.0	0
0.12,93.1	0
0.12,93.2	0
0.12,93.3	0
0.12,93.4	0
0.12,93.5	0
0.12,93.6	0
0.12,93.7	0
0.12,93.8	0
0.12,93.9	0
0.12,94.0	0
0.12,94.1	0
0.12,94.2	0
0.12,94.3	0
0.12,94.4	0
0.12,94.5	0
0.12,94.6	0
0.12,94.7	0
0.12,94.8	0
0.12,94.9	0
0.12,95.0	0
0.12,95.1	0
0.12,95.2	0
0.12,95.3	0
0.12,95.4	0
0.12,95.5	0
0.12,95.6	0
0.12,95.7	0

0.12,95.8	0
0.12,95.9	0
0.12,96.0	0
0.12,96.1	0
0.12,96.2	0
0.12,96.3	0
0.12,96.4	0
0.12,96.5	0
0.12,96.6	0
0.12,96.7	0
0.12,96.8	0
0.12,96.9	0
0.12,97.0	0
0.12,97.1	0
0.12,97.2	0
0.12,97.3	0
0.12,97.4	0
0.12,97.5	0
0.12,97.6	0
0.12,97.7	0
0.12,97.8	0
0.12,97.9	0
0.12,98.0	0
0.12,98.1	0
0.12,98.2	0
0.12,98.3	0
0.12,98.4	0
0.12,98.5	0
0.12,98.6	0
0.12,98.7	0
0.12,98.8	0
0.12,98.9	0
0.12,99.0	0
0.12,99.1	0
0.12,99.2	0
0.12,99.3	0
0.12,99.4	0
0.12,99.5	0
0.12,99.6	0
0.12,99.7	0
0.12,99.8	0
0.12,99.9	0

0.12,100.0	0
0.13,0.0	0
0.13,0.1	0
0.13,0.2	0
0.13,0.3	0
0.13,0.4	0
0.13,0.5	0
0.13,0.6	0
0.13,0.7	0
0.13,0.8	0
0.13,0.9	0
0.13,1.0	0
0.13,1.1	0
0.13,1.2	0
0.13,1.3	0
0.13,1.4	0
0.13,1.5	0
0.13,1.6	0
0.13,1.7	0
0.13,1.8	0
0.13,1.9	0
0.13,2.0	0
0.13,2.1	0
0.13,2.2	0
0.13,2.3	0
0.13,2.4	0
0.13,2.5	0
0.13,2.6	0
0.13,2.7	0
0.13,2.8	0
0.13,2.9	0
0.13,3.0	0
0.13,3.1	0
0.13,3.2	0
0.13,3.3	0
0.13,3.4	0
0.13,3.5	0
0.13,3.6	0
0.13,3.7	0
0.13,3.8	0
0.13,3.9	0
0.13,4.0	0

0.13,4.1	0
0.13,4.2	0
0.13,4.3	0
0.13,4.4	0
0.13,4.5	0
0.13,4.6	0
0.13,4.7	0
0.13,4.8	0
0.13,4.9	0
0.13,5.0	0
0.13,5.1	0
0.13,5.2	0
0.13,5.3	0
0.13,5.4	0
0.13,5.5	0
0.13,5.6	0
0.13,5.7	0
0.13,5.8	0
0.13,5.9	0
0.13,6.0	0
0.13,6.1	0
0.13,6.2	0
0.13,6.3	0
0.13,6.4	0
0.13,6.5	0
0.13,6.6	0
0.13,6.7	0
0.13,6.8	0
0.13,6.9	0
0.13,7.0	0
0.13,7.1	0
0.13,7.2	0
0.13,7.3	0
0.13,7.4	0
0.13,7.5	0
0.13,7.6	0
0.13,7.7	0
0.13,7.8	0
0.13,7.9	0
0.13,8.0	0
0.13,8.1	0
0.13,8.2	0

0.13,8.3	0
0.13,8.4	0
0.13,8.5	0
0.13,8.6	0
0.13,8.7	0
0.13,8.8	0
0.13,8.9	0
0.13,9.0	0
0.13,9.1	0
0.13,9.2	0
0.13,9.3	0
0.13,9.4	0
0.13,9.5	0
0.13,9.6	0
0.13,9.7	0
0.13,9.8	0
0.13,9.9	0
0.13,10.0	0
0.13,10.1	0
0.13,10.2	0
0.13,10.3	0
0.13,10.4	0
0.13,10.5	0
0.13,10.6	0
0.13,10.7	0
0.13,10.8	0
0.13,10.9	0
0.13,11.0	0
0.13,11.1	0
0.13,11.2	0
0.13,11.3	0
0.13,11.4	0
0.13,11.5	0
0.13,11.6	0
0.13,11.7	0
0.13,11.8	0
0.13,11.9	0
0.13,12.0	0
0.13,12.1	0
0.13,12.2	0
0.13,12.3	0
0.13,12.4	0

0.13,12.5	0
0.13,12.6	0
0.13,12.7	0
0.13,12.8	0
0.13,12.9	0
0.13,13.0	0
0.13,13.1	0
0.13,13.2	0
0.13,13.3	0
0.13,13.4	0
0.13,13.5	0
0.13,13.6	0
0.13,13.7	0
0.13,13.8	0
0.13,13.9	0
0.13,14.0	0
0.13,14.1	0
0.13,14.2	0
0.13,14.3	0
0.13,14.4	0
0.13,14.5	0
0.13,14.6	0
0.13,14.7	0
0.13,14.8	0
0.13,14.9	0
0.13,15.0	0
0.13,15.1	0
0.13,15.2	0
0.13,15.3	0
0.13,15.4	0
0.13,15.5	0
0.13,15.6	0
0.13,15.7	0
0.13,15.8	0
0.13,15.9	0
0.13,16.0	0
0.13,16.1	0
0.13,16.2	0
0.13,16.3	0
0.13,16.4	0
0.13,16.5	0
0.13,16.6	0

0.13,16.7	0
0.13,16.8	0
0.13,16.9	0
0.13,17.0	0
0.13,17.1	0
0.13,17.2	0
0.13,17.3	0
0.13,17.4	0
0.13,17.5	0
0.13,17.6	0
0.13,17.7	0
0.13,17.8	0
0.13,17.9	0
0.13,18.0	0
0.13,18.1	0
0.13,18.2	0
0.13,18.3	0
0.13,18.4	0
0.13,18.5	0
0.13,18.6	0
0.13,18.7	0
0.13,18.8	0
0.13,18.9	0
0.13,19.0	0
0.13,19.1	0
0.13,19.2	0
0.13,19.3	0
0.13,19.4	0
0.13,19.5	0
0.13,19.6	0
0.13,19.7	0
0.13,19.8	0
0.13,19.9	0
0.13,20.0	0
0.13,20.1	0
0.13,20.2	0
0.13,20.3	0
0.13,20.4	0
0.13,20.5	0
0.13,20.6	0
0.13,20.7	0
0.13,20.8	0

0.13,20.9	0
0.13,21.0	0
0.13,21.1	0
0.13,21.2	0
0.13,21.3	0
0.13,21.4	0
0.13,21.5	0
0.13,21.6	0
0.13,21.7	0
0.13,21.8	0
0.13,21.9	0
0.13,22.0	0
0.13,22.1	0
0.13,22.2	0
0.13,22.3	0
0.13,22.4	0
0.13,22.5	0
0.13,22.6	0
0.13,22.7	0
0.13,22.8	0
0.13,22.9	0
0.13,23.0	0
0.13,23.1	0
0.13,23.2	0
0.13,23.3	0
0.13,23.4	0
0.13,23.5	0
0.13,23.6	0
0.13,23.7	0
0.13,23.8	0
0.13,23.9	0
0.13,24.0	0
0.13,24.1	0
0.13,24.2	0
0.13,24.3	0
0.13,24.4	0
0.13,24.5	0
0.13,24.6	0
0.13,24.7	0
0.13,24.8	0
0.13,24.9	0
0.13,25.0	0

0.13,25.1	0
0.13,25.2	0
0.13,25.3	0
0.13,25.4	0
0.13,25.5	0
0.13,25.6	0
0.13,25.7	0
0.13,25.8	0
0.13,25.9	0
0.13,26.0	0
0.13,26.1	0
0.13,26.2	0
0.13,26.3	0
0.13,26.4	0
0.13,26.5	0
0.13,26.6	0
0.13,26.7	0
0.13,26.8	0
0.13,26.9	0
0.13,27.0	0
0.13,27.1	0
0.13,27.2	0
0.13,27.3	0
0.13,27.4	0
0.13,27.5	0
0.13,27.6	0
0.13,27.7	0
0.13,27.8	0
0.13,27.9	0
0.13,28.0	0
0.13,28.1	0
0.13,28.2	0
0.13,28.3	0
0.13,28.4	0
0.13,28.5	0
0.13,28.6	0
0.13,28.7	0
0.13,28.8	0
0.13,28.9	0
0.13,29.0	0
0.13,29.1	0
0.13,29.2	0

0.13,29.3	0
0.13,29.4	0
0.13,29.5	0
0.13,29.6	0
0.13,29.7	0
0.13,29.8	0
0.13,29.9	0
0.13,30.0	0
0.13,30.1	0
0.13,30.2	0
0.13,30.3	0
0.13,30.4	0
0.13,30.5	0
0.13,30.6	0
0.13,30.7	0
0.13,30.8	0
0.13,30.9	0
0.13,31.0	0
0.13,31.1	0
0.13,31.2	0
0.13,31.3	0
0.13,31.4	0
0.13,31.5	0
0.13,31.6	0
0.13,31.7	0
0.13,31.8	0
0.13,31.9	0
0.13,32.0	0
0.13,32.1	0
0.13,32.2	0
0.13,32.3	0
0.13,32.4	0
0.13,32.5	0
0.13,32.6	0
0.13,32.7	0
0.13,32.8	0
0.13,32.9	0
0.13,33.0	0
0.13,33.1	0
0.13,33.2	0
0.13,33.3	0
0.13,33.4	0

0.13,33.5	0
0.13,33.6	0
0.13,33.7	0
0.13,33.8	0
0.13,33.9	0
0.13,34.0	0
0.13,34.1	0
0.13,34.2	0
0.13,34.3	0
0.13,34.4	0
0.13,34.5	0
0.13,34.6	0
0.13,34.7	0
0.13,34.8	0
0.13,34.9	0
0.13,35.0	0
0.13,35.1	0
0.13,35.2	0
0.13,35.3	0
0.13,35.4	0
0.13,35.5	0
0.13,35.6	0
0.13,35.7	0
0.13,35.8	0
0.13,35.9	0
0.13,36.0	0
0.13,36.1	0
0.13,36.2	0
0.13,36.3	0
0.13,36.4	0
0.13,36.5	0
0.13,36.6	0
0.13,36.7	0
0.13,36.8	0
0.13,36.9	0
0.13,37.0	0
0.13,37.1	0
0.13,37.2	0
0.13,37.3	0
0.13,37.4	0
0.13,37.5	0
0.13,37.6	0

0.13,37.7	0
0.13,37.8	0
0.13,37.9	0
0.13,38.0	0
0.13,38.1	0
0.13,38.2	0
0.13,38.3	0
0.13,38.4	0
0.13,38.5	0
0.13,38.6	0
0.13,38.7	0
0.13,38.8	0
0.13,38.9	0
0.13,39.0	0
0.13,39.1	0
0.13,39.2	0
0.13,39.3	0
0.13,39.4	0
0.13,39.5	0
0.13,39.6	0
0.13,39.7	0
0.13,39.8	0
0.13,39.9	0
0.13,40.0	0
0.13,40.1	0
0.13,40.2	0
0.13,40.3	0
0.13,40.4	0
0.13,40.5	0
0.13,40.6	0
0.13,40.7	0
0.13,40.8	0
0.13,40.9	0
0.13,41.0	0
0.13,41.1	0
0.13,41.2	0
0.13,41.3	0
0.13,41.4	0
0.13,41.5	0
0.13,41.6	0
0.13,41.7	0
0.13,41.8	0

0.13,41.9	0
0.13,42.0	0
0.13,42.1	0
0.13,42.2	0
0.13,42.3	0
0.13,42.4	0
0.13,42.5	0
0.13,42.6	0
0.13,42.7	0
0.13,42.8	0
0.13,42.9	0
0.13,43.0	0
0.13,43.1	0
0.13,43.2	0
0.13,43.3	0
0.13,43.4	0
0.13,43.5	0
0.13,43.6	0
0.13,43.7	0
0.13,43.8	0
0.13,43.9	0
0.13,44.0	0
0.13,44.1	0
0.13,44.2	0
0.13,44.3	0
0.13,44.4	0
0.13,44.5	0
0.13,44.6	0
0.13,44.7	0
0.13,44.8	0
0.13,44.9	0
0.13,45.0	0
0.13,45.1	0
0.13,45.2	0
0.13,45.3	0
0.13,45.4	0
0.13,45.5	0
0.13,45.6	0
0.13,45.7	0
0.13,45.8	0
0.13,45.9	0
0.13,46.0	0

0.13,46.1	0
0.13,46.2	0
0.13,46.3	0
0.13,46.4	0
0.13,46.5	0
0.13,46.6	0
0.13,46.7	0
0.13,46.8	0
0.13,46.9	0
0.13,47.0	0
0.13,47.1	0
0.13,47.2	0
0.13,47.3	0
0.13,47.4	0
0.13,47.5	0
0.13,47.6	0
0.13,47.7	0
0.13,47.8	0
0.13,47.9	0
0.13,48.0	0
0.13,48.1	0
0.13,48.2	0
0.13,48.3	0
0.13,48.4	0
0.13,48.5	0
0.13,48.6	0
0.13,48.7	0
0.13,48.8	0
0.13,48.9	0
0.13,49.0	0
0.13,49.1	0
0.13,49.2	0
0.13,49.3	0
0.13,49.4	0
0.13,49.5	0
0.13,49.6	0
0.13,49.7	0
0.13,49.8	0
0.13,49.9	0
0.13,50.0	0
0.13,50.1	0
0.13,50.2	0

0.13,50.3	0
0.13,50.4	0
0.13,50.5	0
0.13,50.6	0
0.13,50.7	0
0.13,50.8	0
0.13,50.9	0
0.13,51.0	0
0.13,51.1	0
0.13,51.2	0
0.13,51.3	0
0.13,51.4	0
0.13,51.5	0
0.13,51.6	0
0.13,51.7	0
0.13,51.8	0
0.13,51.9	0
0.13,52.0	0
0.13,52.1	0
0.13,52.2	0
0.13,52.3	0
0.13,52.4	0
0.13,52.5	0
0.13,52.6	0
0.13,52.7	0
0.13,52.8	0
0.13,52.9	0
0.13,53.0	0
0.13,53.1	0
0.13,53.2	0
0.13,53.3	0
0.13,53.4	0
0.13,53.5	0
0.13,53.6	0
0.13,53.7	0
0.13,53.8	0
0.13,53.9	0
0.13,54.0	0
0.13,54.1	0
0.13,54.2	0
0.13,54.3	0
0.13,54.4	0

0.13,54.5	0
0.13,54.6	0
0.13,54.7	0
0.13,54.8	0
0.13,54.9	0
0.13,55.0	0
0.13,55.1	0
0.13,55.2	0
0.13,55.3	0
0.13,55.4	0
0.13,55.5	0
0.13,55.6	0
0.13,55.7	0
0.13,55.8	0
0.13,55.9	0
0.13,56.0	0
0.13,56.1	0
0.13,56.2	0
0.13,56.3	0
0.13,56.4	0
0.13,56.5	0
0.13,56.6	0
0.13,56.7	0
0.13,56.8	0
0.13,56.9	0
0.13,57.0	0
0.13,57.1	0
0.13,57.2	0
0.13,57.3	0
0.13,57.4	0
0.13,57.5	0
0.13,57.6	0
0.13,57.7	0
0.13,57.8	0
0.13,57.9	0
0.13,58.0	0
0.13,58.1	0
0.13,58.2	0
0.13,58.3	0
0.13,58.4	0
0.13,58.5	0
0.13,58.6	0

0.13,58.7	0
0.13,58.8	0
0.13,58.9	0
0.13,59.0	0
0.13,59.1	0
0.13,59.2	0
0.13,59.3	0
0.13,59.4	0
0.13,59.5	0
0.13,59.6	0
0.13,59.7	0
0.13,59.8	0
0.13,59.9	0
0.13,60.0	0
0.13,60.1	0
0.13,60.2	0
0.13,60.3	0
0.13,60.4	0
0.13,60.5	0
0.13,60.6	0
0.13,60.7	0
0.13,60.8	0
0.13,60.9	0
0.13,61.0	0
0.13,61.1	0
0.13,61.2	0
0.13,61.3	0
0.13,61.4	0
0.13,61.5	0
0.13,61.6	0
0.13,61.7	0
0.13,61.8	0
0.13,61.9	0
0.13,62.0	0
0.13,62.1	0
0.13,62.2	0
0.13,62.3	0
0.13,62.4	0
0.13,62.5	0
0.13,62.6	0
0.13,62.7	0
0.13,62.8	0

0.13,62.9	0
0.13,63.0	0
0.13,63.1	0
0.13,63.2	0
0.13,63.3	0
0.13,63.4	0
0.13,63.5	0
0.13,63.6	0
0.13,63.7	0
0.13,63.8	0
0.13,63.9	0
0.13,64.0	0
0.13,64.1	0
0.13,64.2	0
0.13,64.3	0
0.13,64.4	0
0.13,64.5	0
0.13,64.6	0
0.13,64.7	0
0.13,64.8	0
0.13,64.9	0
0.13,65.0	0
0.13,65.1	0
0.13,65.2	0
0.13,65.3	0
0.13,65.4	0
0.13,65.5	0
0.13,65.6	0
0.13,65.7	0
0.13,65.8	0
0.13,65.9	0
0.13,66.0	0
0.13,66.1	0
0.13,66.2	0
0.13,66.3	0
0.13,66.4	0
0.13,66.5	0
0.13,66.6	0
0.13,66.7	0
0.13,66.8	0
0.13,66.9	0
0.13,67.0	0

0.13,67.1	0
0.13,67.2	0
0.13,67.3	0
0.13,67.4	0
0.13,67.5	0
0.13,67.6	0
0.13,67.7	0
0.13,67.8	0
0.13,67.9	0
0.13,68.0	0
0.13,68.1	0
0.13,68.2	0
0.13,68.3	0
0.13,68.4	0
0.13,68.5	0
0.13,68.6	0
0.13,68.7	0
0.13,68.8	0
0.13,68.9	0
0.13,69.0	0
0.13,69.1	0
0.13,69.2	0
0.13,69.3	0
0.13,69.4	0
0.13,69.5	0
0.13,69.6	0
0.13,69.7	0
0.13,69.8	0
0.13,69.9	0
0.13,70.0	0
0.13,70.1	0
0.13,70.2	0
0.13,70.3	0
0.13,70.4	0
0.13,70.5	0
0.13,70.6	0
0.13,70.7	0
0.13,70.8	0
0.13,70.9	0
0.13,71.0	0
0.13,71.1	0
0.13,71.2	0

0.13,71.3	0
0.13,71.4	0
0.13,71.5	0
0.13,71.6	0
0.13,71.7	0
0.13,71.8	0
0.13,71.9	0
0.13,72.0	0
0.13,72.1	0
0.13,72.2	0
0.13,72.3	0
0.13,72.4	0
0.13,72.5	0
0.13,72.6	0
0.13,72.7	0
0.13,72.8	0
0.13,72.9	0
0.13,73.0	0
0.13,73.1	0
0.13,73.2	0
0.13,73.3	0
0.13,73.4	0
0.13,73.5	0
0.13,73.6	0
0.13,73.7	0
0.13,73.8	0
0.13,73.9	0
0.13,74.0	0
0.13,74.1	0
0.13,74.2	0
0.13,74.3	0
0.13,74.4	0
0.13,74.5	0
0.13,74.6	0
0.13,74.7	0
0.13,74.8	0
0.13,74.9	0
0.13,75.0	0
0.13,75.1	0
0.13,75.2	0
0.13,75.3	0
0.13,75.4	0

0.13,75.5	0
0.13,75.6	0
0.13,75.7	0
0.13,75.8	0
0.13,75.9	0
0.13,76.0	0
0.13,76.1	0
0.13,76.2	0
0.13,76.3	0
0.13,76.4	0
0.13,76.5	0
0.13,76.6	0
0.13,76.7	0
0.13,76.8	0
0.13,76.9	0
0.13,77.0	0
0.13,77.1	0
0.13,77.2	0
0.13,77.3	0
0.13,77.4	0
0.13,77.5	0
0.13,77.6	0
0.13,77.7	0
0.13,77.8	0
0.13,77.9	0
0.13,78.0	0
0.13,78.1	0
0.13,78.2	0
0.13,78.3	0
0.13,78.4	0
0.13,78.5	0
0.13,78.6	0
0.13,78.7	0
0.13,78.8	0
0.13,78.9	0
0.13,79.0	0
0.13,79.1	0
0.13,79.2	0
0.13,79.3	0
0.13,79.4	0
0.13,79.5	0
0.13,79.6	0

0.13,79.7	0
0.13,79.8	0
0.13,79.9	0
0.13,80.0	0
0.13,80.1	0
0.13,80.2	0
0.13,80.3	0
0.13,80.4	0
0.13,80.5	0
0.13,80.6	0
0.13,80.7	0
0.13,80.8	0
0.13,80.9	0
0.13,81.0	0
0.13,81.1	0
0.13,81.2	0
0.13,81.3	0
0.13,81.4	0
0.13,81.5	0
0.13,81.6	0
0.13,81.7	0
0.13,81.8	0
0.13,81.9	0
0.13,82.0	0
0.13,82.1	0
0.13,82.2	0
0.13,82.3	0
0.13,82.4	0
0.13,82.5	0
0.13,82.6	0
0.13,82.7	0
0.13,82.8	0
0.13,82.9	0
0.13,83.0	0
0.13,83.1	0
0.13,83.2	0
0.13,83.3	0
0.13,83.4	0
0.13,83.5	0
0.13,83.6	0
0.13,83.7	0
0.13,83.8	0

0.13,83.9	0
0.13,84.0	0
0.13,84.1	0
0.13,84.2	0
0.13,84.3	0
0.13,84.4	0
0.13,84.5	0
0.13,84.6	0
0.13,84.7	0
0.13,84.8	0
0.13,84.9	0
0.13,85.0	0
0.13,85.1	0
0.13,85.2	0
0.13,85.3	0
0.13,85.4	0
0.13,85.5	0
0.13,85.6	0
0.13,85.7	0
0.13,85.8	0
0.13,85.9	0
0.13,86.0	0
0.13,86.1	0
0.13,86.2	0
0.13,86.3	0
0.13,86.4	0
0.13,86.5	0
0.13,86.6	0
0.13,86.7	0
0.13,86.8	0
0.13,86.9	0
0.13,87.0	0
0.13,87.1	0
0.13,87.2	0
0.13,87.3	0
0.13,87.4	0
0.13,87.5	0
0.13,87.6	0
0.13,87.7	0
0.13,87.8	0
0.13,87.9	0
0.13,88.0	0

0.13,88.1	0
0.13,88.2	0
0.13,88.3	0
0.13,88.4	0
0.13,88.5	0
0.13,88.6	0
0.13,88.7	0
0.13,88.8	0
0.13,88.9	0
0.13,89.0	0
0.13,89.1	0
0.13,89.2	0
0.13,89.3	0
0.13,89.4	0
0.13,89.5	0
0.13,89.6	0
0.13,89.7	0
0.13,89.8	0
0.13,89.9	0
0.13,90.0	0
0.13,90.1	0
0.13,90.2	0
0.13,90.3	0
0.13,90.4	0
0.13,90.5	0
0.13,90.6	0
0.13,90.7	0
0.13,90.8	0
0.13,90.9	0
0.13,91.0	0
0.13,91.1	0
0.13,91.2	0
0.13,91.3	0
0.13,91.4	0
0.13,91.5	0
0.13,91.6	0
0.13,91.7	0
0.13,91.8	0
0.13,91.9	0
0.13,92.0	0
0.13,92.1	0
0.13,92.2	0

0.13,92.3	0
0.13,92.4	0
0.13,92.5	0
0.13,92.6	0
0.13,92.7	0
0.13,92.8	0
0.13,92.9	0
0.13,93.0	0
0.13,93.1	0
0.13,93.2	0
0.13,93.3	0
0.13,93.4	0
0.13,93.5	0
0.13,93.6	0
0.13,93.7	0
0.13,93.8	0
0.13,93.9	0
0.13,94.0	0
0.13,94.1	0
0.13,94.2	0
0.13,94.3	0
0.13,94.4	0
0.13,94.5	0
0.13,94.6	0
0.13,94.7	0
0.13,94.8	0
0.13,94.9	0
0.13,95.0	0
0.13,95.1	0
0.13,95.2	0
0.13,95.3	0
0.13,95.4	0
0.13,95.5	0
0.13,95.6	0
0.13,95.7	0
0.13,95.8	0
0.13,95.9	0
0.13,96.0	0
0.13,96.1	0
0.13,96.2	0
0.13,96.3	0
0.13,96.4	0

0.13,96.5	0
0.13,96.6	0
0.13,96.7	0
0.13,96.8	0
0.13,96.9	0
0.13,97.0	0
0.13,97.1	0
0.13,97.2	0
0.13,97.3	0
0.13,97.4	0
0.13,97.5	0
0.13,97.6	0
0.13,97.7	0
0.13,97.8	0
0.13,97.9	0
0.13,98.0	0
0.13,98.1	0
0.13,98.2	0
0.13,98.3	0
0.13,98.4	0
0.13,98.5	0
0.13,98.6	0
0.13,98.7	0
0.13,98.8	0
0.13,98.9	0
0.13,99.0	0
0.13,99.1	0
0.13,99.2	0
0.13,99.3	0
0.13,99.4	0
0.13,99.5	0
0.13,99.6	0
0.13,99.7	0
0.13,99.8	0
0.13,99.9	0
0.13,100.0	0
0.14,0.0	0
0.14,0.1	0
0.14,0.2	0
0.14,0.3	0
0.14,0.4	0
0.14,0.5	0

0.14,0.6	0
0.14,0.7	0
0.14,0.8	0
0.14,0.9	0
0.14,1.0	0
0.14,1.1	0
0.14,1.2	0
0.14,1.3	0
0.14,1.4	0
0.14,1.5	0
0.14,1.6	0
0.14,1.7	0
0.14,1.8	0
0.14,1.9	0
0.14,2.0	0
0.14,2.1	0
0.14,2.2	0
0.14,2.3	0
0.14,2.4	0
0.14,2.5	0
0.14,2.6	0
0.14,2.7	0
0.14,2.8	0
0.14,2.9	0
0.14,3.0	0
0.14,3.1	0
0.14,3.2	0
0.14,3.3	0
0.14,3.4	0
0.14,3.5	0
0.14,3.6	0
0.14,3.7	0
0.14,3.8	0
0.14,3.9	0
0.14,4.0	0
0.14,4.1	0
0.14,4.2	0
0.14,4.3	0
0.14,4.4	0
0.14,4.5	0
0.14,4.6	0
0.14,4.7	0

0.14,4.8	0
0.14,4.9	0
0.14,5.0	0
0.14,5.1	0
0.14,5.2	0
0.14,5.3	0
0.14,5.4	0
0.14,5.5	0
0.14,5.6	0
0.14,5.7	0
0.14,5.8	0
0.14,5.9	0
0.14,6.0	0
0.14,6.1	0
0.14,6.2	0
0.14,6.3	0
0.14,6.4	0
0.14,6.5	0
0.14,6.6	0
0.14,6.7	0
0.14,6.8	0
0.14,6.9	0
0.14,7.0	0
0.14,7.1	0
0.14,7.2	0
0.14,7.3	0
0.14,7.4	0
0.14,7.5	0
0.14,7.6	0
0.14,7.7	0
0.14,7.8	0
0.14,7.9	0
0.14,8.0	0
0.14,8.1	0
0.14,8.2	0
0.14,8.3	0
0.14,8.4	0
0.14,8.5	0
0.14,8.6	0
0.14,8.7	0
0.14,8.8	0
0.14,8.9	0

0.14,9.0	0
0.14,9.1	0
0.14,9.2	0
0.14,9.3	0
0.14,9.4	0
0.14,9.5	0
0.14,9.6	0
0.14,9.7	0
0.14,9.8	0
0.14,9.9	0
0.14,10.0	0
0.14,10.1	0
0.14,10.2	0
0.14,10.3	0
0.14,10.4	0
0.14,10.5	0
0.14,10.6	0
0.14,10.7	0
0.14,10.8	0
0.14,10.9	0
0.14,11.0	0
0.14,11.1	0
0.14,11.2	0
0.14,11.3	0
0.14,11.4	0
0.14,11.5	0
0.14,11.6	0
0.14,11.7	0
0.14,11.8	0
0.14,11.9	0
0.14,12.0	0
0.14,12.1	0
0.14,12.2	0
0.14,12.3	0
0.14,12.4	0
0.14,12.5	0
0.14,12.6	0
0.14,12.7	0
0.14,12.8	0
0.14,12.9	0
0.14,13.0	0
0.14,13.1	0

0.14,13.2	0
0.14,13.3	0
0.14,13.4	0
0.14,13.5	0
0.14,13.6	0
0.14,13.7	0
0.14,13.8	0
0.14,13.9	0
0.14,14.0	0
0.14,14.1	0
0.14,14.2	0
0.14,14.3	0
0.14,14.4	0
0.14,14.5	0
0.14,14.6	0
0.14,14.7	0
0.14,14.8	0
0.14,14.9	0
0.14,15.0	0
0.14,15.1	0
0.14,15.2	0
0.14,15.3	0
0.14,15.4	0
0.14,15.5	0
0.14,15.6	0
0.14,15.7	0
0.14,15.8	0
0.14,15.9	0
0.14,16.0	0
0.14,16.1	0
0.14,16.2	0
0.14,16.3	0
0.14,16.4	0
0.14,16.5	0
0.14,16.6	0
0.14,16.7	0
0.14,16.8	0
0.14,16.9	0
0.14,17.0	0
0.14,17.1	0
0.14,17.2	0
0.14,17.3	0

0.14,17.4	0
0.14,17.5	0
0.14,17.6	0
0.14,17.7	0
0.14,17.8	0
0.14,17.9	0
0.14,18.0	0
0.14,18.1	0
0.14,18.2	0
0.14,18.3	0
0.14,18.4	0
0.14,18.5	0
0.14,18.6	0
0.14,18.7	0
0.14,18.8	0
0.14,18.9	0
0.14,19.0	0
0.14,19.1	0
0.14,19.2	0
0.14,19.3	0
0.14,19.4	0
0.14,19.5	0
0.14,19.6	0
0.14,19.7	0
0.14,19.8	0
0.14,19.9	0
0.14,20.0	0
0.14,20.1	0
0.14,20.2	0
0.14,20.3	0
0.14,20.4	0
0.14,20.5	0
0.14,20.6	0
0.14,20.7	0
0.14,20.8	0
0.14,20.9	0
0.14,21.0	0
0.14,21.1	0
0.14,21.2	0
0.14,21.3	0
0.14,21.4	0
0.14,21.5	0

0.14,21.6	0
0.14,21.7	0
0.14,21.8	0
0.14,21.9	0
0.14,22.0	0
0.14,22.1	0
0.14,22.2	0
0.14,22.3	0
0.14,22.4	0
0.14,22.5	0
0.14,22.6	0
0.14,22.7	0
0.14,22.8	0
0.14,22.9	0
0.14,23.0	0
0.14,23.1	0
0.14,23.2	0
0.14,23.3	0
0.14,23.4	0
0.14,23.5	0
0.14,23.6	0
0.14,23.7	0
0.14,23.8	0
0.14,23.9	0
0.14,24.0	0
0.14,24.1	0
0.14,24.2	0
0.14,24.3	0
0.14,24.4	0
0.14,24.5	0
0.14,24.6	0
0.14,24.7	0
0.14,24.8	0
0.14,24.9	0
0.14,25.0	0
0.14,25.1	0
0.14,25.2	0
0.14,25.3	0
0.14,25.4	0
0.14,25.5	0
0.14,25.6	0
0.14,25.7	0

0.14,25.8	0
0.14,25.9	0
0.14,26.0	0
0.14,26.1	0
0.14,26.2	0
0.14,26.3	0
0.14,26.4	0
0.14,26.5	0
0.14,26.6	0
0.14,26.7	0
0.14,26.8	0
0.14,26.9	0
0.14,27.0	0
0.14,27.1	0
0.14,27.2	0
0.14,27.3	0
0.14,27.4	0
0.14,27.5	0
0.14,27.6	0
0.14,27.7	0
0.14,27.8	0
0.14,27.9	0
0.14,28.0	0
0.14,28.1	0
0.14,28.2	0
0.14,28.3	0
0.14,28.4	0
0.14,28.5	0
0.14,28.6	0
0.14,28.7	0
0.14,28.8	0
0.14,28.9	0
0.14,29.0	0
0.14,29.1	0
0.14,29.2	0
0.14,29.3	0
0.14,29.4	0
0.14,29.5	0
0.14,29.6	0
0.14,29.7	0
0.14,29.8	0
0.14,29.9	0

0.14,30.0	0
0.14,30.1	0
0.14,30.2	0
0.14,30.3	0
0.14,30.4	0
0.14,30.5	0
0.14,30.6	0
0.14,30.7	0
0.14,30.8	0
0.14,30.9	0
0.14,31.0	0
0.14,31.1	0
0.14,31.2	0
0.14,31.3	0
0.14,31.4	0
0.14,31.5	0
0.14,31.6	0
0.14,31.7	0
0.14,31.8	0
0.14,31.9	0
0.14,32.0	0
0.14,32.1	0
0.14,32.2	0
0.14,32.3	0
0.14,32.4	0
0.14,32.5	0
0.14,32.6	0
0.14,32.7	0
0.14,32.8	0
0.14,32.9	0
0.14,33.0	0
0.14,33.1	0
0.14,33.2	0
0.14,33.3	0
0.14,33.4	0
0.14,33.5	0
0.14,33.6	0
0.14,33.7	0
0.14,33.8	0
0.14,33.9	0
0.14,34.0	0
0.14,34.1	0

0.14,34.2	0
0.14,34.3	0
0.14,34.4	0
0.14,34.5	0
0.14,34.6	0
0.14,34.7	0
0.14,34.8	0
0.14,34.9	0
0.14,35.0	0
0.14,35.1	0
0.14,35.2	0
0.14,35.3	0
0.14,35.4	0
0.14,35.5	0
0.14,35.6	0
0.14,35.7	0
0.14,35.8	0
0.14,35.9	0
0.14,36.0	0
0.14,36.1	0
0.14,36.2	0
0.14,36.3	0
0.14,36.4	0
0.14,36.5	0
0.14,36.6	0
0.14,36.7	0
0.14,36.8	0
0.14,36.9	0
0.14,37.0	0
0.14,37.1	0
0.14,37.2	0
0.14,37.3	0
0.14,37.4	0
0.14,37.5	0
0.14,37.6	0
0.14,37.7	0
0.14,37.8	0
0.14,37.9	0
0.14,38.0	0
0.14,38.1	0
0.14,38.2	0
0.14,38.3	0

0.14,38.4	0
0.14,38.5	0
0.14,38.6	0
0.14,38.7	0
0.14,38.8	0
0.14,38.9	0
0.14,39.0	0
0.14,39.1	0
0.14,39.2	0
0.14,39.3	0
0.14,39.4	0
0.14,39.5	0
0.14,39.6	0
0.14,39.7	0
0.14,39.8	0
0.14,39.9	0
0.14,40.0	0
0.14,40.1	0
0.14,40.2	0
0.14,40.3	0
0.14,40.4	0
0.14,40.5	0
0.14,40.6	0
0.14,40.7	0
0.14,40.8	0
0.14,40.9	0
0.14,41.0	0
0.14,41.1	0
0.14,41.2	0
0.14,41.3	0
0.14,41.4	0
0.14,41.5	0
0.14,41.6	0
0.14,41.7	0
0.14,41.8	0
0.14,41.9	0
0.14,42.0	0
0.14,42.1	0
0.14,42.2	0
0.14,42.3	0
0.14,42.4	0
0.14,42.5	0

0.14,42.6	0
0.14,42.7	0
0.14,42.8	0
0.14,42.9	0
0.14,43.0	0
0.14,43.1	0
0.14,43.2	0
0.14,43.3	0
0.14,43.4	0
0.14,43.5	0
0.14,43.6	0
0.14,43.7	0
0.14,43.8	0
0.14,43.9	0
0.14,44.0	0
0.14,44.1	0
0.14,44.2	0
0.14,44.3	0
0.14,44.4	0
0.14,44.5	0
0.14,44.6	0
0.14,44.7	0
0.14,44.8	0
0.14,44.9	0
0.14,45.0	0
0.14,45.1	0
0.14,45.2	0
0.14,45.3	0
0.14,45.4	0
0.14,45.5	0
0.14,45.6	0
0.14,45.7	0
0.14,45.8	0
0.14,45.9	0
0.14,46.0	0
0.14,46.1	0
0.14,46.2	0
0.14,46.3	0
0.14,46.4	0
0.14,46.5	0
0.14,46.6	0
0.14,46.7	0

0.14,46.8	0
0.14,46.9	0
0.14,47.0	0
0.14,47.1	0
0.14,47.2	0
0.14,47.3	0
0.14,47.4	0
0.14,47.5	0
0.14,47.6	0
0.14,47.7	0
0.14,47.8	0
0.14,47.9	0
0.14,48.0	0
0.14,48.1	0
0.14,48.2	0
0.14,48.3	0
0.14,48.4	0
0.14,48.5	0
0.14,48.6	0
0.14,48.7	0
0.14,48.8	0
0.14,48.9	0
0.14,49.0	0
0.14,49.1	0
0.14,49.2	0
0.14,49.3	0
0.14,49.4	0
0.14,49.5	0
0.14,49.6	0
0.14,49.7	0
0.14,49.8	0
0.14,49.9	0
0.14,50.0	0
0.14,50.1	0
0.14,50.2	0
0.14,50.3	0
0.14,50.4	0
0.14,50.5	0
0.14,50.6	0
0.14,50.7	0
0.14,50.8	0
0.14,50.9	0

0.14,51.0	0
0.14,51.1	0
0.14,51.2	0
0.14,51.3	0
0.14,51.4	0
0.14,51.5	0
0.14,51.6	0
0.14,51.7	0
0.14,51.8	0
0.14,51.9	0
0.14,52.0	0
0.14,52.1	0
0.14,52.2	0
0.14,52.3	0
0.14,52.4	0
0.14,52.5	0
0.14,52.6	0
0.14,52.7	0
0.14,52.8	0
0.14,52.9	0
0.14,53.0	0
0.14,53.1	0
0.14,53.2	0
0.14,53.3	0
0.14,53.4	0
0.14,53.5	0
0.14,53.6	0
0.14,53.7	0
0.14,53.8	0
0.14,53.9	0
0.14,54.0	0
0.14,54.1	0
0.14,54.2	0
0.14,54.3	0
0.14,54.4	0
0.14,54.5	0
0.14,54.6	0
0.14,54.7	0
0.14,54.8	0
0.14,54.9	0
0.14,55.0	0
0.14,55.1	0

0.14,55.2	0
0.14,55.3	0
0.14,55.4	0
0.14,55.5	0
0.14,55.6	0
0.14,55.7	0
0.14,55.8	0
0.14,55.9	0
0.14,56.0	0
0.14,56.1	0
0.14,56.2	0
0.14,56.3	0
0.14,56.4	0
0.14,56.5	0
0.14,56.6	0
0.14,56.7	0
0.14,56.8	0
0.14,56.9	0
0.14,57.0	0
0.14,57.1	0
0.14,57.2	0
0.14,57.3	0
0.14,57.4	0
0.14,57.5	0
0.14,57.6	0
0.14,57.7	0
0.14,57.8	0
0.14,57.9	0
0.14,58.0	0
0.14,58.1	0
0.14,58.2	0
0.14,58.3	0
0.14,58.4	0
0.14,58.5	0
0.14,58.6	0
0.14,58.7	0
0.14,58.8	0
0.14,58.9	0
0.14,59.0	0
0.14,59.1	0
0.14,59.2	0
0.14,59.3	0

0.14,59.4	0
0.14,59.5	0
0.14,59.6	0
0.14,59.7	0
0.14,59.8	0
0.14,59.9	0
0.14,60.0	0
0.14,60.1	0
0.14,60.2	0
0.14,60.3	0
0.14,60.4	0
0.14,60.5	0
0.14,60.6	0
0.14,60.7	0
0.14,60.8	0
0.14,60.9	0
0.14,61.0	0
0.14,61.1	0
0.14,61.2	0
0.14,61.3	0
0.14,61.4	0
0.14,61.5	0
0.14,61.6	0
0.14,61.7	0
0.14,61.8	0
0.14,61.9	0
0.14,62.0	0
0.14,62.1	0
0.14,62.2	0
0.14,62.3	0
0.14,62.4	0
0.14,62.5	0
0.14,62.6	0
0.14,62.7	0
0.14,62.8	0
0.14,62.9	0
0.14,63.0	0
0.14,63.1	0
0.14,63.2	0
0.14,63.3	0
0.14,63.4	0
0.14,63.5	0

0.14,63.6	0
0.14,63.7	0
0.14,63.8	0
0.14,63.9	0
0.14,64.0	0
0.14,64.1	0
0.14,64.2	0
0.14,64.3	0
0.14,64.4	0
0.14,64.5	0
0.14,64.6	0
0.14,64.7	0
0.14,64.8	0
0.14,64.9	0
0.14,65.0	0
0.14,65.1	0
0.14,65.2	0
0.14,65.3	0
0.14,65.4	0
0.14,65.5	0
0.14,65.6	0
0.14,65.7	0
0.14,65.8	0
0.14,65.9	0
0.14,66.0	0
0.14,66.1	0
0.14,66.2	0
0.14,66.3	0
0.14,66.4	0
0.14,66.5	0
0.14,66.6	0
0.14,66.7	0
0.14,66.8	0
0.14,66.9	0
0.14,67.0	0
0.14,67.1	0
0.14,67.2	0
0.14,67.3	0
0.14,67.4	0
0.14,67.5	0
0.14,67.6	0
0.14,67.7	0

0.14,67.8	0
0.14,67.9	0
0.14,68.0	0
0.14,68.1	0
0.14,68.2	0
0.14,68.3	0
0.14,68.4	0
0.14,68.5	0
0.14,68.6	0
0.14,68.7	0
0.14,68.8	0
0.14,68.9	0
0.14,69.0	0
0.14,69.1	0
0.14,69.2	0
0.14,69.3	0
0.14,69.4	0
0.14,69.5	0
0.14,69.6	0
0.14,69.7	0
0.14,69.8	0
0.14,69.9	0
0.14,70.0	0
0.14,70.1	0
0.14,70.2	0
0.14,70.3	0
0.14,70.4	0
0.14,70.5	0
0.14,70.6	0
0.14,70.7	0
0.14,70.8	0
0.14,70.9	0
0.14,71.0	0
0.14,71.1	0
0.14,71.2	0
0.14,71.3	0
0.14,71.4	0
0.14,71.5	0
0.14,71.6	0
0.14,71.7	0
0.14,71.8	0
0.14,71.9	0

0.14,72.0	0
0.14,72.1	0
0.14,72.2	0
0.14,72.3	0
0.14,72.4	0
0.14,72.5	0
0.14,72.6	0
0.14,72.7	0
0.14,72.8	0
0.14,72.9	0
0.14,73.0	0
0.14,73.1	0
0.14,73.2	0
0.14,73.3	0
0.14,73.4	0
0.14,73.5	0
0.14,73.6	0
0.14,73.7	0
0.14,73.8	0
0.14,73.9	0
0.14,74.0	0
0.14,74.1	0
0.14,74.2	0
0.14,74.3	0
0.14,74.4	0
0.14,74.5	0
0.14,74.6	0
0.14,74.7	0
0.14,74.8	0
0.14,74.9	0
0.14,75.0	0
0.14,75.1	0
0.14,75.2	0
0.14,75.3	0
0.14,75.4	0
0.14,75.5	0
0.14,75.6	0
0.14,75.7	0
0.14,75.8	0
0.14,75.9	0
0.14,76.0	0
0.14,76.1	0

0.14,76.2	0
0.14,76.3	0
0.14,76.4	0
0.14,76.5	0
0.14,76.6	0
0.14,76.7	0
0.14,76.8	0
0.14,76.9	0
0.14,77.0	0
0.14,77.1	0
0.14,77.2	0
0.14,77.3	0
0.14,77.4	0
0.14,77.5	0
0.14,77.6	0
0.14,77.7	0
0.14,77.8	0
0.14,77.9	0
0.14,78.0	0
0.14,78.1	0
0.14,78.2	0
0.14,78.3	0
0.14,78.4	0
0.14,78.5	0
0.14,78.6	0
0.14,78.7	0
0.14,78.8	0
0.14,78.9	0
0.14,79.0	0
0.14,79.1	0
0.14,79.2	0
0.14,79.3	0
0.14,79.4	0
0.14,79.5	0
0.14,79.6	0
0.14,79.7	0
0.14,79.8	0
0.14,79.9	0
0.14,80.0	0
0.14,80.1	0
0.14,80.2	0
0.14,80.3	0

0.14,80.4	0
0.14,80.5	0
0.14,80.6	0
0.14,80.7	0
0.14,80.8	0
0.14,80.9	0
0.14,81.0	0
0.14,81.1	0
0.14,81.2	0
0.14,81.3	0
0.14,81.4	0
0.14,81.5	0
0.14,81.6	0
0.14,81.7	0
0.14,81.8	0
0.14,81.9	0
0.14,82.0	0
0.14,82.1	0
0.14,82.2	0
0.14,82.3	0
0.14,82.4	0
0.14,82.5	0
0.14,82.6	0
0.14,82.7	0
0.14,82.8	0
0.14,82.9	0
0.14,83.0	0
0.14,83.1	0
0.14,83.2	0
0.14,83.3	0
0.14,83.4	0
0.14,83.5	0
0.14,83.6	0
0.14,83.7	0
0.14,83.8	0
0.14,83.9	0
0.14,84.0	0
0.14,84.1	0
0.14,84.2	0
0.14,84.3	0
0.14,84.4	0
0.14,84.5	0

0.14,84.6	0
0.14,84.7	0
0.14,84.8	0
0.14,84.9	0
0.14,85.0	0
0.14,85.1	0
0.14,85.2	0
0.14,85.3	0
0.14,85.4	0
0.14,85.5	0
0.14,85.6	0
0.14,85.7	0
0.14,85.8	0
0.14,85.9	0
0.14,86.0	0
0.14,86.1	0
0.14,86.2	0
0.14,86.3	0
0.14,86.4	0
0.14,86.5	0
0.14,86.6	0
0.14,86.7	0
0.14,86.8	0
0.14,86.9	0
0.14,87.0	0
0.14,87.1	0
0.14,87.2	0
0.14,87.3	0
0.14,87.4	0
0.14,87.5	0
0.14,87.6	0
0.14,87.7	0
0.14,87.8	0
0.14,87.9	0
0.14,88.0	0
0.14,88.1	0
0.14,88.2	0
0.14,88.3	0
0.14,88.4	0
0.14,88.5	0
0.14,88.6	0
0.14,88.7	0

0.14,88.8	0
0.14,88.9	0
0.14,89.0	0
0.14,89.1	0
0.14,89.2	0
0.14,89.3	0
0.14,89.4	0
0.14,89.5	0
0.14,89.6	0
0.14,89.7	0
0.14,89.8	0
0.14,89.9	0
0.14,90.0	0
0.14,90.1	0
0.14,90.2	0
0.14,90.3	0
0.14,90.4	0
0.14,90.5	0
0.14,90.6	0
0.14,90.7	0
0.14,90.8	0
0.14,90.9	0
0.14,91.0	0
0.14,91.1	0
0.14,91.2	0
0.14,91.3	0
0.14,91.4	0
0.14,91.5	0
0.14,91.6	0
0.14,91.7	0
0.14,91.8	0
0.14,91.9	0
0.14,92.0	0
0.14,92.1	0
0.14,92.2	0
0.14,92.3	0
0.14,92.4	0
0.14,92.5	0
0.14,92.6	0
0.14,92.7	0
0.14,92.8	0
0.14,92.9	0

0.14,93.0	0
0.14,93.1	0
0.14,93.2	0
0.14,93.3	0
0.14,93.4	0
0.14,93.5	0
0.14,93.6	0
0.14,93.7	0
0.14,93.8	0
0.14,93.9	0
0.14,94.0	0
0.14,94.1	0
0.14,94.2	0
0.14,94.3	0
0.14,94.4	0
0.14,94.5	0
0.14,94.6	0
0.14,94.7	0
0.14,94.8	0
0.14,94.9	0
0.14,95.0	0
0.14,95.1	0
0.14,95.2	0
0.14,95.3	0
0.14,95.4	0
0.14,95.5	0
0.14,95.6	0
0.14,95.7	0
0.14,95.8	0
0.14,95.9	0
0.14,96.0	0
0.14,96.1	0
0.14,96.2	0
0.14,96.3	0
0.14,96.4	0
0.14,96.5	0
0.14,96.6	0
0.14,96.7	0
0.14,96.8	0
0.14,96.9	0
0.14,97.0	0
0.14,97.1	0

0.14,97.2	0
0.14,97.3	0
0.14,97.4	0
0.14,97.5	0
0.14,97.6	0
0.14,97.7	0
0.14,97.8	0
0.14,97.9	0
0.14,98.0	0
0.14,98.1	0
0.14,98.2	0
0.14,98.3	0
0.14,98.4	0
0.14,98.5	0
0.14,98.6	0
0.14,98.7	0
0.14,98.8	0
0.14,98.9	0
0.14,99.0	0
0.14,99.1	0
0.14,99.2	0
0.14,99.3	0
0.14,99.4	0
0.14,99.5	0
0.14,99.6	0
0.14,99.7	0
0.14,99.8	0
0.14,99.9	0
0.14,100.0	0
0.15,0.0	0
0.15,0.1	0
0.15,0.2	0
0.15,0.3	0
0.15,0.4	0
0.15,0.5	0
0.15,0.6	0
0.15,0.7	0
0.15,0.8	0
0.15,0.9	0
0.15,1.0	0
0.15,1.1	0
0.15,1.2	0

0.15,1.3	0
0.15,1.4	0
0.15,1.5	0
0.15,1.6	0
0.15,1.7	0
0.15,1.8	0
0.15,1.9	0
0.15,2.0	0
0.15,2.1	0
0.15,2.2	0
0.15,2.3	0
0.15,2.4	0
0.15,2.5	0
0.15,2.6	0
0.15,2.7	0
0.15,2.8	0
0.15,2.9	0
0.15,3.0	0
0.15,3.1	0
0.15,3.2	0
0.15,3.3	0
0.15,3.4	0
0.15,3.5	0
0.15,3.6	0
0.15,3.7	0
0.15,3.8	0
0.15,3.9	0
0.15,4.0	0
0.15,4.1	0
0.15,4.2	0
0.15,4.3	0
0.15,4.4	0
0.15,4.5	0
0.15,4.6	0
0.15,4.7	0
0.15,4.8	0
0.15,4.9	0
0.15,5.0	0
0.15,5.1	0
0.15,5.2	0
0.15,5.3	0
0.15,5.4	0

0.15,5.5	0
0.15,5.6	0
0.15,5.7	0
0.15,5.8	0
0.15,5.9	0
0.15,6.0	0
0.15,6.1	0
0.15,6.2	0
0.15,6.3	0
0.15,6.4	0
0.15,6.5	0
0.15,6.6	0
0.15,6.7	0
0.15,6.8	0
0.15,6.9	0
0.15,7.0	0
0.15,7.1	0
0.15,7.2	0
0.15,7.3	0
0.15,7.4	0
0.15,7.5	0
0.15,7.6	0
0.15,7.7	0
0.15,7.8	0
0.15,7.9	0
0.15,8.0	0
0.15,8.1	0
0.15,8.2	0
0.15,8.3	0
0.15,8.4	0
0.15,8.5	0
0.15,8.6	0
0.15,8.7	0
0.15,8.8	0
0.15,8.9	0
0.15,9.0	0
0.15,9.1	0
0.15,9.2	0
0.15,9.3	0
0.15,9.4	0
0.15,9.5	0
0.15,9.6	0

0.15,9.7	0
0.15,9.8	0
0.15,9.9	0
0.15,10.0	0
0.15,10.1	0
0.15,10.2	0
0.15,10.3	0
0.15,10.4	0
0.15,10.5	0
0.15,10.6	0
0.15,10.7	0
0.15,10.8	0
0.15,10.9	0
0.15,11.0	0
0.15,11.1	0
0.15,11.2	0
0.15,11.3	0
0.15,11.4	0
0.15,11.5	0
0.15,11.6	0
0.15,11.7	0
0.15,11.8	0
0.15,11.9	0
0.15,12.0	0
0.15,12.1	0
0.15,12.2	0
0.15,12.3	0
0.15,12.4	0
0.15,12.5	0
0.15,12.6	0
0.15,12.7	0
0.15,12.8	0
0.15,12.9	0
0.15,13.0	0
0.15,13.1	0
0.15,13.2	0
0.15,13.3	0
0.15,13.4	0
0.15,13.5	0
0.15,13.6	0
0.15,13.7	0
0.15,13.8	0

0.15,13.9	0
0.15,14.0	0
0.15,14.1	0
0.15,14.2	0
0.15,14.3	0
0.15,14.4	0
0.15,14.5	0
0.15,14.6	0
0.15,14.7	0
0.15,14.8	0
0.15,14.9	0
0.15,15.0	0
0.15,15.1	0
0.15,15.2	0
0.15,15.3	0
0.15,15.4	0
0.15,15.5	0
0.15,15.6	0
0.15,15.7	0
0.15,15.8	0
0.15,15.9	0
0.15,16.0	0
0.15,16.1	0
0.15,16.2	0
0.15,16.3	0
0.15,16.4	0
0.15,16.5	0
0.15,16.6	0
0.15,16.7	0
0.15,16.8	0
0.15,16.9	0
0.15,17.0	0
0.15,17.1	0
0.15,17.2	0
0.15,17.3	0
0.15,17.4	0
0.15,17.5	0
0.15,17.6	0
0.15,17.7	0
0.15,17.8	0
0.15,17.9	0
0.15,18.0	0

0.15,18.1	0
0.15,18.2	0
0.15,18.3	0
0.15,18.4	0
0.15,18.5	0
0.15,18.6	0
0.15,18.7	0
0.15,18.8	0
0.15,18.9	0
0.15,19.0	0
0.15,19.1	0
0.15,19.2	0
0.15,19.3	0
0.15,19.4	0
0.15,19.5	0
0.15,19.6	0
0.15,19.7	0
0.15,19.8	0
0.15,19.9	0
0.15,20.0	0
0.15,20.1	0
0.15,20.2	0
0.15,20.3	0
0.15,20.4	0
0.15,20.5	0
0.15,20.6	0
0.15,20.7	0
0.15,20.8	0
0.15,20.9	0
0.15,21.0	0
0.15,21.1	0
0.15,21.2	0
0.15,21.3	0
0.15,21.4	0
0.15,21.5	0
0.15,21.6	0
0.15,21.7	0
0.15,21.8	0
0.15,21.9	0
0.15,22.0	0
0.15,22.1	0
0.15,22.2	0

0.15,22.3	0
0.15,22.4	0
0.15,22.5	0
0.15,22.6	0
0.15,22.7	0
0.15,22.8	0
0.15,22.9	0
0.15,23.0	0
0.15,23.1	0
0.15,23.2	0
0.15,23.3	0
0.15,23.4	0
0.15,23.5	0
0.15,23.6	0
0.15,23.7	0
0.15,23.8	0
0.15,23.9	0
0.15,24.0	0
0.15,24.1	0
0.15,24.2	0
0.15,24.3	0
0.15,24.4	0
0.15,24.5	0
0.15,24.6	0
0.15,24.7	0
0.15,24.8	0
0.15,24.9	0
0.15,25.0	0
0.15,25.1	0
0.15,25.2	0
0.15,25.3	0
0.15,25.4	0
0.15,25.5	0
0.15,25.6	0
0.15,25.7	0
0.15,25.8	0
0.15,25.9	0
0.15,26.0	0
0.15,26.1	0
0.15,26.2	0
0.15,26.3	0
0.15,26.4	0

0.15,26.5	0
0.15,26.6	0
0.15,26.7	0
0.15,26.8	0
0.15,26.9	0
0.15,27.0	0
0.15,27.1	0
0.15,27.2	0
0.15,27.3	0
0.15,27.4	0
0.15,27.5	0
0.15,27.6	0
0.15,27.7	0
0.15,27.8	0
0.15,27.9	0
0.15,28.0	0
0.15,28.1	0
0.15,28.2	0
0.15,28.3	0
0.15,28.4	0
0.15,28.5	0
0.15,28.6	0
0.15,28.7	0
0.15,28.8	0
0.15,28.9	0
0.15,29.0	0
0.15,29.1	0
0.15,29.2	0
0.15,29.3	0
0.15,29.4	0
0.15,29.5	0
0.15,29.6	0
0.15,29.7	0
0.15,29.8	0
0.15,29.9	0
0.15,30.0	0
0.15,30.1	0
0.15,30.2	0
0.15,30.3	0
0.15,30.4	0
0.15,30.5	0
0.15,30.6	0

0.15,30.7	0
0.15,30.8	0
0.15,30.9	0
0.15,31.0	0
0.15,31.1	0
0.15,31.2	0
0.15,31.3	0
0.15,31.4	0
0.15,31.5	0
0.15,31.6	0
0.15,31.7	0
0.15,31.8	0
0.15,31.9	0
0.15,32.0	0
0.15,32.1	0
0.15,32.2	0
0.15,32.3	0
0.15,32.4	0
0.15,32.5	0
0.15,32.6	0
0.15,32.7	0
0.15,32.8	0
0.15,32.9	0
0.15,33.0	0
0.15,33.1	0
0.15,33.2	0
0.15,33.3	0
0.15,33.4	0
0.15,33.5	0
0.15,33.6	0
0.15,33.7	0
0.15,33.8	0
0.15,33.9	0
0.15,34.0	0
0.15,34.1	0
0.15,34.2	0
0.15,34.3	0
0.15,34.4	0
0.15,34.5	0
0.15,34.6	0
0.15,34.7	0
0.15,34.8	0

0.15,34.9	0
0.15,35.0	0
0.15,35.1	0
0.15,35.2	0
0.15,35.3	0
0.15,35.4	0
0.15,35.5	0
0.15,35.6	0
0.15,35.7	0
0.15,35.8	0
0.15,35.9	0
0.15,36.0	0
0.15,36.1	0
0.15,36.2	0
0.15,36.3	0
0.15,36.4	0
0.15,36.5	0
0.15,36.6	0
0.15,36.7	0
0.15,36.8	0
0.15,36.9	0
0.15,37.0	0
0.15,37.1	0
0.15,37.2	0
0.15,37.3	0
0.15,37.4	0
0.15,37.5	0
0.15,37.6	0
0.15,37.7	0
0.15,37.8	0
0.15,37.9	0
0.15,38.0	0
0.15,38.1	0
0.15,38.2	0
0.15,38.3	0
0.15,38.4	0
0.15,38.5	0
0.15,38.6	0
0.15,38.7	0
0.15,38.8	0
0.15,38.9	0
0.15,39.0	0

0.15,39.1	0
0.15,39.2	0
0.15,39.3	0
0.15,39.4	0
0.15,39.5	0
0.15,39.6	0
0.15,39.7	0
0.15,39.8	0
0.15,39.9	0
0.15,40.0	0
0.15,40.1	0
0.15,40.2	0
0.15,40.3	0
0.15,40.4	0
0.15,40.5	0
0.15,40.6	0
0.15,40.7	0
0.15,40.8	0
0.15,40.9	0
0.15,41.0	0
0.15,41.1	0
0.15,41.2	0
0.15,41.3	0
0.15,41.4	0
0.15,41.5	0
0.15,41.6	0
0.15,41.7	0
0.15,41.8	0
0.15,41.9	0
0.15,42.0	0
0.15,42.1	0
0.15,42.2	0
0.15,42.3	0
0.15,42.4	0
0.15,42.5	0
0.15,42.6	0
0.15,42.7	0
0.15,42.8	0
0.15,42.9	0
0.15,43.0	0
0.15,43.1	0
0.15,43.2	0

0.15,43.3	0
0.15,43.4	0
0.15,43.5	0
0.15,43.6	0
0.15,43.7	0
0.15,43.8	0
0.15,43.9	0
0.15,44.0	0
0.15,44.1	0
0.15,44.2	0
0.15,44.3	0
0.15,44.4	0
0.15,44.5	0
0.15,44.6	0
0.15,44.7	0
0.15,44.8	0
0.15,44.9	0
0.15,45.0	0
0.15,45.1	0
0.15,45.2	0
0.15,45.3	0
0.15,45.4	0
0.15,45.5	0
0.15,45.6	0
0.15,45.7	0
0.15,45.8	0
0.15,45.9	0
0.15,46.0	0
0.15,46.1	0
0.15,46.2	0
0.15,46.3	0
0.15,46.4	0
0.15,46.5	0
0.15,46.6	0
0.15,46.7	0
0.15,46.8	0
0.15,46.9	0
0.15,47.0	0
0.15,47.1	0
0.15,47.2	0
0.15,47.3	0
0.15,47.4	0

0.15,47.5	0
0.15,47.6	0
0.15,47.7	0
0.15,47.8	0
0.15,47.9	0
0.15,48.0	0
0.15,48.1	0
0.15,48.2	0
0.15,48.3	0
0.15,48.4	0
0.15,48.5	0
0.15,48.6	0
0.15,48.7	0
0.15,48.8	0
0.15,48.9	0
0.15,49.0	0
0.15,49.1	0
0.15,49.2	0
0.15,49.3	0
0.15,49.4	0
0.15,49.5	0
0.15,49.6	0
0.15,49.7	0
0.15,49.8	0
0.15,49.9	0
0.15,50.0	0
0.15,50.1	0
0.15,50.2	0
0.15,50.3	0
0.15,50.4	0
0.15,50.5	0
0.15,50.6	0
0.15,50.7	0
0.15,50.8	0
0.15,50.9	0
0.15,51.0	0
0.15,51.1	0
0.15,51.2	0
0.15,51.3	0
0.15,51.4	0
0.15,51.5	0
0.15,51.6	0

0.15,51.7	0
0.15,51.8	0
0.15,51.9	0
0.15,52.0	0
0.15,52.1	0
0.15,52.2	0
0.15,52.3	0
0.15,52.4	0
0.15,52.5	0
0.15,52.6	0
0.15,52.7	0
0.15,52.8	0
0.15,52.9	0
0.15,53.0	0
0.15,53.1	0
0.15,53.2	0
0.15,53.3	0
0.15,53.4	0
0.15,53.5	0
0.15,53.6	0
0.15,53.7	0
0.15,53.8	0
0.15,53.9	0
0.15,54.0	0
0.15,54.1	0
0.15,54.2	0
0.15,54.3	0
0.15,54.4	0
0.15,54.5	0
0.15,54.6	0
0.15,54.7	0
0.15,54.8	0
0.15,54.9	0
0.15,55.0	0
0.15,55.1	0
0.15,55.2	0
0.15,55.3	0
0.15,55.4	0
0.15,55.5	0
0.15,55.6	0
0.15,55.7	0
0.15,55.8	0

0.15,55.9	0
0.15,56.0	0
0.15,56.1	0
0.15,56.2	0
0.15,56.3	0
0.15,56.4	0
0.15,56.5	0
0.15,56.6	0
0.15,56.7	0
0.15,56.8	0
0.15,56.9	0
0.15,57.0	0
0.15,57.1	0
0.15,57.2	0
0.15,57.3	0
0.15,57.4	0
0.15,57.5	0
0.15,57.6	0
0.15,57.7	0
0.15,57.8	0
0.15,57.9	0
0.15,58.0	0
0.15,58.1	0
0.15,58.2	0
0.15,58.3	0
0.15,58.4	0
0.15,58.5	0
0.15,58.6	0
0.15,58.7	0
0.15,58.8	0
0.15,58.9	0
0.15,59.0	0
0.15,59.1	0
0.15,59.2	0
0.15,59.3	0
0.15,59.4	0
0.15,59.5	0
0.15,59.6	0
0.15,59.7	0
0.15,59.8	0
0.15,59.9	0
0.15,60.0	0

0.15,60.1	0
0.15,60.2	0
0.15,60.3	0
0.15,60.4	0
0.15,60.5	0
0.15,60.6	0
0.15,60.7	0
0.15,60.8	0
0.15,60.9	0
0.15,61.0	0
0.15,61.1	0
0.15,61.2	0
0.15,61.3	0
0.15,61.4	0
0.15,61.5	0
0.15,61.6	0
0.15,61.7	0
0.15,61.8	0
0.15,61.9	0
0.15,62.0	0
0.15,62.1	0
0.15,62.2	0
0.15,62.3	0
0.15,62.4	0
0.15,62.5	0
0.15,62.6	0
0.15,62.7	0
0.15,62.8	0
0.15,62.9	0
0.15,63.0	0
0.15,63.1	0
0.15,63.2	0
0.15,63.3	0
0.15,63.4	0
0.15,63.5	0
0.15,63.6	0
0.15,63.7	0
0.15,63.8	0
0.15,63.9	0
0.15,64.0	0
0.15,64.1	0
0.15,64.2	0

0.15,64.3	0
0.15,64.4	0
0.15,64.5	0
0.15,64.6	0
0.15,64.7	0
0.15,64.8	0
0.15,64.9	0
0.15,65.0	0
0.15,65.1	0
0.15,65.2	0
0.15,65.3	0
0.15,65.4	0
0.15,65.5	0
0.15,65.6	0
0.15,65.7	0
0.15,65.8	0
0.15,65.9	0
0.15,66.0	0
0.15,66.1	0
0.15,66.2	0
0.15,66.3	0
0.15,66.4	0
0.15,66.5	0
0.15,66.6	0
0.15,66.7	0
0.15,66.8	0
0.15,66.9	0
0.15,67.0	0
0.15,67.1	0
0.15,67.2	0
0.15,67.3	0
0.15,67.4	0
0.15,67.5	0
0.15,67.6	0
0.15,67.7	0
0.15,67.8	0
0.15,67.9	0
0.15,68.0	0
0.15,68.1	0
0.15,68.2	0
0.15,68.3	0
0.15,68.4	0

0.15,68.5	0
0.15,68.6	0
0.15,68.7	0
0.15,68.8	0
0.15,68.9	0
0.15,69.0	0
0.15,69.1	0
0.15,69.2	0
0.15,69.3	0
0.15,69.4	0
0.15,69.5	0
0.15,69.6	0
0.15,69.7	0
0.15,69.8	0
0.15,69.9	0
0.15,70.0	0
0.15,70.1	0
0.15,70.2	0
0.15,70.3	0
0.15,70.4	0
0.15,70.5	0
0.15,70.6	0
0.15,70.7	0
0.15,70.8	0
0.15,70.9	0
0.15,71.0	0
0.15,71.1	0
0.15,71.2	0
0.15,71.3	0
0.15,71.4	0
0.15,71.5	0
0.15,71.6	0
0.15,71.7	0
0.15,71.8	0
0.15,71.9	0
0.15,72.0	0
0.15,72.1	0
0.15,72.2	0
0.15,72.3	0
0.15,72.4	0
0.15,72.5	0
0.15,72.6	0

0.15,72.7	0
0.15,72.8	0
0.15,72.9	0
0.15,73.0	0
0.15,73.1	0
0.15,73.2	0
0.15,73.3	0
0.15,73.4	0
0.15,73.5	0
0.15,73.6	0
0.15,73.7	0
0.15,73.8	0
0.15,73.9	0
0.15,74.0	0
0.15,74.1	0
0.15,74.2	0
0.15,74.3	0
0.15,74.4	0
0.15,74.5	0
0.15,74.6	0
0.15,74.7	0
0.15,74.8	0
0.15,74.9	0
0.15,75.0	0
0.15,75.1	0
0.15,75.2	0
0.15,75.3	0
0.15,75.4	0
0.15,75.5	0
0.15,75.6	0
0.15,75.7	0
0.15,75.8	0
0.15,75.9	0
0.15,76.0	0
0.15,76.1	0
0.15,76.2	0
0.15,76.3	0
0.15,76.4	0
0.15,76.5	0
0.15,76.6	0
0.15,76.7	0
0.15,76.8	0

0.15,76.9	0
0.15,77.0	0
0.15,77.1	0
0.15,77.2	0
0.15,77.3	0
0.15,77.4	0
0.15,77.5	0
0.15,77.6	0
0.15,77.7	0
0.15,77.8	0
0.15,77.9	0
0.15,78.0	0
0.15,78.1	0
0.15,78.2	0
0.15,78.3	0
0.15,78.4	0
0.15,78.5	0
0.15,78.6	0
0.15,78.7	0
0.15,78.8	0
0.15,78.9	0
0.15,79.0	0
0.15,79.1	0
0.15,79.2	0
0.15,79.3	0
0.15,79.4	0
0.15,79.5	0
0.15,79.6	0
0.15,79.7	0
0.15,79.8	0
0.15,79.9	0
0.15,80.0	0
0.15,80.1	0
0.15,80.2	0
0.15,80.3	0
0.15,80.4	0
0.15,80.5	0
0.15,80.6	0
0.15,80.7	0
0.15,80.8	0
0.15,80.9	0
0.15,81.0	0

0.15,81.1	0
0.15,81.2	0
0.15,81.3	0
0.15,81.4	0
0.15,81.5	0
0.15,81.6	0
0.15,81.7	0
0.15,81.8	0
0.15,81.9	0
0.15,82.0	0
0.15,82.1	0
0.15,82.2	0
0.15,82.3	0
0.15,82.4	0
0.15,82.5	0
0.15,82.6	0
0.15,82.7	0
0.15,82.8	0
0.15,82.9	0
0.15,83.0	0
0.15,83.1	0
0.15,83.2	0
0.15,83.3	0
0.15,83.4	0
0.15,83.5	0
0.15,83.6	0
0.15,83.7	0
0.15,83.8	0
0.15,83.9	0
0.15,84.0	0
0.15,84.1	0
0.15,84.2	0
0.15,84.3	0
0.15,84.4	0
0.15,84.5	0
0.15,84.6	0
0.15,84.7	0
0.15,84.8	0
0.15,84.9	0
0.15,85.0	0
0.15,85.1	0
0.15,85.2	0

0.15,85.3	0
0.15,85.4	0
0.15,85.5	0
0.15,85.6	0
0.15,85.7	0
0.15,85.8	0
0.15,85.9	0
0.15,86.0	0
0.15,86.1	0
0.15,86.2	0
0.15,86.3	0
0.15,86.4	0
0.15,86.5	0
0.15,86.6	0
0.15,86.7	0
0.15,86.8	0
0.15,86.9	0
0.15,87.0	0
0.15,87.1	0
0.15,87.2	0
0.15,87.3	0
0.15,87.4	0
0.15,87.5	0
0.15,87.6	0
0.15,87.7	0
0.15,87.8	0
0.15,87.9	0
0.15,88.0	0
0.15,88.1	0
0.15,88.2	0
0.15,88.3	0
0.15,88.4	0
0.15,88.5	0
0.15,88.6	0
0.15,88.7	0
0.15,88.8	0
0.15,88.9	0
0.15,89.0	0
0.15,89.1	0
0.15,89.2	0
0.15,89.3	0
0.15,89.4	0

0.15,89.5	0
0.15,89.6	0
0.15,89.7	0
0.15,89.8	0
0.15,89.9	0
0.15,90.0	0
0.15,90.1	0
0.15,90.2	0
0.15,90.3	0
0.15,90.4	0
0.15,90.5	0
0.15,90.6	0
0.15,90.7	0
0.15,90.8	0
0.15,90.9	0
0.15,91.0	0
0.15,91.1	0
0.15,91.2	0
0.15,91.3	0
0.15,91.4	0
0.15,91.5	0
0.15,91.6	0
0.15,91.7	0
0.15,91.8	0
0.15,91.9	0
0.15,92.0	0
0.15,92.1	0
0.15,92.2	0
0.15,92.3	0
0.15,92.4	0
0.15,92.5	0
0.15,92.6	0
0.15,92.7	0
0.15,92.8	0
0.15,92.9	0
0.15,93.0	0
0.15,93.1	0
0.15,93.2	0
0.15,93.3	0
0.15,93.4	0
0.15,93.5	0
0.15,93.6	0

0.15,93.7	0
0.15,93.8	0
0.15,93.9	0
0.15,94.0	0
0.15,94.1	0
0.15,94.2	0
0.15,94.3	0
0.15,94.4	0
0.15,94.5	0
0.15,94.6	0
0.15,94.7	0
0.15,94.8	0
0.15,94.9	0
0.15,95.0	0
0.15,95.1	0
0.15,95.2	0
0.15,95.3	0
0.15,95.4	0
0.15,95.5	0
0.15,95.6	0
0.15,95.7	0
0.15,95.8	0
0.15,95.9	0
0.15,96.0	0
0.15,96.1	0
0.15,96.2	0
0.15,96.3	0
0.15,96.4	0
0.15,96.5	0
0.15,96.6	0
0.15,96.7	0
0.15,96.8	0
0.15,96.9	0
0.15,97.0	0
0.15,97.1	0
0.15,97.2	0
0.15,97.3	0
0.15,97.4	0
0.15,97.5	0
0.15,97.6	0
0.15,97.7	0
0.15,97.8	0

0.15,97.9	0
0.15,98.0	0
0.15,98.1	0
0.15,98.2	0
0.15,98.3	0
0.15,98.4	0
0.15,98.5	0
0.15,98.6	0
0.15,98.7	0
0.15,98.8	0
0.15,98.9	0
0.15,99.0	0
0.15,99.1	0
0.15,99.2	0
0.15,99.3	0
0.15,99.4	0
0.15,99.5	0
0.15,99.6	0
0.15,99.7	0
0.15,99.8	0
0.15,99.9	0
0.15,100.0	0
0.16,0.0	0
0.16,0.1	0
0.16,0.2	0
0.16,0.3	0
0.16,0.4	0
0.16,0.5	0
0.16,0.6	0
0.16,0.7	0
0.16,0.8	0
0.16,0.9	0
0.16,1.0	0
0.16,1.1	0
0.16,1.2	0
0.16,1.3	0
0.16,1.4	0
0.16,1.5	0
0.16,1.6	0
0.16,1.7	0
0.16,1.8	0
0.16,1.9	0

0.16,2.0	0
0.16,2.1	0
0.16,2.2	0
0.16,2.3	0
0.16,2.4	0
0.16,2.5	0
0.16,2.6	0
0.16,2.7	0
0.16,2.8	0
0.16,2.9	0
0.16,3.0	0
0.16,3.1	0
0.16,3.2	0
0.16,3.3	0
0.16,3.4	0
0.16,3.5	0
0.16,3.6	0
0.16,3.7	0
0.16,3.8	0
0.16,3.9	0
0.16,4.0	0
0.16,4.1	0
0.16,4.2	0
0.16,4.3	0
0.16,4.4	0
0.16,4.5	0
0.16,4.6	0
0.16,4.7	0
0.16,4.8	0
0.16,4.9	0
0.16,5.0	0
0.16,5.1	0
0.16,5.2	0
0.16,5.3	0
0.16,5.4	0
0.16,5.5	0
0.16,5.6	0
0.16,5.7	0
0.16,5.8	0
0.16,5.9	0
0.16,6.0	0
0.16,6.1	0

0.16,6.2	0
0.16,6.3	0
0.16,6.4	0
0.16,6.5	0
0.16,6.6	0
0.16,6.7	0
0.16,6.8	0
0.16,6.9	0
0.16,7.0	0
0.16,7.1	0
0.16,7.2	0
0.16,7.3	0
0.16,7.4	0
0.16,7.5	0
0.16,7.6	0
0.16,7.7	0
0.16,7.8	0
0.16,7.9	0
0.16,8.0	0
0.16,8.1	0
0.16,8.2	0
0.16,8.3	0
0.16,8.4	0
0.16,8.5	0
0.16,8.6	0
0.16,8.7	0
0.16,8.8	0
0.16,8.9	0
0.16,9.0	0
0.16,9.1	0
0.16,9.2	0
0.16,9.3	0
0.16,9.4	0
0.16,9.5	0
0.16,9.6	0
0.16,9.7	0
0.16,9.8	0
0.16,9.9	0
0.16,10.0	0
0.16,10.1	0
0.16,10.2	0
0.16,10.3	0

0.16,10.4	0
0.16,10.5	0
0.16,10.6	0
0.16,10.7	0
0.16,10.8	0
0.16,10.9	0
0.16,11.0	0
0.16,11.1	0
0.16,11.2	0
0.16,11.3	0
0.16,11.4	0
0.16,11.5	0
0.16,11.6	0
0.16,11.7	0
0.16,11.8	0
0.16,11.9	0
0.16,12.0	0
0.16,12.1	0
0.16,12.2	0
0.16,12.3	0
0.16,12.4	0
0.16,12.5	0
0.16,12.6	0
0.16,12.7	0
0.16,12.8	0
0.16,12.9	0
0.16,13.0	0
0.16,13.1	0
0.16,13.2	0
0.16,13.3	0
0.16,13.4	0
0.16,13.5	0
0.16,13.6	0
0.16,13.7	0
0.16,13.8	0
0.16,13.9	0
0.16,14.0	0
0.16,14.1	0
0.16,14.2	0
0.16,14.3	0
0.16,14.4	0
0.16,14.5	0

0.16,14.6	0
0.16,14.7	0
0.16,14.8	0
0.16,14.9	0
0.16,15.0	0
0.16,15.1	0
0.16,15.2	0
0.16,15.3	0
0.16,15.4	0
0.16,15.5	0
0.16,15.6	0
0.16,15.7	0
0.16,15.8	0
0.16,15.9	0
0.16,16.0	0
0.16,16.1	0
0.16,16.2	0
0.16,16.3	0
0.16,16.4	0
0.16,16.5	0
0.16,16.6	0
0.16,16.7	0
0.16,16.8	0
0.16,16.9	0
0.16,17.0	0
0.16,17.1	0
0.16,17.2	0
0.16,17.3	0
0.16,17.4	0
0.16,17.5	0
0.16,17.6	0
0.16,17.7	0
0.16,17.8	0
0.16,17.9	0
0.16,18.0	0
0.16,18.1	0
0.16,18.2	0
0.16,18.3	0
0.16,18.4	0
0.16,18.5	0
0.16,18.6	0
0.16,18.7	0

0.16,18.8	0
0.16,18.9	0
0.16,19.0	0
0.16,19.1	0
0.16,19.2	0
0.16,19.3	0
0.16,19.4	0
0.16,19.5	0
0.16,19.6	0
0.16,19.7	0
0.16,19.8	0
0.16,19.9	0
0.16,20.0	0
0.16,20.1	0
0.16,20.2	0
0.16,20.3	0
0.16,20.4	0
0.16,20.5	0
0.16,20.6	0
0.16,20.7	0
0.16,20.8	0
0.16,20.9	0
0.16,21.0	0
0.16,21.1	0
0.16,21.2	0
0.16,21.3	0
0.16,21.4	0
0.16,21.5	0
0.16,21.6	0
0.16,21.7	0
0.16,21.8	0
0.16,21.9	0
0.16,22.0	0
0.16,22.1	0
0.16,22.2	0
0.16,22.3	0
0.16,22.4	0
0.16,22.5	0
0.16,22.6	0
0.16,22.7	0
0.16,22.8	0
0.16,22.9	0

0.16,23.0	0
0.16,23.1	0
0.16,23.2	0
0.16,23.3	0
0.16,23.4	0
0.16,23.5	0
0.16,23.6	0
0.16,23.7	0
0.16,23.8	0
0.16,23.9	0
0.16,24.0	0
0.16,24.1	0
0.16,24.2	0
0.16,24.3	0
0.16,24.4	0
0.16,24.5	0
0.16,24.6	0
0.16,24.7	0
0.16,24.8	0
0.16,24.9	0
0.16,25.0	0
0.16,25.1	0
0.16,25.2	0
0.16,25.3	0
0.16,25.4	0
0.16,25.5	0
0.16,25.6	0
0.16,25.7	0
0.16,25.8	0
0.16,25.9	0
0.16,26.0	0
0.16,26.1	0
0.16,26.2	0
0.16,26.3	0
0.16,26.4	0
0.16,26.5	0
0.16,26.6	0
0.16,26.7	0
0.16,26.8	0
0.16,26.9	0
0.16,27.0	0
0.16,27.1	0

0.16,27.2	0
0.16,27.3	0
0.16,27.4	0
0.16,27.5	0
0.16,27.6	0
0.16,27.7	0
0.16,27.8	0
0.16,27.9	0
0.16,28.0	0
0.16,28.1	0
0.16,28.2	0
0.16,28.3	0
0.16,28.4	0
0.16,28.5	0
0.16,28.6	0
0.16,28.7	0
0.16,28.8	0
0.16,28.9	0
0.16,29.0	0
0.16,29.1	0
0.16,29.2	0
0.16,29.3	0
0.16,29.4	0
0.16,29.5	0
0.16,29.6	0
0.16,29.7	0
0.16,29.8	0
0.16,29.9	0
0.16,30.0	0
0.16,30.1	0
0.16,30.2	0
0.16,30.3	0
0.16,30.4	0
0.16,30.5	0
0.16,30.6	0
0.16,30.7	0
0.16,30.8	0
0.16,30.9	0
0.16,31.0	0
0.16,31.1	0
0.16,31.2	0
0.16,31.3	0

0.16,31.4	0
0.16,31.5	0
0.16,31.6	0
0.16,31.7	0
0.16,31.8	0
0.16,31.9	0
0.16,32.0	0
0.16,32.1	0
0.16,32.2	0
0.16,32.3	0
0.16,32.4	0
0.16,32.5	0
0.16,32.6	0
0.16,32.7	0
0.16,32.8	0
0.16,32.9	0
0.16,33.0	0
0.16,33.1	0
0.16,33.2	0
0.16,33.3	0
0.16,33.4	0
0.16,33.5	0
0.16,33.6	0
0.16,33.7	0
0.16,33.8	0
0.16,33.9	0
0.16,34.0	0
0.16,34.1	0
0.16,34.2	0
0.16,34.3	0
0.16,34.4	0
0.16,34.5	0
0.16,34.6	0
0.16,34.7	0
0.16,34.8	0
0.16,34.9	0
0.16,35.0	0
0.16,35.1	0
0.16,35.2	0
0.16,35.3	0
0.16,35.4	0
0.16,35.5	0

0.16,35.6	0
0.16,35.7	0
0.16,35.8	0
0.16,35.9	0
0.16,36.0	0
0.16,36.1	0
0.16,36.2	0
0.16,36.3	0
0.16,36.4	0
0.16,36.5	0
0.16,36.6	0
0.16,36.7	0
0.16,36.8	0
0.16,36.9	0
0.16,37.0	0
0.16,37.1	0
0.16,37.2	0
0.16,37.3	0
0.16,37.4	0
0.16,37.5	0
0.16,37.6	0
0.16,37.7	0
0.16,37.8	0
0.16,37.9	0
0.16,38.0	0
0.16,38.1	0
0.16,38.2	0
0.16,38.3	0
0.16,38.4	0
0.16,38.5	0
0.16,38.6	0
0.16,38.7	0
0.16,38.8	0
0.16,38.9	0
0.16,39.0	0
0.16,39.1	0
0.16,39.2	0
0.16,39.3	0
0.16,39.4	0
0.16,39.5	0
0.16,39.6	0
0.16,39.7	0

0.16,39.8	0
0.16,39.9	0
0.16,40.0	0
0.16,40.1	0
0.16,40.2	0
0.16,40.3	0
0.16,40.4	0
0.16,40.5	0
0.16,40.6	0
0.16,40.7	0
0.16,40.8	0
0.16,40.9	0
0.16,41.0	0
0.16,41.1	0
0.16,41.2	0
0.16,41.3	0
0.16,41.4	0
0.16,41.5	0
0.16,41.6	0
0.16,41.7	0
0.16,41.8	0
0.16,41.9	0
0.16,42.0	0
0.16,42.1	0
0.16,42.2	0
0.16,42.3	0
0.16,42.4	0
0.16,42.5	0
0.16,42.6	0
0.16,42.7	0
0.16,42.8	0
0.16,42.9	0
0.16,43.0	0
0.16,43.1	0
0.16,43.2	0
0.16,43.3	0
0.16,43.4	0
0.16,43.5	0
0.16,43.6	0
0.16,43.7	0
0.16,43.8	0
0.16,43.9	0

0.16,44.0	0
0.16,44.1	0
0.16,44.2	0
0.16,44.3	0
0.16,44.4	0
0.16,44.5	0
0.16,44.6	0
0.16,44.7	0
0.16,44.8	0
0.16,44.9	0
0.16,45.0	0
0.16,45.1	0
0.16,45.2	0
0.16,45.3	0
0.16,45.4	0
0.16,45.5	0
0.16,45.6	0
0.16,45.7	0
0.16,45.8	0
0.16,45.9	0
0.16,46.0	0
0.16,46.1	0
0.16,46.2	0
0.16,46.3	0
0.16,46.4	0
0.16,46.5	0
0.16,46.6	0
0.16,46.7	0
0.16,46.8	0
0.16,46.9	0
0.16,47.0	0
0.16,47.1	0
0.16,47.2	0
0.16,47.3	0
0.16,47.4	0
0.16,47.5	0
0.16,47.6	0
0.16,47.7	0
0.16,47.8	0
0.16,47.9	0
0.16,48.0	0
0.16,48.1	0

0.16,48.2	0
0.16,48.3	0
0.16,48.4	0
0.16,48.5	0
0.16,48.6	0
0.16,48.7	0
0.16,48.8	0
0.16,48.9	0
0.16,49.0	0
0.16,49.1	0
0.16,49.2	0
0.16,49.3	0
0.16,49.4	0
0.16,49.5	0
0.16,49.6	0
0.16,49.7	0
0.16,49.8	0
0.16,49.9	0
0.16,50.0	0
0.16,50.1	0
0.16,50.2	0
0.16,50.3	0
0.16,50.4	0
0.16,50.5	0
0.16,50.6	0
0.16,50.7	0
0.16,50.8	0
0.16,50.9	0
0.16,51.0	0
0.16,51.1	0
0.16,51.2	0
0.16,51.3	0
0.16,51.4	0
0.16,51.5	0
0.16,51.6	0
0.16,51.7	0
0.16,51.8	0
0.16,51.9	0
0.16,52.0	0
0.16,52.1	0
0.16,52.2	0
0.16,52.3	0

0.16,52.4	0
0.16,52.5	0
0.16,52.6	0
0.16,52.7	0
0.16,52.8	0
0.16,52.9	0
0.16,53.0	0
0.16,53.1	0
0.16,53.2	0
0.16,53.3	0
0.16,53.4	0
0.16,53.5	0
0.16,53.6	0
0.16,53.7	0
0.16,53.8	0
0.16,53.9	0
0.16,54.0	0
0.16,54.1	0
0.16,54.2	0
0.16,54.3	0
0.16,54.4	0
0.16,54.5	0
0.16,54.6	0
0.16,54.7	0
0.16,54.8	0
0.16,54.9	0
0.16,55.0	0
0.16,55.1	0
0.16,55.2	0
0.16,55.3	0
0.16,55.4	0
0.16,55.5	0
0.16,55.6	0
0.16,55.7	0
0.16,55.8	0
0.16,55.9	0
0.16,56.0	0
0.16,56.1	0
0.16,56.2	0
0.16,56.3	0
0.16,56.4	0
0.16,56.5	0

0.16,56.6	0
0.16,56.7	0
0.16,56.8	0
0.16,56.9	0
0.16,57.0	0
0.16,57.1	0
0.16,57.2	0
0.16,57.3	0
0.16,57.4	0
0.16,57.5	0
0.16,57.6	0
0.16,57.7	0
0.16,57.8	0
0.16,57.9	0
0.16,58.0	0
0.16,58.1	0
0.16,58.2	0
0.16,58.3	0
0.16,58.4	0
0.16,58.5	0
0.16,58.6	0
0.16,58.7	0
0.16,58.8	0
0.16,58.9	0
0.16,59.0	0
0.16,59.1	0
0.16,59.2	0
0.16,59.3	0
0.16,59.4	0
0.16,59.5	0
0.16,59.6	0
0.16,59.7	0
0.16,59.8	0
0.16,59.9	0
0.16,60.0	0
0.16,60.1	0
0.16,60.2	0
0.16,60.3	0
0.16,60.4	0
0.16,60.5	0
0.16,60.6	0
0.16,60.7	0

0.16,60.8	0
0.16,60.9	0
0.16,61.0	0
0.16,61.1	0
0.16,61.2	0
0.16,61.3	0
0.16,61.4	0
0.16,61.5	0
0.16,61.6	0
0.16,61.7	0
0.16,61.8	0
0.16,61.9	0
0.16,62.0	0
0.16,62.1	0
0.16,62.2	0
0.16,62.3	0
0.16,62.4	0
0.16,62.5	0
0.16,62.6	0
0.16,62.7	0
0.16,62.8	0
0.16,62.9	0
0.16,63.0	0
0.16,63.1	0
0.16,63.2	0
0.16,63.3	0
0.16,63.4	0
0.16,63.5	0
0.16,63.6	0
0.16,63.7	0
0.16,63.8	0
0.16,63.9	0
0.16,64.0	0
0.16,64.1	0
0.16,64.2	0
0.16,64.3	0
0.16,64.4	0
0.16,64.5	0
0.16,64.6	0
0.16,64.7	0
0.16,64.8	0
0.16,64.9	0

0.16,65.0	0
0.16,65.1	0
0.16,65.2	0
0.16,65.3	0
0.16,65.4	0
0.16,65.5	0
0.16,65.6	0
0.16,65.7	0
0.16,65.8	0
0.16,65.9	0
0.16,66.0	0
0.16,66.1	0
0.16,66.2	0
0.16,66.3	0
0.16,66.4	0
0.16,66.5	0
0.16,66.6	0
0.16,66.7	0
0.16,66.8	0
0.16,66.9	0
0.16,67.0	0
0.16,67.1	0
0.16,67.2	0
0.16,67.3	0
0.16,67.4	0
0.16,67.5	0
0.16,67.6	0
0.16,67.7	0
0.16,67.8	0
0.16,67.9	0
0.16,68.0	0
0.16,68.1	0
0.16,68.2	0
0.16,68.3	0
0.16,68.4	0
0.16,68.5	0
0.16,68.6	0
0.16,68.7	0
0.16,68.8	0
0.16,68.9	0
0.16,69.0	0
0.16,69.1	0

0.16,69.2	0
0.16,69.3	0
0.16,69.4	0
0.16,69.5	0
0.16,69.6	0
0.16,69.7	0
0.16,69.8	0
0.16,69.9	0
0.16,70.0	0
0.16,70.1	0
0.16,70.2	0
0.16,70.3	0
0.16,70.4	0
0.16,70.5	0
0.16,70.6	0
0.16,70.7	0
0.16,70.8	0
0.16,70.9	0
0.16,71.0	0
0.16,71.1	0
0.16,71.2	0
0.16,71.3	0
0.16,71.4	0
0.16,71.5	0
0.16,71.6	0
0.16,71.7	0
0.16,71.8	0
0.16,71.9	0
0.16,72.0	0
0.16,72.1	0
0.16,72.2	0
0.16,72.3	0
0.16,72.4	0
0.16,72.5	0
0.16,72.6	0
0.16,72.7	0
0.16,72.8	0
0.16,72.9	0
0.16,73.0	0
0.16,73.1	0
0.16,73.2	0
0.16,73.3	0

0.16,73.4	0
0.16,73.5	0
0.16,73.6	0
0.16,73.7	0
0.16,73.8	0
0.16,73.9	0
0.16,74.0	0
0.16,74.1	0
0.16,74.2	0
0.16,74.3	0
0.16,74.4	0
0.16,74.5	0
0.16,74.6	0
0.16,74.7	0
0.16,74.8	0
0.16,74.9	0
0.16,75.0	0
0.16,75.1	0
0.16,75.2	0
0.16,75.3	0
0.16,75.4	0
0.16,75.5	0
0.16,75.6	0
0.16,75.7	0
0.16,75.8	0
0.16,75.9	0
0.16,76.0	0
0.16,76.1	0
0.16,76.2	0
0.16,76.3	0
0.16,76.4	0
0.16,76.5	0
0.16,76.6	0
0.16,76.7	0
0.16,76.8	0
0.16,76.9	0
0.16,77.0	0
0.16,77.1	0
0.16,77.2	0
0.16,77.3	0
0.16,77.4	0
0.16,77.5	0

0.16,77.6	0
0.16,77.7	0
0.16,77.8	0
0.16,77.9	0
0.16,78.0	0
0.16,78.1	0
0.16,78.2	0
0.16,78.3	0
0.16,78.4	0
0.16,78.5	0
0.16,78.6	0
0.16,78.7	0
0.16,78.8	0
0.16,78.9	0
0.16,79.0	0
0.16,79.1	0
0.16,79.2	0
0.16,79.3	0
0.16,79.4	0
0.16,79.5	0
0.16,79.6	0
0.16,79.7	0
0.16,79.8	0
0.16,79.9	0
0.16,80.0	0
0.16,80.1	0
0.16,80.2	0
0.16,80.3	0
0.16,80.4	0
0.16,80.5	0
0.16,80.6	0
0.16,80.7	0
0.16,80.8	0
0.16,80.9	0
0.16,81.0	0
0.16,81.1	0
0.16,81.2	0
0.16,81.3	0
0.16,81.4	0
0.16,81.5	0
0.16,81.6	0
0.16,81.7	0

0.16,81.8	0
0.16,81.9	0
0.16,82.0	0
0.16,82.1	0
0.16,82.2	0
0.16,82.3	0
0.16,82.4	0
0.16,82.5	0
0.16,82.6	0
0.16,82.7	0
0.16,82.8	0
0.16,82.9	0
0.16,83.0	0
0.16,83.1	0
0.16,83.2	0
0.16,83.3	0
0.16,83.4	0
0.16,83.5	0
0.16,83.6	0
0.16,83.7	0
0.16,83.8	0
0.16,83.9	0
0.16,84.0	0
0.16,84.1	0
0.16,84.2	0
0.16,84.3	0
0.16,84.4	0
0.16,84.5	0
0.16,84.6	0
0.16,84.7	0
0.16,84.8	0
0.16,84.9	0
0.16,85.0	0
0.16,85.1	0
0.16,85.2	0
0.16,85.3	0
0.16,85.4	0
0.16,85.5	0
0.16,85.6	0
0.16,85.7	0
0.16,85.8	0
0.16,85.9	0

0.16,86.0	0
0.16,86.1	0
0.16,86.2	0
0.16,86.3	0
0.16,86.4	0
0.16,86.5	0
0.16,86.6	0
0.16,86.7	0
0.16,86.8	0
0.16,86.9	0
0.16,87.0	0
0.16,87.1	0
0.16,87.2	0
0.16,87.3	0
0.16,87.4	0
0.16,87.5	0
0.16,87.6	0
0.16,87.7	0
0.16,87.8	0
0.16,87.9	0
0.16,88.0	0
0.16,88.1	0
0.16,88.2	0
0.16,88.3	0
0.16,88.4	0
0.16,88.5	0
0.16,88.6	0
0.16,88.7	0
0.16,88.8	0
0.16,88.9	0
0.16,89.0	0
0.16,89.1	0
0.16,89.2	0
0.16,89.3	0
0.16,89.4	0
0.16,89.5	0
0.16,89.6	0
0.16,89.7	0
0.16,89.8	0
0.16,89.9	0
0.16,90.0	0
0.16,90.1	0

0.16,90.2	0
0.16,90.3	0
0.16,90.4	0
0.16,90.5	0
0.16,90.6	0
0.16,90.7	0
0.16,90.8	0
0.16,90.9	0
0.16,91.0	0
0.16,91.1	0
0.16,91.2	0
0.16,91.3	0
0.16,91.4	0
0.16,91.5	0
0.16,91.6	0
0.16,91.7	0
0.16,91.8	0
0.16,91.9	0
0.16,92.0	0
0.16,92.1	0
0.16,92.2	0
0.16,92.3	0
0.16,92.4	0
0.16,92.5	0
0.16,92.6	0
0.16,92.7	0
0.16,92.8	0
0.16,92.9	0
0.16,93.0	0
0.16,93.1	0
0.16,93.2	0
0.16,93.3	0
0.16,93.4	0
0.16,93.5	0
0.16,93.6	0
0.16,93.7	0
0.16,93.8	0
0.16,93.9	0
0.16,94.0	0
0.16,94.1	0
0.16,94.2	0
0.16,94.3	0

0.16,94.4	0
0.16,94.5	0
0.16,94.6	0
0.16,94.7	0
0.16,94.8	0
0.16,94.9	0
0.16,95.0	0
0.16,95.1	0
0.16,95.2	0
0.16,95.3	0
0.16,95.4	0
0.16,95.5	0
0.16,95.6	0
0.16,95.7	0
0.16,95.8	0
0.16,95.9	0
0.16,96.0	0
0.16,96.1	0
0.16,96.2	0
0.16,96.3	0
0.16,96.4	0
0.16,96.5	0
0.16,96.6	0
0.16,96.7	0
0.16,96.8	0
0.16,96.9	0
0.16,97.0	0
0.16,97.1	0
0.16,97.2	0
0.16,97.3	0
0.16,97.4	0
0.16,97.5	0
0.16,97.6	0
0.16,97.7	0
0.16,97.8	0
0.16,97.9	0
0.16,98.0	0
0.16,98.1	0
0.16,98.2	0
0.16,98.3	0
0.16,98.4	0
0.16,98.5	0

0.16,98.6	0
0.16,98.7	0
0.16,98.8	0
0.16,98.9	0
0.16,99.0	0
0.16,99.1	0
0.16,99.2	0
0.16,99.3	0
0.16,99.4	0
0.16,99.5	0
0.16,99.6	0
0.16,99.7	0
0.16,99.8	0
0.16,99.9	0
0.16,100.0	0
0.17,0.0	0
0.17,0.1	0
0.17,0.2	0
0.17,0.3	0
0.17,0.4	0
0.17,0.5	0
0.17,0.6	0
0.17,0.7	0
0.17,0.8	0
0.17,0.9	0
0.17,1.0	0
0.17,1.1	0
0.17,1.2	0
0.17,1.3	0
0.17,1.4	0
0.17,1.5	0
0.17,1.6	0
0.17,1.7	0
0.17,1.8	0
0.17,1.9	0
0.17,2.0	0
0.17,2.1	0
0.17,2.2	0
0.17,2.3	0
0.17,2.4	0
0.17,2.5	0
0.17,2.6	0

0.17,2.7	0
0.17,2.8	0
0.17,2.9	0
0.17,3.0	0
0.17,3.1	0
0.17,3.2	0
0.17,3.3	0
0.17,3.4	0
0.17,3.5	0
0.17,3.6	0
0.17,3.7	0
0.17,3.8	0
0.17,3.9	0
0.17,4.0	0
0.17,4.1	0
0.17,4.2	0
0.17,4.3	0
0.17,4.4	0
0.17,4.5	0
0.17,4.6	0
0.17,4.7	0
0.17,4.8	0
0.17,4.9	0
0.17,5.0	0
0.17,5.1	0
0.17,5.2	0
0.17,5.3	0
0.17,5.4	0
0.17,5.5	0
0.17,5.6	0
0.17,5.7	0
0.17,5.8	0
0.17,5.9	0
0.17,6.0	0
0.17,6.1	0
0.17,6.2	0
0.17,6.3	0
0.17,6.4	0
0.17,6.5	0
0.17,6.6	0
0.17,6.7	0
0.17,6.8	0

0.17,6.9	0
0.17,7.0	0
0.17,7.1	0
0.17,7.2	0
0.17,7.3	0
0.17,7.4	0
0.17,7.5	0
0.17,7.6	0
0.17,7.7	0
0.17,7.8	0
0.17,7.9	0
0.17,8.0	0
0.17,8.1	0
0.17,8.2	0
0.17,8.3	0
0.17,8.4	0
0.17,8.5	0
0.17,8.6	0
0.17,8.7	0
0.17,8.8	0
0.17,8.9	0
0.17,9.0	0
0.17,9.1	0
0.17,9.2	0
0.17,9.3	0
0.17,9.4	0
0.17,9.5	0
0.17,9.6	0
0.17,9.7	0
0.17,9.8	0
0.17,9.9	0
0.17,10.0	0
0.17,10.1	0
0.17,10.2	0
0.17,10.3	0
0.17,10.4	0
0.17,10.5	0
0.17,10.6	0
0.17,10.7	0
0.17,10.8	0
0.17,10.9	0
0.17,11.0	0

0.17,11.1	0
0.17,11.2	0
0.17,11.3	0
0.17,11.4	0
0.17,11.5	0
0.17,11.6	0
0.17,11.7	0
0.17,11.8	0
0.17,11.9	0
0.17,12.0	0
0.17,12.1	0
0.17,12.2	0
0.17,12.3	0
0.17,12.4	0
0.17,12.5	0
0.17,12.6	0
0.17,12.7	0
0.17,12.8	0
0.17,12.9	0
0.17,13.0	0
0.17,13.1	0
0.17,13.2	0
0.17,13.3	0
0.17,13.4	0
0.17,13.5	0
0.17,13.6	0
0.17,13.7	0
0.17,13.8	0
0.17,13.9	0
0.17,14.0	0
0.17,14.1	0
0.17,14.2	0
0.17,14.3	0
0.17,14.4	0
0.17,14.5	0
0.17,14.6	0
0.17,14.7	0
0.17,14.8	0
0.17,14.9	0
0.17,15.0	0
0.17,15.1	0
0.17,15.2	0

0.17,15.3	0
0.17,15.4	0
0.17,15.5	0
0.17,15.6	0
0.17,15.7	0
0.17,15.8	0
0.17,15.9	0
0.17,16.0	0
0.17,16.1	0
0.17,16.2	0
0.17,16.3	0
0.17,16.4	0
0.17,16.5	0
0.17,16.6	0
0.17,16.7	0
0.17,16.8	0
0.17,16.9	0
0.17,17.0	0
0.17,17.1	0
0.17,17.2	0
0.17,17.3	0
0.17,17.4	0
0.17,17.5	0
0.17,17.6	0
0.17,17.7	0
0.17,17.8	0
0.17,17.9	0
0.17,18.0	0
0.17,18.1	0
0.17,18.2	0
0.17,18.3	0
0.17,18.4	0
0.17,18.5	0
0.17,18.6	0
0.17,18.7	0
0.17,18.8	0
0.17,18.9	0
0.17,19.0	0
0.17,19.1	0
0.17,19.2	0
0.17,19.3	0
0.17,19.4	0

0.17,19.5	0
0.17,19.6	0
0.17,19.7	0
0.17,19.8	0
0.17,19.9	0
0.17,20.0	0
0.17,20.1	0
0.17,20.2	0
0.17,20.3	0
0.17,20.4	0
0.17,20.5	0
0.17,20.6	0
0.17,20.7	0
0.17,20.8	0
0.17,20.9	0
0.17,21.0	0
0.17,21.1	0
0.17,21.2	0
0.17,21.3	0
0.17,21.4	0
0.17,21.5	0
0.17,21.6	0
0.17,21.7	0
0.17,21.8	0
0.17,21.9	0
0.17,22.0	0
0.17,22.1	0
0.17,22.2	0
0.17,22.3	0
0.17,22.4	0
0.17,22.5	0
0.17,22.6	0
0.17,22.7	0
0.17,22.8	0
0.17,22.9	0
0.17,23.0	0
0.17,23.1	0
0.17,23.2	0
0.17,23.3	0
0.17,23.4	0
0.17,23.5	0
0.17,23.6	0

0.17,23.7	0
0.17,23.8	0
0.17,23.9	0
0.17,24.0	0
0.17,24.1	0
0.17,24.2	0
0.17,24.3	0
0.17,24.4	0
0.17,24.5	0
0.17,24.6	0
0.17,24.7	0
0.17,24.8	0
0.17,24.9	0
0.17,25.0	0
0.17,25.1	0
0.17,25.2	0
0.17,25.3	0
0.17,25.4	0
0.17,25.5	0
0.17,25.6	0
0.17,25.7	0
0.17,25.8	0
0.17,25.9	0
0.17,26.0	0
0.17,26.1	0
0.17,26.2	0
0.17,26.3	0
0.17,26.4	0
0.17,26.5	0
0.17,26.6	0
0.17,26.7	0
0.17,26.8	0
0.17,26.9	0
0.17,27.0	0
0.17,27.1	0
0.17,27.2	0
0.17,27.3	0
0.17,27.4	0
0.17,27.5	0
0.17,27.6	0
0.17,27.7	0
0.17,27.8	0

0.17,27.9	0
0.17,28.0	0
0.17,28.1	0
0.17,28.2	0
0.17,28.3	0
0.17,28.4	0
0.17,28.5	0
0.17,28.6	0
0.17,28.7	0
0.17,28.8	0
0.17,28.9	0
0.17,29.0	0
0.17,29.1	0
0.17,29.2	0
0.17,29.3	0
0.17,29.4	0
0.17,29.5	0
0.17,29.6	0
0.17,29.7	0
0.17,29.8	0
0.17,29.9	0
0.17,30.0	0
0.17,30.1	0
0.17,30.2	0
0.17,30.3	0
0.17,30.4	0
0.17,30.5	0
0.17,30.6	0
0.17,30.7	0
0.17,30.8	0
0.17,30.9	0
0.17,31.0	0
0.17,31.1	0
0.17,31.2	0
0.17,31.3	0
0.17,31.4	0
0.17,31.5	0
0.17,31.6	0
0.17,31.7	0
0.17,31.8	0
0.17,31.9	0
0.17,32.0	0

0.17,32.1	0
0.17,32.2	0
0.17,32.3	0
0.17,32.4	0
0.17,32.5	0
0.17,32.6	0
0.17,32.7	0
0.17,32.8	0
0.17,32.9	0
0.17,33.0	0
0.17,33.1	0
0.17,33.2	0
0.17,33.3	0
0.17,33.4	0
0.17,33.5	0
0.17,33.6	0
0.17,33.7	0
0.17,33.8	0
0.17,33.9	0
0.17,34.0	0
0.17,34.1	0
0.17,34.2	0
0.17,34.3	0
0.17,34.4	0
0.17,34.5	0
0.17,34.6	0
0.17,34.7	0
0.17,34.8	0
0.17,34.9	0
0.17,35.0	0
0.17,35.1	0
0.17,35.2	0
0.17,35.3	0
0.17,35.4	0
0.17,35.5	0
0.17,35.6	0
0.17,35.7	0
0.17,35.8	0
0.17,35.9	0
0.17,36.0	0
0.17,36.1	0
0.17,36.2	0

0.17,36.3	0
0.17,36.4	0
0.17,36.5	0
0.17,36.6	0
0.17,36.7	0
0.17,36.8	0
0.17,36.9	0
0.17,37.0	0
0.17,37.1	0
0.17,37.2	0
0.17,37.3	0
0.17,37.4	0
0.17,37.5	0
0.17,37.6	0
0.17,37.7	0
0.17,37.8	0
0.17,37.9	0
0.17,38.0	0
0.17,38.1	0
0.17,38.2	0
0.17,38.3	0
0.17,38.4	0
0.17,38.5	0
0.17,38.6	0
0.17,38.7	0
0.17,38.8	0
0.17,38.9	0
0.17,39.0	0
0.17,39.1	0
0.17,39.2	0
0.17,39.3	0
0.17,39.4	0
0.17,39.5	0
0.17,39.6	0
0.17,39.7	0
0.17,39.8	0
0.17,39.9	0
0.17,40.0	0
0.17,40.1	0
0.17,40.2	0
0.17,40.3	0
0.17,40.4	0

0.17,40.5	0
0.17,40.6	0
0.17,40.7	0
0.17,40.8	0
0.17,40.9	0
0.17,41.0	0
0.17,41.1	0
0.17,41.2	0
0.17,41.3	0
0.17,41.4	0
0.17,41.5	0
0.17,41.6	0
0.17,41.7	0
0.17,41.8	0
0.17,41.9	0
0.17,42.0	0
0.17,42.1	0
0.17,42.2	0
0.17,42.3	0
0.17,42.4	0
0.17,42.5	0
0.17,42.6	0
0.17,42.7	0
0.17,42.8	0
0.17,42.9	0
0.17,43.0	0
0.17,43.1	0
0.17,43.2	0
0.17,43.3	0
0.17,43.4	0
0.17,43.5	0
0.17,43.6	0
0.17,43.7	0
0.17,43.8	0
0.17,43.9	0
0.17,44.0	0
0.17,44.1	0
0.17,44.2	0
0.17,44.3	0
0.17,44.4	0
0.17,44.5	0
0.17,44.6	0

0.17,44.7	0
0.17,44.8	0
0.17,44.9	0
0.17,45.0	0
0.17,45.1	0
0.17,45.2	0
0.17,45.3	0
0.17,45.4	0
0.17,45.5	0
0.17,45.6	0
0.17,45.7	0
0.17,45.8	0
0.17,45.9	0
0.17,46.0	0
0.17,46.1	0
0.17,46.2	0
0.17,46.3	0
0.17,46.4	0
0.17,46.5	0
0.17,46.6	0
0.17,46.7	0
0.17,46.8	0
0.17,46.9	0
0.17,47.0	0
0.17,47.1	0
0.17,47.2	0
0.17,47.3	0
0.17,47.4	0
0.17,47.5	0
0.17,47.6	0
0.17,47.7	0
0.17,47.8	0
0.17,47.9	0
0.17,48.0	0
0.17,48.1	0
0.17,48.2	0
0.17,48.3	0
0.17,48.4	0
0.17,48.5	0
0.17,48.6	0
0.17,48.7	0
0.17,48.8	0

0.17,48.9	0
0.17,49.0	0
0.17,49.1	0
0.17,49.2	0
0.17,49.3	0
0.17,49.4	0
0.17,49.5	0
0.17,49.6	0
0.17,49.7	0
0.17,49.8	0
0.17,49.9	0
0.17,50.0	0
0.17,50.1	0
0.17,50.2	0
0.17,50.3	0
0.17,50.4	0
0.17,50.5	0
0.17,50.6	0
0.17,50.7	0
0.17,50.8	0
0.17,50.9	0
0.17,51.0	0
0.17,51.1	0
0.17,51.2	0
0.17,51.3	0
0.17,51.4	0
0.17,51.5	0
0.17,51.6	0
0.17,51.7	0
0.17,51.8	0
0.17,51.9	0
0.17,52.0	0
0.17,52.1	0
0.17,52.2	0
0.17,52.3	0
0.17,52.4	0
0.17,52.5	0
0.17,52.6	0
0.17,52.7	0
0.17,52.8	0
0.17,52.9	0
0.17,53.0	0

0.17,53.1	0
0.17,53.2	0
0.17,53.3	0
0.17,53.4	0
0.17,53.5	0
0.17,53.6	0
0.17,53.7	0
0.17,53.8	0
0.17,53.9	0
0.17,54.0	0
0.17,54.1	0
0.17,54.2	0
0.17,54.3	0
0.17,54.4	0
0.17,54.5	0
0.17,54.6	0
0.17,54.7	0
0.17,54.8	0
0.17,54.9	0
0.17,55.0	0
0.17,55.1	0
0.17,55.2	0
0.17,55.3	0
0.17,55.4	0
0.17,55.5	0
0.17,55.6	0
0.17,55.7	0
0.17,55.8	0
0.17,55.9	0
0.17,56.0	0
0.17,56.1	0
0.17,56.2	0
0.17,56.3	0
0.17,56.4	0
0.17,56.5	0
0.17,56.6	0
0.17,56.7	0
0.17,56.8	0
0.17,56.9	0
0.17,57.0	0
0.17,57.1	0
0.17,57.2	0

0.17,57.3	0
0.17,57.4	0
0.17,57.5	0
0.17,57.6	0
0.17,57.7	0
0.17,57.8	0
0.17,57.9	0
0.17,58.0	0
0.17,58.1	0
0.17,58.2	0
0.17,58.3	0
0.17,58.4	0
0.17,58.5	0
0.17,58.6	0
0.17,58.7	0
0.17,58.8	0
0.17,58.9	0
0.17,59.0	0
0.17,59.1	0
0.17,59.2	0
0.17,59.3	0
0.17,59.4	0
0.17,59.5	0
0.17,59.6	0
0.17,59.7	0
0.17,59.8	0
0.17,59.9	0
0.17,60.0	0
0.17,60.1	0
0.17,60.2	0
0.17,60.3	0
0.17,60.4	0
0.17,60.5	0
0.17,60.6	0
0.17,60.7	0
0.17,60.8	0
0.17,60.9	0
0.17,61.0	0
0.17,61.1	0
0.17,61.2	0
0.17,61.3	0
0.17,61.4	0

0.17,61.5	0
0.17,61.6	0
0.17,61.7	0
0.17,61.8	0
0.17,61.9	0
0.17,62.0	0
0.17,62.1	0
0.17,62.2	0
0.17,62.3	0
0.17,62.4	0
0.17,62.5	0
0.17,62.6	0
0.17,62.7	0
0.17,62.8	0
0.17,62.9	0
0.17,63.0	0
0.17,63.1	0
0.17,63.2	0
0.17,63.3	0
0.17,63.4	0
0.17,63.5	0
0.17,63.6	0
0.17,63.7	0
0.17,63.8	0
0.17,63.9	0
0.17,64.0	0
0.17,64.1	0
0.17,64.2	0
0.17,64.3	0
0.17,64.4	0
0.17,64.5	0
0.17,64.6	0
0.17,64.7	0
0.17,64.8	0
0.17,64.9	0
0.17,65.0	0
0.17,65.1	0
0.17,65.2	0
0.17,65.3	0
0.17,65.4	0
0.17,65.5	0
0.17,65.6	0

0.17,65.7	0
0.17,65.8	0
0.17,65.9	0
0.17,66.0	0
0.17,66.1	0
0.17,66.2	0
0.17,66.3	0
0.17,66.4	0
0.17,66.5	0
0.17,66.6	0
0.17,66.7	0
0.17,66.8	0
0.17,66.9	0
0.17,67.0	0
0.17,67.1	0
0.17,67.2	0
0.17,67.3	0
0.17,67.4	0
0.17,67.5	0
0.17,67.6	0
0.17,67.7	0
0.17,67.8	0
0.17,67.9	0
0.17,68.0	0
0.17,68.1	0
0.17,68.2	0
0.17,68.3	0
0.17,68.4	0
0.17,68.5	0
0.17,68.6	0
0.17,68.7	0
0.17,68.8	0
0.17,68.9	0
0.17,69.0	0
0.17,69.1	0
0.17,69.2	0
0.17,69.3	0
0.17,69.4	0
0.17,69.5	0
0.17,69.6	0
0.17,69.7	0
0.17,69.8	0

0.17,69.9	0
0.17,70.0	0
0.17,70.1	0
0.17,70.2	0
0.17,70.3	0
0.17,70.4	0
0.17,70.5	0
0.17,70.6	0
0.17,70.7	0
0.17,70.8	0
0.17,70.9	0
0.17,71.0	0
0.17,71.1	0
0.17,71.2	0
0.17,71.3	0
0.17,71.4	0
0.17,71.5	0
0.17,71.6	0
0.17,71.7	0
0.17,71.8	0
0.17,71.9	0
0.17,72.0	0
0.17,72.1	0
0.17,72.2	0
0.17,72.3	0
0.17,72.4	0
0.17,72.5	0
0.17,72.6	0
0.17,72.7	0
0.17,72.8	0
0.17,72.9	0
0.17,73.0	0
0.17,73.1	0
0.17,73.2	0
0.17,73.3	0
0.17,73.4	0
0.17,73.5	0
0.17,73.6	0
0.17,73.7	0
0.17,73.8	0
0.17,73.9	0
0.17,74.0	0

0.17,74.1	0
0.17,74.2	0
0.17,74.3	0
0.17,74.4	0
0.17,74.5	0
0.17,74.6	0
0.17,74.7	0
0.17,74.8	0
0.17,74.9	0
0.17,75.0	0
0.17,75.1	0
0.17,75.2	0
0.17,75.3	0
0.17,75.4	0
0.17,75.5	0
0.17,75.6	0
0.17,75.7	0
0.17,75.8	0
0.17,75.9	0
0.17,76.0	0
0.17,76.1	0
0.17,76.2	0
0.17,76.3	0
0.17,76.4	0
0.17,76.5	0
0.17,76.6	0
0.17,76.7	0
0.17,76.8	0
0.17,76.9	0
0.17,77.0	0
0.17,77.1	0
0.17,77.2	0
0.17,77.3	0
0.17,77.4	0
0.17,77.5	0
0.17,77.6	0
0.17,77.7	0
0.17,77.8	0
0.17,77.9	0
0.17,78.0	0
0.17,78.1	0
0.17,78.2	0

0.17,78.3	0
0.17,78.4	0
0.17,78.5	0
0.17,78.6	0
0.17,78.7	0
0.17,78.8	0
0.17,78.9	0
0.17,79.0	0
0.17,79.1	0
0.17,79.2	0
0.17,79.3	0
0.17,79.4	0
0.17,79.5	0
0.17,79.6	0
0.17,79.7	0
0.17,79.8	0
0.17,79.9	0
0.17,80.0	0
0.17,80.1	0
0.17,80.2	0
0.17,80.3	0
0.17,80.4	0
0.17,80.5	0
0.17,80.6	0
0.17,80.7	0
0.17,80.8	0
0.17,80.9	0
0.17,81.0	0
0.17,81.1	0
0.17,81.2	0
0.17,81.3	0
0.17,81.4	0
0.17,81.5	0
0.17,81.6	0
0.17,81.7	0
0.17,81.8	0
0.17,81.9	0
0.17,82.0	0
0.17,82.1	0
0.17,82.2	0
0.17,82.3	0
0.17,82.4	0

0.17,82.5	0
0.17,82.6	0
0.17,82.7	0
0.17,82.8	0
0.17,82.9	0
0.17,83.0	0
0.17,83.1	0
0.17,83.2	0
0.17,83.3	0
0.17,83.4	0
0.17,83.5	0
0.17,83.6	0
0.17,83.7	0
0.17,83.8	0
0.17,83.9	0
0.17,84.0	0
0.17,84.1	0
0.17,84.2	0
0.17,84.3	0
0.17,84.4	0
0.17,84.5	0
0.17,84.6	0
0.17,84.7	0
0.17,84.8	0
0.17,84.9	0
0.17,85.0	0
0.17,85.1	0
0.17,85.2	0
0.17,85.3	0
0.17,85.4	0
0.17,85.5	0
0.17,85.6	0
0.17,85.7	0
0.17,85.8	0
0.17,85.9	0
0.17,86.0	0
0.17,86.1	0
0.17,86.2	0
0.17,86.3	0
0.17,86.4	0
0.17,86.5	0
0.17,86.6	0

0.17,86.7	0
0.17,86.8	0
0.17,86.9	0
0.17,87.0	0
0.17,87.1	0
0.17,87.2	0
0.17,87.3	0
0.17,87.4	0
0.17,87.5	0
0.17,87.6	0
0.17,87.7	0
0.17,87.8	0
0.17,87.9	0
0.17,88.0	0
0.17,88.1	0
0.17,88.2	0
0.17,88.3	0
0.17,88.4	0
0.17,88.5	0
0.17,88.6	0
0.17,88.7	0
0.17,88.8	0
0.17,88.9	0
0.17,89.0	0
0.17,89.1	0
0.17,89.2	0
0.17,89.3	0
0.17,89.4	0
0.17,89.5	0
0.17,89.6	0
0.17,89.7	0
0.17,89.8	0
0.17,89.9	0
0.17,90.0	0
0.17,90.1	0
0.17,90.2	0
0.17,90.3	0
0.17,90.4	0
0.17,90.5	0
0.17,90.6	0
0.17,90.7	0
0.17,90.8	0

0.17,90.9	0
0.17,91.0	0
0.17,91.1	0
0.17,91.2	0
0.17,91.3	0
0.17,91.4	0
0.17,91.5	0
0.17,91.6	0
0.17,91.7	0
0.17,91.8	0
0.17,91.9	0
0.17,92.0	0
0.17,92.1	0
0.17,92.2	0
0.17,92.3	0
0.17,92.4	0
0.17,92.5	0
0.17,92.6	0
0.17,92.7	0
0.17,92.8	0
0.17,92.9	0
0.17,93.0	0
0.17,93.1	0
0.17,93.2	0
0.17,93.3	0
0.17,93.4	0
0.17,93.5	0
0.17,93.6	0
0.17,93.7	0
0.17,93.8	0
0.17,93.9	0
0.17,94.0	0
0.17,94.1	0
0.17,94.2	0
0.17,94.3	0
0.17,94.4	0
0.17,94.5	0
0.17,94.6	0
0.17,94.7	0
0.17,94.8	0
0.17,94.9	0
0.17,95.0	0

0.17,95.1	0
0.17,95.2	0
0.17,95.3	0
0.17,95.4	0
0.17,95.5	0
0.17,95.6	0
0.17,95.7	0
0.17,95.8	0
0.17,95.9	0
0.17,96.0	0
0.17,96.1	0
0.17,96.2	0
0.17,96.3	0
0.17,96.4	0
0.17,96.5	0
0.17,96.6	0
0.17,96.7	0
0.17,96.8	0
0.17,96.9	0
0.17,97.0	0
0.17,97.1	0
0.17,97.2	0
0.17,97.3	0
0.17,97.4	0
0.17,97.5	0
0.17,97.6	0
0.17,97.7	0
0.17,97.8	0
0.17,97.9	0
0.17,98.0	0
0.17,98.1	0
0.17,98.2	0
0.17,98.3	0
0.17,98.4	0
0.17,98.5	0
0.17,98.6	0
0.17,98.7	0
0.17,98.8	0
0.17,98.9	0
0.17,99.0	0
0.17,99.1	0
0.17,99.2	0

0.17,99.3	0
0.17,99.4	0
0.17,99.5	0
0.17,99.6	0
0.17,99.7	0
0.17,99.8	0
0.17,99.9	0
0.17,100.0	0
0.18,0.0	0
0.18,0.1	0
0.18,0.2	0
0.18,0.3	0
0.18,0.4	0
0.18,0.5	0
0.18,0.6	0
0.18,0.7	0
0.18,0.8	0
0.18,0.9	0
0.18,1.0	0
0.18,1.1	0
0.18,1.2	0
0.18,1.3	0
0.18,1.4	0
0.18,1.5	0
0.18,1.6	0
0.18,1.7	0
0.18,1.8	0
0.18,1.9	0
0.18,2.0	0
0.18,2.1	0
0.18,2.2	0
0.18,2.3	0
0.18,2.4	0
0.18,2.5	0
0.18,2.6	0
0.18,2.7	0
0.18,2.8	0
0.18,2.9	0
0.18,3.0	0
0.18,3.1	0
0.18,3.2	0
0.18,3.3	0

0.18,3.4	0
0.18,3.5	0
0.18,3.6	0
0.18,3.7	0
0.18,3.8	0
0.18,3.9	0
0.18,4.0	0
0.18,4.1	0
0.18,4.2	0
0.18,4.3	0
0.18,4.4	0
0.18,4.5	0
0.18,4.6	0
0.18,4.7	0
0.18,4.8	0
0.18,4.9	0
0.18,5.0	0
0.18,5.1	0
0.18,5.2	0
0.18,5.3	0
0.18,5.4	0
0.18,5.5	0
0.18,5.6	0
0.18,5.7	0
0.18,5.8	0
0.18,5.9	0
0.18,6.0	0
0.18,6.1	0
0.18,6.2	0
0.18,6.3	0
0.18,6.4	0
0.18,6.5	0
0.18,6.6	0
0.18,6.7	0
0.18,6.8	0
0.18,6.9	0
0.18,7.0	0
0.18,7.1	0
0.18,7.2	0
0.18,7.3	0
0.18,7.4	0
0.18,7.5	0

0.18,7.6	0
0.18,7.7	0
0.18,7.8	0
0.18,7.9	0
0.18,8.0	0
0.18,8.1	0
0.18,8.2	0
0.18,8.3	0
0.18,8.4	0
0.18,8.5	0
0.18,8.6	0
0.18,8.7	0
0.18,8.8	0
0.18,8.9	0
0.18,9.0	0
0.18,9.1	0
0.18,9.2	0
0.18,9.3	0
0.18,9.4	0
0.18,9.5	0
0.18,9.6	0
0.18,9.7	0
0.18,9.8	0
0.18,9.9	0
0.18,10.0	0
0.18,10.1	0
0.18,10.2	0
0.18,10.3	0
0.18,10.4	0
0.18,10.5	0
0.18,10.6	0
0.18,10.7	0
0.18,10.8	0
0.18,10.9	0
0.18,11.0	0
0.18,11.1	0
0.18,11.2	0
0.18,11.3	0
0.18,11.4	0
0.18,11.5	0
0.18,11.6	0
0.18,11.7	0

0.18,11.8	0
0.18,11.9	0
0.18,12.0	0
0.18,12.1	0
0.18,12.2	0
0.18,12.3	0
0.18,12.4	0
0.18,12.5	0
0.18,12.6	0
0.18,12.7	0
0.18,12.8	0
0.18,12.9	0
0.18,13.0	0
0.18,13.1	0
0.18,13.2	0
0.18,13.3	0
0.18,13.4	0
0.18,13.5	0
0.18,13.6	0
0.18,13.7	0
0.18,13.8	0
0.18,13.9	0
0.18,14.0	0
0.18,14.1	0
0.18,14.2	0
0.18,14.3	0
0.18,14.4	0
0.18,14.5	0
0.18,14.6	0
0.18,14.7	0
0.18,14.8	0
0.18,14.9	0
0.18,15.0	0
0.18,15.1	0
0.18,15.2	0
0.18,15.3	0
0.18,15.4	0
0.18,15.5	0
0.18,15.6	0
0.18,15.7	0
0.18,15.8	0
0.18,15.9	0

0.18,16.0	0
0.18,16.1	0
0.18,16.2	0
0.18,16.3	0
0.18,16.4	0
0.18,16.5	0
0.18,16.6	0
0.18,16.7	0
0.18,16.8	0
0.18,16.9	0
0.18,17.0	0
0.18,17.1	0
0.18,17.2	0
0.18,17.3	0
0.18,17.4	0
0.18,17.5	0
0.18,17.6	0
0.18,17.7	0
0.18,17.8	0
0.18,17.9	0
0.18,18.0	0
0.18,18.1	0
0.18,18.2	0
0.18,18.3	0
0.18,18.4	0
0.18,18.5	0
0.18,18.6	0
0.18,18.7	0
0.18,18.8	0
0.18,18.9	0
0.18,19.0	0
0.18,19.1	0
0.18,19.2	0
0.18,19.3	0
0.18,19.4	0
0.18,19.5	0
0.18,19.6	0
0.18,19.7	0
0.18,19.8	0
0.18,19.9	0
0.18,20.0	0
0.18,20.1	0

0.18,20.2	0
0.18,20.3	0
0.18,20.4	0
0.18,20.5	0
0.18,20.6	0
0.18,20.7	0
0.18,20.8	0
0.18,20.9	0
0.18,21.0	0
0.18,21.1	0
0.18,21.2	0
0.18,21.3	0
0.18,21.4	0
0.18,21.5	0
0.18,21.6	0
0.18,21.7	0
0.18,21.8	0
0.18,21.9	0
0.18,22.0	0
0.18,22.1	0
0.18,22.2	0
0.18,22.3	0
0.18,22.4	0
0.18,22.5	0
0.18,22.6	0
0.18,22.7	0
0.18,22.8	0
0.18,22.9	0
0.18,23.0	0
0.18,23.1	0
0.18,23.2	0
0.18,23.3	0
0.18,23.4	0
0.18,23.5	0
0.18,23.6	0
0.18,23.7	0
0.18,23.8	0
0.18,23.9	0
0.18,24.0	0
0.18,24.1	0
0.18,24.2	0
0.18,24.3	0

0.18,24.4	0
0.18,24.5	0
0.18,24.6	0
0.18,24.7	0
0.18,24.8	0
0.18,24.9	0
0.18,25.0	0
0.18,25.1	0
0.18,25.2	0
0.18,25.3	0
0.18,25.4	0
0.18,25.5	0
0.18,25.6	0
0.18,25.7	0
0.18,25.8	0
0.18,25.9	0
0.18,26.0	0
0.18,26.1	0
0.18,26.2	0
0.18,26.3	0
0.18,26.4	0
0.18,26.5	0
0.18,26.6	0
0.18,26.7	0
0.18,26.8	0
0.18,26.9	0
0.18,27.0	0
0.18,27.1	0
0.18,27.2	0
0.18,27.3	0
0.18,27.4	0
0.18,27.5	0
0.18,27.6	0
0.18,27.7	0
0.18,27.8	0
0.18,27.9	0
0.18,28.0	0
0.18,28.1	0
0.18,28.2	0
0.18,28.3	0
0.18,28.4	0
0.18,28.5	0

0.18,28.6	0
0.18,28.7	0
0.18,28.8	0
0.18,28.9	0
0.18,29.0	0
0.18,29.1	0
0.18,29.2	0
0.18,29.3	0
0.18,29.4	0
0.18,29.5	0
0.18,29.6	0
0.18,29.7	0
0.18,29.8	0
0.18,29.9	0
0.18,30.0	0
0.18,30.1	0
0.18,30.2	0
0.18,30.3	0
0.18,30.4	0
0.18,30.5	0
0.18,30.6	0
0.18,30.7	0
0.18,30.8	0
0.18,30.9	0
0.18,31.0	0
0.18,31.1	0
0.18,31.2	0
0.18,31.3	0
0.18,31.4	0
0.18,31.5	0
0.18,31.6	0
0.18,31.7	0
0.18,31.8	0
0.18,31.9	0
0.18,32.0	0
0.18,32.1	0
0.18,32.2	0
0.18,32.3	0
0.18,32.4	0
0.18,32.5	0
0.18,32.6	0
0.18,32.7	0

0.18,32.8	0
0.18,32.9	0
0.18,33.0	0
0.18,33.1	0
0.18,33.2	0
0.18,33.3	0
0.18,33.4	0
0.18,33.5	0
0.18,33.6	0
0.18,33.7	0
0.18,33.8	0
0.18,33.9	0
0.18,34.0	0
0.18,34.1	0
0.18,34.2	0
0.18,34.3	0
0.18,34.4	0
0.18,34.5	0
0.18,34.6	0
0.18,34.7	0
0.18,34.8	0
0.18,34.9	0
0.18,35.0	0
0.18,35.1	0
0.18,35.2	0
0.18,35.3	0
0.18,35.4	0
0.18,35.5	0
0.18,35.6	0
0.18,35.7	0
0.18,35.8	0
0.18,35.9	0
0.18,36.0	0
0.18,36.1	0
0.18,36.2	0
0.18,36.3	0
0.18,36.4	0
0.18,36.5	0
0.18,36.6	0
0.18,36.7	0
0.18,36.8	0
0.18,36.9	0

0.18,37.0	0
0.18,37.1	0
0.18,37.2	0
0.18,37.3	0
0.18,37.4	0
0.18,37.5	0
0.18,37.6	0
0.18,37.7	0
0.18,37.8	0
0.18,37.9	0
0.18,38.0	0
0.18,38.1	0
0.18,38.2	0
0.18,38.3	0
0.18,38.4	0
0.18,38.5	0
0.18,38.6	0
0.18,38.7	0
0.18,38.8	0
0.18,38.9	0
0.18,39.0	0
0.18,39.1	0
0.18,39.2	0
0.18,39.3	0
0.18,39.4	0
0.18,39.5	0
0.18,39.6	0
0.18,39.7	0
0.18,39.8	0
0.18,39.9	0
0.18,40.0	0
0.18,40.1	0
0.18,40.2	0
0.18,40.3	0
0.18,40.4	0
0.18,40.5	0
0.18,40.6	0
0.18,40.7	0
0.18,40.8	0
0.18,40.9	0
0.18,41.0	0
0.18,41.1	0

0.18,41.2	0
0.18,41.3	0
0.18,41.4	0
0.18,41.5	0
0.18,41.6	0
0.18,41.7	0
0.18,41.8	0
0.18,41.9	0
0.18,42.0	0
0.18,42.1	0
0.18,42.2	0
0.18,42.3	0
0.18,42.4	0
0.18,42.5	0
0.18,42.6	0
0.18,42.7	0
0.18,42.8	0
0.18,42.9	0
0.18,43.0	0
0.18,43.1	0
0.18,43.2	0
0.18,43.3	0
0.18,43.4	0
0.18,43.5	0
0.18,43.6	0
0.18,43.7	0
0.18,43.8	0
0.18,43.9	0
0.18,44.0	0
0.18,44.1	0
0.18,44.2	0
0.18,44.3	0
0.18,44.4	0
0.18,44.5	0
0.18,44.6	0
0.18,44.7	0
0.18,44.8	0
0.18,44.9	0
0.18,45.0	0
0.18,45.1	0
0.18,45.2	0
0.18,45.3	0

0.18,45.4	0
0.18,45.5	0
0.18,45.6	0
0.18,45.7	0
0.18,45.8	0
0.18,45.9	0
0.18,46.0	0
0.18,46.1	0
0.18,46.2	0
0.18,46.3	0
0.18,46.4	0
0.18,46.5	0
0.18,46.6	0
0.18,46.7	0
0.18,46.8	0
0.18,46.9	0
0.18,47.0	0
0.18,47.1	0
0.18,47.2	0
0.18,47.3	0
0.18,47.4	0
0.18,47.5	0
0.18,47.6	0
0.18,47.7	0
0.18,47.8	0
0.18,47.9	0
0.18,48.0	0
0.18,48.1	0
0.18,48.2	0
0.18,48.3	0
0.18,48.4	0
0.18,48.5	0
0.18,48.6	0
0.18,48.7	0
0.18,48.8	0
0.18,48.9	0
0.18,49.0	0
0.18,49.1	0
0.18,49.2	0
0.18,49.3	0
0.18,49.4	0
0.18,49.5	0

0.18,49.6	0
0.18,49.7	0
0.18,49.8	0
0.18,49.9	0
0.18,50.0	0
0.18,50.1	0
0.18,50.2	0
0.18,50.3	0
0.18,50.4	0
0.18,50.5	0
0.18,50.6	0
0.18,50.7	0
0.18,50.8	0
0.18,50.9	0
0.18,51.0	0
0.18,51.1	0
0.18,51.2	0
0.18,51.3	0
0.18,51.4	0
0.18,51.5	0
0.18,51.6	0
0.18,51.7	0
0.18,51.8	0
0.18,51.9	0
0.18,52.0	0
0.18,52.1	0
0.18,52.2	0
0.18,52.3	0
0.18,52.4	0
0.18,52.5	0
0.18,52.6	0
0.18,52.7	0
0.18,52.8	0
0.18,52.9	0
0.18,53.0	0
0.18,53.1	0
0.18,53.2	0
0.18,53.3	0
0.18,53.4	0
0.18,53.5	0
0.18,53.6	0
0.18,53.7	0

0.18,53.8	0
0.18,53.9	0
0.18,54.0	0
0.18,54.1	0
0.18,54.2	0
0.18,54.3	0
0.18,54.4	0
0.18,54.5	0
0.18,54.6	0
0.18,54.7	0
0.18,54.8	0
0.18,54.9	0
0.18,55.0	0
0.18,55.1	0
0.18,55.2	0
0.18,55.3	0
0.18,55.4	0
0.18,55.5	0
0.18,55.6	0
0.18,55.7	0
0.18,55.8	0
0.18,55.9	0
0.18,56.0	0
0.18,56.1	0
0.18,56.2	0
0.18,56.3	0
0.18,56.4	0
0.18,56.5	0
0.18,56.6	0
0.18,56.7	0
0.18,56.8	0
0.18,56.9	0
0.18,57.0	0
0.18,57.1	0
0.18,57.2	0
0.18,57.3	0
0.18,57.4	0
0.18,57.5	0
0.18,57.6	0
0.18,57.7	0
0.18,57.8	0
0.18,57.9	0

0.18,58.0	0
0.18,58.1	0
0.18,58.2	0
0.18,58.3	0
0.18,58.4	0
0.18,58.5	0
0.18,58.6	0
0.18,58.7	0
0.18,58.8	0
0.18,58.9	0
0.18,59.0	0
0.18,59.1	0
0.18,59.2	0
0.18,59.3	0
0.18,59.4	0
0.18,59.5	0
0.18,59.6	0
0.18,59.7	0
0.18,59.8	0
0.18,59.9	0
0.18,60.0	0
0.18,60.1	0
0.18,60.2	0
0.18,60.3	0
0.18,60.4	0
0.18,60.5	0
0.18,60.6	0
0.18,60.7	0
0.18,60.8	0
0.18,60.9	0
0.18,61.0	0
0.18,61.1	0
0.18,61.2	0
0.18,61.3	0
0.18,61.4	0
0.18,61.5	0
0.18,61.6	0
0.18,61.7	0
0.18,61.8	0
0.18,61.9	0
0.18,62.0	0
0.18,62.1	0

0.18,62.2	0
0.18,62.3	0
0.18,62.4	0
0.18,62.5	0
0.18,62.6	0
0.18,62.7	0
0.18,62.8	0
0.18,62.9	0
0.18,63.0	0
0.18,63.1	0
0.18,63.2	0
0.18,63.3	0
0.18,63.4	0
0.18,63.5	0
0.18,63.6	0
0.18,63.7	0
0.18,63.8	0
0.18,63.9	0
0.18,64.0	0
0.18,64.1	0
0.18,64.2	0
0.18,64.3	0
0.18,64.4	0
0.18,64.5	0
0.18,64.6	0
0.18,64.7	0
0.18,64.8	0
0.18,64.9	0
0.18,65.0	0
0.18,65.1	0
0.18,65.2	0
0.18,65.3	0
0.18,65.4	0
0.18,65.5	0
0.18,65.6	0
0.18,65.7	0
0.18,65.8	0
0.18,65.9	0
0.18,66.0	0
0.18,66.1	0
0.18,66.2	0
0.18,66.3	0

0.18,66.4	0
0.18,66.5	0
0.18,66.6	0
0.18,66.7	0
0.18,66.8	0
0.18,66.9	0
0.18,67.0	0
0.18,67.1	0
0.18,67.2	0
0.18,67.3	0
0.18,67.4	0
0.18,67.5	0
0.18,67.6	0
0.18,67.7	0
0.18,67.8	0
0.18,67.9	0
0.18,68.0	0
0.18,68.1	0
0.18,68.2	0
0.18,68.3	0
0.18,68.4	0
0.18,68.5	0
0.18,68.6	0
0.18,68.7	0
0.18,68.8	0
0.18,68.9	0
0.18,69.0	0
0.18,69.1	0
0.18,69.2	0
0.18,69.3	0
0.18,69.4	0
0.18,69.5	0
0.18,69.6	0
0.18,69.7	0
0.18,69.8	0
0.18,69.9	0
0.18,70.0	0
0.18,70.1	0
0.18,70.2	0
0.18,70.3	0
0.18,70.4	0
0.18,70.5	0

0.18,70.6	0
0.18,70.7	0
0.18,70.8	0
0.18,70.9	0
0.18,71.0	0
0.18,71.1	0
0.18,71.2	0
0.18,71.3	0
0.18,71.4	0
0.18,71.5	0
0.18,71.6	0
0.18,71.7	0
0.18,71.8	0
0.18,71.9	0
0.18,72.0	0
0.18,72.1	0
0.18,72.2	0
0.18,72.3	0
0.18,72.4	0
0.18,72.5	0
0.18,72.6	0
0.18,72.7	0
0.18,72.8	0
0.18,72.9	0
0.18,73.0	0
0.18,73.1	0
0.18,73.2	0
0.18,73.3	0
0.18,73.4	0
0.18,73.5	0
0.18,73.6	0
0.18,73.7	0
0.18,73.8	0
0.18,73.9	0
0.18,74.0	0
0.18,74.1	0
0.18,74.2	0
0.18,74.3	0
0.18,74.4	0
0.18,74.5	0
0.18,74.6	0
0.18,74.7	0

0.18,74.8	0
0.18,74.9	0
0.18,75.0	0
0.18,75.1	0
0.18,75.2	0
0.18,75.3	0
0.18,75.4	0
0.18,75.5	0
0.18,75.6	0
0.18,75.7	0
0.18,75.8	0
0.18,75.9	0
0.18,76.0	0
0.18,76.1	0
0.18,76.2	0
0.18,76.3	0
0.18,76.4	0
0.18,76.5	0
0.18,76.6	0
0.18,76.7	0
0.18,76.8	0
0.18,76.9	0
0.18,77.0	0
0.18,77.1	0
0.18,77.2	0
0.18,77.3	0
0.18,77.4	0
0.18,77.5	0
0.18,77.6	0
0.18,77.7	0
0.18,77.8	0
0.18,77.9	0
0.18,78.0	0
0.18,78.1	0
0.18,78.2	0
0.18,78.3	0
0.18,78.4	0
0.18,78.5	0
0.18,78.6	0
0.18,78.7	0
0.18,78.8	0
0.18,78.9	0

0.18,79.0	0
0.18,79.1	0
0.18,79.2	0
0.18,79.3	0
0.18,79.4	0
0.18,79.5	0
0.18,79.6	0
0.18,79.7	0
0.18,79.8	0
0.18,79.9	0
0.18,80.0	0
0.18,80.1	0
0.18,80.2	0
0.18,80.3	0
0.18,80.4	0
0.18,80.5	0
0.18,80.6	0
0.18,80.7	0
0.18,80.8	0
0.18,80.9	0
0.18,81.0	0
0.18,81.1	0
0.18,81.2	0
0.18,81.3	0
0.18,81.4	0
0.18,81.5	0
0.18,81.6	0
0.18,81.7	0
0.18,81.8	0
0.18,81.9	0
0.18,82.0	0
0.18,82.1	0
0.18,82.2	0
0.18,82.3	0
0.18,82.4	0
0.18,82.5	0
0.18,82.6	0
0.18,82.7	0
0.18,82.8	0
0.18,82.9	0
0.18,83.0	0
0.18,83.1	0

0.18,83.2	0
0.18,83.3	0
0.18,83.4	0
0.18,83.5	0
0.18,83.6	0
0.18,83.7	0
0.18,83.8	0
0.18,83.9	0
0.18,84.0	0
0.18,84.1	0
0.18,84.2	0
0.18,84.3	0
0.18,84.4	0
0.18,84.5	0
0.18,84.6	0
0.18,84.7	0
0.18,84.8	0
0.18,84.9	0
0.18,85.0	0
0.18,85.1	0
0.18,85.2	0
0.18,85.3	0
0.18,85.4	0
0.18,85.5	0
0.18,85.6	0
0.18,85.7	0
0.18,85.8	0
0.18,85.9	0
0.18,86.0	0
0.18,86.1	0
0.18,86.2	0
0.18,86.3	0
0.18,86.4	0
0.18,86.5	0
0.18,86.6	0
0.18,86.7	0
0.18,86.8	0
0.18,86.9	0
0.18,87.0	0
0.18,87.1	0
0.18,87.2	0
0.18,87.3	0

0.18,87.4	0
0.18,87.5	0
0.18,87.6	0
0.18,87.7	0
0.18,87.8	0
0.18,87.9	0
0.18,88.0	0
0.18,88.1	0
0.18,88.2	0
0.18,88.3	0
0.18,88.4	0
0.18,88.5	0
0.18,88.6	0
0.18,88.7	0
0.18,88.8	0
0.18,88.9	0
0.18,89.0	0
0.18,89.1	0
0.18,89.2	0
0.18,89.3	0
0.18,89.4	0
0.18,89.5	0
0.18,89.6	0
0.18,89.7	0
0.18,89.8	0
0.18,89.9	0
0.18,90.0	0
0.18,90.1	0
0.18,90.2	0
0.18,90.3	0
0.18,90.4	0
0.18,90.5	0
0.18,90.6	0
0.18,90.7	0
0.18,90.8	0
0.18,90.9	0
0.18,91.0	0
0.18,91.1	0
0.18,91.2	0
0.18,91.3	0
0.18,91.4	0
0.18,91.5	0

0.18,91.6	0
0.18,91.7	0
0.18,91.8	0
0.18,91.9	0
0.18,92.0	0
0.18,92.1	0
0.18,92.2	0
0.18,92.3	0
0.18,92.4	0
0.18,92.5	0
0.18,92.6	0
0.18,92.7	0
0.18,92.8	0
0.18,92.9	0
0.18,93.0	0
0.18,93.1	0
0.18,93.2	0
0.18,93.3	0
0.18,93.4	0
0.18,93.5	0
0.18,93.6	0
0.18,93.7	0
0.18,93.8	0
0.18,93.9	0
0.18,94.0	0
0.18,94.1	0
0.18,94.2	0
0.18,94.3	0
0.18,94.4	0
0.18,94.5	0
0.18,94.6	0
0.18,94.7	0
0.18,94.8	0
0.18,94.9	0
0.18,95.0	0
0.18,95.1	0
0.18,95.2	0
0.18,95.3	0
0.18,95.4	0
0.18,95.5	0
0.18,95.6	0
0.18,95.7	0

0.18,95.8	0
0.18,95.9	0
0.18,96.0	0
0.18,96.1	0
0.18,96.2	0
0.18,96.3	0
0.18,96.4	0
0.18,96.5	0
0.18,96.6	0
0.18,96.7	0
0.18,96.8	0
0.18,96.9	0
0.18,97.0	0
0.18,97.1	0
0.18,97.2	0
0.18,97.3	0
0.18,97.4	0
0.18,97.5	0
0.18,97.6	0
0.18,97.7	0
0.18,97.8	0
0.18,97.9	0
0.18,98.0	0
0.18,98.1	0
0.18,98.2	0
0.18,98.3	0
0.18,98.4	0
0.18,98.5	0
0.18,98.6	0
0.18,98.7	0
0.18,98.8	0
0.18,98.9	0
0.18,99.0	0
0.18,99.1	0
0.18,99.2	0
0.18,99.3	0
0.18,99.4	0
0.18,99.5	0
0.18,99.6	0
0.18,99.7	0
0.18,99.8	0
0.18,99.9	0

0.18,100.0	0
0.19,0.0	0
0.19,0.1	0
0.19,0.2	0
0.19,0.3	0
0.19,0.4	0
0.19,0.5	0
0.19,0.6	0
0.19,0.7	0
0.19,0.8	0
0.19,0.9	0
0.19,1.0	0
0.19,1.1	0
0.19,1.2	0
0.19,1.3	0
0.19,1.4	0
0.19,1.5	0
0.19,1.6	0
0.19,1.7	0
0.19,1.8	0
0.19,1.9	0
0.19,2.0	0
0.19,2.1	0
0.19,2.2	0
0.19,2.3	0
0.19,2.4	0
0.19,2.5	0
0.19,2.6	0
0.19,2.7	0
0.19,2.8	0
0.19,2.9	0
0.19,3.0	0
0.19,3.1	0
0.19,3.2	0
0.19,3.3	0
0.19,3.4	0
0.19,3.5	0
0.19,3.6	0
0.19,3.7	0
0.19,3.8	0
0.19,3.9	0
0.19,4.0	0

0.19,4.1	0
0.19,4.2	0
0.19,4.3	0
0.19,4.4	0
0.19,4.5	0
0.19,4.6	0
0.19,4.7	0
0.19,4.8	0
0.19,4.9	0
0.19,5.0	0
0.19,5.1	0
0.19,5.2	0
0.19,5.3	0
0.19,5.4	0
0.19,5.5	0
0.19,5.6	0
0.19,5.7	0
0.19,5.8	0
0.19,5.9	0
0.19,6.0	0
0.19,6.1	0
0.19,6.2	0
0.19,6.3	0
0.19,6.4	0
0.19,6.5	0
0.19,6.6	0
0.19,6.7	0
0.19,6.8	0
0.19,6.9	0
0.19,7.0	0
0.19,7.1	0
0.19,7.2	0
0.19,7.3	0
0.19,7.4	0
0.19,7.5	0
0.19,7.6	0
0.19,7.7	0
0.19,7.8	0
0.19,7.9	0
0.19,8.0	0
0.19,8.1	0
0.19,8.2	0

0.19,8.3	0
0.19,8.4	0
0.19,8.5	0
0.19,8.6	0
0.19,8.7	0
0.19,8.8	0
0.19,8.9	0
0.19,9.0	0
0.19,9.1	0
0.19,9.2	0
0.19,9.3	0
0.19,9.4	0
0.19,9.5	0
0.19,9.6	0
0.19,9.7	0
0.19,9.8	0
0.19,9.9	0
0.19,10.0	0
0.19,10.1	0
0.19,10.2	0
0.19,10.3	0
0.19,10.4	0
0.19,10.5	0
0.19,10.6	0
0.19,10.7	0
0.19,10.8	0
0.19,10.9	0
0.19,11.0	0
0.19,11.1	0
0.19,11.2	0
0.19,11.3	0
0.19,11.4	0
0.19,11.5	0
0.19,11.6	0
0.19,11.7	0
0.19,11.8	0
0.19,11.9	0
0.19,12.0	0
0.19,12.1	0
0.19,12.2	0
0.19,12.3	0
0.19,12.4	0

0.19,12.5	0
0.19,12.6	0
0.19,12.7	0
0.19,12.8	0
0.19,12.9	0
0.19,13.0	0
0.19,13.1	0
0.19,13.2	0
0.19,13.3	0
0.19,13.4	0
0.19,13.5	0
0.19,13.6	0
0.19,13.7	0
0.19,13.8	0
0.19,13.9	0
0.19,14.0	0
0.19,14.1	0
0.19,14.2	0
0.19,14.3	0
0.19,14.4	0
0.19,14.5	0
0.19,14.6	0
0.19,14.7	0
0.19,14.8	0
0.19,14.9	0
0.19,15.0	0
0.19,15.1	0
0.19,15.2	0
0.19,15.3	0
0.19,15.4	0
0.19,15.5	0
0.19,15.6	0
0.19,15.7	0
0.19,15.8	0
0.19,15.9	0
0.19,16.0	0
0.19,16.1	0
0.19,16.2	0
0.19,16.3	0
0.19,16.4	0
0.19,16.5	0
0.19,16.6	0

0.19,16.7	0
0.19,16.8	0
0.19,16.9	0
0.19,17.0	0
0.19,17.1	0
0.19,17.2	0
0.19,17.3	0
0.19,17.4	0
0.19,17.5	0
0.19,17.6	0
0.19,17.7	0
0.19,17.8	0
0.19,17.9	0
0.19,18.0	0
0.19,18.1	0
0.19,18.2	0
0.19,18.3	0
0.19,18.4	0
0.19,18.5	0
0.19,18.6	0
0.19,18.7	0
0.19,18.8	0
0.19,18.9	0
0.19,19.0	0
0.19,19.1	0
0.19,19.2	0
0.19,19.3	0
0.19,19.4	0
0.19,19.5	0
0.19,19.6	0
0.19,19.7	0
0.19,19.8	0
0.19,19.9	0
0.19,20.0	0
0.19,20.1	0
0.19,20.2	0
0.19,20.3	0
0.19,20.4	0
0.19,20.5	0
0.19,20.6	0
0.19,20.7	0
0.19,20.8	0

0.19,20.9	0
0.19,21.0	0
0.19,21.1	0
0.19,21.2	0
0.19,21.3	0
0.19,21.4	0
0.19,21.5	0
0.19,21.6	0
0.19,21.7	0
0.19,21.8	0
0.19,21.9	0
0.19,22.0	0
0.19,22.1	0
0.19,22.2	0
0.19,22.3	0
0.19,22.4	0
0.19,22.5	0
0.19,22.6	0
0.19,22.7	0
0.19,22.8	0
0.19,22.9	0
0.19,23.0	0
0.19,23.1	0
0.19,23.2	0
0.19,23.3	0
0.19,23.4	0
0.19,23.5	0
0.19,23.6	0
0.19,23.7	0
0.19,23.8	0
0.19,23.9	0
0.19,24.0	0
0.19,24.1	0
0.19,24.2	0
0.19,24.3	0
0.19,24.4	0
0.19,24.5	0
0.19,24.6	0
0.19,24.7	0
0.19,24.8	0
0.19,24.9	0
0.19,25.0	0

0.19,25.1	0
0.19,25.2	0
0.19,25.3	0
0.19,25.4	0
0.19,25.5	0
0.19,25.6	0
0.19,25.7	0
0.19,25.8	0
0.19,25.9	0
0.19,26.0	0
0.19,26.1	0
0.19,26.2	0
0.19,26.3	0
0.19,26.4	0
0.19,26.5	0
0.19,26.6	0
0.19,26.7	0
0.19,26.8	0
0.19,26.9	0
0.19,27.0	0
0.19,27.1	0
0.19,27.2	0
0.19,27.3	0
0.19,27.4	0
0.19,27.5	0
0.19,27.6	0
0.19,27.7	0
0.19,27.8	0
0.19,27.9	0
0.19,28.0	0
0.19,28.1	0
0.19,28.2	0
0.19,28.3	0
0.19,28.4	0
0.19,28.5	0
0.19,28.6	0
0.19,28.7	0
0.19,28.8	0
0.19,28.9	0
0.19,29.0	0
0.19,29.1	0
0.19,29.2	0

0.19,29.3	0
0.19,29.4	0
0.19,29.5	0
0.19,29.6	0
0.19,29.7	0
0.19,29.8	0
0.19,29.9	0
0.19,30.0	0
0.19,30.1	0
0.19,30.2	0
0.19,30.3	0
0.19,30.4	0
0.19,30.5	0
0.19,30.6	0
0.19,30.7	0
0.19,30.8	0
0.19,30.9	0
0.19,31.0	0
0.19,31.1	0
0.19,31.2	0
0.19,31.3	0
0.19,31.4	0
0.19,31.5	0
0.19,31.6	0
0.19,31.7	0
0.19,31.8	0
0.19,31.9	0
0.19,32.0	0
0.19,32.1	0
0.19,32.2	0
0.19,32.3	0
0.19,32.4	0
0.19,32.5	0
0.19,32.6	0
0.19,32.7	0
0.19,32.8	0
0.19,32.9	0
0.19,33.0	0
0.19,33.1	0
0.19,33.2	0
0.19,33.3	0
0.19,33.4	0

0.19,33.5	0
0.19,33.6	0
0.19,33.7	0
0.19,33.8	0
0.19,33.9	0
0.19,34.0	0
0.19,34.1	0
0.19,34.2	0
0.19,34.3	0
0.19,34.4	0
0.19,34.5	0
0.19,34.6	0
0.19,34.7	0
0.19,34.8	0
0.19,34.9	0
0.19,35.0	0
0.19,35.1	0
0.19,35.2	0
0.19,35.3	0
0.19,35.4	0
0.19,35.5	0
0.19,35.6	0
0.19,35.7	0
0.19,35.8	0
0.19,35.9	0
0.19,36.0	0
0.19,36.1	0
0.19,36.2	0
0.19,36.3	0
0.19,36.4	0
0.19,36.5	0
0.19,36.6	0
0.19,36.7	0
0.19,36.8	0
0.19,36.9	0
0.19,37.0	0
0.19,37.1	0
0.19,37.2	0
0.19,37.3	0
0.19,37.4	0
0.19,37.5	0
0.19,37.6	0

0.19,37.7	0
0.19,37.8	0
0.19,37.9	0
0.19,38.0	0
0.19,38.1	0
0.19,38.2	0
0.19,38.3	0
0.19,38.4	0
0.19,38.5	0
0.19,38.6	0
0.19,38.7	0
0.19,38.8	0
0.19,38.9	0
0.19,39.0	0
0.19,39.1	0
0.19,39.2	0
0.19,39.3	0
0.19,39.4	0
0.19,39.5	0
0.19,39.6	0
0.19,39.7	0
0.19,39.8	0
0.19,39.9	0
0.19,40.0	0
0.19,40.1	0
0.19,40.2	0
0.19,40.3	0
0.19,40.4	0
0.19,40.5	0
0.19,40.6	0
0.19,40.7	0
0.19,40.8	0
0.19,40.9	0
0.19,41.0	0
0.19,41.1	0
0.19,41.2	0
0.19,41.3	0
0.19,41.4	0
0.19,41.5	0
0.19,41.6	0
0.19,41.7	0
0.19,41.8	0

0.19,41.9	0
0.19,42.0	0
0.19,42.1	0
0.19,42.2	0
0.19,42.3	0
0.19,42.4	0
0.19,42.5	0
0.19,42.6	0
0.19,42.7	0
0.19,42.8	0
0.19,42.9	0
0.19,43.0	0
0.19,43.1	0
0.19,43.2	0
0.19,43.3	0
0.19,43.4	0
0.19,43.5	0
0.19,43.6	0
0.19,43.7	0
0.19,43.8	0
0.19,43.9	0
0.19,44.0	0
0.19,44.1	0
0.19,44.2	0
0.19,44.3	0
0.19,44.4	0
0.19,44.5	0
0.19,44.6	0
0.19,44.7	0
0.19,44.8	0
0.19,44.9	0
0.19,45.0	0
0.19,45.1	0
0.19,45.2	0
0.19,45.3	0
0.19,45.4	0
0.19,45.5	0
0.19,45.6	0
0.19,45.7	0
0.19,45.8	0
0.19,45.9	0
0.19,46.0	0

0.19,46.1	0
0.19,46.2	0
0.19,46.3	0
0.19,46.4	0
0.19,46.5	0
0.19,46.6	0
0.19,46.7	0
0.19,46.8	0
0.19,46.9	0
0.19,47.0	0
0.19,47.1	0
0.19,47.2	0
0.19,47.3	0
0.19,47.4	0
0.19,47.5	0
0.19,47.6	0
0.19,47.7	0
0.19,47.8	0
0.19,47.9	0
0.19,48.0	0
0.19,48.1	0
0.19,48.2	0
0.19,48.3	0
0.19,48.4	0
0.19,48.5	0
0.19,48.6	0
0.19,48.7	0
0.19,48.8	0
0.19,48.9	0
0.19,49.0	0
0.19,49.1	0
0.19,49.2	0
0.19,49.3	0
0.19,49.4	0
0.19,49.5	0
0.19,49.6	0
0.19,49.7	0
0.19,49.8	0
0.19,49.9	0
0.19,50.0	0
0.19,50.1	0
0.19,50.2	0

0.19,50.3	0
0.19,50.4	0
0.19,50.5	0
0.19,50.6	0
0.19,50.7	0
0.19,50.8	0
0.19,50.9	0
0.19,51.0	0
0.19,51.1	0
0.19,51.2	0
0.19,51.3	0
0.19,51.4	0
0.19,51.5	0
0.19,51.6	0
0.19,51.7	0
0.19,51.8	0
0.19,51.9	0
0.19,52.0	0
0.19,52.1	0
0.19,52.2	0
0.19,52.3	0
0.19,52.4	0
0.19,52.5	0
0.19,52.6	0
0.19,52.7	0
0.19,52.8	0
0.19,52.9	0
0.19,53.0	0
0.19,53.1	0
0.19,53.2	0
0.19,53.3	0
0.19,53.4	0
0.19,53.5	0
0.19,53.6	0
0.19,53.7	0
0.19,53.8	0
0.19,53.9	0
0.19,54.0	0
0.19,54.1	0
0.19,54.2	0
0.19,54.3	0
0.19,54.4	0

0.19,54.5	0
0.19,54.6	0
0.19,54.7	0
0.19,54.8	0
0.19,54.9	0
0.19,55.0	0
0.19,55.1	0
0.19,55.2	0
0.19,55.3	0
0.19,55.4	0
0.19,55.5	0
0.19,55.6	0
0.19,55.7	0
0.19,55.8	0
0.19,55.9	0
0.19,56.0	0
0.19,56.1	0
0.19,56.2	0
0.19,56.3	0
0.19,56.4	0
0.19,56.5	0
0.19,56.6	0
0.19,56.7	0
0.19,56.8	0
0.19,56.9	0
0.19,57.0	0
0.19,57.1	0
0.19,57.2	0
0.19,57.3	0
0.19,57.4	0
0.19,57.5	0
0.19,57.6	0
0.19,57.7	0
0.19,57.8	0
0.19,57.9	0
0.19,58.0	0
0.19,58.1	0
0.19,58.2	0
0.19,58.3	0
0.19,58.4	0
0.19,58.5	0
0.19,58.6	0

0.19,58.7	0
0.19,58.8	0
0.19,58.9	0
0.19,59.0	0
0.19,59.1	0
0.19,59.2	0
0.19,59.3	0
0.19,59.4	0
0.19,59.5	0
0.19,59.6	0
0.19,59.7	0
0.19,59.8	0
0.19,59.9	0
0.19,60.0	0
0.19,60.1	0
0.19,60.2	0
0.19,60.3	0
0.19,60.4	0
0.19,60.5	0
0.19,60.6	0
0.19,60.7	0
0.19,60.8	0
0.19,60.9	0
0.19,61.0	0
0.19,61.1	0
0.19,61.2	0
0.19,61.3	0
0.19,61.4	0
0.19,61.5	0
0.19,61.6	0
0.19,61.7	0
0.19,61.8	0
0.19,61.9	0
0.19,62.0	0
0.19,62.1	0
0.19,62.2	0
0.19,62.3	0
0.19,62.4	0
0.19,62.5	0
0.19,62.6	0
0.19,62.7	0
0.19,62.8	0

0.19,62.9	0
0.19,63.0	0
0.19,63.1	0
0.19,63.2	0
0.19,63.3	0
0.19,63.4	0
0.19,63.5	0
0.19,63.6	0
0.19,63.7	0
0.19,63.8	0
0.19,63.9	0
0.19,64.0	0
0.19,64.1	0
0.19,64.2	0
0.19,64.3	0
0.19,64.4	0
0.19,64.5	0
0.19,64.6	0
0.19,64.7	0
0.19,64.8	0
0.19,64.9	0
0.19,65.0	0
0.19,65.1	0
0.19,65.2	0
0.19,65.3	0
0.19,65.4	0
0.19,65.5	0
0.19,65.6	0
0.19,65.7	0
0.19,65.8	0
0.19,65.9	0
0.19,66.0	0
0.19,66.1	0
0.19,66.2	0
0.19,66.3	0
0.19,66.4	0
0.19,66.5	0
0.19,66.6	0
0.19,66.7	0
0.19,66.8	0
0.19,66.9	0
0.19,67.0	0

0.19,67.1	0
0.19,67.2	0
0.19,67.3	0
0.19,67.4	0
0.19,67.5	0
0.19,67.6	0
0.19,67.7	0
0.19,67.8	0
0.19,67.9	0
0.19,68.0	0
0.19,68.1	0
0.19,68.2	0
0.19,68.3	0
0.19,68.4	0
0.19,68.5	0
0.19,68.6	0
0.19,68.7	0
0.19,68.8	0
0.19,68.9	0
0.19,69.0	0
0.19,69.1	0
0.19,69.2	0
0.19,69.3	0
0.19,69.4	0
0.19,69.5	0
0.19,69.6	0
0.19,69.7	0
0.19,69.8	0
0.19,69.9	0
0.19,70.0	0
0.19,70.1	0
0.19,70.2	0
0.19,70.3	0
0.19,70.4	0
0.19,70.5	0
0.19,70.6	0
0.19,70.7	0
0.19,70.8	0
0.19,70.9	0
0.19,71.0	0
0.19,71.1	0
0.19,71.2	0

0.19,71.3	0
0.19,71.4	0
0.19,71.5	0
0.19,71.6	0
0.19,71.7	0
0.19,71.8	0
0.19,71.9	0
0.19,72.0	0
0.19,72.1	0
0.19,72.2	0
0.19,72.3	0
0.19,72.4	0
0.19,72.5	0
0.19,72.6	0
0.19,72.7	0
0.19,72.8	0
0.19,72.9	0
0.19,73.0	0
0.19,73.1	0
0.19,73.2	0
0.19,73.3	0
0.19,73.4	0
0.19,73.5	0
0.19,73.6	0
0.19,73.7	0
0.19,73.8	0
0.19,73.9	0
0.19,74.0	0
0.19,74.1	0
0.19,74.2	0
0.19,74.3	0
0.19,74.4	0
0.19,74.5	0
0.19,74.6	0
0.19,74.7	0
0.19,74.8	0
0.19,74.9	0
0.19,75.0	0
0.19,75.1	0
0.19,75.2	0
0.19,75.3	0
0.19,75.4	0

0.19,75.5	0
0.19,75.6	0
0.19,75.7	0
0.19,75.8	0
0.19,75.9	0
0.19,76.0	0
0.19,76.1	0
0.19,76.2	0
0.19,76.3	0
0.19,76.4	0
0.19,76.5	0
0.19,76.6	0
0.19,76.7	0
0.19,76.8	0
0.19,76.9	0
0.19,77.0	0
0.19,77.1	0
0.19,77.2	0
0.19,77.3	0
0.19,77.4	0
0.19,77.5	0
0.19,77.6	0
0.19,77.7	0
0.19,77.8	0
0.19,77.9	0
0.19,78.0	0
0.19,78.1	0
0.19,78.2	0
0.19,78.3	0
0.19,78.4	0
0.19,78.5	0
0.19,78.6	0
0.19,78.7	0
0.19,78.8	0
0.19,78.9	0
0.19,79.0	0
0.19,79.1	0
0.19,79.2	0
0.19,79.3	0
0.19,79.4	0
0.19,79.5	0
0.19,79.6	0

0.19,79.7	0
0.19,79.8	0
0.19,79.9	0
0.19,80.0	0
0.19,80.1	0
0.19,80.2	0
0.19,80.3	0
0.19,80.4	0
0.19,80.5	0
0.19,80.6	0
0.19,80.7	0
0.19,80.8	0
0.19,80.9	0
0.19,81.0	0
0.19,81.1	0
0.19,81.2	0
0.19,81.3	0
0.19,81.4	0
0.19,81.5	0
0.19,81.6	0
0.19,81.7	0
0.19,81.8	0
0.19,81.9	0
0.19,82.0	0
0.19,82.1	0
0.19,82.2	0
0.19,82.3	0
0.19,82.4	0
0.19,82.5	0
0.19,82.6	0
0.19,82.7	0
0.19,82.8	0
0.19,82.9	0
0.19,83.0	0
0.19,83.1	0
0.19,83.2	0
0.19,83.3	0
0.19,83.4	0
0.19,83.5	0
0.19,83.6	0
0.19,83.7	0
0.19,83.8	0

0.19,83.9	0
0.19,84.0	0
0.19,84.1	0
0.19,84.2	0
0.19,84.3	0
0.19,84.4	0
0.19,84.5	0
0.19,84.6	0
0.19,84.7	0
0.19,84.8	0
0.19,84.9	0
0.19,85.0	0
0.19,85.1	0
0.19,85.2	0
0.19,85.3	0
0.19,85.4	0
0.19,85.5	0
0.19,85.6	0
0.19,85.7	0
0.19,85.8	0
0.19,85.9	0
0.19,86.0	0
0.19,86.1	0
0.19,86.2	0
0.19,86.3	0
0.19,86.4	0
0.19,86.5	0
0.19,86.6	0
0.19,86.7	0
0.19,86.8	0
0.19,86.9	0
0.19,87.0	0
0.19,87.1	0
0.19,87.2	0
0.19,87.3	0
0.19,87.4	0
0.19,87.5	0
0.19,87.6	0
0.19,87.7	0
0.19,87.8	0
0.19,87.9	0
0.19,88.0	0

0.19,88.1	0
0.19,88.2	0
0.19,88.3	0
0.19,88.4	0
0.19,88.5	0
0.19,88.6	0
0.19,88.7	0
0.19,88.8	0
0.19,88.9	0
0.19,89.0	0
0.19,89.1	0
0.19,89.2	0
0.19,89.3	0
0.19,89.4	0
0.19,89.5	0
0.19,89.6	0
0.19,89.7	0
0.19,89.8	0
0.19,89.9	0
0.19,90.0	0
0.19,90.1	0
0.19,90.2	0
0.19,90.3	0
0.19,90.4	0
0.19,90.5	0
0.19,90.6	0
0.19,90.7	0
0.19,90.8	0
0.19,90.9	0
0.19,91.0	0
0.19,91.1	0
0.19,91.2	0
0.19,91.3	0
0.19,91.4	0
0.19,91.5	0
0.19,91.6	0
0.19,91.7	0
0.19,91.8	0
0.19,91.9	0
0.19,92.0	0
0.19,92.1	0
0.19,92.2	0

0.19,92.3	0
0.19,92.4	0
0.19,92.5	0
0.19,92.6	0
0.19,92.7	0
0.19,92.8	0
0.19,92.9	0
0.19,93.0	0
0.19,93.1	0
0.19,93.2	0
0.19,93.3	0
0.19,93.4	0
0.19,93.5	0
0.19,93.6	0
0.19,93.7	0
0.19,93.8	0
0.19,93.9	0
0.19,94.0	0
0.19,94.1	0
0.19,94.2	0
0.19,94.3	0
0.19,94.4	0
0.19,94.5	0
0.19,94.6	0
0.19,94.7	0
0.19,94.8	0
0.19,94.9	0
0.19,95.0	0
0.19,95.1	0
0.19,95.2	0
0.19,95.3	0
0.19,95.4	0
0.19,95.5	0
0.19,95.6	0
0.19,95.7	0
0.19,95.8	0
0.19,95.9	0
0.19,96.0	0
0.19,96.1	0
0.19,96.2	0
0.19,96.3	0
0.19,96.4	0

0.19,96.5	0
0.19,96.6	0
0.19,96.7	0
0.19,96.8	0
0.19,96.9	0
0.19,97.0	0
0.19,97.1	0
0.19,97.2	0
0.19,97.3	0
0.19,97.4	0
0.19,97.5	0
0.19,97.6	0
0.19,97.7	0
0.19,97.8	0
0.19,97.9	0
0.19,98.0	0
0.19,98.1	0
0.19,98.2	0
0.19,98.3	0
0.19,98.4	0
0.19,98.5	0
0.19,98.6	0
0.19,98.7	0
0.19,98.8	0
0.19,98.9	0
0.19,99.0	0
0.19,99.1	0
0.19,99.2	0
0.19,99.3	0
0.19,99.4	0
0.19,99.5	0
0.19,99.6	0
0.19,99.7	0
0.19,99.8	0
0.19,99.9	0
0.19,100.0	0
0.20,0.0	0
0.20,0.1	0
0.20,0.2	0
0.20,0.3	0
0.20,0.4	0
0.20,0.5	0

0.20,0.6	0
0.20,0.7	0
0.20,0.8	0
0.20,0.9	0
0.20,1.0	0
0.20,1.1	0
0.20,1.2	0
0.20,1.3	0
0.20,1.4	0
0.20,1.5	0
0.20,1.6	0
0.20,1.7	0
0.20,1.8	0
0.20,1.9	0
0.20,2.0	0
0.20,2.1	0
0.20,2.2	0
0.20,2.3	0
0.20,2.4	0
0.20,2.5	0
0.20,2.6	0
0.20,2.7	0
0.20,2.8	0
0.20,2.9	0
0.20,3.0	0
0.20,3.1	0
0.20,3.2	0
0.20,3.3	0
0.20,3.4	0
0.20,3.5	0
0.20,3.6	0
0.20,3.7	0
0.20,3.8	0
0.20,3.9	0
0.20,4.0	0
0.20,4.1	0
0.20,4.2	0
0.20,4.3	0
0.20,4.4	0
0.20,4.5	0
0.20,4.6	0
0.20,4.7	0

0.20,4.8	0
0.20,4.9	0
0.20,5.0	0
0.20,5.1	0
0.20,5.2	0
0.20,5.3	0
0.20,5.4	0
0.20,5.5	0
0.20,5.6	0
0.20,5.7	0
0.20,5.8	0
0.20,5.9	0
0.20,6.0	0
0.20,6.1	0
0.20,6.2	0
0.20,6.3	0
0.20,6.4	0
0.20,6.5	0
0.20,6.6	0
0.20,6.7	0
0.20,6.8	0
0.20,6.9	0
0.20,7.0	0
0.20,7.1	0
0.20,7.2	0
0.20,7.3	0
0.20,7.4	0
0.20,7.5	0
0.20,7.6	0
0.20,7.7	0
0.20,7.8	0
0.20,7.9	0
0.20,8.0	0
0.20,8.1	0
0.20,8.2	0
0.20,8.3	0
0.20,8.4	0
0.20,8.5	0
0.20,8.6	0
0.20,8.7	0
0.20,8.8	0
0.20,8.9	0

0.20,9.0	0
0.20,9.1	0
0.20,9.2	0
0.20,9.3	0
0.20,9.4	0
0.20,9.5	0
0.20,9.6	0
0.20,9.7	0
0.20,9.8	0
0.20,9.9	0
0.20,10.0	0
0.20,10.1	0
0.20,10.2	0
0.20,10.3	0
0.20,10.4	0
0.20,10.5	0
0.20,10.6	0
0.20,10.7	0
0.20,10.8	0
0.20,10.9	0
0.20,11.0	0
0.20,11.1	0
0.20,11.2	0
0.20,11.3	0
0.20,11.4	0
0.20,11.5	0
0.20,11.6	0
0.20,11.7	0
0.20,11.8	0
0.20,11.9	0
0.20,12.0	0
0.20,12.1	0
0.20,12.2	0
0.20,12.3	0
0.20,12.4	0
0.20,12.5	0
0.20,12.6	0
0.20,12.7	0
0.20,12.8	0
0.20,12.9	0
0.20,13.0	0
0.20,13.1	0

0.20,13.2	0
0.20,13.3	0
0.20,13.4	0
0.20,13.5	0
0.20,13.6	0
0.20,13.7	0
0.20,13.8	0
0.20,13.9	0
0.20,14.0	0
0.20,14.1	0
0.20,14.2	0
0.20,14.3	0
0.20,14.4	0
0.20,14.5	0
0.20,14.6	0
0.20,14.7	0
0.20,14.8	0
0.20,14.9	0
0.20,15.0	0
0.20,15.1	0
0.20,15.2	0
0.20,15.3	0
0.20,15.4	0
0.20,15.5	0
0.20,15.6	0
0.20,15.7	0
0.20,15.8	0
0.20,15.9	0
0.20,16.0	0
0.20,16.1	0
0.20,16.2	0
0.20,16.3	0
0.20,16.4	0
0.20,16.5	0
0.20,16.6	0
0.20,16.7	0
0.20,16.8	0
0.20,16.9	0
0.20,17.0	0
0.20,17.1	0
0.20,17.2	0
0.20,17.3	0

0.20,17.4	0
0.20,17.5	0
0.20,17.6	0
0.20,17.7	0
0.20,17.8	0
0.20,17.9	0
0.20,18.0	0
0.20,18.1	0
0.20,18.2	0
0.20,18.3	0
0.20,18.4	0
0.20,18.5	0
0.20,18.6	0
0.20,18.7	0
0.20,18.8	0
0.20,18.9	0
0.20,19.0	0
0.20,19.1	0
0.20,19.2	0
0.20,19.3	0
0.20,19.4	0
0.20,19.5	0
0.20,19.6	0
0.20,19.7	0
0.20,19.8	0
0.20,19.9	0
0.20,20.0	0
0.20,20.1	0
0.20,20.2	0
0.20,20.3	0
0.20,20.4	0
0.20,20.5	0
0.20,20.6	0
0.20,20.7	0
0.20,20.8	0
0.20,20.9	0
0.20,21.0	0
0.20,21.1	0
0.20,21.2	0
0.20,21.3	0
0.20,21.4	0
0.20,21.5	0

0.20,21.6	0
0.20,21.7	0
0.20,21.8	0
0.20,21.9	0
0.20,22.0	0
0.20,22.1	0
0.20,22.2	0
0.20,22.3	0
0.20,22.4	0
0.20,22.5	0
0.20,22.6	0
0.20,22.7	0
0.20,22.8	0
0.20,22.9	0
0.20,23.0	0
0.20,23.1	0
0.20,23.2	0
0.20,23.3	0
0.20,23.4	0
0.20,23.5	0
0.20,23.6	0
0.20,23.7	0
0.20,23.8	0
0.20,23.9	0
0.20,24.0	0
0.20,24.1	0
0.20,24.2	0
0.20,24.3	0
0.20,24.4	0
0.20,24.5	0
0.20,24.6	0
0.20,24.7	0
0.20,24.8	0
0.20,24.9	0
0.20,25.0	0
0.20,25.1	0
0.20,25.2	0
0.20,25.3	0
0.20,25.4	0
0.20,25.5	0
0.20,25.6	0
0.20,25.7	0

0.20,25.8	0
0.20,25.9	0
0.20,26.0	0
0.20,26.1	0
0.20,26.2	0
0.20,26.3	0
0.20,26.4	0
0.20,26.5	0
0.20,26.6	0
0.20,26.7	0
0.20,26.8	0
0.20,26.9	0
0.20,27.0	0
0.20,27.1	0
0.20,27.2	0
0.20,27.3	0
0.20,27.4	0
0.20,27.5	0
0.20,27.6	0
0.20,27.7	0
0.20,27.8	0
0.20,27.9	0
0.20,28.0	0
0.20,28.1	0
0.20,28.2	0
0.20,28.3	0
0.20,28.4	0
0.20,28.5	0
0.20,28.6	0
0.20,28.7	0
0.20,28.8	0
0.20,28.9	0
0.20,29.0	0
0.20,29.1	0
0.20,29.2	0
0.20,29.3	0
0.20,29.4	0
0.20,29.5	0
0.20,29.6	0
0.20,29.7	0
0.20,29.8	0
0.20,29.9	0

0.20,30.0	0
0.20,30.1	0
0.20,30.2	0
0.20,30.3	0
0.20,30.4	0
0.20,30.5	0
0.20,30.6	0
0.20,30.7	0
0.20,30.8	0
0.20,30.9	0
0.20,31.0	0
0.20,31.1	0
0.20,31.2	0
0.20,31.3	0
0.20,31.4	0
0.20,31.5	0
0.20,31.6	0
0.20,31.7	0
0.20,31.8	0
0.20,31.9	0
0.20,32.0	0
0.20,32.1	0
0.20,32.2	0
0.20,32.3	0
0.20,32.4	0
0.20,32.5	0
0.20,32.6	0
0.20,32.7	0
0.20,32.8	0
0.20,32.9	0
0.20,33.0	0
0.20,33.1	0
0.20,33.2	0
0.20,33.3	0
0.20,33.4	0
0.20,33.5	0
0.20,33.6	0
0.20,33.7	0
0.20,33.8	0
0.20,33.9	0
0.20,34.0	0
0.20,34.1	0

0.20,34.2	0
0.20,34.3	0
0.20,34.4	0
0.20,34.5	0
0.20,34.6	0
0.20,34.7	0
0.20,34.8	0
0.20,34.9	0
0.20,35.0	0
0.20,35.1	0
0.20,35.2	0
0.20,35.3	0
0.20,35.4	0
0.20,35.5	0
0.20,35.6	0
0.20,35.7	0
0.20,35.8	0
0.20,35.9	0
0.20,36.0	0
0.20,36.1	0
0.20,36.2	0
0.20,36.3	0
0.20,36.4	0
0.20,36.5	0
0.20,36.6	0
0.20,36.7	0
0.20,36.8	0
0.20,36.9	0
0.20,37.0	0
0.20,37.1	0
0.20,37.2	0
0.20,37.3	0
0.20,37.4	0
0.20,37.5	0
0.20,37.6	0
0.20,37.7	0
0.20,37.8	0
0.20,37.9	0
0.20,38.0	0
0.20,38.1	0
0.20,38.2	0
0.20,38.3	0

0.20,38.4	0
0.20,38.5	0
0.20,38.6	0
0.20,38.7	0
0.20,38.8	0
0.20,38.9	0
0.20,39.0	0
0.20,39.1	0
0.20,39.2	0
0.20,39.3	0
0.20,39.4	0
0.20,39.5	0
0.20,39.6	0
0.20,39.7	0
0.20,39.8	0
0.20,39.9	0
0.20,40.0	0
0.20,40.1	0
0.20,40.2	0
0.20,40.3	0
0.20,40.4	0
0.20,40.5	0
0.20,40.6	0
0.20,40.7	0
0.20,40.8	0
0.20,40.9	0
0.20,41.0	0
0.20,41.1	0
0.20,41.2	0
0.20,41.3	0
0.20,41.4	0
0.20,41.5	0
0.20,41.6	0
0.20,41.7	0
0.20,41.8	0
0.20,41.9	0
0.20,42.0	0
0.20,42.1	0
0.20,42.2	0
0.20,42.3	0
0.20,42.4	0
0.20,42.5	0

0.20,42.6	0
0.20,42.7	0
0.20,42.8	0
0.20,42.9	0
0.20,43.0	0
0.20,43.1	0
0.20,43.2	0
0.20,43.3	0
0.20,43.4	0
0.20,43.5	0
0.20,43.6	0
0.20,43.7	0
0.20,43.8	0
0.20,43.9	0
0.20,44.0	0
0.20,44.1	0
0.20,44.2	0
0.20,44.3	0
0.20,44.4	0
0.20,44.5	0
0.20,44.6	0
0.20,44.7	0
0.20,44.8	0
0.20,44.9	0
0.20,45.0	0
0.20,45.1	0
0.20,45.2	0
0.20,45.3	0
0.20,45.4	0
0.20,45.5	0
0.20,45.6	0
0.20,45.7	0
0.20,45.8	0
0.20,45.9	0
0.20,46.0	0
0.20,46.1	0
0.20,46.2	0
0.20,46.3	0
0.20,46.4	0
0.20,46.5	0
0.20,46.6	0
0.20,46.7	0

0.20,46.8	0
0.20,46.9	0
0.20,47.0	0
0.20,47.1	0
0.20,47.2	0
0.20,47.3	0
0.20,47.4	0
0.20,47.5	0
0.20,47.6	0
0.20,47.7	0
0.20,47.8	0
0.20,47.9	0
0.20,48.0	0
0.20,48.1	0
0.20,48.2	0
0.20,48.3	0
0.20,48.4	0
0.20,48.5	0
0.20,48.6	0
0.20,48.7	0
0.20,48.8	0
0.20,48.9	0
0.20,49.0	0
0.20,49.1	0
0.20,49.2	0
0.20,49.3	0
0.20,49.4	0
0.20,49.5	0
0.20,49.6	0
0.20,49.7	0
0.20,49.8	0
0.20,49.9	0
0.20,50.0	0
0.20,50.1	0
0.20,50.2	0
0.20,50.3	0
0.20,50.4	0
0.20,50.5	0
0.20,50.6	0
0.20,50.7	0
0.20,50.8	0
0.20,50.9	0

0.20,51.0	0
0.20,51.1	0
0.20,51.2	0
0.20,51.3	0
0.20,51.4	0
0.20,51.5	0
0.20,51.6	0
0.20,51.7	0
0.20,51.8	0
0.20,51.9	0
0.20,52.0	0
0.20,52.1	0
0.20,52.2	0
0.20,52.3	0
0.20,52.4	0
0.20,52.5	0
0.20,52.6	0
0.20,52.7	0
0.20,52.8	0
0.20,52.9	0
0.20,53.0	0
0.20,53.1	0
0.20,53.2	0
0.20,53.3	0
0.20,53.4	0
0.20,53.5	0
0.20,53.6	0
0.20,53.7	0
0.20,53.8	0
0.20,53.9	0
0.20,54.0	0
0.20,54.1	0
0.20,54.2	0
0.20,54.3	0
0.20,54.4	0
0.20,54.5	0
0.20,54.6	0
0.20,54.7	0
0.20,54.8	0
0.20,54.9	0
0.20,55.0	0
0.20,55.1	0

0.20,55.2	0
0.20,55.3	0
0.20,55.4	0
0.20,55.5	0
0.20,55.6	0
0.20,55.7	0
0.20,55.8	0
0.20,55.9	0
0.20,56.0	0
0.20,56.1	0
0.20,56.2	0
0.20,56.3	0
0.20,56.4	0
0.20,56.5	0
0.20,56.6	0
0.20,56.7	0
0.20,56.8	0
0.20,56.9	0
0.20,57.0	0
0.20,57.1	0
0.20,57.2	0
0.20,57.3	0
0.20,57.4	0
0.20,57.5	0
0.20,57.6	0
0.20,57.7	0
0.20,57.8	0
0.20,57.9	0
0.20,58.0	0
0.20,58.1	0
0.20,58.2	0
0.20,58.3	0
0.20,58.4	0
0.20,58.5	0
0.20,58.6	0
0.20,58.7	0
0.20,58.8	0
0.20,58.9	0
0.20,59.0	0
0.20,59.1	0
0.20,59.2	0
0.20,59.3	0

0.20,59.4	0
0.20,59.5	0
0.20,59.6	0
0.20,59.7	0
0.20,59.8	0
0.20,59.9	0
0.20,60.0	0
0.20,60.1	0
0.20,60.2	0
0.20,60.3	0
0.20,60.4	0
0.20,60.5	0
0.20,60.6	0
0.20,60.7	0
0.20,60.8	0
0.20,60.9	0
0.20,61.0	0
0.20,61.1	0
0.20,61.2	0
0.20,61.3	0
0.20,61.4	0
0.20,61.5	0
0.20,61.6	0
0.20,61.7	0
0.20,61.8	0
0.20,61.9	0
0.20,62.0	0
0.20,62.1	0
0.20,62.2	0
0.20,62.3	0
0.20,62.4	0
0.20,62.5	0
0.20,62.6	0
0.20,62.7	0
0.20,62.8	0
0.20,62.9	0
0.20,63.0	0
0.20,63.1	0
0.20,63.2	0
0.20,63.3	0
0.20,63.4	0
0.20,63.5	0

0.20,63.6	0
0.20,63.7	0
0.20,63.8	0
0.20,63.9	0
0.20,64.0	0
0.20,64.1	0
0.20,64.2	0
0.20,64.3	0
0.20,64.4	0
0.20,64.5	0
0.20,64.6	0
0.20,64.7	0
0.20,64.8	0
0.20,64.9	0
0.20,65.0	0
0.20,65.1	0
0.20,65.2	0
0.20,65.3	0
0.20,65.4	0
0.20,65.5	0
0.20,65.6	0
0.20,65.7	0
0.20,65.8	0
0.20,65.9	0
0.20,66.0	0
0.20,66.1	0
0.20,66.2	0
0.20,66.3	0
0.20,66.4	0
0.20,66.5	0
0.20,66.6	0
0.20,66.7	0
0.20,66.8	0
0.20,66.9	0
0.20,67.0	0
0.20,67.1	0
0.20,67.2	0
0.20,67.3	0
0.20,67.4	0
0.20,67.5	0
0.20,67.6	0
0.20,67.7	0

0.20,67.8	0
0.20,67.9	0
0.20,68.0	0
0.20,68.1	0
0.20,68.2	0
0.20,68.3	0
0.20,68.4	0
0.20,68.5	0
0.20,68.6	0
0.20,68.7	0
0.20,68.8	0
0.20,68.9	0
0.20,69.0	0
0.20,69.1	0
0.20,69.2	0
0.20,69.3	0
0.20,69.4	0
0.20,69.5	0
0.20,69.6	0
0.20,69.7	0
0.20,69.8	0
0.20,69.9	0
0.20,70.0	0
0.20,70.1	0
0.20,70.2	0
0.20,70.3	0
0.20,70.4	0
0.20,70.5	0
0.20,70.6	0
0.20,70.7	0
0.20,70.8	0
0.20,70.9	0
0.20,71.0	0
0.20,71.1	0
0.20,71.2	0
0.20,71.3	0
0.20,71.4	0
0.20,71.5	0
0.20,71.6	0
0.20,71.7	0
0.20,71.8	0
0.20,71.9	0

0.20,72.0	0
0.20,72.1	0
0.20,72.2	0
0.20,72.3	0
0.20,72.4	0
0.20,72.5	0
0.20,72.6	0
0.20,72.7	0
0.20,72.8	0
0.20,72.9	0
0.20,73.0	0
0.20,73.1	0
0.20,73.2	0
0.20,73.3	0
0.20,73.4	0
0.20,73.5	0
0.20,73.6	0
0.20,73.7	0
0.20,73.8	0
0.20,73.9	0
0.20,74.0	0
0.20,74.1	0
0.20,74.2	0
0.20,74.3	0
0.20,74.4	0
0.20,74.5	0
0.20,74.6	0
0.20,74.7	0
0.20,74.8	0
0.20,74.9	0
0.20,75.0	0
0.20,75.1	0
0.20,75.2	0
0.20,75.3	0
0.20,75.4	0
0.20,75.5	0
0.20,75.6	0
0.20,75.7	0
0.20,75.8	0
0.20,75.9	0
0.20,76.0	0
0.20,76.1	0

0.20,76.2	0
0.20,76.3	0
0.20,76.4	0
0.20,76.5	0
0.20,76.6	0
0.20,76.7	0
0.20,76.8	0
0.20,76.9	0
0.20,77.0	0
0.20,77.1	0
0.20,77.2	0
0.20,77.3	0
0.20,77.4	0
0.20,77.5	0
0.20,77.6	0
0.20,77.7	0
0.20,77.8	0
0.20,77.9	0
0.20,78.0	0
0.20,78.1	0
0.20,78.2	0
0.20,78.3	0
0.20,78.4	0
0.20,78.5	0
0.20,78.6	0
0.20,78.7	0
0.20,78.8	0
0.20,78.9	0
0.20,79.0	0
0.20,79.1	0
0.20,79.2	0
0.20,79.3	0
0.20,79.4	0
0.20,79.5	0
0.20,79.6	0
0.20,79.7	0
0.20,79.8	0
0.20,79.9	0
0.20,80.0	0
0.20,80.1	0
0.20,80.2	0
0.20,80.3	0

0.20,80.4	0
0.20,80.5	0
0.20,80.6	0
0.20,80.7	0
0.20,80.8	0
0.20,80.9	0
0.20,81.0	0
0.20,81.1	0
0.20,81.2	0
0.20,81.3	0
0.20,81.4	0
0.20,81.5	0
0.20,81.6	0
0.20,81.7	0
0.20,81.8	0
0.20,81.9	0
0.20,82.0	0
0.20,82.1	0
0.20,82.2	0
0.20,82.3	0
0.20,82.4	0
0.20,82.5	0
0.20,82.6	0
0.20,82.7	0
0.20,82.8	0
0.20,82.9	0
0.20,83.0	0
0.20,83.1	0
0.20,83.2	0
0.20,83.3	0
0.20,83.4	0
0.20,83.5	0
0.20,83.6	0
0.20,83.7	0
0.20,83.8	0
0.20,83.9	0
0.20,84.0	0
0.20,84.1	0
0.20,84.2	0
0.20,84.3	0
0.20,84.4	0
0.20,84.5	0

0.20,84.6	0
0.20,84.7	0
0.20,84.8	0
0.20,84.9	0
0.20,85.0	0
0.20,85.1	0
0.20,85.2	0
0.20,85.3	0
0.20,85.4	0
0.20,85.5	0
0.20,85.6	0
0.20,85.7	0
0.20,85.8	0
0.20,85.9	0
0.20,86.0	0
0.20,86.1	0
0.20,86.2	0
0.20,86.3	0
0.20,86.4	0
0.20,86.5	0
0.20,86.6	0
0.20,86.7	0
0.20,86.8	0
0.20,86.9	0
0.20,87.0	0
0.20,87.1	0
0.20,87.2	0
0.20,87.3	0
0.20,87.4	0
0.20,87.5	0
0.20,87.6	0
0.20,87.7	0
0.20,87.8	0
0.20,87.9	0
0.20,88.0	0
0.20,88.1	0
0.20,88.2	0
0.20,88.3	0
0.20,88.4	0
0.20,88.5	0
0.20,88.6	0
0.20,88.7	0

0.20,88.8	0
0.20,88.9	0
0.20,89.0	0
0.20,89.1	0
0.20,89.2	0
0.20,89.3	0
0.20,89.4	0
0.20,89.5	0
0.20,89.6	0
0.20,89.7	0
0.20,89.8	0
0.20,89.9	0
0.20,90.0	0
0.20,90.1	0
0.20,90.2	0
0.20,90.3	0
0.20,90.4	0
0.20,90.5	0
0.20,90.6	0
0.20,90.7	0
0.20,90.8	0
0.20,90.9	0
0.20,91.0	0
0.20,91.1	0
0.20,91.2	0
0.20,91.3	0
0.20,91.4	0
0.20,91.5	0
0.20,91.6	0
0.20,91.7	0
0.20,91.8	0
0.20,91.9	0
0.20,92.0	0
0.20,92.1	0
0.20,92.2	0
0.20,92.3	0
0.20,92.4	0
0.20,92.5	0
0.20,92.6	0
0.20,92.7	0
0.20,92.8	0
0.20,92.9	0

0.20,93.0	0
0.20,93.1	0
0.20,93.2	0
0.20,93.3	0
0.20,93.4	0
0.20,93.5	0
0.20,93.6	0
0.20,93.7	0
0.20,93.8	0
0.20,93.9	0
0.20,94.0	0
0.20,94.1	0
0.20,94.2	0
0.20,94.3	0
0.20,94.4	0
0.20,94.5	0
0.20,94.6	0
0.20,94.7	0
0.20,94.8	0
0.20,94.9	0
0.20,95.0	0
0.20,95.1	0
0.20,95.2	0
0.20,95.3	0
0.20,95.4	0
0.20,95.5	0
0.20,95.6	0
0.20,95.7	0
0.20,95.8	0
0.20,95.9	0
0.20,96.0	0
0.20,96.1	0
0.20,96.2	0
0.20,96.3	0
0.20,96.4	0
0.20,96.5	0
0.20,96.6	0
0.20,96.7	0
0.20,96.8	0
0.20,96.9	0
0.20,97.0	0
0.20,97.1	0

0.20,97.2	0
0.20,97.3	0
0.20,97.4	0
0.20,97.5	0
0.20,97.6	0
0.20,97.7	0
0.20,97.8	0
0.20,97.9	0
0.20,98.0	0
0.20,98.1	0
0.20,98.2	0
0.20,98.3	0
0.20,98.4	0
0.20,98.5	0
0.20,98.6	0
0.20,98.7	0
0.20,98.8	0
0.20,98.9	0
0.20,99.0	0
0.20,99.1	0
0.20,99.2	0
0.20,99.3	0
0.20,99.4	0
0.20,99.5	0
0.20,99.6	0
0.20,99.7	0
0.20,99.8	0
0.20,99.9	0
0.20,100.0	0
0.21,0.0	0
0.21,0.1	0
0.21,0.2	0
0.21,0.3	0
0.21,0.4	0
0.21,0.5	0
0.21,0.6	0
0.21,0.7	0
0.21,0.8	0
0.21,0.9	0
0.21,1.0	0
0.21,1.1	0
0.21,1.2	0

0.21,1.3	0
0.21,1.4	0
0.21,1.5	0
0.21,1.6	0
0.21,1.7	0
0.21,1.8	0
0.21,1.9	0
0.21,2.0	0
0.21,2.1	0
0.21,2.2	0
0.21,2.3	0
0.21,2.4	0
0.21,2.5	0
0.21,2.6	0
0.21,2.7	0
0.21,2.8	0
0.21,2.9	0
0.21,3.0	0
0.21,3.1	0
0.21,3.2	0
0.21,3.3	0
0.21,3.4	0
0.21,3.5	0
0.21,3.6	0
0.21,3.7	0
0.21,3.8	0
0.21,3.9	0
0.21,4.0	0
0.21,4.1	0
0.21,4.2	0
0.21,4.3	0
0.21,4.4	0
0.21,4.5	0
0.21,4.6	0
0.21,4.7	0
0.21,4.8	0
0.21,4.9	0
0.21,5.0	0
0.21,5.1	0
0.21,5.2	0
0.21,5.3	0
0.21,5.4	0

0.21,5.5	0
0.21,5.6	0
0.21,5.7	0
0.21,5.8	0
0.21,5.9	0
0.21,6.0	0
0.21,6.1	0
0.21,6.2	0
0.21,6.3	0
0.21,6.4	0
0.21,6.5	0
0.21,6.6	0
0.21,6.7	0
0.21,6.8	0
0.21,6.9	0
0.21,7.0	0
0.21,7.1	0
0.21,7.2	0
0.21,7.3	0
0.21,7.4	0
0.21,7.5	0
0.21,7.6	0
0.21,7.7	0
0.21,7.8	0
0.21,7.9	0
0.21,8.0	0
0.21,8.1	0
0.21,8.2	0
0.21,8.3	0
0.21,8.4	0
0.21,8.5	0
0.21,8.6	0
0.21,8.7	0
0.21,8.8	0
0.21,8.9	0
0.21,9.0	0
0.21,9.1	0
0.21,9.2	0
0.21,9.3	0
0.21,9.4	0
0.21,9.5	0
0.21,9.6	0

0.21,9.7	0
0.21,9.8	0
0.21,9.9	0
0.21,10.0	0
0.21,10.1	0
0.21,10.2	0
0.21,10.3	0
0.21,10.4	0
0.21,10.5	0
0.21,10.6	0
0.21,10.7	0
0.21,10.8	0
0.21,10.9	0
0.21,11.0	0
0.21,11.1	0
0.21,11.2	0
0.21,11.3	0
0.21,11.4	0
0.21,11.5	0
0.21,11.6	0
0.21,11.7	0
0.21,11.8	0
0.21,11.9	0
0.21,12.0	0
0.21,12.1	0
0.21,12.2	0
0.21,12.3	0
0.21,12.4	0
0.21,12.5	0
0.21,12.6	0
0.21,12.7	0
0.21,12.8	0
0.21,12.9	0
0.21,13.0	0
0.21,13.1	0
0.21,13.2	0
0.21,13.3	0
0.21,13.4	0
0.21,13.5	0
0.21,13.6	0
0.21,13.7	0
0.21,13.8	0

0.21,13.9	0
0.21,14.0	0
0.21,14.1	0
0.21,14.2	0
0.21,14.3	0
0.21,14.4	0
0.21,14.5	0
0.21,14.6	0
0.21,14.7	0
0.21,14.8	0
0.21,14.9	0
0.21,15.0	0
0.21,15.1	0
0.21,15.2	0
0.21,15.3	0
0.21,15.4	0
0.21,15.5	0
0.21,15.6	0
0.21,15.7	0
0.21,15.8	0
0.21,15.9	0
0.21,16.0	0
0.21,16.1	0
0.21,16.2	0
0.21,16.3	0
0.21,16.4	0
0.21,16.5	0
0.21,16.6	0
0.21,16.7	0
0.21,16.8	0
0.21,16.9	0
0.21,17.0	0
0.21,17.1	0
0.21,17.2	0
0.21,17.3	0
0.21,17.4	0
0.21,17.5	0
0.21,17.6	0
0.21,17.7	0
0.21,17.8	0
0.21,17.9	0
0.21,18.0	0

0.21,18.1	0
0.21,18.2	0
0.21,18.3	0
0.21,18.4	0
0.21,18.5	0
0.21,18.6	0
0.21,18.7	0
0.21,18.8	0
0.21,18.9	0
0.21,19.0	0
0.21,19.1	0
0.21,19.2	0
0.21,19.3	0
0.21,19.4	0
0.21,19.5	0
0.21,19.6	0
0.21,19.7	0
0.21,19.8	0
0.21,19.9	0
0.21,20.0	0
0.21,20.1	0
0.21,20.2	0
0.21,20.3	0
0.21,20.4	0
0.21,20.5	0
0.21,20.6	0
0.21,20.7	0
0.21,20.8	0
0.21,20.9	0
0.21,21.0	0
0.21,21.1	0
0.21,21.2	0
0.21,21.3	0
0.21,21.4	0
0.21,21.5	0
0.21,21.6	0
0.21,21.7	0
0.21,21.8	0
0.21,21.9	0
0.21,22.0	0
0.21,22.1	0
0.21,22.2	0

0.21,22.3	0
0.21,22.4	0
0.21,22.5	0
0.21,22.6	0
0.21,22.7	0
0.21,22.8	0
0.21,22.9	0
0.21,23.0	0
0.21,23.1	0
0.21,23.2	0
0.21,23.3	0
0.21,23.4	0
0.21,23.5	0
0.21,23.6	0
0.21,23.7	0
0.21,23.8	0
0.21,23.9	0
0.21,24.0	0
0.21,24.1	0
0.21,24.2	0
0.21,24.3	0
0.21,24.4	0
0.21,24.5	0
0.21,24.6	0
0.21,24.7	0
0.21,24.8	0
0.21,24.9	0
0.21,25.0	0
0.21,25.1	0
0.21,25.2	0
0.21,25.3	0
0.21,25.4	0
0.21,25.5	0
0.21,25.6	0
0.21,25.7	0
0.21,25.8	0
0.21,25.9	0
0.21,26.0	0
0.21,26.1	0
0.21,26.2	0
0.21,26.3	0
0.21,26.4	0

0.21,26.5	0
0.21,26.6	0
0.21,26.7	0
0.21,26.8	0
0.21,26.9	0
0.21,27.0	0
0.21,27.1	0
0.21,27.2	0
0.21,27.3	0
0.21,27.4	0
0.21,27.5	0
0.21,27.6	0
0.21,27.7	0
0.21,27.8	0
0.21,27.9	0
0.21,28.0	0
0.21,28.1	0
0.21,28.2	0
0.21,28.3	0
0.21,28.4	0
0.21,28.5	0
0.21,28.6	0
0.21,28.7	0
0.21,28.8	0
0.21,28.9	0
0.21,29.0	0
0.21,29.1	0
0.21,29.2	0
0.21,29.3	0
0.21,29.4	0
0.21,29.5	0
0.21,29.6	0
0.21,29.7	0
0.21,29.8	0
0.21,29.9	0
0.21,30.0	0
0.21,30.1	0
0.21,30.2	0
0.21,30.3	0
0.21,30.4	0
0.21,30.5	0
0.21,30.6	0

0.21,30.7	0
0.21,30.8	0
0.21,30.9	0
0.21,31.0	0
0.21,31.1	0
0.21,31.2	0
0.21,31.3	0
0.21,31.4	0
0.21,31.5	0
0.21,31.6	0
0.21,31.7	0
0.21,31.8	0
0.21,31.9	0
0.21,32.0	0
0.21,32.1	0
0.21,32.2	0
0.21,32.3	0
0.21,32.4	0
0.21,32.5	0
0.21,32.6	0
0.21,32.7	0
0.21,32.8	0
0.21,32.9	0
0.21,33.0	0
0.21,33.1	0
0.21,33.2	0
0.21,33.3	0
0.21,33.4	0
0.21,33.5	0
0.21,33.6	0
0.21,33.7	0
0.21,33.8	0
0.21,33.9	0
0.21,34.0	0
0.21,34.1	0
0.21,34.2	0
0.21,34.3	0
0.21,34.4	0
0.21,34.5	0
0.21,34.6	0
0.21,34.7	0
0.21,34.8	0

0.21,34.9	0
0.21,35.0	0
0.21,35.1	0
0.21,35.2	0
0.21,35.3	0
0.21,35.4	0
0.21,35.5	0
0.21,35.6	0
0.21,35.7	0
0.21,35.8	0
0.21,35.9	0
0.21,36.0	0
0.21,36.1	0
0.21,36.2	0
0.21,36.3	0
0.21,36.4	0
0.21,36.5	0
0.21,36.6	0
0.21,36.7	0
0.21,36.8	0
0.21,36.9	0
0.21,37.0	0
0.21,37.1	0
0.21,37.2	0
0.21,37.3	0
0.21,37.4	0
0.21,37.5	0
0.21,37.6	0
0.21,37.7	0
0.21,37.8	0
0.21,37.9	0
0.21,38.0	0
0.21,38.1	0
0.21,38.2	0
0.21,38.3	0
0.21,38.4	0
0.21,38.5	0
0.21,38.6	0
0.21,38.7	0
0.21,38.8	0
0.21,38.9	0
0.21,39.0	0

0.21,39.1	0
0.21,39.2	0
0.21,39.3	0
0.21,39.4	0
0.21,39.5	0
0.21,39.6	0
0.21,39.7	0
0.21,39.8	0
0.21,39.9	0
0.21,40.0	0
0.21,40.1	0
0.21,40.2	0
0.21,40.3	0
0.21,40.4	0
0.21,40.5	0
0.21,40.6	0
0.21,40.7	0
0.21,40.8	0
0.21,40.9	0
0.21,41.0	0
0.21,41.1	0
0.21,41.2	0
0.21,41.3	0
0.21,41.4	0
0.21,41.5	0
0.21,41.6	0
0.21,41.7	0
0.21,41.8	0
0.21,41.9	0
0.21,42.0	0
0.21,42.1	0
0.21,42.2	0
0.21,42.3	0
0.21,42.4	0
0.21,42.5	0
0.21,42.6	0
0.21,42.7	0
0.21,42.8	0
0.21,42.9	0
0.21,43.0	0
0.21,43.1	0
0.21,43.2	0

0.21,43.3	0
0.21,43.4	0
0.21,43.5	0
0.21,43.6	0
0.21,43.7	0
0.21,43.8	0
0.21,43.9	0
0.21,44.0	0
0.21,44.1	0
0.21,44.2	0
0.21,44.3	0
0.21,44.4	0
0.21,44.5	0
0.21,44.6	0
0.21,44.7	0
0.21,44.8	0
0.21,44.9	0
0.21,45.0	0
0.21,45.1	0
0.21,45.2	0
0.21,45.3	0
0.21,45.4	0
0.21,45.5	0
0.21,45.6	0
0.21,45.7	0
0.21,45.8	0
0.21,45.9	0
0.21,46.0	0
0.21,46.1	0
0.21,46.2	0
0.21,46.3	0
0.21,46.4	0
0.21,46.5	0
0.21,46.6	0
0.21,46.7	0
0.21,46.8	0
0.21,46.9	0
0.21,47.0	0
0.21,47.1	0
0.21,47.2	0
0.21,47.3	0
0.21,47.4	0

0.21,47.5	0
0.21,47.6	0
0.21,47.7	0
0.21,47.8	0
0.21,47.9	0
0.21,48.0	0
0.21,48.1	0
0.21,48.2	0
0.21,48.3	0
0.21,48.4	0
0.21,48.5	0
0.21,48.6	0
0.21,48.7	0
0.21,48.8	0
0.21,48.9	0
0.21,49.0	0
0.21,49.1	0
0.21,49.2	0
0.21,49.3	0
0.21,49.4	0
0.21,49.5	0
0.21,49.6	0
0.21,49.7	0
0.21,49.8	0
0.21,49.9	0
0.21,50.0	0
0.21,50.1	0
0.21,50.2	0
0.21,50.3	0
0.21,50.4	0
0.21,50.5	0
0.21,50.6	0
0.21,50.7	0
0.21,50.8	0
0.21,50.9	0
0.21,51.0	0
0.21,51.1	0
0.21,51.2	0
0.21,51.3	0
0.21,51.4	0
0.21,51.5	0
0.21,51.6	0

0.21,51.7	0
0.21,51.8	0
0.21,51.9	0
0.21,52.0	0
0.21,52.1	0
0.21,52.2	0
0.21,52.3	0
0.21,52.4	0
0.21,52.5	0
0.21,52.6	0
0.21,52.7	0
0.21,52.8	0
0.21,52.9	0
0.21,53.0	0
0.21,53.1	0
0.21,53.2	0
0.21,53.3	0
0.21,53.4	0
0.21,53.5	0
0.21,53.6	0
0.21,53.7	0
0.21,53.8	0
0.21,53.9	0
0.21,54.0	0
0.21,54.1	0
0.21,54.2	0
0.21,54.3	0
0.21,54.4	0
0.21,54.5	0
0.21,54.6	0
0.21,54.7	0
0.21,54.8	0
0.21,54.9	0
0.21,55.0	0
0.21,55.1	0
0.21,55.2	0
0.21,55.3	0
0.21,55.4	0
0.21,55.5	0
0.21,55.6	0
0.21,55.7	0
0.21,55.8	0

0.21,55.9	0
0.21,56.0	0
0.21,56.1	0
0.21,56.2	0
0.21,56.3	0
0.21,56.4	0
0.21,56.5	0
0.21,56.6	0
0.21,56.7	0
0.21,56.8	0
0.21,56.9	0
0.21,57.0	0
0.21,57.1	0
0.21,57.2	0
0.21,57.3	0
0.21,57.4	0
0.21,57.5	0
0.21,57.6	0
0.21,57.7	0
0.21,57.8	0
0.21,57.9	0
0.21,58.0	0
0.21,58.1	0
0.21,58.2	0
0.21,58.3	0
0.21,58.4	0
0.21,58.5	0
0.21,58.6	0
0.21,58.7	0
0.21,58.8	0
0.21,58.9	0
0.21,59.0	0
0.21,59.1	0
0.21,59.2	0
0.21,59.3	0
0.21,59.4	0
0.21,59.5	0
0.21,59.6	0
0.21,59.7	0
0.21,59.8	0
0.21,59.9	0
0.21,60.0	0

0.21,60.1	0
0.21,60.2	0
0.21,60.3	0
0.21,60.4	0
0.21,60.5	0
0.21,60.6	0
0.21,60.7	0
0.21,60.8	0
0.21,60.9	0
0.21,61.0	0
0.21,61.1	0
0.21,61.2	0
0.21,61.3	0
0.21,61.4	0
0.21,61.5	0
0.21,61.6	0
0.21,61.7	0
0.21,61.8	0
0.21,61.9	0
0.21,62.0	0
0.21,62.1	0
0.21,62.2	0
0.21,62.3	0
0.21,62.4	0
0.21,62.5	0
0.21,62.6	0
0.21,62.7	0
0.21,62.8	0
0.21,62.9	0
0.21,63.0	0
0.21,63.1	0
0.21,63.2	0
0.21,63.3	0
0.21,63.4	0
0.21,63.5	0
0.21,63.6	0
0.21,63.7	0
0.21,63.8	0
0.21,63.9	0
0.21,64.0	0
0.21,64.1	0
0.21,64.2	0

0.21,64.3	0
0.21,64.4	0
0.21,64.5	0
0.21,64.6	0
0.21,64.7	0
0.21,64.8	0
0.21,64.9	0
0.21,65.0	0
0.21,65.1	0
0.21,65.2	0
0.21,65.3	0
0.21,65.4	0
0.21,65.5	0
0.21,65.6	0
0.21,65.7	0
0.21,65.8	0
0.21,65.9	0
0.21,66.0	0
0.21,66.1	0
0.21,66.2	0
0.21,66.3	0
0.21,66.4	0
0.21,66.5	0
0.21,66.6	0
0.21,66.7	0
0.21,66.8	0
0.21,66.9	0
0.21,67.0	0
0.21,67.1	0
0.21,67.2	0
0.21,67.3	0
0.21,67.4	0
0.21,67.5	0
0.21,67.6	0
0.21,67.7	0
0.21,67.8	0
0.21,67.9	0
0.21,68.0	0
0.21,68.1	0
0.21,68.2	0
0.21,68.3	0
0.21,68.4	0

0.21,68.5	0
0.21,68.6	0
0.21,68.7	0
0.21,68.8	0
0.21,68.9	0
0.21,69.0	0
0.21,69.1	0
0.21,69.2	0
0.21,69.3	0
0.21,69.4	0
0.21,69.5	0
0.21,69.6	0
0.21,69.7	0
0.21,69.8	0
0.21,69.9	0
0.21,70.0	0
0.21,70.1	0
0.21,70.2	0
0.21,70.3	0
0.21,70.4	0
0.21,70.5	0
0.21,70.6	0
0.21,70.7	0
0.21,70.8	0
0.21,70.9	0
0.21,71.0	0
0.21,71.1	0
0.21,71.2	0
0.21,71.3	0
0.21,71.4	0
0.21,71.5	0
0.21,71.6	0
0.21,71.7	0
0.21,71.8	0
0.21,71.9	0
0.21,72.0	0
0.21,72.1	0
0.21,72.2	0
0.21,72.3	0
0.21,72.4	0
0.21,72.5	0
0.21,72.6	0

0.21,72.7	0
0.21,72.8	0
0.21,72.9	0
0.21,73.0	0
0.21,73.1	0
0.21,73.2	0
0.21,73.3	0
0.21,73.4	0
0.21,73.5	0
0.21,73.6	0
0.21,73.7	0
0.21,73.8	0
0.21,73.9	0
0.21,74.0	0
0.21,74.1	0
0.21,74.2	0
0.21,74.3	0
0.21,74.4	0
0.21,74.5	0
0.21,74.6	0
0.21,74.7	0
0.21,74.8	0
0.21,74.9	0
0.21,75.0	0
0.21,75.1	0
0.21,75.2	0
0.21,75.3	0
0.21,75.4	0
0.21,75.5	0
0.21,75.6	0
0.21,75.7	0
0.21,75.8	0
0.21,75.9	0
0.21,76.0	0
0.21,76.1	0
0.21,76.2	0
0.21,76.3	0
0.21,76.4	0
0.21,76.5	0
0.21,76.6	0
0.21,76.7	0
0.21,76.8	0

0.21,76.9	0
0.21,77.0	0
0.21,77.1	0
0.21,77.2	0
0.21,77.3	0
0.21,77.4	0
0.21,77.5	0
0.21,77.6	0
0.21,77.7	0
0.21,77.8	0
0.21,77.9	0
0.21,78.0	0
0.21,78.1	0
0.21,78.2	0
0.21,78.3	0
0.21,78.4	0
0.21,78.5	0
0.21,78.6	0
0.21,78.7	0
0.21,78.8	0
0.21,78.9	0
0.21,79.0	0
0.21,79.1	0
0.21,79.2	0
0.21,79.3	0
0.21,79.4	0
0.21,79.5	0
0.21,79.6	0
0.21,79.7	0
0.21,79.8	0
0.21,79.9	0
0.21,80.0	0
0.21,80.1	0
0.21,80.2	0
0.21,80.3	0
0.21,80.4	0
0.21,80.5	0
0.21,80.6	0
0.21,80.7	0
0.21,80.8	0
0.21,80.9	0
0.21,81.0	0

0.21,81.1	0
0.21,81.2	0
0.21,81.3	0
0.21,81.4	0
0.21,81.5	0
0.21,81.6	0
0.21,81.7	0
0.21,81.8	0
0.21,81.9	0
0.21,82.0	0
0.21,82.1	0
0.21,82.2	0
0.21,82.3	0
0.21,82.4	0
0.21,82.5	0
0.21,82.6	0
0.21,82.7	0
0.21,82.8	0
0.21,82.9	0
0.21,83.0	0
0.21,83.1	0
0.21,83.2	0
0.21,83.3	0
0.21,83.4	0
0.21,83.5	0
0.21,83.6	0
0.21,83.7	0
0.21,83.8	0
0.21,83.9	0
0.21,84.0	0
0.21,84.1	0
0.21,84.2	0
0.21,84.3	0
0.21,84.4	0
0.21,84.5	0
0.21,84.6	0
0.21,84.7	0
0.21,84.8	0
0.21,84.9	0
0.21,85.0	0
0.21,85.1	0
0.21,85.2	0

0.21,85.3	0
0.21,85.4	0
0.21,85.5	0
0.21,85.6	0
0.21,85.7	0
0.21,85.8	0
0.21,85.9	0
0.21,86.0	0
0.21,86.1	0
0.21,86.2	0
0.21,86.3	0
0.21,86.4	0
0.21,86.5	0
0.21,86.6	0
0.21,86.7	0
0.21,86.8	0
0.21,86.9	0
0.21,87.0	0
0.21,87.1	0
0.21,87.2	0
0.21,87.3	0
0.21,87.4	0
0.21,87.5	0
0.21,87.6	0
0.21,87.7	0
0.21,87.8	0
0.21,87.9	0
0.21,88.0	0
0.21,88.1	0
0.21,88.2	0
0.21,88.3	0
0.21,88.4	0
0.21,88.5	0
0.21,88.6	0
0.21,88.7	0
0.21,88.8	0
0.21,88.9	0
0.21,89.0	0
0.21,89.1	0
0.21,89.2	0
0.21,89.3	0
0.21,89.4	0

0.21,89.5	0
0.21,89.6	0
0.21,89.7	0
0.21,89.8	0
0.21,89.9	0
0.21,90.0	0
0.21,90.1	0
0.21,90.2	0
0.21,90.3	0
0.21,90.4	0
0.21,90.5	0
0.21,90.6	0
0.21,90.7	0
0.21,90.8	0
0.21,90.9	0
0.21,91.0	0
0.21,91.1	0
0.21,91.2	0
0.21,91.3	0
0.21,91.4	0
0.21,91.5	0
0.21,91.6	0
0.21,91.7	0
0.21,91.8	0
0.21,91.9	0
0.21,92.0	0
0.21,92.1	0
0.21,92.2	0
0.21,92.3	0
0.21,92.4	0
0.21,92.5	0
0.21,92.6	0
0.21,92.7	0
0.21,92.8	0
0.21,92.9	0
0.21,93.0	0
0.21,93.1	0
0.21,93.2	0
0.21,93.3	0
0.21,93.4	0
0.21,93.5	0
0.21,93.6	0

0.21,93.7	0
0.21,93.8	0
0.21,93.9	0
0.21,94.0	0
0.21,94.1	0
0.21,94.2	0
0.21,94.3	0
0.21,94.4	0
0.21,94.5	0
0.21,94.6	0
0.21,94.7	0
0.21,94.8	0
0.21,94.9	0
0.21,95.0	0
0.21,95.1	0
0.21,95.2	0
0.21,95.3	0
0.21,95.4	0
0.21,95.5	0
0.21,95.6	0
0.21,95.7	0
0.21,95.8	0
0.21,95.9	0
0.21,96.0	0
0.21,96.1	0
0.21,96.2	0
0.21,96.3	0
0.21,96.4	0
0.21,96.5	0
0.21,96.6	0
0.21,96.7	0
0.21,96.8	0
0.21,96.9	0
0.21,97.0	0
0.21,97.1	0
0.21,97.2	0
0.21,97.3	0
0.21,97.4	0
0.21,97.5	0
0.21,97.6	0
0.21,97.7	0
0.21,97.8	0

0.21,97.9	0
0.21,98.0	0
0.21,98.1	0
0.21,98.2	0
0.21,98.3	0
0.21,98.4	0
0.21,98.5	0
0.21,98.6	0
0.21,98.7	0
0.21,98.8	0
0.21,98.9	0
0.21,99.0	0
0.21,99.1	0
0.21,99.2	0
0.21,99.3	0
0.21,99.4	0
0.21,99.5	0
0.21,99.6	0
0.21,99.7	0
0.21,99.8	0
0.21,99.9	0
0.21,100.0	0
0.22,0.0	0
0.22,0.1	0
0.22,0.2	0
0.22,0.3	0
0.22,0.4	0
0.22,0.5	0
0.22,0.6	0
0.22,0.7	0
0.22,0.8	0
0.22,0.9	0
0.22,1.0	0
0.22,1.1	0
0.22,1.2	0
0.22,1.3	0
0.22,1.4	0
0.22,1.5	0
0.22,1.6	0
0.22,1.7	0
0.22,1.8	0
0.22,1.9	0

0.22,2.0	0
0.22,2.1	0
0.22,2.2	0
0.22,2.3	0
0.22,2.4	0
0.22,2.5	0
0.22,2.6	0
0.22,2.7	0
0.22,2.8	0
0.22,2.9	0
0.22,3.0	0
0.22,3.1	0
0.22,3.2	0
0.22,3.3	0
0.22,3.4	0
0.22,3.5	0
0.22,3.6	0
0.22,3.7	0
0.22,3.8	0
0.22,3.9	0
0.22,4.0	0
0.22,4.1	0
0.22,4.2	0
0.22,4.3	0
0.22,4.4	0
0.22,4.5	0
0.22,4.6	0
0.22,4.7	0
0.22,4.8	0
0.22,4.9	0
0.22,5.0	0
0.22,5.1	0
0.22,5.2	0
0.22,5.3	0
0.22,5.4	0
0.22,5.5	0
0.22,5.6	0
0.22,5.7	0
0.22,5.8	0
0.22,5.9	0
0.22,6.0	0
0.22,6.1	0

0.22,6.2	0
0.22,6.3	0
0.22,6.4	0
0.22,6.5	0
0.22,6.6	0
0.22,6.7	0
0.22,6.8	0
0.22,6.9	0
0.22,7.0	0
0.22,7.1	0
0.22,7.2	0
0.22,7.3	0
0.22,7.4	0
0.22,7.5	0
0.22,7.6	0
0.22,7.7	0
0.22,7.8	0
0.22,7.9	0
0.22,8.0	0
0.22,8.1	0
0.22,8.2	0
0.22,8.3	0
0.22,8.4	0
0.22,8.5	0
0.22,8.6	0
0.22,8.7	0
0.22,8.8	0
0.22,8.9	0
0.22,9.0	0
0.22,9.1	0
0.22,9.2	0
0.22,9.3	0
0.22,9.4	0
0.22,9.5	0
0.22,9.6	0
0.22,9.7	0
0.22,9.8	0
0.22,9.9	0
0.22,10.0	0
0.22,10.1	0
0.22,10.2	0
0.22,10.3	0

0.22,10.4	0
0.22,10.5	0
0.22,10.6	0
0.22,10.7	0
0.22,10.8	0
0.22,10.9	0
0.22,11.0	0
0.22,11.1	0
0.22,11.2	0
0.22,11.3	0
0.22,11.4	0
0.22,11.5	0
0.22,11.6	0
0.22,11.7	0
0.22,11.8	0
0.22,11.9	0
0.22,12.0	0
0.22,12.1	0
0.22,12.2	0
0.22,12.3	0
0.22,12.4	0
0.22,12.5	0
0.22,12.6	0
0.22,12.7	0
0.22,12.8	0
0.22,12.9	0
0.22,13.0	0
0.22,13.1	0
0.22,13.2	0
0.22,13.3	0
0.22,13.4	0
0.22,13.5	0
0.22,13.6	0
0.22,13.7	0
0.22,13.8	0
0.22,13.9	0
0.22,14.0	0
0.22,14.1	0
0.22,14.2	0
0.22,14.3	0
0.22,14.4	0
0.22,14.5	0

0.22,14.6	0
0.22,14.7	0
0.22,14.8	0
0.22,14.9	0
0.22,15.0	0
0.22,15.1	0
0.22,15.2	0
0.22,15.3	0
0.22,15.4	0
0.22,15.5	0
0.22,15.6	0
0.22,15.7	0
0.22,15.8	0
0.22,15.9	0
0.22,16.0	0
0.22,16.1	0
0.22,16.2	0
0.22,16.3	0
0.22,16.4	0
0.22,16.5	0
0.22,16.6	0
0.22,16.7	0
0.22,16.8	0
0.22,16.9	0
0.22,17.0	0
0.22,17.1	0
0.22,17.2	0
0.22,17.3	0
0.22,17.4	0
0.22,17.5	0
0.22,17.6	0
0.22,17.7	0
0.22,17.8	0
0.22,17.9	0
0.22,18.0	0
0.22,18.1	0
0.22,18.2	0
0.22,18.3	0
0.22,18.4	0
0.22,18.5	0
0.22,18.6	0
0.22,18.7	0

0.22,18.8	0
0.22,18.9	0
0.22,19.0	0
0.22,19.1	0
0.22,19.2	0
0.22,19.3	0
0.22,19.4	0
0.22,19.5	0
0.22,19.6	0
0.22,19.7	0
0.22,19.8	0
0.22,19.9	0
0.22,20.0	0
0.22,20.1	0
0.22,20.2	0
0.22,20.3	0
0.22,20.4	0
0.22,20.5	0
0.22,20.6	0
0.22,20.7	0
0.22,20.8	0
0.22,20.9	0
0.22,21.0	0
0.22,21.1	0
0.22,21.2	0
0.22,21.3	0
0.22,21.4	0
0.22,21.5	0
0.22,21.6	0
0.22,21.7	0
0.22,21.8	0
0.22,21.9	0
0.22,22.0	0
0.22,22.1	0
0.22,22.2	0
0.22,22.3	0
0.22,22.4	0
0.22,22.5	0
0.22,22.6	0
0.22,22.7	0
0.22,22.8	0
0.22,22.9	0

0.22,23.0	0
0.22,23.1	0
0.22,23.2	0
0.22,23.3	0
0.22,23.4	0
0.22,23.5	0
0.22,23.6	0
0.22,23.7	0
0.22,23.8	0
0.22,23.9	0
0.22,24.0	0
0.22,24.1	0
0.22,24.2	0
0.22,24.3	0
0.22,24.4	0
0.22,24.5	0
0.22,24.6	0
0.22,24.7	0
0.22,24.8	0
0.22,24.9	0
0.22,25.0	0
0.22,25.1	0
0.22,25.2	0
0.22,25.3	0
0.22,25.4	0
0.22,25.5	0
0.22,25.6	0
0.22,25.7	0
0.22,25.8	0
0.22,25.9	0
0.22,26.0	0
0.22,26.1	0
0.22,26.2	0
0.22,26.3	0
0.22,26.4	0
0.22,26.5	0
0.22,26.6	0
0.22,26.7	0
0.22,26.8	0
0.22,26.9	0
0.22,27.0	0
0.22,27.1	0

0.22,27.2	0
0.22,27.3	0
0.22,27.4	0
0.22,27.5	0
0.22,27.6	0
0.22,27.7	0
0.22,27.8	0
0.22,27.9	0
0.22,28.0	0
0.22,28.1	0
0.22,28.2	0
0.22,28.3	0
0.22,28.4	0
0.22,28.5	0
0.22,28.6	0
0.22,28.7	0
0.22,28.8	0
0.22,28.9	0
0.22,29.0	0
0.22,29.1	0
0.22,29.2	0
0.22,29.3	0
0.22,29.4	0
0.22,29.5	0
0.22,29.6	0
0.22,29.7	0
0.22,29.8	0
0.22,29.9	0
0.22,30.0	0
0.22,30.1	0
0.22,30.2	0
0.22,30.3	0
0.22,30.4	0
0.22,30.5	0
0.22,30.6	0
0.22,30.7	0
0.22,30.8	0
0.22,30.9	0
0.22,31.0	0
0.22,31.1	0
0.22,31.2	0
0.22,31.3	0

0.22,31.4	0
0.22,31.5	0
0.22,31.6	0
0.22,31.7	0
0.22,31.8	0
0.22,31.9	0
0.22,32.0	0
0.22,32.1	0
0.22,32.2	0
0.22,32.3	0
0.22,32.4	0
0.22,32.5	0
0.22,32.6	0
0.22,32.7	0
0.22,32.8	0
0.22,32.9	0
0.22,33.0	0
0.22,33.1	0
0.22,33.2	0
0.22,33.3	0
0.22,33.4	0
0.22,33.5	0
0.22,33.6	0
0.22,33.7	0
0.22,33.8	0
0.22,33.9	0
0.22,34.0	0
0.22,34.1	0
0.22,34.2	0
0.22,34.3	0
0.22,34.4	0
0.22,34.5	0
0.22,34.6	0
0.22,34.7	0
0.22,34.8	0
0.22,34.9	0
0.22,35.0	0
0.22,35.1	0
0.22,35.2	0
0.22,35.3	0
0.22,35.4	0
0.22,35.5	0

0.22,35.6	0
0.22,35.7	0
0.22,35.8	0
0.22,35.9	0
0.22,36.0	0
0.22,36.1	0
0.22,36.2	0
0.22,36.3	0
0.22,36.4	0
0.22,36.5	0
0.22,36.6	0
0.22,36.7	0
0.22,36.8	0
0.22,36.9	0
0.22,37.0	0
0.22,37.1	0
0.22,37.2	0
0.22,37.3	0
0.22,37.4	0
0.22,37.5	0
0.22,37.6	0
0.22,37.7	0
0.22,37.8	0
0.22,37.9	0
0.22,38.0	0
0.22,38.1	0
0.22,38.2	0
0.22,38.3	0
0.22,38.4	0
0.22,38.5	0
0.22,38.6	0
0.22,38.7	0
0.22,38.8	0
0.22,38.9	0
0.22,39.0	0
0.22,39.1	0
0.22,39.2	0
0.22,39.3	0
0.22,39.4	0
0.22,39.5	0
0.22,39.6	0
0.22,39.7	0

0.22,39.8	0
0.22,39.9	0
0.22,40.0	0
0.22,40.1	0
0.22,40.2	0
0.22,40.3	0
0.22,40.4	0
0.22,40.5	0
0.22,40.6	0
0.22,40.7	0
0.22,40.8	0
0.22,40.9	0
0.22,41.0	0
0.22,41.1	0
0.22,41.2	0
0.22,41.3	0
0.22,41.4	0
0.22,41.5	0
0.22,41.6	0
0.22,41.7	0
0.22,41.8	0
0.22,41.9	0
0.22,42.0	0
0.22,42.1	0
0.22,42.2	0
0.22,42.3	0
0.22,42.4	0
0.22,42.5	0
0.22,42.6	0
0.22,42.7	0
0.22,42.8	0
0.22,42.9	0
0.22,43.0	0
0.22,43.1	0
0.22,43.2	0
0.22,43.3	0
0.22,43.4	0
0.22,43.5	0
0.22,43.6	0
0.22,43.7	0
0.22,43.8	0
0.22,43.9	0

0.22,44.0	0
0.22,44.1	0
0.22,44.2	0
0.22,44.3	0
0.22,44.4	0
0.22,44.5	0
0.22,44.6	0
0.22,44.7	0
0.22,44.8	0
0.22,44.9	0
0.22,45.0	0
0.22,45.1	0
0.22,45.2	0
0.22,45.3	0
0.22,45.4	0
0.22,45.5	0
0.22,45.6	0
0.22,45.7	0
0.22,45.8	0
0.22,45.9	0
0.22,46.0	0
0.22,46.1	0
0.22,46.2	0
0.22,46.3	0
0.22,46.4	0
0.22,46.5	0
0.22,46.6	0
0.22,46.7	0
0.22,46.8	0
0.22,46.9	0
0.22,47.0	0
0.22,47.1	0
0.22,47.2	0
0.22,47.3	0
0.22,47.4	0
0.22,47.5	0
0.22,47.6	0
0.22,47.7	0
0.22,47.8	0
0.22,47.9	0
0.22,48.0	0
0.22,48.1	0

0.22,48.2	0
0.22,48.3	0
0.22,48.4	0
0.22,48.5	0
0.22,48.6	0
0.22,48.7	0
0.22,48.8	0
0.22,48.9	0
0.22,49.0	0
0.22,49.1	0
0.22,49.2	0
0.22,49.3	0
0.22,49.4	0
0.22,49.5	0
0.22,49.6	0
0.22,49.7	0
0.22,49.8	0
0.22,49.9	0
0.22,50.0	0
0.22,50.1	0
0.22,50.2	0
0.22,50.3	0
0.22,50.4	0
0.22,50.5	0
0.22,50.6	0
0.22,50.7	0
0.22,50.8	0
0.22,50.9	0
0.22,51.0	0
0.22,51.1	0
0.22,51.2	0
0.22,51.3	0
0.22,51.4	0
0.22,51.5	0
0.22,51.6	0
0.22,51.7	0
0.22,51.8	0
0.22,51.9	0
0.22,52.0	0
0.22,52.1	0
0.22,52.2	0
0.22,52.3	0

0.22,52.4	0
0.22,52.5	0
0.22,52.6	0
0.22,52.7	0
0.22,52.8	0
0.22,52.9	0
0.22,53.0	0
0.22,53.1	0
0.22,53.2	0
0.22,53.3	0
0.22,53.4	0
0.22,53.5	0
0.22,53.6	0
0.22,53.7	0
0.22,53.8	0
0.22,53.9	0
0.22,54.0	0
0.22,54.1	0
0.22,54.2	0
0.22,54.3	0
0.22,54.4	0
0.22,54.5	0
0.22,54.6	0
0.22,54.7	0
0.22,54.8	0
0.22,54.9	0
0.22,55.0	0
0.22,55.1	0
0.22,55.2	0
0.22,55.3	0
0.22,55.4	0
0.22,55.5	0
0.22,55.6	0
0.22,55.7	0
0.22,55.8	0
0.22,55.9	0
0.22,56.0	0
0.22,56.1	0
0.22,56.2	0
0.22,56.3	0
0.22,56.4	0
0.22,56.5	0

0.22,56.6	0
0.22,56.7	0
0.22,56.8	0
0.22,56.9	0
0.22,57.0	0
0.22,57.1	0
0.22,57.2	0
0.22,57.3	0
0.22,57.4	0
0.22,57.5	0
0.22,57.6	0
0.22,57.7	0
0.22,57.8	0
0.22,57.9	0
0.22,58.0	0
0.22,58.1	0
0.22,58.2	0
0.22,58.3	0
0.22,58.4	0
0.22,58.5	0
0.22,58.6	0
0.22,58.7	0
0.22,58.8	0
0.22,58.9	0
0.22,59.0	0
0.22,59.1	0
0.22,59.2	0
0.22,59.3	0
0.22,59.4	0
0.22,59.5	0
0.22,59.6	0
0.22,59.7	0
0.22,59.8	0
0.22,59.9	0
0.22,60.0	0
0.22,60.1	0
0.22,60.2	0
0.22,60.3	0
0.22,60.4	0
0.22,60.5	0
0.22,60.6	0
0.22,60.7	0

0.22,60.8	0
0.22,60.9	0
0.22,61.0	0
0.22,61.1	0
0.22,61.2	0
0.22,61.3	0
0.22,61.4	0
0.22,61.5	0
0.22,61.6	0
0.22,61.7	0
0.22,61.8	0
0.22,61.9	0
0.22,62.0	0
0.22,62.1	0
0.22,62.2	0
0.22,62.3	0
0.22,62.4	0
0.22,62.5	0
0.22,62.6	0
0.22,62.7	0
0.22,62.8	0
0.22,62.9	0
0.22,63.0	0
0.22,63.1	0
0.22,63.2	0
0.22,63.3	0
0.22,63.4	0
0.22,63.5	0
0.22,63.6	0
0.22,63.7	0
0.22,63.8	0
0.22,63.9	0
0.22,64.0	0
0.22,64.1	0
0.22,64.2	0
0.22,64.3	0
0.22,64.4	0
0.22,64.5	0
0.22,64.6	0
0.22,64.7	0
0.22,64.8	0
0.22,64.9	0

0.22,65.0	0
0.22,65.1	0
0.22,65.2	0
0.22,65.3	0
0.22,65.4	0
0.22,65.5	0
0.22,65.6	0
0.22,65.7	0
0.22,65.8	0
0.22,65.9	0
0.22,66.0	0
0.22,66.1	0
0.22,66.2	0
0.22,66.3	0
0.22,66.4	0
0.22,66.5	0
0.22,66.6	0
0.22,66.7	0
0.22,66.8	0
0.22,66.9	0
0.22,67.0	0
0.22,67.1	0
0.22,67.2	0
0.22,67.3	0
0.22,67.4	0
0.22,67.5	0
0.22,67.6	0
0.22,67.7	0
0.22,67.8	0
0.22,67.9	0
0.22,68.0	0
0.22,68.1	0
0.22,68.2	0
0.22,68.3	0
0.22,68.4	0
0.22,68.5	0
0.22,68.6	0
0.22,68.7	0
0.22,68.8	0
0.22,68.9	0
0.22,69.0	0
0.22,69.1	0

0.22,69.2	0
0.22,69.3	0
0.22,69.4	0
0.22,69.5	0
0.22,69.6	0
0.22,69.7	0
0.22,69.8	0
0.22,69.9	0
0.22,70.0	0
0.22,70.1	0
0.22,70.2	0
0.22,70.3	0
0.22,70.4	0
0.22,70.5	0
0.22,70.6	0
0.22,70.7	0
0.22,70.8	0
0.22,70.9	0
0.22,71.0	0
0.22,71.1	0
0.22,71.2	0
0.22,71.3	0
0.22,71.4	0
0.22,71.5	0
0.22,71.6	0
0.22,71.7	0
0.22,71.8	0
0.22,71.9	0
0.22,72.0	0
0.22,72.1	0
0.22,72.2	0
0.22,72.3	0
0.22,72.4	0
0.22,72.5	0
0.22,72.6	0
0.22,72.7	0
0.22,72.8	0
0.22,72.9	0
0.22,73.0	0
0.22,73.1	0
0.22,73.2	0
0.22,73.3	0

0.22,73.4	0
0.22,73.5	0
0.22,73.6	0
0.22,73.7	0
0.22,73.8	0
0.22,73.9	0
0.22,74.0	0
0.22,74.1	0
0.22,74.2	0
0.22,74.3	0
0.22,74.4	0
0.22,74.5	0
0.22,74.6	0
0.22,74.7	0
0.22,74.8	0
0.22,74.9	0
0.22,75.0	0
0.22,75.1	0
0.22,75.2	0
0.22,75.3	0
0.22,75.4	0
0.22,75.5	0
0.22,75.6	0
0.22,75.7	0
0.22,75.8	0
0.22,75.9	0
0.22,76.0	0
0.22,76.1	0
0.22,76.2	0
0.22,76.3	0
0.22,76.4	0
0.22,76.5	0
0.22,76.6	0
0.22,76.7	0
0.22,76.8	0
0.22,76.9	0
0.22,77.0	0
0.22,77.1	0
0.22,77.2	0
0.22,77.3	0
0.22,77.4	0
0.22,77.5	0

0.22,77.6	0
0.22,77.7	0
0.22,77.8	0
0.22,77.9	0
0.22,78.0	0
0.22,78.1	0
0.22,78.2	0
0.22,78.3	0
0.22,78.4	0
0.22,78.5	0
0.22,78.6	0
0.22,78.7	0
0.22,78.8	0
0.22,78.9	0
0.22,79.0	0
0.22,79.1	0
0.22,79.2	0
0.22,79.3	0
0.22,79.4	0
0.22,79.5	0
0.22,79.6	0
0.22,79.7	0
0.22,79.8	0
0.22,79.9	0
0.22,80.0	0
0.22,80.1	0
0.22,80.2	0
0.22,80.3	0
0.22,80.4	0
0.22,80.5	0
0.22,80.6	0
0.22,80.7	0
0.22,80.8	0
0.22,80.9	0
0.22,81.0	0
0.22,81.1	0
0.22,81.2	0
0.22,81.3	0
0.22,81.4	0
0.22,81.5	0
0.22,81.6	0
0.22,81.7	0

0.22,81.8	0
0.22,81.9	0
0.22,82.0	0
0.22,82.1	0
0.22,82.2	0
0.22,82.3	0
0.22,82.4	0
0.22,82.5	0
0.22,82.6	0
0.22,82.7	0
0.22,82.8	0
0.22,82.9	0
0.22,83.0	0
0.22,83.1	0
0.22,83.2	0
0.22,83.3	0
0.22,83.4	0
0.22,83.5	0
0.22,83.6	0
0.22,83.7	0
0.22,83.8	0
0.22,83.9	0
0.22,84.0	0
0.22,84.1	0
0.22,84.2	0
0.22,84.3	0
0.22,84.4	0
0.22,84.5	0
0.22,84.6	0
0.22,84.7	0
0.22,84.8	0
0.22,84.9	0
0.22,85.0	0
0.22,85.1	0
0.22,85.2	0
0.22,85.3	0
0.22,85.4	0
0.22,85.5	0
0.22,85.6	0
0.22,85.7	0
0.22,85.8	0
0.22,85.9	0

0.22,86.0	0
0.22,86.1	0
0.22,86.2	0
0.22,86.3	0
0.22,86.4	0
0.22,86.5	0
0.22,86.6	0
0.22,86.7	0
0.22,86.8	0
0.22,86.9	0
0.22,87.0	0
0.22,87.1	0
0.22,87.2	0
0.22,87.3	0
0.22,87.4	0
0.22,87.5	0
0.22,87.6	0
0.22,87.7	0
0.22,87.8	0
0.22,87.9	0
0.22,88.0	0
0.22,88.1	0
0.22,88.2	0
0.22,88.3	0
0.22,88.4	0
0.22,88.5	0
0.22,88.6	0
0.22,88.7	0
0.22,88.8	0
0.22,88.9	0
0.22,89.0	0
0.22,89.1	0
0.22,89.2	0
0.22,89.3	0
0.22,89.4	0
0.22,89.5	0
0.22,89.6	0
0.22,89.7	0
0.22,89.8	0
0.22,89.9	0
0.22,90.0	0
0.22,90.1	0

0.22,90.2	0
0.22,90.3	0
0.22,90.4	0
0.22,90.5	0
0.22,90.6	0
0.22,90.7	0
0.22,90.8	0
0.22,90.9	0
0.22,91.0	0
0.22,91.1	0
0.22,91.2	0
0.22,91.3	0
0.22,91.4	0
0.22,91.5	0
0.22,91.6	0
0.22,91.7	0
0.22,91.8	0
0.22,91.9	0
0.22,92.0	0
0.22,92.1	0
0.22,92.2	0
0.22,92.3	0
0.22,92.4	0
0.22,92.5	0
0.22,92.6	0
0.22,92.7	0
0.22,92.8	0
0.22,92.9	0
0.22,93.0	0
0.22,93.1	0
0.22,93.2	0
0.22,93.3	0
0.22,93.4	0
0.22,93.5	0
0.22,93.6	0
0.22,93.7	0
0.22,93.8	0
0.22,93.9	0
0.22,94.0	0
0.22,94.1	0
0.22,94.2	0
0.22,94.3	0

0.22,94.4	0
0.22,94.5	0
0.22,94.6	0
0.22,94.7	0
0.22,94.8	0
0.22,94.9	0
0.22,95.0	0
0.22,95.1	0
0.22,95.2	0
0.22,95.3	0
0.22,95.4	0
0.22,95.5	0
0.22,95.6	0
0.22,95.7	0
0.22,95.8	0
0.22,95.9	0
0.22,96.0	0
0.22,96.1	0
0.22,96.2	0
0.22,96.3	0
0.22,96.4	0
0.22,96.5	0
0.22,96.6	0
0.22,96.7	0
0.22,96.8	0
0.22,96.9	0
0.22,97.0	0
0.22,97.1	0
0.22,97.2	0
0.22,97.3	0
0.22,97.4	0
0.22,97.5	0
0.22,97.6	0
0.22,97.7	0
0.22,97.8	0
0.22,97.9	0
0.22,98.0	0
0.22,98.1	0
0.22,98.2	0
0.22,98.3	0
0.22,98.4	0
0.22,98.5	0

0.22,98.6	0
0.22,98.7	0
0.22,98.8	0
0.22,98.9	0
0.22,99.0	0
0.22,99.1	0
0.22,99.2	0
0.22,99.3	0
0.22,99.4	0
0.22,99.5	0
0.22,99.6	0
0.22,99.7	0
0.22,99.8	0
0.22,99.9	0
0.22,100.0	0
0.23,0.0	0
0.23,0.1	0
0.23,0.2	0
0.23,0.3	0
0.23,0.4	0
0.23,0.5	0
0.23,0.6	0
0.23,0.7	0
0.23,0.8	0
0.23,0.9	0
0.23,1.0	0
0.23,1.1	0
0.23,1.2	0
0.23,1.3	0
0.23,1.4	0
0.23,1.5	0
0.23,1.6	0
0.23,1.7	0
0.23,1.8	0
0.23,1.9	0
0.23,2.0	0
0.23,2.1	0
0.23,2.2	0
0.23,2.3	0
0.23,2.4	0
0.23,2.5	0
0.23,2.6	0

0.23,2.7	0
0.23,2.8	0
0.23,2.9	0
0.23,3.0	0
0.23,3.1	0
0.23,3.2	0
0.23,3.3	0
0.23,3.4	0
0.23,3.5	0
0.23,3.6	0
0.23,3.7	0
0.23,3.8	0
0.23,3.9	0
0.23,4.0	0
0.23,4.1	0
0.23,4.2	0
0.23,4.3	0
0.23,4.4	0
0.23,4.5	0
0.23,4.6	0
0.23,4.7	0
0.23,4.8	0
0.23,4.9	0
0.23,5.0	0
0.23,5.1	0
0.23,5.2	0
0.23,5.3	0
0.23,5.4	0
0.23,5.5	0
0.23,5.6	0
0.23,5.7	0
0.23,5.8	0
0.23,5.9	0
0.23,6.0	0
0.23,6.1	0
0.23,6.2	0
0.23,6.3	0
0.23,6.4	0
0.23,6.5	0
0.23,6.6	0
0.23,6.7	0
0.23,6.8	0

0.23,6.9	0
0.23,7.0	0
0.23,7.1	0
0.23,7.2	0
0.23,7.3	0
0.23,7.4	0
0.23,7.5	0
0.23,7.6	0
0.23,7.7	0
0.23,7.8	0
0.23,7.9	0
0.23,8.0	0
0.23,8.1	0
0.23,8.2	0
0.23,8.3	0
0.23,8.4	0
0.23,8.5	0
0.23,8.6	0
0.23,8.7	0
0.23,8.8	0
0.23,8.9	0
0.23,9.0	0
0.23,9.1	0
0.23,9.2	0
0.23,9.3	0
0.23,9.4	0
0.23,9.5	0
0.23,9.6	0
0.23,9.7	0
0.23,9.8	0
0.23,9.9	0
0.23,10.0	0
0.23,10.1	0
0.23,10.2	0
0.23,10.3	0
0.23,10.4	0
0.23,10.5	0
0.23,10.6	0
0.23,10.7	0
0.23,10.8	0
0.23,10.9	0
0.23,11.0	0

0.23,11.1	0
0.23,11.2	0
0.23,11.3	0
0.23,11.4	0
0.23,11.5	0
0.23,11.6	0
0.23,11.7	0
0.23,11.8	0
0.23,11.9	0
0.23,12.0	0
0.23,12.1	0
0.23,12.2	0
0.23,12.3	0
0.23,12.4	0
0.23,12.5	0
0.23,12.6	0
0.23,12.7	0
0.23,12.8	0
0.23,12.9	0
0.23,13.0	0
0.23,13.1	0
0.23,13.2	0
0.23,13.3	0
0.23,13.4	0
0.23,13.5	0
0.23,13.6	0
0.23,13.7	0
0.23,13.8	0
0.23,13.9	0
0.23,14.0	0
0.23,14.1	0
0.23,14.2	0
0.23,14.3	0
0.23,14.4	0
0.23,14.5	0
0.23,14.6	0
0.23,14.7	0
0.23,14.8	0
0.23,14.9	0
0.23,15.0	0
0.23,15.1	0
0.23,15.2	0

0.23,15.3	0
0.23,15.4	0
0.23,15.5	0
0.23,15.6	0
0.23,15.7	0
0.23,15.8	0
0.23,15.9	0
0.23,16.0	0
0.23,16.1	0
0.23,16.2	0
0.23,16.3	0
0.23,16.4	0
0.23,16.5	0
0.23,16.6	0
0.23,16.7	0
0.23,16.8	0
0.23,16.9	0
0.23,17.0	0
0.23,17.1	0
0.23,17.2	0
0.23,17.3	0
0.23,17.4	0
0.23,17.5	0
0.23,17.6	0
0.23,17.7	0
0.23,17.8	0
0.23,17.9	0
0.23,18.0	0
0.23,18.1	0
0.23,18.2	0
0.23,18.3	0
0.23,18.4	0
0.23,18.5	0
0.23,18.6	0
0.23,18.7	0
0.23,18.8	0
0.23,18.9	0
0.23,19.0	0
0.23,19.1	0
0.23,19.2	0
0.23,19.3	0
0.23,19.4	0

0.23,19.5	0
0.23,19.6	0
0.23,19.7	0
0.23,19.8	0
0.23,19.9	0
0.23,20.0	0
0.23,20.1	0
0.23,20.2	0
0.23,20.3	0
0.23,20.4	0
0.23,20.5	0
0.23,20.6	0
0.23,20.7	0
0.23,20.8	0
0.23,20.9	0
0.23,21.0	0
0.23,21.1	0
0.23,21.2	0
0.23,21.3	0
0.23,21.4	0
0.23,21.5	0
0.23,21.6	0
0.23,21.7	0
0.23,21.8	0
0.23,21.9	0
0.23,22.0	0
0.23,22.1	0
0.23,22.2	0
0.23,22.3	0
0.23,22.4	0
0.23,22.5	0
0.23,22.6	0
0.23,22.7	0
0.23,22.8	0
0.23,22.9	0
0.23,23.0	0
0.23,23.1	0
0.23,23.2	0
0.23,23.3	0
0.23,23.4	0
0.23,23.5	0
0.23,23.6	0

0.23,23.7	0
0.23,23.8	0
0.23,23.9	0
0.23,24.0	0
0.23,24.1	0
0.23,24.2	0
0.23,24.3	0
0.23,24.4	0
0.23,24.5	0
0.23,24.6	0
0.23,24.7	0
0.23,24.8	0
0.23,24.9	0
0.23,25.0	0
0.23,25.1	0
0.23,25.2	0
0.23,25.3	0
0.23,25.4	0
0.23,25.5	0
0.23,25.6	0
0.23,25.7	0
0.23,25.8	0
0.23,25.9	0
0.23,26.0	0
0.23,26.1	0
0.23,26.2	0
0.23,26.3	0
0.23,26.4	0
0.23,26.5	0
0.23,26.6	0
0.23,26.7	0
0.23,26.8	0
0.23,26.9	0
0.23,27.0	0
0.23,27.1	0
0.23,27.2	0
0.23,27.3	0
0.23,27.4	0
0.23,27.5	0
0.23,27.6	0
0.23,27.7	0
0.23,27.8	0

0.23,27.9	0
0.23,28.0	0
0.23,28.1	0
0.23,28.2	0
0.23,28.3	0
0.23,28.4	0
0.23,28.5	0
0.23,28.6	0
0.23,28.7	0
0.23,28.8	0
0.23,28.9	0
0.23,29.0	0
0.23,29.1	0
0.23,29.2	0
0.23,29.3	0
0.23,29.4	0
0.23,29.5	0
0.23,29.6	0
0.23,29.7	0
0.23,29.8	0
0.23,29.9	0
0.23,30.0	0
0.23,30.1	0
0.23,30.2	0
0.23,30.3	0
0.23,30.4	0
0.23,30.5	0
0.23,30.6	0
0.23,30.7	0
0.23,30.8	0
0.23,30.9	0
0.23,31.0	0
0.23,31.1	0
0.23,31.2	0
0.23,31.3	0
0.23,31.4	0
0.23,31.5	0
0.23,31.6	0
0.23,31.7	0
0.23,31.8	0
0.23,31.9	0
0.23,32.0	0

0.23,32.1	0
0.23,32.2	0
0.23,32.3	0
0.23,32.4	0
0.23,32.5	0
0.23,32.6	0
0.23,32.7	0
0.23,32.8	0
0.23,32.9	0
0.23,33.0	0
0.23,33.1	0
0.23,33.2	0
0.23,33.3	0
0.23,33.4	0
0.23,33.5	0
0.23,33.6	0
0.23,33.7	0
0.23,33.8	0
0.23,33.9	0
0.23,34.0	0
0.23,34.1	0
0.23,34.2	0
0.23,34.3	0
0.23,34.4	0
0.23,34.5	0
0.23,34.6	0
0.23,34.7	0
0.23,34.8	0
0.23,34.9	0
0.23,35.0	0
0.23,35.1	0
0.23,35.2	0
0.23,35.3	0
0.23,35.4	0
0.23,35.5	0
0.23,35.6	0
0.23,35.7	0
0.23,35.8	0
0.23,35.9	0
0.23,36.0	0
0.23,36.1	0
0.23,36.2	0

0.23,36.3	0
0.23,36.4	0
0.23,36.5	0
0.23,36.6	0
0.23,36.7	0
0.23,36.8	0
0.23,36.9	0
0.23,37.0	0
0.23,37.1	0
0.23,37.2	0
0.23,37.3	0
0.23,37.4	0
0.23,37.5	0
0.23,37.6	0
0.23,37.7	0
0.23,37.8	0
0.23,37.9	0
0.23,38.0	0
0.23,38.1	0
0.23,38.2	0
0.23,38.3	0
0.23,38.4	0
0.23,38.5	0
0.23,38.6	0
0.23,38.7	0
0.23,38.8	0
0.23,38.9	0
0.23,39.0	0
0.23,39.1	0
0.23,39.2	0
0.23,39.3	0
0.23,39.4	0
0.23,39.5	0
0.23,39.6	0
0.23,39.7	0
0.23,39.8	0
0.23,39.9	0
0.23,40.0	0
0.23,40.1	0
0.23,40.2	0
0.23,40.3	0
0.23,40.4	0

0.23,40.5	0
0.23,40.6	0
0.23,40.7	0
0.23,40.8	0
0.23,40.9	0
0.23,41.0	0
0.23,41.1	0
0.23,41.2	0
0.23,41.3	0
0.23,41.4	0
0.23,41.5	0
0.23,41.6	0
0.23,41.7	0
0.23,41.8	0
0.23,41.9	0
0.23,42.0	0
0.23,42.1	0
0.23,42.2	0
0.23,42.3	0
0.23,42.4	0
0.23,42.5	0
0.23,42.6	0
0.23,42.7	0
0.23,42.8	0
0.23,42.9	0
0.23,43.0	0
0.23,43.1	0
0.23,43.2	0
0.23,43.3	0
0.23,43.4	0
0.23,43.5	0
0.23,43.6	0
0.23,43.7	0
0.23,43.8	0
0.23,43.9	0
0.23,44.0	0
0.23,44.1	0
0.23,44.2	0
0.23,44.3	0
0.23,44.4	0
0.23,44.5	0
0.23,44.6	0

0.23,44.7	0
0.23,44.8	0
0.23,44.9	0
0.23,45.0	0
0.23,45.1	0
0.23,45.2	0
0.23,45.3	0
0.23,45.4	0
0.23,45.5	0
0.23,45.6	0
0.23,45.7	0
0.23,45.8	0
0.23,45.9	0
0.23,46.0	0
0.23,46.1	0
0.23,46.2	0
0.23,46.3	0
0.23,46.4	0
0.23,46.5	0
0.23,46.6	0
0.23,46.7	0
0.23,46.8	0
0.23,46.9	0
0.23,47.0	0
0.23,47.1	0
0.23,47.2	0
0.23,47.3	0
0.23,47.4	0
0.23,47.5	0
0.23,47.6	0
0.23,47.7	0
0.23,47.8	0
0.23,47.9	0
0.23,48.0	0
0.23,48.1	0
0.23,48.2	0
0.23,48.3	0
0.23,48.4	0
0.23,48.5	0
0.23,48.6	0
0.23,48.7	0
0.23,48.8	0

0.23,48.9	0
0.23,49.0	0
0.23,49.1	0
0.23,49.2	0
0.23,49.3	0
0.23,49.4	0
0.23,49.5	0
0.23,49.6	0
0.23,49.7	0
0.23,49.8	0
0.23,49.9	0
0.23,50.0	0
0.23,50.1	0
0.23,50.2	0
0.23,50.3	0
0.23,50.4	0
0.23,50.5	0
0.23,50.6	0
0.23,50.7	0
0.23,50.8	0
0.23,50.9	0
0.23,51.0	0
0.23,51.1	0
0.23,51.2	0
0.23,51.3	0
0.23,51.4	0
0.23,51.5	0
0.23,51.6	0
0.23,51.7	0
0.23,51.8	0
0.23,51.9	0
0.23,52.0	0
0.23,52.1	0
0.23,52.2	0
0.23,52.3	0
0.23,52.4	0
0.23,52.5	0
0.23,52.6	0
0.23,52.7	0
0.23,52.8	0
0.23,52.9	0
0.23,53.0	0

0.23,53.1	0
0.23,53.2	0
0.23,53.3	0
0.23,53.4	0
0.23,53.5	0
0.23,53.6	0
0.23,53.7	0
0.23,53.8	0
0.23,53.9	0
0.23,54.0	0
0.23,54.1	0
0.23,54.2	0
0.23,54.3	0
0.23,54.4	0
0.23,54.5	0
0.23,54.6	0
0.23,54.7	0
0.23,54.8	0
0.23,54.9	0
0.23,55.0	0
0.23,55.1	0
0.23,55.2	0
0.23,55.3	0
0.23,55.4	0
0.23,55.5	0
0.23,55.6	0
0.23,55.7	0
0.23,55.8	0
0.23,55.9	0
0.23,56.0	0
0.23,56.1	0
0.23,56.2	0
0.23,56.3	0
0.23,56.4	0
0.23,56.5	0
0.23,56.6	0
0.23,56.7	0
0.23,56.8	0
0.23,56.9	0
0.23,57.0	0
0.23,57.1	0
0.23,57.2	0

0.23,57.3	0
0.23,57.4	0
0.23,57.5	0
0.23,57.6	0
0.23,57.7	0
0.23,57.8	0
0.23,57.9	0
0.23,58.0	0
0.23,58.1	0
0.23,58.2	0
0.23,58.3	0
0.23,58.4	0
0.23,58.5	0
0.23,58.6	0
0.23,58.7	0
0.23,58.8	0
0.23,58.9	0
0.23,59.0	0
0.23,59.1	0
0.23,59.2	0
0.23,59.3	0
0.23,59.4	0
0.23,59.5	0
0.23,59.6	0
0.23,59.7	0
0.23,59.8	0
0.23,59.9	0
0.23,60.0	0
0.23,60.1	0
0.23,60.2	0
0.23,60.3	0
0.23,60.4	0
0.23,60.5	0
0.23,60.6	0
0.23,60.7	0
0.23,60.8	0
0.23,60.9	0
0.23,61.0	0
0.23,61.1	0
0.23,61.2	0
0.23,61.3	0
0.23,61.4	0

0.23,61.5	0
0.23,61.6	0
0.23,61.7	0
0.23,61.8	0
0.23,61.9	0
0.23,62.0	0
0.23,62.1	0
0.23,62.2	0
0.23,62.3	0
0.23,62.4	0
0.23,62.5	0
0.23,62.6	0
0.23,62.7	0
0.23,62.8	0
0.23,62.9	0
0.23,63.0	0
0.23,63.1	0
0.23,63.2	0
0.23,63.3	0
0.23,63.4	0
0.23,63.5	0
0.23,63.6	0
0.23,63.7	0
0.23,63.8	0
0.23,63.9	0
0.23,64.0	0
0.23,64.1	0
0.23,64.2	0
0.23,64.3	0
0.23,64.4	0
0.23,64.5	0
0.23,64.6	0
0.23,64.7	0
0.23,64.8	0
0.23,64.9	0
0.23,65.0	0
0.23,65.1	0
0.23,65.2	0
0.23,65.3	0
0.23,65.4	0
0.23,65.5	0
0.23,65.6	0

0.23,65.7	0
0.23,65.8	0
0.23,65.9	0
0.23,66.0	0
0.23,66.1	0
0.23,66.2	0
0.23,66.3	0
0.23,66.4	0
0.23,66.5	0
0.23,66.6	0
0.23,66.7	0
0.23,66.8	0
0.23,66.9	0
0.23,67.0	0
0.23,67.1	0
0.23,67.2	0
0.23,67.3	0
0.23,67.4	0
0.23,67.5	0
0.23,67.6	0
0.23,67.7	0
0.23,67.8	0
0.23,67.9	0
0.23,68.0	0
0.23,68.1	0
0.23,68.2	0
0.23,68.3	0
0.23,68.4	0
0.23,68.5	0
0.23,68.6	0
0.23,68.7	0
0.23,68.8	0
0.23,68.9	0
0.23,69.0	0
0.23,69.1	0
0.23,69.2	0
0.23,69.3	0
0.23,69.4	0
0.23,69.5	0
0.23,69.6	0
0.23,69.7	0
0.23,69.8	0

0.23,69.9	0
0.23,70.0	0
0.23,70.1	0
0.23,70.2	0
0.23,70.3	0
0.23,70.4	0
0.23,70.5	0
0.23,70.6	0
0.23,70.7	0
0.23,70.8	0
0.23,70.9	0
0.23,71.0	0
0.23,71.1	0
0.23,71.2	0
0.23,71.3	0
0.23,71.4	0
0.23,71.5	0
0.23,71.6	0
0.23,71.7	0
0.23,71.8	0
0.23,71.9	0
0.23,72.0	0
0.23,72.1	0
0.23,72.2	0
0.23,72.3	0
0.23,72.4	0
0.23,72.5	0
0.23,72.6	0
0.23,72.7	0
0.23,72.8	0
0.23,72.9	0
0.23,73.0	0
0.23,73.1	0
0.23,73.2	0
0.23,73.3	0
0.23,73.4	0
0.23,73.5	0
0.23,73.6	0
0.23,73.7	0
0.23,73.8	0
0.23,73.9	0
0.23,74.0	0

0.23,74.1	0
0.23,74.2	0
0.23,74.3	0
0.23,74.4	0
0.23,74.5	0
0.23,74.6	0
0.23,74.7	0
0.23,74.8	0
0.23,74.9	0
0.23,75.0	0
0.23,75.1	0
0.23,75.2	0
0.23,75.3	0
0.23,75.4	0
0.23,75.5	0
0.23,75.6	0
0.23,75.7	0
0.23,75.8	0
0.23,75.9	0
0.23,76.0	0
0.23,76.1	0
0.23,76.2	0
0.23,76.3	0
0.23,76.4	0
0.23,76.5	0
0.23,76.6	0
0.23,76.7	0
0.23,76.8	0
0.23,76.9	0
0.23,77.0	0
0.23,77.1	0
0.23,77.2	0
0.23,77.3	0
0.23,77.4	0
0.23,77.5	0
0.23,77.6	0
0.23,77.7	0
0.23,77.8	0
0.23,77.9	0
0.23,78.0	0
0.23,78.1	0
0.23,78.2	0

0.23,78.3	0
0.23,78.4	0
0.23,78.5	0
0.23,78.6	0
0.23,78.7	0
0.23,78.8	0
0.23,78.9	0
0.23,79.0	0
0.23,79.1	0
0.23,79.2	0
0.23,79.3	0
0.23,79.4	0
0.23,79.5	0
0.23,79.6	0
0.23,79.7	0
0.23,79.8	0
0.23,79.9	0
0.23,80.0	0
0.23,80.1	0
0.23,80.2	0
0.23,80.3	0
0.23,80.4	0
0.23,80.5	0
0.23,80.6	0
0.23,80.7	0
0.23,80.8	0
0.23,80.9	0
0.23,81.0	0
0.23,81.1	0
0.23,81.2	0
0.23,81.3	0
0.23,81.4	0
0.23,81.5	0
0.23,81.6	0
0.23,81.7	0
0.23,81.8	0
0.23,81.9	0
0.23,82.0	0
0.23,82.1	0
0.23,82.2	0
0.23,82.3	0
0.23,82.4	0

0.23,82.5	0
0.23,82.6	0
0.23,82.7	0
0.23,82.8	0
0.23,82.9	0
0.23,83.0	0
0.23,83.1	0
0.23,83.2	0
0.23,83.3	0
0.23,83.4	0
0.23,83.5	0
0.23,83.6	0
0.23,83.7	0
0.23,83.8	0
0.23,83.9	0
0.23,84.0	0
0.23,84.1	0
0.23,84.2	0
0.23,84.3	0
0.23,84.4	0
0.23,84.5	0
0.23,84.6	0
0.23,84.7	0
0.23,84.8	0
0.23,84.9	0
0.23,85.0	0
0.23,85.1	0
0.23,85.2	0
0.23,85.3	0
0.23,85.4	0
0.23,85.5	0
0.23,85.6	0
0.23,85.7	0
0.23,85.8	0
0.23,85.9	0
0.23,86.0	0
0.23,86.1	0
0.23,86.2	0
0.23,86.3	0
0.23,86.4	0
0.23,86.5	0
0.23,86.6	0

0.23,86.7	0
0.23,86.8	0
0.23,86.9	0
0.23,87.0	0
0.23,87.1	0
0.23,87.2	0
0.23,87.3	0
0.23,87.4	0
0.23,87.5	0
0.23,87.6	0
0.23,87.7	0
0.23,87.8	0
0.23,87.9	0
0.23,88.0	0
0.23,88.1	0
0.23,88.2	0
0.23,88.3	0
0.23,88.4	0
0.23,88.5	0
0.23,88.6	0
0.23,88.7	0
0.23,88.8	0
0.23,88.9	0
0.23,89.0	0
0.23,89.1	0
0.23,89.2	0
0.23,89.3	0
0.23,89.4	0
0.23,89.5	0
0.23,89.6	0
0.23,89.7	0
0.23,89.8	0
0.23,89.9	0
0.23,90.0	0
0.23,90.1	0
0.23,90.2	0
0.23,90.3	0
0.23,90.4	0
0.23,90.5	0
0.23,90.6	0
0.23,90.7	0
0.23,90.8	0

0.23,90.9	0
0.23,91.0	0
0.23,91.1	0
0.23,91.2	0
0.23,91.3	0
0.23,91.4	0
0.23,91.5	0
0.23,91.6	0
0.23,91.7	0
0.23,91.8	0
0.23,91.9	0
0.23,92.0	0
0.23,92.1	0
0.23,92.2	0
0.23,92.3	0
0.23,92.4	0
0.23,92.5	0
0.23,92.6	0
0.23,92.7	0
0.23,92.8	0
0.23,92.9	0
0.23,93.0	0
0.23,93.1	0
0.23,93.2	0
0.23,93.3	0
0.23,93.4	0
0.23,93.5	0
0.23,93.6	0
0.23,93.7	0
0.23,93.8	0
0.23,93.9	0
0.23,94.0	0
0.23,94.1	0
0.23,94.2	0
0.23,94.3	0
0.23,94.4	0
0.23,94.5	0
0.23,94.6	0
0.23,94.7	0
0.23,94.8	0
0.23,94.9	0
0.23,95.0	0

0.23,95.1	0
0.23,95.2	0
0.23,95.3	0
0.23,95.4	0
0.23,95.5	0
0.23,95.6	0
0.23,95.7	0
0.23,95.8	0
0.23,95.9	0
0.23,96.0	0
0.23,96.1	0
0.23,96.2	0
0.23,96.3	0
0.23,96.4	0
0.23,96.5	0
0.23,96.6	0
0.23,96.7	0
0.23,96.8	0
0.23,96.9	0
0.23,97.0	0
0.23,97.1	0
0.23,97.2	0
0.23,97.3	0
0.23,97.4	0
0.23,97.5	0
0.23,97.6	0
0.23,97.7	0
0.23,97.8	0
0.23,97.9	0
0.23,98.0	0
0.23,98.1	0
0.23,98.2	0
0.23,98.3	0
0.23,98.4	0
0.23,98.5	0
0.23,98.6	0
0.23,98.7	0
0.23,98.8	0
0.23,98.9	0
0.23,99.0	0
0.23,99.1	0
0.23,99.2	0

0.23,99.3	0
0.23,99.4	0
0.23,99.5	0
0.23,99.6	0
0.23,99.7	0
0.23,99.8	0
0.23,99.9	0
0.23,100.0	0
0.24,0.0	0
0.24,0.1	0
0.24,0.2	0
0.24,0.3	0
0.24,0.4	0
0.24,0.5	0
0.24,0.6	0
0.24,0.7	0
0.24,0.8	0
0.24,0.9	0
0.24,1.0	0
0.24,1.1	0
0.24,1.2	0
0.24,1.3	0
0.24,1.4	0
0.24,1.5	0
0.24,1.6	0
0.24,1.7	0
0.24,1.8	0
0.24,1.9	0
0.24,2.0	0
0.24,2.1	0
0.24,2.2	0
0.24,2.3	0
0.24,2.4	0
0.24,2.5	0
0.24,2.6	0
0.24,2.7	0
0.24,2.8	0
0.24,2.9	0
0.24,3.0	0
0.24,3.1	0
0.24,3.2	0
0.24,3.3	0

0.24,3.4	0
0.24,3.5	0
0.24,3.6	0
0.24,3.7	0
0.24,3.8	0
0.24,3.9	0
0.24,4.0	0
0.24,4.1	0
0.24,4.2	0
0.24,4.3	0
0.24,4.4	0
0.24,4.5	0
0.24,4.6	0
0.24,4.7	0
0.24,4.8	0
0.24,4.9	0
0.24,5.0	0
0.24,5.1	0
0.24,5.2	0
0.24,5.3	0
0.24,5.4	0
0.24,5.5	0
0.24,5.6	0
0.24,5.7	0
0.24,5.8	0
0.24,5.9	0
0.24,6.0	0
0.24,6.1	0
0.24,6.2	0
0.24,6.3	0
0.24,6.4	0
0.24,6.5	0
0.24,6.6	0
0.24,6.7	0
0.24,6.8	0
0.24,6.9	0
0.24,7.0	0
0.24,7.1	0
0.24,7.2	0
0.24,7.3	0
0.24,7.4	0
0.24,7.5	0

0.24,7.6	0
0.24,7.7	0
0.24,7.8	0
0.24,7.9	0
0.24,8.0	0
0.24,8.1	0
0.24,8.2	0
0.24,8.3	0
0.24,8.4	0
0.24,8.5	0
0.24,8.6	0
0.24,8.7	0
0.24,8.8	0
0.24,8.9	0
0.24,9.0	0
0.24,9.1	0
0.24,9.2	0
0.24,9.3	0
0.24,9.4	0
0.24,9.5	0
0.24,9.6	0
0.24,9.7	0
0.24,9.8	0
0.24,9.9	0
0.24,10.0	0
0.24,10.1	0
0.24,10.2	0
0.24,10.3	0
0.24,10.4	0
0.24,10.5	0
0.24,10.6	0
0.24,10.7	0
0.24,10.8	0
0.24,10.9	0
0.24,11.0	0
0.24,11.1	0
0.24,11.2	0
0.24,11.3	0
0.24,11.4	0
0.24,11.5	0
0.24,11.6	0
0.24,11.7	0

0.24,11.8	0
0.24,11.9	0
0.24,12.0	0
0.24,12.1	0
0.24,12.2	0
0.24,12.3	0
0.24,12.4	0
0.24,12.5	0
0.24,12.6	0
0.24,12.7	0
0.24,12.8	0
0.24,12.9	0
0.24,13.0	0
0.24,13.1	0
0.24,13.2	0
0.24,13.3	0
0.24,13.4	0
0.24,13.5	0
0.24,13.6	0
0.24,13.7	0
0.24,13.8	0
0.24,13.9	0
0.24,14.0	0
0.24,14.1	0
0.24,14.2	0
0.24,14.3	0
0.24,14.4	0
0.24,14.5	0
0.24,14.6	0
0.24,14.7	0
0.24,14.8	0
0.24,14.9	0
0.24,15.0	0
0.24,15.1	0
0.24,15.2	0
0.24,15.3	0
0.24,15.4	0
0.24,15.5	0
0.24,15.6	0
0.24,15.7	0
0.24,15.8	0
0.24,15.9	0

0.24,16.0	0
0.24,16.1	0
0.24,16.2	0
0.24,16.3	0
0.24,16.4	0
0.24,16.5	0
0.24,16.6	0
0.24,16.7	0
0.24,16.8	0
0.24,16.9	0
0.24,17.0	0
0.24,17.1	0
0.24,17.2	0
0.24,17.3	0
0.24,17.4	0
0.24,17.5	0
0.24,17.6	0
0.24,17.7	0
0.24,17.8	0
0.24,17.9	0
0.24,18.0	0
0.24,18.1	0
0.24,18.2	0
0.24,18.3	0
0.24,18.4	0
0.24,18.5	0
0.24,18.6	0
0.24,18.7	0
0.24,18.8	0
0.24,18.9	0
0.24,19.0	0
0.24,19.1	0
0.24,19.2	0
0.24,19.3	0
0.24,19.4	0
0.24,19.5	0
0.24,19.6	0
0.24,19.7	0
0.24,19.8	0
0.24,19.9	0
0.24,20.0	0
0.24,20.1	0

0.24,20.2	0
0.24,20.3	0
0.24,20.4	0
0.24,20.5	0
0.24,20.6	0
0.24,20.7	0
0.24,20.8	0
0.24,20.9	0
0.24,21.0	0
0.24,21.1	0
0.24,21.2	0
0.24,21.3	0
0.24,21.4	0
0.24,21.5	0
0.24,21.6	0
0.24,21.7	0
0.24,21.8	0
0.24,21.9	0
0.24,22.0	0
0.24,22.1	0
0.24,22.2	0
0.24,22.3	0
0.24,22.4	0
0.24,22.5	0
0.24,22.6	0
0.24,22.7	0
0.24,22.8	0
0.24,22.9	0
0.24,23.0	0
0.24,23.1	0
0.24,23.2	0
0.24,23.3	0
0.24,23.4	0
0.24,23.5	0
0.24,23.6	0
0.24,23.7	0
0.24,23.8	0
0.24,23.9	0
0.24,24.0	0
0.24,24.1	0
0.24,24.2	0
0.24,24.3	0

0.24,24.4	0
0.24,24.5	0
0.24,24.6	0
0.24,24.7	0
0.24,24.8	0
0.24,24.9	0
0.24,25.0	0
0.24,25.1	0
0.24,25.2	0
0.24,25.3	0
0.24,25.4	0
0.24,25.5	0
0.24,25.6	0
0.24,25.7	0
0.24,25.8	0
0.24,25.9	0
0.24,26.0	0
0.24,26.1	0
0.24,26.2	0
0.24,26.3	0
0.24,26.4	0
0.24,26.5	0
0.24,26.6	0
0.24,26.7	0
0.24,26.8	0
0.24,26.9	0
0.24,27.0	0
0.24,27.1	0
0.24,27.2	0
0.24,27.3	0
0.24,27.4	0
0.24,27.5	0
0.24,27.6	0
0.24,27.7	0
0.24,27.8	0
0.24,27.9	0
0.24,28.0	0
0.24,28.1	0
0.24,28.2	0
0.24,28.3	0
0.24,28.4	0
0.24,28.5	0

0.24,28.6	0
0.24,28.7	0
0.24,28.8	0
0.24,28.9	0
0.24,29.0	0
0.24,29.1	0
0.24,29.2	0
0.24,29.3	0
0.24,29.4	0
0.24,29.5	0
0.24,29.6	0
0.24,29.7	0
0.24,29.8	0
0.24,29.9	0
0.24,30.0	0
0.24,30.1	0
0.24,30.2	0
0.24,30.3	0
0.24,30.4	0
0.24,30.5	0
0.24,30.6	0
0.24,30.7	0
0.24,30.8	0
0.24,30.9	0
0.24,31.0	0
0.24,31.1	0
0.24,31.2	0
0.24,31.3	0
0.24,31.4	0
0.24,31.5	0
0.24,31.6	0
0.24,31.7	0
0.24,31.8	0
0.24,31.9	0
0.24,32.0	0
0.24,32.1	0
0.24,32.2	0
0.24,32.3	0
0.24,32.4	0
0.24,32.5	0
0.24,32.6	0
0.24,32.7	0

0.24,32.8	0
0.24,32.9	0
0.24,33.0	0
0.24,33.1	0
0.24,33.2	0
0.24,33.3	0
0.24,33.4	0
0.24,33.5	0
0.24,33.6	0
0.24,33.7	0
0.24,33.8	0
0.24,33.9	0
0.24,34.0	0
0.24,34.1	0
0.24,34.2	0
0.24,34.3	0
0.24,34.4	0
0.24,34.5	0
0.24,34.6	0
0.24,34.7	0
0.24,34.8	0
0.24,34.9	0
0.24,35.0	0
0.24,35.1	0
0.24,35.2	0
0.24,35.3	0
0.24,35.4	0
0.24,35.5	0
0.24,35.6	0
0.24,35.7	0
0.24,35.8	0
0.24,35.9	0
0.24,36.0	0
0.24,36.1	0
0.24,36.2	0
0.24,36.3	0
0.24,36.4	0
0.24,36.5	0
0.24,36.6	0
0.24,36.7	0
0.24,36.8	0
0.24,36.9	0

0.24,37.0	0
0.24,37.1	0
0.24,37.2	0
0.24,37.3	0
0.24,37.4	0
0.24,37.5	0
0.24,37.6	0
0.24,37.7	0
0.24,37.8	0
0.24,37.9	0
0.24,38.0	0
0.24,38.1	0
0.24,38.2	0
0.24,38.3	0
0.24,38.4	0
0.24,38.5	0
0.24,38.6	0
0.24,38.7	0
0.24,38.8	0
0.24,38.9	0
0.24,39.0	0
0.24,39.1	0
0.24,39.2	0
0.24,39.3	0
0.24,39.4	0
0.24,39.5	0
0.24,39.6	0
0.24,39.7	0
0.24,39.8	0
0.24,39.9	0
0.24,40.0	0
0.24,40.1	0
0.24,40.2	0
0.24,40.3	0
0.24,40.4	0
0.24,40.5	0
0.24,40.6	0
0.24,40.7	0
0.24,40.8	0
0.24,40.9	0
0.24,41.0	0
0.24,41.1	0

0.24,41.2	0
0.24,41.3	0
0.24,41.4	0
0.24,41.5	0
0.24,41.6	0
0.24,41.7	0
0.24,41.8	0
0.24,41.9	0
0.24,42.0	0
0.24,42.1	0
0.24,42.2	0
0.24,42.3	0
0.24,42.4	0
0.24,42.5	0
0.24,42.6	0
0.24,42.7	0
0.24,42.8	0
0.24,42.9	0
0.24,43.0	0
0.24,43.1	0
0.24,43.2	0
0.24,43.3	0
0.24,43.4	0
0.24,43.5	0
0.24,43.6	0
0.24,43.7	0
0.24,43.8	0
0.24,43.9	0
0.24,44.0	0
0.24,44.1	0
0.24,44.2	0
0.24,44.3	0
0.24,44.4	0
0.24,44.5	0
0.24,44.6	0
0.24,44.7	0
0.24,44.8	0
0.24,44.9	0
0.24,45.0	0
0.24,45.1	0
0.24,45.2	0
0.24,45.3	0

0.24,45.4	0
0.24,45.5	0
0.24,45.6	0
0.24,45.7	0
0.24,45.8	0
0.24,45.9	0
0.24,46.0	0
0.24,46.1	0
0.24,46.2	0
0.24,46.3	0
0.24,46.4	0
0.24,46.5	0
0.24,46.6	0
0.24,46.7	0
0.24,46.8	0
0.24,46.9	0
0.24,47.0	0
0.24,47.1	0
0.24,47.2	0
0.24,47.3	0
0.24,47.4	0
0.24,47.5	0
0.24,47.6	0
0.24,47.7	0
0.24,47.8	0
0.24,47.9	0
0.24,48.0	0
0.24,48.1	0
0.24,48.2	0
0.24,48.3	0
0.24,48.4	0
0.24,48.5	0
0.24,48.6	0
0.24,48.7	0
0.24,48.8	0
0.24,48.9	0
0.24,49.0	0
0.24,49.1	0
0.24,49.2	0
0.24,49.3	0
0.24,49.4	0
0.24,49.5	0

0.24,49.6	0
0.24,49.7	0
0.24,49.8	0
0.24,49.9	0
0.24,50.0	0
0.24,50.1	0
0.24,50.2	0
0.24,50.3	0
0.24,50.4	0
0.24,50.5	0
0.24,50.6	0
0.24,50.7	0
0.24,50.8	0
0.24,50.9	0
0.24,51.0	0
0.24,51.1	0
0.24,51.2	0
0.24,51.3	0
0.24,51.4	0
0.24,51.5	0
0.24,51.6	0
0.24,51.7	0
0.24,51.8	0
0.24,51.9	0
0.24,52.0	0
0.24,52.1	0
0.24,52.2	0
0.24,52.3	0
0.24,52.4	0
0.24,52.5	0
0.24,52.6	0
0.24,52.7	0
0.24,52.8	0
0.24,52.9	0
0.24,53.0	0
0.24,53.1	0
0.24,53.2	0
0.24,53.3	0
0.24,53.4	0
0.24,53.5	0
0.24,53.6	0
0.24,53.7	0

0.24,53.8	0
0.24,53.9	0
0.24,54.0	0
0.24,54.1	0
0.24,54.2	0
0.24,54.3	0
0.24,54.4	0
0.24,54.5	0
0.24,54.6	0
0.24,54.7	0
0.24,54.8	0
0.24,54.9	0
0.24,55.0	0
0.24,55.1	0
0.24,55.2	0
0.24,55.3	0
0.24,55.4	0
0.24,55.5	0
0.24,55.6	0
0.24,55.7	0
0.24,55.8	0
0.24,55.9	0
0.24,56.0	0
0.24,56.1	0
0.24,56.2	0
0.24,56.3	0
0.24,56.4	0
0.24,56.5	0
0.24,56.6	0
0.24,56.7	0
0.24,56.8	0
0.24,56.9	0
0.24,57.0	0
0.24,57.1	0
0.24,57.2	0
0.24,57.3	0
0.24,57.4	0
0.24,57.5	0
0.24,57.6	0
0.24,57.7	0
0.24,57.8	0
0.24,57.9	0

0.24,58.0	0
0.24,58.1	0
0.24,58.2	0
0.24,58.3	0
0.24,58.4	0
0.24,58.5	0
0.24,58.6	0
0.24,58.7	0
0.24,58.8	0
0.24,58.9	0
0.24,59.0	0
0.24,59.1	0
0.24,59.2	0
0.24,59.3	0
0.24,59.4	0
0.24,59.5	0
0.24,59.6	0
0.24,59.7	0
0.24,59.8	0
0.24,59.9	0
0.24,60.0	0
0.24,60.1	0
0.24,60.2	0
0.24,60.3	0
0.24,60.4	0
0.24,60.5	0
0.24,60.6	0
0.24,60.7	0
0.24,60.8	0
0.24,60.9	0
0.24,61.0	0
0.24,61.1	0
0.24,61.2	0
0.24,61.3	0
0.24,61.4	0
0.24,61.5	0
0.24,61.6	0
0.24,61.7	0
0.24,61.8	0
0.24,61.9	0
0.24,62.0	0
0.24,62.1	0

0.24,62.2	0
0.24,62.3	0
0.24,62.4	0
0.24,62.5	0
0.24,62.6	0
0.24,62.7	0
0.24,62.8	0
0.24,62.9	0
0.24,63.0	0
0.24,63.1	0
0.24,63.2	0
0.24,63.3	0
0.24,63.4	0
0.24,63.5	0
0.24,63.6	0
0.24,63.7	0
0.24,63.8	0
0.24,63.9	0
0.24,64.0	0
0.24,64.1	0
0.24,64.2	0
0.24,64.3	0
0.24,64.4	0
0.24,64.5	0
0.24,64.6	0
0.24,64.7	0
0.24,64.8	0
0.24,64.9	0
0.24,65.0	0
0.24,65.1	0
0.24,65.2	0
0.24,65.3	0
0.24,65.4	0
0.24,65.5	0
0.24,65.6	0
0.24,65.7	0
0.24,65.8	0
0.24,65.9	0
0.24,66.0	0
0.24,66.1	0
0.24,66.2	0
0.24,66.3	0

0.24,66.4	0
0.24,66.5	0
0.24,66.6	0
0.24,66.7	0
0.24,66.8	0
0.24,66.9	0
0.24,67.0	0
0.24,67.1	0
0.24,67.2	0
0.24,67.3	0
0.24,67.4	0
0.24,67.5	0
0.24,67.6	0
0.24,67.7	0
0.24,67.8	0
0.24,67.9	0
0.24,68.0	0
0.24,68.1	0
0.24,68.2	0
0.24,68.3	0
0.24,68.4	0
0.24,68.5	0
0.24,68.6	0
0.24,68.7	0
0.24,68.8	0
0.24,68.9	0
0.24,69.0	0
0.24,69.1	0
0.24,69.2	0
0.24,69.3	0
0.24,69.4	0
0.24,69.5	0
0.24,69.6	0
0.24,69.7	0
0.24,69.8	0
0.24,69.9	0
0.24,70.0	0
0.24,70.1	0
0.24,70.2	0
0.24,70.3	0
0.24,70.4	0
0.24,70.5	0

0.24,70.6	0
0.24,70.7	0
0.24,70.8	0
0.24,70.9	0
0.24,71.0	0
0.24,71.1	0
0.24,71.2	0
0.24,71.3	0
0.24,71.4	0
0.24,71.5	0
0.24,71.6	0
0.24,71.7	0
0.24,71.8	0
0.24,71.9	0
0.24,72.0	0
0.24,72.1	0
0.24,72.2	0
0.24,72.3	0
0.24,72.4	0
0.24,72.5	0
0.24,72.6	0
0.24,72.7	0
0.24,72.8	0
0.24,72.9	0
0.24,73.0	0
0.24,73.1	0
0.24,73.2	0
0.24,73.3	0
0.24,73.4	0
0.24,73.5	0
0.24,73.6	0
0.24,73.7	0
0.24,73.8	0
0.24,73.9	0
0.24,74.0	0
0.24,74.1	0
0.24,74.2	0
0.24,74.3	0
0.24,74.4	0
0.24,74.5	0
0.24,74.6	0
0.24,74.7	0

0.24,74.8	0
0.24,74.9	0
0.24,75.0	0
0.24,75.1	0
0.24,75.2	0
0.24,75.3	0
0.24,75.4	0
0.24,75.5	0
0.24,75.6	0
0.24,75.7	0
0.24,75.8	0
0.24,75.9	0
0.24,76.0	0
0.24,76.1	0
0.24,76.2	0
0.24,76.3	0
0.24,76.4	0
0.24,76.5	0
0.24,76.6	0
0.24,76.7	0
0.24,76.8	0
0.24,76.9	0
0.24,77.0	0
0.24,77.1	0
0.24,77.2	0
0.24,77.3	0
0.24,77.4	0
0.24,77.5	0
0.24,77.6	0
0.24,77.7	0
0.24,77.8	0
0.24,77.9	0
0.24,78.0	0
0.24,78.1	0
0.24,78.2	0
0.24,78.3	0
0.24,78.4	0
0.24,78.5	0
0.24,78.6	0
0.24,78.7	0
0.24,78.8	0
0.24,78.9	0

0.24,79.0	0
0.24,79.1	0
0.24,79.2	0
0.24,79.3	0
0.24,79.4	0
0.24,79.5	0
0.24,79.6	0
0.24,79.7	0
0.24,79.8	0
0.24,79.9	0
0.24,80.0	0
0.24,80.1	0
0.24,80.2	0
0.24,80.3	0
0.24,80.4	0
0.24,80.5	0
0.24,80.6	0
0.24,80.7	0
0.24,80.8	0
0.24,80.9	0
0.24,81.0	0
0.24,81.1	0
0.24,81.2	0
0.24,81.3	0
0.24,81.4	0
0.24,81.5	0
0.24,81.6	0
0.24,81.7	0
0.24,81.8	0
0.24,81.9	0
0.24,82.0	0
0.24,82.1	0
0.24,82.2	0
0.24,82.3	0
0.24,82.4	0
0.24,82.5	0
0.24,82.6	0
0.24,82.7	0
0.24,82.8	0
0.24,82.9	0
0.24,83.0	0
0.24,83.1	0

0.24,83.2	0
0.24,83.3	0
0.24,83.4	0
0.24,83.5	0
0.24,83.6	0
0.24,83.7	0
0.24,83.8	0
0.24,83.9	0
0.24,84.0	0
0.24,84.1	0
0.24,84.2	0
0.24,84.3	0
0.24,84.4	0
0.24,84.5	0
0.24,84.6	0
0.24,84.7	0
0.24,84.8	0
0.24,84.9	0
0.24,85.0	0
0.24,85.1	0
0.24,85.2	0
0.24,85.3	0
0.24,85.4	0
0.24,85.5	0
0.24,85.6	0
0.24,85.7	0
0.24,85.8	0
0.24,85.9	0
0.24,86.0	0
0.24,86.1	0
0.24,86.2	0
0.24,86.3	0
0.24,86.4	0
0.24,86.5	0
0.24,86.6	0
0.24,86.7	0
0.24,86.8	0
0.24,86.9	0
0.24,87.0	0
0.24,87.1	0
0.24,87.2	0
0.24,87.3	0

0.24,87.4	0
0.24,87.5	0
0.24,87.6	0
0.24,87.7	0
0.24,87.8	0
0.24,87.9	0
0.24,88.0	0
0.24,88.1	0
0.24,88.2	0
0.24,88.3	0
0.24,88.4	0
0.24,88.5	0
0.24,88.6	0
0.24,88.7	0
0.24,88.8	0
0.24,88.9	0
0.24,89.0	0
0.24,89.1	0
0.24,89.2	0
0.24,89.3	0
0.24,89.4	0
0.24,89.5	0
0.24,89.6	0
0.24,89.7	0
0.24,89.8	0
0.24,89.9	0
0.24,90.0	0
0.24,90.1	0
0.24,90.2	0
0.24,90.3	0
0.24,90.4	0
0.24,90.5	0
0.24,90.6	0
0.24,90.7	0
0.24,90.8	0
0.24,90.9	0
0.24,91.0	0
0.24,91.1	0
0.24,91.2	0
0.24,91.3	0
0.24,91.4	0
0.24,91.5	0

0.24,91.6	0
0.24,91.7	0
0.24,91.8	0
0.24,91.9	0
0.24,92.0	0
0.24,92.1	0
0.24,92.2	0
0.24,92.3	0
0.24,92.4	0
0.24,92.5	0
0.24,92.6	0
0.24,92.7	0
0.24,92.8	0
0.24,92.9	0
0.24,93.0	0
0.24,93.1	0
0.24,93.2	0
0.24,93.3	0
0.24,93.4	0
0.24,93.5	0
0.24,93.6	0
0.24,93.7	0
0.24,93.8	0
0.24,93.9	0
0.24,94.0	0
0.24,94.1	0
0.24,94.2	0
0.24,94.3	0
0.24,94.4	0
0.24,94.5	0
0.24,94.6	0
0.24,94.7	0
0.24,94.8	0
0.24,94.9	0
0.24,95.0	0
0.24,95.1	0
0.24,95.2	0
0.24,95.3	0
0.24,95.4	0
0.24,95.5	0
0.24,95.6	0
0.24,95.7	0

0.24,95.8	0
0.24,95.9	0
0.24,96.0	0
0.24,96.1	0
0.24,96.2	0
0.24,96.3	0
0.24,96.4	0
0.24,96.5	0
0.24,96.6	0
0.24,96.7	0
0.24,96.8	0
0.24,96.9	0
0.24,97.0	0
0.24,97.1	0
0.24,97.2	0
0.24,97.3	0
0.24,97.4	0
0.24,97.5	0
0.24,97.6	0
0.24,97.7	0
0.24,97.8	0
0.24,97.9	0
0.24,98.0	0
0.24,98.1	0
0.24,98.2	0
0.24,98.3	0
0.24,98.4	0
0.24,98.5	0
0.24,98.6	0
0.24,98.7	0
0.24,98.8	0
0.24,98.9	0
0.24,99.0	0
0.24,99.1	0
0.24,99.2	0
0.24,99.3	0
0.24,99.4	0
0.24,99.5	0
0.24,99.6	0
0.24,99.7	0
0.24,99.8	0
0.24,99.9	0

0.24,100.0	0
0.25,0.0	0
0.25,0.1	0
0.25,0.2	0
0.25,0.3	0
0.25,0.4	0
0.25,0.5	0
0.25,0.6	0
0.25,0.7	0
0.25,0.8	0
0.25,0.9	0
0.25,1.0	0
0.25,1.1	0
0.25,1.2	0
0.25,1.3	0
0.25,1.4	0
0.25,1.5	0
0.25,1.6	0
0.25,1.7	0
0.25,1.8	0
0.25,1.9	0
0.25,2.0	0
0.25,2.1	0
0.25,2.2	0
0.25,2.3	0
0.25,2.4	0
0.25,2.5	0
0.25,2.6	0
0.25,2.7	0
0.25,2.8	0
0.25,2.9	0
0.25,3.0	0
0.25,3.1	0
0.25,3.2	0
0.25,3.3	0
0.25,3.4	0
0.25,3.5	0
0.25,3.6	0
0.25,3.7	0
0.25,3.8	0
0.25,3.9	0
0.25,4.0	0

0.25,4.1	0
0.25,4.2	0
0.25,4.3	0
0.25,4.4	0
0.25,4.5	0
0.25,4.6	0
0.25,4.7	0
0.25,4.8	0
0.25,4.9	0
0.25,5.0	0
0.25,5.1	0
0.25,5.2	0
0.25,5.3	0
0.25,5.4	0
0.25,5.5	0
0.25,5.6	0
0.25,5.7	0
0.25,5.8	0
0.25,5.9	0
0.25,6.0	0
0.25,6.1	0
0.25,6.2	0
0.25,6.3	0
0.25,6.4	0
0.25,6.5	0
0.25,6.6	0
0.25,6.7	0
0.25,6.8	0
0.25,6.9	0
0.25,7.0	0
0.25,7.1	0
0.25,7.2	0
0.25,7.3	0
0.25,7.4	0
0.25,7.5	0
0.25,7.6	0
0.25,7.7	0
0.25,7.8	0
0.25,7.9	0
0.25,8.0	0
0.25,8.1	0
0.25,8.2	0

0.25,8.3	0
0.25,8.4	0
0.25,8.5	0
0.25,8.6	0
0.25,8.7	0
0.25,8.8	0
0.25,8.9	0
0.25,9.0	0
0.25,9.1	0
0.25,9.2	0
0.25,9.3	0
0.25,9.4	0
0.25,9.5	0
0.25,9.6	0
0.25,9.7	0
0.25,9.8	0
0.25,9.9	0
0.25,10.0	0
0.25,10.1	0
0.25,10.2	0
0.25,10.3	0
0.25,10.4	0
0.25,10.5	0
0.25,10.6	0
0.25,10.7	0
0.25,10.8	0
0.25,10.9	0
0.25,11.0	0
0.25,11.1	0
0.25,11.2	0
0.25,11.3	0
0.25,11.4	0
0.25,11.5	0
0.25,11.6	0
0.25,11.7	0
0.25,11.8	0
0.25,11.9	0
0.25,12.0	0
0.25,12.1	0
0.25,12.2	0
0.25,12.3	0
0.25,12.4	0

0.25,12.5	0
0.25,12.6	0
0.25,12.7	0
0.25,12.8	0
0.25,12.9	0
0.25,13.0	0
0.25,13.1	0
0.25,13.2	0
0.25,13.3	0
0.25,13.4	0
0.25,13.5	0
0.25,13.6	0
0.25,13.7	0
0.25,13.8	0
0.25,13.9	0
0.25,14.0	0
0.25,14.1	0
0.25,14.2	0
0.25,14.3	0
0.25,14.4	0
0.25,14.5	0
0.25,14.6	0
0.25,14.7	0
0.25,14.8	0
0.25,14.9	0
0.25,15.0	0
0.25,15.1	0
0.25,15.2	0
0.25,15.3	0
0.25,15.4	0
0.25,15.5	0
0.25,15.6	0
0.25,15.7	0
0.25,15.8	0
0.25,15.9	0
0.25,16.0	0
0.25,16.1	0
0.25,16.2	0
0.25,16.3	0
0.25,16.4	0
0.25,16.5	0
0.25,16.6	0

0.25,16.7	0
0.25,16.8	0
0.25,16.9	0
0.25,17.0	0
0.25,17.1	0
0.25,17.2	0
0.25,17.3	0
0.25,17.4	0
0.25,17.5	0
0.25,17.6	0
0.25,17.7	0
0.25,17.8	0
0.25,17.9	0
0.25,18.0	0
0.25,18.1	0
0.25,18.2	0
0.25,18.3	0
0.25,18.4	0
0.25,18.5	0
0.25,18.6	0
0.25,18.7	0
0.25,18.8	0
0.25,18.9	0
0.25,19.0	0
0.25,19.1	0
0.25,19.2	0
0.25,19.3	0
0.25,19.4	0
0.25,19.5	0
0.25,19.6	0
0.25,19.7	0
0.25,19.8	0
0.25,19.9	0
0.25,20.0	0
0.25,20.1	0
0.25,20.2	0
0.25,20.3	0
0.25,20.4	0
0.25,20.5	0
0.25,20.6	0
0.25,20.7	0
0.25,20.8	0

0.25,20.9	0
0.25,21.0	0
0.25,21.1	0
0.25,21.2	0
0.25,21.3	0
0.25,21.4	0
0.25,21.5	0
0.25,21.6	0
0.25,21.7	0
0.25,21.8	0
0.25,21.9	0
0.25,22.0	0
0.25,22.1	0
0.25,22.2	0
0.25,22.3	0
0.25,22.4	0
0.25,22.5	0
0.25,22.6	0
0.25,22.7	0
0.25,22.8	0
0.25,22.9	0
0.25,23.0	0
0.25,23.1	0
0.25,23.2	0
0.25,23.3	0
0.25,23.4	0
0.25,23.5	0
0.25,23.6	0
0.25,23.7	0
0.25,23.8	0
0.25,23.9	0
0.25,24.0	0
0.25,24.1	0
0.25,24.2	0
0.25,24.3	0
0.25,24.4	0
0.25,24.5	0
0.25,24.6	0
0.25,24.7	0
0.25,24.8	0
0.25,24.9	0
0.25,25.0	0

0.25,25.1	0
0.25,25.2	0
0.25,25.3	0
0.25,25.4	0
0.25,25.5	0
0.25,25.6	0
0.25,25.7	0
0.25,25.8	0
0.25,25.9	0
0.25,26.0	0
0.25,26.1	0
0.25,26.2	0
0.25,26.3	0
0.25,26.4	0
0.25,26.5	0
0.25,26.6	0
0.25,26.7	0
0.25,26.8	0
0.25,26.9	0
0.25,27.0	0
0.25,27.1	0
0.25,27.2	0
0.25,27.3	0
0.25,27.4	0
0.25,27.5	0
0.25,27.6	0
0.25,27.7	0
0.25,27.8	0
0.25,27.9	0
0.25,28.0	0
0.25,28.1	0
0.25,28.2	0
0.25,28.3	0
0.25,28.4	0
0.25,28.5	0
0.25,28.6	0
0.25,28.7	0
0.25,28.8	0
0.25,28.9	0
0.25,29.0	0
0.25,29.1	0
0.25,29.2	0

0.25,29.3	0
0.25,29.4	0
0.25,29.5	0
0.25,29.6	0
0.25,29.7	0
0.25,29.8	0
0.25,29.9	0
0.25,30.0	0
0.25,30.1	0
0.25,30.2	0
0.25,30.3	0
0.25,30.4	0
0.25,30.5	0
0.25,30.6	0
0.25,30.7	0
0.25,30.8	0
0.25,30.9	0
0.25,31.0	0
0.25,31.1	0
0.25,31.2	0
0.25,31.3	0
0.25,31.4	0
0.25,31.5	0
0.25,31.6	0
0.25,31.7	0
0.25,31.8	0
0.25,31.9	0
0.25,32.0	0
0.25,32.1	0
0.25,32.2	0
0.25,32.3	0
0.25,32.4	0
0.25,32.5	0
0.25,32.6	0
0.25,32.7	0
0.25,32.8	0
0.25,32.9	0
0.25,33.0	0
0.25,33.1	0
0.25,33.2	0
0.25,33.3	0
0.25,33.4	0

0.25,33.5	0
0.25,33.6	0
0.25,33.7	0
0.25,33.8	0
0.25,33.9	0
0.25,34.0	0
0.25,34.1	0
0.25,34.2	0
0.25,34.3	0
0.25,34.4	0
0.25,34.5	0
0.25,34.6	0
0.25,34.7	0
0.25,34.8	0
0.25,34.9	0
0.25,35.0	0
0.25,35.1	0
0.25,35.2	0
0.25,35.3	0
0.25,35.4	0
0.25,35.5	0
0.25,35.6	0
0.25,35.7	0
0.25,35.8	0
0.25,35.9	0
0.25,36.0	0
0.25,36.1	0
0.25,36.2	0
0.25,36.3	0
0.25,36.4	0
0.25,36.5	0
0.25,36.6	0
0.25,36.7	0
0.25,36.8	0
0.25,36.9	0
0.25,37.0	0
0.25,37.1	0
0.25,37.2	0
0.25,37.3	0
0.25,37.4	0
0.25,37.5	0
0.25,37.6	0

0.25,37.7	0
0.25,37.8	0
0.25,37.9	0
0.25,38.0	0
0.25,38.1	0
0.25,38.2	0
0.25,38.3	0
0.25,38.4	0
0.25,38.5	0
0.25,38.6	0
0.25,38.7	0
0.25,38.8	0
0.25,38.9	0
0.25,39.0	0
0.25,39.1	0
0.25,39.2	0
0.25,39.3	0
0.25,39.4	0
0.25,39.5	0
0.25,39.6	0
0.25,39.7	0
0.25,39.8	0
0.25,39.9	0
0.25,40.0	0
0.25,40.1	0
0.25,40.2	0
0.25,40.3	0
0.25,40.4	0
0.25,40.5	0
0.25,40.6	0
0.25,40.7	0
0.25,40.8	0
0.25,40.9	0
0.25,41.0	0
0.25,41.1	0
0.25,41.2	0
0.25,41.3	0
0.25,41.4	0
0.25,41.5	0
0.25,41.6	0
0.25,41.7	0
0.25,41.8	0

0.25,41.9	0
0.25,42.0	0
0.25,42.1	0
0.25,42.2	0
0.25,42.3	0
0.25,42.4	0
0.25,42.5	0
0.25,42.6	0
0.25,42.7	0
0.25,42.8	0
0.25,42.9	0
0.25,43.0	0
0.25,43.1	0
0.25,43.2	0
0.25,43.3	0
0.25,43.4	0
0.25,43.5	0
0.25,43.6	0
0.25,43.7	0
0.25,43.8	0
0.25,43.9	0
0.25,44.0	0
0.25,44.1	0
0.25,44.2	0
0.25,44.3	0
0.25,44.4	0
0.25,44.5	0
0.25,44.6	0
0.25,44.7	0
0.25,44.8	0
0.25,44.9	0
0.25,45.0	0
0.25,45.1	0
0.25,45.2	0
0.25,45.3	0
0.25,45.4	0
0.25,45.5	0
0.25,45.6	0
0.25,45.7	0
0.25,45.8	0
0.25,45.9	0
0.25,46.0	0

0.25,46.1	0
0.25,46.2	0
0.25,46.3	0
0.25,46.4	0
0.25,46.5	0
0.25,46.6	0
0.25,46.7	0
0.25,46.8	0
0.25,46.9	0
0.25,47.0	0
0.25,47.1	0
0.25,47.2	0
0.25,47.3	0
0.25,47.4	0
0.25,47.5	0
0.25,47.6	0
0.25,47.7	0
0.25,47.8	0
0.25,47.9	0
0.25,48.0	0
0.25,48.1	0
0.25,48.2	0
0.25,48.3	0
0.25,48.4	0
0.25,48.5	0
0.25,48.6	0
0.25,48.7	0
0.25,48.8	0
0.25,48.9	0
0.25,49.0	0
0.25,49.1	0
0.25,49.2	0
0.25,49.3	0
0.25,49.4	0
0.25,49.5	0
0.25,49.6	0
0.25,49.7	0
0.25,49.8	0
0.25,49.9	0
0.25,50.0	0
0.25,50.1	0
0.25,50.2	0

0.25,50.3	0
0.25,50.4	0
0.25,50.5	0
0.25,50.6	0
0.25,50.7	0
0.25,50.8	0
0.25,50.9	0
0.25,51.0	0
0.25,51.1	0
0.25,51.2	0
0.25,51.3	0
0.25,51.4	0
0.25,51.5	0
0.25,51.6	0
0.25,51.7	0
0.25,51.8	0
0.25,51.9	0
0.25,52.0	0
0.25,52.1	0
0.25,52.2	0
0.25,52.3	0
0.25,52.4	0
0.25,52.5	0
0.25,52.6	0
0.25,52.7	0
0.25,52.8	0
0.25,52.9	0
0.25,53.0	0
0.25,53.1	0
0.25,53.2	0
0.25,53.3	0
0.25,53.4	0
0.25,53.5	0
0.25,53.6	0
0.25,53.7	0
0.25,53.8	0
0.25,53.9	0
0.25,54.0	0
0.25,54.1	0
0.25,54.2	0
0.25,54.3	0
0.25,54.4	0

0.25,54.5	0
0.25,54.6	0
0.25,54.7	0
0.25,54.8	0
0.25,54.9	0
0.25,55.0	0
0.25,55.1	0
0.25,55.2	0
0.25,55.3	0
0.25,55.4	0
0.25,55.5	0
0.25,55.6	0
0.25,55.7	0
0.25,55.8	0
0.25,55.9	0
0.25,56.0	0
0.25,56.1	0
0.25,56.2	0
0.25,56.3	0
0.25,56.4	0
0.25,56.5	0
0.25,56.6	0
0.25,56.7	0
0.25,56.8	0
0.25,56.9	0
0.25,57.0	0
0.25,57.1	0
0.25,57.2	0
0.25,57.3	0
0.25,57.4	0
0.25,57.5	0
0.25,57.6	0
0.25,57.7	0
0.25,57.8	0
0.25,57.9	0
0.25,58.0	0
0.25,58.1	0
0.25,58.2	0
0.25,58.3	0
0.25,58.4	0
0.25,58.5	0
0.25,58.6	0

0.25,58.7	0
0.25,58.8	0
0.25,58.9	0
0.25,59.0	0
0.25,59.1	0
0.25,59.2	0
0.25,59.3	0
0.25,59.4	0
0.25,59.5	0
0.25,59.6	0
0.25,59.7	0
0.25,59.8	0
0.25,59.9	0
0.25,60.0	0
0.25,60.1	0
0.25,60.2	0
0.25,60.3	0
0.25,60.4	0
0.25,60.5	0
0.25,60.6	0
0.25,60.7	0
0.25,60.8	0
0.25,60.9	0
0.25,61.0	0
0.25,61.1	0
0.25,61.2	0
0.25,61.3	0
0.25,61.4	0
0.25,61.5	0
0.25,61.6	0
0.25,61.7	0
0.25,61.8	0
0.25,61.9	0
0.25,62.0	0
0.25,62.1	0
0.25,62.2	0
0.25,62.3	0
0.25,62.4	0
0.25,62.5	0
0.25,62.6	0
0.25,62.7	0
0.25,62.8	0

0.25,62.9	0
0.25,63.0	0
0.25,63.1	0
0.25,63.2	0
0.25,63.3	0
0.25,63.4	0
0.25,63.5	0
0.25,63.6	0
0.25,63.7	0
0.25,63.8	0
0.25,63.9	0
0.25,64.0	0
0.25,64.1	0
0.25,64.2	0
0.25,64.3	0
0.25,64.4	0
0.25,64.5	0
0.25,64.6	0
0.25,64.7	0
0.25,64.8	0
0.25,64.9	0
0.25,65.0	0
0.25,65.1	0
0.25,65.2	0
0.25,65.3	0
0.25,65.4	0
0.25,65.5	0
0.25,65.6	0
0.25,65.7	0
0.25,65.8	0
0.25,65.9	0
0.25,66.0	0
0.25,66.1	0
0.25,66.2	0
0.25,66.3	0
0.25,66.4	0
0.25,66.5	0
0.25,66.6	0
0.25,66.7	0
0.25,66.8	0
0.25,66.9	0
0.25,67.0	0

0.25,67.1	0
0.25,67.2	0
0.25,67.3	0
0.25,67.4	0
0.25,67.5	0
0.25,67.6	0
0.25,67.7	0
0.25,67.8	0
0.25,67.9	0
0.25,68.0	0
0.25,68.1	0
0.25,68.2	0
0.25,68.3	0
0.25,68.4	0
0.25,68.5	0
0.25,68.6	0
0.25,68.7	0
0.25,68.8	0
0.25,68.9	0
0.25,69.0	0
0.25,69.1	0
0.25,69.2	0
0.25,69.3	0
0.25,69.4	0
0.25,69.5	0
0.25,69.6	0
0.25,69.7	0
0.25,69.8	0
0.25,69.9	0
0.25,70.0	0
0.25,70.1	0
0.25,70.2	0
0.25,70.3	0
0.25,70.4	0
0.25,70.5	0
0.25,70.6	0
0.25,70.7	0
0.25,70.8	0
0.25,70.9	0
0.25,71.0	0
0.25,71.1	0
0.25,71.2	0

0.25,71.3	0
0.25,71.4	0
0.25,71.5	0
0.25,71.6	0
0.25,71.7	0
0.25,71.8	0
0.25,71.9	0
0.25,72.0	0
0.25,72.1	0
0.25,72.2	0
0.25,72.3	0
0.25,72.4	0
0.25,72.5	0
0.25,72.6	0
0.25,72.7	0
0.25,72.8	0
0.25,72.9	0
0.25,73.0	0
0.25,73.1	0
0.25,73.2	0
0.25,73.3	0
0.25,73.4	0
0.25,73.5	0
0.25,73.6	0
0.25,73.7	0
0.25,73.8	0
0.25,73.9	0
0.25,74.0	0
0.25,74.1	0
0.25,74.2	0
0.25,74.3	0
0.25,74.4	0
0.25,74.5	0
0.25,74.6	0
0.25,74.7	0
0.25,74.8	0
0.25,74.9	0
0.25,75.0	0
0.25,75.1	0
0.25,75.2	0
0.25,75.3	0
0.25,75.4	0

0.25,75.5	0
0.25,75.6	0
0.25,75.7	0
0.25,75.8	0
0.25,75.9	0
0.25,76.0	0
0.25,76.1	0
0.25,76.2	0
0.25,76.3	0
0.25,76.4	0
0.25,76.5	0
0.25,76.6	0
0.25,76.7	0
0.25,76.8	0
0.25,76.9	0
0.25,77.0	0
0.25,77.1	0
0.25,77.2	0
0.25,77.3	0
0.25,77.4	0
0.25,77.5	0
0.25,77.6	0
0.25,77.7	0
0.25,77.8	0
0.25,77.9	0
0.25,78.0	0
0.25,78.1	0
0.25,78.2	0
0.25,78.3	0
0.25,78.4	0
0.25,78.5	0
0.25,78.6	0
0.25,78.7	0
0.25,78.8	0
0.25,78.9	0
0.25,79.0	0
0.25,79.1	0
0.25,79.2	0
0.25,79.3	0
0.25,79.4	0
0.25,79.5	0
0.25,79.6	0

0.25,79.7	0
0.25,79.8	0
0.25,79.9	0
0.25,80.0	0
0.25,80.1	0
0.25,80.2	0
0.25,80.3	0
0.25,80.4	0
0.25,80.5	0
0.25,80.6	0
0.25,80.7	0
0.25,80.8	0
0.25,80.9	0
0.25,81.0	0
0.25,81.1	0
0.25,81.2	0
0.25,81.3	0
0.25,81.4	0
0.25,81.5	0
0.25,81.6	0
0.25,81.7	0
0.25,81.8	0
0.25,81.9	0
0.25,82.0	0
0.25,82.1	0
0.25,82.2	0
0.25,82.3	0
0.25,82.4	0
0.25,82.5	0
0.25,82.6	0
0.25,82.7	0
0.25,82.8	0
0.25,82.9	0
0.25,83.0	0
0.25,83.1	0
0.25,83.2	0
0.25,83.3	0
0.25,83.4	0
0.25,83.5	0
0.25,83.6	0
0.25,83.7	0
0.25,83.8	0

0.25,83.9	0
0.25,84.0	0
0.25,84.1	0
0.25,84.2	0
0.25,84.3	0
0.25,84.4	0
0.25,84.5	0
0.25,84.6	0
0.25,84.7	0
0.25,84.8	0
0.25,84.9	0
0.25,85.0	0
0.25,85.1	0
0.25,85.2	0
0.25,85.3	0
0.25,85.4	0
0.25,85.5	0
0.25,85.6	0
0.25,85.7	0
0.25,85.8	0
0.25,85.9	0
0.25,86.0	0
0.25,86.1	0
0.25,86.2	0
0.25,86.3	0
0.25,86.4	0
0.25,86.5	0
0.25,86.6	0
0.25,86.7	0
0.25,86.8	0
0.25,86.9	0
0.25,87.0	0
0.25,87.1	0
0.25,87.2	0
0.25,87.3	0
0.25,87.4	0
0.25,87.5	0
0.25,87.6	0
0.25,87.7	0
0.25,87.8	0
0.25,87.9	0
0.25,88.0	0

0.25,88.1	0
0.25,88.2	0
0.25,88.3	0
0.25,88.4	0
0.25,88.5	0
0.25,88.6	0
0.25,88.7	0
0.25,88.8	0
0.25,88.9	0
0.25,89.0	0
0.25,89.1	0
0.25,89.2	0
0.25,89.3	0
0.25,89.4	0
0.25,89.5	0
0.25,89.6	0
0.25,89.7	0
0.25,89.8	0
0.25,89.9	0
0.25,90.0	0
0.25,90.1	0
0.25,90.2	0
0.25,90.3	0
0.25,90.4	0
0.25,90.5	0
0.25,90.6	0
0.25,90.7	0
0.25,90.8	0
0.25,90.9	0
0.25,91.0	0
0.25,91.1	0
0.25,91.2	0
0.25,91.3	0
0.25,91.4	0
0.25,91.5	0
0.25,91.6	0
0.25,91.7	0
0.25,91.8	0
0.25,91.9	0
0.25,92.0	0
0.25,92.1	0
0.25,92.2	0

0.25,92.3	0
0.25,92.4	0
0.25,92.5	0
0.25,92.6	0
0.25,92.7	0
0.25,92.8	0
0.25,92.9	0
0.25,93.0	0
0.25,93.1	0
0.25,93.2	0
0.25,93.3	0
0.25,93.4	0
0.25,93.5	0
0.25,93.6	0
0.25,93.7	0
0.25,93.8	0
0.25,93.9	0
0.25,94.0	0
0.25,94.1	0
0.25,94.2	0
0.25,94.3	0
0.25,94.4	0
0.25,94.5	0
0.25,94.6	0
0.25,94.7	0
0.25,94.8	0
0.25,94.9	0
0.25,95.0	0
0.25,95.1	0
0.25,95.2	0
0.25,95.3	0
0.25,95.4	0
0.25,95.5	0
0.25,95.6	0
0.25,95.7	0
0.25,95.8	0
0.25,95.9	0
0.25,96.0	0
0.25,96.1	0
0.25,96.2	0
0.25,96.3	0
0.25,96.4	0

0.25,96.5	0
0.25,96.6	0
0.25,96.7	0
0.25,96.8	0
0.25,96.9	0
0.25,97.0	0
0.25,97.1	0
0.25,97.2	0
0.25,97.3	0
0.25,97.4	0
0.25,97.5	0
0.25,97.6	0
0.25,97.7	0
0.25,97.8	0
0.25,97.9	0
0.25,98.0	0
0.25,98.1	0
0.25,98.2	0
0.25,98.3	0
0.25,98.4	0
0.25,98.5	0
0.25,98.6	0
0.25,98.7	0
0.25,98.8	0
0.25,98.9	0
0.25,99.0	0
0.25,99.1	0
0.25,99.2	0
0.25,99.3	0
0.25,99.4	0
0.25,99.5	0
0.25,99.6	0
0.25,99.7	0
0.25,99.8	0
0.25,99.9	0
0.25,100.0	0
0.26,0.0	0
0.26,0.1	0
0.26,0.2	0
0.26,0.3	0
0.26,0.4	0
0.26,0.5	0

0.26,0.6	0
0.26,0.7	0
0.26,0.8	0
0.26,0.9	0
0.26,1.0	0
0.26,1.1	0
0.26,1.2	0
0.26,1.3	0
0.26,1.4	0
0.26,1.5	0
0.26,1.6	0
0.26,1.7	0
0.26,1.8	0
0.26,1.9	0
0.26,2.0	0
0.26,2.1	0
0.26,2.2	0
0.26,2.3	0
0.26,2.4	0
0.26,2.5	0
0.26,2.6	0
0.26,2.7	0
0.26,2.8	0
0.26,2.9	0
0.26,3.0	0
0.26,3.1	0
0.26,3.2	0
0.26,3.3	0
0.26,3.4	0
0.26,3.5	0
0.26,3.6	0
0.26,3.7	0
0.26,3.8	0
0.26,3.9	0
0.26,4.0	0
0.26,4.1	0
0.26,4.2	0
0.26,4.3	0
0.26,4.4	0
0.26,4.5	0
0.26,4.6	0
0.26,4.7	0

0.26,4.8	0
0.26,4.9	0
0.26,5.0	0
0.26,5.1	0
0.26,5.2	0
0.26,5.3	0
0.26,5.4	0
0.26,5.5	0
0.26,5.6	0
0.26,5.7	0
0.26,5.8	0
0.26,5.9	0
0.26,6.0	0
0.26,6.1	0
0.26,6.2	0
0.26,6.3	0
0.26,6.4	0
0.26,6.5	0
0.26,6.6	0
0.26,6.7	0
0.26,6.8	0
0.26,6.9	0
0.26,7.0	0
0.26,7.1	0
0.26,7.2	0
0.26,7.3	0
0.26,7.4	0
0.26,7.5	0
0.26,7.6	0
0.26,7.7	0
0.26,7.8	0
0.26,7.9	0
0.26,8.0	0
0.26,8.1	0
0.26,8.2	0
0.26,8.3	0
0.26,8.4	0
0.26,8.5	0
0.26,8.6	0
0.26,8.7	0
0.26,8.8	0
0.26,8.9	0

0.26,9.0	0
0.26,9.1	0
0.26,9.2	0
0.26,9.3	0
0.26,9.4	0
0.26,9.5	0
0.26,9.6	0
0.26,9.7	0
0.26,9.8	0
0.26,9.9	0
0.26,10.0	0
0.26,10.1	0
0.26,10.2	0
0.26,10.3	0
0.26,10.4	0
0.26,10.5	0
0.26,10.6	0
0.26,10.7	0
0.26,10.8	0
0.26,10.9	0
0.26,11.0	0
0.26,11.1	0
0.26,11.2	0
0.26,11.3	0
0.26,11.4	0
0.26,11.5	0
0.26,11.6	0
0.26,11.7	0
0.26,11.8	0
0.26,11.9	0
0.26,12.0	0
0.26,12.1	0
0.26,12.2	0
0.26,12.3	0
0.26,12.4	0
0.26,12.5	0
0.26,12.6	0
0.26,12.7	0
0.26,12.8	0
0.26,12.9	0
0.26,13.0	0
0.26,13.1	0

0.26,13.2	0
0.26,13.3	0
0.26,13.4	0
0.26,13.5	0
0.26,13.6	0
0.26,13.7	0
0.26,13.8	0
0.26,13.9	0
0.26,14.0	0
0.26,14.1	0
0.26,14.2	0
0.26,14.3	0
0.26,14.4	0
0.26,14.5	0
0.26,14.6	0
0.26,14.7	0
0.26,14.8	0
0.26,14.9	0
0.26,15.0	0
0.26,15.1	0
0.26,15.2	0
0.26,15.3	0
0.26,15.4	0
0.26,15.5	0
0.26,15.6	0
0.26,15.7	0
0.26,15.8	0
0.26,15.9	0
0.26,16.0	0
0.26,16.1	0
0.26,16.2	0
0.26,16.3	0
0.26,16.4	0
0.26,16.5	0
0.26,16.6	0
0.26,16.7	0
0.26,16.8	0
0.26,16.9	0
0.26,17.0	0
0.26,17.1	0
0.26,17.2	0
0.26,17.3	0

0.26,17.4	0
0.26,17.5	0
0.26,17.6	0
0.26,17.7	0
0.26,17.8	0
0.26,17.9	0
0.26,18.0	0
0.26,18.1	0
0.26,18.2	0
0.26,18.3	0
0.26,18.4	0
0.26,18.5	0
0.26,18.6	0
0.26,18.7	0
0.26,18.8	0
0.26,18.9	0
0.26,19.0	0
0.26,19.1	0
0.26,19.2	0
0.26,19.3	0
0.26,19.4	0
0.26,19.5	0
0.26,19.6	0
0.26,19.7	0
0.26,19.8	0
0.26,19.9	0
0.26,20.0	0
0.26,20.1	0
0.26,20.2	0
0.26,20.3	0
0.26,20.4	0
0.26,20.5	0
0.26,20.6	0
0.26,20.7	0
0.26,20.8	0
0.26,20.9	0
0.26,21.0	0
0.26,21.1	0
0.26,21.2	0
0.26,21.3	0
0.26,21.4	0
0.26,21.5	0

0.26,21.6	0
0.26,21.7	0
0.26,21.8	0
0.26,21.9	0
0.26,22.0	0
0.26,22.1	0
0.26,22.2	0
0.26,22.3	0
0.26,22.4	0
0.26,22.5	0
0.26,22.6	0
0.26,22.7	0
0.26,22.8	0
0.26,22.9	0
0.26,23.0	0
0.26,23.1	0
0.26,23.2	0
0.26,23.3	0
0.26,23.4	0
0.26,23.5	0
0.26,23.6	0
0.26,23.7	0
0.26,23.8	0
0.26,23.9	0
0.26,24.0	0
0.26,24.1	0
0.26,24.2	0
0.26,24.3	0
0.26,24.4	0
0.26,24.5	0
0.26,24.6	0
0.26,24.7	0
0.26,24.8	0
0.26,24.9	0
0.26,25.0	0
0.26,25.1	0
0.26,25.2	0
0.26,25.3	0
0.26,25.4	0
0.26,25.5	0
0.26,25.6	0
0.26,25.7	0

0.26,25.8	0
0.26,25.9	0
0.26,26.0	0
0.26,26.1	0
0.26,26.2	0
0.26,26.3	0
0.26,26.4	0
0.26,26.5	0
0.26,26.6	0
0.26,26.7	0
0.26,26.8	0
0.26,26.9	0
0.26,27.0	0
0.26,27.1	0
0.26,27.2	0
0.26,27.3	0
0.26,27.4	0
0.26,27.5	0
0.26,27.6	0
0.26,27.7	0
0.26,27.8	0
0.26,27.9	0
0.26,28.0	0
0.26,28.1	0
0.26,28.2	0
0.26,28.3	0
0.26,28.4	0
0.26,28.5	0
0.26,28.6	0
0.26,28.7	0
0.26,28.8	0
0.26,28.9	0
0.26,29.0	0
0.26,29.1	0
0.26,29.2	0
0.26,29.3	0
0.26,29.4	0
0.26,29.5	0
0.26,29.6	0
0.26,29.7	0
0.26,29.8	0
0.26,29.9	0

0.26,30.0	0
0.26,30.1	0
0.26,30.2	0
0.26,30.3	0
0.26,30.4	0
0.26,30.5	0
0.26,30.6	0
0.26,30.7	0
0.26,30.8	0
0.26,30.9	0
0.26,31.0	0
0.26,31.1	0
0.26,31.2	0
0.26,31.3	0
0.26,31.4	0
0.26,31.5	0
0.26,31.6	0
0.26,31.7	0
0.26,31.8	0
0.26,31.9	0
0.26,32.0	0
0.26,32.1	0
0.26,32.2	0
0.26,32.3	0
0.26,32.4	0
0.26,32.5	0
0.26,32.6	0
0.26,32.7	0
0.26,32.8	0
0.26,32.9	0
0.26,33.0	0
0.26,33.1	0
0.26,33.2	0
0.26,33.3	0
0.26,33.4	0
0.26,33.5	0
0.26,33.6	0
0.26,33.7	0
0.26,33.8	0
0.26,33.9	0
0.26,34.0	0
0.26,34.1	0

0.26,34.2	0
0.26,34.3	0
0.26,34.4	0
0.26,34.5	0
0.26,34.6	0
0.26,34.7	0
0.26,34.8	0
0.26,34.9	0
0.26,35.0	0
0.26,35.1	0
0.26,35.2	0
0.26,35.3	0
0.26,35.4	0
0.26,35.5	0
0.26,35.6	0
0.26,35.7	0
0.26,35.8	0
0.26,35.9	0
0.26,36.0	0
0.26,36.1	0
0.26,36.2	0
0.26,36.3	0
0.26,36.4	0
0.26,36.5	0
0.26,36.6	0
0.26,36.7	0
0.26,36.8	0
0.26,36.9	0
0.26,37.0	0
0.26,37.1	0
0.26,37.2	0
0.26,37.3	0
0.26,37.4	0
0.26,37.5	0
0.26,37.6	0
0.26,37.7	0
0.26,37.8	0
0.26,37.9	0
0.26,38.0	0
0.26,38.1	0
0.26,38.2	0
0.26,38.3	0

0.26,38.4	0
0.26,38.5	0
0.26,38.6	0
0.26,38.7	0
0.26,38.8	0
0.26,38.9	0
0.26,39.0	0
0.26,39.1	0
0.26,39.2	0
0.26,39.3	0
0.26,39.4	0
0.26,39.5	0
0.26,39.6	0
0.26,39.7	0
0.26,39.8	0
0.26,39.9	0
0.26,40.0	0
0.26,40.1	0
0.26,40.2	0
0.26,40.3	0
0.26,40.4	0
0.26,40.5	0
0.26,40.6	0
0.26,40.7	0
0.26,40.8	0
0.26,40.9	0
0.26,41.0	0
0.26,41.1	0
0.26,41.2	0
0.26,41.3	0
0.26,41.4	0
0.26,41.5	0
0.26,41.6	0
0.26,41.7	0
0.26,41.8	0
0.26,41.9	0
0.26,42.0	0
0.26,42.1	0
0.26,42.2	0
0.26,42.3	0
0.26,42.4	0
0.26,42.5	0

0.26,42.6	0
0.26,42.7	0
0.26,42.8	0
0.26,42.9	0
0.26,43.0	0
0.26,43.1	0
0.26,43.2	0
0.26,43.3	0
0.26,43.4	0
0.26,43.5	0
0.26,43.6	0
0.26,43.7	0
0.26,43.8	0
0.26,43.9	0
0.26,44.0	0
0.26,44.1	0
0.26,44.2	0
0.26,44.3	0
0.26,44.4	0
0.26,44.5	0
0.26,44.6	0
0.26,44.7	0
0.26,44.8	0
0.26,44.9	0
0.26,45.0	0
0.26,45.1	0
0.26,45.2	0
0.26,45.3	0
0.26,45.4	0
0.26,45.5	0
0.26,45.6	0
0.26,45.7	0
0.26,45.8	0
0.26,45.9	0
0.26,46.0	0
0.26,46.1	0
0.26,46.2	0
0.26,46.3	0
0.26,46.4	0
0.26,46.5	0
0.26,46.6	0
0.26,46.7	0

0.26,46.8	0
0.26,46.9	0
0.26,47.0	0
0.26,47.1	0
0.26,47.2	0
0.26,47.3	0
0.26,47.4	0
0.26,47.5	0
0.26,47.6	0
0.26,47.7	0
0.26,47.8	0
0.26,47.9	0
0.26,48.0	0
0.26,48.1	0
0.26,48.2	0
0.26,48.3	0
0.26,48.4	0
0.26,48.5	0
0.26,48.6	0
0.26,48.7	0
0.26,48.8	0
0.26,48.9	0
0.26,49.0	0
0.26,49.1	0
0.26,49.2	0
0.26,49.3	0
0.26,49.4	0
0.26,49.5	0
0.26,49.6	0
0.26,49.7	0
0.26,49.8	0
0.26,49.9	0
0.26,50.0	0
0.26,50.1	0
0.26,50.2	0
0.26,50.3	0
0.26,50.4	0
0.26,50.5	0
0.26,50.6	0
0.26,50.7	0
0.26,50.8	0
0.26,50.9	0

0.26,51.0	0
0.26,51.1	0
0.26,51.2	0
0.26,51.3	0
0.26,51.4	0
0.26,51.5	0
0.26,51.6	0
0.26,51.7	0
0.26,51.8	0
0.26,51.9	0
0.26,52.0	0
0.26,52.1	0
0.26,52.2	0
0.26,52.3	0
0.26,52.4	0
0.26,52.5	0
0.26,52.6	0
0.26,52.7	0
0.26,52.8	0
0.26,52.9	0
0.26,53.0	0
0.26,53.1	0
0.26,53.2	0
0.26,53.3	0
0.26,53.4	0
0.26,53.5	0
0.26,53.6	0
0.26,53.7	0
0.26,53.8	0
0.26,53.9	0
0.26,54.0	0
0.26,54.1	0
0.26,54.2	0
0.26,54.3	0
0.26,54.4	0
0.26,54.5	0
0.26,54.6	0
0.26,54.7	0
0.26,54.8	0
0.26,54.9	0
0.26,55.0	0
0.26,55.1	0

0.26,55.2	0
0.26,55.3	0
0.26,55.4	0
0.26,55.5	0
0.26,55.6	0
0.26,55.7	0
0.26,55.8	0
0.26,55.9	0
0.26,56.0	0
0.26,56.1	0
0.26,56.2	0
0.26,56.3	0
0.26,56.4	0
0.26,56.5	0
0.26,56.6	0
0.26,56.7	0
0.26,56.8	0
0.26,56.9	0
0.26,57.0	0
0.26,57.1	0
0.26,57.2	0
0.26,57.3	0
0.26,57.4	0
0.26,57.5	0
0.26,57.6	0
0.26,57.7	0
0.26,57.8	0
0.26,57.9	0
0.26,58.0	0
0.26,58.1	0
0.26,58.2	0
0.26,58.3	0
0.26,58.4	0
0.26,58.5	0
0.26,58.6	0
0.26,58.7	0
0.26,58.8	0
0.26,58.9	0
0.26,59.0	0
0.26,59.1	0
0.26,59.2	0
0.26,59.3	0

0.26,59.4	0
0.26,59.5	0
0.26,59.6	0
0.26,59.7	0
0.26,59.8	0
0.26,59.9	0
0.26,60.0	0
0.26,60.1	0
0.26,60.2	0
0.26,60.3	0
0.26,60.4	0
0.26,60.5	0
0.26,60.6	0
0.26,60.7	0
0.26,60.8	0
0.26,60.9	0
0.26,61.0	0
0.26,61.1	0
0.26,61.2	0
0.26,61.3	0
0.26,61.4	0
0.26,61.5	0
0.26,61.6	0
0.26,61.7	0
0.26,61.8	0
0.26,61.9	0
0.26,62.0	0
0.26,62.1	0
0.26,62.2	0
0.26,62.3	0
0.26,62.4	0
0.26,62.5	0
0.26,62.6	0
0.26,62.7	0
0.26,62.8	0
0.26,62.9	0
0.26,63.0	0
0.26,63.1	0
0.26,63.2	0
0.26,63.3	0
0.26,63.4	0
0.26,63.5	0

0.26,63.6	0
0.26,63.7	0
0.26,63.8	0
0.26,63.9	0
0.26,64.0	0
0.26,64.1	0
0.26,64.2	0
0.26,64.3	0
0.26,64.4	0
0.26,64.5	0
0.26,64.6	0
0.26,64.7	0
0.26,64.8	0
0.26,64.9	0
0.26,65.0	0
0.26,65.1	0
0.26,65.2	0
0.26,65.3	0
0.26,65.4	0
0.26,65.5	0
0.26,65.6	0
0.26,65.7	0
0.26,65.8	0
0.26,65.9	0
0.26,66.0	0
0.26,66.1	0
0.26,66.2	0
0.26,66.3	0
0.26,66.4	0
0.26,66.5	0
0.26,66.6	0
0.26,66.7	0
0.26,66.8	0
0.26,66.9	0
0.26,67.0	0
0.26,67.1	0
0.26,67.2	0
0.26,67.3	0
0.26,67.4	0
0.26,67.5	0
0.26,67.6	0
0.26,67.7	0

0.26,67.8	0
0.26,67.9	0
0.26,68.0	0
0.26,68.1	0
0.26,68.2	0
0.26,68.3	0
0.26,68.4	0
0.26,68.5	0
0.26,68.6	0
0.26,68.7	0
0.26,68.8	0
0.26,68.9	0
0.26,69.0	0
0.26,69.1	0
0.26,69.2	0
0.26,69.3	0
0.26,69.4	0
0.26,69.5	0
0.26,69.6	0
0.26,69.7	0
0.26,69.8	0
0.26,69.9	0
0.26,70.0	0
0.26,70.1	0
0.26,70.2	0
0.26,70.3	0
0.26,70.4	0
0.26,70.5	0
0.26,70.6	0
0.26,70.7	0
0.26,70.8	0
0.26,70.9	0
0.26,71.0	0
0.26,71.1	0
0.26,71.2	0
0.26,71.3	0
0.26,71.4	0
0.26,71.5	0
0.26,71.6	0
0.26,71.7	0
0.26,71.8	0
0.26,71.9	0

0.26,72.0	0
0.26,72.1	0
0.26,72.2	0
0.26,72.3	0
0.26,72.4	0
0.26,72.5	0
0.26,72.6	0
0.26,72.7	0
0.26,72.8	0
0.26,72.9	0
0.26,73.0	0
0.26,73.1	0
0.26,73.2	0
0.26,73.3	0
0.26,73.4	0
0.26,73.5	0
0.26,73.6	0
0.26,73.7	0
0.26,73.8	0
0.26,73.9	0
0.26,74.0	0
0.26,74.1	0
0.26,74.2	0
0.26,74.3	0
0.26,74.4	0
0.26,74.5	0
0.26,74.6	0
0.26,74.7	0
0.26,74.8	0
0.26,74.9	0
0.26,75.0	0
0.26,75.1	0
0.26,75.2	0
0.26,75.3	0
0.26,75.4	0
0.26,75.5	0
0.26,75.6	0
0.26,75.7	0
0.26,75.8	0
0.26,75.9	0
0.26,76.0	0
0.26,76.1	0

0.26,76.2	0
0.26,76.3	0
0.26,76.4	0
0.26,76.5	0
0.26,76.6	0
0.26,76.7	0
0.26,76.8	0
0.26,76.9	0
0.26,77.0	0
0.26,77.1	0
0.26,77.2	0
0.26,77.3	0
0.26,77.4	0
0.26,77.5	0
0.26,77.6	0
0.26,77.7	0
0.26,77.8	0
0.26,77.9	0
0.26,78.0	0
0.26,78.1	0
0.26,78.2	0
0.26,78.3	0
0.26,78.4	0
0.26,78.5	0
0.26,78.6	0
0.26,78.7	0
0.26,78.8	0
0.26,78.9	0
0.26,79.0	0
0.26,79.1	0
0.26,79.2	0
0.26,79.3	0
0.26,79.4	0
0.26,79.5	0
0.26,79.6	0
0.26,79.7	0
0.26,79.8	0
0.26,79.9	0
0.26,80.0	0
0.26,80.1	0
0.26,80.2	0
0.26,80.3	0

0.26,80.4	0
0.26,80.5	0
0.26,80.6	0
0.26,80.7	0
0.26,80.8	0
0.26,80.9	0
0.26,81.0	0
0.26,81.1	0
0.26,81.2	0
0.26,81.3	0
0.26,81.4	0
0.26,81.5	0
0.26,81.6	0
0.26,81.7	0
0.26,81.8	0
0.26,81.9	0
0.26,82.0	0
0.26,82.1	0
0.26,82.2	0
0.26,82.3	0
0.26,82.4	0
0.26,82.5	0
0.26,82.6	0
0.26,82.7	0
0.26,82.8	0
0.26,82.9	0
0.26,83.0	0
0.26,83.1	0
0.26,83.2	0
0.26,83.3	0
0.26,83.4	0
0.26,83.5	0
0.26,83.6	0
0.26,83.7	0
0.26,83.8	0
0.26,83.9	0
0.26,84.0	0
0.26,84.1	0
0.26,84.2	0
0.26,84.3	0
0.26,84.4	0
0.26,84.5	0

0.26,84.6	0
0.26,84.7	0
0.26,84.8	0
0.26,84.9	0
0.26,85.0	0
0.26,85.1	0
0.26,85.2	0
0.26,85.3	0
0.26,85.4	0
0.26,85.5	0
0.26,85.6	0
0.26,85.7	0
0.26,85.8	0
0.26,85.9	0
0.26,86.0	0
0.26,86.1	0
0.26,86.2	0
0.26,86.3	0
0.26,86.4	0
0.26,86.5	0
0.26,86.6	0
0.26,86.7	0
0.26,86.8	0
0.26,86.9	0
0.26,87.0	0
0.26,87.1	0
0.26,87.2	0
0.26,87.3	0
0.26,87.4	0
0.26,87.5	0
0.26,87.6	0
0.26,87.7	0
0.26,87.8	0
0.26,87.9	0
0.26,88.0	0
0.26,88.1	0
0.26,88.2	0
0.26,88.3	0
0.26,88.4	0
0.26,88.5	0
0.26,88.6	0
0.26,88.7	0

0.26,88.8	0
0.26,88.9	0
0.26,89.0	0
0.26,89.1	0
0.26,89.2	0
0.26,89.3	0
0.26,89.4	0
0.26,89.5	0
0.26,89.6	0
0.26,89.7	0
0.26,89.8	0
0.26,89.9	0
0.26,90.0	0
0.26,90.1	0
0.26,90.2	0
0.26,90.3	0
0.26,90.4	0
0.26,90.5	0
0.26,90.6	0
0.26,90.7	0
0.26,90.8	0
0.26,90.9	0
0.26,91.0	0
0.26,91.1	0
0.26,91.2	0
0.26,91.3	0
0.26,91.4	0
0.26,91.5	0
0.26,91.6	0
0.26,91.7	0
0.26,91.8	0
0.26,91.9	0
0.26,92.0	0
0.26,92.1	0
0.26,92.2	0
0.26,92.3	0
0.26,92.4	0
0.26,92.5	0
0.26,92.6	0
0.26,92.7	0
0.26,92.8	0
0.26,92.9	0

0.26,93.0	0
0.26,93.1	0
0.26,93.2	0
0.26,93.3	0
0.26,93.4	0
0.26,93.5	0
0.26,93.6	0
0.26,93.7	0
0.26,93.8	0
0.26,93.9	0
0.26,94.0	0
0.26,94.1	0
0.26,94.2	0
0.26,94.3	0
0.26,94.4	0
0.26,94.5	0
0.26,94.6	0
0.26,94.7	0
0.26,94.8	0
0.26,94.9	0
0.26,95.0	0
0.26,95.1	0
0.26,95.2	0
0.26,95.3	0
0.26,95.4	0
0.26,95.5	0
0.26,95.6	0
0.26,95.7	0
0.26,95.8	0
0.26,95.9	0
0.26,96.0	0
0.26,96.1	0
0.26,96.2	0
0.26,96.3	0
0.26,96.4	0
0.26,96.5	0
0.26,96.6	0
0.26,96.7	0
0.26,96.8	0
0.26,96.9	0
0.26,97.0	0
0.26,97.1	0

0.26,97.2	0
0.26,97.3	0
0.26,97.4	0
0.26,97.5	0
0.26,97.6	0
0.26,97.7	0
0.26,97.8	0
0.26,97.9	0
0.26,98.0	0
0.26,98.1	0
0.26,98.2	0
0.26,98.3	0
0.26,98.4	0
0.26,98.5	0
0.26,98.6	0
0.26,98.7	0
0.26,98.8	0
0.26,98.9	0
0.26,99.0	0
0.26,99.1	0
0.26,99.2	0
0.26,99.3	0
0.26,99.4	0
0.26,99.5	0
0.26,99.6	0
0.26,99.7	0
0.26,99.8	0
0.26,99.9	0
0.26,100.0	0
0.27,0.0	0
0.27,0.1	0
0.27,0.2	0
0.27,0.3	0
0.27,0.4	0
0.27,0.5	0
0.27,0.6	0
0.27,0.7	0
0.27,0.8	0
0.27,0.9	0
0.27,1.0	0
0.27,1.1	0
0.27,1.2	0

0.27,1.3	0
0.27,1.4	0
0.27,1.5	0
0.27,1.6	0
0.27,1.7	0
0.27,1.8	0
0.27,1.9	0
0.27,2.0	0
0.27,2.1	0
0.27,2.2	0
0.27,2.3	0
0.27,2.4	0
0.27,2.5	0
0.27,2.6	0
0.27,2.7	0
0.27,2.8	0
0.27,2.9	0
0.27,3.0	0
0.27,3.1	0
0.27,3.2	0
0.27,3.3	0
0.27,3.4	0
0.27,3.5	0
0.27,3.6	0
0.27,3.7	0
0.27,3.8	0
0.27,3.9	0
0.27,4.0	0
0.27,4.1	0
0.27,4.2	0
0.27,4.3	0
0.27,4.4	0
0.27,4.5	0
0.27,4.6	0
0.27,4.7	0
0.27,4.8	0
0.27,4.9	0
0.27,5.0	0
0.27,5.1	0
0.27,5.2	0
0.27,5.3	0
0.27,5.4	0

0.27,5.5	0
0.27,5.6	0
0.27,5.7	0
0.27,5.8	0
0.27,5.9	0
0.27,6.0	0
0.27,6.1	0
0.27,6.2	0
0.27,6.3	0
0.27,6.4	0
0.27,6.5	0
0.27,6.6	0
0.27,6.7	0
0.27,6.8	0
0.27,6.9	0
0.27,7.0	0
0.27,7.1	0
0.27,7.2	0
0.27,7.3	0
0.27,7.4	0
0.27,7.5	0
0.27,7.6	0
0.27,7.7	0
0.27,7.8	0
0.27,7.9	0
0.27,8.0	0
0.27,8.1	0
0.27,8.2	0
0.27,8.3	0
0.27,8.4	0
0.27,8.5	0
0.27,8.6	0
0.27,8.7	0
0.27,8.8	0
0.27,8.9	0
0.27,9.0	0
0.27,9.1	0
0.27,9.2	0
0.27,9.3	0
0.27,9.4	0
0.27,9.5	0
0.27,9.6	0

0.27,9.7	0
0.27,9.8	0
0.27,9.9	0
0.27,10.0	0
0.27,10.1	0
0.27,10.2	0
0.27,10.3	0
0.27,10.4	0
0.27,10.5	0
0.27,10.6	0
0.27,10.7	0
0.27,10.8	0
0.27,10.9	0
0.27,11.0	0
0.27,11.1	0
0.27,11.2	0
0.27,11.3	0
0.27,11.4	0
0.27,11.5	0
0.27,11.6	0
0.27,11.7	0
0.27,11.8	0
0.27,11.9	0
0.27,12.0	0
0.27,12.1	0
0.27,12.2	0
0.27,12.3	0
0.27,12.4	0
0.27,12.5	0
0.27,12.6	0
0.27,12.7	0
0.27,12.8	0
0.27,12.9	0
0.27,13.0	0
0.27,13.1	0
0.27,13.2	0
0.27,13.3	0
0.27,13.4	0
0.27,13.5	0
0.27,13.6	0
0.27,13.7	0
0.27,13.8	0

0.27,13.9	0
0.27,14.0	0
0.27,14.1	0
0.27,14.2	0
0.27,14.3	0
0.27,14.4	0
0.27,14.5	0
0.27,14.6	0
0.27,14.7	0
0.27,14.8	0
0.27,14.9	0
0.27,15.0	0
0.27,15.1	0
0.27,15.2	0
0.27,15.3	0
0.27,15.4	0
0.27,15.5	0
0.27,15.6	0
0.27,15.7	0
0.27,15.8	0
0.27,15.9	0
0.27,16.0	0
0.27,16.1	0
0.27,16.2	0
0.27,16.3	0
0.27,16.4	0
0.27,16.5	0
0.27,16.6	0
0.27,16.7	0
0.27,16.8	0
0.27,16.9	0
0.27,17.0	0
0.27,17.1	0
0.27,17.2	0
0.27,17.3	0
0.27,17.4	0
0.27,17.5	0
0.27,17.6	0
0.27,17.7	0
0.27,17.8	0
0.27,17.9	0
0.27,18.0	0

0.27,18.1	0
0.27,18.2	0
0.27,18.3	0
0.27,18.4	0
0.27,18.5	0
0.27,18.6	0
0.27,18.7	0
0.27,18.8	0
0.27,18.9	0
0.27,19.0	0
0.27,19.1	0
0.27,19.2	0
0.27,19.3	0
0.27,19.4	0
0.27,19.5	0
0.27,19.6	0
0.27,19.7	0
0.27,19.8	0
0.27,19.9	0
0.27,20.0	0
0.27,20.1	0
0.27,20.2	0
0.27,20.3	0
0.27,20.4	0
0.27,20.5	0
0.27,20.6	0
0.27,20.7	0
0.27,20.8	0
0.27,20.9	0
0.27,21.0	0
0.27,21.1	0
0.27,21.2	0
0.27,21.3	0
0.27,21.4	0
0.27,21.5	0
0.27,21.6	0
0.27,21.7	0
0.27,21.8	0
0.27,21.9	0
0.27,22.0	0
0.27,22.1	0
0.27,22.2	0

0.27,22.3	0
0.27,22.4	0
0.27,22.5	0
0.27,22.6	0
0.27,22.7	0
0.27,22.8	0
0.27,22.9	0
0.27,23.0	0
0.27,23.1	0
0.27,23.2	0
0.27,23.3	0
0.27,23.4	0
0.27,23.5	0
0.27,23.6	0
0.27,23.7	0
0.27,23.8	0
0.27,23.9	0
0.27,24.0	0
0.27,24.1	0
0.27,24.2	0
0.27,24.3	0
0.27,24.4	0
0.27,24.5	0
0.27,24.6	0
0.27,24.7	0
0.27,24.8	0
0.27,24.9	0
0.27,25.0	0
0.27,25.1	0
0.27,25.2	0
0.27,25.3	0
0.27,25.4	0
0.27,25.5	0
0.27,25.6	0
0.27,25.7	0
0.27,25.8	0
0.27,25.9	0
0.27,26.0	0
0.27,26.1	0
0.27,26.2	0
0.27,26.3	0
0.27,26.4	0

0.27,26.5	0
0.27,26.6	0
0.27,26.7	0
0.27,26.8	0
0.27,26.9	0
0.27,27.0	0
0.27,27.1	0
0.27,27.2	0
0.27,27.3	0
0.27,27.4	0
0.27,27.5	0
0.27,27.6	0
0.27,27.7	0
0.27,27.8	0
0.27,27.9	0
0.27,28.0	0
0.27,28.1	0
0.27,28.2	0
0.27,28.3	0
0.27,28.4	0
0.27,28.5	0
0.27,28.6	0
0.27,28.7	0
0.27,28.8	0
0.27,28.9	0
0.27,29.0	0
0.27,29.1	0
0.27,29.2	0
0.27,29.3	0
0.27,29.4	0
0.27,29.5	0
0.27,29.6	0
0.27,29.7	0
0.27,29.8	0
0.27,29.9	0
0.27,30.0	0
0.27,30.1	0
0.27,30.2	0
0.27,30.3	0
0.27,30.4	0
0.27,30.5	0
0.27,30.6	0

0.27,30.7	0
0.27,30.8	0
0.27,30.9	0
0.27,31.0	0
0.27,31.1	0
0.27,31.2	0
0.27,31.3	0
0.27,31.4	0
0.27,31.5	0
0.27,31.6	0
0.27,31.7	0
0.27,31.8	0
0.27,31.9	0
0.27,32.0	0
0.27,32.1	0
0.27,32.2	0
0.27,32.3	0
0.27,32.4	0
0.27,32.5	0
0.27,32.6	0
0.27,32.7	0
0.27,32.8	0
0.27,32.9	0
0.27,33.0	0
0.27,33.1	0
0.27,33.2	0
0.27,33.3	0
0.27,33.4	0
0.27,33.5	0
0.27,33.6	0
0.27,33.7	0
0.27,33.8	0
0.27,33.9	0
0.27,34.0	0
0.27,34.1	0
0.27,34.2	0
0.27,34.3	0
0.27,34.4	0
0.27,34.5	0
0.27,34.6	0
0.27,34.7	0
0.27,34.8	0

0.27,34.9	0
0.27,35.0	0
0.27,35.1	0
0.27,35.2	0
0.27,35.3	0
0.27,35.4	0
0.27,35.5	0
0.27,35.6	0
0.27,35.7	0
0.27,35.8	0
0.27,35.9	0
0.27,36.0	0
0.27,36.1	0
0.27,36.2	0
0.27,36.3	0
0.27,36.4	0
0.27,36.5	0
0.27,36.6	0
0.27,36.7	0
0.27,36.8	0
0.27,36.9	0
0.27,37.0	0
0.27,37.1	0
0.27,37.2	0
0.27,37.3	0
0.27,37.4	0
0.27,37.5	0
0.27,37.6	0
0.27,37.7	0
0.27,37.8	0
0.27,37.9	0
0.27,38.0	0
0.27,38.1	0
0.27,38.2	0
0.27,38.3	0
0.27,38.4	0
0.27,38.5	0
0.27,38.6	0
0.27,38.7	0
0.27,38.8	0
0.27,38.9	0
0.27,39.0	0

0.27,39.1	0
0.27,39.2	0
0.27,39.3	0
0.27,39.4	0
0.27,39.5	0
0.27,39.6	0
0.27,39.7	0
0.27,39.8	0
0.27,39.9	0
0.27,40.0	0
0.27,40.1	0
0.27,40.2	0
0.27,40.3	0
0.27,40.4	0
0.27,40.5	0
0.27,40.6	0
0.27,40.7	0
0.27,40.8	0
0.27,40.9	0
0.27,41.0	0
0.27,41.1	0
0.27,41.2	0
0.27,41.3	0
0.27,41.4	0
0.27,41.5	0
0.27,41.6	0
0.27,41.7	0
0.27,41.8	0
0.27,41.9	0
0.27,42.0	0
0.27,42.1	0
0.27,42.2	0
0.27,42.3	0
0.27,42.4	0
0.27,42.5	0
0.27,42.6	0
0.27,42.7	0
0.27,42.8	0
0.27,42.9	0
0.27,43.0	0
0.27,43.1	0
0.27,43.2	0

0.27,43.3	0
0.27,43.4	0
0.27,43.5	0
0.27,43.6	0
0.27,43.7	0
0.27,43.8	0
0.27,43.9	0
0.27,44.0	0
0.27,44.1	0
0.27,44.2	0
0.27,44.3	0
0.27,44.4	0
0.27,44.5	0
0.27,44.6	0
0.27,44.7	0
0.27,44.8	0
0.27,44.9	0
0.27,45.0	0
0.27,45.1	0
0.27,45.2	0
0.27,45.3	0
0.27,45.4	0
0.27,45.5	0
0.27,45.6	0
0.27,45.7	0
0.27,45.8	0
0.27,45.9	0
0.27,46.0	0
0.27,46.1	0
0.27,46.2	0
0.27,46.3	0
0.27,46.4	0
0.27,46.5	0
0.27,46.6	0
0.27,46.7	0
0.27,46.8	0
0.27,46.9	0
0.27,47.0	0
0.27,47.1	0
0.27,47.2	0
0.27,47.3	0
0.27,47.4	0

0.27,47.5	0
0.27,47.6	0
0.27,47.7	0
0.27,47.8	0
0.27,47.9	0
0.27,48.0	0
0.27,48.1	0
0.27,48.2	0
0.27,48.3	0
0.27,48.4	0
0.27,48.5	0
0.27,48.6	0
0.27,48.7	0
0.27,48.8	0
0.27,48.9	0
0.27,49.0	0
0.27,49.1	0
0.27,49.2	0
0.27,49.3	0
0.27,49.4	0
0.27,49.5	0
0.27,49.6	0
0.27,49.7	0
0.27,49.8	0
0.27,49.9	0
0.27,50.0	0
0.27,50.1	0
0.27,50.2	0
0.27,50.3	0
0.27,50.4	0
0.27,50.5	0
0.27,50.6	0
0.27,50.7	0
0.27,50.8	0
0.27,50.9	0
0.27,51.0	0
0.27,51.1	0
0.27,51.2	0
0.27,51.3	0
0.27,51.4	0
0.27,51.5	0
0.27,51.6	0

0.27,51.7	0
0.27,51.8	0
0.27,51.9	0
0.27,52.0	0
0.27,52.1	0
0.27,52.2	0
0.27,52.3	0
0.27,52.4	0
0.27,52.5	0
0.27,52.6	0
0.27,52.7	0
0.27,52.8	0
0.27,52.9	0
0.27,53.0	0
0.27,53.1	0
0.27,53.2	0
0.27,53.3	0
0.27,53.4	0
0.27,53.5	0
0.27,53.6	0
0.27,53.7	0
0.27,53.8	0
0.27,53.9	0
0.27,54.0	0
0.27,54.1	0
0.27,54.2	0
0.27,54.3	0
0.27,54.4	0
0.27,54.5	0
0.27,54.6	0
0.27,54.7	0
0.27,54.8	0
0.27,54.9	0
0.27,55.0	0
0.27,55.1	0
0.27,55.2	0
0.27,55.3	0
0.27,55.4	0
0.27,55.5	0
0.27,55.6	0
0.27,55.7	0
0.27,55.8	0

0.27,55.9	0
0.27,56.0	0
0.27,56.1	0
0.27,56.2	0
0.27,56.3	0
0.27,56.4	0
0.27,56.5	0
0.27,56.6	0
0.27,56.7	0
0.27,56.8	0
0.27,56.9	0
0.27,57.0	0
0.27,57.1	0
0.27,57.2	0
0.27,57.3	0
0.27,57.4	0
0.27,57.5	0
0.27,57.6	0
0.27,57.7	0
0.27,57.8	0
0.27,57.9	0
0.27,58.0	0
0.27,58.1	0
0.27,58.2	0
0.27,58.3	0
0.27,58.4	0
0.27,58.5	0
0.27,58.6	0
0.27,58.7	0
0.27,58.8	0
0.27,58.9	0
0.27,59.0	0
0.27,59.1	0
0.27,59.2	0
0.27,59.3	0
0.27,59.4	0
0.27,59.5	0
0.27,59.6	0
0.27,59.7	0
0.27,59.8	0
0.27,59.9	0
0.27,60.0	0

0.27,60.1	0
0.27,60.2	0
0.27,60.3	0
0.27,60.4	0
0.27,60.5	0
0.27,60.6	0
0.27,60.7	0
0.27,60.8	0
0.27,60.9	0
0.27,61.0	0
0.27,61.1	0
0.27,61.2	0
0.27,61.3	0
0.27,61.4	0
0.27,61.5	0
0.27,61.6	0
0.27,61.7	0
0.27,61.8	0
0.27,61.9	0
0.27,62.0	0
0.27,62.1	0
0.27,62.2	0
0.27,62.3	0
0.27,62.4	0
0.27,62.5	0
0.27,62.6	0
0.27,62.7	0
0.27,62.8	0
0.27,62.9	0
0.27,63.0	0
0.27,63.1	0
0.27,63.2	0
0.27,63.3	0
0.27,63.4	0
0.27,63.5	0
0.27,63.6	0
0.27,63.7	0
0.27,63.8	0
0.27,63.9	0
0.27,64.0	0
0.27,64.1	0
0.27,64.2	0

0.27,64.3	0
0.27,64.4	0
0.27,64.5	0
0.27,64.6	0
0.27,64.7	0
0.27,64.8	0
0.27,64.9	0
0.27,65.0	0
0.27,65.1	0
0.27,65.2	0
0.27,65.3	0
0.27,65.4	0
0.27,65.5	0
0.27,65.6	0
0.27,65.7	0
0.27,65.8	0
0.27,65.9	0
0.27,66.0	0
0.27,66.1	0
0.27,66.2	0
0.27,66.3	0
0.27,66.4	0
0.27,66.5	0
0.27,66.6	0
0.27,66.7	0
0.27,66.8	0
0.27,66.9	0
0.27,67.0	0
0.27,67.1	0
0.27,67.2	0
0.27,67.3	0
0.27,67.4	0
0.27,67.5	0
0.27,67.6	0
0.27,67.7	0
0.27,67.8	0
0.27,67.9	0
0.27,68.0	0
0.27,68.1	0
0.27,68.2	0
0.27,68.3	0
0.27,68.4	0

0.27,68.5	0
0.27,68.6	0
0.27,68.7	0
0.27,68.8	0
0.27,68.9	0
0.27,69.0	0
0.27,69.1	0
0.27,69.2	0
0.27,69.3	0
0.27,69.4	0
0.27,69.5	0
0.27,69.6	0
0.27,69.7	0
0.27,69.8	0
0.27,69.9	0
0.27,70.0	0
0.27,70.1	0
0.27,70.2	0
0.27,70.3	0
0.27,70.4	0
0.27,70.5	0
0.27,70.6	0
0.27,70.7	0
0.27,70.8	0
0.27,70.9	0
0.27,71.0	0
0.27,71.1	0
0.27,71.2	0
0.27,71.3	0
0.27,71.4	0
0.27,71.5	0
0.27,71.6	0
0.27,71.7	0
0.27,71.8	0
0.27,71.9	0
0.27,72.0	0
0.27,72.1	0
0.27,72.2	0
0.27,72.3	0
0.27,72.4	0
0.27,72.5	0
0.27,72.6	0

0.27,72.7	0
0.27,72.8	0
0.27,72.9	0
0.27,73.0	0
0.27,73.1	0
0.27,73.2	0
0.27,73.3	0
0.27,73.4	0
0.27,73.5	0
0.27,73.6	0
0.27,73.7	0
0.27,73.8	0
0.27,73.9	0
0.27,74.0	0
0.27,74.1	0
0.27,74.2	0
0.27,74.3	0
0.27,74.4	0
0.27,74.5	0
0.27,74.6	0
0.27,74.7	0
0.27,74.8	0
0.27,74.9	0
0.27,75.0	0
0.27,75.1	0
0.27,75.2	0
0.27,75.3	0
0.27,75.4	0
0.27,75.5	0
0.27,75.6	0
0.27,75.7	0
0.27,75.8	0
0.27,75.9	0
0.27,76.0	0
0.27,76.1	0
0.27,76.2	0
0.27,76.3	0
0.27,76.4	0
0.27,76.5	0
0.27,76.6	0
0.27,76.7	0
0.27,76.8	0

0.27,76.9	0
0.27,77.0	0
0.27,77.1	0
0.27,77.2	0
0.27,77.3	0
0.27,77.4	0
0.27,77.5	0
0.27,77.6	0
0.27,77.7	0
0.27,77.8	0
0.27,77.9	0
0.27,78.0	0
0.27,78.1	0
0.27,78.2	0
0.27,78.3	0
0.27,78.4	0
0.27,78.5	0
0.27,78.6	0
0.27,78.7	0
0.27,78.8	0
0.27,78.9	0
0.27,79.0	0
0.27,79.1	0
0.27,79.2	0
0.27,79.3	0
0.27,79.4	0
0.27,79.5	0
0.27,79.6	0
0.27,79.7	0
0.27,79.8	0
0.27,79.9	0
0.27,80.0	0
0.27,80.1	0
0.27,80.2	0
0.27,80.3	0
0.27,80.4	0
0.27,80.5	0
0.27,80.6	0
0.27,80.7	0
0.27,80.8	0
0.27,80.9	0
0.27,81.0	0

0.27,81.1	0
0.27,81.2	0
0.27,81.3	0
0.27,81.4	0
0.27,81.5	0
0.27,81.6	0
0.27,81.7	0
0.27,81.8	0
0.27,81.9	0
0.27,82.0	0
0.27,82.1	0
0.27,82.2	0
0.27,82.3	0
0.27,82.4	0
0.27,82.5	0
0.27,82.6	0
0.27,82.7	0
0.27,82.8	0
0.27,82.9	0
0.27,83.0	0
0.27,83.1	0
0.27,83.2	0
0.27,83.3	0
0.27,83.4	0
0.27,83.5	0
0.27,83.6	0
0.27,83.7	0
0.27,83.8	0
0.27,83.9	0
0.27,84.0	0
0.27,84.1	0
0.27,84.2	0
0.27,84.3	0
0.27,84.4	0
0.27,84.5	0
0.27,84.6	0
0.27,84.7	0
0.27,84.8	0
0.27,84.9	0
0.27,85.0	0
0.27,85.1	0
0.27,85.2	0

0.27,85.3	0
0.27,85.4	0
0.27,85.5	0
0.27,85.6	0
0.27,85.7	0
0.27,85.8	0
0.27,85.9	0
0.27,86.0	0
0.27,86.1	0
0.27,86.2	0
0.27,86.3	0
0.27,86.4	0
0.27,86.5	0
0.27,86.6	0
0.27,86.7	0
0.27,86.8	0
0.27,86.9	0
0.27,87.0	0
0.27,87.1	0
0.27,87.2	0
0.27,87.3	0
0.27,87.4	0
0.27,87.5	0
0.27,87.6	0
0.27,87.7	0
0.27,87.8	0
0.27,87.9	0
0.27,88.0	0
0.27,88.1	0
0.27,88.2	0
0.27,88.3	0
0.27,88.4	0
0.27,88.5	0
0.27,88.6	0
0.27,88.7	0
0.27,88.8	0
0.27,88.9	0
0.27,89.0	0
0.27,89.1	0
0.27,89.2	0
0.27,89.3	0
0.27,89.4	0

0.27,89.5	0
0.27,89.6	0
0.27,89.7	0
0.27,89.8	0
0.27,89.9	0
0.27,90.0	0
0.27,90.1	0
0.27,90.2	0
0.27,90.3	0
0.27,90.4	0
0.27,90.5	0
0.27,90.6	0
0.27,90.7	0
0.27,90.8	0
0.27,90.9	0
0.27,91.0	0
0.27,91.1	0
0.27,91.2	0
0.27,91.3	0
0.27,91.4	0
0.27,91.5	0
0.27,91.6	0
0.27,91.7	0
0.27,91.8	0
0.27,91.9	0
0.27,92.0	0
0.27,92.1	0
0.27,92.2	0
0.27,92.3	0
0.27,92.4	0
0.27,92.5	0
0.27,92.6	0
0.27,92.7	0
0.27,92.8	0
0.27,92.9	0
0.27,93.0	0
0.27,93.1	0
0.27,93.2	0
0.27,93.3	0
0.27,93.4	0
0.27,93.5	0
0.27,93.6	0

0.27,93.7	0
0.27,93.8	0
0.27,93.9	0
0.27,94.0	0
0.27,94.1	0
0.27,94.2	0
0.27,94.3	0
0.27,94.4	0
0.27,94.5	0
0.27,94.6	0
0.27,94.7	0
0.27,94.8	0
0.27,94.9	0
0.27,95.0	0
0.27,95.1	0
0.27,95.2	0
0.27,95.3	0
0.27,95.4	0
0.27,95.5	0
0.27,95.6	0
0.27,95.7	0
0.27,95.8	0
0.27,95.9	0
0.27,96.0	0
0.27,96.1	0
0.27,96.2	0
0.27,96.3	0
0.27,96.4	0
0.27,96.5	0
0.27,96.6	0
0.27,96.7	0
0.27,96.8	0
0.27,96.9	0
0.27,97.0	0
0.27,97.1	0
0.27,97.2	0
0.27,97.3	0
0.27,97.4	0
0.27,97.5	0
0.27,97.6	0
0.27,97.7	0
0.27,97.8	0

0.27,97.9	0
0.27,98.0	0
0.27,98.1	0
0.27,98.2	0
0.27,98.3	0
0.27,98.4	0
0.27,98.5	0
0.27,98.6	0
0.27,98.7	0
0.27,98.8	0
0.27,98.9	0
0.27,99.0	0
0.27,99.1	0
0.27,99.2	0
0.27,99.3	0
0.27,99.4	0
0.27,99.5	0
0.27,99.6	0
0.27,99.7	0
0.27,99.8	0
0.27,99.9	0
0.27,100.0	0
0.28,0.0	0
0.28,0.1	0
0.28,0.2	0
0.28,0.3	0
0.28,0.4	0
0.28,0.5	0
0.28,0.6	0
0.28,0.7	0
0.28,0.8	0
0.28,0.9	0
0.28,1.0	0
0.28,1.1	0
0.28,1.2	0
0.28,1.3	0
0.28,1.4	0
0.28,1.5	0
0.28,1.6	0
0.28,1.7	0
0.28,1.8	0
0.28,1.9	0

0.28,2.0	0
0.28,2.1	0
0.28,2.2	0
0.28,2.3	0
0.28,2.4	0
0.28,2.5	0
0.28,2.6	0
0.28,2.7	0
0.28,2.8	0
0.28,2.9	0
0.28,3.0	0
0.28,3.1	0
0.28,3.2	0
0.28,3.3	0
0.28,3.4	0
0.28,3.5	0
0.28,3.6	0
0.28,3.7	0
0.28,3.8	0
0.28,3.9	0
0.28,4.0	0
0.28,4.1	0
0.28,4.2	0
0.28,4.3	0
0.28,4.4	0
0.28,4.5	0
0.28,4.6	0
0.28,4.7	0
0.28,4.8	0
0.28,4.9	0
0.28,5.0	0
0.28,5.1	0
0.28,5.2	0
0.28,5.3	0
0.28,5.4	0
0.28,5.5	0
0.28,5.6	0
0.28,5.7	0
0.28,5.8	0
0.28,5.9	0
0.28,6.0	0
0.28,6.1	0

0.28,6.2	0
0.28,6.3	0
0.28,6.4	0
0.28,6.5	0
0.28,6.6	0
0.28,6.7	0
0.28,6.8	0
0.28,6.9	0
0.28,7.0	0
0.28,7.1	0
0.28,7.2	0
0.28,7.3	0
0.28,7.4	0
0.28,7.5	0
0.28,7.6	0
0.28,7.7	0
0.28,7.8	0
0.28,7.9	0
0.28,8.0	0
0.28,8.1	0
0.28,8.2	0
0.28,8.3	0
0.28,8.4	0
0.28,8.5	0
0.28,8.6	0
0.28,8.7	0
0.28,8.8	0
0.28,8.9	0
0.28,9.0	0
0.28,9.1	0
0.28,9.2	0
0.28,9.3	0
0.28,9.4	0
0.28,9.5	0
0.28,9.6	0
0.28,9.7	0
0.28,9.8	0
0.28,9.9	0
0.28,10.0	0
0.28,10.1	0
0.28,10.2	0
0.28,10.3	0

0.28,10.4	0
0.28,10.5	0
0.28,10.6	0
0.28,10.7	0
0.28,10.8	0
0.28,10.9	0
0.28,11.0	0
0.28,11.1	0
0.28,11.2	0
0.28,11.3	0
0.28,11.4	0
0.28,11.5	0
0.28,11.6	0
0.28,11.7	0
0.28,11.8	0
0.28,11.9	0
0.28,12.0	0
0.28,12.1	0
0.28,12.2	0
0.28,12.3	0
0.28,12.4	0
0.28,12.5	0
0.28,12.6	0
0.28,12.7	0
0.28,12.8	0
0.28,12.9	0
0.28,13.0	0
0.28,13.1	0
0.28,13.2	0
0.28,13.3	0
0.28,13.4	0
0.28,13.5	0
0.28,13.6	0
0.28,13.7	0
0.28,13.8	0
0.28,13.9	0
0.28,14.0	0
0.28,14.1	0
0.28,14.2	0
0.28,14.3	0
0.28,14.4	0
0.28,14.5	0

0.28,14.6	0
0.28,14.7	0
0.28,14.8	0
0.28,14.9	0
0.28,15.0	0
0.28,15.1	0
0.28,15.2	0
0.28,15.3	0
0.28,15.4	0
0.28,15.5	0
0.28,15.6	0
0.28,15.7	0
0.28,15.8	0
0.28,15.9	0
0.28,16.0	0
0.28,16.1	0
0.28,16.2	0
0.28,16.3	0
0.28,16.4	0
0.28,16.5	0
0.28,16.6	0
0.28,16.7	0
0.28,16.8	0
0.28,16.9	0
0.28,17.0	0
0.28,17.1	0
0.28,17.2	0
0.28,17.3	0
0.28,17.4	0
0.28,17.5	0
0.28,17.6	0
0.28,17.7	0
0.28,17.8	0
0.28,17.9	0
0.28,18.0	0
0.28,18.1	0
0.28,18.2	0
0.28,18.3	0
0.28,18.4	0
0.28,18.5	0
0.28,18.6	0
0.28,18.7	0

0.28,18.8	0
0.28,18.9	0
0.28,19.0	0
0.28,19.1	0
0.28,19.2	0
0.28,19.3	0
0.28,19.4	0
0.28,19.5	0
0.28,19.6	0
0.28,19.7	0
0.28,19.8	0
0.28,19.9	0
0.28,20.0	0
0.28,20.1	0
0.28,20.2	0
0.28,20.3	0
0.28,20.4	0
0.28,20.5	0
0.28,20.6	0
0.28,20.7	0
0.28,20.8	0
0.28,20.9	0
0.28,21.0	0
0.28,21.1	0
0.28,21.2	0
0.28,21.3	0
0.28,21.4	0
0.28,21.5	0
0.28,21.6	0
0.28,21.7	0
0.28,21.8	0
0.28,21.9	0
0.28,22.0	0
0.28,22.1	0
0.28,22.2	0
0.28,22.3	0
0.28,22.4	0
0.28,22.5	0
0.28,22.6	0
0.28,22.7	0
0.28,22.8	0
0.28,22.9	0

0.28,23.0	0
0.28,23.1	0
0.28,23.2	0
0.28,23.3	0
0.28,23.4	0
0.28,23.5	0
0.28,23.6	0
0.28,23.7	0
0.28,23.8	0
0.28,23.9	0
0.28,24.0	0
0.28,24.1	0
0.28,24.2	0
0.28,24.3	0
0.28,24.4	0
0.28,24.5	0
0.28,24.6	0
0.28,24.7	0
0.28,24.8	0
0.28,24.9	0
0.28,25.0	0
0.28,25.1	0
0.28,25.2	0
0.28,25.3	0
0.28,25.4	0
0.28,25.5	0
0.28,25.6	0
0.28,25.7	0
0.28,25.8	0
0.28,25.9	0
0.28,26.0	0
0.28,26.1	0
0.28,26.2	0
0.28,26.3	0
0.28,26.4	0
0.28,26.5	0
0.28,26.6	0
0.28,26.7	0
0.28,26.8	0
0.28,26.9	0
0.28,27.0	0
0.28,27.1	0

0.28,27.2	0
0.28,27.3	0
0.28,27.4	0
0.28,27.5	0
0.28,27.6	0
0.28,27.7	0
0.28,27.8	0
0.28,27.9	0
0.28,28.0	0
0.28,28.1	0
0.28,28.2	0
0.28,28.3	0
0.28,28.4	0
0.28,28.5	0
0.28,28.6	0
0.28,28.7	0
0.28,28.8	0
0.28,28.9	0
0.28,29.0	0
0.28,29.1	0
0.28,29.2	0
0.28,29.3	0
0.28,29.4	0
0.28,29.5	0
0.28,29.6	0
0.28,29.7	0
0.28,29.8	0
0.28,29.9	0
0.28,30.0	0
0.28,30.1	0
0.28,30.2	0
0.28,30.3	0
0.28,30.4	0
0.28,30.5	0
0.28,30.6	0
0.28,30.7	0
0.28,30.8	0
0.28,30.9	0
0.28,31.0	0
0.28,31.1	0
0.28,31.2	0
0.28,31.3	0

0.28,31.4	0
0.28,31.5	0
0.28,31.6	0
0.28,31.7	0
0.28,31.8	0
0.28,31.9	0
0.28,32.0	0
0.28,32.1	0
0.28,32.2	0
0.28,32.3	0
0.28,32.4	0
0.28,32.5	0
0.28,32.6	0
0.28,32.7	0
0.28,32.8	0
0.28,32.9	0
0.28,33.0	0
0.28,33.1	0
0.28,33.2	0
0.28,33.3	0
0.28,33.4	0
0.28,33.5	0
0.28,33.6	0
0.28,33.7	0
0.28,33.8	0
0.28,33.9	0
0.28,34.0	0
0.28,34.1	0
0.28,34.2	0
0.28,34.3	0
0.28,34.4	0
0.28,34.5	0
0.28,34.6	0
0.28,34.7	0
0.28,34.8	0
0.28,34.9	0
0.28,35.0	0
0.28,35.1	0
0.28,35.2	0
0.28,35.3	0
0.28,35.4	0
0.28,35.5	0

0.28,35.6	0
0.28,35.7	0
0.28,35.8	0
0.28,35.9	0
0.28,36.0	0
0.28,36.1	0
0.28,36.2	0
0.28,36.3	0
0.28,36.4	0
0.28,36.5	0
0.28,36.6	0
0.28,36.7	0
0.28,36.8	0
0.28,36.9	0
0.28,37.0	0
0.28,37.1	0
0.28,37.2	0
0.28,37.3	0
0.28,37.4	0
0.28,37.5	0
0.28,37.6	0
0.28,37.7	0
0.28,37.8	0
0.28,37.9	0
0.28,38.0	0
0.28,38.1	0
0.28,38.2	0
0.28,38.3	0
0.28,38.4	0
0.28,38.5	0
0.28,38.6	0
0.28,38.7	0
0.28,38.8	0
0.28,38.9	0
0.28,39.0	0
0.28,39.1	0
0.28,39.2	0
0.28,39.3	0
0.28,39.4	0
0.28,39.5	0
0.28,39.6	0
0.28,39.7	0

0.28,39.8	0
0.28,39.9	0
0.28,40.0	0
0.28,40.1	0
0.28,40.2	0
0.28,40.3	0
0.28,40.4	0
0.28,40.5	0
0.28,40.6	0
0.28,40.7	0
0.28,40.8	0
0.28,40.9	0
0.28,41.0	0
0.28,41.1	0
0.28,41.2	0
0.28,41.3	0
0.28,41.4	0
0.28,41.5	0
0.28,41.6	0
0.28,41.7	0
0.28,41.8	0
0.28,41.9	0
0.28,42.0	0
0.28,42.1	0
0.28,42.2	0
0.28,42.3	0
0.28,42.4	0
0.28,42.5	0
0.28,42.6	0
0.28,42.7	0
0.28,42.8	0
0.28,42.9	0
0.28,43.0	0
0.28,43.1	0
0.28,43.2	0
0.28,43.3	0
0.28,43.4	0
0.28,43.5	0
0.28,43.6	0
0.28,43.7	0
0.28,43.8	0
0.28,43.9	0

0.28,44.0	0
0.28,44.1	0
0.28,44.2	0
0.28,44.3	0
0.28,44.4	0
0.28,44.5	0
0.28,44.6	0
0.28,44.7	0
0.28,44.8	0
0.28,44.9	0
0.28,45.0	0
0.28,45.1	0
0.28,45.2	0
0.28,45.3	0
0.28,45.4	0
0.28,45.5	0
0.28,45.6	0
0.28,45.7	0
0.28,45.8	0
0.28,45.9	0
0.28,46.0	0
0.28,46.1	0
0.28,46.2	0
0.28,46.3	0
0.28,46.4	0
0.28,46.5	0
0.28,46.6	0
0.28,46.7	0
0.28,46.8	0
0.28,46.9	0
0.28,47.0	0
0.28,47.1	0
0.28,47.2	0
0.28,47.3	0
0.28,47.4	0
0.28,47.5	0
0.28,47.6	0
0.28,47.7	0
0.28,47.8	0
0.28,47.9	0
0.28,48.0	0
0.28,48.1	0

0.28,48.2	0
0.28,48.3	0
0.28,48.4	0
0.28,48.5	0
0.28,48.6	0
0.28,48.7	0
0.28,48.8	0
0.28,48.9	0
0.28,49.0	0
0.28,49.1	0
0.28,49.2	0
0.28,49.3	0
0.28,49.4	0
0.28,49.5	0
0.28,49.6	0
0.28,49.7	0
0.28,49.8	0
0.28,49.9	0
0.28,50.0	0
0.28,50.1	0
0.28,50.2	0
0.28,50.3	0
0.28,50.4	0
0.28,50.5	0
0.28,50.6	0
0.28,50.7	0
0.28,50.8	0
0.28,50.9	0
0.28,51.0	0
0.28,51.1	0
0.28,51.2	0
0.28,51.3	0
0.28,51.4	0
0.28,51.5	0
0.28,51.6	0
0.28,51.7	0
0.28,51.8	0
0.28,51.9	0
0.28,52.0	0
0.28,52.1	0
0.28,52.2	0
0.28,52.3	0

0.28,52.4	0
0.28,52.5	0
0.28,52.6	0
0.28,52.7	0
0.28,52.8	0
0.28,52.9	0
0.28,53.0	0
0.28,53.1	0
0.28,53.2	0
0.28,53.3	0
0.28,53.4	0
0.28,53.5	0
0.28,53.6	0
0.28,53.7	0
0.28,53.8	0
0.28,53.9	0
0.28,54.0	0
0.28,54.1	0
0.28,54.2	0
0.28,54.3	0
0.28,54.4	0
0.28,54.5	0
0.28,54.6	0
0.28,54.7	0
0.28,54.8	0
0.28,54.9	0
0.28,55.0	0
0.28,55.1	0
0.28,55.2	0
0.28,55.3	0
0.28,55.4	0
0.28,55.5	0
0.28,55.6	0
0.28,55.7	0
0.28,55.8	0
0.28,55.9	0
0.28,56.0	0
0.28,56.1	0
0.28,56.2	0
0.28,56.3	0
0.28,56.4	0
0.28,56.5	0

0.28,56.6	0
0.28,56.7	0
0.28,56.8	0
0.28,56.9	0
0.28,57.0	0
0.28,57.1	0
0.28,57.2	0
0.28,57.3	0
0.28,57.4	0
0.28,57.5	0
0.28,57.6	0
0.28,57.7	0
0.28,57.8	0
0.28,57.9	0
0.28,58.0	0
0.28,58.1	0
0.28,58.2	0
0.28,58.3	0
0.28,58.4	0
0.28,58.5	0
0.28,58.6	0
0.28,58.7	0
0.28,58.8	0
0.28,58.9	0
0.28,59.0	0
0.28,59.1	0
0.28,59.2	0
0.28,59.3	0
0.28,59.4	0
0.28,59.5	0
0.28,59.6	0
0.28,59.7	0
0.28,59.8	0
0.28,59.9	0
0.28,60.0	0
0.28,60.1	0
0.28,60.2	0
0.28,60.3	0
0.28,60.4	0
0.28,60.5	0
0.28,60.6	0
0.28,60.7	0

0.28,60.8	0
0.28,60.9	0
0.28,61.0	0
0.28,61.1	0
0.28,61.2	0
0.28,61.3	0
0.28,61.4	0
0.28,61.5	0
0.28,61.6	0
0.28,61.7	0
0.28,61.8	0
0.28,61.9	0
0.28,62.0	0
0.28,62.1	0
0.28,62.2	0
0.28,62.3	0
0.28,62.4	0
0.28,62.5	0
0.28,62.6	0
0.28,62.7	0
0.28,62.8	0
0.28,62.9	0
0.28,63.0	0
0.28,63.1	0
0.28,63.2	0
0.28,63.3	0
0.28,63.4	0
0.28,63.5	0
0.28,63.6	0
0.28,63.7	0
0.28,63.8	0
0.28,63.9	0
0.28,64.0	0
0.28,64.1	0
0.28,64.2	0
0.28,64.3	0
0.28,64.4	0
0.28,64.5	0
0.28,64.6	0
0.28,64.7	0
0.28,64.8	0
0.28,64.9	0

0.28,65.0	0
0.28,65.1	0
0.28,65.2	0
0.28,65.3	0
0.28,65.4	0
0.28,65.5	0
0.28,65.6	0
0.28,65.7	0
0.28,65.8	0
0.28,65.9	0
0.28,66.0	0
0.28,66.1	0
0.28,66.2	0
0.28,66.3	0
0.28,66.4	0
0.28,66.5	0
0.28,66.6	0
0.28,66.7	0
0.28,66.8	0
0.28,66.9	0
0.28,67.0	0
0.28,67.1	0
0.28,67.2	0
0.28,67.3	0
0.28,67.4	0
0.28,67.5	0
0.28,67.6	0
0.28,67.7	0
0.28,67.8	0
0.28,67.9	0
0.28,68.0	0
0.28,68.1	0
0.28,68.2	0
0.28,68.3	0
0.28,68.4	0
0.28,68.5	0
0.28,68.6	0
0.28,68.7	0
0.28,68.8	0
0.28,68.9	0
0.28,69.0	0
0.28,69.1	0

0.28,69.2	0
0.28,69.3	0
0.28,69.4	0
0.28,69.5	0
0.28,69.6	0
0.28,69.7	0
0.28,69.8	0
0.28,69.9	0
0.28,70.0	0
0.28,70.1	0
0.28,70.2	0
0.28,70.3	0
0.28,70.4	0
0.28,70.5	0
0.28,70.6	0
0.28,70.7	0
0.28,70.8	0
0.28,70.9	0
0.28,71.0	0
0.28,71.1	0
0.28,71.2	0
0.28,71.3	0
0.28,71.4	0
0.28,71.5	0
0.28,71.6	0
0.28,71.7	0
0.28,71.8	0
0.28,71.9	0
0.28,72.0	0
0.28,72.1	0
0.28,72.2	0
0.28,72.3	0
0.28,72.4	0
0.28,72.5	0
0.28,72.6	0
0.28,72.7	0
0.28,72.8	0
0.28,72.9	0
0.28,73.0	0
0.28,73.1	0
0.28,73.2	0
0.28,73.3	0

0.28,73.4	0
0.28,73.5	0
0.28,73.6	0
0.28,73.7	0
0.28,73.8	0
0.28,73.9	0
0.28,74.0	0
0.28,74.1	0
0.28,74.2	0
0.28,74.3	0
0.28,74.4	0
0.28,74.5	0
0.28,74.6	0
0.28,74.7	0
0.28,74.8	0
0.28,74.9	0
0.28,75.0	0
0.28,75.1	0
0.28,75.2	0
0.28,75.3	0
0.28,75.4	0
0.28,75.5	0
0.28,75.6	0
0.28,75.7	0
0.28,75.8	0
0.28,75.9	0
0.28,76.0	0
0.28,76.1	0
0.28,76.2	0
0.28,76.3	0
0.28,76.4	0
0.28,76.5	0
0.28,76.6	0
0.28,76.7	0
0.28,76.8	0
0.28,76.9	0
0.28,77.0	0
0.28,77.1	0
0.28,77.2	0
0.28,77.3	0
0.28,77.4	0
0.28,77.5	0

0.28,77.6	0
0.28,77.7	0
0.28,77.8	0
0.28,77.9	0
0.28,78.0	0
0.28,78.1	0
0.28,78.2	0
0.28,78.3	0
0.28,78.4	0
0.28,78.5	0
0.28,78.6	0
0.28,78.7	0
0.28,78.8	0
0.28,78.9	0
0.28,79.0	0
0.28,79.1	0
0.28,79.2	0
0.28,79.3	0
0.28,79.4	0
0.28,79.5	0
0.28,79.6	0
0.28,79.7	0
0.28,79.8	0
0.28,79.9	0
0.28,80.0	0
0.28,80.1	0
0.28,80.2	0
0.28,80.3	0
0.28,80.4	0
0.28,80.5	0
0.28,80.6	0
0.28,80.7	0
0.28,80.8	0
0.28,80.9	0
0.28,81.0	0
0.28,81.1	0
0.28,81.2	0
0.28,81.3	0
0.28,81.4	0
0.28,81.5	0
0.28,81.6	0
0.28,81.7	0

0.28,81.8	0
0.28,81.9	0
0.28,82.0	0
0.28,82.1	0
0.28,82.2	0
0.28,82.3	0
0.28,82.4	0
0.28,82.5	0
0.28,82.6	0
0.28,82.7	0
0.28,82.8	0
0.28,82.9	0
0.28,83.0	0
0.28,83.1	0
0.28,83.2	0
0.28,83.3	0
0.28,83.4	0
0.28,83.5	0
0.28,83.6	0
0.28,83.7	0
0.28,83.8	0
0.28,83.9	0
0.28,84.0	0
0.28,84.1	0
0.28,84.2	0
0.28,84.3	0
0.28,84.4	0
0.28,84.5	0
0.28,84.6	0
0.28,84.7	0
0.28,84.8	0
0.28,84.9	0
0.28,85.0	0
0.28,85.1	0
0.28,85.2	0
0.28,85.3	0
0.28,85.4	0
0.28,85.5	0
0.28,85.6	0
0.28,85.7	0
0.28,85.8	0
0.28,85.9	0

0.28,86.0	0
0.28,86.1	0
0.28,86.2	0
0.28,86.3	0
0.28,86.4	0
0.28,86.5	0
0.28,86.6	0
0.28,86.7	0
0.28,86.8	0
0.28,86.9	0
0.28,87.0	0
0.28,87.1	0
0.28,87.2	0
0.28,87.3	0
0.28,87.4	0
0.28,87.5	0
0.28,87.6	0
0.28,87.7	0
0.28,87.8	0
0.28,87.9	0
0.28,88.0	0
0.28,88.1	0
0.28,88.2	0
0.28,88.3	0
0.28,88.4	0
0.28,88.5	0
0.28,88.6	0
0.28,88.7	0
0.28,88.8	0
0.28,88.9	0
0.28,89.0	0
0.28,89.1	0
0.28,89.2	0
0.28,89.3	0
0.28,89.4	0
0.28,89.5	0
0.28,89.6	0
0.28,89.7	0
0.28,89.8	0
0.28,89.9	0
0.28,90.0	0
0.28,90.1	0

0.28,90.2	0
0.28,90.3	0
0.28,90.4	0
0.28,90.5	0
0.28,90.6	0
0.28,90.7	0
0.28,90.8	0
0.28,90.9	0
0.28,91.0	0
0.28,91.1	0
0.28,91.2	0
0.28,91.3	0
0.28,91.4	0
0.28,91.5	0
0.28,91.6	0
0.28,91.7	0
0.28,91.8	0
0.28,91.9	0
0.28,92.0	0
0.28,92.1	0
0.28,92.2	0
0.28,92.3	0
0.28,92.4	0
0.28,92.5	0
0.28,92.6	0
0.28,92.7	0
0.28,92.8	0
0.28,92.9	0
0.28,93.0	0
0.28,93.1	0
0.28,93.2	0
0.28,93.3	0
0.28,93.4	0
0.28,93.5	0
0.28,93.6	0
0.28,93.7	0
0.28,93.8	0
0.28,93.9	0
0.28,94.0	0
0.28,94.1	0
0.28,94.2	0
0.28,94.3	0

0.28,94.4	0
0.28,94.5	0
0.28,94.6	0
0.28,94.7	0
0.28,94.8	0
0.28,94.9	0
0.28,95.0	0
0.28,95.1	0
0.28,95.2	0
0.28,95.3	0
0.28,95.4	0
0.28,95.5	0
0.28,95.6	0
0.28,95.7	0
0.28,95.8	0
0.28,95.9	0
0.28,96.0	0
0.28,96.1	0
0.28,96.2	0
0.28,96.3	0
0.28,96.4	0
0.28,96.5	0
0.28,96.6	0
0.28,96.7	0
0.28,96.8	0
0.28,96.9	0
0.28,97.0	0
0.28,97.1	0
0.28,97.2	0
0.28,97.3	0
0.28,97.4	0
0.28,97.5	0
0.28,97.6	0
0.28,97.7	0
0.28,97.8	0
0.28,97.9	0
0.28,98.0	0
0.28,98.1	0
0.28,98.2	0
0.28,98.3	0
0.28,98.4	0
0.28,98.5	0

0.28,98.6	0
0.28,98.7	0
0.28,98.8	0
0.28,98.9	0
0.28,99.0	0
0.28,99.1	0
0.28,99.2	0
0.28,99.3	0
0.28,99.4	0
0.28,99.5	0
0.28,99.6	0
0.28,99.7	0
0.28,99.8	0
0.28,99.9	0
0.28,100.0	0
0.29,0.0	0
0.29,0.1	0
0.29,0.2	0
0.29,0.3	0
0.29,0.4	0
0.29,0.5	0
0.29,0.6	0
0.29,0.7	0
0.29,0.8	0
0.29,0.9	0
0.29,1.0	0
0.29,1.1	0
0.29,1.2	0
0.29,1.3	0
0.29,1.4	0
0.29,1.5	0
0.29,1.6	0
0.29,1.7	0
0.29,1.8	0
0.29,1.9	0
0.29,2.0	0
0.29,2.1	0
0.29,2.2	0
0.29,2.3	0
0.29,2.4	0
0.29,2.5	0
0.29,2.6	0

0.29,2.7	0
0.29,2.8	0
0.29,2.9	0
0.29,3.0	0
0.29,3.1	0
0.29,3.2	0
0.29,3.3	0
0.29,3.4	0
0.29,3.5	0
0.29,3.6	0
0.29,3.7	0
0.29,3.8	0
0.29,3.9	0
0.29,4.0	0
0.29,4.1	0
0.29,4.2	0
0.29,4.3	0
0.29,4.4	0
0.29,4.5	0
0.29,4.6	0
0.29,4.7	0
0.29,4.8	0
0.29,4.9	0
0.29,5.0	0
0.29,5.1	0
0.29,5.2	0
0.29,5.3	0
0.29,5.4	0
0.29,5.5	0
0.29,5.6	0
0.29,5.7	0
0.29,5.8	0
0.29,5.9	0
0.29,6.0	0
0.29,6.1	0
0.29,6.2	0
0.29,6.3	0
0.29,6.4	0
0.29,6.5	0
0.29,6.6	0
0.29,6.7	0
0.29,6.8	0

0.29,6.9	0
0.29,7.0	0
0.29,7.1	0
0.29,7.2	0
0.29,7.3	0
0.29,7.4	0
0.29,7.5	0
0.29,7.6	0
0.29,7.7	0
0.29,7.8	0
0.29,7.9	0
0.29,8.0	0
0.29,8.1	0
0.29,8.2	0
0.29,8.3	0
0.29,8.4	0
0.29,8.5	0
0.29,8.6	0
0.29,8.7	0
0.29,8.8	0
0.29,8.9	0
0.29,9.0	0
0.29,9.1	0
0.29,9.2	0
0.29,9.3	0
0.29,9.4	0
0.29,9.5	0
0.29,9.6	0
0.29,9.7	0
0.29,9.8	0
0.29,9.9	0
0.29,10.0	0
0.29,10.1	0
0.29,10.2	0
0.29,10.3	0
0.29,10.4	0
0.29,10.5	0
0.29,10.6	0
0.29,10.7	0
0.29,10.8	0
0.29,10.9	0
0.29,11.0	0

0.29,11.1	0
0.29,11.2	0
0.29,11.3	0
0.29,11.4	0
0.29,11.5	0
0.29,11.6	0
0.29,11.7	0
0.29,11.8	0
0.29,11.9	0
0.29,12.0	0
0.29,12.1	0
0.29,12.2	0
0.29,12.3	0
0.29,12.4	0
0.29,12.5	0
0.29,12.6	0
0.29,12.7	0
0.29,12.8	0
0.29,12.9	0
0.29,13.0	0
0.29,13.1	0
0.29,13.2	0
0.29,13.3	0
0.29,13.4	0
0.29,13.5	0
0.29,13.6	0
0.29,13.7	0
0.29,13.8	0
0.29,13.9	0
0.29,14.0	0
0.29,14.1	0
0.29,14.2	0
0.29,14.3	0
0.29,14.4	0
0.29,14.5	0
0.29,14.6	0
0.29,14.7	0
0.29,14.8	0
0.29,14.9	0
0.29,15.0	0
0.29,15.1	0
0.29,15.2	0

0.29,15.3	0
0.29,15.4	0
0.29,15.5	0
0.29,15.6	0
0.29,15.7	0
0.29,15.8	0
0.29,15.9	0
0.29,16.0	0
0.29,16.1	0
0.29,16.2	0
0.29,16.3	0
0.29,16.4	0
0.29,16.5	0
0.29,16.6	0
0.29,16.7	0
0.29,16.8	0
0.29,16.9	0
0.29,17.0	0
0.29,17.1	0
0.29,17.2	0
0.29,17.3	0
0.29,17.4	0
0.29,17.5	0
0.29,17.6	0
0.29,17.7	0
0.29,17.8	0
0.29,17.9	0
0.29,18.0	0
0.29,18.1	0
0.29,18.2	0
0.29,18.3	0
0.29,18.4	0
0.29,18.5	0
0.29,18.6	0
0.29,18.7	0
0.29,18.8	0
0.29,18.9	0
0.29,19.0	0
0.29,19.1	0
0.29,19.2	0
0.29,19.3	0
0.29,19.4	0

0.29,19.5	0
0.29,19.6	0
0.29,19.7	0
0.29,19.8	0
0.29,19.9	0
0.29,20.0	0
0.29,20.1	0
0.29,20.2	0
0.29,20.3	0
0.29,20.4	0
0.29,20.5	0
0.29,20.6	0
0.29,20.7	0
0.29,20.8	0
0.29,20.9	0
0.29,21.0	0
0.29,21.1	0
0.29,21.2	0
0.29,21.3	0
0.29,21.4	0
0.29,21.5	0
0.29,21.6	0
0.29,21.7	0
0.29,21.8	0
0.29,21.9	0
0.29,22.0	0
0.29,22.1	0
0.29,22.2	0
0.29,22.3	0
0.29,22.4	0
0.29,22.5	0
0.29,22.6	0
0.29,22.7	0
0.29,22.8	0
0.29,22.9	0
0.29,23.0	0
0.29,23.1	0
0.29,23.2	0
0.29,23.3	0
0.29,23.4	0
0.29,23.5	0
0.29,23.6	0

0.29,23.7	0
0.29,23.8	0
0.29,23.9	0
0.29,24.0	0
0.29,24.1	0
0.29,24.2	0
0.29,24.3	0
0.29,24.4	0
0.29,24.5	0
0.29,24.6	0
0.29,24.7	0
0.29,24.8	0
0.29,24.9	0
0.29,25.0	0
0.29,25.1	0
0.29,25.2	0
0.29,25.3	0
0.29,25.4	0
0.29,25.5	0
0.29,25.6	0
0.29,25.7	0
0.29,25.8	0
0.29,25.9	0
0.29,26.0	0
0.29,26.1	0
0.29,26.2	0
0.29,26.3	0
0.29,26.4	0
0.29,26.5	0
0.29,26.6	0
0.29,26.7	0
0.29,26.8	0
0.29,26.9	0
0.29,27.0	0
0.29,27.1	0
0.29,27.2	0
0.29,27.3	0
0.29,27.4	0
0.29,27.5	0
0.29,27.6	0
0.29,27.7	0
0.29,27.8	0

0.29,27.9	0
0.29,28.0	0
0.29,28.1	0
0.29,28.2	0
0.29,28.3	0
0.29,28.4	0
0.29,28.5	0
0.29,28.6	0
0.29,28.7	0
0.29,28.8	0
0.29,28.9	0
0.29,29.0	0
0.29,29.1	0
0.29,29.2	0
0.29,29.3	0
0.29,29.4	0
0.29,29.5	0
0.29,29.6	0
0.29,29.7	0
0.29,29.8	0
0.29,29.9	0
0.29,30.0	0
0.29,30.1	0
0.29,30.2	0
0.29,30.3	0
0.29,30.4	0
0.29,30.5	0
0.29,30.6	0
0.29,30.7	0
0.29,30.8	0
0.29,30.9	0
0.29,31.0	0
0.29,31.1	0
0.29,31.2	0
0.29,31.3	0
0.29,31.4	0
0.29,31.5	0
0.29,31.6	0
0.29,31.7	0
0.29,31.8	0
0.29,31.9	0
0.29,32.0	0

0.29,32.1	0
0.29,32.2	0
0.29,32.3	0
0.29,32.4	0
0.29,32.5	0
0.29,32.6	0
0.29,32.7	0
0.29,32.8	0
0.29,32.9	0
0.29,33.0	0
0.29,33.1	0
0.29,33.2	0
0.29,33.3	0
0.29,33.4	0
0.29,33.5	0
0.29,33.6	0
0.29,33.7	0
0.29,33.8	0
0.29,33.9	0
0.29,34.0	0
0.29,34.1	0
0.29,34.2	0
0.29,34.3	0
0.29,34.4	0
0.29,34.5	0
0.29,34.6	0
0.29,34.7	0
0.29,34.8	0
0.29,34.9	0
0.29,35.0	0
0.29,35.1	0
0.29,35.2	0
0.29,35.3	0
0.29,35.4	0
0.29,35.5	0
0.29,35.6	0
0.29,35.7	0
0.29,35.8	0
0.29,35.9	0
0.29,36.0	0
0.29,36.1	0
0.29,36.2	0

0.29,36.3	0
0.29,36.4	0
0.29,36.5	0
0.29,36.6	0
0.29,36.7	0
0.29,36.8	0
0.29,36.9	0
0.29,37.0	0
0.29,37.1	0
0.29,37.2	0
0.29,37.3	0
0.29,37.4	0
0.29,37.5	0
0.29,37.6	0
0.29,37.7	0
0.29,37.8	0
0.29,37.9	0
0.29,38.0	0
0.29,38.1	0
0.29,38.2	0
0.29,38.3	0
0.29,38.4	0
0.29,38.5	0
0.29,38.6	0
0.29,38.7	0
0.29,38.8	0
0.29,38.9	0
0.29,39.0	0
0.29,39.1	0
0.29,39.2	0
0.29,39.3	0
0.29,39.4	0
0.29,39.5	0
0.29,39.6	0
0.29,39.7	0
0.29,39.8	0
0.29,39.9	0
0.29,40.0	0
0.29,40.1	0
0.29,40.2	0
0.29,40.3	0
0.29,40.4	0

0.29,40.5	0
0.29,40.6	0
0.29,40.7	0
0.29,40.8	0
0.29,40.9	0
0.29,41.0	0
0.29,41.1	0
0.29,41.2	0
0.29,41.3	0
0.29,41.4	0
0.29,41.5	0
0.29,41.6	0
0.29,41.7	0
0.29,41.8	0
0.29,41.9	0
0.29,42.0	0
0.29,42.1	0
0.29,42.2	0
0.29,42.3	0
0.29,42.4	0
0.29,42.5	0
0.29,42.6	0
0.29,42.7	0
0.29,42.8	0
0.29,42.9	0
0.29,43.0	0
0.29,43.1	0
0.29,43.2	0
0.29,43.3	0
0.29,43.4	0
0.29,43.5	0
0.29,43.6	0
0.29,43.7	0
0.29,43.8	0
0.29,43.9	0
0.29,44.0	0
0.29,44.1	0
0.29,44.2	0
0.29,44.3	0
0.29,44.4	0
0.29,44.5	0
0.29,44.6	0

0.29,44.7	0
0.29,44.8	0
0.29,44.9	0
0.29,45.0	0
0.29,45.1	0
0.29,45.2	0
0.29,45.3	0
0.29,45.4	0
0.29,45.5	0
0.29,45.6	0
0.29,45.7	0
0.29,45.8	0
0.29,45.9	0
0.29,46.0	0
0.29,46.1	0
0.29,46.2	0
0.29,46.3	0
0.29,46.4	0
0.29,46.5	0
0.29,46.6	0
0.29,46.7	0
0.29,46.8	0
0.29,46.9	0
0.29,47.0	0
0.29,47.1	0
0.29,47.2	0
0.29,47.3	0
0.29,47.4	0
0.29,47.5	0
0.29,47.6	0
0.29,47.7	0
0.29,47.8	0
0.29,47.9	0
0.29,48.0	0
0.29,48.1	0
0.29,48.2	0
0.29,48.3	0
0.29,48.4	0
0.29,48.5	0
0.29,48.6	0
0.29,48.7	0
0.29,48.8	0

0.29,48.9	0
0.29,49.0	0
0.29,49.1	0
0.29,49.2	0
0.29,49.3	0
0.29,49.4	0
0.29,49.5	0
0.29,49.6	0
0.29,49.7	0
0.29,49.8	0
0.29,49.9	0
0.29,50.0	0
0.29,50.1	0
0.29,50.2	0
0.29,50.3	0
0.29,50.4	0
0.29,50.5	0
0.29,50.6	0
0.29,50.7	0
0.29,50.8	0
0.29,50.9	0
0.29,51.0	0
0.29,51.1	0
0.29,51.2	0
0.29,51.3	0
0.29,51.4	0
0.29,51.5	0
0.29,51.6	0
0.29,51.7	0
0.29,51.8	0
0.29,51.9	0
0.29,52.0	0
0.29,52.1	0
0.29,52.2	0
0.29,52.3	0
0.29,52.4	0
0.29,52.5	0
0.29,52.6	0
0.29,52.7	0
0.29,52.8	0
0.29,52.9	0
0.29,53.0	0

0.29,53.1	0
0.29,53.2	0
0.29,53.3	0
0.29,53.4	0
0.29,53.5	0
0.29,53.6	0
0.29,53.7	0
0.29,53.8	0
0.29,53.9	0
0.29,54.0	0
0.29,54.1	0
0.29,54.2	0
0.29,54.3	0
0.29,54.4	0
0.29,54.5	0
0.29,54.6	0
0.29,54.7	0
0.29,54.8	0
0.29,54.9	0
0.29,55.0	0
0.29,55.1	0
0.29,55.2	0
0.29,55.3	0
0.29,55.4	0
0.29,55.5	0
0.29,55.6	0
0.29,55.7	0
0.29,55.8	0
0.29,55.9	0
0.29,56.0	0
0.29,56.1	0
0.29,56.2	0
0.29,56.3	0
0.29,56.4	0
0.29,56.5	0
0.29,56.6	0
0.29,56.7	0
0.29,56.8	0
0.29,56.9	0
0.29,57.0	0
0.29,57.1	0
0.29,57.2	0

0.29,57.3	0
0.29,57.4	0
0.29,57.5	0
0.29,57.6	0
0.29,57.7	0
0.29,57.8	0
0.29,57.9	0
0.29,58.0	0
0.29,58.1	0
0.29,58.2	0
0.29,58.3	0
0.29,58.4	0
0.29,58.5	0
0.29,58.6	0
0.29,58.7	0
0.29,58.8	0
0.29,58.9	0
0.29,59.0	0
0.29,59.1	0
0.29,59.2	0
0.29,59.3	0
0.29,59.4	0
0.29,59.5	0
0.29,59.6	0
0.29,59.7	0
0.29,59.8	0
0.29,59.9	0
0.29,60.0	0
0.29,60.1	0
0.29,60.2	0
0.29,60.3	0
0.29,60.4	0
0.29,60.5	0
0.29,60.6	0
0.29,60.7	0
0.29,60.8	0
0.29,60.9	0
0.29,61.0	0
0.29,61.1	0
0.29,61.2	0
0.29,61.3	0
0.29,61.4	0

0.29,61.5	0
0.29,61.6	0
0.29,61.7	0
0.29,61.8	0
0.29,61.9	0
0.29,62.0	0
0.29,62.1	0
0.29,62.2	0
0.29,62.3	0
0.29,62.4	0
0.29,62.5	0
0.29,62.6	0
0.29,62.7	0
0.29,62.8	0
0.29,62.9	0
0.29,63.0	0
0.29,63.1	0
0.29,63.2	0
0.29,63.3	0
0.29,63.4	0
0.29,63.5	0
0.29,63.6	0
0.29,63.7	0
0.29,63.8	0
0.29,63.9	0
0.29,64.0	0
0.29,64.1	0
0.29,64.2	0
0.29,64.3	0
0.29,64.4	0
0.29,64.5	0
0.29,64.6	0
0.29,64.7	0
0.29,64.8	0
0.29,64.9	0
0.29,65.0	0
0.29,65.1	0
0.29,65.2	0
0.29,65.3	0
0.29,65.4	0
0.29,65.5	0
0.29,65.6	0

0.29,65.7	0
0.29,65.8	0
0.29,65.9	0
0.29,66.0	0
0.29,66.1	0
0.29,66.2	0
0.29,66.3	0
0.29,66.4	0
0.29,66.5	0
0.29,66.6	0
0.29,66.7	0
0.29,66.8	0
0.29,66.9	0
0.29,67.0	0
0.29,67.1	0
0.29,67.2	0
0.29,67.3	0
0.29,67.4	0
0.29,67.5	0
0.29,67.6	0
0.29,67.7	0
0.29,67.8	0
0.29,67.9	0
0.29,68.0	0
0.29,68.1	0
0.29,68.2	0
0.29,68.3	0
0.29,68.4	0
0.29,68.5	0
0.29,68.6	0
0.29,68.7	0
0.29,68.8	0
0.29,68.9	0
0.29,69.0	0
0.29,69.1	0
0.29,69.2	0
0.29,69.3	0
0.29,69.4	0
0.29,69.5	0
0.29,69.6	0
0.29,69.7	0
0.29,69.8	0

0.29,69.9	0
0.29,70.0	0
0.29,70.1	0
0.29,70.2	0
0.29,70.3	0
0.29,70.4	0
0.29,70.5	0
0.29,70.6	0
0.29,70.7	0
0.29,70.8	0
0.29,70.9	0
0.29,71.0	0
0.29,71.1	0
0.29,71.2	0
0.29,71.3	0
0.29,71.4	0
0.29,71.5	0
0.29,71.6	0
0.29,71.7	0
0.29,71.8	0
0.29,71.9	0
0.29,72.0	0
0.29,72.1	0
0.29,72.2	0
0.29,72.3	0
0.29,72.4	0
0.29,72.5	0
0.29,72.6	0
0.29,72.7	0
0.29,72.8	0
0.29,72.9	0
0.29,73.0	0
0.29,73.1	0
0.29,73.2	0
0.29,73.3	0
0.29,73.4	0
0.29,73.5	0
0.29,73.6	0
0.29,73.7	0
0.29,73.8	0
0.29,73.9	0
0.29,74.0	0

0.29,74.1	0
0.29,74.2	0
0.29,74.3	0
0.29,74.4	0
0.29,74.5	0
0.29,74.6	0
0.29,74.7	0
0.29,74.8	0
0.29,74.9	0
0.29,75.0	0
0.29,75.1	0
0.29,75.2	0
0.29,75.3	0
0.29,75.4	0
0.29,75.5	0
0.29,75.6	0
0.29,75.7	0
0.29,75.8	0
0.29,75.9	0
0.29,76.0	0
0.29,76.1	0
0.29,76.2	0
0.29,76.3	0
0.29,76.4	0
0.29,76.5	0
0.29,76.6	0
0.29,76.7	0
0.29,76.8	0
0.29,76.9	0
0.29,77.0	0
0.29,77.1	0
0.29,77.2	0
0.29,77.3	0
0.29,77.4	0
0.29,77.5	0
0.29,77.6	0
0.29,77.7	0
0.29,77.8	0
0.29,77.9	0
0.29,78.0	0
0.29,78.1	0
0.29,78.2	0

0.29,78.3	0
0.29,78.4	0
0.29,78.5	0
0.29,78.6	0
0.29,78.7	0
0.29,78.8	0
0.29,78.9	0
0.29,79.0	0
0.29,79.1	0
0.29,79.2	0
0.29,79.3	0
0.29,79.4	0
0.29,79.5	0
0.29,79.6	0
0.29,79.7	0
0.29,79.8	0
0.29,79.9	0
0.29,80.0	0
0.29,80.1	0
0.29,80.2	0
0.29,80.3	0
0.29,80.4	0
0.29,80.5	0
0.29,80.6	0
0.29,80.7	0
0.29,80.8	0
0.29,80.9	0
0.29,81.0	0
0.29,81.1	0
0.29,81.2	0
0.29,81.3	0
0.29,81.4	0
0.29,81.5	0
0.29,81.6	0
0.29,81.7	0
0.29,81.8	0
0.29,81.9	0
0.29,82.0	0
0.29,82.1	0
0.29,82.2	0
0.29,82.3	0
0.29,82.4	0

0.29,82.5	0
0.29,82.6	0
0.29,82.7	0
0.29,82.8	0
0.29,82.9	0
0.29,83.0	0
0.29,83.1	0
0.29,83.2	0
0.29,83.3	0
0.29,83.4	0
0.29,83.5	0
0.29,83.6	0
0.29,83.7	0
0.29,83.8	0
0.29,83.9	0
0.29,84.0	0
0.29,84.1	0
0.29,84.2	0
0.29,84.3	0
0.29,84.4	0
0.29,84.5	0
0.29,84.6	0
0.29,84.7	0
0.29,84.8	0
0.29,84.9	0
0.29,85.0	0
0.29,85.1	0
0.29,85.2	0
0.29,85.3	0
0.29,85.4	0
0.29,85.5	0
0.29,85.6	0
0.29,85.7	0
0.29,85.8	0
0.29,85.9	0
0.29,86.0	0
0.29,86.1	0
0.29,86.2	0
0.29,86.3	0
0.29,86.4	0
0.29,86.5	0
0.29,86.6	0

0.29,86.7	0
0.29,86.8	0
0.29,86.9	0
0.29,87.0	0
0.29,87.1	0
0.29,87.2	0
0.29,87.3	0
0.29,87.4	0
0.29,87.5	0
0.29,87.6	0
0.29,87.7	0
0.29,87.8	0
0.29,87.9	0
0.29,88.0	0
0.29,88.1	0
0.29,88.2	0
0.29,88.3	0
0.29,88.4	0
0.29,88.5	0
0.29,88.6	0
0.29,88.7	0
0.29,88.8	0
0.29,88.9	0
0.29,89.0	0
0.29,89.1	0
0.29,89.2	0
0.29,89.3	0
0.29,89.4	0
0.29,89.5	0
0.29,89.6	0
0.29,89.7	0
0.29,89.8	0
0.29,89.9	0
0.29,90.0	0
0.29,90.1	0
0.29,90.2	0
0.29,90.3	0
0.29,90.4	0
0.29,90.5	0
0.29,90.6	0
0.29,90.7	0
0.29,90.8	0

0.29,90.9	0
0.29,91.0	0
0.29,91.1	0
0.29,91.2	0
0.29,91.3	0
0.29,91.4	0
0.29,91.5	0
0.29,91.6	0
0.29,91.7	0
0.29,91.8	0
0.29,91.9	0
0.29,92.0	0
0.29,92.1	0
0.29,92.2	0
0.29,92.3	0
0.29,92.4	0
0.29,92.5	0
0.29,92.6	0
0.29,92.7	0
0.29,92.8	0
0.29,92.9	0
0.29,93.0	0
0.29,93.1	0
0.29,93.2	0
0.29,93.3	0
0.29,93.4	0
0.29,93.5	0
0.29,93.6	0
0.29,93.7	0
0.29,93.8	0
0.29,93.9	0
0.29,94.0	0
0.29,94.1	0
0.29,94.2	0
0.29,94.3	0
0.29,94.4	0
0.29,94.5	0
0.29,94.6	0
0.29,94.7	0
0.29,94.8	0
0.29,94.9	0
0.29,95.0	0

0.29,95.1	0
0.29,95.2	0
0.29,95.3	0
0.29,95.4	0
0.29,95.5	0
0.29,95.6	0
0.29,95.7	0
0.29,95.8	0
0.29,95.9	0
0.29,96.0	0
0.29,96.1	0
0.29,96.2	0
0.29,96.3	0
0.29,96.4	0
0.29,96.5	0
0.29,96.6	0
0.29,96.7	0
0.29,96.8	0
0.29,96.9	0
0.29,97.0	0
0.29,97.1	0
0.29,97.2	0
0.29,97.3	0
0.29,97.4	0
0.29,97.5	0
0.29,97.6	0
0.29,97.7	0
0.29,97.8	0
0.29,97.9	0
0.29,98.0	0
0.29,98.1	0
0.29,98.2	0
0.29,98.3	0
0.29,98.4	0
0.29,98.5	0
0.29,98.6	0
0.29,98.7	0
0.29,98.8	0
0.29,98.9	0
0.29,99.0	0
0.29,99.1	0
0.29,99.2	0

0.29,99.3	0
0.29,99.4	0
0.29,99.5	0
0.29,99.6	0
0.29,99.7	0
0.29,99.8	0
0.29,99.9	0
0.29,100.0	0
0.30,0.0	0
0.30,0.1	0
0.30,0.2	0
0.30,0.3	0
0.30,0.4	0
0.30,0.5	0
0.30,0.6	0
0.30,0.7	0
0.30,0.8	0
0.30,0.9	0
0.30,1.0	0
0.30,1.1	0
0.30,1.2	0
0.30,1.3	0
0.30,1.4	0
0.30,1.5	0
0.30,1.6	0
0.30,1.7	0
0.30,1.8	0
0.30,1.9	0
0.30,2.0	0
0.30,2.1	0
0.30,2.2	0
0.30,2.3	0
0.30,2.4	0
0.30,2.5	0
0.30,2.6	0
0.30,2.7	0
0.30,2.8	0
0.30,2.9	0
0.30,3.0	0
0.30,3.1	0
0.30,3.2	0
0.30,3.3	0

0.30,3.4	0
0.30,3.5	0
0.30,3.6	0
0.30,3.7	0
0.30,3.8	0
0.30,3.9	0
0.30,4.0	0
0.30,4.1	0
0.30,4.2	0
0.30,4.3	0
0.30,4.4	0
0.30,4.5	0
0.30,4.6	0
0.30,4.7	0
0.30,4.8	0
0.30,4.9	0
0.30,5.0	0
0.30,5.1	0
0.30,5.2	0
0.30,5.3	0
0.30,5.4	0
0.30,5.5	0
0.30,5.6	0
0.30,5.7	0
0.30,5.8	0
0.30,5.9	0
0.30,6.0	0
0.30,6.1	0
0.30,6.2	0
0.30,6.3	0
0.30,6.4	0
0.30,6.5	0
0.30,6.6	0
0.30,6.7	0
0.30,6.8	0
0.30,6.9	0
0.30,7.0	0
0.30,7.1	0
0.30,7.2	0
0.30,7.3	0
0.30,7.4	0
0.30,7.5	0

0.30,7.6	0
0.30,7.7	0
0.30,7.8	0
0.30,7.9	0
0.30,8.0	0
0.30,8.1	0
0.30,8.2	0
0.30,8.3	0
0.30,8.4	0
0.30,8.5	0
0.30,8.6	0
0.30,8.7	0
0.30,8.8	0
0.30,8.9	0
0.30,9.0	0
0.30,9.1	0
0.30,9.2	0
0.30,9.3	0
0.30,9.4	0
0.30,9.5	0
0.30,9.6	0
0.30,9.7	0
0.30,9.8	0
0.30,9.9	0
0.30,10.0	0
0.30,10.1	0
0.30,10.2	0
0.30,10.3	0
0.30,10.4	0
0.30,10.5	0
0.30,10.6	0
0.30,10.7	0
0.30,10.8	0
0.30,10.9	0
0.30,11.0	0
0.30,11.1	0
0.30,11.2	0
0.30,11.3	0
0.30,11.4	0
0.30,11.5	0
0.30,11.6	0
0.30,11.7	0

0.30,11.8	0
0.30,11.9	0
0.30,12.0	0
0.30,12.1	0
0.30,12.2	0
0.30,12.3	0
0.30,12.4	0
0.30,12.5	0
0.30,12.6	0
0.30,12.7	0
0.30,12.8	0
0.30,12.9	0
0.30,13.0	0
0.30,13.1	0
0.30,13.2	0
0.30,13.3	0
0.30,13.4	0
0.30,13.5	0
0.30,13.6	0
0.30,13.7	0
0.30,13.8	0
0.30,13.9	0
0.30,14.0	0
0.30,14.1	0
0.30,14.2	0
0.30,14.3	0
0.30,14.4	0
0.30,14.5	0
0.30,14.6	0
0.30,14.7	0
0.30,14.8	0
0.30,14.9	0
0.30,15.0	0
0.30,15.1	0
0.30,15.2	0
0.30,15.3	0
0.30,15.4	0
0.30,15.5	0
0.30,15.6	0
0.30,15.7	0
0.30,15.8	0
0.30,15.9	0

0.30,16.0	0
0.30,16.1	0
0.30,16.2	0
0.30,16.3	0
0.30,16.4	0
0.30,16.5	0
0.30,16.6	0
0.30,16.7	0
0.30,16.8	0
0.30,16.9	0
0.30,17.0	0
0.30,17.1	0
0.30,17.2	0
0.30,17.3	0
0.30,17.4	0
0.30,17.5	0
0.30,17.6	0
0.30,17.7	0
0.30,17.8	0
0.30,17.9	0
0.30,18.0	0
0.30,18.1	0
0.30,18.2	0
0.30,18.3	0
0.30,18.4	0
0.30,18.5	0
0.30,18.6	0
0.30,18.7	0
0.30,18.8	0
0.30,18.9	0
0.30,19.0	0
0.30,19.1	0
0.30,19.2	0
0.30,19.3	0
0.30,19.4	0
0.30,19.5	0
0.30,19.6	0
0.30,19.7	0
0.30,19.8	0
0.30,19.9	0
0.30,20.0	0
0.30,20.1	0

0.30,20.2	0
0.30,20.3	0
0.30,20.4	0
0.30,20.5	0
0.30,20.6	0
0.30,20.7	0
0.30,20.8	0
0.30,20.9	0
0.30,21.0	0
0.30,21.1	0
0.30,21.2	0
0.30,21.3	0
0.30,21.4	0
0.30,21.5	0
0.30,21.6	0
0.30,21.7	0
0.30,21.8	0
0.30,21.9	0
0.30,22.0	0
0.30,22.1	0
0.30,22.2	0
0.30,22.3	0
0.30,22.4	0
0.30,22.5	0
0.30,22.6	0
0.30,22.7	0
0.30,22.8	0
0.30,22.9	0
0.30,23.0	0
0.30,23.1	0
0.30,23.2	0
0.30,23.3	0
0.30,23.4	0
0.30,23.5	0
0.30,23.6	0
0.30,23.7	0
0.30,23.8	0
0.30,23.9	0
0.30,24.0	0
0.30,24.1	0
0.30,24.2	0
0.30,24.3	0

0.30,24.4	0
0.30,24.5	0
0.30,24.6	0
0.30,24.7	0
0.30,24.8	0
0.30,24.9	0
0.30,25.0	0
0.30,25.1	0
0.30,25.2	0
0.30,25.3	0
0.30,25.4	0
0.30,25.5	0
0.30,25.6	0
0.30,25.7	0
0.30,25.8	0
0.30,25.9	0
0.30,26.0	0
0.30,26.1	0
0.30,26.2	0
0.30,26.3	0
0.30,26.4	0
0.30,26.5	0
0.30,26.6	0
0.30,26.7	0
0.30,26.8	0
0.30,26.9	0
0.30,27.0	0
0.30,27.1	0
0.30,27.2	0
0.30,27.3	0
0.30,27.4	0
0.30,27.5	0
0.30,27.6	0
0.30,27.7	0
0.30,27.8	0
0.30,27.9	0
0.30,28.0	0
0.30,28.1	0
0.30,28.2	0
0.30,28.3	0
0.30,28.4	0
0.30,28.5	0

0.30,28.6	0
0.30,28.7	0
0.30,28.8	0
0.30,28.9	0
0.30,29.0	0
0.30,29.1	0
0.30,29.2	0
0.30,29.3	0
0.30,29.4	0
0.30,29.5	0
0.30,29.6	0
0.30,29.7	0
0.30,29.8	0
0.30,29.9	0
0.30,30.0	0
0.30,30.1	0
0.30,30.2	0
0.30,30.3	0
0.30,30.4	0
0.30,30.5	0
0.30,30.6	0
0.30,30.7	0
0.30,30.8	0
0.30,30.9	0
0.30,31.0	0
0.30,31.1	0
0.30,31.2	0
0.30,31.3	0
0.30,31.4	0
0.30,31.5	0
0.30,31.6	0
0.30,31.7	0
0.30,31.8	0
0.30,31.9	0
0.30,32.0	0
0.30,32.1	0
0.30,32.2	0
0.30,32.3	0
0.30,32.4	0
0.30,32.5	0
0.30,32.6	0
0.30,32.7	0

0.30,32.8	0
0.30,32.9	0
0.30,33.0	0
0.30,33.1	0
0.30,33.2	0
0.30,33.3	0
0.30,33.4	0
0.30,33.5	0
0.30,33.6	0
0.30,33.7	0
0.30,33.8	0
0.30,33.9	0
0.30,34.0	0
0.30,34.1	0
0.30,34.2	0
0.30,34.3	0
0.30,34.4	0
0.30,34.5	0
0.30,34.6	0
0.30,34.7	0
0.30,34.8	0
0.30,34.9	0
0.30,35.0	0
0.30,35.1	0
0.30,35.2	0
0.30,35.3	0
0.30,35.4	0
0.30,35.5	0
0.30,35.6	0
0.30,35.7	0
0.30,35.8	0
0.30,35.9	0
0.30,36.0	0
0.30,36.1	0
0.30,36.2	0
0.30,36.3	0
0.30,36.4	0
0.30,36.5	0
0.30,36.6	0
0.30,36.7	0
0.30,36.8	0
0.30,36.9	0

0.30,37.0	0
0.30,37.1	0
0.30,37.2	0
0.30,37.3	0
0.30,37.4	0
0.30,37.5	0
0.30,37.6	0
0.30,37.7	0
0.30,37.8	0
0.30,37.9	0
0.30,38.0	0
0.30,38.1	0
0.30,38.2	0
0.30,38.3	0
0.30,38.4	0
0.30,38.5	0
0.30,38.6	0
0.30,38.7	0
0.30,38.8	0
0.30,38.9	0
0.30,39.0	0
0.30,39.1	0
0.30,39.2	0
0.30,39.3	0
0.30,39.4	0
0.30,39.5	0
0.30,39.6	0
0.30,39.7	0
0.30,39.8	0
0.30,39.9	0
0.30,40.0	0
0.30,40.1	0
0.30,40.2	0
0.30,40.3	0
0.30,40.4	0
0.30,40.5	0
0.30,40.6	0
0.30,40.7	0
0.30,40.8	0
0.30,40.9	0
0.30,41.0	0
0.30,41.1	0

0.30,41.2	0
0.30,41.3	0
0.30,41.4	0
0.30,41.5	0
0.30,41.6	0
0.30,41.7	0
0.30,41.8	0
0.30,41.9	0
0.30,42.0	0
0.30,42.1	0
0.30,42.2	0
0.30,42.3	0
0.30,42.4	0
0.30,42.5	0
0.30,42.6	0
0.30,42.7	0
0.30,42.8	0
0.30,42.9	0
0.30,43.0	0
0.30,43.1	0
0.30,43.2	0
0.30,43.3	0
0.30,43.4	0
0.30,43.5	0
0.30,43.6	0
0.30,43.7	0
0.30,43.8	0
0.30,43.9	0
0.30,44.0	0
0.30,44.1	0
0.30,44.2	0
0.30,44.3	0
0.30,44.4	0
0.30,44.5	0
0.30,44.6	0
0.30,44.7	0
0.30,44.8	0
0.30,44.9	0
0.30,45.0	0
0.30,45.1	0
0.30,45.2	0
0.30,45.3	0

0.30,45.4	0
0.30,45.5	0
0.30,45.6	0
0.30,45.7	0
0.30,45.8	0
0.30,45.9	0
0.30,46.0	0
0.30,46.1	0
0.30,46.2	0
0.30,46.3	0
0.30,46.4	0
0.30,46.5	0
0.30,46.6	0
0.30,46.7	0
0.30,46.8	0
0.30,46.9	0
0.30,47.0	0
0.30,47.1	0
0.30,47.2	0
0.30,47.3	0
0.30,47.4	0
0.30,47.5	0
0.30,47.6	0
0.30,47.7	0
0.30,47.8	0
0.30,47.9	0
0.30,48.0	0
0.30,48.1	0
0.30,48.2	0
0.30,48.3	0
0.30,48.4	0
0.30,48.5	0
0.30,48.6	0
0.30,48.7	0
0.30,48.8	0
0.30,48.9	0
0.30,49.0	0
0.30,49.1	0
0.30,49.2	0
0.30,49.3	0
0.30,49.4	0
0.30,49.5	0

0.30,49.6	0
0.30,49.7	0
0.30,49.8	0
0.30,49.9	0
0.30,50.0	0
0.30,50.1	0
0.30,50.2	0
0.30,50.3	0
0.30,50.4	0
0.30,50.5	0
0.30,50.6	0
0.30,50.7	0
0.30,50.8	0
0.30,50.9	0
0.30,51.0	0
0.30,51.1	0
0.30,51.2	0
0.30,51.3	0
0.30,51.4	0
0.30,51.5	0
0.30,51.6	0
0.30,51.7	0
0.30,51.8	0
0.30,51.9	0
0.30,52.0	0
0.30,52.1	0
0.30,52.2	0
0.30,52.3	0
0.30,52.4	0
0.30,52.5	0
0.30,52.6	0
0.30,52.7	0
0.30,52.8	0
0.30,52.9	0
0.30,53.0	0
0.30,53.1	0
0.30,53.2	0
0.30,53.3	0
0.30,53.4	0
0.30,53.5	0
0.30,53.6	0
0.30,53.7	0

0.30,53.8	0
0.30,53.9	0
0.30,54.0	0
0.30,54.1	0
0.30,54.2	0
0.30,54.3	0
0.30,54.4	0
0.30,54.5	0
0.30,54.6	0
0.30,54.7	0
0.30,54.8	0
0.30,54.9	0
0.30,55.0	0
0.30,55.1	0
0.30,55.2	0
0.30,55.3	0
0.30,55.4	0
0.30,55.5	0
0.30,55.6	0
0.30,55.7	0
0.30,55.8	0
0.30,55.9	0
0.30,56.0	0
0.30,56.1	0
0.30,56.2	0
0.30,56.3	0
0.30,56.4	0
0.30,56.5	0
0.30,56.6	0
0.30,56.7	0
0.30,56.8	0
0.30,56.9	0
0.30,57.0	0
0.30,57.1	0
0.30,57.2	0
0.30,57.3	0
0.30,57.4	0
0.30,57.5	0
0.30,57.6	0
0.30,57.7	0
0.30,57.8	0
0.30,57.9	0

0.30,58.0	0
0.30,58.1	0
0.30,58.2	0
0.30,58.3	0
0.30,58.4	0
0.30,58.5	0
0.30,58.6	0
0.30,58.7	0
0.30,58.8	0
0.30,58.9	0
0.30,59.0	0
0.30,59.1	0
0.30,59.2	0
0.30,59.3	0
0.30,59.4	0
0.30,59.5	0
0.30,59.6	0
0.30,59.7	0
0.30,59.8	0
0.30,59.9	0
0.30,60.0	0
0.30,60.1	0
0.30,60.2	0
0.30,60.3	0
0.30,60.4	0
0.30,60.5	0
0.30,60.6	0
0.30,60.7	0
0.30,60.8	0
0.30,60.9	0
0.30,61.0	0
0.30,61.1	0
0.30,61.2	0
0.30,61.3	0
0.30,61.4	0
0.30,61.5	0
0.30,61.6	0
0.30,61.7	0
0.30,61.8	0
0.30,61.9	0
0.30,62.0	0
0.30,62.1	0

0.30,62.2	0
0.30,62.3	0
0.30,62.4	0
0.30,62.5	0
0.30,62.6	0
0.30,62.7	0
0.30,62.8	0
0.30,62.9	0
0.30,63.0	0
0.30,63.1	0
0.30,63.2	0
0.30,63.3	0
0.30,63.4	0
0.30,63.5	0
0.30,63.6	0
0.30,63.7	0
0.30,63.8	0
0.30,63.9	0
0.30,64.0	0
0.30,64.1	0
0.30,64.2	0
0.30,64.3	0
0.30,64.4	0
0.30,64.5	0
0.30,64.6	0
0.30,64.7	0
0.30,64.8	0
0.30,64.9	0
0.30,65.0	0
0.30,65.1	0
0.30,65.2	0
0.30,65.3	0
0.30,65.4	0
0.30,65.5	0
0.30,65.6	0
0.30,65.7	0
0.30,65.8	0
0.30,65.9	0
0.30,66.0	0
0.30,66.1	0
0.30,66.2	0
0.30,66.3	0

0.30,66.4	0
0.30,66.5	0
0.30,66.6	0
0.30,66.7	0
0.30,66.8	0
0.30,66.9	0
0.30,67.0	0
0.30,67.1	0
0.30,67.2	0
0.30,67.3	0
0.30,67.4	0
0.30,67.5	0
0.30,67.6	0
0.30,67.7	0
0.30,67.8	0
0.30,67.9	0
0.30,68.0	0
0.30,68.1	0
0.30,68.2	0
0.30,68.3	0
0.30,68.4	0
0.30,68.5	0
0.30,68.6	0
0.30,68.7	0
0.30,68.8	0
0.30,68.9	0
0.30,69.0	0
0.30,69.1	0
0.30,69.2	0
0.30,69.3	0
0.30,69.4	0
0.30,69.5	0
0.30,69.6	0
0.30,69.7	0
0.30,69.8	0
0.30,69.9	0
0.30,70.0	0
0.30,70.1	0
0.30,70.2	0
0.30,70.3	0
0.30,70.4	0
0.30,70.5	0

0.30,70.6	0
0.30,70.7	0
0.30,70.8	0
0.30,70.9	0
0.30,71.0	0
0.30,71.1	0
0.30,71.2	0
0.30,71.3	0
0.30,71.4	0
0.30,71.5	0
0.30,71.6	0
0.30,71.7	0
0.30,71.8	0
0.30,71.9	0
0.30,72.0	0
0.30,72.1	0
0.30,72.2	0
0.30,72.3	0
0.30,72.4	0
0.30,72.5	0
0.30,72.6	0
0.30,72.7	0
0.30,72.8	0
0.30,72.9	0
0.30,73.0	0
0.30,73.1	0
0.30,73.2	0
0.30,73.3	0
0.30,73.4	0
0.30,73.5	0
0.30,73.6	0
0.30,73.7	0
0.30,73.8	0
0.30,73.9	0
0.30,74.0	0
0.30,74.1	0
0.30,74.2	0
0.30,74.3	0
0.30,74.4	0
0.30,74.5	0
0.30,74.6	0
0.30,74.7	0

0.30,74.8	0
0.30,74.9	0
0.30,75.0	0
0.30,75.1	0
0.30,75.2	0
0.30,75.3	0
0.30,75.4	0
0.30,75.5	0
0.30,75.6	0
0.30,75.7	0
0.30,75.8	0
0.30,75.9	0
0.30,76.0	0
0.30,76.1	0
0.30,76.2	0
0.30,76.3	0
0.30,76.4	0
0.30,76.5	0
0.30,76.6	0
0.30,76.7	0
0.30,76.8	0
0.30,76.9	0
0.30,77.0	0
0.30,77.1	0
0.30,77.2	0
0.30,77.3	0
0.30,77.4	0
0.30,77.5	0
0.30,77.6	0
0.30,77.7	0
0.30,77.8	0
0.30,77.9	0
0.30,78.0	0
0.30,78.1	0
0.30,78.2	0
0.30,78.3	0
0.30,78.4	0
0.30,78.5	0
0.30,78.6	0
0.30,78.7	0
0.30,78.8	0
0.30,78.9	0

0.30,79.0	0
0.30,79.1	0
0.30,79.2	0
0.30,79.3	0
0.30,79.4	0
0.30,79.5	0
0.30,79.6	0
0.30,79.7	0
0.30,79.8	0
0.30,79.9	0
0.30,80.0	0
0.30,80.1	0
0.30,80.2	0
0.30,80.3	0
0.30,80.4	0
0.30,80.5	0
0.30,80.6	0
0.30,80.7	0
0.30,80.8	0
0.30,80.9	0
0.30,81.0	0
0.30,81.1	0
0.30,81.2	0
0.30,81.3	0
0.30,81.4	0
0.30,81.5	0
0.30,81.6	0
0.30,81.7	0
0.30,81.8	0
0.30,81.9	0
0.30,82.0	0
0.30,82.1	0
0.30,82.2	0
0.30,82.3	0
0.30,82.4	0
0.30,82.5	0
0.30,82.6	0
0.30,82.7	0
0.30,82.8	0
0.30,82.9	0
0.30,83.0	0
0.30,83.1	0

0.30,83.2	0
0.30,83.3	0
0.30,83.4	0
0.30,83.5	0
0.30,83.6	0
0.30,83.7	0
0.30,83.8	0
0.30,83.9	0
0.30,84.0	0
0.30,84.1	0
0.30,84.2	0
0.30,84.3	0
0.30,84.4	0
0.30,84.5	0
0.30,84.6	0
0.30,84.7	0
0.30,84.8	0
0.30,84.9	0
0.30,85.0	0
0.30,85.1	0
0.30,85.2	0
0.30,85.3	0
0.30,85.4	0
0.30,85.5	0
0.30,85.6	0
0.30,85.7	0
0.30,85.8	0
0.30,85.9	0
0.30,86.0	0
0.30,86.1	0
0.30,86.2	0
0.30,86.3	0
0.30,86.4	0
0.30,86.5	0
0.30,86.6	0
0.30,86.7	0
0.30,86.8	0
0.30,86.9	0
0.30,87.0	0
0.30,87.1	0
0.30,87.2	0
0.30,87.3	0

0.30,87.4	0
0.30,87.5	0
0.30,87.6	0
0.30,87.7	0
0.30,87.8	0
0.30,87.9	0
0.30,88.0	0
0.30,88.1	0
0.30,88.2	0
0.30,88.3	0
0.30,88.4	0
0.30,88.5	0
0.30,88.6	0
0.30,88.7	0
0.30,88.8	0
0.30,88.9	0
0.30,89.0	0
0.30,89.1	0
0.30,89.2	0
0.30,89.3	0
0.30,89.4	0
0.30,89.5	0
0.30,89.6	0
0.30,89.7	0
0.30,89.8	0
0.30,89.9	0
0.30,90.0	0
0.30,90.1	0
0.30,90.2	0
0.30,90.3	0
0.30,90.4	0
0.30,90.5	0
0.30,90.6	0
0.30,90.7	0
0.30,90.8	0
0.30,90.9	0
0.30,91.0	0
0.30,91.1	0
0.30,91.2	0
0.30,91.3	0
0.30,91.4	0
0.30,91.5	0

0.30,91.6	0
0.30,91.7	0
0.30,91.8	0
0.30,91.9	0
0.30,92.0	0
0.30,92.1	0
0.30,92.2	0
0.30,92.3	0
0.30,92.4	0
0.30,92.5	0
0.30,92.6	0
0.30,92.7	0
0.30,92.8	0
0.30,92.9	0
0.30,93.0	0
0.30,93.1	0
0.30,93.2	0
0.30,93.3	0
0.30,93.4	0
0.30,93.5	0
0.30,93.6	0
0.30,93.7	0
0.30,93.8	0
0.30,93.9	0
0.30,94.0	0
0.30,94.1	0
0.30,94.2	0
0.30,94.3	0
0.30,94.4	0
0.30,94.5	0
0.30,94.6	0
0.30,94.7	0
0.30,94.8	0
0.30,94.9	0
0.30,95.0	0
0.30,95.1	0
0.30,95.2	0
0.30,95.3	0
0.30,95.4	0
0.30,95.5	0
0.30,95.6	0
0.30,95.7	0

0.30,95.8	0
0.30,95.9	0
0.30,96.0	0
0.30,96.1	0
0.30,96.2	0
0.30,96.3	0
0.30,96.4	0
0.30,96.5	0
0.30,96.6	0
0.30,96.7	0
0.30,96.8	0
0.30,96.9	0
0.30,97.0	0
0.30,97.1	0
0.30,97.2	0
0.30,97.3	0
0.30,97.4	0
0.30,97.5	0
0.30,97.6	0
0.30,97.7	0
0.30,97.8	0
0.30,97.9	0
0.30,98.0	0
0.30,98.1	0
0.30,98.2	0
0.30,98.3	0
0.30,98.4	0
0.30,98.5	0
0.30,98.6	0
0.30,98.7	0
0.30,98.8	0
0.30,98.9	0
0.30,99.0	0
0.30,99.1	0
0.30,99.2	0
0.30,99.3	0
0.30,99.4	0
0.30,99.5	0
0.30,99.6	0
0.30,99.7	0
0.30,99.8	0
0.30,99.9	0

0.30,100.0	0
0.31,0.0	0
0.31,0.1	0
0.31,0.2	0
0.31,0.3	0
0.31,0.4	0
0.31,0.5	0
0.31,0.6	0
0.31,0.7	0
0.31,0.8	0
0.31,0.9	0
0.31,1.0	0
0.31,1.1	0
0.31,1.2	0
0.31,1.3	0
0.31,1.4	0
0.31,1.5	0
0.31,1.6	0
0.31,1.7	0
0.31,1.8	0
0.31,1.9	0
0.31,2.0	0
0.31,2.1	0
0.31,2.2	0
0.31,2.3	0
0.31,2.4	0
0.31,2.5	0
0.31,2.6	0
0.31,2.7	0
0.31,2.8	0
0.31,2.9	0
0.31,3.0	0
0.31,3.1	0
0.31,3.2	0
0.31,3.3	0
0.31,3.4	0
0.31,3.5	0
0.31,3.6	0
0.31,3.7	0
0.31,3.8	0
0.31,3.9	0
0.31,4.0	0

0.31,4.1	0
0.31,4.2	0
0.31,4.3	0
0.31,4.4	0
0.31,4.5	0
0.31,4.6	0
0.31,4.7	0
0.31,4.8	0
0.31,4.9	0
0.31,5.0	0
0.31,5.1	0
0.31,5.2	0
0.31,5.3	0
0.31,5.4	0
0.31,5.5	0
0.31,5.6	0
0.31,5.7	0
0.31,5.8	0
0.31,5.9	0
0.31,6.0	0
0.31,6.1	0
0.31,6.2	0
0.31,6.3	0
0.31,6.4	0
0.31,6.5	0
0.31,6.6	0
0.31,6.7	0
0.31,6.8	0
0.31,6.9	0
0.31,7.0	0
0.31,7.1	0
0.31,7.2	0
0.31,7.3	0
0.31,7.4	0
0.31,7.5	0
0.31,7.6	0
0.31,7.7	0
0.31,7.8	0
0.31,7.9	0
0.31,8.0	0
0.31,8.1	0
0.31,8.2	0

0.31,8.3	0
0.31,8.4	0
0.31,8.5	0
0.31,8.6	0
0.31,8.7	0
0.31,8.8	0
0.31,8.9	0
0.31,9.0	0
0.31,9.1	0
0.31,9.2	0
0.31,9.3	0
0.31,9.4	0
0.31,9.5	0
0.31,9.6	0
0.31,9.7	0
0.31,9.8	0
0.31,9.9	0
0.31,10.0	0
0.31,10.1	0
0.31,10.2	0
0.31,10.3	0
0.31,10.4	0
0.31,10.5	0
0.31,10.6	0
0.31,10.7	0
0.31,10.8	0
0.31,10.9	0
0.31,11.0	0
0.31,11.1	0
0.31,11.2	0
0.31,11.3	0
0.31,11.4	0
0.31,11.5	0
0.31,11.6	0
0.31,11.7	0
0.31,11.8	0
0.31,11.9	0
0.31,12.0	0
0.31,12.1	0
0.31,12.2	0
0.31,12.3	0
0.31,12.4	0

0.31,12.5	0
0.31,12.6	0
0.31,12.7	0
0.31,12.8	0
0.31,12.9	0
0.31,13.0	0
0.31,13.1	0
0.31,13.2	0
0.31,13.3	0
0.31,13.4	0
0.31,13.5	0
0.31,13.6	0
0.31,13.7	0
0.31,13.8	0
0.31,13.9	0
0.31,14.0	0
0.31,14.1	0
0.31,14.2	0
0.31,14.3	0
0.31,14.4	0
0.31,14.5	0
0.31,14.6	0
0.31,14.7	0
0.31,14.8	0
0.31,14.9	0
0.31,15.0	0
0.31,15.1	0
0.31,15.2	0
0.31,15.3	0
0.31,15.4	0
0.31,15.5	0
0.31,15.6	0
0.31,15.7	0
0.31,15.8	0
0.31,15.9	0
0.31,16.0	0
0.31,16.1	0
0.31,16.2	0
0.31,16.3	0
0.31,16.4	0
0.31,16.5	0
0.31,16.6	0

0.31,16.7	0
0.31,16.8	0
0.31,16.9	0
0.31,17.0	0
0.31,17.1	0
0.31,17.2	0
0.31,17.3	0
0.31,17.4	0
0.31,17.5	0
0.31,17.6	0
0.31,17.7	0
0.31,17.8	0
0.31,17.9	0
0.31,18.0	0
0.31,18.1	0
0.31,18.2	0
0.31,18.3	0
0.31,18.4	0
0.31,18.5	0
0.31,18.6	0
0.31,18.7	0
0.31,18.8	0
0.31,18.9	0
0.31,19.0	0
0.31,19.1	0
0.31,19.2	0
0.31,19.3	0
0.31,19.4	0
0.31,19.5	0
0.31,19.6	0
0.31,19.7	0
0.31,19.8	0
0.31,19.9	0
0.31,20.0	0
0.31,20.1	0
0.31,20.2	0
0.31,20.3	0
0.31,20.4	0
0.31,20.5	0
0.31,20.6	0
0.31,20.7	0
0.31,20.8	0

0.31,20.9	0
0.31,21.0	0
0.31,21.1	0
0.31,21.2	0
0.31,21.3	0
0.31,21.4	0
0.31,21.5	0
0.31,21.6	0
0.31,21.7	0
0.31,21.8	0
0.31,21.9	0
0.31,22.0	0
0.31,22.1	0
0.31,22.2	0
0.31,22.3	0
0.31,22.4	0
0.31,22.5	0
0.31,22.6	0
0.31,22.7	0
0.31,22.8	0
0.31,22.9	0
0.31,23.0	0
0.31,23.1	0
0.31,23.2	0
0.31,23.3	0
0.31,23.4	0
0.31,23.5	0
0.31,23.6	0
0.31,23.7	0
0.31,23.8	0
0.31,23.9	0
0.31,24.0	0
0.31,24.1	0
0.31,24.2	0
0.31,24.3	0
0.31,24.4	0
0.31,24.5	0
0.31,24.6	0
0.31,24.7	0
0.31,24.8	0
0.31,24.9	0
0.31,25.0	0

0.31,25.1	0
0.31,25.2	0
0.31,25.3	0
0.31,25.4	0
0.31,25.5	0
0.31,25.6	0
0.31,25.7	0
0.31,25.8	0
0.31,25.9	0
0.31,26.0	0
0.31,26.1	0
0.31,26.2	0
0.31,26.3	0
0.31,26.4	0
0.31,26.5	0
0.31,26.6	0
0.31,26.7	0
0.31,26.8	0
0.31,26.9	0
0.31,27.0	0
0.31,27.1	0
0.31,27.2	0
0.31,27.3	0
0.31,27.4	0
0.31,27.5	0
0.31,27.6	0
0.31,27.7	0
0.31,27.8	0
0.31,27.9	0
0.31,28.0	0
0.31,28.1	0
0.31,28.2	0
0.31,28.3	0
0.31,28.4	0
0.31,28.5	0
0.31,28.6	0
0.31,28.7	0
0.31,28.8	0
0.31,28.9	0
0.31,29.0	0
0.31,29.1	0
0.31,29.2	0

0.31,29.3	0
0.31,29.4	0
0.31,29.5	0
0.31,29.6	0
0.31,29.7	0
0.31,29.8	0
0.31,29.9	0
0.31,30.0	0
0.31,30.1	0
0.31,30.2	0
0.31,30.3	0
0.31,30.4	0
0.31,30.5	0
0.31,30.6	0
0.31,30.7	0
0.31,30.8	0
0.31,30.9	0
0.31,31.0	0
0.31,31.1	0
0.31,31.2	0
0.31,31.3	0
0.31,31.4	0
0.31,31.5	0
0.31,31.6	0
0.31,31.7	0
0.31,31.8	0
0.31,31.9	0
0.31,32.0	0
0.31,32.1	0
0.31,32.2	0
0.31,32.3	0
0.31,32.4	0
0.31,32.5	0
0.31,32.6	0
0.31,32.7	0
0.31,32.8	0
0.31,32.9	0
0.31,33.0	0
0.31,33.1	0
0.31,33.2	0
0.31,33.3	0
0.31,33.4	0

0.31,33.5	0
0.31,33.6	0
0.31,33.7	0
0.31,33.8	0
0.31,33.9	0
0.31,34.0	0
0.31,34.1	0
0.31,34.2	0
0.31,34.3	0
0.31,34.4	0
0.31,34.5	0
0.31,34.6	0
0.31,34.7	0
0.31,34.8	0
0.31,34.9	0
0.31,35.0	0
0.31,35.1	0
0.31,35.2	0
0.31,35.3	0
0.31,35.4	0
0.31,35.5	0
0.31,35.6	0
0.31,35.7	0
0.31,35.8	0
0.31,35.9	0
0.31,36.0	0
0.31,36.1	0
0.31,36.2	0
0.31,36.3	0
0.31,36.4	0
0.31,36.5	0
0.31,36.6	0
0.31,36.7	0
0.31,36.8	0
0.31,36.9	0
0.31,37.0	0
0.31,37.1	0
0.31,37.2	0
0.31,37.3	0
0.31,37.4	0
0.31,37.5	0
0.31,37.6	0

0.31,37.7	0
0.31,37.8	0
0.31,37.9	0
0.31,38.0	0
0.31,38.1	0
0.31,38.2	0
0.31,38.3	0
0.31,38.4	0
0.31,38.5	0
0.31,38.6	0
0.31,38.7	0
0.31,38.8	0
0.31,38.9	0
0.31,39.0	0
0.31,39.1	0
0.31,39.2	0
0.31,39.3	0
0.31,39.4	0
0.31,39.5	0
0.31,39.6	0
0.31,39.7	0
0.31,39.8	0
0.31,39.9	0
0.31,40.0	0
0.31,40.1	0
0.31,40.2	0
0.31,40.3	0
0.31,40.4	0
0.31,40.5	0
0.31,40.6	0
0.31,40.7	0
0.31,40.8	0
0.31,40.9	0
0.31,41.0	0
0.31,41.1	0
0.31,41.2	0
0.31,41.3	0
0.31,41.4	0
0.31,41.5	0
0.31,41.6	0
0.31,41.7	0
0.31,41.8	0

0.31,41.9	0
0.31,42.0	0
0.31,42.1	0
0.31,42.2	0
0.31,42.3	0
0.31,42.4	0
0.31,42.5	0
0.31,42.6	0
0.31,42.7	0
0.31,42.8	0
0.31,42.9	0
0.31,43.0	0
0.31,43.1	0
0.31,43.2	0
0.31,43.3	0
0.31,43.4	0
0.31,43.5	0
0.31,43.6	0
0.31,43.7	0
0.31,43.8	0
0.31,43.9	0
0.31,44.0	0
0.31,44.1	0
0.31,44.2	0
0.31,44.3	0
0.31,44.4	0
0.31,44.5	0
0.31,44.6	0
0.31,44.7	0
0.31,44.8	0
0.31,44.9	0
0.31,45.0	0
0.31,45.1	0
0.31,45.2	0
0.31,45.3	0
0.31,45.4	0
0.31,45.5	0
0.31,45.6	0
0.31,45.7	0
0.31,45.8	0
0.31,45.9	0
0.31,46.0	0

0.31,46.1	0
0.31,46.2	0
0.31,46.3	0
0.31,46.4	0
0.31,46.5	0
0.31,46.6	0
0.31,46.7	0
0.31,46.8	0
0.31,46.9	0
0.31,47.0	0
0.31,47.1	0
0.31,47.2	0
0.31,47.3	0
0.31,47.4	0
0.31,47.5	0
0.31,47.6	0
0.31,47.7	0
0.31,47.8	0
0.31,47.9	0
0.31,48.0	0
0.31,48.1	0
0.31,48.2	0
0.31,48.3	0
0.31,48.4	0
0.31,48.5	0
0.31,48.6	0
0.31,48.7	0
0.31,48.8	0
0.31,48.9	0
0.31,49.0	0
0.31,49.1	0
0.31,49.2	0
0.31,49.3	0
0.31,49.4	0
0.31,49.5	0
0.31,49.6	0
0.31,49.7	0
0.31,49.8	0
0.31,49.9	0
0.31,50.0	0
0.31,50.1	0
0.31,50.2	0

0.31,50.3	0
0.31,50.4	0
0.31,50.5	0
0.31,50.6	0
0.31,50.7	0
0.31,50.8	0
0.31,50.9	0
0.31,51.0	0
0.31,51.1	0
0.31,51.2	0
0.31,51.3	0
0.31,51.4	0
0.31,51.5	0
0.31,51.6	0
0.31,51.7	0
0.31,51.8	0
0.31,51.9	0
0.31,52.0	0
0.31,52.1	0
0.31,52.2	0
0.31,52.3	0
0.31,52.4	0
0.31,52.5	0
0.31,52.6	0
0.31,52.7	0
0.31,52.8	0
0.31,52.9	0
0.31,53.0	0
0.31,53.1	0
0.31,53.2	0
0.31,53.3	0
0.31,53.4	0
0.31,53.5	0
0.31,53.6	0
0.31,53.7	0
0.31,53.8	0
0.31,53.9	0
0.31,54.0	0
0.31,54.1	0
0.31,54.2	0
0.31,54.3	0
0.31,54.4	0

0.31,54.5	0
0.31,54.6	0
0.31,54.7	0
0.31,54.8	0
0.31,54.9	0
0.31,55.0	0
0.31,55.1	0
0.31,55.2	0
0.31,55.3	0
0.31,55.4	0
0.31,55.5	0
0.31,55.6	0
0.31,55.7	0
0.31,55.8	0
0.31,55.9	0
0.31,56.0	0
0.31,56.1	0
0.31,56.2	0
0.31,56.3	0
0.31,56.4	0
0.31,56.5	0
0.31,56.6	0
0.31,56.7	0
0.31,56.8	0
0.31,56.9	0
0.31,57.0	0
0.31,57.1	0
0.31,57.2	0
0.31,57.3	0
0.31,57.4	0
0.31,57.5	0
0.31,57.6	0
0.31,57.7	0
0.31,57.8	0
0.31,57.9	0
0.31,58.0	0
0.31,58.1	0
0.31,58.2	0
0.31,58.3	0
0.31,58.4	0
0.31,58.5	0
0.31,58.6	0

0.31,58.7	0
0.31,58.8	0
0.31,58.9	0
0.31,59.0	0
0.31,59.1	0
0.31,59.2	0
0.31,59.3	0
0.31,59.4	0
0.31,59.5	0
0.31,59.6	0
0.31,59.7	0
0.31,59.8	0
0.31,59.9	0
0.31,60.0	0
0.31,60.1	0
0.31,60.2	0
0.31,60.3	0
0.31,60.4	0
0.31,60.5	0
0.31,60.6	0
0.31,60.7	0
0.31,60.8	0
0.31,60.9	0
0.31,61.0	0
0.31,61.1	0
0.31,61.2	0
0.31,61.3	0
0.31,61.4	0
0.31,61.5	0
0.31,61.6	0
0.31,61.7	0
0.31,61.8	0
0.31,61.9	0
0.31,62.0	0
0.31,62.1	0
0.31,62.2	0
0.31,62.3	0
0.31,62.4	0
0.31,62.5	0
0.31,62.6	0
0.31,62.7	0
0.31,62.8	0

0.31,62.9	0
0.31,63.0	0
0.31,63.1	0
0.31,63.2	0
0.31,63.3	0
0.31,63.4	0
0.31,63.5	0
0.31,63.6	0
0.31,63.7	0
0.31,63.8	0
0.31,63.9	0
0.31,64.0	0
0.31,64.1	0
0.31,64.2	0
0.31,64.3	0
0.31,64.4	0
0.31,64.5	0
0.31,64.6	0
0.31,64.7	0
0.31,64.8	0
0.31,64.9	0
0.31,65.0	0
0.31,65.1	0
0.31,65.2	0
0.31,65.3	0
0.31,65.4	0
0.31,65.5	0
0.31,65.6	0
0.31,65.7	0
0.31,65.8	0
0.31,65.9	0
0.31,66.0	0
0.31,66.1	0
0.31,66.2	0
0.31,66.3	0
0.31,66.4	0
0.31,66.5	0
0.31,66.6	0
0.31,66.7	0
0.31,66.8	0
0.31,66.9	0
0.31,67.0	0

0.31,67.1	0
0.31,67.2	0
0.31,67.3	0
0.31,67.4	0
0.31,67.5	0
0.31,67.6	0
0.31,67.7	0
0.31,67.8	0
0.31,67.9	0
0.31,68.0	0
0.31,68.1	0
0.31,68.2	0
0.31,68.3	0
0.31,68.4	0
0.31,68.5	0
0.31,68.6	0
0.31,68.7	0
0.31,68.8	0
0.31,68.9	0
0.31,69.0	0
0.31,69.1	0
0.31,69.2	0
0.31,69.3	0
0.31,69.4	0
0.31,69.5	0
0.31,69.6	0
0.31,69.7	0
0.31,69.8	0
0.31,69.9	0
0.31,70.0	0
0.31,70.1	0
0.31,70.2	0
0.31,70.3	0
0.31,70.4	0
0.31,70.5	0
0.31,70.6	0
0.31,70.7	0
0.31,70.8	0
0.31,70.9	0
0.31,71.0	0
0.31,71.1	0
0.31,71.2	0

0.31,71.3	0
0.31,71.4	0
0.31,71.5	0
0.31,71.6	0
0.31,71.7	0
0.31,71.8	0
0.31,71.9	0
0.31,72.0	0
0.31,72.1	0
0.31,72.2	0
0.31,72.3	0
0.31,72.4	0
0.31,72.5	0
0.31,72.6	0
0.31,72.7	0
0.31,72.8	0
0.31,72.9	0
0.31,73.0	0
0.31,73.1	0
0.31,73.2	0
0.31,73.3	0
0.31,73.4	0
0.31,73.5	0
0.31,73.6	0
0.31,73.7	0
0.31,73.8	0
0.31,73.9	0
0.31,74.0	0
0.31,74.1	0
0.31,74.2	0
0.31,74.3	0
0.31,74.4	0
0.31,74.5	0
0.31,74.6	0
0.31,74.7	0
0.31,74.8	0
0.31,74.9	0
0.31,75.0	0
0.31,75.1	0
0.31,75.2	0
0.31,75.3	0
0.31,75.4	0

0.31,75.5	0
0.31,75.6	0
0.31,75.7	0
0.31,75.8	0
0.31,75.9	0
0.31,76.0	0
0.31,76.1	0
0.31,76.2	0
0.31,76.3	0
0.31,76.4	0
0.31,76.5	0
0.31,76.6	0
0.31,76.7	0
0.31,76.8	0
0.31,76.9	0
0.31,77.0	0
0.31,77.1	0
0.31,77.2	0
0.31,77.3	0
0.31,77.4	0
0.31,77.5	0
0.31,77.6	0
0.31,77.7	0
0.31,77.8	0
0.31,77.9	0
0.31,78.0	0
0.31,78.1	0
0.31,78.2	0
0.31,78.3	0
0.31,78.4	0
0.31,78.5	0
0.31,78.6	0
0.31,78.7	0
0.31,78.8	0
0.31,78.9	0
0.31,79.0	0
0.31,79.1	0
0.31,79.2	0
0.31,79.3	0
0.31,79.4	0
0.31,79.5	0
0.31,79.6	0

0.31,79.7	0
0.31,79.8	0
0.31,79.9	0
0.31,80.0	0
0.31,80.1	0
0.31,80.2	0
0.31,80.3	0
0.31,80.4	0
0.31,80.5	0
0.31,80.6	0
0.31,80.7	0
0.31,80.8	0
0.31,80.9	0
0.31,81.0	0
0.31,81.1	0
0.31,81.2	0
0.31,81.3	0
0.31,81.4	0
0.31,81.5	0
0.31,81.6	0
0.31,81.7	0
0.31,81.8	0
0.31,81.9	0
0.31,82.0	0
0.31,82.1	0
0.31,82.2	0
0.31,82.3	0
0.31,82.4	0
0.31,82.5	0
0.31,82.6	0
0.31,82.7	0
0.31,82.8	0
0.31,82.9	0
0.31,83.0	0
0.31,83.1	0
0.31,83.2	0
0.31,83.3	0
0.31,83.4	0
0.31,83.5	0
0.31,83.6	0
0.31,83.7	0
0.31,83.8	0

0.31,83.9	0
0.31,84.0	0
0.31,84.1	0
0.31,84.2	0
0.31,84.3	0
0.31,84.4	0
0.31,84.5	0
0.31,84.6	0
0.31,84.7	0
0.31,84.8	0
0.31,84.9	0
0.31,85.0	0
0.31,85.1	0
0.31,85.2	0
0.31,85.3	0
0.31,85.4	0
0.31,85.5	0
0.31,85.6	0
0.31,85.7	0
0.31,85.8	0
0.31,85.9	0
0.31,86.0	0
0.31,86.1	0
0.31,86.2	0
0.31,86.3	0
0.31,86.4	0
0.31,86.5	0
0.31,86.6	0
0.31,86.7	0
0.31,86.8	0
0.31,86.9	0
0.31,87.0	0
0.31,87.1	0
0.31,87.2	0
0.31,87.3	0
0.31,87.4	0
0.31,87.5	0
0.31,87.6	0
0.31,87.7	0
0.31,87.8	0
0.31,87.9	0
0.31,88.0	0

0.31,88.1	0
0.31,88.2	0
0.31,88.3	0
0.31,88.4	0
0.31,88.5	0
0.31,88.6	0
0.31,88.7	0
0.31,88.8	0
0.31,88.9	0
0.31,89.0	0
0.31,89.1	0
0.31,89.2	0
0.31,89.3	0
0.31,89.4	0
0.31,89.5	0
0.31,89.6	0
0.31,89.7	0
0.31,89.8	0
0.31,89.9	0
0.31,90.0	0
0.31,90.1	0
0.31,90.2	0
0.31,90.3	0
0.31,90.4	0
0.31,90.5	0
0.31,90.6	0
0.31,90.7	0
0.31,90.8	0
0.31,90.9	0
0.31,91.0	0
0.31,91.1	0
0.31,91.2	0
0.31,91.3	0
0.31,91.4	0
0.31,91.5	0
0.31,91.6	0
0.31,91.7	0
0.31,91.8	0
0.31,91.9	0
0.31,92.0	0
0.31,92.1	0
0.31,92.2	0

0.31,92.3	0
0.31,92.4	0
0.31,92.5	0
0.31,92.6	0
0.31,92.7	0
0.31,92.8	0
0.31,92.9	0
0.31,93.0	0
0.31,93.1	0
0.31,93.2	0
0.31,93.3	0
0.31,93.4	0
0.31,93.5	0
0.31,93.6	0
0.31,93.7	0
0.31,93.8	0
0.31,93.9	0
0.31,94.0	0
0.31,94.1	0
0.31,94.2	0
0.31,94.3	0
0.31,94.4	0
0.31,94.5	0
0.31,94.6	0
0.31,94.7	0
0.31,94.8	0
0.31,94.9	0
0.31,95.0	0
0.31,95.1	0
0.31,95.2	0
0.31,95.3	0
0.31,95.4	0
0.31,95.5	0
0.31,95.6	0
0.31,95.7	0
0.31,95.8	0
0.31,95.9	0
0.31,96.0	0
0.31,96.1	0
0.31,96.2	0
0.31,96.3	0
0.31,96.4	0

0.31,96.5	0
0.31,96.6	0
0.31,96.7	0
0.31,96.8	0
0.31,96.9	0
0.31,97.0	0
0.31,97.1	0
0.31,97.2	0
0.31,97.3	0
0.31,97.4	0
0.31,97.5	0
0.31,97.6	0
0.31,97.7	0
0.31,97.8	0
0.31,97.9	0
0.31,98.0	0
0.31,98.1	0
0.31,98.2	0
0.31,98.3	0
0.31,98.4	0
0.31,98.5	0
0.31,98.6	0
0.31,98.7	0
0.31,98.8	0
0.31,98.9	0
0.31,99.0	0
0.31,99.1	0
0.31,99.2	0
0.31,99.3	0
0.31,99.4	0
0.31,99.5	0
0.31,99.6	0
0.31,99.7	0
0.31,99.8	0
0.31,99.9	0
0.31,100.0	0
0.32,0.0	0
0.32,0.1	0
0.32,0.2	0
0.32,0.3	0
0.32,0.4	0
0.32,0.5	0

0.32,0.6	0
0.32,0.7	0
0.32,0.8	0
0.32,0.9	0
0.32,1.0	0
0.32,1.1	0
0.32,1.2	0
0.32,1.3	0
0.32,1.4	0
0.32,1.5	0
0.32,1.6	0
0.32,1.7	0
0.32,1.8	0
0.32,1.9	0
0.32,2.0	0
0.32,2.1	0
0.32,2.2	0
0.32,2.3	0
0.32,2.4	0
0.32,2.5	0
0.32,2.6	0
0.32,2.7	0
0.32,2.8	0
0.32,2.9	0
0.32,3.0	0
0.32,3.1	0
0.32,3.2	0
0.32,3.3	0
0.32,3.4	0
0.32,3.5	0
0.32,3.6	0
0.32,3.7	0
0.32,3.8	0
0.32,3.9	0
0.32,4.0	0
0.32,4.1	0
0.32,4.2	0
0.32,4.3	0
0.32,4.4	0
0.32,4.5	0
0.32,4.6	0
0.32,4.7	0

0.32,4.8	0
0.32,4.9	0
0.32,5.0	0
0.32,5.1	0
0.32,5.2	0
0.32,5.3	0
0.32,5.4	0
0.32,5.5	0
0.32,5.6	0
0.32,5.7	0
0.32,5.8	0
0.32,5.9	0
0.32,6.0	0
0.32,6.1	0
0.32,6.2	0
0.32,6.3	0
0.32,6.4	0
0.32,6.5	0
0.32,6.6	0
0.32,6.7	0
0.32,6.8	0
0.32,6.9	0
0.32,7.0	0
0.32,7.1	0
0.32,7.2	0
0.32,7.3	0
0.32,7.4	0
0.32,7.5	0
0.32,7.6	0
0.32,7.7	0
0.32,7.8	0
0.32,7.9	0
0.32,8.0	0
0.32,8.1	0
0.32,8.2	0
0.32,8.3	0
0.32,8.4	0
0.32,8.5	0
0.32,8.6	0
0.32,8.7	0
0.32,8.8	0
0.32,8.9	0

0.32,9.0	0
0.32,9.1	0
0.32,9.2	0
0.32,9.3	0
0.32,9.4	0
0.32,9.5	0
0.32,9.6	0
0.32,9.7	0
0.32,9.8	0
0.32,9.9	0
0.32,10.0	0
0.32,10.1	0
0.32,10.2	0
0.32,10.3	0
0.32,10.4	0
0.32,10.5	0
0.32,10.6	0
0.32,10.7	0
0.32,10.8	0
0.32,10.9	0
0.32,11.0	0
0.32,11.1	0
0.32,11.2	0
0.32,11.3	0
0.32,11.4	0
0.32,11.5	0
0.32,11.6	0
0.32,11.7	0
0.32,11.8	0
0.32,11.9	0
0.32,12.0	0
0.32,12.1	0
0.32,12.2	0
0.32,12.3	0
0.32,12.4	0
0.32,12.5	0
0.32,12.6	0
0.32,12.7	0
0.32,12.8	0
0.32,12.9	0
0.32,13.0	0
0.32,13.1	0

0.32,13.2	0
0.32,13.3	0
0.32,13.4	0
0.32,13.5	0
0.32,13.6	0
0.32,13.7	0
0.32,13.8	0
0.32,13.9	0
0.32,14.0	0
0.32,14.1	0
0.32,14.2	0
0.32,14.3	0
0.32,14.4	0
0.32,14.5	0
0.32,14.6	0
0.32,14.7	0
0.32,14.8	0
0.32,14.9	0
0.32,15.0	0
0.32,15.1	0
0.32,15.2	0
0.32,15.3	0
0.32,15.4	0
0.32,15.5	0
0.32,15.6	0
0.32,15.7	0
0.32,15.8	0
0.32,15.9	0
0.32,16.0	0
0.32,16.1	0
0.32,16.2	0
0.32,16.3	0
0.32,16.4	0
0.32,16.5	0
0.32,16.6	0
0.32,16.7	0
0.32,16.8	0
0.32,16.9	0
0.32,17.0	0
0.32,17.1	0
0.32,17.2	0
0.32,17.3	0

0.32,17.4	0
0.32,17.5	0
0.32,17.6	0
0.32,17.7	0
0.32,17.8	0
0.32,17.9	0
0.32,18.0	0
0.32,18.1	0
0.32,18.2	0
0.32,18.3	0
0.32,18.4	0
0.32,18.5	0
0.32,18.6	0
0.32,18.7	0
0.32,18.8	0
0.32,18.9	0
0.32,19.0	0
0.32,19.1	0
0.32,19.2	0
0.32,19.3	0
0.32,19.4	0
0.32,19.5	0
0.32,19.6	0
0.32,19.7	0
0.32,19.8	0
0.32,19.9	0
0.32,20.0	0
0.32,20.1	0
0.32,20.2	0
0.32,20.3	0
0.32,20.4	0
0.32,20.5	0
0.32,20.6	0
0.32,20.7	0
0.32,20.8	0
0.32,20.9	0
0.32,21.0	0
0.32,21.1	0
0.32,21.2	0
0.32,21.3	0
0.32,21.4	0
0.32,21.5	0

0.32,21.6	0
0.32,21.7	0
0.32,21.8	0
0.32,21.9	0
0.32,22.0	0
0.32,22.1	0
0.32,22.2	0
0.32,22.3	0
0.32,22.4	0
0.32,22.5	0
0.32,22.6	0
0.32,22.7	0
0.32,22.8	0
0.32,22.9	0
0.32,23.0	0
0.32,23.1	0
0.32,23.2	0
0.32,23.3	0
0.32,23.4	0
0.32,23.5	0
0.32,23.6	0
0.32,23.7	0
0.32,23.8	0
0.32,23.9	0
0.32,24.0	0
0.32,24.1	0
0.32,24.2	0
0.32,24.3	0
0.32,24.4	0
0.32,24.5	0
0.32,24.6	0
0.32,24.7	0
0.32,24.8	0
0.32,24.9	0
0.32,25.0	0
0.32,25.1	0
0.32,25.2	0
0.32,25.3	0
0.32,25.4	0
0.32,25.5	0
0.32,25.6	0
0.32,25.7	0

0.32,25.8	0
0.32,25.9	0
0.32,26.0	0
0.32,26.1	0
0.32,26.2	0
0.32,26.3	0
0.32,26.4	0
0.32,26.5	0
0.32,26.6	0
0.32,26.7	0
0.32,26.8	0
0.32,26.9	0
0.32,27.0	0
0.32,27.1	0
0.32,27.2	0
0.32,27.3	0
0.32,27.4	0
0.32,27.5	0
0.32,27.6	0
0.32,27.7	0
0.32,27.8	0
0.32,27.9	0
0.32,28.0	0
0.32,28.1	0
0.32,28.2	0
0.32,28.3	0
0.32,28.4	0
0.32,28.5	0
0.32,28.6	0
0.32,28.7	0
0.32,28.8	0
0.32,28.9	0
0.32,29.0	0
0.32,29.1	0
0.32,29.2	0
0.32,29.3	0
0.32,29.4	0
0.32,29.5	0
0.32,29.6	0
0.32,29.7	0
0.32,29.8	0
0.32,29.9	0

0.32,30.0	0
0.32,30.1	0
0.32,30.2	0
0.32,30.3	0
0.32,30.4	0
0.32,30.5	0
0.32,30.6	0
0.32,30.7	0
0.32,30.8	0
0.32,30.9	0
0.32,31.0	0
0.32,31.1	0
0.32,31.2	0
0.32,31.3	0
0.32,31.4	0
0.32,31.5	0
0.32,31.6	0
0.32,31.7	0
0.32,31.8	0
0.32,31.9	0
0.32,32.0	0
0.32,32.1	0
0.32,32.2	0
0.32,32.3	0
0.32,32.4	0
0.32,32.5	0
0.32,32.6	0
0.32,32.7	0
0.32,32.8	0
0.32,32.9	0
0.32,33.0	0
0.32,33.1	0
0.32,33.2	0
0.32,33.3	0
0.32,33.4	0
0.32,33.5	0
0.32,33.6	0
0.32,33.7	0
0.32,33.8	0
0.32,33.9	0
0.32,34.0	0
0.32,34.1	0

0.32,34.2	0
0.32,34.3	0
0.32,34.4	0
0.32,34.5	0
0.32,34.6	0
0.32,34.7	0
0.32,34.8	0
0.32,34.9	0
0.32,35.0	0
0.32,35.1	0
0.32,35.2	0
0.32,35.3	0
0.32,35.4	0
0.32,35.5	0
0.32,35.6	0
0.32,35.7	0
0.32,35.8	0
0.32,35.9	0
0.32,36.0	0
0.32,36.1	0
0.32,36.2	0
0.32,36.3	0
0.32,36.4	0
0.32,36.5	0
0.32,36.6	0
0.32,36.7	0
0.32,36.8	0
0.32,36.9	0
0.32,37.0	0
0.32,37.1	0
0.32,37.2	0
0.32,37.3	0
0.32,37.4	0
0.32,37.5	0
0.32,37.6	0
0.32,37.7	0
0.32,37.8	0
0.32,37.9	0
0.32,38.0	0
0.32,38.1	0
0.32,38.2	0
0.32,38.3	0

0.32,38.4	0
0.32,38.5	0
0.32,38.6	0
0.32,38.7	0
0.32,38.8	0
0.32,38.9	0
0.32,39.0	0
0.32,39.1	0
0.32,39.2	0
0.32,39.3	0
0.32,39.4	0
0.32,39.5	0
0.32,39.6	0
0.32,39.7	0
0.32,39.8	0
0.32,39.9	0
0.32,40.0	0
0.32,40.1	0
0.32,40.2	0
0.32,40.3	0
0.32,40.4	0
0.32,40.5	0
0.32,40.6	0
0.32,40.7	0
0.32,40.8	0
0.32,40.9	0
0.32,41.0	0
0.32,41.1	0
0.32,41.2	0
0.32,41.3	0
0.32,41.4	0
0.32,41.5	0
0.32,41.6	0
0.32,41.7	0
0.32,41.8	0
0.32,41.9	0
0.32,42.0	0
0.32,42.1	0
0.32,42.2	0
0.32,42.3	0
0.32,42.4	0
0.32,42.5	0

0.32,42.6	0
0.32,42.7	0
0.32,42.8	0
0.32,42.9	0
0.32,43.0	0
0.32,43.1	0
0.32,43.2	0
0.32,43.3	0
0.32,43.4	0
0.32,43.5	0
0.32,43.6	0
0.32,43.7	0
0.32,43.8	0
0.32,43.9	0
0.32,44.0	0
0.32,44.1	0
0.32,44.2	0
0.32,44.3	0
0.32,44.4	0
0.32,44.5	0
0.32,44.6	0
0.32,44.7	0
0.32,44.8	0
0.32,44.9	0
0.32,45.0	0
0.32,45.1	0
0.32,45.2	0
0.32,45.3	0
0.32,45.4	0
0.32,45.5	0
0.32,45.6	0
0.32,45.7	0
0.32,45.8	0
0.32,45.9	0
0.32,46.0	0
0.32,46.1	0
0.32,46.2	0
0.32,46.3	0
0.32,46.4	0
0.32,46.5	0
0.32,46.6	0
0.32,46.7	0

0.32,46.8	0
0.32,46.9	0
0.32,47.0	0
0.32,47.1	0
0.32,47.2	0
0.32,47.3	0
0.32,47.4	0
0.32,47.5	0
0.32,47.6	0
0.32,47.7	0
0.32,47.8	0
0.32,47.9	0
0.32,48.0	0
0.32,48.1	0
0.32,48.2	0
0.32,48.3	0
0.32,48.4	0
0.32,48.5	0
0.32,48.6	0
0.32,48.7	0
0.32,48.8	0
0.32,48.9	0
0.32,49.0	0
0.32,49.1	0
0.32,49.2	0
0.32,49.3	0
0.32,49.4	0
0.32,49.5	0
0.32,49.6	0
0.32,49.7	0
0.32,49.8	0
0.32,49.9	0
0.32,50.0	0
0.32,50.1	0
0.32,50.2	0
0.32,50.3	0
0.32,50.4	0
0.32,50.5	0
0.32,50.6	0
0.32,50.7	0
0.32,50.8	0
0.32,50.9	0

0.32,51.0	0
0.32,51.1	0
0.32,51.2	0
0.32,51.3	0
0.32,51.4	0
0.32,51.5	0
0.32,51.6	0
0.32,51.7	0
0.32,51.8	0
0.32,51.9	0
0.32,52.0	0
0.32,52.1	0
0.32,52.2	0
0.32,52.3	0
0.32,52.4	0
0.32,52.5	0
0.32,52.6	0
0.32,52.7	0
0.32,52.8	0
0.32,52.9	0
0.32,53.0	0
0.32,53.1	0
0.32,53.2	0
0.32,53.3	0
0.32,53.4	0
0.32,53.5	0
0.32,53.6	0
0.32,53.7	0
0.32,53.8	0
0.32,53.9	0
0.32,54.0	0
0.32,54.1	0
0.32,54.2	0
0.32,54.3	0
0.32,54.4	0
0.32,54.5	0
0.32,54.6	0
0.32,54.7	0
0.32,54.8	0
0.32,54.9	0
0.32,55.0	0
0.32,55.1	0

0.32,55.2	0
0.32,55.3	0
0.32,55.4	0
0.32,55.5	0
0.32,55.6	0
0.32,55.7	0
0.32,55.8	0
0.32,55.9	0
0.32,56.0	0
0.32,56.1	0
0.32,56.2	0
0.32,56.3	0
0.32,56.4	0
0.32,56.5	0
0.32,56.6	0
0.32,56.7	0
0.32,56.8	0
0.32,56.9	0
0.32,57.0	0
0.32,57.1	0
0.32,57.2	0
0.32,57.3	0
0.32,57.4	0
0.32,57.5	0
0.32,57.6	0
0.32,57.7	0
0.32,57.8	0
0.32,57.9	0
0.32,58.0	0
0.32,58.1	0
0.32,58.2	0
0.32,58.3	0
0.32,58.4	0
0.32,58.5	0
0.32,58.6	0
0.32,58.7	0
0.32,58.8	0
0.32,58.9	0
0.32,59.0	0
0.32,59.1	0
0.32,59.2	0
0.32,59.3	0

0.32,59.4	0
0.32,59.5	0
0.32,59.6	0
0.32,59.7	0
0.32,59.8	0
0.32,59.9	0
0.32,60.0	0
0.32,60.1	0
0.32,60.2	0
0.32,60.3	0
0.32,60.4	0
0.32,60.5	0
0.32,60.6	0
0.32,60.7	0
0.32,60.8	0
0.32,60.9	0
0.32,61.0	0
0.32,61.1	0
0.32,61.2	0
0.32,61.3	0
0.32,61.4	0
0.32,61.5	0
0.32,61.6	0
0.32,61.7	0
0.32,61.8	0
0.32,61.9	0
0.32,62.0	0
0.32,62.1	0
0.32,62.2	0
0.32,62.3	0
0.32,62.4	0
0.32,62.5	0
0.32,62.6	0
0.32,62.7	0
0.32,62.8	0
0.32,62.9	0
0.32,63.0	0
0.32,63.1	0
0.32,63.2	0
0.32,63.3	0
0.32,63.4	0
0.32,63.5	0

0.32,63.6	0
0.32,63.7	0
0.32,63.8	0
0.32,63.9	0
0.32,64.0	0
0.32,64.1	0
0.32,64.2	0
0.32,64.3	0
0.32,64.4	0
0.32,64.5	0
0.32,64.6	0
0.32,64.7	0
0.32,64.8	0
0.32,64.9	0
0.32,65.0	0
0.32,65.1	0
0.32,65.2	0
0.32,65.3	0
0.32,65.4	0
0.32,65.5	0
0.32,65.6	0
0.32,65.7	0
0.32,65.8	0
0.32,65.9	0
0.32,66.0	0
0.32,66.1	0
0.32,66.2	0
0.32,66.3	0
0.32,66.4	0
0.32,66.5	0
0.32,66.6	0
0.32,66.7	0
0.32,66.8	0
0.32,66.9	0
0.32,67.0	0
0.32,67.1	0
0.32,67.2	0
0.32,67.3	0
0.32,67.4	0
0.32,67.5	0
0.32,67.6	0
0.32,67.7	0

0.32,67.8	0
0.32,67.9	0
0.32,68.0	0
0.32,68.1	0
0.32,68.2	0
0.32,68.3	0
0.32,68.4	0
0.32,68.5	0
0.32,68.6	0
0.32,68.7	0
0.32,68.8	0
0.32,68.9	0
0.32,69.0	0
0.32,69.1	0
0.32,69.2	0
0.32,69.3	0
0.32,69.4	0
0.32,69.5	0
0.32,69.6	0
0.32,69.7	0
0.32,69.8	0
0.32,69.9	0
0.32,70.0	0
0.32,70.1	0
0.32,70.2	0
0.32,70.3	0
0.32,70.4	0
0.32,70.5	0
0.32,70.6	0
0.32,70.7	0
0.32,70.8	0
0.32,70.9	0
0.32,71.0	0
0.32,71.1	0
0.32,71.2	0
0.32,71.3	0
0.32,71.4	0
0.32,71.5	0
0.32,71.6	0
0.32,71.7	0
0.32,71.8	0
0.32,71.9	0

0.32,72.0	0
0.32,72.1	0
0.32,72.2	0
0.32,72.3	0
0.32,72.4	0
0.32,72.5	0
0.32,72.6	0
0.32,72.7	0
0.32,72.8	0
0.32,72.9	0
0.32,73.0	0
0.32,73.1	0
0.32,73.2	0
0.32,73.3	0
0.32,73.4	0
0.32,73.5	0
0.32,73.6	0
0.32,73.7	0
0.32,73.8	0
0.32,73.9	0
0.32,74.0	0
0.32,74.1	0
0.32,74.2	0
0.32,74.3	0
0.32,74.4	0
0.32,74.5	0
0.32,74.6	0
0.32,74.7	0
0.32,74.8	0
0.32,74.9	0
0.32,75.0	0
0.32,75.1	0
0.32,75.2	0
0.32,75.3	0
0.32,75.4	0
0.32,75.5	0
0.32,75.6	0
0.32,75.7	0
0.32,75.8	0
0.32,75.9	0
0.32,76.0	0
0.32,76.1	0

0.32,76.2	0
0.32,76.3	0
0.32,76.4	0
0.32,76.5	0
0.32,76.6	0
0.32,76.7	0
0.32,76.8	0
0.32,76.9	0
0.32,77.0	0
0.32,77.1	0
0.32,77.2	0
0.32,77.3	0
0.32,77.4	0
0.32,77.5	0
0.32,77.6	0
0.32,77.7	0
0.32,77.8	0
0.32,77.9	0
0.32,78.0	0
0.32,78.1	0
0.32,78.2	0
0.32,78.3	0
0.32,78.4	0
0.32,78.5	0
0.32,78.6	0
0.32,78.7	0
0.32,78.8	0
0.32,78.9	0
0.32,79.0	0
0.32,79.1	0
0.32,79.2	0
0.32,79.3	0
0.32,79.4	0
0.32,79.5	0
0.32,79.6	0
0.32,79.7	0
0.32,79.8	0
0.32,79.9	0
0.32,80.0	0
0.32,80.1	0
0.32,80.2	0
0.32,80.3	0

0.32,80.4	0
0.32,80.5	0
0.32,80.6	0
0.32,80.7	0
0.32,80.8	0
0.32,80.9	0
0.32,81.0	0
0.32,81.1	0
0.32,81.2	0
0.32,81.3	0
0.32,81.4	0
0.32,81.5	0
0.32,81.6	0
0.32,81.7	0
0.32,81.8	0
0.32,81.9	0
0.32,82.0	0
0.32,82.1	0
0.32,82.2	0
0.32,82.3	0
0.32,82.4	0
0.32,82.5	0
0.32,82.6	0
0.32,82.7	0
0.32,82.8	0
0.32,82.9	0
0.32,83.0	0
0.32,83.1	0
0.32,83.2	0
0.32,83.3	0
0.32,83.4	0
0.32,83.5	0
0.32,83.6	0
0.32,83.7	0
0.32,83.8	0
0.32,83.9	0
0.32,84.0	0
0.32,84.1	0
0.32,84.2	0
0.32,84.3	0
0.32,84.4	0
0.32,84.5	0

0.32,84.6	0
0.32,84.7	0
0.32,84.8	0
0.32,84.9	0
0.32,85.0	0
0.32,85.1	0
0.32,85.2	0
0.32,85.3	0
0.32,85.4	0
0.32,85.5	0
0.32,85.6	0
0.32,85.7	0
0.32,85.8	0
0.32,85.9	0
0.32,86.0	0
0.32,86.1	0
0.32,86.2	0
0.32,86.3	0
0.32,86.4	0
0.32,86.5	0
0.32,86.6	0
0.32,86.7	0
0.32,86.8	0
0.32,86.9	0
0.32,87.0	0
0.32,87.1	0
0.32,87.2	0
0.32,87.3	0
0.32,87.4	0
0.32,87.5	0
0.32,87.6	0
0.32,87.7	0
0.32,87.8	0
0.32,87.9	0
0.32,88.0	0
0.32,88.1	0
0.32,88.2	0
0.32,88.3	0
0.32,88.4	0
0.32,88.5	0
0.32,88.6	0
0.32,88.7	0

0.32,88.8	0
0.32,88.9	0
0.32,89.0	0
0.32,89.1	0
0.32,89.2	0
0.32,89.3	0
0.32,89.4	0
0.32,89.5	0
0.32,89.6	0
0.32,89.7	0
0.32,89.8	0
0.32,89.9	0
0.32,90.0	0
0.32,90.1	0
0.32,90.2	0
0.32,90.3	0
0.32,90.4	0
0.32,90.5	0
0.32,90.6	0
0.32,90.7	0
0.32,90.8	0
0.32,90.9	0
0.32,91.0	0
0.32,91.1	0
0.32,91.2	0
0.32,91.3	0
0.32,91.4	0
0.32,91.5	0
0.32,91.6	0
0.32,91.7	0
0.32,91.8	0
0.32,91.9	0
0.32,92.0	0
0.32,92.1	0
0.32,92.2	0
0.32,92.3	0
0.32,92.4	0
0.32,92.5	0
0.32,92.6	0
0.32,92.7	0
0.32,92.8	0
0.32,92.9	0

0.32,93.0	0
0.32,93.1	0
0.32,93.2	0
0.32,93.3	0
0.32,93.4	0
0.32,93.5	0
0.32,93.6	0
0.32,93.7	0
0.32,93.8	0
0.32,93.9	0
0.32,94.0	0
0.32,94.1	0
0.32,94.2	0
0.32,94.3	0
0.32,94.4	0
0.32,94.5	0
0.32,94.6	0
0.32,94.7	0
0.32,94.8	0
0.32,94.9	0
0.32,95.0	0
0.32,95.1	0
0.32,95.2	0
0.32,95.3	0
0.32,95.4	0
0.32,95.5	0
0.32,95.6	0
0.32,95.7	0
0.32,95.8	0
0.32,95.9	0
0.32,96.0	0
0.32,96.1	0
0.32,96.2	0
0.32,96.3	0
0.32,96.4	0
0.32,96.5	0
0.32,96.6	0
0.32,96.7	0
0.32,96.8	0
0.32,96.9	0
0.32,97.0	0
0.32,97.1	0

0.32,97.2	0
0.32,97.3	0
0.32,97.4	0
0.32,97.5	0
0.32,97.6	0
0.32,97.7	0
0.32,97.8	0
0.32,97.9	0
0.32,98.0	0
0.32,98.1	0
0.32,98.2	0
0.32,98.3	0
0.32,98.4	0
0.32,98.5	0
0.32,98.6	0
0.32,98.7	0
0.32,98.8	0
0.32,98.9	0
0.32,99.0	0
0.32,99.1	0
0.32,99.2	0
0.32,99.3	0
0.32,99.4	0
0.32,99.5	0
0.32,99.6	0
0.32,99.7	0
0.32,99.8	0
0.32,99.9	0
0.32,100.0	0
0.33,0.0	0
0.33,0.1	0
0.33,0.2	0
0.33,0.3	0
0.33,0.4	0
0.33,0.5	0
0.33,0.6	0
0.33,0.7	0
0.33,0.8	0
0.33,0.9	0
0.33,1.0	0
0.33,1.1	0
0.33,1.2	0

0.33,1.3	0
0.33,1.4	0
0.33,1.5	0
0.33,1.6	0
0.33,1.7	0
0.33,1.8	0
0.33,1.9	0
0.33,2.0	0
0.33,2.1	0
0.33,2.2	0
0.33,2.3	0
0.33,2.4	0
0.33,2.5	0
0.33,2.6	0
0.33,2.7	0
0.33,2.8	0
0.33,2.9	0
0.33,3.0	0
0.33,3.1	0
0.33,3.2	0
0.33,3.3	0
0.33,3.4	0
0.33,3.5	0
0.33,3.6	0
0.33,3.7	0
0.33,3.8	0
0.33,3.9	0
0.33,4.0	0
0.33,4.1	0
0.33,4.2	0
0.33,4.3	0
0.33,4.4	0
0.33,4.5	0
0.33,4.6	0
0.33,4.7	0
0.33,4.8	0
0.33,4.9	0
0.33,5.0	0
0.33,5.1	0
0.33,5.2	0
0.33,5.3	0
0.33,5.4	0

0.33,5.5	0
0.33,5.6	0
0.33,5.7	0
0.33,5.8	0
0.33,5.9	0
0.33,6.0	0
0.33,6.1	0
0.33,6.2	0
0.33,6.3	0
0.33,6.4	0
0.33,6.5	0
0.33,6.6	0
0.33,6.7	0
0.33,6.8	0
0.33,6.9	0
0.33,7.0	0
0.33,7.1	0
0.33,7.2	0
0.33,7.3	0
0.33,7.4	0
0.33,7.5	0
0.33,7.6	0
0.33,7.7	0
0.33,7.8	0
0.33,7.9	0
0.33,8.0	0
0.33,8.1	0
0.33,8.2	0
0.33,8.3	0
0.33,8.4	0
0.33,8.5	0
0.33,8.6	0
0.33,8.7	0
0.33,8.8	0
0.33,8.9	0
0.33,9.0	0
0.33,9.1	0
0.33,9.2	0
0.33,9.3	0
0.33,9.4	0
0.33,9.5	0
0.33,9.6	0

0.33,9.7	0
0.33,9.8	0
0.33,9.9	0
0.33,10.0	0
0.33,10.1	0
0.33,10.2	0
0.33,10.3	0
0.33,10.4	0
0.33,10.5	0
0.33,10.6	0
0.33,10.7	0
0.33,10.8	0
0.33,10.9	0
0.33,11.0	0
0.33,11.1	0
0.33,11.2	0
0.33,11.3	0
0.33,11.4	0
0.33,11.5	0
0.33,11.6	0
0.33,11.7	0
0.33,11.8	0
0.33,11.9	0
0.33,12.0	0
0.33,12.1	0
0.33,12.2	0
0.33,12.3	0
0.33,12.4	0
0.33,12.5	0
0.33,12.6	0
0.33,12.7	0
0.33,12.8	0
0.33,12.9	0
0.33,13.0	0
0.33,13.1	0
0.33,13.2	0
0.33,13.3	0
0.33,13.4	0
0.33,13.5	0
0.33,13.6	0
0.33,13.7	0
0.33,13.8	0

0.33,13.9	0
0.33,14.0	0
0.33,14.1	0
0.33,14.2	0
0.33,14.3	0
0.33,14.4	0
0.33,14.5	0
0.33,14.6	0
0.33,14.7	0
0.33,14.8	0
0.33,14.9	0
0.33,15.0	0
0.33,15.1	0
0.33,15.2	0
0.33,15.3	0
0.33,15.4	0
0.33,15.5	0
0.33,15.6	0
0.33,15.7	0
0.33,15.8	0
0.33,15.9	0
0.33,16.0	0
0.33,16.1	0
0.33,16.2	0
0.33,16.3	0
0.33,16.4	0
0.33,16.5	0
0.33,16.6	0
0.33,16.7	0
0.33,16.8	0
0.33,16.9	0
0.33,17.0	0
0.33,17.1	0
0.33,17.2	0
0.33,17.3	0
0.33,17.4	0
0.33,17.5	0
0.33,17.6	0
0.33,17.7	0
0.33,17.8	0
0.33,17.9	0
0.33,18.0	0

0.33,18.1	0
0.33,18.2	0
0.33,18.3	0
0.33,18.4	0
0.33,18.5	0
0.33,18.6	0
0.33,18.7	0
0.33,18.8	0
0.33,18.9	0
0.33,19.0	0
0.33,19.1	0
0.33,19.2	0
0.33,19.3	0
0.33,19.4	0
0.33,19.5	0
0.33,19.6	0
0.33,19.7	0
0.33,19.8	0
0.33,19.9	0
0.33,20.0	0
0.33,20.1	0
0.33,20.2	0
0.33,20.3	0
0.33,20.4	0
0.33,20.5	0
0.33,20.6	0
0.33,20.7	0
0.33,20.8	0
0.33,20.9	0
0.33,21.0	0
0.33,21.1	0
0.33,21.2	0
0.33,21.3	0
0.33,21.4	0
0.33,21.5	0
0.33,21.6	0
0.33,21.7	0
0.33,21.8	0
0.33,21.9	0
0.33,22.0	0
0.33,22.1	0
0.33,22.2	0

0.33,22.3	0
0.33,22.4	0
0.33,22.5	0
0.33,22.6	0
0.33,22.7	0
0.33,22.8	0
0.33,22.9	0
0.33,23.0	0
0.33,23.1	0
0.33,23.2	0
0.33,23.3	0
0.33,23.4	0
0.33,23.5	0
0.33,23.6	0
0.33,23.7	0
0.33,23.8	0
0.33,23.9	0
0.33,24.0	0
0.33,24.1	0
0.33,24.2	0
0.33,24.3	0
0.33,24.4	0
0.33,24.5	0
0.33,24.6	0
0.33,24.7	0
0.33,24.8	0
0.33,24.9	0
0.33,25.0	0
0.33,25.1	0
0.33,25.2	0
0.33,25.3	0
0.33,25.4	0
0.33,25.5	0
0.33,25.6	0
0.33,25.7	0
0.33,25.8	0
0.33,25.9	0
0.33,26.0	0
0.33,26.1	0
0.33,26.2	0
0.33,26.3	0
0.33,26.4	0

0.33,26.5	0
0.33,26.6	0
0.33,26.7	0
0.33,26.8	0
0.33,26.9	0
0.33,27.0	0
0.33,27.1	0
0.33,27.2	0
0.33,27.3	0
0.33,27.4	0
0.33,27.5	0
0.33,27.6	0
0.33,27.7	0
0.33,27.8	0
0.33,27.9	0
0.33,28.0	0
0.33,28.1	0
0.33,28.2	0
0.33,28.3	0
0.33,28.4	0
0.33,28.5	0
0.33,28.6	0
0.33,28.7	0
0.33,28.8	0
0.33,28.9	0
0.33,29.0	0
0.33,29.1	0
0.33,29.2	0
0.33,29.3	0
0.33,29.4	0
0.33,29.5	0
0.33,29.6	0
0.33,29.7	0
0.33,29.8	0
0.33,29.9	0
0.33,30.0	0
0.33,30.1	0
0.33,30.2	0
0.33,30.3	0
0.33,30.4	0
0.33,30.5	0
0.33,30.6	0

0.33,30.7	0
0.33,30.8	0
0.33,30.9	0
0.33,31.0	0
0.33,31.1	0
0.33,31.2	0
0.33,31.3	0
0.33,31.4	0
0.33,31.5	0
0.33,31.6	0
0.33,31.7	0
0.33,31.8	0
0.33,31.9	0
0.33,32.0	0
0.33,32.1	0
0.33,32.2	0
0.33,32.3	0
0.33,32.4	0
0.33,32.5	0
0.33,32.6	0
0.33,32.7	0
0.33,32.8	0
0.33,32.9	0
0.33,33.0	0
0.33,33.1	0
0.33,33.2	0
0.33,33.3	0
0.33,33.4	0
0.33,33.5	0
0.33,33.6	0
0.33,33.7	0
0.33,33.8	0
0.33,33.9	0
0.33,34.0	0
0.33,34.1	0
0.33,34.2	0
0.33,34.3	0
0.33,34.4	0
0.33,34.5	0
0.33,34.6	0
0.33,34.7	0
0.33,34.8	0

0.33,34.9	0
0.33,35.0	0
0.33,35.1	0
0.33,35.2	0
0.33,35.3	0
0.33,35.4	0
0.33,35.5	0
0.33,35.6	0
0.33,35.7	0
0.33,35.8	0
0.33,35.9	0
0.33,36.0	0
0.33,36.1	0
0.33,36.2	0
0.33,36.3	0
0.33,36.4	0
0.33,36.5	0
0.33,36.6	0
0.33,36.7	0
0.33,36.8	0
0.33,36.9	0
0.33,37.0	0
0.33,37.1	0
0.33,37.2	0
0.33,37.3	0
0.33,37.4	0
0.33,37.5	0
0.33,37.6	0
0.33,37.7	0
0.33,37.8	0
0.33,37.9	0
0.33,38.0	0
0.33,38.1	0
0.33,38.2	0
0.33,38.3	0
0.33,38.4	0
0.33,38.5	0
0.33,38.6	0
0.33,38.7	0
0.33,38.8	0
0.33,38.9	0
0.33,39.0	0

0.33,39.1	0
0.33,39.2	0
0.33,39.3	0
0.33,39.4	0
0.33,39.5	0
0.33,39.6	0
0.33,39.7	0
0.33,39.8	0
0.33,39.9	0
0.33,40.0	0
0.33,40.1	0
0.33,40.2	0
0.33,40.3	0
0.33,40.4	0
0.33,40.5	0
0.33,40.6	0
0.33,40.7	0
0.33,40.8	0
0.33,40.9	0
0.33,41.0	0
0.33,41.1	0
0.33,41.2	0
0.33,41.3	0
0.33,41.4	0
0.33,41.5	0
0.33,41.6	0
0.33,41.7	0
0.33,41.8	0
0.33,41.9	0
0.33,42.0	0
0.33,42.1	0
0.33,42.2	0
0.33,42.3	0
0.33,42.4	0
0.33,42.5	0
0.33,42.6	0
0.33,42.7	0
0.33,42.8	0
0.33,42.9	0
0.33,43.0	0
0.33,43.1	0
0.33,43.2	0

0.33,43.3	0
0.33,43.4	0
0.33,43.5	0
0.33,43.6	0
0.33,43.7	0
0.33,43.8	0
0.33,43.9	0
0.33,44.0	0
0.33,44.1	0
0.33,44.2	0
0.33,44.3	0
0.33,44.4	0
0.33,44.5	0
0.33,44.6	0
0.33,44.7	0
0.33,44.8	0
0.33,44.9	0
0.33,45.0	0
0.33,45.1	0
0.33,45.2	0
0.33,45.3	0
0.33,45.4	0
0.33,45.5	0
0.33,45.6	0
0.33,45.7	0
0.33,45.8	0
0.33,45.9	0
0.33,46.0	0
0.33,46.1	0
0.33,46.2	0
0.33,46.3	0
0.33,46.4	0
0.33,46.5	0
0.33,46.6	0
0.33,46.7	0
0.33,46.8	0
0.33,46.9	0
0.33,47.0	0
0.33,47.1	0
0.33,47.2	0
0.33,47.3	0
0.33,47.4	0

0.33,47.5	0
0.33,47.6	0
0.33,47.7	0
0.33,47.8	0
0.33,47.9	0
0.33,48.0	0
0.33,48.1	0
0.33,48.2	0
0.33,48.3	0
0.33,48.4	0
0.33,48.5	0
0.33,48.6	0
0.33,48.7	0
0.33,48.8	0
0.33,48.9	0
0.33,49.0	0
0.33,49.1	0
0.33,49.2	0
0.33,49.3	0
0.33,49.4	0
0.33,49.5	0
0.33,49.6	0
0.33,49.7	0
0.33,49.8	0
0.33,49.9	0
0.33,50.0	0
0.33,50.1	0
0.33,50.2	0
0.33,50.3	0
0.33,50.4	0
0.33,50.5	0
0.33,50.6	0
0.33,50.7	0
0.33,50.8	0
0.33,50.9	0
0.33,51.0	0
0.33,51.1	0
0.33,51.2	0
0.33,51.3	0
0.33,51.4	0
0.33,51.5	0
0.33,51.6	0

0.33,51.7	0
0.33,51.8	0
0.33,51.9	0
0.33,52.0	0
0.33,52.1	0
0.33,52.2	0
0.33,52.3	0
0.33,52.4	0
0.33,52.5	0
0.33,52.6	0
0.33,52.7	0
0.33,52.8	0
0.33,52.9	0
0.33,53.0	0
0.33,53.1	0
0.33,53.2	0
0.33,53.3	0
0.33,53.4	0
0.33,53.5	0
0.33,53.6	0
0.33,53.7	0
0.33,53.8	0
0.33,53.9	0
0.33,54.0	0
0.33,54.1	0
0.33,54.2	0
0.33,54.3	0
0.33,54.4	0
0.33,54.5	0
0.33,54.6	0
0.33,54.7	0
0.33,54.8	0
0.33,54.9	0
0.33,55.0	0
0.33,55.1	0
0.33,55.2	0
0.33,55.3	0
0.33,55.4	0
0.33,55.5	0
0.33,55.6	0
0.33,55.7	0
0.33,55.8	0

0.33,55.9	0
0.33,56.0	0
0.33,56.1	0
0.33,56.2	0
0.33,56.3	0
0.33,56.4	0
0.33,56.5	0
0.33,56.6	0
0.33,56.7	0
0.33,56.8	0
0.33,56.9	0
0.33,57.0	0
0.33,57.1	0
0.33,57.2	0
0.33,57.3	0
0.33,57.4	0
0.33,57.5	0
0.33,57.6	0
0.33,57.7	0
0.33,57.8	0
0.33,57.9	0
0.33,58.0	0
0.33,58.1	0
0.33,58.2	0
0.33,58.3	0
0.33,58.4	0
0.33,58.5	0
0.33,58.6	0
0.33,58.7	0
0.33,58.8	0
0.33,58.9	0
0.33,59.0	0
0.33,59.1	0
0.33,59.2	0
0.33,59.3	0
0.33,59.4	0
0.33,59.5	0
0.33,59.6	0
0.33,59.7	0
0.33,59.8	0
0.33,59.9	0
0.33,60.0	0

0.33,60.1	0
0.33,60.2	0
0.33,60.3	0
0.33,60.4	0
0.33,60.5	0
0.33,60.6	0
0.33,60.7	0
0.33,60.8	0
0.33,60.9	0
0.33,61.0	0
0.33,61.1	0
0.33,61.2	0
0.33,61.3	0
0.33,61.4	0
0.33,61.5	0
0.33,61.6	0
0.33,61.7	0
0.33,61.8	0
0.33,61.9	0
0.33,62.0	0
0.33,62.1	0
0.33,62.2	0
0.33,62.3	0
0.33,62.4	0
0.33,62.5	0
0.33,62.6	0
0.33,62.7	0
0.33,62.8	0
0.33,62.9	0
0.33,63.0	0
0.33,63.1	0
0.33,63.2	0
0.33,63.3	0
0.33,63.4	0
0.33,63.5	0
0.33,63.6	0
0.33,63.7	0
0.33,63.8	0
0.33,63.9	0
0.33,64.0	0
0.33,64.1	0
0.33,64.2	0

0.33,64.3	0
0.33,64.4	0
0.33,64.5	0
0.33,64.6	0
0.33,64.7	0
0.33,64.8	0
0.33,64.9	0
0.33,65.0	0
0.33,65.1	0
0.33,65.2	0
0.33,65.3	0
0.33,65.4	0
0.33,65.5	0
0.33,65.6	0
0.33,65.7	0
0.33,65.8	0
0.33,65.9	0
0.33,66.0	0
0.33,66.1	0
0.33,66.2	0
0.33,66.3	0
0.33,66.4	0
0.33,66.5	0
0.33,66.6	0
0.33,66.7	0
0.33,66.8	0
0.33,66.9	0
0.33,67.0	0
0.33,67.1	0
0.33,67.2	0
0.33,67.3	0
0.33,67.4	0
0.33,67.5	0
0.33,67.6	0
0.33,67.7	0
0.33,67.8	0
0.33,67.9	0
0.33,68.0	0
0.33,68.1	0
0.33,68.2	0
0.33,68.3	0
0.33,68.4	0

0.33,68.5	0
0.33,68.6	0
0.33,68.7	0
0.33,68.8	0
0.33,68.9	0
0.33,69.0	0
0.33,69.1	0
0.33,69.2	0
0.33,69.3	0
0.33,69.4	0
0.33,69.5	0
0.33,69.6	0
0.33,69.7	0
0.33,69.8	0
0.33,69.9	0
0.33,70.0	0
0.33,70.1	0
0.33,70.2	0
0.33,70.3	0
0.33,70.4	0
0.33,70.5	0
0.33,70.6	0
0.33,70.7	0
0.33,70.8	0
0.33,70.9	0
0.33,71.0	0
0.33,71.1	0
0.33,71.2	0
0.33,71.3	0
0.33,71.4	0
0.33,71.5	0
0.33,71.6	0
0.33,71.7	0
0.33,71.8	0
0.33,71.9	0
0.33,72.0	0
0.33,72.1	0
0.33,72.2	0
0.33,72.3	0
0.33,72.4	0
0.33,72.5	0
0.33,72.6	0

0.33,72.7	0
0.33,72.8	0
0.33,72.9	0
0.33,73.0	0
0.33,73.1	0
0.33,73.2	0
0.33,73.3	0
0.33,73.4	0
0.33,73.5	0
0.33,73.6	0
0.33,73.7	0
0.33,73.8	0
0.33,73.9	0
0.33,74.0	0
0.33,74.1	0
0.33,74.2	0
0.33,74.3	0
0.33,74.4	0
0.33,74.5	0
0.33,74.6	0
0.33,74.7	0
0.33,74.8	0
0.33,74.9	0
0.33,75.0	0
0.33,75.1	0
0.33,75.2	0
0.33,75.3	0
0.33,75.4	0
0.33,75.5	0
0.33,75.6	0
0.33,75.7	0
0.33,75.8	0
0.33,75.9	0
0.33,76.0	0
0.33,76.1	0
0.33,76.2	0
0.33,76.3	0
0.33,76.4	0
0.33,76.5	0
0.33,76.6	0
0.33,76.7	0
0.33,76.8	0

0.33,76.9	0
0.33,77.0	0
0.33,77.1	0
0.33,77.2	0
0.33,77.3	0
0.33,77.4	0
0.33,77.5	0
0.33,77.6	0
0.33,77.7	0
0.33,77.8	0
0.33,77.9	0
0.33,78.0	0
0.33,78.1	0
0.33,78.2	0
0.33,78.3	0
0.33,78.4	0
0.33,78.5	0
0.33,78.6	0
0.33,78.7	0
0.33,78.8	0
0.33,78.9	0
0.33,79.0	0
0.33,79.1	0
0.33,79.2	0
0.33,79.3	0
0.33,79.4	0
0.33,79.5	0
0.33,79.6	0
0.33,79.7	0
0.33,79.8	0
0.33,79.9	0
0.33,80.0	0
0.33,80.1	0
0.33,80.2	0
0.33,80.3	0
0.33,80.4	0
0.33,80.5	0
0.33,80.6	0
0.33,80.7	0
0.33,80.8	0
0.33,80.9	0
0.33,81.0	0

0.33,81.1	0
0.33,81.2	0
0.33,81.3	0
0.33,81.4	0
0.33,81.5	0
0.33,81.6	0
0.33,81.7	0
0.33,81.8	0
0.33,81.9	0
0.33,82.0	0
0.33,82.1	0
0.33,82.2	0
0.33,82.3	0
0.33,82.4	0
0.33,82.5	0
0.33,82.6	0
0.33,82.7	0
0.33,82.8	0
0.33,82.9	0
0.33,83.0	0
0.33,83.1	0
0.33,83.2	0
0.33,83.3	0
0.33,83.4	0
0.33,83.5	0
0.33,83.6	0
0.33,83.7	0
0.33,83.8	0
0.33,83.9	0
0.33,84.0	0
0.33,84.1	0
0.33,84.2	0
0.33,84.3	0
0.33,84.4	0
0.33,84.5	0
0.33,84.6	0
0.33,84.7	0
0.33,84.8	0
0.33,84.9	0
0.33,85.0	0
0.33,85.1	0
0.33,85.2	0

0.33,85.3	0
0.33,85.4	0
0.33,85.5	0
0.33,85.6	0
0.33,85.7	0
0.33,85.8	0
0.33,85.9	0
0.33,86.0	0
0.33,86.1	0
0.33,86.2	0
0.33,86.3	0
0.33,86.4	0
0.33,86.5	0
0.33,86.6	0
0.33,86.7	0
0.33,86.8	0
0.33,86.9	0
0.33,87.0	0
0.33,87.1	0
0.33,87.2	0
0.33,87.3	0
0.33,87.4	0
0.33,87.5	0
0.33,87.6	0
0.33,87.7	0
0.33,87.8	0
0.33,87.9	0
0.33,88.0	0
0.33,88.1	0
0.33,88.2	0
0.33,88.3	0
0.33,88.4	0
0.33,88.5	0
0.33,88.6	0
0.33,88.7	0
0.33,88.8	0
0.33,88.9	0
0.33,89.0	0
0.33,89.1	0
0.33,89.2	0
0.33,89.3	0
0.33,89.4	0

0.33,89.5	0
0.33,89.6	0
0.33,89.7	0
0.33,89.8	0
0.33,89.9	0
0.33,90.0	0
0.33,90.1	0
0.33,90.2	0
0.33,90.3	0
0.33,90.4	0
0.33,90.5	0
0.33,90.6	0
0.33,90.7	0
0.33,90.8	0
0.33,90.9	0
0.33,91.0	0
0.33,91.1	0
0.33,91.2	0
0.33,91.3	0
0.33,91.4	0
0.33,91.5	0
0.33,91.6	0
0.33,91.7	0
0.33,91.8	0
0.33,91.9	0
0.33,92.0	0
0.33,92.1	0
0.33,92.2	0
0.33,92.3	0
0.33,92.4	0
0.33,92.5	0
0.33,92.6	0
0.33,92.7	0
0.33,92.8	0
0.33,92.9	0
0.33,93.0	0
0.33,93.1	0
0.33,93.2	0
0.33,93.3	0
0.33,93.4	0
0.33,93.5	0
0.33,93.6	0

0.33,93.7	0
0.33,93.8	0
0.33,93.9	0
0.33,94.0	0
0.33,94.1	0
0.33,94.2	0
0.33,94.3	0
0.33,94.4	0
0.33,94.5	0
0.33,94.6	0
0.33,94.7	0
0.33,94.8	0
0.33,94.9	0
0.33,95.0	0
0.33,95.1	0
0.33,95.2	0
0.33,95.3	0
0.33,95.4	0
0.33,95.5	0
0.33,95.6	0
0.33,95.7	0
0.33,95.8	0
0.33,95.9	0
0.33,96.0	0
0.33,96.1	0
0.33,96.2	0
0.33,96.3	0
0.33,96.4	0
0.33,96.5	0
0.33,96.6	0
0.33,96.7	0
0.33,96.8	0
0.33,96.9	0
0.33,97.0	0
0.33,97.1	0
0.33,97.2	0
0.33,97.3	0
0.33,97.4	0
0.33,97.5	0
0.33,97.6	0
0.33,97.7	0
0.33,97.8	0

0.33,97.9	0
0.33,98.0	0
0.33,98.1	0
0.33,98.2	0
0.33,98.3	0
0.33,98.4	0
0.33,98.5	0
0.33,98.6	0
0.33,98.7	0
0.33,98.8	0
0.33,98.9	0
0.33,99.0	0
0.33,99.1	0
0.33,99.2	0
0.33,99.3	0
0.33,99.4	0
0.33,99.5	0
0.33,99.6	0
0.33,99.7	0
0.33,99.8	0
0.33,99.9	0
0.33,100.0	0
0.34,0.0	0
0.34,0.1	0
0.34,0.2	0
0.34,0.3	0
0.34,0.4	0
0.34,0.5	0
0.34,0.6	0
0.34,0.7	0
0.34,0.8	0
0.34,0.9	0
0.34,1.0	0
0.34,1.1	0
0.34,1.2	0
0.34,1.3	0
0.34,1.4	0
0.34,1.5	0
0.34,1.6	0
0.34,1.7	0
0.34,1.8	0
0.34,1.9	0

0.34,2.0	0
0.34,2.1	0
0.34,2.2	0
0.34,2.3	0
0.34,2.4	0
0.34,2.5	0
0.34,2.6	0
0.34,2.7	0
0.34,2.8	0
0.34,2.9	0
0.34,3.0	0
0.34,3.1	0
0.34,3.2	0
0.34,3.3	0
0.34,3.4	0
0.34,3.5	0
0.34,3.6	0
0.34,3.7	0
0.34,3.8	0
0.34,3.9	0
0.34,4.0	0
0.34,4.1	0
0.34,4.2	0
0.34,4.3	0
0.34,4.4	0
0.34,4.5	0
0.34,4.6	0
0.34,4.7	0
0.34,4.8	0
0.34,4.9	0
0.34,5.0	0
0.34,5.1	0
0.34,5.2	0
0.34,5.3	0
0.34,5.4	0
0.34,5.5	0
0.34,5.6	0
0.34,5.7	0
0.34,5.8	0
0.34,5.9	0
0.34,6.0	0
0.34,6.1	0

0.34,6.2	0
0.34,6.3	0
0.34,6.4	0
0.34,6.5	0
0.34,6.6	0
0.34,6.7	0
0.34,6.8	0
0.34,6.9	0
0.34,7.0	0
0.34,7.1	0
0.34,7.2	0
0.34,7.3	0
0.34,7.4	0
0.34,7.5	0
0.34,7.6	0
0.34,7.7	0
0.34,7.8	0
0.34,7.9	0
0.34,8.0	0
0.34,8.1	0
0.34,8.2	0
0.34,8.3	0
0.34,8.4	0
0.34,8.5	0
0.34,8.6	0
0.34,8.7	0
0.34,8.8	0
0.34,8.9	0
0.34,9.0	0
0.34,9.1	0
0.34,9.2	0
0.34,9.3	0
0.34,9.4	0
0.34,9.5	0
0.34,9.6	0
0.34,9.7	0
0.34,9.8	0
0.34,9.9	0
0.34,10.0	0
0.34,10.1	0
0.34,10.2	0
0.34,10.3	0

0.34,10.4	0
0.34,10.5	0
0.34,10.6	0
0.34,10.7	0
0.34,10.8	0
0.34,10.9	0
0.34,11.0	0
0.34,11.1	0
0.34,11.2	0
0.34,11.3	0
0.34,11.4	0
0.34,11.5	0
0.34,11.6	0
0.34,11.7	0
0.34,11.8	0
0.34,11.9	0
0.34,12.0	0
0.34,12.1	0
0.34,12.2	0
0.34,12.3	0
0.34,12.4	0
0.34,12.5	0
0.34,12.6	0
0.34,12.7	0
0.34,12.8	0
0.34,12.9	0
0.34,13.0	0
0.34,13.1	0
0.34,13.2	0
0.34,13.3	0
0.34,13.4	0
0.34,13.5	0
0.34,13.6	0
0.34,13.7	0
0.34,13.8	0
0.34,13.9	0
0.34,14.0	0
0.34,14.1	0
0.34,14.2	0
0.34,14.3	0
0.34,14.4	0
0.34,14.5	0

0.34,14.6	0
0.34,14.7	0
0.34,14.8	0
0.34,14.9	0
0.34,15.0	0
0.34,15.1	0
0.34,15.2	0
0.34,15.3	0
0.34,15.4	0
0.34,15.5	0
0.34,15.6	0
0.34,15.7	0
0.34,15.8	0
0.34,15.9	0
0.34,16.0	0
0.34,16.1	0
0.34,16.2	0
0.34,16.3	0
0.34,16.4	0
0.34,16.5	0
0.34,16.6	0
0.34,16.7	0
0.34,16.8	0
0.34,16.9	0
0.34,17.0	0
0.34,17.1	0
0.34,17.2	0
0.34,17.3	0
0.34,17.4	0
0.34,17.5	0
0.34,17.6	0
0.34,17.7	0
0.34,17.8	0
0.34,17.9	0
0.34,18.0	0
0.34,18.1	0
0.34,18.2	0
0.34,18.3	0
0.34,18.4	0
0.34,18.5	0
0.34,18.6	0
0.34,18.7	0

0.34,18.8	0
0.34,18.9	0
0.34,19.0	0
0.34,19.1	0
0.34,19.2	0
0.34,19.3	0
0.34,19.4	0
0.34,19.5	0
0.34,19.6	0
0.34,19.7	0
0.34,19.8	0
0.34,19.9	0
0.34,20.0	0
0.34,20.1	0
0.34,20.2	0
0.34,20.3	0
0.34,20.4	0
0.34,20.5	0
0.34,20.6	0
0.34,20.7	0
0.34,20.8	0
0.34,20.9	0
0.34,21.0	0
0.34,21.1	0
0.34,21.2	0
0.34,21.3	0
0.34,21.4	0
0.34,21.5	0
0.34,21.6	0
0.34,21.7	0
0.34,21.8	0
0.34,21.9	0
0.34,22.0	0
0.34,22.1	0
0.34,22.2	0
0.34,22.3	0
0.34,22.4	0
0.34,22.5	0
0.34,22.6	0
0.34,22.7	0
0.34,22.8	0
0.34,22.9	0

0.34,23.0	0
0.34,23.1	0
0.34,23.2	0
0.34,23.3	0
0.34,23.4	0
0.34,23.5	0
0.34,23.6	0
0.34,23.7	0
0.34,23.8	0
0.34,23.9	0
0.34,24.0	0
0.34,24.1	0
0.34,24.2	0
0.34,24.3	0
0.34,24.4	0
0.34,24.5	0
0.34,24.6	0
0.34,24.7	0
0.34,24.8	0
0.34,24.9	0
0.34,25.0	0
0.34,25.1	0
0.34,25.2	0
0.34,25.3	0
0.34,25.4	0
0.34,25.5	0
0.34,25.6	0
0.34,25.7	0
0.34,25.8	0
0.34,25.9	0
0.34,26.0	0
0.34,26.1	0
0.34,26.2	0
0.34,26.3	0
0.34,26.4	0
0.34,26.5	0
0.34,26.6	0
0.34,26.7	0
0.34,26.8	0
0.34,26.9	0
0.34,27.0	0
0.34,27.1	0

0.34,27.2	0
0.34,27.3	0
0.34,27.4	0
0.34,27.5	0
0.34,27.6	0
0.34,27.7	0
0.34,27.8	0
0.34,27.9	0
0.34,28.0	0
0.34,28.1	0
0.34,28.2	0
0.34,28.3	0
0.34,28.4	0
0.34,28.5	0
0.34,28.6	0
0.34,28.7	0
0.34,28.8	0
0.34,28.9	0
0.34,29.0	0
0.34,29.1	0
0.34,29.2	0
0.34,29.3	0
0.34,29.4	0
0.34,29.5	0
0.34,29.6	0
0.34,29.7	0
0.34,29.8	0
0.34,29.9	0
0.34,30.0	0
0.34,30.1	0
0.34,30.2	0
0.34,30.3	0
0.34,30.4	0
0.34,30.5	0
0.34,30.6	0
0.34,30.7	0
0.34,30.8	0
0.34,30.9	0
0.34,31.0	0
0.34,31.1	0
0.34,31.2	0
0.34,31.3	0

0.34,31.4	0
0.34,31.5	0
0.34,31.6	0
0.34,31.7	0
0.34,31.8	0
0.34,31.9	0
0.34,32.0	0
0.34,32.1	0
0.34,32.2	0
0.34,32.3	0
0.34,32.4	0
0.34,32.5	0
0.34,32.6	0
0.34,32.7	0
0.34,32.8	0
0.34,32.9	0
0.34,33.0	0
0.34,33.1	0
0.34,33.2	0
0.34,33.3	0
0.34,33.4	0
0.34,33.5	0
0.34,33.6	0
0.34,33.7	0
0.34,33.8	0
0.34,33.9	0
0.34,34.0	0
0.34,34.1	0
0.34,34.2	0
0.34,34.3	0
0.34,34.4	0
0.34,34.5	0
0.34,34.6	0
0.34,34.7	0
0.34,34.8	0
0.34,34.9	0
0.34,35.0	0
0.34,35.1	0
0.34,35.2	0
0.34,35.3	0
0.34,35.4	0
0.34,35.5	0

0.34,35.6	0
0.34,35.7	0
0.34,35.8	0
0.34,35.9	0
0.34,36.0	0
0.34,36.1	0
0.34,36.2	0
0.34,36.3	0
0.34,36.4	0
0.34,36.5	0
0.34,36.6	0
0.34,36.7	0
0.34,36.8	0
0.34,36.9	0
0.34,37.0	0
0.34,37.1	0
0.34,37.2	0
0.34,37.3	0
0.34,37.4	0
0.34,37.5	0
0.34,37.6	0
0.34,37.7	0
0.34,37.8	0
0.34,37.9	0
0.34,38.0	0
0.34,38.1	0
0.34,38.2	0
0.34,38.3	0
0.34,38.4	0
0.34,38.5	0
0.34,38.6	0
0.34,38.7	0
0.34,38.8	0
0.34,38.9	0
0.34,39.0	0
0.34,39.1	0
0.34,39.2	0
0.34,39.3	0
0.34,39.4	0
0.34,39.5	0
0.34,39.6	0
0.34,39.7	0

0.34,39.8	0
0.34,39.9	0
0.34,40.0	0
0.34,40.1	0
0.34,40.2	0
0.34,40.3	0
0.34,40.4	0
0.34,40.5	0
0.34,40.6	0
0.34,40.7	0
0.34,40.8	0
0.34,40.9	0
0.34,41.0	0
0.34,41.1	0
0.34,41.2	0
0.34,41.3	0
0.34,41.4	0
0.34,41.5	0
0.34,41.6	0
0.34,41.7	0
0.34,41.8	0
0.34,41.9	0
0.34,42.0	0
0.34,42.1	0
0.34,42.2	0
0.34,42.3	0
0.34,42.4	0
0.34,42.5	0
0.34,42.6	0
0.34,42.7	0
0.34,42.8	0
0.34,42.9	0
0.34,43.0	0
0.34,43.1	0
0.34,43.2	0
0.34,43.3	0
0.34,43.4	0
0.34,43.5	0
0.34,43.6	0
0.34,43.7	0
0.34,43.8	0
0.34,43.9	0

0.34,44.0	0
0.34,44.1	0
0.34,44.2	0
0.34,44.3	0
0.34,44.4	0
0.34,44.5	0
0.34,44.6	0
0.34,44.7	0
0.34,44.8	0
0.34,44.9	0
0.34,45.0	0
0.34,45.1	0
0.34,45.2	0
0.34,45.3	0
0.34,45.4	0
0.34,45.5	0
0.34,45.6	0
0.34,45.7	0
0.34,45.8	0
0.34,45.9	0
0.34,46.0	0
0.34,46.1	0
0.34,46.2	0
0.34,46.3	0
0.34,46.4	0
0.34,46.5	0
0.34,46.6	0
0.34,46.7	0
0.34,46.8	0
0.34,46.9	0
0.34,47.0	0
0.34,47.1	0
0.34,47.2	0
0.34,47.3	0
0.34,47.4	0
0.34,47.5	0
0.34,47.6	0
0.34,47.7	0
0.34,47.8	0
0.34,47.9	0
0.34,48.0	0
0.34,48.1	0

0.34,48.2	0
0.34,48.3	0
0.34,48.4	0
0.34,48.5	0
0.34,48.6	0
0.34,48.7	0
0.34,48.8	0
0.34,48.9	0
0.34,49.0	0
0.34,49.1	0
0.34,49.2	0
0.34,49.3	0
0.34,49.4	0
0.34,49.5	0
0.34,49.6	0
0.34,49.7	0
0.34,49.8	0
0.34,49.9	0
0.34,50.0	0
0.34,50.1	0
0.34,50.2	0
0.34,50.3	0
0.34,50.4	0
0.34,50.5	0
0.34,50.6	0
0.34,50.7	0
0.34,50.8	0
0.34,50.9	0
0.34,51.0	0
0.34,51.1	0
0.34,51.2	0
0.34,51.3	0
0.34,51.4	0
0.34,51.5	0
0.34,51.6	0
0.34,51.7	0
0.34,51.8	0
0.34,51.9	0
0.34,52.0	0
0.34,52.1	0
0.34,52.2	0
0.34,52.3	0

0.34,52.4	0
0.34,52.5	0
0.34,52.6	0
0.34,52.7	0
0.34,52.8	0
0.34,52.9	0
0.34,53.0	0
0.34,53.1	0
0.34,53.2	0
0.34,53.3	0
0.34,53.4	0
0.34,53.5	0
0.34,53.6	0
0.34,53.7	0
0.34,53.8	0
0.34,53.9	0
0.34,54.0	0
0.34,54.1	0
0.34,54.2	0
0.34,54.3	0
0.34,54.4	0
0.34,54.5	0
0.34,54.6	0
0.34,54.7	0
0.34,54.8	0
0.34,54.9	0
0.34,55.0	0
0.34,55.1	0
0.34,55.2	0
0.34,55.3	0
0.34,55.4	0
0.34,55.5	0
0.34,55.6	0
0.34,55.7	0
0.34,55.8	0
0.34,55.9	0
0.34,56.0	0
0.34,56.1	0
0.34,56.2	0
0.34,56.3	0
0.34,56.4	0
0.34,56.5	0

0.34,56.6	0
0.34,56.7	0
0.34,56.8	0
0.34,56.9	0
0.34,57.0	0
0.34,57.1	0
0.34,57.2	0
0.34,57.3	0
0.34,57.4	0
0.34,57.5	0
0.34,57.6	0
0.34,57.7	0
0.34,57.8	0
0.34,57.9	0
0.34,58.0	0
0.34,58.1	0
0.34,58.2	0
0.34,58.3	0
0.34,58.4	0
0.34,58.5	0
0.34,58.6	0
0.34,58.7	0
0.34,58.8	0
0.34,58.9	0
0.34,59.0	0
0.34,59.1	0
0.34,59.2	0
0.34,59.3	0
0.34,59.4	0
0.34,59.5	0
0.34,59.6	0
0.34,59.7	0
0.34,59.8	0
0.34,59.9	0
0.34,60.0	0
0.34,60.1	0
0.34,60.2	0
0.34,60.3	0
0.34,60.4	0
0.34,60.5	0
0.34,60.6	0
0.34,60.7	0

0.34,60.8	0
0.34,60.9	0
0.34,61.0	0
0.34,61.1	0
0.34,61.2	0
0.34,61.3	0
0.34,61.4	0
0.34,61.5	0
0.34,61.6	0
0.34,61.7	0
0.34,61.8	0
0.34,61.9	0
0.34,62.0	0
0.34,62.1	0
0.34,62.2	0
0.34,62.3	0
0.34,62.4	0
0.34,62.5	0
0.34,62.6	0
0.34,62.7	0
0.34,62.8	0
0.34,62.9	0
0.34,63.0	0
0.34,63.1	0
0.34,63.2	0
0.34,63.3	0
0.34,63.4	0
0.34,63.5	0
0.34,63.6	0
0.34,63.7	0
0.34,63.8	0
0.34,63.9	0
0.34,64.0	0
0.34,64.1	0
0.34,64.2	0
0.34,64.3	0
0.34,64.4	0
0.34,64.5	0
0.34,64.6	0
0.34,64.7	0
0.34,64.8	0
0.34,64.9	0

0.34,65.0	0
0.34,65.1	0
0.34,65.2	0
0.34,65.3	0
0.34,65.4	0
0.34,65.5	0
0.34,65.6	0
0.34,65.7	0
0.34,65.8	0
0.34,65.9	0
0.34,66.0	0
0.34,66.1	0
0.34,66.2	0
0.34,66.3	0
0.34,66.4	0
0.34,66.5	0
0.34,66.6	0
0.34,66.7	0
0.34,66.8	0
0.34,66.9	0
0.34,67.0	0
0.34,67.1	0
0.34,67.2	0
0.34,67.3	0
0.34,67.4	0
0.34,67.5	0
0.34,67.6	0
0.34,67.7	0
0.34,67.8	0
0.34,67.9	0
0.34,68.0	0
0.34,68.1	0
0.34,68.2	0
0.34,68.3	0
0.34,68.4	0
0.34,68.5	0
0.34,68.6	0
0.34,68.7	0
0.34,68.8	0
0.34,68.9	0
0.34,69.0	0
0.34,69.1	0

0.34,69.2	0
0.34,69.3	0
0.34,69.4	0
0.34,69.5	0
0.34,69.6	0
0.34,69.7	0
0.34,69.8	0
0.34,69.9	0
0.34,70.0	0
0.34,70.1	0
0.34,70.2	0
0.34,70.3	0
0.34,70.4	0
0.34,70.5	0
0.34,70.6	0
0.34,70.7	0
0.34,70.8	0
0.34,70.9	0
0.34,71.0	0
0.34,71.1	0
0.34,71.2	0
0.34,71.3	0
0.34,71.4	0
0.34,71.5	0
0.34,71.6	0
0.34,71.7	0
0.34,71.8	0
0.34,71.9	0
0.34,72.0	0
0.34,72.1	0
0.34,72.2	0
0.34,72.3	0
0.34,72.4	0
0.34,72.5	0
0.34,72.6	0
0.34,72.7	0
0.34,72.8	0
0.34,72.9	0
0.34,73.0	0
0.34,73.1	0
0.34,73.2	0
0.34,73.3	0

0.34,73.4	0
0.34,73.5	0
0.34,73.6	0
0.34,73.7	0
0.34,73.8	0
0.34,73.9	0
0.34,74.0	0
0.34,74.1	0
0.34,74.2	0
0.34,74.3	0
0.34,74.4	0
0.34,74.5	0
0.34,74.6	0
0.34,74.7	0
0.34,74.8	0
0.34,74.9	0
0.34,75.0	0
0.34,75.1	0
0.34,75.2	0
0.34,75.3	0
0.34,75.4	0
0.34,75.5	0
0.34,75.6	0
0.34,75.7	0
0.34,75.8	0
0.34,75.9	0
0.34,76.0	0
0.34,76.1	0
0.34,76.2	0
0.34,76.3	0
0.34,76.4	0
0.34,76.5	0
0.34,76.6	0
0.34,76.7	0
0.34,76.8	0
0.34,76.9	0
0.34,77.0	0
0.34,77.1	0
0.34,77.2	0
0.34,77.3	0
0.34,77.4	0
0.34,77.5	0

0.34,77.6	0
0.34,77.7	0
0.34,77.8	0
0.34,77.9	0
0.34,78.0	0
0.34,78.1	0
0.34,78.2	0
0.34,78.3	0
0.34,78.4	0
0.34,78.5	0
0.34,78.6	0
0.34,78.7	0
0.34,78.8	0
0.34,78.9	0
0.34,79.0	0
0.34,79.1	0
0.34,79.2	0
0.34,79.3	0
0.34,79.4	0
0.34,79.5	0
0.34,79.6	0
0.34,79.7	0
0.34,79.8	0
0.34,79.9	0
0.34,80.0	0
0.34,80.1	0
0.34,80.2	0
0.34,80.3	0
0.34,80.4	0
0.34,80.5	0
0.34,80.6	0
0.34,80.7	0
0.34,80.8	0
0.34,80.9	0
0.34,81.0	0
0.34,81.1	0
0.34,81.2	0
0.34,81.3	0
0.34,81.4	0
0.34,81.5	0
0.34,81.6	0
0.34,81.7	0

0.34,81.8	0
0.34,81.9	0
0.34,82.0	0
0.34,82.1	0
0.34,82.2	0
0.34,82.3	0
0.34,82.4	0
0.34,82.5	0
0.34,82.6	0
0.34,82.7	0
0.34,82.8	0
0.34,82.9	0
0.34,83.0	0
0.34,83.1	0
0.34,83.2	0
0.34,83.3	0
0.34,83.4	0
0.34,83.5	0
0.34,83.6	0
0.34,83.7	0
0.34,83.8	0
0.34,83.9	0
0.34,84.0	0
0.34,84.1	0
0.34,84.2	0
0.34,84.3	0
0.34,84.4	0
0.34,84.5	0
0.34,84.6	0
0.34,84.7	0
0.34,84.8	0
0.34,84.9	0
0.34,85.0	0
0.34,85.1	0
0.34,85.2	0
0.34,85.3	0
0.34,85.4	0
0.34,85.5	0
0.34,85.6	0
0.34,85.7	0
0.34,85.8	0
0.34,85.9	0

0.34,86.0	0
0.34,86.1	0
0.34,86.2	0
0.34,86.3	0
0.34,86.4	0
0.34,86.5	0
0.34,86.6	0
0.34,86.7	0
0.34,86.8	0
0.34,86.9	0
0.34,87.0	0
0.34,87.1	0
0.34,87.2	0
0.34,87.3	0
0.34,87.4	0
0.34,87.5	0
0.34,87.6	0
0.34,87.7	0
0.34,87.8	0
0.34,87.9	0
0.34,88.0	0
0.34,88.1	0
0.34,88.2	0
0.34,88.3	0
0.34,88.4	0
0.34,88.5	0
0.34,88.6	0
0.34,88.7	0
0.34,88.8	0
0.34,88.9	0
0.34,89.0	0
0.34,89.1	0
0.34,89.2	0
0.34,89.3	0
0.34,89.4	0
0.34,89.5	0
0.34,89.6	0
0.34,89.7	0
0.34,89.8	0
0.34,89.9	0
0.34,90.0	0
0.34,90.1	0

0.34,90.2	0
0.34,90.3	0
0.34,90.4	0
0.34,90.5	0
0.34,90.6	0
0.34,90.7	0
0.34,90.8	0
0.34,90.9	0
0.34,91.0	0
0.34,91.1	0
0.34,91.2	0
0.34,91.3	0
0.34,91.4	0
0.34,91.5	0
0.34,91.6	0
0.34,91.7	0
0.34,91.8	0
0.34,91.9	0
0.34,92.0	0
0.34,92.1	0
0.34,92.2	0
0.34,92.3	0
0.34,92.4	0
0.34,92.5	0
0.34,92.6	0
0.34,92.7	0
0.34,92.8	0
0.34,92.9	0
0.34,93.0	0
0.34,93.1	0
0.34,93.2	0
0.34,93.3	0
0.34,93.4	0
0.34,93.5	0
0.34,93.6	0
0.34,93.7	0
0.34,93.8	0
0.34,93.9	0
0.34,94.0	0
0.34,94.1	0
0.34,94.2	0
0.34,94.3	0

0.34,94.4	0
0.34,94.5	0
0.34,94.6	0
0.34,94.7	0
0.34,94.8	0
0.34,94.9	0
0.34,95.0	0
0.34,95.1	0
0.34,95.2	0
0.34,95.3	0
0.34,95.4	0
0.34,95.5	0
0.34,95.6	0
0.34,95.7	0
0.34,95.8	0
0.34,95.9	0
0.34,96.0	0
0.34,96.1	0
0.34,96.2	0
0.34,96.3	0
0.34,96.4	0
0.34,96.5	0
0.34,96.6	0
0.34,96.7	0
0.34,96.8	0
0.34,96.9	0
0.34,97.0	0
0.34,97.1	0
0.34,97.2	0
0.34,97.3	0
0.34,97.4	0
0.34,97.5	0
0.34,97.6	0
0.34,97.7	0
0.34,97.8	0
0.34,97.9	0
0.34,98.0	0
0.34,98.1	0
0.34,98.2	0
0.34,98.3	0
0.34,98.4	0
0.34,98.5	0

0.34,98.6	0
0.34,98.7	0
0.34,98.8	0
0.34,98.9	0
0.34,99.0	0
0.34,99.1	0
0.34,99.2	0
0.34,99.3	0
0.34,99.4	0
0.34,99.5	0
0.34,99.6	0
0.34,99.7	0
0.34,99.8	0
0.34,99.9	0
0.34,100.0	0
0.35,0.0	0
0.35,0.1	0
0.35,0.2	0
0.35,0.3	0
0.35,0.4	0
0.35,0.5	0
0.35,0.6	0
0.35,0.7	0
0.35,0.8	0
0.35,0.9	0
0.35,1.0	0
0.35,1.1	0
0.35,1.2	0
0.35,1.3	0
0.35,1.4	0
0.35,1.5	0
0.35,1.6	0
0.35,1.7	0
0.35,1.8	0
0.35,1.9	0
0.35,2.0	0
0.35,2.1	0
0.35,2.2	0
0.35,2.3	0
0.35,2.4	0
0.35,2.5	0
0.35,2.6	0

0.35,2.7	0
0.35,2.8	0
0.35,2.9	0
0.35,3.0	0
0.35,3.1	0
0.35,3.2	0
0.35,3.3	0
0.35,3.4	0
0.35,3.5	0
0.35,3.6	0
0.35,3.7	0
0.35,3.8	0
0.35,3.9	0
0.35,4.0	0
0.35,4.1	0
0.35,4.2	0
0.35,4.3	0
0.35,4.4	0
0.35,4.5	0
0.35,4.6	0
0.35,4.7	0
0.35,4.8	0
0.35,4.9	0
0.35,5.0	0
0.35,5.1	0
0.35,5.2	0
0.35,5.3	0
0.35,5.4	0
0.35,5.5	0
0.35,5.6	0
0.35,5.7	0
0.35,5.8	0
0.35,5.9	0
0.35,6.0	0
0.35,6.1	0
0.35,6.2	0
0.35,6.3	0
0.35,6.4	0
0.35,6.5	0
0.35,6.6	0
0.35,6.7	0
0.35,6.8	0

0.35,6.9	0
0.35,7.0	0
0.35,7.1	0
0.35,7.2	0
0.35,7.3	0
0.35,7.4	0
0.35,7.5	0
0.35,7.6	0
0.35,7.7	0
0.35,7.8	0
0.35,7.9	0
0.35,8.0	0
0.35,8.1	0
0.35,8.2	0
0.35,8.3	0
0.35,8.4	0
0.35,8.5	0
0.35,8.6	0
0.35,8.7	0
0.35,8.8	0
0.35,8.9	0
0.35,9.0	0
0.35,9.1	0
0.35,9.2	0
0.35,9.3	0
0.35,9.4	0
0.35,9.5	0
0.35,9.6	0
0.35,9.7	0
0.35,9.8	0
0.35,9.9	0
0.35,10.0	0
0.35,10.1	0
0.35,10.2	0
0.35,10.3	0
0.35,10.4	0
0.35,10.5	0
0.35,10.6	0
0.35,10.7	0
0.35,10.8	0
0.35,10.9	0
0.35,11.0	0

0.35,11.1	0
0.35,11.2	0
0.35,11.3	0
0.35,11.4	0
0.35,11.5	0
0.35,11.6	0
0.35,11.7	0
0.35,11.8	0
0.35,11.9	0
0.35,12.0	0
0.35,12.1	0
0.35,12.2	0
0.35,12.3	0
0.35,12.4	0
0.35,12.5	0
0.35,12.6	0
0.35,12.7	0
0.35,12.8	0
0.35,12.9	0
0.35,13.0	0
0.35,13.1	0
0.35,13.2	0
0.35,13.3	0
0.35,13.4	0
0.35,13.5	0
0.35,13.6	0
0.35,13.7	0
0.35,13.8	0
0.35,13.9	0
0.35,14.0	0
0.35,14.1	0
0.35,14.2	0
0.35,14.3	0
0.35,14.4	0
0.35,14.5	0
0.35,14.6	0
0.35,14.7	0
0.35,14.8	0
0.35,14.9	0
0.35,15.0	0
0.35,15.1	0
0.35,15.2	0

0.35,15.3	0
0.35,15.4	0
0.35,15.5	0
0.35,15.6	0
0.35,15.7	0
0.35,15.8	0
0.35,15.9	0
0.35,16.0	0
0.35,16.1	0
0.35,16.2	0
0.35,16.3	0
0.35,16.4	0
0.35,16.5	0
0.35,16.6	0
0.35,16.7	0
0.35,16.8	0
0.35,16.9	0
0.35,17.0	0
0.35,17.1	0
0.35,17.2	0
0.35,17.3	0
0.35,17.4	0
0.35,17.5	0
0.35,17.6	0
0.35,17.7	0
0.35,17.8	0
0.35,17.9	0
0.35,18.0	0
0.35,18.1	0
0.35,18.2	0
0.35,18.3	0
0.35,18.4	0
0.35,18.5	0
0.35,18.6	0
0.35,18.7	0
0.35,18.8	0
0.35,18.9	0
0.35,19.0	0
0.35,19.1	0
0.35,19.2	0
0.35,19.3	0
0.35,19.4	0

0.35,19.5	0
0.35,19.6	0
0.35,19.7	0
0.35,19.8	0
0.35,19.9	0
0.35,20.0	0
0.35,20.1	0
0.35,20.2	0
0.35,20.3	0
0.35,20.4	0
0.35,20.5	0
0.35,20.6	0
0.35,20.7	0
0.35,20.8	0
0.35,20.9	0
0.35,21.0	0
0.35,21.1	0
0.35,21.2	0
0.35,21.3	0
0.35,21.4	0
0.35,21.5	0
0.35,21.6	0
0.35,21.7	0
0.35,21.8	0
0.35,21.9	0
0.35,22.0	0
0.35,22.1	0
0.35,22.2	0
0.35,22.3	0
0.35,22.4	0
0.35,22.5	0
0.35,22.6	0
0.35,22.7	0
0.35,22.8	0
0.35,22.9	0
0.35,23.0	0
0.35,23.1	0
0.35,23.2	0
0.35,23.3	0
0.35,23.4	0
0.35,23.5	0
0.35,23.6	0

0.35,23.7	0
0.35,23.8	0
0.35,23.9	0
0.35,24.0	0
0.35,24.1	0
0.35,24.2	0
0.35,24.3	0
0.35,24.4	0
0.35,24.5	0
0.35,24.6	0
0.35,24.7	0
0.35,24.8	0
0.35,24.9	0
0.35,25.0	0
0.35,25.1	0
0.35,25.2	0
0.35,25.3	0
0.35,25.4	0
0.35,25.5	0
0.35,25.6	0
0.35,25.7	0
0.35,25.8	0
0.35,25.9	0
0.35,26.0	0
0.35,26.1	0
0.35,26.2	0
0.35,26.3	0
0.35,26.4	0
0.35,26.5	0
0.35,26.6	0
0.35,26.7	0
0.35,26.8	0
0.35,26.9	0
0.35,27.0	0
0.35,27.1	0
0.35,27.2	0
0.35,27.3	0
0.35,27.4	0
0.35,27.5	0
0.35,27.6	0
0.35,27.7	0
0.35,27.8	0

0.35,27.9	0
0.35,28.0	0
0.35,28.1	0
0.35,28.2	0
0.35,28.3	0
0.35,28.4	0
0.35,28.5	0
0.35,28.6	0
0.35,28.7	0
0.35,28.8	0
0.35,28.9	0
0.35,29.0	0
0.35,29.1	0
0.35,29.2	0
0.35,29.3	0
0.35,29.4	0
0.35,29.5	0
0.35,29.6	0
0.35,29.7	0
0.35,29.8	0
0.35,29.9	0
0.35,30.0	0
0.35,30.1	0
0.35,30.2	0
0.35,30.3	0
0.35,30.4	0
0.35,30.5	0
0.35,30.6	0
0.35,30.7	0
0.35,30.8	0
0.35,30.9	0
0.35,31.0	0
0.35,31.1	0
0.35,31.2	0
0.35,31.3	0
0.35,31.4	0
0.35,31.5	0
0.35,31.6	0
0.35,31.7	0
0.35,31.8	0
0.35,31.9	0
0.35,32.0	0

0.35,32.1	0
0.35,32.2	0
0.35,32.3	0
0.35,32.4	0
0.35,32.5	0
0.35,32.6	0
0.35,32.7	0
0.35,32.8	0
0.35,32.9	0
0.35,33.0	0
0.35,33.1	0
0.35,33.2	0
0.35,33.3	0
0.35,33.4	0
0.35,33.5	0
0.35,33.6	0
0.35,33.7	0
0.35,33.8	0
0.35,33.9	0
0.35,34.0	0
0.35,34.1	0
0.35,34.2	0
0.35,34.3	0
0.35,34.4	0
0.35,34.5	0
0.35,34.6	0
0.35,34.7	0
0.35,34.8	0
0.35,34.9	0
0.35,35.0	0
0.35,35.1	0
0.35,35.2	0
0.35,35.3	0
0.35,35.4	0
0.35,35.5	0
0.35,35.6	0
0.35,35.7	0
0.35,35.8	0
0.35,35.9	0
0.35,36.0	0
0.35,36.1	0
0.35,36.2	0

0.35,36.3	0
0.35,36.4	0
0.35,36.5	0
0.35,36.6	0
0.35,36.7	0
0.35,36.8	0
0.35,36.9	0
0.35,37.0	0
0.35,37.1	0
0.35,37.2	0
0.35,37.3	0
0.35,37.4	0
0.35,37.5	0
0.35,37.6	0
0.35,37.7	0
0.35,37.8	0
0.35,37.9	0
0.35,38.0	0
0.35,38.1	0
0.35,38.2	0
0.35,38.3	0
0.35,38.4	0
0.35,38.5	0
0.35,38.6	0
0.35,38.7	0
0.35,38.8	0
0.35,38.9	0
0.35,39.0	0
0.35,39.1	0
0.35,39.2	0
0.35,39.3	0
0.35,39.4	0
0.35,39.5	0
0.35,39.6	0
0.35,39.7	0
0.35,39.8	0
0.35,39.9	0
0.35,40.0	0
0.35,40.1	0
0.35,40.2	0
0.35,40.3	0
0.35,40.4	0

0.35,40.5	0
0.35,40.6	0
0.35,40.7	0
0.35,40.8	0
0.35,40.9	0
0.35,41.0	0
0.35,41.1	0
0.35,41.2	0
0.35,41.3	0
0.35,41.4	0
0.35,41.5	0
0.35,41.6	0
0.35,41.7	0
0.35,41.8	0
0.35,41.9	0
0.35,42.0	0
0.35,42.1	0
0.35,42.2	0
0.35,42.3	0
0.35,42.4	0
0.35,42.5	0
0.35,42.6	0
0.35,42.7	0
0.35,42.8	0
0.35,42.9	0
0.35,43.0	0
0.35,43.1	0
0.35,43.2	0
0.35,43.3	0
0.35,43.4	0
0.35,43.5	0
0.35,43.6	0
0.35,43.7	0
0.35,43.8	0
0.35,43.9	0
0.35,44.0	0
0.35,44.1	0
0.35,44.2	0
0.35,44.3	0
0.35,44.4	0
0.35,44.5	0
0.35,44.6	0

0.35,44.7	0
0.35,44.8	0
0.35,44.9	0
0.35,45.0	0
0.35,45.1	0
0.35,45.2	0
0.35,45.3	0
0.35,45.4	0
0.35,45.5	0
0.35,45.6	0
0.35,45.7	0
0.35,45.8	0
0.35,45.9	0
0.35,46.0	0
0.35,46.1	0
0.35,46.2	0
0.35,46.3	0
0.35,46.4	0
0.35,46.5	0
0.35,46.6	0
0.35,46.7	0
0.35,46.8	0
0.35,46.9	0
0.35,47.0	0
0.35,47.1	0
0.35,47.2	0
0.35,47.3	0
0.35,47.4	0
0.35,47.5	0
0.35,47.6	0
0.35,47.7	0
0.35,47.8	0
0.35,47.9	0
0.35,48.0	0
0.35,48.1	0
0.35,48.2	0
0.35,48.3	0
0.35,48.4	0
0.35,48.5	0
0.35,48.6	0
0.35,48.7	0
0.35,48.8	0

0.35,48.9	0
0.35,49.0	0
0.35,49.1	0
0.35,49.2	0
0.35,49.3	0
0.35,49.4	0
0.35,49.5	0
0.35,49.6	0
0.35,49.7	0
0.35,49.8	0
0.35,49.9	0
0.35,50.0	0
0.35,50.1	0
0.35,50.2	0
0.35,50.3	0
0.35,50.4	0
0.35,50.5	0
0.35,50.6	0
0.35,50.7	0
0.35,50.8	0
0.35,50.9	0
0.35,51.0	0
0.35,51.1	0
0.35,51.2	0
0.35,51.3	0
0.35,51.4	0
0.35,51.5	0
0.35,51.6	0
0.35,51.7	0
0.35,51.8	0
0.35,51.9	0
0.35,52.0	0
0.35,52.1	0
0.35,52.2	0
0.35,52.3	0
0.35,52.4	0
0.35,52.5	0
0.35,52.6	0
0.35,52.7	0
0.35,52.8	0
0.35,52.9	0
0.35,53.0	0

0.35,53.1	0
0.35,53.2	0
0.35,53.3	0
0.35,53.4	0
0.35,53.5	0
0.35,53.6	0
0.35,53.7	0
0.35,53.8	0
0.35,53.9	0
0.35,54.0	0
0.35,54.1	0
0.35,54.2	0
0.35,54.3	0
0.35,54.4	0
0.35,54.5	0
0.35,54.6	0
0.35,54.7	0
0.35,54.8	0
0.35,54.9	0
0.35,55.0	0
0.35,55.1	0
0.35,55.2	0
0.35,55.3	0
0.35,55.4	0
0.35,55.5	0
0.35,55.6	0
0.35,55.7	0
0.35,55.8	0
0.35,55.9	0
0.35,56.0	0
0.35,56.1	0
0.35,56.2	0
0.35,56.3	0
0.35,56.4	0
0.35,56.5	0
0.35,56.6	0
0.35,56.7	0
0.35,56.8	0
0.35,56.9	0
0.35,57.0	0
0.35,57.1	0
0.35,57.2	0

0.35,57.3	0
0.35,57.4	0
0.35,57.5	0
0.35,57.6	0
0.35,57.7	0
0.35,57.8	0
0.35,57.9	0
0.35,58.0	0
0.35,58.1	0
0.35,58.2	0
0.35,58.3	0
0.35,58.4	0
0.35,58.5	0
0.35,58.6	0
0.35,58.7	0
0.35,58.8	0
0.35,58.9	0
0.35,59.0	0
0.35,59.1	0
0.35,59.2	0
0.35,59.3	0
0.35,59.4	0
0.35,59.5	0
0.35,59.6	0
0.35,59.7	0
0.35,59.8	0
0.35,59.9	0
0.35,60.0	0
0.35,60.1	0
0.35,60.2	0
0.35,60.3	0
0.35,60.4	0
0.35,60.5	0
0.35,60.6	0
0.35,60.7	0
0.35,60.8	0
0.35,60.9	0
0.35,61.0	0
0.35,61.1	0
0.35,61.2	0
0.35,61.3	0
0.35,61.4	0

0.35,61.5	0
0.35,61.6	0
0.35,61.7	0
0.35,61.8	0
0.35,61.9	0
0.35,62.0	0
0.35,62.1	0
0.35,62.2	0
0.35,62.3	0
0.35,62.4	0
0.35,62.5	0
0.35,62.6	0
0.35,62.7	0
0.35,62.8	0
0.35,62.9	0
0.35,63.0	0
0.35,63.1	0
0.35,63.2	0
0.35,63.3	0
0.35,63.4	0
0.35,63.5	0
0.35,63.6	0
0.35,63.7	0
0.35,63.8	0
0.35,63.9	0
0.35,64.0	0
0.35,64.1	0
0.35,64.2	0
0.35,64.3	0
0.35,64.4	0
0.35,64.5	0
0.35,64.6	0
0.35,64.7	0
0.35,64.8	0
0.35,64.9	0
0.35,65.0	0
0.35,65.1	0
0.35,65.2	0
0.35,65.3	0
0.35,65.4	0
0.35,65.5	0
0.35,65.6	0

0.35,65.7	0
0.35,65.8	0
0.35,65.9	0
0.35,66.0	0
0.35,66.1	0
0.35,66.2	0
0.35,66.3	0
0.35,66.4	0
0.35,66.5	0
0.35,66.6	0
0.35,66.7	0
0.35,66.8	0
0.35,66.9	0
0.35,67.0	0
0.35,67.1	0
0.35,67.2	0
0.35,67.3	0
0.35,67.4	0
0.35,67.5	0
0.35,67.6	0
0.35,67.7	0
0.35,67.8	0
0.35,67.9	0
0.35,68.0	0
0.35,68.1	0
0.35,68.2	0
0.35,68.3	0
0.35,68.4	0
0.35,68.5	0
0.35,68.6	0
0.35,68.7	0
0.35,68.8	0
0.35,68.9	0
0.35,69.0	0
0.35,69.1	0
0.35,69.2	0
0.35,69.3	0
0.35,69.4	0
0.35,69.5	0
0.35,69.6	0
0.35,69.7	0
0.35,69.8	0

0.35,69.9	0
0.35,70.0	0
0.35,70.1	0
0.35,70.2	0
0.35,70.3	0
0.35,70.4	0
0.35,70.5	0
0.35,70.6	0
0.35,70.7	0
0.35,70.8	0
0.35,70.9	0
0.35,71.0	0
0.35,71.1	0
0.35,71.2	0
0.35,71.3	0
0.35,71.4	0
0.35,71.5	0
0.35,71.6	0
0.35,71.7	0
0.35,71.8	0
0.35,71.9	0
0.35,72.0	0
0.35,72.1	0
0.35,72.2	0
0.35,72.3	0
0.35,72.4	0
0.35,72.5	0
0.35,72.6	0
0.35,72.7	0
0.35,72.8	0
0.35,72.9	0
0.35,73.0	0
0.35,73.1	0
0.35,73.2	0
0.35,73.3	0
0.35,73.4	0
0.35,73.5	0
0.35,73.6	0
0.35,73.7	0
0.35,73.8	0
0.35,73.9	0
0.35,74.0	0

0.35,74.1	0
0.35,74.2	0
0.35,74.3	0
0.35,74.4	0
0.35,74.5	0
0.35,74.6	0
0.35,74.7	0
0.35,74.8	0
0.35,74.9	0
0.35,75.0	0
0.35,75.1	0
0.35,75.2	0
0.35,75.3	0
0.35,75.4	0
0.35,75.5	0
0.35,75.6	0
0.35,75.7	0
0.35,75.8	0
0.35,75.9	0
0.35,76.0	0
0.35,76.1	0
0.35,76.2	0
0.35,76.3	0
0.35,76.4	0
0.35,76.5	0
0.35,76.6	0
0.35,76.7	0
0.35,76.8	0
0.35,76.9	0
0.35,77.0	0
0.35,77.1	0
0.35,77.2	0
0.35,77.3	0
0.35,77.4	0
0.35,77.5	0
0.35,77.6	0
0.35,77.7	0
0.35,77.8	0
0.35,77.9	0
0.35,78.0	0
0.35,78.1	0
0.35,78.2	0

0.35,78.3	0
0.35,78.4	0
0.35,78.5	0
0.35,78.6	0
0.35,78.7	0
0.35,78.8	0
0.35,78.9	0
0.35,79.0	0
0.35,79.1	0
0.35,79.2	0
0.35,79.3	0
0.35,79.4	0
0.35,79.5	0
0.35,79.6	0
0.35,79.7	0
0.35,79.8	0
0.35,79.9	0
0.35,80.0	0
0.35,80.1	0
0.35,80.2	0
0.35,80.3	0
0.35,80.4	0
0.35,80.5	0
0.35,80.6	0
0.35,80.7	0
0.35,80.8	0
0.35,80.9	0
0.35,81.0	0
0.35,81.1	0
0.35,81.2	0
0.35,81.3	0
0.35,81.4	0
0.35,81.5	0
0.35,81.6	0
0.35,81.7	0
0.35,81.8	0
0.35,81.9	0
0.35,82.0	0
0.35,82.1	0
0.35,82.2	0
0.35,82.3	0
0.35,82.4	0

0.35,82.5	0
0.35,82.6	0
0.35,82.7	0
0.35,82.8	0
0.35,82.9	0
0.35,83.0	0
0.35,83.1	0
0.35,83.2	0
0.35,83.3	0
0.35,83.4	0
0.35,83.5	0
0.35,83.6	0
0.35,83.7	0
0.35,83.8	0
0.35,83.9	0
0.35,84.0	0
0.35,84.1	0
0.35,84.2	0
0.35,84.3	0
0.35,84.4	0
0.35,84.5	0
0.35,84.6	0
0.35,84.7	0
0.35,84.8	0
0.35,84.9	0
0.35,85.0	0
0.35,85.1	0
0.35,85.2	0
0.35,85.3	0
0.35,85.4	0
0.35,85.5	0
0.35,85.6	0
0.35,85.7	0
0.35,85.8	0
0.35,85.9	0
0.35,86.0	0
0.35,86.1	0
0.35,86.2	0
0.35,86.3	0
0.35,86.4	0
0.35,86.5	0
0.35,86.6	0

0.35,86.7	0
0.35,86.8	0
0.35,86.9	0
0.35,87.0	0
0.35,87.1	0
0.35,87.2	0
0.35,87.3	0
0.35,87.4	0
0.35,87.5	0
0.35,87.6	0
0.35,87.7	0
0.35,87.8	0
0.35,87.9	0
0.35,88.0	0
0.35,88.1	0
0.35,88.2	0
0.35,88.3	0
0.35,88.4	0
0.35,88.5	0
0.35,88.6	0
0.35,88.7	0
0.35,88.8	0
0.35,88.9	0
0.35,89.0	0
0.35,89.1	0
0.35,89.2	0
0.35,89.3	0
0.35,89.4	0
0.35,89.5	0
0.35,89.6	0
0.35,89.7	0
0.35,89.8	0
0.35,89.9	0
0.35,90.0	0
0.35,90.1	0
0.35,90.2	0
0.35,90.3	0
0.35,90.4	0
0.35,90.5	0
0.35,90.6	0
0.35,90.7	0
0.35,90.8	0

0.35,90.9	0
0.35,91.0	0
0.35,91.1	0
0.35,91.2	0
0.35,91.3	0
0.35,91.4	0
0.35,91.5	0
0.35,91.6	0
0.35,91.7	0
0.35,91.8	0
0.35,91.9	0
0.35,92.0	0
0.35,92.1	0
0.35,92.2	0
0.35,92.3	0
0.35,92.4	0
0.35,92.5	0
0.35,92.6	0
0.35,92.7	0
0.35,92.8	0
0.35,92.9	0
0.35,93.0	0
0.35,93.1	0
0.35,93.2	0
0.35,93.3	0
0.35,93.4	0
0.35,93.5	0
0.35,93.6	0
0.35,93.7	0
0.35,93.8	0
0.35,93.9	0
0.35,94.0	0
0.35,94.1	0
0.35,94.2	0
0.35,94.3	0
0.35,94.4	0
0.35,94.5	0
0.35,94.6	0
0.35,94.7	0
0.35,94.8	0
0.35,94.9	0
0.35,95.0	0

0.35,95.1	0
0.35,95.2	0
0.35,95.3	0
0.35,95.4	0
0.35,95.5	0
0.35,95.6	0
0.35,95.7	0
0.35,95.8	0
0.35,95.9	0
0.35,96.0	0
0.35,96.1	0
0.35,96.2	0
0.35,96.3	0
0.35,96.4	0
0.35,96.5	0
0.35,96.6	0
0.35,96.7	0
0.35,96.8	0
0.35,96.9	0
0.35,97.0	0
0.35,97.1	0
0.35,97.2	0
0.35,97.3	0
0.35,97.4	0
0.35,97.5	0
0.35,97.6	0
0.35,97.7	0
0.35,97.8	0
0.35,97.9	0
0.35,98.0	0
0.35,98.1	0
0.35,98.2	0
0.35,98.3	0
0.35,98.4	0
0.35,98.5	0
0.35,98.6	0
0.35,98.7	0
0.35,98.8	0
0.35,98.9	0
0.35,99.0	0
0.35,99.1	0
0.35,99.2	0

0.35,99.3	0
0.35,99.4	0
0.35,99.5	0
0.35,99.6	0
0.35,99.7	0
0.35,99.8	0
0.35,99.9	0
0.35,100.0	0
0.36,0.0	0
0.36,0.1	0
0.36,0.2	0
0.36,0.3	0
0.36,0.4	0
0.36,0.5	0
0.36,0.6	0
0.36,0.7	0
0.36,0.8	0
0.36,0.9	0
0.36,1.0	0
0.36,1.1	0
0.36,1.2	0
0.36,1.3	0
0.36,1.4	0
0.36,1.5	0
0.36,1.6	0
0.36,1.7	0
0.36,1.8	0
0.36,1.9	0
0.36,2.0	0
0.36,2.1	0
0.36,2.2	0
0.36,2.3	0
0.36,2.4	0
0.36,2.5	0
0.36,2.6	0
0.36,2.7	0
0.36,2.8	0
0.36,2.9	0
0.36,3.0	0
0.36,3.1	0
0.36,3.2	0
0.36,3.3	0

0.36,3.4	0
0.36,3.5	0
0.36,3.6	0
0.36,3.7	0
0.36,3.8	0
0.36,3.9	0
0.36,4.0	0
0.36,4.1	0
0.36,4.2	0
0.36,4.3	0
0.36,4.4	0
0.36,4.5	0
0.36,4.6	0
0.36,4.7	0
0.36,4.8	0
0.36,4.9	0
0.36,5.0	0
0.36,5.1	0
0.36,5.2	0
0.36,5.3	0
0.36,5.4	0
0.36,5.5	0
0.36,5.6	0
0.36,5.7	0
0.36,5.8	0
0.36,5.9	0
0.36,6.0	0
0.36,6.1	0
0.36,6.2	0
0.36,6.3	0
0.36,6.4	0
0.36,6.5	0
0.36,6.6	0
0.36,6.7	0
0.36,6.8	0
0.36,6.9	0
0.36,7.0	0
0.36,7.1	0
0.36,7.2	0
0.36,7.3	0
0.36,7.4	0
0.36,7.5	0

0.36,7.6	0
0.36,7.7	0
0.36,7.8	0
0.36,7.9	0
0.36,8.0	0
0.36,8.1	0
0.36,8.2	0
0.36,8.3	0
0.36,8.4	0
0.36,8.5	0
0.36,8.6	0
0.36,8.7	0
0.36,8.8	0
0.36,8.9	0
0.36,9.0	0
0.36,9.1	0
0.36,9.2	0
0.36,9.3	0
0.36,9.4	0
0.36,9.5	0
0.36,9.6	0
0.36,9.7	0
0.36,9.8	0
0.36,9.9	0
0.36,10.0	0
0.36,10.1	0
0.36,10.2	0
0.36,10.3	0
0.36,10.4	0
0.36,10.5	0
0.36,10.6	0
0.36,10.7	0
0.36,10.8	0
0.36,10.9	0
0.36,11.0	0
0.36,11.1	0
0.36,11.2	0
0.36,11.3	0
0.36,11.4	0
0.36,11.5	0
0.36,11.6	0
0.36,11.7	0

0.36,11.8	0
0.36,11.9	0
0.36,12.0	0
0.36,12.1	0
0.36,12.2	0
0.36,12.3	0
0.36,12.4	0
0.36,12.5	0
0.36,12.6	0
0.36,12.7	0
0.36,12.8	0
0.36,12.9	0
0.36,13.0	0
0.36,13.1	0
0.36,13.2	0
0.36,13.3	0
0.36,13.4	0
0.36,13.5	0
0.36,13.6	0
0.36,13.7	0
0.36,13.8	0
0.36,13.9	0
0.36,14.0	0
0.36,14.1	0
0.36,14.2	0
0.36,14.3	0
0.36,14.4	0
0.36,14.5	0
0.36,14.6	0
0.36,14.7	0
0.36,14.8	0
0.36,14.9	0
0.36,15.0	0
0.36,15.1	0
0.36,15.2	0
0.36,15.3	0
0.36,15.4	0
0.36,15.5	0
0.36,15.6	0
0.36,15.7	0
0.36,15.8	0
0.36,15.9	0

0.36,16.0	0
0.36,16.1	0
0.36,16.2	0
0.36,16.3	0
0.36,16.4	0
0.36,16.5	0
0.36,16.6	0
0.36,16.7	0
0.36,16.8	0
0.36,16.9	0
0.36,17.0	0
0.36,17.1	0
0.36,17.2	0
0.36,17.3	0
0.36,17.4	0
0.36,17.5	0
0.36,17.6	0
0.36,17.7	0
0.36,17.8	0
0.36,17.9	0
0.36,18.0	0
0.36,18.1	0
0.36,18.2	0
0.36,18.3	0
0.36,18.4	0
0.36,18.5	0
0.36,18.6	0
0.36,18.7	0
0.36,18.8	0
0.36,18.9	0
0.36,19.0	0
0.36,19.1	0
0.36,19.2	0
0.36,19.3	0
0.36,19.4	0
0.36,19.5	0
0.36,19.6	0
0.36,19.7	0
0.36,19.8	0
0.36,19.9	0
0.36,20.0	0
0.36,20.1	0

0.36,20.2	0
0.36,20.3	0
0.36,20.4	0
0.36,20.5	0
0.36,20.6	0
0.36,20.7	0
0.36,20.8	0
0.36,20.9	0
0.36,21.0	0
0.36,21.1	0
0.36,21.2	0
0.36,21.3	0
0.36,21.4	0
0.36,21.5	0
0.36,21.6	0
0.36,21.7	0
0.36,21.8	0
0.36,21.9	0
0.36,22.0	0
0.36,22.1	0
0.36,22.2	0
0.36,22.3	0
0.36,22.4	0
0.36,22.5	0
0.36,22.6	0
0.36,22.7	0
0.36,22.8	0
0.36,22.9	0
0.36,23.0	0
0.36,23.1	0
0.36,23.2	0
0.36,23.3	0
0.36,23.4	0
0.36,23.5	0
0.36,23.6	0
0.36,23.7	0
0.36,23.8	0
0.36,23.9	0
0.36,24.0	0
0.36,24.1	0
0.36,24.2	0
0.36,24.3	0

0.36,24.4	0
0.36,24.5	0
0.36,24.6	0
0.36,24.7	0
0.36,24.8	0
0.36,24.9	0
0.36,25.0	0
0.36,25.1	0
0.36,25.2	0
0.36,25.3	0
0.36,25.4	0
0.36,25.5	0
0.36,25.6	0
0.36,25.7	0
0.36,25.8	0
0.36,25.9	0
0.36,26.0	0
0.36,26.1	0
0.36,26.2	0
0.36,26.3	0
0.36,26.4	0
0.36,26.5	0
0.36,26.6	0
0.36,26.7	0
0.36,26.8	0
0.36,26.9	0
0.36,27.0	0
0.36,27.1	0
0.36,27.2	0
0.36,27.3	0
0.36,27.4	0
0.36,27.5	0
0.36,27.6	0
0.36,27.7	0
0.36,27.8	0
0.36,27.9	0
0.36,28.0	0
0.36,28.1	0
0.36,28.2	0
0.36,28.3	0
0.36,28.4	0
0.36,28.5	0

0.36,28.6	0
0.36,28.7	0
0.36,28.8	0
0.36,28.9	0
0.36,29.0	0
0.36,29.1	0
0.36,29.2	0
0.36,29.3	0
0.36,29.4	0
0.36,29.5	0
0.36,29.6	0
0.36,29.7	0
0.36,29.8	0
0.36,29.9	0
0.36,30.0	0
0.36,30.1	0
0.36,30.2	0
0.36,30.3	0
0.36,30.4	0
0.36,30.5	0
0.36,30.6	0
0.36,30.7	0
0.36,30.8	0
0.36,30.9	0
0.36,31.0	0
0.36,31.1	0
0.36,31.2	0
0.36,31.3	0
0.36,31.4	0
0.36,31.5	0
0.36,31.6	0
0.36,31.7	0
0.36,31.8	0
0.36,31.9	0
0.36,32.0	0
0.36,32.1	0
0.36,32.2	0
0.36,32.3	0
0.36,32.4	0
0.36,32.5	0
0.36,32.6	0
0.36,32.7	0

0.36,32.8	0
0.36,32.9	0
0.36,33.0	0
0.36,33.1	0
0.36,33.2	0
0.36,33.3	0
0.36,33.4	0
0.36,33.5	0
0.36,33.6	0
0.36,33.7	0
0.36,33.8	0
0.36,33.9	0
0.36,34.0	0
0.36,34.1	0
0.36,34.2	0
0.36,34.3	0
0.36,34.4	0
0.36,34.5	0
0.36,34.6	0
0.36,34.7	0
0.36,34.8	0
0.36,34.9	0
0.36,35.0	0
0.36,35.1	0
0.36,35.2	0
0.36,35.3	0
0.36,35.4	0
0.36,35.5	0
0.36,35.6	0
0.36,35.7	0
0.36,35.8	0
0.36,35.9	0
0.36,36.0	0
0.36,36.1	0
0.36,36.2	0
0.36,36.3	0
0.36,36.4	0
0.36,36.5	0
0.36,36.6	0
0.36,36.7	0
0.36,36.8	0
0.36,36.9	0

0.36,37.0	0
0.36,37.1	0
0.36,37.2	0
0.36,37.3	0
0.36,37.4	0
0.36,37.5	0
0.36,37.6	0
0.36,37.7	0
0.36,37.8	0
0.36,37.9	0
0.36,38.0	0
0.36,38.1	0
0.36,38.2	0
0.36,38.3	0
0.36,38.4	0
0.36,38.5	0
0.36,38.6	0
0.36,38.7	0
0.36,38.8	0
0.36,38.9	0
0.36,39.0	0
0.36,39.1	0
0.36,39.2	0
0.36,39.3	0
0.36,39.4	0
0.36,39.5	0
0.36,39.6	0
0.36,39.7	0
0.36,39.8	0
0.36,39.9	0
0.36,40.0	0
0.36,40.1	0
0.36,40.2	0
0.36,40.3	0
0.36,40.4	0
0.36,40.5	0
0.36,40.6	0
0.36,40.7	0
0.36,40.8	0
0.36,40.9	0
0.36,41.0	0
0.36,41.1	0

0.36,41.2	0
0.36,41.3	0
0.36,41.4	0
0.36,41.5	0
0.36,41.6	0
0.36,41.7	0
0.36,41.8	0
0.36,41.9	0
0.36,42.0	0
0.36,42.1	0
0.36,42.2	0
0.36,42.3	0
0.36,42.4	0
0.36,42.5	0
0.36,42.6	0
0.36,42.7	0
0.36,42.8	0
0.36,42.9	0
0.36,43.0	0
0.36,43.1	0
0.36,43.2	0
0.36,43.3	0
0.36,43.4	0
0.36,43.5	0
0.36,43.6	0
0.36,43.7	0
0.36,43.8	0
0.36,43.9	0
0.36,44.0	0
0.36,44.1	0
0.36,44.2	0
0.36,44.3	0
0.36,44.4	0
0.36,44.5	0
0.36,44.6	0
0.36,44.7	0
0.36,44.8	0
0.36,44.9	0
0.36,45.0	0
0.36,45.1	0
0.36,45.2	0
0.36,45.3	0

0.36,45.4	0
0.36,45.5	0
0.36,45.6	0
0.36,45.7	0
0.36,45.8	0
0.36,45.9	0
0.36,46.0	0
0.36,46.1	0
0.36,46.2	0
0.36,46.3	0
0.36,46.4	0
0.36,46.5	0
0.36,46.6	0
0.36,46.7	0
0.36,46.8	0
0.36,46.9	0
0.36,47.0	0
0.36,47.1	0
0.36,47.2	0
0.36,47.3	0
0.36,47.4	0
0.36,47.5	0
0.36,47.6	0
0.36,47.7	0
0.36,47.8	0
0.36,47.9	0
0.36,48.0	0
0.36,48.1	0
0.36,48.2	0
0.36,48.3	0
0.36,48.4	0
0.36,48.5	0
0.36,48.6	0
0.36,48.7	0
0.36,48.8	0
0.36,48.9	0
0.36,49.0	0
0.36,49.1	0
0.36,49.2	0
0.36,49.3	0
0.36,49.4	0
0.36,49.5	0

0.36,49.6	0
0.36,49.7	0
0.36,49.8	0
0.36,49.9	0
0.36,50.0	0
0.36,50.1	0
0.36,50.2	0
0.36,50.3	0
0.36,50.4	0
0.36,50.5	0
0.36,50.6	0
0.36,50.7	0
0.36,50.8	0
0.36,50.9	0
0.36,51.0	0
0.36,51.1	0
0.36,51.2	0
0.36,51.3	0
0.36,51.4	0
0.36,51.5	0
0.36,51.6	0
0.36,51.7	0
0.36,51.8	0
0.36,51.9	0
0.36,52.0	0
0.36,52.1	0
0.36,52.2	0
0.36,52.3	0
0.36,52.4	0
0.36,52.5	0
0.36,52.6	0
0.36,52.7	0
0.36,52.8	0
0.36,52.9	0
0.36,53.0	0
0.36,53.1	0
0.36,53.2	0
0.36,53.3	0
0.36,53.4	0
0.36,53.5	0
0.36,53.6	0
0.36,53.7	0

0.36,53.8	0
0.36,53.9	0
0.36,54.0	0
0.36,54.1	0
0.36,54.2	0
0.36,54.3	0
0.36,54.4	0
0.36,54.5	0
0.36,54.6	0
0.36,54.7	0
0.36,54.8	0
0.36,54.9	0
0.36,55.0	0
0.36,55.1	0
0.36,55.2	0
0.36,55.3	0
0.36,55.4	0
0.36,55.5	0
0.36,55.6	0
0.36,55.7	0
0.36,55.8	0
0.36,55.9	0
0.36,56.0	0
0.36,56.1	0
0.36,56.2	0
0.36,56.3	0
0.36,56.4	0
0.36,56.5	0
0.36,56.6	0
0.36,56.7	0
0.36,56.8	0
0.36,56.9	0
0.36,57.0	0
0.36,57.1	0
0.36,57.2	0
0.36,57.3	0
0.36,57.4	0
0.36,57.5	0
0.36,57.6	0
0.36,57.7	0
0.36,57.8	0
0.36,57.9	0

0.36,58.0	0
0.36,58.1	0
0.36,58.2	0
0.36,58.3	0
0.36,58.4	0
0.36,58.5	0
0.36,58.6	0
0.36,58.7	0
0.36,58.8	0
0.36,58.9	0
0.36,59.0	0
0.36,59.1	0
0.36,59.2	0
0.36,59.3	0
0.36,59.4	0
0.36,59.5	0
0.36,59.6	0
0.36,59.7	0
0.36,59.8	0
0.36,59.9	0
0.36,60.0	0
0.36,60.1	0
0.36,60.2	0
0.36,60.3	0
0.36,60.4	0
0.36,60.5	0
0.36,60.6	0
0.36,60.7	0
0.36,60.8	0
0.36,60.9	0
0.36,61.0	0
0.36,61.1	0
0.36,61.2	0
0.36,61.3	0
0.36,61.4	0
0.36,61.5	0
0.36,61.6	0
0.36,61.7	0
0.36,61.8	0
0.36,61.9	0
0.36,62.0	0
0.36,62.1	0

0.36,62.2	0
0.36,62.3	0
0.36,62.4	0
0.36,62.5	0
0.36,62.6	0
0.36,62.7	0
0.36,62.8	0
0.36,62.9	0
0.36,63.0	0
0.36,63.1	0
0.36,63.2	0
0.36,63.3	0
0.36,63.4	0
0.36,63.5	0
0.36,63.6	0
0.36,63.7	0
0.36,63.8	0
0.36,63.9	0
0.36,64.0	0
0.36,64.1	0
0.36,64.2	0
0.36,64.3	0
0.36,64.4	0
0.36,64.5	0
0.36,64.6	0
0.36,64.7	0
0.36,64.8	0
0.36,64.9	0
0.36,65.0	0
0.36,65.1	0
0.36,65.2	0
0.36,65.3	0
0.36,65.4	0
0.36,65.5	0
0.36,65.6	0
0.36,65.7	0
0.36,65.8	0
0.36,65.9	0
0.36,66.0	0
0.36,66.1	0
0.36,66.2	0
0.36,66.3	0

0.36,66.4	0
0.36,66.5	0
0.36,66.6	0
0.36,66.7	0
0.36,66.8	0
0.36,66.9	0
0.36,67.0	0
0.36,67.1	0
0.36,67.2	0
0.36,67.3	0
0.36,67.4	0
0.36,67.5	0
0.36,67.6	0
0.36,67.7	0
0.36,67.8	0
0.36,67.9	0
0.36,68.0	0
0.36,68.1	0
0.36,68.2	0
0.36,68.3	0
0.36,68.4	0
0.36,68.5	0
0.36,68.6	0
0.36,68.7	0
0.36,68.8	0
0.36,68.9	0
0.36,69.0	0
0.36,69.1	0
0.36,69.2	0
0.36,69.3	0
0.36,69.4	0
0.36,69.5	0
0.36,69.6	0
0.36,69.7	0
0.36,69.8	0
0.36,69.9	0
0.36,70.0	0
0.36,70.1	0
0.36,70.2	0
0.36,70.3	0
0.36,70.4	0
0.36,70.5	0

0.36,70.6	0
0.36,70.7	0
0.36,70.8	0
0.36,70.9	0
0.36,71.0	0
0.36,71.1	0
0.36,71.2	0
0.36,71.3	0
0.36,71.4	0
0.36,71.5	0
0.36,71.6	0
0.36,71.7	0
0.36,71.8	0
0.36,71.9	0
0.36,72.0	0
0.36,72.1	0
0.36,72.2	0
0.36,72.3	0
0.36,72.4	0
0.36,72.5	0
0.36,72.6	0
0.36,72.7	0
0.36,72.8	0
0.36,72.9	0
0.36,73.0	0
0.36,73.1	0
0.36,73.2	0
0.36,73.3	0
0.36,73.4	0
0.36,73.5	0
0.36,73.6	0
0.36,73.7	0
0.36,73.8	0
0.36,73.9	0
0.36,74.0	0
0.36,74.1	0
0.36,74.2	0
0.36,74.3	0
0.36,74.4	0
0.36,74.5	0
0.36,74.6	0
0.36,74.7	0

0.36,74.8	0
0.36,74.9	0
0.36,75.0	0
0.36,75.1	0
0.36,75.2	0
0.36,75.3	0
0.36,75.4	0
0.36,75.5	0
0.36,75.6	0
0.36,75.7	0
0.36,75.8	0
0.36,75.9	0
0.36,76.0	0
0.36,76.1	0
0.36,76.2	0
0.36,76.3	0
0.36,76.4	0
0.36,76.5	0
0.36,76.6	0
0.36,76.7	0
0.36,76.8	0
0.36,76.9	0
0.36,77.0	0
0.36,77.1	0
0.36,77.2	0
0.36,77.3	0
0.36,77.4	0
0.36,77.5	0
0.36,77.6	0
0.36,77.7	0
0.36,77.8	0
0.36,77.9	0
0.36,78.0	0
0.36,78.1	0
0.36,78.2	0
0.36,78.3	0
0.36,78.4	0
0.36,78.5	0
0.36,78.6	0
0.36,78.7	0
0.36,78.8	0
0.36,78.9	0

0.36,79.0	0
0.36,79.1	0
0.36,79.2	0
0.36,79.3	0
0.36,79.4	0
0.36,79.5	0
0.36,79.6	0
0.36,79.7	0
0.36,79.8	0
0.36,79.9	0
0.36,80.0	0
0.36,80.1	0
0.36,80.2	0
0.36,80.3	0
0.36,80.4	0
0.36,80.5	0
0.36,80.6	0
0.36,80.7	0
0.36,80.8	0
0.36,80.9	0
0.36,81.0	0
0.36,81.1	0
0.36,81.2	0
0.36,81.3	0
0.36,81.4	0
0.36,81.5	0
0.36,81.6	0
0.36,81.7	0
0.36,81.8	0
0.36,81.9	0
0.36,82.0	0
0.36,82.1	0
0.36,82.2	0
0.36,82.3	0
0.36,82.4	0
0.36,82.5	0
0.36,82.6	0
0.36,82.7	0
0.36,82.8	0
0.36,82.9	0
0.36,83.0	0
0.36,83.1	0

0.36,83.2	0
0.36,83.3	0
0.36,83.4	0
0.36,83.5	0
0.36,83.6	0
0.36,83.7	0
0.36,83.8	0
0.36,83.9	0
0.36,84.0	0
0.36,84.1	0
0.36,84.2	0
0.36,84.3	0
0.36,84.4	0
0.36,84.5	0
0.36,84.6	0
0.36,84.7	0
0.36,84.8	0
0.36,84.9	0
0.36,85.0	0
0.36,85.1	0
0.36,85.2	0
0.36,85.3	0
0.36,85.4	0
0.36,85.5	0
0.36,85.6	0
0.36,85.7	0
0.36,85.8	0
0.36,85.9	0
0.36,86.0	0
0.36,86.1	0
0.36,86.2	0
0.36,86.3	0
0.36,86.4	0
0.36,86.5	0
0.36,86.6	0
0.36,86.7	0
0.36,86.8	0
0.36,86.9	0
0.36,87.0	0
0.36,87.1	0
0.36,87.2	0
0.36,87.3	0

0.36,87.4	0
0.36,87.5	0
0.36,87.6	0
0.36,87.7	0
0.36,87.8	0
0.36,87.9	0
0.36,88.0	0
0.36,88.1	0
0.36,88.2	0
0.36,88.3	0
0.36,88.4	0
0.36,88.5	0
0.36,88.6	0
0.36,88.7	0
0.36,88.8	0
0.36,88.9	0
0.36,89.0	0
0.36,89.1	0
0.36,89.2	0
0.36,89.3	0
0.36,89.4	0
0.36,89.5	0
0.36,89.6	0
0.36,89.7	0
0.36,89.8	0
0.36,89.9	0
0.36,90.0	0
0.36,90.1	0
0.36,90.2	0
0.36,90.3	0
0.36,90.4	0
0.36,90.5	0
0.36,90.6	0
0.36,90.7	0
0.36,90.8	0
0.36,90.9	0
0.36,91.0	0
0.36,91.1	0
0.36,91.2	0
0.36,91.3	0
0.36,91.4	0
0.36,91.5	0

0.36,91.6	0
0.36,91.7	0
0.36,91.8	0
0.36,91.9	0
0.36,92.0	0
0.36,92.1	0
0.36,92.2	0
0.36,92.3	0
0.36,92.4	0
0.36,92.5	0
0.36,92.6	0
0.36,92.7	0
0.36,92.8	0
0.36,92.9	0
0.36,93.0	0
0.36,93.1	0
0.36,93.2	0
0.36,93.3	0
0.36,93.4	0
0.36,93.5	0
0.36,93.6	0
0.36,93.7	0
0.36,93.8	0
0.36,93.9	0
0.36,94.0	0
0.36,94.1	0
0.36,94.2	0
0.36,94.3	0
0.36,94.4	0
0.36,94.5	0
0.36,94.6	0
0.36,94.7	0
0.36,94.8	0
0.36,94.9	0
0.36,95.0	0
0.36,95.1	0
0.36,95.2	0
0.36,95.3	0
0.36,95.4	0
0.36,95.5	0
0.36,95.6	0
0.36,95.7	0

0.36,95.8	0
0.36,95.9	0
0.36,96.0	0
0.36,96.1	0
0.36,96.2	0
0.36,96.3	0
0.36,96.4	0
0.36,96.5	0
0.36,96.6	0
0.36,96.7	0
0.36,96.8	0
0.36,96.9	0
0.36,97.0	0
0.36,97.1	0
0.36,97.2	0
0.36,97.3	0
0.36,97.4	0
0.36,97.5	0
0.36,97.6	0
0.36,97.7	0
0.36,97.8	0
0.36,97.9	0
0.36,98.0	0
0.36,98.1	0
0.36,98.2	0
0.36,98.3	0
0.36,98.4	0
0.36,98.5	0
0.36,98.6	0
0.36,98.7	0
0.36,98.8	0
0.36,98.9	0
0.36,99.0	0
0.36,99.1	0
0.36,99.2	0
0.36,99.3	0
0.36,99.4	0
0.36,99.5	0
0.36,99.6	0
0.36,99.7	0
0.36,99.8	0
0.36,99.9	0

0.36,100.0	0
0.37,0.0	0
0.37,0.1	0
0.37,0.2	0
0.37,0.3	0
0.37,0.4	0
0.37,0.5	0
0.37,0.6	0
0.37,0.7	0
0.37,0.8	0
0.37,0.9	0
0.37,1.0	0
0.37,1.1	0
0.37,1.2	0
0.37,1.3	0
0.37,1.4	0
0.37,1.5	0
0.37,1.6	0
0.37,1.7	0
0.37,1.8	0
0.37,1.9	0
0.37,2.0	0
0.37,2.1	0
0.37,2.2	0
0.37,2.3	0
0.37,2.4	0
0.37,2.5	0
0.37,2.6	0
0.37,2.7	0
0.37,2.8	0
0.37,2.9	0
0.37,3.0	0
0.37,3.1	0
0.37,3.2	0
0.37,3.3	0
0.37,3.4	0
0.37,3.5	0
0.37,3.6	0
0.37,3.7	0
0.37,3.8	0
0.37,3.9	0
0.37,4.0	0

0.37,4.1	0
0.37,4.2	0
0.37,4.3	0
0.37,4.4	0
0.37,4.5	0
0.37,4.6	0
0.37,4.7	0
0.37,4.8	0
0.37,4.9	0
0.37,5.0	0
0.37,5.1	0
0.37,5.2	0
0.37,5.3	0
0.37,5.4	0
0.37,5.5	0
0.37,5.6	0
0.37,5.7	0
0.37,5.8	0
0.37,5.9	0
0.37,6.0	0
0.37,6.1	0
0.37,6.2	0
0.37,6.3	0
0.37,6.4	0
0.37,6.5	0
0.37,6.6	0
0.37,6.7	0
0.37,6.8	0
0.37,6.9	0
0.37,7.0	0
0.37,7.1	0
0.37,7.2	0
0.37,7.3	0
0.37,7.4	0
0.37,7.5	0
0.37,7.6	0
0.37,7.7	0
0.37,7.8	0
0.37,7.9	0
0.37,8.0	0
0.37,8.1	0
0.37,8.2	0

0.37,8.3	0
0.37,8.4	0
0.37,8.5	0
0.37,8.6	0
0.37,8.7	0
0.37,8.8	0
0.37,8.9	0
0.37,9.0	0
0.37,9.1	0
0.37,9.2	0
0.37,9.3	0
0.37,9.4	0
0.37,9.5	0
0.37,9.6	0
0.37,9.7	0
0.37,9.8	0
0.37,9.9	0
0.37,10.0	0
0.37,10.1	0
0.37,10.2	0
0.37,10.3	0
0.37,10.4	0
0.37,10.5	0
0.37,10.6	0
0.37,10.7	0
0.37,10.8	0
0.37,10.9	0
0.37,11.0	0
0.37,11.1	0
0.37,11.2	0
0.37,11.3	0
0.37,11.4	0
0.37,11.5	0
0.37,11.6	0
0.37,11.7	0
0.37,11.8	0
0.37,11.9	0
0.37,12.0	0
0.37,12.1	0
0.37,12.2	0
0.37,12.3	0
0.37,12.4	0

0.37,12.5	0
0.37,12.6	0
0.37,12.7	0
0.37,12.8	0
0.37,12.9	0
0.37,13.0	0
0.37,13.1	0
0.37,13.2	0
0.37,13.3	0
0.37,13.4	0
0.37,13.5	0
0.37,13.6	0
0.37,13.7	0
0.37,13.8	0
0.37,13.9	0
0.37,14.0	0
0.37,14.1	0
0.37,14.2	0
0.37,14.3	0
0.37,14.4	0
0.37,14.5	0
0.37,14.6	0
0.37,14.7	0
0.37,14.8	0
0.37,14.9	0
0.37,15.0	0
0.37,15.1	0
0.37,15.2	0
0.37,15.3	0
0.37,15.4	0
0.37,15.5	0
0.37,15.6	0
0.37,15.7	0
0.37,15.8	0
0.37,15.9	0
0.37,16.0	0
0.37,16.1	0
0.37,16.2	0
0.37,16.3	0
0.37,16.4	0
0.37,16.5	0
0.37,16.6	0

0.37,16.7	0
0.37,16.8	0
0.37,16.9	0
0.37,17.0	0
0.37,17.1	0
0.37,17.2	0
0.37,17.3	0
0.37,17.4	0
0.37,17.5	0
0.37,17.6	0
0.37,17.7	0
0.37,17.8	0
0.37,17.9	0
0.37,18.0	0
0.37,18.1	0
0.37,18.2	0
0.37,18.3	0
0.37,18.4	0
0.37,18.5	0
0.37,18.6	0
0.37,18.7	0
0.37,18.8	0
0.37,18.9	0
0.37,19.0	0
0.37,19.1	0
0.37,19.2	0
0.37,19.3	0
0.37,19.4	0
0.37,19.5	0
0.37,19.6	0
0.37,19.7	0
0.37,19.8	0
0.37,19.9	0
0.37,20.0	0
0.37,20.1	0
0.37,20.2	0
0.37,20.3	0
0.37,20.4	0
0.37,20.5	0
0.37,20.6	0
0.37,20.7	0
0.37,20.8	0

0.37,20.9	0
0.37,21.0	0
0.37,21.1	0
0.37,21.2	0
0.37,21.3	0
0.37,21.4	0
0.37,21.5	0
0.37,21.6	0
0.37,21.7	0
0.37,21.8	0
0.37,21.9	0
0.37,22.0	0
0.37,22.1	0
0.37,22.2	0
0.37,22.3	0
0.37,22.4	0
0.37,22.5	0
0.37,22.6	0
0.37,22.7	0
0.37,22.8	0
0.37,22.9	0
0.37,23.0	0
0.37,23.1	0
0.37,23.2	0
0.37,23.3	0
0.37,23.4	0
0.37,23.5	0
0.37,23.6	0
0.37,23.7	0
0.37,23.8	0
0.37,23.9	0
0.37,24.0	0
0.37,24.1	0
0.37,24.2	0
0.37,24.3	0
0.37,24.4	0
0.37,24.5	0
0.37,24.6	0
0.37,24.7	0
0.37,24.8	0
0.37,24.9	0
0.37,25.0	0

0.37,25.1	0
0.37,25.2	0
0.37,25.3	0
0.37,25.4	0
0.37,25.5	0
0.37,25.6	0
0.37,25.7	0
0.37,25.8	0
0.37,25.9	0
0.37,26.0	0
0.37,26.1	0
0.37,26.2	0
0.37,26.3	0
0.37,26.4	0
0.37,26.5	0
0.37,26.6	0
0.37,26.7	0
0.37,26.8	0
0.37,26.9	0
0.37,27.0	0
0.37,27.1	0
0.37,27.2	0
0.37,27.3	0
0.37,27.4	0
0.37,27.5	0
0.37,27.6	0
0.37,27.7	0
0.37,27.8	0
0.37,27.9	0
0.37,28.0	0
0.37,28.1	0
0.37,28.2	0
0.37,28.3	0
0.37,28.4	0
0.37,28.5	0
0.37,28.6	0
0.37,28.7	0
0.37,28.8	0
0.37,28.9	0
0.37,29.0	0
0.37,29.1	0
0.37,29.2	0

0.37,29.3	0
0.37,29.4	0
0.37,29.5	0
0.37,29.6	0
0.37,29.7	0
0.37,29.8	0
0.37,29.9	0
0.37,30.0	0
0.37,30.1	0
0.37,30.2	0
0.37,30.3	0
0.37,30.4	0
0.37,30.5	0
0.37,30.6	0
0.37,30.7	0
0.37,30.8	0
0.37,30.9	0
0.37,31.0	0
0.37,31.1	0
0.37,31.2	0
0.37,31.3	0
0.37,31.4	0
0.37,31.5	0
0.37,31.6	0
0.37,31.7	0
0.37,31.8	0
0.37,31.9	0
0.37,32.0	0
0.37,32.1	0
0.37,32.2	0
0.37,32.3	0
0.37,32.4	0
0.37,32.5	0
0.37,32.6	0
0.37,32.7	0
0.37,32.8	0
0.37,32.9	0
0.37,33.0	0
0.37,33.1	0
0.37,33.2	0
0.37,33.3	0
0.37,33.4	0

0.37,33.5	0
0.37,33.6	0
0.37,33.7	0
0.37,33.8	0
0.37,33.9	0
0.37,34.0	0
0.37,34.1	0
0.37,34.2	0
0.37,34.3	0
0.37,34.4	0
0.37,34.5	0
0.37,34.6	0
0.37,34.7	0
0.37,34.8	0
0.37,34.9	0
0.37,35.0	0
0.37,35.1	0
0.37,35.2	0
0.37,35.3	0
0.37,35.4	0
0.37,35.5	0
0.37,35.6	0
0.37,35.7	0
0.37,35.8	0
0.37,35.9	0
0.37,36.0	0
0.37,36.1	0
0.37,36.2	0
0.37,36.3	0
0.37,36.4	0
0.37,36.5	0
0.37,36.6	0
0.37,36.7	0
0.37,36.8	0
0.37,36.9	0
0.37,37.0	0
0.37,37.1	0
0.37,37.2	0
0.37,37.3	0
0.37,37.4	0
0.37,37.5	0
0.37,37.6	0

0.37,37.7	0
0.37,37.8	0
0.37,37.9	0
0.37,38.0	0
0.37,38.1	0
0.37,38.2	0
0.37,38.3	0
0.37,38.4	0
0.37,38.5	0
0.37,38.6	0
0.37,38.7	0
0.37,38.8	0
0.37,38.9	0
0.37,39.0	0
0.37,39.1	0
0.37,39.2	0
0.37,39.3	0
0.37,39.4	0
0.37,39.5	0
0.37,39.6	0
0.37,39.7	0
0.37,39.8	0
0.37,39.9	0
0.37,40.0	0
0.37,40.1	0
0.37,40.2	0
0.37,40.3	0
0.37,40.4	0
0.37,40.5	0
0.37,40.6	0
0.37,40.7	0
0.37,40.8	0
0.37,40.9	0
0.37,41.0	0
0.37,41.1	0
0.37,41.2	0
0.37,41.3	0
0.37,41.4	0
0.37,41.5	0
0.37,41.6	0
0.37,41.7	0
0.37,41.8	0

0.37,41.9	0
0.37,42.0	0
0.37,42.1	0
0.37,42.2	0
0.37,42.3	0
0.37,42.4	0
0.37,42.5	0
0.37,42.6	0
0.37,42.7	0
0.37,42.8	0
0.37,42.9	0
0.37,43.0	0
0.37,43.1	0
0.37,43.2	0
0.37,43.3	0
0.37,43.4	0
0.37,43.5	0
0.37,43.6	0
0.37,43.7	0
0.37,43.8	0
0.37,43.9	0
0.37,44.0	0
0.37,44.1	0
0.37,44.2	0
0.37,44.3	0
0.37,44.4	0
0.37,44.5	0
0.37,44.6	0
0.37,44.7	0
0.37,44.8	0
0.37,44.9	0
0.37,45.0	0
0.37,45.1	0
0.37,45.2	0
0.37,45.3	0
0.37,45.4	0
0.37,45.5	0
0.37,45.6	0
0.37,45.7	0
0.37,45.8	0
0.37,45.9	0
0.37,46.0	0

0.37,46.1	0
0.37,46.2	0
0.37,46.3	0
0.37,46.4	0
0.37,46.5	0
0.37,46.6	0
0.37,46.7	0
0.37,46.8	0
0.37,46.9	0
0.37,47.0	0
0.37,47.1	0
0.37,47.2	0
0.37,47.3	0
0.37,47.4	0
0.37,47.5	0
0.37,47.6	0
0.37,47.7	0
0.37,47.8	0
0.37,47.9	0
0.37,48.0	0
0.37,48.1	0
0.37,48.2	0
0.37,48.3	0
0.37,48.4	0
0.37,48.5	0
0.37,48.6	0
0.37,48.7	0
0.37,48.8	0
0.37,48.9	0
0.37,49.0	0
0.37,49.1	0
0.37,49.2	0
0.37,49.3	0
0.37,49.4	0
0.37,49.5	0
0.37,49.6	0
0.37,49.7	0
0.37,49.8	0
0.37,49.9	0
0.37,50.0	0
0.37,50.1	0
0.37,50.2	0

0.37,50.3	0
0.37,50.4	0
0.37,50.5	0
0.37,50.6	0
0.37,50.7	0
0.37,50.8	0
0.37,50.9	0
0.37,51.0	0
0.37,51.1	0
0.37,51.2	0
0.37,51.3	0
0.37,51.4	0
0.37,51.5	0
0.37,51.6	0
0.37,51.7	0
0.37,51.8	0
0.37,51.9	0
0.37,52.0	0
0.37,52.1	0
0.37,52.2	0
0.37,52.3	0
0.37,52.4	0
0.37,52.5	0
0.37,52.6	0
0.37,52.7	0
0.37,52.8	0
0.37,52.9	0
0.37,53.0	0
0.37,53.1	0
0.37,53.2	0
0.37,53.3	0
0.37,53.4	0
0.37,53.5	0
0.37,53.6	0
0.37,53.7	0
0.37,53.8	0
0.37,53.9	0
0.37,54.0	0
0.37,54.1	0
0.37,54.2	0
0.37,54.3	0
0.37,54.4	0

0.37,54.5	0
0.37,54.6	0
0.37,54.7	0
0.37,54.8	0
0.37,54.9	0
0.37,55.0	0
0.37,55.1	0
0.37,55.2	0
0.37,55.3	0
0.37,55.4	0
0.37,55.5	0
0.37,55.6	0
0.37,55.7	0
0.37,55.8	0
0.37,55.9	0
0.37,56.0	0
0.37,56.1	0
0.37,56.2	0
0.37,56.3	0
0.37,56.4	0
0.37,56.5	0
0.37,56.6	0
0.37,56.7	0
0.37,56.8	0
0.37,56.9	0
0.37,57.0	0
0.37,57.1	0
0.37,57.2	0
0.37,57.3	0
0.37,57.4	0
0.37,57.5	0
0.37,57.6	0
0.37,57.7	0
0.37,57.8	0
0.37,57.9	0
0.37,58.0	0
0.37,58.1	0
0.37,58.2	0
0.37,58.3	0
0.37,58.4	0
0.37,58.5	0
0.37,58.6	0

0.37,58.7	0
0.37,58.8	0
0.37,58.9	0
0.37,59.0	0
0.37,59.1	0
0.37,59.2	0
0.37,59.3	0
0.37,59.4	0
0.37,59.5	0
0.37,59.6	0
0.37,59.7	0
0.37,59.8	0
0.37,59.9	0
0.37,60.0	0
0.37,60.1	0
0.37,60.2	0
0.37,60.3	0
0.37,60.4	0
0.37,60.5	0
0.37,60.6	0
0.37,60.7	0
0.37,60.8	0
0.37,60.9	0
0.37,61.0	0
0.37,61.1	0
0.37,61.2	0
0.37,61.3	0
0.37,61.4	0
0.37,61.5	0
0.37,61.6	0
0.37,61.7	0
0.37,61.8	0
0.37,61.9	0
0.37,62.0	0
0.37,62.1	0
0.37,62.2	0
0.37,62.3	0
0.37,62.4	0
0.37,62.5	0
0.37,62.6	0
0.37,62.7	0
0.37,62.8	0

0.37,62.9	0
0.37,63.0	0
0.37,63.1	0
0.37,63.2	0
0.37,63.3	0
0.37,63.4	0
0.37,63.5	0
0.37,63.6	0
0.37,63.7	0
0.37,63.8	0
0.37,63.9	0
0.37,64.0	0
0.37,64.1	0
0.37,64.2	0
0.37,64.3	0
0.37,64.4	0
0.37,64.5	0
0.37,64.6	0
0.37,64.7	0
0.37,64.8	0
0.37,64.9	0
0.37,65.0	0
0.37,65.1	0
0.37,65.2	0
0.37,65.3	0
0.37,65.4	0
0.37,65.5	0
0.37,65.6	0
0.37,65.7	0
0.37,65.8	0
0.37,65.9	0
0.37,66.0	0
0.37,66.1	0
0.37,66.2	0
0.37,66.3	0
0.37,66.4	0
0.37,66.5	0
0.37,66.6	0
0.37,66.7	0
0.37,66.8	0
0.37,66.9	0
0.37,67.0	0

0.37,67.1	0
0.37,67.2	0
0.37,67.3	0
0.37,67.4	0
0.37,67.5	0
0.37,67.6	0
0.37,67.7	0
0.37,67.8	0
0.37,67.9	0
0.37,68.0	0
0.37,68.1	0
0.37,68.2	0
0.37,68.3	0
0.37,68.4	0
0.37,68.5	0
0.37,68.6	0
0.37,68.7	0
0.37,68.8	0
0.37,68.9	0
0.37,69.0	0
0.37,69.1	0
0.37,69.2	0
0.37,69.3	0
0.37,69.4	0
0.37,69.5	0
0.37,69.6	0
0.37,69.7	0
0.37,69.8	0
0.37,69.9	0
0.37,70.0	0
0.37,70.1	0
0.37,70.2	0
0.37,70.3	0
0.37,70.4	0
0.37,70.5	0
0.37,70.6	0
0.37,70.7	0
0.37,70.8	0
0.37,70.9	0
0.37,71.0	0
0.37,71.1	0
0.37,71.2	0

0.37,71.3	0
0.37,71.4	0
0.37,71.5	0
0.37,71.6	0
0.37,71.7	0
0.37,71.8	0
0.37,71.9	0
0.37,72.0	0
0.37,72.1	0
0.37,72.2	0
0.37,72.3	0
0.37,72.4	0
0.37,72.5	0
0.37,72.6	0
0.37,72.7	0
0.37,72.8	0
0.37,72.9	0
0.37,73.0	0
0.37,73.1	0
0.37,73.2	0
0.37,73.3	0
0.37,73.4	0
0.37,73.5	0
0.37,73.6	0
0.37,73.7	0
0.37,73.8	0
0.37,73.9	0
0.37,74.0	0
0.37,74.1	0
0.37,74.2	0
0.37,74.3	0
0.37,74.4	0
0.37,74.5	0
0.37,74.6	0
0.37,74.7	0
0.37,74.8	0
0.37,74.9	0
0.37,75.0	0
0.37,75.1	0
0.37,75.2	0
0.37,75.3	0
0.37,75.4	0

0.37,75.5	0
0.37,75.6	0
0.37,75.7	0
0.37,75.8	0
0.37,75.9	0
0.37,76.0	0
0.37,76.1	0
0.37,76.2	0
0.37,76.3	0
0.37,76.4	0
0.37,76.5	0
0.37,76.6	0
0.37,76.7	0
0.37,76.8	0
0.37,76.9	0
0.37,77.0	0
0.37,77.1	0
0.37,77.2	0
0.37,77.3	0
0.37,77.4	0
0.37,77.5	0
0.37,77.6	0
0.37,77.7	0
0.37,77.8	0
0.37,77.9	0
0.37,78.0	0
0.37,78.1	0
0.37,78.2	0
0.37,78.3	0
0.37,78.4	0
0.37,78.5	0
0.37,78.6	0
0.37,78.7	0
0.37,78.8	0
0.37,78.9	0
0.37,79.0	0
0.37,79.1	0
0.37,79.2	0
0.37,79.3	0
0.37,79.4	0
0.37,79.5	0
0.37,79.6	0

0.37,79.7	0
0.37,79.8	0
0.37,79.9	0
0.37,80.0	0
0.37,80.1	0
0.37,80.2	0
0.37,80.3	0
0.37,80.4	0
0.37,80.5	0
0.37,80.6	0
0.37,80.7	0
0.37,80.8	0
0.37,80.9	0
0.37,81.0	0
0.37,81.1	0
0.37,81.2	0
0.37,81.3	0
0.37,81.4	0
0.37,81.5	0
0.37,81.6	0
0.37,81.7	0
0.37,81.8	0
0.37,81.9	0
0.37,82.0	0
0.37,82.1	0
0.37,82.2	0
0.37,82.3	0
0.37,82.4	0
0.37,82.5	0
0.37,82.6	0
0.37,82.7	0
0.37,82.8	0
0.37,82.9	0
0.37,83.0	0
0.37,83.1	0
0.37,83.2	0
0.37,83.3	0
0.37,83.4	0
0.37,83.5	0
0.37,83.6	0
0.37,83.7	0
0.37,83.8	0

0.37,83.9	0
0.37,84.0	0
0.37,84.1	0
0.37,84.2	0
0.37,84.3	0
0.37,84.4	0
0.37,84.5	0
0.37,84.6	0
0.37,84.7	0
0.37,84.8	0
0.37,84.9	0
0.37,85.0	0
0.37,85.1	0
0.37,85.2	0
0.37,85.3	0
0.37,85.4	0
0.37,85.5	0
0.37,85.6	0
0.37,85.7	0
0.37,85.8	0
0.37,85.9	0
0.37,86.0	0
0.37,86.1	0
0.37,86.2	0
0.37,86.3	0
0.37,86.4	0
0.37,86.5	0
0.37,86.6	0
0.37,86.7	0
0.37,86.8	0
0.37,86.9	0
0.37,87.0	0
0.37,87.1	0
0.37,87.2	0
0.37,87.3	0
0.37,87.4	0
0.37,87.5	0
0.37,87.6	0
0.37,87.7	0
0.37,87.8	0
0.37,87.9	0
0.37,88.0	0

0.37,88.1	0
0.37,88.2	0
0.37,88.3	0
0.37,88.4	0
0.37,88.5	0
0.37,88.6	0
0.37,88.7	0
0.37,88.8	0
0.37,88.9	0
0.37,89.0	0
0.37,89.1	0
0.37,89.2	0
0.37,89.3	0
0.37,89.4	0
0.37,89.5	0
0.37,89.6	0
0.37,89.7	0
0.37,89.8	0
0.37,89.9	0
0.37,90.0	0
0.37,90.1	0
0.37,90.2	0
0.37,90.3	0
0.37,90.4	0
0.37,90.5	0
0.37,90.6	0
0.37,90.7	0
0.37,90.8	0
0.37,90.9	0
0.37,91.0	0
0.37,91.1	0
0.37,91.2	0
0.37,91.3	0
0.37,91.4	0
0.37,91.5	0
0.37,91.6	0
0.37,91.7	0
0.37,91.8	0
0.37,91.9	0
0.37,92.0	0
0.37,92.1	0
0.37,92.2	0

0.37,92.3	0
0.37,92.4	0
0.37,92.5	0
0.37,92.6	0
0.37,92.7	0
0.37,92.8	0
0.37,92.9	0
0.37,93.0	0
0.37,93.1	0
0.37,93.2	0
0.37,93.3	0
0.37,93.4	0
0.37,93.5	0
0.37,93.6	0
0.37,93.7	0
0.37,93.8	0
0.37,93.9	0
0.37,94.0	0
0.37,94.1	0
0.37,94.2	0
0.37,94.3	0
0.37,94.4	0
0.37,94.5	0
0.37,94.6	0
0.37,94.7	0
0.37,94.8	0
0.37,94.9	0
0.37,95.0	0
0.37,95.1	0
0.37,95.2	0
0.37,95.3	0
0.37,95.4	0
0.37,95.5	0
0.37,95.6	0
0.37,95.7	0
0.37,95.8	0
0.37,95.9	0
0.37,96.0	0
0.37,96.1	0
0.37,96.2	0
0.37,96.3	0
0.37,96.4	0

0.37,96.5	0
0.37,96.6	0
0.37,96.7	0
0.37,96.8	0
0.37,96.9	0
0.37,97.0	0
0.37,97.1	0
0.37,97.2	0
0.37,97.3	0
0.37,97.4	0
0.37,97.5	0
0.37,97.6	0
0.37,97.7	0
0.37,97.8	0
0.37,97.9	0
0.37,98.0	0
0.37,98.1	0
0.37,98.2	0
0.37,98.3	0
0.37,98.4	0
0.37,98.5	0
0.37,98.6	0
0.37,98.7	0
0.37,98.8	0
0.37,98.9	0
0.37,99.0	0
0.37,99.1	0
0.37,99.2	0
0.37,99.3	0
0.37,99.4	0
0.37,99.5	0
0.37,99.6	0
0.37,99.7	0
0.37,99.8	0
0.37,99.9	0
0.37,100.0	0
0.38,0.0	0
0.38,0.1	0
0.38,0.2	0
0.38,0.3	0
0.38,0.4	0
0.38,0.5	0

0.38,0.6	0
0.38,0.7	0
0.38,0.8	0
0.38,0.9	0
0.38,1.0	0
0.38,1.1	0
0.38,1.2	0
0.38,1.3	0
0.38,1.4	0
0.38,1.5	0
0.38,1.6	0
0.38,1.7	0
0.38,1.8	0
0.38,1.9	0
0.38,2.0	0
0.38,2.1	0
0.38,2.2	0
0.38,2.3	0
0.38,2.4	0
0.38,2.5	0
0.38,2.6	0
0.38,2.7	0
0.38,2.8	0
0.38,2.9	0
0.38,3.0	0
0.38,3.1	0
0.38,3.2	0
0.38,3.3	0
0.38,3.4	0
0.38,3.5	0
0.38,3.6	0
0.38,3.7	0
0.38,3.8	0
0.38,3.9	0
0.38,4.0	0
0.38,4.1	0
0.38,4.2	0
0.38,4.3	0
0.38,4.4	0
0.38,4.5	0
0.38,4.6	0
0.38,4.7	0

0.38,4.8	0
0.38,4.9	0
0.38,5.0	0
0.38,5.1	0
0.38,5.2	0
0.38,5.3	0
0.38,5.4	0
0.38,5.5	0
0.38,5.6	0
0.38,5.7	0
0.38,5.8	0
0.38,5.9	0
0.38,6.0	0
0.38,6.1	0
0.38,6.2	0
0.38,6.3	0
0.38,6.4	0
0.38,6.5	0
0.38,6.6	0
0.38,6.7	0
0.38,6.8	0
0.38,6.9	0
0.38,7.0	0
0.38,7.1	0
0.38,7.2	0
0.38,7.3	0
0.38,7.4	0
0.38,7.5	0
0.38,7.6	0
0.38,7.7	0
0.38,7.8	0
0.38,7.9	0
0.38,8.0	0
0.38,8.1	0
0.38,8.2	0
0.38,8.3	0
0.38,8.4	0
0.38,8.5	0
0.38,8.6	0
0.38,8.7	0
0.38,8.8	0
0.38,8.9	0

0.38,9.0	0
0.38,9.1	0
0.38,9.2	0
0.38,9.3	0
0.38,9.4	0
0.38,9.5	0
0.38,9.6	0
0.38,9.7	0
0.38,9.8	0
0.38,9.9	0
0.38,10.0	0
0.38,10.1	0
0.38,10.2	0
0.38,10.3	0
0.38,10.4	0
0.38,10.5	0
0.38,10.6	0
0.38,10.7	0
0.38,10.8	0
0.38,10.9	0
0.38,11.0	0
0.38,11.1	0
0.38,11.2	0
0.38,11.3	0
0.38,11.4	0
0.38,11.5	0
0.38,11.6	0
0.38,11.7	0
0.38,11.8	0
0.38,11.9	0
0.38,12.0	0
0.38,12.1	0
0.38,12.2	0
0.38,12.3	0
0.38,12.4	0
0.38,12.5	0
0.38,12.6	0
0.38,12.7	0
0.38,12.8	0
0.38,12.9	0
0.38,13.0	0
0.38,13.1	0

0.38,13.2	0
0.38,13.3	0
0.38,13.4	0
0.38,13.5	0
0.38,13.6	0
0.38,13.7	0
0.38,13.8	0
0.38,13.9	0
0.38,14.0	0
0.38,14.1	0
0.38,14.2	0
0.38,14.3	0
0.38,14.4	0
0.38,14.5	0
0.38,14.6	0
0.38,14.7	0
0.38,14.8	0
0.38,14.9	0
0.38,15.0	0
0.38,15.1	0
0.38,15.2	0
0.38,15.3	0
0.38,15.4	0
0.38,15.5	0
0.38,15.6	0
0.38,15.7	0
0.38,15.8	0
0.38,15.9	0
0.38,16.0	0
0.38,16.1	0
0.38,16.2	0
0.38,16.3	0
0.38,16.4	0
0.38,16.5	0
0.38,16.6	0
0.38,16.7	0
0.38,16.8	0
0.38,16.9	0
0.38,17.0	0
0.38,17.1	0
0.38,17.2	0
0.38,17.3	0

0.38,17.4	0
0.38,17.5	0
0.38,17.6	0
0.38,17.7	0
0.38,17.8	0
0.38,17.9	0
0.38,18.0	0
0.38,18.1	0
0.38,18.2	0
0.38,18.3	0
0.38,18.4	0
0.38,18.5	0
0.38,18.6	0
0.38,18.7	0
0.38,18.8	0
0.38,18.9	0
0.38,19.0	0
0.38,19.1	0
0.38,19.2	0
0.38,19.3	0
0.38,19.4	0
0.38,19.5	0
0.38,19.6	0
0.38,19.7	0
0.38,19.8	0
0.38,19.9	0
0.38,20.0	0
0.38,20.1	0
0.38,20.2	0
0.38,20.3	0
0.38,20.4	0
0.38,20.5	0
0.38,20.6	0
0.38,20.7	0
0.38,20.8	0
0.38,20.9	0
0.38,21.0	0
0.38,21.1	0
0.38,21.2	0
0.38,21.3	0
0.38,21.4	0
0.38,21.5	0

0.38,21.6	0
0.38,21.7	0
0.38,21.8	0
0.38,21.9	0
0.38,22.0	0
0.38,22.1	0
0.38,22.2	0
0.38,22.3	0
0.38,22.4	0
0.38,22.5	0
0.38,22.6	0
0.38,22.7	0
0.38,22.8	0
0.38,22.9	0
0.38,23.0	0
0.38,23.1	0
0.38,23.2	0
0.38,23.3	0
0.38,23.4	0
0.38,23.5	0
0.38,23.6	0
0.38,23.7	0
0.38,23.8	0
0.38,23.9	0
0.38,24.0	0
0.38,24.1	0
0.38,24.2	0
0.38,24.3	0
0.38,24.4	0
0.38,24.5	0
0.38,24.6	0
0.38,24.7	0
0.38,24.8	0
0.38,24.9	0
0.38,25.0	0
0.38,25.1	0
0.38,25.2	0
0.38,25.3	0
0.38,25.4	0
0.38,25.5	0
0.38,25.6	0
0.38,25.7	0

0.38,25.8	0
0.38,25.9	0
0.38,26.0	0
0.38,26.1	0
0.38,26.2	0
0.38,26.3	0
0.38,26.4	0
0.38,26.5	0
0.38,26.6	0
0.38,26.7	0
0.38,26.8	0
0.38,26.9	0
0.38,27.0	0
0.38,27.1	0
0.38,27.2	0
0.38,27.3	0
0.38,27.4	0
0.38,27.5	0
0.38,27.6	0
0.38,27.7	0
0.38,27.8	0
0.38,27.9	0
0.38,28.0	0
0.38,28.1	0
0.38,28.2	0
0.38,28.3	0
0.38,28.4	0
0.38,28.5	0
0.38,28.6	0
0.38,28.7	0
0.38,28.8	0
0.38,28.9	0
0.38,29.0	0
0.38,29.1	0
0.38,29.2	0
0.38,29.3	0
0.38,29.4	0
0.38,29.5	0
0.38,29.6	0
0.38,29.7	0
0.38,29.8	0
0.38,29.9	0

0.38,30.0	0
0.38,30.1	0
0.38,30.2	0
0.38,30.3	0
0.38,30.4	0
0.38,30.5	0
0.38,30.6	0
0.38,30.7	0
0.38,30.8	0
0.38,30.9	0
0.38,31.0	0
0.38,31.1	0
0.38,31.2	0
0.38,31.3	0
0.38,31.4	0
0.38,31.5	0
0.38,31.6	0
0.38,31.7	0
0.38,31.8	0
0.38,31.9	0
0.38,32.0	0
0.38,32.1	0
0.38,32.2	0
0.38,32.3	0
0.38,32.4	0
0.38,32.5	0
0.38,32.6	0
0.38,32.7	0
0.38,32.8	0
0.38,32.9	0
0.38,33.0	0
0.38,33.1	0
0.38,33.2	0
0.38,33.3	0
0.38,33.4	0
0.38,33.5	0
0.38,33.6	0
0.38,33.7	0
0.38,33.8	0
0.38,33.9	0
0.38,34.0	0
0.38,34.1	0

0.38,34.2	0
0.38,34.3	0
0.38,34.4	0
0.38,34.5	0
0.38,34.6	0
0.38,34.7	0
0.38,34.8	0
0.38,34.9	0
0.38,35.0	0
0.38,35.1	0
0.38,35.2	0
0.38,35.3	0
0.38,35.4	0
0.38,35.5	0
0.38,35.6	0
0.38,35.7	0
0.38,35.8	0
0.38,35.9	0
0.38,36.0	0
0.38,36.1	0
0.38,36.2	0
0.38,36.3	0
0.38,36.4	0
0.38,36.5	0
0.38,36.6	0
0.38,36.7	0
0.38,36.8	0
0.38,36.9	0
0.38,37.0	0
0.38,37.1	0
0.38,37.2	0
0.38,37.3	0
0.38,37.4	0
0.38,37.5	0
0.38,37.6	0
0.38,37.7	0
0.38,37.8	0
0.38,37.9	0
0.38,38.0	0
0.38,38.1	0
0.38,38.2	0
0.38,38.3	0

0.38,38.4	0
0.38,38.5	0
0.38,38.6	0
0.38,38.7	0
0.38,38.8	0
0.38,38.9	0
0.38,39.0	0
0.38,39.1	0
0.38,39.2	0
0.38,39.3	0
0.38,39.4	0
0.38,39.5	0
0.38,39.6	0
0.38,39.7	0
0.38,39.8	0
0.38,39.9	0
0.38,40.0	0
0.38,40.1	0
0.38,40.2	0
0.38,40.3	0
0.38,40.4	0
0.38,40.5	0
0.38,40.6	0
0.38,40.7	0
0.38,40.8	0
0.38,40.9	0
0.38,41.0	0
0.38,41.1	0
0.38,41.2	0
0.38,41.3	0
0.38,41.4	0
0.38,41.5	0
0.38,41.6	0
0.38,41.7	0
0.38,41.8	0
0.38,41.9	0
0.38,42.0	0
0.38,42.1	0
0.38,42.2	0
0.38,42.3	0
0.38,42.4	0
0.38,42.5	0

0.38,42.6	0
0.38,42.7	0
0.38,42.8	0
0.38,42.9	0
0.38,43.0	0
0.38,43.1	0
0.38,43.2	0
0.38,43.3	0
0.38,43.4	0
0.38,43.5	0
0.38,43.6	0
0.38,43.7	0
0.38,43.8	0
0.38,43.9	0
0.38,44.0	0
0.38,44.1	0
0.38,44.2	0
0.38,44.3	0
0.38,44.4	0
0.38,44.5	0
0.38,44.6	0
0.38,44.7	0
0.38,44.8	0
0.38,44.9	0
0.38,45.0	0
0.38,45.1	0
0.38,45.2	0
0.38,45.3	0
0.38,45.4	0
0.38,45.5	0
0.38,45.6	0
0.38,45.7	0
0.38,45.8	0
0.38,45.9	0
0.38,46.0	0
0.38,46.1	0
0.38,46.2	0
0.38,46.3	0
0.38,46.4	0
0.38,46.5	0
0.38,46.6	0
0.38,46.7	0

0.38,46.8	0
0.38,46.9	0
0.38,47.0	0
0.38,47.1	0
0.38,47.2	0
0.38,47.3	0
0.38,47.4	0
0.38,47.5	0
0.38,47.6	0
0.38,47.7	0
0.38,47.8	0
0.38,47.9	0
0.38,48.0	0
0.38,48.1	0
0.38,48.2	0
0.38,48.3	0
0.38,48.4	0
0.38,48.5	0
0.38,48.6	0
0.38,48.7	0
0.38,48.8	0
0.38,48.9	0
0.38,49.0	0
0.38,49.1	0
0.38,49.2	0
0.38,49.3	0
0.38,49.4	0
0.38,49.5	0
0.38,49.6	0
0.38,49.7	0
0.38,49.8	0
0.38,49.9	0
0.38,50.0	0
0.38,50.1	0
0.38,50.2	0
0.38,50.3	0
0.38,50.4	0
0.38,50.5	0
0.38,50.6	0
0.38,50.7	0
0.38,50.8	0
0.38,50.9	0

0.38,51.0	0
0.38,51.1	0
0.38,51.2	0
0.38,51.3	0
0.38,51.4	0
0.38,51.5	0
0.38,51.6	0
0.38,51.7	0
0.38,51.8	0
0.38,51.9	0
0.38,52.0	0
0.38,52.1	0
0.38,52.2	0
0.38,52.3	0
0.38,52.4	0
0.38,52.5	0
0.38,52.6	0
0.38,52.7	0
0.38,52.8	0
0.38,52.9	0
0.38,53.0	0
0.38,53.1	0
0.38,53.2	0
0.38,53.3	0
0.38,53.4	0
0.38,53.5	0
0.38,53.6	0
0.38,53.7	0
0.38,53.8	0
0.38,53.9	0
0.38,54.0	0
0.38,54.1	0
0.38,54.2	0
0.38,54.3	0
0.38,54.4	0
0.38,54.5	0
0.38,54.6	0
0.38,54.7	0
0.38,54.8	0
0.38,54.9	0
0.38,55.0	0
0.38,55.1	0

0.38,55.2	0
0.38,55.3	0
0.38,55.4	0
0.38,55.5	0
0.38,55.6	0
0.38,55.7	0
0.38,55.8	0
0.38,55.9	0
0.38,56.0	0
0.38,56.1	0
0.38,56.2	0
0.38,56.3	0
0.38,56.4	0
0.38,56.5	0
0.38,56.6	0
0.38,56.7	0
0.38,56.8	0
0.38,56.9	0
0.38,57.0	0
0.38,57.1	0
0.38,57.2	0
0.38,57.3	0
0.38,57.4	0
0.38,57.5	0
0.38,57.6	0
0.38,57.7	0
0.38,57.8	0
0.38,57.9	0
0.38,58.0	0
0.38,58.1	0
0.38,58.2	0
0.38,58.3	0
0.38,58.4	0
0.38,58.5	0
0.38,58.6	0
0.38,58.7	0
0.38,58.8	0
0.38,58.9	0
0.38,59.0	0
0.38,59.1	0
0.38,59.2	0
0.38,59.3	0

0.38,59.4	0
0.38,59.5	0
0.38,59.6	0
0.38,59.7	0
0.38,59.8	0
0.38,59.9	0
0.38,60.0	0
0.38,60.1	0
0.38,60.2	0
0.38,60.3	0
0.38,60.4	0
0.38,60.5	0
0.38,60.6	0
0.38,60.7	0
0.38,60.8	0
0.38,60.9	0
0.38,61.0	0
0.38,61.1	0
0.38,61.2	0
0.38,61.3	0
0.38,61.4	0
0.38,61.5	0
0.38,61.6	0
0.38,61.7	0
0.38,61.8	0
0.38,61.9	0
0.38,62.0	0
0.38,62.1	0
0.38,62.2	0
0.38,62.3	0
0.38,62.4	0
0.38,62.5	0
0.38,62.6	0
0.38,62.7	0
0.38,62.8	0
0.38,62.9	0
0.38,63.0	0
0.38,63.1	0
0.38,63.2	0
0.38,63.3	0
0.38,63.4	0
0.38,63.5	0

0.38,63.6	0
0.38,63.7	0
0.38,63.8	0
0.38,63.9	0
0.38,64.0	0
0.38,64.1	0
0.38,64.2	0
0.38,64.3	0
0.38,64.4	0
0.38,64.5	0
0.38,64.6	0
0.38,64.7	0
0.38,64.8	0
0.38,64.9	0
0.38,65.0	0
0.38,65.1	0
0.38,65.2	0
0.38,65.3	0
0.38,65.4	0
0.38,65.5	0
0.38,65.6	0
0.38,65.7	0
0.38,65.8	0
0.38,65.9	0
0.38,66.0	0
0.38,66.1	0
0.38,66.2	0
0.38,66.3	0
0.38,66.4	0
0.38,66.5	0
0.38,66.6	0
0.38,66.7	0
0.38,66.8	0
0.38,66.9	0
0.38,67.0	0
0.38,67.1	0
0.38,67.2	0
0.38,67.3	0
0.38,67.4	0
0.38,67.5	0
0.38,67.6	0
0.38,67.7	0

0.38,67.8	0
0.38,67.9	0
0.38,68.0	0
0.38,68.1	0
0.38,68.2	0
0.38,68.3	0
0.38,68.4	0
0.38,68.5	0
0.38,68.6	0
0.38,68.7	0
0.38,68.8	0
0.38,68.9	0
0.38,69.0	0
0.38,69.1	0
0.38,69.2	0
0.38,69.3	0
0.38,69.4	0
0.38,69.5	0
0.38,69.6	0
0.38,69.7	0
0.38,69.8	0
0.38,69.9	0
0.38,70.0	0
0.38,70.1	0
0.38,70.2	0
0.38,70.3	0
0.38,70.4	0
0.38,70.5	0
0.38,70.6	0
0.38,70.7	0
0.38,70.8	0
0.38,70.9	0
0.38,71.0	0
0.38,71.1	0
0.38,71.2	0
0.38,71.3	0
0.38,71.4	0
0.38,71.5	0
0.38,71.6	0
0.38,71.7	0
0.38,71.8	0
0.38,71.9	0

0.38,72.0	0
0.38,72.1	0
0.38,72.2	0
0.38,72.3	0
0.38,72.4	0
0.38,72.5	0
0.38,72.6	0
0.38,72.7	0
0.38,72.8	0
0.38,72.9	0
0.38,73.0	0
0.38,73.1	0
0.38,73.2	0
0.38,73.3	0
0.38,73.4	0
0.38,73.5	0
0.38,73.6	0
0.38,73.7	0
0.38,73.8	0
0.38,73.9	0
0.38,74.0	0
0.38,74.1	0
0.38,74.2	0
0.38,74.3	0
0.38,74.4	0
0.38,74.5	0
0.38,74.6	0
0.38,74.7	0
0.38,74.8	0
0.38,74.9	0
0.38,75.0	0
0.38,75.1	0
0.38,75.2	0
0.38,75.3	0
0.38,75.4	0
0.38,75.5	0
0.38,75.6	0
0.38,75.7	0
0.38,75.8	0
0.38,75.9	0
0.38,76.0	0
0.38,76.1	0

0.38,76.2	0
0.38,76.3	0
0.38,76.4	0
0.38,76.5	0
0.38,76.6	0
0.38,76.7	0
0.38,76.8	0
0.38,76.9	0
0.38,77.0	0
0.38,77.1	0
0.38,77.2	0
0.38,77.3	0
0.38,77.4	0
0.38,77.5	0
0.38,77.6	0
0.38,77.7	0
0.38,77.8	0
0.38,77.9	0
0.38,78.0	0
0.38,78.1	0
0.38,78.2	0
0.38,78.3	0
0.38,78.4	0
0.38,78.5	0
0.38,78.6	0
0.38,78.7	0
0.38,78.8	0
0.38,78.9	0
0.38,79.0	0
0.38,79.1	0
0.38,79.2	0
0.38,79.3	0
0.38,79.4	0
0.38,79.5	0
0.38,79.6	0
0.38,79.7	0
0.38,79.8	0
0.38,79.9	0
0.38,80.0	0
0.38,80.1	0
0.38,80.2	0
0.38,80.3	0

0.38,80.4	0
0.38,80.5	0
0.38,80.6	0
0.38,80.7	0
0.38,80.8	0
0.38,80.9	0
0.38,81.0	0
0.38,81.1	0
0.38,81.2	0
0.38,81.3	0
0.38,81.4	0
0.38,81.5	0
0.38,81.6	0
0.38,81.7	0
0.38,81.8	0
0.38,81.9	0
0.38,82.0	0
0.38,82.1	0
0.38,82.2	0
0.38,82.3	0
0.38,82.4	0
0.38,82.5	0
0.38,82.6	0
0.38,82.7	0
0.38,82.8	0
0.38,82.9	0
0.38,83.0	0
0.38,83.1	0
0.38,83.2	0
0.38,83.3	0
0.38,83.4	0
0.38,83.5	0
0.38,83.6	0
0.38,83.7	0
0.38,83.8	0
0.38,83.9	0
0.38,84.0	0
0.38,84.1	0
0.38,84.2	0
0.38,84.3	0
0.38,84.4	0
0.38,84.5	0

0.38,84.6	0
0.38,84.7	0
0.38,84.8	0
0.38,84.9	0
0.38,85.0	0
0.38,85.1	0
0.38,85.2	0
0.38,85.3	0
0.38,85.4	0
0.38,85.5	0
0.38,85.6	0
0.38,85.7	0
0.38,85.8	0
0.38,85.9	0
0.38,86.0	0
0.38,86.1	0
0.38,86.2	0
0.38,86.3	0
0.38,86.4	0
0.38,86.5	0
0.38,86.6	0
0.38,86.7	0
0.38,86.8	0
0.38,86.9	0
0.38,87.0	0
0.38,87.1	0
0.38,87.2	0
0.38,87.3	0
0.38,87.4	0
0.38,87.5	0
0.38,87.6	0
0.38,87.7	0
0.38,87.8	0
0.38,87.9	0
0.38,88.0	0
0.38,88.1	0
0.38,88.2	0
0.38,88.3	0
0.38,88.4	0
0.38,88.5	0
0.38,88.6	0
0.38,88.7	0

0.38,88.8	0
0.38,88.9	0
0.38,89.0	0
0.38,89.1	0
0.38,89.2	0
0.38,89.3	0
0.38,89.4	0
0.38,89.5	0
0.38,89.6	0
0.38,89.7	0
0.38,89.8	0
0.38,89.9	0
0.38,90.0	0
0.38,90.1	0
0.38,90.2	0
0.38,90.3	0
0.38,90.4	0
0.38,90.5	0
0.38,90.6	0
0.38,90.7	0
0.38,90.8	0
0.38,90.9	0
0.38,91.0	0
0.38,91.1	0
0.38,91.2	0
0.38,91.3	0
0.38,91.4	0
0.38,91.5	0
0.38,91.6	0
0.38,91.7	0
0.38,91.8	0
0.38,91.9	0
0.38,92.0	0
0.38,92.1	0
0.38,92.2	0
0.38,92.3	0
0.38,92.4	0
0.38,92.5	0
0.38,92.6	0
0.38,92.7	0
0.38,92.8	0
0.38,92.9	0

0.38,93.0	0
0.38,93.1	0
0.38,93.2	0
0.38,93.3	0
0.38,93.4	0
0.38,93.5	0
0.38,93.6	0
0.38,93.7	0
0.38,93.8	0
0.38,93.9	0
0.38,94.0	0
0.38,94.1	0
0.38,94.2	0
0.38,94.3	0
0.38,94.4	0
0.38,94.5	0
0.38,94.6	0
0.38,94.7	0
0.38,94.8	0
0.38,94.9	0
0.38,95.0	0
0.38,95.1	0
0.38,95.2	0
0.38,95.3	0
0.38,95.4	0
0.38,95.5	0
0.38,95.6	0
0.38,95.7	0
0.38,95.8	0
0.38,95.9	0
0.38,96.0	0
0.38,96.1	0
0.38,96.2	0
0.38,96.3	0
0.38,96.4	0
0.38,96.5	0
0.38,96.6	0
0.38,96.7	0
0.38,96.8	0
0.38,96.9	0
0.38,97.0	0
0.38,97.1	0

0.38,97.2	0
0.38,97.3	0
0.38,97.4	0
0.38,97.5	0
0.38,97.6	0
0.38,97.7	0
0.38,97.8	0
0.38,97.9	0
0.38,98.0	0
0.38,98.1	0
0.38,98.2	0
0.38,98.3	0
0.38,98.4	0
0.38,98.5	0
0.38,98.6	0
0.38,98.7	0
0.38,98.8	0
0.38,98.9	0
0.38,99.0	0
0.38,99.1	0
0.38,99.2	0
0.38,99.3	0
0.38,99.4	0
0.38,99.5	0
0.38,99.6	0
0.38,99.7	0
0.38,99.8	0
0.38,99.9	0
0.38,100.0	0
0.39,0.0	0
0.39,0.1	0
0.39,0.2	0
0.39,0.3	0
0.39,0.4	0
0.39,0.5	0
0.39,0.6	0
0.39,0.7	0
0.39,0.8	0
0.39,0.9	0
0.39,1.0	0
0.39,1.1	0
0.39,1.2	0

0.39,1.3	0
0.39,1.4	0
0.39,1.5	0
0.39,1.6	0
0.39,1.7	0
0.39,1.8	0
0.39,1.9	0
0.39,2.0	0
0.39,2.1	0
0.39,2.2	0
0.39,2.3	0
0.39,2.4	0
0.39,2.5	0
0.39,2.6	0
0.39,2.7	0
0.39,2.8	0
0.39,2.9	0
0.39,3.0	0
0.39,3.1	0
0.39,3.2	0
0.39,3.3	0
0.39,3.4	0
0.39,3.5	0
0.39,3.6	0
0.39,3.7	0
0.39,3.8	0
0.39,3.9	0
0.39,4.0	0
0.39,4.1	0
0.39,4.2	0
0.39,4.3	0
0.39,4.4	0
0.39,4.5	0
0.39,4.6	0
0.39,4.7	0
0.39,4.8	0
0.39,4.9	0
0.39,5.0	0
0.39,5.1	0
0.39,5.2	0
0.39,5.3	0
0.39,5.4	0

0.39,5.5	0
0.39,5.6	0
0.39,5.7	0
0.39,5.8	0
0.39,5.9	0
0.39,6.0	0
0.39,6.1	0
0.39,6.2	0
0.39,6.3	0
0.39,6.4	0
0.39,6.5	0
0.39,6.6	0
0.39,6.7	0
0.39,6.8	0
0.39,6.9	0
0.39,7.0	0
0.39,7.1	0
0.39,7.2	0
0.39,7.3	0
0.39,7.4	0
0.39,7.5	0
0.39,7.6	0
0.39,7.7	0
0.39,7.8	0
0.39,7.9	0
0.39,8.0	0
0.39,8.1	0
0.39,8.2	0
0.39,8.3	0
0.39,8.4	0
0.39,8.5	0
0.39,8.6	0
0.39,8.7	0
0.39,8.8	0
0.39,8.9	0
0.39,9.0	0
0.39,9.1	0
0.39,9.2	0
0.39,9.3	0
0.39,9.4	0
0.39,9.5	0
0.39,9.6	0

0.39,9.7	0
0.39,9.8	0
0.39,9.9	0
0.39,10.0	0
0.39,10.1	0
0.39,10.2	0
0.39,10.3	0
0.39,10.4	0
0.39,10.5	0
0.39,10.6	0
0.39,10.7	0
0.39,10.8	0
0.39,10.9	0
0.39,11.0	0
0.39,11.1	0
0.39,11.2	0
0.39,11.3	0
0.39,11.4	0
0.39,11.5	0
0.39,11.6	0
0.39,11.7	0
0.39,11.8	0
0.39,11.9	0
0.39,12.0	0
0.39,12.1	0
0.39,12.2	0
0.39,12.3	0
0.39,12.4	0
0.39,12.5	0
0.39,12.6	0
0.39,12.7	0
0.39,12.8	0
0.39,12.9	0
0.39,13.0	0
0.39,13.1	0
0.39,13.2	0
0.39,13.3	0
0.39,13.4	0
0.39,13.5	0
0.39,13.6	0
0.39,13.7	0
0.39,13.8	0

0.39,13.9	0
0.39,14.0	0
0.39,14.1	0
0.39,14.2	0
0.39,14.3	0
0.39,14.4	0
0.39,14.5	0
0.39,14.6	0
0.39,14.7	0
0.39,14.8	0
0.39,14.9	0
0.39,15.0	0
0.39,15.1	0
0.39,15.2	0
0.39,15.3	0
0.39,15.4	0
0.39,15.5	0
0.39,15.6	0
0.39,15.7	0
0.39,15.8	0
0.39,15.9	0
0.39,16.0	0
0.39,16.1	0
0.39,16.2	0
0.39,16.3	0
0.39,16.4	0
0.39,16.5	0
0.39,16.6	0
0.39,16.7	0
0.39,16.8	0
0.39,16.9	0
0.39,17.0	0
0.39,17.1	0
0.39,17.2	0
0.39,17.3	0
0.39,17.4	0
0.39,17.5	0
0.39,17.6	0
0.39,17.7	0
0.39,17.8	0
0.39,17.9	0
0.39,18.0	0

0.39,18.1	0
0.39,18.2	0
0.39,18.3	0
0.39,18.4	0
0.39,18.5	0
0.39,18.6	0
0.39,18.7	0
0.39,18.8	0
0.39,18.9	0
0.39,19.0	0
0.39,19.1	0
0.39,19.2	0
0.39,19.3	0
0.39,19.4	0
0.39,19.5	0
0.39,19.6	0
0.39,19.7	0
0.39,19.8	0
0.39,19.9	0
0.39,20.0	0
0.39,20.1	0
0.39,20.2	0
0.39,20.3	0
0.39,20.4	0
0.39,20.5	0
0.39,20.6	0
0.39,20.7	0
0.39,20.8	0
0.39,20.9	0
0.39,21.0	0
0.39,21.1	0
0.39,21.2	0
0.39,21.3	0
0.39,21.4	0
0.39,21.5	0
0.39,21.6	0
0.39,21.7	0
0.39,21.8	0
0.39,21.9	0
0.39,22.0	0
0.39,22.1	0
0.39,22.2	0

0.39,22.3	0
0.39,22.4	0
0.39,22.5	0
0.39,22.6	0
0.39,22.7	0
0.39,22.8	0
0.39,22.9	0
0.39,23.0	0
0.39,23.1	0
0.39,23.2	0
0.39,23.3	0
0.39,23.4	0
0.39,23.5	0
0.39,23.6	0
0.39,23.7	0
0.39,23.8	0
0.39,23.9	0
0.39,24.0	0
0.39,24.1	0
0.39,24.2	0
0.39,24.3	0
0.39,24.4	0
0.39,24.5	0
0.39,24.6	0
0.39,24.7	0
0.39,24.8	0
0.39,24.9	0
0.39,25.0	0
0.39,25.1	0
0.39,25.2	0
0.39,25.3	0
0.39,25.4	0
0.39,25.5	0
0.39,25.6	0
0.39,25.7	0
0.39,25.8	0
0.39,25.9	0
0.39,26.0	0
0.39,26.1	0
0.39,26.2	0
0.39,26.3	0
0.39,26.4	0

0.39,26.5	0
0.39,26.6	0
0.39,26.7	0
0.39,26.8	0
0.39,26.9	0
0.39,27.0	0
0.39,27.1	0
0.39,27.2	0
0.39,27.3	0
0.39,27.4	0
0.39,27.5	0
0.39,27.6	0
0.39,27.7	0
0.39,27.8	0
0.39,27.9	0
0.39,28.0	0
0.39,28.1	0
0.39,28.2	0
0.39,28.3	0
0.39,28.4	0
0.39,28.5	0
0.39,28.6	0
0.39,28.7	0
0.39,28.8	0
0.39,28.9	0
0.39,29.0	0
0.39,29.1	0
0.39,29.2	0
0.39,29.3	0
0.39,29.4	0
0.39,29.5	0
0.39,29.6	0
0.39,29.7	0
0.39,29.8	0
0.39,29.9	0
0.39,30.0	0
0.39,30.1	0
0.39,30.2	0
0.39,30.3	0
0.39,30.4	0
0.39,30.5	0
0.39,30.6	0

0.39,30.7	0
0.39,30.8	0
0.39,30.9	0
0.39,31.0	0
0.39,31.1	0
0.39,31.2	0
0.39,31.3	0
0.39,31.4	0
0.39,31.5	0
0.39,31.6	0
0.39,31.7	0
0.39,31.8	0
0.39,31.9	0
0.39,32.0	0
0.39,32.1	0
0.39,32.2	0
0.39,32.3	0
0.39,32.4	0
0.39,32.5	0
0.39,32.6	0
0.39,32.7	0
0.39,32.8	0
0.39,32.9	0
0.39,33.0	0
0.39,33.1	0
0.39,33.2	0
0.39,33.3	0
0.39,33.4	0
0.39,33.5	0
0.39,33.6	0
0.39,33.7	0
0.39,33.8	0
0.39,33.9	0
0.39,34.0	0
0.39,34.1	0
0.39,34.2	0
0.39,34.3	0
0.39,34.4	0
0.39,34.5	0
0.39,34.6	0
0.39,34.7	0
0.39,34.8	0

0.39,34.9	0
0.39,35.0	0
0.39,35.1	0
0.39,35.2	0
0.39,35.3	0
0.39,35.4	0
0.39,35.5	0
0.39,35.6	0
0.39,35.7	0
0.39,35.8	0
0.39,35.9	0
0.39,36.0	0
0.39,36.1	0
0.39,36.2	0
0.39,36.3	0
0.39,36.4	0
0.39,36.5	0
0.39,36.6	0
0.39,36.7	0
0.39,36.8	0
0.39,36.9	0
0.39,37.0	0
0.39,37.1	0
0.39,37.2	0
0.39,37.3	0
0.39,37.4	0
0.39,37.5	0
0.39,37.6	0
0.39,37.7	0
0.39,37.8	0
0.39,37.9	0
0.39,38.0	0
0.39,38.1	0
0.39,38.2	0
0.39,38.3	0
0.39,38.4	0
0.39,38.5	0
0.39,38.6	0
0.39,38.7	0
0.39,38.8	0
0.39,38.9	0
0.39,39.0	0

0.39,39.1	0
0.39,39.2	0
0.39,39.3	0
0.39,39.4	0
0.39,39.5	0
0.39,39.6	0
0.39,39.7	0
0.39,39.8	0
0.39,39.9	0
0.39,40.0	0
0.39,40.1	0
0.39,40.2	0
0.39,40.3	0
0.39,40.4	0
0.39,40.5	0
0.39,40.6	0
0.39,40.7	0
0.39,40.8	0
0.39,40.9	0
0.39,41.0	0
0.39,41.1	0
0.39,41.2	0
0.39,41.3	0
0.39,41.4	0
0.39,41.5	0
0.39,41.6	0
0.39,41.7	0
0.39,41.8	0
0.39,41.9	0
0.39,42.0	0
0.39,42.1	0
0.39,42.2	0
0.39,42.3	0
0.39,42.4	0
0.39,42.5	0
0.39,42.6	0
0.39,42.7	0
0.39,42.8	0
0.39,42.9	0
0.39,43.0	0
0.39,43.1	0
0.39,43.2	0

0.39,43.3	0
0.39,43.4	0
0.39,43.5	0
0.39,43.6	0
0.39,43.7	0
0.39,43.8	0
0.39,43.9	0
0.39,44.0	0
0.39,44.1	0
0.39,44.2	0
0.39,44.3	0
0.39,44.4	0
0.39,44.5	0
0.39,44.6	0
0.39,44.7	0
0.39,44.8	0
0.39,44.9	0
0.39,45.0	0
0.39,45.1	0
0.39,45.2	0
0.39,45.3	0
0.39,45.4	0
0.39,45.5	0
0.39,45.6	0
0.39,45.7	0
0.39,45.8	0
0.39,45.9	0
0.39,46.0	0
0.39,46.1	0
0.39,46.2	0
0.39,46.3	0
0.39,46.4	0
0.39,46.5	0
0.39,46.6	0
0.39,46.7	0
0.39,46.8	0
0.39,46.9	0
0.39,47.0	0
0.39,47.1	0
0.39,47.2	0
0.39,47.3	0
0.39,47.4	0

0.39,47.5	0
0.39,47.6	0
0.39,47.7	0
0.39,47.8	0
0.39,47.9	0
0.39,48.0	0
0.39,48.1	0
0.39,48.2	0
0.39,48.3	0
0.39,48.4	0
0.39,48.5	0
0.39,48.6	0
0.39,48.7	0
0.39,48.8	0
0.39,48.9	0
0.39,49.0	0
0.39,49.1	0
0.39,49.2	0
0.39,49.3	0
0.39,49.4	0
0.39,49.5	0
0.39,49.6	0
0.39,49.7	0
0.39,49.8	0
0.39,49.9	0
0.39,50.0	0
0.39,50.1	0
0.39,50.2	0
0.39,50.3	0
0.39,50.4	0
0.39,50.5	0
0.39,50.6	0
0.39,50.7	0
0.39,50.8	0
0.39,50.9	0
0.39,51.0	0
0.39,51.1	0
0.39,51.2	0
0.39,51.3	0
0.39,51.4	0
0.39,51.5	0
0.39,51.6	0

0.39,51.7	0
0.39,51.8	0
0.39,51.9	0
0.39,52.0	0
0.39,52.1	0
0.39,52.2	0
0.39,52.3	0
0.39,52.4	0
0.39,52.5	0
0.39,52.6	0
0.39,52.7	0
0.39,52.8	0
0.39,52.9	0
0.39,53.0	0
0.39,53.1	0
0.39,53.2	0
0.39,53.3	0
0.39,53.4	0
0.39,53.5	0
0.39,53.6	0
0.39,53.7	0
0.39,53.8	0
0.39,53.9	0
0.39,54.0	0
0.39,54.1	0
0.39,54.2	0
0.39,54.3	0
0.39,54.4	0
0.39,54.5	0
0.39,54.6	0
0.39,54.7	0
0.39,54.8	0
0.39,54.9	0
0.39,55.0	0
0.39,55.1	0
0.39,55.2	0
0.39,55.3	0
0.39,55.4	0
0.39,55.5	0
0.39,55.6	0
0.39,55.7	0
0.39,55.8	0

0.39,55.9	0
0.39,56.0	0
0.39,56.1	0
0.39,56.2	0
0.39,56.3	0
0.39,56.4	0
0.39,56.5	0
0.39,56.6	0
0.39,56.7	0
0.39,56.8	0
0.39,56.9	0
0.39,57.0	0
0.39,57.1	0
0.39,57.2	0
0.39,57.3	0
0.39,57.4	0
0.39,57.5	0
0.39,57.6	0
0.39,57.7	0
0.39,57.8	0
0.39,57.9	0
0.39,58.0	0
0.39,58.1	0
0.39,58.2	0
0.39,58.3	0
0.39,58.4	0
0.39,58.5	0
0.39,58.6	0
0.39,58.7	0
0.39,58.8	0
0.39,58.9	0
0.39,59.0	0
0.39,59.1	0
0.39,59.2	0
0.39,59.3	0
0.39,59.4	0
0.39,59.5	0
0.39,59.6	0
0.39,59.7	0
0.39,59.8	0
0.39,59.9	0
0.39,60.0	0

0.39,60.1	0
0.39,60.2	0
0.39,60.3	0
0.39,60.4	0
0.39,60.5	0
0.39,60.6	0
0.39,60.7	0
0.39,60.8	0
0.39,60.9	0
0.39,61.0	0
0.39,61.1	0
0.39,61.2	0
0.39,61.3	0
0.39,61.4	0
0.39,61.5	0
0.39,61.6	0
0.39,61.7	0
0.39,61.8	0
0.39,61.9	0
0.39,62.0	0
0.39,62.1	0
0.39,62.2	0
0.39,62.3	0
0.39,62.4	0
0.39,62.5	0
0.39,62.6	0
0.39,62.7	0
0.39,62.8	0
0.39,62.9	0
0.39,63.0	0
0.39,63.1	0
0.39,63.2	0
0.39,63.3	0
0.39,63.4	0
0.39,63.5	0
0.39,63.6	0
0.39,63.7	0
0.39,63.8	0
0.39,63.9	0
0.39,64.0	0
0.39,64.1	0
0.39,64.2	0

0.39,64.3	0
0.39,64.4	0
0.39,64.5	0
0.39,64.6	0
0.39,64.7	0
0.39,64.8	0
0.39,64.9	0
0.39,65.0	0
0.39,65.1	0
0.39,65.2	0
0.39,65.3	0
0.39,65.4	0
0.39,65.5	0
0.39,65.6	0
0.39,65.7	0
0.39,65.8	0
0.39,65.9	0
0.39,66.0	0
0.39,66.1	0
0.39,66.2	0
0.39,66.3	0
0.39,66.4	0
0.39,66.5	0
0.39,66.6	0
0.39,66.7	0
0.39,66.8	0
0.39,66.9	0
0.39,67.0	0
0.39,67.1	0
0.39,67.2	0
0.39,67.3	0
0.39,67.4	0
0.39,67.5	0
0.39,67.6	0
0.39,67.7	0
0.39,67.8	0
0.39,67.9	0
0.39,68.0	0
0.39,68.1	0
0.39,68.2	0
0.39,68.3	0
0.39,68.4	0

0.39,68.5	0
0.39,68.6	0
0.39,68.7	0
0.39,68.8	0
0.39,68.9	0
0.39,69.0	0
0.39,69.1	0
0.39,69.2	0
0.39,69.3	0
0.39,69.4	0
0.39,69.5	0
0.39,69.6	0
0.39,69.7	0
0.39,69.8	0
0.39,69.9	0
0.39,70.0	0
0.39,70.1	0
0.39,70.2	0
0.39,70.3	0
0.39,70.4	0
0.39,70.5	0
0.39,70.6	0
0.39,70.7	0
0.39,70.8	0
0.39,70.9	0
0.39,71.0	0
0.39,71.1	0
0.39,71.2	0
0.39,71.3	0
0.39,71.4	0
0.39,71.5	0
0.39,71.6	0
0.39,71.7	0
0.39,71.8	0
0.39,71.9	0
0.39,72.0	0
0.39,72.1	0
0.39,72.2	0
0.39,72.3	0
0.39,72.4	0
0.39,72.5	0
0.39,72.6	0

0.39,72.7	0
0.39,72.8	0
0.39,72.9	0
0.39,73.0	0
0.39,73.1	0
0.39,73.2	0
0.39,73.3	0
0.39,73.4	0
0.39,73.5	0
0.39,73.6	0
0.39,73.7	0
0.39,73.8	0
0.39,73.9	0
0.39,74.0	0
0.39,74.1	0
0.39,74.2	0
0.39,74.3	0
0.39,74.4	0
0.39,74.5	0
0.39,74.6	0
0.39,74.7	0
0.39,74.8	0
0.39,74.9	0
0.39,75.0	0
0.39,75.1	0
0.39,75.2	0
0.39,75.3	0
0.39,75.4	0
0.39,75.5	0
0.39,75.6	0
0.39,75.7	0
0.39,75.8	0
0.39,75.9	0
0.39,76.0	0
0.39,76.1	0
0.39,76.2	0
0.39,76.3	0
0.39,76.4	0
0.39,76.5	0
0.39,76.6	0
0.39,76.7	0
0.39,76.8	0

0.39,76.9	0
0.39,77.0	0
0.39,77.1	0
0.39,77.2	0
0.39,77.3	0
0.39,77.4	0
0.39,77.5	0
0.39,77.6	0
0.39,77.7	0
0.39,77.8	0
0.39,77.9	0
0.39,78.0	0
0.39,78.1	0
0.39,78.2	0
0.39,78.3	0
0.39,78.4	0
0.39,78.5	0
0.39,78.6	0
0.39,78.7	0
0.39,78.8	0
0.39,78.9	0
0.39,79.0	0
0.39,79.1	0
0.39,79.2	0
0.39,79.3	0
0.39,79.4	0
0.39,79.5	0
0.39,79.6	0
0.39,79.7	0
0.39,79.8	0
0.39,79.9	0
0.39,80.0	0
0.39,80.1	0
0.39,80.2	0
0.39,80.3	0
0.39,80.4	0
0.39,80.5	0
0.39,80.6	0
0.39,80.7	0
0.39,80.8	0
0.39,80.9	0
0.39,81.0	0

0.39,81.1	0
0.39,81.2	0
0.39,81.3	0
0.39,81.4	0
0.39,81.5	0
0.39,81.6	0
0.39,81.7	0
0.39,81.8	0
0.39,81.9	0
0.39,82.0	0
0.39,82.1	0
0.39,82.2	0
0.39,82.3	0
0.39,82.4	0
0.39,82.5	0
0.39,82.6	0
0.39,82.7	0
0.39,82.8	0
0.39,82.9	0
0.39,83.0	0
0.39,83.1	0
0.39,83.2	0
0.39,83.3	0
0.39,83.4	0
0.39,83.5	0
0.39,83.6	0
0.39,83.7	0
0.39,83.8	0
0.39,83.9	0
0.39,84.0	0
0.39,84.1	0
0.39,84.2	0
0.39,84.3	0
0.39,84.4	0
0.39,84.5	0
0.39,84.6	0
0.39,84.7	0
0.39,84.8	0
0.39,84.9	0
0.39,85.0	0
0.39,85.1	0
0.39,85.2	0

0.39,85.3	0
0.39,85.4	0
0.39,85.5	0
0.39,85.6	0
0.39,85.7	0
0.39,85.8	0
0.39,85.9	0
0.39,86.0	0
0.39,86.1	0
0.39,86.2	0
0.39,86.3	0
0.39,86.4	0
0.39,86.5	0
0.39,86.6	0
0.39,86.7	0
0.39,86.8	0
0.39,86.9	0
0.39,87.0	0
0.39,87.1	0
0.39,87.2	0
0.39,87.3	0
0.39,87.4	0
0.39,87.5	0
0.39,87.6	0
0.39,87.7	0
0.39,87.8	0
0.39,87.9	0
0.39,88.0	0
0.39,88.1	0
0.39,88.2	0
0.39,88.3	0
0.39,88.4	0
0.39,88.5	0
0.39,88.6	0
0.39,88.7	0
0.39,88.8	0
0.39,88.9	0
0.39,89.0	0
0.39,89.1	0
0.39,89.2	0
0.39,89.3	0
0.39,89.4	0

0.39,89.5	0
0.39,89.6	0
0.39,89.7	0
0.39,89.8	0
0.39,89.9	0
0.39,90.0	0
0.39,90.1	0
0.39,90.2	0
0.39,90.3	0
0.39,90.4	0
0.39,90.5	0
0.39,90.6	0
0.39,90.7	0
0.39,90.8	0
0.39,90.9	0
0.39,91.0	0
0.39,91.1	0
0.39,91.2	0
0.39,91.3	0
0.39,91.4	0
0.39,91.5	0
0.39,91.6	0
0.39,91.7	0
0.39,91.8	0
0.39,91.9	0
0.39,92.0	0
0.39,92.1	0
0.39,92.2	0
0.39,92.3	0
0.39,92.4	0
0.39,92.5	0
0.39,92.6	0
0.39,92.7	0
0.39,92.8	0
0.39,92.9	0
0.39,93.0	0
0.39,93.1	0
0.39,93.2	0
0.39,93.3	0
0.39,93.4	0
0.39,93.5	0
0.39,93.6	0

0.39,93.7	0
0.39,93.8	0
0.39,93.9	0
0.39,94.0	0
0.39,94.1	0
0.39,94.2	0
0.39,94.3	0
0.39,94.4	0
0.39,94.5	0
0.39,94.6	0
0.39,94.7	0
0.39,94.8	0
0.39,94.9	0
0.39,95.0	0
0.39,95.1	0
0.39,95.2	0
0.39,95.3	0
0.39,95.4	0
0.39,95.5	0
0.39,95.6	0
0.39,95.7	0
0.39,95.8	0
0.39,95.9	0
0.39,96.0	0
0.39,96.1	0
0.39,96.2	0
0.39,96.3	0
0.39,96.4	0
0.39,96.5	0
0.39,96.6	0
0.39,96.7	0
0.39,96.8	0
0.39,96.9	0
0.39,97.0	0
0.39,97.1	0
0.39,97.2	0
0.39,97.3	0
0.39,97.4	0
0.39,97.5	0
0.39,97.6	0
0.39,97.7	0
0.39,97.8	0

0.39,97.9	0
0.39,98.0	0
0.39,98.1	0
0.39,98.2	0
0.39,98.3	0
0.39,98.4	0
0.39,98.5	0
0.39,98.6	0
0.39,98.7	0
0.39,98.8	0
0.39,98.9	0
0.39,99.0	0
0.39,99.1	0
0.39,99.2	0
0.39,99.3	0
0.39,99.4	0
0.39,99.5	0
0.39,99.6	0
0.39,99.7	0
0.39,99.8	0
0.39,99.9	0
0.39,100.0	0
0.40,0.0	0
0.40,0.1	0
0.40,0.2	0
0.40,0.3	0
0.40,0.4	0
0.40,0.5	0
0.40,0.6	0
0.40,0.7	0
0.40,0.8	0
0.40,0.9	0
0.40,1.0	0
0.40,1.1	0
0.40,1.2	0
0.40,1.3	0
0.40,1.4	0
0.40,1.5	0
0.40,1.6	0
0.40,1.7	0
0.40,1.8	0
0.40,1.9	0

0.40,2.0	0
0.40,2.1	0
0.40,2.2	0
0.40,2.3	0
0.40,2.4	0
0.40,2.5	0
0.40,2.6	0
0.40,2.7	0
0.40,2.8	0
0.40,2.9	0
0.40,3.0	0
0.40,3.1	0
0.40,3.2	0
0.40,3.3	0
0.40,3.4	0
0.40,3.5	0
0.40,3.6	0
0.40,3.7	0
0.40,3.8	0
0.40,3.9	0
0.40,4.0	0
0.40,4.1	0
0.40,4.2	0
0.40,4.3	0
0.40,4.4	0
0.40,4.5	0
0.40,4.6	0
0.40,4.7	0
0.40,4.8	0
0.40,4.9	0
0.40,5.0	0
0.40,5.1	0
0.40,5.2	0
0.40,5.3	0
0.40,5.4	0
0.40,5.5	0
0.40,5.6	0
0.40,5.7	0
0.40,5.8	0
0.40,5.9	0
0.40,6.0	0
0.40,6.1	0

0.40,6.2	0
0.40,6.3	0
0.40,6.4	0
0.40,6.5	0
0.40,6.6	0
0.40,6.7	0
0.40,6.8	0
0.40,6.9	0
0.40,7.0	0
0.40,7.1	0
0.40,7.2	0
0.40,7.3	0
0.40,7.4	0
0.40,7.5	0
0.40,7.6	0
0.40,7.7	0
0.40,7.8	0
0.40,7.9	0
0.40,8.0	0
0.40,8.1	0
0.40,8.2	0
0.40,8.3	0
0.40,8.4	0
0.40,8.5	0
0.40,8.6	0
0.40,8.7	0
0.40,8.8	0
0.40,8.9	0
0.40,9.0	0
0.40,9.1	0
0.40,9.2	0
0.40,9.3	0
0.40,9.4	0
0.40,9.5	0
0.40,9.6	0
0.40,9.7	0
0.40,9.8	0
0.40,9.9	0
0.40,10.0	0
0.40,10.1	0
0.40,10.2	0
0.40,10.3	0

0.40,10.4	0
0.40,10.5	0
0.40,10.6	0
0.40,10.7	0
0.40,10.8	0
0.40,10.9	0
0.40,11.0	0
0.40,11.1	0
0.40,11.2	0
0.40,11.3	0
0.40,11.4	0
0.40,11.5	0
0.40,11.6	0
0.40,11.7	0
0.40,11.8	0
0.40,11.9	0
0.40,12.0	0
0.40,12.1	0
0.40,12.2	0
0.40,12.3	0
0.40,12.4	0
0.40,12.5	0
0.40,12.6	0
0.40,12.7	0
0.40,12.8	0
0.40,12.9	0
0.40,13.0	0
0.40,13.1	0
0.40,13.2	0
0.40,13.3	0
0.40,13.4	0
0.40,13.5	0
0.40,13.6	0
0.40,13.7	0
0.40,13.8	0
0.40,13.9	0
0.40,14.0	0
0.40,14.1	0
0.40,14.2	0
0.40,14.3	0
0.40,14.4	0
0.40,14.5	0

0.40,14.6	0
0.40,14.7	0
0.40,14.8	0
0.40,14.9	0
0.40,15.0	0
0.40,15.1	0
0.40,15.2	0
0.40,15.3	0
0.40,15.4	0
0.40,15.5	0
0.40,15.6	0
0.40,15.7	0
0.40,15.8	0
0.40,15.9	0
0.40,16.0	0
0.40,16.1	0
0.40,16.2	0
0.40,16.3	0
0.40,16.4	0
0.40,16.5	0
0.40,16.6	0
0.40,16.7	0
0.40,16.8	0
0.40,16.9	0
0.40,17.0	0
0.40,17.1	0
0.40,17.2	0
0.40,17.3	0
0.40,17.4	0
0.40,17.5	0
0.40,17.6	0
0.40,17.7	0
0.40,17.8	0
0.40,17.9	0
0.40,18.0	0
0.40,18.1	0
0.40,18.2	0
0.40,18.3	0
0.40,18.4	0
0.40,18.5	0
0.40,18.6	0
0.40,18.7	0

0.40,18.8	0
0.40,18.9	0
0.40,19.0	0
0.40,19.1	0
0.40,19.2	0
0.40,19.3	0
0.40,19.4	0
0.40,19.5	0
0.40,19.6	0
0.40,19.7	0
0.40,19.8	0
0.40,19.9	0
0.40,20.0	0
0.40,20.1	0
0.40,20.2	0
0.40,20.3	0
0.40,20.4	0
0.40,20.5	0
0.40,20.6	0
0.40,20.7	0
0.40,20.8	0
0.40,20.9	0
0.40,21.0	0
0.40,21.1	0
0.40,21.2	0
0.40,21.3	0
0.40,21.4	0
0.40,21.5	0
0.40,21.6	0
0.40,21.7	0
0.40,21.8	0
0.40,21.9	0
0.40,22.0	0
0.40,22.1	0
0.40,22.2	0
0.40,22.3	0
0.40,22.4	0
0.40,22.5	0
0.40,22.6	0
0.40,22.7	0
0.40,22.8	0
0.40,22.9	0

0.40,23.0	0
0.40,23.1	0
0.40,23.2	0
0.40,23.3	0
0.40,23.4	0
0.40,23.5	0
0.40,23.6	0
0.40,23.7	0
0.40,23.8	0
0.40,23.9	0
0.40,24.0	0
0.40,24.1	0
0.40,24.2	0
0.40,24.3	0
0.40,24.4	0
0.40,24.5	0
0.40,24.6	0
0.40,24.7	0
0.40,24.8	0
0.40,24.9	0
0.40,25.0	0
0.40,25.1	0
0.40,25.2	0
0.40,25.3	0
0.40,25.4	0
0.40,25.5	0
0.40,25.6	0
0.40,25.7	0
0.40,25.8	0
0.40,25.9	0
0.40,26.0	0
0.40,26.1	0
0.40,26.2	0
0.40,26.3	0
0.40,26.4	0
0.40,26.5	0
0.40,26.6	0
0.40,26.7	0
0.40,26.8	0
0.40,26.9	0
0.40,27.0	0
0.40,27.1	0

0.40,27.2	0
0.40,27.3	0
0.40,27.4	0
0.40,27.5	0
0.40,27.6	0
0.40,27.7	0
0.40,27.8	0
0.40,27.9	0
0.40,28.0	0
0.40,28.1	0
0.40,28.2	0
0.40,28.3	0
0.40,28.4	0
0.40,28.5	0
0.40,28.6	0
0.40,28.7	0
0.40,28.8	0
0.40,28.9	0
0.40,29.0	0
0.40,29.1	0
0.40,29.2	0
0.40,29.3	0
0.40,29.4	0
0.40,29.5	0
0.40,29.6	0
0.40,29.7	0
0.40,29.8	0
0.40,29.9	0
0.40,30.0	0
0.40,30.1	0
0.40,30.2	0
0.40,30.3	0
0.40,30.4	0
0.40,30.5	0
0.40,30.6	0
0.40,30.7	0
0.40,30.8	0
0.40,30.9	0
0.40,31.0	0
0.40,31.1	0
0.40,31.2	0
0.40,31.3	0

0.40,31.4	0
0.40,31.5	0
0.40,31.6	0
0.40,31.7	0
0.40,31.8	0
0.40,31.9	0
0.40,32.0	0
0.40,32.1	0
0.40,32.2	0
0.40,32.3	0
0.40,32.4	0
0.40,32.5	0
0.40,32.6	0
0.40,32.7	0
0.40,32.8	0
0.40,32.9	0
0.40,33.0	0
0.40,33.1	0
0.40,33.2	0
0.40,33.3	0
0.40,33.4	0
0.40,33.5	0
0.40,33.6	0
0.40,33.7	0
0.40,33.8	0
0.40,33.9	0
0.40,34.0	0
0.40,34.1	0
0.40,34.2	0
0.40,34.3	0
0.40,34.4	0
0.40,34.5	0
0.40,34.6	0
0.40,34.7	0
0.40,34.8	0
0.40,34.9	0
0.40,35.0	0
0.40,35.1	0
0.40,35.2	0
0.40,35.3	0
0.40,35.4	0
0.40,35.5	0

0.40,35.6	0
0.40,35.7	0
0.40,35.8	0
0.40,35.9	0
0.40,36.0	0
0.40,36.1	0
0.40,36.2	0
0.40,36.3	0
0.40,36.4	0
0.40,36.5	0
0.40,36.6	0
0.40,36.7	0
0.40,36.8	0
0.40,36.9	0
0.40,37.0	0
0.40,37.1	0
0.40,37.2	0
0.40,37.3	0
0.40,37.4	0
0.40,37.5	0
0.40,37.6	0
0.40,37.7	0
0.40,37.8	0
0.40,37.9	0
0.40,38.0	0
0.40,38.1	0
0.40,38.2	0
0.40,38.3	0
0.40,38.4	0
0.40,38.5	0
0.40,38.6	0
0.40,38.7	0
0.40,38.8	0
0.40,38.9	0
0.40,39.0	0
0.40,39.1	0
0.40,39.2	0
0.40,39.3	0
0.40,39.4	0
0.40,39.5	0
0.40,39.6	0
0.40,39.7	0

0.40,39.8	0
0.40,39.9	0
0.40,40.0	0
0.40,40.1	0
0.40,40.2	0
0.40,40.3	0
0.40,40.4	0
0.40,40.5	0
0.40,40.6	0
0.40,40.7	0
0.40,40.8	0
0.40,40.9	0
0.40,41.0	0
0.40,41.1	0
0.40,41.2	0
0.40,41.3	0
0.40,41.4	0
0.40,41.5	0
0.40,41.6	0
0.40,41.7	0
0.40,41.8	0
0.40,41.9	0
0.40,42.0	0
0.40,42.1	0
0.40,42.2	0
0.40,42.3	0
0.40,42.4	0
0.40,42.5	0
0.40,42.6	0
0.40,42.7	0
0.40,42.8	0
0.40,42.9	0
0.40,43.0	0
0.40,43.1	0
0.40,43.2	0
0.40,43.3	0
0.40,43.4	0
0.40,43.5	0
0.40,43.6	0
0.40,43.7	0
0.40,43.8	0
0.40,43.9	0

0.40,44.0	0
0.40,44.1	0
0.40,44.2	0
0.40,44.3	0
0.40,44.4	0
0.40,44.5	0
0.40,44.6	0
0.40,44.7	0
0.40,44.8	0
0.40,44.9	0
0.40,45.0	0
0.40,45.1	0
0.40,45.2	0
0.40,45.3	0
0.40,45.4	0
0.40,45.5	0
0.40,45.6	0
0.40,45.7	0
0.40,45.8	0
0.40,45.9	0
0.40,46.0	0
0.40,46.1	0
0.40,46.2	0
0.40,46.3	0
0.40,46.4	0
0.40,46.5	0
0.40,46.6	0
0.40,46.7	0
0.40,46.8	0
0.40,46.9	0
0.40,47.0	0
0.40,47.1	0
0.40,47.2	0
0.40,47.3	0
0.40,47.4	0
0.40,47.5	0
0.40,47.6	0
0.40,47.7	0
0.40,47.8	0
0.40,47.9	0
0.40,48.0	0
0.40,48.1	0

0.40,48.2	0
0.40,48.3	0
0.40,48.4	0
0.40,48.5	0
0.40,48.6	0
0.40,48.7	0
0.40,48.8	0
0.40,48.9	0
0.40,49.0	0
0.40,49.1	0
0.40,49.2	0
0.40,49.3	0
0.40,49.4	0
0.40,49.5	0
0.40,49.6	0
0.40,49.7	0
0.40,49.8	0
0.40,49.9	0
0.40,50.0	0
0.40,50.1	0
0.40,50.2	0
0.40,50.3	0
0.40,50.4	0
0.40,50.5	0
0.40,50.6	0
0.40,50.7	0
0.40,50.8	0
0.40,50.9	0
0.40,51.0	0
0.40,51.1	0
0.40,51.2	0
0.40,51.3	0
0.40,51.4	0
0.40,51.5	0
0.40,51.6	0
0.40,51.7	0
0.40,51.8	0
0.40,51.9	0
0.40,52.0	0
0.40,52.1	0
0.40,52.2	0
0.40,52.3	0

0.40,52.4	0
0.40,52.5	0
0.40,52.6	0
0.40,52.7	0
0.40,52.8	0
0.40,52.9	0
0.40,53.0	0
0.40,53.1	0
0.40,53.2	0
0.40,53.3	0
0.40,53.4	0
0.40,53.5	0
0.40,53.6	0
0.40,53.7	0
0.40,53.8	0
0.40,53.9	0
0.40,54.0	0
0.40,54.1	0
0.40,54.2	0
0.40,54.3	0
0.40,54.4	0
0.40,54.5	0
0.40,54.6	0
0.40,54.7	0
0.40,54.8	0
0.40,54.9	0
0.40,55.0	0
0.40,55.1	0
0.40,55.2	0
0.40,55.3	0
0.40,55.4	0
0.40,55.5	0
0.40,55.6	0
0.40,55.7	0
0.40,55.8	0
0.40,55.9	0
0.40,56.0	0
0.40,56.1	0
0.40,56.2	0
0.40,56.3	0
0.40,56.4	0
0.40,56.5	0

0.40,56.6	0
0.40,56.7	0
0.40,56.8	0
0.40,56.9	0
0.40,57.0	0
0.40,57.1	0
0.40,57.2	0
0.40,57.3	0
0.40,57.4	0
0.40,57.5	0
0.40,57.6	0
0.40,57.7	0
0.40,57.8	0
0.40,57.9	0
0.40,58.0	0
0.40,58.1	0
0.40,58.2	0
0.40,58.3	0
0.40,58.4	0
0.40,58.5	0
0.40,58.6	0
0.40,58.7	0
0.40,58.8	0
0.40,58.9	0
0.40,59.0	0
0.40,59.1	0
0.40,59.2	0
0.40,59.3	0
0.40,59.4	0
0.40,59.5	0
0.40,59.6	0
0.40,59.7	0
0.40,59.8	0
0.40,59.9	0
0.40,60.0	0
0.40,60.1	0
0.40,60.2	0
0.40,60.3	0
0.40,60.4	0
0.40,60.5	0
0.40,60.6	0
0.40,60.7	0

0.40,60.8	0
0.40,60.9	0
0.40,61.0	0
0.40,61.1	0
0.40,61.2	0
0.40,61.3	0
0.40,61.4	0
0.40,61.5	0
0.40,61.6	0
0.40,61.7	0
0.40,61.8	0
0.40,61.9	0
0.40,62.0	0
0.40,62.1	0
0.40,62.2	0
0.40,62.3	0
0.40,62.4	0
0.40,62.5	0
0.40,62.6	0
0.40,62.7	0
0.40,62.8	0
0.40,62.9	0
0.40,63.0	0
0.40,63.1	0
0.40,63.2	0
0.40,63.3	0
0.40,63.4	0
0.40,63.5	0
0.40,63.6	0
0.40,63.7	0
0.40,63.8	0
0.40,63.9	0
0.40,64.0	0
0.40,64.1	0
0.40,64.2	0
0.40,64.3	0
0.40,64.4	0
0.40,64.5	0
0.40,64.6	0
0.40,64.7	0
0.40,64.8	0
0.40,64.9	0

0.40,65.0	0
0.40,65.1	0
0.40,65.2	0
0.40,65.3	0
0.40,65.4	0
0.40,65.5	0
0.40,65.6	0
0.40,65.7	0
0.40,65.8	0
0.40,65.9	0
0.40,66.0	0
0.40,66.1	0
0.40,66.2	0
0.40,66.3	0
0.40,66.4	0
0.40,66.5	0
0.40,66.6	0
0.40,66.7	0
0.40,66.8	0
0.40,66.9	0
0.40,67.0	0
0.40,67.1	0
0.40,67.2	0
0.40,67.3	0
0.40,67.4	0
0.40,67.5	0
0.40,67.6	0
0.40,67.7	0
0.40,67.8	0
0.40,67.9	0
0.40,68.0	0
0.40,68.1	0
0.40,68.2	0
0.40,68.3	0
0.40,68.4	0
0.40,68.5	0
0.40,68.6	0
0.40,68.7	0
0.40,68.8	0
0.40,68.9	0
0.40,69.0	0
0.40,69.1	0

0.40,69.2	0
0.40,69.3	0
0.40,69.4	0
0.40,69.5	0
0.40,69.6	0
0.40,69.7	0
0.40,69.8	0
0.40,69.9	0
0.40,70.0	0
0.40,70.1	0
0.40,70.2	0
0.40,70.3	0
0.40,70.4	0
0.40,70.5	0
0.40,70.6	0
0.40,70.7	0
0.40,70.8	0
0.40,70.9	0
0.40,71.0	0
0.40,71.1	0
0.40,71.2	0
0.40,71.3	0
0.40,71.4	0
0.40,71.5	0
0.40,71.6	0
0.40,71.7	0
0.40,71.8	0
0.40,71.9	0
0.40,72.0	0
0.40,72.1	0
0.40,72.2	0
0.40,72.3	0
0.40,72.4	0
0.40,72.5	0
0.40,72.6	0
0.40,72.7	0
0.40,72.8	0
0.40,72.9	0
0.40,73.0	0
0.40,73.1	0
0.40,73.2	0
0.40,73.3	0

0.40,73.4	0
0.40,73.5	0
0.40,73.6	0
0.40,73.7	0
0.40,73.8	0
0.40,73.9	0
0.40,74.0	0
0.40,74.1	0
0.40,74.2	0
0.40,74.3	0
0.40,74.4	0
0.40,74.5	0
0.40,74.6	0
0.40,74.7	0
0.40,74.8	0
0.40,74.9	0
0.40,75.0	0
0.40,75.1	0
0.40,75.2	0
0.40,75.3	0
0.40,75.4	0
0.40,75.5	0
0.40,75.6	0
0.40,75.7	0
0.40,75.8	0
0.40,75.9	0
0.40,76.0	0
0.40,76.1	0
0.40,76.2	0
0.40,76.3	0
0.40,76.4	0
0.40,76.5	0
0.40,76.6	0
0.40,76.7	0
0.40,76.8	0
0.40,76.9	0
0.40,77.0	0
0.40,77.1	0
0.40,77.2	0
0.40,77.3	0
0.40,77.4	0
0.40,77.5	0

0.40,77.6	0
0.40,77.7	0
0.40,77.8	0
0.40,77.9	0
0.40,78.0	0
0.40,78.1	0
0.40,78.2	0
0.40,78.3	0
0.40,78.4	0
0.40,78.5	0
0.40,78.6	0
0.40,78.7	0
0.40,78.8	0
0.40,78.9	0
0.40,79.0	0
0.40,79.1	0
0.40,79.2	0
0.40,79.3	0
0.40,79.4	0
0.40,79.5	0
0.40,79.6	0
0.40,79.7	0
0.40,79.8	0
0.40,79.9	0
0.40,80.0	0
0.40,80.1	0
0.40,80.2	0
0.40,80.3	0
0.40,80.4	0
0.40,80.5	0
0.40,80.6	0
0.40,80.7	0
0.40,80.8	0
0.40,80.9	0
0.40,81.0	0
0.40,81.1	0
0.40,81.2	0
0.40,81.3	0
0.40,81.4	0
0.40,81.5	0
0.40,81.6	0
0.40,81.7	0

0.40,81.8	0
0.40,81.9	0
0.40,82.0	0
0.40,82.1	0
0.40,82.2	0
0.40,82.3	0
0.40,82.4	0
0.40,82.5	0
0.40,82.6	0
0.40,82.7	0
0.40,82.8	0
0.40,82.9	0
0.40,83.0	0
0.40,83.1	0
0.40,83.2	0
0.40,83.3	0
0.40,83.4	0
0.40,83.5	0
0.40,83.6	0
0.40,83.7	0
0.40,83.8	0
0.40,83.9	0
0.40,84.0	0
0.40,84.1	0
0.40,84.2	0
0.40,84.3	0
0.40,84.4	0
0.40,84.5	0
0.40,84.6	0
0.40,84.7	0
0.40,84.8	0
0.40,84.9	0
0.40,85.0	0
0.40,85.1	0
0.40,85.2	0
0.40,85.3	0
0.40,85.4	0
0.40,85.5	0
0.40,85.6	0
0.40,85.7	0
0.40,85.8	0
0.40,85.9	0

0.40,86.0	0
0.40,86.1	0
0.40,86.2	0
0.40,86.3	0
0.40,86.4	0
0.40,86.5	0
0.40,86.6	0
0.40,86.7	0
0.40,86.8	0
0.40,86.9	0
0.40,87.0	0
0.40,87.1	0
0.40,87.2	0
0.40,87.3	0
0.40,87.4	0
0.40,87.5	0
0.40,87.6	0
0.40,87.7	0
0.40,87.8	0
0.40,87.9	0
0.40,88.0	0
0.40,88.1	0
0.40,88.2	0
0.40,88.3	0
0.40,88.4	0
0.40,88.5	0
0.40,88.6	0
0.40,88.7	0
0.40,88.8	0
0.40,88.9	0
0.40,89.0	0
0.40,89.1	0
0.40,89.2	0
0.40,89.3	0
0.40,89.4	0
0.40,89.5	0
0.40,89.6	0
0.40,89.7	0
0.40,89.8	0
0.40,89.9	0
0.40,90.0	0
0.40,90.1	0

0.40,90.2	0
0.40,90.3	0
0.40,90.4	0
0.40,90.5	0
0.40,90.6	0
0.40,90.7	0
0.40,90.8	0
0.40,90.9	0
0.40,91.0	0
0.40,91.1	0
0.40,91.2	0
0.40,91.3	0
0.40,91.4	0
0.40,91.5	0
0.40,91.6	0
0.40,91.7	0
0.40,91.8	0
0.40,91.9	0
0.40,92.0	0
0.40,92.1	0
0.40,92.2	0
0.40,92.3	0
0.40,92.4	0
0.40,92.5	0
0.40,92.6	0
0.40,92.7	0
0.40,92.8	0
0.40,92.9	0
0.40,93.0	0
0.40,93.1	0
0.40,93.2	0
0.40,93.3	0
0.40,93.4	0
0.40,93.5	0
0.40,93.6	0
0.40,93.7	0
0.40,93.8	0
0.40,93.9	0
0.40,94.0	0
0.40,94.1	0
0.40,94.2	0
0.40,94.3	0

0.40,94.4	0
0.40,94.5	0
0.40,94.6	0
0.40,94.7	0
0.40,94.8	0
0.40,94.9	0
0.40,95.0	0
0.40,95.1	0
0.40,95.2	0
0.40,95.3	0
0.40,95.4	0
0.40,95.5	0
0.40,95.6	0
0.40,95.7	0
0.40,95.8	0
0.40,95.9	0
0.40,96.0	0
0.40,96.1	0
0.40,96.2	0
0.40,96.3	0
0.40,96.4	0
0.40,96.5	0
0.40,96.6	0
0.40,96.7	0
0.40,96.8	0
0.40,96.9	0
0.40,97.0	0
0.40,97.1	0
0.40,97.2	0
0.40,97.3	0
0.40,97.4	0
0.40,97.5	0
0.40,97.6	0
0.40,97.7	0
0.40,97.8	0
0.40,97.9	0
0.40,98.0	0
0.40,98.1	0
0.40,98.2	0
0.40,98.3	0
0.40,98.4	0
0.40,98.5	0

0.40,98.6	0
0.40,98.7	0
0.40,98.8	0
0.40,98.9	0
0.40,99.0	0
0.40,99.1	0
0.40,99.2	0
0.40,99.3	0
0.40,99.4	0
0.40,99.5	0
0.40,99.6	0
0.40,99.7	0
0.40,99.8	0
0.40,99.9	0
0.40,100.0	0
0.41,0.0	0
0.41,0.1	0
0.41,0.2	0
0.41,0.3	0
0.41,0.4	0
0.41,0.5	0
0.41,0.6	0
0.41,0.7	0
0.41,0.8	0
0.41,0.9	0
0.41,1.0	0
0.41,1.1	0
0.41,1.2	0
0.41,1.3	0
0.41,1.4	0
0.41,1.5	0
0.41,1.6	0
0.41,1.7	0
0.41,1.8	0
0.41,1.9	0
0.41,2.0	0
0.41,2.1	0
0.41,2.2	0
0.41,2.3	0
0.41,2.4	0
0.41,2.5	0
0.41,2.6	0

0.41,2.7	0
0.41,2.8	0
0.41,2.9	0
0.41,3.0	0
0.41,3.1	0
0.41,3.2	0
0.41,3.3	0
0.41,3.4	0
0.41,3.5	0
0.41,3.6	0
0.41,3.7	0
0.41,3.8	0
0.41,3.9	0
0.41,4.0	0
0.41,4.1	0
0.41,4.2	0
0.41,4.3	0
0.41,4.4	0
0.41,4.5	0
0.41,4.6	0
0.41,4.7	0
0.41,4.8	0
0.41,4.9	0
0.41,5.0	0
0.41,5.1	0
0.41,5.2	0
0.41,5.3	0
0.41,5.4	0
0.41,5.5	0
0.41,5.6	0
0.41,5.7	0
0.41,5.8	0
0.41,5.9	0
0.41,6.0	0
0.41,6.1	0
0.41,6.2	0
0.41,6.3	0
0.41,6.4	0
0.41,6.5	0
0.41,6.6	0
0.41,6.7	0
0.41,6.8	0

0.41,6.9	0
0.41,7.0	0
0.41,7.1	0
0.41,7.2	0
0.41,7.3	0
0.41,7.4	0
0.41,7.5	0
0.41,7.6	0
0.41,7.7	0
0.41,7.8	0
0.41,7.9	0
0.41,8.0	0
0.41,8.1	0
0.41,8.2	0
0.41,8.3	0
0.41,8.4	0
0.41,8.5	0
0.41,8.6	0
0.41,8.7	0
0.41,8.8	0
0.41,8.9	0
0.41,9.0	0
0.41,9.1	0
0.41,9.2	0
0.41,9.3	0
0.41,9.4	0
0.41,9.5	0
0.41,9.6	0
0.41,9.7	0
0.41,9.8	0
0.41,9.9	0
0.41,10.0	0
0.41,10.1	0
0.41,10.2	0
0.41,10.3	0
0.41,10.4	0
0.41,10.5	0
0.41,10.6	0
0.41,10.7	0
0.41,10.8	0
0.41,10.9	0
0.41,11.0	0

0.41,11.1	0
0.41,11.2	0
0.41,11.3	0
0.41,11.4	0
0.41,11.5	0
0.41,11.6	0
0.41,11.7	0
0.41,11.8	0
0.41,11.9	0
0.41,12.0	0
0.41,12.1	0
0.41,12.2	0
0.41,12.3	0
0.41,12.4	0
0.41,12.5	0
0.41,12.6	0
0.41,12.7	0
0.41,12.8	0
0.41,12.9	0
0.41,13.0	0
0.41,13.1	0
0.41,13.2	0
0.41,13.3	0
0.41,13.4	0
0.41,13.5	0
0.41,13.6	0
0.41,13.7	0
0.41,13.8	0
0.41,13.9	0
0.41,14.0	0
0.41,14.1	0
0.41,14.2	0
0.41,14.3	0
0.41,14.4	0
0.41,14.5	0
0.41,14.6	0
0.41,14.7	0
0.41,14.8	0
0.41,14.9	0
0.41,15.0	0
0.41,15.1	0
0.41,15.2	0

0.41,15.3	0
0.41,15.4	0
0.41,15.5	0
0.41,15.6	0
0.41,15.7	0
0.41,15.8	0
0.41,15.9	0
0.41,16.0	0
0.41,16.1	0
0.41,16.2	0
0.41,16.3	0
0.41,16.4	0
0.41,16.5	0
0.41,16.6	0
0.41,16.7	0
0.41,16.8	0
0.41,16.9	0
0.41,17.0	0
0.41,17.1	0
0.41,17.2	0
0.41,17.3	0
0.41,17.4	0
0.41,17.5	0
0.41,17.6	0
0.41,17.7	0
0.41,17.8	0
0.41,17.9	0
0.41,18.0	0
0.41,18.1	0
0.41,18.2	0
0.41,18.3	0
0.41,18.4	0
0.41,18.5	0
0.41,18.6	0
0.41,18.7	0
0.41,18.8	0
0.41,18.9	0
0.41,19.0	0
0.41,19.1	0
0.41,19.2	0
0.41,19.3	0
0.41,19.4	0

0.41,19.5	0
0.41,19.6	0
0.41,19.7	0
0.41,19.8	0
0.41,19.9	0
0.41,20.0	0
0.41,20.1	0
0.41,20.2	0
0.41,20.3	0
0.41,20.4	0
0.41,20.5	0
0.41,20.6	0
0.41,20.7	0
0.41,20.8	0
0.41,20.9	0
0.41,21.0	0
0.41,21.1	0
0.41,21.2	0
0.41,21.3	0
0.41,21.4	0
0.41,21.5	0
0.41,21.6	0
0.41,21.7	0
0.41,21.8	0
0.41,21.9	0
0.41,22.0	0
0.41,22.1	0
0.41,22.2	0
0.41,22.3	0
0.41,22.4	0
0.41,22.5	0
0.41,22.6	0
0.41,22.7	0
0.41,22.8	0
0.41,22.9	0
0.41,23.0	0
0.41,23.1	0
0.41,23.2	0
0.41,23.3	0
0.41,23.4	0
0.41,23.5	0
0.41,23.6	0

0.41,23.7	0
0.41,23.8	0
0.41,23.9	0
0.41,24.0	0
0.41,24.1	0
0.41,24.2	0
0.41,24.3	0
0.41,24.4	0
0.41,24.5	0
0.41,24.6	0
0.41,24.7	0
0.41,24.8	0
0.41,24.9	0
0.41,25.0	0
0.41,25.1	0
0.41,25.2	0
0.41,25.3	0
0.41,25.4	0
0.41,25.5	0
0.41,25.6	0
0.41,25.7	0
0.41,25.8	0
0.41,25.9	0
0.41,26.0	0
0.41,26.1	0
0.41,26.2	0
0.41,26.3	0
0.41,26.4	0
0.41,26.5	0
0.41,26.6	0
0.41,26.7	0
0.41,26.8	0
0.41,26.9	0
0.41,27.0	0
0.41,27.1	0
0.41,27.2	0
0.41,27.3	0
0.41,27.4	0
0.41,27.5	0
0.41,27.6	0
0.41,27.7	0
0.41,27.8	0

0.41,27.9	0
0.41,28.0	0
0.41,28.1	0
0.41,28.2	0
0.41,28.3	0
0.41,28.4	0
0.41,28.5	0
0.41,28.6	0
0.41,28.7	0
0.41,28.8	0
0.41,28.9	0
0.41,29.0	0
0.41,29.1	0
0.41,29.2	0
0.41,29.3	0
0.41,29.4	0
0.41,29.5	0
0.41,29.6	0
0.41,29.7	0
0.41,29.8	0
0.41,29.9	0
0.41,30.0	0
0.41,30.1	0
0.41,30.2	0
0.41,30.3	0
0.41,30.4	0
0.41,30.5	0
0.41,30.6	0
0.41,30.7	0
0.41,30.8	0
0.41,30.9	0
0.41,31.0	0
0.41,31.1	0
0.41,31.2	0
0.41,31.3	0
0.41,31.4	0
0.41,31.5	0
0.41,31.6	0
0.41,31.7	0
0.41,31.8	0
0.41,31.9	0
0.41,32.0	0

0.41,32.1	0
0.41,32.2	0
0.41,32.3	0
0.41,32.4	0
0.41,32.5	0
0.41,32.6	0
0.41,32.7	0
0.41,32.8	0
0.41,32.9	0
0.41,33.0	0
0.41,33.1	0
0.41,33.2	0
0.41,33.3	0
0.41,33.4	0
0.41,33.5	0
0.41,33.6	0
0.41,33.7	0
0.41,33.8	0
0.41,33.9	0
0.41,34.0	0
0.41,34.1	0
0.41,34.2	0
0.41,34.3	0
0.41,34.4	0
0.41,34.5	0
0.41,34.6	0
0.41,34.7	0
0.41,34.8	0
0.41,34.9	0
0.41,35.0	0
0.41,35.1	0
0.41,35.2	0
0.41,35.3	0
0.41,35.4	0
0.41,35.5	0
0.41,35.6	0
0.41,35.7	0
0.41,35.8	0
0.41,35.9	0
0.41,36.0	0
0.41,36.1	0
0.41,36.2	0

0.41,36.3	0
0.41,36.4	0
0.41,36.5	0
0.41,36.6	0
0.41,36.7	0
0.41,36.8	0
0.41,36.9	0
0.41,37.0	0
0.41,37.1	0
0.41,37.2	0
0.41,37.3	0
0.41,37.4	0
0.41,37.5	0
0.41,37.6	0
0.41,37.7	0
0.41,37.8	0
0.41,37.9	0
0.41,38.0	0
0.41,38.1	0
0.41,38.2	0
0.41,38.3	0
0.41,38.4	0
0.41,38.5	0
0.41,38.6	0
0.41,38.7	0
0.41,38.8	0
0.41,38.9	0
0.41,39.0	0
0.41,39.1	0
0.41,39.2	0
0.41,39.3	0
0.41,39.4	0
0.41,39.5	0
0.41,39.6	0
0.41,39.7	0
0.41,39.8	0
0.41,39.9	0
0.41,40.0	0
0.41,40.1	0
0.41,40.2	0
0.41,40.3	0
0.41,40.4	0

0.41,40.5	0
0.41,40.6	0
0.41,40.7	0
0.41,40.8	0
0.41,40.9	0
0.41,41.0	0
0.41,41.1	0
0.41,41.2	0
0.41,41.3	0
0.41,41.4	0
0.41,41.5	0
0.41,41.6	0
0.41,41.7	0
0.41,41.8	0
0.41,41.9	0
0.41,42.0	0
0.41,42.1	0
0.41,42.2	0
0.41,42.3	0
0.41,42.4	0
0.41,42.5	0
0.41,42.6	0
0.41,42.7	0
0.41,42.8	0
0.41,42.9	0
0.41,43.0	0
0.41,43.1	0
0.41,43.2	0
0.41,43.3	0
0.41,43.4	0
0.41,43.5	0
0.41,43.6	0
0.41,43.7	0
0.41,43.8	0
0.41,43.9	0
0.41,44.0	0
0.41,44.1	0
0.41,44.2	0
0.41,44.3	0
0.41,44.4	0
0.41,44.5	0
0.41,44.6	0

0.41,44.7	0
0.41,44.8	0
0.41,44.9	0
0.41,45.0	0
0.41,45.1	0
0.41,45.2	0
0.41,45.3	0
0.41,45.4	0
0.41,45.5	0
0.41,45.6	0
0.41,45.7	0
0.41,45.8	0
0.41,45.9	0
0.41,46.0	0
0.41,46.1	0
0.41,46.2	0
0.41,46.3	0
0.41,46.4	0
0.41,46.5	0
0.41,46.6	0
0.41,46.7	0
0.41,46.8	0
0.41,46.9	0
0.41,47.0	0
0.41,47.1	0
0.41,47.2	0
0.41,47.3	0
0.41,47.4	0
0.41,47.5	0
0.41,47.6	0
0.41,47.7	0
0.41,47.8	0
0.41,47.9	0
0.41,48.0	0
0.41,48.1	0
0.41,48.2	0
0.41,48.3	0
0.41,48.4	0
0.41,48.5	0
0.41,48.6	0
0.41,48.7	0
0.41,48.8	0

0.41,48.9	0
0.41,49.0	0
0.41,49.1	0
0.41,49.2	0
0.41,49.3	0
0.41,49.4	0
0.41,49.5	0
0.41,49.6	0
0.41,49.7	0
0.41,49.8	0
0.41,49.9	0
0.41,50.0	0
0.41,50.1	0
0.41,50.2	0
0.41,50.3	0
0.41,50.4	0
0.41,50.5	0
0.41,50.6	0
0.41,50.7	0
0.41,50.8	0
0.41,50.9	0
0.41,51.0	0
0.41,51.1	0
0.41,51.2	0
0.41,51.3	0
0.41,51.4	0
0.41,51.5	0
0.41,51.6	0
0.41,51.7	0
0.41,51.8	0
0.41,51.9	0
0.41,52.0	0
0.41,52.1	0
0.41,52.2	0
0.41,52.3	0
0.41,52.4	0
0.41,52.5	0
0.41,52.6	0
0.41,52.7	0
0.41,52.8	0
0.41,52.9	0
0.41,53.0	0

0.41,53.1	0
0.41,53.2	0
0.41,53.3	0
0.41,53.4	0
0.41,53.5	0
0.41,53.6	0
0.41,53.7	0
0.41,53.8	0
0.41,53.9	0
0.41,54.0	0
0.41,54.1	0
0.41,54.2	0
0.41,54.3	0
0.41,54.4	0
0.41,54.5	0
0.41,54.6	0
0.41,54.7	0
0.41,54.8	0
0.41,54.9	0
0.41,55.0	0
0.41,55.1	0
0.41,55.2	0
0.41,55.3	0
0.41,55.4	0
0.41,55.5	0
0.41,55.6	0
0.41,55.7	0
0.41,55.8	0
0.41,55.9	0
0.41,56.0	0
0.41,56.1	0
0.41,56.2	0
0.41,56.3	0
0.41,56.4	0
0.41,56.5	0
0.41,56.6	0
0.41,56.7	0
0.41,56.8	0
0.41,56.9	0
0.41,57.0	0
0.41,57.1	0
0.41,57.2	0

0.41,57.3	0
0.41,57.4	0
0.41,57.5	0
0.41,57.6	0
0.41,57.7	0
0.41,57.8	0
0.41,57.9	0
0.41,58.0	0
0.41,58.1	0
0.41,58.2	0
0.41,58.3	0
0.41,58.4	0
0.41,58.5	0
0.41,58.6	0
0.41,58.7	0
0.41,58.8	0
0.41,58.9	0
0.41,59.0	0
0.41,59.1	0
0.41,59.2	0
0.41,59.3	0
0.41,59.4	0
0.41,59.5	0
0.41,59.6	0
0.41,59.7	0
0.41,59.8	0
0.41,59.9	0
0.41,60.0	0
0.41,60.1	0
0.41,60.2	0
0.41,60.3	0
0.41,60.4	0
0.41,60.5	0
0.41,60.6	0
0.41,60.7	0
0.41,60.8	0
0.41,60.9	0
0.41,61.0	0
0.41,61.1	0
0.41,61.2	0
0.41,61.3	0
0.41,61.4	0

0.41,61.5	0
0.41,61.6	0
0.41,61.7	0
0.41,61.8	0
0.41,61.9	0
0.41,62.0	0
0.41,62.1	0
0.41,62.2	0
0.41,62.3	0
0.41,62.4	0
0.41,62.5	0
0.41,62.6	0
0.41,62.7	0
0.41,62.8	0
0.41,62.9	0
0.41,63.0	0
0.41,63.1	0
0.41,63.2	0
0.41,63.3	0
0.41,63.4	0
0.41,63.5	0
0.41,63.6	0
0.41,63.7	0
0.41,63.8	0
0.41,63.9	0
0.41,64.0	0
0.41,64.1	0
0.41,64.2	0
0.41,64.3	0
0.41,64.4	0
0.41,64.5	0
0.41,64.6	0
0.41,64.7	0
0.41,64.8	0
0.41,64.9	0
0.41,65.0	0
0.41,65.1	0
0.41,65.2	0
0.41,65.3	0
0.41,65.4	0
0.41,65.5	0
0.41,65.6	0

0.41,65.7	0
0.41,65.8	0
0.41,65.9	0
0.41,66.0	0
0.41,66.1	0
0.41,66.2	0
0.41,66.3	0
0.41,66.4	0
0.41,66.5	0
0.41,66.6	0
0.41,66.7	0
0.41,66.8	0
0.41,66.9	0
0.41,67.0	0
0.41,67.1	0
0.41,67.2	0
0.41,67.3	0
0.41,67.4	0
0.41,67.5	0
0.41,67.6	0
0.41,67.7	0
0.41,67.8	0
0.41,67.9	0
0.41,68.0	0
0.41,68.1	0
0.41,68.2	0
0.41,68.3	0
0.41,68.4	0
0.41,68.5	0
0.41,68.6	0
0.41,68.7	0
0.41,68.8	0
0.41,68.9	0
0.41,69.0	0
0.41,69.1	0
0.41,69.2	0
0.41,69.3	0
0.41,69.4	0
0.41,69.5	0
0.41,69.6	0
0.41,69.7	0
0.41,69.8	0

0.41,69.9	0
0.41,70.0	0
0.41,70.1	0
0.41,70.2	0
0.41,70.3	0
0.41,70.4	0
0.41,70.5	0
0.41,70.6	0
0.41,70.7	0
0.41,70.8	0
0.41,70.9	0
0.41,71.0	0
0.41,71.1	0
0.41,71.2	0
0.41,71.3	0
0.41,71.4	0
0.41,71.5	0
0.41,71.6	0
0.41,71.7	0
0.41,71.8	0
0.41,71.9	0
0.41,72.0	0
0.41,72.1	0
0.41,72.2	0
0.41,72.3	0
0.41,72.4	0
0.41,72.5	0
0.41,72.6	0
0.41,72.7	0
0.41,72.8	0
0.41,72.9	0
0.41,73.0	0
0.41,73.1	0
0.41,73.2	0
0.41,73.3	0
0.41,73.4	0
0.41,73.5	0
0.41,73.6	0
0.41,73.7	0
0.41,73.8	0
0.41,73.9	0
0.41,74.0	0

0.41,74.1	0
0.41,74.2	0
0.41,74.3	0
0.41,74.4	0
0.41,74.5	0
0.41,74.6	0
0.41,74.7	0
0.41,74.8	0
0.41,74.9	0
0.41,75.0	0
0.41,75.1	0
0.41,75.2	0
0.41,75.3	0
0.41,75.4	0
0.41,75.5	0
0.41,75.6	0
0.41,75.7	0
0.41,75.8	0
0.41,75.9	0
0.41,76.0	0
0.41,76.1	0
0.41,76.2	0
0.41,76.3	0
0.41,76.4	0
0.41,76.5	0
0.41,76.6	0
0.41,76.7	0
0.41,76.8	0
0.41,76.9	0
0.41,77.0	0
0.41,77.1	0
0.41,77.2	0
0.41,77.3	0
0.41,77.4	0
0.41,77.5	0
0.41,77.6	0
0.41,77.7	0
0.41,77.8	0
0.41,77.9	0
0.41,78.0	0
0.41,78.1	0
0.41,78.2	0

0.41,78.3	0
0.41,78.4	0
0.41,78.5	0
0.41,78.6	0
0.41,78.7	0
0.41,78.8	0
0.41,78.9	0
0.41,79.0	0
0.41,79.1	0
0.41,79.2	0
0.41,79.3	0
0.41,79.4	0
0.41,79.5	0
0.41,79.6	0
0.41,79.7	0
0.41,79.8	0
0.41,79.9	0
0.41,80.0	0
0.41,80.1	0
0.41,80.2	0
0.41,80.3	0
0.41,80.4	0
0.41,80.5	0
0.41,80.6	0
0.41,80.7	0
0.41,80.8	0
0.41,80.9	0
0.41,81.0	0
0.41,81.1	0
0.41,81.2	0
0.41,81.3	0
0.41,81.4	0
0.41,81.5	0
0.41,81.6	0
0.41,81.7	0
0.41,81.8	0
0.41,81.9	0
0.41,82.0	0
0.41,82.1	0
0.41,82.2	0
0.41,82.3	0
0.41,82.4	0

0.41,82.5	0
0.41,82.6	0
0.41,82.7	0
0.41,82.8	0
0.41,82.9	0
0.41,83.0	0
0.41,83.1	0
0.41,83.2	0
0.41,83.3	0
0.41,83.4	0
0.41,83.5	0
0.41,83.6	0
0.41,83.7	0
0.41,83.8	0
0.41,83.9	0
0.41,84.0	0
0.41,84.1	0
0.41,84.2	0
0.41,84.3	0
0.41,84.4	0
0.41,84.5	0
0.41,84.6	0
0.41,84.7	0
0.41,84.8	0
0.41,84.9	0
0.41,85.0	0
0.41,85.1	0
0.41,85.2	0
0.41,85.3	0
0.41,85.4	0
0.41,85.5	0
0.41,85.6	0
0.41,85.7	0
0.41,85.8	0
0.41,85.9	0
0.41,86.0	0
0.41,86.1	0
0.41,86.2	0
0.41,86.3	0
0.41,86.4	0
0.41,86.5	0
0.41,86.6	0

0.41,86.7	0
0.41,86.8	0
0.41,86.9	0
0.41,87.0	0
0.41,87.1	0
0.41,87.2	0
0.41,87.3	0
0.41,87.4	0
0.41,87.5	0
0.41,87.6	0
0.41,87.7	0
0.41,87.8	0
0.41,87.9	0
0.41,88.0	0
0.41,88.1	0
0.41,88.2	0
0.41,88.3	0
0.41,88.4	0
0.41,88.5	0
0.41,88.6	0
0.41,88.7	0
0.41,88.8	0
0.41,88.9	0
0.41,89.0	0
0.41,89.1	0
0.41,89.2	0
0.41,89.3	0
0.41,89.4	0
0.41,89.5	0
0.41,89.6	0
0.41,89.7	0
0.41,89.8	0
0.41,89.9	0
0.41,90.0	0
0.41,90.1	0
0.41,90.2	0
0.41,90.3	0
0.41,90.4	0
0.41,90.5	0
0.41,90.6	0
0.41,90.7	0
0.41,90.8	0

0.41,90.9	0
0.41,91.0	0
0.41,91.1	0
0.41,91.2	0
0.41,91.3	0
0.41,91.4	0
0.41,91.5	0
0.41,91.6	0
0.41,91.7	0
0.41,91.8	0
0.41,91.9	0
0.41,92.0	0
0.41,92.1	0
0.41,92.2	0
0.41,92.3	0
0.41,92.4	0
0.41,92.5	0
0.41,92.6	0
0.41,92.7	0
0.41,92.8	0
0.41,92.9	0
0.41,93.0	0
0.41,93.1	0
0.41,93.2	0
0.41,93.3	0
0.41,93.4	0
0.41,93.5	0
0.41,93.6	0
0.41,93.7	0
0.41,93.8	0
0.41,93.9	0
0.41,94.0	0
0.41,94.1	0
0.41,94.2	0
0.41,94.3	0
0.41,94.4	0
0.41,94.5	0
0.41,94.6	0
0.41,94.7	0
0.41,94.8	0
0.41,94.9	0
0.41,95.0	0

0.41,95.1	0
0.41,95.2	0
0.41,95.3	0
0.41,95.4	0
0.41,95.5	0
0.41,95.6	0
0.41,95.7	0
0.41,95.8	0
0.41,95.9	0
0.41,96.0	0
0.41,96.1	0
0.41,96.2	0
0.41,96.3	0
0.41,96.4	0
0.41,96.5	0
0.41,96.6	0
0.41,96.7	0
0.41,96.8	0
0.41,96.9	0
0.41,97.0	0
0.41,97.1	0
0.41,97.2	0
0.41,97.3	0
0.41,97.4	0
0.41,97.5	0
0.41,97.6	0
0.41,97.7	0
0.41,97.8	0
0.41,97.9	0
0.41,98.0	0
0.41,98.1	0
0.41,98.2	0
0.41,98.3	0
0.41,98.4	0
0.41,98.5	0
0.41,98.6	0
0.41,98.7	0
0.41,98.8	0
0.41,98.9	0
0.41,99.0	0
0.41,99.1	0
0.41,99.2	0

0.41,99.3	0
0.41,99.4	0
0.41,99.5	0
0.41,99.6	0
0.41,99.7	0
0.41,99.8	0
0.41,99.9	0
0.41,100.0	0
0.42,0.0	0
0.42,0.1	0
0.42,0.2	0
0.42,0.3	0
0.42,0.4	0
0.42,0.5	0
0.42,0.6	0
0.42,0.7	0
0.42,0.8	0
0.42,0.9	0
0.42,1.0	0
0.42,1.1	0
0.42,1.2	0
0.42,1.3	0
0.42,1.4	0
0.42,1.5	0
0.42,1.6	0
0.42,1.7	0
0.42,1.8	0
0.42,1.9	0
0.42,2.0	0
0.42,2.1	0
0.42,2.2	0
0.42,2.3	0
0.42,2.4	0
0.42,2.5	0
0.42,2.6	0
0.42,2.7	0
0.42,2.8	0
0.42,2.9	0
0.42,3.0	0
0.42,3.1	0
0.42,3.2	0
0.42,3.3	0

0.42,3.4	0
0.42,3.5	0
0.42,3.6	0
0.42,3.7	0
0.42,3.8	0
0.42,3.9	0
0.42,4.0	0
0.42,4.1	0
0.42,4.2	0
0.42,4.3	0
0.42,4.4	0
0.42,4.5	0
0.42,4.6	0
0.42,4.7	0
0.42,4.8	0
0.42,4.9	0
0.42,5.0	0
0.42,5.1	0
0.42,5.2	0
0.42,5.3	0
0.42,5.4	0
0.42,5.5	0
0.42,5.6	0
0.42,5.7	0
0.42,5.8	0
0.42,5.9	0
0.42,6.0	0
0.42,6.1	0
0.42,6.2	0
0.42,6.3	0
0.42,6.4	0
0.42,6.5	0
0.42,6.6	0
0.42,6.7	0
0.42,6.8	0
0.42,6.9	0
0.42,7.0	0
0.42,7.1	0
0.42,7.2	0
0.42,7.3	0
0.42,7.4	0
0.42,7.5	0

0.42,7.6	0
0.42,7.7	0
0.42,7.8	0
0.42,7.9	0
0.42,8.0	0
0.42,8.1	0
0.42,8.2	0
0.42,8.3	0
0.42,8.4	0
0.42,8.5	0
0.42,8.6	0
0.42,8.7	0
0.42,8.8	0
0.42,8.9	0
0.42,9.0	0
0.42,9.1	0
0.42,9.2	0
0.42,9.3	0
0.42,9.4	0
0.42,9.5	0
0.42,9.6	0
0.42,9.7	0
0.42,9.8	0
0.42,9.9	0
0.42,10.0	0
0.42,10.1	0
0.42,10.2	0
0.42,10.3	0
0.42,10.4	0
0.42,10.5	0
0.42,10.6	0
0.42,10.7	0
0.42,10.8	0
0.42,10.9	0
0.42,11.0	0
0.42,11.1	0
0.42,11.2	0
0.42,11.3	0
0.42,11.4	0
0.42,11.5	0
0.42,11.6	0
0.42,11.7	0

0.42,11.8	0
0.42,11.9	0
0.42,12.0	0
0.42,12.1	0
0.42,12.2	0
0.42,12.3	0
0.42,12.4	0
0.42,12.5	0
0.42,12.6	0
0.42,12.7	0
0.42,12.8	0
0.42,12.9	0
0.42,13.0	0
0.42,13.1	0
0.42,13.2	0
0.42,13.3	0
0.42,13.4	0
0.42,13.5	0
0.42,13.6	0
0.42,13.7	0
0.42,13.8	0
0.42,13.9	0
0.42,14.0	0
0.42,14.1	0
0.42,14.2	0
0.42,14.3	0
0.42,14.4	0
0.42,14.5	0
0.42,14.6	0
0.42,14.7	0
0.42,14.8	0
0.42,14.9	0
0.42,15.0	0
0.42,15.1	0
0.42,15.2	0
0.42,15.3	0
0.42,15.4	0
0.42,15.5	0
0.42,15.6	0
0.42,15.7	0
0.42,15.8	0
0.42,15.9	0

0.42,16.0	0
0.42,16.1	0
0.42,16.2	0
0.42,16.3	0
0.42,16.4	0
0.42,16.5	0
0.42,16.6	0
0.42,16.7	0
0.42,16.8	0
0.42,16.9	0
0.42,17.0	0
0.42,17.1	0
0.42,17.2	0
0.42,17.3	0
0.42,17.4	0
0.42,17.5	0
0.42,17.6	0
0.42,17.7	0
0.42,17.8	0
0.42,17.9	0
0.42,18.0	0
0.42,18.1	0
0.42,18.2	0
0.42,18.3	0
0.42,18.4	0
0.42,18.5	0
0.42,18.6	0
0.42,18.7	0
0.42,18.8	0
0.42,18.9	0
0.42,19.0	0
0.42,19.1	0
0.42,19.2	0
0.42,19.3	0
0.42,19.4	0
0.42,19.5	0
0.42,19.6	0
0.42,19.7	0
0.42,19.8	0
0.42,19.9	0
0.42,20.0	0
0.42,20.1	0

0.42,20.2	0
0.42,20.3	0
0.42,20.4	0
0.42,20.5	0
0.42,20.6	0
0.42,20.7	0
0.42,20.8	0
0.42,20.9	0
0.42,21.0	0
0.42,21.1	0
0.42,21.2	0
0.42,21.3	0
0.42,21.4	0
0.42,21.5	0
0.42,21.6	0
0.42,21.7	0
0.42,21.8	0
0.42,21.9	0
0.42,22.0	0
0.42,22.1	0
0.42,22.2	0
0.42,22.3	0
0.42,22.4	0
0.42,22.5	0
0.42,22.6	0
0.42,22.7	0
0.42,22.8	0
0.42,22.9	0
0.42,23.0	0
0.42,23.1	0
0.42,23.2	0
0.42,23.3	0
0.42,23.4	0
0.42,23.5	0
0.42,23.6	0
0.42,23.7	0
0.42,23.8	0
0.42,23.9	0
0.42,24.0	0
0.42,24.1	0
0.42,24.2	0
0.42,24.3	0

0.42,24.4	0
0.42,24.5	0
0.42,24.6	0
0.42,24.7	0
0.42,24.8	0
0.42,24.9	0
0.42,25.0	0
0.42,25.1	0
0.42,25.2	0
0.42,25.3	0
0.42,25.4	0
0.42,25.5	0
0.42,25.6	0
0.42,25.7	0
0.42,25.8	0
0.42,25.9	0
0.42,26.0	0
0.42,26.1	0
0.42,26.2	0
0.42,26.3	0
0.42,26.4	0
0.42,26.5	0
0.42,26.6	0
0.42,26.7	0
0.42,26.8	0
0.42,26.9	0
0.42,27.0	0
0.42,27.1	0
0.42,27.2	0
0.42,27.3	0
0.42,27.4	0
0.42,27.5	0
0.42,27.6	0
0.42,27.7	0
0.42,27.8	0
0.42,27.9	0
0.42,28.0	0
0.42,28.1	0
0.42,28.2	0
0.42,28.3	0
0.42,28.4	0
0.42,28.5	0

0.42,28.6	0
0.42,28.7	0
0.42,28.8	0
0.42,28.9	0
0.42,29.0	0
0.42,29.1	0
0.42,29.2	0
0.42,29.3	0
0.42,29.4	0
0.42,29.5	0
0.42,29.6	0
0.42,29.7	0
0.42,29.8	0
0.42,29.9	0
0.42,30.0	0
0.42,30.1	0
0.42,30.2	0
0.42,30.3	0
0.42,30.4	0
0.42,30.5	0
0.42,30.6	0
0.42,30.7	0
0.42,30.8	0
0.42,30.9	0
0.42,31.0	0
0.42,31.1	0
0.42,31.2	0
0.42,31.3	0
0.42,31.4	0
0.42,31.5	0
0.42,31.6	0
0.42,31.7	0
0.42,31.8	0
0.42,31.9	0
0.42,32.0	0
0.42,32.1	0
0.42,32.2	0
0.42,32.3	0
0.42,32.4	0
0.42,32.5	0
0.42,32.6	0
0.42,32.7	0

0.42,32.8	0
0.42,32.9	0
0.42,33.0	0
0.42,33.1	0
0.42,33.2	0
0.42,33.3	0
0.42,33.4	0
0.42,33.5	0
0.42,33.6	0
0.42,33.7	0
0.42,33.8	0
0.42,33.9	0
0.42,34.0	0
0.42,34.1	0
0.42,34.2	0
0.42,34.3	0
0.42,34.4	0
0.42,34.5	0
0.42,34.6	0
0.42,34.7	0
0.42,34.8	0
0.42,34.9	0
0.42,35.0	0
0.42,35.1	0
0.42,35.2	0
0.42,35.3	0
0.42,35.4	0
0.42,35.5	0
0.42,35.6	0
0.42,35.7	0
0.42,35.8	0
0.42,35.9	0
0.42,36.0	0
0.42,36.1	0
0.42,36.2	0
0.42,36.3	0
0.42,36.4	0
0.42,36.5	0
0.42,36.6	0
0.42,36.7	0
0.42,36.8	0
0.42,36.9	0

0.42,37.0	0
0.42,37.1	0
0.42,37.2	0
0.42,37.3	0
0.42,37.4	0
0.42,37.5	0
0.42,37.6	0
0.42,37.7	0
0.42,37.8	0
0.42,37.9	0
0.42,38.0	0
0.42,38.1	0
0.42,38.2	0
0.42,38.3	0
0.42,38.4	0
0.42,38.5	0
0.42,38.6	0
0.42,38.7	0
0.42,38.8	0
0.42,38.9	0
0.42,39.0	0
0.42,39.1	0
0.42,39.2	0
0.42,39.3	0
0.42,39.4	0
0.42,39.5	0
0.42,39.6	0
0.42,39.7	0
0.42,39.8	0
0.42,39.9	0
0.42,40.0	0
0.42,40.1	0
0.42,40.2	0
0.42,40.3	0
0.42,40.4	0
0.42,40.5	0
0.42,40.6	0
0.42,40.7	0
0.42,40.8	0
0.42,40.9	0
0.42,41.0	0
0.42,41.1	0

0.42,41.2	0
0.42,41.3	0
0.42,41.4	0
0.42,41.5	0
0.42,41.6	0
0.42,41.7	0
0.42,41.8	0
0.42,41.9	0
0.42,42.0	0
0.42,42.1	0
0.42,42.2	0
0.42,42.3	0
0.42,42.4	0
0.42,42.5	0
0.42,42.6	0
0.42,42.7	0
0.42,42.8	0
0.42,42.9	0
0.42,43.0	0
0.42,43.1	0
0.42,43.2	0
0.42,43.3	0
0.42,43.4	0
0.42,43.5	0
0.42,43.6	0
0.42,43.7	0
0.42,43.8	0
0.42,43.9	0
0.42,44.0	0
0.42,44.1	0
0.42,44.2	0
0.42,44.3	0
0.42,44.4	0
0.42,44.5	0
0.42,44.6	0
0.42,44.7	0
0.42,44.8	0
0.42,44.9	0
0.42,45.0	0
0.42,45.1	0
0.42,45.2	0
0.42,45.3	0

0.42,45.4	0
0.42,45.5	0
0.42,45.6	0
0.42,45.7	0
0.42,45.8	0
0.42,45.9	0
0.42,46.0	0
0.42,46.1	0
0.42,46.2	0
0.42,46.3	0
0.42,46.4	0
0.42,46.5	0
0.42,46.6	0
0.42,46.7	0
0.42,46.8	0
0.42,46.9	0
0.42,47.0	0
0.42,47.1	0
0.42,47.2	0
0.42,47.3	0
0.42,47.4	0
0.42,47.5	0
0.42,47.6	0
0.42,47.7	0
0.42,47.8	0
0.42,47.9	0
0.42,48.0	0
0.42,48.1	0
0.42,48.2	0
0.42,48.3	0
0.42,48.4	0
0.42,48.5	0
0.42,48.6	0
0.42,48.7	0
0.42,48.8	0
0.42,48.9	0
0.42,49.0	0
0.42,49.1	0
0.42,49.2	0
0.42,49.3	0
0.42,49.4	0
0.42,49.5	0

0.42,49.6	0
0.42,49.7	0
0.42,49.8	0
0.42,49.9	0
0.42,50.0	0
0.42,50.1	0
0.42,50.2	0
0.42,50.3	0
0.42,50.4	0
0.42,50.5	0
0.42,50.6	0
0.42,50.7	0
0.42,50.8	0
0.42,50.9	0
0.42,51.0	0
0.42,51.1	0
0.42,51.2	0
0.42,51.3	0
0.42,51.4	0
0.42,51.5	0
0.42,51.6	0
0.42,51.7	0
0.42,51.8	0
0.42,51.9	0
0.42,52.0	0
0.42,52.1	0
0.42,52.2	0
0.42,52.3	0
0.42,52.4	0
0.42,52.5	0
0.42,52.6	0
0.42,52.7	0
0.42,52.8	0
0.42,52.9	0
0.42,53.0	0
0.42,53.1	0
0.42,53.2	0
0.42,53.3	0
0.42,53.4	0
0.42,53.5	0
0.42,53.6	0
0.42,53.7	0

0.42,53.8	0
0.42,53.9	0
0.42,54.0	0
0.42,54.1	0
0.42,54.2	0
0.42,54.3	0
0.42,54.4	0
0.42,54.5	0
0.42,54.6	0
0.42,54.7	0
0.42,54.8	0
0.42,54.9	0
0.42,55.0	0
0.42,55.1	0
0.42,55.2	0
0.42,55.3	0
0.42,55.4	0
0.42,55.5	0
0.42,55.6	0
0.42,55.7	0
0.42,55.8	0
0.42,55.9	0
0.42,56.0	0
0.42,56.1	0
0.42,56.2	0
0.42,56.3	0
0.42,56.4	0
0.42,56.5	0
0.42,56.6	0
0.42,56.7	0
0.42,56.8	0
0.42,56.9	0
0.42,57.0	0
0.42,57.1	0
0.42,57.2	0
0.42,57.3	0
0.42,57.4	0
0.42,57.5	0
0.42,57.6	0
0.42,57.7	0
0.42,57.8	0
0.42,57.9	0

0.42,58.0	0
0.42,58.1	0
0.42,58.2	0
0.42,58.3	0
0.42,58.4	0
0.42,58.5	0
0.42,58.6	0
0.42,58.7	0
0.42,58.8	0
0.42,58.9	0
0.42,59.0	0
0.42,59.1	0
0.42,59.2	0
0.42,59.3	0
0.42,59.4	0
0.42,59.5	0
0.42,59.6	0
0.42,59.7	0
0.42,59.8	0
0.42,59.9	0
0.42,60.0	0
0.42,60.1	0
0.42,60.2	0
0.42,60.3	0
0.42,60.4	0
0.42,60.5	0
0.42,60.6	0
0.42,60.7	0
0.42,60.8	0
0.42,60.9	0
0.42,61.0	0
0.42,61.1	0
0.42,61.2	0
0.42,61.3	0
0.42,61.4	0
0.42,61.5	0
0.42,61.6	0
0.42,61.7	0
0.42,61.8	0
0.42,61.9	0
0.42,62.0	0
0.42,62.1	0

0.42,62.2	0
0.42,62.3	0
0.42,62.4	0
0.42,62.5	0
0.42,62.6	0
0.42,62.7	0
0.42,62.8	0
0.42,62.9	0
0.42,63.0	0
0.42,63.1	0
0.42,63.2	0
0.42,63.3	0
0.42,63.4	0
0.42,63.5	0
0.42,63.6	0
0.42,63.7	0
0.42,63.8	0
0.42,63.9	0
0.42,64.0	0
0.42,64.1	0
0.42,64.2	0
0.42,64.3	0
0.42,64.4	0
0.42,64.5	0
0.42,64.6	0
0.42,64.7	0
0.42,64.8	0
0.42,64.9	0
0.42,65.0	0
0.42,65.1	0
0.42,65.2	0
0.42,65.3	0
0.42,65.4	0
0.42,65.5	0
0.42,65.6	0
0.42,65.7	0
0.42,65.8	0
0.42,65.9	0
0.42,66.0	0
0.42,66.1	0
0.42,66.2	0
0.42,66.3	0

0.42,66.4	0
0.42,66.5	0
0.42,66.6	0
0.42,66.7	0
0.42,66.8	0
0.42,66.9	0
0.42,67.0	0
0.42,67.1	0
0.42,67.2	0
0.42,67.3	0
0.42,67.4	0
0.42,67.5	0
0.42,67.6	0
0.42,67.7	0
0.42,67.8	0
0.42,67.9	0
0.42,68.0	0
0.42,68.1	0
0.42,68.2	0
0.42,68.3	0
0.42,68.4	0
0.42,68.5	0
0.42,68.6	0
0.42,68.7	0
0.42,68.8	0
0.42,68.9	0
0.42,69.0	0
0.42,69.1	0
0.42,69.2	0
0.42,69.3	0
0.42,69.4	0
0.42,69.5	0
0.42,69.6	0
0.42,69.7	0
0.42,69.8	0
0.42,69.9	0
0.42,70.0	0
0.42,70.1	0
0.42,70.2	0
0.42,70.3	0
0.42,70.4	0
0.42,70.5	0

0.42,70.6	0
0.42,70.7	0
0.42,70.8	0
0.42,70.9	0
0.42,71.0	0
0.42,71.1	0
0.42,71.2	0
0.42,71.3	0
0.42,71.4	0
0.42,71.5	0
0.42,71.6	0
0.42,71.7	0
0.42,71.8	0
0.42,71.9	0
0.42,72.0	0
0.42,72.1	0
0.42,72.2	0
0.42,72.3	0
0.42,72.4	0
0.42,72.5	0
0.42,72.6	0
0.42,72.7	0
0.42,72.8	0
0.42,72.9	0
0.42,73.0	0
0.42,73.1	0
0.42,73.2	0
0.42,73.3	0
0.42,73.4	0
0.42,73.5	0
0.42,73.6	0
0.42,73.7	0
0.42,73.8	0
0.42,73.9	0
0.42,74.0	0
0.42,74.1	0
0.42,74.2	0
0.42,74.3	0
0.42,74.4	0
0.42,74.5	0
0.42,74.6	0
0.42,74.7	0

0.42,74.8	0
0.42,74.9	0
0.42,75.0	0
0.42,75.1	0
0.42,75.2	0
0.42,75.3	0
0.42,75.4	0
0.42,75.5	0
0.42,75.6	0
0.42,75.7	0
0.42,75.8	0
0.42,75.9	0
0.42,76.0	0
0.42,76.1	0
0.42,76.2	0
0.42,76.3	0
0.42,76.4	0
0.42,76.5	0
0.42,76.6	0
0.42,76.7	0
0.42,76.8	0
0.42,76.9	0
0.42,77.0	0
0.42,77.1	0
0.42,77.2	0
0.42,77.3	0
0.42,77.4	0
0.42,77.5	0
0.42,77.6	0
0.42,77.7	0
0.42,77.8	0
0.42,77.9	0
0.42,78.0	0
0.42,78.1	0
0.42,78.2	0
0.42,78.3	0
0.42,78.4	0
0.42,78.5	0
0.42,78.6	0
0.42,78.7	0
0.42,78.8	0
0.42,78.9	0

0.42,79.0	0
0.42,79.1	0
0.42,79.2	0
0.42,79.3	0
0.42,79.4	0
0.42,79.5	0
0.42,79.6	0
0.42,79.7	0
0.42,79.8	0
0.42,79.9	0
0.42,80.0	0
0.42,80.1	0
0.42,80.2	0
0.42,80.3	0
0.42,80.4	0
0.42,80.5	0
0.42,80.6	0
0.42,80.7	0
0.42,80.8	0
0.42,80.9	0
0.42,81.0	0
0.42,81.1	0
0.42,81.2	0
0.42,81.3	0
0.42,81.4	0
0.42,81.5	0
0.42,81.6	0
0.42,81.7	0
0.42,81.8	0
0.42,81.9	0
0.42,82.0	0
0.42,82.1	0
0.42,82.2	0
0.42,82.3	0
0.42,82.4	0
0.42,82.5	0
0.42,82.6	0
0.42,82.7	0
0.42,82.8	0
0.42,82.9	0
0.42,83.0	0
0.42,83.1	0

0.42,83.2	0
0.42,83.3	0
0.42,83.4	0
0.42,83.5	0
0.42,83.6	0
0.42,83.7	0
0.42,83.8	0
0.42,83.9	0
0.42,84.0	0
0.42,84.1	0
0.42,84.2	0
0.42,84.3	0
0.42,84.4	0
0.42,84.5	0
0.42,84.6	0
0.42,84.7	0
0.42,84.8	0
0.42,84.9	0
0.42,85.0	0
0.42,85.1	0
0.42,85.2	0
0.42,85.3	0
0.42,85.4	0
0.42,85.5	0
0.42,85.6	0
0.42,85.7	0
0.42,85.8	0
0.42,85.9	0
0.42,86.0	0
0.42,86.1	0
0.42,86.2	0
0.42,86.3	0
0.42,86.4	0
0.42,86.5	0
0.42,86.6	0
0.42,86.7	0
0.42,86.8	0
0.42,86.9	0
0.42,87.0	0
0.42,87.1	0
0.42,87.2	0
0.42,87.3	0

0.42,87.4	0
0.42,87.5	0
0.42,87.6	0
0.42,87.7	0
0.42,87.8	0
0.42,87.9	0
0.42,88.0	0
0.42,88.1	0
0.42,88.2	0
0.42,88.3	0
0.42,88.4	0
0.42,88.5	0
0.42,88.6	0
0.42,88.7	0
0.42,88.8	0
0.42,88.9	0
0.42,89.0	0
0.42,89.1	0
0.42,89.2	0
0.42,89.3	0
0.42,89.4	0
0.42,89.5	0
0.42,89.6	0
0.42,89.7	0
0.42,89.8	0
0.42,89.9	0
0.42,90.0	0
0.42,90.1	0
0.42,90.2	0
0.42,90.3	0
0.42,90.4	0
0.42,90.5	0
0.42,90.6	0
0.42,90.7	0
0.42,90.8	0
0.42,90.9	0
0.42,91.0	0
0.42,91.1	0
0.42,91.2	0
0.42,91.3	0
0.42,91.4	0
0.42,91.5	0

0.42,91.6	0
0.42,91.7	0
0.42,91.8	0
0.42,91.9	0
0.42,92.0	0
0.42,92.1	0
0.42,92.2	0
0.42,92.3	0
0.42,92.4	0
0.42,92.5	0
0.42,92.6	0
0.42,92.7	0
0.42,92.8	0
0.42,92.9	0
0.42,93.0	0
0.42,93.1	0
0.42,93.2	0
0.42,93.3	0
0.42,93.4	0
0.42,93.5	0
0.42,93.6	0
0.42,93.7	0
0.42,93.8	0
0.42,93.9	0
0.42,94.0	0
0.42,94.1	0
0.42,94.2	0
0.42,94.3	0
0.42,94.4	0
0.42,94.5	0
0.42,94.6	0
0.42,94.7	0
0.42,94.8	0
0.42,94.9	0
0.42,95.0	0
0.42,95.1	0
0.42,95.2	0
0.42,95.3	0
0.42,95.4	0
0.42,95.5	0
0.42,95.6	0
0.42,95.7	0

0.42,95.8	0
0.42,95.9	0
0.42,96.0	0
0.42,96.1	0
0.42,96.2	0
0.42,96.3	0
0.42,96.4	0
0.42,96.5	0
0.42,96.6	0
0.42,96.7	0
0.42,96.8	0
0.42,96.9	0
0.42,97.0	0
0.42,97.1	0
0.42,97.2	0
0.42,97.3	0
0.42,97.4	0
0.42,97.5	0
0.42,97.6	0
0.42,97.7	0
0.42,97.8	0
0.42,97.9	0
0.42,98.0	0
0.42,98.1	0
0.42,98.2	0
0.42,98.3	0
0.42,98.4	0
0.42,98.5	0
0.42,98.6	0
0.42,98.7	0
0.42,98.8	0
0.42,98.9	0
0.42,99.0	0
0.42,99.1	0
0.42,99.2	0
0.42,99.3	0
0.42,99.4	0
0.42,99.5	0
0.42,99.6	0
0.42,99.7	0
0.42,99.8	0
0.42,99.9	0

0.42,100.0	0
0.43,0.0	0
0.43,0.1	0
0.43,0.2	0
0.43,0.3	0
0.43,0.4	0
0.43,0.5	0
0.43,0.6	0
0.43,0.7	0
0.43,0.8	0
0.43,0.9	0
0.43,1.0	0
0.43,1.1	0
0.43,1.2	0
0.43,1.3	0
0.43,1.4	0
0.43,1.5	0
0.43,1.6	0
0.43,1.7	0
0.43,1.8	0
0.43,1.9	0
0.43,2.0	0
0.43,2.1	0
0.43,2.2	0
0.43,2.3	0
0.43,2.4	0
0.43,2.5	0
0.43,2.6	0
0.43,2.7	0
0.43,2.8	0
0.43,2.9	0
0.43,3.0	0
0.43,3.1	0
0.43,3.2	0
0.43,3.3	0
0.43,3.4	0
0.43,3.5	0
0.43,3.6	0
0.43,3.7	0
0.43,3.8	0
0.43,3.9	0
0.43,4.0	0

0.43,4.1	0
0.43,4.2	0
0.43,4.3	0
0.43,4.4	0
0.43,4.5	0
0.43,4.6	0
0.43,4.7	0
0.43,4.8	0
0.43,4.9	0
0.43,5.0	0
0.43,5.1	0
0.43,5.2	0
0.43,5.3	0
0.43,5.4	0
0.43,5.5	0
0.43,5.6	0
0.43,5.7	0
0.43,5.8	0
0.43,5.9	0
0.43,6.0	0
0.43,6.1	0
0.43,6.2	0
0.43,6.3	0
0.43,6.4	0
0.43,6.5	0
0.43,6.6	0
0.43,6.7	0
0.43,6.8	0
0.43,6.9	0
0.43,7.0	0
0.43,7.1	0
0.43,7.2	0
0.43,7.3	0
0.43,7.4	0
0.43,7.5	0
0.43,7.6	0
0.43,7.7	0
0.43,7.8	0
0.43,7.9	0
0.43,8.0	0
0.43,8.1	0
0.43,8.2	0

0.43,8.3	0
0.43,8.4	0
0.43,8.5	0
0.43,8.6	0
0.43,8.7	0
0.43,8.8	0
0.43,8.9	0
0.43,9.0	0
0.43,9.1	0
0.43,9.2	0
0.43,9.3	0
0.43,9.4	0
0.43,9.5	0
0.43,9.6	0
0.43,9.7	0
0.43,9.8	0
0.43,9.9	0
0.43,10.0	0
0.43,10.1	0
0.43,10.2	0
0.43,10.3	0
0.43,10.4	0
0.43,10.5	0
0.43,10.6	0
0.43,10.7	0
0.43,10.8	0
0.43,10.9	0
0.43,11.0	0
0.43,11.1	0
0.43,11.2	0
0.43,11.3	0
0.43,11.4	0
0.43,11.5	0
0.43,11.6	0
0.43,11.7	0
0.43,11.8	0
0.43,11.9	0
0.43,12.0	0
0.43,12.1	0
0.43,12.2	0
0.43,12.3	0
0.43,12.4	0

0.43,12.5	0
0.43,12.6	0
0.43,12.7	0
0.43,12.8	0
0.43,12.9	0
0.43,13.0	0
0.43,13.1	0
0.43,13.2	0
0.43,13.3	0
0.43,13.4	0
0.43,13.5	0
0.43,13.6	0
0.43,13.7	0
0.43,13.8	0
0.43,13.9	0
0.43,14.0	0
0.43,14.1	0
0.43,14.2	0
0.43,14.3	0
0.43,14.4	0
0.43,14.5	0
0.43,14.6	0
0.43,14.7	0
0.43,14.8	0
0.43,14.9	0
0.43,15.0	0
0.43,15.1	0
0.43,15.2	0
0.43,15.3	0
0.43,15.4	0
0.43,15.5	0
0.43,15.6	0
0.43,15.7	0
0.43,15.8	0
0.43,15.9	0
0.43,16.0	0
0.43,16.1	0
0.43,16.2	0
0.43,16.3	0
0.43,16.4	0
0.43,16.5	0
0.43,16.6	0

0.43,16.7	0
0.43,16.8	0
0.43,16.9	0
0.43,17.0	0
0.43,17.1	0
0.43,17.2	0
0.43,17.3	0
0.43,17.4	0
0.43,17.5	0
0.43,17.6	0
0.43,17.7	0
0.43,17.8	0
0.43,17.9	0
0.43,18.0	0
0.43,18.1	0
0.43,18.2	0
0.43,18.3	0
0.43,18.4	0
0.43,18.5	0
0.43,18.6	0
0.43,18.7	0
0.43,18.8	0
0.43,18.9	0
0.43,19.0	0
0.43,19.1	0
0.43,19.2	0
0.43,19.3	0
0.43,19.4	0
0.43,19.5	0
0.43,19.6	0
0.43,19.7	0
0.43,19.8	0
0.43,19.9	0
0.43,20.0	0
0.43,20.1	0
0.43,20.2	0
0.43,20.3	0
0.43,20.4	0
0.43,20.5	0
0.43,20.6	0
0.43,20.7	0
0.43,20.8	0

0.43,20.9	0
0.43,21.0	0
0.43,21.1	0
0.43,21.2	0
0.43,21.3	0
0.43,21.4	0
0.43,21.5	0
0.43,21.6	0
0.43,21.7	0
0.43,21.8	0
0.43,21.9	0
0.43,22.0	0
0.43,22.1	0
0.43,22.2	0
0.43,22.3	0
0.43,22.4	0
0.43,22.5	0
0.43,22.6	0
0.43,22.7	0
0.43,22.8	0
0.43,22.9	0
0.43,23.0	0
0.43,23.1	0
0.43,23.2	0
0.43,23.3	0
0.43,23.4	0
0.43,23.5	0
0.43,23.6	0
0.43,23.7	0
0.43,23.8	0
0.43,23.9	0
0.43,24.0	0
0.43,24.1	0
0.43,24.2	0
0.43,24.3	0
0.43,24.4	0
0.43,24.5	0
0.43,24.6	0
0.43,24.7	0
0.43,24.8	0
0.43,24.9	0
0.43,25.0	0

0.43,25.1	0
0.43,25.2	0
0.43,25.3	0
0.43,25.4	0
0.43,25.5	0
0.43,25.6	0
0.43,25.7	0
0.43,25.8	0
0.43,25.9	0
0.43,26.0	0
0.43,26.1	0
0.43,26.2	0
0.43,26.3	0
0.43,26.4	0
0.43,26.5	0
0.43,26.6	0
0.43,26.7	0
0.43,26.8	0
0.43,26.9	0
0.43,27.0	0
0.43,27.1	0
0.43,27.2	0
0.43,27.3	0
0.43,27.4	0
0.43,27.5	0
0.43,27.6	0
0.43,27.7	0
0.43,27.8	0
0.43,27.9	0
0.43,28.0	0
0.43,28.1	0
0.43,28.2	0
0.43,28.3	0
0.43,28.4	0
0.43,28.5	0
0.43,28.6	0
0.43,28.7	0
0.43,28.8	0
0.43,28.9	0
0.43,29.0	0
0.43,29.1	0
0.43,29.2	0

0.43,29.3	0
0.43,29.4	0
0.43,29.5	0
0.43,29.6	0
0.43,29.7	0
0.43,29.8	0
0.43,29.9	0
0.43,30.0	0
0.43,30.1	0
0.43,30.2	0
0.43,30.3	0
0.43,30.4	0
0.43,30.5	0
0.43,30.6	0
0.43,30.7	0
0.43,30.8	0
0.43,30.9	0
0.43,31.0	0
0.43,31.1	0
0.43,31.2	0
0.43,31.3	0
0.43,31.4	0
0.43,31.5	0
0.43,31.6	0
0.43,31.7	0
0.43,31.8	0
0.43,31.9	0
0.43,32.0	0
0.43,32.1	0
0.43,32.2	0
0.43,32.3	0
0.43,32.4	0
0.43,32.5	0
0.43,32.6	0
0.43,32.7	0
0.43,32.8	0
0.43,32.9	0
0.43,33.0	0
0.43,33.1	0
0.43,33.2	0
0.43,33.3	0
0.43,33.4	0

0.43,33.5	0
0.43,33.6	0
0.43,33.7	0
0.43,33.8	0
0.43,33.9	0
0.43,34.0	0
0.43,34.1	0
0.43,34.2	0
0.43,34.3	0
0.43,34.4	0
0.43,34.5	0
0.43,34.6	0
0.43,34.7	0
0.43,34.8	0
0.43,34.9	0
0.43,35.0	0
0.43,35.1	0
0.43,35.2	0
0.43,35.3	0
0.43,35.4	0
0.43,35.5	0
0.43,35.6	0
0.43,35.7	0
0.43,35.8	0
0.43,35.9	0
0.43,36.0	0
0.43,36.1	0
0.43,36.2	0
0.43,36.3	0
0.43,36.4	0
0.43,36.5	0
0.43,36.6	0
0.43,36.7	0
0.43,36.8	0
0.43,36.9	0
0.43,37.0	0
0.43,37.1	0
0.43,37.2	0
0.43,37.3	0
0.43,37.4	0
0.43,37.5	0
0.43,37.6	0

0.43,37.7	0
0.43,37.8	0
0.43,37.9	0
0.43,38.0	0
0.43,38.1	0
0.43,38.2	0
0.43,38.3	0
0.43,38.4	0
0.43,38.5	0
0.43,38.6	0
0.43,38.7	0
0.43,38.8	0
0.43,38.9	0
0.43,39.0	0
0.43,39.1	0
0.43,39.2	0
0.43,39.3	0
0.43,39.4	0
0.43,39.5	0
0.43,39.6	0
0.43,39.7	0
0.43,39.8	0
0.43,39.9	0
0.43,40.0	0
0.43,40.1	0
0.43,40.2	0
0.43,40.3	0
0.43,40.4	0
0.43,40.5	0
0.43,40.6	0
0.43,40.7	0
0.43,40.8	0
0.43,40.9	0
0.43,41.0	0
0.43,41.1	0
0.43,41.2	0
0.43,41.3	0
0.43,41.4	0
0.43,41.5	0
0.43,41.6	0
0.43,41.7	0
0.43,41.8	0

0.43,41.9	0
0.43,42.0	0
0.43,42.1	0
0.43,42.2	0
0.43,42.3	0
0.43,42.4	0
0.43,42.5	0
0.43,42.6	0
0.43,42.7	0
0.43,42.8	0
0.43,42.9	0
0.43,43.0	0
0.43,43.1	0
0.43,43.2	0
0.43,43.3	0
0.43,43.4	0
0.43,43.5	0
0.43,43.6	0
0.43,43.7	0
0.43,43.8	0
0.43,43.9	0
0.43,44.0	0
0.43,44.1	0
0.43,44.2	0
0.43,44.3	0
0.43,44.4	0
0.43,44.5	0
0.43,44.6	0
0.43,44.7	0
0.43,44.8	0
0.43,44.9	0
0.43,45.0	0
0.43,45.1	0
0.43,45.2	0
0.43,45.3	0
0.43,45.4	0
0.43,45.5	0
0.43,45.6	0
0.43,45.7	0
0.43,45.8	0
0.43,45.9	0
0.43,46.0	0

0.43,46.1	0
0.43,46.2	0
0.43,46.3	0
0.43,46.4	0
0.43,46.5	0
0.43,46.6	0
0.43,46.7	0
0.43,46.8	0
0.43,46.9	0
0.43,47.0	0
0.43,47.1	0
0.43,47.2	0
0.43,47.3	0
0.43,47.4	0
0.43,47.5	0
0.43,47.6	0
0.43,47.7	0
0.43,47.8	0
0.43,47.9	0
0.43,48.0	0
0.43,48.1	0
0.43,48.2	0
0.43,48.3	0
0.43,48.4	0
0.43,48.5	0
0.43,48.6	0
0.43,48.7	0
0.43,48.8	0
0.43,48.9	0
0.43,49.0	0
0.43,49.1	0
0.43,49.2	0
0.43,49.3	0
0.43,49.4	0
0.43,49.5	0
0.43,49.6	0
0.43,49.7	0
0.43,49.8	0
0.43,49.9	0
0.43,50.0	0
0.43,50.1	0
0.43,50.2	0

0.43,50.3	0
0.43,50.4	0
0.43,50.5	0
0.43,50.6	0
0.43,50.7	0
0.43,50.8	0
0.43,50.9	0
0.43,51.0	0
0.43,51.1	0
0.43,51.2	0
0.43,51.3	0
0.43,51.4	0
0.43,51.5	0
0.43,51.6	0
0.43,51.7	0
0.43,51.8	0
0.43,51.9	0
0.43,52.0	0
0.43,52.1	0
0.43,52.2	0
0.43,52.3	0
0.43,52.4	0
0.43,52.5	0
0.43,52.6	0
0.43,52.7	0
0.43,52.8	0
0.43,52.9	0
0.43,53.0	0
0.43,53.1	0
0.43,53.2	0
0.43,53.3	0
0.43,53.4	0
0.43,53.5	0
0.43,53.6	0
0.43,53.7	0
0.43,53.8	0
0.43,53.9	0
0.43,54.0	0
0.43,54.1	0
0.43,54.2	0
0.43,54.3	0
0.43,54.4	0

0.43,54.5	0
0.43,54.6	0
0.43,54.7	0
0.43,54.8	0
0.43,54.9	0
0.43,55.0	0
0.43,55.1	0
0.43,55.2	0
0.43,55.3	0
0.43,55.4	0
0.43,55.5	0
0.43,55.6	0
0.43,55.7	0
0.43,55.8	0
0.43,55.9	0
0.43,56.0	0
0.43,56.1	0
0.43,56.2	0
0.43,56.3	0
0.43,56.4	0
0.43,56.5	0
0.43,56.6	0
0.43,56.7	0
0.43,56.8	0
0.43,56.9	0
0.43,57.0	0
0.43,57.1	0
0.43,57.2	0
0.43,57.3	0
0.43,57.4	0
0.43,57.5	0
0.43,57.6	0
0.43,57.7	0
0.43,57.8	0
0.43,57.9	0
0.43,58.0	0
0.43,58.1	0
0.43,58.2	0
0.43,58.3	0
0.43,58.4	0
0.43,58.5	0
0.43,58.6	0

0.43,58.7	0
0.43,58.8	0
0.43,58.9	0
0.43,59.0	0
0.43,59.1	0
0.43,59.2	0
0.43,59.3	0
0.43,59.4	0
0.43,59.5	0
0.43,59.6	0
0.43,59.7	0
0.43,59.8	0
0.43,59.9	0
0.43,60.0	0
0.43,60.1	0
0.43,60.2	0
0.43,60.3	0
0.43,60.4	0
0.43,60.5	0
0.43,60.6	0
0.43,60.7	0
0.43,60.8	0
0.43,60.9	0
0.43,61.0	0
0.43,61.1	0
0.43,61.2	0
0.43,61.3	0
0.43,61.4	0
0.43,61.5	0
0.43,61.6	0
0.43,61.7	0
0.43,61.8	0
0.43,61.9	0
0.43,62.0	0
0.43,62.1	0
0.43,62.2	0
0.43,62.3	0
0.43,62.4	0
0.43,62.5	0
0.43,62.6	0
0.43,62.7	0
0.43,62.8	0

0.43,62.9	0
0.43,63.0	0
0.43,63.1	0
0.43,63.2	0
0.43,63.3	0
0.43,63.4	0
0.43,63.5	0
0.43,63.6	0
0.43,63.7	0
0.43,63.8	0
0.43,63.9	0
0.43,64.0	0
0.43,64.1	0
0.43,64.2	0
0.43,64.3	0
0.43,64.4	0
0.43,64.5	0
0.43,64.6	0
0.43,64.7	0
0.43,64.8	0
0.43,64.9	0
0.43,65.0	0
0.43,65.1	0
0.43,65.2	0
0.43,65.3	0
0.43,65.4	0
0.43,65.5	0
0.43,65.6	0
0.43,65.7	0
0.43,65.8	0
0.43,65.9	0
0.43,66.0	0
0.43,66.1	0
0.43,66.2	0
0.43,66.3	0
0.43,66.4	0
0.43,66.5	0
0.43,66.6	0
0.43,66.7	0
0.43,66.8	0
0.43,66.9	0
0.43,67.0	0

0.43,67.1	0
0.43,67.2	0
0.43,67.3	0
0.43,67.4	0
0.43,67.5	0
0.43,67.6	0
0.43,67.7	0
0.43,67.8	0
0.43,67.9	0
0.43,68.0	0
0.43,68.1	0
0.43,68.2	0
0.43,68.3	0
0.43,68.4	0
0.43,68.5	0
0.43,68.6	0
0.43,68.7	0
0.43,68.8	0
0.43,68.9	0
0.43,69.0	0
0.43,69.1	0
0.43,69.2	0
0.43,69.3	0
0.43,69.4	0
0.43,69.5	0
0.43,69.6	0
0.43,69.7	0
0.43,69.8	0
0.43,69.9	0
0.43,70.0	0
0.43,70.1	0
0.43,70.2	0
0.43,70.3	0
0.43,70.4	0
0.43,70.5	0
0.43,70.6	0
0.43,70.7	0
0.43,70.8	0
0.43,70.9	0
0.43,71.0	0
0.43,71.1	0
0.43,71.2	0

0.43,71.3	0
0.43,71.4	0
0.43,71.5	0
0.43,71.6	0
0.43,71.7	0
0.43,71.8	0
0.43,71.9	0
0.43,72.0	0
0.43,72.1	0
0.43,72.2	0
0.43,72.3	0
0.43,72.4	0
0.43,72.5	0
0.43,72.6	0
0.43,72.7	0
0.43,72.8	0
0.43,72.9	0
0.43,73.0	0
0.43,73.1	0
0.43,73.2	0
0.43,73.3	0
0.43,73.4	0
0.43,73.5	0
0.43,73.6	0
0.43,73.7	0
0.43,73.8	0
0.43,73.9	0
0.43,74.0	0
0.43,74.1	0
0.43,74.2	0
0.43,74.3	0
0.43,74.4	0
0.43,74.5	0
0.43,74.6	0
0.43,74.7	0
0.43,74.8	0
0.43,74.9	0
0.43,75.0	0
0.43,75.1	0
0.43,75.2	0
0.43,75.3	0
0.43,75.4	0

0.43,75.5	0
0.43,75.6	0
0.43,75.7	0
0.43,75.8	0
0.43,75.9	0
0.43,76.0	0
0.43,76.1	0
0.43,76.2	0
0.43,76.3	0
0.43,76.4	0
0.43,76.5	0
0.43,76.6	0
0.43,76.7	0
0.43,76.8	0
0.43,76.9	0
0.43,77.0	0
0.43,77.1	0
0.43,77.2	0
0.43,77.3	0
0.43,77.4	0
0.43,77.5	0
0.43,77.6	0
0.43,77.7	0
0.43,77.8	0
0.43,77.9	0
0.43,78.0	0
0.43,78.1	0
0.43,78.2	0
0.43,78.3	0
0.43,78.4	0
0.43,78.5	0
0.43,78.6	0
0.43,78.7	0
0.43,78.8	0
0.43,78.9	0
0.43,79.0	0
0.43,79.1	0
0.43,79.2	0
0.43,79.3	0
0.43,79.4	0
0.43,79.5	0
0.43,79.6	0

0.43,79.7	0
0.43,79.8	0
0.43,79.9	0
0.43,80.0	0
0.43,80.1	0
0.43,80.2	0
0.43,80.3	0
0.43,80.4	0
0.43,80.5	0
0.43,80.6	0
0.43,80.7	0
0.43,80.8	0
0.43,80.9	0
0.43,81.0	0
0.43,81.1	0
0.43,81.2	0
0.43,81.3	0
0.43,81.4	0
0.43,81.5	0
0.43,81.6	0
0.43,81.7	0
0.43,81.8	0
0.43,81.9	0
0.43,82.0	0
0.43,82.1	0
0.43,82.2	0
0.43,82.3	0
0.43,82.4	0
0.43,82.5	0
0.43,82.6	0
0.43,82.7	0
0.43,82.8	0
0.43,82.9	0
0.43,83.0	0
0.43,83.1	0
0.43,83.2	0
0.43,83.3	0
0.43,83.4	0
0.43,83.5	0
0.43,83.6	0
0.43,83.7	0
0.43,83.8	0

0.43,83.9	0
0.43,84.0	0
0.43,84.1	0
0.43,84.2	0
0.43,84.3	0
0.43,84.4	0
0.43,84.5	0
0.43,84.6	0
0.43,84.7	0
0.43,84.8	0
0.43,84.9	0
0.43,85.0	0
0.43,85.1	0
0.43,85.2	0
0.43,85.3	0
0.43,85.4	0
0.43,85.5	0
0.43,85.6	0
0.43,85.7	0
0.43,85.8	0
0.43,85.9	0
0.43,86.0	0
0.43,86.1	0
0.43,86.2	0
0.43,86.3	0
0.43,86.4	0
0.43,86.5	0
0.43,86.6	0
0.43,86.7	0
0.43,86.8	0
0.43,86.9	0
0.43,87.0	0
0.43,87.1	0
0.43,87.2	0
0.43,87.3	0
0.43,87.4	0
0.43,87.5	0
0.43,87.6	0
0.43,87.7	0
0.43,87.8	0
0.43,87.9	0
0.43,88.0	0

0.43,88.1	0
0.43,88.2	0
0.43,88.3	0
0.43,88.4	0
0.43,88.5	0
0.43,88.6	0
0.43,88.7	0
0.43,88.8	0
0.43,88.9	0
0.43,89.0	0
0.43,89.1	0
0.43,89.2	0
0.43,89.3	0
0.43,89.4	0
0.43,89.5	0
0.43,89.6	0
0.43,89.7	0
0.43,89.8	0
0.43,89.9	0
0.43,90.0	0
0.43,90.1	0
0.43,90.2	0
0.43,90.3	0
0.43,90.4	0
0.43,90.5	0
0.43,90.6	0
0.43,90.7	0
0.43,90.8	0
0.43,90.9	0
0.43,91.0	0
0.43,91.1	0
0.43,91.2	0
0.43,91.3	0
0.43,91.4	0
0.43,91.5	0
0.43,91.6	0
0.43,91.7	0
0.43,91.8	0
0.43,91.9	0
0.43,92.0	0
0.43,92.1	0
0.43,92.2	0

0.43,92.3	0
0.43,92.4	0
0.43,92.5	0
0.43,92.6	0
0.43,92.7	0
0.43,92.8	0
0.43,92.9	0
0.43,93.0	0
0.43,93.1	0
0.43,93.2	0
0.43,93.3	0
0.43,93.4	0
0.43,93.5	0
0.43,93.6	0
0.43,93.7	0
0.43,93.8	0
0.43,93.9	0
0.43,94.0	0
0.43,94.1	0
0.43,94.2	0
0.43,94.3	0
0.43,94.4	0
0.43,94.5	0
0.43,94.6	0
0.43,94.7	0
0.43,94.8	0
0.43,94.9	0
0.43,95.0	0
0.43,95.1	0
0.43,95.2	0
0.43,95.3	0
0.43,95.4	0
0.43,95.5	0
0.43,95.6	0
0.43,95.7	0
0.43,95.8	0
0.43,95.9	0
0.43,96.0	0
0.43,96.1	0
0.43,96.2	0
0.43,96.3	0
0.43,96.4	0

0.43,96.5	0
0.43,96.6	0
0.43,96.7	0
0.43,96.8	0
0.43,96.9	0
0.43,97.0	0
0.43,97.1	0
0.43,97.2	0
0.43,97.3	0
0.43,97.4	0
0.43,97.5	0
0.43,97.6	0
0.43,97.7	0
0.43,97.8	0
0.43,97.9	0
0.43,98.0	0
0.43,98.1	0
0.43,98.2	0
0.43,98.3	0
0.43,98.4	0
0.43,98.5	0
0.43,98.6	0
0.43,98.7	0
0.43,98.8	0
0.43,98.9	0
0.43,99.0	0
0.43,99.1	0
0.43,99.2	0
0.43,99.3	0
0.43,99.4	0
0.43,99.5	0
0.43,99.6	0
0.43,99.7	0
0.43,99.8	0
0.43,99.9	0
0.43,100.0	0
0.44,0.0	0
0.44,0.1	0
0.44,0.2	0
0.44,0.3	0
0.44,0.4	0
0.44,0.5	0

0.44,0.6	0
0.44,0.7	0
0.44,0.8	0
0.44,0.9	0
0.44,1.0	0
0.44,1.1	0
0.44,1.2	0
0.44,1.3	0
0.44,1.4	0
0.44,1.5	0
0.44,1.6	0
0.44,1.7	0
0.44,1.8	0
0.44,1.9	0
0.44,2.0	0
0.44,2.1	0
0.44,2.2	0
0.44,2.3	0
0.44,2.4	0
0.44,2.5	0
0.44,2.6	0
0.44,2.7	0
0.44,2.8	0
0.44,2.9	0
0.44,3.0	0
0.44,3.1	0
0.44,3.2	0
0.44,3.3	0
0.44,3.4	0
0.44,3.5	0
0.44,3.6	0
0.44,3.7	0
0.44,3.8	0
0.44,3.9	0
0.44,4.0	0
0.44,4.1	0
0.44,4.2	0
0.44,4.3	0
0.44,4.4	0
0.44,4.5	0
0.44,4.6	0
0.44,4.7	0

0.44,4.8	0
0.44,4.9	0
0.44,5.0	0
0.44,5.1	0
0.44,5.2	0
0.44,5.3	0
0.44,5.4	0
0.44,5.5	0
0.44,5.6	0
0.44,5.7	0
0.44,5.8	0
0.44,5.9	0
0.44,6.0	0
0.44,6.1	0
0.44,6.2	0
0.44,6.3	0
0.44,6.4	0
0.44,6.5	0
0.44,6.6	0
0.44,6.7	0
0.44,6.8	0
0.44,6.9	0
0.44,7.0	0
0.44,7.1	0
0.44,7.2	0
0.44,7.3	0
0.44,7.4	0
0.44,7.5	0
0.44,7.6	0
0.44,7.7	0
0.44,7.8	0
0.44,7.9	0
0.44,8.0	0
0.44,8.1	0
0.44,8.2	0
0.44,8.3	0
0.44,8.4	0
0.44,8.5	0
0.44,8.6	0
0.44,8.7	0
0.44,8.8	0
0.44,8.9	0

0.44,9.0	0
0.44,9.1	0
0.44,9.2	0
0.44,9.3	0
0.44,9.4	0
0.44,9.5	0
0.44,9.6	0
0.44,9.7	0
0.44,9.8	0
0.44,9.9	0
0.44,10.0	0
0.44,10.1	0
0.44,10.2	0
0.44,10.3	0
0.44,10.4	0
0.44,10.5	0
0.44,10.6	0
0.44,10.7	0
0.44,10.8	0
0.44,10.9	0
0.44,11.0	0
0.44,11.1	0
0.44,11.2	0
0.44,11.3	0
0.44,11.4	0
0.44,11.5	0
0.44,11.6	0
0.44,11.7	0
0.44,11.8	0
0.44,11.9	0
0.44,12.0	0
0.44,12.1	0
0.44,12.2	0
0.44,12.3	0
0.44,12.4	0
0.44,12.5	0
0.44,12.6	0
0.44,12.7	0
0.44,12.8	0
0.44,12.9	0
0.44,13.0	0
0.44,13.1	0

0.44,13.2	0
0.44,13.3	0
0.44,13.4	0
0.44,13.5	0
0.44,13.6	0
0.44,13.7	0
0.44,13.8	0
0.44,13.9	0
0.44,14.0	0
0.44,14.1	0
0.44,14.2	0
0.44,14.3	0
0.44,14.4	0
0.44,14.5	0
0.44,14.6	0
0.44,14.7	0
0.44,14.8	0
0.44,14.9	0
0.44,15.0	0
0.44,15.1	0
0.44,15.2	0
0.44,15.3	0
0.44,15.4	0
0.44,15.5	0
0.44,15.6	0
0.44,15.7	0
0.44,15.8	0
0.44,15.9	0
0.44,16.0	0
0.44,16.1	0
0.44,16.2	0
0.44,16.3	0
0.44,16.4	0
0.44,16.5	0
0.44,16.6	0
0.44,16.7	0
0.44,16.8	0
0.44,16.9	0
0.44,17.0	0
0.44,17.1	0
0.44,17.2	0
0.44,17.3	0

0.44,17.4	0
0.44,17.5	0
0.44,17.6	0
0.44,17.7	0
0.44,17.8	0
0.44,17.9	0
0.44,18.0	0
0.44,18.1	0
0.44,18.2	0
0.44,18.3	0
0.44,18.4	0
0.44,18.5	0
0.44,18.6	0
0.44,18.7	0
0.44,18.8	0
0.44,18.9	0
0.44,19.0	0
0.44,19.1	0
0.44,19.2	0
0.44,19.3	0
0.44,19.4	0
0.44,19.5	0
0.44,19.6	0
0.44,19.7	0
0.44,19.8	0
0.44,19.9	0
0.44,20.0	0
0.44,20.1	0
0.44,20.2	0
0.44,20.3	0
0.44,20.4	0
0.44,20.5	0
0.44,20.6	0
0.44,20.7	0
0.44,20.8	0
0.44,20.9	0
0.44,21.0	0
0.44,21.1	0
0.44,21.2	0
0.44,21.3	0
0.44,21.4	0
0.44,21.5	0

0.44,21.6	0
0.44,21.7	0
0.44,21.8	0
0.44,21.9	0
0.44,22.0	0
0.44,22.1	0
0.44,22.2	0
0.44,22.3	0
0.44,22.4	0
0.44,22.5	0
0.44,22.6	0
0.44,22.7	0
0.44,22.8	0
0.44,22.9	0
0.44,23.0	0
0.44,23.1	0
0.44,23.2	0
0.44,23.3	0
0.44,23.4	0
0.44,23.5	0
0.44,23.6	0
0.44,23.7	0
0.44,23.8	0
0.44,23.9	0
0.44,24.0	0
0.44,24.1	0
0.44,24.2	0
0.44,24.3	0
0.44,24.4	0
0.44,24.5	0
0.44,24.6	0
0.44,24.7	0
0.44,24.8	0
0.44,24.9	0
0.44,25.0	0
0.44,25.1	0
0.44,25.2	0
0.44,25.3	0
0.44,25.4	0
0.44,25.5	0
0.44,25.6	0
0.44,25.7	0

0.44,25.8	0
0.44,25.9	0
0.44,26.0	0
0.44,26.1	0
0.44,26.2	0
0.44,26.3	0
0.44,26.4	0
0.44,26.5	0
0.44,26.6	0
0.44,26.7	0
0.44,26.8	0
0.44,26.9	0
0.44,27.0	0
0.44,27.1	0
0.44,27.2	0
0.44,27.3	0
0.44,27.4	0
0.44,27.5	0
0.44,27.6	0
0.44,27.7	0
0.44,27.8	0
0.44,27.9	0
0.44,28.0	0
0.44,28.1	0
0.44,28.2	0
0.44,28.3	0
0.44,28.4	0
0.44,28.5	0
0.44,28.6	0
0.44,28.7	0
0.44,28.8	0
0.44,28.9	0
0.44,29.0	0
0.44,29.1	0
0.44,29.2	0
0.44,29.3	0
0.44,29.4	0
0.44,29.5	0
0.44,29.6	0
0.44,29.7	0
0.44,29.8	0
0.44,29.9	0

0.44,30.0	0
0.44,30.1	0
0.44,30.2	0
0.44,30.3	0
0.44,30.4	0
0.44,30.5	0
0.44,30.6	0
0.44,30.7	0
0.44,30.8	0
0.44,30.9	0
0.44,31.0	0
0.44,31.1	0
0.44,31.2	0
0.44,31.3	0
0.44,31.4	0
0.44,31.5	0
0.44,31.6	0
0.44,31.7	0
0.44,31.8	0
0.44,31.9	0
0.44,32.0	0
0.44,32.1	0
0.44,32.2	0
0.44,32.3	0
0.44,32.4	0
0.44,32.5	0
0.44,32.6	0
0.44,32.7	0
0.44,32.8	0
0.44,32.9	0
0.44,33.0	0
0.44,33.1	0
0.44,33.2	0
0.44,33.3	0
0.44,33.4	0
0.44,33.5	0
0.44,33.6	0
0.44,33.7	0
0.44,33.8	0
0.44,33.9	0
0.44,34.0	0
0.44,34.1	0

0.44,34.2	0
0.44,34.3	0
0.44,34.4	0
0.44,34.5	0
0.44,34.6	0
0.44,34.7	0
0.44,34.8	0
0.44,34.9	0
0.44,35.0	0
0.44,35.1	0
0.44,35.2	0
0.44,35.3	0
0.44,35.4	0
0.44,35.5	0
0.44,35.6	0
0.44,35.7	0
0.44,35.8	0
0.44,35.9	0
0.44,36.0	0
0.44,36.1	0
0.44,36.2	0
0.44,36.3	0
0.44,36.4	0
0.44,36.5	0
0.44,36.6	0
0.44,36.7	0
0.44,36.8	0
0.44,36.9	0
0.44,37.0	0
0.44,37.1	0
0.44,37.2	0
0.44,37.3	0
0.44,37.4	0
0.44,37.5	0
0.44,37.6	0
0.44,37.7	0
0.44,37.8	0
0.44,37.9	0
0.44,38.0	0
0.44,38.1	0
0.44,38.2	0
0.44,38.3	0

0.44,38.4	0
0.44,38.5	0
0.44,38.6	0
0.44,38.7	0
0.44,38.8	0
0.44,38.9	0
0.44,39.0	0
0.44,39.1	0
0.44,39.2	0
0.44,39.3	0
0.44,39.4	0
0.44,39.5	0
0.44,39.6	0
0.44,39.7	0
0.44,39.8	0
0.44,39.9	0
0.44,40.0	0
0.44,40.1	0
0.44,40.2	0
0.44,40.3	0
0.44,40.4	0
0.44,40.5	0
0.44,40.6	0
0.44,40.7	0
0.44,40.8	0
0.44,40.9	0
0.44,41.0	0
0.44,41.1	0
0.44,41.2	0
0.44,41.3	0
0.44,41.4	0
0.44,41.5	0
0.44,41.6	0
0.44,41.7	0
0.44,41.8	0
0.44,41.9	0
0.44,42.0	0
0.44,42.1	0
0.44,42.2	0
0.44,42.3	0
0.44,42.4	0
0.44,42.5	0

0.44,42.6	0
0.44,42.7	0
0.44,42.8	0
0.44,42.9	0
0.44,43.0	0
0.44,43.1	0
0.44,43.2	0
0.44,43.3	0
0.44,43.4	0
0.44,43.5	0
0.44,43.6	0
0.44,43.7	0
0.44,43.8	0
0.44,43.9	0
0.44,44.0	0
0.44,44.1	0
0.44,44.2	0
0.44,44.3	0
0.44,44.4	0
0.44,44.5	0
0.44,44.6	0
0.44,44.7	0
0.44,44.8	0
0.44,44.9	0
0.44,45.0	0
0.44,45.1	0
0.44,45.2	0
0.44,45.3	0
0.44,45.4	0
0.44,45.5	0
0.44,45.6	0
0.44,45.7	0
0.44,45.8	0
0.44,45.9	0
0.44,46.0	0
0.44,46.1	0
0.44,46.2	0
0.44,46.3	0
0.44,46.4	0
0.44,46.5	0
0.44,46.6	0
0.44,46.7	0

0.44,46.8	0
0.44,46.9	0
0.44,47.0	0
0.44,47.1	0
0.44,47.2	0
0.44,47.3	0
0.44,47.4	0
0.44,47.5	0
0.44,47.6	0
0.44,47.7	0
0.44,47.8	0
0.44,47.9	0
0.44,48.0	0
0.44,48.1	0
0.44,48.2	0
0.44,48.3	0
0.44,48.4	0
0.44,48.5	0
0.44,48.6	0
0.44,48.7	0
0.44,48.8	0
0.44,48.9	0
0.44,49.0	0
0.44,49.1	0
0.44,49.2	0
0.44,49.3	0
0.44,49.4	0
0.44,49.5	0
0.44,49.6	0
0.44,49.7	0
0.44,49.8	0
0.44,49.9	0
0.44,50.0	0
0.44,50.1	0
0.44,50.2	0
0.44,50.3	0
0.44,50.4	0
0.44,50.5	0
0.44,50.6	0
0.44,50.7	0
0.44,50.8	0
0.44,50.9	0

0.44,51.0	0
0.44,51.1	0
0.44,51.2	0
0.44,51.3	0
0.44,51.4	0
0.44,51.5	0
0.44,51.6	0
0.44,51.7	0
0.44,51.8	0
0.44,51.9	0
0.44,52.0	0
0.44,52.1	0
0.44,52.2	0
0.44,52.3	0
0.44,52.4	0
0.44,52.5	0
0.44,52.6	0
0.44,52.7	0
0.44,52.8	0
0.44,52.9	0
0.44,53.0	0
0.44,53.1	0
0.44,53.2	0
0.44,53.3	0
0.44,53.4	0
0.44,53.5	0
0.44,53.6	0
0.44,53.7	0
0.44,53.8	0
0.44,53.9	0
0.44,54.0	0
0.44,54.1	0
0.44,54.2	0
0.44,54.3	0
0.44,54.4	0
0.44,54.5	0
0.44,54.6	0
0.44,54.7	0
0.44,54.8	0
0.44,54.9	0
0.44,55.0	0
0.44,55.1	0

0.44,55.2	0
0.44,55.3	0
0.44,55.4	0
0.44,55.5	0
0.44,55.6	0
0.44,55.7	0
0.44,55.8	0
0.44,55.9	0
0.44,56.0	0
0.44,56.1	0
0.44,56.2	0
0.44,56.3	0
0.44,56.4	0
0.44,56.5	0
0.44,56.6	0
0.44,56.7	0
0.44,56.8	0
0.44,56.9	0
0.44,57.0	0
0.44,57.1	0
0.44,57.2	0
0.44,57.3	0
0.44,57.4	0
0.44,57.5	0
0.44,57.6	0
0.44,57.7	0
0.44,57.8	0
0.44,57.9	0
0.44,58.0	0
0.44,58.1	0
0.44,58.2	0
0.44,58.3	0
0.44,58.4	0
0.44,58.5	0
0.44,58.6	0
0.44,58.7	0
0.44,58.8	0
0.44,58.9	0
0.44,59.0	0
0.44,59.1	0
0.44,59.2	0
0.44,59.3	0

0.44,59.4	0
0.44,59.5	0
0.44,59.6	0
0.44,59.7	0
0.44,59.8	0
0.44,59.9	0
0.44,60.0	0
0.44,60.1	0
0.44,60.2	0
0.44,60.3	0
0.44,60.4	0
0.44,60.5	0
0.44,60.6	0
0.44,60.7	0
0.44,60.8	0
0.44,60.9	0
0.44,61.0	0
0.44,61.1	0
0.44,61.2	0
0.44,61.3	0
0.44,61.4	0
0.44,61.5	0
0.44,61.6	0
0.44,61.7	0
0.44,61.8	0
0.44,61.9	0
0.44,62.0	0
0.44,62.1	0
0.44,62.2	0
0.44,62.3	0
0.44,62.4	0
0.44,62.5	0
0.44,62.6	0
0.44,62.7	0
0.44,62.8	0
0.44,62.9	0
0.44,63.0	0
0.44,63.1	0
0.44,63.2	0
0.44,63.3	0
0.44,63.4	0
0.44,63.5	0

0.44,63.6	0
0.44,63.7	0
0.44,63.8	0
0.44,63.9	0
0.44,64.0	0
0.44,64.1	0
0.44,64.2	0
0.44,64.3	0
0.44,64.4	0
0.44,64.5	0
0.44,64.6	0
0.44,64.7	0
0.44,64.8	0
0.44,64.9	0
0.44,65.0	0
0.44,65.1	0
0.44,65.2	0
0.44,65.3	0
0.44,65.4	0
0.44,65.5	0
0.44,65.6	0
0.44,65.7	0
0.44,65.8	0
0.44,65.9	0
0.44,66.0	0
0.44,66.1	0
0.44,66.2	0
0.44,66.3	0
0.44,66.4	0
0.44,66.5	0
0.44,66.6	0
0.44,66.7	0
0.44,66.8	0
0.44,66.9	0
0.44,67.0	0
0.44,67.1	0
0.44,67.2	0
0.44,67.3	0
0.44,67.4	0
0.44,67.5	0
0.44,67.6	0
0.44,67.7	0

0.44,67.8	0
0.44,67.9	0
0.44,68.0	0
0.44,68.1	0
0.44,68.2	0
0.44,68.3	0
0.44,68.4	0
0.44,68.5	0
0.44,68.6	0
0.44,68.7	0
0.44,68.8	0
0.44,68.9	0
0.44,69.0	0
0.44,69.1	0
0.44,69.2	0
0.44,69.3	0
0.44,69.4	0
0.44,69.5	0
0.44,69.6	0
0.44,69.7	0
0.44,69.8	0
0.44,69.9	0
0.44,70.0	0
0.44,70.1	0
0.44,70.2	0
0.44,70.3	0
0.44,70.4	0
0.44,70.5	0
0.44,70.6	0
0.44,70.7	0
0.44,70.8	0
0.44,70.9	0
0.44,71.0	0
0.44,71.1	0
0.44,71.2	0
0.44,71.3	0
0.44,71.4	0
0.44,71.5	0
0.44,71.6	0
0.44,71.7	0
0.44,71.8	0
0.44,71.9	0

0.44,72.0	0
0.44,72.1	0
0.44,72.2	0
0.44,72.3	0
0.44,72.4	0
0.44,72.5	0
0.44,72.6	0
0.44,72.7	0
0.44,72.8	0
0.44,72.9	0
0.44,73.0	0
0.44,73.1	0
0.44,73.2	0
0.44,73.3	0
0.44,73.4	0
0.44,73.5	0
0.44,73.6	0
0.44,73.7	0
0.44,73.8	0
0.44,73.9	0
0.44,74.0	0
0.44,74.1	0
0.44,74.2	0
0.44,74.3	0
0.44,74.4	0
0.44,74.5	0
0.44,74.6	0
0.44,74.7	0
0.44,74.8	0
0.44,74.9	0
0.44,75.0	0
0.44,75.1	0
0.44,75.2	0
0.44,75.3	0
0.44,75.4	0
0.44,75.5	0
0.44,75.6	0
0.44,75.7	0
0.44,75.8	0
0.44,75.9	0
0.44,76.0	0
0.44,76.1	0

0.44,76.2	0
0.44,76.3	0
0.44,76.4	0
0.44,76.5	0
0.44,76.6	0
0.44,76.7	0
0.44,76.8	0
0.44,76.9	0
0.44,77.0	0
0.44,77.1	0
0.44,77.2	0
0.44,77.3	0
0.44,77.4	0
0.44,77.5	0
0.44,77.6	0
0.44,77.7	0
0.44,77.8	0
0.44,77.9	0
0.44,78.0	0
0.44,78.1	0
0.44,78.2	0
0.44,78.3	0
0.44,78.4	0
0.44,78.5	0
0.44,78.6	0
0.44,78.7	0
0.44,78.8	0
0.44,78.9	0
0.44,79.0	0
0.44,79.1	0
0.44,79.2	0
0.44,79.3	0
0.44,79.4	0
0.44,79.5	0
0.44,79.6	0
0.44,79.7	0
0.44,79.8	0
0.44,79.9	0
0.44,80.0	0
0.44,80.1	0
0.44,80.2	0
0.44,80.3	0

0.44,80.4	0
0.44,80.5	0
0.44,80.6	0
0.44,80.7	0
0.44,80.8	0
0.44,80.9	0
0.44,81.0	0
0.44,81.1	0
0.44,81.2	0
0.44,81.3	0
0.44,81.4	0
0.44,81.5	0
0.44,81.6	0
0.44,81.7	0
0.44,81.8	0
0.44,81.9	0
0.44,82.0	0
0.44,82.1	0
0.44,82.2	0
0.44,82.3	0
0.44,82.4	0
0.44,82.5	0
0.44,82.6	0
0.44,82.7	0
0.44,82.8	0
0.44,82.9	0
0.44,83.0	0
0.44,83.1	0
0.44,83.2	0
0.44,83.3	0
0.44,83.4	0
0.44,83.5	0
0.44,83.6	0
0.44,83.7	0
0.44,83.8	0
0.44,83.9	0
0.44,84.0	0
0.44,84.1	0
0.44,84.2	0
0.44,84.3	0
0.44,84.4	0
0.44,84.5	0

0.44,84.6	0
0.44,84.7	0
0.44,84.8	0
0.44,84.9	0
0.44,85.0	0
0.44,85.1	0
0.44,85.2	0
0.44,85.3	0
0.44,85.4	0
0.44,85.5	0
0.44,85.6	0
0.44,85.7	0
0.44,85.8	0
0.44,85.9	0
0.44,86.0	0
0.44,86.1	0
0.44,86.2	0
0.44,86.3	0
0.44,86.4	0
0.44,86.5	0
0.44,86.6	0
0.44,86.7	0
0.44,86.8	0
0.44,86.9	0
0.44,87.0	0
0.44,87.1	0
0.44,87.2	0
0.44,87.3	0
0.44,87.4	0
0.44,87.5	0
0.44,87.6	0
0.44,87.7	0
0.44,87.8	0
0.44,87.9	0
0.44,88.0	0
0.44,88.1	0
0.44,88.2	0
0.44,88.3	0
0.44,88.4	0
0.44,88.5	0
0.44,88.6	0
0.44,88.7	0

0.44,88.8	0
0.44,88.9	0
0.44,89.0	0
0.44,89.1	0
0.44,89.2	0
0.44,89.3	0
0.44,89.4	0
0.44,89.5	0
0.44,89.6	0
0.44,89.7	0
0.44,89.8	0
0.44,89.9	0
0.44,90.0	0
0.44,90.1	0
0.44,90.2	0
0.44,90.3	0
0.44,90.4	0
0.44,90.5	0
0.44,90.6	0
0.44,90.7	0
0.44,90.8	0
0.44,90.9	0
0.44,91.0	0
0.44,91.1	0
0.44,91.2	0
0.44,91.3	0
0.44,91.4	0
0.44,91.5	0
0.44,91.6	0
0.44,91.7	0
0.44,91.8	0
0.44,91.9	0
0.44,92.0	0
0.44,92.1	0
0.44,92.2	0
0.44,92.3	0
0.44,92.4	0
0.44,92.5	0
0.44,92.6	0
0.44,92.7	0
0.44,92.8	0
0.44,92.9	0

0.44,93.0	0
0.44,93.1	0
0.44,93.2	0
0.44,93.3	0
0.44,93.4	0
0.44,93.5	0
0.44,93.6	0
0.44,93.7	0
0.44,93.8	0
0.44,93.9	0
0.44,94.0	0
0.44,94.1	0
0.44,94.2	0
0.44,94.3	0
0.44,94.4	0
0.44,94.5	0
0.44,94.6	0
0.44,94.7	0
0.44,94.8	0
0.44,94.9	0
0.44,95.0	0
0.44,95.1	0
0.44,95.2	0
0.44,95.3	0
0.44,95.4	0
0.44,95.5	0
0.44,95.6	0
0.44,95.7	0
0.44,95.8	0
0.44,95.9	0
0.44,96.0	0
0.44,96.1	0
0.44,96.2	0
0.44,96.3	0
0.44,96.4	0
0.44,96.5	0
0.44,96.6	0
0.44,96.7	0
0.44,96.8	0
0.44,96.9	0
0.44,97.0	0
0.44,97.1	0

0.44,97.2	0
0.44,97.3	0
0.44,97.4	0
0.44,97.5	0
0.44,97.6	0
0.44,97.7	0
0.44,97.8	0
0.44,97.9	0
0.44,98.0	0
0.44,98.1	0
0.44,98.2	0
0.44,98.3	0
0.44,98.4	0
0.44,98.5	0
0.44,98.6	0
0.44,98.7	0
0.44,98.8	0
0.44,98.9	0
0.44,99.0	0
0.44,99.1	0
0.44,99.2	0
0.44,99.3	0
0.44,99.4	0
0.44,99.5	0
0.44,99.6	0
0.44,99.7	0
0.44,99.8	0
0.44,99.9	0
0.44,100.0	0
0.45,0.0	0
0.45,0.1	0
0.45,0.2	0
0.45,0.3	0
0.45,0.4	0
0.45,0.5	0
0.45,0.6	0
0.45,0.7	0
0.45,0.8	0
0.45,0.9	0
0.45,1.0	0
0.45,1.1	0
0.45,1.2	0

0.45,1.3	0
0.45,1.4	0
0.45,1.5	0
0.45,1.6	0
0.45,1.7	0
0.45,1.8	0
0.45,1.9	0
0.45,2.0	0
0.45,2.1	0
0.45,2.2	0
0.45,2.3	0
0.45,2.4	0
0.45,2.5	0
0.45,2.6	0
0.45,2.7	0
0.45,2.8	0
0.45,2.9	0
0.45,3.0	0
0.45,3.1	0
0.45,3.2	0
0.45,3.3	0
0.45,3.4	0
0.45,3.5	0
0.45,3.6	0
0.45,3.7	0
0.45,3.8	0
0.45,3.9	0
0.45,4.0	0
0.45,4.1	0
0.45,4.2	0
0.45,4.3	0
0.45,4.4	0
0.45,4.5	0
0.45,4.6	0
0.45,4.7	0
0.45,4.8	0
0.45,4.9	0
0.45,5.0	0
0.45,5.1	0
0.45,5.2	0
0.45,5.3	0
0.45,5.4	0

0.45,5.5	0
0.45,5.6	0
0.45,5.7	0
0.45,5.8	0
0.45,5.9	0
0.45,6.0	0
0.45,6.1	0
0.45,6.2	0
0.45,6.3	0
0.45,6.4	0
0.45,6.5	0
0.45,6.6	0
0.45,6.7	0
0.45,6.8	0
0.45,6.9	0
0.45,7.0	0
0.45,7.1	0
0.45,7.2	0
0.45,7.3	0
0.45,7.4	0
0.45,7.5	0
0.45,7.6	0
0.45,7.7	0
0.45,7.8	0
0.45,7.9	0
0.45,8.0	0
0.45,8.1	0
0.45,8.2	0
0.45,8.3	0
0.45,8.4	0
0.45,8.5	0
0.45,8.6	0
0.45,8.7	0
0.45,8.8	0
0.45,8.9	0
0.45,9.0	0
0.45,9.1	0
0.45,9.2	0
0.45,9.3	0
0.45,9.4	0
0.45,9.5	0
0.45,9.6	0

0.45,9.7	0
0.45,9.8	0
0.45,9.9	0
0.45,10.0	0
0.45,10.1	0
0.45,10.2	0
0.45,10.3	0
0.45,10.4	0
0.45,10.5	0
0.45,10.6	0
0.45,10.7	0
0.45,10.8	0
0.45,10.9	0
0.45,11.0	0
0.45,11.1	0
0.45,11.2	0
0.45,11.3	0
0.45,11.4	0
0.45,11.5	0
0.45,11.6	0
0.45,11.7	0
0.45,11.8	0
0.45,11.9	0
0.45,12.0	0
0.45,12.1	0
0.45,12.2	0
0.45,12.3	0
0.45,12.4	0
0.45,12.5	0
0.45,12.6	0
0.45,12.7	0
0.45,12.8	0
0.45,12.9	0
0.45,13.0	0
0.45,13.1	0
0.45,13.2	0
0.45,13.3	0
0.45,13.4	0
0.45,13.5	0
0.45,13.6	0
0.45,13.7	0
0.45,13.8	0

0.45,13.9	0
0.45,14.0	0
0.45,14.1	0
0.45,14.2	0
0.45,14.3	0
0.45,14.4	0
0.45,14.5	0
0.45,14.6	0
0.45,14.7	0
0.45,14.8	0
0.45,14.9	0
0.45,15.0	0
0.45,15.1	0
0.45,15.2	0
0.45,15.3	0
0.45,15.4	0
0.45,15.5	0
0.45,15.6	0
0.45,15.7	0
0.45,15.8	0
0.45,15.9	0
0.45,16.0	0
0.45,16.1	0
0.45,16.2	0
0.45,16.3	0
0.45,16.4	0
0.45,16.5	0
0.45,16.6	0
0.45,16.7	0
0.45,16.8	0
0.45,16.9	0
0.45,17.0	0
0.45,17.1	0
0.45,17.2	0
0.45,17.3	0
0.45,17.4	0
0.45,17.5	0
0.45,17.6	0
0.45,17.7	0
0.45,17.8	0
0.45,17.9	0
0.45,18.0	0

0.45,18.1	0
0.45,18.2	0
0.45,18.3	0
0.45,18.4	0
0.45,18.5	0
0.45,18.6	0
0.45,18.7	0
0.45,18.8	0
0.45,18.9	0
0.45,19.0	0
0.45,19.1	0
0.45,19.2	0
0.45,19.3	0
0.45,19.4	0
0.45,19.5	0
0.45,19.6	0
0.45,19.7	0
0.45,19.8	0
0.45,19.9	0
0.45,20.0	0
0.45,20.1	0
0.45,20.2	0
0.45,20.3	0
0.45,20.4	0
0.45,20.5	0
0.45,20.6	0
0.45,20.7	0
0.45,20.8	0
0.45,20.9	0
0.45,21.0	0
0.45,21.1	0
0.45,21.2	0
0.45,21.3	0
0.45,21.4	0
0.45,21.5	0
0.45,21.6	0
0.45,21.7	0
0.45,21.8	0
0.45,21.9	0
0.45,22.0	0
0.45,22.1	0
0.45,22.2	0

0.45,22.3	0
0.45,22.4	0
0.45,22.5	0
0.45,22.6	0
0.45,22.7	0
0.45,22.8	0
0.45,22.9	0
0.45,23.0	0
0.45,23.1	0
0.45,23.2	0
0.45,23.3	0
0.45,23.4	0
0.45,23.5	0
0.45,23.6	0
0.45,23.7	0
0.45,23.8	0
0.45,23.9	0
0.45,24.0	0
0.45,24.1	0
0.45,24.2	0
0.45,24.3	0
0.45,24.4	0
0.45,24.5	0
0.45,24.6	0
0.45,24.7	0
0.45,24.8	0
0.45,24.9	0
0.45,25.0	0
0.45,25.1	0
0.45,25.2	0
0.45,25.3	0
0.45,25.4	0
0.45,25.5	0
0.45,25.6	0
0.45,25.7	0
0.45,25.8	0
0.45,25.9	0
0.45,26.0	0
0.45,26.1	0
0.45,26.2	0
0.45,26.3	0
0.45,26.4	0

0.45,26.5	0
0.45,26.6	0
0.45,26.7	0
0.45,26.8	0
0.45,26.9	0
0.45,27.0	0
0.45,27.1	0
0.45,27.2	0
0.45,27.3	0
0.45,27.4	0
0.45,27.5	0
0.45,27.6	0
0.45,27.7	0
0.45,27.8	0
0.45,27.9	0
0.45,28.0	0
0.45,28.1	0
0.45,28.2	0
0.45,28.3	0
0.45,28.4	0
0.45,28.5	0
0.45,28.6	0
0.45,28.7	0
0.45,28.8	0
0.45,28.9	0
0.45,29.0	0
0.45,29.1	0
0.45,29.2	0
0.45,29.3	0
0.45,29.4	0
0.45,29.5	0
0.45,29.6	0
0.45,29.7	0
0.45,29.8	0
0.45,29.9	0
0.45,30.0	0
0.45,30.1	0
0.45,30.2	0
0.45,30.3	0
0.45,30.4	0
0.45,30.5	0
0.45,30.6	0

0.45,30.7	0
0.45,30.8	0
0.45,30.9	0
0.45,31.0	0
0.45,31.1	0
0.45,31.2	0
0.45,31.3	0
0.45,31.4	0
0.45,31.5	0
0.45,31.6	0
0.45,31.7	0
0.45,31.8	0
0.45,31.9	0
0.45,32.0	0
0.45,32.1	0
0.45,32.2	0
0.45,32.3	0
0.45,32.4	0
0.45,32.5	0
0.45,32.6	0
0.45,32.7	0
0.45,32.8	0
0.45,32.9	0
0.45,33.0	0
0.45,33.1	0
0.45,33.2	0
0.45,33.3	0
0.45,33.4	0
0.45,33.5	0
0.45,33.6	0
0.45,33.7	0
0.45,33.8	0
0.45,33.9	0
0.45,34.0	0
0.45,34.1	0
0.45,34.2	0
0.45,34.3	0
0.45,34.4	0
0.45,34.5	0
0.45,34.6	0
0.45,34.7	0
0.45,34.8	0

0.45,34.9	0
0.45,35.0	0
0.45,35.1	0
0.45,35.2	0
0.45,35.3	0
0.45,35.4	0
0.45,35.5	0
0.45,35.6	0
0.45,35.7	0
0.45,35.8	0
0.45,35.9	0
0.45,36.0	0
0.45,36.1	0
0.45,36.2	0
0.45,36.3	0
0.45,36.4	0
0.45,36.5	0
0.45,36.6	0
0.45,36.7	0
0.45,36.8	0
0.45,36.9	0
0.45,37.0	0
0.45,37.1	0
0.45,37.2	0
0.45,37.3	0
0.45,37.4	0
0.45,37.5	0
0.45,37.6	0
0.45,37.7	0
0.45,37.8	0
0.45,37.9	0
0.45,38.0	0
0.45,38.1	0
0.45,38.2	0
0.45,38.3	0
0.45,38.4	0
0.45,38.5	0
0.45,38.6	0
0.45,38.7	0
0.45,38.8	0
0.45,38.9	0
0.45,39.0	0

0.45,39.1	0
0.45,39.2	0
0.45,39.3	0
0.45,39.4	0
0.45,39.5	0
0.45,39.6	0
0.45,39.7	0
0.45,39.8	0
0.45,39.9	0
0.45,40.0	0
0.45,40.1	0
0.45,40.2	0
0.45,40.3	0
0.45,40.4	0
0.45,40.5	0
0.45,40.6	0
0.45,40.7	0
0.45,40.8	0
0.45,40.9	0
0.45,41.0	0
0.45,41.1	0
0.45,41.2	0
0.45,41.3	0
0.45,41.4	0
0.45,41.5	0
0.45,41.6	0
0.45,41.7	0
0.45,41.8	0
0.45,41.9	0
0.45,42.0	0
0.45,42.1	0
0.45,42.2	0
0.45,42.3	0
0.45,42.4	0
0.45,42.5	0
0.45,42.6	0
0.45,42.7	0
0.45,42.8	0
0.45,42.9	0
0.45,43.0	0
0.45,43.1	0
0.45,43.2	0

0.45,43.3	0
0.45,43.4	0
0.45,43.5	0
0.45,43.6	0
0.45,43.7	0
0.45,43.8	0
0.45,43.9	0
0.45,44.0	0
0.45,44.1	0
0.45,44.2	0
0.45,44.3	0
0.45,44.4	0
0.45,44.5	0
0.45,44.6	0
0.45,44.7	0
0.45,44.8	0
0.45,44.9	0
0.45,45.0	0
0.45,45.1	0
0.45,45.2	0
0.45,45.3	0
0.45,45.4	0
0.45,45.5	0
0.45,45.6	0
0.45,45.7	0
0.45,45.8	0
0.45,45.9	0
0.45,46.0	0
0.45,46.1	0
0.45,46.2	0
0.45,46.3	0
0.45,46.4	0
0.45,46.5	0
0.45,46.6	0
0.45,46.7	0
0.45,46.8	0
0.45,46.9	0
0.45,47.0	0
0.45,47.1	0
0.45,47.2	0
0.45,47.3	0
0.45,47.4	0

0.45,47.5	0
0.45,47.6	0
0.45,47.7	0
0.45,47.8	0
0.45,47.9	0
0.45,48.0	0
0.45,48.1	0
0.45,48.2	0
0.45,48.3	0
0.45,48.4	0
0.45,48.5	0
0.45,48.6	0
0.45,48.7	0
0.45,48.8	0
0.45,48.9	0
0.45,49.0	0
0.45,49.1	0
0.45,49.2	0
0.45,49.3	0
0.45,49.4	0
0.45,49.5	0
0.45,49.6	0
0.45,49.7	0
0.45,49.8	0
0.45,49.9	0
0.45,50.0	0
0.45,50.1	0
0.45,50.2	0
0.45,50.3	0
0.45,50.4	0
0.45,50.5	0
0.45,50.6	0
0.45,50.7	0
0.45,50.8	0
0.45,50.9	0
0.45,51.0	0
0.45,51.1	0
0.45,51.2	0
0.45,51.3	0
0.45,51.4	0
0.45,51.5	0
0.45,51.6	0

0.45,51.7	0
0.45,51.8	0
0.45,51.9	0
0.45,52.0	0
0.45,52.1	0
0.45,52.2	0
0.45,52.3	0
0.45,52.4	0
0.45,52.5	0
0.45,52.6	0
0.45,52.7	0
0.45,52.8	0
0.45,52.9	0
0.45,53.0	0
0.45,53.1	0
0.45,53.2	0
0.45,53.3	0
0.45,53.4	0
0.45,53.5	0
0.45,53.6	0
0.45,53.7	0
0.45,53.8	0
0.45,53.9	0
0.45,54.0	0
0.45,54.1	0
0.45,54.2	0
0.45,54.3	0
0.45,54.4	0
0.45,54.5	0
0.45,54.6	0
0.45,54.7	0
0.45,54.8	0
0.45,54.9	0
0.45,55.0	0
0.45,55.1	0
0.45,55.2	0
0.45,55.3	0
0.45,55.4	0
0.45,55.5	0
0.45,55.6	0
0.45,55.7	0
0.45,55.8	0

0.45,55.9	0
0.45,56.0	0
0.45,56.1	0
0.45,56.2	0
0.45,56.3	0
0.45,56.4	0
0.45,56.5	0
0.45,56.6	0
0.45,56.7	0
0.45,56.8	0
0.45,56.9	0
0.45,57.0	0
0.45,57.1	0
0.45,57.2	0
0.45,57.3	0
0.45,57.4	0
0.45,57.5	0
0.45,57.6	0
0.45,57.7	0
0.45,57.8	0
0.45,57.9	0
0.45,58.0	0
0.45,58.1	0
0.45,58.2	0
0.45,58.3	0
0.45,58.4	0
0.45,58.5	0
0.45,58.6	0
0.45,58.7	0
0.45,58.8	0
0.45,58.9	0
0.45,59.0	0
0.45,59.1	0
0.45,59.2	0
0.45,59.3	0
0.45,59.4	0
0.45,59.5	0
0.45,59.6	0
0.45,59.7	0
0.45,59.8	0
0.45,59.9	0
0.45,60.0	0

0.45,60.1	0
0.45,60.2	0
0.45,60.3	0
0.45,60.4	0
0.45,60.5	0
0.45,60.6	0
0.45,60.7	0
0.45,60.8	0
0.45,60.9	0
0.45,61.0	0
0.45,61.1	0
0.45,61.2	0
0.45,61.3	0
0.45,61.4	0
0.45,61.5	0
0.45,61.6	0
0.45,61.7	0
0.45,61.8	0
0.45,61.9	0
0.45,62.0	0
0.45,62.1	0
0.45,62.2	0
0.45,62.3	0
0.45,62.4	0
0.45,62.5	0
0.45,62.6	0
0.45,62.7	0
0.45,62.8	0
0.45,62.9	0
0.45,63.0	0
0.45,63.1	0
0.45,63.2	0
0.45,63.3	0
0.45,63.4	0
0.45,63.5	0
0.45,63.6	0
0.45,63.7	0
0.45,63.8	0
0.45,63.9	0
0.45,64.0	0
0.45,64.1	0
0.45,64.2	0

0.45,64.3	0
0.45,64.4	0
0.45,64.5	0
0.45,64.6	0
0.45,64.7	0
0.45,64.8	0
0.45,64.9	0
0.45,65.0	0
0.45,65.1	0
0.45,65.2	0
0.45,65.3	0
0.45,65.4	0
0.45,65.5	0
0.45,65.6	0
0.45,65.7	0
0.45,65.8	0
0.45,65.9	0
0.45,66.0	0
0.45,66.1	0
0.45,66.2	0
0.45,66.3	0
0.45,66.4	0
0.45,66.5	0
0.45,66.6	0
0.45,66.7	0
0.45,66.8	0
0.45,66.9	0
0.45,67.0	0
0.45,67.1	0
0.45,67.2	0
0.45,67.3	0
0.45,67.4	0
0.45,67.5	0
0.45,67.6	0
0.45,67.7	0
0.45,67.8	0
0.45,67.9	0
0.45,68.0	0
0.45,68.1	0
0.45,68.2	0
0.45,68.3	0
0.45,68.4	0

0.45,68.5	0
0.45,68.6	0
0.45,68.7	0
0.45,68.8	0
0.45,68.9	0
0.45,69.0	0
0.45,69.1	0
0.45,69.2	0
0.45,69.3	0
0.45,69.4	0
0.45,69.5	0
0.45,69.6	0
0.45,69.7	0
0.45,69.8	0
0.45,69.9	0
0.45,70.0	0
0.45,70.1	0
0.45,70.2	0
0.45,70.3	0
0.45,70.4	0
0.45,70.5	0
0.45,70.6	0
0.45,70.7	0
0.45,70.8	0
0.45,70.9	0
0.45,71.0	0
0.45,71.1	0
0.45,71.2	0
0.45,71.3	0
0.45,71.4	0
0.45,71.5	0
0.45,71.6	0
0.45,71.7	0
0.45,71.8	0
0.45,71.9	0
0.45,72.0	0
0.45,72.1	0
0.45,72.2	0
0.45,72.3	0
0.45,72.4	0
0.45,72.5	0
0.45,72.6	0

0.45,72.7	0
0.45,72.8	0
0.45,72.9	0
0.45,73.0	0
0.45,73.1	0
0.45,73.2	0
0.45,73.3	0
0.45,73.4	0
0.45,73.5	0
0.45,73.6	0
0.45,73.7	0
0.45,73.8	0
0.45,73.9	0
0.45,74.0	0
0.45,74.1	0
0.45,74.2	0
0.45,74.3	0
0.45,74.4	0
0.45,74.5	0
0.45,74.6	0
0.45,74.7	0
0.45,74.8	0
0.45,74.9	0
0.45,75.0	0
0.45,75.1	0
0.45,75.2	0
0.45,75.3	0
0.45,75.4	0
0.45,75.5	0
0.45,75.6	0
0.45,75.7	0
0.45,75.8	0
0.45,75.9	0
0.45,76.0	0
0.45,76.1	0
0.45,76.2	0
0.45,76.3	0
0.45,76.4	0
0.45,76.5	0
0.45,76.6	0
0.45,76.7	0
0.45,76.8	0

0.45,76.9	0
0.45,77.0	0
0.45,77.1	0
0.45,77.2	0
0.45,77.3	0
0.45,77.4	0
0.45,77.5	0
0.45,77.6	0
0.45,77.7	0
0.45,77.8	0
0.45,77.9	0
0.45,78.0	0
0.45,78.1	0
0.45,78.2	0
0.45,78.3	0
0.45,78.4	0
0.45,78.5	0
0.45,78.6	0
0.45,78.7	0
0.45,78.8	0
0.45,78.9	0
0.45,79.0	0
0.45,79.1	0
0.45,79.2	0
0.45,79.3	0
0.45,79.4	0
0.45,79.5	0
0.45,79.6	0
0.45,79.7	0
0.45,79.8	0
0.45,79.9	0
0.45,80.0	0
0.45,80.1	0
0.45,80.2	0
0.45,80.3	0
0.45,80.4	0
0.45,80.5	0
0.45,80.6	0
0.45,80.7	0
0.45,80.8	0
0.45,80.9	0
0.45,81.0	0

0.45,81.1	0
0.45,81.2	0
0.45,81.3	0
0.45,81.4	0
0.45,81.5	0
0.45,81.6	0
0.45,81.7	0
0.45,81.8	0
0.45,81.9	0
0.45,82.0	0
0.45,82.1	0
0.45,82.2	0
0.45,82.3	0
0.45,82.4	0
0.45,82.5	0
0.45,82.6	0
0.45,82.7	0
0.45,82.8	0
0.45,82.9	0
0.45,83.0	0
0.45,83.1	0
0.45,83.2	0
0.45,83.3	0
0.45,83.4	0
0.45,83.5	0
0.45,83.6	0
0.45,83.7	0
0.45,83.8	0
0.45,83.9	0
0.45,84.0	0
0.45,84.1	0
0.45,84.2	0
0.45,84.3	0
0.45,84.4	0
0.45,84.5	0
0.45,84.6	0
0.45,84.7	0
0.45,84.8	0
0.45,84.9	0
0.45,85.0	0
0.45,85.1	0
0.45,85.2	0

0.45,85.3	0
0.45,85.4	0
0.45,85.5	0
0.45,85.6	0
0.45,85.7	0
0.45,85.8	0
0.45,85.9	0
0.45,86.0	0
0.45,86.1	0
0.45,86.2	0
0.45,86.3	0
0.45,86.4	0
0.45,86.5	0
0.45,86.6	0
0.45,86.7	0
0.45,86.8	0
0.45,86.9	0
0.45,87.0	0
0.45,87.1	0
0.45,87.2	0
0.45,87.3	0
0.45,87.4	0
0.45,87.5	0
0.45,87.6	0
0.45,87.7	0
0.45,87.8	0
0.45,87.9	0
0.45,88.0	0
0.45,88.1	0
0.45,88.2	0
0.45,88.3	0
0.45,88.4	0
0.45,88.5	0
0.45,88.6	0
0.45,88.7	0
0.45,88.8	0
0.45,88.9	0
0.45,89.0	0
0.45,89.1	0
0.45,89.2	0
0.45,89.3	0
0.45,89.4	0

0.45,89.5	0
0.45,89.6	0
0.45,89.7	0
0.45,89.8	0
0.45,89.9	0
0.45,90.0	0
0.45,90.1	0
0.45,90.2	0
0.45,90.3	0
0.45,90.4	0
0.45,90.5	0
0.45,90.6	0
0.45,90.7	0
0.45,90.8	0
0.45,90.9	0
0.45,91.0	0
0.45,91.1	0
0.45,91.2	0
0.45,91.3	0
0.45,91.4	0
0.45,91.5	0
0.45,91.6	0
0.45,91.7	0
0.45,91.8	0
0.45,91.9	0
0.45,92.0	0
0.45,92.1	0
0.45,92.2	0
0.45,92.3	0
0.45,92.4	0
0.45,92.5	0
0.45,92.6	0
0.45,92.7	0
0.45,92.8	0
0.45,92.9	0
0.45,93.0	0
0.45,93.1	0
0.45,93.2	0
0.45,93.3	0
0.45,93.4	0
0.45,93.5	0
0.45,93.6	0

0.45,93.7	0
0.45,93.8	0
0.45,93.9	0
0.45,94.0	0
0.45,94.1	0
0.45,94.2	0
0.45,94.3	0
0.45,94.4	0
0.45,94.5	0
0.45,94.6	0
0.45,94.7	0
0.45,94.8	0
0.45,94.9	0
0.45,95.0	0
0.45,95.1	0
0.45,95.2	0
0.45,95.3	0
0.45,95.4	0
0.45,95.5	0
0.45,95.6	0
0.45,95.7	0
0.45,95.8	0
0.45,95.9	0
0.45,96.0	0
0.45,96.1	0
0.45,96.2	0
0.45,96.3	0
0.45,96.4	0
0.45,96.5	0
0.45,96.6	0
0.45,96.7	0
0.45,96.8	0
0.45,96.9	0
0.45,97.0	0
0.45,97.1	0
0.45,97.2	0
0.45,97.3	0
0.45,97.4	0
0.45,97.5	0
0.45,97.6	0
0.45,97.7	0
0.45,97.8	0

0.45,97.9	0
0.45,98.0	0
0.45,98.1	0
0.45,98.2	0
0.45,98.3	0
0.45,98.4	0
0.45,98.5	0
0.45,98.6	0
0.45,98.7	0
0.45,98.8	0
0.45,98.9	0
0.45,99.0	0
0.45,99.1	0
0.45,99.2	0
0.45,99.3	0
0.45,99.4	0
0.45,99.5	0
0.45,99.6	0
0.45,99.7	0
0.45,99.8	0
0.45,99.9	0
0.45,100.0	0
0.46,0.0	0
0.46,0.1	0
0.46,0.2	0
0.46,0.3	0
0.46,0.4	0
0.46,0.5	0
0.46,0.6	0
0.46,0.7	0
0.46,0.8	0
0.46,0.9	0
0.46,1.0	0
0.46,1.1	0
0.46,1.2	0
0.46,1.3	0
0.46,1.4	0
0.46,1.5	0
0.46,1.6	0
0.46,1.7	0
0.46,1.8	0
0.46,1.9	0

0.46,2.0	0
0.46,2.1	0
0.46,2.2	0
0.46,2.3	0
0.46,2.4	0
0.46,2.5	0
0.46,2.6	0
0.46,2.7	0
0.46,2.8	0
0.46,2.9	0
0.46,3.0	0
0.46,3.1	0
0.46,3.2	0
0.46,3.3	0
0.46,3.4	0
0.46,3.5	0
0.46,3.6	0
0.46,3.7	0
0.46,3.8	0
0.46,3.9	0
0.46,4.0	0
0.46,4.1	0
0.46,4.2	0
0.46,4.3	0
0.46,4.4	0
0.46,4.5	0
0.46,4.6	0
0.46,4.7	0
0.46,4.8	0
0.46,4.9	0
0.46,5.0	0
0.46,5.1	0
0.46,5.2	0
0.46,5.3	0
0.46,5.4	0
0.46,5.5	0
0.46,5.6	0
0.46,5.7	0
0.46,5.8	0
0.46,5.9	0
0.46,6.0	0
0.46,6.1	0

0.46,6.2	0
0.46,6.3	0
0.46,6.4	0
0.46,6.5	0
0.46,6.6	0
0.46,6.7	0
0.46,6.8	0
0.46,6.9	0
0.46,7.0	0
0.46,7.1	0
0.46,7.2	0
0.46,7.3	0
0.46,7.4	0
0.46,7.5	0
0.46,7.6	0
0.46,7.7	0
0.46,7.8	0
0.46,7.9	0
0.46,8.0	0
0.46,8.1	0
0.46,8.2	0
0.46,8.3	0
0.46,8.4	0
0.46,8.5	0
0.46,8.6	0
0.46,8.7	0
0.46,8.8	0
0.46,8.9	0
0.46,9.0	0
0.46,9.1	0
0.46,9.2	0
0.46,9.3	0
0.46,9.4	0
0.46,9.5	0
0.46,9.6	0
0.46,9.7	0
0.46,9.8	0
0.46,9.9	0
0.46,10.0	0
0.46,10.1	0
0.46,10.2	0
0.46,10.3	0

0.46,10.4	0
0.46,10.5	0
0.46,10.6	0
0.46,10.7	0
0.46,10.8	0
0.46,10.9	0
0.46,11.0	0
0.46,11.1	0
0.46,11.2	0
0.46,11.3	0
0.46,11.4	0
0.46,11.5	0
0.46,11.6	0
0.46,11.7	0
0.46,11.8	0
0.46,11.9	0
0.46,12.0	0
0.46,12.1	0
0.46,12.2	0
0.46,12.3	0
0.46,12.4	0
0.46,12.5	0
0.46,12.6	0
0.46,12.7	0
0.46,12.8	0
0.46,12.9	0
0.46,13.0	0
0.46,13.1	0
0.46,13.2	0
0.46,13.3	0
0.46,13.4	0
0.46,13.5	0
0.46,13.6	0
0.46,13.7	0
0.46,13.8	0
0.46,13.9	0
0.46,14.0	0
0.46,14.1	0
0.46,14.2	0
0.46,14.3	0
0.46,14.4	0
0.46,14.5	0

0.46,14.6	0
0.46,14.7	0
0.46,14.8	0
0.46,14.9	0
0.46,15.0	0
0.46,15.1	0
0.46,15.2	0
0.46,15.3	0
0.46,15.4	0
0.46,15.5	0
0.46,15.6	0
0.46,15.7	0
0.46,15.8	0
0.46,15.9	0
0.46,16.0	0
0.46,16.1	0
0.46,16.2	0
0.46,16.3	0
0.46,16.4	0
0.46,16.5	0
0.46,16.6	0
0.46,16.7	0
0.46,16.8	0
0.46,16.9	0
0.46,17.0	0
0.46,17.1	0
0.46,17.2	0
0.46,17.3	0
0.46,17.4	0
0.46,17.5	0
0.46,17.6	0
0.46,17.7	0
0.46,17.8	0
0.46,17.9	0
0.46,18.0	0
0.46,18.1	0
0.46,18.2	0
0.46,18.3	0
0.46,18.4	0
0.46,18.5	0
0.46,18.6	0
0.46,18.7	0

0.46,18.8	0
0.46,18.9	0
0.46,19.0	0
0.46,19.1	0
0.46,19.2	0
0.46,19.3	0
0.46,19.4	0
0.46,19.5	0
0.46,19.6	0
0.46,19.7	0
0.46,19.8	0
0.46,19.9	0
0.46,20.0	0
0.46,20.1	0
0.46,20.2	0
0.46,20.3	0
0.46,20.4	0
0.46,20.5	0
0.46,20.6	0
0.46,20.7	0
0.46,20.8	0
0.46,20.9	0
0.46,21.0	0
0.46,21.1	0
0.46,21.2	0
0.46,21.3	0
0.46,21.4	0
0.46,21.5	0
0.46,21.6	0
0.46,21.7	0
0.46,21.8	0
0.46,21.9	0
0.46,22.0	0
0.46,22.1	0
0.46,22.2	0
0.46,22.3	0
0.46,22.4	0
0.46,22.5	0
0.46,22.6	0
0.46,22.7	0
0.46,22.8	0
0.46,22.9	0

0.46,23.0	0
0.46,23.1	0
0.46,23.2	0
0.46,23.3	0
0.46,23.4	0
0.46,23.5	0
0.46,23.6	0
0.46,23.7	0
0.46,23.8	0
0.46,23.9	0
0.46,24.0	0
0.46,24.1	0
0.46,24.2	0
0.46,24.3	0
0.46,24.4	0
0.46,24.5	0
0.46,24.6	0
0.46,24.7	0
0.46,24.8	0
0.46,24.9	0
0.46,25.0	0
0.46,25.1	0
0.46,25.2	0
0.46,25.3	0
0.46,25.4	0
0.46,25.5	0
0.46,25.6	0
0.46,25.7	0
0.46,25.8	0
0.46,25.9	0
0.46,26.0	0
0.46,26.1	0
0.46,26.2	0
0.46,26.3	0
0.46,26.4	0
0.46,26.5	0
0.46,26.6	0
0.46,26.7	0
0.46,26.8	0
0.46,26.9	0
0.46,27.0	0
0.46,27.1	0

0.46,27.2	0
0.46,27.3	0
0.46,27.4	0
0.46,27.5	0
0.46,27.6	0
0.46,27.7	0
0.46,27.8	0
0.46,27.9	0
0.46,28.0	0
0.46,28.1	0
0.46,28.2	0
0.46,28.3	0
0.46,28.4	0
0.46,28.5	0
0.46,28.6	0
0.46,28.7	0
0.46,28.8	0
0.46,28.9	0
0.46,29.0	0
0.46,29.1	0
0.46,29.2	0
0.46,29.3	0
0.46,29.4	0
0.46,29.5	0
0.46,29.6	0
0.46,29.7	0
0.46,29.8	0
0.46,29.9	0
0.46,30.0	0
0.46,30.1	0
0.46,30.2	0
0.46,30.3	0
0.46,30.4	0
0.46,30.5	0
0.46,30.6	0
0.46,30.7	0
0.46,30.8	0
0.46,30.9	0
0.46,31.0	0
0.46,31.1	0
0.46,31.2	0
0.46,31.3	0

0.46,31.4	0
0.46,31.5	0
0.46,31.6	0
0.46,31.7	0
0.46,31.8	0
0.46,31.9	0
0.46,32.0	0
0.46,32.1	0
0.46,32.2	0
0.46,32.3	0
0.46,32.4	0
0.46,32.5	0
0.46,32.6	0
0.46,32.7	0
0.46,32.8	0
0.46,32.9	0
0.46,33.0	0
0.46,33.1	0
0.46,33.2	0
0.46,33.3	0
0.46,33.4	0
0.46,33.5	0
0.46,33.6	0
0.46,33.7	0
0.46,33.8	0
0.46,33.9	0
0.46,34.0	0
0.46,34.1	0
0.46,34.2	0
0.46,34.3	0
0.46,34.4	0
0.46,34.5	0
0.46,34.6	0
0.46,34.7	0
0.46,34.8	0
0.46,34.9	0
0.46,35.0	0
0.46,35.1	0
0.46,35.2	0
0.46,35.3	0
0.46,35.4	0
0.46,35.5	0

0.46,35.6	0
0.46,35.7	0
0.46,35.8	0
0.46,35.9	0
0.46,36.0	0
0.46,36.1	0
0.46,36.2	0
0.46,36.3	0
0.46,36.4	0
0.46,36.5	0
0.46,36.6	0
0.46,36.7	0
0.46,36.8	0
0.46,36.9	0
0.46,37.0	0
0.46,37.1	0
0.46,37.2	0
0.46,37.3	0
0.46,37.4	0
0.46,37.5	0
0.46,37.6	0
0.46,37.7	0
0.46,37.8	0
0.46,37.9	0
0.46,38.0	0
0.46,38.1	0
0.46,38.2	0
0.46,38.3	0
0.46,38.4	0
0.46,38.5	0
0.46,38.6	0
0.46,38.7	0
0.46,38.8	0
0.46,38.9	0
0.46,39.0	0
0.46,39.1	0
0.46,39.2	0
0.46,39.3	0
0.46,39.4	0
0.46,39.5	0
0.46,39.6	0
0.46,39.7	0

0.46,39.8	0
0.46,39.9	0
0.46,40.0	0
0.46,40.1	0
0.46,40.2	0
0.46,40.3	0
0.46,40.4	0
0.46,40.5	0
0.46,40.6	0
0.46,40.7	0
0.46,40.8	0
0.46,40.9	0
0.46,41.0	0
0.46,41.1	0
0.46,41.2	0
0.46,41.3	0
0.46,41.4	0
0.46,41.5	0
0.46,41.6	0
0.46,41.7	0
0.46,41.8	0
0.46,41.9	0
0.46,42.0	0
0.46,42.1	0
0.46,42.2	0
0.46,42.3	0
0.46,42.4	0
0.46,42.5	0
0.46,42.6	0
0.46,42.7	0
0.46,42.8	0
0.46,42.9	0
0.46,43.0	0
0.46,43.1	0
0.46,43.2	0
0.46,43.3	0
0.46,43.4	0
0.46,43.5	0
0.46,43.6	0
0.46,43.7	0
0.46,43.8	0
0.46,43.9	0

0.46,44.0	0
0.46,44.1	0
0.46,44.2	0
0.46,44.3	0
0.46,44.4	0
0.46,44.5	0
0.46,44.6	0
0.46,44.7	0
0.46,44.8	0
0.46,44.9	0
0.46,45.0	0
0.46,45.1	0
0.46,45.2	0
0.46,45.3	0
0.46,45.4	0
0.46,45.5	0
0.46,45.6	0
0.46,45.7	0
0.46,45.8	0
0.46,45.9	0
0.46,46.0	0
0.46,46.1	0
0.46,46.2	0
0.46,46.3	0
0.46,46.4	0
0.46,46.5	0
0.46,46.6	0
0.46,46.7	0
0.46,46.8	0
0.46,46.9	0
0.46,47.0	0
0.46,47.1	0
0.46,47.2	0
0.46,47.3	0
0.46,47.4	0
0.46,47.5	0
0.46,47.6	0
0.46,47.7	0
0.46,47.8	0
0.46,47.9	0
0.46,48.0	0
0.46,48.1	0

0.46,48.2	0
0.46,48.3	0
0.46,48.4	0
0.46,48.5	0
0.46,48.6	0
0.46,48.7	0
0.46,48.8	0
0.46,48.9	0
0.46,49.0	0
0.46,49.1	0
0.46,49.2	0
0.46,49.3	0
0.46,49.4	0
0.46,49.5	0
0.46,49.6	0
0.46,49.7	0
0.46,49.8	0
0.46,49.9	0
0.46,50.0	0
0.46,50.1	0
0.46,50.2	0
0.46,50.3	0
0.46,50.4	0
0.46,50.5	0
0.46,50.6	0
0.46,50.7	0
0.46,50.8	0
0.46,50.9	0
0.46,51.0	0
0.46,51.1	0
0.46,51.2	0
0.46,51.3	0
0.46,51.4	0
0.46,51.5	0
0.46,51.6	0
0.46,51.7	0
0.46,51.8	0
0.46,51.9	0
0.46,52.0	0
0.46,52.1	0
0.46,52.2	0
0.46,52.3	0

0.46,52.4	0
0.46,52.5	0
0.46,52.6	0
0.46,52.7	0
0.46,52.8	0
0.46,52.9	0
0.46,53.0	0
0.46,53.1	0
0.46,53.2	0
0.46,53.3	0
0.46,53.4	0
0.46,53.5	0
0.46,53.6	0
0.46,53.7	0
0.46,53.8	0
0.46,53.9	0
0.46,54.0	0
0.46,54.1	0
0.46,54.2	0
0.46,54.3	0
0.46,54.4	0
0.46,54.5	0
0.46,54.6	0
0.46,54.7	0
0.46,54.8	0
0.46,54.9	0
0.46,55.0	0
0.46,55.1	0
0.46,55.2	0
0.46,55.3	0
0.46,55.4	0
0.46,55.5	0
0.46,55.6	0
0.46,55.7	0
0.46,55.8	0
0.46,55.9	0
0.46,56.0	0
0.46,56.1	0
0.46,56.2	0
0.46,56.3	0
0.46,56.4	0
0.46,56.5	0

0.46,56.6	0
0.46,56.7	0
0.46,56.8	0
0.46,56.9	0
0.46,57.0	0
0.46,57.1	0
0.46,57.2	0
0.46,57.3	0
0.46,57.4	0
0.46,57.5	0
0.46,57.6	0
0.46,57.7	0
0.46,57.8	0
0.46,57.9	0
0.46,58.0	0
0.46,58.1	0
0.46,58.2	0
0.46,58.3	0
0.46,58.4	0
0.46,58.5	0
0.46,58.6	0
0.46,58.7	0
0.46,58.8	0
0.46,58.9	0
0.46,59.0	0
0.46,59.1	0
0.46,59.2	0
0.46,59.3	0
0.46,59.4	0
0.46,59.5	0
0.46,59.6	0
0.46,59.7	0
0.46,59.8	0
0.46,59.9	0
0.46,60.0	0
0.46,60.1	0
0.46,60.2	0
0.46,60.3	0
0.46,60.4	0
0.46,60.5	0
0.46,60.6	0
0.46,60.7	0

0.46,60.8	0
0.46,60.9	0
0.46,61.0	0
0.46,61.1	0
0.46,61.2	0
0.46,61.3	0
0.46,61.4	0
0.46,61.5	0
0.46,61.6	0
0.46,61.7	0
0.46,61.8	0
0.46,61.9	0
0.46,62.0	0
0.46,62.1	0
0.46,62.2	0
0.46,62.3	0
0.46,62.4	0
0.46,62.5	0
0.46,62.6	0
0.46,62.7	0
0.46,62.8	0
0.46,62.9	0
0.46,63.0	0
0.46,63.1	0
0.46,63.2	0
0.46,63.3	0
0.46,63.4	0
0.46,63.5	0
0.46,63.6	0
0.46,63.7	0
0.46,63.8	0
0.46,63.9	0
0.46,64.0	0
0.46,64.1	0
0.46,64.2	0
0.46,64.3	0
0.46,64.4	0
0.46,64.5	0
0.46,64.6	0
0.46,64.7	0
0.46,64.8	0
0.46,64.9	0

0.46,65.0	0
0.46,65.1	0
0.46,65.2	0
0.46,65.3	0
0.46,65.4	0
0.46,65.5	0
0.46,65.6	0
0.46,65.7	0
0.46,65.8	0
0.46,65.9	0
0.46,66.0	0
0.46,66.1	0
0.46,66.2	0
0.46,66.3	0
0.46,66.4	0
0.46,66.5	0
0.46,66.6	0
0.46,66.7	0
0.46,66.8	0
0.46,66.9	0
0.46,67.0	0
0.46,67.1	0
0.46,67.2	0
0.46,67.3	0
0.46,67.4	0
0.46,67.5	0
0.46,67.6	0
0.46,67.7	0
0.46,67.8	0
0.46,67.9	0
0.46,68.0	0
0.46,68.1	0
0.46,68.2	0
0.46,68.3	0
0.46,68.4	0
0.46,68.5	0
0.46,68.6	0
0.46,68.7	0
0.46,68.8	0
0.46,68.9	0
0.46,69.0	0
0.46,69.1	0

0.46,69.2	0
0.46,69.3	0
0.46,69.4	0
0.46,69.5	0
0.46,69.6	0
0.46,69.7	0
0.46,69.8	0
0.46,69.9	0
0.46,70.0	0
0.46,70.1	0
0.46,70.2	0
0.46,70.3	0
0.46,70.4	0
0.46,70.5	0
0.46,70.6	0
0.46,70.7	0
0.46,70.8	0
0.46,70.9	0
0.46,71.0	0
0.46,71.1	0
0.46,71.2	0
0.46,71.3	0
0.46,71.4	0
0.46,71.5	0
0.46,71.6	0
0.46,71.7	0
0.46,71.8	0
0.46,71.9	0
0.46,72.0	0
0.46,72.1	0
0.46,72.2	0
0.46,72.3	0
0.46,72.4	0
0.46,72.5	0
0.46,72.6	0
0.46,72.7	0
0.46,72.8	0
0.46,72.9	0
0.46,73.0	0
0.46,73.1	0
0.46,73.2	0
0.46,73.3	0

0.46,73.4	0
0.46,73.5	0
0.46,73.6	0
0.46,73.7	0
0.46,73.8	0
0.46,73.9	0
0.46,74.0	0
0.46,74.1	0
0.46,74.2	0
0.46,74.3	0
0.46,74.4	0
0.46,74.5	0
0.46,74.6	0
0.46,74.7	0
0.46,74.8	0
0.46,74.9	0
0.46,75.0	0
0.46,75.1	0
0.46,75.2	0
0.46,75.3	0
0.46,75.4	0
0.46,75.5	0
0.46,75.6	0
0.46,75.7	0
0.46,75.8	0
0.46,75.9	0
0.46,76.0	0
0.46,76.1	0
0.46,76.2	0
0.46,76.3	0
0.46,76.4	0
0.46,76.5	0
0.46,76.6	0
0.46,76.7	0
0.46,76.8	0
0.46,76.9	0
0.46,77.0	0
0.46,77.1	0
0.46,77.2	0
0.46,77.3	0
0.46,77.4	0
0.46,77.5	0

0.46,77.6	0
0.46,77.7	0
0.46,77.8	0
0.46,77.9	0
0.46,78.0	0
0.46,78.1	0
0.46,78.2	0
0.46,78.3	0
0.46,78.4	0
0.46,78.5	0
0.46,78.6	0
0.46,78.7	0
0.46,78.8	0
0.46,78.9	0
0.46,79.0	0
0.46,79.1	0
0.46,79.2	0
0.46,79.3	0
0.46,79.4	0
0.46,79.5	0
0.46,79.6	0
0.46,79.7	0
0.46,79.8	0
0.46,79.9	0
0.46,80.0	0
0.46,80.1	0
0.46,80.2	0
0.46,80.3	0
0.46,80.4	0
0.46,80.5	0
0.46,80.6	0
0.46,80.7	0
0.46,80.8	0
0.46,80.9	0
0.46,81.0	0
0.46,81.1	0
0.46,81.2	0
0.46,81.3	0
0.46,81.4	0
0.46,81.5	0
0.46,81.6	0
0.46,81.7	0

0.46,81.8	0
0.46,81.9	0
0.46,82.0	0
0.46,82.1	0
0.46,82.2	0
0.46,82.3	0
0.46,82.4	0
0.46,82.5	0
0.46,82.6	0
0.46,82.7	0
0.46,82.8	0
0.46,82.9	0
0.46,83.0	0
0.46,83.1	0
0.46,83.2	0
0.46,83.3	0
0.46,83.4	0
0.46,83.5	0
0.46,83.6	0
0.46,83.7	0
0.46,83.8	0
0.46,83.9	0
0.46,84.0	0
0.46,84.1	0
0.46,84.2	0
0.46,84.3	0
0.46,84.4	0
0.46,84.5	0
0.46,84.6	0
0.46,84.7	0
0.46,84.8	0
0.46,84.9	0
0.46,85.0	0
0.46,85.1	0
0.46,85.2	0
0.46,85.3	0
0.46,85.4	0
0.46,85.5	0
0.46,85.6	0
0.46,85.7	0
0.46,85.8	0
0.46,85.9	0

0.46,86.0	0
0.46,86.1	0
0.46,86.2	0
0.46,86.3	0
0.46,86.4	0
0.46,86.5	0
0.46,86.6	0
0.46,86.7	0
0.46,86.8	0
0.46,86.9	0
0.46,87.0	0
0.46,87.1	0
0.46,87.2	0
0.46,87.3	0
0.46,87.4	0
0.46,87.5	0
0.46,87.6	0
0.46,87.7	0
0.46,87.8	0
0.46,87.9	0
0.46,88.0	0
0.46,88.1	0
0.46,88.2	0
0.46,88.3	0
0.46,88.4	0
0.46,88.5	0
0.46,88.6	0
0.46,88.7	0
0.46,88.8	0
0.46,88.9	0
0.46,89.0	0
0.46,89.1	0
0.46,89.2	0
0.46,89.3	0
0.46,89.4	0
0.46,89.5	0
0.46,89.6	0
0.46,89.7	0
0.46,89.8	0
0.46,89.9	0
0.46,90.0	0
0.46,90.1	0

0.46,90.2	0
0.46,90.3	0
0.46,90.4	0
0.46,90.5	0
0.46,90.6	0
0.46,90.7	0
0.46,90.8	0
0.46,90.9	0
0.46,91.0	0
0.46,91.1	0
0.46,91.2	0
0.46,91.3	0
0.46,91.4	0
0.46,91.5	0
0.46,91.6	0
0.46,91.7	0
0.46,91.8	0
0.46,91.9	0
0.46,92.0	0
0.46,92.1	0
0.46,92.2	0
0.46,92.3	0
0.46,92.4	0
0.46,92.5	0
0.46,92.6	0
0.46,92.7	0
0.46,92.8	0
0.46,92.9	0
0.46,93.0	0
0.46,93.1	0
0.46,93.2	0
0.46,93.3	0
0.46,93.4	0
0.46,93.5	0
0.46,93.6	0
0.46,93.7	0
0.46,93.8	0
0.46,93.9	0
0.46,94.0	0
0.46,94.1	0
0.46,94.2	0
0.46,94.3	0

0.46,94.4	0
0.46,94.5	0
0.46,94.6	0
0.46,94.7	0
0.46,94.8	0
0.46,94.9	0
0.46,95.0	0
0.46,95.1	0
0.46,95.2	0
0.46,95.3	0
0.46,95.4	0
0.46,95.5	0
0.46,95.6	0
0.46,95.7	0
0.46,95.8	0
0.46,95.9	0
0.46,96.0	0
0.46,96.1	0
0.46,96.2	0
0.46,96.3	0
0.46,96.4	0
0.46,96.5	0
0.46,96.6	0
0.46,96.7	0
0.46,96.8	0
0.46,96.9	0
0.46,97.0	0
0.46,97.1	0
0.46,97.2	0
0.46,97.3	0
0.46,97.4	0
0.46,97.5	0
0.46,97.6	0
0.46,97.7	0
0.46,97.8	0
0.46,97.9	0
0.46,98.0	0
0.46,98.1	0
0.46,98.2	0
0.46,98.3	0
0.46,98.4	0
0.46,98.5	0

0.46,98.6	0
0.46,98.7	0
0.46,98.8	0
0.46,98.9	0
0.46,99.0	0
0.46,99.1	0
0.46,99.2	0
0.46,99.3	0
0.46,99.4	0
0.46,99.5	0
0.46,99.6	0
0.46,99.7	0
0.46,99.8	0
0.46,99.9	0
0.46,100.0	0
0.47,0.0	0
0.47,0.1	0
0.47,0.2	0
0.47,0.3	0
0.47,0.4	0
0.47,0.5	0
0.47,0.6	0
0.47,0.7	0
0.47,0.8	0
0.47,0.9	0
0.47,1.0	0
0.47,1.1	0
0.47,1.2	0
0.47,1.3	0
0.47,1.4	0
0.47,1.5	0
0.47,1.6	0
0.47,1.7	0
0.47,1.8	0
0.47,1.9	0
0.47,2.0	0
0.47,2.1	0
0.47,2.2	0
0.47,2.3	0
0.47,2.4	0
0.47,2.5	0
0.47,2.6	0

0.47,2.7	0
0.47,2.8	0
0.47,2.9	0
0.47,3.0	0
0.47,3.1	0
0.47,3.2	0
0.47,3.3	0
0.47,3.4	0
0.47,3.5	0
0.47,3.6	0
0.47,3.7	0
0.47,3.8	0
0.47,3.9	0
0.47,4.0	0
0.47,4.1	0
0.47,4.2	0
0.47,4.3	0
0.47,4.4	0
0.47,4.5	0
0.47,4.6	0
0.47,4.7	0
0.47,4.8	0
0.47,4.9	0
0.47,5.0	0
0.47,5.1	0
0.47,5.2	0
0.47,5.3	0
0.47,5.4	0
0.47,5.5	0
0.47,5.6	0
0.47,5.7	0
0.47,5.8	0
0.47,5.9	0
0.47,6.0	0
0.47,6.1	0
0.47,6.2	0
0.47,6.3	0
0.47,6.4	0
0.47,6.5	0
0.47,6.6	0
0.47,6.7	0
0.47,6.8	0

0.47,6.9	0
0.47,7.0	0
0.47,7.1	0
0.47,7.2	0
0.47,7.3	0
0.47,7.4	0
0.47,7.5	0
0.47,7.6	0
0.47,7.7	0
0.47,7.8	0
0.47,7.9	0
0.47,8.0	0
0.47,8.1	0
0.47,8.2	0
0.47,8.3	0
0.47,8.4	0
0.47,8.5	0
0.47,8.6	0
0.47,8.7	0
0.47,8.8	0
0.47,8.9	0
0.47,9.0	0
0.47,9.1	0
0.47,9.2	0
0.47,9.3	0
0.47,9.4	0
0.47,9.5	0
0.47,9.6	0
0.47,9.7	0
0.47,9.8	0
0.47,9.9	0
0.47,10.0	0
0.47,10.1	0
0.47,10.2	0
0.47,10.3	0
0.47,10.4	0
0.47,10.5	0
0.47,10.6	0
0.47,10.7	0
0.47,10.8	0
0.47,10.9	0
0.47,11.0	0

0.47,11.1	0
0.47,11.2	0
0.47,11.3	0
0.47,11.4	0
0.47,11.5	0
0.47,11.6	0
0.47,11.7	0
0.47,11.8	0
0.47,11.9	0
0.47,12.0	0
0.47,12.1	0
0.47,12.2	0
0.47,12.3	0
0.47,12.4	0
0.47,12.5	0
0.47,12.6	0
0.47,12.7	0
0.47,12.8	0
0.47,12.9	0
0.47,13.0	0
0.47,13.1	0
0.47,13.2	0
0.47,13.3	0
0.47,13.4	0
0.47,13.5	0
0.47,13.6	0
0.47,13.7	0
0.47,13.8	0
0.47,13.9	0
0.47,14.0	0
0.47,14.1	0
0.47,14.2	0
0.47,14.3	0
0.47,14.4	0
0.47,14.5	0
0.47,14.6	0
0.47,14.7	0
0.47,14.8	0
0.47,14.9	0
0.47,15.0	0
0.47,15.1	0
0.47,15.2	0

0.47,15.3	0
0.47,15.4	0
0.47,15.5	0
0.47,15.6	0
0.47,15.7	0
0.47,15.8	0
0.47,15.9	0
0.47,16.0	0
0.47,16.1	0
0.47,16.2	0
0.47,16.3	0
0.47,16.4	0
0.47,16.5	0
0.47,16.6	0
0.47,16.7	0
0.47,16.8	0
0.47,16.9	0
0.47,17.0	0
0.47,17.1	0
0.47,17.2	0
0.47,17.3	0
0.47,17.4	0
0.47,17.5	0
0.47,17.6	0
0.47,17.7	0
0.47,17.8	0
0.47,17.9	0
0.47,18.0	0
0.47,18.1	0
0.47,18.2	0
0.47,18.3	0
0.47,18.4	0
0.47,18.5	0
0.47,18.6	0
0.47,18.7	0
0.47,18.8	0
0.47,18.9	0
0.47,19.0	0
0.47,19.1	0
0.47,19.2	0
0.47,19.3	0
0.47,19.4	0

0.47,19.5	0
0.47,19.6	0
0.47,19.7	0
0.47,19.8	0
0.47,19.9	0
0.47,20.0	0
0.47,20.1	0
0.47,20.2	0
0.47,20.3	0
0.47,20.4	0
0.47,20.5	0
0.47,20.6	0
0.47,20.7	0
0.47,20.8	0
0.47,20.9	0
0.47,21.0	0
0.47,21.1	0
0.47,21.2	0
0.47,21.3	0
0.47,21.4	0
0.47,21.5	0
0.47,21.6	0
0.47,21.7	0
0.47,21.8	0
0.47,21.9	0
0.47,22.0	0
0.47,22.1	0
0.47,22.2	0
0.47,22.3	0
0.47,22.4	0
0.47,22.5	0
0.47,22.6	0
0.47,22.7	0
0.47,22.8	0
0.47,22.9	0
0.47,23.0	0
0.47,23.1	0
0.47,23.2	0
0.47,23.3	0
0.47,23.4	0
0.47,23.5	0
0.47,23.6	0

0.47,23.7	0
0.47,23.8	0
0.47,23.9	0
0.47,24.0	0
0.47,24.1	0
0.47,24.2	0
0.47,24.3	0
0.47,24.4	0
0.47,24.5	0
0.47,24.6	0
0.47,24.7	0
0.47,24.8	0
0.47,24.9	0
0.47,25.0	0
0.47,25.1	0
0.47,25.2	0
0.47,25.3	0
0.47,25.4	0
0.47,25.5	0
0.47,25.6	0
0.47,25.7	0
0.47,25.8	0
0.47,25.9	0
0.47,26.0	0
0.47,26.1	0
0.47,26.2	0
0.47,26.3	0
0.47,26.4	0
0.47,26.5	0
0.47,26.6	0
0.47,26.7	0
0.47,26.8	0
0.47,26.9	0
0.47,27.0	0
0.47,27.1	0
0.47,27.2	0
0.47,27.3	0
0.47,27.4	0
0.47,27.5	0
0.47,27.6	0
0.47,27.7	0
0.47,27.8	0

0.47,27.9	0
0.47,28.0	0
0.47,28.1	0
0.47,28.2	0
0.47,28.3	0
0.47,28.4	0
0.47,28.5	0
0.47,28.6	0
0.47,28.7	0
0.47,28.8	0
0.47,28.9	0
0.47,29.0	0
0.47,29.1	0
0.47,29.2	0
0.47,29.3	0
0.47,29.4	0
0.47,29.5	0
0.47,29.6	0
0.47,29.7	0
0.47,29.8	0
0.47,29.9	0
0.47,30.0	0
0.47,30.1	0
0.47,30.2	0
0.47,30.3	0
0.47,30.4	0
0.47,30.5	0
0.47,30.6	0
0.47,30.7	0
0.47,30.8	0
0.47,30.9	0
0.47,31.0	0
0.47,31.1	0
0.47,31.2	0
0.47,31.3	0
0.47,31.4	0
0.47,31.5	0
0.47,31.6	0
0.47,31.7	0
0.47,31.8	0
0.47,31.9	0
0.47,32.0	0

0.47,32.1	0
0.47,32.2	0
0.47,32.3	0
0.47,32.4	0
0.47,32.5	0
0.47,32.6	0
0.47,32.7	0
0.47,32.8	0
0.47,32.9	0
0.47,33.0	0
0.47,33.1	0
0.47,33.2	0
0.47,33.3	0
0.47,33.4	0
0.47,33.5	0
0.47,33.6	0
0.47,33.7	0
0.47,33.8	0
0.47,33.9	0
0.47,34.0	0
0.47,34.1	0
0.47,34.2	0
0.47,34.3	0
0.47,34.4	0
0.47,34.5	0
0.47,34.6	0
0.47,34.7	0
0.47,34.8	0
0.47,34.9	0
0.47,35.0	0
0.47,35.1	0
0.47,35.2	0
0.47,35.3	0
0.47,35.4	0
0.47,35.5	0
0.47,35.6	0
0.47,35.7	0
0.47,35.8	0
0.47,35.9	0
0.47,36.0	0
0.47,36.1	0
0.47,36.2	0

0.47,36.3	0
0.47,36.4	0
0.47,36.5	0
0.47,36.6	0
0.47,36.7	0
0.47,36.8	0
0.47,36.9	0
0.47,37.0	0
0.47,37.1	0
0.47,37.2	0
0.47,37.3	0
0.47,37.4	0
0.47,37.5	0
0.47,37.6	0
0.47,37.7	0
0.47,37.8	0
0.47,37.9	0
0.47,38.0	0
0.47,38.1	0
0.47,38.2	0
0.47,38.3	0
0.47,38.4	0
0.47,38.5	0
0.47,38.6	0
0.47,38.7	0
0.47,38.8	0
0.47,38.9	0
0.47,39.0	0
0.47,39.1	0
0.47,39.2	0
0.47,39.3	0
0.47,39.4	0
0.47,39.5	0
0.47,39.6	0
0.47,39.7	0
0.47,39.8	0
0.47,39.9	0
0.47,40.0	0
0.47,40.1	0
0.47,40.2	0
0.47,40.3	0
0.47,40.4	0

0.47,40.5	0
0.47,40.6	0
0.47,40.7	0
0.47,40.8	0
0.47,40.9	0
0.47,41.0	0
0.47,41.1	0
0.47,41.2	0
0.47,41.3	0
0.47,41.4	0
0.47,41.5	0
0.47,41.6	0
0.47,41.7	0
0.47,41.8	0
0.47,41.9	0
0.47,42.0	0
0.47,42.1	0
0.47,42.2	0
0.47,42.3	0
0.47,42.4	0
0.47,42.5	0
0.47,42.6	0
0.47,42.7	0
0.47,42.8	0
0.47,42.9	0
0.47,43.0	0
0.47,43.1	0
0.47,43.2	0
0.47,43.3	0
0.47,43.4	0
0.47,43.5	0
0.47,43.6	0
0.47,43.7	0
0.47,43.8	0
0.47,43.9	0
0.47,44.0	0
0.47,44.1	0
0.47,44.2	0
0.47,44.3	0
0.47,44.4	0
0.47,44.5	0
0.47,44.6	0

0.47,44.7	0
0.47,44.8	0
0.47,44.9	0
0.47,45.0	0
0.47,45.1	0
0.47,45.2	0
0.47,45.3	0
0.47,45.4	0
0.47,45.5	0
0.47,45.6	0
0.47,45.7	0
0.47,45.8	0
0.47,45.9	0
0.47,46.0	0
0.47,46.1	0
0.47,46.2	0
0.47,46.3	0
0.47,46.4	0
0.47,46.5	0
0.47,46.6	0
0.47,46.7	0
0.47,46.8	0
0.47,46.9	0
0.47,47.0	0
0.47,47.1	0
0.47,47.2	0
0.47,47.3	0
0.47,47.4	0
0.47,47.5	0
0.47,47.6	0
0.47,47.7	0
0.47,47.8	0
0.47,47.9	0
0.47,48.0	0
0.47,48.1	0
0.47,48.2	0
0.47,48.3	0
0.47,48.4	0
0.47,48.5	0
0.47,48.6	0
0.47,48.7	0
0.47,48.8	0

0.47,48.9	0
0.47,49.0	0
0.47,49.1	0
0.47,49.2	0
0.47,49.3	0
0.47,49.4	0
0.47,49.5	0
0.47,49.6	0
0.47,49.7	0
0.47,49.8	0
0.47,49.9	0
0.47,50.0	0
0.47,50.1	0
0.47,50.2	0
0.47,50.3	0
0.47,50.4	0
0.47,50.5	0
0.47,50.6	0
0.47,50.7	0
0.47,50.8	0
0.47,50.9	0
0.47,51.0	0
0.47,51.1	0
0.47,51.2	0
0.47,51.3	0
0.47,51.4	0
0.47,51.5	0
0.47,51.6	0
0.47,51.7	0
0.47,51.8	0
0.47,51.9	0
0.47,52.0	0
0.47,52.1	0
0.47,52.2	0
0.47,52.3	0
0.47,52.4	0
0.47,52.5	0
0.47,52.6	0
0.47,52.7	0
0.47,52.8	0
0.47,52.9	0
0.47,53.0	0

0.47,53.1	0
0.47,53.2	0
0.47,53.3	0
0.47,53.4	0
0.47,53.5	0
0.47,53.6	0
0.47,53.7	0
0.47,53.8	0
0.47,53.9	0
0.47,54.0	0
0.47,54.1	0
0.47,54.2	0
0.47,54.3	0
0.47,54.4	0
0.47,54.5	0
0.47,54.6	0
0.47,54.7	0
0.47,54.8	0
0.47,54.9	0
0.47,55.0	0
0.47,55.1	0
0.47,55.2	0
0.47,55.3	0
0.47,55.4	0
0.47,55.5	0
0.47,55.6	0
0.47,55.7	0
0.47,55.8	0
0.47,55.9	0
0.47,56.0	0
0.47,56.1	0
0.47,56.2	0
0.47,56.3	0
0.47,56.4	0
0.47,56.5	0
0.47,56.6	0
0.47,56.7	0
0.47,56.8	0
0.47,56.9	0
0.47,57.0	0
0.47,57.1	0
0.47,57.2	0

0.47,57.3	0
0.47,57.4	0
0.47,57.5	0
0.47,57.6	0
0.47,57.7	0
0.47,57.8	0
0.47,57.9	0
0.47,58.0	0
0.47,58.1	0
0.47,58.2	0
0.47,58.3	0
0.47,58.4	0
0.47,58.5	0
0.47,58.6	0
0.47,58.7	0
0.47,58.8	0
0.47,58.9	0
0.47,59.0	0
0.47,59.1	0
0.47,59.2	0
0.47,59.3	0
0.47,59.4	0
0.47,59.5	0
0.47,59.6	0
0.47,59.7	0
0.47,59.8	0
0.47,59.9	0
0.47,60.0	0
0.47,60.1	0
0.47,60.2	0
0.47,60.3	0
0.47,60.4	0
0.47,60.5	0
0.47,60.6	0
0.47,60.7	0
0.47,60.8	0
0.47,60.9	0
0.47,61.0	0
0.47,61.1	0
0.47,61.2	0
0.47,61.3	0
0.47,61.4	0

0.47,61.5	0
0.47,61.6	0
0.47,61.7	0
0.47,61.8	0
0.47,61.9	0
0.47,62.0	0
0.47,62.1	0
0.47,62.2	0
0.47,62.3	0
0.47,62.4	0
0.47,62.5	0
0.47,62.6	0
0.47,62.7	0
0.47,62.8	0
0.47,62.9	0
0.47,63.0	0
0.47,63.1	0
0.47,63.2	0
0.47,63.3	0
0.47,63.4	0
0.47,63.5	0
0.47,63.6	0
0.47,63.7	0
0.47,63.8	0
0.47,63.9	0
0.47,64.0	0
0.47,64.1	0
0.47,64.2	0
0.47,64.3	0
0.47,64.4	0
0.47,64.5	0
0.47,64.6	0
0.47,64.7	0
0.47,64.8	0
0.47,64.9	0
0.47,65.0	0
0.47,65.1	0
0.47,65.2	0
0.47,65.3	0
0.47,65.4	0
0.47,65.5	0
0.47,65.6	0

0.47,65.7	0
0.47,65.8	0
0.47,65.9	0
0.47,66.0	0
0.47,66.1	0
0.47,66.2	0
0.47,66.3	0
0.47,66.4	0
0.47,66.5	0
0.47,66.6	0
0.47,66.7	0
0.47,66.8	0
0.47,66.9	0
0.47,67.0	0
0.47,67.1	0
0.47,67.2	0
0.47,67.3	0
0.47,67.4	0
0.47,67.5	0
0.47,67.6	0
0.47,67.7	0
0.47,67.8	0
0.47,67.9	0
0.47,68.0	0
0.47,68.1	0
0.47,68.2	0
0.47,68.3	0
0.47,68.4	0
0.47,68.5	0
0.47,68.6	0
0.47,68.7	0
0.47,68.8	0
0.47,68.9	0
0.47,69.0	0
0.47,69.1	0
0.47,69.2	0
0.47,69.3	0
0.47,69.4	0
0.47,69.5	0
0.47,69.6	0
0.47,69.7	0
0.47,69.8	0

0.47,69.9	0
0.47,70.0	0
0.47,70.1	0
0.47,70.2	0
0.47,70.3	0
0.47,70.4	0
0.47,70.5	0
0.47,70.6	0
0.47,70.7	0
0.47,70.8	0
0.47,70.9	0
0.47,71.0	0
0.47,71.1	0
0.47,71.2	0
0.47,71.3	0
0.47,71.4	0
0.47,71.5	0
0.47,71.6	0
0.47,71.7	0
0.47,71.8	0
0.47,71.9	0
0.47,72.0	0
0.47,72.1	0
0.47,72.2	0
0.47,72.3	0
0.47,72.4	0
0.47,72.5	0
0.47,72.6	0
0.47,72.7	0
0.47,72.8	0
0.47,72.9	0
0.47,73.0	0
0.47,73.1	0
0.47,73.2	0
0.47,73.3	0
0.47,73.4	0
0.47,73.5	0
0.47,73.6	0
0.47,73.7	0
0.47,73.8	0
0.47,73.9	0
0.47,74.0	0

0.47,74.1	0
0.47,74.2	0
0.47,74.3	0
0.47,74.4	0
0.47,74.5	0
0.47,74.6	0
0.47,74.7	0
0.47,74.8	0
0.47,74.9	0
0.47,75.0	0
0.47,75.1	0
0.47,75.2	0
0.47,75.3	0
0.47,75.4	0
0.47,75.5	0
0.47,75.6	0
0.47,75.7	0
0.47,75.8	0
0.47,75.9	0
0.47,76.0	0
0.47,76.1	0
0.47,76.2	0
0.47,76.3	0
0.47,76.4	0
0.47,76.5	0
0.47,76.6	0
0.47,76.7	0
0.47,76.8	0
0.47,76.9	0
0.47,77.0	0
0.47,77.1	0
0.47,77.2	0
0.47,77.3	0
0.47,77.4	0
0.47,77.5	0
0.47,77.6	0
0.47,77.7	0
0.47,77.8	0
0.47,77.9	0
0.47,78.0	0
0.47,78.1	0
0.47,78.2	0

0.47,78.3	0
0.47,78.4	0
0.47,78.5	0
0.47,78.6	0
0.47,78.7	0
0.47,78.8	0
0.47,78.9	0
0.47,79.0	0
0.47,79.1	0
0.47,79.2	0
0.47,79.3	0
0.47,79.4	0
0.47,79.5	0
0.47,79.6	0
0.47,79.7	0
0.47,79.8	0
0.47,79.9	0
0.47,80.0	0
0.47,80.1	0
0.47,80.2	0
0.47,80.3	0
0.47,80.4	0
0.47,80.5	0
0.47,80.6	0
0.47,80.7	0
0.47,80.8	0
0.47,80.9	0
0.47,81.0	0
0.47,81.1	0
0.47,81.2	0
0.47,81.3	0
0.47,81.4	0
0.47,81.5	0
0.47,81.6	0
0.47,81.7	0
0.47,81.8	0
0.47,81.9	0
0.47,82.0	0
0.47,82.1	0
0.47,82.2	0
0.47,82.3	0
0.47,82.4	0

0.47,82.5	0
0.47,82.6	0
0.47,82.7	0
0.47,82.8	0
0.47,82.9	0
0.47,83.0	0
0.47,83.1	0
0.47,83.2	0
0.47,83.3	0
0.47,83.4	0
0.47,83.5	0
0.47,83.6	0
0.47,83.7	0
0.47,83.8	0
0.47,83.9	0
0.47,84.0	0
0.47,84.1	0
0.47,84.2	0
0.47,84.3	0
0.47,84.4	0
0.47,84.5	0
0.47,84.6	0
0.47,84.7	0
0.47,84.8	0
0.47,84.9	0
0.47,85.0	0
0.47,85.1	0
0.47,85.2	0
0.47,85.3	0
0.47,85.4	0
0.47,85.5	0
0.47,85.6	0
0.47,85.7	0
0.47,85.8	0
0.47,85.9	0
0.47,86.0	0
0.47,86.1	0
0.47,86.2	0
0.47,86.3	0
0.47,86.4	0
0.47,86.5	0
0.47,86.6	0

0.47,86.7	0
0.47,86.8	0
0.47,86.9	0
0.47,87.0	0
0.47,87.1	0
0.47,87.2	0
0.47,87.3	0
0.47,87.4	0
0.47,87.5	0
0.47,87.6	0
0.47,87.7	0
0.47,87.8	0
0.47,87.9	0
0.47,88.0	0
0.47,88.1	0
0.47,88.2	0
0.47,88.3	0
0.47,88.4	0
0.47,88.5	0
0.47,88.6	0
0.47,88.7	0
0.47,88.8	0
0.47,88.9	0
0.47,89.0	0
0.47,89.1	0
0.47,89.2	0
0.47,89.3	0
0.47,89.4	0
0.47,89.5	0
0.47,89.6	0
0.47,89.7	0
0.47,89.8	0
0.47,89.9	0
0.47,90.0	0
0.47,90.1	0
0.47,90.2	0
0.47,90.3	0
0.47,90.4	0
0.47,90.5	0
0.47,90.6	0
0.47,90.7	0
0.47,90.8	0

0.47,90.9	0
0.47,91.0	0
0.47,91.1	0
0.47,91.2	0
0.47,91.3	0
0.47,91.4	0
0.47,91.5	0
0.47,91.6	0
0.47,91.7	0
0.47,91.8	0
0.47,91.9	0
0.47,92.0	0
0.47,92.1	0
0.47,92.2	0
0.47,92.3	0
0.47,92.4	0
0.47,92.5	0
0.47,92.6	0
0.47,92.7	0
0.47,92.8	0
0.47,92.9	0
0.47,93.0	0
0.47,93.1	0
0.47,93.2	0
0.47,93.3	0
0.47,93.4	0
0.47,93.5	0
0.47,93.6	0
0.47,93.7	0
0.47,93.8	0
0.47,93.9	0
0.47,94.0	0
0.47,94.1	0
0.47,94.2	0
0.47,94.3	0
0.47,94.4	0
0.47,94.5	0
0.47,94.6	0
0.47,94.7	0
0.47,94.8	0
0.47,94.9	0
0.47,95.0	0

0.47,95.1	0
0.47,95.2	0
0.47,95.3	0
0.47,95.4	0
0.47,95.5	0
0.47,95.6	0
0.47,95.7	0
0.47,95.8	0
0.47,95.9	0
0.47,96.0	0
0.47,96.1	0
0.47,96.2	0
0.47,96.3	0
0.47,96.4	0
0.47,96.5	0
0.47,96.6	0
0.47,96.7	0
0.47,96.8	0
0.47,96.9	0
0.47,97.0	0
0.47,97.1	0
0.47,97.2	0
0.47,97.3	0
0.47,97.4	0
0.47,97.5	0
0.47,97.6	0
0.47,97.7	0
0.47,97.8	0
0.47,97.9	0
0.47,98.0	0
0.47,98.1	0
0.47,98.2	0
0.47,98.3	0
0.47,98.4	0
0.47,98.5	0
0.47,98.6	0
0.47,98.7	0
0.47,98.8	0
0.47,98.9	0
0.47,99.0	0
0.47,99.1	0
0.47,99.2	0

0.47,99.3	0
0.47,99.4	0
0.47,99.5	0
0.47,99.6	0
0.47,99.7	0
0.47,99.8	0
0.47,99.9	0
0.47,100.0	0
0.48,0.0	0
0.48,0.1	0
0.48,0.2	0
0.48,0.3	0
0.48,0.4	0
0.48,0.5	0
0.48,0.6	0
0.48,0.7	0
0.48,0.8	0
0.48,0.9	0
0.48,1.0	0
0.48,1.1	0
0.48,1.2	0
0.48,1.3	0
0.48,1.4	0
0.48,1.5	0
0.48,1.6	0
0.48,1.7	0
0.48,1.8	0
0.48,1.9	0
0.48,2.0	0
0.48,2.1	0
0.48,2.2	0
0.48,2.3	0
0.48,2.4	0
0.48,2.5	0
0.48,2.6	0
0.48,2.7	0
0.48,2.8	0
0.48,2.9	0
0.48,3.0	0
0.48,3.1	0
0.48,3.2	0
0.48,3.3	0

0.48,3.4	0
0.48,3.5	0
0.48,3.6	0
0.48,3.7	0
0.48,3.8	0
0.48,3.9	0
0.48,4.0	0
0.48,4.1	0
0.48,4.2	0
0.48,4.3	0
0.48,4.4	0
0.48,4.5	0
0.48,4.6	0
0.48,4.7	0
0.48,4.8	0
0.48,4.9	0
0.48,5.0	0
0.48,5.1	0
0.48,5.2	0
0.48,5.3	0
0.48,5.4	0
0.48,5.5	0
0.48,5.6	0
0.48,5.7	0
0.48,5.8	0
0.48,5.9	0
0.48,6.0	0
0.48,6.1	0
0.48,6.2	0
0.48,6.3	0
0.48,6.4	0
0.48,6.5	0
0.48,6.6	0
0.48,6.7	0
0.48,6.8	0
0.48,6.9	0
0.48,7.0	0
0.48,7.1	0
0.48,7.2	0
0.48,7.3	0
0.48,7.4	0
0.48,7.5	0

0.48,7.6	0
0.48,7.7	0
0.48,7.8	0
0.48,7.9	0
0.48,8.0	0
0.48,8.1	0
0.48,8.2	0
0.48,8.3	0
0.48,8.4	0
0.48,8.5	0
0.48,8.6	0
0.48,8.7	0
0.48,8.8	0
0.48,8.9	0
0.48,9.0	0
0.48,9.1	0
0.48,9.2	0
0.48,9.3	0
0.48,9.4	0
0.48,9.5	0
0.48,9.6	0
0.48,9.7	0
0.48,9.8	0
0.48,9.9	0
0.48,10.0	0
0.48,10.1	0
0.48,10.2	0
0.48,10.3	0
0.48,10.4	0
0.48,10.5	0
0.48,10.6	0
0.48,10.7	0
0.48,10.8	0
0.48,10.9	0
0.48,11.0	0
0.48,11.1	0
0.48,11.2	0
0.48,11.3	0
0.48,11.4	0
0.48,11.5	0
0.48,11.6	0
0.48,11.7	0

0.48,11.8	0
0.48,11.9	0
0.48,12.0	0
0.48,12.1	0
0.48,12.2	0
0.48,12.3	0
0.48,12.4	0
0.48,12.5	0
0.48,12.6	0
0.48,12.7	0
0.48,12.8	0
0.48,12.9	0
0.48,13.0	0
0.48,13.1	0
0.48,13.2	0
0.48,13.3	0
0.48,13.4	0
0.48,13.5	0
0.48,13.6	0
0.48,13.7	0
0.48,13.8	0
0.48,13.9	0
0.48,14.0	0
0.48,14.1	0
0.48,14.2	0
0.48,14.3	0
0.48,14.4	0
0.48,14.5	0
0.48,14.6	0
0.48,14.7	0
0.48,14.8	0
0.48,14.9	0
0.48,15.0	0
0.48,15.1	0
0.48,15.2	0
0.48,15.3	0
0.48,15.4	0
0.48,15.5	0
0.48,15.6	0
0.48,15.7	0
0.48,15.8	0
0.48,15.9	0

0.48,16.0	0
0.48,16.1	0
0.48,16.2	0
0.48,16.3	0
0.48,16.4	0
0.48,16.5	0
0.48,16.6	0
0.48,16.7	0
0.48,16.8	0
0.48,16.9	0
0.48,17.0	0
0.48,17.1	0
0.48,17.2	0
0.48,17.3	0
0.48,17.4	0
0.48,17.5	0
0.48,17.6	0
0.48,17.7	0
0.48,17.8	0
0.48,17.9	0
0.48,18.0	0
0.48,18.1	0
0.48,18.2	0
0.48,18.3	0
0.48,18.4	0
0.48,18.5	0
0.48,18.6	0
0.48,18.7	0
0.48,18.8	0
0.48,18.9	0
0.48,19.0	0
0.48,19.1	0
0.48,19.2	0
0.48,19.3	0
0.48,19.4	0
0.48,19.5	0
0.48,19.6	0
0.48,19.7	0
0.48,19.8	0
0.48,19.9	0
0.48,20.0	0
0.48,20.1	0

0.48,20.2	0
0.48,20.3	0
0.48,20.4	0
0.48,20.5	0
0.48,20.6	0
0.48,20.7	0
0.48,20.8	0
0.48,20.9	0
0.48,21.0	0
0.48,21.1	0
0.48,21.2	0
0.48,21.3	0
0.48,21.4	0
0.48,21.5	0
0.48,21.6	0
0.48,21.7	0
0.48,21.8	0
0.48,21.9	0
0.48,22.0	0
0.48,22.1	0
0.48,22.2	0
0.48,22.3	0
0.48,22.4	0
0.48,22.5	0
0.48,22.6	0
0.48,22.7	0
0.48,22.8	0
0.48,22.9	0
0.48,23.0	0
0.48,23.1	0
0.48,23.2	0
0.48,23.3	0
0.48,23.4	0
0.48,23.5	0
0.48,23.6	0
0.48,23.7	0
0.48,23.8	0
0.48,23.9	0
0.48,24.0	0
0.48,24.1	0
0.48,24.2	0
0.48,24.3	0

0.48,24.4	0
0.48,24.5	0
0.48,24.6	0
0.48,24.7	0
0.48,24.8	0
0.48,24.9	0
0.48,25.0	0
0.48,25.1	0
0.48,25.2	0
0.48,25.3	0
0.48,25.4	0
0.48,25.5	0
0.48,25.6	0
0.48,25.7	0
0.48,25.8	0
0.48,25.9	0
0.48,26.0	0
0.48,26.1	0
0.48,26.2	0
0.48,26.3	0
0.48,26.4	0
0.48,26.5	0
0.48,26.6	0
0.48,26.7	0
0.48,26.8	0
0.48,26.9	0
0.48,27.0	0
0.48,27.1	0
0.48,27.2	0
0.48,27.3	0
0.48,27.4	0
0.48,27.5	0
0.48,27.6	0
0.48,27.7	0
0.48,27.8	0
0.48,27.9	0
0.48,28.0	0
0.48,28.1	0
0.48,28.2	0
0.48,28.3	0
0.48,28.4	0
0.48,28.5	0

0.48,28.6	0
0.48,28.7	0
0.48,28.8	0
0.48,28.9	0
0.48,29.0	0
0.48,29.1	0
0.48,29.2	0
0.48,29.3	0
0.48,29.4	0
0.48,29.5	0
0.48,29.6	0
0.48,29.7	0
0.48,29.8	0
0.48,29.9	0
0.48,30.0	0
0.48,30.1	0
0.48,30.2	0
0.48,30.3	0
0.48,30.4	0
0.48,30.5	0
0.48,30.6	0
0.48,30.7	0
0.48,30.8	0
0.48,30.9	0
0.48,31.0	0
0.48,31.1	0
0.48,31.2	0
0.48,31.3	0
0.48,31.4	0
0.48,31.5	0
0.48,31.6	0
0.48,31.7	0
0.48,31.8	0
0.48,31.9	0
0.48,32.0	0
0.48,32.1	0
0.48,32.2	0
0.48,32.3	0
0.48,32.4	0
0.48,32.5	0
0.48,32.6	0
0.48,32.7	0

0.48,32.8	0
0.48,32.9	0
0.48,33.0	0
0.48,33.1	0
0.48,33.2	0
0.48,33.3	0
0.48,33.4	0
0.48,33.5	0
0.48,33.6	0
0.48,33.7	0
0.48,33.8	0
0.48,33.9	0
0.48,34.0	0
0.48,34.1	0
0.48,34.2	0
0.48,34.3	0
0.48,34.4	0
0.48,34.5	0
0.48,34.6	0
0.48,34.7	0
0.48,34.8	0
0.48,34.9	0
0.48,35.0	0
0.48,35.1	0
0.48,35.2	0
0.48,35.3	0
0.48,35.4	0
0.48,35.5	0
0.48,35.6	0
0.48,35.7	0
0.48,35.8	0
0.48,35.9	0
0.48,36.0	0
0.48,36.1	0
0.48,36.2	0
0.48,36.3	0
0.48,36.4	0
0.48,36.5	0
0.48,36.6	0
0.48,36.7	0
0.48,36.8	0
0.48,36.9	0

0.48,37.0	0
0.48,37.1	0
0.48,37.2	0
0.48,37.3	0
0.48,37.4	0
0.48,37.5	0
0.48,37.6	0
0.48,37.7	0
0.48,37.8	0
0.48,37.9	0
0.48,38.0	0
0.48,38.1	0
0.48,38.2	0
0.48,38.3	0
0.48,38.4	0
0.48,38.5	0
0.48,38.6	0
0.48,38.7	0
0.48,38.8	0
0.48,38.9	0
0.48,39.0	0
0.48,39.1	0
0.48,39.2	0
0.48,39.3	0
0.48,39.4	0
0.48,39.5	0
0.48,39.6	0
0.48,39.7	0
0.48,39.8	0
0.48,39.9	0
0.48,40.0	0
0.48,40.1	0
0.48,40.2	0
0.48,40.3	0
0.48,40.4	0
0.48,40.5	0
0.48,40.6	0
0.48,40.7	0
0.48,40.8	0
0.48,40.9	0
0.48,41.0	0
0.48,41.1	0

0.48,41.2	0
0.48,41.3	0
0.48,41.4	0
0.48,41.5	0
0.48,41.6	0
0.48,41.7	0
0.48,41.8	0
0.48,41.9	0
0.48,42.0	0
0.48,42.1	0
0.48,42.2	0
0.48,42.3	0
0.48,42.4	0
0.48,42.5	0
0.48,42.6	0
0.48,42.7	0
0.48,42.8	0
0.48,42.9	0
0.48,43.0	0
0.48,43.1	0
0.48,43.2	0
0.48,43.3	0
0.48,43.4	0
0.48,43.5	0
0.48,43.6	0
0.48,43.7	0
0.48,43.8	0
0.48,43.9	0
0.48,44.0	0
0.48,44.1	0
0.48,44.2	0
0.48,44.3	0
0.48,44.4	0
0.48,44.5	0
0.48,44.6	0
0.48,44.7	0
0.48,44.8	0
0.48,44.9	0
0.48,45.0	0
0.48,45.1	0
0.48,45.2	0
0.48,45.3	0

0.48,45.4	0
0.48,45.5	0
0.48,45.6	0
0.48,45.7	0
0.48,45.8	0
0.48,45.9	0
0.48,46.0	0
0.48,46.1	0
0.48,46.2	0
0.48,46.3	0
0.48,46.4	0
0.48,46.5	0
0.48,46.6	0
0.48,46.7	0
0.48,46.8	0
0.48,46.9	0
0.48,47.0	0
0.48,47.1	0
0.48,47.2	0
0.48,47.3	0
0.48,47.4	0
0.48,47.5	0
0.48,47.6	0
0.48,47.7	0
0.48,47.8	0
0.48,47.9	0
0.48,48.0	0
0.48,48.1	0
0.48,48.2	0
0.48,48.3	0
0.48,48.4	0
0.48,48.5	0
0.48,48.6	0
0.48,48.7	0
0.48,48.8	0
0.48,48.9	0
0.48,49.0	0
0.48,49.1	0
0.48,49.2	0
0.48,49.3	0
0.48,49.4	0
0.48,49.5	0

0.48,49.6	0
0.48,49.7	0
0.48,49.8	0
0.48,49.9	0
0.48,50.0	0
0.48,50.1	0
0.48,50.2	0
0.48,50.3	0
0.48,50.4	0
0.48,50.5	0
0.48,50.6	0
0.48,50.7	0
0.48,50.8	0
0.48,50.9	0
0.48,51.0	0
0.48,51.1	0
0.48,51.2	0
0.48,51.3	0
0.48,51.4	0
0.48,51.5	0
0.48,51.6	0
0.48,51.7	0
0.48,51.8	0
0.48,51.9	0
0.48,52.0	0
0.48,52.1	0
0.48,52.2	0
0.48,52.3	0
0.48,52.4	0
0.48,52.5	0
0.48,52.6	0
0.48,52.7	0
0.48,52.8	0
0.48,52.9	0
0.48,53.0	0
0.48,53.1	0
0.48,53.2	0
0.48,53.3	0
0.48,53.4	0
0.48,53.5	0
0.48,53.6	0
0.48,53.7	0

0.48,53.8	0
0.48,53.9	0
0.48,54.0	0
0.48,54.1	0
0.48,54.2	0
0.48,54.3	0
0.48,54.4	0
0.48,54.5	0
0.48,54.6	0
0.48,54.7	0
0.48,54.8	0
0.48,54.9	0
0.48,55.0	0
0.48,55.1	0
0.48,55.2	0
0.48,55.3	0
0.48,55.4	0
0.48,55.5	0
0.48,55.6	0
0.48,55.7	0
0.48,55.8	0
0.48,55.9	0
0.48,56.0	0
0.48,56.1	0
0.48,56.2	0
0.48,56.3	0
0.48,56.4	0
0.48,56.5	0
0.48,56.6	0
0.48,56.7	0
0.48,56.8	0
0.48,56.9	0
0.48,57.0	0
0.48,57.1	0
0.48,57.2	0
0.48,57.3	0
0.48,57.4	0
0.48,57.5	0
0.48,57.6	0
0.48,57.7	0
0.48,57.8	0
0.48,57.9	0

0.48,58.0	0
0.48,58.1	0
0.48,58.2	0
0.48,58.3	0
0.48,58.4	0
0.48,58.5	0
0.48,58.6	0
0.48,58.7	0
0.48,58.8	0
0.48,58.9	0
0.48,59.0	0
0.48,59.1	0
0.48,59.2	0
0.48,59.3	0
0.48,59.4	0
0.48,59.5	0
0.48,59.6	0
0.48,59.7	0
0.48,59.8	0
0.48,59.9	0
0.48,60.0	0
0.48,60.1	0
0.48,60.2	0
0.48,60.3	0
0.48,60.4	0
0.48,60.5	0
0.48,60.6	0
0.48,60.7	0
0.48,60.8	0
0.48,60.9	0
0.48,61.0	0
0.48,61.1	0
0.48,61.2	0
0.48,61.3	0
0.48,61.4	0
0.48,61.5	0
0.48,61.6	0
0.48,61.7	0
0.48,61.8	0
0.48,61.9	0
0.48,62.0	0
0.48,62.1	0

0.48,62.2	0
0.48,62.3	0
0.48,62.4	0
0.48,62.5	0
0.48,62.6	0
0.48,62.7	0
0.48,62.8	0
0.48,62.9	0
0.48,63.0	0
0.48,63.1	0
0.48,63.2	0
0.48,63.3	0
0.48,63.4	0
0.48,63.5	0
0.48,63.6	0
0.48,63.7	0
0.48,63.8	0
0.48,63.9	0
0.48,64.0	0
0.48,64.1	0
0.48,64.2	0
0.48,64.3	0
0.48,64.4	0
0.48,64.5	0
0.48,64.6	0
0.48,64.7	0
0.48,64.8	0
0.48,64.9	0
0.48,65.0	0
0.48,65.1	0
0.48,65.2	0
0.48,65.3	0
0.48,65.4	0
0.48,65.5	0
0.48,65.6	0
0.48,65.7	0
0.48,65.8	0
0.48,65.9	0
0.48,66.0	0
0.48,66.1	0
0.48,66.2	0
0.48,66.3	0

0.48,66.4	0
0.48,66.5	0
0.48,66.6	0
0.48,66.7	0
0.48,66.8	0
0.48,66.9	0
0.48,67.0	0
0.48,67.1	0
0.48,67.2	0
0.48,67.3	0
0.48,67.4	0
0.48,67.5	0
0.48,67.6	0
0.48,67.7	0
0.48,67.8	0
0.48,67.9	0
0.48,68.0	0
0.48,68.1	0
0.48,68.2	0
0.48,68.3	0
0.48,68.4	0
0.48,68.5	0
0.48,68.6	0
0.48,68.7	0
0.48,68.8	0
0.48,68.9	0
0.48,69.0	0
0.48,69.1	0
0.48,69.2	0
0.48,69.3	0
0.48,69.4	0
0.48,69.5	0
0.48,69.6	0
0.48,69.7	0
0.48,69.8	0
0.48,69.9	0
0.48,70.0	0
0.48,70.1	0
0.48,70.2	0
0.48,70.3	0
0.48,70.4	0
0.48,70.5	0

0.48,70.6	0
0.48,70.7	0
0.48,70.8	0
0.48,70.9	0
0.48,71.0	0
0.48,71.1	0
0.48,71.2	0
0.48,71.3	0
0.48,71.4	0
0.48,71.5	0
0.48,71.6	0
0.48,71.7	0
0.48,71.8	0
0.48,71.9	0
0.48,72.0	0
0.48,72.1	0
0.48,72.2	0
0.48,72.3	0
0.48,72.4	0
0.48,72.5	0
0.48,72.6	0
0.48,72.7	0
0.48,72.8	0
0.48,72.9	0
0.48,73.0	0
0.48,73.1	0
0.48,73.2	0
0.48,73.3	0
0.48,73.4	0
0.48,73.5	0
0.48,73.6	0
0.48,73.7	0
0.48,73.8	0
0.48,73.9	0
0.48,74.0	0
0.48,74.1	0
0.48,74.2	0
0.48,74.3	0
0.48,74.4	0
0.48,74.5	0
0.48,74.6	0
0.48,74.7	0

0.48,74.8	0
0.48,74.9	0
0.48,75.0	0
0.48,75.1	0
0.48,75.2	0
0.48,75.3	0
0.48,75.4	0
0.48,75.5	0
0.48,75.6	0
0.48,75.7	0
0.48,75.8	0
0.48,75.9	0
0.48,76.0	0
0.48,76.1	0
0.48,76.2	0
0.48,76.3	0
0.48,76.4	0
0.48,76.5	0
0.48,76.6	0
0.48,76.7	0
0.48,76.8	0
0.48,76.9	0
0.48,77.0	0
0.48,77.1	0
0.48,77.2	0
0.48,77.3	0
0.48,77.4	0
0.48,77.5	0
0.48,77.6	0
0.48,77.7	0
0.48,77.8	0
0.48,77.9	0
0.48,78.0	0
0.48,78.1	0
0.48,78.2	0
0.48,78.3	0
0.48,78.4	0
0.48,78.5	0
0.48,78.6	0
0.48,78.7	0
0.48,78.8	0
0.48,78.9	0

0.48,79.0	0
0.48,79.1	0
0.48,79.2	0
0.48,79.3	0
0.48,79.4	0
0.48,79.5	0
0.48,79.6	0
0.48,79.7	0
0.48,79.8	0
0.48,79.9	0
0.48,80.0	0
0.48,80.1	0
0.48,80.2	0
0.48,80.3	0
0.48,80.4	0
0.48,80.5	0
0.48,80.6	0
0.48,80.7	0
0.48,80.8	0
0.48,80.9	0
0.48,81.0	0
0.48,81.1	0
0.48,81.2	0
0.48,81.3	0
0.48,81.4	0
0.48,81.5	0
0.48,81.6	0
0.48,81.7	0
0.48,81.8	0
0.48,81.9	0
0.48,82.0	0
0.48,82.1	0
0.48,82.2	0
0.48,82.3	0
0.48,82.4	0
0.48,82.5	0
0.48,82.6	0
0.48,82.7	0
0.48,82.8	0
0.48,82.9	0
0.48,83.0	0
0.48,83.1	0

0.48,83.2	0
0.48,83.3	0
0.48,83.4	0
0.48,83.5	0
0.48,83.6	0
0.48,83.7	0
0.48,83.8	0
0.48,83.9	0
0.48,84.0	0
0.48,84.1	0
0.48,84.2	0
0.48,84.3	0
0.48,84.4	0
0.48,84.5	0
0.48,84.6	0
0.48,84.7	0
0.48,84.8	0
0.48,84.9	0
0.48,85.0	0
0.48,85.1	0
0.48,85.2	0
0.48,85.3	0
0.48,85.4	0
0.48,85.5	0
0.48,85.6	0
0.48,85.7	0
0.48,85.8	0
0.48,85.9	0
0.48,86.0	0
0.48,86.1	0
0.48,86.2	0
0.48,86.3	0
0.48,86.4	0
0.48,86.5	0
0.48,86.6	0
0.48,86.7	0
0.48,86.8	0
0.48,86.9	0
0.48,87.0	0
0.48,87.1	0
0.48,87.2	0
0.48,87.3	0

0.48,87.4	0
0.48,87.5	0
0.48,87.6	0
0.48,87.7	0
0.48,87.8	0
0.48,87.9	0
0.48,88.0	0
0.48,88.1	0
0.48,88.2	0
0.48,88.3	0
0.48,88.4	0
0.48,88.5	0
0.48,88.6	0
0.48,88.7	0
0.48,88.8	0
0.48,88.9	0
0.48,89.0	0
0.48,89.1	0
0.48,89.2	0
0.48,89.3	0
0.48,89.4	0
0.48,89.5	0
0.48,89.6	0
0.48,89.7	0
0.48,89.8	0
0.48,89.9	0
0.48,90.0	0
0.48,90.1	0
0.48,90.2	0
0.48,90.3	0
0.48,90.4	0
0.48,90.5	0
0.48,90.6	0
0.48,90.7	0
0.48,90.8	0
0.48,90.9	0
0.48,91.0	0
0.48,91.1	0
0.48,91.2	0
0.48,91.3	0
0.48,91.4	0
0.48,91.5	0

0.48,91.6	0
0.48,91.7	0
0.48,91.8	0
0.48,91.9	0
0.48,92.0	0
0.48,92.1	0
0.48,92.2	0
0.48,92.3	0
0.48,92.4	0
0.48,92.5	0
0.48,92.6	0
0.48,92.7	0
0.48,92.8	0
0.48,92.9	0
0.48,93.0	0
0.48,93.1	0
0.48,93.2	0
0.48,93.3	0
0.48,93.4	0
0.48,93.5	0
0.48,93.6	0
0.48,93.7	0
0.48,93.8	0
0.48,93.9	0
0.48,94.0	0
0.48,94.1	0
0.48,94.2	0
0.48,94.3	0
0.48,94.4	0
0.48,94.5	0
0.48,94.6	0
0.48,94.7	0
0.48,94.8	0
0.48,94.9	0
0.48,95.0	0
0.48,95.1	0
0.48,95.2	0
0.48,95.3	0
0.48,95.4	0
0.48,95.5	0
0.48,95.6	0
0.48,95.7	0

0.48,95.8	0
0.48,95.9	0
0.48,96.0	0
0.48,96.1	0
0.48,96.2	0
0.48,96.3	0
0.48,96.4	0
0.48,96.5	0
0.48,96.6	0
0.48,96.7	0
0.48,96.8	0
0.48,96.9	0
0.48,97.0	0
0.48,97.1	0
0.48,97.2	0
0.48,97.3	0
0.48,97.4	0
0.48,97.5	0
0.48,97.6	0
0.48,97.7	0
0.48,97.8	0
0.48,97.9	0
0.48,98.0	0
0.48,98.1	0
0.48,98.2	0
0.48,98.3	0
0.48,98.4	0
0.48,98.5	0
0.48,98.6	0
0.48,98.7	0
0.48,98.8	0
0.48,98.9	0
0.48,99.0	0
0.48,99.1	0
0.48,99.2	0
0.48,99.3	0
0.48,99.4	0
0.48,99.5	0
0.48,99.6	0
0.48,99.7	0
0.48,99.8	0
0.48,99.9	0

0.48,100.0	0
0.49,0.0	0
0.49,0.1	0
0.49,0.2	0
0.49,0.3	0
0.49,0.4	0
0.49,0.5	0
0.49,0.6	0
0.49,0.7	0
0.49,0.8	0
0.49,0.9	0
0.49,1.0	0
0.49,1.1	0
0.49,1.2	0
0.49,1.3	0
0.49,1.4	0
0.49,1.5	0
0.49,1.6	0
0.49,1.7	0
0.49,1.8	0
0.49,1.9	0
0.49,2.0	0
0.49,2.1	0
0.49,2.2	0
0.49,2.3	0
0.49,2.4	0
0.49,2.5	0
0.49,2.6	0
0.49,2.7	0
0.49,2.8	0
0.49,2.9	0
0.49,3.0	0
0.49,3.1	0
0.49,3.2	0
0.49,3.3	0
0.49,3.4	0
0.49,3.5	0
0.49,3.6	0
0.49,3.7	0
0.49,3.8	0
0.49,3.9	0
0.49,4.0	0

0.49,4.1	0
0.49,4.2	0
0.49,4.3	0
0.49,4.4	0
0.49,4.5	0
0.49,4.6	0
0.49,4.7	0
0.49,4.8	0
0.49,4.9	0
0.49,5.0	0
0.49,5.1	0
0.49,5.2	0
0.49,5.3	0
0.49,5.4	0
0.49,5.5	0
0.49,5.6	0
0.49,5.7	0
0.49,5.8	0
0.49,5.9	0
0.49,6.0	0
0.49,6.1	0
0.49,6.2	0
0.49,6.3	0
0.49,6.4	0
0.49,6.5	0
0.49,6.6	0
0.49,6.7	0
0.49,6.8	0
0.49,6.9	0
0.49,7.0	0
0.49,7.1	0
0.49,7.2	0
0.49,7.3	0
0.49,7.4	0
0.49,7.5	0
0.49,7.6	0
0.49,7.7	0
0.49,7.8	0
0.49,7.9	0
0.49,8.0	0
0.49,8.1	0
0.49,8.2	0

0.49,8.3	0
0.49,8.4	0
0.49,8.5	0
0.49,8.6	0
0.49,8.7	0
0.49,8.8	0
0.49,8.9	0
0.49,9.0	0
0.49,9.1	0
0.49,9.2	0
0.49,9.3	0
0.49,9.4	0
0.49,9.5	0
0.49,9.6	0
0.49,9.7	0
0.49,9.8	0
0.49,9.9	0
0.49,10.0	0
0.49,10.1	0
0.49,10.2	0
0.49,10.3	0
0.49,10.4	0
0.49,10.5	0
0.49,10.6	0
0.49,10.7	0
0.49,10.8	0
0.49,10.9	0
0.49,11.0	0
0.49,11.1	0
0.49,11.2	0
0.49,11.3	0
0.49,11.4	0
0.49,11.5	0
0.49,11.6	0
0.49,11.7	0
0.49,11.8	0
0.49,11.9	0
0.49,12.0	0
0.49,12.1	0
0.49,12.2	0
0.49,12.3	0
0.49,12.4	0

0.49,12.5	0
0.49,12.6	0
0.49,12.7	0
0.49,12.8	0
0.49,12.9	0
0.49,13.0	0
0.49,13.1	0
0.49,13.2	0
0.49,13.3	0
0.49,13.4	0
0.49,13.5	0
0.49,13.6	0
0.49,13.7	0
0.49,13.8	0
0.49,13.9	0
0.49,14.0	0
0.49,14.1	0
0.49,14.2	0
0.49,14.3	0
0.49,14.4	0
0.49,14.5	0
0.49,14.6	0
0.49,14.7	0
0.49,14.8	0
0.49,14.9	0
0.49,15.0	0
0.49,15.1	0
0.49,15.2	0
0.49,15.3	0
0.49,15.4	0
0.49,15.5	0
0.49,15.6	0
0.49,15.7	0
0.49,15.8	0
0.49,15.9	0
0.49,16.0	0
0.49,16.1	0
0.49,16.2	0
0.49,16.3	0
0.49,16.4	0
0.49,16.5	0
0.49,16.6	0

0.49,16.7	0
0.49,16.8	0
0.49,16.9	0
0.49,17.0	0
0.49,17.1	0
0.49,17.2	0
0.49,17.3	0
0.49,17.4	0
0.49,17.5	0
0.49,17.6	0
0.49,17.7	0
0.49,17.8	0
0.49,17.9	0
0.49,18.0	0
0.49,18.1	0
0.49,18.2	0
0.49,18.3	0
0.49,18.4	0
0.49,18.5	0
0.49,18.6	0
0.49,18.7	0
0.49,18.8	0
0.49,18.9	0
0.49,19.0	0
0.49,19.1	0
0.49,19.2	0
0.49,19.3	0
0.49,19.4	0
0.49,19.5	0
0.49,19.6	0
0.49,19.7	0
0.49,19.8	0
0.49,19.9	0
0.49,20.0	0
0.49,20.1	0
0.49,20.2	0
0.49,20.3	0
0.49,20.4	0
0.49,20.5	0
0.49,20.6	0
0.49,20.7	0
0.49,20.8	0

0.49,20.9	0
0.49,21.0	0
0.49,21.1	0
0.49,21.2	0
0.49,21.3	0
0.49,21.4	0
0.49,21.5	0
0.49,21.6	0
0.49,21.7	0
0.49,21.8	0
0.49,21.9	0
0.49,22.0	0
0.49,22.1	0
0.49,22.2	0
0.49,22.3	0
0.49,22.4	0
0.49,22.5	0
0.49,22.6	0
0.49,22.7	0
0.49,22.8	0
0.49,22.9	0
0.49,23.0	0
0.49,23.1	0
0.49,23.2	0
0.49,23.3	0
0.49,23.4	0
0.49,23.5	0
0.49,23.6	0
0.49,23.7	0
0.49,23.8	0
0.49,23.9	0
0.49,24.0	0
0.49,24.1	0
0.49,24.2	0
0.49,24.3	0
0.49,24.4	0
0.49,24.5	0
0.49,24.6	0
0.49,24.7	0
0.49,24.8	0
0.49,24.9	0
0.49,25.0	0

0.49,25.1	0
0.49,25.2	0
0.49,25.3	0
0.49,25.4	0
0.49,25.5	0
0.49,25.6	0
0.49,25.7	0
0.49,25.8	0
0.49,25.9	0
0.49,26.0	0
0.49,26.1	0
0.49,26.2	0
0.49,26.3	0
0.49,26.4	0
0.49,26.5	0
0.49,26.6	0
0.49,26.7	0
0.49,26.8	0
0.49,26.9	0
0.49,27.0	0
0.49,27.1	0
0.49,27.2	0
0.49,27.3	0
0.49,27.4	0
0.49,27.5	0
0.49,27.6	0
0.49,27.7	0
0.49,27.8	0
0.49,27.9	0
0.49,28.0	0
0.49,28.1	0
0.49,28.2	0
0.49,28.3	0
0.49,28.4	0
0.49,28.5	0
0.49,28.6	0
0.49,28.7	0
0.49,28.8	0
0.49,28.9	0
0.49,29.0	0
0.49,29.1	0
0.49,29.2	0

0.49,29.3	0
0.49,29.4	0
0.49,29.5	0
0.49,29.6	0
0.49,29.7	0
0.49,29.8	0
0.49,29.9	0
0.49,30.0	0
0.49,30.1	0
0.49,30.2	0
0.49,30.3	0
0.49,30.4	0
0.49,30.5	0
0.49,30.6	0
0.49,30.7	0
0.49,30.8	0
0.49,30.9	0
0.49,31.0	0
0.49,31.1	0
0.49,31.2	0
0.49,31.3	0
0.49,31.4	0
0.49,31.5	0
0.49,31.6	0
0.49,31.7	0
0.49,31.8	0
0.49,31.9	0
0.49,32.0	0
0.49,32.1	0
0.49,32.2	0
0.49,32.3	0
0.49,32.4	0
0.49,32.5	0
0.49,32.6	0
0.49,32.7	0
0.49,32.8	0
0.49,32.9	0
0.49,33.0	0
0.49,33.1	0
0.49,33.2	0
0.49,33.3	0
0.49,33.4	0

0.49,33.5	0
0.49,33.6	0
0.49,33.7	0
0.49,33.8	0
0.49,33.9	0
0.49,34.0	0
0.49,34.1	0
0.49,34.2	0
0.49,34.3	0
0.49,34.4	0
0.49,34.5	0
0.49,34.6	0
0.49,34.7	0
0.49,34.8	0
0.49,34.9	0
0.49,35.0	0
0.49,35.1	0
0.49,35.2	0
0.49,35.3	0
0.49,35.4	0
0.49,35.5	0
0.49,35.6	0
0.49,35.7	0
0.49,35.8	0
0.49,35.9	0
0.49,36.0	0
0.49,36.1	0
0.49,36.2	0
0.49,36.3	0
0.49,36.4	0
0.49,36.5	0
0.49,36.6	0
0.49,36.7	0
0.49,36.8	0
0.49,36.9	0
0.49,37.0	0
0.49,37.1	0
0.49,37.2	0
0.49,37.3	0
0.49,37.4	0
0.49,37.5	0
0.49,37.6	0

0.49,37.7	0
0.49,37.8	0
0.49,37.9	0
0.49,38.0	0
0.49,38.1	0
0.49,38.2	0
0.49,38.3	0
0.49,38.4	0
0.49,38.5	0
0.49,38.6	0
0.49,38.7	0
0.49,38.8	0
0.49,38.9	0
0.49,39.0	0
0.49,39.1	0
0.49,39.2	0
0.49,39.3	0
0.49,39.4	0
0.49,39.5	0
0.49,39.6	0
0.49,39.7	0
0.49,39.8	0
0.49,39.9	0
0.49,40.0	0
0.49,40.1	0
0.49,40.2	0
0.49,40.3	0
0.49,40.4	0
0.49,40.5	0
0.49,40.6	0
0.49,40.7	0
0.49,40.8	0
0.49,40.9	0
0.49,41.0	0
0.49,41.1	0
0.49,41.2	0
0.49,41.3	0
0.49,41.4	0
0.49,41.5	0
0.49,41.6	0
0.49,41.7	0
0.49,41.8	0

0.49,41.9	0
0.49,42.0	0
0.49,42.1	0
0.49,42.2	0
0.49,42.3	0
0.49,42.4	0
0.49,42.5	0
0.49,42.6	0
0.49,42.7	0
0.49,42.8	0
0.49,42.9	0
0.49,43.0	0
0.49,43.1	0
0.49,43.2	0
0.49,43.3	0
0.49,43.4	0
0.49,43.5	0
0.49,43.6	0
0.49,43.7	0
0.49,43.8	0
0.49,43.9	0
0.49,44.0	0
0.49,44.1	0
0.49,44.2	0
0.49,44.3	0
0.49,44.4	0
0.49,44.5	0
0.49,44.6	0
0.49,44.7	0
0.49,44.8	0
0.49,44.9	0
0.49,45.0	0
0.49,45.1	0
0.49,45.2	0
0.49,45.3	0
0.49,45.4	0
0.49,45.5	0
0.49,45.6	0
0.49,45.7	0
0.49,45.8	0
0.49,45.9	0
0.49,46.0	0

0.49,46.1	0
0.49,46.2	0
0.49,46.3	0
0.49,46.4	0
0.49,46.5	0
0.49,46.6	0
0.49,46.7	0
0.49,46.8	0
0.49,46.9	0
0.49,47.0	0
0.49,47.1	0
0.49,47.2	0
0.49,47.3	0
0.49,47.4	0
0.49,47.5	0
0.49,47.6	0
0.49,47.7	0
0.49,47.8	0
0.49,47.9	0
0.49,48.0	0
0.49,48.1	0
0.49,48.2	0
0.49,48.3	0
0.49,48.4	0
0.49,48.5	0
0.49,48.6	0
0.49,48.7	0
0.49,48.8	0
0.49,48.9	0
0.49,49.0	0
0.49,49.1	0
0.49,49.2	0
0.49,49.3	0
0.49,49.4	0
0.49,49.5	0
0.49,49.6	0
0.49,49.7	0
0.49,49.8	0
0.49,49.9	0
0.49,50.0	0
0.49,50.1	0
0.49,50.2	0

0.49,50.3	0
0.49,50.4	0
0.49,50.5	0
0.49,50.6	0
0.49,50.7	0
0.49,50.8	0
0.49,50.9	0
0.49,51.0	0
0.49,51.1	0
0.49,51.2	0
0.49,51.3	0
0.49,51.4	0
0.49,51.5	0
0.49,51.6	0
0.49,51.7	0
0.49,51.8	0
0.49,51.9	0
0.49,52.0	0
0.49,52.1	0
0.49,52.2	0
0.49,52.3	0
0.49,52.4	0
0.49,52.5	0
0.49,52.6	0
0.49,52.7	0
0.49,52.8	0
0.49,52.9	0
0.49,53.0	0
0.49,53.1	0
0.49,53.2	0
0.49,53.3	0
0.49,53.4	0
0.49,53.5	0
0.49,53.6	0
0.49,53.7	0
0.49,53.8	0
0.49,53.9	0
0.49,54.0	0
0.49,54.1	0
0.49,54.2	0
0.49,54.3	0
0.49,54.4	0

0.49,54.5	0
0.49,54.6	0
0.49,54.7	0
0.49,54.8	0
0.49,54.9	0
0.49,55.0	0
0.49,55.1	0
0.49,55.2	0
0.49,55.3	0
0.49,55.4	0
0.49,55.5	0
0.49,55.6	0
0.49,55.7	0
0.49,55.8	0
0.49,55.9	0
0.49,56.0	0
0.49,56.1	0
0.49,56.2	0
0.49,56.3	0
0.49,56.4	0
0.49,56.5	0
0.49,56.6	0
0.49,56.7	0
0.49,56.8	0
0.49,56.9	0
0.49,57.0	0
0.49,57.1	0
0.49,57.2	0
0.49,57.3	0
0.49,57.4	0
0.49,57.5	0
0.49,57.6	0
0.49,57.7	0
0.49,57.8	0
0.49,57.9	0
0.49,58.0	0
0.49,58.1	0
0.49,58.2	0
0.49,58.3	0
0.49,58.4	0
0.49,58.5	0
0.49,58.6	0

0.49,58.7	0
0.49,58.8	0
0.49,58.9	0
0.49,59.0	0
0.49,59.1	0
0.49,59.2	0
0.49,59.3	0
0.49,59.4	0
0.49,59.5	0
0.49,59.6	0
0.49,59.7	0
0.49,59.8	0
0.49,59.9	0
0.49,60.0	0
0.49,60.1	0
0.49,60.2	0
0.49,60.3	0
0.49,60.4	0
0.49,60.5	0
0.49,60.6	0
0.49,60.7	0
0.49,60.8	0
0.49,60.9	0
0.49,61.0	0
0.49,61.1	0
0.49,61.2	0
0.49,61.3	0
0.49,61.4	0
0.49,61.5	0
0.49,61.6	0
0.49,61.7	0
0.49,61.8	0
0.49,61.9	0
0.49,62.0	0
0.49,62.1	0
0.49,62.2	0
0.49,62.3	0
0.49,62.4	0
0.49,62.5	0
0.49,62.6	0
0.49,62.7	0
0.49,62.8	0

0.49,62.9	0
0.49,63.0	0
0.49,63.1	0
0.49,63.2	0
0.49,63.3	0
0.49,63.4	0
0.49,63.5	0
0.49,63.6	0
0.49,63.7	0
0.49,63.8	0
0.49,63.9	0
0.49,64.0	0
0.49,64.1	0
0.49,64.2	0
0.49,64.3	0
0.49,64.4	0
0.49,64.5	0
0.49,64.6	0
0.49,64.7	0
0.49,64.8	0
0.49,64.9	0
0.49,65.0	0
0.49,65.1	0
0.49,65.2	0
0.49,65.3	0
0.49,65.4	0
0.49,65.5	0
0.49,65.6	0
0.49,65.7	0
0.49,65.8	0
0.49,65.9	0
0.49,66.0	0
0.49,66.1	0
0.49,66.2	0
0.49,66.3	0
0.49,66.4	0
0.49,66.5	0
0.49,66.6	0
0.49,66.7	0
0.49,66.8	0
0.49,66.9	0
0.49,67.0	0

0.49,67.1	0
0.49,67.2	0
0.49,67.3	0
0.49,67.4	0
0.49,67.5	0
0.49,67.6	0
0.49,67.7	0
0.49,67.8	0
0.49,67.9	0
0.49,68.0	0
0.49,68.1	0
0.49,68.2	0
0.49,68.3	0
0.49,68.4	0
0.49,68.5	0
0.49,68.6	0
0.49,68.7	0
0.49,68.8	0
0.49,68.9	0
0.49,69.0	0
0.49,69.1	0
0.49,69.2	0
0.49,69.3	0
0.49,69.4	0
0.49,69.5	0
0.49,69.6	0
0.49,69.7	0
0.49,69.8	0
0.49,69.9	0
0.49,70.0	0
0.49,70.1	0
0.49,70.2	0
0.49,70.3	0
0.49,70.4	0
0.49,70.5	0
0.49,70.6	0
0.49,70.7	0
0.49,70.8	0
0.49,70.9	0
0.49,71.0	0
0.49,71.1	0
0.49,71.2	0

0.49,71.3	0
0.49,71.4	0
0.49,71.5	0
0.49,71.6	0
0.49,71.7	0
0.49,71.8	0
0.49,71.9	0
0.49,72.0	0
0.49,72.1	0
0.49,72.2	0
0.49,72.3	0
0.49,72.4	0
0.49,72.5	0
0.49,72.6	0
0.49,72.7	0
0.49,72.8	0
0.49,72.9	0
0.49,73.0	0
0.49,73.1	0
0.49,73.2	0
0.49,73.3	0
0.49,73.4	0
0.49,73.5	0
0.49,73.6	0
0.49,73.7	0
0.49,73.8	0
0.49,73.9	0
0.49,74.0	0
0.49,74.1	0
0.49,74.2	0
0.49,74.3	0
0.49,74.4	0
0.49,74.5	0
0.49,74.6	0
0.49,74.7	0
0.49,74.8	0
0.49,74.9	0
0.49,75.0	0
0.49,75.1	0
0.49,75.2	0
0.49,75.3	0
0.49,75.4	0

0.49,75.5	0
0.49,75.6	0
0.49,75.7	0
0.49,75.8	0
0.49,75.9	0
0.49,76.0	0
0.49,76.1	0
0.49,76.2	0
0.49,76.3	0
0.49,76.4	0
0.49,76.5	0
0.49,76.6	0
0.49,76.7	0
0.49,76.8	0
0.49,76.9	0
0.49,77.0	0
0.49,77.1	0
0.49,77.2	0
0.49,77.3	0
0.49,77.4	0
0.49,77.5	0
0.49,77.6	0
0.49,77.7	0
0.49,77.8	0
0.49,77.9	0
0.49,78.0	0
0.49,78.1	0
0.49,78.2	0
0.49,78.3	0
0.49,78.4	0
0.49,78.5	0
0.49,78.6	0
0.49,78.7	0
0.49,78.8	0
0.49,78.9	0
0.49,79.0	0
0.49,79.1	0
0.49,79.2	0
0.49,79.3	0
0.49,79.4	0
0.49,79.5	0
0.49,79.6	0

0.49,79.7	0
0.49,79.8	0
0.49,79.9	0
0.49,80.0	0
0.49,80.1	0
0.49,80.2	0
0.49,80.3	0
0.49,80.4	0
0.49,80.5	0
0.49,80.6	0
0.49,80.7	0
0.49,80.8	0
0.49,80.9	0
0.49,81.0	0
0.49,81.1	0
0.49,81.2	0
0.49,81.3	0
0.49,81.4	0
0.49,81.5	0
0.49,81.6	0
0.49,81.7	0
0.49,81.8	0
0.49,81.9	0
0.49,82.0	0
0.49,82.1	0
0.49,82.2	0
0.49,82.3	0
0.49,82.4	0
0.49,82.5	0
0.49,82.6	0
0.49,82.7	0
0.49,82.8	0
0.49,82.9	0
0.49,83.0	0
0.49,83.1	0
0.49,83.2	0
0.49,83.3	0
0.49,83.4	0
0.49,83.5	0
0.49,83.6	0
0.49,83.7	0
0.49,83.8	0

0.49,83.9	0
0.49,84.0	0
0.49,84.1	0
0.49,84.2	0
0.49,84.3	0
0.49,84.4	0
0.49,84.5	0
0.49,84.6	0
0.49,84.7	0
0.49,84.8	0
0.49,84.9	0
0.49,85.0	0
0.49,85.1	0
0.49,85.2	0
0.49,85.3	0
0.49,85.4	0
0.49,85.5	0
0.49,85.6	0
0.49,85.7	0
0.49,85.8	0
0.49,85.9	0
0.49,86.0	0
0.49,86.1	0
0.49,86.2	0
0.49,86.3	0
0.49,86.4	0
0.49,86.5	0
0.49,86.6	0
0.49,86.7	0
0.49,86.8	0
0.49,86.9	0
0.49,87.0	0
0.49,87.1	0
0.49,87.2	0
0.49,87.3	0
0.49,87.4	0
0.49,87.5	0
0.49,87.6	0
0.49,87.7	0
0.49,87.8	0
0.49,87.9	0
0.49,88.0	0

0.49,88.1	0
0.49,88.2	0
0.49,88.3	0
0.49,88.4	0
0.49,88.5	0
0.49,88.6	0
0.49,88.7	0
0.49,88.8	0
0.49,88.9	0
0.49,89.0	0
0.49,89.1	0
0.49,89.2	0
0.49,89.3	0
0.49,89.4	0
0.49,89.5	0
0.49,89.6	0
0.49,89.7	0
0.49,89.8	0
0.49,89.9	0
0.49,90.0	0
0.49,90.1	0
0.49,90.2	0
0.49,90.3	0
0.49,90.4	0
0.49,90.5	0
0.49,90.6	0
0.49,90.7	0
0.49,90.8	0
0.49,90.9	0
0.49,91.0	0
0.49,91.1	0
0.49,91.2	0
0.49,91.3	0
0.49,91.4	0
0.49,91.5	0
0.49,91.6	0
0.49,91.7	0
0.49,91.8	0
0.49,91.9	0
0.49,92.0	0
0.49,92.1	0
0.49,92.2	0

0.49,92.3	0
0.49,92.4	0
0.49,92.5	0
0.49,92.6	0
0.49,92.7	0
0.49,92.8	0
0.49,92.9	0
0.49,93.0	0
0.49,93.1	0
0.49,93.2	0
0.49,93.3	0
0.49,93.4	0
0.49,93.5	0
0.49,93.6	0
0.49,93.7	0
0.49,93.8	0
0.49,93.9	0
0.49,94.0	0
0.49,94.1	0
0.49,94.2	0
0.49,94.3	0
0.49,94.4	0
0.49,94.5	0
0.49,94.6	0
0.49,94.7	0
0.49,94.8	0
0.49,94.9	0
0.49,95.0	0
0.49,95.1	0
0.49,95.2	0
0.49,95.3	0
0.49,95.4	0
0.49,95.5	0
0.49,95.6	0
0.49,95.7	0
0.49,95.8	0
0.49,95.9	0
0.49,96.0	0
0.49,96.1	0
0.49,96.2	0
0.49,96.3	0
0.49,96.4	0

0.49,96.5	0
0.49,96.6	0
0.49,96.7	0
0.49,96.8	0
0.49,96.9	0
0.49,97.0	0
0.49,97.1	0
0.49,97.2	0
0.49,97.3	0
0.49,97.4	0
0.49,97.5	0
0.49,97.6	0
0.49,97.7	0
0.49,97.8	0
0.49,97.9	0
0.49,98.0	0
0.49,98.1	0
0.49,98.2	0
0.49,98.3	0
0.49,98.4	0
0.49,98.5	0
0.49,98.6	0
0.49,98.7	0
0.49,98.8	0
0.49,98.9	0
0.49,99.0	0
0.49,99.1	0
0.49,99.2	0
0.49,99.3	0
0.49,99.4	0
0.49,99.5	0
0.49,99.6	0
0.49,99.7	0
0.49,99.8	0
0.49,99.9	0
0.49,100.0	0
0.50,0.0	0
0.50,0.1	0
0.50,0.2	0
0.50,0.3	0
0.50,0.4	0
0.50,0.5	0

0.50,0.6	0
0.50,0.7	0
0.50,0.8	0
0.50,0.9	0
0.50,1.0	0
0.50,1.1	0
0.50,1.2	0
0.50,1.3	0
0.50,1.4	0
0.50,1.5	0
0.50,1.6	0
0.50,1.7	0
0.50,1.8	0
0.50,1.9	0
0.50,2.0	0
0.50,2.1	0
0.50,2.2	0
0.50,2.3	0
0.50,2.4	0
0.50,2.5	0
0.50,2.6	0
0.50,2.7	0
0.50,2.8	0
0.50,2.9	0
0.50,3.0	0
0.50,3.1	0
0.50,3.2	0
0.50,3.3	0
0.50,3.4	0
0.50,3.5	0
0.50,3.6	0
0.50,3.7	0
0.50,3.8	0
0.50,3.9	0
0.50,4.0	0
0.50,4.1	0
0.50,4.2	0
0.50,4.3	0
0.50,4.4	0
0.50,4.5	0
0.50,4.6	0
0.50,4.7	0

0.50,4.8	0
0.50,4.9	0
0.50,5.0	0
0.50,5.1	0
0.50,5.2	0
0.50,5.3	0
0.50,5.4	0
0.50,5.5	0
0.50,5.6	0
0.50,5.7	0
0.50,5.8	0
0.50,5.9	0
0.50,6.0	0
0.50,6.1	0
0.50,6.2	0
0.50,6.3	0
0.50,6.4	0
0.50,6.5	0
0.50,6.6	0
0.50,6.7	0
0.50,6.8	0
0.50,6.9	0
0.50,7.0	0
0.50,7.1	0
0.50,7.2	0
0.50,7.3	0
0.50,7.4	0
0.50,7.5	0
0.50,7.6	0
0.50,7.7	0
0.50,7.8	0
0.50,7.9	0
0.50,8.0	0
0.50,8.1	0
0.50,8.2	0
0.50,8.3	0
0.50,8.4	0
0.50,8.5	0
0.50,8.6	0
0.50,8.7	0
0.50,8.8	0
0.50,8.9	0

0.50,9.0	0
0.50,9.1	0
0.50,9.2	0
0.50,9.3	0
0.50,9.4	0
0.50,9.5	0
0.50,9.6	0
0.50,9.7	0
0.50,9.8	0
0.50,9.9	0
0.50,10.0	0
0.50,10.1	0
0.50,10.2	0
0.50,10.3	0
0.50,10.4	0
0.50,10.5	0
0.50,10.6	0
0.50,10.7	0
0.50,10.8	0
0.50,10.9	0
0.50,11.0	0
0.50,11.1	0
0.50,11.2	0
0.50,11.3	0
0.50,11.4	0
0.50,11.5	0
0.50,11.6	0
0.50,11.7	0
0.50,11.8	0
0.50,11.9	0
0.50,12.0	0
0.50,12.1	0
0.50,12.2	0
0.50,12.3	0
0.50,12.4	0
0.50,12.5	0
0.50,12.6	0
0.50,12.7	0
0.50,12.8	0
0.50,12.9	0
0.50,13.0	0
0.50,13.1	0

0.50,13.2	0
0.50,13.3	0
0.50,13.4	0
0.50,13.5	0
0.50,13.6	0
0.50,13.7	0
0.50,13.8	0
0.50,13.9	0
0.50,14.0	0
0.50,14.1	0
0.50,14.2	0
0.50,14.3	0
0.50,14.4	0
0.50,14.5	0
0.50,14.6	0
0.50,14.7	0
0.50,14.8	0
0.50,14.9	0
0.50,15.0	0
0.50,15.1	0
0.50,15.2	0
0.50,15.3	0
0.50,15.4	0
0.50,15.5	0
0.50,15.6	0
0.50,15.7	0
0.50,15.8	0
0.50,15.9	0
0.50,16.0	0
0.50,16.1	0
0.50,16.2	0
0.50,16.3	0
0.50,16.4	0
0.50,16.5	0
0.50,16.6	0
0.50,16.7	0
0.50,16.8	0
0.50,16.9	0
0.50,17.0	0
0.50,17.1	0
0.50,17.2	0
0.50,17.3	0

0.50,17.4	0
0.50,17.5	0
0.50,17.6	0
0.50,17.7	0
0.50,17.8	0
0.50,17.9	0
0.50,18.0	0
0.50,18.1	0
0.50,18.2	0
0.50,18.3	0
0.50,18.4	0
0.50,18.5	0
0.50,18.6	0
0.50,18.7	0
0.50,18.8	0
0.50,18.9	0
0.50,19.0	0
0.50,19.1	0
0.50,19.2	0
0.50,19.3	0
0.50,19.4	0
0.50,19.5	0
0.50,19.6	0
0.50,19.7	0
0.50,19.8	0
0.50,19.9	0
0.50,20.0	0
0.50,20.1	0
0.50,20.2	0
0.50,20.3	0
0.50,20.4	0
0.50,20.5	0
0.50,20.6	0
0.50,20.7	0
0.50,20.8	0
0.50,20.9	0
0.50,21.0	0
0.50,21.1	0
0.50,21.2	0
0.50,21.3	0
0.50,21.4	0
0.50,21.5	0

0.50,21.6	0
0.50,21.7	0
0.50,21.8	0
0.50,21.9	0
0.50,22.0	0
0.50,22.1	0
0.50,22.2	0
0.50,22.3	0
0.50,22.4	0
0.50,22.5	0
0.50,22.6	0
0.50,22.7	0
0.50,22.8	0
0.50,22.9	0
0.50,23.0	0
0.50,23.1	0
0.50,23.2	0
0.50,23.3	0
0.50,23.4	0
0.50,23.5	0
0.50,23.6	0
0.50,23.7	0
0.50,23.8	0
0.50,23.9	0
0.50,24.0	0
0.50,24.1	0
0.50,24.2	0
0.50,24.3	0
0.50,24.4	0
0.50,24.5	0
0.50,24.6	0
0.50,24.7	0
0.50,24.8	0
0.50,24.9	0
0.50,25.0	0
0.50,25.1	0
0.50,25.2	0
0.50,25.3	0
0.50,25.4	0
0.50,25.5	0
0.50,25.6	0
0.50,25.7	0

0.50,25.8	0
0.50,25.9	0
0.50,26.0	0
0.50,26.1	0
0.50,26.2	0
0.50,26.3	0
0.50,26.4	0
0.50,26.5	0
0.50,26.6	0
0.50,26.7	0
0.50,26.8	0
0.50,26.9	0
0.50,27.0	0
0.50,27.1	0
0.50,27.2	0
0.50,27.3	0
0.50,27.4	0
0.50,27.5	0
0.50,27.6	0
0.50,27.7	0
0.50,27.8	0
0.50,27.9	0
0.50,28.0	0
0.50,28.1	0
0.50,28.2	0
0.50,28.3	0
0.50,28.4	0
0.50,28.5	0
0.50,28.6	0
0.50,28.7	0
0.50,28.8	0
0.50,28.9	0
0.50,29.0	0
0.50,29.1	0
0.50,29.2	0
0.50,29.3	0
0.50,29.4	0
0.50,29.5	0
0.50,29.6	0
0.50,29.7	0
0.50,29.8	0
0.50,29.9	0

0.50,30.0	0
0.50,30.1	0
0.50,30.2	0
0.50,30.3	0
0.50,30.4	0
0.50,30.5	0
0.50,30.6	0
0.50,30.7	0
0.50,30.8	0
0.50,30.9	0
0.50,31.0	0
0.50,31.1	0
0.50,31.2	0
0.50,31.3	0
0.50,31.4	0
0.50,31.5	0
0.50,31.6	0
0.50,31.7	0
0.50,31.8	0
0.50,31.9	0
0.50,32.0	0
0.50,32.1	0
0.50,32.2	0
0.50,32.3	0
0.50,32.4	0
0.50,32.5	0
0.50,32.6	0
0.50,32.7	0
0.50,32.8	0
0.50,32.9	0
0.50,33.0	0
0.50,33.1	0
0.50,33.2	0
0.50,33.3	0
0.50,33.4	0
0.50,33.5	0
0.50,33.6	0
0.50,33.7	0
0.50,33.8	0
0.50,33.9	0
0.50,34.0	0
0.50,34.1	0

0.50,34.2	0
0.50,34.3	0
0.50,34.4	0
0.50,34.5	0
0.50,34.6	0
0.50,34.7	0
0.50,34.8	0
0.50,34.9	0
0.50,35.0	0
0.50,35.1	0
0.50,35.2	0
0.50,35.3	0
0.50,35.4	0
0.50,35.5	0
0.50,35.6	0
0.50,35.7	0
0.50,35.8	0
0.50,35.9	0
0.50,36.0	0
0.50,36.1	0
0.50,36.2	0
0.50,36.3	0
0.50,36.4	0
0.50,36.5	0
0.50,36.6	0
0.50,36.7	0
0.50,36.8	0
0.50,36.9	0
0.50,37.0	0
0.50,37.1	0
0.50,37.2	0
0.50,37.3	0
0.50,37.4	0
0.50,37.5	0
0.50,37.6	0
0.50,37.7	0
0.50,37.8	0
0.50,37.9	0
0.50,38.0	0
0.50,38.1	0
0.50,38.2	0
0.50,38.3	0

0.50,38.4	0
0.50,38.5	0
0.50,38.6	0
0.50,38.7	0
0.50,38.8	0
0.50,38.9	0
0.50,39.0	0
0.50,39.1	0
0.50,39.2	0
0.50,39.3	0
0.50,39.4	0
0.50,39.5	0
0.50,39.6	0
0.50,39.7	0
0.50,39.8	0
0.50,39.9	0
0.50,40.0	0
0.50,40.1	0
0.50,40.2	0
0.50,40.3	0
0.50,40.4	0
0.50,40.5	0
0.50,40.6	0
0.50,40.7	0
0.50,40.8	0
0.50,40.9	0
0.50,41.0	0
0.50,41.1	0
0.50,41.2	0
0.50,41.3	0
0.50,41.4	0
0.50,41.5	0
0.50,41.6	0
0.50,41.7	0
0.50,41.8	0
0.50,41.9	0
0.50,42.0	0
0.50,42.1	0
0.50,42.2	0
0.50,42.3	0
0.50,42.4	0
0.50,42.5	0

0.50,42.6	0
0.50,42.7	0
0.50,42.8	0
0.50,42.9	0
0.50,43.0	0
0.50,43.1	0
0.50,43.2	0
0.50,43.3	0
0.50,43.4	0
0.50,43.5	0
0.50,43.6	0
0.50,43.7	0
0.50,43.8	0
0.50,43.9	0
0.50,44.0	0
0.50,44.1	0
0.50,44.2	0
0.50,44.3	0
0.50,44.4	0
0.50,44.5	0
0.50,44.6	0
0.50,44.7	0
0.50,44.8	0
0.50,44.9	0
0.50,45.0	0
0.50,45.1	0
0.50,45.2	0
0.50,45.3	0
0.50,45.4	0
0.50,45.5	0
0.50,45.6	0
0.50,45.7	0
0.50,45.8	0
0.50,45.9	0
0.50,46.0	0
0.50,46.1	0
0.50,46.2	0
0.50,46.3	0
0.50,46.4	0
0.50,46.5	0
0.50,46.6	0
0.50,46.7	0

0.50,46.8	0
0.50,46.9	0
0.50,47.0	0
0.50,47.1	0
0.50,47.2	0
0.50,47.3	0
0.50,47.4	0
0.50,47.5	0
0.50,47.6	0
0.50,47.7	0
0.50,47.8	0
0.50,47.9	0
0.50,48.0	0
0.50,48.1	0
0.50,48.2	0
0.50,48.3	0
0.50,48.4	0
0.50,48.5	0
0.50,48.6	0
0.50,48.7	0
0.50,48.8	0
0.50,48.9	0
0.50,49.0	0
0.50,49.1	0
0.50,49.2	0
0.50,49.3	0
0.50,49.4	0
0.50,49.5	0
0.50,49.6	0
0.50,49.7	0
0.50,49.8	0
0.50,49.9	0
0.50,50.0	0
0.50,50.1	0
0.50,50.2	0
0.50,50.3	0
0.50,50.4	0
0.50,50.5	0
0.50,50.6	0
0.50,50.7	0
0.50,50.8	0
0.50,50.9	0

0.50,51.0	0
0.50,51.1	0
0.50,51.2	0
0.50,51.3	0
0.50,51.4	0
0.50,51.5	0
0.50,51.6	0
0.50,51.7	0
0.50,51.8	0
0.50,51.9	0
0.50,52.0	0
0.50,52.1	0
0.50,52.2	0
0.50,52.3	0
0.50,52.4	0
0.50,52.5	0
0.50,52.6	0
0.50,52.7	0
0.50,52.8	0
0.50,52.9	0
0.50,53.0	0
0.50,53.1	0
0.50,53.2	0
0.50,53.3	0
0.50,53.4	0
0.50,53.5	0
0.50,53.6	0
0.50,53.7	0
0.50,53.8	0
0.50,53.9	0
0.50,54.0	0
0.50,54.1	0
0.50,54.2	0
0.50,54.3	0
0.50,54.4	0
0.50,54.5	0
0.50,54.6	0
0.50,54.7	0
0.50,54.8	0
0.50,54.9	0
0.50,55.0	0
0.50,55.1	0

0.50,55.2	0
0.50,55.3	0
0.50,55.4	0
0.50,55.5	0
0.50,55.6	0
0.50,55.7	0
0.50,55.8	0
0.50,55.9	0
0.50,56.0	0
0.50,56.1	0
0.50,56.2	0
0.50,56.3	0
0.50,56.4	0
0.50,56.5	0
0.50,56.6	0
0.50,56.7	0
0.50,56.8	0
0.50,56.9	0
0.50,57.0	0
0.50,57.1	0
0.50,57.2	0
0.50,57.3	0
0.50,57.4	0
0.50,57.5	0
0.50,57.6	0
0.50,57.7	0
0.50,57.8	0
0.50,57.9	0
0.50,58.0	0
0.50,58.1	0
0.50,58.2	0
0.50,58.3	0
0.50,58.4	0
0.50,58.5	0
0.50,58.6	0
0.50,58.7	0
0.50,58.8	0
0.50,58.9	0
0.50,59.0	0
0.50,59.1	0
0.50,59.2	0
0.50,59.3	0

0.50,59.4	0
0.50,59.5	0
0.50,59.6	0
0.50,59.7	0
0.50,59.8	0
0.50,59.9	0
0.50,60.0	0
0.50,60.1	0
0.50,60.2	0
0.50,60.3	0
0.50,60.4	0
0.50,60.5	0
0.50,60.6	0
0.50,60.7	0
0.50,60.8	0
0.50,60.9	0
0.50,61.0	0
0.50,61.1	0
0.50,61.2	0
0.50,61.3	0
0.50,61.4	0
0.50,61.5	0
0.50,61.6	0
0.50,61.7	0
0.50,61.8	0
0.50,61.9	0
0.50,62.0	0
0.50,62.1	0
0.50,62.2	0
0.50,62.3	0
0.50,62.4	0
0.50,62.5	0
0.50,62.6	0
0.50,62.7	0
0.50,62.8	0
0.50,62.9	0
0.50,63.0	0
0.50,63.1	0
0.50,63.2	0
0.50,63.3	0
0.50,63.4	0
0.50,63.5	0

0.50,63.6	0
0.50,63.7	0
0.50,63.8	0
0.50,63.9	0
0.50,64.0	0
0.50,64.1	0
0.50,64.2	0
0.50,64.3	0
0.50,64.4	0
0.50,64.5	0
0.50,64.6	0
0.50,64.7	0
0.50,64.8	0
0.50,64.9	0
0.50,65.0	0
0.50,65.1	0
0.50,65.2	0
0.50,65.3	0
0.50,65.4	0
0.50,65.5	0
0.50,65.6	0
0.50,65.7	0
0.50,65.8	0
0.50,65.9	0
0.50,66.0	0
0.50,66.1	0
0.50,66.2	0
0.50,66.3	0
0.50,66.4	0
0.50,66.5	0
0.50,66.6	0
0.50,66.7	0
0.50,66.8	0
0.50,66.9	0
0.50,67.0	0
0.50,67.1	0
0.50,67.2	0
0.50,67.3	0
0.50,67.4	0
0.50,67.5	0
0.50,67.6	0
0.50,67.7	0

0.50,67.8	0
0.50,67.9	0
0.50,68.0	0
0.50,68.1	0
0.50,68.2	0
0.50,68.3	0
0.50,68.4	0
0.50,68.5	0
0.50,68.6	0
0.50,68.7	0
0.50,68.8	0
0.50,68.9	0
0.50,69.0	0
0.50,69.1	0
0.50,69.2	0
0.50,69.3	0
0.50,69.4	0
0.50,69.5	0
0.50,69.6	0
0.50,69.7	0
0.50,69.8	0
0.50,69.9	0
0.50,70.0	0
0.50,70.1	0
0.50,70.2	0
0.50,70.3	0
0.50,70.4	0
0.50,70.5	0
0.50,70.6	0
0.50,70.7	0
0.50,70.8	0
0.50,70.9	0
0.50,71.0	0
0.50,71.1	0
0.50,71.2	0
0.50,71.3	0
0.50,71.4	0
0.50,71.5	0
0.50,71.6	0
0.50,71.7	0
0.50,71.8	0
0.50,71.9	0

0.50,72.0	0
0.50,72.1	0
0.50,72.2	0
0.50,72.3	0
0.50,72.4	0
0.50,72.5	0
0.50,72.6	0
0.50,72.7	0
0.50,72.8	0
0.50,72.9	0
0.50,73.0	0
0.50,73.1	0
0.50,73.2	0
0.50,73.3	0
0.50,73.4	0
0.50,73.5	0
0.50,73.6	0
0.50,73.7	0
0.50,73.8	0
0.50,73.9	0
0.50,74.0	0
0.50,74.1	0
0.50,74.2	0
0.50,74.3	0
0.50,74.4	0
0.50,74.5	0
0.50,74.6	0
0.50,74.7	0
0.50,74.8	0
0.50,74.9	0
0.50,75.0	0
0.50,75.1	0
0.50,75.2	0
0.50,75.3	0
0.50,75.4	0
0.50,75.5	0
0.50,75.6	0
0.50,75.7	0
0.50,75.8	0
0.50,75.9	0
0.50,76.0	0
0.50,76.1	0

0.50,76.2	0
0.50,76.3	0
0.50,76.4	0
0.50,76.5	0
0.50,76.6	0
0.50,76.7	0
0.50,76.8	0
0.50,76.9	0
0.50,77.0	0
0.50,77.1	0
0.50,77.2	0
0.50,77.3	0
0.50,77.4	0
0.50,77.5	0
0.50,77.6	0
0.50,77.7	0
0.50,77.8	0
0.50,77.9	0
0.50,78.0	0
0.50,78.1	0
0.50,78.2	0
0.50,78.3	0
0.50,78.4	0
0.50,78.5	0
0.50,78.6	0
0.50,78.7	0
0.50,78.8	0
0.50,78.9	0
0.50,79.0	0
0.50,79.1	0
0.50,79.2	0
0.50,79.3	0
0.50,79.4	0
0.50,79.5	0
0.50,79.6	0
0.50,79.7	0
0.50,79.8	0
0.50,79.9	0
0.50,80.0	0
0.50,80.1	0
0.50,80.2	0
0.50,80.3	0

0.50,80.4	0
0.50,80.5	0
0.50,80.6	0
0.50,80.7	0
0.50,80.8	0
0.50,80.9	0
0.50,81.0	0
0.50,81.1	0
0.50,81.2	0
0.50,81.3	0
0.50,81.4	0
0.50,81.5	0
0.50,81.6	0
0.50,81.7	0
0.50,81.8	0
0.50,81.9	0
0.50,82.0	0
0.50,82.1	0
0.50,82.2	0
0.50,82.3	0
0.50,82.4	0
0.50,82.5	0
0.50,82.6	0
0.50,82.7	0
0.50,82.8	0
0.50,82.9	0
0.50,83.0	0
0.50,83.1	0
0.50,83.2	0
0.50,83.3	0
0.50,83.4	0
0.50,83.5	0
0.50,83.6	0
0.50,83.7	0
0.50,83.8	0
0.50,83.9	0
0.50,84.0	0
0.50,84.1	0
0.50,84.2	0
0.50,84.3	0
0.50,84.4	0
0.50,84.5	0

0.50,84.6	0
0.50,84.7	0
0.50,84.8	0
0.50,84.9	0
0.50,85.0	0
0.50,85.1	0
0.50,85.2	0
0.50,85.3	0
0.50,85.4	0
0.50,85.5	0
0.50,85.6	0
0.50,85.7	0
0.50,85.8	0
0.50,85.9	0
0.50,86.0	0
0.50,86.1	0
0.50,86.2	0
0.50,86.3	0
0.50,86.4	0
0.50,86.5	0
0.50,86.6	0
0.50,86.7	0
0.50,86.8	0
0.50,86.9	0
0.50,87.0	0
0.50,87.1	0
0.50,87.2	0
0.50,87.3	0
0.50,87.4	0.000001
0.50,87.5	0.000001
0.50,87.6	0.000001
0.50,87.7	0.000001
0.50,87.8	0.000001
0.50,87.9	0.000001
0.50,88.0	0.000001
0.50,88.1	0.000001
0.50,88.2	0.000001
0.50,88.3	0.000001
0.50,88.4	0.000001
0.50,88.5	0.000001
0.50,88.6	0.000001
0.50,88.7	0.000001

0.50,88.8	0.000001
0.50,88.9	0.000001
0.50,89.0	0.000001
0.50,89.1	0.000001
0.50,89.2	0.000001
0.50,89.3	0.000001
0.50,89.4	0.000001
0.50,89.5	0.000001
0.50,89.6	0.000001
0.50,89.7	0.000001
0.50,89.8	0.000001
0.50,89.9	0.000001
0.50,90.0	0.000001
0.50,90.1	0.000001
0.50,90.2	0.000001
0.50,90.3	0.000001
0.50,90.4	0.000001
0.50,90.5	0.000001
0.50,90.6	0.000001
0.50,90.7	0.000001
0.50,90.8	0.000001
0.50,90.9	0.000001
0.50,91.0	0.000001
0.50,91.1	0.000001
0.50,91.2	0.000001
0.50,91.3	0.000001
0.50,91.4	0.000001
0.50,91.5	0.000001
0.50,91.6	0.000001
0.50,91.7	0.000001
0.50,91.8	0.000001
0.50,91.9	0.000001
0.50,92.0	0.000001
0.50,92.1	0.000001
0.50,92.2	0.000001
0.50,92.3	0.000001
0.50,92.4	0.000001
0.50,92.5	0.000001
0.50,92.6	0.000001
0.50,92.7	0.000001
0.50,92.8	0.000001
0.50,92.9	0.000001

0.50,93.0	0.000001
0.50,93.1	0.000001
0.50,93.2	0.000001
0.50,93.3	0.000001
0.50,93.4	0.000001
0.50,93.5	0.000001
0.50,93.6	0.000001
0.50,93.7	0.000001
0.50,93.8	0.000001
0.50,93.9	0.000001
0.50,94.0	0.000001
0.50,94.1	0.000001
0.50,94.2	0.000001
0.50,94.3	0.000001
0.50,94.4	0.000001
0.50,94.5	0.000001
0.50,94.6	0.000001
0.50,94.7	0.000001
0.50,94.8	0.000001
0.50,94.9	0.000001
0.50,95.0	0.000001
0.50,95.1	0.000001
0.50,95.2	0.000001
0.50,95.3	0.000001
0.50,95.4	0.000001
0.50,95.5	0.000002
0.50,95.6	0.000002
0.50,95.7	0.000002
0.50,95.8	0.000002
0.50,95.9	0.000002
0.50,96.0	0.000002
0.50,96.1	0.000002
0.50,96.2	0.000002
0.50,96.3	0.000002
0.50,96.4	0.000002
0.50,96.5	0.000002
0.50,96.6	0.000002
0.50,96.7	0.000002
0.50,96.8	0.000002
0.50,96.9	0.000002
0.50,97.0	0.000002
0.50,97.1	0.000002

0.50,97.2	0.000002
0.50,97.3	0.000002
0.50,97.4	0.000002
0.50,97.5	0.000002
0.50,97.6	0.000002
0.50,97.7	0.000002
0.50,97.8	0.000002
0.50,97.9	0.000002
0.50,98.0	0.000002
0.50,98.1	0.000002
0.50,98.2	0.000002
0.50,98.3	0.000002
0.50,98.4	0.000002
0.50,98.5	0.000002
0.50,98.6	0.000002
0.50,98.7	0.000002
0.50,98.8	0.000002
0.50,98.9	0.000002
0.50,99.0	0.000002
0.50,99.1	0.000002
0.50,99.2	0.000002
0.50,99.3	0.000002
0.50,99.4	0.000002
0.50,99.5	0.000002
0.50,99.6	0.000003
0.50,99.7	0.000003
0.50,99.8	0.000003
0.50,99.9	0
0.50,100.0	0
0.51,0.0	0
0.51,0.1	0
0.51,0.2	0
0.51,0.3	0
0.51,0.4	0
0.51,0.5	0
0.51,0.6	0
0.51,0.7	0
0.51,0.8	0
0.51,0.9	0
0.51,1.0	0
0.51,1.1	0
0.51,1.2	0

0.51,1.3	0
0.51,1.4	0
0.51,1.5	0
0.51,1.6	0
0.51,1.7	0
0.51,1.8	0
0.51,1.9	0
0.51,2.0	0
0.51,2.1	0
0.51,2.2	0
0.51,2.3	0
0.51,2.4	0
0.51,2.5	0
0.51,2.6	0
0.51,2.7	0
0.51,2.8	0
0.51,2.9	0
0.51,3.0	0
0.51,3.1	0
0.51,3.2	0
0.51,3.3	0
0.51,3.4	0
0.51,3.5	0
0.51,3.6	0
0.51,3.7	0
0.51,3.8	0
0.51,3.9	0
0.51,4.0	0
0.51,4.1	0
0.51,4.2	0
0.51,4.3	0
0.51,4.4	0
0.51,4.5	0
0.51,4.6	0
0.51,4.7	0
0.51,4.8	0
0.51,4.9	0
0.51,5.0	0
0.51,5.1	0
0.51,5.2	0
0.51,5.3	0
0.51,5.4	0

0.51,5.5	0
0.51,5.6	0
0.51,5.7	0
0.51,5.8	0
0.51,5.9	0
0.51,6.0	0
0.51,6.1	0
0.51,6.2	0
0.51,6.3	0
0.51,6.4	0
0.51,6.5	0
0.51,6.6	0
0.51,6.7	0
0.51,6.8	0
0.51,6.9	0
0.51,7.0	0
0.51,7.1	0
0.51,7.2	0
0.51,7.3	0
0.51,7.4	0
0.51,7.5	0
0.51,7.6	0
0.51,7.7	0
0.51,7.8	0
0.51,7.9	0
0.51,8.0	0
0.51,8.1	0
0.51,8.2	0
0.51,8.3	0
0.51,8.4	0
0.51,8.5	0
0.51,8.6	0
0.51,8.7	0
0.51,8.8	0
0.51,8.9	0
0.51,9.0	0
0.51,9.1	0
0.51,9.2	0
0.51,9.3	0
0.51,9.4	0
0.51,9.5	0
0.51,9.6	0

0.51,9.7	0
0.51,9.8	0
0.51,9.9	0
0.51,10.0	0
0.51,10.1	0
0.51,10.2	0
0.51,10.3	0
0.51,10.4	0
0.51,10.5	0
0.51,10.6	0
0.51,10.7	0
0.51,10.8	0
0.51,10.9	0
0.51,11.0	0
0.51,11.1	0
0.51,11.2	0
0.51,11.3	0
0.51,11.4	0
0.51,11.5	0
0.51,11.6	0
0.51,11.7	0
0.51,11.8	0
0.51,11.9	0
0.51,12.0	0
0.51,12.1	0
0.51,12.2	0
0.51,12.3	0
0.51,12.4	0
0.51,12.5	0
0.51,12.6	0
0.51,12.7	0
0.51,12.8	0
0.51,12.9	0
0.51,13.0	0
0.51,13.1	0
0.51,13.2	0
0.51,13.3	0
0.51,13.4	0
0.51,13.5	0
0.51,13.6	0
0.51,13.7	0
0.51,13.8	0

0.51,13.9	0
0.51,14.0	0
0.51,14.1	0
0.51,14.2	0
0.51,14.3	0
0.51,14.4	0
0.51,14.5	0
0.51,14.6	0
0.51,14.7	0
0.51,14.8	0
0.51,14.9	0
0.51,15.0	0
0.51,15.1	0
0.51,15.2	0
0.51,15.3	0
0.51,15.4	0
0.51,15.5	0
0.51,15.6	0
0.51,15.7	0
0.51,15.8	0
0.51,15.9	0
0.51,16.0	0
0.51,16.1	0
0.51,16.2	0
0.51,16.3	0
0.51,16.4	0
0.51,16.5	0
0.51,16.6	0
0.51,16.7	0
0.51,16.8	0
0.51,16.9	0
0.51,17.0	0
0.51,17.1	0
0.51,17.2	0
0.51,17.3	0
0.51,17.4	0
0.51,17.5	0
0.51,17.6	0
0.51,17.7	0
0.51,17.8	0
0.51,17.9	0
0.51,18.0	0

0.51,18.1	0
0.51,18.2	0
0.51,18.3	0
0.51,18.4	0
0.51,18.5	0
0.51,18.6	0
0.51,18.7	0
0.51,18.8	0
0.51,18.9	0
0.51,19.0	0
0.51,19.1	0
0.51,19.2	0
0.51,19.3	0
0.51,19.4	0
0.51,19.5	0
0.51,19.6	0
0.51,19.7	0
0.51,19.8	0
0.51,19.9	0
0.51,20.0	0
0.51,20.1	0
0.51,20.2	0
0.51,20.3	0
0.51,20.4	0
0.51,20.5	0
0.51,20.6	0
0.51,20.7	0
0.51,20.8	0
0.51,20.9	0
0.51,21.0	0
0.51,21.1	0
0.51,21.2	0
0.51,21.3	0
0.51,21.4	0
0.51,21.5	0
0.51,21.6	0
0.51,21.7	0
0.51,21.8	0
0.51,21.9	0
0.51,22.0	0
0.51,22.1	0
0.51,22.2	0

0.51,22.3	0
0.51,22.4	0
0.51,22.5	0
0.51,22.6	0
0.51,22.7	0
0.51,22.8	0
0.51,22.9	0
0.51,23.0	0
0.51,23.1	0
0.51,23.2	0
0.51,23.3	0
0.51,23.4	0
0.51,23.5	0
0.51,23.6	0
0.51,23.7	0
0.51,23.8	0
0.51,23.9	0
0.51,24.0	0
0.51,24.1	0
0.51,24.2	0
0.51,24.3	0
0.51,24.4	0
0.51,24.5	0
0.51,24.6	0
0.51,24.7	0
0.51,24.8	0
0.51,24.9	0
0.51,25.0	0
0.51,25.1	0
0.51,25.2	0
0.51,25.3	0
0.51,25.4	0
0.51,25.5	0
0.51,25.6	0
0.51,25.7	0
0.51,25.8	0
0.51,25.9	0
0.51,26.0	0
0.51,26.1	0
0.51,26.2	0
0.51,26.3	0
0.51,26.4	0

0.51,26.5	0
0.51,26.6	0
0.51,26.7	0
0.51,26.8	0
0.51,26.9	0
0.51,27.0	0
0.51,27.1	0
0.51,27.2	0
0.51,27.3	0
0.51,27.4	0
0.51,27.5	0
0.51,27.6	0
0.51,27.7	0
0.51,27.8	0
0.51,27.9	0
0.51,28.0	0
0.51,28.1	0
0.51,28.2	0
0.51,28.3	0
0.51,28.4	0
0.51,28.5	0
0.51,28.6	0
0.51,28.7	0
0.51,28.8	0
0.51,28.9	0
0.51,29.0	0
0.51,29.1	0
0.51,29.2	0
0.51,29.3	0
0.51,29.4	0
0.51,29.5	0
0.51,29.6	0
0.51,29.7	0
0.51,29.8	0
0.51,29.9	0
0.51,30.0	0
0.51,30.1	0
0.51,30.2	0
0.51,30.3	0
0.51,30.4	0
0.51,30.5	0
0.51,30.6	0

0.51,30.7	0
0.51,30.8	0
0.51,30.9	0
0.51,31.0	0
0.51,31.1	0
0.51,31.2	0
0.51,31.3	0
0.51,31.4	0
0.51,31.5	0
0.51,31.6	0
0.51,31.7	0
0.51,31.8	0
0.51,31.9	0
0.51,32.0	0
0.51,32.1	0
0.51,32.2	0
0.51,32.3	0
0.51,32.4	0
0.51,32.5	0
0.51,32.6	0
0.51,32.7	0
0.51,32.8	0
0.51,32.9	0
0.51,33.0	0
0.51,33.1	0
0.51,33.2	0
0.51,33.3	0
0.51,33.4	0
0.51,33.5	0
0.51,33.6	0
0.51,33.7	0
0.51,33.8	0
0.51,33.9	0
0.51,34.0	0
0.51,34.1	0
0.51,34.2	0
0.51,34.3	0
0.51,34.4	0
0.51,34.5	0
0.51,34.6	0
0.51,34.7	0
0.51,34.8	0

0.51,34.9	0
0.51,35.0	0
0.51,35.1	0
0.51,35.2	0
0.51,35.3	0
0.51,35.4	0
0.51,35.5	0
0.51,35.6	0
0.51,35.7	0
0.51,35.8	0
0.51,35.9	0
0.51,36.0	0
0.51,36.1	0
0.51,36.2	0
0.51,36.3	0
0.51,36.4	0
0.51,36.5	0
0.51,36.6	0
0.51,36.7	0
0.51,36.8	0
0.51,36.9	0
0.51,37.0	0
0.51,37.1	0
0.51,37.2	0
0.51,37.3	0
0.51,37.4	0
0.51,37.5	0
0.51,37.6	0
0.51,37.7	0
0.51,37.8	0
0.51,37.9	0
0.51,38.0	0
0.51,38.1	0
0.51,38.2	0
0.51,38.3	0
0.51,38.4	0
0.51,38.5	0
0.51,38.6	0
0.51,38.7	0
0.51,38.8	0
0.51,38.9	0
0.51,39.0	0

0.51,39.1	0
0.51,39.2	0
0.51,39.3	0
0.51,39.4	0
0.51,39.5	0
0.51,39.6	0
0.51,39.7	0
0.51,39.8	0
0.51,39.9	0
0.51,40.0	0
0.51,40.1	0
0.51,40.2	0
0.51,40.3	0
0.51,40.4	0
0.51,40.5	0
0.51,40.6	0
0.51,40.7	0
0.51,40.8	0
0.51,40.9	0
0.51,41.0	0
0.51,41.1	0
0.51,41.2	0
0.51,41.3	0
0.51,41.4	0
0.51,41.5	0
0.51,41.6	0
0.51,41.7	0
0.51,41.8	0
0.51,41.9	0
0.51,42.0	0
0.51,42.1	0
0.51,42.2	0
0.51,42.3	0
0.51,42.4	0
0.51,42.5	0
0.51,42.6	0
0.51,42.7	0
0.51,42.8	0
0.51,42.9	0
0.51,43.0	0
0.51,43.1	0
0.51,43.2	0

0.51,43.3	0
0.51,43.4	0
0.51,43.5	0
0.51,43.6	0
0.51,43.7	0
0.51,43.8	0
0.51,43.9	0
0.51,44.0	0
0.51,44.1	0
0.51,44.2	0
0.51,44.3	0
0.51,44.4	0
0.51,44.5	0
0.51,44.6	0
0.51,44.7	0
0.51,44.8	0
0.51,44.9	0
0.51,45.0	0
0.51,45.1	0
0.51,45.2	0
0.51,45.3	0
0.51,45.4	0
0.51,45.5	0
0.51,45.6	0
0.51,45.7	0
0.51,45.8	0
0.51,45.9	0
0.51,46.0	0
0.51,46.1	0
0.51,46.2	0
0.51,46.3	0
0.51,46.4	0
0.51,46.5	0
0.51,46.6	0
0.51,46.7	0
0.51,46.8	0
0.51,46.9	0
0.51,47.0	0
0.51,47.1	0
0.51,47.2	0
0.51,47.3	0
0.51,47.4	0

0.51,47.5	0
0.51,47.6	0
0.51,47.7	0
0.51,47.8	0
0.51,47.9	0
0.51,48.0	0
0.51,48.1	0
0.51,48.2	0
0.51,48.3	0
0.51,48.4	0
0.51,48.5	0
0.51,48.6	0
0.51,48.7	0
0.51,48.8	0
0.51,48.9	0
0.51,49.0	0
0.51,49.1	0
0.51,49.2	0
0.51,49.3	0
0.51,49.4	0
0.51,49.5	0
0.51,49.6	0
0.51,49.7	0
0.51,49.8	0
0.51,49.9	0
0.51,50.0	0
0.51,50.1	0
0.51,50.2	0
0.51,50.3	0
0.51,50.4	0
0.51,50.5	0
0.51,50.6	0
0.51,50.7	0
0.51,50.8	0
0.51,50.9	0
0.51,51.0	0
0.51,51.1	0
0.51,51.2	0
0.51,51.3	0
0.51,51.4	0
0.51,51.5	0
0.51,51.6	0

0.51,51.7	0
0.51,51.8	0
0.51,51.9	0
0.51,52.0	0
0.51,52.1	0
0.51,52.2	0
0.51,52.3	0
0.51,52.4	0
0.51,52.5	0
0.51,52.6	0
0.51,52.7	0
0.51,52.8	0
0.51,52.9	0
0.51,53.0	0
0.51,53.1	0
0.51,53.2	0
0.51,53.3	0
0.51,53.4	0
0.51,53.5	0
0.51,53.6	0
0.51,53.7	0
0.51,53.8	0
0.51,53.9	0
0.51,54.0	0
0.51,54.1	0
0.51,54.2	0
0.51,54.3	0
0.51,54.4	0
0.51,54.5	0
0.51,54.6	0
0.51,54.7	0
0.51,54.8	0
0.51,54.9	0
0.51,55.0	0
0.51,55.1	0
0.51,55.2	0
0.51,55.3	0
0.51,55.4	0
0.51,55.5	0
0.51,55.6	0
0.51,55.7	0
0.51,55.8	0

0.51,55.9	0
0.51,56.0	0
0.51,56.1	0
0.51,56.2	0
0.51,56.3	0
0.51,56.4	0
0.51,56.5	0
0.51,56.6	0
0.51,56.7	0
0.51,56.8	0
0.51,56.9	0
0.51,57.0	0
0.51,57.1	0
0.51,57.2	0
0.51,57.3	0
0.51,57.4	0
0.51,57.5	0
0.51,57.6	0
0.51,57.7	0
0.51,57.8	0
0.51,57.9	0
0.51,58.0	0
0.51,58.1	0
0.51,58.2	0
0.51,58.3	0
0.51,58.4	0
0.51,58.5	0
0.51,58.6	0
0.51,58.7	0
0.51,58.8	0
0.51,58.9	0
0.51,59.0	0
0.51,59.1	0
0.51,59.2	0
0.51,59.3	0
0.51,59.4	0
0.51,59.5	0
0.51,59.6	0
0.51,59.7	0
0.51,59.8	0
0.51,59.9	0
0.51,60.0	0

0.51,60.1	0
0.51,60.2	0
0.51,60.3	0
0.51,60.4	0
0.51,60.5	0
0.51,60.6	0
0.51,60.7	0
0.51,60.8	0
0.51,60.9	0
0.51,61.0	0
0.51,61.1	0
0.51,61.2	0
0.51,61.3	0
0.51,61.4	0
0.51,61.5	0
0.51,61.6	0
0.51,61.7	0
0.51,61.8	0
0.51,61.9	0
0.51,62.0	0
0.51,62.1	0
0.51,62.2	0
0.51,62.3	0
0.51,62.4	0
0.51,62.5	0
0.51,62.6	0
0.51,62.7	0
0.51,62.8	0
0.51,62.9	0
0.51,63.0	0
0.51,63.1	0
0.51,63.2	0
0.51,63.3	0
0.51,63.4	0
0.51,63.5	0
0.51,63.6	0
0.51,63.7	0
0.51,63.8	0
0.51,63.9	0
0.51,64.0	0
0.51,64.1	0
0.51,64.2	0

0.51,64.3	0
0.51,64.4	0
0.51,64.5	0
0.51,64.6	0
0.51,64.7	0
0.51,64.8	0
0.51,64.9	0
0.51,65.0	0
0.51,65.1	0
0.51,65.2	0
0.51,65.3	0
0.51,65.4	0
0.51,65.5	0
0.51,65.6	0
0.51,65.7	0
0.51,65.8	0
0.51,65.9	0
0.51,66.0	0
0.51,66.1	0
0.51,66.2	0
0.51,66.3	0
0.51,66.4	0
0.51,66.5	0
0.51,66.6	0
0.51,66.7	0
0.51,66.8	0
0.51,66.9	0
0.51,67.0	0
0.51,67.1	0
0.51,67.2	0
0.51,67.3	0
0.51,67.4	0
0.51,67.5	0
0.51,67.6	0
0.51,67.7	0
0.51,67.8	0
0.51,67.9	0
0.51,68.0	0
0.51,68.1	0
0.51,68.2	0
0.51,68.3	0
0.51,68.4	0

0.51,68.5	0
0.51,68.6	0
0.51,68.7	0
0.51,68.8	0
0.51,68.9	0
0.51,69.0	0
0.51,69.1	0
0.51,69.2	0
0.51,69.3	0
0.51,69.4	0
0.51,69.5	0
0.51,69.6	0
0.51,69.7	0
0.51,69.8	0
0.51,69.9	0
0.51,70.0	0
0.51,70.1	0
0.51,70.2	0
0.51,70.3	0
0.51,70.4	0
0.51,70.5	0
0.51,70.6	0
0.51,70.7	0
0.51,70.8	0
0.51,70.9	0
0.51,71.0	0
0.51,71.1	0
0.51,71.2	0
0.51,71.3	0
0.51,71.4	0
0.51,71.5	0
0.51,71.6	0
0.51,71.7	0
0.51,71.8	0
0.51,71.9	0
0.51,72.0	0
0.51,72.1	0
0.51,72.2	0
0.51,72.3	0
0.51,72.4	0
0.51,72.5	0
0.51,72.6	0

0.51,72.7	0
0.51,72.8	0
0.51,72.9	0
0.51,73.0	0
0.51,73.1	0
0.51,73.2	0
0.51,73.3	0
0.51,73.4	0
0.51,73.5	0
0.51,73.6	0
0.51,73.7	0
0.51,73.8	0
0.51,73.9	0
0.51,74.0	0
0.51,74.1	0
0.51,74.2	0
0.51,74.3	0
0.51,74.4	0
0.51,74.5	0
0.51,74.6	0
0.51,74.7	0
0.51,74.8	0
0.51,74.9	0
0.51,75.0	0
0.51,75.1	0
0.51,75.2	0
0.51,75.3	0
0.51,75.4	0
0.51,75.5	0
0.51,75.6	0
0.51,75.7	0
0.51,75.8	0
0.51,75.9	0
0.51,76.0	0
0.51,76.1	0
0.51,76.2	0
0.51,76.3	0
0.51,76.4	0
0.51,76.5	0
0.51,76.6	0
0.51,76.7	0
0.51,76.8	0

0.51,76.9	0
0.51,77.0	0
0.51,77.1	0
0.51,77.2	0
0.51,77.3	0
0.51,77.4	0
0.51,77.5	0
0.51,77.6	0
0.51,77.7	0
0.51,77.8	0
0.51,77.9	0
0.51,78.0	0
0.51,78.1	0
0.51,78.2	0
0.51,78.3	0
0.51,78.4	0
0.51,78.5	0
0.51,78.6	0
0.51,78.7	0
0.51,78.8	0
0.51,78.9	0
0.51,79.0	0
0.51,79.1	0
0.51,79.2	0
0.51,79.3	0
0.51,79.4	0
0.51,79.5	0
0.51,79.6	0
0.51,79.7	0
0.51,79.8	0
0.51,79.9	0
0.51,80.0	0
0.51,80.1	0
0.51,80.2	0
0.51,80.3	0
0.51,80.4	0
0.51,80.5	0
0.51,80.6	0
0.51,80.7	0
0.51,80.8	0
0.51,80.9	0
0.51,81.0	0

0.51,81.1	0
0.51,81.2	0
0.51,81.3	0
0.51,81.4	0
0.51,81.5	0
0.51,81.6	0
0.51,81.7	0
0.51,81.8	0
0.51,81.9	0
0.51,82.0	0
0.51,82.1	0
0.51,82.2	0
0.51,82.3	0
0.51,82.4	0
0.51,82.5	0
0.51,82.6	0
0.51,82.7	0
0.51,82.8	0
0.51,82.9	0
0.51,83.0	0
0.51,83.1	0
0.51,83.2	0
0.51,83.3	0
0.51,83.4	0
0.51,83.5	0
0.51,83.6	0
0.51,83.7	0
0.51,83.8	0
0.51,83.9	0
0.51,84.0	0
0.51,84.1	0
0.51,84.2	0
0.51,84.3	0
0.51,84.4	0
0.51,84.5	0
0.51,84.6	0
0.51,84.7	0
0.51,84.8	0
0.51,84.9	0
0.51,85.0	0
0.51,85.1	0
0.51,85.2	0

0.51,85.3	0
0.51,85.4	0
0.51,85.5	0
0.51,85.6	0
0.51,85.7	0
0.51,85.8	0
0.51,85.9	0
0.51,86.0	0
0.51,86.1	0
0.51,86.2	0
0.51,86.3	0
0.51,86.4	0
0.51,86.5	0
0.51,86.6	0
0.51,86.7	0
0.51,86.8	0
0.51,86.9	0
0.51,87.0	0
0.51,87.1	0
0.51,87.2	0
0.51,87.3	0
0.51,87.4	0
0.51,87.5	0
0.51,87.6	0
0.51,87.7	0
0.51,87.8	0
0.51,87.9	0
0.51,88.0	0
0.51,88.1	0
0.51,88.2	0
0.51,88.3	0
0.51,88.4	0
0.51,88.5	0
0.51,88.6	0
0.51,88.7	0
0.51,88.8	0
0.51,88.9	0
0.51,89.0	0
0.51,89.1	0
0.51,89.2	0
0.51,89.3	0
0.51,89.4	0

0.51,89.5	0
0.51,89.6	0
0.51,89.7	0
0.51,89.8	0
0.51,89.9	0
0.51,90.0	0
0.51,90.1	0
0.51,90.2	0
0.51,90.3	0
0.51,90.4	0
0.51,90.5	0
0.51,90.6	0
0.51,90.7	0
0.51,90.8	0
0.51,90.9	0
0.51,91.0	0
0.51,91.1	0
0.51,91.2	0
0.51,91.3	0
0.51,91.4	0
0.51,91.5	0
0.51,91.6	0
0.51,91.7	0
0.51,91.8	0
0.51,91.9	0
0.51,92.0	0
0.51,92.1	0
0.51,92.2	0
0.51,92.3	0
0.51,92.4	0
0.51,92.5	0
0.51,92.6	0
0.51,92.7	0
0.51,92.8	0
0.51,92.9	0
0.51,93.0	0
0.51,93.1	0
0.51,93.2	0
0.51,93.3	0
0.51,93.4	0
0.51,93.5	0
0.51,93.6	0

0.51,93.7	0
0.51,93.8	0
0.51,93.9	0
0.51,94.0	0
0.51,94.1	0
0.51,94.2	0
0.51,94.3	0
0.51,94.4	0
0.51,94.5	0
0.51,94.6	0
0.51,94.7	0
0.51,94.8	0
0.51,94.9	0
0.51,95.0	0
0.51,95.1	0
0.51,95.2	0
0.51,95.3	0
0.51,95.4	0
0.51,95.5	0
0.51,95.6	0
0.51,95.7	0
0.51,95.8	0
0.51,95.9	0
0.51,96.0	0
0.51,96.1	0
0.51,96.2	0
0.51,96.3	0
0.51,96.4	0
0.51,96.5	0
0.51,96.6	0
0.51,96.7	0
0.51,96.8	0
0.51,96.9	0
0.51,97.0	0
0.51,97.1	0
0.51,97.2	0
0.51,97.3	0
0.51,97.4	0
0.51,97.5	0
0.51,97.6	0
0.51,97.7	0
0.51,97.8	0

0.51,97.9	0
0.51,98.0	0
0.51,98.1	0
0.51,98.2	0
0.51,98.3	0
0.51,98.4	0
0.51,98.5	0
0.51,98.6	0
0.51,98.7	0
0.51,98.8	0
0.51,98.9	0
0.51,99.0	0
0.51,99.1	0
0.51,99.2	0
0.51,99.3	0
0.51,99.4	0
0.51,99.5	0
0.51,99.6	0
0.51,99.7	0
0.51,99.8	0
0.51,99.9	0
0.51,100.0	0
0.52,0.0	0
0.52,0.1	0
0.52,0.2	0
0.52,0.3	0
0.52,0.4	0
0.52,0.5	0
0.52,0.6	0
0.52,0.7	0
0.52,0.8	0
0.52,0.9	0
0.52,1.0	0
0.52,1.1	0
0.52,1.2	0
0.52,1.3	0
0.52,1.4	0
0.52,1.5	0
0.52,1.6	0
0.52,1.7	0
0.52,1.8	0
0.52,1.9	0

0.52,2.0	0
0.52,2.1	0
0.52,2.2	0
0.52,2.3	0
0.52,2.4	0
0.52,2.5	0
0.52,2.6	0
0.52,2.7	0
0.52,2.8	0
0.52,2.9	0
0.52,3.0	0
0.52,3.1	0
0.52,3.2	0
0.52,3.3	0
0.52,3.4	0
0.52,3.5	0
0.52,3.6	0
0.52,3.7	0
0.52,3.8	0
0.52,3.9	0
0.52,4.0	0
0.52,4.1	0
0.52,4.2	0
0.52,4.3	0
0.52,4.4	0
0.52,4.5	0
0.52,4.6	0
0.52,4.7	0
0.52,4.8	0
0.52,4.9	0
0.52,5.0	0
0.52,5.1	0
0.52,5.2	0
0.52,5.3	0
0.52,5.4	0
0.52,5.5	0
0.52,5.6	0
0.52,5.7	0
0.52,5.8	0
0.52,5.9	0
0.52,6.0	0
0.52,6.1	0

0.52,6.2	0
0.52,6.3	0
0.52,6.4	0
0.52,6.5	0
0.52,6.6	0
0.52,6.7	0
0.52,6.8	0
0.52,6.9	0
0.52,7.0	0
0.52,7.1	0
0.52,7.2	0
0.52,7.3	0
0.52,7.4	0
0.52,7.5	0
0.52,7.6	0
0.52,7.7	0
0.52,7.8	0
0.52,7.9	0
0.52,8.0	0
0.52,8.1	0
0.52,8.2	0
0.52,8.3	0
0.52,8.4	0
0.52,8.5	0
0.52,8.6	0
0.52,8.7	0
0.52,8.8	0
0.52,8.9	0
0.52,9.0	0
0.52,9.1	0
0.52,9.2	0
0.52,9.3	0
0.52,9.4	0
0.52,9.5	0
0.52,9.6	0
0.52,9.7	0
0.52,9.8	0
0.52,9.9	0
0.52,10.0	0
0.52,10.1	0
0.52,10.2	0
0.52,10.3	0

0.52,10.4	0
0.52,10.5	0
0.52,10.6	0
0.52,10.7	0
0.52,10.8	0
0.52,10.9	0
0.52,11.0	0
0.52,11.1	0
0.52,11.2	0
0.52,11.3	0
0.52,11.4	0
0.52,11.5	0
0.52,11.6	0
0.52,11.7	0
0.52,11.8	0
0.52,11.9	0
0.52,12.0	0
0.52,12.1	0
0.52,12.2	0
0.52,12.3	0
0.52,12.4	0
0.52,12.5	0
0.52,12.6	0
0.52,12.7	0
0.52,12.8	0
0.52,12.9	0
0.52,13.0	0
0.52,13.1	0
0.52,13.2	0
0.52,13.3	0
0.52,13.4	0
0.52,13.5	0
0.52,13.6	0
0.52,13.7	0
0.52,13.8	0
0.52,13.9	0
0.52,14.0	0
0.52,14.1	0
0.52,14.2	0
0.52,14.3	0
0.52,14.4	0
0.52,14.5	0

0.52,14.6	0
0.52,14.7	0
0.52,14.8	0
0.52,14.9	0
0.52,15.0	0
0.52,15.1	0
0.52,15.2	0
0.52,15.3	0
0.52,15.4	0
0.52,15.5	0
0.52,15.6	0
0.52,15.7	0
0.52,15.8	0
0.52,15.9	0
0.52,16.0	0
0.52,16.1	0
0.52,16.2	0
0.52,16.3	0
0.52,16.4	0
0.52,16.5	0
0.52,16.6	0
0.52,16.7	0
0.52,16.8	0
0.52,16.9	0
0.52,17.0	0
0.52,17.1	0
0.52,17.2	0
0.52,17.3	0
0.52,17.4	0
0.52,17.5	0
0.52,17.6	0
0.52,17.7	0
0.52,17.8	0
0.52,17.9	0
0.52,18.0	0
0.52,18.1	0
0.52,18.2	0
0.52,18.3	0
0.52,18.4	0
0.52,18.5	0
0.52,18.6	0
0.52,18.7	0

0.52,18.8	0
0.52,18.9	0
0.52,19.0	0
0.52,19.1	0
0.52,19.2	0
0.52,19.3	0
0.52,19.4	0
0.52,19.5	0
0.52,19.6	0
0.52,19.7	0
0.52,19.8	0
0.52,19.9	0
0.52,20.0	0
0.52,20.1	0
0.52,20.2	0
0.52,20.3	0
0.52,20.4	0
0.52,20.5	0
0.52,20.6	0
0.52,20.7	0
0.52,20.8	0
0.52,20.9	0
0.52,21.0	0
0.52,21.1	0
0.52,21.2	0
0.52,21.3	0
0.52,21.4	0
0.52,21.5	0
0.52,21.6	0
0.52,21.7	0
0.52,21.8	0
0.52,21.9	0
0.52,22.0	0
0.52,22.1	0
0.52,22.2	0
0.52,22.3	0
0.52,22.4	0
0.52,22.5	0
0.52,22.6	0
0.52,22.7	0
0.52,22.8	0
0.52,22.9	0

0.52,23.0	0
0.52,23.1	0
0.52,23.2	0
0.52,23.3	0
0.52,23.4	0
0.52,23.5	0
0.52,23.6	0
0.52,23.7	0
0.52,23.8	0
0.52,23.9	0
0.52,24.0	0
0.52,24.1	0
0.52,24.2	0
0.52,24.3	0
0.52,24.4	0
0.52,24.5	0
0.52,24.6	0
0.52,24.7	0
0.52,24.8	0
0.52,24.9	0
0.52,25.0	0
0.52,25.1	0
0.52,25.2	0
0.52,25.3	0
0.52,25.4	0
0.52,25.5	0
0.52,25.6	0
0.52,25.7	0
0.52,25.8	0
0.52,25.9	0
0.52,26.0	0
0.52,26.1	0
0.52,26.2	0
0.52,26.3	0
0.52,26.4	0
0.52,26.5	0
0.52,26.6	0
0.52,26.7	0
0.52,26.8	0
0.52,26.9	0
0.52,27.0	0
0.52,27.1	0

0.52,27.2	0
0.52,27.3	0
0.52,27.4	0
0.52,27.5	0
0.52,27.6	0
0.52,27.7	0
0.52,27.8	0
0.52,27.9	0
0.52,28.0	0
0.52,28.1	0
0.52,28.2	0
0.52,28.3	0
0.52,28.4	0
0.52,28.5	0
0.52,28.6	0
0.52,28.7	0
0.52,28.8	0
0.52,28.9	0
0.52,29.0	0
0.52,29.1	0
0.52,29.2	0
0.52,29.3	0
0.52,29.4	0
0.52,29.5	0
0.52,29.6	0
0.52,29.7	0
0.52,29.8	0
0.52,29.9	0
0.52,30.0	0
0.52,30.1	0
0.52,30.2	0
0.52,30.3	0
0.52,30.4	0
0.52,30.5	0
0.52,30.6	0
0.52,30.7	0
0.52,30.8	0
0.52,30.9	0
0.52,31.0	0
0.52,31.1	0
0.52,31.2	0
0.52,31.3	0

0.52,31.4	0
0.52,31.5	0
0.52,31.6	0
0.52,31.7	0
0.52,31.8	0
0.52,31.9	0
0.52,32.0	0
0.52,32.1	0
0.52,32.2	0
0.52,32.3	0
0.52,32.4	0
0.52,32.5	0
0.52,32.6	0
0.52,32.7	0
0.52,32.8	0
0.52,32.9	0
0.52,33.0	0
0.52,33.1	0
0.52,33.2	0
0.52,33.3	0
0.52,33.4	0
0.52,33.5	0
0.52,33.6	0
0.52,33.7	0
0.52,33.8	0
0.52,33.9	0
0.52,34.0	0
0.52,34.1	0
0.52,34.2	0
0.52,34.3	0
0.52,34.4	0
0.52,34.5	0
0.52,34.6	0
0.52,34.7	0
0.52,34.8	0
0.52,34.9	0
0.52,35.0	0
0.52,35.1	0
0.52,35.2	0
0.52,35.3	0
0.52,35.4	0
0.52,35.5	0

0.52,35.6	0
0.52,35.7	0
0.52,35.8	0
0.52,35.9	0
0.52,36.0	0
0.52,36.1	0
0.52,36.2	0
0.52,36.3	0
0.52,36.4	0
0.52,36.5	0
0.52,36.6	0
0.52,36.7	0
0.52,36.8	0
0.52,36.9	0
0.52,37.0	0
0.52,37.1	0
0.52,37.2	0
0.52,37.3	0
0.52,37.4	0
0.52,37.5	0
0.52,37.6	0
0.52,37.7	0
0.52,37.8	0
0.52,37.9	0
0.52,38.0	0
0.52,38.1	0
0.52,38.2	0
0.52,38.3	0
0.52,38.4	0
0.52,38.5	0
0.52,38.6	0
0.52,38.7	0
0.52,38.8	0
0.52,38.9	0
0.52,39.0	0
0.52,39.1	0
0.52,39.2	0
0.52,39.3	0
0.52,39.4	0
0.52,39.5	0
0.52,39.6	0
0.52,39.7	0

0.52,39.8	0
0.52,39.9	0
0.52,40.0	0
0.52,40.1	0
0.52,40.2	0
0.52,40.3	0
0.52,40.4	0
0.52,40.5	0
0.52,40.6	0
0.52,40.7	0
0.52,40.8	0
0.52,40.9	0
0.52,41.0	0
0.52,41.1	0
0.52,41.2	0
0.52,41.3	0
0.52,41.4	0
0.52,41.5	0
0.52,41.6	0
0.52,41.7	0
0.52,41.8	0
0.52,41.9	0
0.52,42.0	0
0.52,42.1	0
0.52,42.2	0
0.52,42.3	0
0.52,42.4	0
0.52,42.5	0
0.52,42.6	0
0.52,42.7	0
0.52,42.8	0
0.52,42.9	0
0.52,43.0	0
0.52,43.1	0
0.52,43.2	0
0.52,43.3	0
0.52,43.4	0
0.52,43.5	0
0.52,43.6	0
0.52,43.7	0
0.52,43.8	0
0.52,43.9	0

0.52,44.0	0
0.52,44.1	0
0.52,44.2	0
0.52,44.3	0
0.52,44.4	0
0.52,44.5	0
0.52,44.6	0
0.52,44.7	0
0.52,44.8	0
0.52,44.9	0
0.52,45.0	0
0.52,45.1	0
0.52,45.2	0
0.52,45.3	0
0.52,45.4	0
0.52,45.5	0
0.52,45.6	0
0.52,45.7	0
0.52,45.8	0
0.52,45.9	0
0.52,46.0	0
0.52,46.1	0
0.52,46.2	0
0.52,46.3	0
0.52,46.4	0
0.52,46.5	0
0.52,46.6	0
0.52,46.7	0
0.52,46.8	0
0.52,46.9	0
0.52,47.0	0
0.52,47.1	0
0.52,47.2	0
0.52,47.3	0
0.52,47.4	0
0.52,47.5	0
0.52,47.6	0
0.52,47.7	0
0.52,47.8	0
0.52,47.9	0
0.52,48.0	0
0.52,48.1	0

0.52,48.2	0
0.52,48.3	0
0.52,48.4	0
0.52,48.5	0
0.52,48.6	0
0.52,48.7	0
0.52,48.8	0
0.52,48.9	0
0.52,49.0	0
0.52,49.1	0
0.52,49.2	0
0.52,49.3	0
0.52,49.4	0
0.52,49.5	0
0.52,49.6	0
0.52,49.7	0
0.52,49.8	0
0.52,49.9	0
0.52,50.0	0
0.52,50.1	0
0.52,50.2	0
0.52,50.3	0
0.52,50.4	0
0.52,50.5	0
0.52,50.6	0
0.52,50.7	0
0.52,50.8	0
0.52,50.9	0
0.52,51.0	0
0.52,51.1	0
0.52,51.2	0
0.52,51.3	0
0.52,51.4	0
0.52,51.5	0
0.52,51.6	0
0.52,51.7	0
0.52,51.8	0
0.52,51.9	0
0.52,52.0	0
0.52,52.1	0
0.52,52.2	0
0.52,52.3	0

0.52,52.4	0
0.52,52.5	0
0.52,52.6	0
0.52,52.7	0
0.52,52.8	0
0.52,52.9	0
0.52,53.0	0
0.52,53.1	0
0.52,53.2	0
0.52,53.3	0
0.52,53.4	0
0.52,53.5	0
0.52,53.6	0
0.52,53.7	0
0.52,53.8	0
0.52,53.9	0
0.52,54.0	0
0.52,54.1	0
0.52,54.2	0
0.52,54.3	0
0.52,54.4	0
0.52,54.5	0
0.52,54.6	0
0.52,54.7	0
0.52,54.8	0
0.52,54.9	0
0.52,55.0	0
0.52,55.1	0
0.52,55.2	0
0.52,55.3	0
0.52,55.4	0
0.52,55.5	0
0.52,55.6	0
0.52,55.7	0
0.52,55.8	0
0.52,55.9	0
0.52,56.0	0
0.52,56.1	0
0.52,56.2	0
0.52,56.3	0
0.52,56.4	0
0.52,56.5	0

0.52,56.6	0
0.52,56.7	0
0.52,56.8	0
0.52,56.9	0
0.52,57.0	0
0.52,57.1	0
0.52,57.2	0
0.52,57.3	0
0.52,57.4	0
0.52,57.5	0
0.52,57.6	0
0.52,57.7	0
0.52,57.8	0
0.52,57.9	0
0.52,58.0	0
0.52,58.1	0
0.52,58.2	0
0.52,58.3	0
0.52,58.4	0
0.52,58.5	0
0.52,58.6	0
0.52,58.7	0
0.52,58.8	0
0.52,58.9	0
0.52,59.0	0
0.52,59.1	0
0.52,59.2	0
0.52,59.3	0
0.52,59.4	0
0.52,59.5	0
0.52,59.6	0
0.52,59.7	0
0.52,59.8	0
0.52,59.9	0
0.52,60.0	0
0.52,60.1	0
0.52,60.2	0
0.52,60.3	0
0.52,60.4	0
0.52,60.5	0
0.52,60.6	0
0.52,60.7	0

0.52,60.8	0
0.52,60.9	0
0.52,61.0	0
0.52,61.1	0
0.52,61.2	0
0.52,61.3	0
0.52,61.4	0
0.52,61.5	0
0.52,61.6	0
0.52,61.7	0
0.52,61.8	0
0.52,61.9	0
0.52,62.0	0
0.52,62.1	0
0.52,62.2	0
0.52,62.3	0
0.52,62.4	0
0.52,62.5	0
0.52,62.6	0
0.52,62.7	0
0.52,62.8	0
0.52,62.9	0
0.52,63.0	0
0.52,63.1	0
0.52,63.2	0
0.52,63.3	0
0.52,63.4	0
0.52,63.5	0
0.52,63.6	0
0.52,63.7	0
0.52,63.8	0
0.52,63.9	0
0.52,64.0	0
0.52,64.1	0
0.52,64.2	0
0.52,64.3	0
0.52,64.4	0
0.52,64.5	0
0.52,64.6	0
0.52,64.7	0
0.52,64.8	0
0.52,64.9	0

0.52,65.0	0
0.52,65.1	0
0.52,65.2	0
0.52,65.3	0
0.52,65.4	0
0.52,65.5	0
0.52,65.6	0
0.52,65.7	0
0.52,65.8	0
0.52,65.9	0
0.52,66.0	0
0.52,66.1	0
0.52,66.2	0
0.52,66.3	0
0.52,66.4	0
0.52,66.5	0
0.52,66.6	0
0.52,66.7	0
0.52,66.8	0
0.52,66.9	0
0.52,67.0	0
0.52,67.1	0
0.52,67.2	0
0.52,67.3	0
0.52,67.4	0
0.52,67.5	0
0.52,67.6	0
0.52,67.7	0
0.52,67.8	0
0.52,67.9	0
0.52,68.0	0
0.52,68.1	0
0.52,68.2	0
0.52,68.3	0
0.52,68.4	0
0.52,68.5	0
0.52,68.6	0
0.52,68.7	0
0.52,68.8	0
0.52,68.9	0
0.52,69.0	0
0.52,69.1	0

0.52,69.2	0
0.52,69.3	0
0.52,69.4	0
0.52,69.5	0
0.52,69.6	0
0.52,69.7	0
0.52,69.8	0
0.52,69.9	0
0.52,70.0	0
0.52,70.1	0
0.52,70.2	0
0.52,70.3	0
0.52,70.4	0
0.52,70.5	0
0.52,70.6	0
0.52,70.7	0
0.52,70.8	0
0.52,70.9	0
0.52,71.0	0
0.52,71.1	0
0.52,71.2	0
0.52,71.3	0
0.52,71.4	0
0.52,71.5	0
0.52,71.6	0
0.52,71.7	0
0.52,71.8	0
0.52,71.9	0
0.52,72.0	0
0.52,72.1	0
0.52,72.2	0
0.52,72.3	0
0.52,72.4	0
0.52,72.5	0
0.52,72.6	0
0.52,72.7	0
0.52,72.8	0
0.52,72.9	0
0.52,73.0	0
0.52,73.1	0
0.52,73.2	0
0.52,73.3	0

0.52,73.4	0
0.52,73.5	0
0.52,73.6	0
0.52,73.7	0
0.52,73.8	0
0.52,73.9	0
0.52,74.0	0
0.52,74.1	0
0.52,74.2	0
0.52,74.3	0
0.52,74.4	0
0.52,74.5	0
0.52,74.6	0
0.52,74.7	0
0.52,74.8	0
0.52,74.9	0
0.52,75.0	0
0.52,75.1	0
0.52,75.2	0
0.52,75.3	0
0.52,75.4	0
0.52,75.5	0
0.52,75.6	0
0.52,75.7	0
0.52,75.8	0
0.52,75.9	0
0.52,76.0	0
0.52,76.1	0
0.52,76.2	0
0.52,76.3	0
0.52,76.4	0
0.52,76.5	0
0.52,76.6	0
0.52,76.7	0
0.52,76.8	0
0.52,76.9	0
0.52,77.0	0
0.52,77.1	0
0.52,77.2	0
0.52,77.3	0
0.52,77.4	0
0.52,77.5	0

0.52,77.6	0
0.52,77.7	0
0.52,77.8	0
0.52,77.9	0
0.52,78.0	0
0.52,78.1	0
0.52,78.2	0
0.52,78.3	0
0.52,78.4	0
0.52,78.5	0
0.52,78.6	0
0.52,78.7	0
0.52,78.8	0
0.52,78.9	0
0.52,79.0	0
0.52,79.1	0
0.52,79.2	0
0.52,79.3	0
0.52,79.4	0
0.52,79.5	0
0.52,79.6	0
0.52,79.7	0
0.52,79.8	0
0.52,79.9	0
0.52,80.0	0
0.52,80.1	0
0.52,80.2	0
0.52,80.3	0
0.52,80.4	0
0.52,80.5	0
0.52,80.6	0
0.52,80.7	0
0.52,80.8	0
0.52,80.9	0
0.52,81.0	0
0.52,81.1	0
0.52,81.2	0
0.52,81.3	0
0.52,81.4	0
0.52,81.5	0
0.52,81.6	0
0.52,81.7	0

0.52,81.8	0
0.52,81.9	0
0.52,82.0	0
0.52,82.1	0
0.52,82.2	0
0.52,82.3	0
0.52,82.4	0
0.52,82.5	0
0.52,82.6	0
0.52,82.7	0
0.52,82.8	0
0.52,82.9	0
0.52,83.0	0
0.52,83.1	0
0.52,83.2	0
0.52,83.3	0
0.52,83.4	0
0.52,83.5	0
0.52,83.6	0
0.52,83.7	0
0.52,83.8	0
0.52,83.9	0
0.52,84.0	0
0.52,84.1	0
0.52,84.2	0
0.52,84.3	0
0.52,84.4	0
0.52,84.5	0
0.52,84.6	0
0.52,84.7	0
0.52,84.8	0
0.52,84.9	0
0.52,85.0	0
0.52,85.1	0
0.52,85.2	0
0.52,85.3	0
0.52,85.4	0
0.52,85.5	0
0.52,85.6	0
0.52,85.7	0
0.52,85.8	0
0.52,85.9	0

0.52,86.0	0
0.52,86.1	0
0.52,86.2	0
0.52,86.3	0
0.52,86.4	0.000001
0.52,86.5	0.000001
0.52,86.6	0.000001
0.52,86.7	0.000001
0.52,86.8	0.000001
0.52,86.9	0.000001
0.52,87.0	0.000001
0.52,87.1	0.000001
0.52,87.2	0.000001
0.52,87.3	0.000001
0.52,87.4	0.000001
0.52,87.5	0.000001
0.52,87.6	0.000001
0.52,87.7	0.000001
0.52,87.8	0.000001
0.52,87.9	0.000001
0.52,88.0	0.000001
0.52,88.1	0.000001
0.52,88.2	0.000001
0.52,88.3	0.000001
0.52,88.4	0.000001
0.52,88.5	0.000001
0.52,88.6	0.000001
0.52,88.7	0.000001
0.52,88.8	0.000001
0.52,88.9	0.000001
0.52,89.0	0.000001
0.52,89.1	0.000001
0.52,89.2	0.000001
0.52,89.3	0.000001
0.52,89.4	0.000001
0.52,89.5	0.000001
0.52,89.6	0.000001
0.52,89.7	0.000001
0.52,89.8	0.000001
0.52,89.9	0.000001
0.52,90.0	0.000001
0.52,90.1	0.000001

0.52,90.2	0.000001
0.52,90.3	0.000001
0.52,90.4	0.000001
0.52,90.5	0.000001
0.52,90.6	0.000001
0.52,90.7	0.000001
0.52,90.8	0.000001
0.52,90.9	0.000001
0.52,91.0	0.000001
0.52,91.1	0.000001
0.52,91.2	0.000001
0.52,91.3	0.000001
0.52,91.4	0.000001
0.52,91.5	0.000001
0.52,91.6	0.000001
0.52,91.7	0.000001
0.52,91.8	0.000001
0.52,91.9	0.000001
0.52,92.0	0.000001
0.52,92.1	0.000001
0.52,92.2	0.000001
0.52,92.3	0.000001
0.52,92.4	0.000001
0.52,92.5	0.000001
0.52,92.6	0.000001
0.52,92.7	0.000001
0.52,92.8	0.000001
0.52,92.9	0.000001
0.52,93.0	0.000001
0.52,93.1	0.000001
0.52,93.2	0.000001
0.52,93.3	0.000001
0.52,93.4	0.000001
0.52,93.5	0.000001
0.52,93.6	0.000001
0.52,93.7	0.000001
0.52,93.8	0.000001
0.52,93.9	0.000001
0.52,94.0	0.000001
0.52,94.1	0.000001
0.52,94.2	0.000001
0.52,94.3	0.000001

0.52,94.4	0.000001
0.52,94.5	0.000001
0.52,94.6	0.000001
0.52,94.7	0.000001
0.52,94.8	0.000001
0.52,94.9	0.000001
0.52,95.0	0.000001
0.52,95.1	0.000001
0.52,95.2	0.000001
0.52,95.3	0.000001
0.52,95.4	0.000002
0.52,95.5	0.000002
0.52,95.6	0.000002
0.52,95.7	0.000002
0.52,95.8	0.000002
0.52,95.9	0.000002
0.52,96.0	0.000002
0.52,96.1	0.000002
0.52,96.2	0.000002
0.52,96.3	0.000002
0.52,96.4	0.000002
0.52,96.5	0.000002
0.52,96.6	0.000002
0.52,96.7	0.000002
0.52,96.8	0.000002
0.52,96.9	0.000002
0.52,97.0	0.000002
0.52,97.1	0.000002
0.52,97.2	0.000002
0.52,97.3	0.000003
0.52,97.4	0.000003
0.52,97.5	0.000003
0.52,97.6	0.000003
0.52,97.7	0.000003
0.52,97.8	0
0.52,97.9	0
0.52,98.0	0
0.52,98.1	0
0.52,98.2	0
0.52,98.3	0
0.52,98.4	0
0.52,98.5	0

0.52,98.6	0
0.52,98.7	0
0.52,98.8	0
0.52,98.9	0
0.52,99.0	0
0.52,99.1	0
0.52,99.2	0
0.52,99.3	0
0.52,99.4	0
0.52,99.5	0
0.52,99.6	0
0.52,99.7	0
0.52,99.8	0
0.52,99.9	0
0.52,100.0	0
0.53,0.0	0
0.53,0.1	0
0.53,0.2	0
0.53,0.3	0
0.53,0.4	0
0.53,0.5	0
0.53,0.6	0
0.53,0.7	0
0.53,0.8	0
0.53,0.9	0
0.53,1.0	0
0.53,1.1	0
0.53,1.2	0
0.53,1.3	0
0.53,1.4	0
0.53,1.5	0
0.53,1.6	0
0.53,1.7	0
0.53,1.8	0
0.53,1.9	0
0.53,2.0	0
0.53,2.1	0
0.53,2.2	0
0.53,2.3	0
0.53,2.4	0
0.53,2.5	0
0.53,2.6	0

0.53,2.7	0
0.53,2.8	0
0.53,2.9	0
0.53,3.0	0
0.53,3.1	0
0.53,3.2	0
0.53,3.3	0
0.53,3.4	0
0.53,3.5	0
0.53,3.6	0
0.53,3.7	0
0.53,3.8	0
0.53,3.9	0
0.53,4.0	0
0.53,4.1	0
0.53,4.2	0
0.53,4.3	0
0.53,4.4	0
0.53,4.5	0
0.53,4.6	0
0.53,4.7	0
0.53,4.8	0
0.53,4.9	0
0.53,5.0	0
0.53,5.1	0
0.53,5.2	0
0.53,5.3	0
0.53,5.4	0
0.53,5.5	0
0.53,5.6	0
0.53,5.7	0
0.53,5.8	0
0.53,5.9	0
0.53,6.0	0
0.53,6.1	0
0.53,6.2	0
0.53,6.3	0
0.53,6.4	0
0.53,6.5	0
0.53,6.6	0
0.53,6.7	0
0.53,6.8	0

0.53,6.9	0
0.53,7.0	0
0.53,7.1	0
0.53,7.2	0
0.53,7.3	0
0.53,7.4	0
0.53,7.5	0
0.53,7.6	0
0.53,7.7	0
0.53,7.8	0
0.53,7.9	0
0.53,8.0	0
0.53,8.1	0
0.53,8.2	0
0.53,8.3	0
0.53,8.4	0
0.53,8.5	0
0.53,8.6	0
0.53,8.7	0
0.53,8.8	0
0.53,8.9	0
0.53,9.0	0
0.53,9.1	0
0.53,9.2	0
0.53,9.3	0
0.53,9.4	0
0.53,9.5	0
0.53,9.6	0
0.53,9.7	0
0.53,9.8	0
0.53,9.9	0
0.53,10.0	0
0.53,10.1	0
0.53,10.2	0
0.53,10.3	0
0.53,10.4	0
0.53,10.5	0
0.53,10.6	0
0.53,10.7	0
0.53,10.8	0
0.53,10.9	0
0.53,11.0	0

0.53,11.1	0
0.53,11.2	0
0.53,11.3	0
0.53,11.4	0
0.53,11.5	0
0.53,11.6	0
0.53,11.7	0
0.53,11.8	0
0.53,11.9	0
0.53,12.0	0
0.53,12.1	0
0.53,12.2	0
0.53,12.3	0
0.53,12.4	0
0.53,12.5	0
0.53,12.6	0
0.53,12.7	0
0.53,12.8	0
0.53,12.9	0
0.53,13.0	0
0.53,13.1	0
0.53,13.2	0
0.53,13.3	0
0.53,13.4	0
0.53,13.5	0
0.53,13.6	0
0.53,13.7	0
0.53,13.8	0
0.53,13.9	0
0.53,14.0	0
0.53,14.1	0
0.53,14.2	0
0.53,14.3	0
0.53,14.4	0
0.53,14.5	0
0.53,14.6	0
0.53,14.7	0
0.53,14.8	0
0.53,14.9	0
0.53,15.0	0
0.53,15.1	0
0.53,15.2	0

0.53,15.3	0
0.53,15.4	0
0.53,15.5	0
0.53,15.6	0
0.53,15.7	0
0.53,15.8	0
0.53,15.9	0
0.53,16.0	0
0.53,16.1	0
0.53,16.2	0
0.53,16.3	0
0.53,16.4	0
0.53,16.5	0
0.53,16.6	0
0.53,16.7	0
0.53,16.8	0
0.53,16.9	0
0.53,17.0	0
0.53,17.1	0
0.53,17.2	0
0.53,17.3	0
0.53,17.4	0
0.53,17.5	0
0.53,17.6	0
0.53,17.7	0
0.53,17.8	0
0.53,17.9	0
0.53,18.0	0
0.53,18.1	0
0.53,18.2	0
0.53,18.3	0
0.53,18.4	0
0.53,18.5	0
0.53,18.6	0
0.53,18.7	0
0.53,18.8	0
0.53,18.9	0
0.53,19.0	0
0.53,19.1	0
0.53,19.2	0
0.53,19.3	0
0.53,19.4	0

0.53,19.5	0
0.53,19.6	0
0.53,19.7	0
0.53,19.8	0
0.53,19.9	0
0.53,20.0	0
0.53,20.1	0
0.53,20.2	0
0.53,20.3	0
0.53,20.4	0
0.53,20.5	0
0.53,20.6	0
0.53,20.7	0
0.53,20.8	0
0.53,20.9	0
0.53,21.0	0
0.53,21.1	0
0.53,21.2	0
0.53,21.3	0
0.53,21.4	0
0.53,21.5	0
0.53,21.6	0
0.53,21.7	0
0.53,21.8	0
0.53,21.9	0
0.53,22.0	0
0.53,22.1	0
0.53,22.2	0
0.53,22.3	0
0.53,22.4	0
0.53,22.5	0
0.53,22.6	0
0.53,22.7	0
0.53,22.8	0
0.53,22.9	0
0.53,23.0	0
0.53,23.1	0
0.53,23.2	0
0.53,23.3	0
0.53,23.4	0
0.53,23.5	0
0.53,23.6	0

0.53,23.7	0
0.53,23.8	0
0.53,23.9	0
0.53,24.0	0
0.53,24.1	0
0.53,24.2	0
0.53,24.3	0
0.53,24.4	0
0.53,24.5	0
0.53,24.6	0
0.53,24.7	0
0.53,24.8	0
0.53,24.9	0
0.53,25.0	0
0.53,25.1	0
0.53,25.2	0
0.53,25.3	0
0.53,25.4	0
0.53,25.5	0
0.53,25.6	0
0.53,25.7	0
0.53,25.8	0
0.53,25.9	0
0.53,26.0	0
0.53,26.1	0
0.53,26.2	0
0.53,26.3	0
0.53,26.4	0
0.53,26.5	0
0.53,26.6	0
0.53,26.7	0
0.53,26.8	0
0.53,26.9	0
0.53,27.0	0
0.53,27.1	0
0.53,27.2	0
0.53,27.3	0
0.53,27.4	0
0.53,27.5	0
0.53,27.6	0
0.53,27.7	0
0.53,27.8	0

0.53,27.9	0
0.53,28.0	0
0.53,28.1	0
0.53,28.2	0
0.53,28.3	0
0.53,28.4	0
0.53,28.5	0
0.53,28.6	0
0.53,28.7	0
0.53,28.8	0
0.53,28.9	0
0.53,29.0	0
0.53,29.1	0
0.53,29.2	0
0.53,29.3	0
0.53,29.4	0
0.53,29.5	0
0.53,29.6	0
0.53,29.7	0
0.53,29.8	0
0.53,29.9	0
0.53,30.0	0
0.53,30.1	0
0.53,30.2	0
0.53,30.3	0
0.53,30.4	0
0.53,30.5	0
0.53,30.6	0
0.53,30.7	0
0.53,30.8	0
0.53,30.9	0
0.53,31.0	0
0.53,31.1	0
0.53,31.2	0
0.53,31.3	0
0.53,31.4	0
0.53,31.5	0
0.53,31.6	0
0.53,31.7	0
0.53,31.8	0
0.53,31.9	0
0.53,32.0	0

0.53,32.1	0
0.53,32.2	0
0.53,32.3	0
0.53,32.4	0
0.53,32.5	0
0.53,32.6	0
0.53,32.7	0
0.53,32.8	0
0.53,32.9	0
0.53,33.0	0
0.53,33.1	0
0.53,33.2	0
0.53,33.3	0
0.53,33.4	0
0.53,33.5	0
0.53,33.6	0
0.53,33.7	0
0.53,33.8	0
0.53,33.9	0
0.53,34.0	0
0.53,34.1	0
0.53,34.2	0
0.53,34.3	0
0.53,34.4	0
0.53,34.5	0
0.53,34.6	0
0.53,34.7	0
0.53,34.8	0
0.53,34.9	0
0.53,35.0	0
0.53,35.1	0
0.53,35.2	0
0.53,35.3	0
0.53,35.4	0
0.53,35.5	0
0.53,35.6	0
0.53,35.7	0
0.53,35.8	0
0.53,35.9	0
0.53,36.0	0
0.53,36.1	0
0.53,36.2	0

0.53,36.3	0
0.53,36.4	0
0.53,36.5	0
0.53,36.6	0
0.53,36.7	0
0.53,36.8	0
0.53,36.9	0
0.53,37.0	0
0.53,37.1	0
0.53,37.2	0
0.53,37.3	0
0.53,37.4	0
0.53,37.5	0
0.53,37.6	0
0.53,37.7	0
0.53,37.8	0
0.53,37.9	0
0.53,38.0	0
0.53,38.1	0
0.53,38.2	0
0.53,38.3	0
0.53,38.4	0
0.53,38.5	0
0.53,38.6	0
0.53,38.7	0
0.53,38.8	0
0.53,38.9	0
0.53,39.0	0
0.53,39.1	0
0.53,39.2	0
0.53,39.3	0
0.53,39.4	0
0.53,39.5	0
0.53,39.6	0
0.53,39.7	0
0.53,39.8	0
0.53,39.9	0
0.53,40.0	0
0.53,40.1	0
0.53,40.2	0
0.53,40.3	0
0.53,40.4	0

0.53,40.5	0
0.53,40.6	0
0.53,40.7	0
0.53,40.8	0
0.53,40.9	0
0.53,41.0	0
0.53,41.1	0
0.53,41.2	0
0.53,41.3	0
0.53,41.4	0
0.53,41.5	0
0.53,41.6	0
0.53,41.7	0
0.53,41.8	0
0.53,41.9	0
0.53,42.0	0
0.53,42.1	0
0.53,42.2	0
0.53,42.3	0
0.53,42.4	0
0.53,42.5	0
0.53,42.6	0
0.53,42.7	0
0.53,42.8	0
0.53,42.9	0
0.53,43.0	0
0.53,43.1	0
0.53,43.2	0
0.53,43.3	0
0.53,43.4	0
0.53,43.5	0
0.53,43.6	0
0.53,43.7	0
0.53,43.8	0
0.53,43.9	0
0.53,44.0	0
0.53,44.1	0
0.53,44.2	0
0.53,44.3	0
0.53,44.4	0
0.53,44.5	0
0.53,44.6	0

0.53,44.7	0
0.53,44.8	0
0.53,44.9	0
0.53,45.0	0
0.53,45.1	0
0.53,45.2	0
0.53,45.3	0
0.53,45.4	0
0.53,45.5	0
0.53,45.6	0
0.53,45.7	0
0.53,45.8	0
0.53,45.9	0
0.53,46.0	0
0.53,46.1	0
0.53,46.2	0
0.53,46.3	0
0.53,46.4	0
0.53,46.5	0
0.53,46.6	0
0.53,46.7	0
0.53,46.8	0
0.53,46.9	0
0.53,47.0	0
0.53,47.1	0
0.53,47.2	0
0.53,47.3	0
0.53,47.4	0
0.53,47.5	0
0.53,47.6	0
0.53,47.7	0
0.53,47.8	0
0.53,47.9	0
0.53,48.0	0
0.53,48.1	0
0.53,48.2	0
0.53,48.3	0
0.53,48.4	0
0.53,48.5	0
0.53,48.6	0
0.53,48.7	0
0.53,48.8	0

0.53,48.9	0
0.53,49.0	0
0.53,49.1	0
0.53,49.2	0
0.53,49.3	0
0.53,49.4	0
0.53,49.5	0
0.53,49.6	0
0.53,49.7	0
0.53,49.8	0
0.53,49.9	0
0.53,50.0	0
0.53,50.1	0
0.53,50.2	0
0.53,50.3	0
0.53,50.4	0
0.53,50.5	0
0.53,50.6	0
0.53,50.7	0
0.53,50.8	0
0.53,50.9	0
0.53,51.0	0
0.53,51.1	0
0.53,51.2	0
0.53,51.3	0
0.53,51.4	0
0.53,51.5	0
0.53,51.6	0
0.53,51.7	0
0.53,51.8	0
0.53,51.9	0
0.53,52.0	0
0.53,52.1	0
0.53,52.2	0
0.53,52.3	0
0.53,52.4	0
0.53,52.5	0
0.53,52.6	0
0.53,52.7	0
0.53,52.8	0
0.53,52.9	0
0.53,53.0	0

0.53,53.1	0
0.53,53.2	0
0.53,53.3	0
0.53,53.4	0
0.53,53.5	0
0.53,53.6	0
0.53,53.7	0
0.53,53.8	0
0.53,53.9	0
0.53,54.0	0
0.53,54.1	0
0.53,54.2	0
0.53,54.3	0
0.53,54.4	0
0.53,54.5	0
0.53,54.6	0
0.53,54.7	0
0.53,54.8	0
0.53,54.9	0
0.53,55.0	0
0.53,55.1	0
0.53,55.2	0
0.53,55.3	0
0.53,55.4	0
0.53,55.5	0
0.53,55.6	0
0.53,55.7	0
0.53,55.8	0
0.53,55.9	0
0.53,56.0	0
0.53,56.1	0
0.53,56.2	0
0.53,56.3	0
0.53,56.4	0
0.53,56.5	0
0.53,56.6	0
0.53,56.7	0
0.53,56.8	0
0.53,56.9	0
0.53,57.0	0
0.53,57.1	0
0.53,57.2	0

0.53,57.3	0
0.53,57.4	0
0.53,57.5	0
0.53,57.6	0
0.53,57.7	0
0.53,57.8	0
0.53,57.9	0
0.53,58.0	0
0.53,58.1	0
0.53,58.2	0
0.53,58.3	0
0.53,58.4	0
0.53,58.5	0
0.53,58.6	0
0.53,58.7	0
0.53,58.8	0
0.53,58.9	0
0.53,59.0	0
0.53,59.1	0
0.53,59.2	0
0.53,59.3	0
0.53,59.4	0
0.53,59.5	0
0.53,59.6	0
0.53,59.7	0
0.53,59.8	0
0.53,59.9	0
0.53,60.0	0
0.53,60.1	0
0.53,60.2	0
0.53,60.3	0
0.53,60.4	0
0.53,60.5	0
0.53,60.6	0
0.53,60.7	0
0.53,60.8	0
0.53,60.9	0
0.53,61.0	0
0.53,61.1	0
0.53,61.2	0
0.53,61.3	0
0.53,61.4	0

0.53,61.5	0
0.53,61.6	0
0.53,61.7	0
0.53,61.8	0
0.53,61.9	0
0.53,62.0	0
0.53,62.1	0
0.53,62.2	0
0.53,62.3	0
0.53,62.4	0
0.53,62.5	0
0.53,62.6	0
0.53,62.7	0
0.53,62.8	0
0.53,62.9	0
0.53,63.0	0
0.53,63.1	0
0.53,63.2	0
0.53,63.3	0
0.53,63.4	0
0.53,63.5	0
0.53,63.6	0
0.53,63.7	0
0.53,63.8	0
0.53,63.9	0
0.53,64.0	0
0.53,64.1	0
0.53,64.2	0
0.53,64.3	0
0.53,64.4	0
0.53,64.5	0
0.53,64.6	0
0.53,64.7	0
0.53,64.8	0
0.53,64.9	0
0.53,65.0	0
0.53,65.1	0
0.53,65.2	0
0.53,65.3	0
0.53,65.4	0
0.53,65.5	0
0.53,65.6	0

0.53,65.7	0
0.53,65.8	0
0.53,65.9	0
0.53,66.0	0
0.53,66.1	0
0.53,66.2	0
0.53,66.3	0
0.53,66.4	0
0.53,66.5	0
0.53,66.6	0
0.53,66.7	0
0.53,66.8	0
0.53,66.9	0
0.53,67.0	0
0.53,67.1	0
0.53,67.2	0
0.53,67.3	0
0.53,67.4	0
0.53,67.5	0
0.53,67.6	0
0.53,67.7	0
0.53,67.8	0
0.53,67.9	0
0.53,68.0	0
0.53,68.1	0
0.53,68.2	0
0.53,68.3	0
0.53,68.4	0
0.53,68.5	0
0.53,68.6	0
0.53,68.7	0
0.53,68.8	0
0.53,68.9	0
0.53,69.0	0
0.53,69.1	0
0.53,69.2	0
0.53,69.3	0
0.53,69.4	0
0.53,69.5	0
0.53,69.6	0
0.53,69.7	0
0.53,69.8	0

0.53,69.9	0
0.53,70.0	0
0.53,70.1	0
0.53,70.2	0
0.53,70.3	0
0.53,70.4	0
0.53,70.5	0
0.53,70.6	0
0.53,70.7	0
0.53,70.8	0
0.53,70.9	0
0.53,71.0	0
0.53,71.1	0
0.53,71.2	0
0.53,71.3	0
0.53,71.4	0
0.53,71.5	0
0.53,71.6	0
0.53,71.7	0
0.53,71.8	0
0.53,71.9	0
0.53,72.0	0
0.53,72.1	0
0.53,72.2	0
0.53,72.3	0
0.53,72.4	0
0.53,72.5	0
0.53,72.6	0
0.53,72.7	0
0.53,72.8	0
0.53,72.9	0
0.53,73.0	0
0.53,73.1	0
0.53,73.2	0
0.53,73.3	0
0.53,73.4	0
0.53,73.5	0
0.53,73.6	0
0.53,73.7	0
0.53,73.8	0
0.53,73.9	0
0.53,74.0	0

0.53,74.1	0
0.53,74.2	0
0.53,74.3	0
0.53,74.4	0
0.53,74.5	0
0.53,74.6	0
0.53,74.7	0
0.53,74.8	0
0.53,74.9	0
0.53,75.0	0
0.53,75.1	0
0.53,75.2	0
0.53,75.3	0
0.53,75.4	0
0.53,75.5	0
0.53,75.6	0
0.53,75.7	0
0.53,75.8	0
0.53,75.9	0
0.53,76.0	0
0.53,76.1	0
0.53,76.2	0
0.53,76.3	0
0.53,76.4	0
0.53,76.5	0
0.53,76.6	0
0.53,76.7	0
0.53,76.8	0
0.53,76.9	0
0.53,77.0	0
0.53,77.1	0
0.53,77.2	0
0.53,77.3	0
0.53,77.4	0
0.53,77.5	0
0.53,77.6	0
0.53,77.7	0
0.53,77.8	0
0.53,77.9	0
0.53,78.0	0
0.53,78.1	0
0.53,78.2	0

0.53,78.3	0
0.53,78.4	0
0.53,78.5	0
0.53,78.6	0
0.53,78.7	0
0.53,78.8	0
0.53,78.9	0
0.53,79.0	0
0.53,79.1	0
0.53,79.2	0
0.53,79.3	0
0.53,79.4	0
0.53,79.5	0
0.53,79.6	0
0.53,79.7	0
0.53,79.8	0
0.53,79.9	0
0.53,80.0	0
0.53,80.1	0
0.53,80.2	0
0.53,80.3	0
0.53,80.4	0
0.53,80.5	0
0.53,80.6	0
0.53,80.7	0
0.53,80.8	0
0.53,80.9	0
0.53,81.0	0
0.53,81.1	0
0.53,81.2	0
0.53,81.3	0
0.53,81.4	0
0.53,81.5	0
0.53,81.6	0
0.53,81.7	0
0.53,81.8	0
0.53,81.9	0
0.53,82.0	0
0.53,82.1	0
0.53,82.2	0
0.53,82.3	0
0.53,82.4	0

0.53,82.5	0
0.53,82.6	0
0.53,82.7	0
0.53,82.8	0
0.53,82.9	0
0.53,83.0	0
0.53,83.1	0
0.53,83.2	0
0.53,83.3	0
0.53,83.4	0
0.53,83.5	0
0.53,83.6	0
0.53,83.7	0
0.53,83.8	0
0.53,83.9	0.000001
0.53,84.0	0.000001
0.53,84.1	0.000001
0.53,84.2	0.000001
0.53,84.3	0.000001
0.53,84.4	0.000001
0.53,84.5	0.000001
0.53,84.6	0.000001
0.53,84.7	0.000001
0.53,84.8	0.000002
0.53,84.9	0.000002
0.53,85.0	0.000002
0.53,85.1	0.000003
0.53,85.2	0.000003
0.53,85.3	0.000004
0.53,85.4	0.000004
0.53,85.5	0.000004
0.53,85.6	0.000004
0.53,85.7	0.000005
0.53,85.8	0.000005
0.53,85.9	0.000006
0.53,86.0	0.000006
0.53,86.1	0.000006
0.53,86.2	0.000006
0.53,86.3	0.000006
0.53,86.4	0.000006
0.53,86.5	0.000006
0.53,86.6	0.000007

0.53,86.7	0.000007
0.53,86.8	0.000007
0.53,86.9	0.000007
0.53,87.0	0.000008
0.53,87.1	0.000008
0.53,87.2	0.000008
0.53,87.3	0.000008
0.53,87.4	0.000008
0.53,87.5	0.000008
0.53,87.6	0.000008
0.53,87.7	0.000008
0.53,87.8	0.000008
0.53,87.9	0.000008
0.53,88.0	0.000008
0.53,88.1	0.000009
0.53,88.2	0.000009
0.53,88.3	0.000009
0.53,88.4	0.000009
0.53,88.5	0.000009
0.53,88.6	0.000009
0.53,88.7	0.000009
0.53,88.8	0.000009
0.53,88.9	0.000009
0.53,89.0	0.000009
0.53,89.1	0.000009
0.53,89.2	0.000009
0.53,89.3	0.000009
0.53,89.4	0.000009
0.53,89.5	0.000009
0.53,89.6	0.000009
0.53,89.7	0.000009
0.53,89.8	0.000009
0.53,89.9	0.000009
0.53,90.0	0.000009
0.53,90.1	0.000009
0.53,90.2	0.000009
0.53,90.3	0.000009
0.53,90.4	0.000009
0.53,90.5	0.000009
0.53,90.6	0.000009
0.53,90.7	0.000009
0.53,90.8	0.00001

0.53,90.9	0.00001
0.53,91.0	0.00001
0.53,91.1	0.00001
0.53,91.2	0.00001
0.53,91.3	0.000011
0.53,91.4	0.000011
0.53,91.5	0.000011
0.53,91.6	0.000011
0.53,91.7	0.000011
0.53,91.8	0.000011
0.53,91.9	0.000011
0.53,92.0	0.000011
0.53,92.1	0.000011
0.53,92.2	0.000011
0.53,92.3	0.000011
0.53,92.4	0.000012
0.53,92.5	0.000012
0.53,92.6	0.000012
0.53,92.7	0.000012
0.53,92.8	0.000014
0.53,92.9	0.000015
0.53,93.0	0.000015
0.53,93.1	0.000015
0.53,93.2	0.000015
0.53,93.3	0.000015
0.53,93.4	0.000015
0.53,93.5	0.000015
0.53,93.6	0.000015
0.53,93.7	0.000015
0.53,93.8	0.000015
0.53,93.9	0.000015
0.53,94.0	0.000015
0.53,94.1	0.000015
0.53,94.2	0.000015
0.53,94.3	0.000015
0.53,94.4	0.000015
0.53,94.5	0.000015
0.53,94.6	0.000015
0.53,94.7	0.000015
0.53,94.8	0.000017
0.53,94.9	0.000017
0.53,95.0	0.000017

0.53,95.1	0.000017
0.53,95.2	0.000017
0.53,95.3	0.000017
0.53,95.4	0.000017
0.53,95.5	0.000017
0.53,95.6	0.000017
0.53,95.7	0.000017
0.53,95.8	0.000017
0.53,95.9	0.000017
0.53,96.0	0.000019
0.53,96.1	0.000019
0.53,96.2	0.000021
0.53,96.3	0.000021
0.53,96.4	0.000021
0.53,96.5	0.000021
0.53,96.6	0.000021
0.53,96.7	0.000021
0.53,96.8	0.000021
0.53,96.9	0.000023
0.53,97.0	0.000023
0.53,97.1	0.000023
0.53,97.2	0.000023
0.53,97.3	0.000023
0.53,97.4	0.000023
0.53,97.5	0.000023
0.53,97.6	0.000023
0.53,97.7	0.000023
0.53,97.8	0.000026
0.53,97.9	0.000026
0.53,98.0	0.000026
0.53,98.1	0.000026
0.53,98.2	0.000026
0.53,98.3	0.000026
0.53,98.4	0.000026
0.53,98.5	0.000026
0.53,98.6	0.000026
0.53,98.7	0.000017
0.53,98.8	0.00001
0.53,98.9	0.00001
0.53,99.0	0.00001
0.53,99.1	0.00001
0.53,99.2	0.00001

0.53,99.3	0.00001
0.53,99.4	0.00001
0.53,99.5	0.00001
0.53,99.6	0.00001
0.53,99.7	0.000012
0.53,99.8	0.000012
0.53,99.9	0.000012
0.53,100.0	0
0.54,0.0	0
0.54,0.1	0
0.54,0.2	0
0.54,0.3	0
0.54,0.4	0
0.54,0.5	0
0.54,0.6	0
0.54,0.7	0
0.54,0.8	0
0.54,0.9	0
0.54,1.0	0
0.54,1.1	0
0.54,1.2	0
0.54,1.3	0
0.54,1.4	0
0.54,1.5	0
0.54,1.6	0
0.54,1.7	0
0.54,1.8	0
0.54,1.9	0
0.54,2.0	0
0.54,2.1	0
0.54,2.2	0
0.54,2.3	0
0.54,2.4	0
0.54,2.5	0
0.54,2.6	0
0.54,2.7	0
0.54,2.8	0
0.54,2.9	0
0.54,3.0	0
0.54,3.1	0
0.54,3.2	0
0.54,3.3	0

0.54,3.4	0
0.54,3.5	0
0.54,3.6	0
0.54,3.7	0
0.54,3.8	0
0.54,3.9	0
0.54,4.0	0
0.54,4.1	0
0.54,4.2	0
0.54,4.3	0
0.54,4.4	0
0.54,4.5	0
0.54,4.6	0
0.54,4.7	0
0.54,4.8	0
0.54,4.9	0
0.54,5.0	0
0.54,5.1	0
0.54,5.2	0
0.54,5.3	0
0.54,5.4	0
0.54,5.5	0
0.54,5.6	0
0.54,5.7	0
0.54,5.8	0
0.54,5.9	0
0.54,6.0	0
0.54,6.1	0
0.54,6.2	0
0.54,6.3	0
0.54,6.4	0
0.54,6.5	0
0.54,6.6	0
0.54,6.7	0
0.54,6.8	0
0.54,6.9	0
0.54,7.0	0
0.54,7.1	0
0.54,7.2	0
0.54,7.3	0
0.54,7.4	0
0.54,7.5	0

0.54,7.6	0
0.54,7.7	0
0.54,7.8	0
0.54,7.9	0
0.54,8.0	0
0.54,8.1	0
0.54,8.2	0
0.54,8.3	0
0.54,8.4	0
0.54,8.5	0
0.54,8.6	0
0.54,8.7	0
0.54,8.8	0
0.54,8.9	0
0.54,9.0	0
0.54,9.1	0
0.54,9.2	0
0.54,9.3	0
0.54,9.4	0
0.54,9.5	0
0.54,9.6	0
0.54,9.7	0
0.54,9.8	0
0.54,9.9	0
0.54,10.0	0
0.54,10.1	0
0.54,10.2	0
0.54,10.3	0
0.54,10.4	0
0.54,10.5	0
0.54,10.6	0
0.54,10.7	0
0.54,10.8	0
0.54,10.9	0
0.54,11.0	0
0.54,11.1	0
0.54,11.2	0
0.54,11.3	0
0.54,11.4	0
0.54,11.5	0
0.54,11.6	0
0.54,11.7	0

0.54,11.8	0
0.54,11.9	0
0.54,12.0	0
0.54,12.1	0
0.54,12.2	0
0.54,12.3	0
0.54,12.4	0
0.54,12.5	0
0.54,12.6	0
0.54,12.7	0
0.54,12.8	0
0.54,12.9	0
0.54,13.0	0
0.54,13.1	0
0.54,13.2	0
0.54,13.3	0
0.54,13.4	0
0.54,13.5	0
0.54,13.6	0
0.54,13.7	0
0.54,13.8	0
0.54,13.9	0
0.54,14.0	0
0.54,14.1	0
0.54,14.2	0
0.54,14.3	0
0.54,14.4	0
0.54,14.5	0
0.54,14.6	0
0.54,14.7	0
0.54,14.8	0
0.54,14.9	0
0.54,15.0	0
0.54,15.1	0
0.54,15.2	0
0.54,15.3	0
0.54,15.4	0
0.54,15.5	0
0.54,15.6	0
0.54,15.7	0
0.54,15.8	0
0.54,15.9	0

0.54,16.0	0
0.54,16.1	0
0.54,16.2	0
0.54,16.3	0
0.54,16.4	0
0.54,16.5	0
0.54,16.6	0
0.54,16.7	0
0.54,16.8	0
0.54,16.9	0
0.54,17.0	0
0.54,17.1	0
0.54,17.2	0
0.54,17.3	0
0.54,17.4	0
0.54,17.5	0
0.54,17.6	0
0.54,17.7	0
0.54,17.8	0
0.54,17.9	0
0.54,18.0	0
0.54,18.1	0
0.54,18.2	0
0.54,18.3	0
0.54,18.4	0
0.54,18.5	0
0.54,18.6	0
0.54,18.7	0
0.54,18.8	0
0.54,18.9	0
0.54,19.0	0
0.54,19.1	0
0.54,19.2	0
0.54,19.3	0
0.54,19.4	0
0.54,19.5	0
0.54,19.6	0
0.54,19.7	0
0.54,19.8	0
0.54,19.9	0
0.54,20.0	0
0.54,20.1	0

0.54,20.2	0
0.54,20.3	0
0.54,20.4	0
0.54,20.5	0
0.54,20.6	0
0.54,20.7	0
0.54,20.8	0
0.54,20.9	0
0.54,21.0	0
0.54,21.1	0
0.54,21.2	0
0.54,21.3	0
0.54,21.4	0
0.54,21.5	0
0.54,21.6	0
0.54,21.7	0
0.54,21.8	0
0.54,21.9	0
0.54,22.0	0
0.54,22.1	0
0.54,22.2	0
0.54,22.3	0
0.54,22.4	0
0.54,22.5	0
0.54,22.6	0
0.54,22.7	0
0.54,22.8	0
0.54,22.9	0
0.54,23.0	0
0.54,23.1	0
0.54,23.2	0
0.54,23.3	0
0.54,23.4	0
0.54,23.5	0
0.54,23.6	0
0.54,23.7	0
0.54,23.8	0
0.54,23.9	0
0.54,24.0	0
0.54,24.1	0
0.54,24.2	0
0.54,24.3	0

0.54,24.4	0
0.54,24.5	0
0.54,24.6	0
0.54,24.7	0
0.54,24.8	0
0.54,24.9	0
0.54,25.0	0
0.54,25.1	0
0.54,25.2	0
0.54,25.3	0
0.54,25.4	0
0.54,25.5	0
0.54,25.6	0
0.54,25.7	0
0.54,25.8	0
0.54,25.9	0
0.54,26.0	0
0.54,26.1	0
0.54,26.2	0
0.54,26.3	0
0.54,26.4	0
0.54,26.5	0
0.54,26.6	0
0.54,26.7	0
0.54,26.8	0
0.54,26.9	0
0.54,27.0	0
0.54,27.1	0
0.54,27.2	0
0.54,27.3	0
0.54,27.4	0
0.54,27.5	0
0.54,27.6	0
0.54,27.7	0
0.54,27.8	0
0.54,27.9	0
0.54,28.0	0
0.54,28.1	0
0.54,28.2	0
0.54,28.3	0
0.54,28.4	0
0.54,28.5	0

0.54,28.6	0
0.54,28.7	0
0.54,28.8	0
0.54,28.9	0
0.54,29.0	0
0.54,29.1	0
0.54,29.2	0
0.54,29.3	0
0.54,29.4	0
0.54,29.5	0
0.54,29.6	0
0.54,29.7	0
0.54,29.8	0
0.54,29.9	0
0.54,30.0	0
0.54,30.1	0
0.54,30.2	0
0.54,30.3	0
0.54,30.4	0
0.54,30.5	0
0.54,30.6	0
0.54,30.7	0
0.54,30.8	0
0.54,30.9	0
0.54,31.0	0
0.54,31.1	0
0.54,31.2	0
0.54,31.3	0
0.54,31.4	0
0.54,31.5	0
0.54,31.6	0
0.54,31.7	0
0.54,31.8	0
0.54,31.9	0
0.54,32.0	0
0.54,32.1	0
0.54,32.2	0
0.54,32.3	0
0.54,32.4	0
0.54,32.5	0
0.54,32.6	0
0.54,32.7	0

0.54,32.8	0
0.54,32.9	0
0.54,33.0	0
0.54,33.1	0
0.54,33.2	0
0.54,33.3	0
0.54,33.4	0
0.54,33.5	0
0.54,33.6	0
0.54,33.7	0
0.54,33.8	0
0.54,33.9	0
0.54,34.0	0
0.54,34.1	0
0.54,34.2	0
0.54,34.3	0
0.54,34.4	0
0.54,34.5	0
0.54,34.6	0
0.54,34.7	0
0.54,34.8	0
0.54,34.9	0
0.54,35.0	0
0.54,35.1	0
0.54,35.2	0
0.54,35.3	0
0.54,35.4	0
0.54,35.5	0
0.54,35.6	0
0.54,35.7	0
0.54,35.8	0
0.54,35.9	0
0.54,36.0	0
0.54,36.1	0
0.54,36.2	0
0.54,36.3	0
0.54,36.4	0
0.54,36.5	0
0.54,36.6	0
0.54,36.7	0
0.54,36.8	0
0.54,36.9	0

0.54,37.0	0
0.54,37.1	0
0.54,37.2	0
0.54,37.3	0
0.54,37.4	0
0.54,37.5	0
0.54,37.6	0
0.54,37.7	0
0.54,37.8	0
0.54,37.9	0
0.54,38.0	0
0.54,38.1	0
0.54,38.2	0
0.54,38.3	0
0.54,38.4	0
0.54,38.5	0
0.54,38.6	0
0.54,38.7	0
0.54,38.8	0
0.54,38.9	0
0.54,39.0	0
0.54,39.1	0
0.54,39.2	0
0.54,39.3	0
0.54,39.4	0
0.54,39.5	0
0.54,39.6	0
0.54,39.7	0
0.54,39.8	0
0.54,39.9	0
0.54,40.0	0
0.54,40.1	0
0.54,40.2	0
0.54,40.3	0
0.54,40.4	0
0.54,40.5	0
0.54,40.6	0
0.54,40.7	0
0.54,40.8	0
0.54,40.9	0
0.54,41.0	0
0.54,41.1	0

0.54,41.2	0
0.54,41.3	0
0.54,41.4	0
0.54,41.5	0
0.54,41.6	0
0.54,41.7	0
0.54,41.8	0
0.54,41.9	0
0.54,42.0	0
0.54,42.1	0
0.54,42.2	0
0.54,42.3	0
0.54,42.4	0
0.54,42.5	0
0.54,42.6	0
0.54,42.7	0
0.54,42.8	0
0.54,42.9	0
0.54,43.0	0
0.54,43.1	0
0.54,43.2	0
0.54,43.3	0
0.54,43.4	0
0.54,43.5	0
0.54,43.6	0
0.54,43.7	0
0.54,43.8	0
0.54,43.9	0
0.54,44.0	0
0.54,44.1	0
0.54,44.2	0
0.54,44.3	0
0.54,44.4	0
0.54,44.5	0
0.54,44.6	0
0.54,44.7	0
0.54,44.8	0
0.54,44.9	0
0.54,45.0	0
0.54,45.1	0
0.54,45.2	0
0.54,45.3	0

0.54,45.4	0
0.54,45.5	0
0.54,45.6	0
0.54,45.7	0
0.54,45.8	0
0.54,45.9	0
0.54,46.0	0
0.54,46.1	0
0.54,46.2	0
0.54,46.3	0
0.54,46.4	0
0.54,46.5	0
0.54,46.6	0
0.54,46.7	0
0.54,46.8	0
0.54,46.9	0
0.54,47.0	0
0.54,47.1	0
0.54,47.2	0
0.54,47.3	0
0.54,47.4	0
0.54,47.5	0
0.54,47.6	0
0.54,47.7	0
0.54,47.8	0
0.54,47.9	0
0.54,48.0	0
0.54,48.1	0
0.54,48.2	0
0.54,48.3	0
0.54,48.4	0
0.54,48.5	0
0.54,48.6	0
0.54,48.7	0
0.54,48.8	0
0.54,48.9	0
0.54,49.0	0
0.54,49.1	0
0.54,49.2	0
0.54,49.3	0
0.54,49.4	0
0.54,49.5	0

0.54,49.6	0
0.54,49.7	0
0.54,49.8	0
0.54,49.9	0
0.54,50.0	0
0.54,50.1	0
0.54,50.2	0
0.54,50.3	0
0.54,50.4	0
0.54,50.5	0
0.54,50.6	0
0.54,50.7	0
0.54,50.8	0
0.54,50.9	0
0.54,51.0	0
0.54,51.1	0
0.54,51.2	0
0.54,51.3	0
0.54,51.4	0
0.54,51.5	0
0.54,51.6	0
0.54,51.7	0
0.54,51.8	0
0.54,51.9	0
0.54,52.0	0
0.54,52.1	0
0.54,52.2	0
0.54,52.3	0
0.54,52.4	0
0.54,52.5	0
0.54,52.6	0
0.54,52.7	0
0.54,52.8	0
0.54,52.9	0
0.54,53.0	0
0.54,53.1	0
0.54,53.2	0
0.54,53.3	0
0.54,53.4	0
0.54,53.5	0
0.54,53.6	0
0.54,53.7	0

0.54,53.8	0
0.54,53.9	0
0.54,54.0	0
0.54,54.1	0
0.54,54.2	0
0.54,54.3	0
0.54,54.4	0
0.54,54.5	0
0.54,54.6	0
0.54,54.7	0
0.54,54.8	0
0.54,54.9	0
0.54,55.0	0
0.54,55.1	0
0.54,55.2	0
0.54,55.3	0
0.54,55.4	0
0.54,55.5	0
0.54,55.6	0
0.54,55.7	0
0.54,55.8	0
0.54,55.9	0
0.54,56.0	0
0.54,56.1	0
0.54,56.2	0
0.54,56.3	0
0.54,56.4	0
0.54,56.5	0
0.54,56.6	0
0.54,56.7	0
0.54,56.8	0
0.54,56.9	0
0.54,57.0	0
0.54,57.1	0
0.54,57.2	0
0.54,57.3	0
0.54,57.4	0
0.54,57.5	0
0.54,57.6	0
0.54,57.7	0
0.54,57.8	0
0.54,57.9	0

0.54,58.0	0
0.54,58.1	0
0.54,58.2	0
0.54,58.3	0
0.54,58.4	0
0.54,58.5	0
0.54,58.6	0
0.54,58.7	0
0.54,58.8	0
0.54,58.9	0
0.54,59.0	0
0.54,59.1	0
0.54,59.2	0
0.54,59.3	0
0.54,59.4	0
0.54,59.5	0
0.54,59.6	0
0.54,59.7	0
0.54,59.8	0
0.54,59.9	0
0.54,60.0	0
0.54,60.1	0
0.54,60.2	0
0.54,60.3	0
0.54,60.4	0
0.54,60.5	0
0.54,60.6	0
0.54,60.7	0
0.54,60.8	0
0.54,60.9	0
0.54,61.0	0
0.54,61.1	0
0.54,61.2	0
0.54,61.3	0
0.54,61.4	0
0.54,61.5	0
0.54,61.6	0
0.54,61.7	0
0.54,61.8	0
0.54,61.9	0
0.54,62.0	0
0.54,62.1	0

0.54,62.2	0
0.54,62.3	0
0.54,62.4	0
0.54,62.5	0
0.54,62.6	0
0.54,62.7	0
0.54,62.8	0
0.54,62.9	0
0.54,63.0	0
0.54,63.1	0
0.54,63.2	0
0.54,63.3	0
0.54,63.4	0
0.54,63.5	0
0.54,63.6	0
0.54,63.7	0
0.54,63.8	0
0.54,63.9	0
0.54,64.0	0
0.54,64.1	0
0.54,64.2	0
0.54,64.3	0
0.54,64.4	0
0.54,64.5	0
0.54,64.6	0
0.54,64.7	0
0.54,64.8	0
0.54,64.9	0
0.54,65.0	0
0.54,65.1	0
0.54,65.2	0
0.54,65.3	0
0.54,65.4	0
0.54,65.5	0
0.54,65.6	0
0.54,65.7	0
0.54,65.8	0
0.54,65.9	0
0.54,66.0	0
0.54,66.1	0
0.54,66.2	0
0.54,66.3	0

0.54,66.4	0
0.54,66.5	0
0.54,66.6	0
0.54,66.7	0
0.54,66.8	0
0.54,66.9	0
0.54,67.0	0
0.54,67.1	0
0.54,67.2	0
0.54,67.3	0
0.54,67.4	0
0.54,67.5	0
0.54,67.6	0
0.54,67.7	0
0.54,67.8	0
0.54,67.9	0
0.54,68.0	0
0.54,68.1	0
0.54,68.2	0
0.54,68.3	0
0.54,68.4	0
0.54,68.5	0
0.54,68.6	0
0.54,68.7	0
0.54,68.8	0
0.54,68.9	0
0.54,69.0	0
0.54,69.1	0
0.54,69.2	0
0.54,69.3	0
0.54,69.4	0
0.54,69.5	0
0.54,69.6	0
0.54,69.7	0
0.54,69.8	0
0.54,69.9	0
0.54,70.0	0
0.54,70.1	0
0.54,70.2	0
0.54,70.3	0
0.54,70.4	0
0.54,70.5	0

0.54,70.6	0
0.54,70.7	0
0.54,70.8	0
0.54,70.9	0
0.54,71.0	0
0.54,71.1	0
0.54,71.2	0
0.54,71.3	0
0.54,71.4	0
0.54,71.5	0
0.54,71.6	0
0.54,71.7	0
0.54,71.8	0
0.54,71.9	0
0.54,72.0	0
0.54,72.1	0
0.54,72.2	0
0.54,72.3	0
0.54,72.4	0
0.54,72.5	0
0.54,72.6	0
0.54,72.7	0
0.54,72.8	0
0.54,72.9	0
0.54,73.0	0
0.54,73.1	0
0.54,73.2	0
0.54,73.3	0
0.54,73.4	0
0.54,73.5	0
0.54,73.6	0
0.54,73.7	0
0.54,73.8	0
0.54,73.9	0
0.54,74.0	0
0.54,74.1	0
0.54,74.2	0
0.54,74.3	0
0.54,74.4	0
0.54,74.5	0
0.54,74.6	0
0.54,74.7	0

0.54,74.8	0
0.54,74.9	0
0.54,75.0	0
0.54,75.1	0
0.54,75.2	0
0.54,75.3	0
0.54,75.4	0
0.54,75.5	0
0.54,75.6	0
0.54,75.7	0
0.54,75.8	0
0.54,75.9	0
0.54,76.0	0
0.54,76.1	0
0.54,76.2	0
0.54,76.3	0
0.54,76.4	0
0.54,76.5	0
0.54,76.6	0
0.54,76.7	0
0.54,76.8	0
0.54,76.9	0
0.54,77.0	0
0.54,77.1	0
0.54,77.2	0
0.54,77.3	0
0.54,77.4	0
0.54,77.5	0
0.54,77.6	0
0.54,77.7	0
0.54,77.8	0
0.54,77.9	0
0.54,78.0	0
0.54,78.1	0
0.54,78.2	0
0.54,78.3	0
0.54,78.4	0
0.54,78.5	0
0.54,78.6	0
0.54,78.7	0
0.54,78.8	0
0.54,78.9	0

0.54,79.0	0
0.54,79.1	0
0.54,79.2	0
0.54,79.3	0
0.54,79.4	0
0.54,79.5	0
0.54,79.6	0
0.54,79.7	0
0.54,79.8	0
0.54,79.9	0
0.54,80.0	0
0.54,80.1	0
0.54,80.2	0
0.54,80.3	0
0.54,80.4	0
0.54,80.5	0
0.54,80.6	0
0.54,80.7	0
0.54,80.8	0
0.54,80.9	0
0.54,81.0	0
0.54,81.1	0
0.54,81.2	0
0.54,81.3	0
0.54,81.4	0
0.54,81.5	0
0.54,81.6	0
0.54,81.7	0
0.54,81.8	0
0.54,81.9	0
0.54,82.0	0
0.54,82.1	0
0.54,82.2	0
0.54,82.3	0
0.54,82.4	0
0.54,82.5	0
0.54,82.6	0
0.54,82.7	0
0.54,82.8	0
0.54,82.9	0
0.54,83.0	0
0.54,83.1	0

0.54,83.2	0
0.54,83.3	0
0.54,83.4	0
0.54,83.5	0
0.54,83.6	0
0.54,83.7	0
0.54,83.8	0
0.54,83.9	0
0.54,84.0	0
0.54,84.1	0
0.54,84.2	0
0.54,84.3	0
0.54,84.4	0
0.54,84.5	0.000001
0.54,84.6	0.000001
0.54,84.7	0.000001
0.54,84.8	0.000001
0.54,84.9	0.000001
0.54,85.0	0.000001
0.54,85.1	0.000001
0.54,85.2	0.000001
0.54,85.3	0.000001
0.54,85.4	0.000001
0.54,85.5	0.000002
0.54,85.6	0.000002
0.54,85.7	0.000002
0.54,85.8	0.000002
0.54,85.9	0.000002
0.54,86.0	0.000002
0.54,86.1	0.000002
0.54,86.2	0.000002
0.54,86.3	0.000002
0.54,86.4	0.000002
0.54,86.5	0.000002
0.54,86.6	0.000002
0.54,86.7	0.000002
0.54,86.8	0.000002
0.54,86.9	0.000002
0.54,87.0	0.000002
0.54,87.1	0.000003
0.54,87.2	0.000003
0.54,87.3	0.000003

0.54,87.4	0.000003
0.54,87.5	0.000003
0.54,87.6	0.000003
0.54,87.7	0.000003
0.54,87.8	0.000003
0.54,87.9	0.000003
0.54,88.0	0.000003
0.54,88.1	0.000003
0.54,88.2	0.000003
0.54,88.3	0.000003
0.54,88.4	0.000003
0.54,88.5	0.000003
0.54,88.6	0.000003
0.54,88.7	0.000003
0.54,88.8	0.000003
0.54,88.9	0.000003
0.54,89.0	0.000003
0.54,89.1	0.000003
0.54,89.2	0.000003
0.54,89.3	0.000003
0.54,89.4	0.000003
0.54,89.5	0.000003
0.54,89.6	0.000003
0.54,89.7	0.000004
0.54,89.8	0.000004
0.54,89.9	0.000004
0.54,90.0	0.000004
0.54,90.1	0.000004
0.54,90.2	0.000004
0.54,90.3	0.000004
0.54,90.4	0.000004
0.54,90.5	0.000004
0.54,90.6	0.000004
0.54,90.7	0.000004
0.54,90.8	0.000004
0.54,90.9	0.000004
0.54,91.0	0.000004
0.54,91.1	0.000004
0.54,91.2	0.000004
0.54,91.3	0.000004
0.54,91.4	0.000004
0.54,91.5	0.000004

0.54,91.6	0.000004
0.54,91.7	0.000004
0.54,91.8	0.000004
0.54,91.9	0.000004
0.54,92.0	0.000004
0.54,92.1	0.000004
0.54,92.2	0.000004
0.54,92.3	0.000005
0.54,92.4	0.000005
0.54,92.5	0.000005
0.54,92.6	0.000005
0.54,92.7	0.000005
0.54,92.8	0.000005
0.54,92.9	0.000006
0.54,93.0	0.000006
0.54,93.1	0.000006
0.54,93.2	0.000006
0.54,93.3	0.000006
0.54,93.4	0.000006
0.54,93.5	0.000006
0.54,93.6	0.000006
0.54,93.7	0.000006
0.54,93.8	0.000006
0.54,93.9	0.000006
0.54,94.0	0.000006
0.54,94.1	0.000006
0.54,94.2	0.000006
0.54,94.3	0.000006
0.54,94.4	0.000006
0.54,94.5	0.000006
0.54,94.6	0.000006
0.54,94.7	0.000006
0.54,94.8	0.000006
0.54,94.9	0.000006
0.54,95.0	0.000006
0.54,95.1	0.000006
0.54,95.2	0.000007
0.54,95.3	0.000007
0.54,95.4	0.000007
0.54,95.5	0.000007
0.54,95.6	0.000007
0.54,95.7	0.000007

0.54,95.8	0.000007
0.54,95.9	0.000007
0.54,96.0	0.000007
0.54,96.1	0.000007
0.54,96.2	0.000007
0.54,96.3	0.000007
0.54,96.4	0.000007
0.54,96.5	0.000007
0.54,96.6	0.000007
0.54,96.7	0.000007
0.54,96.8	0.000007
0.54,96.9	0.000008
0.54,97.0	0.000009
0.54,97.1	0.000009
0.54,97.2	0.000009
0.54,97.3	0.000009
0.54,97.4	0.000009
0.54,97.5	0.00001
0.54,97.6	0.000011
0.54,97.7	0.000011
0.54,97.8	0.000011
0.54,97.9	0.000008
0.54,98.0	0.000008
0.54,98.1	0.000008
0.54,98.2	0.000008
0.54,98.3	0.000008
0.54,98.4	0.000008
0.54,98.5	0.000008
0.54,98.6	0.000008
0.54,98.7	0.000004
0.54,98.8	0.000004
0.54,98.9	0.000004
0.54,99.0	0.000004
0.54,99.1	0.000004
0.54,99.2	0.000004
0.54,99.3	0.000004
0.54,99.4	0.000004
0.54,99.5	0.000004
0.54,99.6	0
0.54,99.7	0
0.54,99.8	0
0.54,99.9	0

0.54,100.0	0
0.55,0.0	0
0.55,0.1	0
0.55,0.2	0
0.55,0.3	0
0.55,0.4	0
0.55,0.5	0
0.55,0.6	0
0.55,0.7	0
0.55,0.8	0
0.55,0.9	0
0.55,1.0	0
0.55,1.1	0
0.55,1.2	0
0.55,1.3	0
0.55,1.4	0
0.55,1.5	0
0.55,1.6	0
0.55,1.7	0
0.55,1.8	0
0.55,1.9	0
0.55,2.0	0
0.55,2.1	0
0.55,2.2	0
0.55,2.3	0
0.55,2.4	0
0.55,2.5	0
0.55,2.6	0
0.55,2.7	0
0.55,2.8	0
0.55,2.9	0
0.55,3.0	0
0.55,3.1	0
0.55,3.2	0
0.55,3.3	0
0.55,3.4	0
0.55,3.5	0
0.55,3.6	0
0.55,3.7	0
0.55,3.8	0
0.55,3.9	0
0.55,4.0	0

0.55,4.1	0
0.55,4.2	0
0.55,4.3	0
0.55,4.4	0
0.55,4.5	0
0.55,4.6	0
0.55,4.7	0
0.55,4.8	0
0.55,4.9	0
0.55,5.0	0
0.55,5.1	0
0.55,5.2	0
0.55,5.3	0
0.55,5.4	0
0.55,5.5	0
0.55,5.6	0
0.55,5.7	0
0.55,5.8	0
0.55,5.9	0
0.55,6.0	0
0.55,6.1	0
0.55,6.2	0
0.55,6.3	0
0.55,6.4	0
0.55,6.5	0
0.55,6.6	0
0.55,6.7	0
0.55,6.8	0
0.55,6.9	0
0.55,7.0	0
0.55,7.1	0
0.55,7.2	0
0.55,7.3	0
0.55,7.4	0
0.55,7.5	0
0.55,7.6	0
0.55,7.7	0
0.55,7.8	0
0.55,7.9	0
0.55,8.0	0
0.55,8.1	0
0.55,8.2	0

0.55,8.3	0
0.55,8.4	0
0.55,8.5	0
0.55,8.6	0
0.55,8.7	0
0.55,8.8	0
0.55,8.9	0
0.55,9.0	0
0.55,9.1	0
0.55,9.2	0
0.55,9.3	0
0.55,9.4	0
0.55,9.5	0
0.55,9.6	0
0.55,9.7	0
0.55,9.8	0
0.55,9.9	0
0.55,10.0	0
0.55,10.1	0
0.55,10.2	0
0.55,10.3	0
0.55,10.4	0
0.55,10.5	0
0.55,10.6	0
0.55,10.7	0
0.55,10.8	0
0.55,10.9	0
0.55,11.0	0
0.55,11.1	0
0.55,11.2	0
0.55,11.3	0
0.55,11.4	0
0.55,11.5	0
0.55,11.6	0
0.55,11.7	0
0.55,11.8	0
0.55,11.9	0
0.55,12.0	0
0.55,12.1	0
0.55,12.2	0
0.55,12.3	0
0.55,12.4	0

0.55,12.5	0
0.55,12.6	0
0.55,12.7	0
0.55,12.8	0
0.55,12.9	0
0.55,13.0	0
0.55,13.1	0
0.55,13.2	0
0.55,13.3	0
0.55,13.4	0
0.55,13.5	0
0.55,13.6	0
0.55,13.7	0
0.55,13.8	0
0.55,13.9	0
0.55,14.0	0
0.55,14.1	0
0.55,14.2	0
0.55,14.3	0
0.55,14.4	0
0.55,14.5	0
0.55,14.6	0
0.55,14.7	0
0.55,14.8	0
0.55,14.9	0
0.55,15.0	0
0.55,15.1	0
0.55,15.2	0
0.55,15.3	0
0.55,15.4	0
0.55,15.5	0
0.55,15.6	0
0.55,15.7	0
0.55,15.8	0
0.55,15.9	0
0.55,16.0	0
0.55,16.1	0
0.55,16.2	0
0.55,16.3	0
0.55,16.4	0
0.55,16.5	0
0.55,16.6	0

0.55,16.7	0
0.55,16.8	0
0.55,16.9	0
0.55,17.0	0
0.55,17.1	0
0.55,17.2	0
0.55,17.3	0
0.55,17.4	0
0.55,17.5	0
0.55,17.6	0
0.55,17.7	0
0.55,17.8	0
0.55,17.9	0
0.55,18.0	0
0.55,18.1	0
0.55,18.2	0
0.55,18.3	0
0.55,18.4	0
0.55,18.5	0
0.55,18.6	0
0.55,18.7	0
0.55,18.8	0
0.55,18.9	0
0.55,19.0	0
0.55,19.1	0
0.55,19.2	0
0.55,19.3	0
0.55,19.4	0
0.55,19.5	0
0.55,19.6	0
0.55,19.7	0
0.55,19.8	0
0.55,19.9	0
0.55,20.0	0
0.55,20.1	0
0.55,20.2	0
0.55,20.3	0
0.55,20.4	0
0.55,20.5	0
0.55,20.6	0
0.55,20.7	0
0.55,20.8	0

0.55,20.9	0
0.55,21.0	0
0.55,21.1	0
0.55,21.2	0
0.55,21.3	0
0.55,21.4	0
0.55,21.5	0
0.55,21.6	0
0.55,21.7	0
0.55,21.8	0
0.55,21.9	0
0.55,22.0	0
0.55,22.1	0
0.55,22.2	0
0.55,22.3	0
0.55,22.4	0
0.55,22.5	0
0.55,22.6	0
0.55,22.7	0
0.55,22.8	0
0.55,22.9	0
0.55,23.0	0
0.55,23.1	0
0.55,23.2	0
0.55,23.3	0
0.55,23.4	0
0.55,23.5	0
0.55,23.6	0
0.55,23.7	0
0.55,23.8	0
0.55,23.9	0
0.55,24.0	0
0.55,24.1	0
0.55,24.2	0
0.55,24.3	0
0.55,24.4	0
0.55,24.5	0
0.55,24.6	0
0.55,24.7	0
0.55,24.8	0
0.55,24.9	0
0.55,25.0	0

0.55,25.1	0
0.55,25.2	0
0.55,25.3	0
0.55,25.4	0
0.55,25.5	0
0.55,25.6	0
0.55,25.7	0
0.55,25.8	0
0.55,25.9	0
0.55,26.0	0
0.55,26.1	0
0.55,26.2	0
0.55,26.3	0
0.55,26.4	0
0.55,26.5	0
0.55,26.6	0
0.55,26.7	0
0.55,26.8	0
0.55,26.9	0
0.55,27.0	0
0.55,27.1	0
0.55,27.2	0
0.55,27.3	0
0.55,27.4	0
0.55,27.5	0
0.55,27.6	0
0.55,27.7	0
0.55,27.8	0
0.55,27.9	0
0.55,28.0	0
0.55,28.1	0
0.55,28.2	0
0.55,28.3	0
0.55,28.4	0
0.55,28.5	0
0.55,28.6	0
0.55,28.7	0
0.55,28.8	0
0.55,28.9	0
0.55,29.0	0
0.55,29.1	0
0.55,29.2	0

0.55,29.3	0
0.55,29.4	0
0.55,29.5	0
0.55,29.6	0
0.55,29.7	0
0.55,29.8	0
0.55,29.9	0
0.55,30.0	0
0.55,30.1	0
0.55,30.2	0
0.55,30.3	0
0.55,30.4	0
0.55,30.5	0
0.55,30.6	0
0.55,30.7	0
0.55,30.8	0
0.55,30.9	0
0.55,31.0	0
0.55,31.1	0
0.55,31.2	0
0.55,31.3	0
0.55,31.4	0
0.55,31.5	0
0.55,31.6	0
0.55,31.7	0
0.55,31.8	0
0.55,31.9	0
0.55,32.0	0
0.55,32.1	0
0.55,32.2	0
0.55,32.3	0
0.55,32.4	0
0.55,32.5	0
0.55,32.6	0
0.55,32.7	0
0.55,32.8	0
0.55,32.9	0
0.55,33.0	0
0.55,33.1	0
0.55,33.2	0
0.55,33.3	0
0.55,33.4	0

0.55,33.5	0
0.55,33.6	0
0.55,33.7	0
0.55,33.8	0
0.55,33.9	0
0.55,34.0	0
0.55,34.1	0
0.55,34.2	0
0.55,34.3	0
0.55,34.4	0
0.55,34.5	0
0.55,34.6	0
0.55,34.7	0
0.55,34.8	0
0.55,34.9	0
0.55,35.0	0
0.55,35.1	0
0.55,35.2	0
0.55,35.3	0
0.55,35.4	0
0.55,35.5	0
0.55,35.6	0
0.55,35.7	0
0.55,35.8	0
0.55,35.9	0
0.55,36.0	0
0.55,36.1	0
0.55,36.2	0
0.55,36.3	0
0.55,36.4	0
0.55,36.5	0
0.55,36.6	0
0.55,36.7	0
0.55,36.8	0
0.55,36.9	0
0.55,37.0	0
0.55,37.1	0
0.55,37.2	0
0.55,37.3	0
0.55,37.4	0
0.55,37.5	0
0.55,37.6	0

0.55,37.7	0
0.55,37.8	0
0.55,37.9	0
0.55,38.0	0
0.55,38.1	0
0.55,38.2	0
0.55,38.3	0
0.55,38.4	0
0.55,38.5	0
0.55,38.6	0
0.55,38.7	0
0.55,38.8	0
0.55,38.9	0
0.55,39.0	0
0.55,39.1	0
0.55,39.2	0
0.55,39.3	0
0.55,39.4	0
0.55,39.5	0
0.55,39.6	0
0.55,39.7	0
0.55,39.8	0
0.55,39.9	0
0.55,40.0	0
0.55,40.1	0
0.55,40.2	0
0.55,40.3	0
0.55,40.4	0
0.55,40.5	0
0.55,40.6	0
0.55,40.7	0
0.55,40.8	0
0.55,40.9	0
0.55,41.0	0
0.55,41.1	0
0.55,41.2	0
0.55,41.3	0
0.55,41.4	0
0.55,41.5	0
0.55,41.6	0
0.55,41.7	0
0.55,41.8	0

0.55,41.9	0
0.55,42.0	0
0.55,42.1	0
0.55,42.2	0
0.55,42.3	0
0.55,42.4	0
0.55,42.5	0
0.55,42.6	0
0.55,42.7	0
0.55,42.8	0
0.55,42.9	0
0.55,43.0	0
0.55,43.1	0
0.55,43.2	0
0.55,43.3	0
0.55,43.4	0
0.55,43.5	0
0.55,43.6	0
0.55,43.7	0
0.55,43.8	0
0.55,43.9	0
0.55,44.0	0
0.55,44.1	0
0.55,44.2	0
0.55,44.3	0
0.55,44.4	0
0.55,44.5	0
0.55,44.6	0
0.55,44.7	0
0.55,44.8	0
0.55,44.9	0
0.55,45.0	0
0.55,45.1	0
0.55,45.2	0
0.55,45.3	0
0.55,45.4	0
0.55,45.5	0
0.55,45.6	0
0.55,45.7	0
0.55,45.8	0
0.55,45.9	0
0.55,46.0	0

0.55,46.1	0
0.55,46.2	0
0.55,46.3	0
0.55,46.4	0
0.55,46.5	0
0.55,46.6	0
0.55,46.7	0
0.55,46.8	0
0.55,46.9	0
0.55,47.0	0
0.55,47.1	0
0.55,47.2	0
0.55,47.3	0
0.55,47.4	0
0.55,47.5	0
0.55,47.6	0
0.55,47.7	0
0.55,47.8	0
0.55,47.9	0
0.55,48.0	0
0.55,48.1	0
0.55,48.2	0
0.55,48.3	0
0.55,48.4	0
0.55,48.5	0
0.55,48.6	0
0.55,48.7	0
0.55,48.8	0
0.55,48.9	0
0.55,49.0	0
0.55,49.1	0
0.55,49.2	0
0.55,49.3	0
0.55,49.4	0
0.55,49.5	0
0.55,49.6	0
0.55,49.7	0
0.55,49.8	0
0.55,49.9	0
0.55,50.0	0
0.55,50.1	0
0.55,50.2	0

0.55,50.3	0
0.55,50.4	0
0.55,50.5	0
0.55,50.6	0
0.55,50.7	0
0.55,50.8	0
0.55,50.9	0
0.55,51.0	0
0.55,51.1	0
0.55,51.2	0
0.55,51.3	0
0.55,51.4	0
0.55,51.5	0
0.55,51.6	0
0.55,51.7	0
0.55,51.8	0
0.55,51.9	0
0.55,52.0	0
0.55,52.1	0
0.55,52.2	0
0.55,52.3	0
0.55,52.4	0
0.55,52.5	0
0.55,52.6	0
0.55,52.7	0
0.55,52.8	0
0.55,52.9	0
0.55,53.0	0
0.55,53.1	0
0.55,53.2	0
0.55,53.3	0
0.55,53.4	0
0.55,53.5	0
0.55,53.6	0
0.55,53.7	0
0.55,53.8	0
0.55,53.9	0
0.55,54.0	0
0.55,54.1	0
0.55,54.2	0
0.55,54.3	0
0.55,54.4	0

0.55,54.5	0
0.55,54.6	0
0.55,54.7	0
0.55,54.8	0
0.55,54.9	0
0.55,55.0	0
0.55,55.1	0
0.55,55.2	0
0.55,55.3	0
0.55,55.4	0
0.55,55.5	0
0.55,55.6	0
0.55,55.7	0
0.55,55.8	0
0.55,55.9	0
0.55,56.0	0
0.55,56.1	0
0.55,56.2	0
0.55,56.3	0
0.55,56.4	0
0.55,56.5	0
0.55,56.6	0
0.55,56.7	0
0.55,56.8	0
0.55,56.9	0
0.55,57.0	0
0.55,57.1	0
0.55,57.2	0
0.55,57.3	0
0.55,57.4	0
0.55,57.5	0
0.55,57.6	0
0.55,57.7	0
0.55,57.8	0
0.55,57.9	0
0.55,58.0	0
0.55,58.1	0
0.55,58.2	0
0.55,58.3	0
0.55,58.4	0
0.55,58.5	0
0.55,58.6	0

0.55,58.7	0
0.55,58.8	0
0.55,58.9	0
0.55,59.0	0
0.55,59.1	0
0.55,59.2	0
0.55,59.3	0
0.55,59.4	0
0.55,59.5	0
0.55,59.6	0
0.55,59.7	0
0.55,59.8	0
0.55,59.9	0
0.55,60.0	0
0.55,60.1	0
0.55,60.2	0
0.55,60.3	0
0.55,60.4	0
0.55,60.5	0
0.55,60.6	0
0.55,60.7	0
0.55,60.8	0
0.55,60.9	0
0.55,61.0	0
0.55,61.1	0
0.55,61.2	0
0.55,61.3	0
0.55,61.4	0
0.55,61.5	0
0.55,61.6	0
0.55,61.7	0
0.55,61.8	0
0.55,61.9	0
0.55,62.0	0
0.55,62.1	0
0.55,62.2	0
0.55,62.3	0
0.55,62.4	0
0.55,62.5	0
0.55,62.6	0
0.55,62.7	0
0.55,62.8	0

0.55,62.9	0
0.55,63.0	0
0.55,63.1	0
0.55,63.2	0
0.55,63.3	0
0.55,63.4	0
0.55,63.5	0
0.55,63.6	0
0.55,63.7	0
0.55,63.8	0
0.55,63.9	0
0.55,64.0	0
0.55,64.1	0
0.55,64.2	0
0.55,64.3	0
0.55,64.4	0
0.55,64.5	0
0.55,64.6	0
0.55,64.7	0
0.55,64.8	0
0.55,64.9	0
0.55,65.0	0
0.55,65.1	0
0.55,65.2	0
0.55,65.3	0
0.55,65.4	0
0.55,65.5	0
0.55,65.6	0
0.55,65.7	0
0.55,65.8	0
0.55,65.9	0
0.55,66.0	0
0.55,66.1	0
0.55,66.2	0
0.55,66.3	0
0.55,66.4	0
0.55,66.5	0
0.55,66.6	0
0.55,66.7	0
0.55,66.8	0
0.55,66.9	0
0.55,67.0	0

0.55,67.1	0
0.55,67.2	0
0.55,67.3	0
0.55,67.4	0
0.55,67.5	0
0.55,67.6	0
0.55,67.7	0
0.55,67.8	0
0.55,67.9	0
0.55,68.0	0
0.55,68.1	0
0.55,68.2	0
0.55,68.3	0
0.55,68.4	0
0.55,68.5	0
0.55,68.6	0
0.55,68.7	0
0.55,68.8	0
0.55,68.9	0
0.55,69.0	0
0.55,69.1	0
0.55,69.2	0
0.55,69.3	0
0.55,69.4	0
0.55,69.5	0
0.55,69.6	0
0.55,69.7	0
0.55,69.8	0
0.55,69.9	0
0.55,70.0	0
0.55,70.1	0
0.55,70.2	0
0.55,70.3	0
0.55,70.4	0
0.55,70.5	0
0.55,70.6	0
0.55,70.7	0
0.55,70.8	0
0.55,70.9	0
0.55,71.0	0
0.55,71.1	0
0.55,71.2	0

0.55,71.3	0
0.55,71.4	0
0.55,71.5	0
0.55,71.6	0
0.55,71.7	0
0.55,71.8	0
0.55,71.9	0
0.55,72.0	0
0.55,72.1	0
0.55,72.2	0
0.55,72.3	0
0.55,72.4	0
0.55,72.5	0
0.55,72.6	0
0.55,72.7	0
0.55,72.8	0
0.55,72.9	0
0.55,73.0	0
0.55,73.1	0
0.55,73.2	0
0.55,73.3	0
0.55,73.4	0
0.55,73.5	0
0.55,73.6	0
0.55,73.7	0
0.55,73.8	0
0.55,73.9	0
0.55,74.0	0
0.55,74.1	0
0.55,74.2	0
0.55,74.3	0
0.55,74.4	0
0.55,74.5	0
0.55,74.6	0
0.55,74.7	0
0.55,74.8	0
0.55,74.9	0
0.55,75.0	0
0.55,75.1	0
0.55,75.2	0
0.55,75.3	0
0.55,75.4	0

0.55,75.5	0
0.55,75.6	0
0.55,75.7	0
0.55,75.8	0
0.55,75.9	0
0.55,76.0	0
0.55,76.1	0
0.55,76.2	0
0.55,76.3	0
0.55,76.4	0
0.55,76.5	0
0.55,76.6	0
0.55,76.7	0
0.55,76.8	0
0.55,76.9	0
0.55,77.0	0
0.55,77.1	0
0.55,77.2	0
0.55,77.3	0
0.55,77.4	0
0.55,77.5	0
0.55,77.6	0
0.55,77.7	0
0.55,77.8	0
0.55,77.9	0
0.55,78.0	0
0.55,78.1	0
0.55,78.2	0
0.55,78.3	0
0.55,78.4	0
0.55,78.5	0
0.55,78.6	0
0.55,78.7	0
0.55,78.8	0
0.55,78.9	0
0.55,79.0	0
0.55,79.1	0
0.55,79.2	0
0.55,79.3	0
0.55,79.4	0
0.55,79.5	0
0.55,79.6	0

0.55,79.7	0
0.55,79.8	0
0.55,79.9	0
0.55,80.0	0
0.55,80.1	0
0.55,80.2	0
0.55,80.3	0.000001
0.55,80.4	0.000001
0.55,80.5	0.000001
0.55,80.6	0.000001
0.55,80.7	0.000001
0.55,80.8	0.000001
0.55,80.9	0.000001
0.55,81.0	0.000001
0.55,81.1	0.000001
0.55,81.2	0.000001
0.55,81.3	0.000001
0.55,81.4	0.000001
0.55,81.5	0.000002
0.55,81.6	0.000003
0.55,81.7	0.000003
0.55,81.8	0.000003
0.55,81.9	0.000003
0.55,82.0	0.000004
0.55,82.1	0.000004
0.55,82.2	0.000004
0.55,82.3	0.000005
0.55,82.4	0.000005
0.55,82.5	0.000005
0.55,82.6	0.000005
0.55,82.7	0.000005
0.55,82.8	0.000005
0.55,82.9	0.000006
0.55,83.0	0.000006
0.55,83.1	0.000006
0.55,83.2	0.000006
0.55,83.3	0.000006
0.55,83.4	0.000006
0.55,83.5	0.000006
0.55,83.6	0.000006
0.55,83.7	0.000006
0.55,83.8	0.000006

0.55,83.9	0.000006
0.55,84.0	0.000007
0.55,84.1	0.000007
0.55,84.2	0.000007
0.55,84.3	0.000008
0.55,84.4	0.000011
0.55,84.5	0.000012
0.55,84.6	0.000013
0.55,84.7	0.000014
0.55,84.8	0.000014
0.55,84.9	0.000018
0.55,85.0	0.000021
0.55,85.1	0.000023
0.55,85.2	0.000023
0.55,85.3	0.000026
0.55,85.4	0.000027
0.55,85.5	0.000028
0.55,85.6	0.000033
0.55,85.7	0.000035
0.55,85.8	0.000036
0.55,85.9	0.000036
0.55,86.0	0.000037
0.55,86.1	0.000038
0.55,86.2	0.000039
0.55,86.3	0.00004
0.55,86.4	0.000042
0.55,86.5	0.000048
0.55,86.6	0.000059
0.55,86.7	0.000063
0.55,86.8	0.000063
0.55,86.9	0.000064
0.55,87.0	0.000064
0.55,87.1	0.000065
0.55,87.2	0.000068
0.55,87.3	0.00007
0.55,87.4	0.00007
0.55,87.5	0.00007
0.55,87.6	0.00007
0.55,87.7	0.00007
0.55,87.8	0.00007
0.55,87.9	0.000071
0.55,88.0	0.000073

0.55,88.1	0.000076
0.55,88.2	0.000076
0.55,88.3	0.000076
0.55,88.4	0.000076
0.55,88.5	0.000076
0.55,88.6	0.00008
0.55,88.7	0.00008
0.55,88.8	0.00008
0.55,88.9	0.000082
0.55,89.0	0.000082
0.55,89.1	0.000087
0.55,89.2	0.000087
0.55,89.3	0.000089
0.55,89.4	0.000092
0.55,89.5	0.000094
0.55,89.6	0.000097
0.55,89.7	0.000097
0.55,89.8	0.000097
0.55,89.9	0.000097
0.55,90.0	0.000097
0.55,90.1	0.0001
0.55,90.2	0.0001
0.55,90.3	0.0001
0.55,90.4	0.0001
0.55,90.5	0.0001
0.55,90.6	0.000103
0.55,90.7	0.000103
0.55,90.8	0.000107
0.55,90.9	0.000107
0.55,91.0	0.00011
0.55,91.1	0.00011
0.55,91.2	0.00011
0.55,91.3	0.00011
0.55,91.4	0.00011
0.55,91.5	0.00011
0.55,91.6	0.00011
0.55,91.7	0.00011
0.55,91.8	0.00011
0.55,91.9	0.00011
0.55,92.0	0.00011
0.55,92.1	0.00011
0.55,92.2	0.000119

0.55,92.3	0.000119
0.55,92.4	0.000123
0.55,92.5	0.000123
0.55,92.6	0.000123
0.55,92.7	0.000123
0.55,92.8	0.000128
0.55,92.9	0.000133
0.55,93.0	0.000133
0.55,93.1	0.000133
0.55,93.2	0.000133
0.55,93.3	0.000133
0.55,93.4	0.000133
0.55,93.5	0.000133
0.55,93.6	0.000133
0.55,93.7	0.000133
0.55,93.8	0.000133
0.55,93.9	0.000139
0.55,94.0	0.000139
0.55,94.1	0.000139
0.55,94.2	0.000146
0.55,94.3	0.000146
0.55,94.4	0.000146
0.55,94.5	0.000153
0.55,94.6	0.000153
0.55,94.7	0.000153
0.55,94.8	0.000153
0.55,94.9	0.00016
0.55,95.0	0.00016
0.55,95.1	0.00016
0.55,95.2	0.00016
0.55,95.3	0.00016
0.55,95.4	0.00016
0.55,95.5	0.00016
0.55,95.6	0.00016
0.55,95.7	0.00016
0.55,95.8	0.00016
0.55,95.9	0.00016
0.55,96.0	0.00016
0.55,96.1	0.00016
0.55,96.2	0.00016
0.55,96.3	0.00016
0.55,96.4	0.00016

0.55,96.5	0.000163
0.55,96.6	0.000157
0.55,96.7	0.000157
0.55,96.8	0.00015
0.55,96.9	0.00015
0.55,97.0	0.00015
0.55,97.1	0.00016
0.55,97.2	0.00016
0.55,97.3	0.00016
0.55,97.4	0.00016
0.55,97.5	0.00016
0.55,97.6	0.00016
0.55,97.7	0.000164
0.55,97.8	0.000156
0.55,97.9	0.000156
0.55,98.0	0.000156
0.55,98.1	0.000146
0.55,98.2	0.000146
0.55,98.3	0.000146
0.55,98.4	0.000133
0.55,98.5	0.000133
0.55,98.6	0.000133
0.55,98.7	0.000119
0.55,98.8	0.0001
0.55,98.9	0.000076
0.55,99.0	0.000076
0.55,99.1	0.000076
0.55,99.2	0.000076
0.55,99.3	0.000045
0.55,99.4	0.000045
0.55,99.5	0.000053
0.55,99.6	0
0.55,99.7	0
0.55,99.8	0
0.55,99.9	0
0.55,100.0	0
0.56,0.0	0
0.56,0.1	0
0.56,0.2	0
0.56,0.3	0
0.56,0.4	0
0.56,0.5	0

0.56,0.6	0
0.56,0.7	0
0.56,0.8	0
0.56,0.9	0
0.56,1.0	0
0.56,1.1	0
0.56,1.2	0
0.56,1.3	0
0.56,1.4	0
0.56,1.5	0
0.56,1.6	0
0.56,1.7	0
0.56,1.8	0
0.56,1.9	0
0.56,2.0	0
0.56,2.1	0
0.56,2.2	0
0.56,2.3	0
0.56,2.4	0
0.56,2.5	0
0.56,2.6	0
0.56,2.7	0
0.56,2.8	0
0.56,2.9	0
0.56,3.0	0
0.56,3.1	0
0.56,3.2	0
0.56,3.3	0
0.56,3.4	0
0.56,3.5	0
0.56,3.6	0
0.56,3.7	0
0.56,3.8	0
0.56,3.9	0
0.56,4.0	0
0.56,4.1	0
0.56,4.2	0
0.56,4.3	0
0.56,4.4	0
0.56,4.5	0
0.56,4.6	0
0.56,4.7	0

0.56,4.8	0
0.56,4.9	0
0.56,5.0	0
0.56,5.1	0
0.56,5.2	0
0.56,5.3	0
0.56,5.4	0
0.56,5.5	0
0.56,5.6	0
0.56,5.7	0
0.56,5.8	0
0.56,5.9	0
0.56,6.0	0
0.56,6.1	0
0.56,6.2	0
0.56,6.3	0
0.56,6.4	0
0.56,6.5	0
0.56,6.6	0
0.56,6.7	0
0.56,6.8	0
0.56,6.9	0
0.56,7.0	0
0.56,7.1	0
0.56,7.2	0
0.56,7.3	0
0.56,7.4	0
0.56,7.5	0
0.56,7.6	0
0.56,7.7	0
0.56,7.8	0
0.56,7.9	0
0.56,8.0	0
0.56,8.1	0
0.56,8.2	0
0.56,8.3	0
0.56,8.4	0
0.56,8.5	0
0.56,8.6	0
0.56,8.7	0
0.56,8.8	0
0.56,8.9	0

0.56,9.0	0
0.56,9.1	0
0.56,9.2	0
0.56,9.3	0
0.56,9.4	0
0.56,9.5	0
0.56,9.6	0
0.56,9.7	0
0.56,9.8	0
0.56,9.9	0
0.56,10.0	0
0.56,10.1	0
0.56,10.2	0
0.56,10.3	0
0.56,10.4	0
0.56,10.5	0
0.56,10.6	0
0.56,10.7	0
0.56,10.8	0
0.56,10.9	0
0.56,11.0	0
0.56,11.1	0
0.56,11.2	0
0.56,11.3	0
0.56,11.4	0
0.56,11.5	0
0.56,11.6	0
0.56,11.7	0
0.56,11.8	0
0.56,11.9	0
0.56,12.0	0
0.56,12.1	0
0.56,12.2	0
0.56,12.3	0
0.56,12.4	0
0.56,12.5	0
0.56,12.6	0
0.56,12.7	0
0.56,12.8	0
0.56,12.9	0
0.56,13.0	0
0.56,13.1	0

0.56,13.2	0
0.56,13.3	0
0.56,13.4	0
0.56,13.5	0
0.56,13.6	0
0.56,13.7	0
0.56,13.8	0
0.56,13.9	0
0.56,14.0	0
0.56,14.1	0
0.56,14.2	0
0.56,14.3	0
0.56,14.4	0
0.56,14.5	0
0.56,14.6	0
0.56,14.7	0
0.56,14.8	0
0.56,14.9	0
0.56,15.0	0
0.56,15.1	0
0.56,15.2	0
0.56,15.3	0
0.56,15.4	0
0.56,15.5	0
0.56,15.6	0
0.56,15.7	0
0.56,15.8	0
0.56,15.9	0
0.56,16.0	0
0.56,16.1	0
0.56,16.2	0
0.56,16.3	0
0.56,16.4	0
0.56,16.5	0
0.56,16.6	0
0.56,16.7	0
0.56,16.8	0
0.56,16.9	0
0.56,17.0	0
0.56,17.1	0
0.56,17.2	0
0.56,17.3	0

0.56,17.4	0
0.56,17.5	0
0.56,17.6	0
0.56,17.7	0
0.56,17.8	0
0.56,17.9	0
0.56,18.0	0
0.56,18.1	0
0.56,18.2	0
0.56,18.3	0
0.56,18.4	0
0.56,18.5	0
0.56,18.6	0
0.56,18.7	0
0.56,18.8	0
0.56,18.9	0
0.56,19.0	0
0.56,19.1	0
0.56,19.2	0
0.56,19.3	0
0.56,19.4	0
0.56,19.5	0
0.56,19.6	0
0.56,19.7	0
0.56,19.8	0
0.56,19.9	0
0.56,20.0	0
0.56,20.1	0
0.56,20.2	0
0.56,20.3	0
0.56,20.4	0
0.56,20.5	0
0.56,20.6	0
0.56,20.7	0
0.56,20.8	0
0.56,20.9	0
0.56,21.0	0
0.56,21.1	0
0.56,21.2	0
0.56,21.3	0
0.56,21.4	0
0.56,21.5	0

0.56,21.6	0
0.56,21.7	0
0.56,21.8	0
0.56,21.9	0
0.56,22.0	0
0.56,22.1	0
0.56,22.2	0
0.56,22.3	0
0.56,22.4	0
0.56,22.5	0
0.56,22.6	0
0.56,22.7	0
0.56,22.8	0
0.56,22.9	0
0.56,23.0	0
0.56,23.1	0
0.56,23.2	0
0.56,23.3	0
0.56,23.4	0
0.56,23.5	0
0.56,23.6	0
0.56,23.7	0
0.56,23.8	0
0.56,23.9	0
0.56,24.0	0
0.56,24.1	0
0.56,24.2	0
0.56,24.3	0
0.56,24.4	0
0.56,24.5	0
0.56,24.6	0
0.56,24.7	0
0.56,24.8	0
0.56,24.9	0
0.56,25.0	0
0.56,25.1	0
0.56,25.2	0
0.56,25.3	0
0.56,25.4	0
0.56,25.5	0
0.56,25.6	0
0.56,25.7	0

0.56,25.8	0
0.56,25.9	0
0.56,26.0	0
0.56,26.1	0
0.56,26.2	0
0.56,26.3	0
0.56,26.4	0
0.56,26.5	0
0.56,26.6	0
0.56,26.7	0
0.56,26.8	0
0.56,26.9	0
0.56,27.0	0
0.56,27.1	0
0.56,27.2	0
0.56,27.3	0
0.56,27.4	0
0.56,27.5	0
0.56,27.6	0
0.56,27.7	0
0.56,27.8	0
0.56,27.9	0
0.56,28.0	0
0.56,28.1	0
0.56,28.2	0
0.56,28.3	0
0.56,28.4	0
0.56,28.5	0
0.56,28.6	0
0.56,28.7	0
0.56,28.8	0
0.56,28.9	0
0.56,29.0	0
0.56,29.1	0
0.56,29.2	0
0.56,29.3	0
0.56,29.4	0
0.56,29.5	0
0.56,29.6	0
0.56,29.7	0
0.56,29.8	0
0.56,29.9	0

0.56,30.0	0
0.56,30.1	0
0.56,30.2	0
0.56,30.3	0
0.56,30.4	0
0.56,30.5	0
0.56,30.6	0
0.56,30.7	0
0.56,30.8	0
0.56,30.9	0
0.56,31.0	0
0.56,31.1	0
0.56,31.2	0
0.56,31.3	0
0.56,31.4	0
0.56,31.5	0
0.56,31.6	0
0.56,31.7	0
0.56,31.8	0
0.56,31.9	0
0.56,32.0	0
0.56,32.1	0
0.56,32.2	0
0.56,32.3	0
0.56,32.4	0
0.56,32.5	0
0.56,32.6	0
0.56,32.7	0
0.56,32.8	0
0.56,32.9	0
0.56,33.0	0
0.56,33.1	0
0.56,33.2	0
0.56,33.3	0
0.56,33.4	0
0.56,33.5	0
0.56,33.6	0
0.56,33.7	0
0.56,33.8	0
0.56,33.9	0
0.56,34.0	0
0.56,34.1	0

0.56,34.2	0
0.56,34.3	0
0.56,34.4	0
0.56,34.5	0
0.56,34.6	0
0.56,34.7	0
0.56,34.8	0
0.56,34.9	0
0.56,35.0	0
0.56,35.1	0
0.56,35.2	0
0.56,35.3	0
0.56,35.4	0
0.56,35.5	0
0.56,35.6	0
0.56,35.7	0
0.56,35.8	0
0.56,35.9	0
0.56,36.0	0
0.56,36.1	0
0.56,36.2	0
0.56,36.3	0
0.56,36.4	0
0.56,36.5	0
0.56,36.6	0
0.56,36.7	0
0.56,36.8	0
0.56,36.9	0
0.56,37.0	0
0.56,37.1	0
0.56,37.2	0
0.56,37.3	0
0.56,37.4	0
0.56,37.5	0
0.56,37.6	0
0.56,37.7	0
0.56,37.8	0
0.56,37.9	0
0.56,38.0	0
0.56,38.1	0
0.56,38.2	0
0.56,38.3	0

0.56,38.4	0
0.56,38.5	0
0.56,38.6	0
0.56,38.7	0
0.56,38.8	0
0.56,38.9	0
0.56,39.0	0
0.56,39.1	0
0.56,39.2	0
0.56,39.3	0
0.56,39.4	0
0.56,39.5	0
0.56,39.6	0
0.56,39.7	0
0.56,39.8	0
0.56,39.9	0
0.56,40.0	0
0.56,40.1	0
0.56,40.2	0
0.56,40.3	0
0.56,40.4	0
0.56,40.5	0
0.56,40.6	0
0.56,40.7	0
0.56,40.8	0
0.56,40.9	0
0.56,41.0	0
0.56,41.1	0
0.56,41.2	0
0.56,41.3	0
0.56,41.4	0
0.56,41.5	0
0.56,41.6	0
0.56,41.7	0
0.56,41.8	0
0.56,41.9	0
0.56,42.0	0
0.56,42.1	0
0.56,42.2	0
0.56,42.3	0
0.56,42.4	0
0.56,42.5	0

0.56,42.6	0
0.56,42.7	0
0.56,42.8	0
0.56,42.9	0
0.56,43.0	0
0.56,43.1	0
0.56,43.2	0
0.56,43.3	0
0.56,43.4	0
0.56,43.5	0
0.56,43.6	0
0.56,43.7	0
0.56,43.8	0
0.56,43.9	0
0.56,44.0	0
0.56,44.1	0
0.56,44.2	0
0.56,44.3	0
0.56,44.4	0
0.56,44.5	0
0.56,44.6	0
0.56,44.7	0
0.56,44.8	0
0.56,44.9	0
0.56,45.0	0
0.56,45.1	0
0.56,45.2	0
0.56,45.3	0
0.56,45.4	0
0.56,45.5	0
0.56,45.6	0
0.56,45.7	0
0.56,45.8	0
0.56,45.9	0
0.56,46.0	0
0.56,46.1	0
0.56,46.2	0
0.56,46.3	0
0.56,46.4	0
0.56,46.5	0
0.56,46.6	0
0.56,46.7	0

0.56,46.8	0
0.56,46.9	0
0.56,47.0	0
0.56,47.1	0
0.56,47.2	0
0.56,47.3	0
0.56,47.4	0
0.56,47.5	0
0.56,47.6	0
0.56,47.7	0
0.56,47.8	0
0.56,47.9	0
0.56,48.0	0
0.56,48.1	0
0.56,48.2	0
0.56,48.3	0
0.56,48.4	0
0.56,48.5	0
0.56,48.6	0
0.56,48.7	0
0.56,48.8	0
0.56,48.9	0
0.56,49.0	0
0.56,49.1	0
0.56,49.2	0
0.56,49.3	0
0.56,49.4	0
0.56,49.5	0
0.56,49.6	0
0.56,49.7	0
0.56,49.8	0
0.56,49.9	0
0.56,50.0	0
0.56,50.1	0
0.56,50.2	0
0.56,50.3	0
0.56,50.4	0
0.56,50.5	0
0.56,50.6	0
0.56,50.7	0
0.56,50.8	0
0.56,50.9	0

0.56,51.0	0
0.56,51.1	0
0.56,51.2	0
0.56,51.3	0
0.56,51.4	0
0.56,51.5	0
0.56,51.6	0
0.56,51.7	0
0.56,51.8	0
0.56,51.9	0
0.56,52.0	0
0.56,52.1	0
0.56,52.2	0
0.56,52.3	0
0.56,52.4	0
0.56,52.5	0
0.56,52.6	0
0.56,52.7	0
0.56,52.8	0
0.56,52.9	0
0.56,53.0	0
0.56,53.1	0
0.56,53.2	0
0.56,53.3	0
0.56,53.4	0
0.56,53.5	0
0.56,53.6	0
0.56,53.7	0
0.56,53.8	0
0.56,53.9	0
0.56,54.0	0
0.56,54.1	0
0.56,54.2	0
0.56,54.3	0
0.56,54.4	0
0.56,54.5	0
0.56,54.6	0
0.56,54.7	0
0.56,54.8	0
0.56,54.9	0
0.56,55.0	0
0.56,55.1	0

0.56,55.2	0
0.56,55.3	0
0.56,55.4	0
0.56,55.5	0
0.56,55.6	0
0.56,55.7	0
0.56,55.8	0
0.56,55.9	0
0.56,56.0	0
0.56,56.1	0
0.56,56.2	0
0.56,56.3	0
0.56,56.4	0
0.56,56.5	0
0.56,56.6	0
0.56,56.7	0
0.56,56.8	0
0.56,56.9	0
0.56,57.0	0
0.56,57.1	0
0.56,57.2	0
0.56,57.3	0
0.56,57.4	0
0.56,57.5	0
0.56,57.6	0
0.56,57.7	0
0.56,57.8	0
0.56,57.9	0
0.56,58.0	0
0.56,58.1	0
0.56,58.2	0
0.56,58.3	0
0.56,58.4	0
0.56,58.5	0
0.56,58.6	0
0.56,58.7	0
0.56,58.8	0
0.56,58.9	0
0.56,59.0	0
0.56,59.1	0
0.56,59.2	0
0.56,59.3	0

0.56,59.4	0
0.56,59.5	0
0.56,59.6	0
0.56,59.7	0
0.56,59.8	0
0.56,59.9	0
0.56,60.0	0
0.56,60.1	0
0.56,60.2	0
0.56,60.3	0
0.56,60.4	0
0.56,60.5	0
0.56,60.6	0
0.56,60.7	0
0.56,60.8	0
0.56,60.9	0
0.56,61.0	0
0.56,61.1	0
0.56,61.2	0
0.56,61.3	0
0.56,61.4	0
0.56,61.5	0
0.56,61.6	0
0.56,61.7	0
0.56,61.8	0
0.56,61.9	0
0.56,62.0	0
0.56,62.1	0
0.56,62.2	0
0.56,62.3	0
0.56,62.4	0
0.56,62.5	0
0.56,62.6	0
0.56,62.7	0
0.56,62.8	0
0.56,62.9	0
0.56,63.0	0
0.56,63.1	0
0.56,63.2	0
0.56,63.3	0
0.56,63.4	0
0.56,63.5	0

0.56,63.6	0
0.56,63.7	0
0.56,63.8	0
0.56,63.9	0
0.56,64.0	0
0.56,64.1	0
0.56,64.2	0
0.56,64.3	0
0.56,64.4	0
0.56,64.5	0
0.56,64.6	0
0.56,64.7	0
0.56,64.8	0
0.56,64.9	0
0.56,65.0	0
0.56,65.1	0
0.56,65.2	0
0.56,65.3	0
0.56,65.4	0
0.56,65.5	0
0.56,65.6	0
0.56,65.7	0
0.56,65.8	0
0.56,65.9	0
0.56,66.0	0
0.56,66.1	0
0.56,66.2	0
0.56,66.3	0
0.56,66.4	0
0.56,66.5	0
0.56,66.6	0
0.56,66.7	0
0.56,66.8	0
0.56,66.9	0
0.56,67.0	0
0.56,67.1	0
0.56,67.2	0
0.56,67.3	0
0.56,67.4	0
0.56,67.5	0
0.56,67.6	0
0.56,67.7	0

0.56,67.8	0
0.56,67.9	0
0.56,68.0	0
0.56,68.1	0
0.56,68.2	0
0.56,68.3	0
0.56,68.4	0
0.56,68.5	0
0.56,68.6	0
0.56,68.7	0
0.56,68.8	0
0.56,68.9	0
0.56,69.0	0
0.56,69.1	0
0.56,69.2	0
0.56,69.3	0
0.56,69.4	0
0.56,69.5	0
0.56,69.6	0
0.56,69.7	0
0.56,69.8	0
0.56,69.9	0
0.56,70.0	0
0.56,70.1	0
0.56,70.2	0
0.56,70.3	0
0.56,70.4	0
0.56,70.5	0
0.56,70.6	0
0.56,70.7	0
0.56,70.8	0
0.56,70.9	0
0.56,71.0	0
0.56,71.1	0
0.56,71.2	0
0.56,71.3	0
0.56,71.4	0
0.56,71.5	0
0.56,71.6	0
0.56,71.7	0
0.56,71.8	0
0.56,71.9	0

0.56,72.0	0
0.56,72.1	0
0.56,72.2	0
0.56,72.3	0
0.56,72.4	0
0.56,72.5	0
0.56,72.6	0
0.56,72.7	0
0.56,72.8	0
0.56,72.9	0
0.56,73.0	0
0.56,73.1	0
0.56,73.2	0
0.56,73.3	0
0.56,73.4	0
0.56,73.5	0
0.56,73.6	0
0.56,73.7	0
0.56,73.8	0
0.56,73.9	0
0.56,74.0	0
0.56,74.1	0
0.56,74.2	0
0.56,74.3	0
0.56,74.4	0
0.56,74.5	0
0.56,74.6	0
0.56,74.7	0
0.56,74.8	0
0.56,74.9	0
0.56,75.0	0
0.56,75.1	0
0.56,75.2	0
0.56,75.3	0
0.56,75.4	0
0.56,75.5	0
0.56,75.6	0
0.56,75.7	0
0.56,75.8	0
0.56,75.9	0
0.56,76.0	0
0.56,76.1	0

0.56,76.2	0
0.56,76.3	0
0.56,76.4	0
0.56,76.5	0
0.56,76.6	0
0.56,76.7	0
0.56,76.8	0
0.56,76.9	0
0.56,77.0	0
0.56,77.1	0
0.56,77.2	0
0.56,77.3	0
0.56,77.4	0
0.56,77.5	0
0.56,77.6	0
0.56,77.7	0
0.56,77.8	0
0.56,77.9	0
0.56,78.0	0
0.56,78.1	0
0.56,78.2	0
0.56,78.3	0
0.56,78.4	0
0.56,78.5	0
0.56,78.6	0
0.56,78.7	0
0.56,78.8	0
0.56,78.9	0
0.56,79.0	0
0.56,79.1	0
0.56,79.2	0.000001
0.56,79.3	0.000001
0.56,79.4	0.000001
0.56,79.5	0.000001
0.56,79.6	0.000001
0.56,79.7	0.000001
0.56,79.8	0.000001
0.56,79.9	0.000001
0.56,80.0	0.000001
0.56,80.1	0.000001
0.56,80.2	0.000001
0.56,80.3	0.000001

0.56,80.4	0.000001
0.56,80.5	0.000001
0.56,80.6	0.000001
0.56,80.7	0.000001
0.56,80.8	0.000001
0.56,80.9	0.000001
0.56,81.0	0.000001
0.56,81.1	0.000001
0.56,81.2	0.000001
0.56,81.3	0.000002
0.56,81.4	0.000003
0.56,81.5	0.000006
0.56,81.6	0.000008
0.56,81.7	0.000009
0.56,81.8	0.00001
0.56,81.9	0.00001
0.56,82.0	0.000011
0.56,82.1	0.000011
0.56,82.2	0.000012
0.56,82.3	0.000012
0.56,82.4	0.000013
0.56,82.5	0.000014
0.56,82.6	0.000014
0.56,82.7	0.000014
0.56,82.8	0.000015
0.56,82.9	0.000016
0.56,83.0	0.000016
0.56,83.1	0.000017
0.56,83.2	0.000018
0.56,83.3	0.000018
0.56,83.4	0.000018
0.56,83.5	0.000019
0.56,83.6	0.000019
0.56,83.7	0.000019
0.56,83.8	0.000019
0.56,83.9	0.000019
0.56,84.0	0.000019
0.56,84.1	0.000019
0.56,84.2	0.00002
0.56,84.3	0.00002
0.56,84.4	0.000022
0.56,84.5	0.000022

0.56,84.6	0.000023
0.56,84.7	0.000023
0.56,84.8	0.000025
0.56,84.9	0.000029
0.56,85.0	0.000032
0.56,85.1	0.000034
0.56,85.2	0.000034
0.56,85.3	0.000036
0.56,85.4	0.000037
0.56,85.5	0.000037
0.56,85.6	0.000039
0.56,85.7	0.000039
0.56,85.8	0.000039
0.56,85.9	0.00004
0.56,86.0	0.00004
0.56,86.1	0.000041
0.56,86.2	0.000041
0.56,86.3	0.000043
0.56,86.4	0.000054
0.56,86.5	0.000079
0.56,86.6	0.000105
0.56,86.7	0.000116
0.56,86.8	0.000116
0.56,86.9	0.000119
0.56,87.0	0.000122
0.56,87.1	0.000128
0.56,87.2	0.000129
0.56,87.3	0.000129
0.56,87.4	0.000129
0.56,87.5	0.000129
0.56,87.6	0.000131
0.56,87.7	0.000133
0.56,87.8	0.000133
0.56,87.9	0.000139
0.56,88.0	0.000139
0.56,88.1	0.000142
0.56,88.2	0.000142
0.56,88.3	0.000144
0.56,88.4	0.000149
0.56,88.5	0.000151
0.56,88.6	0.000151
0.56,88.7	0.000159

0.56,88.8	0.000162
0.56,88.9	0.000165
0.56,89.0	0.000168
0.56,89.1	0.000168
0.56,89.2	0.000172
0.56,89.3	0.000186
0.56,89.4	0.00019
0.56,89.5	0.000194
0.56,89.6	0.000198
0.56,89.7	0.000198
0.56,89.8	0.000203
0.56,89.9	0.000203
0.56,90.0	0.000203
0.56,90.1	0.000203
0.56,90.2	0.000203
0.56,90.3	0.000208
0.56,90.4	0.000208
0.56,90.5	0.000213
0.56,90.6	0.000213
0.56,90.7	0.000213
0.56,90.8	0.000213
0.56,90.9	0.000218
0.56,91.0	0.000223
0.56,91.1	0.000229
0.56,91.2	0.000229
0.56,91.3	0.000229
0.56,91.4	0.000229
0.56,91.5	0.000229
0.56,91.6	0.000248
0.56,91.7	0.000262
0.56,91.8	0.000279
0.56,91.9	0.000308
0.56,92.0	0.000343
0.56,92.1	0.000357
0.56,92.2	0.000357
0.56,92.3	0.000357
0.56,92.4	0.000372
0.56,92.5	0.000372
0.56,92.6	0.000372
0.56,92.7	0.000372
0.56,92.8	0.000372
0.56,92.9	0.000406

0.56,93.0	0.000425
0.56,93.1	0.000425
0.56,93.2	0.000425
0.56,93.3	0.000425
0.56,93.4	0.000446
0.56,93.5	0.000446
0.56,93.6	0.000446
0.56,93.7	0.000446
0.56,93.8	0.000446
0.56,93.9	0.000446
0.56,94.0	0.000446
0.56,94.1	0.000446
0.56,94.2	0.000446
0.56,94.3	0.000446
0.56,94.4	0.00047
0.56,94.5	0.00047
0.56,94.6	0.00047
0.56,94.7	0.00047
0.56,94.8	0.00047
0.56,94.9	0.00047
0.56,95.0	0.00047
0.56,95.1	0.000525
0.56,95.2	0.000525
0.56,95.3	0.000525
0.56,95.4	0.000525
0.56,95.5	0.000525
0.56,95.6	0.000525
0.56,95.7	0.000525
0.56,95.8	0.000525
0.56,95.9	0.000525
0.56,96.0	0.000525
0.56,96.1	0.000525
0.56,96.2	0.000525
0.56,96.3	0.000525
0.56,96.4	0.000525
0.56,96.5	0.000521
0.56,96.6	0.000516
0.56,96.7	0.000516
0.56,96.8	0.000516
0.56,96.9	0.000516
0.56,97.0	0.000516
0.56,97.1	0.000516

0.56,97.2	0.000516
0.56,97.3	0.000516
0.56,97.4	0.000516
0.56,97.5	0.000516
0.56,97.6	0.000516
0.56,97.7	0.000516
0.56,97.8	0.00051
0.56,97.9	0.000476
0.56,98.0	0.000446
0.56,98.1	0.00051
0.56,98.2	0.00051
0.56,98.3	0.000595
0.56,98.4	0.000595
0.56,98.5	0.000595
0.56,98.6	0.000595
0.56,98.7	0.000595
0.56,98.8	0.000595
0.56,98.9	0.000595
0.56,99.0	0.000595
0.56,99.1	0.000595
0.56,99.2	0.000595
0.56,99.3	0.000595
0.56,99.4	0.000595
0.56,99.5	0.000595
0.56,99.6	0
0.56,99.7	0
0.56,99.8	0
0.56,99.9	0
0.56,100.0	0
0.57,0.0	0.000003
0.57,0.1	0.000003
0.57,0.2	0.000003
0.57,0.3	0.000003
0.57,0.4	0.000003
0.57,0.5	0.000003
0.57,0.6	0.000003
0.57,0.7	0.000003
0.57,0.8	0.000003
0.57,0.9	0.000003
0.57,1.0	0.000003
0.57,1.1	0.000003
0.57,1.2	0.000003

0.57,1.3	0.000003
0.57,1.4	0.000003
0.57,1.5	0.000003
0.57,1.6	0.000003
0.57,1.7	0.000003
0.57,1.8	0.000003
0.57,1.9	0.000003
0.57,2.0	0.000003
0.57,2.1	0.000003
0.57,2.2	0.000003
0.57,2.3	0.000003
0.57,2.4	0.000003
0.57,2.5	0.000003
0.57,2.6	0.000003
0.57,2.7	0.000003
0.57,2.8	0.000003
0.57,2.9	0.000003
0.57,3.0	0.000003
0.57,3.1	0.000003
0.57,3.2	0.000003
0.57,3.3	0.000003
0.57,3.4	0.000003
0.57,3.5	0.000003
0.57,3.6	0.000003
0.57,3.7	0.000003
0.57,3.8	0.000003
0.57,3.9	0.000003
0.57,4.0	0.000003
0.57,4.1	0.000003
0.57,4.2	0.000003
0.57,4.3	0.000003
0.57,4.4	0.000003
0.57,4.5	0.000003
0.57,4.6	0.000003
0.57,4.7	0.000003
0.57,4.8	0.000003
0.57,4.9	0.000003
0.57,5.0	0.000003
0.57,5.1	0.000003
0.57,5.2	0.000003
0.57,5.3	0.000003
0.57,5.4	0.000003

0.57,5.5	0.000003
0.57,5.6	0.000003
0.57,5.7	0.000003
0.57,5.8	0.000003
0.57,5.9	0.000003
0.57,6.0	0.000003
0.57,6.1	0.000003
0.57,6.2	0.000003
0.57,6.3	0.000003
0.57,6.4	0.000003
0.57,6.5	0.000003
0.57,6.6	0.000003
0.57,6.7	0.000003
0.57,6.8	0.000003
0.57,6.9	0.000003
0.57,7.0	0.000003
0.57,7.1	0.000003
0.57,7.2	0.000003
0.57,7.3	0.000003
0.57,7.4	0.000003
0.57,7.5	0.000003
0.57,7.6	0.000003
0.57,7.7	0.000003
0.57,7.8	0.000003
0.57,7.9	0.000003
0.57,8.0	0.000003
0.57,8.1	0.000003
0.57,8.2	0.000003
0.57,8.3	0.000003
0.57,8.4	0.000003
0.57,8.5	0.000003
0.57,8.6	0.000003
0.57,8.7	0.000003
0.57,8.8	0.000003
0.57,8.9	0.000003
0.57,9.0	0.000003
0.57,9.1	0.000003
0.57,9.2	0.000003
0.57,9.3	0.000003
0.57,9.4	0.000003
0.57,9.5	0.000003
0.57,9.6	0.000003

0.57,9.7	0.000003
0.57,9.8	0.000003
0.57,9.9	0.000003
0.57,10.0	0.000003
0.57,10.1	0.000003
0.57,10.2	0.000003
0.57,10.3	0.000003
0.57,10.4	0.000003
0.57,10.5	0.000003
0.57,10.6	0.000003
0.57,10.7	0.000003
0.57,10.8	0.000003
0.57,10.9	0.000003
0.57,11.0	0.000003
0.57,11.1	0.000003
0.57,11.2	0.000003
0.57,11.3	0.000003
0.57,11.4	0.000003
0.57,11.5	0.000003
0.57,11.6	0.000003
0.57,11.7	0.000003
0.57,11.8	0.000003
0.57,11.9	0.000003
0.57,12.0	0.000003
0.57,12.1	0.000003
0.57,12.2	0.000003
0.57,12.3	0.000003
0.57,12.4	0.000003
0.57,12.5	0.000003
0.57,12.6	0.000003
0.57,12.7	0.000003
0.57,12.8	0.000003
0.57,12.9	0.000003
0.57,13.0	0.000003
0.57,13.1	0.000003
0.57,13.2	0.000003
0.57,13.3	0.000003
0.57,13.4	0.000003
0.57,13.5	0.000003
0.57,13.6	0.000003
0.57,13.7	0.000003
0.57,13.8	0.000003

0.57,13.9	0.000003
0.57,14.0	0.000003
0.57,14.1	0.000003
0.57,14.2	0.000003
0.57,14.3	0.000003
0.57,14.4	0.000003
0.57,14.5	0.000003
0.57,14.6	0.000003
0.57,14.7	0.000003
0.57,14.8	0.000003
0.57,14.9	0.000003
0.57,15.0	0.000003
0.57,15.1	0.000003
0.57,15.2	0.000003
0.57,15.3	0.000003
0.57,15.4	0.000003
0.57,15.5	0.000003
0.57,15.6	0.000003
0.57,15.7	0.000003
0.57,15.8	0.000003
0.57,15.9	0.000003
0.57,16.0	0.000003
0.57,16.1	0.000003
0.57,16.2	0.000003
0.57,16.3	0.000003
0.57,16.4	0.000003
0.57,16.5	0.000003
0.57,16.6	0.000003
0.57,16.7	0.000003
0.57,16.8	0.000003
0.57,16.9	0.000003
0.57,17.0	0.000003
0.57,17.1	0.000003
0.57,17.2	0.000003
0.57,17.3	0.000003
0.57,17.4	0.000003
0.57,17.5	0.000003
0.57,17.6	0.000003
0.57,17.7	0.000003
0.57,17.8	0.000003
0.57,17.9	0.000003
0.57,18.0	0.000003

0.57,18.1	0.000003
0.57,18.2	0.000003
0.57,18.3	0.000003
0.57,18.4	0.000003
0.57,18.5	0.000003
0.57,18.6	0.000003
0.57,18.7	0.000003
0.57,18.8	0.000003
0.57,18.9	0.000003
0.57,19.0	0.000003
0.57,19.1	0.000003
0.57,19.2	0.000003
0.57,19.3	0.000003
0.57,19.4	0.000003
0.57,19.5	0.000003
0.57,19.6	0.000003
0.57,19.7	0.000003
0.57,19.8	0.000003
0.57,19.9	0.000003
0.57,20.0	0.000003
0.57,20.1	0.000003
0.57,20.2	0.000003
0.57,20.3	0.000003
0.57,20.4	0.000003
0.57,20.5	0.000003
0.57,20.6	0.000003
0.57,20.7	0.000003
0.57,20.8	0.000003
0.57,20.9	0.000003
0.57,21.0	0.000003
0.57,21.1	0.000003
0.57,21.2	0.000003
0.57,21.3	0.000003
0.57,21.4	0.000003
0.57,21.5	0.000003
0.57,21.6	0.000003
0.57,21.7	0.000003
0.57,21.8	0.000003
0.57,21.9	0.000003
0.57,22.0	0.000003
0.57,22.1	0.000003
0.57,22.2	0.000003

0.57,22.3	0.000003
0.57,22.4	0.000003
0.57,22.5	0.000003
0.57,22.6	0.000003
0.57,22.7	0.000003
0.57,22.8	0.000003
0.57,22.9	0.000003
0.57,23.0	0.000003
0.57,23.1	0.000003
0.57,23.2	0.000003
0.57,23.3	0.000003
0.57,23.4	0.000003
0.57,23.5	0.000003
0.57,23.6	0.000003
0.57,23.7	0.000003
0.57,23.8	0.000003
0.57,23.9	0.000003
0.57,24.0	0.000003
0.57,24.1	0.000003
0.57,24.2	0.000003
0.57,24.3	0.000003
0.57,24.4	0.000003
0.57,24.5	0.000003
0.57,24.6	0.000003
0.57,24.7	0.000003
0.57,24.8	0.000003
0.57,24.9	0.000003
0.57,25.0	0.000003
0.57,25.1	0.000003
0.57,25.2	0.000003
0.57,25.3	0.000003
0.57,25.4	0.000003
0.57,25.5	0.000003
0.57,25.6	0.000003
0.57,25.7	0.000003
0.57,25.8	0.000003
0.57,25.9	0.000003
0.57,26.0	0.000003
0.57,26.1	0.000003
0.57,26.2	0.000003
0.57,26.3	0.000003
0.57,26.4	0.000003

0.57,26.5	0.000003
0.57,26.6	0.000003
0.57,26.7	0.000003
0.57,26.8	0.000003
0.57,26.9	0.000003
0.57,27.0	0.000003
0.57,27.1	0.000003
0.57,27.2	0.000003
0.57,27.3	0.000003
0.57,27.4	0.000003
0.57,27.5	0.000003
0.57,27.6	0.000003
0.57,27.7	0.000003
0.57,27.8	0.000003
0.57,27.9	0.000003
0.57,28.0	0.000003
0.57,28.1	0.000003
0.57,28.2	0.000003
0.57,28.3	0.000003
0.57,28.4	0.000003
0.57,28.5	0.000003
0.57,28.6	0.000003
0.57,28.7	0.000003
0.57,28.8	0.000003
0.57,28.9	0.000003
0.57,29.0	0.000003
0.57,29.1	0.000003
0.57,29.2	0.000003
0.57,29.3	0.000003
0.57,29.4	0.000003
0.57,29.5	0.000003
0.57,29.6	0.000003
0.57,29.7	0.000003
0.57,29.8	0.000003
0.57,29.9	0.000003
0.57,30.0	0.000003
0.57,30.1	0.000003
0.57,30.2	0.000003
0.57,30.3	0.000003
0.57,30.4	0.000003
0.57,30.5	0.000003
0.57,30.6	0.000003

0.57,30.7	0.000003
0.57,30.8	0.000003
0.57,30.9	0.000003
0.57,31.0	0.000003
0.57,31.1	0.000003
0.57,31.2	0.000003
0.57,31.3	0.000003
0.57,31.4	0.000003
0.57,31.5	0.000003
0.57,31.6	0.000003
0.57,31.7	0.000003
0.57,31.8	0.000003
0.57,31.9	0.000003
0.57,32.0	0.000003
0.57,32.1	0.000003
0.57,32.2	0.000003
0.57,32.3	0.000003
0.57,32.4	0.000003
0.57,32.5	0.000003
0.57,32.6	0.000003
0.57,32.7	0.000003
0.57,32.8	0.000003
0.57,32.9	0.000003
0.57,33.0	0.000003
0.57,33.1	0.000003
0.57,33.2	0.000003
0.57,33.3	0.000003
0.57,33.4	0.000003
0.57,33.5	0.000003
0.57,33.6	0.000003
0.57,33.7	0.000003
0.57,33.8	0.000003
0.57,33.9	0.000003
0.57,34.0	0.000003
0.57,34.1	0.000003
0.57,34.2	0.000003
0.57,34.3	0.000003
0.57,34.4	0.000003
0.57,34.5	0.000003
0.57,34.6	0.000003
0.57,34.7	0.000003
0.57,34.8	0.000003

0.57,34.9	0.000003
0.57,35.0	0.000003
0.57,35.1	0.000003
0.57,35.2	0.000003
0.57,35.3	0.000003
0.57,35.4	0.000003
0.57,35.5	0.000003
0.57,35.6	0.000003
0.57,35.7	0.000003
0.57,35.8	0.000003
0.57,35.9	0.000003
0.57,36.0	0.000003
0.57,36.1	0.000003
0.57,36.2	0.000003
0.57,36.3	0.000003
0.57,36.4	0.000003
0.57,36.5	0.000003
0.57,36.6	0.000003
0.57,36.7	0.000003
0.57,36.8	0.000003
0.57,36.9	0.000003
0.57,37.0	0.000003
0.57,37.1	0.000003
0.57,37.2	0.000003
0.57,37.3	0.000003
0.57,37.4	0.000003
0.57,37.5	0.000003
0.57,37.6	0.000003
0.57,37.7	0.000003
0.57,37.8	0.000003
0.57,37.9	0.000003
0.57,38.0	0.000003
0.57,38.1	0.000003
0.57,38.2	0.000003
0.57,38.3	0.000003
0.57,38.4	0.000003
0.57,38.5	0.000003
0.57,38.6	0.000003
0.57,38.7	0.000003
0.57,38.8	0.000003
0.57,38.9	0.000003
0.57,39.0	0.000003

0.57,39.1	0.000003
0.57,39.2	0.000003
0.57,39.3	0.000003
0.57,39.4	0.000003
0.57,39.5	0.000003
0.57,39.6	0.000003
0.57,39.7	0.000003
0.57,39.8	0.000003
0.57,39.9	0.000003
0.57,40.0	0.000003
0.57,40.1	0.000003
0.57,40.2	0.000003
0.57,40.3	0.000003
0.57,40.4	0.000003
0.57,40.5	0.000003
0.57,40.6	0.000003
0.57,40.7	0.000003
0.57,40.8	0.000003
0.57,40.9	0.000003
0.57,41.0	0.000003
0.57,41.1	0.000003
0.57,41.2	0.000003
0.57,41.3	0.000003
0.57,41.4	0.000003
0.57,41.5	0.000003
0.57,41.6	0.000003
0.57,41.7	0.000003
0.57,41.8	0.000003
0.57,41.9	0.000003
0.57,42.0	0.000003
0.57,42.1	0.000003
0.57,42.2	0.000003
0.57,42.3	0.000003
0.57,42.4	0.000003
0.57,42.5	0.000003
0.57,42.6	0.000003
0.57,42.7	0.000003
0.57,42.8	0.000003
0.57,42.9	0.000003
0.57,43.0	0.000003
0.57,43.1	0.000003
0.57,43.2	0.000003

0.57,43.3	0.000003
0.57,43.4	0.000003
0.57,43.5	0.000003
0.57,43.6	0.000003
0.57,43.7	0.000003
0.57,43.8	0.000003
0.57,43.9	0.000003
0.57,44.0	0.000003
0.57,44.1	0.000003
0.57,44.2	0.000003
0.57,44.3	0.000003
0.57,44.4	0.000003
0.57,44.5	0.000003
0.57,44.6	0.000003
0.57,44.7	0.000003
0.57,44.8	0.000003
0.57,44.9	0.000003
0.57,45.0	0.000003
0.57,45.1	0.000003
0.57,45.2	0.000003
0.57,45.3	0.000003
0.57,45.4	0.000003
0.57,45.5	0.000003
0.57,45.6	0.000003
0.57,45.7	0.000003
0.57,45.8	0.000003
0.57,45.9	0.000003
0.57,46.0	0.000003
0.57,46.1	0.000003
0.57,46.2	0.000003
0.57,46.3	0.000003
0.57,46.4	0.000003
0.57,46.5	0.000003
0.57,46.6	0.000003
0.57,46.7	0.000003
0.57,46.8	0.000003
0.57,46.9	0.000003
0.57,47.0	0.000003
0.57,47.1	0.000003
0.57,47.2	0.000003
0.57,47.3	0.000003
0.57,47.4	0.000003

0.57,47.5	0.000003
0.57,47.6	0.000003
0.57,47.7	0.000003
0.57,47.8	0.000003
0.57,47.9	0.000003
0.57,48.0	0.000003
0.57,48.1	0.000003
0.57,48.2	0.000003
0.57,48.3	0.000003
0.57,48.4	0.000003
0.57,48.5	0.000003
0.57,48.6	0.000003
0.57,48.7	0.000003
0.57,48.8	0.000003
0.57,48.9	0.000003
0.57,49.0	0.000003
0.57,49.1	0.000003
0.57,49.2	0.000003
0.57,49.3	0.000003
0.57,49.4	0.000003
0.57,49.5	0.000003
0.57,49.6	0.000003
0.57,49.7	0.000003
0.57,49.8	0.000003
0.57,49.9	0.000003
0.57,50.0	0.000003
0.57,50.1	0.000003
0.57,50.2	0.000003
0.57,50.3	0.000003
0.57,50.4	0.000003
0.57,50.5	0.000003
0.57,50.6	0.000003
0.57,50.7	0.000003
0.57,50.8	0.000003
0.57,50.9	0.000003
0.57,51.0	0.000003
0.57,51.1	0.000003
0.57,51.2	0.000003
0.57,51.3	0.000003
0.57,51.4	0.000003
0.57,51.5	0.000003
0.57,51.6	0.000003

0.57,51.7	0.000003
0.57,51.8	0.000003
0.57,51.9	0.000003
0.57,52.0	0.000003
0.57,52.1	0.000003
0.57,52.2	0.000003
0.57,52.3	0.000003
0.57,52.4	0.000003
0.57,52.5	0.000003
0.57,52.6	0.000003
0.57,52.7	0.000003
0.57,52.8	0.000003
0.57,52.9	0.000003
0.57,53.0	0.000003
0.57,53.1	0.000003
0.57,53.2	0.000003
0.57,53.3	0.000003
0.57,53.4	0.000003
0.57,53.5	0.000003
0.57,53.6	0.000003
0.57,53.7	0.000003
0.57,53.8	0.000003
0.57,53.9	0.000003
0.57,54.0	0.000003
0.57,54.1	0.000003
0.57,54.2	0.000003
0.57,54.3	0.000003
0.57,54.4	0.000003
0.57,54.5	0.000003
0.57,54.6	0.000003
0.57,54.7	0.000003
0.57,54.8	0.000003
0.57,54.9	0.000003
0.57,55.0	0.000003
0.57,55.1	0.000003
0.57,55.2	0.000003
0.57,55.3	0.000003
0.57,55.4	0.000003
0.57,55.5	0.000003
0.57,55.6	0.000003
0.57,55.7	0.000003
0.57,55.8	0.000003

0.57,55.9	0.000003
0.57,56.0	0.000003
0.57,56.1	0.000003
0.57,56.2	0.000003
0.57,56.3	0.000003
0.57,56.4	0.000003
0.57,56.5	0.000003
0.57,56.6	0.000003
0.57,56.7	0.000003
0.57,56.8	0.000003
0.57,56.9	0.000003
0.57,57.0	0.000003
0.57,57.1	0.000003
0.57,57.2	0.000003
0.57,57.3	0.000003
0.57,57.4	0.000003
0.57,57.5	0.000003
0.57,57.6	0.000003
0.57,57.7	0.000003
0.57,57.8	0.000003
0.57,57.9	0.000003
0.57,58.0	0.000003
0.57,58.1	0.000003
0.57,58.2	0.000003
0.57,58.3	0.000003
0.57,58.4	0.000003
0.57,58.5	0.000003
0.57,58.6	0.000003
0.57,58.7	0.000003
0.57,58.8	0.000003
0.57,58.9	0.000003
0.57,59.0	0.000003
0.57,59.1	0.000003
0.57,59.2	0.000003
0.57,59.3	0.000003
0.57,59.4	0.000003
0.57,59.5	0.000003
0.57,59.6	0.000003
0.57,59.7	0.000003
0.57,59.8	0.000003
0.57,59.9	0.000003
0.57,60.0	0.000003

0.57,60.1	0.000003
0.57,60.2	0.000003
0.57,60.3	0.000003
0.57,60.4	0.000003
0.57,60.5	0.000003
0.57,60.6	0.000003
0.57,60.7	0.000003
0.57,60.8	0.000003
0.57,60.9	0.000003
0.57,61.0	0.000003
0.57,61.1	0.000003
0.57,61.2	0.000003
0.57,61.3	0.000003
0.57,61.4	0.000003
0.57,61.5	0.000003
0.57,61.6	0.000003
0.57,61.7	0.000003
0.57,61.8	0.000003
0.57,61.9	0.000003
0.57,62.0	0.000003
0.57,62.1	0.000003
0.57,62.2	0.000003
0.57,62.3	0.000003
0.57,62.4	0.000003
0.57,62.5	0.000003
0.57,62.6	0.000003
0.57,62.7	0.000003
0.57,62.8	0.000003
0.57,62.9	0.000003
0.57,63.0	0.000003
0.57,63.1	0.000003
0.57,63.2	0.000003
0.57,63.3	0.000003
0.57,63.4	0.000003
0.57,63.5	0.000003
0.57,63.6	0.000003
0.57,63.7	0.000003
0.57,63.8	0.000003
0.57,63.9	0.000003
0.57,64.0	0.000003
0.57,64.1	0.000003
0.57,64.2	0.000003

0.57,64.3	0.000003
0.57,64.4	0.000003
0.57,64.5	0.000003
0.57,64.6	0.000003
0.57,64.7	0.000003
0.57,64.8	0.000003
0.57,64.9	0.000003
0.57,65.0	0.000003
0.57,65.1	0.000003
0.57,65.2	0.000003
0.57,65.3	0.000003
0.57,65.4	0.000003
0.57,65.5	0.000003
0.57,65.6	0.000003
0.57,65.7	0.000003
0.57,65.8	0.000003
0.57,65.9	0.000003
0.57,66.0	0.000003
0.57,66.1	0.000003
0.57,66.2	0.000003
0.57,66.3	0.000003
0.57,66.4	0.000003
0.57,66.5	0.000003
0.57,66.6	0.000003
0.57,66.7	0.000003
0.57,66.8	0.000003
0.57,66.9	0.000003
0.57,67.0	0.000003
0.57,67.1	0.000003
0.57,67.2	0.000003
0.57,67.3	0.000003
0.57,67.4	0.000003
0.57,67.5	0.000003
0.57,67.6	0.000003
0.57,67.7	0.000003
0.57,67.8	0.000003
0.57,67.9	0.000003
0.57,68.0	0.000003
0.57,68.1	0.000003
0.57,68.2	0.000003
0.57,68.3	0.000003
0.57,68.4	0.000003

0.57,68.5	0.000003
0.57,68.6	0.000003
0.57,68.7	0.000003
0.57,68.8	0.000003
0.57,68.9	0.000003
0.57,69.0	0.000003
0.57,69.1	0.000003
0.57,69.2	0.000003
0.57,69.3	0.000003
0.57,69.4	0.000003
0.57,69.5	0.000003
0.57,69.6	0.000003
0.57,69.7	0.000003
0.57,69.8	0.000003
0.57,69.9	0.000003
0.57,70.0	0.000003
0.57,70.1	0.000003
0.57,70.2	0.000003
0.57,70.3	0.000003
0.57,70.4	0.000003
0.57,70.5	0.000003
0.57,70.6	0.000003
0.57,70.7	0.000003
0.57,70.8	0.000003
0.57,70.9	0.000003
0.57,71.0	0.000003
0.57,71.1	0.000003
0.57,71.2	0.000003
0.57,71.3	0.000003
0.57,71.4	0.000003
0.57,71.5	0.000003
0.57,71.6	0.000003
0.57,71.7	0.000003
0.57,71.8	0.000003
0.57,71.9	0.000003
0.57,72.0	0.000003
0.57,72.1	0.000003
0.57,72.2	0.000003
0.57,72.3	0.000003
0.57,72.4	0.000003
0.57,72.5	0.000003
0.57,72.6	0.000003

0.57,72.7	0.000003
0.57,72.8	0.000003
0.57,72.9	0.000003
0.57,73.0	0.000003
0.57,73.1	0.000003
0.57,73.2	0.000003
0.57,73.3	0.000003
0.57,73.4	0.000003
0.57,73.5	0.000003
0.57,73.6	0.000003
0.57,73.7	0.000003
0.57,73.8	0.000003
0.57,73.9	0.000003
0.57,74.0	0.000003
0.57,74.1	0.000003
0.57,74.2	0.000003
0.57,74.3	0.000003
0.57,74.4	0.000003
0.57,74.5	0.000003
0.57,74.6	0.000003
0.57,74.7	0.000003
0.57,74.8	0.000003
0.57,74.9	0.000003
0.57,75.0	0.000003
0.57,75.1	0.000003
0.57,75.2	0.000003
0.57,75.3	0.000003
0.57,75.4	0.000003
0.57,75.5	0.000003
0.57,75.6	0.000003
0.57,75.7	0.000003
0.57,75.8	0.000003
0.57,75.9	0.000003
0.57,76.0	0.000003
0.57,76.1	0.000003
0.57,76.2	0.000003
0.57,76.3	0.000003
0.57,76.4	0.000003
0.57,76.5	0.000003
0.57,76.6	0.000003
0.57,76.7	0.000003
0.57,76.8	0.000003

0.57,76.9	0.000003
0.57,77.0	0.000003
0.57,77.1	0.000003
0.57,77.2	0.000003
0.57,77.3	0.000003
0.57,77.4	0.000003
0.57,77.5	0.000003
0.57,77.6	0.000003
0.57,77.7	0.000003
0.57,77.8	0.000003
0.57,77.9	0.000003
0.57,78.0	0.000003
0.57,78.1	0.000003
0.57,78.2	0.000003
0.57,78.3	0.000003
0.57,78.4	0.000003
0.57,78.5	0.000003
0.57,78.6	0.000003
0.57,78.7	0.000003
0.57,78.8	0.000003
0.57,78.9	0.000003
0.57,79.0	0.000003
0.57,79.1	0.000003
0.57,79.2	0.000004
0.57,79.3	0.000004
0.57,79.4	0.000004
0.57,79.5	0.000004
0.57,79.6	0.000005
0.57,79.7	0.000005
0.57,79.8	0.000005
0.57,79.9	0.000006
0.57,80.0	0.000006
0.57,80.1	0.000006
0.57,80.2	0.000006
0.57,80.3	0.000006
0.57,80.4	0.000006
0.57,80.5	0.000006
0.57,80.6	0.000006
0.57,80.7	0.000006
0.57,80.8	0.000006
0.57,80.9	0.000006
0.57,81.0	0.000006

0.57,81.1	0.000006
0.57,81.2	0.000007
0.57,81.3	0.000009
0.57,81.4	0.000019
0.57,81.5	0.000032
0.57,81.6	0.000045
0.57,81.7	0.000054
0.57,81.8	0.000059
0.57,81.9	0.000064
0.57,82.0	0.000067
0.57,82.1	0.000069
0.57,82.2	0.000071
0.57,82.3	0.000074
0.57,82.4	0.00008
0.57,82.5	0.000083
0.57,82.6	0.000086
0.57,82.7	0.000087
0.57,82.8	0.00009
0.57,82.9	0.000097
0.57,83.0	0.000102
0.57,83.1	0.000106
0.57,83.2	0.000108
0.57,83.3	0.000112
0.57,83.4	0.000114
0.57,83.5	0.000114
0.57,83.6	0.000116
0.57,83.7	0.000117
0.57,83.8	0.000119
0.57,83.9	0.00012
0.57,84.0	0.000122
0.57,84.1	0.000123
0.57,84.2	0.000124
0.57,84.3	0.000129
0.57,84.4	0.000132
0.57,84.5	0.000134
0.57,84.6	0.000138
0.57,84.7	0.000141
0.57,84.8	0.000147
0.57,84.9	0.00015
0.57,85.0	0.000154
0.57,85.1	0.000158
0.57,85.2	0.000163

0.57,85.3	0.000167
0.57,85.4	0.000169
0.57,85.5	0.000169
0.57,85.6	0.00017
0.57,85.7	0.00017
0.57,85.8	0.00017
0.57,85.9	0.000171
0.57,86.0	0.000172
0.57,86.1	0.000172
0.57,86.2	0.000176
0.57,86.3	0.000189
0.57,86.4	0.000236
0.57,86.5	0.000351
0.57,86.6	0.00042
0.57,86.7	0.000428
0.57,86.8	0.000438
0.57,86.9	0.000438
0.57,87.0	0.000441
0.57,87.1	0.000444
0.57,87.2	0.000447
0.57,87.3	0.000453
0.57,87.4	0.000457
0.57,87.5	0.00046
0.57,87.6	0.00046
0.57,87.7	0.000467
0.57,87.8	0.000467
0.57,87.9	0.000471
0.57,88.0	0.000478
0.57,88.1	0.000489
0.57,88.2	0.000489
0.57,88.3	0.000501
0.57,88.4	0.000522
0.57,88.5	0.000526
0.57,88.6	0.00055
0.57,88.7	0.000555
0.57,88.8	0.000555
0.57,88.9	0.000555
0.57,89.0	0.00056
0.57,89.1	0.000565
0.57,89.2	0.00057
0.57,89.3	0.000586
0.57,89.4	0.000597

0.57,89.5	0.000609
0.57,89.6	0.000615
0.57,89.7	0.000628
0.57,89.8	0.000647
0.57,89.9	0.000647
0.57,90.0	0.000654
0.57,90.1	0.000654
0.57,90.2	0.000654
0.57,90.3	0.000654
0.57,90.4	0.000668
0.57,90.5	0.000675
0.57,90.6	0.000698
0.57,90.7	0.000698
0.57,90.8	0.000698
0.57,90.9	0.000698
0.57,91.0	0.000722
0.57,91.1	0.000722
0.57,91.2	0.000722
0.57,91.3	0.000722
0.57,91.4	0.000722
0.57,91.5	0.000731
0.57,91.6	0.000731
0.57,91.7	0.00074
0.57,91.8	0.000758
0.57,91.9	0.000777
0.57,92.0	0.000817
0.57,92.1	0.000875
0.57,92.2	0.000914
0.57,92.3	0.000941
0.57,92.4	0.000941
0.57,92.5	0.000956
0.57,92.6	0.000971
0.57,92.7	0.001002
0.57,92.8	0.001002
0.57,92.9	0.001071
0.57,93.0	0.00109
0.57,93.1	0.00113
0.57,93.2	0.00113
0.57,93.3	0.00115
0.57,93.4	0.001218
0.57,93.5	0.001218
0.57,93.6	0.001218

0.57,93.7	0.001218
0.57,93.8	0.001218
0.57,93.9	0.001218
0.57,94.0	0.001243
0.57,94.1	0.001268
0.57,94.2	0.001322
0.57,94.3	0.001322
0.57,94.4	0.001322
0.57,94.5	0.001322
0.57,94.6	0.001322
0.57,94.7	0.001381
0.57,94.8	0.001479
0.57,94.9	0.001479
0.57,95.0	0.001479
0.57,95.1	0.001479
0.57,95.2	0.001479
0.57,95.3	0.001479
0.57,95.4	0.001479
0.57,95.5	0.001479
0.57,95.6	0.001479
0.57,95.7	0.00147
0.57,95.8	0.00147
0.57,95.9	0.00147
0.57,96.0	0.00147
0.57,96.1	0.00147
0.57,96.2	0.00147
0.57,96.3	0.001464
0.57,96.4	0.001464
0.57,96.5	0.001464
0.57,96.6	0.00146
0.57,96.7	0.00146
0.57,96.8	0.00146
0.57,96.9	0.00146
0.57,97.0	0.001456
0.57,97.1	0.001499
0.57,97.2	0.001499
0.57,97.3	0.001499
0.57,97.4	0.001499
0.57,97.5	0.001499
0.57,97.6	0.001499
0.57,97.7	0.001499
0.57,97.8	0.00149

0.57,97.9	0.001454
0.57,98.0	0.001504
0.57,98.1	0.001499
0.57,98.2	0.001499
0.57,98.3	0.001499
0.57,98.4	0.001487
0.57,98.5	0.001487
0.57,98.6	0.001487
0.57,98.7	0.001479
0.57,98.8	0.00146
0.57,98.9	0.00146
0.57,99.0	0.001434
0.57,99.1	0.001434
0.57,99.2	0.001434
0.57,99.3	0.001416
0.57,99.4	0.001274
0.57,99.5	0.001195
0.57,99.6	0.001195
0.57,99.7	0.001062
0.57,99.8	0
0.57,99.9	0
0.57,100.0	0
0.58,0.0	0.000007
0.58,0.1	0.000007
0.58,0.2	0.000007
0.58,0.3	0.000007
0.58,0.4	0.000007
0.58,0.5	0.000007
0.58,0.6	0.000007
0.58,0.7	0.000007
0.58,0.8	0.000007
0.58,0.9	0.000007
0.58,1.0	0.000007
0.58,1.1	0.000007
0.58,1.2	0.000007
0.58,1.3	0.000007
0.58,1.4	0.000007
0.58,1.5	0.000007
0.58,1.6	0.000007
0.58,1.7	0.000007
0.58,1.8	0.000007
0.58,1.9	0.000007

0.58,2.0	0.000007
0.58,2.1	0.000007
0.58,2.2	0.000007
0.58,2.3	0.000007
0.58,2.4	0.000007
0.58,2.5	0.000007
0.58,2.6	0.000007
0.58,2.7	0.000007
0.58,2.8	0.000007
0.58,2.9	0.000007
0.58,3.0	0.000007
0.58,3.1	0.000007
0.58,3.2	0.000007
0.58,3.3	0.000007
0.58,3.4	0.000007
0.58,3.5	0.000007
0.58,3.6	0.000007
0.58,3.7	0.000007
0.58,3.8	0.000007
0.58,3.9	0.000007
0.58,4.0	0.000007
0.58,4.1	0.000007
0.58,4.2	0.000007
0.58,4.3	0.000007
0.58,4.4	0.000007
0.58,4.5	0.000007
0.58,4.6	0.000007
0.58,4.7	0.000007
0.58,4.8	0.000007
0.58,4.9	0.000007
0.58,5.0	0.000007
0.58,5.1	0.000007
0.58,5.2	0.000007
0.58,5.3	0.000007
0.58,5.4	0.000007
0.58,5.5	0.000007
0.58,5.6	0.000007
0.58,5.7	0.000007
0.58,5.8	0.000007
0.58,5.9	0.000007
0.58,6.0	0.000007
0.58,6.1	0.000007

0.58,6.2	0.000007
0.58,6.3	0.000007
0.58,6.4	0.000007
0.58,6.5	0.000007
0.58,6.6	0.000007
0.58,6.7	0.000007
0.58,6.8	0.000007
0.58,6.9	0.000007
0.58,7.0	0.000007
0.58,7.1	0.000007
0.58,7.2	0.000007
0.58,7.3	0.000007
0.58,7.4	0.000007
0.58,7.5	0.000007
0.58,7.6	0.000007
0.58,7.7	0.000007
0.58,7.8	0.000007
0.58,7.9	0.000007
0.58,8.0	0.000007
0.58,8.1	0.000007
0.58,8.2	0.000007
0.58,8.3	0.000007
0.58,8.4	0.000007
0.58,8.5	0.000007
0.58,8.6	0.000007
0.58,8.7	0.000007
0.58,8.8	0.000007
0.58,8.9	0.000007
0.58,9.0	0.000007
0.58,9.1	0.000007
0.58,9.2	0.000007
0.58,9.3	0.000007
0.58,9.4	0.000007
0.58,9.5	0.000007
0.58,9.6	0.000007
0.58,9.7	0.000007
0.58,9.8	0.000007
0.58,9.9	0.000007
0.58,10.0	0.000007
0.58,10.1	0.000007
0.58,10.2	0.000007
0.58,10.3	0.000007

0.58,10.4	0.000007
0.58,10.5	0.000007
0.58,10.6	0.000007
0.58,10.7	0.000007
0.58,10.8	0.000007
0.58,10.9	0.000007
0.58,11.0	0.000007
0.58,11.1	0.000007
0.58,11.2	0.000007
0.58,11.3	0.000007
0.58,11.4	0.000007
0.58,11.5	0.000007
0.58,11.6	0.000007
0.58,11.7	0.000007
0.58,11.8	0.000007
0.58,11.9	0.000007
0.58,12.0	0.000007
0.58,12.1	0.000007
0.58,12.2	0.000007
0.58,12.3	0.000007
0.58,12.4	0.000007
0.58,12.5	0.000007
0.58,12.6	0.000007
0.58,12.7	0.000007
0.58,12.8	0.000007
0.58,12.9	0.000007
0.58,13.0	0.000007
0.58,13.1	0.000007
0.58,13.2	0.000007
0.58,13.3	0.000007
0.58,13.4	0.000007
0.58,13.5	0.000007
0.58,13.6	0.000007
0.58,13.7	0.000007
0.58,13.8	0.000007
0.58,13.9	0.000007
0.58,14.0	0.000007
0.58,14.1	0.000007
0.58,14.2	0.000007
0.58,14.3	0.000007
0.58,14.4	0.000007
0.58,14.5	0.000007

0.58,14.6	0.000007
0.58,14.7	0.000007
0.58,14.8	0.000007
0.58,14.9	0.000007
0.58,15.0	0.000007
0.58,15.1	0.000007
0.58,15.2	0.000007
0.58,15.3	0.000007
0.58,15.4	0.000007
0.58,15.5	0.000007
0.58,15.6	0.000007
0.58,15.7	0.000007
0.58,15.8	0.000007
0.58,15.9	0.000007
0.58,16.0	0.000007
0.58,16.1	0.000007
0.58,16.2	0.000007
0.58,16.3	0.000007
0.58,16.4	0.000007
0.58,16.5	0.000007
0.58,16.6	0.000007
0.58,16.7	0.000007
0.58,16.8	0.000007
0.58,16.9	0.000007
0.58,17.0	0.000007
0.58,17.1	0.000007
0.58,17.2	0.000007
0.58,17.3	0.000007
0.58,17.4	0.000007
0.58,17.5	0.000007
0.58,17.6	0.000007
0.58,17.7	0.000007
0.58,17.8	0.000007
0.58,17.9	0.000007
0.58,18.0	0.000007
0.58,18.1	0.000007
0.58,18.2	0.000007
0.58,18.3	0.000007
0.58,18.4	0.000007
0.58,18.5	0.000007
0.58,18.6	0.000007
0.58,18.7	0.000007

0.58,18.8	0.000007
0.58,18.9	0.000007
0.58,19.0	0.000007
0.58,19.1	0.000007
0.58,19.2	0.000007
0.58,19.3	0.000007
0.58,19.4	0.000007
0.58,19.5	0.000007
0.58,19.6	0.000007
0.58,19.7	0.000007
0.58,19.8	0.000007
0.58,19.9	0.000007
0.58,20.0	0.000007
0.58,20.1	0.000007
0.58,20.2	0.000007
0.58,20.3	0.000007
0.58,20.4	0.000007
0.58,20.5	0.000007
0.58,20.6	0.000007
0.58,20.7	0.000007
0.58,20.8	0.000007
0.58,20.9	0.000007
0.58,21.0	0.000007
0.58,21.1	0.000007
0.58,21.2	0.000007
0.58,21.3	0.000007
0.58,21.4	0.000007
0.58,21.5	0.000007
0.58,21.6	0.000007
0.58,21.7	0.000007
0.58,21.8	0.000007
0.58,21.9	0.000007
0.58,22.0	0.000007
0.58,22.1	0.000007
0.58,22.2	0.000007
0.58,22.3	0.000007
0.58,22.4	0.000007
0.58,22.5	0.000007
0.58,22.6	0.000007
0.58,22.7	0.000007
0.58,22.8	0.000007
0.58,22.9	0.000007

0.58,23.0	0.000007
0.58,23.1	0.000007
0.58,23.2	0.000007
0.58,23.3	0.000007
0.58,23.4	0.000007
0.58,23.5	0.000007
0.58,23.6	0.000007
0.58,23.7	0.000007
0.58,23.8	0.000007
0.58,23.9	0.000007
0.58,24.0	0.000007
0.58,24.1	0.000007
0.58,24.2	0.000007
0.58,24.3	0.000007
0.58,24.4	0.000007
0.58,24.5	0.000007
0.58,24.6	0.000007
0.58,24.7	0.000007
0.58,24.8	0.000007
0.58,24.9	0.000007
0.58,25.0	0.000007
0.58,25.1	0.000007
0.58,25.2	0.000007
0.58,25.3	0.000007
0.58,25.4	0.000007
0.58,25.5	0.000007
0.58,25.6	0.000007
0.58,25.7	0.000007
0.58,25.8	0.000007
0.58,25.9	0.000007
0.58,26.0	0.000007
0.58,26.1	0.000007
0.58,26.2	0.000007
0.58,26.3	0.000007
0.58,26.4	0.000007
0.58,26.5	0.000007
0.58,26.6	0.000007
0.58,26.7	0.000007
0.58,26.8	0.000007
0.58,26.9	0.000007
0.58,27.0	0.000007
0.58,27.1	0.000007

0.58,27.2	0.000007
0.58,27.3	0.000007
0.58,27.4	0.000007
0.58,27.5	0.000007
0.58,27.6	0.000007
0.58,27.7	0.000007
0.58,27.8	0.000007
0.58,27.9	0.000007
0.58,28.0	0.000007
0.58,28.1	0.000007
0.58,28.2	0.000007
0.58,28.3	0.000007
0.58,28.4	0.000007
0.58,28.5	0.000007
0.58,28.6	0.000007
0.58,28.7	0.000007
0.58,28.8	0.000007
0.58,28.9	0.000007
0.58,29.0	0.000007
0.58,29.1	0.000007
0.58,29.2	0.000007
0.58,29.3	0.000007
0.58,29.4	0.000007
0.58,29.5	0.000007
0.58,29.6	0.000007
0.58,29.7	0.000007
0.58,29.8	0.000007
0.58,29.9	0.000007
0.58,30.0	0.000007
0.58,30.1	0.000007
0.58,30.2	0.000007
0.58,30.3	0.000007
0.58,30.4	0.000007
0.58,30.5	0.000007
0.58,30.6	0.000007
0.58,30.7	0.000007
0.58,30.8	0.000007
0.58,30.9	0.000007
0.58,31.0	0.000007
0.58,31.1	0.000007
0.58,31.2	0.000007
0.58,31.3	0.000007

0.58,31.4	0.000007
0.58,31.5	0.000007
0.58,31.6	0.000007
0.58,31.7	0.000007
0.58,31.8	0.000007
0.58,31.9	0.000007
0.58,32.0	0.000007
0.58,32.1	0.000007
0.58,32.2	0.000007
0.58,32.3	0.000007
0.58,32.4	0.000007
0.58,32.5	0.000007
0.58,32.6	0.000007
0.58,32.7	0.000007
0.58,32.8	0.000007
0.58,32.9	0.000007
0.58,33.0	0.000007
0.58,33.1	0.000007
0.58,33.2	0.000007
0.58,33.3	0.000007
0.58,33.4	0.000007
0.58,33.5	0.000007
0.58,33.6	0.000007
0.58,33.7	0.000007
0.58,33.8	0.000007
0.58,33.9	0.000007
0.58,34.0	0.000007
0.58,34.1	0.000007
0.58,34.2	0.000007
0.58,34.3	0.000007
0.58,34.4	0.000007
0.58,34.5	0.000007
0.58,34.6	0.000007
0.58,34.7	0.000007
0.58,34.8	0.000007
0.58,34.9	0.000007
0.58,35.0	0.000007
0.58,35.1	0.000007
0.58,35.2	0.000007
0.58,35.3	0.000007
0.58,35.4	0.000007
0.58,35.5	0.000007

0.58,35.6	0.000007
0.58,35.7	0.000007
0.58,35.8	0.000007
0.58,35.9	0.000007
0.58,36.0	0.000007
0.58,36.1	0.000007
0.58,36.2	0.000007
0.58,36.3	0.000007
0.58,36.4	0.000007
0.58,36.5	0.000007
0.58,36.6	0.000007
0.58,36.7	0.000007
0.58,36.8	0.000007
0.58,36.9	0.000007
0.58,37.0	0.000007
0.58,37.1	0.000007
0.58,37.2	0.000007
0.58,37.3	0.000007
0.58,37.4	0.000007
0.58,37.5	0.000007
0.58,37.6	0.000007
0.58,37.7	0.000007
0.58,37.8	0.000007
0.58,37.9	0.000007
0.58,38.0	0.000007
0.58,38.1	0.000007
0.58,38.2	0.000007
0.58,38.3	0.000007
0.58,38.4	0.000007
0.58,38.5	0.000007
0.58,38.6	0.000007
0.58,38.7	0.000007
0.58,38.8	0.000007
0.58,38.9	0.000007
0.58,39.0	0.000007
0.58,39.1	0.000007
0.58,39.2	0.000007
0.58,39.3	0.000007
0.58,39.4	0.000007
0.58,39.5	0.000007
0.58,39.6	0.000007
0.58,39.7	0.000007

0.58,39.8	0.000007
0.58,39.9	0.000007
0.58,40.0	0.000007
0.58,40.1	0.000007
0.58,40.2	0.000007
0.58,40.3	0.000007
0.58,40.4	0.000007
0.58,40.5	0.000007
0.58,40.6	0.000007
0.58,40.7	0.000007
0.58,40.8	0.000007
0.58,40.9	0.000007
0.58,41.0	0.000007
0.58,41.1	0.000007
0.58,41.2	0.000007
0.58,41.3	0.000007
0.58,41.4	0.000007
0.58,41.5	0.000007
0.58,41.6	0.000007
0.58,41.7	0.000007
0.58,41.8	0.000007
0.58,41.9	0.000007
0.58,42.0	0.000007
0.58,42.1	0.000007
0.58,42.2	0.000007
0.58,42.3	0.000007
0.58,42.4	0.000007
0.58,42.5	0.000007
0.58,42.6	0.000007
0.58,42.7	0.000007
0.58,42.8	0.000007
0.58,42.9	0.000007
0.58,43.0	0.000007
0.58,43.1	0.000007
0.58,43.2	0.000007
0.58,43.3	0.000007
0.58,43.4	0.000007
0.58,43.5	0.000007
0.58,43.6	0.000007
0.58,43.7	0.000007
0.58,43.8	0.000007
0.58,43.9	0.000007

0.58,44.0	0.000007
0.58,44.1	0.000007
0.58,44.2	0.000007
0.58,44.3	0.000007
0.58,44.4	0.000007
0.58,44.5	0.000007
0.58,44.6	0.000007
0.58,44.7	0.000007
0.58,44.8	0.000007
0.58,44.9	0.000007
0.58,45.0	0.000007
0.58,45.1	0.000007
0.58,45.2	0.000007
0.58,45.3	0.000007
0.58,45.4	0.000007
0.58,45.5	0.000007
0.58,45.6	0.000007
0.58,45.7	0.000007
0.58,45.8	0.000007
0.58,45.9	0.000007
0.58,46.0	0.000007
0.58,46.1	0.000007
0.58,46.2	0.000007
0.58,46.3	0.000007
0.58,46.4	0.000007
0.58,46.5	0.000007
0.58,46.6	0.000007
0.58,46.7	0.000007
0.58,46.8	0.000007
0.58,46.9	0.000007
0.58,47.0	0.000007
0.58,47.1	0.000007
0.58,47.2	0.000007
0.58,47.3	0.000007
0.58,47.4	0.000007
0.58,47.5	0.000007
0.58,47.6	0.000007
0.58,47.7	0.000007
0.58,47.8	0.000007
0.58,47.9	0.000007
0.58,48.0	0.000007
0.58,48.1	0.000007

0.58,48.2	0.000007
0.58,48.3	0.000007
0.58,48.4	0.000007
0.58,48.5	0.000007
0.58,48.6	0.000007
0.58,48.7	0.000007
0.58,48.8	0.000007
0.58,48.9	0.000007
0.58,49.0	0.000007
0.58,49.1	0.000007
0.58,49.2	0.000007
0.58,49.3	0.000007
0.58,49.4	0.000007
0.58,49.5	0.000007
0.58,49.6	0.000007
0.58,49.7	0.000007
0.58,49.8	0.000007
0.58,49.9	0.000007
0.58,50.0	0.000007
0.58,50.1	0.000007
0.58,50.2	0.000007
0.58,50.3	0.000007
0.58,50.4	0.000007
0.58,50.5	0.000007
0.58,50.6	0.000007
0.58,50.7	0.000007
0.58,50.8	0.000007
0.58,50.9	0.000007
0.58,51.0	0.000007
0.58,51.1	0.000007
0.58,51.2	0.000007
0.58,51.3	0.000007
0.58,51.4	0.000007
0.58,51.5	0.000007
0.58,51.6	0.000007
0.58,51.7	0.000007
0.58,51.8	0.000007
0.58,51.9	0.000007
0.58,52.0	0.000007
0.58,52.1	0.000007
0.58,52.2	0.000007
0.58,52.3	0.000007

0.58,52.4	0.000007
0.58,52.5	0.000007
0.58,52.6	0.000007
0.58,52.7	0.000007
0.58,52.8	0.000007
0.58,52.9	0.000007
0.58,53.0	0.000007
0.58,53.1	0.000007
0.58,53.2	0.000007
0.58,53.3	0.000007
0.58,53.4	0.000007
0.58,53.5	0.000007
0.58,53.6	0.000007
0.58,53.7	0.000007
0.58,53.8	0.000007
0.58,53.9	0.000007
0.58,54.0	0.000007
0.58,54.1	0.000007
0.58,54.2	0.000007
0.58,54.3	0.000007
0.58,54.4	0.000007
0.58,54.5	0.000007
0.58,54.6	0.000007
0.58,54.7	0.000007
0.58,54.8	0.000007
0.58,54.9	0.000007
0.58,55.0	0.000007
0.58,55.1	0.000007
0.58,55.2	0.000007
0.58,55.3	0.000007
0.58,55.4	0.000007
0.58,55.5	0.000007
0.58,55.6	0.000007
0.58,55.7	0.000007
0.58,55.8	0.000007
0.58,55.9	0.000007
0.58,56.0	0.000007
0.58,56.1	0.000007
0.58,56.2	0.000007
0.58,56.3	0.000007
0.58,56.4	0.000007
0.58,56.5	0.000007

0.58,56.6	0.000007
0.58,56.7	0.000007
0.58,56.8	0.000007
0.58,56.9	0.000007
0.58,57.0	0.000007
0.58,57.1	0.000007
0.58,57.2	0.000007
0.58,57.3	0.000007
0.58,57.4	0.000007
0.58,57.5	0.000007
0.58,57.6	0.000007
0.58,57.7	0.000007
0.58,57.8	0.000007
0.58,57.9	0.000007
0.58,58.0	0.000007
0.58,58.1	0.000007
0.58,58.2	0.000007
0.58,58.3	0.000007
0.58,58.4	0.000007
0.58,58.5	0.000007
0.58,58.6	0.000007
0.58,58.7	0.000007
0.58,58.8	0.000007
0.58,58.9	0.000007
0.58,59.0	0.000007
0.58,59.1	0.000007
0.58,59.2	0.000007
0.58,59.3	0.000007
0.58,59.4	0.000007
0.58,59.5	0.000007
0.58,59.6	0.000007
0.58,59.7	0.000007
0.58,59.8	0.000007
0.58,59.9	0.000007
0.58,60.0	0.000007
0.58,60.1	0.000007
0.58,60.2	0.000007
0.58,60.3	0.000007
0.58,60.4	0.000007
0.58,60.5	0.000007
0.58,60.6	0.000007
0.58,60.7	0.000007

0.58,60.8	0.000007
0.58,60.9	0.000007
0.58,61.0	0.000007
0.58,61.1	0.000007
0.58,61.2	0.000007
0.58,61.3	0.000007
0.58,61.4	0.000007
0.58,61.5	0.000007
0.58,61.6	0.000007
0.58,61.7	0.000007
0.58,61.8	0.000007
0.58,61.9	0.000007
0.58,62.0	0.000007
0.58,62.1	0.000007
0.58,62.2	0.000007
0.58,62.3	0.000007
0.58,62.4	0.000007
0.58,62.5	0.000007
0.58,62.6	0.000007
0.58,62.7	0.000007
0.58,62.8	0.000007
0.58,62.9	0.000007
0.58,63.0	0.000007
0.58,63.1	0.000007
0.58,63.2	0.000007
0.58,63.3	0.000007
0.58,63.4	0.000007
0.58,63.5	0.000007
0.58,63.6	0.000007
0.58,63.7	0.000007
0.58,63.8	0.000007
0.58,63.9	0.000007
0.58,64.0	0.000007
0.58,64.1	0.000007
0.58,64.2	0.000007
0.58,64.3	0.000007
0.58,64.4	0.000007
0.58,64.5	0.000007
0.58,64.6	0.000007
0.58,64.7	0.000007
0.58,64.8	0.000007
0.58,64.9	0.000007

0.58,65.0	0.000007
0.58,65.1	0.000007
0.58,65.2	0.000007
0.58,65.3	0.000007
0.58,65.4	0.000007
0.58,65.5	0.000007
0.58,65.6	0.000007
0.58,65.7	0.000007
0.58,65.8	0.000007
0.58,65.9	0.000007
0.58,66.0	0.000007
0.58,66.1	0.000007
0.58,66.2	0.000007
0.58,66.3	0.000007
0.58,66.4	0.000007
0.58,66.5	0.000007
0.58,66.6	0.000007
0.58,66.7	0.000007
0.58,66.8	0.000007
0.58,66.9	0.000007
0.58,67.0	0.000007
0.58,67.1	0.000007
0.58,67.2	0.000007
0.58,67.3	0.000007
0.58,67.4	0.000007
0.58,67.5	0.000007
0.58,67.6	0.000007
0.58,67.7	0.000007
0.58,67.8	0.000007
0.58,67.9	0.000007
0.58,68.0	0.000007
0.58,68.1	0.000007
0.58,68.2	0.000007
0.58,68.3	0.000007
0.58,68.4	0.000007
0.58,68.5	0.000007
0.58,68.6	0.000007
0.58,68.7	0.000007
0.58,68.8	0.000007
0.58,68.9	0.000007
0.58,69.0	0.000007
0.58,69.1	0.000007

0.58,69.2	0.000007
0.58,69.3	0.000007
0.58,69.4	0.000007
0.58,69.5	0.000007
0.58,69.6	0.000007
0.58,69.7	0.000007
0.58,69.8	0.000007
0.58,69.9	0.000007
0.58,70.0	0.000007
0.58,70.1	0.000007
0.58,70.2	0.000007
0.58,70.3	0.000007
0.58,70.4	0.000007
0.58,70.5	0.000007
0.58,70.6	0.000007
0.58,70.7	0.000007
0.58,70.8	0.000007
0.58,70.9	0.000007
0.58,71.0	0.000007
0.58,71.1	0.000007
0.58,71.2	0.000007
0.58,71.3	0.000007
0.58,71.4	0.000007
0.58,71.5	0.000007
0.58,71.6	0.000007
0.58,71.7	0.000007
0.58,71.8	0.000007
0.58,71.9	0.000007
0.58,72.0	0.000007
0.58,72.1	0.000007
0.58,72.2	0.000007
0.58,72.3	0.000007
0.58,72.4	0.000007
0.58,72.5	0.000007
0.58,72.6	0.000007
0.58,72.7	0.000007
0.58,72.8	0.000007
0.58,72.9	0.000007
0.58,73.0	0.000007
0.58,73.1	0.000007
0.58,73.2	0.000007
0.58,73.3	0.000007

0.58,73.4	0.000007
0.58,73.5	0.000007
0.58,73.6	0.000007
0.58,73.7	0.000007
0.58,73.8	0.000007
0.58,73.9	0.000007
0.58,74.0	0.000007
0.58,74.1	0.000007
0.58,74.2	0.000007
0.58,74.3	0.000007
0.58,74.4	0.000007
0.58,74.5	0.000007
0.58,74.6	0.000007
0.58,74.7	0.000007
0.58,74.8	0.000007
0.58,74.9	0.000007
0.58,75.0	0.000007
0.58,75.1	0.000007
0.58,75.2	0.000007
0.58,75.3	0.000007
0.58,75.4	0.000007
0.58,75.5	0.000007
0.58,75.6	0.000007
0.58,75.7	0.000007
0.58,75.8	0.000007
0.58,75.9	0.000007
0.58,76.0	0.000007
0.58,76.1	0.000007
0.58,76.2	0.000007
0.58,76.3	0.000007
0.58,76.4	0.000007
0.58,76.5	0.000007
0.58,76.6	0.000007
0.58,76.7	0.000008
0.58,76.8	0.000008
0.58,76.9	0.000008
0.58,77.0	0.000008
0.58,77.1	0.000008
0.58,77.2	0.000008
0.58,77.3	0.000008
0.58,77.4	0.000008
0.58,77.5	0.000008

0.58,77.6	0.000008
0.58,77.7	0.000008
0.58,77.8	0.000008
0.58,77.9	0.000008
0.58,78.0	0.000008
0.58,78.1	0.000008
0.58,78.2	0.000008
0.58,78.3	0.000008
0.58,78.4	0.000008
0.58,78.5	0.000008
0.58,78.6	0.000008
0.58,78.7	0.000008
0.58,78.8	0.000008
0.58,78.9	0.000008
0.58,79.0	0.000008
0.58,79.1	0.000009
0.58,79.2	0.000009
0.58,79.3	0.000009
0.58,79.4	0.000009
0.58,79.5	0.000009
0.58,79.6	0.00001
0.58,79.7	0.00001
0.58,79.8	0.000011
0.58,79.9	0.000012
0.58,80.0	0.000012
0.58,80.1	0.000012
0.58,80.2	0.000012
0.58,80.3	0.000012
0.58,80.4	0.000012
0.58,80.5	0.000012
0.58,80.6	0.000012
0.58,80.7	0.000012
0.58,80.8	0.000012
0.58,80.9	0.000012
0.58,81.0	0.000012
0.58,81.1	0.000013
0.58,81.2	0.000013
0.58,81.3	0.000022
0.58,81.4	0.000051
0.58,81.5	0.000083
0.58,81.6	0.000119
0.58,81.7	0.000141

0.58,81.8	0.000154
0.58,81.9	0.000168
0.58,82.0	0.000178
0.58,82.1	0.000185
0.58,82.2	0.000188
0.58,82.3	0.000194
0.58,82.4	0.000202
0.58,82.5	0.000217
0.58,82.6	0.000233
0.58,82.7	0.000239
0.58,82.8	0.00025
0.58,82.9	0.000274
0.58,83.0	0.000297
0.58,83.1	0.000311
0.58,83.2	0.000329
0.58,83.3	0.000343
0.58,83.4	0.000348
0.58,83.5	0.000354
0.58,83.6	0.000357
0.58,83.7	0.000363
0.58,83.8	0.000365
0.58,83.9	0.000368
0.58,84.0	0.00037
0.58,84.1	0.000373
0.58,84.2	0.000389
0.58,84.3	0.000407
0.58,84.4	0.000416
0.58,84.5	0.000419
0.58,84.6	0.000426
0.58,84.7	0.00044
0.58,84.8	0.000452
0.58,84.9	0.000454
0.58,85.0	0.000454
0.58,85.1	0.000458
0.58,85.2	0.000463
0.58,85.3	0.000467
0.58,85.4	0.000469
0.58,85.5	0.000469
0.58,85.6	0.000473
0.58,85.7	0.000473
0.58,85.8	0.000473
0.58,85.9	0.000474

0.58,86.0	0.000474
0.58,86.1	0.000475
0.58,86.2	0.000484
0.58,86.3	0.000513
0.58,86.4	0.0006
0.58,86.5	0.000778
0.58,86.6	0.000886
0.58,86.7	0.000925
0.58,86.8	0.000934
0.58,86.9	0.000939
0.58,87.0	0.000957
0.58,87.1	0.000987
0.58,87.2	0.001003
0.58,87.3	0.001003
0.58,87.4	0.001003
0.58,87.5	0.001008
0.58,87.6	0.001013
0.58,87.7	0.001035
0.58,87.8	0.001035
0.58,87.9	0.001035
0.58,88.0	0.001058
0.58,88.1	0.001088
0.58,88.2	0.001094
0.58,88.3	0.00112
0.58,88.4	0.00114
0.58,88.5	0.001182
0.58,88.6	0.001189
0.58,88.7	0.001197
0.58,88.8	0.001197
0.58,88.9	0.001204
0.58,89.0	0.001212
0.58,89.1	0.001227
0.58,89.2	0.001243
0.58,89.3	0.001251
0.58,89.4	0.001251
0.58,89.5	0.001285
0.58,89.6	0.001311
0.58,89.7	0.00133
0.58,89.8	0.001348
0.58,89.9	0.001368
0.58,90.0	0.001368
0.58,90.1	0.001378

0.58,90.2	0.001388
0.58,90.3	0.001398
0.58,90.4	0.001398
0.58,90.5	0.001418
0.58,90.6	0.001462
0.58,90.7	0.001473
0.58,90.8	0.001473
0.58,90.9	0.001484
0.58,91.0	0.001532
0.58,91.1	0.001544
0.58,91.2	0.001557
0.58,91.3	0.00157
0.58,91.4	0.00157
0.58,91.5	0.001582
0.58,91.6	0.001623
0.58,91.7	0.001637
0.58,91.8	0.001637
0.58,91.9	0.00168
0.58,92.0	0.00171
0.58,92.1	0.001725
0.58,92.2	0.00179
0.58,92.3	0.001877
0.58,92.4	0.001877
0.58,92.5	0.001877
0.58,92.6	0.001877
0.58,92.7	0.001877
0.58,92.8	0.001877
0.58,92.9	0.001934
0.58,93.0	0.001934
0.58,93.1	0.001954
0.58,93.2	0.001954
0.58,93.3	0.001995
0.58,93.4	0.002016
0.58,93.5	0.002037
0.58,93.6	0.002037
0.58,93.7	0.002037
0.58,93.8	0.002059
0.58,93.9	0.002059
0.58,94.0	0.002081
0.58,94.1	0.002081
0.58,94.2	0.002128
0.58,94.3	0.002151

0.58,94.4	0.00228
0.58,94.5	0.002364
0.58,94.6	0.002364
0.58,94.7	0.002364
0.58,94.8	0.002487
0.58,94.9	0.002487
0.58,95.0	0.002487
0.58,95.1	0.002487
0.58,95.2	0.002487
0.58,95.3	0.002487
0.58,95.4	0.002487
0.58,95.5	0.002487
0.58,95.6	0.002487
0.58,95.7	0.002487
0.58,95.8	0.002487
0.58,95.9	0.002487
0.58,96.0	0.002487
0.58,96.1	0.002487
0.58,96.2	0.002487
0.58,96.3	0.002483
0.58,96.4	0.002483
0.58,96.5	0.002481
0.58,96.6	0.002481
0.58,96.7	0.002481
0.58,96.8	0.002481
0.58,96.9	0.002481
0.58,97.0	0.002479
0.58,97.1	0.002479
0.58,97.2	0.002479
0.58,97.3	0.002477
0.58,97.4	0.002475
0.58,97.5	0.002475
0.58,97.6	0.002475
0.58,97.7	0.002475
0.58,97.8	0.002469
0.58,97.9	0.00248
0.58,98.0	0.002448
0.58,98.1	0.002492
0.58,98.2	0.002492
0.58,98.3	0.002492
0.58,98.4	0.00255
0.58,98.5	0.002548

0.58,98.6	0.002548
0.58,98.7	0.002536
0.58,98.8	0.002536
0.58,98.9	0.002526
0.58,99.0	0.002518
0.58,99.1	0.002514
0.58,99.2	0.002514
0.58,99.3	0.002509
0.58,99.4	0.002404
0.58,99.5	0.002098
0.58,99.6	0.001967
0.58,99.7	0.001311
0.58,99.8	0.001311
0.58,99.9	0
0.58,100.0	0
0.59,0.0	0.000018
0.59,0.1	0.000018
0.59,0.2	0.000018
0.59,0.3	0.000018
0.59,0.4	0.000018
0.59,0.5	0.000018
0.59,0.6	0.000018
0.59,0.7	0.000018
0.59,0.8	0.000018
0.59,0.9	0.000018
0.59,1.0	0.000018
0.59,1.1	0.000018
0.59,1.2	0.000018
0.59,1.3	0.000018
0.59,1.4	0.000018
0.59,1.5	0.000018
0.59,1.6	0.000018
0.59,1.7	0.000018
0.59,1.8	0.000018
0.59,1.9	0.000018
0.59,2.0	0.000018
0.59,2.1	0.000018
0.59,2.2	0.000018
0.59,2.3	0.000018
0.59,2.4	0.000018
0.59,2.5	0.000018
0.59,2.6	0.000018

0.59,2.7	0.000018
0.59,2.8	0.000018
0.59,2.9	0.000018
0.59,3.0	0.000018
0.59,3.1	0.000018
0.59,3.2	0.000018
0.59,3.3	0.000018
0.59,3.4	0.000018
0.59,3.5	0.000018
0.59,3.6	0.000018
0.59,3.7	0.000018
0.59,3.8	0.000018
0.59,3.9	0.000018
0.59,4.0	0.000018
0.59,4.1	0.000018
0.59,4.2	0.000018
0.59,4.3	0.000018
0.59,4.4	0.000018
0.59,4.5	0.000018
0.59,4.6	0.000018
0.59,4.7	0.000018
0.59,4.8	0.000018
0.59,4.9	0.000018
0.59,5.0	0.000018
0.59,5.1	0.000018
0.59,5.2	0.000018
0.59,5.3	0.000018
0.59,5.4	0.000018
0.59,5.5	0.000018
0.59,5.6	0.000018
0.59,5.7	0.000018
0.59,5.8	0.000018
0.59,5.9	0.000018
0.59,6.0	0.000018
0.59,6.1	0.000018
0.59,6.2	0.000018
0.59,6.3	0.000018
0.59,6.4	0.000018
0.59,6.5	0.000018
0.59,6.6	0.000018
0.59,6.7	0.000018
0.59,6.8	0.000018

0.59,6.9	0.000018
0.59,7.0	0.000018
0.59,7.1	0.000018
0.59,7.2	0.000018
0.59,7.3	0.000018
0.59,7.4	0.000018
0.59,7.5	0.000018
0.59,7.6	0.000018
0.59,7.7	0.000018
0.59,7.8	0.000018
0.59,7.9	0.000018
0.59,8.0	0.000018
0.59,8.1	0.000018
0.59,8.2	0.000018
0.59,8.3	0.000018
0.59,8.4	0.000018
0.59,8.5	0.000018
0.59,8.6	0.000018
0.59,8.7	0.000018
0.59,8.8	0.000018
0.59,8.9	0.000018
0.59,9.0	0.000018
0.59,9.1	0.000018
0.59,9.2	0.000018
0.59,9.3	0.000018
0.59,9.4	0.000018
0.59,9.5	0.000018
0.59,9.6	0.000018
0.59,9.7	0.000018
0.59,9.8	0.000018
0.59,9.9	0.000018
0.59,10.0	0.000018
0.59,10.1	0.000018
0.59,10.2	0.000018
0.59,10.3	0.000018
0.59,10.4	0.000018
0.59,10.5	0.000018
0.59,10.6	0.000018
0.59,10.7	0.000018
0.59,10.8	0.000018
0.59,10.9	0.000018
0.59,11.0	0.000018

0.59,11.1	0.000018
0.59,11.2	0.000018
0.59,11.3	0.000018
0.59,11.4	0.000018
0.59,11.5	0.000018
0.59,11.6	0.000018
0.59,11.7	0.000018
0.59,11.8	0.000018
0.59,11.9	0.000018
0.59,12.0	0.000018
0.59,12.1	0.000018
0.59,12.2	0.000018
0.59,12.3	0.000018
0.59,12.4	0.000018
0.59,12.5	0.000018
0.59,12.6	0.000018
0.59,12.7	0.000018
0.59,12.8	0.000018
0.59,12.9	0.000018
0.59,13.0	0.000018
0.59,13.1	0.000018
0.59,13.2	0.000018
0.59,13.3	0.000018
0.59,13.4	0.000018
0.59,13.5	0.000018
0.59,13.6	0.000018
0.59,13.7	0.000018
0.59,13.8	0.000018
0.59,13.9	0.000018
0.59,14.0	0.000018
0.59,14.1	0.000018
0.59,14.2	0.000018
0.59,14.3	0.000018
0.59,14.4	0.000018
0.59,14.5	0.000018
0.59,14.6	0.000018
0.59,14.7	0.000018
0.59,14.8	0.000018
0.59,14.9	0.000018
0.59,15.0	0.000018
0.59,15.1	0.000018
0.59,15.2	0.000018

0.59,15.3	0.000018
0.59,15.4	0.000018
0.59,15.5	0.000018
0.59,15.6	0.000018
0.59,15.7	0.000018
0.59,15.8	0.000018
0.59,15.9	0.000018
0.59,16.0	0.000018
0.59,16.1	0.000018
0.59,16.2	0.000018
0.59,16.3	0.000018
0.59,16.4	0.000018
0.59,16.5	0.000018
0.59,16.6	0.000018
0.59,16.7	0.000018
0.59,16.8	0.000018
0.59,16.9	0.000018
0.59,17.0	0.000018
0.59,17.1	0.000018
0.59,17.2	0.000018
0.59,17.3	0.000018
0.59,17.4	0.000018
0.59,17.5	0.000018
0.59,17.6	0.000018
0.59,17.7	0.000018
0.59,17.8	0.000018
0.59,17.9	0.000018
0.59,18.0	0.000018
0.59,18.1	0.000018
0.59,18.2	0.000018
0.59,18.3	0.000018
0.59,18.4	0.000018
0.59,18.5	0.000018
0.59,18.6	0.000018
0.59,18.7	0.000018
0.59,18.8	0.000018
0.59,18.9	0.000018
0.59,19.0	0.000018
0.59,19.1	0.000018
0.59,19.2	0.000018
0.59,19.3	0.000018
0.59,19.4	0.000018

0.59,19.5	0.000018
0.59,19.6	0.000018
0.59,19.7	0.000018
0.59,19.8	0.000018
0.59,19.9	0.000018
0.59,20.0	0.000018
0.59,20.1	0.000018
0.59,20.2	0.000018
0.59,20.3	0.000018
0.59,20.4	0.000018
0.59,20.5	0.000018
0.59,20.6	0.000018
0.59,20.7	0.000018
0.59,20.8	0.000018
0.59,20.9	0.000018
0.59,21.0	0.000018
0.59,21.1	0.000018
0.59,21.2	0.000018
0.59,21.3	0.000018
0.59,21.4	0.000018
0.59,21.5	0.000018
0.59,21.6	0.000018
0.59,21.7	0.000018
0.59,21.8	0.000018
0.59,21.9	0.000018
0.59,22.0	0.000018
0.59,22.1	0.000018
0.59,22.2	0.000018
0.59,22.3	0.000018
0.59,22.4	0.000018
0.59,22.5	0.000018
0.59,22.6	0.000018
0.59,22.7	0.000018
0.59,22.8	0.000018
0.59,22.9	0.000018
0.59,23.0	0.000018
0.59,23.1	0.000018
0.59,23.2	0.000018
0.59,23.3	0.000018
0.59,23.4	0.000018
0.59,23.5	0.000018
0.59,23.6	0.000018

0.59,23.7	0.000018
0.59,23.8	0.000018
0.59,23.9	0.000018
0.59,24.0	0.000018
0.59,24.1	0.000018
0.59,24.2	0.000018
0.59,24.3	0.000018
0.59,24.4	0.000018
0.59,24.5	0.000018
0.59,24.6	0.000018
0.59,24.7	0.000018
0.59,24.8	0.000018
0.59,24.9	0.000018
0.59,25.0	0.000018
0.59,25.1	0.000018
0.59,25.2	0.000018
0.59,25.3	0.000018
0.59,25.4	0.000018
0.59,25.5	0.000018
0.59,25.6	0.000018
0.59,25.7	0.000018
0.59,25.8	0.000018
0.59,25.9	0.000018
0.59,26.0	0.000018
0.59,26.1	0.000018
0.59,26.2	0.000018
0.59,26.3	0.000018
0.59,26.4	0.000018
0.59,26.5	0.000018
0.59,26.6	0.000018
0.59,26.7	0.000018
0.59,26.8	0.000018
0.59,26.9	0.000018
0.59,27.0	0.000018
0.59,27.1	0.000018
0.59,27.2	0.000018
0.59,27.3	0.000018
0.59,27.4	0.000018
0.59,27.5	0.000018
0.59,27.6	0.000018
0.59,27.7	0.000018
0.59,27.8	0.000018

0.59,27.9	0.000018
0.59,28.0	0.000018
0.59,28.1	0.000018
0.59,28.2	0.000018
0.59,28.3	0.000018
0.59,28.4	0.000018
0.59,28.5	0.000018
0.59,28.6	0.000018
0.59,28.7	0.000018
0.59,28.8	0.000018
0.59,28.9	0.000018
0.59,29.0	0.000018
0.59,29.1	0.000018
0.59,29.2	0.000018
0.59,29.3	0.000018
0.59,29.4	0.000018
0.59,29.5	0.000018
0.59,29.6	0.000018
0.59,29.7	0.000018
0.59,29.8	0.000018
0.59,29.9	0.000018
0.59,30.0	0.000018
0.59,30.1	0.000018
0.59,30.2	0.000018
0.59,30.3	0.000018
0.59,30.4	0.000018
0.59,30.5	0.000018
0.59,30.6	0.000018
0.59,30.7	0.000018
0.59,30.8	0.000018
0.59,30.9	0.000018
0.59,31.0	0.000018
0.59,31.1	0.000018
0.59,31.2	0.000018
0.59,31.3	0.000018
0.59,31.4	0.000018
0.59,31.5	0.000018
0.59,31.6	0.000018
0.59,31.7	0.000018
0.59,31.8	0.000018
0.59,31.9	0.000018
0.59,32.0	0.000018

0.59,32.1	0.000018
0.59,32.2	0.000018
0.59,32.3	0.000018
0.59,32.4	0.000018
0.59,32.5	0.000018
0.59,32.6	0.000018
0.59,32.7	0.000018
0.59,32.8	0.000018
0.59,32.9	0.000018
0.59,33.0	0.000018
0.59,33.1	0.000018
0.59,33.2	0.000018
0.59,33.3	0.000018
0.59,33.4	0.000018
0.59,33.5	0.000018
0.59,33.6	0.000018
0.59,33.7	0.000018
0.59,33.8	0.000018
0.59,33.9	0.000018
0.59,34.0	0.000018
0.59,34.1	0.000018
0.59,34.2	0.000018
0.59,34.3	0.000018
0.59,34.4	0.000018
0.59,34.5	0.000018
0.59,34.6	0.000018
0.59,34.7	0.000018
0.59,34.8	0.000018
0.59,34.9	0.000018
0.59,35.0	0.000018
0.59,35.1	0.000018
0.59,35.2	0.000018
0.59,35.3	0.000018
0.59,35.4	0.000018
0.59,35.5	0.000018
0.59,35.6	0.000018
0.59,35.7	0.000018
0.59,35.8	0.000018
0.59,35.9	0.000018
0.59,36.0	0.000018
0.59,36.1	0.000018
0.59,36.2	0.000018

0.59,36.3	0.000018
0.59,36.4	0.000018
0.59,36.5	0.000018
0.59,36.6	0.000018
0.59,36.7	0.000018
0.59,36.8	0.000018
0.59,36.9	0.000018
0.59,37.0	0.000018
0.59,37.1	0.000018
0.59,37.2	0.000018
0.59,37.3	0.000018
0.59,37.4	0.000018
0.59,37.5	0.000018
0.59,37.6	0.000018
0.59,37.7	0.000018
0.59,37.8	0.000018
0.59,37.9	0.000018
0.59,38.0	0.000018
0.59,38.1	0.000018
0.59,38.2	0.000018
0.59,38.3	0.000018
0.59,38.4	0.000018
0.59,38.5	0.000018
0.59,38.6	0.000018
0.59,38.7	0.000018
0.59,38.8	0.000018
0.59,38.9	0.000018
0.59,39.0	0.000018
0.59,39.1	0.000018
0.59,39.2	0.000018
0.59,39.3	0.000018
0.59,39.4	0.000018
0.59,39.5	0.000018
0.59,39.6	0.000018
0.59,39.7	0.000018
0.59,39.8	0.000018
0.59,39.9	0.000018
0.59,40.0	0.000018
0.59,40.1	0.000018
0.59,40.2	0.000018
0.59,40.3	0.000018
0.59,40.4	0.000018

0.59,40.5	0.000018
0.59,40.6	0.000018
0.59,40.7	0.000018
0.59,40.8	0.000018
0.59,40.9	0.000018
0.59,41.0	0.000018
0.59,41.1	0.000018
0.59,41.2	0.000018
0.59,41.3	0.000018
0.59,41.4	0.000018
0.59,41.5	0.000018
0.59,41.6	0.000018
0.59,41.7	0.000018
0.59,41.8	0.000018
0.59,41.9	0.000018
0.59,42.0	0.000018
0.59,42.1	0.000018
0.59,42.2	0.000018
0.59,42.3	0.000018
0.59,42.4	0.000018
0.59,42.5	0.000018
0.59,42.6	0.000018
0.59,42.7	0.000018
0.59,42.8	0.000018
0.59,42.9	0.000018
0.59,43.0	0.000018
0.59,43.1	0.000018
0.59,43.2	0.000018
0.59,43.3	0.000018
0.59,43.4	0.000018
0.59,43.5	0.000018
0.59,43.6	0.000018
0.59,43.7	0.000018
0.59,43.8	0.000018
0.59,43.9	0.000018
0.59,44.0	0.000018
0.59,44.1	0.000018
0.59,44.2	0.000018
0.59,44.3	0.000018
0.59,44.4	0.000018
0.59,44.5	0.000018
0.59,44.6	0.000018

0.59,44.7	0.000018
0.59,44.8	0.000018
0.59,44.9	0.000018
0.59,45.0	0.000018
0.59,45.1	0.000018
0.59,45.2	0.000018
0.59,45.3	0.000018
0.59,45.4	0.000018
0.59,45.5	0.000018
0.59,45.6	0.000018
0.59,45.7	0.000018
0.59,45.8	0.000018
0.59,45.9	0.000018
0.59,46.0	0.000018
0.59,46.1	0.000018
0.59,46.2	0.000018
0.59,46.3	0.000018
0.59,46.4	0.000018
0.59,46.5	0.000018
0.59,46.6	0.000018
0.59,46.7	0.000018
0.59,46.8	0.000018
0.59,46.9	0.000018
0.59,47.0	0.000018
0.59,47.1	0.000018
0.59,47.2	0.000018
0.59,47.3	0.000018
0.59,47.4	0.000018
0.59,47.5	0.000018
0.59,47.6	0.000018
0.59,47.7	0.000018
0.59,47.8	0.000018
0.59,47.9	0.000018
0.59,48.0	0.000018
0.59,48.1	0.000018
0.59,48.2	0.000018
0.59,48.3	0.000018
0.59,48.4	0.000018
0.59,48.5	0.000018
0.59,48.6	0.000018
0.59,48.7	0.000018
0.59,48.8	0.000018

0.59,48.9	0.000018
0.59,49.0	0.000018
0.59,49.1	0.000018
0.59,49.2	0.000018
0.59,49.3	0.000018
0.59,49.4	0.000018
0.59,49.5	0.000018
0.59,49.6	0.000018
0.59,49.7	0.000018
0.59,49.8	0.000018
0.59,49.9	0.000018
0.59,50.0	0.000018
0.59,50.1	0.000018
0.59,50.2	0.000018
0.59,50.3	0.000018
0.59,50.4	0.000018
0.59,50.5	0.000018
0.59,50.6	0.000018
0.59,50.7	0.000018
0.59,50.8	0.000018
0.59,50.9	0.000018
0.59,51.0	0.000018
0.59,51.1	0.000018
0.59,51.2	0.000018
0.59,51.3	0.000018
0.59,51.4	0.000018
0.59,51.5	0.000018
0.59,51.6	0.000018
0.59,51.7	0.000018
0.59,51.8	0.000018
0.59,51.9	0.000018
0.59,52.0	0.000018
0.59,52.1	0.000018
0.59,52.2	0.000018
0.59,52.3	0.000018
0.59,52.4	0.000018
0.59,52.5	0.000018
0.59,52.6	0.000018
0.59,52.7	0.000018
0.59,52.8	0.000018
0.59,52.9	0.000018
0.59,53.0	0.000018

0.59,53.1	0.000018
0.59,53.2	0.000018
0.59,53.3	0.000018
0.59,53.4	0.000018
0.59,53.5	0.000018
0.59,53.6	0.000018
0.59,53.7	0.000018
0.59,53.8	0.000018
0.59,53.9	0.000018
0.59,54.0	0.000018
0.59,54.1	0.000018
0.59,54.2	0.000018
0.59,54.3	0.000018
0.59,54.4	0.000018
0.59,54.5	0.000018
0.59,54.6	0.000018
0.59,54.7	0.000018
0.59,54.8	0.000018
0.59,54.9	0.000018
0.59,55.0	0.000018
0.59,55.1	0.000018
0.59,55.2	0.000018
0.59,55.3	0.000018
0.59,55.4	0.000018
0.59,55.5	0.000018
0.59,55.6	0.000018
0.59,55.7	0.000018
0.59,55.8	0.000018
0.59,55.9	0.000018
0.59,56.0	0.000018
0.59,56.1	0.000018
0.59,56.2	0.000018
0.59,56.3	0.000018
0.59,56.4	0.000018
0.59,56.5	0.000018
0.59,56.6	0.000018
0.59,56.7	0.000018
0.59,56.8	0.000018
0.59,56.9	0.000018
0.59,57.0	0.000018
0.59,57.1	0.000018
0.59,57.2	0.000018

0.59,57.3	0.000018
0.59,57.4	0.000018
0.59,57.5	0.000018
0.59,57.6	0.000018
0.59,57.7	0.000018
0.59,57.8	0.000018
0.59,57.9	0.000018
0.59,58.0	0.000018
0.59,58.1	0.000018
0.59,58.2	0.000018
0.59,58.3	0.000018
0.59,58.4	0.000018
0.59,58.5	0.000018
0.59,58.6	0.000018
0.59,58.7	0.000018
0.59,58.8	0.000018
0.59,58.9	0.000018
0.59,59.0	0.000018
0.59,59.1	0.000018
0.59,59.2	0.000018
0.59,59.3	0.000018
0.59,59.4	0.000018
0.59,59.5	0.000018
0.59,59.6	0.000018
0.59,59.7	0.000018
0.59,59.8	0.000018
0.59,59.9	0.000018
0.59,60.0	0.000018
0.59,60.1	0.000018
0.59,60.2	0.000018
0.59,60.3	0.000018
0.59,60.4	0.000018
0.59,60.5	0.000018
0.59,60.6	0.000018
0.59,60.7	0.000018
0.59,60.8	0.000018
0.59,60.9	0.000018
0.59,61.0	0.000018
0.59,61.1	0.000018
0.59,61.2	0.000018
0.59,61.3	0.000018
0.59,61.4	0.000018

0.59,61.5	0.000018
0.59,61.6	0.000018
0.59,61.7	0.000018
0.59,61.8	0.000018
0.59,61.9	0.000018
0.59,62.0	0.000018
0.59,62.1	0.000018
0.59,62.2	0.000018
0.59,62.3	0.000018
0.59,62.4	0.000018
0.59,62.5	0.000018
0.59,62.6	0.000018
0.59,62.7	0.000018
0.59,62.8	0.000018
0.59,62.9	0.000018
0.59,63.0	0.000018
0.59,63.1	0.000018
0.59,63.2	0.000018
0.59,63.3	0.000018
0.59,63.4	0.000018
0.59,63.5	0.000018
0.59,63.6	0.000018
0.59,63.7	0.000018
0.59,63.8	0.000018
0.59,63.9	0.000018
0.59,64.0	0.000018
0.59,64.1	0.000018
0.59,64.2	0.000018
0.59,64.3	0.000018
0.59,64.4	0.000018
0.59,64.5	0.000018
0.59,64.6	0.000018
0.59,64.7	0.000018
0.59,64.8	0.000018
0.59,64.9	0.000018
0.59,65.0	0.000018
0.59,65.1	0.000018
0.59,65.2	0.000018
0.59,65.3	0.000018
0.59,65.4	0.000018
0.59,65.5	0.000018
0.59,65.6	0.000018

0.59,65.7	0.000018
0.59,65.8	0.000018
0.59,65.9	0.000018
0.59,66.0	0.000018
0.59,66.1	0.000018
0.59,66.2	0.000018
0.59,66.3	0.000018
0.59,66.4	0.000018
0.59,66.5	0.000018
0.59,66.6	0.000018
0.59,66.7	0.000018
0.59,66.8	0.000018
0.59,66.9	0.000018
0.59,67.0	0.000018
0.59,67.1	0.000018
0.59,67.2	0.000018
0.59,67.3	0.000018
0.59,67.4	0.000018
0.59,67.5	0.000018
0.59,67.6	0.000018
0.59,67.7	0.000018
0.59,67.8	0.000018
0.59,67.9	0.000018
0.59,68.0	0.000018
0.59,68.1	0.000018
0.59,68.2	0.000018
0.59,68.3	0.000018
0.59,68.4	0.000018
0.59,68.5	0.000018
0.59,68.6	0.000018
0.59,68.7	0.000018
0.59,68.8	0.000018
0.59,68.9	0.000018
0.59,69.0	0.000018
0.59,69.1	0.000018
0.59,69.2	0.000018
0.59,69.3	0.000018
0.59,69.4	0.000018
0.59,69.5	0.000018
0.59,69.6	0.000018
0.59,69.7	0.000018
0.59,69.8	0.000018

0.59,69.9	0.000018
0.59,70.0	0.000018
0.59,70.1	0.000018
0.59,70.2	0.000018
0.59,70.3	0.000018
0.59,70.4	0.000018
0.59,70.5	0.000018
0.59,70.6	0.000018
0.59,70.7	0.000018
0.59,70.8	0.000018
0.59,70.9	0.000018
0.59,71.0	0.000018
0.59,71.1	0.000018
0.59,71.2	0.000018
0.59,71.3	0.000018
0.59,71.4	0.000018
0.59,71.5	0.000018
0.59,71.6	0.000018
0.59,71.7	0.000018
0.59,71.8	0.000018
0.59,71.9	0.000018
0.59,72.0	0.000018
0.59,72.1	0.000018
0.59,72.2	0.000018
0.59,72.3	0.000018
0.59,72.4	0.000018
0.59,72.5	0.000018
0.59,72.6	0.000018
0.59,72.7	0.000018
0.59,72.8	0.000018
0.59,72.9	0.000018
0.59,73.0	0.000018
0.59,73.1	0.000018
0.59,73.2	0.000018
0.59,73.3	0.000018
0.59,73.4	0.000018
0.59,73.5	0.000018
0.59,73.6	0.000018
0.59,73.7	0.000018
0.59,73.8	0.000018
0.59,73.9	0.000018
0.59,74.0	0.000018

0.59,74.1	0.000018
0.59,74.2	0.000018
0.59,74.3	0.000018
0.59,74.4	0.000018
0.59,74.5	0.000018
0.59,74.6	0.000018
0.59,74.7	0.000018
0.59,74.8	0.000018
0.59,74.9	0.000018
0.59,75.0	0.000018
0.59,75.1	0.000018
0.59,75.2	0.000018
0.59,75.3	0.000018
0.59,75.4	0.000018
0.59,75.5	0.000018
0.59,75.6	0.000018
0.59,75.7	0.000018
0.59,75.8	0.000018
0.59,75.9	0.000018
0.59,76.0	0.000018
0.59,76.1	0.000018
0.59,76.2	0.000018
0.59,76.3	0.000018
0.59,76.4	0.000019
0.59,76.5	0.000019
0.59,76.6	0.00002
0.59,76.7	0.00002
0.59,76.8	0.00002
0.59,76.9	0.00002
0.59,77.0	0.000021
0.59,77.1	0.000021
0.59,77.2	0.000021
0.59,77.3	0.000021
0.59,77.4	0.000021
0.59,77.5	0.000021
0.59,77.6	0.000021
0.59,77.7	0.000021
0.59,77.8	0.000021
0.59,77.9	0.000021
0.59,78.0	0.000021
0.59,78.1	0.000021
0.59,78.2	0.000021

0.59,78.3	0.000021
0.59,78.4	0.000021
0.59,78.5	0.000021
0.59,78.6	0.000021
0.59,78.7	0.000021
0.59,78.8	0.000021
0.59,78.9	0.000021
0.59,79.0	0.000021
0.59,79.1	0.000021
0.59,79.2	0.000021
0.59,79.3	0.000022
0.59,79.4	0.000022
0.59,79.5	0.000022
0.59,79.6	0.000022
0.59,79.7	0.000023
0.59,79.8	0.000023
0.59,79.9	0.000024
0.59,80.0	0.000024
0.59,80.1	0.000024
0.59,80.2	0.000025
0.59,80.3	0.000025
0.59,80.4	0.000025
0.59,80.5	0.000025
0.59,80.6	0.000025
0.59,80.7	0.000025
0.59,80.8	0.000025
0.59,80.9	0.000025
0.59,81.0	0.000025
0.59,81.1	0.000026
0.59,81.2	0.000028
0.59,81.3	0.000057
0.59,81.4	0.000138
0.59,81.5	0.000214
0.59,81.6	0.000304
0.59,81.7	0.000357
0.59,81.8	0.000393
0.59,81.9	0.00042
0.59,82.0	0.000436
0.59,82.1	0.000447
0.59,82.2	0.000459
0.59,82.3	0.000474
0.59,82.4	0.000493

0.59,82.5	0.000509
0.59,82.6	0.000538
0.59,82.7	0.000549
0.59,82.8	0.00057
0.59,82.9	0.000609
0.59,83.0	0.000743
0.59,83.1	0.000783
0.59,83.2	0.000806
0.59,83.3	0.00083
0.59,83.4	0.000839
0.59,83.5	0.000853
0.59,83.6	0.000866
0.59,83.7	0.000869
0.59,83.8	0.000876
0.59,83.9	0.000888
0.59,84.0	0.000893
0.59,84.1	0.000909
0.59,84.2	0.000968
0.59,84.3	0.001043
0.59,84.4	0.001056
0.59,84.5	0.001056
0.59,84.6	0.001065
0.59,84.7	0.001073
0.59,84.8	0.001078
0.59,84.9	0.001091
0.59,85.0	0.001092
0.59,85.1	0.001104
0.59,85.2	0.001116
0.59,85.3	0.001127
0.59,85.4	0.001128
0.59,85.5	0.001137
0.59,85.6	0.001137
0.59,85.7	0.001137
0.59,85.8	0.001139
0.59,85.9	0.001156
0.59,86.0	0.001163
0.59,86.1	0.001169
0.59,86.2	0.001182
0.59,86.3	0.001239
0.59,86.4	0.001406
0.59,86.5	0.001682
0.59,86.6	0.002

0.59,86.7	0.002087
0.59,86.8	0.002099
0.59,86.9	0.002099
0.59,87.0	0.002149
0.59,87.1	0.002257
0.59,87.2	0.002257
0.59,87.3	0.002257
0.59,87.4	0.002264
0.59,87.5	0.002278
0.59,87.6	0.002286
0.59,87.7	0.0023
0.59,87.8	0.002308
0.59,87.9	0.002322
0.59,88.0	0.002337
0.59,88.1	0.002353
0.59,88.2	0.00236
0.59,88.3	0.002424
0.59,88.4	0.002509
0.59,88.5	0.002553
0.59,88.6	0.002571
0.59,88.7	0.00258
0.59,88.8	0.002599
0.59,88.9	0.002599
0.59,89.0	0.002608
0.59,89.1	0.002618
0.59,89.2	0.002637
0.59,89.3	0.002666
0.59,89.4	0.002686
0.59,89.5	0.002707
0.59,89.6	0.002758
0.59,89.7	0.002758
0.59,89.8	0.00278
0.59,89.9	0.002823
0.59,90.0	0.002834
0.59,90.1	0.00288
0.59,90.2	0.002903
0.59,90.3	0.002939
0.59,90.4	0.002951
0.59,90.5	0.002963
0.59,90.6	0.003
0.59,90.7	0.003
0.59,90.8	0.003051

0.59,90.9	0.003051
0.59,91.0	0.003064
0.59,91.1	0.003064
0.59,91.2	0.003077
0.59,91.3	0.003077
0.59,91.4	0.003077
0.59,91.5	0.003117
0.59,91.6	0.003144
0.59,91.7	0.003144
0.59,91.8	0.003158
0.59,91.9	0.003158
0.59,92.0	0.003172
0.59,92.1	0.003228
0.59,92.2	0.003272
0.59,92.3	0.003287
0.59,92.4	0.003302
0.59,92.5	0.003333
0.59,92.6	0.003333
0.59,92.7	0.003333
0.59,92.8	0.00338
0.59,92.9	0.003396
0.59,93.0	0.003396
0.59,93.1	0.003428
0.59,93.2	0.003428
0.59,93.3	0.003445
0.59,93.4	0.003478
0.59,93.5	0.003478
0.59,93.6	0.003478
0.59,93.7	0.003478
0.59,93.8	0.003478
0.59,93.9	0.003478
0.59,94.0	0.003495
0.59,94.1	0.003495
0.59,94.2	0.003547
0.59,94.3	0.003618
0.59,94.4	0.003711
0.59,94.5	0.00373
0.59,94.6	0.00375
0.59,94.7	0.00385
0.59,94.8	0.003913
0.59,94.9	0.003934
0.59,95.0	0.003978

0.59,95.1	0.004022
0.59,95.2	0.004067
0.59,95.3	0.004091
0.59,95.4	0.004091
0.59,95.5	0.004091
0.59,95.6	0.004091
0.59,95.7	0.004091
0.59,95.8	0.00409
0.59,95.9	0.004112
0.59,96.0	0.004112
0.59,96.1	0.004136
0.59,96.2	0.004135
0.59,96.3	0.004135
0.59,96.4	0.004135
0.59,96.5	0.004157
0.59,96.6	0.004182
0.59,96.7	0.004181
0.59,96.8	0.00418
0.59,96.9	0.00418
0.59,97.0	0.00418
0.59,97.1	0.00418
0.59,97.2	0.004179
0.59,97.3	0.004178
0.59,97.4	0.004178
0.59,97.5	0.004178
0.59,97.6	0.004178
0.59,97.7	0.004178
0.59,97.8	0.004174
0.59,97.9	0.004196
0.59,98.0	0.004187
0.59,98.1	0.004185
0.59,98.2	0.004185
0.59,98.3	0.004184
0.59,98.4	0.004183
0.59,98.5	0.004181
0.59,98.6	0.004179
0.59,98.7	0.004174
0.59,98.8	0.004214
0.59,98.9	0.004213
0.59,99.0	0.004212
0.59,99.1	0.004211
0.59,99.2	0.00421

0.59,99.3	0.004209
0.59,99.4	0.004197
0.59,99.5	0.003651
0.59,99.6	0.00426
0.59,99.7	0.00426
0.59,99.8	0.00426
0.59,99.9	0.00426
0.59,100.0	0
0.60,0.0	0.000013
0.60,0.1	0.000013
0.60,0.2	0.000013
0.60,0.3	0.000013
0.60,0.4	0.000013
0.60,0.5	0.000013
0.60,0.6	0.000013
0.60,0.7	0.000013
0.60,0.8	0.000013
0.60,0.9	0.000013
0.60,1.0	0.000013
0.60,1.1	0.000013
0.60,1.2	0.000013
0.60,1.3	0.000013
0.60,1.4	0.000013
0.60,1.5	0.000013
0.60,1.6	0.000013
0.60,1.7	0.000013
0.60,1.8	0.000013
0.60,1.9	0.000013
0.60,2.0	0.000013
0.60,2.1	0.000013
0.60,2.2	0.000013
0.60,2.3	0.000013
0.60,2.4	0.000013
0.60,2.5	0.000013
0.60,2.6	0.000013
0.60,2.7	0.000013
0.60,2.8	0.000013
0.60,2.9	0.000013
0.60,3.0	0.000013
0.60,3.1	0.000013
0.60,3.2	0.000013
0.60,3.3	0.000013

0.60,3.4	0.000013
0.60,3.5	0.000013
0.60,3.6	0.000013
0.60,3.7	0.000013
0.60,3.8	0.000013
0.60,3.9	0.000013
0.60,4.0	0.000013
0.60,4.1	0.000013
0.60,4.2	0.000013
0.60,4.3	0.000013
0.60,4.4	0.000013
0.60,4.5	0.000013
0.60,4.6	0.000013
0.60,4.7	0.000013
0.60,4.8	0.000013
0.60,4.9	0.000013
0.60,5.0	0.000013
0.60,5.1	0.000013
0.60,5.2	0.000013
0.60,5.3	0.000013
0.60,5.4	0.000013
0.60,5.5	0.000013
0.60,5.6	0.000013
0.60,5.7	0.000013
0.60,5.8	0.000013
0.60,5.9	0.000013
0.60,6.0	0.000013
0.60,6.1	0.000013
0.60,6.2	0.000013
0.60,6.3	0.000013
0.60,6.4	0.000013
0.60,6.5	0.000013
0.60,6.6	0.000013
0.60,6.7	0.000013
0.60,6.8	0.000013
0.60,6.9	0.000013
0.60,7.0	0.000013
0.60,7.1	0.000013
0.60,7.2	0.000013
0.60,7.3	0.000013
0.60,7.4	0.000013
0.60,7.5	0.000013

0.60,7.6	0.000013
0.60,7.7	0.000013
0.60,7.8	0.000013
0.60,7.9	0.000013
0.60,8.0	0.000013
0.60,8.1	0.000013
0.60,8.2	0.000013
0.60,8.3	0.000013
0.60,8.4	0.000013
0.60,8.5	0.000013
0.60,8.6	0.000013
0.60,8.7	0.000013
0.60,8.8	0.000013
0.60,8.9	0.000013
0.60,9.0	0.000013
0.60,9.1	0.000013
0.60,9.2	0.000013
0.60,9.3	0.000013
0.60,9.4	0.000013
0.60,9.5	0.000013
0.60,9.6	0.000013
0.60,9.7	0.000013
0.60,9.8	0.000013
0.60,9.9	0.000013
0.60,10.0	0.000013
0.60,10.1	0.000013
0.60,10.2	0.000013
0.60,10.3	0.000013
0.60,10.4	0.000013
0.60,10.5	0.000013
0.60,10.6	0.000013
0.60,10.7	0.000013
0.60,10.8	0.000013
0.60,10.9	0.000013
0.60,11.0	0.000013
0.60,11.1	0.000013
0.60,11.2	0.000013
0.60,11.3	0.000013
0.60,11.4	0.000013
0.60,11.5	0.000013
0.60,11.6	0.000013
0.60,11.7	0.000013

0.60,11.8	0.000013
0.60,11.9	0.000013
0.60,12.0	0.000013
0.60,12.1	0.000013
0.60,12.2	0.000013
0.60,12.3	0.000013
0.60,12.4	0.000013
0.60,12.5	0.000013
0.60,12.6	0.000013
0.60,12.7	0.000013
0.60,12.8	0.000013
0.60,12.9	0.000013
0.60,13.0	0.000013
0.60,13.1	0.000013
0.60,13.2	0.000013
0.60,13.3	0.000013
0.60,13.4	0.000013
0.60,13.5	0.000013
0.60,13.6	0.000013
0.60,13.7	0.000013
0.60,13.8	0.000013
0.60,13.9	0.000013
0.60,14.0	0.000013
0.60,14.1	0.000013
0.60,14.2	0.000013
0.60,14.3	0.000013
0.60,14.4	0.000013
0.60,14.5	0.000013
0.60,14.6	0.000013
0.60,14.7	0.000013
0.60,14.8	0.000013
0.60,14.9	0.000013
0.60,15.0	0.000013
0.60,15.1	0.000013
0.60,15.2	0.000013
0.60,15.3	0.000013
0.60,15.4	0.000013
0.60,15.5	0.000013
0.60,15.6	0.000013
0.60,15.7	0.000013
0.60,15.8	0.000013
0.60,15.9	0.000013

0.60,16.0	0.000013
0.60,16.1	0.000013
0.60,16.2	0.000013
0.60,16.3	0.000013
0.60,16.4	0.000013
0.60,16.5	0.000013
0.60,16.6	0.000013
0.60,16.7	0.000013
0.60,16.8	0.000013
0.60,16.9	0.000013
0.60,17.0	0.000013
0.60,17.1	0.000013
0.60,17.2	0.000013
0.60,17.3	0.000013
0.60,17.4	0.000013
0.60,17.5	0.000013
0.60,17.6	0.000013
0.60,17.7	0.000013
0.60,17.8	0.000013
0.60,17.9	0.000013
0.60,18.0	0.000013
0.60,18.1	0.000013
0.60,18.2	0.000013
0.60,18.3	0.000013
0.60,18.4	0.000013
0.60,18.5	0.000013
0.60,18.6	0.000013
0.60,18.7	0.000013
0.60,18.8	0.000013
0.60,18.9	0.000013
0.60,19.0	0.000013
0.60,19.1	0.000013
0.60,19.2	0.000013
0.60,19.3	0.000013
0.60,19.4	0.000013
0.60,19.5	0.000013
0.60,19.6	0.000013
0.60,19.7	0.000013
0.60,19.8	0.000013
0.60,19.9	0.000013
0.60,20.0	0.000013
0.60,20.1	0.000013

0.60,20.2	0.000013
0.60,20.3	0.000013
0.60,20.4	0.000013
0.60,20.5	0.000013
0.60,20.6	0.000013
0.60,20.7	0.000013
0.60,20.8	0.000013
0.60,20.9	0.000013
0.60,21.0	0.000013
0.60,21.1	0.000013
0.60,21.2	0.000013
0.60,21.3	0.000013
0.60,21.4	0.000013
0.60,21.5	0.000013
0.60,21.6	0.000013
0.60,21.7	0.000013
0.60,21.8	0.000013
0.60,21.9	0.000013
0.60,22.0	0.000013
0.60,22.1	0.000013
0.60,22.2	0.000013
0.60,22.3	0.000013
0.60,22.4	0.000013
0.60,22.5	0.000013
0.60,22.6	0.000013
0.60,22.7	0.000013
0.60,22.8	0.000013
0.60,22.9	0.000013
0.60,23.0	0.000013
0.60,23.1	0.000013
0.60,23.2	0.000013
0.60,23.3	0.000013
0.60,23.4	0.000013
0.60,23.5	0.000013
0.60,23.6	0.000013
0.60,23.7	0.000013
0.60,23.8	0.000013
0.60,23.9	0.000013
0.60,24.0	0.000013
0.60,24.1	0.000013
0.60,24.2	0.000013
0.60,24.3	0.000013

0.60,24.4	0.000013
0.60,24.5	0.000013
0.60,24.6	0.000013
0.60,24.7	0.000013
0.60,24.8	0.000013
0.60,24.9	0.000013
0.60,25.0	0.000013
0.60,25.1	0.000013
0.60,25.2	0.000013
0.60,25.3	0.000013
0.60,25.4	0.000013
0.60,25.5	0.000013
0.60,25.6	0.000013
0.60,25.7	0.000013
0.60,25.8	0.000013
0.60,25.9	0.000013
0.60,26.0	0.000013
0.60,26.1	0.000013
0.60,26.2	0.000013
0.60,26.3	0.000013
0.60,26.4	0.000013
0.60,26.5	0.000013
0.60,26.6	0.000013
0.60,26.7	0.000013
0.60,26.8	0.000013
0.60,26.9	0.000013
0.60,27.0	0.000013
0.60,27.1	0.000013
0.60,27.2	0.000013
0.60,27.3	0.000013
0.60,27.4	0.000013
0.60,27.5	0.000013
0.60,27.6	0.000013
0.60,27.7	0.000013
0.60,27.8	0.000013
0.60,27.9	0.000013
0.60,28.0	0.000013
0.60,28.1	0.000013
0.60,28.2	0.000013
0.60,28.3	0.000013
0.60,28.4	0.000013
0.60,28.5	0.000013

0.60,28.6	0.000013
0.60,28.7	0.000013
0.60,28.8	0.000013
0.60,28.9	0.000013
0.60,29.0	0.000013
0.60,29.1	0.000013
0.60,29.2	0.000013
0.60,29.3	0.000013
0.60,29.4	0.000013
0.60,29.5	0.000013
0.60,29.6	0.000013
0.60,29.7	0.000013
0.60,29.8	0.000013
0.60,29.9	0.000013
0.60,30.0	0.000013
0.60,30.1	0.000013
0.60,30.2	0.000013
0.60,30.3	0.000013
0.60,30.4	0.000013
0.60,30.5	0.000013
0.60,30.6	0.000013
0.60,30.7	0.000013
0.60,30.8	0.000013
0.60,30.9	0.000013
0.60,31.0	0.000013
0.60,31.1	0.000013
0.60,31.2	0.000013
0.60,31.3	0.000013
0.60,31.4	0.000013
0.60,31.5	0.000013
0.60,31.6	0.000013
0.60,31.7	0.000013
0.60,31.8	0.000013
0.60,31.9	0.000013
0.60,32.0	0.000013
0.60,32.1	0.000013
0.60,32.2	0.000013
0.60,32.3	0.000013
0.60,32.4	0.000013
0.60,32.5	0.000013
0.60,32.6	0.000013
0.60,32.7	0.000013

0.60,32.8	0.000013
0.60,32.9	0.000013
0.60,33.0	0.000013
0.60,33.1	0.000013
0.60,33.2	0.000013
0.60,33.3	0.000013
0.60,33.4	0.000013
0.60,33.5	0.000013
0.60,33.6	0.000013
0.60,33.7	0.000013
0.60,33.8	0.000013
0.60,33.9	0.000013
0.60,34.0	0.000013
0.60,34.1	0.000013
0.60,34.2	0.000013
0.60,34.3	0.000013
0.60,34.4	0.000013
0.60,34.5	0.000013
0.60,34.6	0.000013
0.60,34.7	0.000013
0.60,34.8	0.000013
0.60,34.9	0.000013
0.60,35.0	0.000013
0.60,35.1	0.000013
0.60,35.2	0.000013
0.60,35.3	0.000013
0.60,35.4	0.000013
0.60,35.5	0.000013
0.60,35.6	0.000013
0.60,35.7	0.000013
0.60,35.8	0.000013
0.60,35.9	0.000013
0.60,36.0	0.000013
0.60,36.1	0.000013
0.60,36.2	0.000013
0.60,36.3	0.000013
0.60,36.4	0.000013
0.60,36.5	0.000013
0.60,36.6	0.000013
0.60,36.7	0.000013
0.60,36.8	0.000013
0.60,36.9	0.000013

0.60,37.0	0.000013
0.60,37.1	0.000013
0.60,37.2	0.000013
0.60,37.3	0.000013
0.60,37.4	0.000013
0.60,37.5	0.000013
0.60,37.6	0.000013
0.60,37.7	0.000013
0.60,37.8	0.000013
0.60,37.9	0.000013
0.60,38.0	0.000013
0.60,38.1	0.000013
0.60,38.2	0.000013
0.60,38.3	0.000013
0.60,38.4	0.000013
0.60,38.5	0.000013
0.60,38.6	0.000013
0.60,38.7	0.000013
0.60,38.8	0.000013
0.60,38.9	0.000013
0.60,39.0	0.000013
0.60,39.1	0.000013
0.60,39.2	0.000013
0.60,39.3	0.000013
0.60,39.4	0.000013
0.60,39.5	0.000013
0.60,39.6	0.000013
0.60,39.7	0.000013
0.60,39.8	0.000013
0.60,39.9	0.000013
0.60,40.0	0.000013
0.60,40.1	0.000013
0.60,40.2	0.000013
0.60,40.3	0.000013
0.60,40.4	0.000013
0.60,40.5	0.000013
0.60,40.6	0.000013
0.60,40.7	0.000013
0.60,40.8	0.000013
0.60,40.9	0.000013
0.60,41.0	0.000013
0.60,41.1	0.000013

0.60,41.2	0.000013
0.60,41.3	0.000013
0.60,41.4	0.000013
0.60,41.5	0.000013
0.60,41.6	0.000013
0.60,41.7	0.000013
0.60,41.8	0.000013
0.60,41.9	0.000013
0.60,42.0	0.000013
0.60,42.1	0.000013
0.60,42.2	0.000013
0.60,42.3	0.000013
0.60,42.4	0.000013
0.60,42.5	0.000013
0.60,42.6	0.000013
0.60,42.7	0.000013
0.60,42.8	0.000013
0.60,42.9	0.000013
0.60,43.0	0.000013
0.60,43.1	0.000013
0.60,43.2	0.000013
0.60,43.3	0.000013
0.60,43.4	0.000013
0.60,43.5	0.000013
0.60,43.6	0.000013
0.60,43.7	0.000013
0.60,43.8	0.000013
0.60,43.9	0.000013
0.60,44.0	0.000013
0.60,44.1	0.000013
0.60,44.2	0.000013
0.60,44.3	0.000013
0.60,44.4	0.000013
0.60,44.5	0.000013
0.60,44.6	0.000013
0.60,44.7	0.000013
0.60,44.8	0.000013
0.60,44.9	0.000013
0.60,45.0	0.000013
0.60,45.1	0.000013
0.60,45.2	0.000013
0.60,45.3	0.000013

0.60,45.4	0.000013
0.60,45.5	0.000013
0.60,45.6	0.000013
0.60,45.7	0.000013
0.60,45.8	0.000013
0.60,45.9	0.000013
0.60,46.0	0.000013
0.60,46.1	0.000013
0.60,46.2	0.000013
0.60,46.3	0.000013
0.60,46.4	0.000013
0.60,46.5	0.000013
0.60,46.6	0.000013
0.60,46.7	0.000013
0.60,46.8	0.000013
0.60,46.9	0.000013
0.60,47.0	0.000013
0.60,47.1	0.000013
0.60,47.2	0.000013
0.60,47.3	0.000013
0.60,47.4	0.000013
0.60,47.5	0.000013
0.60,47.6	0.000013
0.60,47.7	0.000013
0.60,47.8	0.000013
0.60,47.9	0.000013
0.60,48.0	0.000013
0.60,48.1	0.000013
0.60,48.2	0.000013
0.60,48.3	0.000013
0.60,48.4	0.000013
0.60,48.5	0.000013
0.60,48.6	0.000013
0.60,48.7	0.000013
0.60,48.8	0.000013
0.60,48.9	0.000013
0.60,49.0	0.000013
0.60,49.1	0.000013
0.60,49.2	0.000013
0.60,49.3	0.000013
0.60,49.4	0.000013
0.60,49.5	0.000013

0.60,49.6	0.000013
0.60,49.7	0.000013
0.60,49.8	0.000013
0.60,49.9	0.000013
0.60,50.0	0.000013
0.60,50.1	0.000013
0.60,50.2	0.000013
0.60,50.3	0.000013
0.60,50.4	0.000013
0.60,50.5	0.000013
0.60,50.6	0.000013
0.60,50.7	0.000013
0.60,50.8	0.000013
0.60,50.9	0.000013
0.60,51.0	0.000013
0.60,51.1	0.000013
0.60,51.2	0.000013
0.60,51.3	0.000013
0.60,51.4	0.000013
0.60,51.5	0.000013
0.60,51.6	0.000013
0.60,51.7	0.000013
0.60,51.8	0.000013
0.60,51.9	0.000013
0.60,52.0	0.000013
0.60,52.1	0.000013
0.60,52.2	0.000013
0.60,52.3	0.000013
0.60,52.4	0.000013
0.60,52.5	0.000013
0.60,52.6	0.000013
0.60,52.7	0.000013
0.60,52.8	0.000013
0.60,52.9	0.000013
0.60,53.0	0.000013
0.60,53.1	0.000013
0.60,53.2	0.000013
0.60,53.3	0.000013
0.60,53.4	0.000013
0.60,53.5	0.000013
0.60,53.6	0.000013
0.60,53.7	0.000013

0.60,53.8	0.000013
0.60,53.9	0.000013
0.60,54.0	0.000013
0.60,54.1	0.000013
0.60,54.2	0.000013
0.60,54.3	0.000013
0.60,54.4	0.000013
0.60,54.5	0.000013
0.60,54.6	0.000013
0.60,54.7	0.000013
0.60,54.8	0.000013
0.60,54.9	0.000013
0.60,55.0	0.000013
0.60,55.1	0.000013
0.60,55.2	0.000013
0.60,55.3	0.000013
0.60,55.4	0.000013
0.60,55.5	0.000013
0.60,55.6	0.000013
0.60,55.7	0.000013
0.60,55.8	0.000013
0.60,55.9	0.000013
0.60,56.0	0.000013
0.60,56.1	0.000013
0.60,56.2	0.000013
0.60,56.3	0.000013
0.60,56.4	0.000013
0.60,56.5	0.000013
0.60,56.6	0.000013
0.60,56.7	0.000013
0.60,56.8	0.000013
0.60,56.9	0.000013
0.60,57.0	0.000013
0.60,57.1	0.000013
0.60,57.2	0.000013
0.60,57.3	0.000013
0.60,57.4	0.000013
0.60,57.5	0.000013
0.60,57.6	0.000013
0.60,57.7	0.000013
0.60,57.8	0.000013
0.60,57.9	0.000013

0.60,58.0	0.000013
0.60,58.1	0.000013
0.60,58.2	0.000013
0.60,58.3	0.000013
0.60,58.4	0.000013
0.60,58.5	0.000013
0.60,58.6	0.000013
0.60,58.7	0.000013
0.60,58.8	0.000013
0.60,58.9	0.000013
0.60,59.0	0.000013
0.60,59.1	0.000013
0.60,59.2	0.000013
0.60,59.3	0.000013
0.60,59.4	0.000013
0.60,59.5	0.000013
0.60,59.6	0.000013
0.60,59.7	0.000013
0.60,59.8	0.000013
0.60,59.9	0.000013
0.60,60.0	0.000013
0.60,60.1	0.000013
0.60,60.2	0.000013
0.60,60.3	0.000013
0.60,60.4	0.000013
0.60,60.5	0.000013
0.60,60.6	0.000013
0.60,60.7	0.000013
0.60,60.8	0.000013
0.60,60.9	0.000013
0.60,61.0	0.000013
0.60,61.1	0.000013
0.60,61.2	0.000013
0.60,61.3	0.000013
0.60,61.4	0.000013
0.60,61.5	0.000013
0.60,61.6	0.000013
0.60,61.7	0.000013
0.60,61.8	0.000013
0.60,61.9	0.000013
0.60,62.0	0.000013
0.60,62.1	0.000013

0.60,62.2	0.000013
0.60,62.3	0.000013
0.60,62.4	0.000013
0.60,62.5	0.000013
0.60,62.6	0.000013
0.60,62.7	0.000013
0.60,62.8	0.000013
0.60,62.9	0.000013
0.60,63.0	0.000013
0.60,63.1	0.000013
0.60,63.2	0.000013
0.60,63.3	0.000013
0.60,63.4	0.000013
0.60,63.5	0.000013
0.60,63.6	0.000013
0.60,63.7	0.000013
0.60,63.8	0.000013
0.60,63.9	0.000013
0.60,64.0	0.000013
0.60,64.1	0.000013
0.60,64.2	0.000013
0.60,64.3	0.000013
0.60,64.4	0.000013
0.60,64.5	0.000013
0.60,64.6	0.000013
0.60,64.7	0.000013
0.60,64.8	0.000013
0.60,64.9	0.000013
0.60,65.0	0.000013
0.60,65.1	0.000013
0.60,65.2	0.000013
0.60,65.3	0.000013
0.60,65.4	0.000013
0.60,65.5	0.000013
0.60,65.6	0.000013
0.60,65.7	0.000013
0.60,65.8	0.000013
0.60,65.9	0.000013
0.60,66.0	0.000013
0.60,66.1	0.000013
0.60,66.2	0.000013
0.60,66.3	0.000013

0.60,66.4	0.000013
0.60,66.5	0.000013
0.60,66.6	0.000013
0.60,66.7	0.000013
0.60,66.8	0.000013
0.60,66.9	0.000013
0.60,67.0	0.000013
0.60,67.1	0.000013
0.60,67.2	0.000013
0.60,67.3	0.000013
0.60,67.4	0.000013
0.60,67.5	0.000013
0.60,67.6	0.000013
0.60,67.7	0.000013
0.60,67.8	0.000013
0.60,67.9	0.000013
0.60,68.0	0.000013
0.60,68.1	0.000013
0.60,68.2	0.000013
0.60,68.3	0.000013
0.60,68.4	0.000013
0.60,68.5	0.000013
0.60,68.6	0.000013
0.60,68.7	0.000013
0.60,68.8	0.000013
0.60,68.9	0.000013
0.60,69.0	0.000013
0.60,69.1	0.000013
0.60,69.2	0.000013
0.60,69.3	0.000013
0.60,69.4	0.000013
0.60,69.5	0.000013
0.60,69.6	0.000013
0.60,69.7	0.000013
0.60,69.8	0.000013
0.60,69.9	0.000013
0.60,70.0	0.000013
0.60,70.1	0.000013
0.60,70.2	0.000013
0.60,70.3	0.000013
0.60,70.4	0.000013
0.60,70.5	0.000013

0.60,70.6	0.000013
0.60,70.7	0.000013
0.60,70.8	0.000013
0.60,70.9	0.000013
0.60,71.0	0.000013
0.60,71.1	0.000013
0.60,71.2	0.000013
0.60,71.3	0.000013
0.60,71.4	0.000013
0.60,71.5	0.000013
0.60,71.6	0.000013
0.60,71.7	0.000013
0.60,71.8	0.000013
0.60,71.9	0.000013
0.60,72.0	0.000013
0.60,72.1	0.000013
0.60,72.2	0.000013
0.60,72.3	0.000013
0.60,72.4	0.000013
0.60,72.5	0.000013
0.60,72.6	0.000013
0.60,72.7	0.000013
0.60,72.8	0.000013
0.60,72.9	0.000013
0.60,73.0	0.000013
0.60,73.1	0.000013
0.60,73.2	0.000013
0.60,73.3	0.000013
0.60,73.4	0.000013
0.60,73.5	0.000013
0.60,73.6	0.000013
0.60,73.7	0.000013
0.60,73.8	0.000013
0.60,73.9	0.000013
0.60,74.0	0.000013
0.60,74.1	0.000013
0.60,74.2	0.000013
0.60,74.3	0.000013
0.60,74.4	0.000013
0.60,74.5	0.000013
0.60,74.6	0.000013
0.60,74.7	0.000013

0.60,74.8	0.000013
0.60,74.9	0.000013
0.60,75.0	0.000013
0.60,75.1	0.000013
0.60,75.2	0.000013
0.60,75.3	0.000013
0.60,75.4	0.000013
0.60,75.5	0.000013
0.60,75.6	0.000013
0.60,75.7	0.000013
0.60,75.8	0.000013
0.60,75.9	0.000013
0.60,76.0	0.000013
0.60,76.1	0.000013
0.60,76.2	0.000013
0.60,76.3	0.000013
0.60,76.4	0.000014
0.60,76.5	0.000014
0.60,76.6	0.000014
0.60,76.7	0.000014
0.60,76.8	0.000014
0.60,76.9	0.000015
0.60,77.0	0.000015
0.60,77.1	0.000015
0.60,77.2	0.000015
0.60,77.3	0.000015
0.60,77.4	0.000015
0.60,77.5	0.000015
0.60,77.6	0.000015
0.60,77.7	0.000015
0.60,77.8	0.000015
0.60,77.9	0.000015
0.60,78.0	0.000015
0.60,78.1	0.000015
0.60,78.2	0.000015
0.60,78.3	0.000015
0.60,78.4	0.000015
0.60,78.5	0.000015
0.60,78.6	0.000015
0.60,78.7	0.000015
0.60,78.8	0.000015
0.60,78.9	0.000015

0.60,79.0	0.000015
0.60,79.1	0.000015
0.60,79.2	0.000015
0.60,79.3	0.000015
0.60,79.4	0.000015
0.60,79.5	0.000015
0.60,79.6	0.000015
0.60,79.7	0.000015
0.60,79.8	0.000016
0.60,79.9	0.000016
0.60,80.0	0.000016
0.60,80.1	0.000016
0.60,80.2	0.000016
0.60,80.3	0.000016
0.60,80.4	0.000016
0.60,80.5	0.000016
0.60,80.6	0.000016
0.60,80.7	0.000016
0.60,80.8	0.000017
0.60,80.9	0.000017
0.60,81.0	0.000017
0.60,81.1	0.000017
0.60,81.2	0.000018
0.60,81.3	0.000043
0.60,81.4	0.000102
0.60,81.5	0.000182
0.60,81.6	0.000262
0.60,81.7	0.000323
0.60,81.8	0.000353
0.60,81.9	0.000376
0.60,82.0	0.000394
0.60,82.1	0.00041
0.60,82.2	0.000422
0.60,82.3	0.000445
0.60,82.4	0.000459
0.60,82.5	0.000479
0.60,82.6	0.000496
0.60,82.7	0.000509
0.60,82.8	0.000529
0.60,82.9	0.000596
0.60,83.0	0.000697
0.60,83.1	0.000718

0.60,83.2	0.000737
0.60,83.3	0.000753
0.60,83.4	0.000763
0.60,83.5	0.000779
0.60,83.6	0.000789
0.60,83.7	0.000793
0.60,83.8	0.000802
0.60,83.9	0.000811
0.60,84.0	0.000814
0.60,84.1	0.000846
0.60,84.2	0.000887
0.60,84.3	0.000921
0.60,84.4	0.000929
0.60,84.5	0.000933
0.60,84.6	0.000942
0.60,84.7	0.000942
0.60,84.8	0.000945
0.60,84.9	0.000945
0.60,85.0	0.00095
0.60,85.1	0.00096
0.60,85.2	0.00097
0.60,85.3	0.000976
0.60,85.4	0.000979
0.60,85.5	0.000983
0.60,85.6	0.000987
0.60,85.7	0.000989
0.60,85.8	0.000993
0.60,85.9	0.000998
0.60,86.0	0.001
0.60,86.1	0.001004
0.60,86.2	0.001007
0.60,86.3	0.001044
0.60,86.4	0.001119
0.60,86.5	0.001233
0.60,86.6	0.001469
0.60,86.7	0.001565
0.60,86.8	0.001574
0.60,86.9	0.00159
0.60,87.0	0.001692
0.60,87.1	0.001729
0.60,87.2	0.001745
0.60,87.3	0.001748

0.60,87.4	0.001752
0.60,87.5	0.00178
0.60,87.6	0.001788
0.60,87.7	0.001788
0.60,87.8	0.001788
0.60,87.9	0.001792
0.60,88.0	0.0018
0.60,88.1	0.001817
0.60,88.2	0.001829
0.60,88.3	0.001855
0.60,88.4	0.001864
0.60,88.5	0.001886
0.60,88.6	0.001904
0.60,88.7	0.001914
0.60,88.8	0.001914
0.60,88.9	0.001914
0.60,89.0	0.001932
0.60,89.1	0.001942
0.60,89.2	0.001947
0.60,89.3	0.001961
0.60,89.4	0.001966
0.60,89.5	0.001986
0.60,89.6	0.002016
0.60,89.7	0.002053
0.60,89.8	0.002085
0.60,89.9	0.002091
0.60,90.0	0.002102
0.60,90.1	0.002113
0.60,90.2	0.002136
0.60,90.3	0.002177
0.60,90.4	0.002189
0.60,90.5	0.002226
0.60,90.6	0.002239
0.60,90.7	0.002245
0.60,90.8	0.002252
0.60,90.9	0.002252
0.60,91.0	0.002278
0.60,91.1	0.002284
0.60,91.2	0.002311
0.60,91.3	0.002324
0.60,91.4	0.002331
0.60,91.5	0.002388

0.60,91.6	0.002493
0.60,91.7	0.002525
0.60,91.8	0.002558
0.60,91.9	0.002591
0.60,92.0	0.002626
0.60,92.1	0.002652
0.60,92.2	0.00267
0.60,92.3	0.00267
0.60,92.4	0.002679
0.60,92.5	0.002793
0.60,92.6	0.002843
0.60,92.7	0.002863
0.60,92.8	0.002895
0.60,92.9	0.002938
0.60,93.0	0.00296
0.60,93.1	0.002971
0.60,93.2	0.002971
0.60,93.3	0.003005
0.60,93.4	0.00304
0.60,93.5	0.003051
0.60,93.6	0.003075
0.60,93.7	0.003087
0.60,93.8	0.003087
0.60,93.9	0.003111
0.60,94.0	0.003149
0.60,94.1	0.003161
0.60,94.2	0.003174
0.60,94.3	0.003226
0.60,94.4	0.003279
0.60,94.5	0.003307
0.60,94.6	0.003321
0.60,94.7	0.003321
0.60,94.8	0.003363
0.60,94.9	0.003466
0.60,95.0	0.003466
0.60,95.1	0.003482
0.60,95.2	0.003497
0.60,95.3	0.003513
0.60,95.4	0.003544
0.60,95.5	0.003592
0.60,95.6	0.003592
0.60,95.7	0.003592

0.60,95.8	0.003592
0.60,95.9	0.003592
0.60,96.0	0.003592
0.60,96.1	0.003625
0.60,96.2	0.003625
0.60,96.3	0.003625
0.60,96.4	0.003642
0.60,96.5	0.003642
0.60,96.6	0.003642
0.60,96.7	0.003642
0.60,96.8	0.003642
0.60,96.9	0.003642
0.60,97.0	0.003642
0.60,97.1	0.003642
0.60,97.2	0.003642
0.60,97.3	0.003642
0.60,97.4	0.003642
0.60,97.5	0.003642
0.60,97.6	0.003642
0.60,97.7	0.003642
0.60,97.8	0.003642
0.60,97.9	0.003642
0.60,98.0	0.003642
0.60,98.1	0.003642
0.60,98.2	0.003642
0.60,98.3	0.003642
0.60,98.4	0.003642
0.60,98.5	0.003642
0.60,98.6	0.003642
0.60,98.7	0.003642
0.60,98.8	0.003642
0.60,98.9	0.003642
0.60,99.0	0.003642
0.60,99.1	0.003642
0.60,99.2	0.003642
0.60,99.3	0.003642
0.60,99.4	0.003642
0.60,99.5	0.003642
0.60,99.6	0.003642
0.60,99.7	0.003642
0.60,99.8	0.003642
0.60,99.9	0.003642

0.60,100.0	0.003642
0.61,0.0	0.00002
0.61,0.1	0.00002
0.61,0.2	0.00002
0.61,0.3	0.00002
0.61,0.4	0.00002
0.61,0.5	0.00002
0.61,0.6	0.00002
0.61,0.7	0.00002
0.61,0.8	0.00002
0.61,0.9	0.00002
0.61,1.0	0.00002
0.61,1.1	0.00002
0.61,1.2	0.00002
0.61,1.3	0.00002
0.61,1.4	0.00002
0.61,1.5	0.00002
0.61,1.6	0.00002
0.61,1.7	0.00002
0.61,1.8	0.00002
0.61,1.9	0.00002
0.61,2.0	0.00002
0.61,2.1	0.00002
0.61,2.2	0.00002
0.61,2.3	0.00002
0.61,2.4	0.00002
0.61,2.5	0.00002
0.61,2.6	0.00002
0.61,2.7	0.00002
0.61,2.8	0.00002
0.61,2.9	0.00002
0.61,3.0	0.00002
0.61,3.1	0.00002
0.61,3.2	0.00002
0.61,3.3	0.00002
0.61,3.4	0.00002
0.61,3.5	0.00002
0.61,3.6	0.00002
0.61,3.7	0.00002
0.61,3.8	0.00002
0.61,3.9	0.00002
0.61,4.0	0.00002

0.61,4.1	0.00002
0.61,4.2	0.00002
0.61,4.3	0.00002
0.61,4.4	0.00002
0.61,4.5	0.00002
0.61,4.6	0.00002
0.61,4.7	0.00002
0.61,4.8	0.00002
0.61,4.9	0.00002
0.61,5.0	0.00002
0.61,5.1	0.00002
0.61,5.2	0.00002
0.61,5.3	0.00002
0.61,5.4	0.00002
0.61,5.5	0.00002
0.61,5.6	0.00002
0.61,5.7	0.00002
0.61,5.8	0.00002
0.61,5.9	0.00002
0.61,6.0	0.00002
0.61,6.1	0.00002
0.61,6.2	0.00002
0.61,6.3	0.00002
0.61,6.4	0.00002
0.61,6.5	0.00002
0.61,6.6	0.00002
0.61,6.7	0.00002
0.61,6.8	0.00002
0.61,6.9	0.00002
0.61,7.0	0.00002
0.61,7.1	0.00002
0.61,7.2	0.00002
0.61,7.3	0.00002
0.61,7.4	0.00002
0.61,7.5	0.00002
0.61,7.6	0.00002
0.61,7.7	0.00002
0.61,7.8	0.00002
0.61,7.9	0.00002
0.61,8.0	0.00002
0.61,8.1	0.00002
0.61,8.2	0.00002

0.61,8.3	0.00002
0.61,8.4	0.00002
0.61,8.5	0.00002
0.61,8.6	0.00002
0.61,8.7	0.00002
0.61,8.8	0.00002
0.61,8.9	0.00002
0.61,9.0	0.00002
0.61,9.1	0.00002
0.61,9.2	0.00002
0.61,9.3	0.00002
0.61,9.4	0.00002
0.61,9.5	0.00002
0.61,9.6	0.00002
0.61,9.7	0.00002
0.61,9.8	0.00002
0.61,9.9	0.00002
0.61,10.0	0.00002
0.61,10.1	0.00002
0.61,10.2	0.00002
0.61,10.3	0.00002
0.61,10.4	0.00002
0.61,10.5	0.00002
0.61,10.6	0.00002
0.61,10.7	0.00002
0.61,10.8	0.00002
0.61,10.9	0.00002
0.61,11.0	0.00002
0.61,11.1	0.00002
0.61,11.2	0.00002
0.61,11.3	0.00002
0.61,11.4	0.00002
0.61,11.5	0.00002
0.61,11.6	0.00002
0.61,11.7	0.00002
0.61,11.8	0.00002
0.61,11.9	0.00002
0.61,12.0	0.00002
0.61,12.1	0.00002
0.61,12.2	0.00002
0.61,12.3	0.00002
0.61,12.4	0.00002

0.61,12.5	0.00002
0.61,12.6	0.00002
0.61,12.7	0.00002
0.61,12.8	0.00002
0.61,12.9	0.00002
0.61,13.0	0.00002
0.61,13.1	0.00002
0.61,13.2	0.00002
0.61,13.3	0.00002
0.61,13.4	0.00002
0.61,13.5	0.00002
0.61,13.6	0.00002
0.61,13.7	0.00002
0.61,13.8	0.00002
0.61,13.9	0.00002
0.61,14.0	0.00002
0.61,14.1	0.00002
0.61,14.2	0.00002
0.61,14.3	0.00002
0.61,14.4	0.00002
0.61,14.5	0.00002
0.61,14.6	0.00002
0.61,14.7	0.00002
0.61,14.8	0.00002
0.61,14.9	0.00002
0.61,15.0	0.00002
0.61,15.1	0.00002
0.61,15.2	0.00002
0.61,15.3	0.00002
0.61,15.4	0.00002
0.61,15.5	0.00002
0.61,15.6	0.00002
0.61,15.7	0.00002
0.61,15.8	0.00002
0.61,15.9	0.00002
0.61,16.0	0.00002
0.61,16.1	0.00002
0.61,16.2	0.00002
0.61,16.3	0.00002
0.61,16.4	0.00002
0.61,16.5	0.00002
0.61,16.6	0.00002

0.61,16.7	0.00002
0.61,16.8	0.00002
0.61,16.9	0.00002
0.61,17.0	0.00002
0.61,17.1	0.00002
0.61,17.2	0.00002
0.61,17.3	0.00002
0.61,17.4	0.00002
0.61,17.5	0.00002
0.61,17.6	0.00002
0.61,17.7	0.00002
0.61,17.8	0.00002
0.61,17.9	0.00002
0.61,18.0	0.00002
0.61,18.1	0.00002
0.61,18.2	0.00002
0.61,18.3	0.00002
0.61,18.4	0.00002
0.61,18.5	0.00002
0.61,18.6	0.00002
0.61,18.7	0.00002
0.61,18.8	0.00002
0.61,18.9	0.00002
0.61,19.0	0.00002
0.61,19.1	0.00002
0.61,19.2	0.00002
0.61,19.3	0.00002
0.61,19.4	0.00002
0.61,19.5	0.00002
0.61,19.6	0.00002
0.61,19.7	0.00002
0.61,19.8	0.00002
0.61,19.9	0.00002
0.61,20.0	0.00002
0.61,20.1	0.00002
0.61,20.2	0.00002
0.61,20.3	0.00002
0.61,20.4	0.00002
0.61,20.5	0.00002
0.61,20.6	0.00002
0.61,20.7	0.00002
0.61,20.8	0.00002

0.61,20.9	0.00002
0.61,21.0	0.00002
0.61,21.1	0.00002
0.61,21.2	0.00002
0.61,21.3	0.00002
0.61,21.4	0.00002
0.61,21.5	0.00002
0.61,21.6	0.00002
0.61,21.7	0.00002
0.61,21.8	0.00002
0.61,21.9	0.00002
0.61,22.0	0.00002
0.61,22.1	0.00002
0.61,22.2	0.00002
0.61,22.3	0.00002
0.61,22.4	0.00002
0.61,22.5	0.00002
0.61,22.6	0.00002
0.61,22.7	0.00002
0.61,22.8	0.00002
0.61,22.9	0.00002
0.61,23.0	0.00002
0.61,23.1	0.00002
0.61,23.2	0.00002
0.61,23.3	0.00002
0.61,23.4	0.00002
0.61,23.5	0.00002
0.61,23.6	0.00002
0.61,23.7	0.00002
0.61,23.8	0.00002
0.61,23.9	0.00002
0.61,24.0	0.00002
0.61,24.1	0.00002
0.61,24.2	0.00002
0.61,24.3	0.00002
0.61,24.4	0.00002
0.61,24.5	0.00002
0.61,24.6	0.00002
0.61,24.7	0.00002
0.61,24.8	0.00002
0.61,24.9	0.00002
0.61,25.0	0.00002

0.61,25.1	0.00002
0.61,25.2	0.00002
0.61,25.3	0.00002
0.61,25.4	0.00002
0.61,25.5	0.00002
0.61,25.6	0.00002
0.61,25.7	0.00002
0.61,25.8	0.00002
0.61,25.9	0.00002
0.61,26.0	0.00002
0.61,26.1	0.00002
0.61,26.2	0.00002
0.61,26.3	0.00002
0.61,26.4	0.00002
0.61,26.5	0.00002
0.61,26.6	0.00002
0.61,26.7	0.00002
0.61,26.8	0.00002
0.61,26.9	0.00002
0.61,27.0	0.00002
0.61,27.1	0.00002
0.61,27.2	0.00002
0.61,27.3	0.00002
0.61,27.4	0.00002
0.61,27.5	0.00002
0.61,27.6	0.00002
0.61,27.7	0.00002
0.61,27.8	0.00002
0.61,27.9	0.00002
0.61,28.0	0.00002
0.61,28.1	0.00002
0.61,28.2	0.00002
0.61,28.3	0.00002
0.61,28.4	0.00002
0.61,28.5	0.00002
0.61,28.6	0.00002
0.61,28.7	0.00002
0.61,28.8	0.00002
0.61,28.9	0.00002
0.61,29.0	0.00002
0.61,29.1	0.00002
0.61,29.2	0.00002

0.61,29.3	0.00002
0.61,29.4	0.00002
0.61,29.5	0.00002
0.61,29.6	0.00002
0.61,29.7	0.00002
0.61,29.8	0.00002
0.61,29.9	0.00002
0.61,30.0	0.00002
0.61,30.1	0.00002
0.61,30.2	0.00002
0.61,30.3	0.00002
0.61,30.4	0.00002
0.61,30.5	0.00002
0.61,30.6	0.00002
0.61,30.7	0.00002
0.61,30.8	0.00002
0.61,30.9	0.00002
0.61,31.0	0.00002
0.61,31.1	0.00002
0.61,31.2	0.00002
0.61,31.3	0.00002
0.61,31.4	0.00002
0.61,31.5	0.00002
0.61,31.6	0.00002
0.61,31.7	0.00002
0.61,31.8	0.00002
0.61,31.9	0.00002
0.61,32.0	0.00002
0.61,32.1	0.00002
0.61,32.2	0.00002
0.61,32.3	0.00002
0.61,32.4	0.00002
0.61,32.5	0.00002
0.61,32.6	0.00002
0.61,32.7	0.00002
0.61,32.8	0.00002
0.61,32.9	0.00002
0.61,33.0	0.00002
0.61,33.1	0.00002
0.61,33.2	0.00002
0.61,33.3	0.00002
0.61,33.4	0.00002

0.61,33.5	0.00002
0.61,33.6	0.00002
0.61,33.7	0.00002
0.61,33.8	0.00002
0.61,33.9	0.00002
0.61,34.0	0.00002
0.61,34.1	0.00002
0.61,34.2	0.00002
0.61,34.3	0.00002
0.61,34.4	0.00002
0.61,34.5	0.00002
0.61,34.6	0.00002
0.61,34.7	0.00002
0.61,34.8	0.00002
0.61,34.9	0.00002
0.61,35.0	0.00002
0.61,35.1	0.00002
0.61,35.2	0.00002
0.61,35.3	0.00002
0.61,35.4	0.00002
0.61,35.5	0.00002
0.61,35.6	0.00002
0.61,35.7	0.00002
0.61,35.8	0.00002
0.61,35.9	0.00002
0.61,36.0	0.00002
0.61,36.1	0.00002
0.61,36.2	0.00002
0.61,36.3	0.00002
0.61,36.4	0.00002
0.61,36.5	0.00002
0.61,36.6	0.00002
0.61,36.7	0.00002
0.61,36.8	0.00002
0.61,36.9	0.00002
0.61,37.0	0.00002
0.61,37.1	0.00002
0.61,37.2	0.00002
0.61,37.3	0.00002
0.61,37.4	0.00002
0.61,37.5	0.00002
0.61,37.6	0.00002

0.61,37.7	0.00002
0.61,37.8	0.00002
0.61,37.9	0.00002
0.61,38.0	0.00002
0.61,38.1	0.00002
0.61,38.2	0.00002
0.61,38.3	0.00002
0.61,38.4	0.00002
0.61,38.5	0.00002
0.61,38.6	0.00002
0.61,38.7	0.00002
0.61,38.8	0.00002
0.61,38.9	0.00002
0.61,39.0	0.00002
0.61,39.1	0.00002
0.61,39.2	0.00002
0.61,39.3	0.00002
0.61,39.4	0.00002
0.61,39.5	0.00002
0.61,39.6	0.00002
0.61,39.7	0.00002
0.61,39.8	0.00002
0.61,39.9	0.00002
0.61,40.0	0.00002
0.61,40.1	0.00002
0.61,40.2	0.00002
0.61,40.3	0.00002
0.61,40.4	0.00002
0.61,40.5	0.00002
0.61,40.6	0.00002
0.61,40.7	0.00002
0.61,40.8	0.00002
0.61,40.9	0.00002
0.61,41.0	0.00002
0.61,41.1	0.00002
0.61,41.2	0.00002
0.61,41.3	0.00002
0.61,41.4	0.00002
0.61,41.5	0.00002
0.61,41.6	0.00002
0.61,41.7	0.00002
0.61,41.8	0.00002

0.61,41.9	0.00002
0.61,42.0	0.00002
0.61,42.1	0.00002
0.61,42.2	0.00002
0.61,42.3	0.00002
0.61,42.4	0.00002
0.61,42.5	0.00002
0.61,42.6	0.00002
0.61,42.7	0.00002
0.61,42.8	0.00002
0.61,42.9	0.00002
0.61,43.0	0.00002
0.61,43.1	0.00002
0.61,43.2	0.00002
0.61,43.3	0.00002
0.61,43.4	0.00002
0.61,43.5	0.00002
0.61,43.6	0.00002
0.61,43.7	0.00002
0.61,43.8	0.00002
0.61,43.9	0.00002
0.61,44.0	0.00002
0.61,44.1	0.00002
0.61,44.2	0.00002
0.61,44.3	0.00002
0.61,44.4	0.00002
0.61,44.5	0.00002
0.61,44.6	0.00002
0.61,44.7	0.00002
0.61,44.8	0.00002
0.61,44.9	0.00002
0.61,45.0	0.00002
0.61,45.1	0.00002
0.61,45.2	0.00002
0.61,45.3	0.00002
0.61,45.4	0.00002
0.61,45.5	0.00002
0.61,45.6	0.00002
0.61,45.7	0.00002
0.61,45.8	0.00002
0.61,45.9	0.00002
0.61,46.0	0.00002

0.61,46.1	0.00002
0.61,46.2	0.00002
0.61,46.3	0.00002
0.61,46.4	0.00002
0.61,46.5	0.00002
0.61,46.6	0.00002
0.61,46.7	0.00002
0.61,46.8	0.00002
0.61,46.9	0.00002
0.61,47.0	0.00002
0.61,47.1	0.00002
0.61,47.2	0.00002
0.61,47.3	0.00002
0.61,47.4	0.00002
0.61,47.5	0.00002
0.61,47.6	0.00002
0.61,47.7	0.00002
0.61,47.8	0.00002
0.61,47.9	0.00002
0.61,48.0	0.00002
0.61,48.1	0.00002
0.61,48.2	0.00002
0.61,48.3	0.00002
0.61,48.4	0.00002
0.61,48.5	0.00002
0.61,48.6	0.00002
0.61,48.7	0.00002
0.61,48.8	0.00002
0.61,48.9	0.00002
0.61,49.0	0.00002
0.61,49.1	0.00002
0.61,49.2	0.00002
0.61,49.3	0.00002
0.61,49.4	0.00002
0.61,49.5	0.00002
0.61,49.6	0.00002
0.61,49.7	0.00002
0.61,49.8	0.00002
0.61,49.9	0.00002
0.61,50.0	0.00002
0.61,50.1	0.00002
0.61,50.2	0.00002

0.61,50.3	0.00002
0.61,50.4	0.00002
0.61,50.5	0.00002
0.61,50.6	0.00002
0.61,50.7	0.00002
0.61,50.8	0.00002
0.61,50.9	0.00002
0.61,51.0	0.00002
0.61,51.1	0.00002
0.61,51.2	0.00002
0.61,51.3	0.00002
0.61,51.4	0.00002
0.61,51.5	0.00002
0.61,51.6	0.00002
0.61,51.7	0.00002
0.61,51.8	0.00002
0.61,51.9	0.00002
0.61,52.0	0.00002
0.61,52.1	0.00002
0.61,52.2	0.00002
0.61,52.3	0.00002
0.61,52.4	0.00002
0.61,52.5	0.00002
0.61,52.6	0.00002
0.61,52.7	0.00002
0.61,52.8	0.00002
0.61,52.9	0.00002
0.61,53.0	0.00002
0.61,53.1	0.00002
0.61,53.2	0.00002
0.61,53.3	0.00002
0.61,53.4	0.00002
0.61,53.5	0.00002
0.61,53.6	0.00002
0.61,53.7	0.00002
0.61,53.8	0.00002
0.61,53.9	0.00002
0.61,54.0	0.00002
0.61,54.1	0.00002
0.61,54.2	0.00002
0.61,54.3	0.00002
0.61,54.4	0.00002

0.61,54.5	0.00002
0.61,54.6	0.00002
0.61,54.7	0.00002
0.61,54.8	0.00002
0.61,54.9	0.00002
0.61,55.0	0.00002
0.61,55.1	0.00002
0.61,55.2	0.00002
0.61,55.3	0.00002
0.61,55.4	0.00002
0.61,55.5	0.00002
0.61,55.6	0.00002
0.61,55.7	0.00002
0.61,55.8	0.00002
0.61,55.9	0.00002
0.61,56.0	0.00002
0.61,56.1	0.00002
0.61,56.2	0.00002
0.61,56.3	0.00002
0.61,56.4	0.00002
0.61,56.5	0.00002
0.61,56.6	0.00002
0.61,56.7	0.00002
0.61,56.8	0.00002
0.61,56.9	0.00002
0.61,57.0	0.00002
0.61,57.1	0.00002
0.61,57.2	0.00002
0.61,57.3	0.00002
0.61,57.4	0.00002
0.61,57.5	0.00002
0.61,57.6	0.00002
0.61,57.7	0.00002
0.61,57.8	0.00002
0.61,57.9	0.00002
0.61,58.0	0.00002
0.61,58.1	0.00002
0.61,58.2	0.00002
0.61,58.3	0.00002
0.61,58.4	0.00002
0.61,58.5	0.00002
0.61,58.6	0.00002

0.61,58.7	0.00002
0.61,58.8	0.00002
0.61,58.9	0.00002
0.61,59.0	0.00002
0.61,59.1	0.00002
0.61,59.2	0.00002
0.61,59.3	0.00002
0.61,59.4	0.00002
0.61,59.5	0.00002
0.61,59.6	0.00002
0.61,59.7	0.00002
0.61,59.8	0.00002
0.61,59.9	0.00002
0.61,60.0	0.00002
0.61,60.1	0.00002
0.61,60.2	0.00002
0.61,60.3	0.00002
0.61,60.4	0.00002
0.61,60.5	0.00002
0.61,60.6	0.00002
0.61,60.7	0.00002
0.61,60.8	0.00002
0.61,60.9	0.00002
0.61,61.0	0.00002
0.61,61.1	0.00002
0.61,61.2	0.00002
0.61,61.3	0.00002
0.61,61.4	0.00002
0.61,61.5	0.00002
0.61,61.6	0.00002
0.61,61.7	0.00002
0.61,61.8	0.00002
0.61,61.9	0.00002
0.61,62.0	0.00002
0.61,62.1	0.00002
0.61,62.2	0.00002
0.61,62.3	0.00002
0.61,62.4	0.00002
0.61,62.5	0.00002
0.61,62.6	0.00002
0.61,62.7	0.00002
0.61,62.8	0.00002

0.61,62.9	0.00002
0.61,63.0	0.00002
0.61,63.1	0.00002
0.61,63.2	0.00002
0.61,63.3	0.00002
0.61,63.4	0.00002
0.61,63.5	0.00002
0.61,63.6	0.00002
0.61,63.7	0.00002
0.61,63.8	0.00002
0.61,63.9	0.00002
0.61,64.0	0.00002
0.61,64.1	0.00002
0.61,64.2	0.00002
0.61,64.3	0.00002
0.61,64.4	0.00002
0.61,64.5	0.00002
0.61,64.6	0.00002
0.61,64.7	0.00002
0.61,64.8	0.00002
0.61,64.9	0.00002
0.61,65.0	0.00002
0.61,65.1	0.00002
0.61,65.2	0.00002
0.61,65.3	0.00002
0.61,65.4	0.00002
0.61,65.5	0.00002
0.61,65.6	0.00002
0.61,65.7	0.00002
0.61,65.8	0.00002
0.61,65.9	0.00002
0.61,66.0	0.00002
0.61,66.1	0.00002
0.61,66.2	0.00002
0.61,66.3	0.00002
0.61,66.4	0.00002
0.61,66.5	0.00002
0.61,66.6	0.00002
0.61,66.7	0.00002
0.61,66.8	0.00002
0.61,66.9	0.00002
0.61,67.0	0.00002

0.61,67.1	0.00002
0.61,67.2	0.00002
0.61,67.3	0.00002
0.61,67.4	0.00002
0.61,67.5	0.00002
0.61,67.6	0.00002
0.61,67.7	0.00002
0.61,67.8	0.00002
0.61,67.9	0.00002
0.61,68.0	0.00002
0.61,68.1	0.00002
0.61,68.2	0.00002
0.61,68.3	0.00002
0.61,68.4	0.00002
0.61,68.5	0.00002
0.61,68.6	0.00002
0.61,68.7	0.00002
0.61,68.8	0.00002
0.61,68.9	0.00002
0.61,69.0	0.00002
0.61,69.1	0.00002
0.61,69.2	0.00002
0.61,69.3	0.00002
0.61,69.4	0.00002
0.61,69.5	0.00002
0.61,69.6	0.00002
0.61,69.7	0.00002
0.61,69.8	0.00002
0.61,69.9	0.00002
0.61,70.0	0.00002
0.61,70.1	0.00002
0.61,70.2	0.00002
0.61,70.3	0.00002
0.61,70.4	0.00002
0.61,70.5	0.00002
0.61,70.6	0.00002
0.61,70.7	0.00002
0.61,70.8	0.00002
0.61,70.9	0.00002
0.61,71.0	0.00002
0.61,71.1	0.00002
0.61,71.2	0.00002

0.61,71.3	0.00002
0.61,71.4	0.00002
0.61,71.5	0.00002
0.61,71.6	0.00002
0.61,71.7	0.00002
0.61,71.8	0.00002
0.61,71.9	0.00002
0.61,72.0	0.00002
0.61,72.1	0.00002
0.61,72.2	0.00002
0.61,72.3	0.00002
0.61,72.4	0.00002
0.61,72.5	0.00002
0.61,72.6	0.00002
0.61,72.7	0.00002
0.61,72.8	0.00002
0.61,72.9	0.00002
0.61,73.0	0.00002
0.61,73.1	0.00002
0.61,73.2	0.00002
0.61,73.3	0.00002
0.61,73.4	0.00002
0.61,73.5	0.00002
0.61,73.6	0.00002
0.61,73.7	0.00002
0.61,73.8	0.00002
0.61,73.9	0.00002
0.61,74.0	0.00002
0.61,74.1	0.00002
0.61,74.2	0.00002
0.61,74.3	0.00002
0.61,74.4	0.00002
0.61,74.5	0.00002
0.61,74.6	0.00002
0.61,74.7	0.00002
0.61,74.8	0.00002
0.61,74.9	0.00002
0.61,75.0	0.00002
0.61,75.1	0.00002
0.61,75.2	0.00002
0.61,75.3	0.00002
0.61,75.4	0.00002

0.61,75.5	0.00002
0.61,75.6	0.00002
0.61,75.7	0.00002
0.61,75.8	0.00002
0.61,75.9	0.00002
0.61,76.0	0.00002
0.61,76.1	0.000021
0.61,76.2	0.000021
0.61,76.3	0.000021
0.61,76.4	0.000021
0.61,76.5	0.000021
0.61,76.6	0.000021
0.61,76.7	0.000022
0.61,76.8	0.000022
0.61,76.9	0.000022
0.61,77.0	0.000022
0.61,77.1	0.000022
0.61,77.2	0.000022
0.61,77.3	0.000022
0.61,77.4	0.000022
0.61,77.5	0.000022
0.61,77.6	0.000022
0.61,77.7	0.000022
0.61,77.8	0.000022
0.61,77.9	0.000022
0.61,78.0	0.000022
0.61,78.1	0.000022
0.61,78.2	0.000022
0.61,78.3	0.000022
0.61,78.4	0.000022
0.61,78.5	0.000022
0.61,78.6	0.000022
0.61,78.7	0.000022
0.61,78.8	0.000022
0.61,78.9	0.000022
0.61,79.0	0.000022
0.61,79.1	0.000022
0.61,79.2	0.000022
0.61,79.3	0.000022
0.61,79.4	0.000022
0.61,79.5	0.000022
0.61,79.6	0.000022

0.61,79.7	0.000023
0.61,79.8	0.000023
0.61,79.9	0.000023
0.61,80.0	0.000023
0.61,80.1	0.000023
0.61,80.2	0.000023
0.61,80.3	0.000024
0.61,80.4	0.000024
0.61,80.5	0.000024
0.61,80.6	0.000024
0.61,80.7	0.000024
0.61,80.8	0.000024
0.61,80.9	0.000024
0.61,81.0	0.000024
0.61,81.1	0.000024
0.61,81.2	0.000027
0.61,81.3	0.000073
0.61,81.4	0.000173
0.61,81.5	0.000258
0.61,81.6	0.000403
0.61,81.7	0.000548
0.61,81.8	0.000601
0.61,81.9	0.000648
0.61,82.0	0.000694
0.61,82.1	0.000737
0.61,82.2	0.000774
0.61,82.3	0.000819
0.61,82.4	0.000878
0.61,82.5	0.000945
0.61,82.6	0.000981
0.61,82.7	0.001035
0.61,82.8	0.001092
0.61,82.9	0.001177
0.61,83.0	0.00124
0.61,83.1	0.001294
0.61,83.2	0.001314
0.61,83.3	0.001358
0.61,83.4	0.001409
0.61,83.5	0.00157
0.61,83.6	0.001669
0.61,83.7	0.001693
0.61,83.8	0.001723

0.61,83.9	0.001738
0.61,84.0	0.001747
0.61,84.1	0.001811
0.61,84.2	0.001868
0.61,84.3	0.001944
0.61,84.4	0.001965
0.61,84.5	0.002007
0.61,84.6	0.002018
0.61,84.7	0.002018
0.61,84.8	0.002024
0.61,84.9	0.002038
0.61,85.0	0.002041
0.61,85.1	0.002053
0.61,85.2	0.002053
0.61,85.3	0.002068
0.61,85.4	0.002086
0.61,85.5	0.002101
0.61,85.6	0.002117
0.61,85.7	0.00212
0.61,85.8	0.00212
0.61,85.9	0.00212
0.61,86.0	0.002126
0.61,86.1	0.002136
0.61,86.2	0.002155
0.61,86.3	0.002195
0.61,86.4	0.00225
0.61,86.5	0.002301
0.61,86.6	0.002369
0.61,86.7	0.002413
0.61,86.8	0.002455
0.61,86.9	0.002485
0.61,87.0	0.002511
0.61,87.1	0.00252
0.61,87.2	0.002529
0.61,87.3	0.002551
0.61,87.4	0.002551
0.61,87.5	0.00257
0.61,87.6	0.002593
0.61,87.7	0.002612
0.61,87.8	0.002612
0.61,87.9	0.002622
0.61,88.0	0.002641

0.61,88.1	0.002686
0.61,88.2	0.002706
0.61,88.3	0.002753
0.61,88.4	0.002763
0.61,88.5	0.002774
0.61,88.6	0.00278
0.61,88.7	0.002785
0.61,88.8	0.002785
0.61,88.9	0.002796
0.61,89.0	0.002807
0.61,89.1	0.002824
0.61,89.2	0.00284
0.61,89.3	0.00284
0.61,89.4	0.002852
0.61,89.5	0.002863
0.61,89.6	0.002892
0.61,89.7	0.00294
0.61,89.8	0.002989
0.61,89.9	0.003027
0.61,90.0	0.003059
0.61,90.1	0.003073
0.61,90.2	0.003086
0.61,90.3	0.003106
0.61,90.4	0.003161
0.61,90.5	0.003196
0.61,90.6	0.003211
0.61,90.7	0.003232
0.61,90.8	0.003247
0.61,90.9	0.003254
0.61,91.0	0.003262
0.61,91.1	0.003269
0.61,91.2	0.003307
0.61,91.3	0.00341
0.61,91.4	0.00341
0.61,91.5	0.003426
0.61,91.6	0.003493
0.61,91.7	0.00351
0.61,91.8	0.003599
0.61,91.9	0.003701
0.61,92.0	0.00379
0.61,92.1	0.003872
0.61,92.2	0.003914

0.61,92.3	0.003936
0.61,92.4	0.003936
0.61,92.5	0.003958
0.61,92.6	0.004002
0.61,92.7	0.004013
0.61,92.8	0.004047
0.61,92.9	0.00407
0.61,93.0	0.004082
0.61,93.1	0.004117
0.61,93.2	0.004117
0.61,93.3	0.004129
0.61,93.4	0.004153
0.61,93.5	0.004153
0.61,93.6	0.004153
0.61,93.7	0.004153
0.61,93.8	0.004153
0.61,93.9	0.004165
0.61,94.0	0.004178
0.61,94.1	0.004178
0.61,94.2	0.004215
0.61,94.3	0.004215
0.61,94.4	0.004227
0.61,94.5	0.00424
0.61,94.6	0.00424
0.61,94.7	0.00424
0.61,94.8	0.004252
0.61,94.9	0.00429
0.61,95.0	0.004316
0.61,95.1	0.004343
0.61,95.2	0.004396
0.61,95.3	0.00441
0.61,95.4	0.004423
0.61,95.5	0.004437
0.61,95.6	0.004437
0.61,95.7	0.004437
0.61,95.8	0.004451
0.61,95.9	0.00445
0.61,96.0	0.00445
0.61,96.1	0.00445
0.61,96.2	0.004464
0.61,96.3	0.004478
0.61,96.4	0.004507

0.61,96.5	0.004507
0.61,96.6	0.004507
0.61,96.7	0.004507
0.61,96.8	0.004507
0.61,96.9	0.004507
0.61,97.0	0.004507
0.61,97.1	0.004507
0.61,97.2	0.004507
0.61,97.3	0.004507
0.61,97.4	0.004507
0.61,97.5	0.004507
0.61,97.6	0.004507
0.61,97.7	0.004507
0.61,97.8	0.004507
0.61,97.9	0.004507
0.61,98.0	0.004507
0.61,98.1	0.004507
0.61,98.2	0.004507
0.61,98.3	0.004507
0.61,98.4	0.004507
0.61,98.5	0.004507
0.61,98.6	0.004507
0.61,98.7	0.004507
0.61,98.8	0.004507
0.61,98.9	0.004507
0.61,99.0	0.004507
0.61,99.1	0.004507
0.61,99.2	0.004507
0.61,99.3	0.004507
0.61,99.4	0.004507
0.61,99.5	0.004507
0.61,99.6	0.004507
0.61,99.7	0.004507
0.61,99.8	0.004507
0.61,99.9	0.004507
0.61,100.0	0.004507
0.62,0.0	0.000058
0.62,0.1	0.000058
0.62,0.2	0.000058
0.62,0.3	0.000058
0.62,0.4	0.000058
0.62,0.5	0.000058

0.62,0.6	0.000058
0.62,0.7	0.000058
0.62,0.8	0.000058
0.62,0.9	0.000058
0.62,1.0	0.000058
0.62,1.1	0.000058
0.62,1.2	0.000058
0.62,1.3	0.000058
0.62,1.4	0.000058
0.62,1.5	0.000058
0.62,1.6	0.000058
0.62,1.7	0.000058
0.62,1.8	0.000058
0.62,1.9	0.000058
0.62,2.0	0.000058
0.62,2.1	0.000058
0.62,2.2	0.000058
0.62,2.3	0.000058
0.62,2.4	0.000058
0.62,2.5	0.000058
0.62,2.6	0.000058
0.62,2.7	0.000058
0.62,2.8	0.000058
0.62,2.9	0.000058
0.62,3.0	0.000058
0.62,3.1	0.000058
0.62,3.2	0.000058
0.62,3.3	0.000058
0.62,3.4	0.000058
0.62,3.5	0.000058
0.62,3.6	0.000058
0.62,3.7	0.000058
0.62,3.8	0.000058
0.62,3.9	0.000058
0.62,4.0	0.000058
0.62,4.1	0.000058
0.62,4.2	0.000058
0.62,4.3	0.000058
0.62,4.4	0.000058
0.62,4.5	0.000058
0.62,4.6	0.000058
0.62,4.7	0.000058

0.62,4.8	0.000058
0.62,4.9	0.000058
0.62,5.0	0.000058
0.62,5.1	0.000058
0.62,5.2	0.000058
0.62,5.3	0.000058
0.62,5.4	0.000058
0.62,5.5	0.000058
0.62,5.6	0.000058
0.62,5.7	0.000058
0.62,5.8	0.000058
0.62,5.9	0.000058
0.62,6.0	0.000058
0.62,6.1	0.000058
0.62,6.2	0.000058
0.62,6.3	0.000058
0.62,6.4	0.000058
0.62,6.5	0.000058
0.62,6.6	0.000058
0.62,6.7	0.000058
0.62,6.8	0.000058
0.62,6.9	0.000058
0.62,7.0	0.000058
0.62,7.1	0.000058
0.62,7.2	0.000058
0.62,7.3	0.000058
0.62,7.4	0.000058
0.62,7.5	0.000058
0.62,7.6	0.000058
0.62,7.7	0.000058
0.62,7.8	0.000058
0.62,7.9	0.000058
0.62,8.0	0.000058
0.62,8.1	0.000058
0.62,8.2	0.000058
0.62,8.3	0.000058
0.62,8.4	0.000058
0.62,8.5	0.000058
0.62,8.6	0.000058
0.62,8.7	0.000058
0.62,8.8	0.000058
0.62,8.9	0.000058

0.62,9.0	0.000058
0.62,9.1	0.000058
0.62,9.2	0.000058
0.62,9.3	0.000058
0.62,9.4	0.000058
0.62,9.5	0.000058
0.62,9.6	0.000058
0.62,9.7	0.000058
0.62,9.8	0.000058
0.62,9.9	0.000058
0.62,10.0	0.000058
0.62,10.1	0.000058
0.62,10.2	0.000058
0.62,10.3	0.000058
0.62,10.4	0.000058
0.62,10.5	0.000058
0.62,10.6	0.000058
0.62,10.7	0.000058
0.62,10.8	0.000058
0.62,10.9	0.000058
0.62,11.0	0.000058
0.62,11.1	0.000058
0.62,11.2	0.000058
0.62,11.3	0.000058
0.62,11.4	0.000058
0.62,11.5	0.000058
0.62,11.6	0.000058
0.62,11.7	0.000058
0.62,11.8	0.000058
0.62,11.9	0.000058
0.62,12.0	0.000058
0.62,12.1	0.000058
0.62,12.2	0.000058
0.62,12.3	0.000058
0.62,12.4	0.000058
0.62,12.5	0.000058
0.62,12.6	0.000058
0.62,12.7	0.000058
0.62,12.8	0.000058
0.62,12.9	0.000058
0.62,13.0	0.000058
0.62,13.1	0.000058

0.62,13.2	0.000058
0.62,13.3	0.000058
0.62,13.4	0.000058
0.62,13.5	0.000058
0.62,13.6	0.000058
0.62,13.7	0.000058
0.62,13.8	0.000058
0.62,13.9	0.000058
0.62,14.0	0.000058
0.62,14.1	0.000058
0.62,14.2	0.000058
0.62,14.3	0.000058
0.62,14.4	0.000058
0.62,14.5	0.000058
0.62,14.6	0.000058
0.62,14.7	0.000058
0.62,14.8	0.000058
0.62,14.9	0.000058
0.62,15.0	0.000058
0.62,15.1	0.000058
0.62,15.2	0.000058
0.62,15.3	0.000058
0.62,15.4	0.000058
0.62,15.5	0.000058
0.62,15.6	0.000058
0.62,15.7	0.000058
0.62,15.8	0.000058
0.62,15.9	0.000058
0.62,16.0	0.000058
0.62,16.1	0.000058
0.62,16.2	0.000058
0.62,16.3	0.000058
0.62,16.4	0.000058
0.62,16.5	0.000058
0.62,16.6	0.000058
0.62,16.7	0.000058
0.62,16.8	0.000058
0.62,16.9	0.000058
0.62,17.0	0.000058
0.62,17.1	0.000058
0.62,17.2	0.000058
0.62,17.3	0.000058

0.62,17.4	0.000058
0.62,17.5	0.000058
0.62,17.6	0.000058
0.62,17.7	0.000058
0.62,17.8	0.000058
0.62,17.9	0.000058
0.62,18.0	0.000058
0.62,18.1	0.000058
0.62,18.2	0.000058
0.62,18.3	0.000058
0.62,18.4	0.000058
0.62,18.5	0.000058
0.62,18.6	0.000058
0.62,18.7	0.000058
0.62,18.8	0.000058
0.62,18.9	0.000058
0.62,19.0	0.000058
0.62,19.1	0.000058
0.62,19.2	0.000058
0.62,19.3	0.000058
0.62,19.4	0.000058
0.62,19.5	0.000058
0.62,19.6	0.000058
0.62,19.7	0.000058
0.62,19.8	0.000058
0.62,19.9	0.000058
0.62,20.0	0.000058
0.62,20.1	0.000058
0.62,20.2	0.000058
0.62,20.3	0.000058
0.62,20.4	0.000058
0.62,20.5	0.000058
0.62,20.6	0.000058
0.62,20.7	0.000058
0.62,20.8	0.000058
0.62,20.9	0.000058
0.62,21.0	0.000058
0.62,21.1	0.000058
0.62,21.2	0.000058
0.62,21.3	0.000058
0.62,21.4	0.000058
0.62,21.5	0.000058

0.62,21.6	0.000058
0.62,21.7	0.000058
0.62,21.8	0.000058
0.62,21.9	0.000058
0.62,22.0	0.000058
0.62,22.1	0.000058
0.62,22.2	0.000058
0.62,22.3	0.000058
0.62,22.4	0.000058
0.62,22.5	0.000058
0.62,22.6	0.000058
0.62,22.7	0.000058
0.62,22.8	0.000058
0.62,22.9	0.000058
0.62,23.0	0.000058
0.62,23.1	0.000058
0.62,23.2	0.000058
0.62,23.3	0.000058
0.62,23.4	0.000058
0.62,23.5	0.000058
0.62,23.6	0.000058
0.62,23.7	0.000058
0.62,23.8	0.000058
0.62,23.9	0.000058
0.62,24.0	0.000058
0.62,24.1	0.000058
0.62,24.2	0.000058
0.62,24.3	0.000058
0.62,24.4	0.000058
0.62,24.5	0.000058
0.62,24.6	0.000058
0.62,24.7	0.000058
0.62,24.8	0.000058
0.62,24.9	0.000058
0.62,25.0	0.000058
0.62,25.1	0.000058
0.62,25.2	0.000058
0.62,25.3	0.000058
0.62,25.4	0.000058
0.62,25.5	0.000058
0.62,25.6	0.000058
0.62,25.7	0.000058

0.62,25.8	0.000058
0.62,25.9	0.000058
0.62,26.0	0.000058
0.62,26.1	0.000058
0.62,26.2	0.000058
0.62,26.3	0.000058
0.62,26.4	0.000058
0.62,26.5	0.000058
0.62,26.6	0.000058
0.62,26.7	0.000058
0.62,26.8	0.000058
0.62,26.9	0.000058
0.62,27.0	0.000058
0.62,27.1	0.000058
0.62,27.2	0.000058
0.62,27.3	0.000058
0.62,27.4	0.000058
0.62,27.5	0.000058
0.62,27.6	0.000058
0.62,27.7	0.000058
0.62,27.8	0.000058
0.62,27.9	0.000058
0.62,28.0	0.000058
0.62,28.1	0.000058
0.62,28.2	0.000058
0.62,28.3	0.000058
0.62,28.4	0.000058
0.62,28.5	0.000058
0.62,28.6	0.000058
0.62,28.7	0.000058
0.62,28.8	0.000058
0.62,28.9	0.000058
0.62,29.0	0.000058
0.62,29.1	0.000058
0.62,29.2	0.000058
0.62,29.3	0.000058
0.62,29.4	0.000058
0.62,29.5	0.000058
0.62,29.6	0.000058
0.62,29.7	0.000058
0.62,29.8	0.000058
0.62,29.9	0.000058

0.62,30.0	0.000058
0.62,30.1	0.000058
0.62,30.2	0.000058
0.62,30.3	0.000058
0.62,30.4	0.000058
0.62,30.5	0.000058
0.62,30.6	0.000058
0.62,30.7	0.000058
0.62,30.8	0.000058
0.62,30.9	0.000058
0.62,31.0	0.000058
0.62,31.1	0.000058
0.62,31.2	0.000058
0.62,31.3	0.000058
0.62,31.4	0.000058
0.62,31.5	0.000058
0.62,31.6	0.000058
0.62,31.7	0.000058
0.62,31.8	0.000058
0.62,31.9	0.000058
0.62,32.0	0.000058
0.62,32.1	0.000058
0.62,32.2	0.000058
0.62,32.3	0.000058
0.62,32.4	0.000058
0.62,32.5	0.000058
0.62,32.6	0.000058
0.62,32.7	0.000058
0.62,32.8	0.000058
0.62,32.9	0.000058
0.62,33.0	0.000058
0.62,33.1	0.000058
0.62,33.2	0.000058
0.62,33.3	0.000058
0.62,33.4	0.000058
0.62,33.5	0.000058
0.62,33.6	0.000058
0.62,33.7	0.000058
0.62,33.8	0.000058
0.62,33.9	0.000058
0.62,34.0	0.000058
0.62,34.1	0.000058

0.62,34.2	0.000058
0.62,34.3	0.000058
0.62,34.4	0.000058
0.62,34.5	0.000058
0.62,34.6	0.000058
0.62,34.7	0.000058
0.62,34.8	0.000058
0.62,34.9	0.000058
0.62,35.0	0.000058
0.62,35.1	0.000058
0.62,35.2	0.000058
0.62,35.3	0.000058
0.62,35.4	0.000058
0.62,35.5	0.000058
0.62,35.6	0.000058
0.62,35.7	0.000058
0.62,35.8	0.000058
0.62,35.9	0.000058
0.62,36.0	0.000058
0.62,36.1	0.000058
0.62,36.2	0.000058
0.62,36.3	0.000058
0.62,36.4	0.000058
0.62,36.5	0.000058
0.62,36.6	0.000058
0.62,36.7	0.000058
0.62,36.8	0.000058
0.62,36.9	0.000058
0.62,37.0	0.000058
0.62,37.1	0.000058
0.62,37.2	0.000058
0.62,37.3	0.000058
0.62,37.4	0.000058
0.62,37.5	0.000058
0.62,37.6	0.000058
0.62,37.7	0.000058
0.62,37.8	0.000058
0.62,37.9	0.000058
0.62,38.0	0.000058
0.62,38.1	0.000058
0.62,38.2	0.000058
0.62,38.3	0.000058

0.62,38.4	0.000058
0.62,38.5	0.000058
0.62,38.6	0.000058
0.62,38.7	0.000058
0.62,38.8	0.000058
0.62,38.9	0.000058
0.62,39.0	0.000058
0.62,39.1	0.000058
0.62,39.2	0.000058
0.62,39.3	0.000058
0.62,39.4	0.000058
0.62,39.5	0.000058
0.62,39.6	0.000058
0.62,39.7	0.000058
0.62,39.8	0.000058
0.62,39.9	0.000058
0.62,40.0	0.000058
0.62,40.1	0.000058
0.62,40.2	0.000058
0.62,40.3	0.000058
0.62,40.4	0.000058
0.62,40.5	0.000058
0.62,40.6	0.000058
0.62,40.7	0.000058
0.62,40.8	0.000058
0.62,40.9	0.000058
0.62,41.0	0.000058
0.62,41.1	0.000058
0.62,41.2	0.000058
0.62,41.3	0.000058
0.62,41.4	0.000058
0.62,41.5	0.000058
0.62,41.6	0.000058
0.62,41.7	0.000058
0.62,41.8	0.000058
0.62,41.9	0.000058
0.62,42.0	0.000058
0.62,42.1	0.000058
0.62,42.2	0.000058
0.62,42.3	0.000058
0.62,42.4	0.000058
0.62,42.5	0.000058

0.62,42.6	0.000058
0.62,42.7	0.000058
0.62,42.8	0.000058
0.62,42.9	0.000058
0.62,43.0	0.000058
0.62,43.1	0.000058
0.62,43.2	0.000058
0.62,43.3	0.000058
0.62,43.4	0.000058
0.62,43.5	0.000058
0.62,43.6	0.000058
0.62,43.7	0.000058
0.62,43.8	0.000058
0.62,43.9	0.000058
0.62,44.0	0.000058
0.62,44.1	0.000058
0.62,44.2	0.000058
0.62,44.3	0.000058
0.62,44.4	0.000058
0.62,44.5	0.000058
0.62,44.6	0.000058
0.62,44.7	0.000058
0.62,44.8	0.000058
0.62,44.9	0.000058
0.62,45.0	0.000058
0.62,45.1	0.000058
0.62,45.2	0.000058
0.62,45.3	0.000058
0.62,45.4	0.000058
0.62,45.5	0.000058
0.62,45.6	0.000058
0.62,45.7	0.000058
0.62,45.8	0.000058
0.62,45.9	0.000058
0.62,46.0	0.000058
0.62,46.1	0.000058
0.62,46.2	0.000058
0.62,46.3	0.000058
0.62,46.4	0.000058
0.62,46.5	0.000058
0.62,46.6	0.000058
0.62,46.7	0.000058

0.62,46.8	0.000058
0.62,46.9	0.000058
0.62,47.0	0.000058
0.62,47.1	0.000058
0.62,47.2	0.000058
0.62,47.3	0.000058
0.62,47.4	0.000058
0.62,47.5	0.000058
0.62,47.6	0.000058
0.62,47.7	0.000058
0.62,47.8	0.000058
0.62,47.9	0.000058
0.62,48.0	0.000058
0.62,48.1	0.000058
0.62,48.2	0.000058
0.62,48.3	0.000058
0.62,48.4	0.000058
0.62,48.5	0.000058
0.62,48.6	0.000058
0.62,48.7	0.000058
0.62,48.8	0.000058
0.62,48.9	0.000058
0.62,49.0	0.000058
0.62,49.1	0.000058
0.62,49.2	0.000058
0.62,49.3	0.000058
0.62,49.4	0.000058
0.62,49.5	0.000058
0.62,49.6	0.000058
0.62,49.7	0.000058
0.62,49.8	0.000058
0.62,49.9	0.000058
0.62,50.0	0.000058
0.62,50.1	0.000058
0.62,50.2	0.000058
0.62,50.3	0.000058
0.62,50.4	0.000058
0.62,50.5	0.000058
0.62,50.6	0.000058
0.62,50.7	0.000058
0.62,50.8	0.000058
0.62,50.9	0.000058

0.62,51.0	0.000058
0.62,51.1	0.000058
0.62,51.2	0.000058
0.62,51.3	0.000058
0.62,51.4	0.000058
0.62,51.5	0.000058
0.62,51.6	0.000058
0.62,51.7	0.000058
0.62,51.8	0.000058
0.62,51.9	0.000058
0.62,52.0	0.000058
0.62,52.1	0.000058
0.62,52.2	0.000058
0.62,52.3	0.000058
0.62,52.4	0.000058
0.62,52.5	0.000058
0.62,52.6	0.000058
0.62,52.7	0.000058
0.62,52.8	0.000058
0.62,52.9	0.000058
0.62,53.0	0.000058
0.62,53.1	0.000058
0.62,53.2	0.000058
0.62,53.3	0.000058
0.62,53.4	0.000058
0.62,53.5	0.000058
0.62,53.6	0.000058
0.62,53.7	0.000058
0.62,53.8	0.000058
0.62,53.9	0.000058
0.62,54.0	0.000058
0.62,54.1	0.000058
0.62,54.2	0.000058
0.62,54.3	0.000058
0.62,54.4	0.000058
0.62,54.5	0.000058
0.62,54.6	0.000058
0.62,54.7	0.000058
0.62,54.8	0.000058
0.62,54.9	0.000058
0.62,55.0	0.000058
0.62,55.1	0.000058

0.62,55.2	0.000058
0.62,55.3	0.000058
0.62,55.4	0.000058
0.62,55.5	0.000058
0.62,55.6	0.000058
0.62,55.7	0.000058
0.62,55.8	0.000058
0.62,55.9	0.000058
0.62,56.0	0.000058
0.62,56.1	0.000058
0.62,56.2	0.000058
0.62,56.3	0.000058
0.62,56.4	0.000058
0.62,56.5	0.000058
0.62,56.6	0.000058
0.62,56.7	0.000058
0.62,56.8	0.000058
0.62,56.9	0.000058
0.62,57.0	0.000058
0.62,57.1	0.000058
0.62,57.2	0.000058
0.62,57.3	0.000058
0.62,57.4	0.000058
0.62,57.5	0.000058
0.62,57.6	0.000058
0.62,57.7	0.000058
0.62,57.8	0.000058
0.62,57.9	0.000058
0.62,58.0	0.000058
0.62,58.1	0.000058
0.62,58.2	0.000058
0.62,58.3	0.000058
0.62,58.4	0.000058
0.62,58.5	0.000058
0.62,58.6	0.000058
0.62,58.7	0.000058
0.62,58.8	0.000058
0.62,58.9	0.000058
0.62,59.0	0.000058
0.62,59.1	0.000058
0.62,59.2	0.000058
0.62,59.3	0.000058

0.62,59.4	0.000058
0.62,59.5	0.000058
0.62,59.6	0.000058
0.62,59.7	0.000058
0.62,59.8	0.000058
0.62,59.9	0.000058
0.62,60.0	0.000058
0.62,60.1	0.000058
0.62,60.2	0.000058
0.62,60.3	0.000058
0.62,60.4	0.000058
0.62,60.5	0.000058
0.62,60.6	0.000058
0.62,60.7	0.000058
0.62,60.8	0.000058
0.62,60.9	0.000058
0.62,61.0	0.000058
0.62,61.1	0.000058
0.62,61.2	0.000058
0.62,61.3	0.000058
0.62,61.4	0.000058
0.62,61.5	0.000058
0.62,61.6	0.000058
0.62,61.7	0.000058
0.62,61.8	0.000058
0.62,61.9	0.000058
0.62,62.0	0.000058
0.62,62.1	0.000058
0.62,62.2	0.000058
0.62,62.3	0.000058
0.62,62.4	0.000058
0.62,62.5	0.000058
0.62,62.6	0.000058
0.62,62.7	0.000058
0.62,62.8	0.000058
0.62,62.9	0.000058
0.62,63.0	0.000058
0.62,63.1	0.000058
0.62,63.2	0.000058
0.62,63.3	0.000058
0.62,63.4	0.000058
0.62,63.5	0.000058

0.62,63.6	0.000058
0.62,63.7	0.000058
0.62,63.8	0.000058
0.62,63.9	0.000058
0.62,64.0	0.000058
0.62,64.1	0.000058
0.62,64.2	0.000058
0.62,64.3	0.000058
0.62,64.4	0.000058
0.62,64.5	0.000058
0.62,64.6	0.000058
0.62,64.7	0.000058
0.62,64.8	0.000058
0.62,64.9	0.000058
0.62,65.0	0.000058
0.62,65.1	0.000058
0.62,65.2	0.000058
0.62,65.3	0.000058
0.62,65.4	0.000058
0.62,65.5	0.000058
0.62,65.6	0.000058
0.62,65.7	0.000058
0.62,65.8	0.000058
0.62,65.9	0.000058
0.62,66.0	0.000058
0.62,66.1	0.000058
0.62,66.2	0.000058
0.62,66.3	0.000058
0.62,66.4	0.000058
0.62,66.5	0.000058
0.62,66.6	0.000058
0.62,66.7	0.000058
0.62,66.8	0.000058
0.62,66.9	0.000058
0.62,67.0	0.000058
0.62,67.1	0.000058
0.62,67.2	0.000058
0.62,67.3	0.000058
0.62,67.4	0.000058
0.62,67.5	0.000058
0.62,67.6	0.000058
0.62,67.7	0.000058

0.62,67.8	0.000058
0.62,67.9	0.000058
0.62,68.0	0.000058
0.62,68.1	0.000058
0.62,68.2	0.000058
0.62,68.3	0.000058
0.62,68.4	0.000058
0.62,68.5	0.000058
0.62,68.6	0.000058
0.62,68.7	0.000058
0.62,68.8	0.000058
0.62,68.9	0.000058
0.62,69.0	0.000058
0.62,69.1	0.000058
0.62,69.2	0.000058
0.62,69.3	0.000058
0.62,69.4	0.000058
0.62,69.5	0.000058
0.62,69.6	0.000058
0.62,69.7	0.000058
0.62,69.8	0.000058
0.62,69.9	0.000058
0.62,70.0	0.000058
0.62,70.1	0.000058
0.62,70.2	0.000058
0.62,70.3	0.000058
0.62,70.4	0.000058
0.62,70.5	0.000058
0.62,70.6	0.000058
0.62,70.7	0.000058
0.62,70.8	0.000058
0.62,70.9	0.000058
0.62,71.0	0.000058
0.62,71.1	0.000058
0.62,71.2	0.000058
0.62,71.3	0.000058
0.62,71.4	0.000058
0.62,71.5	0.000058
0.62,71.6	0.000058
0.62,71.7	0.000058
0.62,71.8	0.000058
0.62,71.9	0.000058

0.62,72.0	0.000058
0.62,72.1	0.000058
0.62,72.2	0.000058
0.62,72.3	0.000058
0.62,72.4	0.000058
0.62,72.5	0.000058
0.62,72.6	0.000058
0.62,72.7	0.000058
0.62,72.8	0.000058
0.62,72.9	0.000058
0.62,73.0	0.000058
0.62,73.1	0.000058
0.62,73.2	0.000058
0.62,73.3	0.000058
0.62,73.4	0.000058
0.62,73.5	0.000058
0.62,73.6	0.000058
0.62,73.7	0.000058
0.62,73.8	0.000058
0.62,73.9	0.000058
0.62,74.0	0.000058
0.62,74.1	0.000058
0.62,74.2	0.000058
0.62,74.3	0.000058
0.62,74.4	0.000058
0.62,74.5	0.000058
0.62,74.6	0.000058
0.62,74.7	0.000058
0.62,74.8	0.000058
0.62,74.9	0.000059
0.62,75.0	0.000059
0.62,75.1	0.000059
0.62,75.2	0.000059
0.62,75.3	0.000059
0.62,75.4	0.000059
0.62,75.5	0.000059
0.62,75.6	0.000059
0.62,75.7	0.000059
0.62,75.8	0.000059
0.62,75.9	0.000059
0.62,76.0	0.000059
0.62,76.1	0.000059

0.62,76.2	0.000059
0.62,76.3	0.000059
0.62,76.4	0.00006
0.62,76.5	0.00006
0.62,76.6	0.00006
0.62,76.7	0.000061
0.62,76.8	0.000061
0.62,76.9	0.000061
0.62,77.0	0.000061
0.62,77.1	0.000061
0.62,77.2	0.000061
0.62,77.3	0.000061
0.62,77.4	0.000061
0.62,77.5	0.000061
0.62,77.6	0.000061
0.62,77.7	0.000061
0.62,77.8	0.000061
0.62,77.9	0.000061
0.62,78.0	0.000061
0.62,78.1	0.000061
0.62,78.2	0.000061
0.62,78.3	0.000061
0.62,78.4	0.000061
0.62,78.5	0.000061
0.62,78.6	0.000061
0.62,78.7	0.000061
0.62,78.8	0.000061
0.62,78.9	0.000061
0.62,79.0	0.000061
0.62,79.1	0.000062
0.62,79.2	0.000062
0.62,79.3	0.000062
0.62,79.4	0.000062
0.62,79.5	0.000062
0.62,79.6	0.000062
0.62,79.7	0.000062
0.62,79.8	0.000063
0.62,79.9	0.000063
0.62,80.0	0.000063
0.62,80.1	0.000064
0.62,80.2	0.000064
0.62,80.3	0.000064

0.62,80.4	0.000064
0.62,80.5	0.000064
0.62,80.6	0.000064
0.62,80.7	0.000064
0.62,80.8	0.000064
0.62,80.9	0.000065
0.62,81.0	0.000065
0.62,81.1	0.000066
0.62,81.2	0.000073
0.62,81.3	0.000188
0.62,81.4	0.000424
0.62,81.5	0.000627
0.62,81.6	0.000949
0.62,81.7	0.001207
0.62,81.8	0.001287
0.62,81.9	0.00136
0.62,82.0	0.001492
0.62,82.1	0.001636
0.62,82.2	0.001748
0.62,82.3	0.00186
0.62,82.4	0.001983
0.62,82.5	0.002097
0.62,82.6	0.002171
0.62,82.7	0.002423
0.62,82.8	0.00257
0.62,82.9	0.002758
0.62,83.0	0.002862
0.62,83.1	0.003018
0.62,83.2	0.003051
0.62,83.3	0.003093
0.62,83.4	0.003141
0.62,83.5	0.003247
0.62,83.6	0.003314
0.62,83.7	0.003377
0.62,83.8	0.003421
0.62,83.9	0.003459
0.62,84.0	0.003491
0.62,84.1	0.003519
0.62,84.2	0.003567
0.62,84.3	0.003622
0.62,84.4	0.003663
0.62,84.5	0.003694

0.62,84.6	0.003719
0.62,84.7	0.003734
0.62,84.8	0.003747
0.62,84.9	0.003753
0.62,85.0	0.003756
0.62,85.1	0.003769
0.62,85.2	0.003782
0.62,85.3	0.003811
0.62,85.4	0.003831
0.62,85.5	0.00385
0.62,85.6	0.003864
0.62,85.7	0.003871
0.62,85.8	0.003877
0.62,85.9	0.003881
0.62,86.0	0.003887
0.62,86.1	0.003908
0.62,86.2	0.003943
0.62,86.3	0.00405
0.62,86.4	0.004133
0.62,86.5	0.004183
0.62,86.6	0.004227
0.62,86.7	0.004272
0.62,86.8	0.004276
0.62,86.9	0.004296
0.62,87.0	0.004326
0.62,87.1	0.004334
0.62,87.2	0.004343
0.62,87.3	0.004351
0.62,87.4	0.004377
0.62,87.5	0.004398
0.62,87.6	0.004416
0.62,87.7	0.004438
0.62,87.8	0.004447
0.62,87.9	0.004469
0.62,88.0	0.004483
0.62,88.1	0.004505
0.62,88.2	0.004537
0.62,88.3	0.004584
0.62,88.4	0.004598
0.62,88.5	0.004613
0.62,88.6	0.004641
0.62,88.7	0.004646

0.62,88.8	0.004656
0.62,88.9	0.004685
0.62,89.0	0.00469
0.62,89.1	0.004705
0.62,89.2	0.00472
0.62,89.3	0.00474
0.62,89.4	0.004755
0.62,89.5	0.004791
0.62,89.6	0.004865
0.62,89.7	0.004897
0.62,89.8	0.004962
0.62,89.9	0.005035
0.62,90.0	0.00507
0.62,90.1	0.005122
0.62,90.2	0.005146
0.62,90.3	0.005224
0.62,90.4	0.005312
0.62,90.5	0.005324
0.62,90.6	0.005324
0.62,90.7	0.005324
0.62,90.8	0.005331
0.62,90.9	0.005337
0.62,91.0	0.00535
0.62,91.1	0.005376
0.62,91.2	0.005395
0.62,91.3	0.005455
0.62,91.4	0.005462
0.62,91.5	0.005592
0.62,91.6	0.005677
0.62,91.7	0.005841
0.62,91.8	0.006106
0.62,91.9	0.006305
0.62,92.0	0.006585
0.62,92.1	0.006724
0.62,92.2	0.006765
0.62,92.3	0.006775
0.62,92.4	0.006785
0.62,92.5	0.006806
0.62,92.6	0.006837
0.62,92.7	0.006858
0.62,92.8	0.00688
0.62,92.9	0.006966

0.62,93.0	0.006977
0.62,93.1	0.006977
0.62,93.2	0.006977
0.62,93.3	0.006988
0.62,93.4	0.006988
0.62,93.5	0.006999
0.62,93.6	0.006999
0.62,93.7	0.007021
0.62,93.8	0.007032
0.62,93.9	0.007043
0.62,94.0	0.007043
0.62,94.1	0.007065
0.62,94.2	0.007088
0.62,94.3	0.00711
0.62,94.4	0.007156
0.62,94.5	0.007226
0.62,94.6	0.007226
0.62,94.7	0.007238
0.62,94.8	0.007238
0.62,94.9	0.007321
0.62,95.0	0.007333
0.62,95.1	0.007345
0.62,95.2	0.007382
0.62,95.3	0.007443
0.62,95.4	0.007456
0.62,95.5	0.007468
0.62,95.6	0.007493
0.62,95.7	0.007506
0.62,95.8	0.007531
0.62,95.9	0.007557
0.62,96.0	0.007569
0.62,96.1	0.007635
0.62,96.2	0.007635
0.62,96.3	0.007635
0.62,96.4	0.007648
0.62,96.5	0.007648
0.62,96.6	0.007648
0.62,96.7	0.007648
0.62,96.8	0.007648
0.62,96.9	0.007648
0.62,97.0	0.007648
0.62,97.1	0.007648

0.62,97.2	0.007648
0.62,97.3	0.007648
0.62,97.4	0.007648
0.62,97.5	0.007648
0.62,97.6	0.007648
0.62,97.7	0.007648
0.62,97.8	0.007648
0.62,97.9	0.007648
0.62,98.0	0.007648
0.62,98.1	0.007648
0.62,98.2	0.007648
0.62,98.3	0.007648
0.62,98.4	0.007648
0.62,98.5	0.007648
0.62,98.6	0.007648
0.62,98.7	0.007648
0.62,98.8	0.007648
0.62,98.9	0.007648
0.62,99.0	0.007648
0.62,99.1	0.007648
0.62,99.2	0.007648
0.62,99.3	0.007648
0.62,99.4	0.007648
0.62,99.5	0.007648
0.62,99.6	0.007648
0.62,99.7	0.007648
0.62,99.8	0.007648
0.62,99.9	0.007648
0.62,100.0	0.007648
0.63,0.0	0.000238
0.63,0.1	0.000238
0.63,0.2	0.000238
0.63,0.3	0.000238
0.63,0.4	0.000238
0.63,0.5	0.000238
0.63,0.6	0.000238
0.63,0.7	0.000238
0.63,0.8	0.000238
0.63,0.9	0.000238
0.63,1.0	0.000238
0.63,1.1	0.000238
0.63,1.2	0.000238

0.63,1.3	0.000238
0.63,1.4	0.000238
0.63,1.5	0.000238
0.63,1.6	0.000238
0.63,1.7	0.000238
0.63,1.8	0.000238
0.63,1.9	0.000238
0.63,2.0	0.000238
0.63,2.1	0.000238
0.63,2.2	0.000238
0.63,2.3	0.000238
0.63,2.4	0.000238
0.63,2.5	0.000238
0.63,2.6	0.000238
0.63,2.7	0.000238
0.63,2.8	0.000238
0.63,2.9	0.000238
0.63,3.0	0.000238
0.63,3.1	0.000238
0.63,3.2	0.000238
0.63,3.3	0.000238
0.63,3.4	0.000238
0.63,3.5	0.000238
0.63,3.6	0.000238
0.63,3.7	0.000238
0.63,3.8	0.000238
0.63,3.9	0.000238
0.63,4.0	0.000238
0.63,4.1	0.000238
0.63,4.2	0.000238
0.63,4.3	0.000238
0.63,4.4	0.000238
0.63,4.5	0.000238
0.63,4.6	0.000238
0.63,4.7	0.000238
0.63,4.8	0.000238
0.63,4.9	0.000238
0.63,5.0	0.000238
0.63,5.1	0.000238
0.63,5.2	0.000238
0.63,5.3	0.000238
0.63,5.4	0.000238

0.63,5.5	0.000238
0.63,5.6	0.000238
0.63,5.7	0.000238
0.63,5.8	0.000238
0.63,5.9	0.000238
0.63,6.0	0.000238
0.63,6.1	0.000238
0.63,6.2	0.000238
0.63,6.3	0.000238
0.63,6.4	0.000238
0.63,6.5	0.000238
0.63,6.6	0.000238
0.63,6.7	0.000238
0.63,6.8	0.000238
0.63,6.9	0.000238
0.63,7.0	0.000238
0.63,7.1	0.000238
0.63,7.2	0.000238
0.63,7.3	0.000238
0.63,7.4	0.000238
0.63,7.5	0.000238
0.63,7.6	0.000238
0.63,7.7	0.000238
0.63,7.8	0.000238
0.63,7.9	0.000238
0.63,8.0	0.000238
0.63,8.1	0.000238
0.63,8.2	0.000238
0.63,8.3	0.000238
0.63,8.4	0.000238
0.63,8.5	0.000238
0.63,8.6	0.000238
0.63,8.7	0.000238
0.63,8.8	0.000238
0.63,8.9	0.000238
0.63,9.0	0.000238
0.63,9.1	0.000238
0.63,9.2	0.000238
0.63,9.3	0.000238
0.63,9.4	0.000238
0.63,9.5	0.000238
0.63,9.6	0.000238

0.63,9.7	0.000238
0.63,9.8	0.000238
0.63,9.9	0.000238
0.63,10.0	0.000238
0.63,10.1	0.000238
0.63,10.2	0.000238
0.63,10.3	0.000238
0.63,10.4	0.000238
0.63,10.5	0.000238
0.63,10.6	0.000238
0.63,10.7	0.000238
0.63,10.8	0.000238
0.63,10.9	0.000238
0.63,11.0	0.000238
0.63,11.1	0.000238
0.63,11.2	0.000238
0.63,11.3	0.000238
0.63,11.4	0.000238
0.63,11.5	0.000238
0.63,11.6	0.000238
0.63,11.7	0.000238
0.63,11.8	0.000238
0.63,11.9	0.000238
0.63,12.0	0.000238
0.63,12.1	0.000238
0.63,12.2	0.000238
0.63,12.3	0.000238
0.63,12.4	0.000238
0.63,12.5	0.000238
0.63,12.6	0.000238
0.63,12.7	0.000238
0.63,12.8	0.000238
0.63,12.9	0.000238
0.63,13.0	0.000238
0.63,13.1	0.000238
0.63,13.2	0.000238
0.63,13.3	0.000238
0.63,13.4	0.000238
0.63,13.5	0.000238
0.63,13.6	0.000238
0.63,13.7	0.000238
0.63,13.8	0.000238

0.63,13.9	0.000238
0.63,14.0	0.000238
0.63,14.1	0.000238
0.63,14.2	0.000238
0.63,14.3	0.000238
0.63,14.4	0.000238
0.63,14.5	0.000238
0.63,14.6	0.000238
0.63,14.7	0.000238
0.63,14.8	0.000238
0.63,14.9	0.000238
0.63,15.0	0.000238
0.63,15.1	0.000238
0.63,15.2	0.000238
0.63,15.3	0.000238
0.63,15.4	0.000238
0.63,15.5	0.000238
0.63,15.6	0.000238
0.63,15.7	0.000238
0.63,15.8	0.000238
0.63,15.9	0.000238
0.63,16.0	0.000238
0.63,16.1	0.000238
0.63,16.2	0.000238
0.63,16.3	0.000238
0.63,16.4	0.000238
0.63,16.5	0.000238
0.63,16.6	0.000238
0.63,16.7	0.000238
0.63,16.8	0.000238
0.63,16.9	0.000238
0.63,17.0	0.000238
0.63,17.1	0.000238
0.63,17.2	0.000238
0.63,17.3	0.000238
0.63,17.4	0.000238
0.63,17.5	0.000238
0.63,17.6	0.000238
0.63,17.7	0.000238
0.63,17.8	0.000238
0.63,17.9	0.000238
0.63,18.0	0.000238

0.63,18.1	0.000238
0.63,18.2	0.000238
0.63,18.3	0.000238
0.63,18.4	0.000238
0.63,18.5	0.000238
0.63,18.6	0.000238
0.63,18.7	0.000238
0.63,18.8	0.000238
0.63,18.9	0.000238
0.63,19.0	0.000238
0.63,19.1	0.000238
0.63,19.2	0.000238
0.63,19.3	0.000238
0.63,19.4	0.000238
0.63,19.5	0.000238
0.63,19.6	0.000238
0.63,19.7	0.000238
0.63,19.8	0.000238
0.63,19.9	0.000238
0.63,20.0	0.000238
0.63,20.1	0.000238
0.63,20.2	0.000238
0.63,20.3	0.000238
0.63,20.4	0.000238
0.63,20.5	0.000238
0.63,20.6	0.000238
0.63,20.7	0.000238
0.63,20.8	0.000238
0.63,20.9	0.000238
0.63,21.0	0.000238
0.63,21.1	0.000238
0.63,21.2	0.000238
0.63,21.3	0.000238
0.63,21.4	0.000238
0.63,21.5	0.000238
0.63,21.6	0.000238
0.63,21.7	0.000238
0.63,21.8	0.000238
0.63,21.9	0.000238
0.63,22.0	0.000238
0.63,22.1	0.000238
0.63,22.2	0.000238

0.63,22.3	0.000238
0.63,22.4	0.000238
0.63,22.5	0.000238
0.63,22.6	0.000238
0.63,22.7	0.000238
0.63,22.8	0.000238
0.63,22.9	0.000238
0.63,23.0	0.000238
0.63,23.1	0.000238
0.63,23.2	0.000238
0.63,23.3	0.000238
0.63,23.4	0.000238
0.63,23.5	0.000238
0.63,23.6	0.000238
0.63,23.7	0.000238
0.63,23.8	0.000238
0.63,23.9	0.000238
0.63,24.0	0.000238
0.63,24.1	0.000238
0.63,24.2	0.000238
0.63,24.3	0.000238
0.63,24.4	0.000238
0.63,24.5	0.000238
0.63,24.6	0.000238
0.63,24.7	0.000238
0.63,24.8	0.000238
0.63,24.9	0.000238
0.63,25.0	0.000238
0.63,25.1	0.000238
0.63,25.2	0.000238
0.63,25.3	0.000238
0.63,25.4	0.000238
0.63,25.5	0.000238
0.63,25.6	0.000238
0.63,25.7	0.000238
0.63,25.8	0.000238
0.63,25.9	0.000238
0.63,26.0	0.000238
0.63,26.1	0.000238
0.63,26.2	0.000238
0.63,26.3	0.000238
0.63,26.4	0.000238

0.63,26.5	0.000238
0.63,26.6	0.000238
0.63,26.7	0.000238
0.63,26.8	0.000238
0.63,26.9	0.000238
0.63,27.0	0.000238
0.63,27.1	0.000238
0.63,27.2	0.000238
0.63,27.3	0.000238
0.63,27.4	0.000238
0.63,27.5	0.000238
0.63,27.6	0.000238
0.63,27.7	0.000238
0.63,27.8	0.000238
0.63,27.9	0.000238
0.63,28.0	0.000238
0.63,28.1	0.000238
0.63,28.2	0.000238
0.63,28.3	0.000238
0.63,28.4	0.000238
0.63,28.5	0.000238
0.63,28.6	0.000238
0.63,28.7	0.000238
0.63,28.8	0.000238
0.63,28.9	0.000238
0.63,29.0	0.000238
0.63,29.1	0.000238
0.63,29.2	0.000238
0.63,29.3	0.000238
0.63,29.4	0.000238
0.63,29.5	0.000238
0.63,29.6	0.000238
0.63,29.7	0.000238
0.63,29.8	0.000238
0.63,29.9	0.000238
0.63,30.0	0.000238
0.63,30.1	0.000238
0.63,30.2	0.000238
0.63,30.3	0.000238
0.63,30.4	0.000238
0.63,30.5	0.000238
0.63,30.6	0.000238

0.63,30.7	0.000238
0.63,30.8	0.000238
0.63,30.9	0.000238
0.63,31.0	0.000238
0.63,31.1	0.000238
0.63,31.2	0.000238
0.63,31.3	0.000238
0.63,31.4	0.000238
0.63,31.5	0.000238
0.63,31.6	0.000238
0.63,31.7	0.000238
0.63,31.8	0.000238
0.63,31.9	0.000238
0.63,32.0	0.000238
0.63,32.1	0.000238
0.63,32.2	0.000238
0.63,32.3	0.000238
0.63,32.4	0.000238
0.63,32.5	0.000238
0.63,32.6	0.000238
0.63,32.7	0.000238
0.63,32.8	0.000238
0.63,32.9	0.000238
0.63,33.0	0.000238
0.63,33.1	0.000238
0.63,33.2	0.000238
0.63,33.3	0.000238
0.63,33.4	0.000238
0.63,33.5	0.000238
0.63,33.6	0.000238
0.63,33.7	0.000238
0.63,33.8	0.000238
0.63,33.9	0.000238
0.63,34.0	0.000238
0.63,34.1	0.000238
0.63,34.2	0.000238
0.63,34.3	0.000238
0.63,34.4	0.000238
0.63,34.5	0.000238
0.63,34.6	0.000238
0.63,34.7	0.000238
0.63,34.8	0.000238

0.63,34.9	0.000238
0.63,35.0	0.000238
0.63,35.1	0.000238
0.63,35.2	0.000238
0.63,35.3	0.000238
0.63,35.4	0.000238
0.63,35.5	0.000238
0.63,35.6	0.000238
0.63,35.7	0.000238
0.63,35.8	0.000238
0.63,35.9	0.000238
0.63,36.0	0.000238
0.63,36.1	0.000238
0.63,36.2	0.000238
0.63,36.3	0.000238
0.63,36.4	0.000238
0.63,36.5	0.000238
0.63,36.6	0.000238
0.63,36.7	0.000238
0.63,36.8	0.000238
0.63,36.9	0.000238
0.63,37.0	0.000238
0.63,37.1	0.000238
0.63,37.2	0.000238
0.63,37.3	0.000238
0.63,37.4	0.000238
0.63,37.5	0.000238
0.63,37.6	0.000238
0.63,37.7	0.000238
0.63,37.8	0.000238
0.63,37.9	0.000238
0.63,38.0	0.000238
0.63,38.1	0.000238
0.63,38.2	0.000238
0.63,38.3	0.000238
0.63,38.4	0.000238
0.63,38.5	0.000238
0.63,38.6	0.000238
0.63,38.7	0.000238
0.63,38.8	0.000238
0.63,38.9	0.000238
0.63,39.0	0.000238

0.63,39.1	0.000238
0.63,39.2	0.000238
0.63,39.3	0.000238
0.63,39.4	0.000238
0.63,39.5	0.000238
0.63,39.6	0.000238
0.63,39.7	0.000238
0.63,39.8	0.000238
0.63,39.9	0.000238
0.63,40.0	0.000238
0.63,40.1	0.000238
0.63,40.2	0.000238
0.63,40.3	0.000238
0.63,40.4	0.000238
0.63,40.5	0.000238
0.63,40.6	0.000238
0.63,40.7	0.000238
0.63,40.8	0.000238
0.63,40.9	0.000238
0.63,41.0	0.000238
0.63,41.1	0.000238
0.63,41.2	0.000238
0.63,41.3	0.000238
0.63,41.4	0.000238
0.63,41.5	0.000238
0.63,41.6	0.000238
0.63,41.7	0.000238
0.63,41.8	0.000238
0.63,41.9	0.000238
0.63,42.0	0.000238
0.63,42.1	0.000238
0.63,42.2	0.000238
0.63,42.3	0.000238
0.63,42.4	0.000238
0.63,42.5	0.000238
0.63,42.6	0.000238
0.63,42.7	0.000238
0.63,42.8	0.000238
0.63,42.9	0.000238
0.63,43.0	0.000238
0.63,43.1	0.000238
0.63,43.2	0.000238

0.63,43.3	0.000238
0.63,43.4	0.000238
0.63,43.5	0.000238
0.63,43.6	0.000238
0.63,43.7	0.000238
0.63,43.8	0.000238
0.63,43.9	0.000238
0.63,44.0	0.000238
0.63,44.1	0.000238
0.63,44.2	0.000238
0.63,44.3	0.000238
0.63,44.4	0.000238
0.63,44.5	0.000238
0.63,44.6	0.000238
0.63,44.7	0.000238
0.63,44.8	0.000238
0.63,44.9	0.000238
0.63,45.0	0.000238
0.63,45.1	0.000238
0.63,45.2	0.000238
0.63,45.3	0.000238
0.63,45.4	0.000238
0.63,45.5	0.000238
0.63,45.6	0.000238
0.63,45.7	0.000238
0.63,45.8	0.000238
0.63,45.9	0.000238
0.63,46.0	0.000238
0.63,46.1	0.000238
0.63,46.2	0.000238
0.63,46.3	0.000238
0.63,46.4	0.000238
0.63,46.5	0.000238
0.63,46.6	0.000238
0.63,46.7	0.000238
0.63,46.8	0.000238
0.63,46.9	0.000238
0.63,47.0	0.000238
0.63,47.1	0.000238
0.63,47.2	0.000238
0.63,47.3	0.000238
0.63,47.4	0.000238

0.63,47.5	0.000238
0.63,47.6	0.000238
0.63,47.7	0.000238
0.63,47.8	0.000238
0.63,47.9	0.000238
0.63,48.0	0.000238
0.63,48.1	0.000238
0.63,48.2	0.000238
0.63,48.3	0.000238
0.63,48.4	0.000238
0.63,48.5	0.000238
0.63,48.6	0.000238
0.63,48.7	0.000238
0.63,48.8	0.000238
0.63,48.9	0.000238
0.63,49.0	0.000238
0.63,49.1	0.000238
0.63,49.2	0.000238
0.63,49.3	0.000238
0.63,49.4	0.000238
0.63,49.5	0.000238
0.63,49.6	0.000238
0.63,49.7	0.000238
0.63,49.8	0.000238
0.63,49.9	0.000238
0.63,50.0	0.000238
0.63,50.1	0.000238
0.63,50.2	0.000238
0.63,50.3	0.000238
0.63,50.4	0.000238
0.63,50.5	0.000238
0.63,50.6	0.000238
0.63,50.7	0.000238
0.63,50.8	0.000238
0.63,50.9	0.000238
0.63,51.0	0.000238
0.63,51.1	0.000238
0.63,51.2	0.000238
0.63,51.3	0.000238
0.63,51.4	0.000238
0.63,51.5	0.000238
0.63,51.6	0.000238

0.63,51.7	0.000238
0.63,51.8	0.000238
0.63,51.9	0.000238
0.63,52.0	0.000238
0.63,52.1	0.000238
0.63,52.2	0.000238
0.63,52.3	0.000238
0.63,52.4	0.000238
0.63,52.5	0.000238
0.63,52.6	0.000238
0.63,52.7	0.000238
0.63,52.8	0.000238
0.63,52.9	0.000238
0.63,53.0	0.000238
0.63,53.1	0.000238
0.63,53.2	0.000238
0.63,53.3	0.000238
0.63,53.4	0.000238
0.63,53.5	0.000238
0.63,53.6	0.000238
0.63,53.7	0.000238
0.63,53.8	0.000238
0.63,53.9	0.000238
0.63,54.0	0.000238
0.63,54.1	0.000238
0.63,54.2	0.000238
0.63,54.3	0.000238
0.63,54.4	0.000238
0.63,54.5	0.000238
0.63,54.6	0.000238
0.63,54.7	0.000238
0.63,54.8	0.000238
0.63,54.9	0.000238
0.63,55.0	0.000238
0.63,55.1	0.000238
0.63,55.2	0.000238
0.63,55.3	0.000238
0.63,55.4	0.000238
0.63,55.5	0.000238
0.63,55.6	0.000238
0.63,55.7	0.000238
0.63,55.8	0.000238

0.63,55.9	0.000238
0.63,56.0	0.000238
0.63,56.1	0.000238
0.63,56.2	0.000238
0.63,56.3	0.000238
0.63,56.4	0.000238
0.63,56.5	0.000238
0.63,56.6	0.000238
0.63,56.7	0.000238
0.63,56.8	0.000238
0.63,56.9	0.000238
0.63,57.0	0.000238
0.63,57.1	0.000238
0.63,57.2	0.000238
0.63,57.3	0.000238
0.63,57.4	0.000238
0.63,57.5	0.000238
0.63,57.6	0.000238
0.63,57.7	0.000238
0.63,57.8	0.000238
0.63,57.9	0.000238
0.63,58.0	0.000238
0.63,58.1	0.000238
0.63,58.2	0.000238
0.63,58.3	0.000238
0.63,58.4	0.000238
0.63,58.5	0.000238
0.63,58.6	0.000238
0.63,58.7	0.000238
0.63,58.8	0.000238
0.63,58.9	0.000238
0.63,59.0	0.000238
0.63,59.1	0.000238
0.63,59.2	0.000238
0.63,59.3	0.000238
0.63,59.4	0.000238
0.63,59.5	0.000238
0.63,59.6	0.000238
0.63,59.7	0.000238
0.63,59.8	0.000238
0.63,59.9	0.000238
0.63,60.0	0.000238

0.63,60.1	0.000238
0.63,60.2	0.000238
0.63,60.3	0.000238
0.63,60.4	0.000238
0.63,60.5	0.000238
0.63,60.6	0.000238
0.63,60.7	0.000238
0.63,60.8	0.000238
0.63,60.9	0.000238
0.63,61.0	0.000238
0.63,61.1	0.000238
0.63,61.2	0.000238
0.63,61.3	0.000238
0.63,61.4	0.000238
0.63,61.5	0.000238
0.63,61.6	0.000238
0.63,61.7	0.000238
0.63,61.8	0.000238
0.63,61.9	0.000238
0.63,62.0	0.000238
0.63,62.1	0.000238
0.63,62.2	0.000238
0.63,62.3	0.000238
0.63,62.4	0.000238
0.63,62.5	0.000238
0.63,62.6	0.000238
0.63,62.7	0.000238
0.63,62.8	0.000238
0.63,62.9	0.000238
0.63,63.0	0.000238
0.63,63.1	0.000238
0.63,63.2	0.000238
0.63,63.3	0.000238
0.63,63.4	0.000238
0.63,63.5	0.000238
0.63,63.6	0.000238
0.63,63.7	0.000238
0.63,63.8	0.000238
0.63,63.9	0.000238
0.63,64.0	0.000238
0.63,64.1	0.000238
0.63,64.2	0.000238

0.63,64.3	0.000238
0.63,64.4	0.000238
0.63,64.5	0.000238
0.63,64.6	0.000238
0.63,64.7	0.000238
0.63,64.8	0.000238
0.63,64.9	0.000238
0.63,65.0	0.000238
0.63,65.1	0.000238
0.63,65.2	0.000238
0.63,65.3	0.000238
0.63,65.4	0.000238
0.63,65.5	0.000238
0.63,65.6	0.000238
0.63,65.7	0.000238
0.63,65.8	0.000238
0.63,65.9	0.000238
0.63,66.0	0.000238
0.63,66.1	0.000238
0.63,66.2	0.000238
0.63,66.3	0.000238
0.63,66.4	0.000238
0.63,66.5	0.000238
0.63,66.6	0.000238
0.63,66.7	0.000238
0.63,66.8	0.000238
0.63,66.9	0.000238
0.63,67.0	0.000238
0.63,67.1	0.000238
0.63,67.2	0.000238
0.63,67.3	0.000238
0.63,67.4	0.000238
0.63,67.5	0.000238
0.63,67.6	0.000238
0.63,67.7	0.000238
0.63,67.8	0.000238
0.63,67.9	0.000238
0.63,68.0	0.000238
0.63,68.1	0.000238
0.63,68.2	0.000238
0.63,68.3	0.000238
0.63,68.4	0.000238

0.63,68.5	0.000238
0.63,68.6	0.000238
0.63,68.7	0.000238
0.63,68.8	0.000238
0.63,68.9	0.000238
0.63,69.0	0.000238
0.63,69.1	0.000238
0.63,69.2	0.000238
0.63,69.3	0.000238
0.63,69.4	0.000238
0.63,69.5	0.000238
0.63,69.6	0.000238
0.63,69.7	0.000238
0.63,69.8	0.000238
0.63,69.9	0.000238
0.63,70.0	0.000238
0.63,70.1	0.000238
0.63,70.2	0.000238
0.63,70.3	0.000238
0.63,70.4	0.000238
0.63,70.5	0.000238
0.63,70.6	0.000238
0.63,70.7	0.000238
0.63,70.8	0.000238
0.63,70.9	0.000238
0.63,71.0	0.000238
0.63,71.1	0.000238
0.63,71.2	0.000238
0.63,71.3	0.000238
0.63,71.4	0.000238
0.63,71.5	0.000238
0.63,71.6	0.000238
0.63,71.7	0.000238
0.63,71.8	0.000238
0.63,71.9	0.000238
0.63,72.0	0.000238
0.63,72.1	0.000238
0.63,72.2	0.000238
0.63,72.3	0.000238
0.63,72.4	0.000238
0.63,72.5	0.000238
0.63,72.6	0.000238

0.63,72.7	0.000238
0.63,72.8	0.000238
0.63,72.9	0.000238
0.63,73.0	0.000238
0.63,73.1	0.000238
0.63,73.2	0.000238
0.63,73.3	0.000238
0.63,73.4	0.000238
0.63,73.5	0.000238
0.63,73.6	0.000238
0.63,73.7	0.000238
0.63,73.8	0.000238
0.63,73.9	0.000238
0.63,74.0	0.000238
0.63,74.1	0.000238
0.63,74.2	0.000238
0.63,74.3	0.000238
0.63,74.4	0.000238
0.63,74.5	0.000238
0.63,74.6	0.000238
0.63,74.7	0.000238
0.63,74.8	0.000238
0.63,74.9	0.000238
0.63,75.0	0.000238
0.63,75.1	0.000239
0.63,75.2	0.000239
0.63,75.3	0.000239
0.63,75.4	0.000239
0.63,75.5	0.000239
0.63,75.6	0.000239
0.63,75.7	0.000239
0.63,75.8	0.000239
0.63,75.9	0.000239
0.63,76.0	0.000239
0.63,76.1	0.000239
0.63,76.2	0.00024
0.63,76.3	0.00024
0.63,76.4	0.00024
0.63,76.5	0.000241
0.63,76.6	0.000242
0.63,76.7	0.000242
0.63,76.8	0.000242

0.63,76.9	0.000242
0.63,77.0	0.000242
0.63,77.1	0.000242
0.63,77.2	0.000242
0.63,77.3	0.000243
0.63,77.4	0.000243
0.63,77.5	0.000243
0.63,77.6	0.000243
0.63,77.7	0.000243
0.63,77.8	0.000244
0.63,77.9	0.000244
0.63,78.0	0.000244
0.63,78.1	0.000244
0.63,78.2	0.000244
0.63,78.3	0.000244
0.63,78.4	0.000244
0.63,78.5	0.000244
0.63,78.6	0.000244
0.63,78.7	0.000244
0.63,78.8	0.000244
0.63,78.9	0.000244
0.63,79.0	0.000244
0.63,79.1	0.000244
0.63,79.2	0.000245
0.63,79.3	0.000245
0.63,79.4	0.000245
0.63,79.5	0.000245
0.63,79.6	0.000246
0.63,79.7	0.000246
0.63,79.8	0.000246
0.63,79.9	0.000248
0.63,80.0	0.000248
0.63,80.1	0.000249
0.63,80.2	0.000249
0.63,80.3	0.000249
0.63,80.4	0.00025
0.63,80.5	0.00025
0.63,80.6	0.00025
0.63,80.7	0.00025
0.63,80.8	0.00025
0.63,80.9	0.000251
0.63,81.0	0.000253

0.63,81.1	0.000257
0.63,81.2	0.000285
0.63,81.3	0.000659
0.63,81.4	0.001428
0.63,81.5	0.001992
0.63,81.6	0.003278
0.63,81.7	0.003717
0.63,81.8	0.003884
0.63,81.9	0.004036
0.63,82.0	0.004199
0.63,82.1	0.004536
0.63,82.2	0.00473
0.63,82.3	0.005046
0.63,82.4	0.005413
0.63,82.5	0.005616
0.63,82.6	0.005836
0.63,82.7	0.006151
0.63,82.8	0.006456
0.63,82.9	0.00679
0.63,83.0	0.00693
0.63,83.1	0.007124
0.63,83.2	0.007174
0.63,83.3	0.007262
0.63,83.4	0.007379
0.63,83.5	0.007469
0.63,83.6	0.007561
0.63,83.7	0.007656
0.63,83.8	0.007731
0.63,83.9	0.007826
0.63,84.0	0.007882
0.63,84.1	0.007931
0.63,84.2	0.008063
0.63,84.3	0.008123
0.63,84.4	0.008175
0.63,84.5	0.008211
0.63,84.6	0.008236
0.63,84.7	0.008273
0.63,84.8	0.008294
0.63,84.9	0.00831
0.63,85.0	0.008327
0.63,85.1	0.008348
0.63,85.2	0.008365

0.63,85.3	0.008408
0.63,85.4	0.008442
0.63,85.5	0.008477
0.63,85.6	0.00849
0.63,85.7	0.008516
0.63,85.8	0.008547
0.63,85.9	0.008565
0.63,86.0	0.008587
0.63,86.1	0.008614
0.63,86.2	0.008723
0.63,86.3	0.008911
0.63,86.4	0.009108
0.63,86.5	0.009271
0.63,86.6	0.009441
0.63,86.7	0.009473
0.63,86.8	0.009506
0.63,86.9	0.00955
0.63,87.0	0.009583
0.63,87.1	0.0096
0.63,87.2	0.009628
0.63,87.3	0.009673
0.63,87.4	0.00969
0.63,87.5	0.009701
0.63,87.6	0.00973
0.63,87.7	0.009736
0.63,87.8	0.009793
0.63,87.9	0.009822
0.63,88.0	0.009875
0.63,88.1	0.009899
0.63,88.2	0.009941
0.63,88.3	0.010025
0.63,88.4	0.010062
0.63,88.5	0.010086
0.63,88.6	0.01013
0.63,88.7	0.010142
0.63,88.8	0.010154
0.63,88.9	0.010167
0.63,89.0	0.010198
0.63,89.1	0.010224
0.63,89.2	0.01023
0.63,89.3	0.010236
0.63,89.4	0.010268

0.63,89.5	0.0103
0.63,89.6	0.01043
0.63,89.7	0.01047
0.63,89.8	0.010611
0.63,89.9	0.010693
0.63,90.0	0.010735
0.63,90.1	0.010827
0.63,90.2	0.010876
0.63,90.3	0.011058
0.63,90.4	0.011224
0.63,90.5	0.01127
0.63,90.6	0.011285
0.63,90.7	0.011355
0.63,90.8	0.011378
0.63,90.9	0.011378
0.63,91.0	0.011394
0.63,91.1	0.011418
0.63,91.2	0.011473
0.63,91.3	0.011578
0.63,91.4	0.011602
0.63,91.5	0.011668
0.63,91.6	0.011843
0.63,91.7	0.012211
0.63,91.8	0.012827
0.63,91.9	0.013133
0.63,92.0	0.013509
0.63,92.1	0.013825
0.63,92.2	0.01393
0.63,92.3	0.013989
0.63,92.4	0.014013
0.63,92.5	0.014085
0.63,92.6	0.014157
0.63,92.7	0.014194
0.63,92.8	0.014292
0.63,92.9	0.014354
0.63,93.0	0.014379
0.63,93.1	0.014442
0.63,93.2	0.014492
0.63,93.3	0.014492
0.63,93.4	0.014492
0.63,93.5	0.014505
0.63,93.6	0.014505

0.63,93.7	0.014518
0.63,93.8	0.014556
0.63,93.9	0.014634
0.63,94.0	0.014634
0.63,94.1	0.014673
0.63,94.2	0.014686
0.63,94.3	0.014725
0.63,94.4	0.014844
0.63,94.5	0.014884
0.63,94.6	0.014924
0.63,94.7	0.014965
0.63,94.8	0.014978
0.63,94.9	0.015019
0.63,95.0	0.015116
0.63,95.1	0.015143
0.63,95.2	0.015157
0.63,95.3	0.015185
0.63,95.4	0.015185
0.63,95.5	0.015185
0.63,95.6	0.015199
0.63,95.7	0.015213
0.63,95.8	0.015227
0.63,95.9	0.015226
0.63,96.0	0.015226
0.63,96.1	0.015311
0.63,96.2	0.015354
0.63,96.3	0.015397
0.63,96.4	0.015411
0.63,96.5	0.015426
0.63,96.6	0.015426
0.63,96.7	0.015426
0.63,96.8	0.015426
0.63,96.9	0.015426
0.63,97.0	0.015426
0.63,97.1	0.015426
0.63,97.2	0.015426
0.63,97.3	0.015426
0.63,97.4	0.015426
0.63,97.5	0.015426
0.63,97.6	0.015426
0.63,97.7	0.015426
0.63,97.8	0.015426

0.63,97.9	0.015426
0.63,98.0	0.015426
0.63,98.1	0.015426
0.63,98.2	0.015426
0.63,98.3	0.015426
0.63,98.4	0.015426
0.63,98.5	0.015426
0.63,98.6	0.015426
0.63,98.7	0.015426
0.63,98.8	0.015426
0.63,98.9	0.015426
0.63,99.0	0.015426
0.63,99.1	0.015426
0.63,99.2	0.015426
0.63,99.3	0.015426
0.63,99.4	0.015426
0.63,99.5	0.015426
0.63,99.6	0.015426
0.63,99.7	0.015426
0.63,99.8	0.015426
0.63,99.9	0.015426
0.63,100.0	0.015426
0.64,0.0	0.000861
0.64,0.1	0.000861
0.64,0.2	0.000861
0.64,0.3	0.000861
0.64,0.4	0.000861
0.64,0.5	0.000861
0.64,0.6	0.000861
0.64,0.7	0.000861
0.64,0.8	0.000861
0.64,0.9	0.000861
0.64,1.0	0.000861
0.64,1.1	0.000861
0.64,1.2	0.000861
0.64,1.3	0.000861
0.64,1.4	0.000861
0.64,1.5	0.000861
0.64,1.6	0.000861
0.64,1.7	0.000861
0.64,1.8	0.000861
0.64,1.9	0.000861

0.64,2.0	0.000861
0.64,2.1	0.000861
0.64,2.2	0.000861
0.64,2.3	0.000861
0.64,2.4	0.000861
0.64,2.5	0.000861
0.64,2.6	0.000861
0.64,2.7	0.000861
0.64,2.8	0.000861
0.64,2.9	0.000861
0.64,3.0	0.000861
0.64,3.1	0.000861
0.64,3.2	0.000861
0.64,3.3	0.000861
0.64,3.4	0.000861
0.64,3.5	0.000861
0.64,3.6	0.000861
0.64,3.7	0.000861
0.64,3.8	0.000861
0.64,3.9	0.000861
0.64,4.0	0.000861
0.64,4.1	0.000861
0.64,4.2	0.000861
0.64,4.3	0.000861
0.64,4.4	0.000861
0.64,4.5	0.000861
0.64,4.6	0.000861
0.64,4.7	0.000861
0.64,4.8	0.000861
0.64,4.9	0.000861
0.64,5.0	0.000861
0.64,5.1	0.000861
0.64,5.2	0.000861
0.64,5.3	0.000861
0.64,5.4	0.000861
0.64,5.5	0.000861
0.64,5.6	0.000861
0.64,5.7	0.000861
0.64,5.8	0.000861
0.64,5.9	0.000861
0.64,6.0	0.000861
0.64,6.1	0.000861

0.64,6.2	0.000861
0.64,6.3	0.000861
0.64,6.4	0.000861
0.64,6.5	0.000861
0.64,6.6	0.000861
0.64,6.7	0.000861
0.64,6.8	0.000861
0.64,6.9	0.000861
0.64,7.0	0.000861
0.64,7.1	0.000861
0.64,7.2	0.000861
0.64,7.3	0.000861
0.64,7.4	0.000861
0.64,7.5	0.000861
0.64,7.6	0.000861
0.64,7.7	0.000861
0.64,7.8	0.000861
0.64,7.9	0.000861
0.64,8.0	0.000861
0.64,8.1	0.000861
0.64,8.2	0.000861
0.64,8.3	0.000861
0.64,8.4	0.000861
0.64,8.5	0.000861
0.64,8.6	0.000861
0.64,8.7	0.000861
0.64,8.8	0.000861
0.64,8.9	0.000861
0.64,9.0	0.000861
0.64,9.1	0.000861
0.64,9.2	0.000861
0.64,9.3	0.000861
0.64,9.4	0.000861
0.64,9.5	0.000861
0.64,9.6	0.000861
0.64,9.7	0.000861
0.64,9.8	0.000861
0.64,9.9	0.000861
0.64,10.0	0.000861
0.64,10.1	0.000861
0.64,10.2	0.000861
0.64,10.3	0.000861

0.64,10.4	0.000861
0.64,10.5	0.000861
0.64,10.6	0.000861
0.64,10.7	0.000861
0.64,10.8	0.000861
0.64,10.9	0.000861
0.64,11.0	0.000861
0.64,11.1	0.000861
0.64,11.2	0.000861
0.64,11.3	0.000861
0.64,11.4	0.000861
0.64,11.5	0.000861
0.64,11.6	0.000861
0.64,11.7	0.000861
0.64,11.8	0.000861
0.64,11.9	0.000861
0.64,12.0	0.000861
0.64,12.1	0.000861
0.64,12.2	0.000861
0.64,12.3	0.000861
0.64,12.4	0.000861
0.64,12.5	0.000861
0.64,12.6	0.000861
0.64,12.7	0.000861
0.64,12.8	0.000861
0.64,12.9	0.000861
0.64,13.0	0.000861
0.64,13.1	0.000861
0.64,13.2	0.000861
0.64,13.3	0.000861
0.64,13.4	0.000861
0.64,13.5	0.000861
0.64,13.6	0.000861
0.64,13.7	0.000861
0.64,13.8	0.000861
0.64,13.9	0.000861
0.64,14.0	0.000861
0.64,14.1	0.000861
0.64,14.2	0.000861
0.64,14.3	0.000861
0.64,14.4	0.000861
0.64,14.5	0.000861

0.64,14.6	0.000861
0.64,14.7	0.000861
0.64,14.8	0.000861
0.64,14.9	0.000861
0.64,15.0	0.000861
0.64,15.1	0.000861
0.64,15.2	0.000861
0.64,15.3	0.000861
0.64,15.4	0.000861
0.64,15.5	0.000861
0.64,15.6	0.000861
0.64,15.7	0.000861
0.64,15.8	0.000861
0.64,15.9	0.000861
0.64,16.0	0.000861
0.64,16.1	0.000861
0.64,16.2	0.000861
0.64,16.3	0.000861
0.64,16.4	0.000861
0.64,16.5	0.000861
0.64,16.6	0.000861
0.64,16.7	0.000861
0.64,16.8	0.000861
0.64,16.9	0.000861
0.64,17.0	0.000861
0.64,17.1	0.000861
0.64,17.2	0.000861
0.64,17.3	0.000861
0.64,17.4	0.000861
0.64,17.5	0.000861
0.64,17.6	0.000861
0.64,17.7	0.000861
0.64,17.8	0.000861
0.64,17.9	0.000861
0.64,18.0	0.000861
0.64,18.1	0.000861
0.64,18.2	0.000861
0.64,18.3	0.000861
0.64,18.4	0.000861
0.64,18.5	0.000861
0.64,18.6	0.000861
0.64,18.7	0.000861

0.64,18.8	0.000861
0.64,18.9	0.000861
0.64,19.0	0.000861
0.64,19.1	0.000861
0.64,19.2	0.000861
0.64,19.3	0.000861
0.64,19.4	0.000861
0.64,19.5	0.000861
0.64,19.6	0.000861
0.64,19.7	0.000861
0.64,19.8	0.000861
0.64,19.9	0.000861
0.64,20.0	0.000861
0.64,20.1	0.000861
0.64,20.2	0.000861
0.64,20.3	0.000861
0.64,20.4	0.000861
0.64,20.5	0.000861
0.64,20.6	0.000861
0.64,20.7	0.000861
0.64,20.8	0.000861
0.64,20.9	0.000861
0.64,21.0	0.000861
0.64,21.1	0.000861
0.64,21.2	0.000861
0.64,21.3	0.000861
0.64,21.4	0.000861
0.64,21.5	0.000861
0.64,21.6	0.000861
0.64,21.7	0.000861
0.64,21.8	0.000861
0.64,21.9	0.000861
0.64,22.0	0.000861
0.64,22.1	0.000861
0.64,22.2	0.000861
0.64,22.3	0.000861
0.64,22.4	0.000861
0.64,22.5	0.000861
0.64,22.6	0.000861
0.64,22.7	0.000861
0.64,22.8	0.000861
0.64,22.9	0.000861

0.64,23.0	0.000861
0.64,23.1	0.000861
0.64,23.2	0.000861
0.64,23.3	0.000861
0.64,23.4	0.000861
0.64,23.5	0.000861
0.64,23.6	0.000861
0.64,23.7	0.000861
0.64,23.8	0.000861
0.64,23.9	0.000861
0.64,24.0	0.000861
0.64,24.1	0.000861
0.64,24.2	0.000861
0.64,24.3	0.000861
0.64,24.4	0.000861
0.64,24.5	0.000861
0.64,24.6	0.000861
0.64,24.7	0.000861
0.64,24.8	0.000861
0.64,24.9	0.000861
0.64,25.0	0.000861
0.64,25.1	0.000861
0.64,25.2	0.000861
0.64,25.3	0.000861
0.64,25.4	0.000861
0.64,25.5	0.000861
0.64,25.6	0.000861
0.64,25.7	0.000861
0.64,25.8	0.000861
0.64,25.9	0.000861
0.64,26.0	0.000861
0.64,26.1	0.000861
0.64,26.2	0.000861
0.64,26.3	0.000861
0.64,26.4	0.000861
0.64,26.5	0.000861
0.64,26.6	0.000861
0.64,26.7	0.000861
0.64,26.8	0.000861
0.64,26.9	0.000861
0.64,27.0	0.000861
0.64,27.1	0.000861

0.64,27.2	0.000861
0.64,27.3	0.000861
0.64,27.4	0.000861
0.64,27.5	0.000861
0.64,27.6	0.000861
0.64,27.7	0.000861
0.64,27.8	0.000861
0.64,27.9	0.000861
0.64,28.0	0.000861
0.64,28.1	0.000861
0.64,28.2	0.000861
0.64,28.3	0.000861
0.64,28.4	0.000861
0.64,28.5	0.000861
0.64,28.6	0.000861
0.64,28.7	0.000861
0.64,28.8	0.000861
0.64,28.9	0.000861
0.64,29.0	0.000861
0.64,29.1	0.000861
0.64,29.2	0.000861
0.64,29.3	0.000861
0.64,29.4	0.000861
0.64,29.5	0.000861
0.64,29.6	0.000861
0.64,29.7	0.000861
0.64,29.8	0.000861
0.64,29.9	0.000861
0.64,30.0	0.000861
0.64,30.1	0.000861
0.64,30.2	0.000861
0.64,30.3	0.000861
0.64,30.4	0.000861
0.64,30.5	0.000861
0.64,30.6	0.000861
0.64,30.7	0.000861
0.64,30.8	0.000861
0.64,30.9	0.000861
0.64,31.0	0.000861
0.64,31.1	0.000861
0.64,31.2	0.000861
0.64,31.3	0.000861

0.64,31.4	0.000861
0.64,31.5	0.000861
0.64,31.6	0.000861
0.64,31.7	0.000861
0.64,31.8	0.000861
0.64,31.9	0.000861
0.64,32.0	0.000861
0.64,32.1	0.000861
0.64,32.2	0.000861
0.64,32.3	0.000861
0.64,32.4	0.000861
0.64,32.5	0.000861
0.64,32.6	0.000861
0.64,32.7	0.000861
0.64,32.8	0.000861
0.64,32.9	0.000861
0.64,33.0	0.000861
0.64,33.1	0.000861
0.64,33.2	0.000861
0.64,33.3	0.000861
0.64,33.4	0.000861
0.64,33.5	0.000861
0.64,33.6	0.000861
0.64,33.7	0.000861
0.64,33.8	0.000861
0.64,33.9	0.000861
0.64,34.0	0.000861
0.64,34.1	0.000861
0.64,34.2	0.000861
0.64,34.3	0.000861
0.64,34.4	0.000861
0.64,34.5	0.000861
0.64,34.6	0.000861
0.64,34.7	0.000861
0.64,34.8	0.000861
0.64,34.9	0.000861
0.64,35.0	0.000861
0.64,35.1	0.000861
0.64,35.2	0.000861
0.64,35.3	0.000861
0.64,35.4	0.000861
0.64,35.5	0.000861

0.64,35.6	0.000861
0.64,35.7	0.000861
0.64,35.8	0.000861
0.64,35.9	0.000861
0.64,36.0	0.000861
0.64,36.1	0.000861
0.64,36.2	0.000861
0.64,36.3	0.000861
0.64,36.4	0.000861
0.64,36.5	0.000861
0.64,36.6	0.000861
0.64,36.7	0.000861
0.64,36.8	0.000861
0.64,36.9	0.000861
0.64,37.0	0.000861
0.64,37.1	0.000861
0.64,37.2	0.000861
0.64,37.3	0.000861
0.64,37.4	0.000861
0.64,37.5	0.000861
0.64,37.6	0.000861
0.64,37.7	0.000861
0.64,37.8	0.000861
0.64,37.9	0.000861
0.64,38.0	0.000861
0.64,38.1	0.000861
0.64,38.2	0.000861
0.64,38.3	0.000861
0.64,38.4	0.000861
0.64,38.5	0.000861
0.64,38.6	0.000861
0.64,38.7	0.000861
0.64,38.8	0.000861
0.64,38.9	0.000861
0.64,39.0	0.000861
0.64,39.1	0.000861
0.64,39.2	0.000861
0.64,39.3	0.000861
0.64,39.4	0.000861
0.64,39.5	0.000861
0.64,39.6	0.000861
0.64,39.7	0.000861

0.64,39.8	0.000861
0.64,39.9	0.000861
0.64,40.0	0.000861
0.64,40.1	0.000861
0.64,40.2	0.000861
0.64,40.3	0.000861
0.64,40.4	0.000861
0.64,40.5	0.000861
0.64,40.6	0.000861
0.64,40.7	0.000861
0.64,40.8	0.000861
0.64,40.9	0.000861
0.64,41.0	0.000861
0.64,41.1	0.000861
0.64,41.2	0.000861
0.64,41.3	0.000861
0.64,41.4	0.000861
0.64,41.5	0.000861
0.64,41.6	0.000861
0.64,41.7	0.000861
0.64,41.8	0.000861
0.64,41.9	0.000861
0.64,42.0	0.000861
0.64,42.1	0.000861
0.64,42.2	0.000861
0.64,42.3	0.000861
0.64,42.4	0.000861
0.64,42.5	0.000861
0.64,42.6	0.000861
0.64,42.7	0.000861
0.64,42.8	0.000861
0.64,42.9	0.000861
0.64,43.0	0.000861
0.64,43.1	0.000861
0.64,43.2	0.000861
0.64,43.3	0.000861
0.64,43.4	0.000861
0.64,43.5	0.000861
0.64,43.6	0.000861
0.64,43.7	0.000861
0.64,43.8	0.000861
0.64,43.9	0.000861

0.64,44.0	0.000861
0.64,44.1	0.000861
0.64,44.2	0.000861
0.64,44.3	0.000861
0.64,44.4	0.000861
0.64,44.5	0.000861
0.64,44.6	0.000861
0.64,44.7	0.000861
0.64,44.8	0.000861
0.64,44.9	0.000861
0.64,45.0	0.000861
0.64,45.1	0.000861
0.64,45.2	0.000861
0.64,45.3	0.000861
0.64,45.4	0.000861
0.64,45.5	0.000861
0.64,45.6	0.000861
0.64,45.7	0.000861
0.64,45.8	0.000861
0.64,45.9	0.000861
0.64,46.0	0.000861
0.64,46.1	0.000861
0.64,46.2	0.000861
0.64,46.3	0.000861
0.64,46.4	0.000861
0.64,46.5	0.000861
0.64,46.6	0.000861
0.64,46.7	0.000861
0.64,46.8	0.000861
0.64,46.9	0.000861
0.64,47.0	0.000861
0.64,47.1	0.000861
0.64,47.2	0.000861
0.64,47.3	0.000861
0.64,47.4	0.000861
0.64,47.5	0.000861
0.64,47.6	0.000861
0.64,47.7	0.000861
0.64,47.8	0.000861
0.64,47.9	0.000861
0.64,48.0	0.000861
0.64,48.1	0.000861

0.64,48.2	0.000861
0.64,48.3	0.000861
0.64,48.4	0.000861
0.64,48.5	0.000861
0.64,48.6	0.000861
0.64,48.7	0.000861
0.64,48.8	0.000861
0.64,48.9	0.000861
0.64,49.0	0.000861
0.64,49.1	0.000861
0.64,49.2	0.000861
0.64,49.3	0.000861
0.64,49.4	0.000861
0.64,49.5	0.000861
0.64,49.6	0.000861
0.64,49.7	0.000861
0.64,49.8	0.000861
0.64,49.9	0.000861
0.64,50.0	0.000861
0.64,50.1	0.000861
0.64,50.2	0.000861
0.64,50.3	0.000861
0.64,50.4	0.000861
0.64,50.5	0.000861
0.64,50.6	0.000861
0.64,50.7	0.000861
0.64,50.8	0.000861
0.64,50.9	0.000861
0.64,51.0	0.000861
0.64,51.1	0.000861
0.64,51.2	0.000861
0.64,51.3	0.000861
0.64,51.4	0.000861
0.64,51.5	0.000861
0.64,51.6	0.000861
0.64,51.7	0.000861
0.64,51.8	0.000861
0.64,51.9	0.000861
0.64,52.0	0.000861
0.64,52.1	0.000861
0.64,52.2	0.000861
0.64,52.3	0.000861

0.64,52.4	0.000861
0.64,52.5	0.000861
0.64,52.6	0.000861
0.64,52.7	0.000861
0.64,52.8	0.000861
0.64,52.9	0.000861
0.64,53.0	0.000861
0.64,53.1	0.000861
0.64,53.2	0.000861
0.64,53.3	0.000861
0.64,53.4	0.000861
0.64,53.5	0.000861
0.64,53.6	0.000861
0.64,53.7	0.000861
0.64,53.8	0.000861
0.64,53.9	0.000861
0.64,54.0	0.000861
0.64,54.1	0.000861
0.64,54.2	0.000861
0.64,54.3	0.000861
0.64,54.4	0.000861
0.64,54.5	0.000861
0.64,54.6	0.000861
0.64,54.7	0.000861
0.64,54.8	0.000861
0.64,54.9	0.000861
0.64,55.0	0.000861
0.64,55.1	0.000861
0.64,55.2	0.000861
0.64,55.3	0.000861
0.64,55.4	0.000861
0.64,55.5	0.000861
0.64,55.6	0.000861
0.64,55.7	0.000861
0.64,55.8	0.000861
0.64,55.9	0.000861
0.64,56.0	0.000861
0.64,56.1	0.000861
0.64,56.2	0.000861
0.64,56.3	0.000861
0.64,56.4	0.000861
0.64,56.5	0.000861

0.64,56.6	0.000861
0.64,56.7	0.000861
0.64,56.8	0.000861
0.64,56.9	0.000861
0.64,57.0	0.000861
0.64,57.1	0.000861
0.64,57.2	0.000861
0.64,57.3	0.000861
0.64,57.4	0.000861
0.64,57.5	0.000861
0.64,57.6	0.000861
0.64,57.7	0.000861
0.64,57.8	0.000861
0.64,57.9	0.000861
0.64,58.0	0.000861
0.64,58.1	0.000861
0.64,58.2	0.000861
0.64,58.3	0.000861
0.64,58.4	0.000861
0.64,58.5	0.000861
0.64,58.6	0.000861
0.64,58.7	0.000861
0.64,58.8	0.000861
0.64,58.9	0.000861
0.64,59.0	0.000861
0.64,59.1	0.000861
0.64,59.2	0.000861
0.64,59.3	0.000861
0.64,59.4	0.000861
0.64,59.5	0.000861
0.64,59.6	0.000861
0.64,59.7	0.000861
0.64,59.8	0.000861
0.64,59.9	0.000861
0.64,60.0	0.000861
0.64,60.1	0.000861
0.64,60.2	0.000861
0.64,60.3	0.000861
0.64,60.4	0.000861
0.64,60.5	0.000861
0.64,60.6	0.000861
0.64,60.7	0.000861

0.64,60.8	0.000861
0.64,60.9	0.000861
0.64,61.0	0.000861
0.64,61.1	0.000861
0.64,61.2	0.000861
0.64,61.3	0.000861
0.64,61.4	0.000861
0.64,61.5	0.000861
0.64,61.6	0.000861
0.64,61.7	0.000861
0.64,61.8	0.000861
0.64,61.9	0.000861
0.64,62.0	0.000861
0.64,62.1	0.000861
0.64,62.2	0.000861
0.64,62.3	0.000861
0.64,62.4	0.000861
0.64,62.5	0.000861
0.64,62.6	0.000861
0.64,62.7	0.000861
0.64,62.8	0.000861
0.64,62.9	0.000861
0.64,63.0	0.000861
0.64,63.1	0.000861
0.64,63.2	0.000861
0.64,63.3	0.000861
0.64,63.4	0.000861
0.64,63.5	0.000861
0.64,63.6	0.000861
0.64,63.7	0.000861
0.64,63.8	0.000861
0.64,63.9	0.000861
0.64,64.0	0.000861
0.64,64.1	0.000861
0.64,64.2	0.000861
0.64,64.3	0.000861
0.64,64.4	0.000861
0.64,64.5	0.000861
0.64,64.6	0.000861
0.64,64.7	0.000861
0.64,64.8	0.000861
0.64,64.9	0.000861

0.64,65.0	0.000861
0.64,65.1	0.000861
0.64,65.2	0.000861
0.64,65.3	0.000861
0.64,65.4	0.000861
0.64,65.5	0.000861
0.64,65.6	0.000861
0.64,65.7	0.000861
0.64,65.8	0.000861
0.64,65.9	0.000861
0.64,66.0	0.000861
0.64,66.1	0.000861
0.64,66.2	0.000861
0.64,66.3	0.000861
0.64,66.4	0.000861
0.64,66.5	0.000861
0.64,66.6	0.000861
0.64,66.7	0.000861
0.64,66.8	0.000861
0.64,66.9	0.000861
0.64,67.0	0.000861
0.64,67.1	0.000861
0.64,67.2	0.000861
0.64,67.3	0.000861
0.64,67.4	0.000861
0.64,67.5	0.000861
0.64,67.6	0.000861
0.64,67.7	0.000861
0.64,67.8	0.000861
0.64,67.9	0.000861
0.64,68.0	0.000861
0.64,68.1	0.000861
0.64,68.2	0.000861
0.64,68.3	0.000861
0.64,68.4	0.000861
0.64,68.5	0.000861
0.64,68.6	0.000861
0.64,68.7	0.000861
0.64,68.8	0.000861
0.64,68.9	0.000861
0.64,69.0	0.000861
0.64,69.1	0.000861

0.64,69.2	0.000861
0.64,69.3	0.000861
0.64,69.4	0.000861
0.64,69.5	0.000861
0.64,69.6	0.000861
0.64,69.7	0.000861
0.64,69.8	0.000861
0.64,69.9	0.000861
0.64,70.0	0.000861
0.64,70.1	0.000861
0.64,70.2	0.000861
0.64,70.3	0.000861
0.64,70.4	0.000861
0.64,70.5	0.000861
0.64,70.6	0.000861
0.64,70.7	0.000861
0.64,70.8	0.000861
0.64,70.9	0.000861
0.64,71.0	0.000861
0.64,71.1	0.000861
0.64,71.2	0.000861
0.64,71.3	0.000861
0.64,71.4	0.000861
0.64,71.5	0.000861
0.64,71.6	0.000861
0.64,71.7	0.000861
0.64,71.8	0.000861
0.64,71.9	0.000861
0.64,72.0	0.000861
0.64,72.1	0.000861
0.64,72.2	0.000861
0.64,72.3	0.000861
0.64,72.4	0.000861
0.64,72.5	0.000861
0.64,72.6	0.000861
0.64,72.7	0.000861
0.64,72.8	0.000861
0.64,72.9	0.000861
0.64,73.0	0.000861
0.64,73.1	0.000861
0.64,73.2	0.000861
0.64,73.3	0.000861

0.64,73.4	0.000861
0.64,73.5	0.000861
0.64,73.6	0.000861
0.64,73.7	0.000861
0.64,73.8	0.000861
0.64,73.9	0.000861
0.64,74.0	0.000861
0.64,74.1	0.000861
0.64,74.2	0.000861
0.64,74.3	0.000861
0.64,74.4	0.000861
0.64,74.5	0.000861
0.64,74.6	0.000861
0.64,74.7	0.000861
0.64,74.8	0.000861
0.64,74.9	0.000861
0.64,75.0	0.000861
0.64,75.1	0.000862
0.64,75.2	0.000862
0.64,75.3	0.000862
0.64,75.4	0.000862
0.64,75.5	0.000862
0.64,75.6	0.000862
0.64,75.7	0.000862
0.64,75.8	0.000862
0.64,75.9	0.000862
0.64,76.0	0.000862
0.64,76.1	0.000862
0.64,76.2	0.000863
0.64,76.3	0.000863
0.64,76.4	0.000864
0.64,76.5	0.000865
0.64,76.6	0.000866
0.64,76.7	0.000867
0.64,76.8	0.000867
0.64,76.9	0.000868
0.64,77.0	0.000868
0.64,77.1	0.000869
0.64,77.2	0.000869
0.64,77.3	0.000869
0.64,77.4	0.00087
0.64,77.5	0.000871

0.64,77.6	0.000872
0.64,77.7	0.000872
0.64,77.8	0.000873
0.64,77.9	0.000873
0.64,78.0	0.000873
0.64,78.1	0.000873
0.64,78.2	0.000873
0.64,78.3	0.000873
0.64,78.4	0.000873
0.64,78.5	0.000873
0.64,78.6	0.000873
0.64,78.7	0.000873
0.64,78.8	0.000874
0.64,78.9	0.000874
0.64,79.0	0.000874
0.64,79.1	0.000875
0.64,79.2	0.000877
0.64,79.3	0.000878
0.64,79.4	0.000878
0.64,79.5	0.000878
0.64,79.6	0.000879
0.64,79.7	0.000879
0.64,79.8	0.00088
0.64,79.9	0.00088
0.64,80.0	0.000881
0.64,80.1	0.000881
0.64,80.2	0.000882
0.64,80.3	0.000882
0.64,80.4	0.000884
0.64,80.5	0.000884
0.64,80.6	0.000884
0.64,80.7	0.000884
0.64,80.8	0.000886
0.64,80.9	0.000889
0.64,81.0	0.000893
0.64,81.1	0.000904
0.64,81.2	0.00097
0.64,81.3	0.001902
0.64,81.4	0.004307
0.64,81.5	0.005519
0.64,81.6	0.008107
0.64,81.7	0.009234

0.64,81.8	0.009582
0.64,81.9	0.009817
0.64,82.0	0.009949
0.64,82.1	0.010168
0.64,82.2	0.010417
0.64,82.3	0.01108
0.64,82.4	0.011653
0.64,82.5	0.012129
0.64,82.6	0.012752
0.64,82.7	0.013044
0.64,82.8	0.013654
0.64,82.9	0.014141
0.64,83.0	0.014275
0.64,83.1	0.01446
0.64,83.2	0.014555
0.64,83.3	0.014693
0.64,83.4	0.014817
0.64,83.5	0.014912
0.64,83.6	0.015061
0.64,83.7	0.015222
0.64,83.8	0.015373
0.64,83.9	0.015447
0.64,84.0	0.015485
0.64,84.1	0.015551
0.64,84.2	0.015689
0.64,84.3	0.01585
0.64,84.4	0.015943
0.64,84.5	0.015978
0.64,84.6	0.016018
0.64,84.7	0.016079
0.64,84.8	0.01614
0.64,84.9	0.01617
0.64,85.0	0.016216
0.64,85.1	0.016247
0.64,85.2	0.016294
0.64,85.3	0.016346
0.64,85.4	0.016398
0.64,85.5	0.016419
0.64,85.6	0.016488
0.64,85.7	0.01652
0.64,85.8	0.016542
0.64,85.9	0.016622

0.64,86.0	0.016791
0.64,86.1	0.016891
0.64,86.2	0.017077
0.64,86.3	0.017673
0.64,86.4	0.018035
0.64,86.5	0.018592
0.64,86.6	0.018929
0.64,86.7	0.019028
0.64,86.8	0.019056
0.64,86.9	0.019077
0.64,87.0	0.019106
0.64,87.1	0.019142
0.64,87.2	0.01917
0.64,87.3	0.019221
0.64,87.4	0.019257
0.64,87.5	0.019286
0.64,87.6	0.019352
0.64,87.7	0.019381
0.64,87.8	0.01947
0.64,87.9	0.019566
0.64,88.0	0.019649
0.64,88.1	0.019702
0.64,88.2	0.019801
0.64,88.3	0.019979
0.64,88.4	0.020018
0.64,88.5	0.020065
0.64,88.6	0.020121
0.64,88.7	0.020152
0.64,88.8	0.0202
0.64,88.9	0.020216
0.64,89.0	0.020232
0.64,89.1	0.02028
0.64,89.2	0.020296
0.64,89.3	0.020337
0.64,89.4	0.020402
0.64,89.5	0.020475
0.64,89.6	0.020758
0.64,89.7	0.020936
0.64,89.8	0.021179
0.64,89.9	0.021338
0.64,90.0	0.021391
0.64,90.1	0.021481

0.64,90.2	0.021544
0.64,90.3	0.021961
0.64,90.4	0.022631
0.64,90.5	0.022895
0.64,90.6	0.023029
0.64,90.7	0.023049
0.64,90.8	0.02306
0.64,90.9	0.023101
0.64,91.0	0.023238
0.64,91.1	0.023355
0.64,91.2	0.02343
0.64,91.3	0.023581
0.64,91.4	0.023592
0.64,91.5	0.023646
0.64,91.6	0.023956
0.64,91.7	0.024519
0.64,91.8	0.025183
0.64,91.9	0.025663
0.64,92.0	0.026202
0.64,92.1	0.026708
0.64,92.2	0.026891
0.64,92.3	0.027019
0.64,92.4	0.027047
0.64,92.5	0.027119
0.64,92.6	0.027307
0.64,92.7	0.027395
0.64,92.8	0.027439
0.64,92.9	0.027572
0.64,93.0	0.027572
0.64,93.1	0.027572
0.64,93.2	0.027602
0.64,93.3	0.027632
0.64,93.4	0.027647
0.64,93.5	0.027677
0.64,93.6	0.027677
0.64,93.7	0.027797
0.64,93.8	0.027812
0.64,93.9	0.027903
0.64,94.0	0.027964
0.64,94.1	0.02801
0.64,94.2	0.028118
0.64,94.3	0.028289

0.64,94.4	0.028446
0.64,94.5	0.028494
0.64,94.6	0.028558
0.64,94.7	0.028589
0.64,94.8	0.028621
0.64,94.9	0.028686
0.64,95.0	0.028702
0.64,95.1	0.028734
0.64,95.2	0.028831
0.64,95.3	0.028896
0.64,95.4	0.028913
0.64,95.5	0.028962
0.64,95.6	0.028978
0.64,95.7	0.029011
0.64,95.8	0.029011
0.64,95.9	0.029011
0.64,96.0	0.029043
0.64,96.1	0.029176
0.64,96.2	0.02926
0.64,96.3	0.029276
0.64,96.4	0.02931
0.64,96.5	0.029344
0.64,96.6	0.029344
0.64,96.7	0.029344
0.64,96.8	0.029344
0.64,96.9	0.029344
0.64,97.0	0.029344
0.64,97.1	0.029344
0.64,97.2	0.029344
0.64,97.3	0.029344
0.64,97.4	0.029344
0.64,97.5	0.029344
0.64,97.6	0.029344
0.64,97.7	0.029344
0.64,97.8	0.029344
0.64,97.9	0.029344
0.64,98.0	0.029344
0.64,98.1	0.029344
0.64,98.2	0.029344
0.64,98.3	0.029344
0.64,98.4	0.029344
0.64,98.5	0.029344

0.64,98.6	0.029344
0.64,98.7	0.029344
0.64,98.8	0.029344
0.64,98.9	0.029344
0.64,99.0	0.029344
0.64,99.1	0.029344
0.64,99.2	0.029344
0.64,99.3	0.029344
0.64,99.4	0.029344
0.64,99.5	0.029344
0.64,99.6	0.029344
0.64,99.7	0.029344
0.64,99.8	0.029344
0.64,99.9	0.029344
0.64,100.0	0.029344
0.65,0.0	0.003553
0.65,0.1	0.003553
0.65,0.2	0.003553
0.65,0.3	0.003553
0.65,0.4	0.003553
0.65,0.5	0.003553
0.65,0.6	0.003553
0.65,0.7	0.003553
0.65,0.8	0.003553
0.65,0.9	0.003553
0.65,1.0	0.003553
0.65,1.1	0.003553
0.65,1.2	0.003553
0.65,1.3	0.003553
0.65,1.4	0.003553
0.65,1.5	0.003553
0.65,1.6	0.003553
0.65,1.7	0.003553
0.65,1.8	0.003553
0.65,1.9	0.003553
0.65,2.0	0.003553
0.65,2.1	0.003553
0.65,2.2	0.003553
0.65,2.3	0.003553
0.65,2.4	0.003553
0.65,2.5	0.003553
0.65,2.6	0.003553

0.65,2.7	0.003553
0.65,2.8	0.003553
0.65,2.9	0.003553
0.65,3.0	0.003553
0.65,3.1	0.003553
0.65,3.2	0.003553
0.65,3.3	0.003553
0.65,3.4	0.003553
0.65,3.5	0.003553
0.65,3.6	0.003553
0.65,3.7	0.003553
0.65,3.8	0.003553
0.65,3.9	0.003553
0.65,4.0	0.003553
0.65,4.1	0.003553
0.65,4.2	0.003553
0.65,4.3	0.003553
0.65,4.4	0.003553
0.65,4.5	0.003553
0.65,4.6	0.003553
0.65,4.7	0.003553
0.65,4.8	0.003553
0.65,4.9	0.003553
0.65,5.0	0.003553
0.65,5.1	0.003553
0.65,5.2	0.003553
0.65,5.3	0.003553
0.65,5.4	0.003553
0.65,5.5	0.003553
0.65,5.6	0.003553
0.65,5.7	0.003553
0.65,5.8	0.003553
0.65,5.9	0.003553
0.65,6.0	0.003553
0.65,6.1	0.003553
0.65,6.2	0.003553
0.65,6.3	0.003553
0.65,6.4	0.003553
0.65,6.5	0.003553
0.65,6.6	0.003553
0.65,6.7	0.003553
0.65,6.8	0.003553

0.65,6.9	0.003553
0.65,7.0	0.003553
0.65,7.1	0.003553
0.65,7.2	0.003553
0.65,7.3	0.003553
0.65,7.4	0.003553
0.65,7.5	0.003553
0.65,7.6	0.003553
0.65,7.7	0.003553
0.65,7.8	0.003553
0.65,7.9	0.003553
0.65,8.0	0.003553
0.65,8.1	0.003553
0.65,8.2	0.003553
0.65,8.3	0.003553
0.65,8.4	0.003553
0.65,8.5	0.003553
0.65,8.6	0.003553
0.65,8.7	0.003553
0.65,8.8	0.003553
0.65,8.9	0.003553
0.65,9.0	0.003553
0.65,9.1	0.003553
0.65,9.2	0.003553
0.65,9.3	0.003553
0.65,9.4	0.003553
0.65,9.5	0.003553
0.65,9.6	0.003553
0.65,9.7	0.003553
0.65,9.8	0.003553
0.65,9.9	0.003553
0.65,10.0	0.003553
0.65,10.1	0.003553
0.65,10.2	0.003553
0.65,10.3	0.003553
0.65,10.4	0.003553
0.65,10.5	0.003553
0.65,10.6	0.003553
0.65,10.7	0.003553
0.65,10.8	0.003553
0.65,10.9	0.003553
0.65,11.0	0.003553

0.65,11.1	0.003553
0.65,11.2	0.003553
0.65,11.3	0.003553
0.65,11.4	0.003553
0.65,11.5	0.003553
0.65,11.6	0.003553
0.65,11.7	0.003553
0.65,11.8	0.003553
0.65,11.9	0.003553
0.65,12.0	0.003553
0.65,12.1	0.003553
0.65,12.2	0.003553
0.65,12.3	0.003553
0.65,12.4	0.003553
0.65,12.5	0.003553
0.65,12.6	0.003553
0.65,12.7	0.003553
0.65,12.8	0.003553
0.65,12.9	0.003553
0.65,13.0	0.003553
0.65,13.1	0.003553
0.65,13.2	0.003553
0.65,13.3	0.003553
0.65,13.4	0.003553
0.65,13.5	0.003553
0.65,13.6	0.003553
0.65,13.7	0.003553
0.65,13.8	0.003553
0.65,13.9	0.003553
0.65,14.0	0.003553
0.65,14.1	0.003553
0.65,14.2	0.003553
0.65,14.3	0.003553
0.65,14.4	0.003553
0.65,14.5	0.003553
0.65,14.6	0.003553
0.65,14.7	0.003553
0.65,14.8	0.003553
0.65,14.9	0.003553
0.65,15.0	0.003553
0.65,15.1	0.003553
0.65,15.2	0.003553

0.65,15.3	0.003553
0.65,15.4	0.003553
0.65,15.5	0.003553
0.65,15.6	0.003553
0.65,15.7	0.003553
0.65,15.8	0.003553
0.65,15.9	0.003553
0.65,16.0	0.003553
0.65,16.1	0.003553
0.65,16.2	0.003553
0.65,16.3	0.003553
0.65,16.4	0.003553
0.65,16.5	0.003553
0.65,16.6	0.003553
0.65,16.7	0.003553
0.65,16.8	0.003553
0.65,16.9	0.003553
0.65,17.0	0.003553
0.65,17.1	0.003553
0.65,17.2	0.003553
0.65,17.3	0.003553
0.65,17.4	0.003553
0.65,17.5	0.003553
0.65,17.6	0.003553
0.65,17.7	0.003553
0.65,17.8	0.003553
0.65,17.9	0.003553
0.65,18.0	0.003553
0.65,18.1	0.003553
0.65,18.2	0.003553
0.65,18.3	0.003553
0.65,18.4	0.003553
0.65,18.5	0.003553
0.65,18.6	0.003553
0.65,18.7	0.003553
0.65,18.8	0.003553
0.65,18.9	0.003553
0.65,19.0	0.003553
0.65,19.1	0.003553
0.65,19.2	0.003553
0.65,19.3	0.003553
0.65,19.4	0.003553

0.65,19.5	0.003553
0.65,19.6	0.003553
0.65,19.7	0.003553
0.65,19.8	0.003553
0.65,19.9	0.003553
0.65,20.0	0.003553
0.65,20.1	0.003553
0.65,20.2	0.003553
0.65,20.3	0.003553
0.65,20.4	0.003553
0.65,20.5	0.003553
0.65,20.6	0.003553
0.65,20.7	0.003553
0.65,20.8	0.003553
0.65,20.9	0.003553
0.65,21.0	0.003553
0.65,21.1	0.003553
0.65,21.2	0.003553
0.65,21.3	0.003553
0.65,21.4	0.003553
0.65,21.5	0.003553
0.65,21.6	0.003553
0.65,21.7	0.003553
0.65,21.8	0.003553
0.65,21.9	0.003553
0.65,22.0	0.003553
0.65,22.1	0.003553
0.65,22.2	0.003553
0.65,22.3	0.003553
0.65,22.4	0.003553
0.65,22.5	0.003553
0.65,22.6	0.003553
0.65,22.7	0.003553
0.65,22.8	0.003553
0.65,22.9	0.003553
0.65,23.0	0.003553
0.65,23.1	0.003553
0.65,23.2	0.003553
0.65,23.3	0.003553
0.65,23.4	0.003553
0.65,23.5	0.003553
0.65,23.6	0.003553

0.65,23.7	0.003553
0.65,23.8	0.003553
0.65,23.9	0.003553
0.65,24.0	0.003553
0.65,24.1	0.003553
0.65,24.2	0.003553
0.65,24.3	0.003553
0.65,24.4	0.003553
0.65,24.5	0.003553
0.65,24.6	0.003553
0.65,24.7	0.003553
0.65,24.8	0.003553
0.65,24.9	0.003553
0.65,25.0	0.003553
0.65,25.1	0.003553
0.65,25.2	0.003553
0.65,25.3	0.003553
0.65,25.4	0.003553
0.65,25.5	0.003553
0.65,25.6	0.003553
0.65,25.7	0.003553
0.65,25.8	0.003553
0.65,25.9	0.003553
0.65,26.0	0.003553
0.65,26.1	0.003553
0.65,26.2	0.003553
0.65,26.3	0.003553
0.65,26.4	0.003553
0.65,26.5	0.003553
0.65,26.6	0.003553
0.65,26.7	0.003553
0.65,26.8	0.003553
0.65,26.9	0.003553
0.65,27.0	0.003553
0.65,27.1	0.003553
0.65,27.2	0.003553
0.65,27.3	0.003553
0.65,27.4	0.003553
0.65,27.5	0.003553
0.65,27.6	0.003553
0.65,27.7	0.003553
0.65,27.8	0.003553

0.65,27.9	0.003553
0.65,28.0	0.003553
0.65,28.1	0.003553
0.65,28.2	0.003553
0.65,28.3	0.003553
0.65,28.4	0.003553
0.65,28.5	0.003553
0.65,28.6	0.003553
0.65,28.7	0.003553
0.65,28.8	0.003553
0.65,28.9	0.003553
0.65,29.0	0.003553
0.65,29.1	0.003553
0.65,29.2	0.003553
0.65,29.3	0.003553
0.65,29.4	0.003553
0.65,29.5	0.003553
0.65,29.6	0.003553
0.65,29.7	0.003553
0.65,29.8	0.003553
0.65,29.9	0.003553
0.65,30.0	0.003553
0.65,30.1	0.003553
0.65,30.2	0.003553
0.65,30.3	0.003553
0.65,30.4	0.003553
0.65,30.5	0.003553
0.65,30.6	0.003553
0.65,30.7	0.003553
0.65,30.8	0.003553
0.65,30.9	0.003553
0.65,31.0	0.003553
0.65,31.1	0.003553
0.65,31.2	0.003553
0.65,31.3	0.003553
0.65,31.4	0.003553
0.65,31.5	0.003553
0.65,31.6	0.003553
0.65,31.7	0.003553
0.65,31.8	0.003553
0.65,31.9	0.003553
0.65,32.0	0.003553

0.65,32.1	0.003553
0.65,32.2	0.003553
0.65,32.3	0.003553
0.65,32.4	0.003553
0.65,32.5	0.003553
0.65,32.6	0.003553
0.65,32.7	0.003553
0.65,32.8	0.003553
0.65,32.9	0.003553
0.65,33.0	0.003553
0.65,33.1	0.003553
0.65,33.2	0.003553
0.65,33.3	0.003553
0.65,33.4	0.003553
0.65,33.5	0.003553
0.65,33.6	0.003553
0.65,33.7	0.003553
0.65,33.8	0.003553
0.65,33.9	0.003553
0.65,34.0	0.003553
0.65,34.1	0.003553
0.65,34.2	0.003553
0.65,34.3	0.003553
0.65,34.4	0.003553
0.65,34.5	0.003553
0.65,34.6	0.003553
0.65,34.7	0.003553
0.65,34.8	0.003553
0.65,34.9	0.003553
0.65,35.0	0.003553
0.65,35.1	0.003553
0.65,35.2	0.003553
0.65,35.3	0.003553
0.65,35.4	0.003553
0.65,35.5	0.003553
0.65,35.6	0.003553
0.65,35.7	0.003553
0.65,35.8	0.003553
0.65,35.9	0.003553
0.65,36.0	0.003553
0.65,36.1	0.003553
0.65,36.2	0.003553

0.65,36.3	0.003553
0.65,36.4	0.003553
0.65,36.5	0.003553
0.65,36.6	0.003553
0.65,36.7	0.003553
0.65,36.8	0.003553
0.65,36.9	0.003553
0.65,37.0	0.003553
0.65,37.1	0.003553
0.65,37.2	0.003553
0.65,37.3	0.003553
0.65,37.4	0.003553
0.65,37.5	0.003553
0.65,37.6	0.003553
0.65,37.7	0.003553
0.65,37.8	0.003553
0.65,37.9	0.003553
0.65,38.0	0.003553
0.65,38.1	0.003553
0.65,38.2	0.003553
0.65,38.3	0.003553
0.65,38.4	0.003553
0.65,38.5	0.003553
0.65,38.6	0.003553
0.65,38.7	0.003553
0.65,38.8	0.003553
0.65,38.9	0.003553
0.65,39.0	0.003553
0.65,39.1	0.003553
0.65,39.2	0.003553
0.65,39.3	0.003553
0.65,39.4	0.003553
0.65,39.5	0.003553
0.65,39.6	0.003553
0.65,39.7	0.003553
0.65,39.8	0.003553
0.65,39.9	0.003553
0.65,40.0	0.003553
0.65,40.1	0.003553
0.65,40.2	0.003553
0.65,40.3	0.003553
0.65,40.4	0.003553

0.65,40.5	0.003553
0.65,40.6	0.003553
0.65,40.7	0.003553
0.65,40.8	0.003553
0.65,40.9	0.003553
0.65,41.0	0.003553
0.65,41.1	0.003553
0.65,41.2	0.003553
0.65,41.3	0.003553
0.65,41.4	0.003553
0.65,41.5	0.003553
0.65,41.6	0.003553
0.65,41.7	0.003553
0.65,41.8	0.003553
0.65,41.9	0.003553
0.65,42.0	0.003553
0.65,42.1	0.003553
0.65,42.2	0.003553
0.65,42.3	0.003553
0.65,42.4	0.003553
0.65,42.5	0.003553
0.65,42.6	0.003553
0.65,42.7	0.003553
0.65,42.8	0.003553
0.65,42.9	0.003553
0.65,43.0	0.003553
0.65,43.1	0.003553
0.65,43.2	0.003553
0.65,43.3	0.003553
0.65,43.4	0.003553
0.65,43.5	0.003553
0.65,43.6	0.003553
0.65,43.7	0.003553
0.65,43.8	0.003553
0.65,43.9	0.003553
0.65,44.0	0.003553
0.65,44.1	0.003553
0.65,44.2	0.003553
0.65,44.3	0.003553
0.65,44.4	0.003553
0.65,44.5	0.003553
0.65,44.6	0.003553

0.65,44.7	0.003553
0.65,44.8	0.003553
0.65,44.9	0.003553
0.65,45.0	0.003553
0.65,45.1	0.003553
0.65,45.2	0.003553
0.65,45.3	0.003553
0.65,45.4	0.003553
0.65,45.5	0.003553
0.65,45.6	0.003553
0.65,45.7	0.003553
0.65,45.8	0.003553
0.65,45.9	0.003553
0.65,46.0	0.003553
0.65,46.1	0.003553
0.65,46.2	0.003553
0.65,46.3	0.003553
0.65,46.4	0.003553
0.65,46.5	0.003553
0.65,46.6	0.003553
